# Increased MicroRNA Activity in Human Cancers

**DOI:** 10.1371/journal.pone.0006045

**Published:** 2009-06-25

**Authors:** Ariel Israel, Roded Sharan, Eytan Ruppin, Eithan Galun

**Affiliations:** 1 Goldyne Savad Gene Therapy Institute, Hadassah Hebrew University Hospital, Jerusalem, Israel; 2 School of Computer Science, Tel Aviv University, Tel Aviv, Israel; 3 School of Medicine, Tel Aviv University, Tel Aviv, Israel; Bauer Research Foundation, United States of America

## Abstract

MicroRNAs (miRNAs) are small regulatory RNAs that act by blocking the translation and increasing the degradation of target transcripts. MiRNAs play a critical role in many biological processes including development and differentiation and many studies have shown that major changes in miRNA levels occur in cancer. Since miRNAs degrade target messages, we used this property to develop a novel computational method aimed at determining the actual biological activity of miRNAs using variations in gene expression. Using the method described here, we quantified miRNA activity in papillary thyroid carcinoma and breast cancer, and found a strong and distinctive signal of increased global miRNA activity, embedded in the pertaining gene expression measurements. Interestingly, we found that in these two cancers, miRNA activity is globally increased, and is associated with a global downregulation of miRNA target genes. This downreguation of miRNA regulated genes is particularly noticeable for genes carrying multiple target sites for miRNAs. Among the miRNA-repressed genes, we found a significant enrichment of known tumor suppressors, thereby suggesting that the increased miRNA activity was indeed tumorigenic.

## Introduction

MicroRNAs are single-stranded non-coding RNA molecules of about 22 nucleotides that pair with messenger RNAs (mRNAs) carrying a complementary sequence [1]. MicroRNAs bind to target mRNAs within the RNA-induced silencing complex (RISC), which contains a member of the Argonaute protein family. This binding prevents translation and accelerates the degradation of the targeted mRNAs [2]. In the last few years, miRNAs have been shown to play a key role in the regulation of gene expression, and there is evidence that miRNAs are involved in central biological processes, including development, organogenesis, tissue differentiation, cell cycle, and metabolism [3]–[5]. Remarkably, the spatial and temporal expression of miRNAs is characteristic of tissues and developmental stages, and several studies have shown a link between miRNAs expressed in particular tissue types and regulation of tissue-specific genes [6].

Changes in miRNA expression have been shown to occur in cancer [7] However, the nature and impact of most of these changes remain unclear. Notably, conflicting findings exist over the question of whether miRNA levels are globally decreased or increased in cancer. Mature miRNAs have been shown in some studies to be decreased in cancer [8], while other studies have detected upregulation of miRNAs in many tumors [9]. One possible explanation for these diverging findings may be differences between tumor types, tissues analysed, or even measurement techniques. Another putative explanation is that the deregulation that miRNAs undergo in cancer is a complex process. That is, the outcome of miRNA regulation on gene expression is dependent not only on the levels of the miRNAs, but also on numerous other factors that mediate the influence of miRNAs on their target genes, such as components of the RISC complex. Consequently, the availability of these factors could markedly modulate the overall influence of miRNAs effect on their target genes, and, as a result, provide additional feedback on the levels of miRNAs themselves. Such global variations of miRNA activity are suggested by studies that have shown that the Argonaute2 (*EIF2C2*) gene, which is incorporated in the RISC complex, is frequently duplicated in tumors [10]. Since this gene is not directly involved in miRNA biogenesis, one could expect that when this gene is duplicated, the degradation of miRNA target genes would increase, with no associated increase in miRNA levels. For this reason, we attempted to determine the overall effect of miRNAs on their target genes, their *biological activity*, and designed a method for measuring this effect directly from the pertaining target gene expression levels.

Our method, called MiRABELLE (MicroRNA Activity Based on Expression Levels), is based on the observation that miRNAs are known to accelerate the degradation of their target transcripts, and that this activity leaves a signature on the mRNA levels of their target genes. A decrease in the expression levels of mRNAs carrying a binding site for a miRNA species can be detected upon transfection with the cognate miRNAs [11]. Additionally, several studies have shown a clear correlation between miRNAs that are highly expressed in a given tissue and the downregulation of their target transcripts [6], [12], [13]. Thus, a shift of expression of the set of genes targeted by a miRNA in a sample is an indication that the biological activity of a corresponding miRNA species changes in this sample.

The MiRABELLE approach relies on the principle that gene expression levels reflect the regulatory effect of higher-order modules, and therefore, one could assess the impact of these modules, by observing transcriptional changes [14], [15]. In particular, recent reports have shown that variation in the activity of miRNAs can be detected by comparing the levels of miRNA target genes across various tissues [16], [17]. Our method, though similar in its concept, diverges from previously published approaches in that it is designed to capture characteristic features of miRNA regulation on mRNA transcripts.

It is widely accepted that miRNA binding is determined primarily by the 3′ untranslated region (UTR) of mRNAs. The presence of a 7-mer complementary to a miRNA seed (nucleotides 2–8) in the 3′ UTR of a gene is a key factor of miRNA recognition; and several algorithms, such as TargetScan [18], [19], have succeeded to identify miRNA-gene associations by carefully identifying genes with conserved sequences corresponding to miR recognition patterns. Nevertheless, an established association between a miRNA and a target gene does not imply that all transcripts produced from this gene would be subject to miRNA regulation. About half of human genes can undergo polyadenylation at multiple sites, or be subject to alternative splicing influencing their last exons [20], [21], and thus produce transcripts which differ in their 3′ UTR sequences. Among mRNAs transcribed from such loci, only those that carry the miRNA recognized sequence between the stop codon and the polyA tail would be affected by miRNA regulation. This property, remarkably differentiates miRNA effect from transcription regulation occurring at the gene promoter site, and which affects all isoforms of the gene. We used this property to design a method that would specifically assess the effect of miRNA regulation.

This approach is made possible by the fact that some microarray platforms, like Affymetrix, measure transcript abundance using several sequences taken from the 3′ end of the gene. Affymetrix gene expression data is summarized in probe-sets, and there are several probe-sets available for most genes. Some of these probe-sets are reliable indicators of miRNA activity, meaning that they detect sequences that could only appear in the isoforms of a gene that include a binding site for a given miRNA: all of the transcripts detected by these probe-sets would be affected by a change in the activity of a given miRNA. The first step of our analysis was thus to identify these probe-sets and the miRNAs for which they detect activity. We did this by mapping the sequence of the probes, and determining their position in the gene with regard to the miRNA target sites predicted for the gene.

MiRABELLE uses the expression values measured by these probe-sets to calculate a biological activity score for each miRNA seed. Basically, it takes as input a gene expression dataset and compares, in each sample, the expression levels of probe-sets that are reliable indicators for activity of a given miRNA seed “miR-seed”, with the expression levels of other probe-sets present on the array, which serve as reference. The output of MiRABELLE is a “miR activity matrix”, providing the activity scores for each miRNA family (identified by the seed sequence), and each sample. By convention, positive scores are given when targets for a miRNA family display downregulation, indicating that the biological activity of the cognate miRNAs is increased in the sample, while negative values are obtained for upregulation of targets, suggesting a decreased activity.

## Methods

### MiRABELLE tool

MiRABELLE takes as input an expression dataset and produces a miR-seed activity matrix, giving for each sample in the dataset, the activity scores computed for each of the miRNA species for which predictions exist. Positive values are obtained when targets for a given miR-seed display more downregulation than the reference, indicating that the activity of this miR-seed is increased in the sample, while negative values are obtained for upregulation of targets, suggesting a decreased activity. This tool is written in Perl.

### MiR-seeds activity score calculation

MiRABELLE first standardizes the gene expression levels reported in the input dataset using Z-scores, to correct for the varying sensitivity of probe-sets, and disparity between average level of transcripts present in the tissue. Next, it uses the t-statistic to calculate activity scores, comparing, in each sample, the Z-scores of probe-sets that are reliable detectors of a given miR-seed, with the Z-scores of other probe-sets. We used the two-tailed, two-sample t-statistic, with unequal variance (two sample Welch's t-statistic). On a randomly shuffled gene expression dataset, the variance of this statistic is 1, and its distribution is normal when based on at least 20 detector probe-sets.

### MiRNA targets predictions

In this study, we used TargetScan 4.0 predictions (July 2007) of miRNA targets [18], [19]. TargetScan 4.0 uses both evolutionary conservation, and specific context determinants to predict miRNA targets. Targetscan 4.0 predictions are available for about 158 different conserved miRNA seeds, representative of about 450 mature miRNAs in the human. Among these miR-seeds, four were reliably detected by less than 20 probe-sets in the microarray, and were hence excluded from the analyses.

### Mapping of probe-sets to miR-seeds

In Affymetrix gene expression microarrays, it is common to have several probe-sets for the same gene, each recognizing a different sequence. According to the location of the sequences recognized by probe-sets, it is possible to determine whether a given probe-set detects only isoforms that carry a miR-seed target site, or also isoforms that may be exempt from the miR-seed target site. Probe-sets detecting exclusively transcripts carrying a miR-seed target are more strongly affected by miRNA regulation than probe-sets that detect a region of the gene shared by transcripts that do not carry the miR target sequence. Thus, a prior step to our analysis was to assign to each probe-set available on the microarray a list of miR-seeds that will affect all the isoforms detected by the probe-set. We used the following rule for assigning probe-sets to miR-seeds: when the sequence corresponding to a given miR-seed is directly recognized by a probe-set or is located upstream to the sequences detected by the probe-set in the gene and on the same exon, we consider this probe-set to be a reliable indicator of activity for this miR-seed. However, if the sequence corresponding to this miR-seed is located downstream to the sequences recognized by the probe-set, the probe-set might detect short mRNAs that do not contain a miRNA target sites, and we do not assign it to the miR-seed. We performed this mapping using the UCSC genome browser database [22], human build 17 (http://genome.ucsc.edu). We downloaded the TargetScan predictions from the website (http://www.targetscan.org) and mapped them to the UCSC genome; we used the “knownGene” table for mapping chromosomal locations to genes; we used the “affyU133Plus2” and “affyU133” tables for finding the location of sequences recognized by probe-sets in the genes (Text S1).

### Datasets analyzed

Pappilary thyroid carcinoma and breast cancer datasets were retrieved from the GEO database [23], accession GSE3467, GSE3744, and ArrayExpress database [24], accession E-MEXP-882. We used the normalized expression values from the database. When raw data was available, we downloaded it, and renormalized it using GCRMA, RMA and MAS 5.0 packages on Bioconductor [25]. MiRABELLE predictions and enrichment of downregulated targets were not significantly affected by the normalization algorithm used. Validation experiments of transfection with microRNAs and antagomirs were retrieved from the GEO database, accession GDS1858, GDS2657, and GSE3425.

### TF enrichment analysis

Binding sites (BS) for TF were determined by scanning the promoters of all probe-sets present in the affymetrix microarray for matches with Transfac matrices, as described in [26]. In each promoter, the 500 base pairs (bp) immediately before the transcription start sites were scanned, in accordance with the fact that most active TFBS appear close to the transcription start site [27]. The hypergeometric distribution was used to assess enrichment between the background set of probe-sets and a sample set.

### GO Annotations

We used the GO biological process annotations for the U133Plus2 array from Affymetrix website (http://www.affymetrix.com).

### Statistics

We used the two-tailed, two-sample t-test with equal variance in order to identify the miR-seeds showing the most significant deviations in tumors vs. normal tissues, and ranked these MiRs according to this test. This test was performed in Excel. We performed one sample Kolmogorov-Smirnov (KS) tests to assess the normality of the distribution of miR activity scores computed by MiRABELLE on the dataset, and two-sample KS tests for comparing the distributions of miR activity scores computed from the original and a randomly shuffled dataset. The KS tests and the associated histograms were performed in Matlab 7 (Mathworks). The enrichment tests were performed using the hypergeometric cumulative distribution function in Matlab.

### Enrichment of downregulated genes with increasing number of miR target sites (Table 1)

**Table 1:** Gene expression trends obtained for probe-sets that were mapped to the 77 miR-seeds that were found by t-test to display the most significant upregulation in breast cancer (FWER p<0.05).

minimal number of target sites in transcripts	Up-	Down-	percentage of downregulated probe-sets	P-value for enrichment (hypergeometric distribution)
	regulated probe-sets			
0	23660	16879	41.6%	
1	4473	5069	53.1%	5.94·10^−148^
2	2999	3575	54.4%	1.36·10^−4^
3	2164	2715	55.6%	2.66·10^−4^
4–5	1576	2104	57.2%	9.87·10^−5^
6–7	882	1316	59.9%	3.27·10^−5^
8–9	547	821	60.0%	4.48·10^−1^
10–11	303	547	64.4%	1.81·10^−5^
12–13	191	341	64.1%	6.08·10^−1^
14–15	117	250	68.1%	2.81·10^−3^
16–18	69	159	69.7%	2.30·10^−1^
19–21	42	92	68.7%	7.15·10^−1^
22–24	24	59	71.1%	2.79·10^−1^
25–26	12	32	72.7%	4.56·10^−1^
27–30	6	21	77.8%	2.72·10^−1^
31–33	2	9	81.8%	5.28·10^−1^
> = 34	0	9	100.0%	1.82·10^−1^

The table gives the expression trends observed from probe-sets, according to the number of target sites predicted in the transcripts they detect. We observe that the proportion of probe-sets detecting downregulation in tumors gradually increases with the number of target sites for these *miRs*, and this increase is associated with significant hypergeometric p-values.

For the given set of miR-seeds, we performed enrichment tests iteratively for each *i* between 0 and the maximal number of target sites, as follows. The total population size (N) was the number of informative probe-sets having at least *i* target sites for the considered set of miR-seeds, the number of probe-sets reporting downregulation were counted as successes (m). We tested for enrichment of downregulation in the sample of probe-sets detecting at least *i+1* target sites for these miR-seeds.

### Enrichment of GO annotations (Table S6)

We used the hyper-geometric distribution to identify the most significantly enriched annotations in the sample of 9542 probe-sets associated with the 77 miR-seeds, with regard to the population of all probe-sets on the array (A). 5069 of these miR targets were effectively downregulated in tumors. In the second analysis, we identified the most significantly enriched annotations in the sample of 5069 downregulated probe-sets, with regard to the population of the 9542 predicted targets (B).

## Results

### Validation of the MiRABELLE Method

We first validated the MiRABELLE method in tissues where the abundance of a particular miRNA had been experimentally increased. Lim et al. [11] transfected HeLa cells with miR-124, miR-1, and miR-373, and measured gene expression after 12 and 24 hours. Wang et al. [28] transfected HepG2 cells with miR-124 and measured gene expression at various time intervals. We subjected the gene expression data to MiRABELLE analysis, which computed the miR-seed activity for each sample. In the samples that were transfected with microRNAs, our tool correctly identified very significant increases in microRNA activity, specifically for the miR-seeds miR-124, miR-1, and miR-373, in the corresponding experiments (Text S1). In order to ensure that MiRABELLE is suitable for detection of changes of miRNA activity occurring *in vivo*, we analyzed the gene expression data generated in the experiment of Krutzfeldt et al., where miR-122 was silenced by systemic injection of an antagomir [29]. We found that our tool correctly identified the significant decrease of activity for miR-122 occuring after treatment with the antagomir (Text S1).

### Inferring MicroRNA activity in Human Papillary Thyroid Carcinoma

Papillary Thyroid Carcinoma (PTC) is a malignancy that accounts for ∼80% of human thyroid cancers. Two independent studies have reported specific alterations of miRNA levels in PTC. It was therefore interesting to quantify the biological activity of miRNAs in PTC using the MiRABELLE tool and compare these with the changes in miRNA expression levels reported in these two studies.

He et al. [30] measured miRNA levels in tissue samples from 15 PTC patients using miRNA microarrays. Concomitantly, they also used Affymetrix microarrays to determine gene expression in nine tumors (T-PTC) and nine paired surrounding tissues (N-PTC). We analyzed the gene expression data using MiRABELLE to infer a miR-seed activity matrix (Table S1). As evident from this table, miR-seed activity scores were substantially higher in tumors than in normal tissues, suggesting that PTC tumors are characterized by an intensification of miRNA activity. Indeed, the median miRNA activity score in tumors is higher than the median activity score in normal samples for 97% of the miR-seeds. To determine the miR-seeds for which the increase of activity is the most pronounced in tumors, we used the two-sample t-test to compare the activity scores obtained from normal and tumor samples (Table S2). He et al., reported that miR-146, miR-221, miR-222 and miR-21, displayed the most dramatic overexpression in tumors with levels 19- to 4-fold higher in tumors than in adjacent tissue. The t-test applied to our activity scores (using only the subset of seeds that were used by He et al., in their microRNA array) has successfully identified the 3 miR-seeds belonging to these 4 microRNAs among the six most significant *p*-values (out of 65 different seeds). Thus, in the PTC case, the top miR-seeds identified by their biological activity coincide with the top overexpressed miRNAs.

**Figure 1:**
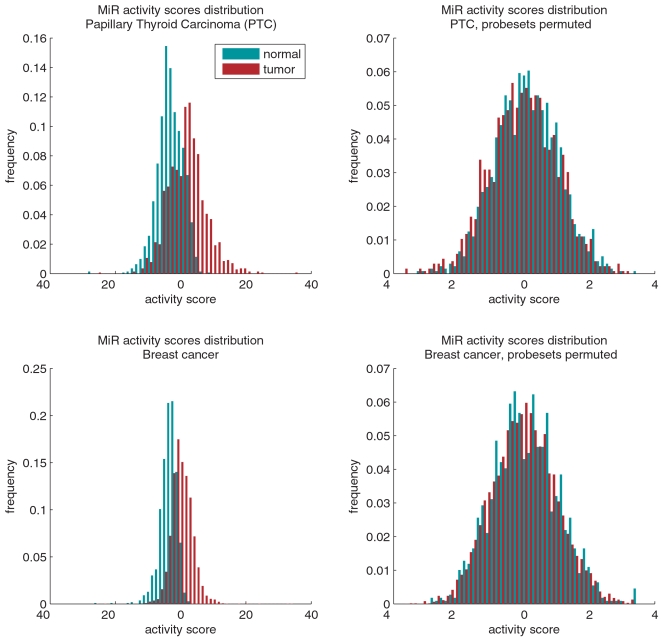
Distribution of the MiR activity scores calculated for tumor samples (red) and normal samples (cyan). (A) Histogram displaying the distribution of miR-seed activity scores computed for tumor (red) and normal samples (cyan) in the papillary thyroid carcinoma dataset. Activity of miRNAs is globally higher in tumor tissues relative to normal tissues. The KS test rejects the hypothesis of equality of the distributions of activity scores in tumor and in normal tissues, with a p-value P≈10^−126^. Normality of the activity scores is rejected with P<10^−300^. (B) To show that the deviation between scores computed for normal and tumor tissues is not due to our method of calculating the activity scores, we computed these scores from a random permutation of the probe-sets from the PTC dataset. For both normal and tumor tissues, the activity scores follow approximately a normal distribution, as expected for a t-statistic. There is no observable deviation between tumor and normal tissues, and the KS test does not reject equality of the two distributions. (C) Histogram displaying the distribution of miR-seed activity scores computed for tumor (red) and normal samples (cyan) in breast cancer dataset. MiRNA activity is significantly higher in tumor tissues relative to normal tissues. The KS test rejects the hypothesis of equality of the distributions of activity scores in tumor and in normal tissues, with a p-value P<10^−298^. (D) Histogram displaying the distribution of miR-seed activity scores for a random permutation of the probe-sets from the breast dataset.

The match between our predictions and the biological measurements is not incidental and as we show below, results from a very strong signal present in gene expression. The activity scores computed by the MiRABELLE tool are based on t-statistics, and in the absence of a common regulation of these genes, these statistics follow a normal distribution. Figure 1A displays the distribution of the miR-seed activity scores in normal and tumor tissues. It can be seen that activity scores are significantly higher in tumors than in normal tissues (*p*<10^−125^ by a Kolmogorov-Smirnov (KS) test). As a control, we computed hypothetical activity scores on the same dataset after random shuffling of the probe-sets (Fig. 1B). In the latter case, the KS test does not detect a significant difference between the distribution of activity scores in tumor and normal tissues, as one would expect.

### Inferring MicroRNA activity in breast cancer

Having found that microRNA activity is globally increased in papillary thyroid carcinoma, we proceeded to investigate miRNA activity in breast cancer, which is the second most common type of cancer, and a subject of extensive studies on gene expression, with some very valuable datasets available in public repositories. Richardson et al., published a gene expression study on breast cancer [31], which is based on a dataset that included seven normal tissue samples and 40 breast tumors, among which 18 were basal-like cancers (BLC), a poorly differentiated and highly aggressive form of cancers. The activity matrix inferred for this dataset appears in Table S3. As demonstrated in Figure 1C, the level of miRNA biological activity in tumor samples is here also significantly higher than in normal samples; the KS test shows that activity scores in tumor and normal samples have a distinct distribution (*p*<10^−298^). Comparing the median activity scores in cancer and normal tissue samples, all but four miR-seeds appear to have higher activity in cancer than in normal tissue. Even after choosing a restrictive cut-off of *p*<3·10^−4^ (corresponding to a 0.05 significance level after a Bonferroni correction for multiple testing), we find that 77 (out of the 150) miR-seeds have a significant increased activity in tumors (Table S4). Altogether, the microRNAs corresponding to these 77 miR-seeds are predicted by TargetScan to regulate about 6,000 genes. The increased miRNA activity observed is indeed reflected in a marked decrease in the expression of these target genes. Out of the 9,542 probe-sets associated with transcripts regulated by one of these 77 miR-seeds, 5,069 (53.1%) have lower average expression in tumors (i.e., the corresponding target genes are downregulated) than in normal tissue, compared with 41.6% of all the 40,539 probe-sets in the array (*p*<10^−147^ by a hypergeometric test, Figure 1).

To provide additional support for our conclusion that microRNAs are the main cause of the massive downregulation of these genes, we examined the extent of gene target downregulation as a function of the number of the associated binding sites for the miR-seeds. This investigation has been motivated by previous observations suggesting that the mRNA degradation following binding of miRNA is more efficient for transcripts carrying multiple target sites for miRNAs [32]–[34]. Table 1 summarizes the expression trends observed for probe-sets that are mapped to the 77 miR-seeds displaying the most significant increase of activity in tumors. As mentioned above, 9,542 probe-sets detect transcripts that have at least one binding site for MiR-seeds, 53.1% of them displaying downregulation in tumors. When one considers the 6,574 probe-sets detecting transcripts carrying at least 2 target sites, the percentage improves to 54.4% (p<1.3·10^−4^ by a hypergeometric test), and it continues to increase for probe-sets detecting transcripts carrying more sites for miRNA binding, reaching about 70% for the 228 probe-sets detecting transcripts with more than 15 target sites. It is remarkable that even though MIRABELLE algorithm did not use the number of miRNA binding sites in its calculations–activity scores were computed from probe-sets identified as indicators of a miR-seed, regardless of the number of binding sites they may carry–the miRNAs that it identified as undergoing a significant change activity, displayed a clear dose-response effect. Hence, the gradual increase in the proportion of downregulated genes with the number of target sites provides strong support to the notion that the decreased expression of target genes is indeed due to increased miRNA activity.

Since many genes are regulated by several miRNA species, we wished to ensure that the detected global downregulation of miRNA target genes was not due to the increase in just a few specific species of miRNAs (which share target genes with the other miRNA species) out of this set of 77 active miR-seeds. To this end, we performed the hypergeometric enrichment test described earlier (i.e,. testing for enrichment of down-regulated genes among probe-sets that detect targets of at least one miR-seed in the set examined) in an iterative manner. In the first iteration, we examined the hypergeometric enrichment obtained when considering the expression patterns of the targets of only the first miR-seed. In the second iteration, we examined the hypergeometric enrichment obtained when considering the expression patterns of the targets of the first and second miR-seeds, and so on. Remarkably, the best score was obtained when considering the targets of the first 74 miR-seeds, confirming that, indeed, almost all of the original 77 biologically-active miRNA species contribute to the observed expression downregulation. To ensure that these results were not due to specific features of the analyzed data, we investigated other breast cancer datasets, such as E-MEXP-882 [35], and processed them with different normalization schemes (GC-RMA, MAS5.0). Using the hypergeometric test, we found a similarly significant downregulation of miRNA target genes (81 biologically-active miR-seeds, p<10^−134^ hypergeometric enrichment test), furthering strengthening the hypothesis that the global downregulation of miRNA targeted genes observed in breast cancer is not specific to a particular study.

A potential cause for the observed downregulation of microRNA target genes could be the action of transcription factors (TFs) that co-regulate those genes [36]. Searching for known TF binding sites in the promoter regions of target transcripts of each of the 77 miR-seeds, we found a significant enrichment (*p*<0.05) for 82 TFs (Methods). Binding sites for these TFs were found in 30% of the probe-sets present in the array. After excluding all transcripts with putative binding sites for those TFs, we still observed a very significant p-value for enrichment of downregulated genes among miRNA targets (p<10^−96^).

Next, we set to list the genes that have predicted target sites for the 77 miR-seeds and are therefore expected to be affected by the increased miRNA activity (Table S5). We functionally characterized them using gene ontology (GO) annotation, and looked for statistical enrichment of specific annotations (Table S6). This analysis shows that several biological processes were overrepresented among gene targets of these miRNAs: regulation of transcription, development and differentiation, ubiquitin cycle, signal transduction, transport, and tumor suppression (synonym category: regulation of progression through cell cycle) (FDR *p*<10^−9^). As we saw earlier, most of these genes displayed decreased expression values in tumors, but not all. We looked for functional annotations that could characterize the probe-sets that were downregulated in tumors, compared to the other predicted targets that were not effectively downregulated. We found that cell-cycle arrest was the most significantly enriched annotation (FDR *p*<2·10^−2^), suggesting that miRNA-regulated genes that cause cell-cycle arrest are indeed downregulated in tumors.

Since the efficiency of gene silencing by microRNAs improves with the number of target sites, we looked for genes carrying the highest number of target sites for our 77 miR-seeds. This corresponds to the top of Table S5. In accordance with the top identified GO annotations, we find genes regulating gene transcription: *CPEB4* (cytoplasmic polyadenylation element binding 4), which encodes a protein believed to control polyadenylation-induced translation in early development, has the highest number of target sites (38), and is downregulated. Two other members of the CPEB family, *CPEB2* and *CPEB3*, also appear high in the list with 26 and 25 target sites, respectively, and are also downregulated; *DDX3X* (DEAD box polypeptide 3, X-linked), a RNA helicase, has 33 target sites, and its associated probe-sets also report downregulation (p = 0.002, 0.00001); *PURB* (Purine-rich element binding protein B), a single-stranded DNA binding protein that is implicated in the control of both DNA replication and transcription has 30 target sites (deletion of this gene has been associated with myelodysplastic syndrome and acute myelogenous leukemia) and is downregulated. *MECP2* (methyl CpG binding protein 2), which participates in the repression of methylated promoters, carries 29 sites, and appears to be efficiently silenced (t-test *p*≈0.001 for 2 probe-sets detecting transcripts with target sites); likewise *ZBTB4*, a CpG binding protein that participates in the repression of transcription from methylated promoters carries 22 sites and its transcript levels appear to be significantly reduced (*p*<10^−7^).

We also find important tumor suppressors: *FOXP1* (Forkhead box P1), a transcription factor that is believed to act as a tumor suppressor as it is lost in several tumor types, carries 15 predicted target sites from the most upregulated miR-seeds, and a significant downregulation is detected. Also, *KLF4* (Kruppel-like factor) that controls the G1/S cell cycle checkpoint upon DNA damage has 14 predicted target sites for the most upregulated miR-seeds; (t-test showing that its expression levels are very significantly reduced in cancer (p<0.000001 for the two probe-sets available for this gene)). *ESR1* (estrogen receptor 1), a gene frequently silenced in breast cancer, carries 12 predicted miRNA targets, and is downregulated. Interestingly, *PDCD4* (Programmed Cell Death 4), a tumor suppressor whose inhibition in breast cancer has been linked to an increase in miR-21 [37], has 7 target sites for this group of miRNAs (including one for miR-21), and here is also significantly downregulated.

**Figure 2:**
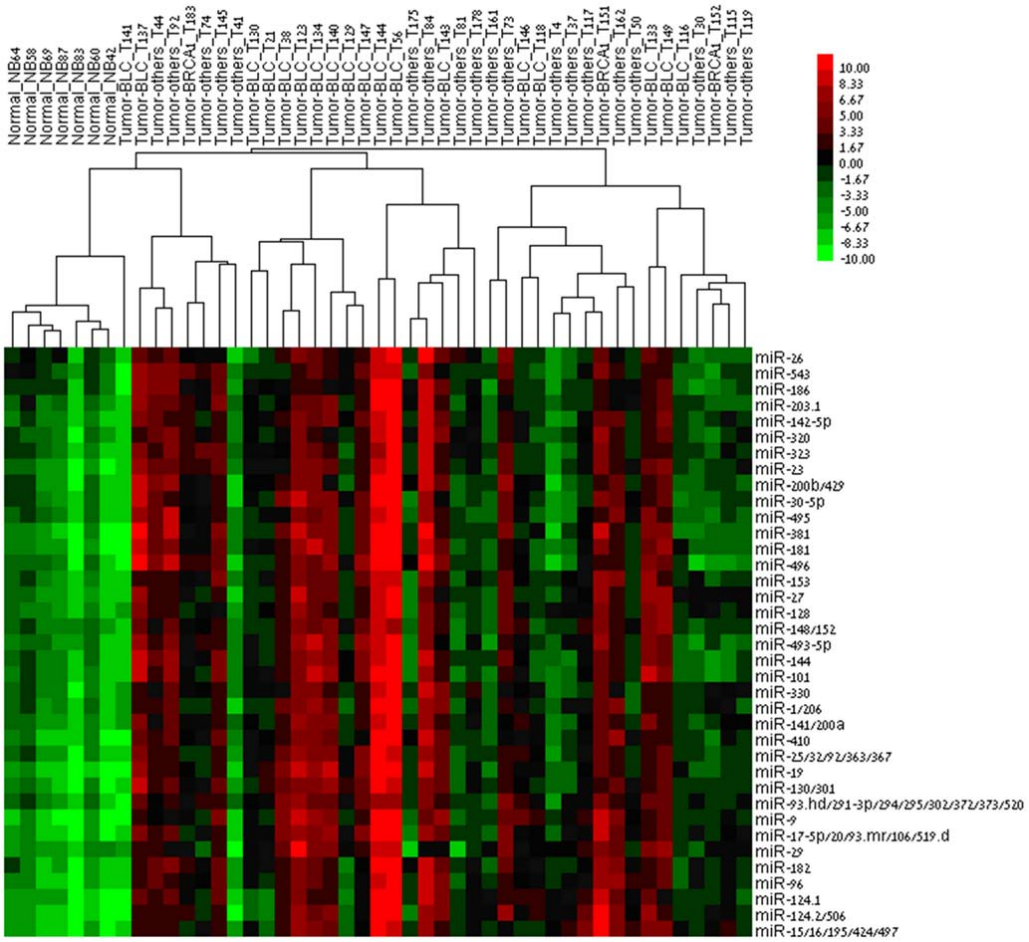
Hierarchical clustering of breast samples by MiR-seed activity scores. Clustering was done by complete linkage on cluster 3.0 according to the correlation (centered) similarity metric, after selecting miR-seeds for which the SD is at least 4. It can be observed that normal samples are clustered together. Most of the BLC tumor samples are also grouped together. Prior to clustering, samples were randomly shuffled. Color code: red means increased activity of miR-seeds; green decreased (Spearman rank correlation, complete linkage).

Lu et al. [8] have shown that microRNA levels measured by bead-flow permit the classification of human cancers by non-supervised methods such as hierarchical clustering. To ascertain that the miRNA activity scores computed by MIRABELLE do also provide this level of information, we subjected the miR-seed activity matrix produced for the breast cancer datasetto hierarchical clustering [38]. Interestingly, hierarchical clustering based on activity scores allowed to distinguish between tumor and non-tumor samples (Fig. 2). Moreover, the clustering procedure appears to distinguish between subtypes of tumors, with most BLC samples grouped together.

## Discussion

We have described here a method that allows inferring of miRNA activity from gene expression data. During the course of this study, other reports were published presenting analogous approaches [16], [17], which also concluded that miRNA regulation information can be extracted from gene expression data. The method we present here, although similar in its principle with the methods described in these reports, is the first to incorporate input from the location of the probes within the gene to detect variations that are specific to changes in miR activity. By applying this method to two types of human cancer, we found a strong signal of increased miRNA activity in tumors. This increase appears to have a very significant impact on gene expression, and we observe a substantial number of miRNA target genes downregulated in tumors, with the downregulation being stronger for genes carrying multiple sites for miRNAs. Among these genes, we identified a significant enrichment of tumor suppressor genes, suggesting a mechanism by which the increase in miRNA activity could be advantageous to tumors. In addition to their destabilizing effect on mRNA transcripts, microRNAs have been shown to repress protein translation, and therefore, one could expect that at the protein level, these miRNAs repressed genes are even more strongly silenced than what their probe-sets suggest.

Our method uses the changes in mRNA levels detected by indicator probe-sets to measure changes in miRNA activity across samples. It is remarkable that the moderate fluctuations exerted by miRNAs at the mRNA level, could give such a strong signal of miRNA activity when collected from these probe-sets, and this finding is consistent with reports that show that each miRNA represses hundreds of genes at the mRNA level [11], [39], [40]. In addition, the statistical signal of miRNA activity in gene expression data has also been observed in two recent reports [16], [17].

Our activity scores are based on variations in levels of mRNA transcripts, and the specific effect of microRNAs on protein translation is not assessed by these scores. Nonetheless, there is increasing evidence that the translational effect of microRNAs is accompanied by changes in mRNA level, and that mRNA destabilization is the dominant component of repression. Baek et al. [39], have shown that genes undergoing microRNA repression by more than a third also display detectable mRNA destabilization, and that highly repressed targets were silenced primarily at the mRNA level. Additionally, Selbach et al. [40] showed that most microRNA targets are repressed both at the mRNA and the translational levels. MicroRNA species typically repress hundreds of targets, and most of them are repressed at least at the mRNA level. Therefore, it is reasonable to think that microRNAs that decrease or increase significantly their activity would be noticed by their effect on mRNA levels.

The increase in activity of miRNAs that we identified in the two types of cancer studied could be due either to increased biogenesis of miRNAs, or to improved efficiency of the mediators of miRNA effect on target genes. In the first case, the increase in miRNA activity would have to be accompanied by an increase in the levels of miRNAs, while in the latter, mature miRNA transcripts levels may not undergo a significant change. In PTC and breast cancer, an increase in the level of few miRNAs species has been reported [30], [41], [42], but not a global increase in the levels of many miRNA species. Such a global increase could suggest an alteration in the post-biogenesis regulation of the miRNA effects. This may possibly occur at the level of the Argonaute proteins, which form the core components of the RISC effector complex RISC mediating miRNA function [1]. In support of this hypothesis, a genome-wide search for copy number alterations in cancer has shown frequent duplication of the Argonaute2 (*EIF2C2*) gene, as well as *DICER1* in tumors [10].

Interestingly, Kumar et al., have found that tumorigenesis is accelerated after DICER conditional deletion in the K-ras model of lung cancer, and that RNA interference against DICER increases cell-growth in several tumor cell lines [43]. This suggests that DICER repression, and hence reduction of miRNA activity, enhances tumorigenesis, and could appear to conflict with our findings that breast cancer and PTC are accompanied by an increase in miRNA activity. In accordance with Kumar et al., who stated that other studies showed that overexpression of miRNAs accelerates tumorigenesis, we do not think that there is a contradiction here, and the effect of changes in miRNA activity are likely to vary with cancer types and stages of tumorigenesis.

Further biological studies would be needed to confirm the increase of miRNA activity identified here bioinformatically in two types of cancer and identify its mechanism. If the downregulation is confirmed, this would suggest that the increase in miRNA activity occurring in certain types of cancer as reported here may play a key role in the pathogenesis of these types of cancers, and could offer an attractive diagnostic and therapeutic avenue.

## Supporting Information

Text S1 Supplementary methods
(1.10 MB DOC)

Table S1 MiR-seed activity matrix - Papillary Thyroid Carcinoma
(0.03 MB PDF)

Table S2 MiR-seeds found to be the most significantly upregulated in PTC
(0.04 MB PDF)

Table S3 MiR-seed activity matrix - Breast cancer
(0.08 MB PDF)

Table S4 MiR-seeds found to be the most significantly upregulated in breast cancer
(0.04 MB PDF)

Table S5 Expression values for genes targeted by the 77 miR-seeds upregulated in breast cancer
(1.13 MB PDF)

Table S6 GO annotation enrichment analysis for the targets of the 77 miR-seeds upregulated in breast cancer
(0.04 MB PDF)
