# Supplementary material for: Increased MicroRNA Activity in Human Cancers
Source: PLoS One. 2009 Jun 25;4(6):e6045. doi: 10.1371/journal.pone.0006045 (PMC2698213; doi:10.1371/journal.pone.0006045)
Supplement: Text S1 — Supplementary methods (1.10 MB DOC) [file pone.0006045.s001.doc]

**Supplementary Methods**

MiRABELLE analysis was designed to detect changes in microRNA (miRNA) activity from gene expression data. The algorithm used for this analysis is presented in this paper.

Its main principles are that:

- Expression values of predicted miRNA target genes are compared with expression values of all other genes on the array to predict changes of activity for each miR-seed.

- The effect of microRNA regulation on each target gene can be small, but when the expression values of many target genes are compared to a background set of genes, a significant change can be detected for targets of miRNA undergoing differential activity.

- We use miRNA target prediction algorithms that take into account the conservation of the seed complementary sequences across species, since it is usually a very good indication that the gene is regulated by the cognate miRNA.

- When available, we use information on the sequence identified by each probe-set, so that only probe-sets that detect only transcripts that must include a miR-target site serve to calculate the activity scores of the associated miR-seeds.

We validated this method using several publicly available gene expression datasets in which specific microRNAs have been transfected or silenced. In this document, we present the miR-seed activity scores computed from these datasets. We also present data from two cancer datasets that we submitted to MiRABELLE analysis, but were not included in the paper.

**Validation of the MiRABELLE algorithm on datasets with known perturbations**

**Transfection experiment with miR-124 and miR-1 (Lim et al., 2005) (GEO dataset GDS1858)**

This experiment is based on gene expression analysis of HeLa cells at 12 or 24 hours after transfection with miR-124 and miR-1. We computed the miR activity scores using MiRABELLE. The highest activity scores obtained from each sample are highlighted in red. We observe that these high scores were found for the cognate miR-seeds. A particularity of Targetscan predictions is that they list the targets of miR-124 under two entries: miR-124.2/506 (AAGGCAC) - which in later versions of TargetScan was renamed miR-124/506 - and miR-124.1 (UAAGGCA), since miR-124, miR‑506, and miR-124a have very close seeds. The miR-seed activity scores corresponding to both miR-124 entries appear to undergo the highest increase following transfection with miR-124, while following transfection with miR-1, the activity scores for miR-seed miR-1/206 undergo the highest increase. We also observe slight changes in miR activity for other miR-seeds that are very small compared to the changes observed for the seeds corresponding to transfected miRNAs. Interestingly, in the samples transfected with miR-124, the activity of other miR-seeds appear to undergo a slight reduction, possibly reflecting the repressing effect of miR-124 on DICER1 (a predicted target of miR-124), which would eventually lead to a depletion of the level of other mature miRNAs.

| **MIR-seed** | **Number of targets** | **miR-124 12 hours** | **miR-124 24 hours** | **miR-1 12 hours** | **miR-1 24 hours** |
| --- | --- | --- | --- | --- | --- |
| miR-124.2/506 | 605 | 9.1 | 13.8 | -6.2 | -5.6 |
| miR-124.1 | 387 | 3.0 | 6.2 | -1.9 | -2.8 |
| miR-1/206 | 270 | -5.3 | -5.0 | 10.5 | 7.6 |
| miR-93.hd/291-3p/294/295/302/372/373/520 | 227 | -2.1 | -2.1 | 1.1 | -1.4 |
| miR-130/301 | 275 | -2.9 | -2.8 | 0.9 | -2.6 |
| miR-17-5p/20/93.mr/106/519.d | 385 | -0.4 | -2.8 | 3.4 | -2.3 |
| miR-29 | 359 | 0.6 | -0.5 | 4.7 | 0.8 |
| let-7/98 | 362 | -0.7 | -2.3 | 3.9 | 0.7 |
| miR-148/152 | 196 | -1.8 | -2.7 | 1.1 | -1.7 |
| miR-30-5p | 436 | -1.8 | -3.0 | 2.9 | -0.3 |
| miR-409-3p | 111 | -3.3 | -2.7 | 3.3 | 2.6 |
| miR-142-5p | 179 | -2.8 | -3.1 | 0.5 | -3.3 |
| miR-23 | 286 | 0.1 | -1.9 | 1.6 | -3.2 |
| miR-144 | 244 | -2.7 | -4.1 | 0.9 | -1.0 |
| miR-138 | 187 | -2.2 | -3.1 | 1.7 | 3.0 |
| miR-25/32/92/363/367 | 252 | 0.5 | -2.5 | 2.2 | -2.3 |
| miR-320 | 261 | -1.7 | -2.2 | 3.1 | 0.6 |
| miR-19 | 358 | -0.7 | -3.5 | 2.4 | -0.8 |
| miR-323 | 243 | 0.5 | -1.5 | 1.5 | -2.4 |
| miR-141/200a | 226 | -1.8 | -1.1 | 1.0 | -2.0 |
| miR-125/351 | 290 | 0.4 | -0.3 | 2.9 | 1.7 |
| miR-18 | 79 | -0.1 | -3.9 | 1.2 | 0.6 |
| miR-224 | 96 | -1.1 | -1.5 | 3.0 | -1.2 |
| miR-27 | 395 | -1.7 | -2.6 | 2.4 | -0.2 |
| miR-24 | 193 | 0.8 | -1.1 | 1.6 | 2.2 |
| miR-324-3p | 80 | -0.8 | -2.5 | 3.0 | 1.7 |
| miR-495 | 249 | -1.1 | -1.6 | 1.7 | -0.1 |
| miR-181 | 346 | -1.8 | -1.6 | 2.6 | -0.6 |
| miR-26 | 318 | -0.2 | -1.6 | 2.4 | -1.4 |
| miR-9 | 474 | -1.3 | -0.5 | 3.0 | 0.1 |
| miR-203.1 | 219 | -1.8 | -2.1 | 1.0 | 0.0 |
| miR-15/16/195/424/497 | 384 | -0.2 | -0.8 | 1.2 | -1.6 |
| miR-329 | 103 | 0.5 | -0.7 | 3.8 | 1.1 |
| miR-199 | 152 | -0.3 | -1.8 | 3.1 | 0.0 |
| miR-132/212 | 120 | -1.7 | -2.4 | 2.5 | 0.8 |
| miR-496 | 249 | -1.0 | -0.7 | -0.7 | -4.6 |
| miR-145 | 192 | -0.8 | -0.6 | 2.3 | -0.7 |
| miR-153 | 206 | -2.2 | -2.3 | 0.4 | -1.4 |
| miR-455 | 48 | -0.3 | -3.8 | 1.7 | 0.6 |
| miR-376 | 62 | 1.2 | 1.8 | -2.4 | -2.4 |
| miR-485-3p | 78 | -0.7 | -2.4 | 2.1 | 1.8 |
| miR-370 | 105 | -0.9 | -3.1 | 1.5 | 0.2 |
| miR-381 | 257 | 0.0 | -0.9 | 0.9 | -2.2 |
| miR-499 | 94 | -2.2 | -1.2 | 0.9 | 0.9 |
| miR-183 | 135 | -1.2 | -0.5 | 2.2 | 0.3 |
| miR-129-5p | 158 | -1.0 | -1.1 | 2.2 | -1.4 |
| miR-140 | 89 | 0.0 | -1.9 | -0.9 | -0.4 |
| miR-218 | 287 | -0.8 | -1.4 | 0.3 | -0.6 |
| miR-196 | 99 | -0.9 | -0.3 | 2.0 | -1.6 |
| miR-10 | 80 | 0.3 | 0.0 | 1.3 | -0.8 |
| miR-96 | 350 | -0.4 | 1.9 | -2.6 | -0.9 |
| miR-493-5p | 219 | 1.6 | 0.1 | 1.1 | -0.9 |
| miR-155 | 114 | -1.1 | -1.5 | 1.6 | 1.2 |
| miR-135 | 230 | -0.5 | -1.7 | 2.3 | -0.5 |
| miR-338 | 113 | -0.2 | -1.4 | 1.4 | 0.4 |
| miR-24* | 38 | -0.1 | -1.5 | -1.0 | -2.2 |
| miR-330 | 205 | -1.4 | -1.4 | 1.5 | -0.9 |
| miR-448 | 202 | -2.1 | -1.6 | 0.1 | -0.5 |
| miR-324-5p | 51 | -0.4 | -1.5 | 2.7 | 0.7 |
| miR-194 | 121 | 0.3 | -0.9 | 2.5 | 0.5 |
| miR-421 | 41 | 1.2 | 1.0 | 1.7 | -0.4 |
| miR-7 | 149 | 0.4 | 0.7 | 1.4 | -1.8 |
| miR-491 | 47 | 0.0 | 0.0 | 0.2 | 3.1 |
| miR-22 | 154 | -1.2 | -1.6 | 1.9 | 1.6 |
| miR-362 | 57 | -1.1 | -0.2 | 1.6 | -1.9 |
| miR-137 | 233 | -2.0 | -1.5 | 1.5 | 0.1 |
| miR-205 | 151 | -0.6 | 1.0 | 2.0 | 0.8 |
| miR-221/222 | 97 | -0.2 | -1.9 | 2.1 | 0.6 |
| miR-128 | 343 | -1.1 | -1.6 | 1.2 | 0.2 |
| miR-28 | 63 | 1.5 | -0.6 | 2.0 | 0.2 |
| miR-101 | 243 | -0.8 | -2.1 | 0.2 | 0.9 |
| miR-34/449 | 222 | -0.5 | 0.1 | 2.5 | 0.7 |
| miR-219 | 131 | -0.5 | -1.2 | 2.2 | -0.5 |
| miR-378 | 158 | -0.1 | -0.8 | 1.2 | -1.1 |
| miR-335 | 84 | 2.3 | 1.8 | 0.1 | -0.2 |
| miR-452 | 85 | -1.5 | -1.4 | -1.1 | 0.4 |
| miR-30-3p | 127 | -1.8 | -1.0 | 0.8 | -1.2 |
| miR-136 | 60 | -1.5 | -0.9 | 1.5 | 1.2 |
| miR-192/215 | 46 | -1.2 | -1.2 | 1.1 | 1.9 |
| miR-186 | 214 | -1.5 | -2.0 | -0.1 | -0.8 |
| miR-193 | 78 | -2.4 | -1.0 | 0.7 | -0.1 |
| miR-374 | 166 | -0.4 | -2.0 | 1.8 | 1.1 |
| miR-149 | 149 | 2.4 | -0.5 | 0.3 | 1.7 |
| miR-543 | 190 | -1.5 | -1.9 | 0.6 | -1.0 |
| miR-200b/429 | 304 | -0.3 | -0.9 | 0.8 | -0.5 |
| miR-365 | 92 | -0.3 | -1.7 | 1.3 | -0.6 |
| miR-377 | 129 | -0.5 | -1.2 | 0.1 | -1.4 |
| miR-134 | 55 | 0.6 | 1.3 | -2.2 | -1.5 |
| miR-539 | 171 | -2.1 | -0.3 | 1.8 | -0.1 |
| miR-21 | 87 | -1.8 | -0.3 | 1.8 | -0.1 |
| miR-214 | 187 | 1.0 | -0.1 | 0.7 | 0.6 |
| miR-33 | 100 | -1.1 | -1.0 | -0.2 | -0.6 |
| miR-299-5p | 94 | -1.4 | -0.1 | 2.0 | -0.7 |
| miR-139 | 109 | -1.6 | -1.5 | -0.2 | 0.5 |
| miR-217 | 95 | -0.7 | -1.0 | 1.8 | -0.8 |
| miR-542-3p | 87 | -2.3 | 0.4 | -0.6 | 0.7 |
| miR-223 | 92 | -1.6 | -0.5 | 0.0 | -1.8 |
| miR-503 | 141 | -0.4 | -0.1 | 0.5 | -0.8 |
| miR-339 | 75 | -0.2 | -1.1 | 1.7 | -1.2 |
| miR-185 | 85 | 1.3 | 0.3 | -0.1 | 1.2 |
| miR-369-3p | 175 | 1.5 | -1.6 | 1.5 | 0.6 |
| miR-494 | 131 | -2.0 | -1.4 | 0.9 | 0.3 |
| miR-378* | 48 | 0.8 | 0.2 | -1.3 | -1.7 |
| miR-133 | 180 | 2.3 | -0.1 | 1.8 | -0.2 |
| miR-208 | 74 | -1.0 | -1.1 | 0.3 | 0.2 |
| miR-143 | 130 | 1.5 | 1.0 | -0.9 | -0.1 |
| miR-379 | 24 | 0.4 | -1.1 | 1.4 | 0.8 |
| miR-150 | 90 | -1.1 | -0.7 | 0.5 | 1.7 |
| miR-188 | 65 | -0.3 | -1.9 | 1.0 | 0.6 |
| miR-182 | 339 | -0.8 | 1.1 | -1.7 | -0.3 |
| miR-485-5p | 95 | 0.4 | 0.8 | 1.4 | 1.0 |
| miR-216 | 64 | -0.7 | -0.7 | 1.2 | 1.4 |
| miR-342 | 73 | 0.5 | 0.9 | -0.6 | -1.2 |
| miR-326 | 154 | 1.2 | -0.9 | 1.2 | 1.6 |
| miR-34b | 132 | -1.3 | 0.1 | 0.2 | -1.7 |
| miR-486 | 44 | 0.1 | 0.1 | 2.0 | 0.1 |
| miR-331 | 76 | -0.1 | -1.5 | 0.5 | 0.8 |
| miR-190 | 42 | -1.9 | -1.2 | 0.0 | -0.7 |
| miR-409-5p | 46 | 0.0 | -0.3 | 0.1 | 1.8 |
| miR-31 | 114 | 0.9 | 1.2 | 0.8 | -1.3 |
| miR-505 | 108 | -0.4 | -1.3 | -0.4 | -1.5 |
| miR-325 | 26 | -0.2 | -0.7 | -1.0 | -1.5 |
| miR-431 | 42 | 1.6 | -0.5 | -0.1 | 0.4 |
| miR-204/211 | 156 | -0.7 | -0.1 | -0.2 | -1.8 |
| miR-380-5p | 32 | -1.3 | -0.1 | 1.2 | 0.8 |
| miR-433-3p | 83 | -0.1 | 0.0 | 1.0 | -1.3 |
| miR-488 | 61 | -1.0 | 0.2 | -0.1 | -1.5 |
| miR-504 | 61 | -0.3 | 0.4 | 0.9 | -1.0 |
| miR-410 | 215 | -0.2 | -1.5 | 1.0 | -0.3 |
| miR-103/107 | 212 | 0.8 | -0.1 | 1.3 | 0.8 |
| miR-142-3p | 104 | -0.3 | -0.7 | 0.5 | -1.2 |
| miR-328 | 82 | -1.5 | -1.3 | 0.6 | 0.0 |
| miR-146 | 48 | -1.2 | -0.3 | 0.4 | 0.5 |
| miR-490 | 50 | 0.6 | 1.2 | 1.8 | 0.2 |
| miR-363 | 66 | 0.1 | 0.2 | -1.4 | -0.2 |
| miR-122 | 63 | 0.4 | 0.4 | 1.4 | 0.4 |
| miR-361 | 75 | 0.3 | 0.0 | 0.2 | -1.5 |
| miR-450 | 95 | -0.4 | -1.0 | 1.1 | -0.2 |
| miR-544 | 134 | 0.4 | -0.7 | -0.3 | -0.3 |
| miR-375 | 94 | 0.9 | -0.2 | -1.0 | -0.9 |
| miR-384 | 104 | -0.9 | 0.6 | 0.5 | -0.4 |
| miR-299-3p | 42 | -0.4 | 0.9 | -0.3 | -0.5 |
| miR-382 | 49 | -0.6 | -0.4 | 1.1 | 0.5 |
| miR-500 | 63 | 0.3 | 0.0 | 0.4 | 0.0 |
| miR-376c | 52 | -1.3 | -0.7 | -0.3 | -1.1 |
| miR-383 | 45 | -0.5 | 0.2 | -0.6 | 0.5 |
| miR-151 | 27 | -1.5 | -0.3 | -0.4 | -0.6 |
| miR-346 | 38 | 0.1 | -0.6 | -0.2 | -0.4 |

**MiR-124 transfection experiment (Wang & Wang, 2006) (GEO dataset GDS2657)**

This experiment is based on gene expression analysis of HepG2 cells over-expressing miR-124 for up to 120 hours.

We computed the miR-seed activity scores using MiRABELLE. The highest activity scores obtained from each sample are highlighted in red. Here again, we observe that the highest scores correspond to the two miR-seeds associated with miR-124.

Interestingly, this analysis gives an insight into the dynamics of miRNA activity following miR transfection. We observe a gradual increase in the activity for miR-124 associated seeds, with a peak occurring 32 hours after the transfection, followed by a gradual decrease of this activity. As in the previous dataset, the activity of other miR-seeds also gradually decreases, and this appeared to be a delayed effect of the increase in miR-124 activity. This observation could again be explained by the repression effect of miR-124 on DICER, causing a decrease in the maturation of all miRNAs.

| MIR-seed | Number of targets | miR-124, 16h | miR-124, 24h | miR-124, 32h | miR-124, 72h | miR-124, 120h | Contr. 16h | contr. 24h | contr. 32h | contr. 72h | Contr. 120h |
| --- | --- | --- | --- | --- | --- | --- | --- | --- | --- | --- | --- |
| miR-124.1 | 1085 | 12.5 | 25.1 | 25.5 | 19.4 | 2.7 | -17.1 | -18.0 | -18.4 | -13.0 | -9.3 |
| miR-124.2/506 | 1535 | 8.3 | 16.3 | 14.2 | 13.3 | -1.7 | -12.0 | -11.2 | -13.4 | -6.7 | -4.2 |
| miR-331 | 203 | 6.3 | -0.8 | -4.4 | 0.4 | -4.2 | -0.7 | 0.7 | 1.7 | 0.0 | -1.4 |
| miR-24 | 464 | 5.1 | 3.8 | -0.8 | -0.4 | -4.5 | -1.0 | 0.1 | -1.0 | 0.7 | -1.5 |
| miR-185 | 220 | 4.4 | -0.6 | -2.0 | -1.5 | -2.6 | 1.4 | 0.0 | -1.9 | -0.2 | 2.3 |
| miR-491 | 131 | 3.5 | 0.2 | -1.9 | -0.7 | -3.3 | 0.1 | -0.4 | 0.4 | -0.1 | 1.4 |
| miR-125/351 | 663 | 3.1 | 2.6 | -2.0 | 0.3 | -8.2 | -2.9 | 1.2 | 0.4 | 2.1 | 0.5 |
| miR-210 | 24 | 3.0 | 0.6 | -0.6 | -2.3 | -0.2 | 1.8 | 1.0 | -0.9 | -2.2 | -1.4 |
| miR-326 | 367 | 2.8 | 1.4 | -0.7 | -1.6 | -3.7 | -0.6 | 1.2 | 0.6 | 2.9 | -0.3 |
| miR-328 | 187 | 2.5 | 2.8 | 0.5 | -0.6 | -2.7 | 0.1 | -1.2 | -2.9 | 2.1 | 0.4 |
| miR-214 | 529 | 2.4 | 0.4 | -1.7 | 0.1 | -6.3 | -0.9 | 0.5 | 1.1 | 2.0 | 2.4 |
| miR-29 | 912 | 2.4 | 1.1 | -3.0 | -1.6 | -7.2 | -0.3 | -1.1 | -3.0 | 0.3 | 2.6 |
| miR-34/449 | 528 | 2.2 | -0.5 | -4.2 | -2.7 | -6.8 | 0.7 | 0.3 | 0.6 | 2.7 | 0.7 |
| miR-346 | 104 | 2.2 | 1.8 | -0.8 | 1.4 | -2.4 | -0.2 | 0.2 | -2.1 | 2.5 | 2.1 |
| miR-410 | 679 | 2.1 | 7.8 | 2.8 | -0.5 | -8.5 | 1.5 | 2.0 | -2.7 | 4.0 | -1.0 |
| miR-150 | 257 | 2.0 | 1.7 | -1.0 | -1.7 | -6.1 | 0.8 | 2.0 | 0.2 | 1.0 | -0.7 |
| miR-21 | 253 | 2.0 | 5.6 | 1.1 | 1.1 | -3.8 | 0.0 | -2.7 | -4.7 | 0.3 | 1.6 |
| miR-138 | 492 | 2.0 | 3.0 | -1.9 | -1.6 | -7.5 | 1.6 | 2.0 | 1.3 | 3.7 | -0.9 |
| miR-99/100 | 51 | 2.0 | 2.1 | 2.0 | 1.1 | -2.1 | -1.5 | -1.6 | -0.4 | -0.3 | -0.5 |
| miR-122 | 211 | 2.0 | 2.1 | -1.8 | 1.9 | -2.7 | 0.6 | -1.9 | -2.3 | 0.3 | 1.1 |
| miR-370 | 283 | 2.0 | 1.5 | -2.6 | 0.0 | -5.1 | -0.6 | 0.7 | 0.6 | 0.3 | 2.2 |
| miR-149 | 332 | 1.9 | 2.5 | -1.9 | 1.0 | -4.5 | -1.7 | -1.9 | -0.8 | -0.7 | 0.9 |
| miR-191 | 50 | 1.9 | 3.0 | 0.8 | 1.8 | -1.3 | -0.2 | -1.1 | -1.9 | -2.8 | -1.4 |
| miR-9 | 1109 | 1.8 | 5.9 | -0.4 | 1.4 | -9.3 | -0.2 | -2.4 | -3.8 | 2.7 | 1.9 |
| miR-126/126-3p | 21 | 1.8 | 0.2 | -2.3 | -0.7 | -2.9 | 0.7 | 0.3 | -0.2 | 2.6 | 2.4 |
| miR-339 | 192 | 1.8 | 2.5 | 0.3 | 1.0 | -2.8 | -1.3 | -0.7 | -0.6 | 1.8 | 1.2 |
| miR-28 | 183 | 1.8 | -0.7 | -2.6 | -1.5 | -2.5 | 1.3 | 2.2 | 0.4 | 0.8 | -1.1 |
| miR-218 | 768 | 1.7 | 5.4 | 1.3 | -0.3 | -8.6 | 1.0 | 0.7 | -2.5 | 2.5 | 1.5 |
| miR-143 | 304 | 1.7 | 4.7 | 1.8 | 1.4 | -3.9 | -1.6 | -0.2 | -2.7 | 1.0 | -1.7 |
| miR-342 | 243 | 1.5 | 4.2 | -1.1 | 2.0 | -2.5 | 0.7 | -0.4 | -3.2 | 2.3 | 2.4 |
| miR-409-5p | 138 | 1.2 | 1.7 | -0.4 | -1.8 | -5.4 | 0.4 | 1.6 | -1.7 | 4.4 | 0.4 |
| miR-135 | 576 | 1.0 | 5.4 | -1.0 | 0.9 | -7.1 | 1.4 | -0.7 | -2.3 | 2.2 | 2.4 |
| miR-153 | 571 | 0.7 | 6.6 | 0.5 | -1.4 | -9.0 | 1.6 | -0.7 | -2.0 | 1.8 | 1.0 |
| miR-34b | 356 | 0.6 | 2.0 | 0.4 | -1.2 | -5.6 | 1.1 | -0.2 | -2.6 | 1.5 | 0.2 |
| miR-380-5p | 69 | 0.6 | 2.0 | 4.0 | 1.2 | -2.4 | -0.3 | -2.1 | -0.5 | -1.2 | -0.4 |
| miR-500 | 183 | 0.6 | 2.5 | 0.5 | 1.2 | -2.4 | 1.7 | -0.3 | -3.7 | 1.9 | 1.8 |
| miR-504 | 162 | 0.5 | 1.0 | -0.7 | 0.7 | -1.6 | 1.4 | 0.8 | -1.8 | 1.8 | 1.0 |
| miR-142-3p | 264 | 0.5 | 4.2 | 1.4 | 0.3 | -5.8 | 1.6 | -0.2 | -3.2 | -0.1 | 2.0 |
| miR-18 | 217 | 0.3 | 1.2 | -1.7 | -3.4 | -5.3 | 1.2 | 1.1 | 0.0 | 2.3 | -0.1 |
| miR-431 | 120 | 0.2 | 2.7 | 1.0 | -2.9 | -3.9 | 1.4 | 0.8 | -0.6 | 0.7 | -0.5 |
| miR-17-5p/20/93.mr/106/519.d | 996 | 0.2 | 5.3 | -2.8 | -1.0 | -12.2 | 3.1 | -0.7 | -4.0 | 1.9 | 2.4 |
| miR-455 | 161 | 0.2 | 4.4 | 1.6 | 0.5 | -7.6 | 1.1 | -0.1 | -0.4 | 0.1 | 0.3 |
| miR-10 | 244 | 0.2 | 2.4 | -2.6 | -2.0 | -5.5 | 0.9 | 2.0 | -1.4 | 4.8 | 2.0 |
| miR-452 | 225 | 0.2 | 4.3 | 2.5 | 1.5 | -1.6 | -0.1 | -1.6 | -2.2 | -0.4 | -0.7 |
| miR-299-3p | 108 | 0.2 | 2.6 | -0.6 | 0.3 | -3.3 | -0.2 | 0.6 | -0.5 | -0.4 | 1.4 |
| miR-183 | 379 | 0.1 | 6.2 | 2.1 | 1.9 | -5.7 | -0.5 | 1.1 | -2.1 | 3.7 | 0.8 |
| miR-499 | 307 | 0.1 | 8.3 | 2.9 | 3.1 | -2.9 | 0.7 | -1.1 | -5.2 | 0.2 | 0.7 |
| miR-133 | 454 | 0.1 | 1.0 | -1.2 | 2.0 | -6.6 | -0.7 | 0.4 | -1.2 | 1.3 | 3.3 |
| miR-151 | 78 | 0.0 | 2.5 | 0.9 | 0.3 | -3.4 | -0.5 | -2.6 | -3.1 | -0.3 | 1.0 |
| miR-208 | 248 | 0.0 | 6.9 | 2.2 | 2.7 | -2.0 | 0.2 | -1.3 | -5.9 | 0.0 | 2.4 |
| miR-324-5p | 146 | 0.0 | 1.6 | -0.9 | 0.2 | -2.7 | 0.0 | -0.9 | -0.9 | 1.1 | 1.7 |
| miR-7 | 394 | -0.1 | 1.2 | -2.8 | -0.3 | -6.1 | 1.8 | 2.1 | -2.3 | 2.5 | 0.6 |
| miR-485-5p | 275 | -0.1 | -0.4 | -2.9 | -0.8 | -5.5 | 0.6 | 1.8 | 2.4 | 2.3 | 0.5 |
| miR-146 | 166 | -0.1 | 2.2 | 0.2 | -0.5 | -3.2 | 0.3 | -0.5 | -1.8 | 0.0 | 2.2 |
| miR-193 | 211 | -0.1 | 3.6 | -1.7 | -0.2 | -4.1 | 2.6 | 0.8 | -1.6 | 1.1 | 0.6 |
| miR-25/32/92/363/367 | 776 | -0.2 | 3.7 | -3.8 | -2.6 | -9.4 | 5.0 | 1.3 | -2.7 | 4.8 | 3.2 |
| miR-204/211 | 431 | -0.3 | 4.1 | -0.7 | 0.5 | -6.2 | -0.9 | -1.0 | -2.5 | 2.2 | 2.0 |
| miR-96 | 945 | -0.3 | 6.1 | 0.6 | 0.3 | -8.1 | 1.4 | -0.3 | -3.5 | 3.1 | 1.6 |
| miR-128 | 908 | -0.4 | 4.7 | -0.7 | -1.2 | -8.0 | 3.0 | 1.4 | -2.2 | 3.6 | 2.5 |
| miR-22 | 377 | -0.4 | 2.7 | -0.8 | -0.3 | -5.8 | 0.8 | 1.6 | -1.4 | 2.6 | 3.2 |
| miR-93.hd/291-3p/294/295/302/372/373/520 | 585 | -0.4 | 3.3 | -0.7 | -0.5 | -7.9 | 1.8 | -0.4 | -3.3 | 1.7 | 5.0 |
| miR-223 | 243 | -0.5 | 5.3 | 2.2 | 2.8 | -4.6 | -0.8 | -0.1 | -3.0 | 2.1 | 0.8 |
| miR-448 | 555 | -0.5 | 6.9 | 1.3 | -1.4 | -9.1 | 1.5 | -0.4 | -2.0 | 2.7 | 1.4 |
| miR-184 | 30 | -0.5 | -0.9 | -1.7 | 1.4 | 0.3 | -0.4 | 1.2 | 0.1 | -0.7 | 0.9 |
| miR-155 | 317 | -0.5 | 5.1 | 1.2 | 1.0 | -4.7 | 1.0 | -0.6 | -3.7 | 1.9 | 3.3 |
| miR-134 | 147 | -0.7 | 3.2 | 0.0 | -0.6 | -4.4 | 2.8 | 2.1 | -2.0 | 1.9 | 1.4 |
| miR-369-3p | 548 | -0.7 | 4.2 | 0.4 | -2.9 | -7.9 | 3.6 | 2.4 | -0.2 | 3.3 | -0.9 |
| miR-299-5p | 313 | -0.7 | 5.0 | 1.9 | 0.7 | -7.0 | -2.3 | -1.6 | -1.9 | 2.4 | 2.5 |
| miR-137 | 628 | -0.7 | 4.1 | -0.2 | -0.8 | -6.8 | 1.3 | 0.9 | -1.5 | 2.0 | 1.2 |
| miR-27 | 1023 | -0.7 | 5.3 | -0.8 | -1.4 | -8.7 | 2.8 | 0.9 | -2.6 | 2.6 | 2.2 |
| miR-362 | 157 | -0.7 | 2.7 | -0.1 | 0.7 | -2.8 | 0.4 | -0.3 | -3.0 | -0.6 | 3.6 |
| miR-148/152 | 587 | -0.8 | 2.5 | -2.4 | -2.1 | -8.5 | 3.0 | 1.7 | -1.2 | 3.8 | 3.5 |
| miR-363 | 198 | -0.8 | 4.1 | 1.7 | 1.4 | -3.5 | 1.1 | 0.5 | -4.0 | 1.0 | 1.1 |
| miR-338 | 297 | -0.8 | 2.2 | 0.7 | 0.8 | -5.1 | -0.2 | 1.0 | -0.5 | 1.7 | 2.5 |
| miR-15/16/195/424/497 | 1017 | -0.9 | 2.4 | -3.7 | -1.4 | -10.3 | 2.6 | -0.1 | -1.5 | 4.8 | 3.9 |
| miR-219 | 320 | -0.9 | 3.5 | -1.1 | -1.7 | -5.4 | 0.9 | 0.3 | -2.7 | 2.2 | 0.3 |
| miR-335 | 226 | -1.0 | 3.0 | -0.7 | -0.4 | -3.6 | -1.0 | -0.6 | -2.1 | 0.7 | 1.3 |
| miR-31 | 273 | -1.0 | 1.4 | -1.2 | -1.1 | -4.0 | -0.9 | 1.0 | -1.6 | 1.7 | 1.1 |
| miR-375 | 273 | -1.0 | 2.2 | -0.6 | -1.0 | -6.4 | 2.4 | 1.9 | -0.5 | 3.4 | -1.1 |
| miR-19 | 938 | -1.0 | 5.3 | -1.3 | -3.8 | -12.6 | 3.7 | 0.0 | -2.0 | 3.4 | 1.9 |
| miR-221/222 | 320 | -1.1 | 3.8 | 0.3 | -0.8 | -6.2 | 2.8 | 0.6 | 0.3 | 1.5 | 1.2 |
| miR-421 | 131 | -1.2 | 2.3 | 0.3 | -0.3 | -3.4 | 0.4 | -0.7 | -0.4 | 2.1 | 2.2 |
| miR-539 | 506 | -1.3 | 4.4 | 1.0 | -0.8 | -5.5 | 0.0 | -0.3 | -1.7 | 0.0 | 1.2 |
| miR-374 | 550 | -1.3 | 5.0 | 0.5 | -1.4 | -7.2 | 3.9 | 1.9 | -2.1 | 2.7 | 0.2 |
| miR-182 | 875 | -1.3 | 6.0 | -1.5 | -1.9 | -9.4 | 2.5 | 0.5 | -3.1 | 4.1 | 2.4 |
| miR-199 | 407 | -1.3 | 2.6 | -2.4 | -0.9 | -6.8 | 0.1 | 0.3 | -2.3 | 3.6 | 3.8 |
| miR-542-3p | 275 | -1.3 | 3.1 | 0.8 | -0.1 | -3.7 | 1.9 | 0.4 | -1.3 | 4.0 | -0.9 |
| miR-1/206 | 715 | -1.4 | 6.1 | 1.0 | 1.6 | -7.8 | -1.8 | -1.1 | -3.9 | 3.0 | 1.2 |
| let-7/98 | 811 | -1.4 | 1.9 | -1.9 | -1.6 | -8.4 | -1.1 | 0.2 | 1.1 | 2.9 | 0.3 |
| miR-23 | 866 | -1.4 | 9.3 | 2.2 | 1.9 | -7.9 | 0.6 | -1.7 | -6.6 | 3.2 | 2.7 |
| miR-365 | 218 | -1.5 | 3.0 | -0.9 | -1.4 | -7.2 | 1.5 | 1.0 | 0.6 | 2.0 | 3.5 |
| miR-33 | 309 | -1.6 | 4.7 | 1.6 | -1.0 | -5.9 | 1.2 | -0.2 | -0.8 | 1.1 | 1.1 |
| miR-485-3p | 270 | -1.7 | 2.1 | -1.7 | -0.4 | -3.7 | -0.2 | 0.2 | -2.8 | 3.4 | 2.1 |
| miR-377 | 378 | -1.8 | 2.2 | -2.1 | -1.1 | -5.7 | 0.2 | 1.8 | 0.0 | 4.5 | 3.6 |
| miR-196 | 235 | -1.8 | 3.0 | 0.4 | 1.0 | -2.2 | 0.9 | -0.9 | -1.3 | -1.5 | 0.6 |
| miR-505 | 391 | -1.8 | 3.8 | 0.0 | 0.1 | -7.7 | 2.3 | 2.7 | -1.4 | 3.7 | 3.0 |
| miR-141/200a | 570 | -1.9 | 4.5 | -1.2 | -0.1 | -7.7 | 3.7 | 1.1 | -2.0 | 5.0 | 2.2 |
| miR-503 | 398 | -1.9 | 2.2 | -0.9 | -0.1 | -5.5 | 1.1 | 0.7 | -0.7 | 4.4 | 2.3 |
| miR-379 | 82 | -1.9 | 1.4 | 1.2 | 0.4 | -3.9 | -2.0 | -0.2 | -0.6 | 2.9 | 3.1 |
| miR-140 | 252 | -2.0 | 1.5 | -1.5 | -0.4 | -4.6 | 0.1 | -0.7 | -2.3 | -0.1 | 0.9 |
| miR-130/301 | 714 | -2.0 | 4.9 | -1.7 | -3.0 | -12.1 | 3.6 | 1.3 | -2.5 | 4.2 | 3.3 |
| miR-451 | 28 | -2.0 | -0.4 | -0.9 | -2.0 | -3.6 | 1.2 | 0.5 | 0.5 | 1.2 | 2.0 |
| miR-129-5p | 470 | -2.1 | 4.0 | 0.1 | -0.2 | -7.9 | -0.1 | 2.0 | -2.8 | 2.5 | 4.7 |
| miR-450 | 274 | -2.1 | 3.4 | 1.0 | -1.1 | -6.3 | 0.4 | 1.7 | -2.3 | 2.9 | 3.1 |
| miR-205 | 345 | -2.1 | 2.9 | 0.7 | 1.6 | -7.1 | -0.3 | -2.7 | -2.0 | 0.5 | 3.1 |
| miR-383 | 105 | -2.1 | 1.3 | -0.3 | 0.9 | -2.2 | -0.5 | 0.7 | 0.2 | 2.0 | 0.8 |
| miR-224 | 294 | -2.2 | 5.0 | -0.8 | 0.0 | -5.1 | 1.3 | 0.7 | -4.2 | 4.0 | 4.3 |
| miR-488 | 182 | -2.2 | 1.7 | -0.2 | 0.1 | -4.3 | 0.2 | -1.0 | -0.5 | 0.7 | 2.3 |
| miR-188 | 169 | -2.2 | 1.9 | -1.2 | 0.0 | -4.0 | 2.1 | 0.4 | -1.6 | 2.5 | 4.3 |
| miR-378 | 434 | -2.2 | 2.4 | 0.2 | 1.7 | -5.4 | -0.4 | -0.2 | -2.9 | 2.2 | 2.6 |
| miR-324-3p | 233 | -2.2 | 0.9 | -2.5 | -0.7 | -5.0 | 1.4 | 1.8 | 0.4 | 4.4 | 3.0 |
| miR-103/107 | 555 | -2.2 | 2.3 | -1.8 | 0.9 | -5.6 | 0.9 | -1.3 | -2.2 | 3.3 | 5.8 |
| miR-30-5p | 1200 | -2.3 | 8.0 | 0.1 | -1.0 | -10.0 | 0.5 | -2.7 | -5.7 | 2.7 | 4.1 |
| miR-378* | 136 | -2.3 | 0.6 | 0.4 | -0.7 | -2.0 | -0.7 | 0.7 | -0.9 | 1.9 | 2.2 |
| miR-145 | 566 | -2.4 | 6.2 | 0.5 | 0.3 | -9.2 | -0.5 | -0.8 | -2.6 | 3.2 | 0.7 |
| miR-376c | 218 | -2.4 | 3.1 | 0.1 | -1.6 | -6.3 | 2.9 | 2.2 | -2.1 | 2.0 | 1.7 |
| miR-216 | 200 | -2.5 | 1.5 | -1.0 | -0.1 | -5.8 | 1.4 | 0.6 | -0.7 | 3.3 | 4.6 |
| miR-486 | 142 | -2.5 | 2.6 | 0.0 | 0.6 | -4.2 | -0.6 | 0.3 | 0.2 | 4.2 | 3.0 |
| miR-329 | 310 | -2.6 | 2.2 | -0.4 | 1.1 | -5.9 | 1.0 | 0.4 | -2.1 | 3.8 | 2.8 |
| miR-132/212 | 379 | -2.7 | 3.4 | -0.3 | 1.0 | -6.0 | -0.8 | 1.6 | -1.4 | 3.5 | 3.6 |
| miR-136 | 157 | -2.8 | 0.2 | -0.3 | 0.5 | -2.8 | 0.3 | -0.3 | -0.8 | -0.2 | 1.6 |
| miR-192/215 | 135 | -2.8 | 1.6 | 0.8 | 0.5 | -1.5 | 1.5 | -0.1 | -3.3 | -0.8 | 1.9 |
| miR-194 | 351 | -2.9 | 4.1 | 1.2 | 2.7 | -4.9 | -1.8 | 0.0 | -3.1 | 4.8 | 4.3 |
| miR-490 | 137 | -2.9 | 1.6 | -0.3 | 1.4 | -2.9 | 0.4 | -0.7 | 0.0 | 1.2 | 2.4 |
| miR-376 | 209 | -3.0 | 2.9 | 1.5 | -0.4 | -5.4 | 0.6 | 1.0 | 0.5 | 2.8 | -0.5 |
| miR-433-3p | 253 | -3.2 | 3.6 | 0.6 | 2.9 | -4.3 | -1.1 | -0.9 | -2.9 | 1.8 | 3.3 |
| miR-330 | 549 | -3.2 | 3.9 | 0.5 | -0.2 | -6.3 | 1.2 | 1.0 | -2.4 | 3.3 | 2.6 |
| miR-323 | 720 | -3.2 | 8.5 | 3.0 | 2.4 | -7.2 | 0.0 | -2.5 | -6.5 | 1.1 | 3.8 |
| miR-24* | 99 | -3.3 | 3.3 | 0.3 | -1.0 | -7.6 | 3.0 | 1.6 | -0.9 | 2.7 | 0.5 |
| miR-144 | 678 | -3.3 | 6.5 | 0.4 | -0.9 | -9.3 | 2.9 | 2.0 | -3.4 | 6.2 | 3.9 |
| miR-181 | 982 | -3.3 | 8.0 | -0.8 | -0.7 | -11.5 | 2.5 | 0.8 | -3.8 | 4.5 | 3.9 |
| miR-217 | 268 | -3.5 | 4.0 | -1.8 | 0.2 | -7.0 | 2.9 | 1.5 | -1.8 | 4.4 | 3.1 |
| miR-409-3p | 312 | -3.5 | 5.2 | 2.5 | 0.9 | -6.1 | 0.3 | 1.1 | -1.2 | 1.5 | -1.5 |
| miR-325 | 81 | -3.7 | 1.8 | -2.5 | -0.8 | -3.1 | 2.1 | 2.0 | -1.1 | 3.9 | 4.9 |
| miR-544 | 397 | -3.7 | 3.6 | 0.6 | 2.1 | -4.2 | 0.2 | 0.9 | -3.1 | 2.1 | 3.1 |
| miR-493-5p | 695 | -3.7 | 5.6 | -1.3 | 1.6 | -9.0 | 0.4 | 0.7 | -2.2 | 5.1 | 3.7 |
| miR-139 | 331 | -3.7 | 2.0 | -1.3 | -0.5 | -6.4 | 1.4 | 0.3 | -1.5 | 2.6 | 3.4 |
| miR-190 | 137 | -3.7 | 2.0 | 0.7 | 0.5 | -1.7 | -1.1 | -0.4 | -2.6 | 1.9 | 3.2 |
| miR-382 | 168 | -3.7 | 2.3 | 0.2 | 3.0 | -3.0 | -1.1 | -0.5 | -3.0 | 1.6 | 4.4 |
| miR-384 | 271 | -3.8 | 3.4 | 0.5 | -0.9 | -5.5 | 0.9 | 0.4 | -0.9 | 3.0 | 2.4 |
| miR-381 | 868 | -3.8 | 8.3 | 2.8 | 2.9 | -8.9 | 0.3 | 0.1 | -4.2 | 5.0 | 2.1 |
| miR-200b/429 | 840 | -3.8 | 6.8 | 0.8 | 0.6 | -11.1 | 1.1 | 1.7 | -2.4 | 5.9 | 2.6 |
| miR-495 | 879 | -3.8 | 3.1 | -1.1 | -0.2 | -9.7 | 2.6 | 1.4 | -3.0 | 4.0 | 4.5 |
| miR-142-5p | 621 | -4.0 | 4.5 | 0.7 | -0.2 | -7.3 | 0.9 | 0.2 | -3.6 | 2.3 | 3.2 |
| miR-101 | 708 | -4.2 | 6.0 | -0.6 | -1.3 | -10.2 | 3.5 | 1.8 | -2.5 | 6.3 | 5.2 |
| miR-361 | 184 | -4.2 | 2.3 | -2.1 | -1.1 | -4.4 | 1.9 | 1.5 | -1.0 | 3.2 | 3.4 |
| miR-30-3p | 425 | -4.3 | 5.8 | 1.4 | 0.8 | -6.5 | 1.1 | 1.9 | -1.2 | 3.2 | 1.6 |
| miR-543 | 624 | -4.5 | 5.4 | 0.1 | 1.4 | -8.1 | 1.4 | 0.6 | -2.8 | 3.4 | 4.5 |
| miR-26 | 811 | -4.6 | 6.1 | 0.0 | 0.1 | -6.9 | 1.0 | -3.0 | -5.5 | 3.6 | 3.7 |
| miR-496 | 825 | -4.8 | 10.6 | 5.5 | 4.4 | -9.2 | 0.1 | 0.2 | -6.4 | 3.3 | 2.9 |
| miR-494 | 441 | -4.9 | 3.9 | 0.3 | 2.1 | -5.0 | 0.3 | 0.9 | -4.0 | 2.8 | 4.2 |
| miR-320 | 685 | -5.0 | 4.9 | -1.6 | 0.6 | -7.9 | 1.4 | 0.4 | -3.5 | 3.5 | 4.5 |
| miR-203.1 | 594 | -5.2 | 5.4 | 0.7 | 0.1 | -6.2 | 1.2 | 0.6 | -4.2 | 3.3 | 4.5 |
| miR-186 | 676 | -5.6 | 6.8 | 1.8 | 1.2 | -10.8 | 0.7 | 0.1 | -3.4 | 5.6 | 3.1 |

**Antagomir (Krutzfeldt et al., 2005) experiment silencing endogenous miR-122 (GSE3425)**

AntagomiRs are a novel class of chemically-engineered oligonucleotides. Krutzfeldt et al.(Krutzfeldt et al., 2005) studied the biological significance of silencing miR-122 in the liver of mice at the mRNA level using an antagomiR. They injected mice on three subsequent days with antagomir-122 or scrambled control (mm-antagomir-122), respectively; 80mg/kg bodyweight/day. 24 hrs after the last treatment, mice were sacrificed and total RNA isolated from the livers.

The highest activity scores obtained from each sample are highlighted in red. Here again, we observe that the most significant change of activity occurs for miR-122, with a very high decrease of miR-122 observable for miR-122.

| MIR-seed | Number of targets | Control | Treated |
| --- | --- | --- | --- |
| miR-122 | 179 | 8.228087 | -8.22809 |
| miR-184 | 28 | 3.43445 | -3.43445 |
| miR-143 | 272 | 3.146685 | -3.14668 |
| miR-218 | 664 | 2.25998 | -2.25998 |
| let-7/98 | 717 | 2.252678 | -2.25268 |
| miR-335 | 200 | 2.156273 | -2.15627 |
| miR-15/16/195/424/497 | 814 | 2.118086 | -2.11809 |
| miR-324-3p | 210 | 2.035811 | -2.03581 |
| miR-193 | 170 | 1.972605 | -1.97261 |
| miR-124.2/506 | 1100 | 1.969098 | -1.9691 |
| miR-204/211 | 418 | 1.813664 | -1.81366 |
| miR-485-5p | 226 | 1.620327 | -1.62033 |
| miR-34/449 | 484 | 1.533428 | -1.53343 |
| miR-22 | 327 | 1.467145 | -1.46714 |
| miR-362 | 141 | 1.338599 | -1.3386 |
| miR-103/107 | 509 | 1.324602 | -1.3246 |
| miR-323 | 460 | 1.315091 | -1.31509 |
| miR-328 | 157 | 1.199407 | -1.19941 |
| miR-433-3p | 242 | 1.145946 | -1.14595 |
| miR-346 | 98 | 1.097085 | -1.09708 |
| miR-448 | 495 | 1.050698 | -1.0507 |
| miR-223 | 236 | 1.050319 | -1.05032 |
| miR-331 | 147 | 1.049676 | -1.04968 |
| miR-503 | 338 | 0.968322 | -0.96832 |
| miR-214 | 433 | 0.93948 | -0.93948 |
| miR-451 | 29 | 0.935654 | -0.93565 |
| miR-216 | 175 | 0.877186 | -0.87719 |
| miR-125/351 | 575 | 0.852783 | -0.85278 |
| miR-153 | 393 | 0.731135 | -0.73113 |
| miR-31 | 288 | 0.704178 | -0.70418 |
| miR-491 | 114 | 0.703552 | -0.70355 |
| miR-148/152 | 503 | 0.699106 | -0.69911 |
| miR-34b | 295 | 0.692963 | -0.69296 |
| miR-29 | 776 | 0.685604 | -0.6856 |
| miR-455 | 124 | 0.654251 | -0.65425 |
| miR-21 | 240 | 0.647528 | -0.64753 |
| miR-17-5p/20/93.mr/106/519.d | 860 | 0.560503 | -0.5605 |
| miR-132/212 | 332 | 0.549772 | -0.54977 |
| miR-128 | 603 | 0.539418 | -0.53942 |
| miR-361 | 185 | 0.508543 | -0.50854 |
| miR-365 | 207 | 0.508543 | -0.50854 |
| miR-192/215 | 107 | 0.496638 | -0.49664 |
| miR-130/301 | 607 | 0.491969 | -0.49197 |
| miR-183 | 355 | 0.417653 | -0.41765 |
| miR-383 | 103 | 0.364611 | -0.36461 |
| miR-28 | 147 | 0.345904 | -0.3459 |
| miR-93.hd/291-3p/294/295/302/372/373/520 | 526 | 0.270282 | -0.27028 |
| miR-382 | 139 | 0.24359 | -0.24359 |
| miR-504 | 154 | 0.242282 | -0.24228 |
| miR-146 | 149 | 0.239676 | -0.23968 |
| miR-23 | 750 | 0.239315 | -0.23931 |
| miR-19 | 835 | 0.231815 | -0.23181 |
| miR-342 | 189 | 0.164012 | -0.16401 |
| miR-486 | 151 | 0.149629 | -0.14963 |
| miR-136 | 140 | 0.148255 | -0.14825 |
| miR-338 | 275 | 0.117615 | -0.11762 |
| miR-217 | 246 | 0.105333 | -0.10533 |
| miR-384 | 234 | 0.095491 | -0.09549 |
| miR-185 | 201 | 0.079489 | -0.07949 |
| miR-200b/429 | 772 | 0.074537 | -0.07454 |
| miR-339 | 179 | 0.057301 | -0.0573 |
| miR-182 | 566 | 0.044825 | -0.04482 |
| miR-141/200a | 528 | 0.018844 | -0.01884 |
| miR-133 | 433 | 0.015537 | -0.01554 |
| miR-324-5p | 121 | -0.00225 | 0.002247 |
| miR-24 | 425 | -0.00803 | 0.00803 |
| miR-24* | 78 | -0.02512 | 0.025118 |
| miR-129-5p | 366 | -0.03218 | 0.032181 |
| miR-142-3p | 262 | -0.08709 | 0.087091 |
| miR-376 | 165 | -0.12768 | 0.127683 |
| miR-196 | 189 | -0.13149 | 0.131487 |
| miR-495 | 752 | -0.15345 | 0.15345 |
| miR-299-3p | 89 | -0.16802 | 0.168018 |
| miR-181 | 809 | -0.18774 | 0.187736 |
| miR-30-3p | 387 | -0.21023 | 0.210228 |
| miR-96 | 859 | -0.22551 | 0.225509 |
| miR-124.1 | 999 | -0.2331 | 0.233098 |
| miR-7 | 314 | -0.23458 | 0.234581 |
| miR-134 | 138 | -0.26985 | 0.26985 |
| miR-25/32/92/363/367 | 656 | -0.27726 | 0.277262 |
| miR-9 | 998 | -0.28283 | 0.28283 |
| miR-378* | 118 | -0.294 | 0.294004 |
| miR-150 | 227 | -0.33094 | 0.330943 |
| miR-101 | 429 | -0.33954 | 0.339545 |
| miR-140 | 206 | -0.36578 | 0.365785 |
| miR-219 | 283 | -0.36689 | 0.366886 |
| miR-139 | 304 | -0.39306 | 0.39306 |
| miR-18 | 168 | -0.40575 | 0.405755 |
| miR-138 | 422 | -0.42142 | 0.42142 |
| miR-377 | 332 | -0.43571 | 0.435712 |
| miR-145 | 490 | -0.43749 | 0.437489 |
| miR-320 | 601 | -0.4375 | 0.437505 |
| miR-190 | 119 | -0.43797 | 0.437968 |
| miR-329 | 283 | -0.4576 | 0.457599 |
| miR-137 | 545 | -0.46205 | 0.462052 |
| miR-505 | 323 | -0.47891 | 0.478906 |
| miR-544 | 324 | -0.48394 | 0.483939 |
| miR-191 | 54 | -0.50696 | 0.506961 |
| miR-450 | 61 | -0.51142 | 0.511417 |
| miR-325 | 57 | -0.52805 | 0.52805 |
| miR-135 | 452 | -0.53674 | 0.536742 |
| miR-379 | 87 | -0.54454 | 0.544544 |
| miR-410 | 599 | -0.55163 | 0.551627 |
| miR-326 | 292 | -0.68466 | 0.684662 |
| miR-30-5p | 1084 | -0.68729 | 0.687286 |
| miR-409-5p | 123 | -0.69155 | 0.691547 |
| miR-10 | 211 | -0.69614 | 0.696137 |
| miR-224 | 252 | -0.82138 | 0.821384 |
| miR-33 | 284 | -0.82622 | 0.82622 |
| miR-496 | 735 | -0.87214 | 0.872139 |
| miR-203.1 | 574 | -0.88585 | 0.885852 |
| miR-221/222 | 281 | -0.88958 | 0.889584 |
| miR-378 | 363 | -0.91066 | 0.910659 |
| miR-374 | 453 | -0.93234 | 0.932338 |
| miR-151 | 79 | -0.93516 | 0.935164 |
| miR-485-3p | 245 | -0.93882 | 0.938817 |
| miR-199 | 350 | -0.94479 | 0.944793 |
| miR-149 | 318 | -0.95957 | 0.959566 |
| miR-370 | 260 | -0.96941 | 0.96941 |
| miR-488 | 158 | -0.98141 | 0.981412 |
| miR-99/100 | 37 | -1.05687 | 1.056872 |
| miR-363 | 148 | -1.09687 | 1.096866 |
| miR-188 | 158 | -1.13368 | 1.133682 |
| miR-490 | 135 | -1.17841 | 1.178412 |
| miR-431 | 126 | -1.23101 | 1.231013 |
| miR-409-3p | 260 | -1.23425 | 1.23425 |
| miR-500 | 158 | -1.24259 | 1.24259 |
| miR-194 | 326 | -1.27507 | 1.275066 |
| miR-421 | 104 | -1.29505 | 1.295046 |
| miR-27 | 974 | -1.29746 | 1.297461 |
| miR-155 | 291 | -1.41073 | 1.410734 |
| miR-375 | 233 | -1.41215 | 1.412153 |
| miR-205 | 354 | -1.43719 | 1.43719 |
| miR-543 | 577 | -1.45511 | 1.455111 |
| miR-144 | 633 | -1.50576 | 1.505755 |
| miR-494 | 379 | -1.51851 | 1.518515 |
| miR-493-5p | 598 | -1.55053 | 1.550533 |
| miR-380-5p | 71 | -1.59457 | 1.594567 |
| miR-542-3p | 238 | -1.73128 | 1.731278 |
| miR-1/206 | 676 | -1.76169 | 1.761689 |
| miR-369-3p | 338 | -1.77539 | 1.775387 |
| miR-186 | 624 | -1.78245 | 1.782452 |
| miR-539 | 462 | -1.79765 | 1.797655 |
| miR-208 | 72 | -1.867 | 1.867003 |
| miR-26 | 736 | -1.90949 | 1.909491 |
| miR-499 | 272 | -1.91583 | 1.915833 |
| miR-376c | 192 | -1.96517 | 1.965165 |
| miR-330 | 486 | -2.43158 | 2.431575 |
| miR-381 | 756 | -2.57274 | 2.57274 |
| miR-452 | 205 | -2.66211 | 2.662114 |
| miR-142-5p | 581 | -2.84767 | 2.847673 |
| miR-299-5p | 272 | -3.00682 | 3.006818 |

**Analysis of a prostate cancer dataset (GSE3325)**

We verified that the miRNA activity measured by MiRABELLE allows to distinguish the different stages of carcinogenesis in other cancer types. Among others, we investigated a dataset of prostate cancer published by Varambally et al. (Varambally et al., 2005).

This dataset included 13 individual benign prostate hyperplasia (BPH), primary and metastatic prostate cancer samples and 6 pooled samples from benign, primary or metastatic prostate cancer tissues.

We found that miRNA activity calculated by miRABELLE allows distinguishing the different stages of carcinogenesis. In this type of cancer, miR activity scores are increased both at the primary carcinoma and the metastatic stages, compared to BPH. Below are the results of hierarchical clustering performed using Cluster 3.0 (Eisen et al., 1998) on the miR-seed activity scores computed on this dataset.


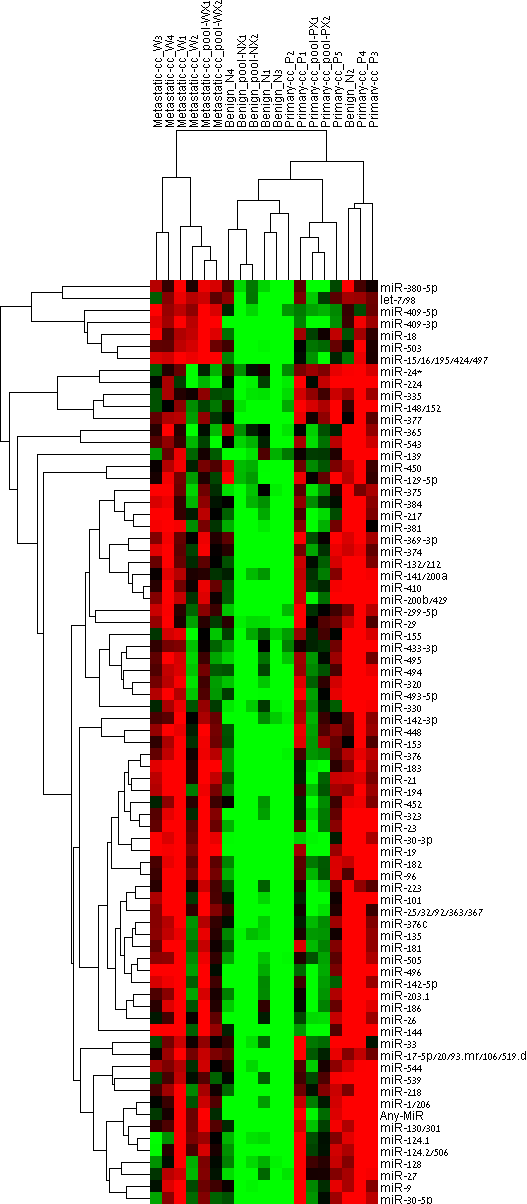


**Analysis of a leukemia dataset (GSE2466)**

We also investigated miRNA activity in a large dataset of B-cell chronic lymphocytic leukemia (B-CLL) published by Haslinger et al.J Clin Oncol 2004.. In this dataset, gene expression profiling was used to profile the five most frequent genomic aberrations in B-CLL, namely deletions affecting chromosome bands 13q14, 11q22-q23, 17p13 and 6q21, and gains of genomic material affecting chromosome band 12q13. This dataset includes 100 B-CLL samples, and 11 control samples.

Similarly, we found that miRNA activity calculated by MiRABELLE allowed to clearly distinguish between most of the normal samples (9 of 11) and the tumors. Interestingly, in this cancer, the miRNA activity appeared to be diminished in many tumors, and this is apparently consistent with the observation that miRNA genes in the affected chromosome bands are frequently deleted in B‑CLL (Calin et al., 2004). Below are the results of hierarchical clustering, performed using Cluster 3.0 on the miR-seed activity scores computed on this dataset.


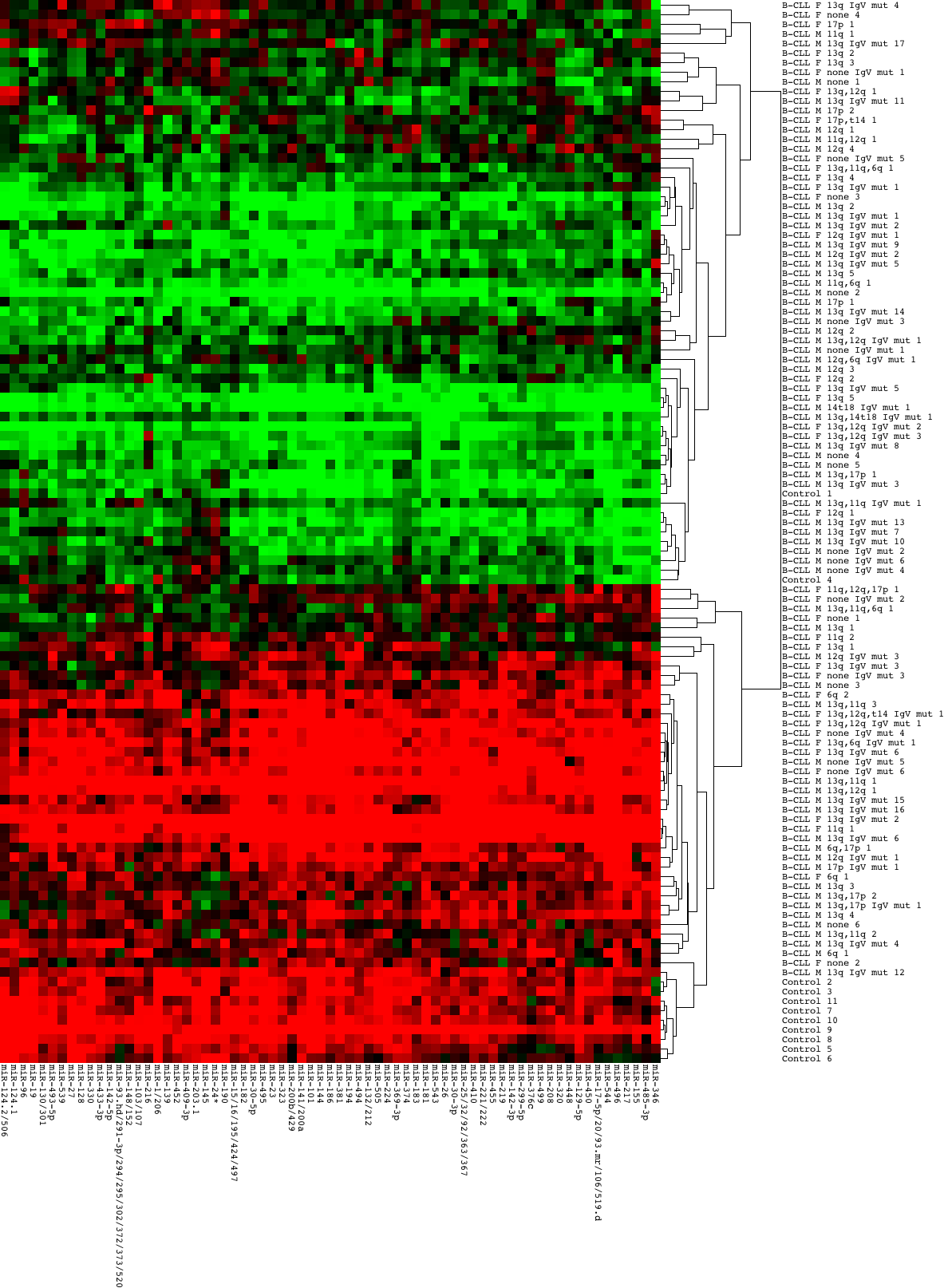


**References**

Lim LP, Lau NC, Garrett-Engele P, Grimson A, Schelter JM, Castle J, Bartel DP, Linsley PS, Johnson JM (2005) Microarray analysis shows that some microRNAs downregulate large numbers of target mRNAs. *Nature* **433:** 769-773.

Wang X, Wang X (2006) Systematic identification of microRNA functions by combining target prediction and expression profiling. *Nucleic Acids Res* **34:** 1646-1652.

Krutzfeldt J, Rajewsky N, Braich R, Rajeev KG, Tuschl T, Manoharan M, Stoffel M (2005) Silencing of microRNAs in vivo with 'antagomirs'. *Nature* **438:** 685-689.

Varambally S, Yu J, Laxman B, Rhodes DR, Mehra R, Tomlins SA, Shah RB, Chandran U, Monzon FA, Becich MJ, Wei JT, Pienta KJ, Ghosh D, Rubin MA, Chinnaiyan AM (2005) Integrative genomic and proteomic analysis of prostate cancer reveals signatures of metastatic progression. *Cancer Cell* **8:** 393-406.

Eisen MB, Spellman PT, Brown PO, Botstein D (1998) Cluster analysis and display of genome-wide expression patterns. *Proc Natl Acad Sci U S A* **95:** 14863-14868.

Calin GA, Sevignani C, Dumitru CD, Hyslop T, Noch E, Yendamuri S, Shimizu M, Rattan S, Bullrich F, Negrini M, Croce CM (2004) Human microRNA genes are frequently located at fragile sites and genomic regions involved in cancers. *Proc Natl Acad Sci U S A* **101:** 2999-3004.
