# Supplementary material for: Increased MicroRNA Activity in Human Cancers
Source: PLoS One. 2009 Jun 25;4(6):e6045. doi: 10.1371/journal.pone.0006045 (PMC2698213; doi:10.1371/journal.pone.0006045)
Supplement: Table S1 — MiR-seed activity matrix - Papillary Thyroid Carcinoma (0.03 MB PDF) [file pone.0006045.s002.pdf]

MIR-seed	nb targets	median diff	Normal N-PTC1	Normal N-PTC2	Normal N-PTC3	Normal N-PTC4	Normal N-PTC5	Normal N-PTC6	Normal N-PTC7	Normal N-PTC8	Normal N-PTC9	Papillary-carcinoma T-PTC1	Papillary-carcinoma T-PTC2	Papillary-carcinoma T-PTC3	Papillary-carcinoma T-PTC4	Papillary-carcinoma T-PTC5	Papillary-carcinoma T-PTC6	Papillary-carcinoma T-PTC7	Papillary-carcinoma T-PTC8	Papillary-carcinoma T-PTC9
miR-381	868	16.83	-7.34	-10.88	-14.54	1.62	-11.45	-17.27	-4.96	-8.77	2.58	-11.11	-10.21	-5.70	16.21	4.01	8.06	11.84	24.13	8.09
miR-496	825	14.94	-7.88	-12.25	-15.44	0.93	-10.65	-16.75	-7.31	-8.20	1.30	-10.71	-6.99	-4.89	16.84	5.81	6.74	11.25	23.94	8.63
miR-181	982	14.36	-6.30	-9.05	-9.51	3.20	-9.50	-14.16	-3.05	-8.20	2.00	-10.20	-7.67	-5.02	15.57	1.63	6.15	8.72	18.54	8.22
miR-495	879	14.23	-5.79	-8.71	-9.51	1.89	-10.15	-14.26	-5.41	-8.53	0.74	-9.53	-6.13	-5.88	13.76	2.59	5.70	8.72	20.69	7.84
miR-323	720	14.22	-7.54	-8.81	-10.19	0.45	-9.15	-11.46	-4.69	-6.14	1.53	-7.78	-6.45	-2.82	11.58	4.07	6.67	9.69	15.61	7.10
miR-30-3p	425	13.63	-5.63	-9.57	-9.85	0.17	-7.89	-11.39	-4.44	-7.52	1.39	-10.07	-3.02	-1.36	9.18	6.65	6.11	9.08	15.33	5.91
miR-493-5p	695	13.36	-4.69	-8.29	-9.34	1.97	-11.12	-11.43	-4.32	-7.81	1.90	-7.82	-5.05	-4.08	12.08	1.50	5.60	5.55	17.81	6.81
miR-23	866	13.15	-7.12	-9.00	-9.74	1.81	-9.76	-12.89	-4.26	-6.72	2.70	-8.82	-7.35	-2.41	12.18	3.05	6.03	9.51	16.50	7.74
miR-142-5p	621	13.06	-7.02	-7.95	-8.62	0.21	-8.35	-13.95	-3.03	-7.86	4.28	-11.22	-5.87	-3.30	10.89	2.93	5.20	8.19	18.24	6.93
miR-186	676	13.00	-5.70	-8.09	-10.75	2.46	-7.76	-11.93	-3.48	-7.61	1.91	-8.57	-8.46	-6.74	13.06	2.13	5.39	8.58	18.83	7.89
miR-320	685	12.97	-7.90	-8.02	-7.95	3.56	-9.24	-14.12	-3.12	-7.62	1.76	-8.56	-7.34	-5.07	13.66	2.06	5.06	9.38	16.53	7.81
miR-182	875	12.61	-6.05	-6.42	-7.57	0.83	-8.53	-13.35	-5.34	-6.67	4.39	-10.03	-4.93	-1.54	9.36	3.57	6.29	6.19	15.67	6.42
miR-194	351	12.28	-3.15	-7.25	-7.89	3.07	-8.18	-10.69	-3.70	-6.87	-0.79	-5.44	-5.23	-2.82	10.28	2.81	5.48	6.59	12.42	5.40
miR-93.hd/291-3p/294/295/302/3	585	12.15	-7.23	-5.83	-6.57	2.26	-8.42	-12.79	-3.32	-5.77	3.09	-7.14	-5.19	-2.84	9.63	3.24	6.32	7.30	11.57	7.47
miR-124.2/506	1535	12.06	-7.01	-3.27	-4.75	2.58	-12.95	-13.32	-7.20	-6.22	3.23	-10.81	-5.86	-2.87	11.75	1.50	6.43	7.40	15.36	5.84
miR-130/301	714	11.71	-5.63	-8.29	-8.30	1.55	-9.20	-11.94	-5.12	-5.38	2.73	-7.72	-5.65	-3.51	9.63	2.49	6.08	6.86	16.06	7.13
miR-19	938	11.66	-4.82	-9.75	-7.55	3.77	-10.06	-14.20	-5.28	-7.23	2.00	-11.80	-5.96	-3.52	13.62	2.06	4.43	9.60	17.20	7.23
miR-21	253	11.52	-3.26	-7.22	-6.71	-0.06	-7.44	-9.58	-2.66	-6.51	-0.63	-3.42	-3.98	-2.31	10.74	3.46	6.25	5.01	10.61	6.09
miR-25/32/92/363/367	776	11.32	-5.51	-7.53	-5.97	3.37	-8.14	-12.56	-2.88	-5.65	4.20	-9.26	-6.76	-3.50	9.14	3.22	5.68	7.05	13.77	5.98
miR-410	679	11.10	-4.84	-5.88	-8.64	3.90	-8.63	-10.29	-5.29	-6.64	0.49	-10.58	-3.47	-2.40	11.08	2.13	5.22	5.55	15.84	5.38
miR-144	678	11.07	-5.49	-9.92	-11.19	2.40	-10.63	-12.78	-4.49	-5.98	0.97	-9.81	-6.22	-4.34	12.80	2.39	5.69	9.45	20.63	5.09
miR-17-5p/20/93.mr/106/519.d	996	11.02	-7.54	-6.29	-5.22	4.39	-9.34	-13.06	-3.82	-6.65	4.46	-9.55	-6.51	-3.58	9.56	2.41	4.73	7.92	14.54	7.22
miR-96	945	11.02	-5.45	-4.72	-6.05	1.60	-8.74	-12.51	-6.13	-5.68	4.24	-9.58	-4.40	-1.38	8.82	2.38	5.79	5.34	14.08	5.47
miR-543	624	10.98	-6.31	-9.21	-12.12	0.49	-9.06	-11.14	-3.87	-6.94	-0.23	-7.55	-7.41	-3.68	14.31	3.98	7.76	9.31	19.38	4.03
miR-9	1109	10.94	-6.94	-3.14	-5.54	4.18	-10.24	-13.88	-3.14	-7.10	4.68	-10.54	-7.03	-2.08	9.14	1.20	5.40	7.94	13.46	6.76
miR-124.1	1085	10.71	-6.43	-4.67	-6.76	2.86	-11.45	-14.03	-6.83	-6.61	1.03	-11.21	-4.29	-0.93	11.95	2.65	6.33	7.65	16.02	4.11
miR-101	708	10.63	-4.65	-9.64	-9.69	4.22	-10.98	-12.11	-4.21	-6.43	1.00	-9.21	-5.53	-5.03	13.19	1.15	4.19	7.76	19.60	5.32
miR-221/222	320	10.60	-4.11	-7.18	-6.66	2.09	-7.97	-9.23	-5.06	-5.71	-0.81	-7.09	-2.58	0.14	8.31	2.60	6.33	6.77	10.79	4.89
miR-148/152	587	10.56	-5.12	-5.03	-6.85	0.66	-8.56	-8.03	-3.48	-3.79	1.76	-4.51	-4.97	-4.58	9.28	1.82	6.05	5.53	10.83	5.88
miR-200b/429	840	10.52	-5.13	-6.77	-10.01	2.84	-9.72	-12.37	-4.35	-6.32	2.74	-10.89	-7.79	-4.93	12.92	2.25	4.19	9.48	18.04	7.62
miR-505	391	10.07	-5.40	-6.36	-8.06	1.14	-7.54	-11.90	-4.40	-6.00	0.24	-6.90	-3.79	-1.74	11.71	3.25	5.42	7.35	12.36	4.07
miR-30-5p	1200	9.98	-6.61	-8.38	-9.22	2.60	-8.23	-12.38	-2.82	-3.69	4.58	-14.00	-7.48	-3.17	11.43	1.32	3.37	7.66	18.93	5.92
miR-183	379	9.98	-5.05	-6.86	-7.21	1.37	-7.40	-10.49	-4.55	-4.83	1.77	-9.24	-4.20	-0.60	9.73	3.69	4.93	7.39	11.22	5.71
miR-374	550	9.76	-5.55	-7.51	-8.53	1.59	-4.59	-9.36	-2.20	-4.73	3.44	-10.07	-5.05	-5.02	9.10	3.82	5.52	5.04	14.21	6.15
miR-494	441	9.74	-4.72	-6.88	-7.78	1.00	-7.00	-10.73	-4.28	-6.12	1.44	-6.16	-4.73	-3.86	10.30	2.84	3.62	6.95	14.52	7.11
miR-141/200a	570	9.74	-6.17	-5.67	-7.79	4.37	-7.10	-11.38	-3.22	-5.90	2.05	-10.49	-5.24	-4.82	11.66	2.14	3.85	7.76	14.35	5.19
miR-26	811	9.74	-6.63	-7.35	-9.01	1.78	-7.86	-15.13	-3.27	-5.25	4.66	-9.62	-9.76	-4.03	12.68	2.13	3.12	9.99	19.82	5.83
miR-376c	218	9.63	-3.99	-5.92	-6.59	-0.48	-5.73	-7.02	-4.79	-4.28	-0.66	-5.02	-3.45	0.46	8.28	4.85	5.78	5.27	9.15	2.21
miR-1/206	715	9.53	-3.81	-4.96	-5.02	4.21	-10.78	-11.37	-3.21	-6.40	-2.98	-6.95	-5.05	-1.91	11.72	0.90	5.78	5.62	12.61	4.57
miR-384	271	9.42	-3.04	-6.76	-5.73	1.10	-6.26	-7.72	-6.12	-5.28	-1.09	-4.22	-2.57	-1.96	9.40	1.54	3.69	5.42	11.81	4.00
miR-330	549	9.37	-4.00	-6.47	-8.29	1.37	-7.09	-10.35	-2.91	-5.90	0.88	-7.41	-3.78	-2.46	9.89	1.23	3.47	6.55	13.75	5.26
miR-132/212	379	9.36	-4.92	-6.69	-6.00	2.82	-8.48	-8.86	-4.29	-6.23	1.34	-7.66	-4.20	-2.36	11.16	2.04	3.37	5.89	12.24	5.61
miR-15/16/195/424/497	1017	9.05	-4.54	-3.05	-3.74	1.63	-9.86	-10.01	-5.77	-5.15	2.33	-6.53	-4.83	-2.40	10.05	0.91	6.06	5.35	10.62	4.51
miR-145	566	9.04	-4.64	-6.53	-7.05	4.29	-8.02	-11.88	-0.72	-6.01	1.31	-7.35	-5.91	-2.58	11.90	1.03	3.04	6.25	14.06	4.76
miR-382	168	8.76	-0.97	-5.62	-8.81	1.56	-5.78	-8.17	-2.21	-6.40	-2.70	-3.36	-0.28	-2.17	7.73	3.02	3.48	4.77	10.08	3.14
miR-500	183	8.71	-5.20	-4.65	-4.78	0.62	-5.30	-5.93	-1.57	-3.72	0.74	-2.82	-1.94	0.32	5.86	4.07	4.48	4.53	5.61	0.24
miR-376	209	8.41	-1.62	-5.76	-6.08	0.69	-6.11	-6.57	-4.97	-4.75	-1.30	-4.50	-2.90	-0.12	6.30	2.68	3.44	4.78	10.62	4.15
miR-369-3p	548	8.34	-4.18	-5.90	-6.88	3.57	-4.49	-8.89	-1.69	-4.00	4.30	-10.92	-6.64	-4.67	8.57	2.21	4.55	4.15	13.02	5.35
miR-299-5p	313	8.26	-4.64	-4.84	-4.48	1.64	-5.84	-7.30	-4.63	-3.60	0.50	-5.04	-2.97	-2.14	8.55	0.89	4.28	4.57	9.69	3.63
miR-448	555	8.24	-5.25	-6.04	-7.38	1.38	-6.87	-10.25	-4.93	-4.74	2.28	-7.68	-4.52	-2.07	9.44	2.12	3.95	2.99	14.79	6.75
miR-409-3p	312	8.02	-4.07	-5.70	-7.74	2.09	-5.91	-8.78	-3.94	-5.19	-0.44	-6.95	-3.55	-2.35	9.46	2.23	2.82	5.14	12.97	5.17
miR-377	378	7.95	-3.61	-5.92	-6.55	1.04	-7.44	-8.09	-2.08	-4.19	-1.00	-3.85	-3.47	-1.98	7.64	1.28	5.53	6.41	9.13	3.76
miR-216	200	7.91	-4.44	-2.70	-6.00	-0.54	-4.18	-7.93	-2.45	-4.00	1.33	-4.75	-3.00	-1.48	5.28	3.41	3.91	5.39	7.38	4.05
miR-223	243	7.87	-4.08	-4.31	-5.88	0.91	-4.61	-9.21	-3.98	-4.02	2.70	-6.23	-3.77	-0.41	7.37	2.25	3.79	4.02	9.32	4.22
miR-218	768	7.71	-4.39	-4.83	-8.00	1.82	-5.01	-8.45	-1.57	-2.23	3.35	-5.55	-4.46	-3.82	6.72	2.17	3.41	4.33	10.73	3.32
miR-203.1	594	7.50	-2.97	-7.18	-8.64	1.43	-5.60	-11.62	-3.84	-5.12	2.47	-8.24	-6.35	-4.69	11.71	1.29	2.39	6.58	16.55	5.03
miR-204/211	431	7.49	-3.28	-3.84	-4.58	2.89	-6.42	-6.34	-5.77	-5.01	0.04	-6.68	-1.62	-1.40	7.34	1.41	2.91	4.30	9.82	4.87
miR-379	82	7.36	-2.96	-4.54	-5.06	0.23	-4.10	-5.78	-3.88	-3.87	-0.41	-3.15	-0.92	-0.13	4.85	3.98	3.48	3.31	5.99	3.86
miR-362	157	7.34	-2.97	-5.40	-5.75	0.52	-5.10	-6.98	-1.41	-3.85	-0.18	-4.58	-0.26	-0.70	4.86	2.73	3.48	3.71	8.14	3.97
miR-544	397	7.29	-2.75	-5.11	-6.40	3.03	-6.60	-9.47	-5.45	-4.67	-0.19	-5.76	-3.21	-1.92	8.62	1.65	4.77	4.95	12.09	2.18
miR-365	218	7.28	-2.73	-3.89	-4.98	-0.44	-5.13	-5.19	-1.71	-3.77	2.48	-5.01	-4.76	-2.99	6.58	1.70	3.67	3.51	8.63	4.74
miR-539	506	7.27	-4.16	-6.87	-7.42	2.52	-6.87	-9.60	-3.65	-5.25	0.09	-7.05	-3.36	-0.98	9.36	1.86	2.02	6.77	13.75	3.95
miR-361	184	7.25	-3.14	-5.83	-4.67	2.00	-3.98	-6.81	-0.66	-4.27	1.97	-4.75	-4.21	-3.46	6.30	1.73	3.73	3.27	10.24	3.69
miR-103/107	555	7.19	-4.75	-4.67	-3.41	3.96	-5.27	-9.25	-2.99	-4.45	1.34	-7.15	-2.25	-2.69	7.96	-0.21	2.77	3.57	11.43	2.74
miR-363	198	7.17	-2.78	-4.68	-5.53	0.05	-3.85	-5.87	-3.91	-4.82	-0.03	-2.13	-2.13	-0.19	6.30	3.27	3.92	2.74	7.27	3.40
miR-155	317	7.17	-3.41	-3.99	-5.11	1.32	-5.77	-7.27	-2.71	-4.65	2.02	-6.20	-4.92	-2.99	8.41	1.41	3.18	6.04	9.50	4.89
miR-217	268	7.14	-3.29	-5.31	-7.02	1.28	-3.38	-8.52	-1.08	-3.72	2.83	-5.33	-7.06	-6.23	8.62	1.44	3.76	4.45	13.99	4.42
miR-455	161	7.10	-3.73	-4.39	-4.36	1.20	-5.33	-5.89	-2.52	-4.13	0.20	-3.11	-3.41	-0.60	8.01	0.42	3.04	2.96	8.69	3.45
miR-27	1023	7.02	-4.41	-4.18	-6.96	3.37	-6.92	-10.00	-3.19	-4.33	1.64	-8.53	-3.07	-4.34	8.19	1.56	3.46	6.10	13.63	2.69
miR-128	908	7.01	-3.81	-3.77	-5.21	4.96	-6.68	-8.04	-4.24	-4.91	1.97	-7.66	-3.26	-3.87	7.77	0.67	3.52	4.88	11.53	2.78
miR-433-3p	253	7.00	-2.74	-5.52	-7.33	1.39	-7.12	-7.88	-5.00	-4.21	-0.95	-4.86	-4.36	-6.30	9.75	1.03	2.00	8.47	15.30	4.90
miR-137	628	7.00	-3.52	-5.13	-5.40	1.47	-4.92	-9.09	-1.67	-4.18	3.11	-7.37	-4.61	-2.76	7.61	0.95	3.79	4.88	11.13	2.82
miR-153	571	6.91	-4.51	-4.95	-5.86	2.17	-7.05	-9.91	-4.36	-4.22	3.00	-8.03	-4.73	-3.31	9.12	1.50	3.54	2.40	13.94	6.54
miR-205	345	6.85	-3.99	-3.82	-7.32	0.55	-7.07	-8.31	-0.36	-3.78	1.77	-4.79	-5.10	-4.49	8.14	2.12	3.45	6.87	10.51	3.04
miR-199	407	6.75	-5.25	-2.92	-3.15	3.19	-6.04	-8.72	-0.62	-4.18	1.75	-6.37	-4.56	-2.71	7.84	0.67	3.76	4.03	9.72	3.60
miR-325	81	6.73	-3.45	-4.61	-4.31	1.32	-4.02	-5.41	-2.08	-4.61	0.41	-3.43	-1.81	-0.98	5.49	2.94	4.42	2.32	7.16	2.71
miR-329	310	6.68	-4.03	-3.32	-6.36	2.20	-5.29	-8.24	-1.24	-3.24	1.09	-5.70	-4.74	-1.65	7.70	1.28	3.36	4.44	9.37	3.60
miR-129-5p	470	6.66	-3.80	-7.05	-6.15	3.35	-5.02	-7.96	-3.23	-4.14	1.68	-7.25	-4.82	-1.10	9.35	1.75	3.23	2.52	12.33	3.31
miR-135	576	6.64	-2.58	-4.64	-5.42	3.13	-5.93	-9.36	-3.46	-5.07	1.99	-7.57	-3.63	-1.87	7.00	1.81	2.00	3.75	12.50	2.80
miR-34b	356	6.58	-3.73	-2.84	-4.55	0.17	-5.89	-6.82	-3.91	-4.09	0.80	-4.62	-1.27	-2.17	6.66	2.63	3.80	4.07	8.94	2.67
miR-503	398	6.46	-5.33	-3.98	-4.03	1.14	-4.42	-8.67	-1.51	-3.85	1.53	-5.00	-3.64	-2.90	7.84	1.34	4.32	5.91	8.42	2.48
miR-485-3p	270	6.44	-3.19	-4.33	-7.26	2.53	-5.28	-7.46	-1.65	-3.53	0.89	-4.76	-2.95	-3.21	7.14	0.34	2.91	4.91	10.96	3.16
miR-452	225	6.34	-2.87	-3.70	-3.91	-0.26	-3.35	-6.42	-3.87	-4.01	0.16	-3.76	-3.83	-1.86	6.84	2.94	2.38	3.33	9.68	2.64
miR-499	307	6.34	-3.18	-7.13	-6.40	0.82	-3.16	-6.09	-1.45	-3.20	1.96	-5.36	-5.26	-3.31	8.45	2.32	3.35	3.16	11.78	3.61
miR-24*	99	6.32	-4.50	-4.56	-4.16	-1.34	-5.02	-4.77	-2.50	-3.26	1.93	-2.97	-2.58	-2.73	5.54	1.78	2.16	4.13	8.94	4.82
miR-139	331	6.23	-0.96	-5.91	-5.13	1.81	-6.12	-6.94	-1.47	-4.34	0.08	-5.46	-4.88	-4.69	9.19	-0.29	1.89	4.47	12.06	3.68
miR-542-3p	275	6.20	-2.83	-3.58	-4.12	2.16	-5.05	-7.23	-3.44	-3.34	-0.59	-5.92	-2.12	-0.35	7.05	2.33	2.76	4.51	7.96	2.95
miR-10	244	6.15	-4.21	-2.12	-4.71	3.51	-3.80	-8.45	-1.16	-2.85	1.31	-5.90	-3.19	-1.88	6.22	2.53	3.41	3.46	7.16	3.30
miR-29	912	6.10	-5.49	-2.49	-3.78	2.73	-5.58	-10.00	-3.12	-2.39	1.33	-8.37	-2.43	-1.16	3.93	2.99	3.70	5.81	10.34	1.51

miR-375	273	6.09	-3.70	-5.94	-5.99	1.93	-4.67	-6.90	-3.34	-3.75	0.97	-5.21	-2.06	-3.10	7.77	3.00	2.34	4.55	11.23	2.14
miR-140	252	6.06	-3.43	-3.61	-4.21	0.75	-4.02	-6.68	-3.48	-5.00	2.13	-5.42	-1.51	-1.95	4.48	1.40	2.46	5.48	8.89	3.21
miR-7	394	5.95	-3.89	-1.32	-3.51	3.14	-6.86	-8.44	-0.88	-5.52	0.57	-5.61	-2.40	-2.81	6.46	1.37	2.44	3.64	9.09	3.18
miR-378	434	5.93	-2.54	-2.24	-4.25	0.41	-5.83	-7.30	-3.37	-2.94	0.68	-4.63	-3.03	-1.62	6.09	1.22	2.99	3.17	9.46	3.10
miR-193	211	5.84	-2.36	-4.73	-4.29	3.44	-5.90	-4.80	-0.82	-3.81	-0.48	-3.41	-1.48	-2.13	6.33	-0.98	2.04	3.72	7.41	3.86
miR-335	226	5.60	-3.60	-3.57	-4.17	1.70	-5.19	-6.48	-2.03	-5.18	1.99	-4.54	-2.97	0.86	4.87	1.05	2.00	3.55	7.88	4.07
miR-338	297	5.44	-1.91	-3.06	-4.02	1.37	-6.80	-5.98	-1.92	-5.36	0.36	-5.60	-1.89	-4.21	5.42	1.56	2.38	5.20	9.53	4.45
miR-224	294	5.27	-3.96	-3.60	-4.65	0.90	-5.07	-6.32	-1.64	-2.95	4.14	-5.57	-4.15	-4.66	6.65	-0.16	1.68	5.03	10.46	3.56
miR-138	492	5.26	-2.89	-3.08	-2.28	1.80	-4.79	-6.14	-2.93	-3.07	-0.21	-4.15	-2.95	-1.60	5.08	1.24	5.29	3.73	5.81	2.33
miR-208	248	5.16	-2.81	-6.68	-5.62	0.65	-2.52	-5.21	-1.35	-2.42	2.35	-5.15	-5.45	-3.07	7.68	1.87	3.06	2.64	11.17	3.40
miR-33	309	5.08	-4.71	-4.14	-4.77	2.48	-3.17	-7.55	0.23	-2.15	3.21	-5.57	-5.79	-2.51	5.26	1.92	3.02	1.91	10.92	1.89
miR-486	142	5.05	-2.24	-3.96	-3.43	0.31	-3.71	-5.59	-1.58	-2.98	0.47	-4.14	-3.18	-1.30	4.45	1.70	2.07	4.36	8.44	2.28
miR-31	273	4.93	-2.59	-2.77	-0.98	2.14	-5.77	-4.92	-4.35	-4.47	0.57	-4.20	-0.61	-0.86	5.28	2.17	1.78	4.23	4.72	2.67
let-7/98	811	4.92	-2.55	-3.36	-2.47	0.88	-7.13	-7.39	-1.99	-2.51	2.37	-7.03	-4.90	-2.60	4.32	1.14	2.41	6.14	9.52	4.74
miR-421	131	4.61	-2.20	-0.73	-2.50	0.26	-4.28	-3.59	-3.39	-2.81	0.77	-1.76	-1.75	-3.10	3.83	1.29	2.11	2.99	5.21	2.83
miR-24	464	4.51	-3.15	-0.53	0.02	3.21	-4.56	-5.65	-2.69	-2.25	1.09	-4.35	-1.96	-1.68	4.51	0.80	2.81	2.26	3.66	3.80
miR-136	157	4.50	-1.01	-1.91	-2.86	2.48	-3.71	-3.92	-3.11	-3.86	-2.42	-2.32	-0.88	-2.01	4.41	-1.55	1.63	2.06	7.60	2.24
miR-342	243	4.41	-4.54	-4.02	-2.84	2.35	-5.28	-6.35	-0.75	-3.09	-0.04	-4.66	-0.14	-1.07	6.20	-0.25	3.10	2.31	7.46	1.32
miR-22	377	4.21	-2.47	-1.48	-2.30	2.46	-3.84	-6.24	-1.94	-1.77	2.47	-4.84	-3.06	-1.43	2.84	1.45	2.27	2.45	6.16	2.38
miR-143	304	4.18	-3.81	-3.12	-2.53	-0.43	-3.34	-5.57	-1.94	-1.78	1.09	-5.70	-1.87	0.36	5.21	0.63	1.81	1.65	8.37	1.81
miR-133	454	4.07	-2.57	0.09	-1.19	2.53	-5.71	-5.28	-3.00	-5.01	0.15	-3.86	-3.54	-1.34	5.43	0.26	3.17	3.03	6.73	1.50
miR-18	217	4.00	-2.93	-2.00	-4.41	2.75	-5.13	-6.07	-2.41	-1.27	0.45	-5.40	-3.06	-1.83	6.11	0.57	1.59	2.12	8.79	2.09
miR-450	274	3.99	-3.73	-5.52	-2.87	3.69	-3.34	-6.17	-2.07	-2.49	0.92	-7.25	-4.75	-0.60	8.47	0.68	1.12	2.07	9.86	2.55
miR-142-3p	264	3.76	-2.73	-4.49	-3.82	6.32	-6.25	-7.05	-2.17	-4.00	1.05	-5.25	-4.47	-2.37	8.99	-0.91	-0.06	2.81	10.10	5.56
miR-34/449	528	3.71	-2.77	-0.04	-2.55	1.24	-3.29	-4.90	-1.54	-2.32	1.86	-4.98	-3.15	0.06	3.48	0.60	2.11	3.21	5.21	1.38
miR-299-3p	108	3.70	-2.50	-2.71	-2.22	0.86	-5.14	-2.65	-0.96	-2.68	-1.07	-2.62	0.14	-1.95	4.34	0.67	2.41	3.01	5.22	1.20
miR-134	147	3.62	-2.43	-2.87	-3.90	1.76	-4.23	-5.24	-2.46	-3.17	-1.25	-2.97	-0.70	0.20	5.25	0.24	1.65	3.50	6.35	0.75
miR-324-3p	233	3.56	-0.94	-3.10	-3.41	4.98	-6.17	-5.42	-1.47	-3.08	-0.96	-3.87	-2.41	-1.73	6.48	0.21	0.48	2.34	8.03	3.74
miR-146	166	3.45	-3.43	-2.69	-1.89	-0.47	-3.52	-3.27	-1.76	-1.17	-0.57	-5.09	-0.14	0.90	2.46	1.56	2.53	2.77	5.51	1.46
miR-219	320	3.42	-1.79	-3.21	-3.33	0.35	-3.30	-6.20	-0.39	-2.22	2.94	-7.94	-3.64	-3.37	6.22	-0.62	1.28	4.15	9.44	1.20
miR-346	104	3.37	-1.05	-2.00	-2.24	1.00	-3.58	-4.11	-0.67	-4.02	-1.92	-1.67	0.14	1.00	3.16	1.23	1.88	2.00	3.65	1.37
miR-488	182	3.27	-0.80	-3.41	-4.35	0.88	-2.75	-5.78	-2.55	-2.40	0.21	-3.94	-1.59	-4.93	5.07	0.56	0.72	3.15	8.61	3.67
miR-504	162	3.26	-3.12	-0.29	-0.62	1.85	-3.92	-4.13	-1.60	-1.88	2.39	-3.79	-0.46	0.64	2.64	1.66	2.27	2.72	2.20	-0.82
miR-190	137	3.26	-1.65	-4.38	-3.86	3.19	-2.04	-4.97	-1.94	-3.47	1.04	-4.92	-2.16	-0.90	5.30	1.22	1.16	1.46	6.70	3.21
miR-383	105	3.04	-0.98	-1.96	-4.43	-0.33	-3.35	-2.61	-1.50	-0.80	1.29	-1.33	-2.35	-1.93	3.69	0.17	2.66	1.59	5.67	1.53
miR-409-5p	138	2.98	-1.00	-3.62	-3.93	1.30	-3.75	-4.24	-2.31	-1.60	-0.19	-3.67	-0.91	-0.97	5.35	0.42	1.31	0.67	6.18	4.02
miR-324-5p	146	2.90	-0.32	-2.46	-1.54	1.78	-4.71	-2.21	-2.34	-2.43	-0.91	-2.93	-0.70	-2.93	4.40	-0.78	0.69	1.85	5.99	2.91
miR-431	120	2.83	-1.73	-1.35	-3.52	1.55	-2.66	-4.89	0.89	-1.52	2.69	-4.58	-3.34	-1.87	3.60	-0.05	1.85	1.30	5.89	2.56
miR-370	283	2.78	-1.20	-0.07	-0.81	1.53	-4.54	-2.79	-1.57	-1.79	-0.72	-2.18	-0.54	-0.02	2.60	1.01	1.96	2.08	2.24	1.58
miR-196	235	2.77	-1.46	-3.30	-4.95	-0.57	-0.95	-5.51	-0.69	-1.00	2.56	-3.81	-1.60	-2.23	1.90	1.77	0.85	2.87	6.23	3.66
miR-125/351	663	2.76	-1.49	1.71	-0.19	2.28	-8.25	-5.18	-2.53	-5.85	2.09	-2.65	-2.94	-1.30	3.75	-0.20	2.19	5.04	5.39	1.27
miR-451	28	2.75	-0.19	-0.90	-1.00	1.54	-4.29	-5.15	-3.37	-2.79	-0.11	-2.54	-1.03	-0.35	2.37	1.82	1.58	2.05	5.05	1.75
miR-490	137	2.60	-0.45	1.23	-1.47	3.50	-4.36	-4.33	-0.79	-2.00	-1.47	-1.67	-3.03	-3.76	6.34	-3.32	1.14	1.86	6.37	1.29
miR-149	332	2.56	-1.72	-1.36	-0.45	2.72	-4.33	-3.68	-2.81	-2.43	-0.10	-3.72	0.03	-1.68	4.23	0.84	0.62	2.47	4.70	0.83
miR-214	529	2.49	-1.67	0.70	-0.12	3.68	-4.66	-5.43	-2.99	-4.20	-0.39	-2.42	-3.39	-1.66	5.39	0.49	2.96	1.19	5.18	0.82
miR-485-5p	275	2.33	-3.03	-0.51	0.03	2.42	-5.06	-4.89	-0.44	-5.11	-0.40	-2.90	-0.18	-0.41	5.15	0.64	3.34	2.77	2.58	1.82
miR-326	367	2.21	-0.47	-0.89	-0.96	2.88	-5.13	-1.53	-1.05	-3.10	-1.37	-2.33	2.41	0.53	1.97	-1.00	1.16	2.22	2.30	0.80
miR-378*	136	2.20	-2.78	-2.98	-2.91	0.42	-1.13	-4.64	1.16	-0.74	1.73	-2.34	-1.85	-0.69	3.89	0.90	1.07	2.46	3.00	1.34
miR-122	211	2.13	-0.83	-1.66	-1.42	3.08	-5.61	-4.99	-2.21	-3.23	0.46	-4.69	-1.17	-1.61	6.65	-0.30	0.47	2.43	5.20	3.26
miR-188	169	2.04	0.48	-2.00	-1.97	1.41	-2.06	-2.55	-1.71	-2.64	0.88	-3.86	-3.00	-2.74	4.56	0.04	0.07	1.95	6.20	2.34
miR-192/215	135	1.88	-0.64	-2.39	-2.80	1.44	-2.92	-3.43	-0.34	-1.38	0.41	-3.13	-2.07	-1.43	4.11	-0.42	1.34	2.96	4.24	0.50
miR-339	192	1.82	-1.43	-1.13	-1.13	4.68	-1.43	-4.43	-1.32	-1.36	0.84	-3.29	-2.42	-1.27	3.94	-0.30	0.50	1.17	3.79	0.64
miR-380-5p	69	1.58	0.07	-2.57	-0.97	0.97	-1.45	-1.93	-1.44	0.66	-1.18	-3.67	-1.76	0.82	1.91	0.40	-0.69	1.92	6.92	-1.64
miR-126/126-3p	21	1.56	-1.95	0.00	0.87	1.06	-1.13	-1.83	-1.42	-1.14	0.69	-3.25	-0.84	-0.74	1.97	-1.03	1.69	1.79	1.36	0.43
miR-151	78	1.32	-0.76	-2.46	-4.93	1.97	-0.92	-3.96	-0.51	-1.12	-1.52	-2.42	-0.42	-2.15	4.40	-0.60	1.30	3.17	5.89	0.20
miR-28	183	1.21	-2.22	-0.49	0.98	1.00	-1.52	-2.62	-0.39	-0.29	0.15	-3.37	0.81	3.13	-0.79	2.32	2.11	0.92	0.16	-1.95
miR-184	30	1.16	1.93	-0.07	-1.50	0.84	-1.29	-0.63	-2.38	0.53	-1.18	-0.44	0.88	1.40	-1.42	0.83	0.52	3.42	-0.02	-0.57
miR-185	220	0.67	-1.18	-0.01	-0.16	1.83	-1.72	-1.07	-0.96	-0.33	0.69	0.56	0.50	-0.50	0.28	-1.10	0.35	0.38	1.16	-0.48
miR-150	257	0.53	-1.51	-1.72	-1.21	3.45	-2.26	-3.61	-1.36	-2.69	-0.69	-2.87	-1.38	-1.66	4.07	-1.35	0.96	1.98	5.83	-0.98
miR-331	203	0.45	0.68	2.00	1.32	4.27	-3.87	-0.04	-1.52	-2.99	-2.02	-2.77	2.74	1.09	0.00	0.41	0.89	0.31	-1.23	0.87
miR-99/100	51	0.45	-0.50	-0.22	-1.23	0.45	-1.44	-2.67	1.35	-1.38	3.23	-0.90	-1.87	-0.66	0.93	-0.61	-0.05	0.71	2.11	0.99
miR-328	187	-0.04	-1.10	-0.46	-1.68	3.07	-0.69	-0.59	-0.29	1.10	1.83	-2.48	-1.89	-0.50	1.82	-0.77	-0.33	0.35	2.17	-1.22
miR-491	131	-0.31	-0.11	2.29	0.75	1.44	-1.31	-0.75	-0.36	-0.20	0.40	-1.68	2.02	1.36	-2.67	0.22	-2.12	0.90	-0.42	-0.86
miR-191	50	-0.47	-1.48	-0.07	-0.40	0.49	-1.49	-1.12	1.33	0.23	1.93	-1.59	-1.43	-0.79	1.12	-0.54	0.58	0.39	2.15	-0.67
miR-210	24	-0.99	0.91	0.71	-1.22	-0.72	1.51	-1.07	1.20	0.56	1.52	1.72	-0.20	-0.72	-0.28	1.17	0.43	-1.82	-1.11	-1.06
