# Supplementary material for: Increased MicroRNA Activity in Human Cancers
Source: PLoS One. 2009 Jun 25;4(6):e6045. doi: 10.1371/journal.pone.0006045 (PMC2698213; doi:10.1371/journal.pone.0006045)
Supplement: Table S2 — MiR-seeds found to be the most significantly upregulated in PTC (0.04 MB PDF) [file pone.0006045.s003.pdf]

Supplemental Table 2: MiR-seeds found to be the most significantly upregulated in PTC.
using two-sample t-test p-values*

rank	miR-seed	p-value
	miR-379	2.35E-04
1	miR-346	3.46E-04
	miR-363	3.85E-04
	miR-376c	8.86E-04
	miR-500	1.11E-03
	miR-382	1.26E-03
2	miR-21	1.33E-03
	miR-362	1.41E-03
	miR-376	1.58E-03
3	miR-221/222	1.90E-03
	miR-384	2.05E-03
	miR-325	2.49E-03
	miR-377	2.54E-03
	miR-134	2.72E-03
	miR-30-3p	3.11E-03
	miR-34b	3.38E-03
	miR-505	3.45E-03
	miR-216	3.91E-03
	miR-299-3p	3.94E-03
4	miR-323	4.23E-03
5	miR-24*	4.45E-03
6	miR-146	4.58E-03
	miR-455	4.95E-03
	miR-496	5.11E-03
7	miR-194	5.51E-03
	miR-543	6.18E-03
	miR-31	6.47E-03
	miR-452	6.75E-03
	miR-299-5p	6.79E-03
	miR-494	7.12E-03
	miR-542-3p	7.35E-03
	miR-183	7.37E-03
	miR-539	8.34E-03
	miR-409-3p	8.60E-03
8	miR-204/211	8.66E-03
9	miR-148/152	8.71E-03
10	miR-23	8.72E-03
	miR-421	8.76E-03
	miR-544	8.90E-03
	miR-370	9.04E-03
11	miR-335	9.30E-03
12	miR-93.hd/291-3p/294	9.32E-03
13	miR-130/301	9.63E-03
	miR-330	9.71E-03
	miR-493-5p	1.02E-02
14	miR-138	1.05E-02
15	miR-223	1.07E-02
	miR-140	1.07E-02
	miR-495	1.08E-02
16	miR-1/206	1.08E-02
	miR-124.1	1.09E-02
	miR-451	1.09E-02
	miR-375	1.14E-02
17	miR-132/212	1.14E-02
	miR-378	1.15E-02
	miR-182	1.19E-02
	miR-409-5p	1.24E-02
	miR-381	1.27E-02
	miR-486	1.31E-02
	miR-144	1.32E-02
18	miR-342	1.32E-02
	miR-448	1.32E-02
	miR-485-5p	1.47E-02
19	miR-15/16/195/424/49	1.48E-02
	miR-410	1.53E-02
	miR-433-3p	1.54E-02
20	miR-136	1.56E-02
21	miR-320	1.63E-02
	miR-503	1.63E-02
22	miR-193	1.66E-02
23	miR-96	1.68E-02
	miR-142-5p	1.73E-02
24	miR-181	1.94E-02
	miR-19	1.97E-02
	miR-338	1.98E-02
	miR-101	2.03E-02
	miR-485-3p	2.10E-02
	miR-383	2.16E-02
	miR-143	2.19E-02
	miR-124.2/506	2.27E-02
25	miR-205	2.29E-02
	miR-186	2.35E-02
26	miR-155	2.39E-02
	miR-329	2.48E-02
	miR-365	2.50E-02
27	miR-326	2.67E-02
28	miR-200b/429	2.89E-02
29	miR-145	2.90E-02
30	miR-129-5p	3.06E-02
31	miR-7	3.18E-02
32	miR-17-5p/20/93.mr/106/51	3.21E-02
33	miR-25/32/92/363/367	3.25E-02
	miR-153	3.32E-02
	miR-499	3.39E-02
	miR-361	3.39E-02
	miR-203.1	3.50E-02
34	miR-29	3.56E-02
	miR-374	3.65E-02
35	miR-27	3.65E-02
36	miR-135	3.73E-02
	miR-141/200a	3.78E-02
37	miR-218	3.79E-02
38	miR-26	3.94E-02
	miR-10	4.00E-02
39	miR-133	4.04E-02
	miR-151	4.09E-02
40	miR-103/107	4.10E-02
	miR-488	4.16E-02
	miR-149	4.30E-02
	miR-324-5p	4.36E-02
41	miR-9	4.49E-02
	miR-137	4.49E-02
	miR-190	4.56E-02
	miR-378*	4.66E-02
	miR-139	4.83E-02
42	miR-196	5.01E-02
43	miR-324-3p	5.08E-02
44	miR-199	5.21E-02
45	miR-128	5.24E-02
	miR-208	5.29E-02
	miR-18	5.68E-02
46	let-7/98	6.20E-02
47	miR-122	6.40E-02
48	miR-30-5p	6.45E-02
49	miR-24	7.02E-02
	miR-504	7.21E-02
	miR-217	7.25E-02
50	miR-192/215	7.42E-02
51	miR-34/449	7.68E-02
52	miR-125/351	7.84E-02
53	miR-224	8.10E-02
54	miR-214	8.60E-02
	miR-142-3p	9.03E-02
	miR-450	9.27E-02
	miR-22	9.45E-02
55	miR-33	1.19E-01
	miR-369-3p	1.20E-01
56	miR-150	1.46E-01
57	miR-184	1.71E-01
	miR-188	1.88E-01
58	miR-219	2.02E-01
	miR-431	2.24E-01
	miR-380-5p	2.36E-01
	miR-28	2.56E-01
	miR-210	2.83E-01
	miR-490	2.84E-01
59	miR-185	3.08E-01
60	miR-126/126-3p	3.42E-01
	miR-491	3.75E-01
61	miR-339	3.78E-01
62	miR-328	5.47E-01
63	miR-331	6.27E-01
64	miR-99/100	6.39E-01
65	miR-191	9.70E-01

* MicroRNA seeds which did not correspond to any microRNA in the array experiment by He et al. are written in grey. The t-test p-values have been used to rank the seeds displaying the most significant deviation of activity in tumors, and are provided here for information.
