# Supplementary material for: Increased MicroRNA Activity in Human Cancers
Source: PLoS One. 2009 Jun 25;4(6):e6045. doi: 10.1371/journal.pone.0006045 (PMC2698213; doi:10.1371/journal.pone.0006045)
Supplement: Table S3 — MiR-seed activity matrix - Breast cancer (0.08 MB PDF) [file pone.0006045.s004.pdf]

Supplemental Table 3: MiR-seed activity matrix - Breast cancer

miR-seed	nb targets	median diff	Normal NB42	Normal NB58	Normal NB60	Normal NB64	Normal NB69	Normal NB83	Normal NB87	Tumor BLC_T116	Tumor BLC_T118	Tumor BLC_T123	Tumor BLC_T129
Any-MiR	11484	14.63	-21.39	-9.54	-14.52	-12.10	-15.90	-27.06	-15.06	-2.69	-0.65	17.81	-11.65
let-7/98	811	5.72	-5.69	-1.81	-2.99	-1.27	-4.15	-6.87	-5.48	0.03	0.93	6.10	1.67
miR-1/206	715	5.18	-7.10	-2.93	-3.90	-3.85	-5.70	-8.76	-4.78	-2.01	-1.92	4.70	-4.26
miR-10	244	4.18	-5.97	-3.26	-1.66	-2.96	-3.70	-4.58	-4.39	-2.74	-2.50	1.46	-3.22
miR-101	708	5.69	-7.67	-2.34	-4.69	-4.52	-5.40	-7.95	-5.17	-4.34	-3.60	4.67	0.31
miR-103/107	555	4.86	-5.21	-2.29	-2.96	-4.55	-5.32	-5.85	-4.27	-2.09	-1.18	5.24	-3.80
miR-122	211	2.12	-1.87	-3.17	-1.56	-0.32	-2.80	-4.01	-1.95	-2.05	0.05	2.93	-2.38
miR-124.1	1085	7.75	-9.55	-5.88	-7.34	-6.27	-6.78	-11.85	-6.58	-2.84	2.23	5.02	-5.06
miR-124.2/506	1535	9.90	-11.43	-5.99	-8.64	-6.80	-8.77	-12.68	-9.02	-3.11	1.88	5.68	-3.93
miR-125/351	663	4.60	-6.17	-3.04	-2.78	-3.50	-4.16	-5.87	-4.07	2.10	0.05	6.49	-1.35
miR-126/126-3p	21	1.65	-3.09	-1.70	-1.45	-0.08	-1.06	-1.21	-1.71	-1.52	-0.02	2.13	-0.73
miR-128	908	5.74	-8.44	-4.53	-5.13	-4.38	-5.57	-8.66	-6.42	-1.69	-1.58	6.87	-2.44
miR-129-5p	470	6.12	-7.96	-3.78	-3.00	-5.20	-7.32	-5.76	-7.48	-3.76	-0.34	3.88	-0.84
miR-130/301	714	7.68	-10.72	-3.52	-5.63	-5.34	-6.67	-10.41	-7.72	-2.11	-1.92	5.54	1.44
miR-132/212	379	4.63	-6.31	-1.76	-2.63	-2.22	-4.47	-5.50	-4.14	-4.15	-2.10	3.48	-2.14
miR-133	454	4.47	-5.91	-3.01	-4.19	-4.10	-4.43	-5.92	-4.05	-1.24	0.46	7.57	-1.49
miR-134	147	3.92	-3.87	-2.10	-3.33	-2.06	-3.90	-4.83	-3.10	-2.10	1.08	4.75	0.70
miR-135	576	4.15	-6.75	-2.23	-4.37	-2.90	-4.08	-6.55	-4.18	-1.30	-0.30	5.15	-1.35
miR-136	157	1.22	-3.37	0.47	-0.62	-1.92	-0.12	-2.35	-1.46	-0.54	-0.65	5.80	0.47
miR-137	628	5.06	-7.11	-3.56	-3.89	-4.42	-4.89	-7.20	-4.77	-0.98	-2.36	4.12	-1.53
miR-138	492	5.70	-7.28	-3.08	-3.68	-4.36	-5.06	-5.90	-6.01	-0.19	-1.13	4.55	-1.69
miR-139	331	0.04	-2.60	0.37	-0.79	-0.27	-1.00	-4.36	-0.70	-2.32	-0.91	1.91	-0.49
miR-140	252	3.60	-5.70	-3.22	-3.24	-2.01	-4.52	-4.08	-3.16	-2.61	-0.29	4.62	-0.87
miR-141/200a	570	5.72	-7.92	-3.90	-5.55	-4.35	-5.90	-7.87	-5.73	-1.82	-0.43	6.06	-2.49
miR-142-3p	264	3.13	-5.70	-2.89	-2.71	-2.45	-2.61	-6.50	-2.87	-2.16	-1.61	3.27	0.03
miR-142-5p	621	5.12	-8.07	-2.41	-5.06	-4.91	-4.81	-8.77	-4.52	-2.15	-1.97	4.43	0.14
miR-143	304	3.99	-3.93	-2.03	-3.09	-4.24	-1.75	-4.20	-3.19	-1.06	-1.94	2.36	-1.65
miR-144	678	5.37	-6.99	-2.54	-3.91	-3.82	-5.43	-8.61	-5.48	-4.02	-3.76	4.80	-0.26
miR-145	566	4.15	-6.66	-2.76	-2.15	-3.31	-4.62	-8.46	-4.83	-4.23	-2.39	3.63	-2.32
miR-146	166	3.22	-3.72	-2.32	-2.03	-4.14	-4.51	-2.15	-2.83	-2.34	0.75	2.48	-0.15
miR-148/152	587	5.59	-6.71	-2.68	-3.43	-3.67	-4.88	-5.06	-5.43	-2.64	-3.25	4.41	-1.11
miR-149	332	4.28	-5.47	-3.04	-2.36	-2.96	-4.09	-4.02	-3.61	0.21	0.59	4.09	-0.84
miR-15/16/195/424/497	1017	8.45	-10.40	-5.76	-6.18	-6.34	-7.29	-11.50	-7.99	0.59	-2.29	5.96	-3.16
miR-150	257	3.80	-4.11	-2.06	-2.81	-2.60	-3.25	-4.36	-3.56	-1.99	-1.50	2.96	-0.09
miR-151	78	0.82	-0.77	-0.94	-0.67	0.23	-1.53	-2.10	-1.89	-0.33	-2.00	2.86	-0.97
miR-153	571	4.89	-8.35	-1.86	-4.47	-3.64	-5.22	-8.39	-6.03	-2.49	-1.59	5.60	-1.54
miR-155	317	2.93	-3.59	-0.94	-1.84	-1.16	-2.35	-5.84	-3.18	0.10	-5.26	1.76	-0.80
miR-17-5p/20/93.mr/106/519.	996	9.22	-10.96	-5.61	-6.16	-6.93	-8.39	-11.59	-8.38	-2.19	0.07	6.06	-1.18
miR-18	217	4.93	-5.92	-2.63	-3.87	-4.32	-3.51	-5.44	-4.63	-1.73	-0.27	3.80	0.67
miR-181	982	6.96	-9.78	-4.87	-5.79	-4.46	-6.16	-10.34	-6.54	-0.77	-3.34	5.85	-2.12
miR-182	875	7.11	-8.65	-4.88	-5.97	-2.93	-8.02	-10.78	-6.74	-4.24	-0.99	5.08	-4.98
miR-183	379	4.78	-6.55	-2.50	-5.64	-3.83	-4.09	-8.36	-4.44	-1.63	-1.98	3.78	-0.67
miR-184	30	2.66	-1.83	-2.18	-1.62	-2.42	-2.73	-0.90	-2.24	-1.57	-0.60	2.50	-0.16
miR-185	220	4.15	-5.08	-1.94	-2.88	-3.25	-4.29	-2.48	-4.37	-0.92	1.07	4.64	-1.49
miR-186	676	2.55	-5.09	-1.42	-1.43	-2.65	-2.18	-7.79	-2.54	-4.21	-1.71	3.09	0.33
miR-188	169	3.32	-3.54	-0.08	-0.71	-1.50	-2.85	-4.13	-3.23	1.00	0.43	1.18	-1.02

miR-seed	nb targets	median diff	Normal NB42	Normal NB58	Normal NB60	Normal NB64	Normal NB69	Normal NB83	Normal NB87	Tumor BLC_T116	Tumor BLC_T118	Tumor BLC_T123	Tumor BLC_T129
miR-19	938	8.60	-12.38	-5.31	-7.98	-6.50	-8.39	-12.27	-8.21	-0.84	-0.46	8.35	0.82
miR-190	137	0.23	0.32	2.13	-0.08	0.08	-0.70	-1.35	-0.09	-3.03	-4.12	0.82	-2.45
miR-191	50	3.40	-3.15	-3.01	-1.22	-3.02	-2.66	-3.36	-3.09	0.48	-1.31	1.13	0.17
miR-192/215	135	4.35	-3.67	-2.20	-3.74	-3.47	-4.46	-3.86	-3.00	-4.06	1.84	4.03	2.22
miR-193	211	4.70	-5.11	-1.84	-2.15	-4.21	-4.49	-4.50	-3.30	0.06	-1.00	2.89	1.39
miR-194	351	3.50	-5.17	-0.81	-2.27	-1.65	-4.10	-4.91	-3.01	-3.65	-2.97	3.03	-2.71
miR-196	235	1.63	-1.55	-0.73	-1.43	-0.53	-2.59	-3.38	-2.24	-2.54	-0.81	-0.08	0.74
miR-199	407	4.14	-5.11	-1.67	-0.86	-4.09	-3.35	-5.76	-3.79	-1.81	-2.33	3.02	-2.35
miR-200b/429	840	6.34	-7.70	-2.03	-3.57	-2.02	-6.40	-9.78	-6.81	-3.75	-0.77	4.48	-4.35
miR-203.1	594	4.23	-6.19	-1.15	-3.91	-4.29	-4.43	-7.52	-3.98	-1.75	-1.61	3.42	-2.01
miR-204/211	431	5.62	-6.62	-2.86	-5.68	-2.86	-5.91	-5.27	-5.01	-0.58	-0.45	5.19	-1.41
miR-205	345	3.20	-5.86	-1.54	-2.57	-2.99	-3.22	-5.05	-3.26	-2.39	-0.42	3.36	-1.33
miR-208	248	1.11	-2.92	-0.44	-0.53	-0.93	-1.19	-3.96	-2.73	-2.09	-1.17	-0.34	-1.77
miR-21	253	4.20	-4.76	-1.96	-3.53	-3.66	-3.85	-5.52	-3.28	-2.63	-3.14	1.67	-0.40
miR-210	24	0.98	-1.59	-0.42	-1.50	0.10	-0.67	-2.17	-0.57	0.18	-0.64	1.96	0.79
miR-214	529	3.12	-4.16	-2.27	-2.81	-2.83	-2.77	-3.52	-3.11	-0.89	-1.20	3.33	-0.92
miR-216	200	3.04	-3.89	-0.74	-0.54	-2.39	-2.50	-3.57	-3.79	-0.68	-1.88	2.03	0.57
miR-217	268	0.88	-1.84	0.20	0.24	-0.94	0.39	-2.94	-1.70	-2.07	-3.56	2.50	-0.20
miR-218	768	4.74	-4.42	-0.62	-4.40	-2.91	-3.84	-5.23	-4.22	-0.89	0.79	5.04	-0.66
miR-219	320	5.88	-5.98	-3.19	-5.35	-3.17	-4.96	-6.25	-4.65	-1.01	0.45	4.26	-0.20
miR-22	377	5.37	-6.88	-3.22	-1.88	-2.76	-4.40	-6.68	-5.63	-2.98	0.50	3.92	0.06
miR-221/222	320	4.53	-6.81	-2.50	-4.14	-2.99	-3.74	-5.56	-4.33	-1.46	-0.94	4.14	0.76
miR-223	243	4.03	-4.39	-1.99	-3.50	-3.10	-3.32	-5.70	-3.15	-2.18	-1.45	2.81	-0.58
miR-224	294	4.76	-5.57	-1.65	-3.53	-2.58	-4.62	-5.43	-4.43	-2.59	-1.49	3.64	-2.04
miR-23	866	6.93	-9.30	-4.36	-5.85	-3.60	-6.08	-9.66	-6.80	-1.85	-1.92	4.75	-1.78
miR-24	464	5.89	-6.87	-3.76	-4.51	-4.25	-5.58	-5.81	-5.09	1.47	-0.02	4.16	1.88
miR-24*	99	-0.48	-0.99	2.33	0.42	0.20	0.39	-1.89	0.73	-0.09	-1.82	0.95	-3.04
miR-25/32/92/363/367	776	6.12	-8.77	-3.07	-5.47	-4.34	-6.76	-10.52	-5.89	-1.48	0.10	5.74	0.35
miR-26	811	2.75	-4.16	-0.28	-2.79	-2.20	-2.62	-8.15	-0.59	-2.24	-1.57	4.46	0.35
miR-27	1023	5.83	-8.11	-4.31	-6.07	-3.83	-5.85	-8.63	-6.44	-1.02	-1.85	7.64	-2.73
miR-28	183	4.52	-5.27	-2.50	-1.63	-4.85	-4.00	-3.93	-5.18	-1.46	0.21	3.39	-2.83
miR-29	912	6.60	-5.54	-4.43	-4.46	-5.92	-6.04	-8.55	-6.84	-2.70	-0.24	10.92	-0.38
miR-299-3p	108	2.27	-3.93	-2.73	-1.15	-2.09	-2.20	-3.20	-1.48	-1.63	-1.82	2.00	-1.09
miR-299-5p	313	4.43	-6.48	-2.99	-3.47	-2.83	-3.71	-5.71	-4.32	-1.61	-1.36	3.04	-0.96
miR-30-3p	425	4.52	-6.55	-2.29	-4.07	-3.86	-4.72	-8.37	-3.25	-0.78	-1.34	4.33	-1.10
miR-30-5p	1200	5.41	-7.25	-1.89	-3.89	-2.31	-5.13	-8.78	-6.40	-4.82	-1.77	7.50	-0.60
miR-31	273	4.63	-5.59	-3.94	-4.28	-2.86	-4.31	-6.77	-3.86	-0.01	-0.23	4.29	-0.89
miR-320	685	4.38	-6.84	-3.25	-4.05	-3.05	-3.63	-9.15	-5.22	-2.51	-3.46	4.67	-2.71
miR-323	720	4.32	-7.31	-2.79	-4.59	-1.42	-4.33	-7.64	-4.98	-1.72	-1.95	4.77	-3.79
miR-324-3p	233	2.28	-3.97	-1.38	-1.82	-0.67	-3.20	-3.54	-2.17	-2.76	-1.09	3.33	-1.69
miR-324-5p	146	0.88	-1.72	-1.34	-0.07	-0.94	-0.54	-1.37	-0.90	-1.52	-4.58	1.70	-2.90
miR-325	81	1.10	-3.20	-0.02	0.46	-1.17	0.65	-4.05	-2.36	-0.75	-1.00	0.34	-2.49
miR-326	367	4.18	-5.13	-3.82	-3.07	-3.90	-5.31	-3.83	-4.82	0.20	0.05	5.50	-0.65
miR-328	187	2.26	-2.99	0.52	-0.24	-1.84	-1.89	-3.16	-3.31	-0.42	-0.85	1.26	-1.88
miR-329	310	3.73	-5.84	-2.09	-1.55	-2.50	-3.79	-4.28	-4.64	-2.07	-2.64	2.13	-1.28
miR-33	309	2.90	-2.62	-0.72	-2.66	-1.87	-2.81	-4.77	-2.74	-0.97	-1.17	-0.19	-0.87
miR-330	549	5.07	-8.92	-2.05	-4.68	-3.56	-4.72	-9.14	-4.43	-1.32	1.15	4.95	-1.42
miR-331	203	3.95	-3.76	-3.32	-3.32	-2.99	-6.20	-3.18	-5.10	1.74	0.56	6.04	-0.63

miR-seed	nb targets	median diff	Normal NB42	Normal NB58	Normal NB60	Normal NB64	Normal NB69	Normal NB83	Normal NB87	Tumor BLC_T116	Tumor BLC_T118	Tumor BLC_T123	Tumor BLC_T129
miR-335	226	3.59	-5.35	-2.13	-2.02	-2.72	-2.89	-5.91	-3.08	-1.90	0.60	1.69	-2.10
miR-338	297	3.17	-5.90	-2.62	-1.20	-2.46	-2.21	-4.27	-2.95	-1.50	-0.37	1.41	-1.03
miR-339	192	3.95	-4.08	-1.54	-1.69	-3.32	-3.41	-5.50	-4.21	1.04	-1.18	3.13	-0.16
miR-34/449	528	6.21	-6.56	-3.88	-4.09	-5.52	-5.57	-7.55	-5.59	-0.01	-0.80	7.38	-1.70
miR-342	243	3.31	-4.72	-1.62	-1.60	-1.07	-3.18	-3.76	-2.79	-4.43	-0.32	4.45	-2.37
miR-346	104	3.00	-3.29	-2.06	-0.56	-2.58	-2.99	-4.23	-1.40	-0.34	1.35	2.08	-1.51
miR-34b	356	3.26	-3.84	-1.39	-3.14	-2.05	-3.21	-4.99	-1.99	-2.94	-1.48	5.42	-1.59
miR-361	184	1.14	-3.22	-0.67	-0.57	-1.78	-1.19	-4.26	-0.49	-3.27	-0.14	0.74	-2.38
miR-362	157	2.37	-3.71	-3.24	-1.87	-1.39	-1.96	-3.47	-1.78	1.65	-1.45	2.53	-1.88
miR-363	198	4.56	-5.39	-2.89	-3.00	-2.96	-4.53	-5.94	-4.14	-0.48	0.24	2.65	0.60
miR-365	218	4.71	-3.90	-3.17	-2.51	-2.49	-4.00	-4.94	-4.61	-0.45	-1.72	2.26	0.55
miR-369-3p	548	6.63	-6.45	-2.39	-3.61	-3.18	-5.96	-6.44	-6.18	-1.59	-0.51	3.19	-0.20
miR-370	283	1.99	-3.36	-0.34	-0.14	-1.45	-1.66	-2.67	-1.76	-1.06	1.52	3.07	-2.48
miR-374	550	5.26	-6.63	-2.66	-4.00	-2.74	-4.77	-6.56	-5.71	-2.78	-3.28	3.11	-0.41
miR-375	273	2.75	-4.32	-1.40	-2.51	-1.67	-1.24	-4.72	-2.48	-1.42	-0.42	3.68	-1.97
miR-376	209	2.40	-3.38	-0.52	-2.36	-2.22	-2.34	-4.13	-2.20	-1.30	-1.23	-0.09	-1.28
miR-376c	218	5.22	-4.09	-2.95	-4.42	-4.25	-4.15	-5.71	-3.80	-1.51	-2.73	2.71	-0.56
miR-377	378	3.85	-6.87	-2.82	-2.05	-3.58	-3.91	-5.02	-5.10	-1.81	-1.17	3.26	-2.39
miR-378	434	0.88	-2.12	1.12	-0.07	0.15	-0.36	-4.73	-2.13	-0.71	-1.45	2.65	-1.77
miR-378*	136	1.53	-2.93	-0.53	-2.35	-1.71	-1.59	-1.44	-1.48	-0.86	0.57	1.85	-0.43
miR-379	82	1.90	-2.25	-1.37	-1.52	-1.81	-1.39	-3.93	-2.50	-1.72	-2.43	2.83	-1.91
miR-380-5p	69	2.96	-0.97	-2.93	-2.15	-0.81	-3.19	-3.96	-3.10	-0.45	0.91	3.09	1.65
miR-381	868	6.84	-10.39	-5.26	-5.14	-5.63	-6.74	-11.03	-7.93	-4.41	-0.58	7.24	-2.13
miR-382	168	1.29	-1.80	-0.76	-0.88	-1.31	-1.67	-2.66	-1.96	-1.53	-0.72	1.77	0.32
miR-383	105	0.03	-0.88	1.31	-0.26	-0.48	-0.97	-0.44	-1.43	-1.49	-0.90	1.60	0.47
miR-384	271	3.69	-5.65	-2.21	-2.65	-2.41	-3.55	-5.72	-3.33	-1.13	-3.98	3.09	1.22
miR-409-3p	312	4.24	-6.23	-3.94	-4.70	-4.39	-4.15	-7.71	-3.28	-1.02	-0.70	1.86	-1.40
miR-409-5p	138	3.22	-4.98	-2.50	-1.86	-3.26	-3.82	-1.82	-3.59	-4.11	-1.72	1.92	-1.05
miR-410	679	7.33	-9.55	-4.38	-5.29	-4.83	-7.02	-8.00	-7.01	-1.52	-0.50	5.24	-2.09
miR-421	131	3.11	-4.69	-2.75	-4.28	-1.61	-1.97	-3.27	-2.81	0.79	-1.45	0.87	-0.05
miR-431	120	4.08	-3.78	-2.59	-2.25	-2.42	-3.56	-4.43	-3.53	-2.86	-1.81	1.08	1.67
miR-433-3p	253	-0.62	-2.68	1.12	0.26	0.43	0.82	-4.25	0.27	-1.28	-4.20	4.44	-1.75
miR-448	555	4.72	-7.42	-1.83	-2.25	-3.30	-4.63	-8.13	-6.59	-2.66	-1.55	4.63	-1.94
miR-450	274	5.83	-7.34	-3.27	-2.85	-4.37	-5.82	-5.96	-5.04	-2.38	-1.43	4.30	-0.83
miR-451	28	1.09	-1.39	-0.80	-0.14	-0.89	-0.94	-3.39	-0.99	-0.46	-2.20	0.05	1.68
miR-452	225	0.26	-0.87	1.23	-1.36	0.98	0.27	-1.88	-0.25	-2.85	-2.22	0.02	-1.10
miR-455	161	4.74	-5.32	-3.68	-4.35	-4.94	-4.62	-5.47	-4.12	-0.13	-2.03	2.13	-0.75
miR-485-3p	270	2.71	-4.34	-0.46	-1.32	-1.82	-2.87	-2.24	-3.46	-0.98	-3.52	1.14	1.15
miR-485-5p	275	5.53	-6.84	-2.17	-4.04	-5.94	-5.98	-5.13	-4.89	-0.24	0.06	5.44	-2.71
miR-486	142	1.81	-1.39	0.55	0.09	-1.91	-1.95	-2.47	-1.72	-1.85	-3.57	0.89	-1.34
miR-488	182	-0.07	-2.26	1.42	-0.52	0.01	-1.13	-3.12	-0.14	-1.22	-0.60	2.71	-2.12
miR-490	137	-0.09	-2.44	2.01	-0.17	0.47	-0.39	-1.20	-0.09	0.50	-2.91	3.07	0.30
miR-491	131	2.69	-2.20	-1.50	-1.99	-2.96	-2.82	-0.81	-2.45	0.47	0.65	2.68	-0.63
miR-493-5p	695	4.60	-8.09	-3.21	-3.39	-4.44	-4.32	-6.89	-6.19	-3.36	-2.10	6.22	-2.76
miR-494	441	2.16	-4.73	-0.26	-0.29	-0.23	-2.07	-4.98	-2.41	-0.14	-1.95	2.11	0.64
miR-495	879	4.32	-7.97	-3.11	-4.31	-3.57	-3.25	-7.57	-4.74	-3.92	-4.31	5.57	-1.50
miR-496	825	5.06	-8.98	-3.19	-4.21	-3.32	-3.80	-9.72	-5.30	-2.80	-3.18	5.46	-0.96
miR-499	307	1.46	-3.33	-1.16	-1.12	-1.12	-1.68	-4.28	-3.07	-2.48	-1.26	0.59	-2.15

miR-seed	nb targets	median diff	Normal NB42	Normal NB58	Normal NB60	Normal NB64	Normal NB69	Normal NB83	Normal NB87	Tumor BLC_T116	Tumor BLC_T118	Tumor BLC_T123	Tumor BLC_T129
miR-500	183	2.94	-4.04	-1.29	-2.43	-2.16	-2.52	-4.25	-2.46	-1.26	-0.37	2.47	0.41
miR-503	398	4.89	-4.79	-2.59	-2.87	-2.26	-4.13	-6.61	-4.34	-0.35	-1.97	4.27	-2.44
miR-504	162	4.44	-5.94	-2.70	-3.54	-3.30	-3.87	-5.09	-4.87	-1.15	-2.03	2.12	0.56
miR-505	391	2.97	-6.77	-1.84	-3.89	-3.10	-2.84	-6.65	-2.90	-1.38	-0.79	2.78	-0.28
miR-539	506	5.10	-6.73	-2.26	-4.81	-3.19	-4.52	-6.39	-5.61	-1.90	-3.57	5.14	-1.23
miR-542-3p	275	5.36	-5.34	-2.96	-5.33	-3.13	-5.16	-6.61	-4.65	-1.59	-0.86	3.28	-2.07
miR-543	624	2.63	-4.92	-0.93	-2.04	-1.18	-2.70	-6.07	-3.95	-3.39	-3.68	2.37	-0.60
miR-544	397	3.79	-5.23	-1.96	-2.94	-2.06	-4.47	-5.09	-3.61	-3.70	-2.43	3.46	-0.31
miR-7	394	3.60	-6.68	-2.20	-1.89	-2.93	-2.18	-5.78	-3.98	0.78	-0.88	5.72	-2.83
miR-9	1109	9.12	-11.43	-5.53	-6.34	-7.02	-8.46	-11.38	-10.15	-2.41	1.95	7.27	-2.61
miR-93.hd/291-3p/294/295/3(	585	6.19	-8.03	-3.02	-4.19	-3.79	-5.52	-8.34	-6.12	-1.86	-0.54	6.18	-1.21
miR-96	945	7.83	-8.97	-5.52	-6.39	-4.73	-8.96	-10.86	-6.99	-3.41	0.86	4.62	-3.76
miR-99/100	51	1.45	-2.51	0.53	-1.72	-1.86	-1.12	-0.82	-1.08	-0.84	1.20	1.51	1.82

Supplemental Table 3:

miR-seed	Tumor BLC_T130	Tumor BLC_T133	Tumor BLC_T134	Tumor BLC_T137	Tumor BLC_T140	Tumor BLC_T141	Tumor BLC_T143	Tumor BLC_T144	Tumor BLC_T146	Tumor BLC_T147	Tumor BLC_T149	Tumor BLC_T21	Tumor BLC_T38
Any-MiR	-12.29	13.72	12.06	2.12	0.17	-22.55	13.12	32.67	-3.74	-2.20	8.25	-15.45	4.81
let-7/98	-3.57	3.71	3.52	2.21	3.72	-4.64	2.58	5.81	-1.42	2.08	5.27	-3.25	2.89
miR-1/206	-2.04	1.68	6.17	1.98	3.42	-5.82	4.60	10.65	1.18	2.17	2.34	-1.41	3.21
miR-10	1.70	3.37	1.61	1.53	2.09	-3.16	-2.10	3.78	-2.38	-1.52	-0.20	-3.59	-0.81
miR-101	0.72	7.00	7.38	7.39	6.82	-8.54	2.51	10.41	1.25	5.05	5.48	-0.99	2.52
miR-103/107	-0.20	3.44	4.61	1.64	2.69	-5.80	1.25	7.82	-0.32	1.89	2.93	-2.88	0.14
miR-122	-1.92	0.53	3.34	-2.50	1.20	-2.92	1.30	6.10	1.34	-1.37	0.43	0.07	-1.92
miR-124.1	-3.56	3.86	2.63	4.10	2.81	-9.55	5.29	10.14	2.66	0.85	1.10	-4.65	1.72
miR-124.2/506	-3.69	5.68	4.20	2.99	4.56	-11.15	5.05	12.31	0.61	0.47	1.65	-4.75	2.63
miR-125/351	-2.06	3.28	3.88	0.04	1.71	-3.96	3.88	7.82	2.17	0.73	2.36	-3.46	2.38
miR-126/126-3p	-1.05	3.95	1.35	-1.97	0.58	-1.05	0.26	3.57	-1.14	0.25	-1.85	-3.10	-1.04
miR-128	-2.63	4.95	4.25	4.90	2.99	-6.90	2.41	8.26	-0.61	2.73	5.91	-0.06	2.47
miR-129-5p	0.87	3.91	3.50	3.34	6.52	-6.53	-0.84	6.34	2.57	0.27	2.74	1.42	1.31
miR-130/301	-1.81	2.52	4.86	7.29	6.71	-8.71	3.81	9.19	-0.23	3.24	4.15	1.76	3.70
miR-132/212	0.14	1.91	3.04	3.04	1.64	-6.14	0.38	8.11	0.59	1.51	0.68	-1.95	2.10
miR-133	-1.57	5.16	4.87	0.12	2.33	-3.39	0.19	8.13	0.40	-0.56	1.58	-3.08	1.39
miR-134	0.73	3.25	2.63	3.69	3.21	-3.98	1.15	3.95	1.18	3.28	2.49	-0.21	0.25
miR-135	-0.38	3.32	3.23	1.64	1.72	-6.46	2.57	7.90	-1.20	0.65	3.10	-1.77	-0.12
miR-136	-0.10	2.85	4.13	2.87	2.83	-2.04	1.29	4.88	0.32	0.76	3.78	1.74	-0.70
miR-137	-0.61	3.85	4.37	4.75	5.91	-6.91	3.64	5.04	0.29	1.42	3.82	-0.44	0.32
miR-138	-0.25	5.57	2.68	2.71	2.74	-5.57	2.67	6.12	0.46	0.18	3.29	-1.25	0.56
miR-139	-2.33	3.32	2.16	4.00	1.27	-5.93	1.76	4.76	0.72	0.22	1.07	-1.44	-1.12
miR-140	-0.01	3.06	1.84	1.43	0.20	-2.78	0.30	4.80	-0.67	3.17	1.10	-2.02	1.93
miR-141/200a	-0.15	5.43	5.28	5.28	5.11	-6.22	1.27	8.80	1.63	1.57	2.95	-1.46	2.33
miR-142-3p	-3.83	1.43	3.05	3.28	2.49	-3.74	4.37	7.39	-2.22	0.52	2.21	0.48	1.05
miR-142-5p	0.29	2.10	3.42	5.63	5.57	-7.55	3.41	7.53	-1.04	3.62	5.33	-0.57	1.32
miR-143	-0.31	2.60	1.34	4.40	2.51	-1.25	3.50	6.12	-0.40	3.66	4.41	2.21	0.42
miR-144	-1.02	5.22	6.72	7.18	4.80	-8.87	2.11	8.92	0.14	4.57	4.00	-1.66	2.58
miR-145	-1.71	3.33	3.59	2.59	0.31	-5.69	1.30	8.95	-2.34	-0.62	2.98	-0.92	1.45
miR-146	0.12	0.91	2.53	0.80	4.95	-1.49	-0.90	4.22	1.18	0.64	2.11	-0.92	1.67
miR-148/152	-2.44	5.30	3.31	3.23	5.12	-7.86	2.05	8.48	2.20	3.27	6.46	-2.21	1.64
miR-149	0.24	6.04	3.64	-1.61	0.74	-2.21	2.18	3.78	1.62	-1.10	1.11	-4.25	1.49
miR-15/16/195/424/497	-0.69	4.65	5.54	1.29	3.47	-8.40	2.05	10.30	-1.41	-0.10	5.05	-3.75	1.29
miR-150	-0.63	2.74	2.67	0.98	1.47	-1.62	-0.50	4.49	0.03	-1.20	0.73	-0.96	2.81
miR-151	-2.32	-0.04	0.79	4.93	-0.35	-2.54	-0.42	3.92	-2.28	1.14	0.44	-1.05	-0.42
miR-153	-1.44	5.80	3.78	2.91	3.66	-7.41	1.99	8.31	-0.28	3.36	2.35	-1.08	2.32
miR-155	-2.57	3.29	0.78	3.61	2.44	-5.34	0.24	5.64	-3.54	1.76	1.71	-2.41	1.84
miR-17-5p/20/93.mr/106/519	-0.37	1.27	4.67	5.34	5.74	-8.70	3.41	10.22	0.91	5.10	3.95	0.93	5.01
miR-18	0.85	0.23	1.68	6.30	5.72	-6.18	0.94	5.89	0.83	2.19	4.07	-0.49	4.35
miR-181	-1.12	3.70	7.05	8.60	4.90	-10.49	3.48	10.76	-0.81	3.52	6.09	-0.72	2.67
miR-182	-0.69	2.51	3.89	2.77	2.16	-8.25	3.27	9.94	-0.88	-0.31	3.61	-1.08	1.96
miR-183	-0.86	0.43	4.58	4.44	2.15	-6.71	1.72	6.42	-1.71	0.95	2.12	-0.93	1.19
miR-184	0.81	-0.42	1.73	-1.55	2.03	-0.08	0.83	1.82	0.34	0.47	0.91	0.18	2.51
miR-185	1.60	5.45	3.05	-0.73	3.02	-1.87	1.25	3.45	4.36	1.26	2.11	0.81	1.48
miR-186	-1.20	2.46	2.83	6.46	1.25	-9.61	3.61	9.84	-1.68	3.09	3.60	-0.30	-0.89
miR-188	-0.47	0.62	0.78	3.26	-0.80	-2.63	0.73	5.40	-0.66	1.76	0.83	-0.48	-0.34

miR-seed	Tumor BLC_T130	Tumor BLC_T133	Tumor BLC_T134	Tumor BLC_T137	Tumor BLC_T140	Tumor BLC_T141	Tumor BLC_T143	Tumor BLC_T144	Tumor BLC_T146	Tumor BLC_T147	Tumor BLC_T149	Tumor BLC_T21	Tumor BLC_T38
miR-19	-1.21	1.57	6.68	6.65	7.10	-9.60	3.44	12.42	-0.42	4.74	4.18	3.05	5.36
miR-190	0.77	2.62	3.57	2.94	2.09	-3.71	1.15	2.78	0.04	0.86	1.04	-1.77	-0.64
miR-191	1.51	3.31	2.08	0.06	1.96	-1.01	0.30	3.09	1.66	1.39	3.02	0.74	1.36
miR-192/215	1.23	1.43	3.37	2.96	3.56	-3.30	0.68	4.44	0.89	2.81	1.41	1.08	-0.48
miR-193	1.28	0.67	3.86	3.92	4.30	-3.14	0.51	5.17	-0.14	2.94	2.44	0.49	2.70
miR-194	-1.42	2.62	2.30	3.71	3.92	-8.30	0.14	4.78	-0.36	2.41	2.37	-2.05	0.83
miR-196	-0.97	-0.33	-0.03	2.69	0.77	-3.46	1.18	0.82	-0.73	1.20	1.84	0.87	-1.24
miR-199	1.31	5.50	2.25	3.85	3.92	-3.98	2.36	7.69	0.40	0.90	3.81	-1.26	3.14
miR-200b/429	-0.11	2.82	4.94	7.25	1.19	-8.61	3.34	10.64	-0.02	2.72	3.59	-0.19	1.95
miR-203.1	-0.05	1.41	3.76	5.97	4.85	-7.77	3.08	9.48	-1.34	2.77	3.18	-1.50	-0.06
miR-204/211	-0.94	3.26	2.81	3.89	3.00	-5.59	1.42	6.77	-1.34	0.44	1.12	-1.43	0.72
miR-205	0.46	6.03	2.88	4.19	2.96	-4.03	3.14	7.13	0.01	-0.69	2.00	-2.44	0.83
miR-208	-2.15	2.05	-0.59	6.15	-0.54	-2.88	1.47	4.21	0.18	1.73	2.39	0.38	-0.44
miR-21	-1.09	2.29	4.76	2.86	1.17	-7.26	-0.52	3.26	-1.34	1.73	2.20	-2.16	-0.24
miR-210	0.35	1.02	1.17	0.85	2.59	0.79	-0.62	-1.60	-0.49	-0.32	1.45	1.61	0.15
miR-214	-0.81	5.74	3.01	3.21	2.32	-3.47	3.80	6.53	1.23	-0.30	2.53	-0.93	1.31
miR-216	-0.21	3.56	1.49	2.24	1.33	-4.45	1.59	3.79	0.13	1.91	1.35	-2.10	-2.24
miR-217	1.19	3.94	0.80	4.84	2.56	-4.89	0.41	5.57	1.08	-0.76	4.89	-1.35	0.12
miR-218	0.56	4.28	1.87	4.20	3.11	-5.57	3.51	5.80	-1.90	2.37	3.11	0.40	0.47
miR-219	1.01	1.75	3.21	3.13	1.11	-3.58	3.62	4.36	0.56	2.59	2.28	0.91	4.17
miR-22	-1.07	2.83	1.66	1.68	1.17	-2.88	1.54	3.72	-1.13	0.70	3.47	-3.21	1.81
miR-221/222	0.03	1.66	2.83	5.25	1.96	-5.47	2.60	7.87	2.56	1.82	3.85	-0.08	3.85
miR-223	-0.93	1.60	3.16	2.45	2.02	-5.64	3.82	4.31	0.98	0.70	4.03	0.91	0.72
miR-224	-2.44	2.69	1.93	3.65	3.30	-4.61	-0.27	3.48	-1.17	1.40	0.13	-0.43	0.59
miR-23	-1.25	3.66	5.45	6.09	3.84	-8.83	2.15	7.92	-1.94	2.13	4.82	-1.04	1.14
miR-24	-0.36	4.70	5.49	1.44	2.56	-3.72	2.51	7.34	1.30	-1.26	2.98	-2.25	2.99
miR-24*	-0.67	0.52	-1.47	1.85	-0.31	-5.04	1.95	2.63	-0.63	1.75	1.60	-1.64	0.45
miR-25/32/92/363/367	-1.07	2.66	4.82	5.45	5.20	-8.13	3.53	9.25	1.44	5.19	4.18	-0.68	4.22
miR-26	-5.18	3.24	3.72	3.75	3.15	-7.38	3.85	8.85	-2.05	2.02	1.94	-1.89	1.99
miR-27	-2.02	6.39	3.36	6.50	3.18	-6.94	1.20	8.78	-1.37	2.54	6.77	-1.71	2.85
miR-28	0.86	1.87	1.39	-0.78	4.02	-1.72	-0.61	2.37	4.13	0.45	3.90	-0.44	1.12
miR-29	0.80	4.70	5.01	1.74	5.11	-5.80	-0.81	8.70	0.99	2.98	6.56	-1.04	4.04
miR-299-3p	1.09	3.25	5.02	-0.20	4.07	-1.93	1.32	4.05	2.52	2.75	1.57	0.34	1.96
miR-299-5p	-1.87	2.51	3.62	1.26	1.21	-5.37	4.15	5.62	-0.67	1.79	1.69	0.14	2.45
miR-30-3p	-1.61	2.95	1.95	3.96	1.28	-5.95	4.28	7.22	-2.14	4.25	2.89	1.65	-0.96
miR-30-5p	-0.70	2.76	4.89	8.87	4.81	-8.44	0.81	11.11	0.19	3.57	3.93	-1.44	4.83
miR-31	-1.09	2.90	2.18	-0.04	0.50	-4.23	3.91	5.71	1.40	2.05	3.67	0.49	0.29
miR-320	-0.29	1.78	2.40	3.12	1.08	-8.16	4.69	7.52	-0.98	1.66	3.79	-1.98	0.76
miR-323	-0.65	2.49	4.12	5.54	2.02	-8.95	1.75	6.01	-2.04	1.43	2.56	-0.79	0.08
miR-324-3p	-1.00	2.72	2.40	1.96	4.96	-1.91	1.43	4.91	0.63	1.46	2.27	0.47	-0.54
miR-324-5p	-2.00	2.67	2.05	-0.39	1.18	-2.74	1.84	4.10	1.63	-1.70	2.26	-2.08	-0.81
miR-325	-0.06	3.98	-0.22	2.62	0.23	-1.47	0.55	3.03	2.02	0.31	2.13	0.51	-0.26
miR-326	0.86	3.27	5.81	-1.36	2.13	-2.40	-1.23	4.32	2.69	-1.92	4.54	-1.29	4.57
miR-328	0.18	1.44	1.38	0.74	1.44	-2.97	-2.40	4.17	1.07	-1.38	3.35	-1.21	2.58
miR-329	-0.23	4.44	4.71	3.11	4.80	-5.04	-0.43	6.24	1.19	0.51	4.89	-1.83	0.73
miR-33	-0.23	1.26	0.82	5.02	3.41	-5.04	2.44	6.69	-0.36	2.82	1.99	0.94	2.04
miR-330	-0.61	2.75	5.38	2.56	2.38	-6.26	4.58	8.15	-0.22	2.49	2.54	-0.79	1.01
miR-331	-0.04	3.19	4.23	-1.89	1.59	-0.22	-0.23	4.61	2.29	-1.72	3.46	-0.86	1.69

miR-seed	Tumor BLC_T130	Tumor BLC_T133	Tumor BLC_T134	Tumor BLC_T137	Tumor BLC_T140	Tumor BLC_T141	Tumor BLC_T143	Tumor BLC_T144	Tumor BLC_T146	Tumor BLC_T147	Tumor BLC_T149	Tumor BLC_T21	Tumor BLC_T38
miR-335	-1.10	1.23	0.47	1.91	1.87	-3.54	2.10	3.58	-1.03	3.29	4.25	1.25	1.13
miR-338	-0.56	1.24	2.99	3.32	1.15	-5.03	0.53	4.77	1.37	-0.73	2.27	-0.47	1.92
miR-339	-0.74	1.42	2.31	1.83	3.48	-0.34	-1.60	3.85	-1.22	-1.12	1.62	0.22	1.64
miR-34/449	1.70	5.08	3.71	2.01	1.84	-1.59	-0.58	5.56	0.62	2.64	5.40	-3.40	2.24
miR-342	-1.83	1.29	2.35	2.05	1.57	-3.72	0.61	4.65	1.57	3.62	0.58	-0.07	2.86
miR-346	-0.19	1.35	3.51	0.03	1.53	-1.87	1.34	4.02	4.34	0.35	2.29	-0.34	1.94
miR-34b	0.12	3.19	3.88	2.82	6.39	-4.93	-1.06	7.01	-1.56	2.19	3.35	-0.52	1.62
miR-361	1.44	3.14	1.97	3.75	2.42	-1.53	2.47	3.59	0.26	0.34	2.50	-2.15	2.48
miR-362	0.74	4.84	2.59	1.77	1.41	-2.59	2.31	4.54	1.48	-1.02	1.91	-2.00	0.24
miR-363	1.80	2.05	4.00	3.98	3.93	-3.60	1.30	5.31	0.09	4.12	3.43	2.32	2.69
miR-365	-0.70	1.92	1.43	3.46	2.20	-4.88	1.63	4.28	-0.42	1.84	2.61	0.35	1.49
miR-369-3p	-1.16	3.11	4.37	6.77	2.89	-6.03	1.43	7.03	0.69	1.42	4.99	1.25	1.69
miR-370	0.45	3.49	2.83	-0.59	2.26	-3.08	-1.34	4.74	1.86	-1.59	0.98	-2.27	2.06
miR-374	-2.33	2.23	3.78	6.34	5.14	-6.75	0.30	5.54	0.05	1.01	4.20	0.95	0.79
miR-375	-3.00	3.08	1.19	2.03	1.22	-4.58	2.51	6.35	0.29	0.92	3.45	-1.29	-1.64
miR-376	-1.94	-0.56	3.44	3.48	1.03	-5.43	0.50	4.55	-1.42	0.95	3.12	-0.92	1.02
miR-376c	2.43	1.20	2.23	3.19	2.54	-6.33	3.91	7.07	0.25	2.21	4.50	-0.59	1.54
miR-377	-0.88	4.74	3.85	3.48	2.91	-4.36	-0.15	6.93	0.97	2.45	4.00	-0.83	3.08
miR-378	-3.02	1.75	-0.19	2.72	0.61	-6.35	1.41	6.38	-3.88	-1.02	1.62	-3.23	-0.43
miR-378*	1.53	3.23	2.50	1.91	3.36	-1.68	-0.30	0.18	-0.74	1.23	2.31	-0.29	-0.41
miR-379	1.16	-0.42	2.06	0.94	0.09	-2.43	2.00	2.37	-1.29	0.08	0.07	-0.93	-0.89
miR-380-5p	-1.93	-0.39	1.86	4.27	2.13	-4.43	-0.49	2.29	-1.01	0.05	0.02	-1.29	2.43
miR-381	-2.72	5.23	5.35	10.17	5.25	-9.85	2.21	10.12	-1.66	3.61	5.31	-2.52	3.20
miR-382	-0.54	1.77	1.81	2.61	-0.43	-1.23	-1.06	4.29	-1.75	-0.45	1.83	0.68	-1.24
miR-383	-0.17	2.64	1.28	3.31	1.31	-0.71	2.69	2.92	-0.03	-0.62	3.63	-0.57	1.18
miR-384	-0.57	1.56	2.86	4.71	0.44	-6.04	0.88	3.83	-2.57	2.29	2.33	-1.07	1.47
miR-409-3p	-1.85	-0.61	3.85	3.66	1.91	-5.38	4.10	7.87	-0.26	1.65	1.85	-1.37	2.47
miR-409-5p	-0.09	2.63	5.08	2.60	4.81	-3.84	-0.37	5.02	0.02	0.07	2.54	-0.23	1.09
miR-410	-0.95	2.27	5.45	4.20	4.36	-9.92	4.11	9.29	1.13	0.37	2.38	-0.20	1.98
miR-421	-5.01	1.68	1.06	2.25	-0.21	-1.05	0.18	3.49	-2.27	-1.26	2.03	1.79	-1.05
miR-431	0.34	2.79	1.33	1.42	1.24	-2.14	1.87	0.43	-0.86	1.45	2.16	-0.78	0.24
miR-433-3p	-3.73	1.86	1.58	3.33	0.10	-4.30	0.89	6.08	-3.38	-0.17	-0.03	-2.95	-0.54
miR-448	-2.15	4.16	2.44	3.43	2.94	-7.61	1.86	8.76	-0.06	2.96	2.29	-1.98	2.31
miR-450	1.38	3.11	4.17	3.74	4.29	-5.52	-1.06	5.16	1.38	1.15	2.76	1.91	2.92
miR-451	-2.98	-1.20	0.96	-0.14	2.36	-1.88	-0.77	1.95	-2.05	0.70	-2.21	-0.44	1.53
miR-452	-1.95	-0.05	-0.87	3.34	1.64	-3.42	1.66	3.51	-1.65	-0.45	0.00	-1.90	-0.93
miR-455	0.22	3.43	3.66	5.12	4.45	-3.61	3.06	5.92	3.21	0.02	5.28	0.59	1.42
miR-485-3p	0.35	2.10	2.80	3.74	3.38	-4.60	-0.18	4.31	-0.71	2.30	4.14	-1.03	1.91
miR-485-5p	2.85	6.27	3.16	-1.64	3.87	-3.12	2.79	4.98	1.76	-0.71	2.28	-1.55	2.65
miR-486	0.80	2.62	0.70	1.74	1.65	-4.23	-0.35	3.48	-0.90	0.00	1.06	-3.64	-0.51
miR-488	-3.16	0.20	2.02	1.90	-0.94	-3.78	1.23	2.89	-1.82	-1.17	1.55	-1.48	0.25
miR-490	-2.80	-0.14	1.04	1.04	1.58	-4.40	-0.60	3.83	-1.13	-1.64	1.55	-2.59	-2.29
miR-491	0.84	3.90	2.70	-1.20	1.58	-0.81	-1.53	3.02	1.32	-1.79	1.65	-1.26	1.75
miR-493-5p	-0.96	5.55	7.38	6.08	5.38	-8.24	-0.24	8.94	0.55	5.68	4.14	-1.81	1.70
miR-494	-2.33	2.11	3.02	4.49	4.14	-4.67	1.59	7.39	-1.27	2.90	2.84	0.32	-0.98
miR-495	-1.68	5.11	4.12	5.99	4.43	-7.75	2.05	7.45	0.64	3.52	2.84	-0.91	-0.57
miR-496	-2.43	4.69	3.48	10.62	6.22	-9.82	4.87	10.60	0.00	5.85	4.03	-1.83	1.58
miR-499	-3.18	1.48	0.72	6.30	-0.42	-4.24	1.25	5.04	-0.90	0.69	2.12	-0.66	-0.97

miR-seed	Tumor BLC_T130	Tumor BLC_T133	Tumor BLC_T134	Tumor BLC_T137	Tumor BLC_T140	Tumor BLC_T141	Tumor BLC_T143	Tumor BLC_T144	Tumor BLC_T146	Tumor BLC_T147	Tumor BLC_T149	Tumor BLC_T21	Tumor BLC_T38
miR-500	1.15	1.71	2.78	4.37	1.34	-3.45	0.95	3.65	0.62	0.07	3.43	2.50	-1.58
miR-503	0.22	2.12	2.44	2.36	1.77	-5.98	0.44	5.07	-2.03	1.32	3.17	-1.11	0.86
miR-504	0.78	2.57	3.43	-0.85	2.76	-2.52	1.48	3.53	0.38	0.58	1.41	0.47	0.94
miR-505	0.43	3.99	4.69	6.42	3.90	-6.04	5.37	7.95	-0.88	0.68	5.09	-0.74	0.38
miR-539	-0.65	3.40	4.43	3.34	2.61	-4.81	2.94	6.50	-1.84	0.33	1.62	-1.28	1.69
miR-542-3p	-0.85	1.27	4.94	3.84	3.17	-5.90	1.49	5.57	-1.32	1.98	2.46	-0.94	1.09
miR-543	-1.85	3.12	4.01	6.68	2.71	-9.15	0.69	8.18	-1.91	4.08	2.62	-1.71	-1.29
miR-544	-0.44	2.50	4.09	3.98	3.59	-6.12	1.66	7.03	-1.14	1.50	3.59	-0.84	-0.28
miR-7	-0.88	4.75	3.18	5.40	3.27	-5.95	1.48	4.83	0.28	1.32	5.40	-0.05	2.27
miR-9	-0.57	3.03	5.98	2.38	4.72	-10.53	6.21	10.04	3.64	2.50	3.80	-1.27	5.16
miR-93.hd/291-3p/294/295/30	-0.30	3.50	3.74	4.27	3.83	-9.35	2.81	6.80	1.42	3.64	3.41	-0.60	5.27
miR-96	-0.48	2.26	3.93	1.38	2.77	-8.79	5.15	8.67	0.00	0.36	3.28	-2.50	2.84
miR-99/100	-0.56	1.79	2.08	0.35	0.26	-1.64	0.88	2.07	-1.31	1.40	1.28	1.36	0.99

Supplemental Table 3:

miR-seed	Tumor BLC_T56	Tumor BRCA1_T151	Tumor BRCA1_T152	Tumor BRCA1_T183	Tumor others_T115	Tumor others_T117	Tumor others_T119	Tumor others_T145	Tumor others_T161	Tumor others_T162	Tumor others_T175	Tumor others_T178	Tumor others_T30
Any-MiR	34.42	23.15	-1.68	-4.27	0.41	8.57	-4.32	2.75	-7.71	11.85	-12.35	-0.45	-6.72
let-7/98	8.11	3.38	-0.03	-1.52	-1.69	1.67	1.76	1.92	-2.98	2.54	-1.93	1.72	-3.54
miR-1/206	11.56	3.86	-0.73	-2.28	-1.10	1.22	-3.49	1.66	-3.90	4.50	-3.98	-0.37	-1.90
miR-10	4.06	5.98	0.32	0.55	-0.85	1.74	2.72	2.99	0.25	3.99	-1.84	0.41	-0.68
miR-101	10.93	3.19	-6.28	-0.49	-4.72	-0.30	-3.00	5.21	-4.86	2.58	-4.82	-1.79	-3.46
miR-103/107	6.35	5.53	-1.34	0.44	0.69	-0.45	-0.35	1.84	-3.83	3.91	-3.69	-1.35	-0.55
miR-122	4.65	0.82	0.90	-3.18	0.38	2.21	-0.85	0.15	-3.83	0.74	0.19	-0.25	-0.04
miR-124.1	10.41	9.03	-1.26	0.60	-0.45	2.43	-3.38	4.04	-1.20	4.02	-2.95	0.32	-2.39
miR-124.2/506	12.67	10.47	-1.18	1.81	0.46	3.59	-2.31	2.74	-0.88	5.28	-5.14	0.61	-2.95
miR-125/351	6.88	6.56	3.34	-1.58	-0.97	1.41	1.44	1.30	-1.82	7.38	-5.62	-0.74	-2.15
miR-126/126-3p	1.07	3.92	0.37	-0.16	2.19	1.82	1.20	-1.18	0.58	2.03	-2.60	-1.17	-0.66
miR-128	9.72	4.66	1.20	-2.09	-1.67	0.39	-0.81	1.25	-3.59	2.83	-4.46	-1.28	-0.80
miR-129-5p	6.82	3.11	-1.57	-1.43	0.33	-0.33	-1.48	2.63	-2.39	5.91	-3.63	-1.91	0.10
miR-130/301	9.05	3.07	-2.12	0.58	-2.70	-0.79	-1.36	4.20	-2.34	3.29	-2.98	-0.89	-2.93
miR-132/212	9.04	4.03	-2.84	1.81	-0.92	-2.07	0.08	3.51	-1.74	2.54	-2.66	-3.60	-1.34
miR-133	5.44	5.20	1.16	-1.72	0.89	2.57	0.36	0.20	-2.82	4.66	-3.97	-0.17	-0.44
miR-134	3.75	2.85	-0.70	0.57	0.05	0.09	-1.09	0.91	-0.89	1.47	-1.44	0.93	-2.34
miR-135	6.33	3.23	-3.58	-0.19	1.30	0.05	-1.45	3.47	-1.82	3.60	-3.71	-1.30	-2.15
miR-136	3.97	1.22	-0.37	-2.55	0.08	1.76	-0.62	-0.01	-4.87	-0.81	-2.70	-1.22	-2.92
miR-137	6.35	3.56	-1.41	0.29	-2.10	1.13	-1.57	3.89	-1.15	2.72	-1.92	-0.55	-4.34
miR-138	3.97	5.37	1.31	-0.41	0.71	1.83	0.77	3.59	-2.11	4.38	-3.55	0.33	-2.17
miR-139	9.31	2.96	-2.01	-1.50	-5.58	-1.42	-2.54	-0.21	-2.89	2.55	-2.14	-1.33	-1.95
miR-140	3.58	2.90	-0.38	1.20	1.78	0.70	-0.79	1.93	0.43	2.32	-0.02	-2.74	-0.56
miR-141/200a	11.82	3.18	-2.12	-0.51	-0.11	0.13	-0.17	2.96	-3.89	2.16	-2.15	-1.04	-3.57
miR-142-3p	8.29	1.05	-1.25	-0.91	-1.34	-2.12	-2.46	-1.06	-1.05	1.31	-2.24	-1.99	-1.97
miR-142-5p	9.98	3.45	-2.73	1.44	-1.06	-0.74	-2.12	2.84	-3.82	3.62	-2.22	-0.78	-3.76
miR-143	6.79	2.79	1.19	-0.33	-1.94	1.56	-2.88	1.84	-3.22	2.79	-3.65	-1.21	-2.43
miR-144	11.71	3.26	-5.86	0.97	-4.56	-0.86	-3.11	4.75	-3.04	1.72	-4.91	-1.71	-4.00
miR-145	8.13	7.58	-1.24	-1.58	-0.97	-1.20	-1.03	-0.10	-1.75	3.01	-1.60	-0.84	-0.32
miR-146	1.96	0.36	-2.33	0.41	-0.84	0.42	-1.15	2.86	-0.19	4.35	-1.26	1.60	-1.25
miR-148/152	6.81	5.14	-1.55	-0.37	-1.88	2.17	-1.53	1.68	-3.28	3.04	-3.60	-2.27	-3.82
miR-149	4.42	4.83	2.09	-3.17	1.80	3.67	1.11	2.03	-2.79	4.03	-3.99	-0.28	-1.62
miR-15/16/195/424/497	8.07	9.40	-0.87	-1.40	2.73	4.67	-1.83	3.07	-3.00	4.87	-2.81	-0.94	-1.45
miR-150	5.80	2.96	2.31	-1.69	1.90	0.99	-0.82	1.24	-1.20	2.58	-1.07	-2.24	-1.27
miR-151	2.14	0.95	-3.97	1.87	1.04	-0.19	1.60	2.02	0.25	1.23	2.43	-1.39	-1.08
miR-153	7.09	6.32	-3.41	-0.43	-2.01	-1.05	-1.67	4.77	-2.97	5.17	-5.03	-0.75	-0.38
miR-155	5.72	5.55	-3.79	1.30	-0.62	-1.42	-0.20	1.61	-0.06	2.40	-1.49	0.37	-1.95
miR-17-5p/20/93.mr/106/519.	10.91	7.32	-1.44	-1.62	-0.28	-0.50	-1.09	2.00	-4.02	3.22	-4.70	-2.32	-1.12
miR-18	4.15	1.66	-2.42	0.61	-0.34	0.60	0.17	3.93	-0.95	1.66	-1.99	-0.90	-1.29
miR-181	10.17	4.74	-4.21	0.84	-3.65	0.75	-3.60	3.39	-4.35	2.95	-1.36	-2.69	-3.90
miR-182	11.76	6.12	-2.38	1.70	-0.45	0.01	-3.13	3.75	-4.33	5.17	-0.85	-1.18	-1.20
miR-183	7.89	2.59	-3.44	1.04	-1.00	-0.48	-0.63	2.71	-1.63	3.90	0.33	0.27	-3.26
miR-184	3.00	3.72	0.48	-0.14	0.33	0.91	0.68	0.89	-0.14	2.35	-1.19	2.45	-1.02
miR-185	2.86	2.20	0.53	-2.04	-0.73	1.07	0.53	1.38	-2.05	2.79	-2.48	0.98	-0.82
miR-186	9.80	2.81	-5.03	1.16	-3.59	-2.72	-2.37	4.28	-5.95	0.49	-1.03	-0.36	-6.15
miR-188	5.93	1.76	1.76	1.16	0.74	-2.43	0.52	1.18	0.29	-0.37	-1.90	-1.68	-2.25

miR-seed	Tumor BLC_T56	Tumor BRCA1_T151	Tumor BRCA1_T152	Tumor BRCA1_T183	Tumor others_T115	Tumor others_T117	Tumor others_T119	Tumor others_T145	Tumor others_T161	Tumor others_T162	Tumor others_T175	Tumor others_T178	Tumor others_T30
miR-19	11.99	4.17	-4.05	-0.04	-1.34	-0.06	-2.21	2.42	-5.28	2.07	-1.48	-0.53	-3.40
miR-190	2.44	0.42	-3.12	1.60	-2.70	-0.94	-1.66	1.96	-0.72	3.61	-2.02	-0.47	-1.45
miR-191	2.72	2.84	1.59	-2.38	-0.87	2.05	-0.12	0.20	-2.23	-0.14	-1.44	1.34	-1.94
miR-192/215	4.86	2.30	-2.59	-1.52	-1.73	-1.97	-0.85	1.25	-1.95	0.67	1.82	-0.67	0.13
miR-193	4.56	2.69	-0.42	-2.01	-1.77	-0.70	1.03	1.97	0.18	3.42	-2.19	-3.53	-0.87
miR-194	7.13	3.80	-4.67	1.71	-0.27	1.38	-2.47	4.29	-2.44	3.13	-2.64	-1.73	-1.80
miR-196	5.32	0.24	-1.67	0.19	1.22	-0.46	-1.18	0.74	-1.19	2.70	-0.61	1.86	-0.15
miR-199	6.78	4.10	-0.80	-1.51	-1.28	0.31	0.15	2.29	-4.82	5.01	-3.47	-2.40	-3.72
miR-200b/429	13.02	3.13	-2.91	0.71	-2.74	-1.46	-3.16	2.55	-2.04	3.69	-0.30	-1.86	-4.41
miR-203.1	9.85	2.03	-3.31	1.42	-3.17	-1.82	-3.50	5.01	-3.22	0.89	-0.54	0.05	-2.06
miR-204/211	6.65	5.04	-0.36	1.36	1.79	-0.20	-1.19	2.00	-1.64	3.47	-4.08	-0.83	-2.39
miR-205	8.94	3.65	-0.05	-1.36	1.22	0.69	-1.25	0.12	-4.45	1.25	-2.56	-2.12	-3.61
miR-208	3.44	1.53	0.38	0.65	-2.40	-2.16	-1.83	2.12	-1.87	0.80	-0.36	0.40	-0.74
miR-21	5.93	3.23	-4.05	2.17	1.71	0.34	1.47	1.86	-1.66	0.84	-0.42	0.41	-1.41
miR-210	-0.31	0.86	2.18	0.30	-0.41	1.51	0.63	-0.27	-2.11	-0.23	0.03	0.67	-0.01
miR-214	5.69	5.30	2.76	-3.04	-0.55	2.05	-1.99	0.25	-4.14	2.09	-4.68	-0.75	-2.97
miR-216	4.41	2.51	-0.96	0.52	0.33	0.47	1.77	3.80	-0.10	1.69	-0.50	-0.52	-2.41
miR-217	4.97	1.49	-3.24	-0.73	-2.15	-1.83	-1.74	4.20	-2.38	0.09	-2.38	-3.19	-5.21
miR-218	7.20	4.23	-2.32	0.51	-0.30	1.31	-0.33	2.96	-3.37	2.84	-3.20	-1.49	-3.74
miR-219	6.52	2.57	-1.55	0.94	0.26	-1.08	-2.62	1.41	-1.81	1.09	-2.17	-1.18	-1.51
miR-22	6.09	5.70	0.39	-0.86	1.77	3.26	1.16	2.18	-2.13	4.33	-1.74	1.75	-0.45
miR-221/222	7.74	2.98	-2.92	1.96	-1.35	-1.88	-0.64	2.64	-1.30	1.53	-3.04	-0.92	-3.74
miR-223	5.72	1.74	-2.53	2.43	-1.87	-0.93	-0.39	1.70	-1.42	2.29	-1.18	-1.72	-2.49
miR-224	6.84	4.94	-0.30	0.66	-0.94	-0.51	-2.18	4.57	-1.54	3.38	0.53	-1.19	-2.95
miR-23	9.58	5.39	-2.97	1.84	-1.34	-0.90	-0.96	4.24	-3.15	2.79	-2.92	0.56	-3.62
miR-24	5.69	5.26	1.62	-3.74	0.67	0.35	0.92	2.58	-2.19	2.57	-4.59	0.65	-2.42
miR-24*	3.81	1.58	-4.12	0.56	-0.45	-0.83	-1.70	1.85	-3.41	-0.02	-0.10	-0.35	-3.48
miR-25/32/92/363/367	9.11	4.07	-4.00	-1.59	-2.41	-1.24	-1.86	2.89	-2.29	2.41	-2.59	-1.45	-2.87
miR-26	11.69	4.21	-3.96	-0.08	-3.30	-2.36	-3.49	0.51	-3.59	0.92	-2.62	-0.69	-5.47
miR-27	10.43	6.09	0.90	-1.10	-0.78	0.06	-0.88	1.62	-4.02	2.79	-5.37	-1.76	-0.54
miR-28	2.83	2.48	2.08	-0.58	-0.04	3.79	1.18	2.76	0.43	3.54	-1.64	0.59	-0.67
miR-29	8.55	5.20	0.57	-1.13	0.34	1.51	0.85	1.62	-0.89	3.57	-7.57	-2.84	-3.99
miR-299-3p	3.41	3.82	0.43	-1.39	-1.20	-0.85	-1.44	-0.64	-3.28	1.57	-1.00	-1.65	-1.02
miR-299-5p	4.91	4.74	-2.35	0.63	0.83	0.21	-1.34	3.17	-4.79	1.87	-3.40	0.51	-1.10
miR-30-3p	8.48	2.87	-2.55	2.36	-2.49	-3.62	-2.37	3.52	-2.59	1.11	-0.72	-0.89	-3.49
miR-30-5p	10.61	4.54	-3.57	0.38	-1.63	-1.49	-3.02	2.84	-3.92	3.78	-4.75	-2.04	-3.63
miR-31	5.83	2.17	0.38	1.41	0.31	0.69	-0.54	0.11	-1.18	2.60	-0.24	2.44	-1.19
miR-320	10.97	6.03	-4.81	1.56	-2.19	0.04	-0.64	2.63	-4.08	2.72	-1.13	-1.01	-3.24
miR-323	9.42	5.00	-3.56	2.77	-0.79	-0.85	-1.84	4.07	-2.59	1.97	-2.32	-0.63	-2.94
miR-324-3p	4.76	1.05	0.69	-2.96	-1.06	-2.09	-2.14	2.58	-2.03	1.74	-3.56	-2.70	0.15
miR-324-5p	5.04	1.14	-2.21	-1.48	-2.96	-0.24	-1.41	2.54	-3.04	1.42	-2.68	-0.86	-0.67
miR-325	6.27	0.88	-0.92	-0.59	-0.08	-1.62	0.04	0.85	-2.90	-0.10	-0.10	-2.05	-1.70
miR-326	3.01	2.86	2.74	-3.47	3.05	2.66	0.27	2.25	-0.70	4.85	-2.86	-1.42	-1.05
miR-328	3.43	1.62	0.17	0.14	0.09	1.43	0.28	0.45	-1.24	0.91	-0.33	1.36	-1.88
miR-329	8.10	4.33	-1.37	-0.29	-1.28	0.11	-0.51	3.04	-2.64	3.03	-3.77	-1.19	-2.34
miR-33	4.80	1.89	-1.92	1.28	-2.10	-3.08	-1.73	0.88	-1.69	0.57	-3.60	-3.62	-1.52
miR-330	11.01	4.36	-1.08	-0.44	-0.90	-1.38	-1.01	2.63	-3.38	1.09	-2.92	2.04	-2.23
miR-331	-0.19	1.94	4.11	-4.52	1.19	3.44	1.56	0.69	-1.56	5.48	-3.70	-1.02	-0.57

miR-seed	Tumor BLC_T56	Tumor BRCA1_T151	Tumor BRCA1_T152	Tumor BRCA1_T183	Tumor others_T115	Tumor others_T117	Tumor others_T119	Tumor others_T145	Tumor others_T161	Tumor others_T162	Tumor others_T175	Tumor others_T178	Tumor others_T30
miR-335	5.76	2.31	0.05	1.45	0.31	0.80	-0.12	0.95	-2.82	-0.50	0.55	-0.30	-1.60
miR-338	6.26	1.97	-1.15	-0.17	-0.44	0.76	-1.30	1.03	-3.16	1.23	0.00	0.68	-0.57
miR-339	2.68	2.03	0.96	-0.83	0.86	-0.19	1.08	1.57	-0.72	1.68	-2.54	0.08	-1.63
miR-34/449	5.17	5.52	1.77	-0.53	0.65	4.45	-0.02	1.56	-4.80	4.26	-3.06	-0.63	-2.84
miR-342	6.60	5.15	-1.16	0.03	-1.23	-0.12	-0.91	1.53	0.45	2.20	-5.11	-3.69	-0.02
miR-346	1.19	1.33	0.49	-2.14	-0.69	0.89	-0.43	1.05	-0.83	1.88	-2.32	-1.19	-1.80
miR-34b	4.58	3.21	-1.06	0.63	-1.95	1.44	-1.37	3.43	-3.90	3.27	-4.38	0.12	-0.74
miR-361	4.21	2.03	-2.16	-1.31	-0.94	-0.60	-1.70	-0.06	-3.92	1.22	-0.65	-2.42	-4.04
miR-362	5.80	0.49	-1.07	0.74	-0.89	0.73	1.04	0.74	-3.39	1.02	-0.06	-0.51	-1.76
miR-363	4.23	2.91	-1.94	0.98	-0.46	-0.78	-1.18	3.23	-2.58	0.22	-1.11	-1.28	-2.06
miR-365	4.64	2.69	-2.42	1.29	0.09	-0.24	-0.83	3.35	-2.73	1.57	-0.43	-0.06	0.04
miR-369-3p	7.39	3.19	-3.87	-0.63	-0.79	0.17	-1.64	2.22	-1.75	0.65	-0.86	-3.79	-3.13
miR-370	2.04	2.83	0.47	-2.24	0.59	2.44	-1.30	-0.96	-0.44	3.65	-5.38	-0.08	-0.55
miR-374	5.18	4.84	-3.87	0.42	-0.81	0.57	-0.95	4.57	-0.65	2.90	-0.92	-1.95	-2.18
miR-375	4.14	2.95	-3.37	0.25	-2.12	-0.81	-1.44	2.93	-1.30	1.00	-2.87	-0.85	-2.35
miR-376	5.60	0.91	-2.79	1.73	-2.38	-1.78	-3.66	3.43	-0.98	0.57	-1.79	0.20	-0.86
miR-376c	4.38	3.07	-3.67	2.52	-1.44	-1.53	-1.64	2.43	-1.14	1.13	-1.79	-0.20	-1.40
miR-377	6.70	3.07	-1.60	-0.20	-0.41	0.81	-0.70	1.11	-2.83	4.44	-3.63	0.04	-2.42
miR-378	6.35	3.03	-3.06	2.51	0.59	0.46	-0.74	2.23	-2.54	1.65	-3.07	3.09	-1.17
miR-378*	2.35	0.83	-1.74	-0.78	2.02	3.97	0.64	2.87	-2.17	2.52	-1.92	0.72	-0.77
miR-379	3.04	2.58	-0.82	-0.15	-1.32	-1.79	-1.66	2.60	0.95	1.16	-0.15	0.38	1.40
miR-380-5p	1.19	0.87	-0.30	-1.00	1.98	-0.37	0.76	1.32	-0.39	-0.63	0.28	0.04	-0.68
miR-381	10.48	7.41	-3.60	1.07	-3.43	-0.28	-4.15	4.63	-2.51	3.12	-2.10	-2.08	-5.57
miR-382	6.27	1.06	-1.56	-1.08	-1.79	-2.36	-0.33	0.91	-2.20	1.27	-0.99	-0.92	0.18
miR-383	2.99	0.98	-1.33	-1.10	-2.09	-0.88	-2.66	-1.00	-0.34	1.44	-2.78	-2.36	-2.66
miR-384	7.00	1.49	-1.05	0.95	-1.32	0.23	-0.40	1.90	-2.74	0.52	0.28	0.12	-1.42
miR-409-3p	7.67	3.08	-4.50	1.72	-0.50	-0.28	-2.71	2.73	-2.63	1.82	-0.36	1.04	-0.71
miR-409-5p	4.29	1.29	-1.09	-0.62	-2.37	0.70	-1.18	3.05	-1.79	2.27	-1.29	-0.20	-1.08
miR-410	10.82	3.46	-4.47	0.27	-0.61	-0.49	-1.41	4.34	-3.56	6.43	0.04	-1.47	-1.60
miR-421	3.46	3.30	-1.89	1.49	-0.08	-0.06	-0.98	1.50	0.77	1.40	-2.02	-1.23	-0.23
miR-431	1.34	2.97	-0.91	1.64	-0.20	2.52	-0.42	3.32	-0.67	3.29	0.65	-1.30	-0.13
miR-433-3p	8.65	3.07	-2.19	1.28	-1.77	-1.07	-0.88	0.12	-1.82	0.23	-2.59	-1.50	-3.75
miR-448	6.74	4.72	-4.41	0.24	-1.35	-1.01	-1.83	4.61	-2.18	4.89	-3.39	-0.47	-0.19
miR-450	5.47	3.06	-1.94	-0.79	0.85	-1.29	-2.60	1.25	-2.90	3.22	-1.55	-1.56	-0.91
miR-451	3.86	2.21	-1.82	2.34	0.41	-0.53	-0.25	0.26	-2.11	2.02	1.99	-1.91	1.82
miR-452	4.34	1.01	-3.38	1.35	-0.21	-1.69	0.89	0.45	-0.31	0.34	0.08	-0.58	-1.44
miR-455	3.64	1.53	-1.52	-1.14	-1.04	-0.44	-2.00	0.48	-1.78	3.45	-3.54	-0.79	-3.25
miR-485-3p	5.86	4.30	-0.91	0.58	-3.32	-0.61	-0.34	0.74	-1.60	2.57	-1.71	0.04	-2.10
miR-485-5p	3.91	4.76	1.00	-2.59	1.72	5.17	0.60	2.55	-1.37	4.45	-3.46	-1.53	-0.50
miR-486	4.51	2.36	-3.59	-0.05	2.55	0.28	1.05	4.32	-0.21	4.39	0.00	-0.01	-1.38
miR-488	4.04	1.87	-2.18	2.39	-1.26	-0.65	-1.24	2.94	-2.62	0.69	-0.09	-1.70	-2.26
miR-490	3.77	4.74	-2.37	-0.38	-0.04	-1.38	-1.35	2.08	0.74	2.52	-2.69	-2.00	-0.56
miR-491	0.15	4.71	1.07	-2.26	-0.17	1.05	-0.87	-0.01	-0.75	4.30	-4.21	-2.05	2.69
miR-493-5p	10.65	6.32	-2.94	1.15	-5.24	-0.58	-4.19	2.93	-5.71	2.23	-4.79	-0.80	-5.90
miR-494	7.00	2.19	-1.50	-1.30	-3.30	-1.85	-2.74	3.49	-2.80	1.54	-3.88	-0.90	-2.39
miR-495	12.56	2.89	-4.54	-0.03	-2.15	0.05	-3.65	3.01	-3.65	5.53	-3.31	-3.38	-3.40
miR-496	10.36	2.46	-5.98	1.84	-5.89	-0.95	-5.44	4.33	-3.96	2.05	0.28	-1.31	-7.10
miR-499	4.53	2.97	-0.67	0.74	-2.73	-1.30	-0.88	2.55	-2.54	1.64	-0.38	0.60	-0.05

miR-seed	Tumor BLC_T56	Tumor BRCA1_T151	Tumor BRCA1_T152	Tumor BRCA1_T183	Tumor others_T115	Tumor others_T117	Tumor others_T119	Tumor others_T145	Tumor others_T161	Tumor others_T162	Tumor others_T175	Tumor others_T178	Tumor others_T30
miR-500	3.52	2.76	-0.56	-0.81	-1.74	0.54	-1.42	1.99	-0.88	-0.47	-1.77	-0.10	-2.00
miR-503	4.58	4.95	-0.29	1.04	2.31	2.43	-0.11	1.73	-2.47	2.55	-0.55	-0.29	-1.22
miR-504	1.64	4.06	2.67	-0.51	-0.33	2.32	1.50	1.98	-0.02	2.39	-2.00	1.48	-1.17
miR-505	10.55	2.41	-1.83	0.57	-3.48	0.02	-1.59	0.43	-2.68	-1.33	-1.65	-3.15	-3.83
miR-539	6.62	3.81	-3.01	0.98	-0.22	1.17	0.26	2.37	-1.24	1.71	-2.52	-1.64	-0.52
miR-542-3p	5.48	4.41	-2.43	-0.20	1.23	1.10	-0.08	4.30	-0.15	3.59	-2.15	-0.45	-0.98
miR-543	8.63	5.47	-6.15	3.45	-1.70	0.45	-1.58	3.37	-2.44	1.54	-3.25	-1.45	-3.26
miR-544	6.59	3.00	-2.37	1.84	0.01	1.86	-1.32	1.97	-0.88	3.32	-1.59	0.36	-2.36
miR-7	7.37	1.93	-1.36	0.94	-0.41	1.91	0.69	1.41	-4.26	1.47	-2.83	-1.44	-4.07
miR-9	10.63	7.86	-1.64	-0.57	-0.60	1.80	-1.41	4.93	-2.64	5.07	-4.31	-1.75	-3.08
miR-93.hd/291-3p/294/295/30	6.87	5.22	-2.40	1.18	-2.03	-0.36	-0.42	2.72	-3.48	1.47	-2.15	-2.61	-0.81
miR-96	11.01	6.56	-1.91	0.81	-0.42	1.24	-2.92	4.13	-2.89	6.80	-1.40	-0.66	-1.02
miR-99/100	2.29	1.64	2.44	-1.74	-1.25	0.30	-0.59	0.80	-2.53	1.10	-0.58	-0.05	-2.14

Supplemental Table 3:

miR-seed	Tumor others_T37	Tumor others_T4	Tumor others_T41	Tumor others_T44	Tumor others_T50	Tumor others_T73	Tumor others_T74	Tumor others_T81	Tumor others_T84	Tumor others_T92
Any-MiR	-1.19	-0.41	-25.26	3.58	-0.68	8.58	-6.04	-8.66	19.92	11.23
let-7/98	-0.27	-5.54	-8.38	0.15	-2.65	4.37	-2.85	-4.25	1.50	1.64
miR-1/206	-1.06	-4.18	-7.32	1.84	-3.33	2.59	-2.61	-3.19	6.63	3.70
miR-10	2.10	-0.51	-0.79	1.73	1.19	3.29	-0.41	-2.02	3.17	4.52
miR-101	-4.18	-5.03	-5.53	3.03	-3.01	1.58	-1.86	-4.61	6.28	5.96
miR-103/107	0.17	-1.42	-4.73	4.38	0.75	0.70	-1.39	-3.62	5.96	3.26
miR-122	2.26	-0.28	-1.78	-0.39	2.18	-0.88	1.38	-0.12	3.59	0.72
miR-124.1	-1.20	-0.77	-7.92	2.93	0.68	2.78	-1.37	-1.86	7.29	3.33
miR-124.2/506	-0.37	-0.88	-10.15	2.43	0.12	6.34	-1.55	-3.05	6.40	1.84
miR-125/351	1.59	-0.83	-7.54	-3.05	-1.62	0.70	-3.58	-4.88	0.35	-0.65
miR-126/126-3p	-1.14	-0.09	-0.74	0.15	1.98	1.12	-0.50	0.35	0.42	1.35
miR-128	-0.51	-0.97	-6.78	1.60	-2.54	3.39	-0.54	-3.93	4.80	3.03
miR-129-5p	0.36	-2.23	-0.80	1.14	0.24	1.77	0.37	-3.91	2.66	4.95
miR-130/301	-3.47	-4.40	-6.17	2.23	-1.07	2.41	-1.19	-3.59	5.45	3.71
miR-132/212	-2.19	-3.06	-2.72	1.33	-2.16	4.35	1.06	-1.10	4.33	2.99
miR-133	1.05	-0.10	-6.19	-0.74	-2.20	0.83	-1.15	-4.39	3.19	1.76
miR-134	-1.50	-2.00	-2.84	-0.58	-1.52	0.61	-2.01	-3.11	1.27	0.42
miR-135	-0.92	-3.83	-4.92	1.59	-1.19	3.71	1.27	-2.11	4.48	5.15
miR-136	-1.92	-0.92	-3.90	-1.83	-4.68	-0.87	-0.40	-5.04	3.78	2.38
miR-137	-1.60	-2.09	-4.14	1.01	-0.91	1.91	-0.31	-3.58	5.72	4.45
miR-138	2.11	-1.24	-3.72	-0.33	-0.72	1.73	-2.57	-6.00	1.97	1.91
miR-139	-1.59	-1.79	-3.60	3.55	-1.13	-0.29	-0.59	-2.52	6.94	5.23
miR-140	-1.68	-2.60	-3.48	1.10	0.17	2.54	-1.49	-1.11	5.12	2.56
miR-141/200a	-3.44	-4.88	-4.18	1.23	-1.62	0.08	-0.69	-3.06	6.41	3.34
miR-142-3p	-1.96	-2.96	-1.74	0.59	-2.35	4.18	1.31	0.50	4.67	4.50
miR-142-5p	-5.23	-5.84	-5.21	3.85	-2.33	2.63	-1.70	-1.96	8.26	2.77
miR-143	-1.88	-0.96	-5.44	1.79	-2.78	1.72	-2.10	-4.04	3.50	2.13
miR-144	-4.42	-4.23	-5.31	4.05	-2.37	3.51	-0.58	-3.03	7.54	6.08
miR-145	-2.60	-2.16	-3.41	2.65	-0.04	3.73	-1.09	0.29	6.29	6.04
miR-146	0.56	-1.39	-1.53	4.36	-0.15	-0.79	-0.70	-3.00	0.75	0.32
miR-148/152	-2.49	-2.20	-5.15	1.25	-0.68	4.35	0.17	-5.13	4.90	3.25
miR-149	2.80	1.80	-4.39	-0.48	-0.22	-1.69	-2.72	-4.09	0.28	2.09
miR-15/16/195/424/497	2.11	0.28	-6.77	1.93	1.75	0.53	1.05	-2.69	5.82	2.78
miR-150	1.90	0.54	-2.94	0.56	-1.41	0.56	-3.17	-1.74	2.39	1.00
miR-151	-2.20	-1.40	-3.02	5.38	-0.29	0.74	-0.19	-0.40	2.30	0.15
miR-153	-0.25	-2.38	-5.14	2.89	-1.43	3.17	-1.21	-3.65	6.05	2.51
miR-155	-0.75	-4.78	-3.34	1.00	-0.85	5.53	0.80	1.20	4.55	2.04
miR-17-5p/20/93.mr/106/519.	-1.08	-3.66	-7.47	1.30	0.78	4.62	-1.20	-2.95	6.11	3.89
miR-18	-1.38	-1.31	-2.78	0.72	-0.85	0.74	-3.72	-2.58	2.31	-0.47
miR-181	-3.60	-5.41	-5.66	3.86	-1.63	2.52	0.92	-4.14	8.93	5.25
miR-182	-2.41	-2.17	-6.82	3.96	0.80	3.98	0.74	-2.12	7.45	4.80
miR-183	-1.89	-4.46	-3.35	2.80	0.16	1.53	-0.10	0.34	7.02	3.09
miR-184	0.69	0.33	-1.89	0.50	2.17	-1.64	-1.10	-1.80	-0.09	1.29
miR-185	1.38	1.05	-2.79	-1.26	-0.57	-0.95	-2.68	-4.40	-0.45	0.09
miR-186	-2.45	-6.24	-6.53	5.65	-0.53	1.53	0.87	-1.78	8.27	5.20
miR-188	0.23	-4.62	-4.17	0.40	-1.17	1.85	1.10	0.07	2.12	0.70

miR-seed	Tumor others_T37	Tumor others_T4	Tumor others_T41	Tumor others_T44	Tumor others_T50	Tumor others_T73	Tumor others_T74	Tumor others_T81	Tumor others_T84	Tumor others_T92
miR-19	-4.19	-4.41	-7.31	2.44	-2.29	1.89	-2.95	-3.50	9.02	2.71
miR-190	-3.67	-2.87	-0.73	1.28	0.27	-0.64	1.91	-2.37	3.23	4.00
miR-191	2.02	0.63	-2.40	-0.11	-0.12	-0.13	-0.57	-1.48	-0.64	0.45
miR-192/215	-0.89	-2.35	-2.52	1.83	-1.17	-0.43	-0.88	-1.02	4.47	-0.27
miR-193	-0.93	-3.88	0.17	-2.05	-1.12	1.80	-0.16	-3.05	1.50	0.76
miR-194	-2.01	-3.84	-2.15	2.16	-0.79	3.37	2.18	-1.90	5.51	4.28
miR-196	-1.44	-2.95	-3.06	2.86	-0.81	3.52	1.38	-0.98	3.90	1.76
miR-199	-0.82	-1.98	-4.12	0.53	-1.71	1.77	-1.27	-4.02	2.61	3.01
miR-200b/429	-3.57	-7.30	-8.34	4.71	-2.41	5.96	-1.06	-3.30	8.78	5.81
miR-203.1	-2.67	-4.28	-4.28	3.85	-1.11	0.78	-0.72	-3.65	8.27	4.29
miR-204/211	0.50	-2.51	-1.88	1.48	0.25	0.16	-1.67	-1.89	2.56	4.11
miR-205	-2.83	-1.33	-6.02	-0.36	-2.28	1.87	-0.18	-1.70	2.10	3.53
miR-208	-0.59	-3.47	-3.41	4.53	-0.90	0.55	2.55	-0.99	2.86	2.89
miR-21	0.01	-4.32	0.04	2.28	-0.03	3.16	0.67	-1.17	5.98	3.29
miR-210	0.41	2.14	0.05	0.31	-1.27	0.43	-0.81	-1.73	1.66	-1.94
miR-214	0.78	0.37	-6.78	0.31	0.96	-0.09	-1.88	-4.94	1.83	0.08
miR-216	1.09	-2.65	-3.82	-1.69	2.04	-0.31	0.66	-1.97	0.84	1.20
miR-217	-2.85	-5.48	-3.77	1.71	-2.74	3.14	3.24	-1.13	4.35	3.62
miR-218	-0.79	-2.12	-5.16	2.60	-0.54	0.55	-2.84	-6.44	4.49	1.90
miR-219	-1.66	-2.88	-4.04	2.86	-0.84	2.05	-0.75	-2.65	4.07	1.21
miR-22	-0.41	-1.92	-5.13	-2.20	-0.41	3.77	0.77	-4.79	2.88	2.11
miR-221/222	-2.45	-4.05	-2.78	3.14	-3.21	-1.30	-1.84	-2.18	4.12	2.54
miR-223	-2.66	-5.03	-1.33	1.58	-2.22	5.18	-0.40	0.33	7.03	3.00
miR-224	-1.09	-4.21	-1.18	1.86	-1.19	2.64	2.48	0.81	5.44	2.35
miR-23	-2.38	-6.54	-4.75	3.34	-1.08	3.59	2.75	-1.85	7.28	4.38
miR-24	2.23	-1.27	-3.92	-3.38	-0.45	0.07	-2.40	-3.64	1.21	0.31
miR-24*	-2.30	-3.78	-0.44	3.53	-0.94	4.07	2.35	-0.06	3.42	2.72
miR-25/32/92/363/367	-1.27	-6.03	-6.53	2.43	-1.27	2.73	-1.65	-4.07	6.65	2.79
miR-26	-3.61	-6.59	-6.95	2.48	-1.90	5.62	-0.58	1.48	9.78	3.70
miR-27	-1.07	-3.26	-8.33	2.96	-2.11	3.53	-2.59	-3.08	4.08	2.96
miR-28	2.22	1.38	-1.28	-1.82	0.19	-0.51	-0.47	-4.06	-0.94	0.64
miR-29	0.03	0.25	-9.39	2.20	-1.32	5.35	-4.01	-6.96	-0.89	1.23
miR-299-3p	0.42	-0.73	-2.01	-0.80	-0.63	-0.24	0.35	-1.17	0.58	2.66
miR-299-5p	-2.50	-2.85	-4.26	2.72	-0.35	3.02	1.65	-2.22	3.72	3.41
miR-30-3p	-3.04	-4.79	-4.06	3.35	-2.13	1.39	1.26	-0.20	7.91	5.08
miR-30-5p	-5.22	-5.76	-7.49	3.15	-0.27	4.20	1.12	-4.27	6.01	2.44
miR-31	-2.53	-1.55	-4.35	0.51	-1.22	-0.08	-2.36	-1.65	2.22	0.87
miR-320	-1.71	-3.73	-5.58	4.22	-1.94	3.01	0.62	-0.73	7.33	4.95
miR-323	-3.19	-5.93	-4.03	2.92	-1.27	4.10	3.17	-1.24	7.24	5.65
miR-324-3p	0.06	-2.27	-2.30	0.02	0.66	-1.00	-0.64	-2.60	3.85	2.46
miR-324-5p	1.43	0.69	-0.94	0.11	1.89	1.25	1.50	-0.77	1.50	2.11
miR-325	-2.51	-1.93	-2.17	0.12	-0.87	2.13	0.33	-1.49	4.29	3.69
miR-326	2.09	1.97	-4.19	-0.88	-0.99	-2.44	-0.49	-6.38	0.28	0.49
miR-328	-0.05	-0.42	-2.23	-0.57	1.02	2.74	0.96	-4.15	1.06	1.19
miR-329	-0.41	-2.43	-4.69	0.73	-2.24	2.10	2.23	-3.27	1.49	3.22
miR-33	-1.16	-4.17	-1.53	4.95	-0.10	0.96	1.94	-1.03	3.73	1.01
miR-330	-3.93	-5.26	-4.87	1.07	-4.39	1.13	-0.28	-3.01	8.22	3.58
miR-331	3.09	3.86	-3.53	-0.67	1.52	-1.61	-0.98	-5.85	-2.36	0.80

miR-seed	Tumor others_T37	Tumor others_T4	Tumor others_T41	Tumor others_T44	Tumor others_T50	Tumor others_T73	Tumor others_T74	Tumor others_T81	Tumor others_T84	Tumor others_T92
miR-335	1.75	-4.13	-2.98	1.47	-1.06	1.44	2.14	-1.98	3.78	-0.32
miR-338	-0.06	-2.64	-3.89	3.13	-1.17	0.59	0.58	-3.06	3.21	3.46
miR-339	5.11	-0.62	-3.01	2.70	1.29	-0.57	-2.56	-2.20	0.96	0.08
miR-34/449	1.97	0.50	-6.27	0.09	0.36	0.27	-1.15	-5.32	1.15	1.06
miR-342	-2.02	-1.84	-1.67	1.30	-1.04	1.94	2.12	-3.09	1.39	1.31
miR-346	-0.63	-1.58	-1.27	0.79	-1.97	0.92	-0.08	-1.62	1.66	0.73
miR-34b	-1.26	-0.47	-4.65	1.26	-0.31	0.58	-2.56	-4.10	2.87	0.69
miR-361	0.17	-1.90	-2.13	-0.04	-2.25	1.14	-1.56	-0.70	5.84	2.54
miR-362	-1.26	-1.93	-3.08	0.31	-0.63	-0.77	-0.22	-2.20	1.61	2.22
miR-363	-1.39	-4.55	-1.64	1.10	-2.01	-0.56	-1.36	-2.90	4.07	1.21
miR-365	-1.50	-4.15	-1.73	1.09	-2.19	1.77	2.93	-1.26	3.60	2.85
miR-369-3p	-1.82	-3.13	-4.75	4.50	-1.00	1.99	1.27	-3.48	6.12	1.92
miR-370	1.31	-0.50	-1.26	-1.65	0.58	0.22	0.80	-2.47	-0.71	2.75
miR-374	-1.45	-2.24	-2.58	4.33	-0.51	1.91	1.33	-2.95	6.39	1.92
miR-375	-0.99	0.51	-2.91	2.23	-0.04	2.07	-0.61	1.31	3.39	1.70
miR-376	-0.83	-2.64	-2.31	2.73	0.24	2.56	1.88	-0.14	3.99	3.61
miR-376c	-1.40	-2.16	-3.52	5.70	1.00	-0.50	1.21	-0.94	4.38	3.09
miR-377	-1.16	-3.04	-3.34	1.60	-1.68	0.40	-0.29	-4.12	3.23	3.44
miR-378	2.90	-2.76	-5.46	1.82	-1.95	4.34	0.80	-3.09	3.73	2.28
miR-378*	-0.60	-2.86	-1.13	0.83	-1.63	-1.11	-0.86	-1.76	0.50	-1.04
miR-379	-0.36	0.06	-1.48	1.20	-1.54	3.25	1.29	2.08	2.25	2.56
miR-380-5p	-0.79	-0.21	-0.71	2.50	1.61	-0.28	-0.02	-1.70	3.12	0.97
miR-381	-3.12	-8.81	-5.49	3.77	-1.73	4.42	0.48	-2.18	9.21	7.11
miR-382	-2.87	-3.74	-1.78	1.80	-2.35	0.59	1.20	0.27	4.24	6.38
miR-383	-2.83	-1.89	-2.08	1.10	-0.89	3.32	-1.83	-2.27	1.57	1.86
miR-384	-1.96	-5.40	-3.57	3.97	-1.06	2.35	0.23	-1.60	7.65	5.23
miR-409-3p	-1.16	-5.16	-1.97	1.76	-2.73	2.49	-1.22	-0.04	6.98	3.52
miR-409-5p	1.55	-0.55	-0.29	-1.11	0.41	-0.91	0.51	-2.45	2.08	1.81
miR-410	-1.96	-5.28	-4.44	2.71	-1.25	0.82	1.07	-3.07	8.43	3.67
miR-421	-1.29	-0.25	-3.28	4.30	0.42	1.71	1.05	-0.73	3.89	2.73
miR-431	0.81	0.39	-0.81	-0.09	0.44	-1.35	0.75	-0.74	2.39	2.46
miR-433-3p	-3.01	-3.65	-2.80	1.67	-2.08	4.05	1.06	0.04	5.80	4.08
miR-448	-0.14	-3.44	-4.94	4.36	-0.96	2.20	0.62	-2.46	5.76	3.83
miR-450	0.26	-2.36	-1.17	0.73	-1.59	1.12	-0.29	-1.19	2.47	3.51
miR-451	-0.54	-2.11	-1.71	2.05	1.85	5.15	-0.09	0.74	3.63	2.50
miR-452	-1.06	-2.57	0.49	3.68	0.35	0.88	0.12	0.28	5.13	0.45
miR-455	-1.68	-2.93	-2.84	-0.62	-0.76	0.67	0.84	-1.33	1.73	3.15
miR-485-3p	-2.36	-2.08	-4.87	1.75	-1.66	2.50	0.65	-3.63	1.55	3.67
miR-485-5p	2.89	0.11	-4.97	-1.79	1.57	-0.32	-3.59	-3.65	0.20	-0.85
miR-486	-0.94	-1.89	-2.67	0.17	-1.66	2.18	1.03	-1.39	2.47	1.22
miR-488	-1.38	-1.57	-2.68	1.70	-1.08	2.23	2.58	-0.57	5.35	2.08
miR-490	1.58	-0.43	-1.58	0.49	-0.85	1.35	-1.06	-0.78	3.51	1.28
miR-491	4.14	5.44	-1.17	-1.84	1.57	0.96	-2.42	-4.26	-2.36	0.52
miR-493-5p	-0.87	-3.17	-4.67	2.85	-1.58	2.55	1.43	-4.69	5.67	5.60
miR-494	-4.60	-5.63	-4.09	2.74	-2.49	1.51	2.04	-1.34	5.26	2.81
miR-495	-4.52	-5.90	-4.60	3.99	-0.73	3.62	0.80	-2.01	6.68	7.50
miR-496	-5.39	-8.27	-8.13	5.56	-2.44	2.61	1.42	-2.53	10.73	7.55
miR-499	-0.78	-2.74	-3.73	4.49	-0.57	1.70	2.47	-1.73	4.16	2.82

miR-seed	Tumor others_T37	Tumor others_T4	Tumor others_T41	Tumor others_T44	Tumor others_T50	Tumor others_T73	Tumor others_T74	Tumor others_T81	Tumor others_T84	Tumor others_T92
miR-500	-1.19	-2.36	-2.50	1.07	-0.69	0.95	0.94	-1.25	2.53	1.00
miR-503	0.67	-1.70	-4.81	-0.11	0.80	0.71	2.06	-2.72	3.26	2.27
miR-504	3.15	0.89	-2.34	0.01	0.37	-0.07	-0.63	-2.22	-1.17	0.09
miR-505	-2.78	-5.93	-5.55	0.82	-3.40	3.38	-1.53	-2.60	7.15	3.27
miR-539	-0.53	-2.38	-3.20	4.49	-0.74	2.34	-1.80	-3.45	4.61	4.27
miR-542-3p	-0.56	-3.68	-4.45	3.33	-0.60	0.47	-0.29	-1.32	4.77	2.83
miR-543	-3.48	-6.06	-5.18	5.45	-1.33	4.07	2.66	-2.91	5.47	4.92
miR-544	-2.69	-3.02	-4.51	2.63	-0.84	0.84	-2.77	-3.70	4.18	4.09
miR-7	0.59	-2.07	-5.84	0.14	-4.49	1.12	-2.99	-3.65	3.52	0.64
miR-9	-0.31	-2.08	-9.06	0.04	0.12	3.46	-1.38	-3.56	4.11	1.19
miR-93.hd/291-3p/294/295/30	-0.77	-5.80	-4.34	0.18	-0.63	4.80	2.48	-3.04	5.45	1.76
miR-96	-0.48	-2.95	-5.91	3.70	1.80	2.28	0.13	-3.28	6.94	3.10
miR-99/100	1.65	-1.00	-1.49	-1.99	0.99	-0.11	0.02	-1.42	0.55	-1.32
