# Supplementary material for: Increased MicroRNA Activity in Human Cancers
Source: PLoS One. 2009 Jun 25;4(6):e6045. doi: 10.1371/journal.pone.0006045 (PMC2698213; doi:10.1371/journal.pone.0006045)
Supplement: Table S4 — MiR-seeds found to be the most significantly upregulated in breast cancer (0.04 MB PDF) [file pone.0006045.s005.pdf]

Supplemental Table 4: MiR-seeds found to be the most significantly upregulated in breast cancer
using two-sample t-test p-values*
We find 77 MiRs with P < 3E-04 (Bonferroni FWER < 0.05)

rank	miR-seed	p-value	Bonferroni
1	miR-504	8.60E-09	1.29E-06
2	miR-431	8.57E-08	1.28E-05
3	miR-28	5.72E-07	8.58E-05
4	miR-15/16/195/424/497	6.36E-07	9.54E-05
5	miR-31	9.19E-07	1.38E-04
6	miR-219	1.11E-06	1.66E-04
7	miR-17-5p/20/93.mr/106/	1.13E-06	1.70E-04
8	miR-450	1.52E-06	2.28E-04
9	miR-129-5p	1.56E-06	2.34E-04
10	miR-9	1.61E-06	2.41E-04
11	miR-96	1.97E-06	2.96E-04
12	miR-191	2.88E-06	4.32E-04
13	miR-34/449	3.62E-06	5.43E-04
14	miR-339	4.34E-06	6.51E-04
15	miR-124.2/506	4.67E-06	7.00E-04
16	miR-455	5.01E-06	7.51E-04
17	miR-204/211	5.23E-06	7.84E-04
18	miR-138	5.74E-06	8.62E-04
19	miR-124.1	5.77E-06	8.65E-04
20	miR-19	6.18E-06	9.26E-04
21	miR-24	6.97E-06	1.05E-03
22	miR-542-3p	7.19E-06	1.08E-03
23	miR-485-5p	7.62E-06	1.14E-03
24	miR-140	8.16E-06	1.22E-03
25	miR-130/301	8.79E-06	1.32E-03
26	miR-365	1.18E-05	1.77E-03
27	miR-363	1.25E-05	1.87E-03
28	miR-22	1.31E-05	1.96E-03
29	miR-146	1.79E-05	2.69E-03
30	miR-539	2.24E-05	3.36E-03
31	miR-18	2.28E-05	3.41E-03
32	miR-183	2.37E-05	3.56E-03
33	miR-137	2.39E-05	3.59E-03
34	miR-128	2.41E-05	3.61E-03
35	miR-410	2.50E-05	3.75E-03
36	miR-182	2.54E-05	3.81E-03
37	miR-503	2.58E-05	3.87E-03
38	miR-185	2.71E-05	4.06E-03
39	miR-23	2.75E-05	4.12E-03
40	miR-193	2.92E-05	4.38E-03
41	miR-10	3.04E-05	4.56E-03
42	miR-150	3.32E-05	4.98E-03
43	miR-335	3.40E-05	5.11E-03
44	miR-409-3p	3.55E-05	5.33E-03
45	miR-184	3.63E-05	5.45E-03
46	miR-376c	3.99E-05	5.99E-03
47	miR-192/215	4.05E-05	6.07E-03
48	miR-326	4.13E-05	6.20E-03
49	miR-93.hd/291-3p/294/29	4.30E-05	6.45E-03
50	miR-134	4.43E-05	6.65E-03
51	miR-25/32/92/363/367	5.10E-05	7.65E-03
52	miR-374	5.24E-05	7.86E-03
53	miR-141/200a	5.26E-05	7.89E-03
54	miR-421	6.95E-05	1.04E-02
55	miR-380-5p	6.97E-05	1.04E-02
56	miR-299-5p	7.09E-05	1.06E-02
57	miR-224	7.61E-05	1.14E-02
58	miR-331	7.91E-05	1.19E-02
59	miR-369-3p	8.40E-05	1.26E-02
60	miR-27	1.04E-04	1.56E-02
61	miR-133	1.20E-04	1.81E-02
62	miR-346	1.23E-04	1.85E-02
63	miR-153	1.25E-04	1.87E-02
64	miR-500	1.33E-04	2.00E-02
65	miR-377	1.43E-04	2.14E-02
66	miR-381	1.43E-04	2.15E-02
67	miR-21	1.59E-04	2.39E-02
68	miR-181	1.69E-04	2.53E-02
69	miR-409-5p	1.85E-04	2.78E-02
70	miR-135	1.98E-04	2.96E-02
71	miR-29	2.14E-04	3.21E-02
72	miR-330	2.17E-04	3.25E-02
73	miR-221/222	2.41E-04	3.62E-02
74	miR-448	2.50E-04	3.75E-02
75	miR-103/107	2.58E-04	3.86E-02
76	miR-149	2.64E-04	3.97E-02
77	miR-142-5p	2.70E-04	4.05E-02

rank	miR-seed	p-value	Bonferroni
78	miR-145	0.00028	0.041699
79	miR-30-3p	0.00034	0.050946
80	miR-338	0.00037	0.055718
81	miR-223	0.00038	0.056899
82	miR-379	0.00044	0.065511
83	miR-320	0.00067	0.100309
84	miR-1/206	0.00078	0.116811
85	miR-299-3p	0.00080	0.120671
86	miR-384	0.00082	0.123366
87	miR-142-3p	0.00086	0.129479
88	miR-323	0.00091	0.135925
89	miR-148/152	0.00101	0.151849
90	miR-216	0.00104	0.156338
91	miR-362	0.00110	0.164385
92	miR-544	0.00117	0.17479
93	miR-132/212	0.00122	0.182873
94	miR-125/351	0.00124	0.186202
95	miR-218	0.00151	0.226205
96	miR-329	0.00158	0.237637
97	miR-143	0.00168	0.252723
98	miR-203.1	0.00177	0.264907
99	miR-493-5p	0.00194	0.290574
100	let-7/98	0.00194	0.291542
101	miR-200b/429	0.00220	0.329485
102	miR-199	0.00230	0.345711
103	miR-144	0.00243	0.363834
104	miR-7	0.00258	0.386297
105	miR-495	0.00262	0.392676
106	miR-101	0.00281	0.421875
107	miR-205	0.00288	0.4316
108	miR-188	0.00317	0.476078
109	miR-30-5p	0.00330	0.49575
110	miR-375	0.00334	0.500453
111	miR-328	0.00405	0.606965
112	miR-378*	0.00414	0.621654
113	miR-122	0.00419	0.629056
114	miR-342	0.00475	0.712557
115	miR-505	0.00501	0.751174
116	miR-214	0.00507	0.760314
117	miR-324-3p	0.00602	0.903117
118	miR-496	0.00615	0.923188
119	miR-34b	0.00618	0.926631
120	miR-376	0.00648	0.971984
121	miR-33	0.00686	1.028291
122	miR-491	0.00718	1.076628
123	miR-210	0.00769	1.154167
124	miR-194	0.00822	1.232256
125	miR-196	0.00887	1.330009
126	miR-155	0.01008	1.512346
127	miR-485-3p	0.01060	1.589516
128	miR-126/126-3p	0.01210	1.815264
129	miR-499	0.01243	1.863857
130	miR-99/100	0.01326	1.988634
131	miR-208	0.02319	3.479152
132	miR-370	0.02930	4.394275
133	miR-543	0.03384	5.075361
134	miR-451	0.04349	6.523888
135	miR-186	0.04468	6.702188
136	miR-361	0.04740	7.110201
137	miR-382	0.04797	7.196214
138	miR-325	0.05878	8.816981
139	miR-26	0.06705	10.05717
140	miR-494	0.06768	10.15218
141	miR-486	0.09159	13.73793
142	miR-151	0.10164	15.24532
143	miR-136	0.16749	25.12317
144	miR-324-5p	0.21946	32.91935
145	miR-139	0.25186	37.77969
146	miR-378	0.25988	38.98153
147	miR-217	0.40571	60.857
148	miR-383	0.53905	80.85796
149	miR-452	0.70222	105.3326
150	miR-190	0.96814	145.2209

* The t-test p-values have been used to rank the seeds displaying the most significant deviation of activity in tumors, and are provided here for information.
