# Supplementary material for: Increased MicroRNA Activity in Human Cancers
Source: PLoS One. 2009 Jun 25;4(6):e6045. doi: 10.1371/journal.pone.0006045 (PMC2698213; doi:10.1371/journal.pone.0006045)
Supplement: Table S5 — Expression values for genes targeted by the 77 miR-seeds upregulated in breast cancer (1.13 MB PDF) [file pone.0006045.s006.pdf]

Supplemental Table 5. Expression values for genes targeted by the 77 miR-seeds upregulated in breast cancer

miR-504 miR-431 miR-28 miR-15/16/195/424/497 miR-31 miR-219 miR-17-5p/20/93.mr/106/519.d miR-450 miR-129-5p miR-9 miR-96 miR-191 miR-34/449 miR-339 miR-124.2/506 miR-455 miR-204/211 miR-138 miR-124.1 miR-19 miR-24 miR-542-3p miR-485-5p miR-140 miR-130/301 miR-365 miR-363 miR-22 miR-146 miR-539 miR-18 miR-183 miR-137 miR-128 miR-410 miR-182 miR-503 miR-185 miR-23 miR-193 miR-10 miR-150 miR-335 miR-409-3p miR-184 miR-376c miR-192/215 miR-326 miR-93.hd/291-3p/294/295/302/372/373/520 miR-134 miR-25/32/92/363/367 miR-374 miR-141/200a miR-421 miR-380-5p miR-299-5p miR-224 miR-331 miR-369-3p miR-27 miR-133 miR-346 miR-153 miR-500 miR-377 miR-381 miR-21 miR-181 miR-409-5p miR-135 miR-29 miR-330 miR-221/222 miR-448 miR-103/107 miR-149 miR-142-5p

GENE			probe-set		normal			tumor				
name	nb targets	title	id	nb targets	avg	s.d.	n	avg	s.d.	n	u/d	t-test
CPEB4	38	cytoplasmic polyadenylation element binding protein 4	224829_at	38	7.373	0.58	7	6.596	1.56	40	d	0.20741
			224828_at	38	6.06	0.95	7	6.052	1.47	40	d	0.989124
			224831_at	38	8.04	0.4	7	7.641	1.18	40	d	0.121494
			242280_x_at	0	4.411	0.28	7	4.286	0.83	40	d	0.484302
RSBN1	36	round spermatid basic protein 1	213694_at	36	7.724	0.15	7	7.125	0.65	40	d	0.000016
			222789_at	15	7.714	0.43	7	7.799	0.67	40	u	0.749036
			222788_s_at	15	7.629	0.38	7	7.071	0.47	40	d	0.005663
			222790_s_at	15	8.342	0.32	7	7.811	0.64	40	d	0.040362
			222791_at	15	8.205	0.42	7	7.677	0.7	40	d	0.066144
DDX3X	33	DEAD (Asp-Glu-Ala-Asp) box polypeptide 3, X-linked	201210_at	33	12.65	0.16	7	12.11	0.42	40	d	0.002164
			212514_x_at	29	9.103	0.71	7	8.939	1.11	40	d	0.713125
			212515_s_at	29	10.56	0.21	7	9.663	0.77	40	d	0.000001
			207617_at	6	2.544	0.09	7	2.608	0.11	40	u	0.17036
			201211_s_at	5	8.172	0.75	7	7.835	1.36	40	d	0.535853
			1558120_at	0	3.542	0.72	7	3.821	0.83	40	u	0.416563
OTUD4	33	OTU domain containing 4	203479_s_at	33	4.587	0.38	7	5.84	0.69	40	u	0.000037
			203480_s_at	33	9.475	0.13	7	9.472	0.43	40	d	0.966843
			220669_at	0	2.631	0.11	7	3.298	0.78	40	u	0.000009
			238848_at	0	3.646	0.25	7	4.679	1.11	40	u	0.000011
PLAG1	32	pleiomorphic adenoma gene 1	205372_at	32	3.579	0.27	7	4.178	1.18	40	u	0.008872
RPS6KB1	31	ribosomal protein S6 kinase, 70kDa, polypeptide 1	226660_at	31	8.719	0.22	7	8.583	0.88	40	d	0.419942
			204171_at	10	6.744	0.28	7	7.457	0.73	40	u	0.016238
			211578_s_at	5	6.058	0.43	7	6.684	0.99	40	u	0.115956
PURB	30	purine-rich element binding protein B	225120_at	30	9.378	0.16	7	9.187	0.64	40	d	0.123387
			226762_at	26	8.432	0.47	7	8.661	0.76	40	u	0.455816
			235711_at	16	4.628	1	7	5.213	1.38	40	u	0.297991
			227718_at	1	4.928	0.31	7	5.648	0.84	40	u	0.03382
			228467_at	0	2.221	0.07	7	2.266	0.18	40	u	0.528614
ATXN1	30	ataxin 1	203231_s_at	30	7.483	0.8	7	6.785	1.09	40	d	0.121376
			203232_s_at	30	8.232	0.34	7	7.387	1.1	40	d	0.000767
			1559249_at	0	4.674	0.69	7	4.999	0.75	40	u	0.300648
			242230_at	0	4.662	0.49	7	4.724	1.09	40	u	0.885178
			230507_at	0	3.982	0.23	7	4.021	0.39	40	u	0.801144
BACH2	30	BTB and CNC homology 1, basic leucine zipper transcription factor 2	227173_s_at	30	6.025	0.47	7	5.403	0.76	40	d	0.045343
			221234_s_at	30	4.655	1.04	7	3.116	1.19	40	d	0.002848
			236796_at	0	5.841	0.89	7	3.92	1.14	40	d	0.000147
ELAVL2	29	ELAV (embryonic lethal, abnormal vision, Drosophila)-like 2 (Hu antigen B)	228260_at	29	2.922	0.47	7	4.564	2.14	40	u	0.000152
			208427_s_at	7	2.904	0.2	7	3.947	1.24	40	u	0.000015
MECP2	29	methyl CpG binding protein 2 (Rett syndrome)	202616_s_at	29	7.43	0.43	7	6.065	1.07	40	d	0.002148
			202617_s_at	29	7.994	0.39	7	7.001	0.76	40	d	0.001794
			202618_s_at	1	5.892	0.22	7	5.899	0.59	40	u	0.976508
CUGBP2	29	CUG triplet repeat, RNA binding protein 2	202156_s_at	36	6.949	0.43	7	6.123	1.37	40	d	0.006651
			202158_s_at	36	7.111	0.49	7	6.603	1.38	40	d	0.101355
			202157_s_at	11	9.197	0.5	7	8.161	1.47	40	d	0.002728
			1554569_a_at	0	4.36	0.36	7	4.485	0.59	40	u	0.598078
			1556323_at	0	3.429	0.33	7	3.764	0.57	40	u	0.145088
			242268_at	0	4.058	1.03	7	3.602	1.06	40	d	0.306678
			227178_at	0	6.561	0.6	7	5.401	1.19	40	d	0.01749
		fragile X mental retardation, autosomal	201636_at	5	8.687	0.14	7	8.847	0.59	40	u	0.161763
			201635_s_at	5	8.517	0.42	7	9.381	0.68	40	u	0.002752

FXR1	27	fragile X mental retardation, autosomal homolog 1	201637_s_at	4	9.894	0.14	7	9.911	0.65	40	u	0.881075
			1569171_a_at	0	1.857	0.09	7	1.928	0.1	40	u	0.083432
			229519_at	0	7.489	0.22	7	8.217	0.88	40	u	0.000106
KPNA3	27	karyopherin alpha 3 (importin alpha 4)	221502_at	27	8.685	0.21	7	9.202	0.62	40	u	0.000552
			221503_s_at	8	7.077	0.58	7	7.839	0.69	40	u	0.009482
BCL11A	27	B-cell CLL/lymphoma 11A (zinc finger protein)	219497_s_at	27	5.36	0.46	7	5.93	2.07	40	u	0.142391
			222891_s_at	27	5.944	0.77	7	6.151	2.37	40	u	0.677078
			219498_s_at	13	4.082	0.8	7	5.062	1.72	40	u	0.15508
			1559078_at	0	4.984	0.86	7	4.997	1.65	40	u	0.985172
			210347_s_at	0	4.768	0.49	7	4.972	1.65	40	u	0.542219
CAMTA1	26	calmodulin binding transcription activator 1	213268_at	26	5.113	0.87	7	3.827	1.09	40	d	0.0059
			1555370_a_at	17	6.513	0.6	7	5.954	1.03	40	d	0.1782
			227328_at	0	6.358	0.4	7	6.206	0.6	40	d	0.52676
			225692_at	0	9.364	0.25	7	9.622	0.78	40	u	0.122701
			225693_s_at	0	9.855	0.23	7	9.722	0.73	40	d	0.385702
			241882_at	0	3.416	0.5	7	3.634	0.54	40	u	0.335408
EPHA4	26	EPH receptor A4	227449_at	6	4.774	0.54	7	3.819	1.21	40	d	0.050703
			228948_at	6	3.286	0.59	7	3.504	0.96	40	u	0.571569
			229374_at	6	2.37	0.36	7	2.486	0.51	40	u	0.573195
			206114_at	3	4.924	0.73	7	4.56	1.22	40	d	0.456434
KLF12	26	Kruppel-like factor 12	227261_at	26	7.649	0.44	7	7.092	0.95	40	d	0.141798
			229881_at	18	4.901	0.32	7	5.081	0.82	40	u	0.57523
			238940_at	16	3.523	0.16	7	3.905	0.43	40	u	0.028224
			239019_at	12	2.162	0.04	7	2.338	0.45	40	u	0.022495
			214276_at	5	4.245	0.32	7	4.576	0.71	40	u	0.245069
			208467_at	0	2.294	0.21	7	2.472	0.57	40	u	0.169341
			206966_s_at	0	5.33	0.54	7	5.164	0.62	40	d	0.520347
			206965_at	0	3.691	0.24	7	3.986	0.75	40	u	0.069698
SRC	26	v-src sarcoma (Schmidt-Ruppin A-2) viral oncogene homolog (avian)	213324_at	5	7.112	0.27	7	6.93	0.99	40	d	0.350625
			221284_s_at	0	3.244	0.43	7	3.083	0.67	40	d	0.548876
			1565082_x_at	0	2.07	0.04	7	2.147	0.17	40	u	0.017931
			221281_at	0	3.203	0.28	7	3.224	0.45	40	u	0.909969
			1565080_at	0	2.099	0.03	7	2.166	0.07	40	u	0.018814
			1558211_s_at	0	3.297	0.15	7	3.37	0.29	40	u	0.53616
			1558210_at	0	2.149	0.02	7	2.204	0.05	40	u	0.006751
EIF2C1	26	eukaryotic translation initiation factor 2C, 1	218287_s_at	26	7.344	0.3	7	7.155	0.57	40	d	0.404511
			222576_s_at	26	4.432	0.48	7	4.912	0.74	40	u	0.111901
CPEB2	26	cytoplasmic polyadenylation element binding protein 2	226939_at	26	8.773	0.46	7	7.794	1.26	40	d	0.001655
			235462_at	0	2.661	0.12	7	2.817	0.29	40	u	0.176943
			235479_at	0	4.459	0.87	7	4.902	0.77	40	u	0.187834
CPEB3	25	cytoplasmic polyadenylation element binding protein 3	205773_at	25	3.858	0.67	7	3.484	0.48	40	d	0.088366
			1555250_a_at	18	4.426	0.34	7	4.044	0.53	40	d	0.077699
			243651_at	0	6.081	0.15	7	6.037	0.32	40	d	0.72699
FMR1	25	fragile X mental retardation 1	215245_x_at	25	8.542	0.31	7	8.904	0.72	40	u	0.205256
			203689_s_at	25	8.342	0.27	7	8.499	0.88	40	u	0.38859
NOVA1	25	neuro-oncological ventral antigen 1	205794_s_at	25	7.634	0.69	7	3.899	2.29	40	d	0
			207437_at	0	3.288	0.33	7	3.734	1.03	40	u	0.045319
MIER3	25	mesoderm induction early response 1, family member 3	228961_at	25	7.026	0.27	7	6.933	0.68	40	d	0.726802
			231975_s_at	23	6.119	0.42	7	5.961	0.87	40	d	0.6475
			1554449_at	18	3.301	0.22	7	3.721	0.65	40	u	0.005563
			1554450_s_at	18	5.997	0.42	7	5.964	0.9	40	d	0.926833
			1553336_a_at	3	2.582	0.33	7	2.692	0.51	40	u	0.591252
STC1	25	stanniocalcin 1	204595_s_at	25	7.292	2.08	7	6.433	2.15	40	d	0.34281
			204597_x_at	25	7.069	2.16	7	6.088	2.04	40	d	0.260727
			230746_s_at	24	7.319	1.93	7	6.789	2.1	40	d	0.545468
			204596_s_at	0	5.018	1.85	7	4.635	1.88	40	d	0.628995
RNF165	25	ring finger protein 165	1569080_at	25	2.404	0.22	7	2.339	0.16	40	d	0.360249
			1557545_s_at	23	3.589	0.78	7	3.29	0.55	40	d	0.232049
			230143_at	0	3.098	0.27	7	3.672	1.31	40	u	0.020073
RNF38	25	ring finger protein 38	218528_s_at	25	8.937	0.22	7	7.939	0.62	40	d	0
			222660_s_at	6	4.028	0.58	7	4.352	0.92	40	u	0.381474
SERTAD2	25	SERTA domain containing 2	202657_s_at	25	8.674	0.24	7	8.854	0.81	40	u	0.283274
			202656_s_at	25	8.044	0.33	7	8.164	0.84	40	u	0.71787
			218669_at	0	7.907	0.36	7	8.505	1.28	40	u	0.022994

RAP2C	25	RAP2C, member of RAS oncogene family	218668_s_at	0	6.182	0.17	7	7.253	1.49	40	u	0.00009
			234344_at	0	3.198	0.28	7	4.198	1.06	40	u	0.000021
HIC2	24	hypermethylated in cancer 2	212965_at	24	5.674	0.28	7	5.645	0.36	40	d	0.845986
			212966_at	24	5.487	0.66	7	4.866	0.73	40	d	0.044827
			212964_at	24	6.462	0.22	7	5.688	0.93	40	d	0.000068
			216911_s_at	1	2.474	0.14	7	2.479	0.15	40	u	0.937295
QKI	24	quaking homolog, KH domain RNA binding (mouse)	212636_at	24	8.481	0.45	7	7.881	0.9	40	d	0.098763
			212262_at	10	7.4	0.25	7	7.626	1.14	40	u	0.28572
			214543_x_at	10	7.335	0.39	7	6.926	0.9	40	d	0.252501
			1555154_a_at	10	6.945	0.33	7	6.56	1.2	40	d	0.111849
			212263_at	10	9.778	0.18	7	9.226	0.87	40	d	0.001086
			212265_at	10	10.21	0.21	7	9.934	0.83	40	d	0.089523
			214541_s_at	0	2.233	0.03	7	2.418	0.43	40	u	0.01081
			241938_at	0	6.404	0.71	7	6.181	0.8	40	d	0.501586
NR4A3	24	nuclear receptor subfamily 4, group A, member 3	209959_at	24	6.267	1.2	7	5.382	1.1	40	d	0.065335
			207978_s_at	9	3.691	1.27	7	3.41	1.05	40	d	0.539162
			216979_at	0	2.734	0.3	7	2.753	0.34	40	u	0.895736
NBEA	24	neurobeachin	226439_s_at	24	5.888	0.34	7	4.583	1.39	40	d	0.000014
			221207_s_at	24	6.781	0.47	7	4.828	1.47	40	d	0.000001
PURA	24	purine-rich element binding protein A	204020_at	24	9.993	0.25	7	8.876	0.69	40	d	0
			204021_s_at	0	7.937	0.26	7	7.621	0.79	40	d	0.065517
			213806_at	0	2.192	0.29	7	2.185	0.22	40	d	0.93906
SOX4	24	SRY (sex determining region Y)-box 4	201417_at	24	10.96	0.47	7	11.22	0.71	40	u	0.365976
			201416_at	24	10.27	0.55	7	10.52	0.86	40	u	0.466365
			1567906_at	0	2.08	0.42	7	2.934	1.31	40	u	0.004046
			213665_at	0	3.516	0.4	7	4.524	1.28	40	u	0.000648
			201418_s_at	0	7.84	0.54	7	8.117	0.91	40	u	0.449671
			213668_s_at	0	7.871	0.61	7	8.509	1.24	40	u	0.199425
KIAA2022	24	KIAA2022	244370_at	24	3.495	0.68	7	3.74	1.19	40	u	0.607782
			244076_at	20	3.601	0.44	7	3.711	0.59	40	u	0.646729
			234333_at	10	2.326	0.24	7	2.432	0.3	40	u	0.389331
ESRRG	24	estrogen-related receptor gamma	207981_s_at	24	5.111	0.7	7	4.85	1.33	40	d	0.623071
			209966_x_at	11	4.542	1.07	7	4.7	1.37	40	u	0.776774
RIT1	24	Ras-like without CAAX 1	236224_at	0	7.162	0.46	7	7.053	0.97	40	d	0.776785
			209882_at	0	6.087	0.53	7	7.343	1.12	40	u	0.00657
			243463_s_at	0	6.287	0.27	7	6.492	0.64	40	u	0.416265
			236223_s_at	0	5.803	0.5	7	5.977	0.96	40	u	0.647626
			239843_at	0	4.956	0.28	7	5.96	0.8	40	u	0.000005
ZFPM2	24	zinc finger protein, multitype 2	219778_at	24	4.938	0.48	7	3.835	1.24	40	d	0.028034
SOX11	24	SRY (sex determining region Y)-box 11	204913_s_at	24	2.794	0.61	7	5.587	2.71	40	u	0.000002
			204914_s_at	24	2.943	0.6	7	6.106	2.6	40	u	0
			204915_s_at	24	3.022	0.63	7	5.164	2.17	40	u	0.000025
NARG1	23	NMDA receptor regulated 1	226998_at	23	5.338	0.52	7	6.308	0.66	40	u	0.000799
			222836_at	12	2.288	0.05	7	2.368	0.17	40	u	0.024856
			222837_s_at	12	5.374	0.54	7	5.864	1.04	40	u	0.24027
			1556382_a_at	0	2.593	0.17	7	2.676	0.19	40	u	0.290814
			219158_s_at	0	7.554	0.33	7	8.807	0.62	40	u	0.000006
			1556381_at	0	2.969	0.45	7	2.638	0.36	40	d	0.041483
MBNL2	23	muscleblind-like 2 (Drosophila)	203640_at	23	9.836	0.33	7	9.084	0.89	40	d	0.035869
			205017_s_at	8	5.737	0.74	7	6.022	1.12	40	u	0.527831
			205018_s_at	1	7.012	0.87	7	6.984	1.58	40	d	0.964357
			232138_at	0	8.329	0.52	7	7.216	1.3	40	d	0.033658
PHF15	23	PHD finger protein 15	212660_at	23	8.188	0.44	7	6.178	1.33	40	d	0
			208202_s_at	1	4.409	0.36	7	4.066	0.43	40	d	0.058311
			232279_at	0	5.316	0.64	7	4.819	0.69	40	d	0.090304
CREB5	23	cAMP responsive element binding protein 5	229228_at	23	5.443	1.2	7	4.566	0.75	40	d	0.015578
			205931_s_at	6	5.192	0.98	7	4.337	0.79	40	d	0.016324
SYT1	23	synaptotagmin I	203999_at	19	3.12	0.52	7	4.068	2.32	40	u	0.032437
			203998_s_at	19	2.866	0.14	7	4.107	2.09	40	u	0.000728
ZFP36L1	22	zinc finger protein 36, C3H type-like 1	211962_s_at	22	11.45	0.42	7	10.39	0.7	40	d	0.000433
			211965_at	22	8.531	1.04	7	8.454	1.09	40	d	0.864734
			213284_at	19	2.432	0.29	7	2.363	0.16	40	d	0.595069
			213277_at	19	3.057	0.09	7	3.02	0.39	40	d	0.621238
			227084_at	22	6.799	0.47	7	6.198	1.57	40	d	0.06635

DTNA	22	dystrobrevin, alpha	210736_x_at	1	4.363	0.46	7	4.649	0.63	40	u	0.263146
			211493_x_at	1	2.487	0.28	7	2.857	0.48	40	u	0.059699
			208430_s_at	0	2.719	0.39	7	3.069	1.04	40	u	0.393806
			210091_s_at	0	2.854	0.22	7	3.325	1.06	40	u	0.018552
			210611_s_at	0	2.991	0.17	7	3.396	0.95	40	u	0.019243
			205741_s_at	0	3.493	0.25	7	4.581	1.51	40	u	0.000158
YTHDF3	22	YTH domain family, member 3	221749_at	22	9.472	0.26	7	9.729	0.62	40	u	0.293216
			1564053_a_at	3	5.751	0.95	7	6.736	1.94	40	u	0.204617
NEUROD1	22	neurogenic differentiation 1	206282_at	22	2.061	0.07	7	2.155	0.32	40	u	0.111563
			1556057_s_at	17	2.403	0.07	7	2.565	0.27	40	u	0.003553
ARID4B	22	AT rich interactive domain 4B (RBP1-like)	223111_x_at	22	6.248	0.4	7	7.398	0.68	40	u	0.000113
			221230_s_at	22	9.573	0.33	7	9.781	0.47	40	u	0.274621
			235959_at	0	5.554	0.57	7	6.576	0.98	40	u	0.01151
			224322_at	0	2.539	0.14	7	2.793	0.32	40	u	0.05183
BCL11B	22	B-cell CLL/lymphoma 11B (zinc finger protein)	222895_s_at	22	4.935	0.61	7	4.513	1.42	40	d	0.454038
			219528_s_at	22	4.491	0.69	7	4.879	0.99	40	u	0.333898
			224310_s_at	0	2.418	0.09	7	2.482	0.19	40	u	0.398772
ZBTB4	22	zinc finger and BTB domain containing 4	225629_s_at	22	10.18	0.26	7	8.586	0.62	40	d	0
			227047_x_at	21	7.636	0.3	7	6.762	0.51	40	d	0.000095
CCND2	22	cyclin D2	200951_s_at	22	3.554	0.53	7	3.431	0.89	40	d	0.732378
			200953_s_at	22	10.42	0.21	7	7.917	1.42	40	d	0
			200952_s_at	22	2.849	0.37	7	2.844	0.86	40	d	0.987441
NFAT5	21	nuclear factor of activated T-cells 5, tonicity-responsive	224984_at	21	10.63	0.24	7	9.203	0.7	40	d	0
			208003_s_at	2	6.764	0.77	7	7.243	1.08	40	u	0.277704
			215092_s_at	0	6.121	0.7	7	6.321	0.62	40	u	0.454544
VCP	21	valosin-containing protein	208648_at	7	8.337	0.29	7	8.227	0.56	40	d	0.619084
			208649_s_at	4	9.828	0.15	7	9.625	0.68	40	d	0.114606
NCOA1	21	nuclear receptor coactivator 1	209105_at	21	7.21	0.24	7	6.833	0.98	40	d	0.049506
			209106_at	21	8.736	0.22	7	7.693	0.75	40	d	0
			210249_s_at	6	8.873	0.38	7	8.003	0.54	40	d	0.000245
			209107_x_at	6	8.897	0.31	7	8.047	0.6	40	d	0.000887
			231289_at	0	2.484	0.19	7	2.502	0.35	40	u	0.897164
RAB10	21	RAB10, member RAS oncogene family	222980_at	21	10.6	0.1	7	11.08	0.45	40	u	0.000001
			222981_s_at	3	9.005	0.34	7	10.27	0.61	40	u	0.000004
SATB2	21	SATB homeobox 2	213435_at	21	3.939	0.56	7	4.255	1.5	40	u	0.592176
			215591_at	0	3.362	0.19	7	3.538	0.48	40	u	0.356444
ZNF238	21	zinc finger protein 238	212774_at	21	9.456	0.88	7	8.976	1.6	40	d	0.453125
			207164_s_at	6	5.058	1.09	7	5.308	1.67	40	u	0.710153
SP1	21	Sp1 transcription factor	224760_at	21	9.423	0.3	7	8.056	0.77	40	d	0.000042
			224754_at	21	9.601	0.2	7	8.936	0.49	40	d	0.001189
			1553685_s_at	11	6.989	0.67	7	6.605	1.32	40	d	0.462898
			214732_at	8	4.629	0.38	7	5.087	1.11	40	u	0.063575
MBNL1	21	muscleblind-like (Drosophila)	201151_s_at	21	7.672	0.72	7	7.87	1.71	40	u	0.768176
			201153_s_at	21	10.1	0.41	7	9.616	0.63	40	d	0.058935
			201152_s_at	21	10.71	0.28	7	10.28	0.74	40	d	0.147676
			1555594_a_at	0	4.5	0.58	7	5.384	1.5	40	u	0.139796
			235879_at	0	8.549	0.3	7	8.096	1.06	40	d	0.037102
			215663_at	0	3.109	0.09	7	3.394	0.66	40	u	0.014714
			1558111_at	0	7.896	0.37	7	7.315	1.25	40	d	0.027572
			233015_at	0	2.46	0.15	7	2.777	0.48	40	u	0.003518
EFNB2	21	ephrin-B2	202668_at	21	8.527	0.48	7	7.753	1.1	40	d	0.078722
			202669_s_at	0	7.151	0.5	7	6.342	1.54	40	d	0.017442
CNOT6	21	CCR4-NOT transcription complex, subunit 6	222476_at	15	8.363	0.28	7	8.034	0.68	40	d	0.221129
			217970_s_at	13	7.795	0.23	7	8.293	0.64	40	u	0.001679
PAPPA	21	pregnancy-associated plasma protein A, pappalysin 1	228128_x_at	21	3.375	0.38	7	3.532	0.69	40	u	0.567543
			224940_s_at	19	4.179	0.34	7	4.639	0.75	40	u	0.12608
			224941_at	19	3.42	0.17	7	3.681	0.38	40	u	0.086719
			224942_at	19	2.232	0.38	7	2.197	0.47	40	d	0.85594
			201981_at	8	4.007	0.26	7	3.762	0.33	40	d	0.071162
			201982_s_at	1	4.31	0.21	7	4.454	0.34	40	u	0.290462
			1559928_at	0	2.172	0.04	7	2.287	0.23	40	u	0.005233
			232748_at	0	2.742	0.11	7	2.872	0.3	40	u	0.27031
			1558608_a_at	0	4.851	0.33	7	4.798	0.53	40	d	0.801388
			1558607_at	0	3.705	0.15	7	3.807	0.29	40	u	0.385386

			1559400_s_at	0	3.988	0.47	7	3.937	0.59	40	d	0.832161
FBXO33	21	F-box protein 33	227521_at	21	5.711	0.39	7	5.605	0.71	40	d	0.707545
			226970_at	11	7.213	0.22	7	6.543	0.67	40	d	0.000055
TEAD1	20	TEA domain family member 1 (SV40 transcriptional enhancer factor)	224955_at	20	9.525	0.32	7	8.378	0.86	40	d	0.001361
			214600_at	9	3.895	0.26	7	4.419	0.9	40	u	0.006133
			1553322_s_at	0	2.726	0.25	7	2.995	0.59	40	u	0.253023
TBL1XR1	20	transducin (beta)-like 1X-linked receptor 1	223013_at	20	11.65	0.27	7	11.56	0.61	40	d	0.703542
			221428_s_at	18	8.618	0.43	7	8.506	1.32	40	d	0.687865
			222633_at	9	10.32	0.17	7	11.04	0.62	40	u	0.000002
			222634_s_at	2	7.408	0.26	7	8.402	0.85	40	u	0.000003
			233633_at	0	2.507	0.04	7	2.72	0.25	40	u	0.00001
NHS	20	Nance-Horan syndrome (congenital cataracts and dental anomalies)	228933_at	20	5.686	0.81	7	4.861	1.24	40	d	0.102388
			242800_at	0	2.644	0.05	7	2.818	0.39	40	u	0.010426
KLF13	20	Kruppel-like factor 13	225390_s_at	20	8.81	0.44	7	8.108	0.91	40	d	0.056785
			219878_s_at	0	5.14	0.67	7	5.217	0.8	40	u	0.814972
ZEB2	20	zinc finger E-box binding homeobox 2	203603_s_at	20	5.838	0.87	7	5.503	0.91	40	d	0.380888
			235593_at	0	2.856	0.19	7	3.246	0.7	40	u	0.007208
			233031_at	0	2.938	0.17	7	3.144	0.35	40	u	0.14594
			233033_at	0	2.64	0.23	7	2.833	0.23	40	u	0.053942
MAP3K3	19	mitogen-activated protein kinase kinase kinase 3	227131_at	24	6.657	0.21	7	5.733	0.86	40	d	0.000001
			203514_at	11	8.541	0.19	7	7.73	0.69	40	d	0.000001
PCDHA13	19	protocadherin alpha 13	no probeset available									
PPARGC1A	19	peroxisome proliferator-activated receptor gamma, coactivator 1 alpha	219195_at	19	3.385	0.36	7	3.637	0.74	40	u	0.392607
			1569141_a_at	0	2.531	0.04	7	2.667	0.21	40	u	0.000768
PCDHA12	19	protocadherin alpha 12	no probeset available									
MAP2K4	19	mitogen-activated protein kinase kinase 4	203266_s_at	19	8.468	0.37	7	7.675	0.99	40	d	0.046142
			203265_s_at	19	6.889	0.54	7	7.031	1	40	u	0.720962
TCF12	19	transcription factor 12 (HTF4, helix-loop-helix transcription factors 4)	208986_at	19	9.078	0.24	7	8.96	0.52	40	d	0.565892
			215611_at	0	5.623	0.55	7	4.637	1.04	40	d	0.020999
			235925_at	0	7.505	0.38	7	6.932	0.95	40	d	0.131696
PCDHA2	19	protocadherin alpha 2	210572_at	0	2.758	0.11	7	2.877	0.25	40	u	0.22878
			211365_s_at	0	2.144	0.06	7	2.198	0.07	40	u	0.067338
PCDHA11	19	protocadherin alpha 11	no probeset available									
PCDHA9	19	protocadherin alpha 9	208205_at	0	4.123	0.73	7	4.155	0.83	40	u	0.924673
PCDHAC2	19	protocadherin alpha subfamily C, 2	224539_s_at	0	2.195	0.03	7	2.246	0.06	40	u	0.045401
PCDHA10	19	protocadherin alpha 10	211867_s_at	0	2.904	0.17	7	2.619	0.31	40	d	0.025465
TP53INP1	19	tumor protein p53 inducible nuclear protein 1	225912_at	19	9.629	0.45	7	9.398	1.37	40	d	0.428664
			244721_at	0	3.825	0.33	7	3.931	0.59	40	u	0.655077
			235602_at	0	2.221	0.04	7	2.324	0.13	40	u	0.000814
NAV1	19	neuron navigator 1	224772_at	19	7.141	0.6	7	6.838	1.52	40	d	0.613716
			224773_at	19	4.831	0.57	7	5.027	1.38	40	u	0.7185
			224774_s_at	19	5.616	0.32	7	6.507	1.13	40	u	0.000368
			224770_s_at	19	5.794	0.31	7	5.939	1.06	40	u	0.500855
			224771_at	19	7.162	0.46	7	7.554	1.46	40	u	0.203967
			233567_at	2	2.494	0.39	7	2.673	0.46	40	u	0.345107
			233870_at	0	2.499	0.17	7	2.733	0.59	40	u	0.055515
PCDHA1	19	protocadherin alpha 1	210674_s_at	19	5.364	0.85	7	4.497	1.52	40	d	0.156567
			223435_s_at	19	6.919	0.81	7	5.481	1.99	40	d	0.072254
			224212_s_at	2	4.675	0.73	7	3.95	1.53	40	d	0.236851
FBXL11	19	F-box and leucine-rich repeat protein 11	208989_s_at	19	7.855	0.31	7	7.466	0.84	40	d	0.240173
			208987_s_at	19	8.65	0.22	7	8.845	0.73	40	u	0.192163
			208988_at	19	8.826	0.44	7	8.904	0.76	40	u	0.798139
PCDHA8	19	protocadherin alpha 8	no probeset available									
KCNJ2	19	potassium inwardly-rectifying channel, subfamily J, member 2	206765_at	19	6.985	0.54	7	5.411	0.95	40	d	0.00013
PCDHAC1	19	protocadherin alpha subfamily C, 1	1553235_at	0	2.161	0.06	7	2.271	0.18	40	u	0.005708
			1553237_x_at	0	2.699	0.09	7	2.873	0.24	40	u	0.072269
FRMD4A	19	FERM domain containing 4A	208476_s_at	0	5.921	0.31	7	5.631	1.3	40	d	0.239247
			1554034_a_at	0	2.208	0.02	7	2.364	0.31	40	u	0.00364
			1560031_at	0	5.471	0.66	7	5.222	0.92	40	d	0.503926
			225167_at	0	7.592	0.3	7	7.842	1.02	40	u	0.232126
			225168_at	0	7.606	0.27	7	7.64	0.92	40	u	0.853378
			225163_at	0	7.223	0.29	7	6.542	1.18	40	d	0.004077
			208475_at	0	5.162	0.32	7	5.227	0.87	40	u	0.84928

			1554033_at	0	2.726	0.05	7	2.825	0.13	40	u	0.056993
PCDHA7	19	protocadherin alpha 7	no probeset available									
LPHN3	19	latrophilin 3	209866_s_at	19	4.467	0.88	7	3.812	1.02	40	d	0.125503
			209867_s_at	19	3.439	0.79	7	3.077	0.79	40	d	0.281404
			236264_at	0	2.744	0.5	7	2.528	0.5	40	d	0.309289
			242186_x_at	0	2.758	0.06	7	2.981	0.38	40	u	0.001498
PCDHA5	19	protocadherin alpha 5	211838_x_at	0	2.695	0.19	7	2.695	0.28	40	u	0.99891
BAZ2B	19	bromodomain adjacent to zinc finger domain, 2B	203080_s_at	19	8.857	0.32	7	8.106	0.87	40	d	0.031567
FBXW7	19	F-box and WD repeat domain containing 7	229419_at	19	7.502	0.58	7	6.703	0.71	40	d	0.008701
			222729_at	16	6.652	0.71	7	6.26	0.75	40	d	0.216971
			218751_s_at	0	7.959	0.45	7	6.938	0.75	40	d	0.001323
PCDHA6	19	protocadherin alpha 6	211811_s_at	0	2.055	0.05	7	2.133	0.37	40	u	0.220753
MLL	19	myeloid/lymphoid or mixed-lineage leukemia (trithorax homolog, Drosophila)	212080_at	14	8.462	0.61	7	6.676	0.89	40	d	0.00001
			212079_s_at	14	5.838	0.57	7	6.079	1.1	40	u	0.579995
			212076_at	14	7.581	0.48	7	6.334	0.91	40	d	0.001163
			212078_s_at	14	7.265	0.46	7	6.865	0.83	40	d	0.23014
			1559856_s_at	0	3.485	0.55	7	3.714	0.66	40	u	0.397597
			226981_at	0	8.665	0.48	7	7.358	0.86	40	d	0.000395
			216624_s_at	0	3.67	0.32	7	3.755	0.56	40	u	0.705752
			244110_at	0	3.534	0.44	7	3.277	0.63	40	d	0.314664
			1565436_s_at	0	3.792	0.27	7	4.024	0.6	40	u	0.328817
PCDHA4	19	protocadherin alpha 4	no probeset available									
JAG1	19	jagged 1 (Alagille syndrome)	209097_s_at	19	4.494	0.37	7	4.636	0.4	40	u	0.390424
			209099_x_at	18	9.524	0.39	7	8.584	1.13	40	d	0.000638
			216268_s_at	18	8.964	0.37	7	7.982	1.12	40	d	0.000297
			231183_s_at	18	6.106	0.74	7	5.548	1.04	40	d	0.189657
			209098_s_at	11	6.032	0.43	7	5.022	1.19	40	d	0.000716
			229924_s_at	0	2.479	0.31	7	2.312	0.18	40	d	0.241953
PCDHA3	19	protocadherin alpha 3	211870_s_at	0	2.482	0.32	7	2.341	0.35	40	d	0.328348
CHD9	18	chromodomain helicase DNA binding protein 9	212616_at	18	10.95	0.2	7	9.737	0.65	40	d	0
			212615_at	18	8.329	0.28	7	7.451	0.68	40	d	0.001895
			240367_at	0	3.759	0.17	7	3.892	0.39	40	u	0.386891
			243660_at	0	2.733	0.36	7	2.773	0.36	40	u	0.792139
			220586_at	0	3.813	0.6	7	3.287	0.56	40	d	0.032359
			239654_at	0	7.628	0.29	7	6.572	0.86	40	d	0.000004
			235388_at	0	4.42	0.56	7	4.878	0.99	40	u	0.24803
			229586_at	0	8.42	0.32	7	8.042	0.73	40	d	0.191836
PAFAH1B1	18	platelet-activating factor acetylhydrolase, isoform Ib, alpha subunit 45kDa	200813_s_at	18	8.422	0.28	7	7.535	0.75	40	d	0.004084
			200816_s_at	18	10.02	0.19	7	9.013	0.55	40	d	0
			211547_s_at	8	6.313	0.69	7	6.366	1.26	40	u	0.914771
			200815_s_at	0	7.771	0.29	7	8.565	0.73	40	u	0.007948
EBF3	18	early B-cell factor 3	227243_s_at	18	4.776	0.63	7	3.805	0.94	40	d	0.01325
			227242_s_at	18	2.618	0.48	7	2.481	0.84	40	d	0.683734
			229621_x_at	15	3.045	0.21	7	3.129	0.4	40	u	0.598357
			1559796_at	0	2.269	0.19	7	2.371	0.34	40	u	0.458351
BAZ2A	18	bromodomain adjacent to zinc finger domain, 2A	201355_s_at	18	5.884	0.18	7	5.688	0.3	40	d	0.105021
			201353_s_at	18	6.971	0.51	7	6.752	0.91	40	d	0.548189
			201354_s_at	18	7.545	0.18	7	7.153	0.64	40	d	0.003767
			215437_x_at	7	2.572	0.2	7	2.655	0.33	40	u	0.526219
TRPS1	18	trichorhinophalangeal syndrome I	222651_s_at	18	11.71	0.36	7	11.66	1	40	d	0.831101
			218502_s_at	18	10.4	0.51	7	10.47	1	40	u	0.848679
			224218_s_at	6	9.073	0.54	7	9.892	1.35	40	u	0.129769
			234351_x_at	3	8.373	0.49	7	9.325	1.31	40	u	0.069261
MORF4LP1	18	mortality factor 4 like pseudogene 1	no probeset available									
IGF2BP1	18	insulin-like growth factor 2 mRNA binding protein 1	227377_at	18	2.105	0.03	7	2.151	0.06	40	u	0.061038
			241574_s_at	1	2.477	0.03	7	2.534	0.07	40	u	0.045035
			223689_at	0	2.307	0.13	7	2.334	0.06	40	u	0.637199
HDAC4	18	histone deacetylase 4	204225_at	18	5.136	0.68	7	4.072	1.26	40	d	0.037967
			1554322_a_at	1	2.919	0.1	7	3.015	0.18	40	u	0.194682
			228813_at	0	4.15	0.73	7	3.256	1.1	40	d	0.048268
SEMA6D	18	sema domain, transmembrane domain (TM), and cytoplasmic domain, (semaphorin) 6D	226492_at	18	8.412	0.6	7	4.587	1.61	40	d	0
			233882_s_at	11	5.254	0.83	7	3.27	1.27	40	d	0.000311
			233801_s_at	5	2.851	0.41	7	2.663	1	40	d	0.632969

		and cytoplasmic domain, (semaphorin) 6D	220574_at	0	4.232	0.78	7	3.401	1.19	40	d	0.08722
			244746_at	0	2.856	0.21	7	2.742	0.15	40	d	0.098899
SP3	18	Sp3 transcription factor	213168_at	18	10.08	0.22	7	10.39	0.53	40	u	0.151297
			232529_at	0	5.568	0.6	7	5.971	1.05	40	u	0.338664
			238035_at	0	7.449	0.36	7	7.157	0.58	40	d	0.213832
			229217_at	0	3.512	0.47	7	3.632	0.49	40	u	0.558323
			227537_s_at	0	2.887	0.21	7	2.917	0.34	40	u	0.822156
VEZF1	18	vascular endothelial zinc finger 1	202172_at	18	8.905	0.32	7	8.406	0.63	40	d	0.051751
			202171_at	18	9.293	0.31	7	9.322	0.67	40	u	0.912676
			202173_s_at	8	8.938	0.19	7	9.421	0.84	40	u	0.003256
ARRDC3	18	arrestin domain containing 3	224797_at	18	8.923	0.37	7	7.557	0.74	40	d	0.000027
HIPK1	18	homeodomain interacting protein kinase 1	212293_at	18	8.665	0.27	7	8.422	0.6	40	d	0.309413
			212291_at	18	7.083	0.5	7	7.065	0.85	40	d	0.958144
			1552515_at	0	2.395	0.23	7	2.396	0.19	40	u	0.991516
			1552516_a_at	0	4.611	0.57	7	4.577	1.15	40	d	0.940015
CPD	17	carboxypeptidase D	201940_at	17	7.753	0.5	7	7.442	1.03	40	d	0.448843
			201941_at	17	8.729	0.34	7	8.01	0.99	40	d	0.002102
			201943_s_at	9	7.336	0.48	7	7.587	1.25	40	u	0.610254
			201942_s_at	1	5.312	0.88	7	5.944	1.62	40	u	0.328941
			229600_s_at	0	2.25	0.12	7	2.338	0.18	40	u	0.224776
RUNX2	17	runt-related transcription factor 2	232231_at	17	6.624	0.46	7	6.598	1.47	40	d	0.932024
			236859_at	0	4.163	0.38	7	4.645	0.82	40	u	0.142377
			221283_at	0	2.239	0.04	7	2.346	0.21	40	u	0.007044
			221282_x_at	0	2.024	0.09	7	2.086	0.12	40	u	0.208177
			216994_s_at	0	3.018	0.34	7	3.079	0.35	40	u	0.678418
MEF2C	17	myocyte enhancer factor 2C	209199_s_at	17	8.326	0.53	7	6.903	1.34	40	d	0.009541
			209200_at	17	8.129	0.58	7	6.722	1.27	40	d	0.007302
			207968_s_at	1	3.155	0.46	7	3.083	0.86	40	d	0.833569
DCUN1D4	17	DCN1, defective in cullin neddylation 1, domain containing 4 (S. cerevisiae)	212853_at	17	4.886	0.4	7	5.444	0.88	40	u	0.11265
			212855_at	17	8.195	0.21	7	8.035	0.55	40	d	0.457862
			212851_at	17	7.032	0.32	7	7.113	0.77	40	u	0.7896
PLAGL2	17	pleiomorphic adenoma gene-like 2	202924_s_at	17	3.281	0.33	7	3.939	0.89	40	u	0.065388
			202925_s_at	17	5.216	0.48	7	5.395	0.87	40	u	0.605933
RBP1	17	retinol binding protein 1, cellular	203423_at	0	9.263	0.67	7	8.335	1.96	40	d	0.035147
MITF	17	microphthalmia-associated transcription factor	226066_at	17	8.16	0.59	7	7.184	0.73	40	d	0.002099
			207233_s_at	4	7.476	0.54	7	6.659	0.63	40	d	0.002814
			1554874_at	0	2.492	0.12	7	2.718	0.36	40	u	0.006498
TRIM2	17	tripartite motif-containing 2	215945_s_at	17	8.301	0.66	7	7.481	1.54	40	d	0.181511
			202342_s_at	17	8.842	0.4	7	7.765	1.64	40	d	0.001333
			202341_s_at	17	9.375	0.76	7	8.608	1.62	40	d	0.236615
			214248_s_at	0	2.793	0.54	7	3.109	1.04	40	u	0.447781
			214249_at	0	2.978	0.49	7	2.767	0.32	40	d	0.154443
			243945_at	0	2.873	0.14	7	3.183	0.51	40	u	0.003432
TRAM1	17	translocation associated membrane protein 1	201398_s_at	10	11.48	0.26	7	11.55	0.73	40	u	0.652839
			210733_at	10	6.05	0.32	7	7.064	1.11	40	u	0.000068
			201399_s_at	0	10.12	0.49	7	10.51	1.4	40	u	0.211289
PHLPPL	17	PH domain and leucine rich repeat protein phosphatase-like	213407_at	17	7.02	0.45	7	7.036	0.73	40	u	0.956612
			214944_at	0	2.262	0.19	7	2.454	0.16	40	u	0.007224
RAP1B	17	RAP1B, member of RAS oncogene family	200833_s_at	24	11.21	0.23	7	11.06	0.58	40	d	0.519014
SIX4	17	SIX homeobox 4	229796_at	17	6.862	0.61	7	7.762	1.16	40	u	0.055697
			231797_at	1	2.933	0.25	7	3.104	0.32	40	u	0.190587
BTBD7	17	BTB (POZ) domain containing 7	224943_at	17	7.005	0.36	7	6.602	0.85	40	d	0.233492
			224945_at	17	8.326	0.36	7	7.386	0.58	40	d	0.000176
			1556000_s_at	6	7.357	0.44	7	6.585	0.48	40	d	0.00036
			217141_at	0	5.046	0.49	7	5.162	0.54	40	u	0.605893
			220297_at	0	3.092	0.21	7	3.209	0.5	40	u	0.553986
DLGAP2	17	discs, large (Drosophila) homolog-associated protein 2	210227_at	17	1.944	0.02	7	1.991	0.07	40	u	0.001031
			216916_s_at	0	1.94	0.01	7	2.011	0.18	40	u	0.017059
MAT2A	17	methionine adenosyltransferase II, alpha	200768_s_at	17	11.03	0.23	7	10.63	0.67	40	d	0.009304
			200769_s_at	1	7.839	0.72	7	7.428	1.65	40	d	0.528179
NR4A2	16	nuclear receptor subfamily 4, group A, member 2	216248_s_at	16	7.796	1.11	7	6.298	1.41	40	d	0.012217
			204621_s_at	16	8.692	1.1	7	6.931	1.36	40	d	0.002626
			204622_x_at	16	7.78	0.91	7	6.29	1.58	40	d	0.022322
			204685_s_at	16	2.486	0.08	7	2.731	0.72	40	u	0.046702

ATP2B2	16	ATPase, Ca++ transporting, plasma membrane 2	216120_s_at	3	2.334	0.15	7	2.392	0.37	40	u	0.6882
			234741_at	0	2.583	0.16	7	2.657	0.23	40	u	0.425261
			234714_x_at	0	4.396	0.18	7	4.486	0.31	40	u	0.463472
			211586_s_at	0	2.332	0.06	7	2.461	0.32	40	u	0.031213
HBP1	16	HMG-box transcription factor 1	209102_s_at	16	9.895	0.32	7	8.973	0.69	40	d	0.001356
			207361_at	0	4.125	0.52	7	3.943	0.85	40	d	0.59352
			236645_at	0	2.645	0.23	7	3.373	0.87	40	u	0.000112
ZBTB41	16	zinc finger and BTB domain containing 41	226962_at	16	7.208	0.59	7	8.034	0.74	40	u	0.008694
WDR26	16	WD repeat domain 26	224897_at	16	8.869	0.26	7	8.673	0.71	40	d	0.479915
			224905_at	16	7.155	0.31	7	7.658	0.56	40	u	0.028123
			224898_at	16	6.947	0.39	7	6.806	0.73	40	d	0.629454
			218107_at	6	9.334	0.21	7	9.794	0.65	40	u	0.002156
			223863_at	0	2.598	0.1	7	2.705	0.17	40	u	0.10995
RNF111	16	ring finger protein 111	218761_at	16	8.24	0.24	7	7.554	0.55	40	d	0.002864
AZIN1	16	antizyme inhibitor 1	212461_at	16	10.26	0.25	7	10.48	0.71	40	u	0.173008
			201772_at	8	8.91	0.2	7	9.958	0.88	40	u	0
MAF	16	v-maf musculoaponeurotic fibrosarcoma oncogene homolog (avian)	206363_at	16	6.62	0.89	7	4.161	0.87	40	d	0
			1566323_at	15	2.838	0.12	7	2.975	0.16	40	u	0.037039
			1566324_a_at	15	3.148	0.55	7	2.96	0.24	40	d	0.440021
			209348_s_at	14	8.748	0.4	7	7.009	0.81	40	d	0.000002
			209347_s_at	14	4.485	0.65	7	3.873	0.37	40	d	0.066082
AKT3	16	v-akt murine thymoma viral oncogene homolog 3 (protein kinase B, gamma)	222880_at	9	4.171	0.52	7	5.375	1.85	40	u	0.002362
			219393_s_at	2	2.685	0.08	7	3.294	0.94	40	u	0.000309
			212609_s_at	0	7.896	0.38	7	7.37	1.5	40	d	0.074576
			242876_at	0	6.341	0.49	7	6.009	1.11	40	d	0.449426
			212607_at	0	7.209	0.54	7	7.251	1.96	40	u	0.912573
			224229_s_at	0	2.851	0.32	7	3.696	1.42	40	u	0.002556
YOD1	16	YOD1 OTU deubiquinating enzyme 1 homolog (S. cerevisiae)	227309_at	16	6.443	0.26	7	6.906	1.52	40	u	0.089398
			215150_at	0	2.732	0.14	7	3.697	1.32	40	u	0.000067
TP53INP2	16	tumor protein p53 inducible nuclear protein 2	224836_at	16	7.381	0.38	7	7.244	0.98	40	d	0.721435
RUNX1	16	runt-related transcription factor 1 (acute myeloid leukemia 1; aml1 oncogene)	209360_s_at	16	9.477	0.47	7	8.317	0.94	40	d	0.003156
			209359_x_at	3	5.971	0.5	7	5.699	0.5	40	d	0.197432
			211180_x_at	3	6.067	0.3	7	5.779	0.44	40	d	0.108846
			208129_x_at	3	6.146	0.34	7	5.722	0.53	40	d	0.0523
			211182_x_at	0	3.372	0.57	7	3.502	0.92	40	u	0.723354
			217263_x_at	0	3.753	0.59	7	3.699	0.86	40	d	0.878707
			211181_x_at	0	3.275	0.64	7	3.537	0.96	40	u	0.502492
			210805_x_at	0	2.69	0.32	7	2.805	0.48	40	u	0.554518
			211620_x_at	0	4.208	0.7	7	3.978	0.71	40	d	0.443806
			211179_at	0	3.115	0.3	7	3.25	0.38	40	u	0.393409
			210365_at	0	6.233	1.02	7	4.526	1.19	40	d	0.001075
SGMS1	16	sphingomyelin synthase 1	212989_at	16	6.951	0.27	7	6.958	0.99	40	u	0.96956
NFIX	16	nuclear factor I/X (CCAAT-binding transcription factor)	227400_at	16	9.05	0.34	7	8.36	1.44	40	d	0.01435
			228278_at	6	8.846	0.35	7	7.779	1.43	40	d	0.000329
			209807_s_at	0	5.12	0.42	7	5.585	1.45	40	u	0.119462
			229834_at	0	2.077	0.02	7	2.133	0.12	40	u	0.009368
LPHN1	16	latrophilin 1	219145_at	16	6.396	0.31	7	6.624	0.76	40	u	0.449476
			47560_at	16	5.883	0.22	7	6.134	1.09	40	u	0.208207
			203488_at	4	6.853	0.17	7	6.621	0.96	40	d	0.180073
ZNF800	16	zinc finger protein 800	227101_at	0	5.025	0.99	7	6.738	0.85	40	u	0.000026
			227104_x_at	0	4.035	0.29	7	4.153	0.4	40	u	0.472772
MTRF1	16	mitochondrial translational release factor 1	219822_at	0	5.826	0.35	7	5.335	0.79	40	d	0.120644
			244763_at	0	3.532	0.34	7	3.837	0.62	40	u	0.219238
			242996_at	0	2.88	0.21	7	3.163	0.53	40	u	0.178548
EPHA7	16	EPH receptor A7	238533_at	16	2.482	0.39	7	2.358	0.05	40	d	0.461268
			206852_at	9	2.552	0.06	7	2.62	0.12	40	u	0.172841
			1554629_at	0	3.043	0.43	7	3.105	0.4	40	u	0.718778
NR3C2	16	nuclear receptor subfamily 3, group C, member 2	205259_at	16	6.564	0.31	7	4.021	0.98	40	d	0
RC3H1	16	ring finger and CCCH-type zinc finger domains 1	228996_at	16	3.8	0.67	7	4.941	0.95	40	u	0.004686
CRIM1	16	cysteine rich transmembrane BMP regulator 1 (chordin-like)	202552_s_at	16	7.723	0.37	7	5.809	1.14	40	d	0
			228496_s_at	16	9.878	0.14	7	8.276	1.11	40	d	0

		1 (chordin-like)	202551_s_at	16	10.35	0.25	7	8.098	1.15	40	d	0
ETF1	16	eukaryotic translation termination factor 1	201574_at	16	10.55	0.3	7	10.08	0.58	40	d	0.050429
			201573_s_at	6	8.631	0.37	7	8.885	0.67	40	u	0.341285
SLC36A1	16	solute carrier family 36 (proton/amino acid symporter), member 1	213119_at	3	6.331	0.31	7	6.908	0.56	40	u	0.012578
			1553272_at	2	2.878	0.43	7	2.997	0.41	40	u	0.498999
FAM46A	16	family with sequence similarity 46, member A	224973_at	16	5.076	0.7	7	4.676	1.06	40	d	0.353622
			221766_s_at	16	9.837	0.63	7	8.554	1.16	40	d	0.007681
			229737_at	0	2.821	0.07	7	2.861	0.12	40	u	0.42429
PCDH10	16	protocadherin 10	228635_at	16	2.622	0.56	7	2.73	0.7	40	u	0.7027
			1552925_at	0	2.404	0.16	7	2.421	0.19	40	u	0.828674
CTDSPL	16	CTD (carboxy-terminal domain, RNA polymerase II, polypeptide A) small phosphatase-like	201906_s_at	16	9.196	0.19	7	7.919	0.78	40	d	0
			201905_s_at	16	5.436	0.46	7	5.388	0.87	40	d	0.889413
			201904_s_at	16	8.465	0.22	7	7.75	0.88	40	d	0.000146
			214187_x_at	0	2.031	0.18	7	2.006	0.15	40	d	0.698585
			213597_s_at	0	2.166	0.03	7	2.263	0.21	40	u	0.007511
OXSR1	16	oxidative-stress responsive 1	202696_at	16	8.565	0.27	7	8.312	0.59	40	d	0.280135
DYRK1A	16	dual-specificity tyrosine-(Y)-phosphorylation regulated kinase 1A	209033_s_at	16	9.562	0.23	7	9.347	0.47	40	d	0.250989
			211541_s_at	0	2.915	0.5	7	3.225	0.49	40	u	0.136362
			211079_s_at	0	3.178	0.08	7	3.371	0.41	40	u	0.011804
			239308_at	0	3.544	0.43	7	3.276	0.25	40	d	0.183865
BRPF3	16	bromodomain and PHD finger containing, 3	225217_s_at	16	6.484	0.63	7	6.123	0.91	40	d	0.327782
			1565844_at	0	2.433	0.19	7	2.5	0.38	40	u	0.659426
			1565843_s_at	0	2.989	0.33	7	3.187	0.52	40	u	0.341962
BTG2	15	BTG family, member 2	201235_s_at	15	7.631	1.17	7	6.556	1.47	40	d	0.079681
			201236_s_at	15	9.901	0.82	7	8.153	1.53	40	d	0.005901
KBTBD8	15	kelch repeat and BTB (POZ) domain containing 8	239835_at	15	3.746	0.53	7	4.301	0.85	40	u	0.108798
ISL1	15	ISL LIM homeobox 1	206104_at	15	2.168	0.13	7	2.261	0.37	40	u	0.249664
SH3PXD2A	15	SH3 and PX domains 2A	224817_at	15	7.928	0.38	7	7.08	1.03	40	d	0.041852
			213252_at	8	6.683	0.69	7	6.324	0.78	40	d	0.269136
			207661_s_at	3	3.076	0.16	7	3.377	0.7	40	u	0.025097
CHD7	15	chromodomain helicase DNA binding protein 7	222755_s_at	15	5.523	0.27	7	5.964	0.91	40	u	0.02311
			218829_s_at	15	6.459	0.23	7	6.688	1.35	40	u	0.334338
			220619_at	0	2.609	0.06	7	2.95	0.51	40	u	0.000211
			226123_at	0	8.553	0.38	7	8.297	1.29	40	d	0.325986
NLK	15	nemo-like kinase	222589_at	14	7.427	0.25	7	7.616	0.89	40	u	0.293138
			218318_s_at	7	5.863	0.65	7	6.308	0.93	40	u	0.239814
			222590_s_at	4	5.648	0.65	7	6.418	0.83	40	u	0.026665
PIK3R1	15	phosphoinositide-3-kinase, regulatory subunit 1 (p85 alpha)	212240_s_at	15	10.63	0.47	7	9.001	1.22	40	d	0.001339
			212249_at	15	9	0.68	7	7.16	1.15	40	d	0.000213
			212239_at	15	10.44	0.17	7	8.322	0.87	40	d	0
PDGFRA	15	platelet-derived growth factor receptor, alpha polypeptide	203131_at	15	10.82	0.22	7	7.804	1.07	40	d	0
			211533_at	1	2.277	0.03	7	2.394	0.2	40	u	0.001341
			215305_at	0	4.058	0.69	7	3.59	0.44	40	d	0.025412
			1554828_at	0	2.046	0.02	7	2.093	0.09	40	u	0.00613
TLE4	15	transducin-like enhancer of split 4 (E(sp1) homolog, Drosophila)	204872_at	15	7.77	0.42	7	6.198	1.16	40	d	0.000002
			214688_at	8	3.769	0.39	7	3.885	0.5	40	u	0.570147
			233575_s_at	0	3.483	0.27	7	2.728	0.4	40	d	0.000024
			216997_x_at	0	4.072	0.93	7	3.34	0.63	40	d	0.014093
			235765_at	0	3.827	0.32	7	4.031	0.59	40	u	0.384045
LONRF1	15	LON peptidase N-terminal domain and ring finger 1	226038_at	15	9.243	0.28	7	8.016	0.9	40	d	0
			236556_s_at	0	3.417	0.13	7	3.437	0.14	40	u	0.72042
DLG3	15	discs, large homolog 3 (neuroendocrine-dlg, Drosophila)	212728_at	15	6.104	0.22	7	5.906	0.88	40	d	0.244105
			212727_at	15	7.815	0.39	7	7.752	0.56	40	d	0.780358
			212729_at	15	6.807	0.54	7	7.076	0.92	40	u	0.468923
			207732_s_at	2	5.547	0.64	7	6.391	1.12	40	u	0.064598
ANK2	15	ankyrin 2, neuronal	202920_at	15	7.644	0.9	7	4.926	1.21	40	d	0.000001
			202921_s_at	6	3.834	1.26	7	2.859	0.55	40	d	0.110659
			216195_at	0	2.501	0.16	7	2.542	0.25	40	u	0.676295
SLC1A2	15	solute carrier family 1 (glial high affinity glutamate transporter), member 2	225491_at	15	5.689	0.73	7	5.005	1.38	40	d	0.217743
			1558010_s_at	10	2.309	0.04	7	2.473	0.23	40	u	0.000232
			1558009_at	10	3.029	0.2	7	2.975	0.15	40	d	0.412243
			208389_s_at	0	3.034	0.28	7	3.258	0.57	40	u	0.323877
JAZF1	15	JAZF zinc finger 1	225798_at	15	7.47	0.32	7	6.761	0.82	40	d	0.031861

JAZF1	15	JAZF zinc finger 1	225800_at	15	5.093	0.61	7	4.706	0.93	40	d	0.30593
SOX5	15	SRY (sex determining region Y)-box 5	238285_at	15	2.497	0.14	7	2.614	0.22	40	u	0.189362
			207336_at	6	2.078	0.04	7	2.283	0.51	40	u	0.017953
			1569638_at	0	2.357	0.02	7	2.458	0.19	40	u	0.00319
TMCC1	15	transmembrane and coiled-coil domain family 1	227112_at	15	7.089	0.26	7	6.792	0.54	40	d	0.166936
			213351_s_at	12	7.623	0.26	7	7.718	0.74	40	u	0.553196
			213352_at	12	6.484	0.34	7	6.601	0.8	40	u	0.712215
			213349_at	12	6.628	0.57	7	7.055	0.67	40	u	0.1267
			239029_at	0	1.848	0.02	7	1.882	0.03	40	u	0.006532
			239662_x_at	0	6.504	0.23	7	6.471	0.46	40	d	0.857352
CDC2L6	15	cell division cycle 2-like 6 (CDK8-like)	212897_at	15	7.084	0.23	7	7.753	1.01	40	u	0.000931
			212899_at	15	8.264	0.35	7	8.582	1.01	40	u	0.156911
			211706_s_at	1	2.374	0.03	7	2.636	0.55	40	u	0.005637
			235226_at	0	3.774	0.65	7	4.389	1.13	40	u	0.176107
PCDH9	15	protocadherin 9	219738_s_at	15	3.013	0.31	7	2.901	0.12	40	d	0.408945
			219737_s_at	15	3.461	0.99	7	2.685	0.85	40	d	0.038371
E2F3	15	E2F transcription factor 3	203693_s_at	15	5.857	0.64	7	7.782	1.08	40	u	0.000052
			203692_s_at	15	5.53	0.41	7	7.31	0.94	40	u	0.000016
PHTF2	15	putative homeodomain transcription factor 2	209780_at	15	8.025	0.28	7	8.721	0.67	40	u	0.011196
			1554822_at	0	2.751	0.26	7	3.036	0.45	40	u	0.119365
			1554780_a_at	0	3.962	0.29	7	4.53	0.84	40	u	0.004026
			215286_s_at	0	3.562	0.44	7	4.198	0.79	40	u	0.047816
			217097_s_at	0	4.118	0.37	7	5.482	1.02	40	u	0.000003
FAM126B	15	family with sequence similarity 126, member B	1554178_a_at	15	7.344	0.26	7	7.602	0.81	40	u	0.133964
			231874_at	13	4.886	0.54	7	5.059	0.8	40	u	0.591848
TOX	15	thymocyte selection-associated high mobility group box	204530_s_at	15	2.696	0.14	7	2.726	0.12	40	u	0.555008
			204529_s_at	15	3.195	0.26	7	3.823	1.14	40	u	0.004884
BCL6	15	B-cell CLL/lymphoma 6 (zinc finger protein 51)	203140_at	15	9.562	0.49	7	8.351	0.95	40	d	0.002383
			215990_s_at	1	6.329	0.77	7	5.411	0.91	40	d	0.017603
			239249_at	0	5.456	0.23	7	5.361	0.41	40	d	0.563537
			228758_at	0	7.869	0.49	7	6.423	1.09	40	d	0.001564
PPM1E	15	protein phosphatase 1E (PP2C domain containing)	236302_at	15	2.887	0.06	7	3.889	1.13	40	u	0.000002
			205938_at	8	2.825	0.24	7	3.293	0.9	40	u	0.011697
KPNA4	15	karyopherin alpha 4 (importin alpha 3)	225268_at	15	8.329	0.3	7	9.173	0.61	40	u	0.001057
			225267_at	15	8.668	0.25	7	9.775	0.77	40	u	0
			209653_at	0	6.324	0.65	7	7.608	1.5	40	u	0.034494
GPR85	15	G protein-coupled receptor 85	219898_at	10	2.813	0.1	7	3.116	0.37	40	u	0.000195
			234303_s_at	10	2.21	0.03	7	2.425	0.38	40	u	0.001188
ELAVL1	15	ELAV (embryonic lethal, abnormal vision, Drosophila)-like 1 (Hu antigen R)	244660_at	15	2.749	0.15	7	3.02	0.84	40	u	0.073656
			227746_at	10	5.751	1.02	7	4.488	1.08	40	d	0.006989
			201726_at	7	9.579	0.28	7	9.976	0.51	40	u	0.055917
			201727_s_at	0	7.452	0.1	7	7.973	0.95	40	u	0.001952
SIRT1	15	sirtuin (silent mating type information regulation 2 homolog) 1 (S. cerevisiae)	218878_s_at	15	8.031	0.28	7	7.422	0.57	40	d	0.010122
SATB1	15	SATB homeobox 1	203408_s_at	2	9.579	0.24	7	8.298	1.19	40	d	0
ABCA1	15	ATP-binding cassette, sub-family A (ABC1), member 1	203505_at	15	7.952	0.29	7	7.597	0.57	40	d	0.121508
			203504_s_at	7	7.093	0.64	7	6.355	0.98	40	d	0.066099
			215876_at	0	2.393	0.07	7	2.568	0.39	40	u	0.012481
			215869_at	0	3.139	0.21	7	3.216	0.3	40	u	0.526248
			216066_at	0	3.234	0.1	7	3.432	0.23	40	u	0.033007
			1570279_at	0	2.419	0.03	7	2.496	0.1	40	u	0.001211
RAB11FIP2	15	RAB11 family interacting protein 2 (class I)	203883_s_at	15	7.604	0.29	7	7.57	0.49	40	d	0.864381
			203884_s_at	15	5.815	0.8	7	5.364	0.78	40	d	0.174377
FURIN	15	furin (paired basic amino acid cleaving enzyme)	201945_at	15	7.101	0.27	7	7.273	0.67	40	u	0.513956
PHF6	15	PHD finger protein 6	225501_at	15	8.466	0.3	7	9.055	0.67	40	u	0.031252
			224442_at	0	3.658	0.12	7	3.961	0.4	40	u	0.000916
FNDC3A	15	fibronectin type III domain containing 3A	202304_at	15	8.936	0.27	7	8.267	0.75	40	d	0.000454
			215910_s_at	0	3.827	0.6	7	3.726	0.78	40	d	0.750352
			241611_s_at	0	3.749	0.32	7	3.926	0.35	40	u	0.224005
			238961_s_at	0	2.118	0.03	7	2.206	0.18	40	u	0.008295
ADCY6	15	adenylate cyclase 6	209195_s_at	14	8.149	0.43	7	7.249	0.92	40	d	0.0171
			224837_at	15	10.35	0.36	7	9.134	0.98	40	d	0.000009
			224838_at	15	10.66	0.46	7	9.322	1.04	40	d	0.00198

FOXP1	15	forkhead box P1	235444_at	0	7.459	0.57	7	6.274	1.28	40	d	0.022588
			1558996_at	0	6.607	0.58	7	5.961	1.12	40	d	0.150093
			223287_s_at	0	10.53	0.33	7	9.161	1.06	40	d	0.000001
			223937_at	0	5.219	0.2	7	5.31	0.48	40	u	0.629949
			223936_s_at	0	5.621	0.41	7	5.865	0.88	40	u	0.48519
MED13L	14	mediator complex subunit 13-like	212208_at	14	8.508	0.32	7	8.312	0.77	40	d	0.519311
			212207_at	14	7.779	0.32	7	7.767	0.69	40	d	0.964068
			212209_at	14	8.866	0.36	7	8.394	0.97	40	d	0.219278
			242911_at	0	5.624	0.43	7	6.249	0.93	40	u	0.095074
			216109_at	0	6.335	0.73	7	4.961	1.21	40	d	0.006504
LUZP1	14	leucine zipper protein 1	221832_s_at	14	6.846	0.57	7	6.391	0.69	40	d	0.114212
			225831_at	14	7.301	0.3	7	6.487	0.52	40	d	0.000304
			221831_at	14	6.172	0.23	7	5.814	0.48	40	d	0.063614
			1553224_at	0	2.418	0.04	7	2.525	0.2	40	u	0.006014
			1558173_a_at	0	6.847	0.28	7	7.38	0.77	40	u	0.004452
BCL2	14	B-cell CLL/lymphoma 2	203685_at	14	9.219	0.4	7	7.257	2.02	40	d	0.000003
			203684_s_at	14	4.706	0.72	7	4.634	0.91	40	d	0.847182
			207005_s_at	0	4.581	0.28	7	4.295	0.63	40	d	0.25615
			207004_at	0	6.224	0.31	7	5.924	0.53	40	d	0.165455
KLF4	14	Kruppel-like factor 4 (gut)	221841_s_at	14	10.34	1.01	7	7.836	0.97	40	d	0
			220266_s_at	1	8.403	1.23	7	5.887	1.04	40	d	0.000001
DCX	14	doublecortex; lissencephaly, X-linked (doublecortin)	204850_s_at	14	4.05	1.45	7	3.039	1.28	40	d	0.070715
			204851_s_at	3	3.861	1.48	7	2.981	1.51	40	d	0.169903
VEGFA	14	vascular endothelial growth factor A	210512_s_at	14	9.201	0.45	7	10.35	1.01	40	u	0.005559
			212171_x_at	14	8.363	0.52	7	9.392	1.16	40	u	0.028694
			211527_x_at	4	5.927	0.61	7	7.615	1.78	40	u	0.000146
			210513_s_at	0	5.853	0.95	7	7.515	1.65	40	u	0.014784
STAG1	14	stromal antigen 1	202293_at	14	7.292	0.29	7	7.556	0.69	40	u	0.331921
			202294_at	14	9.172	0.23	7	8.527	0.57	40	d	0.005918
			232588_at	0	4.166	0.27	7	4.38	0.62	40	u	0.385867
KLF5	14	Kruppel-like factor 5 (intestinal)	209211_at	14	7.257	0.35	7	7.109	1.44	40	d	0.589339
			209212_s_at	0	7.485	0.67	7	7.057	1.81	40	d	0.548512
BAHD1	14	bromo adjacent homology domain containing 1	203051_at	14	7.8	0.28	7	7.438	0.59	40	d	0.126475
PAN3	14	PAN3 polyA specific ribonuclease subunit homolog (S. cerevisiae)	225563_at	14	10.5	0.25	7	9.675	0.68	40	d	0.000012
MTPN	14	myotrophin	224656_s_at	14	11.14	0.12	7	11.36	0.54	40	u	0.029951
ZER1	14	zer-1 homolog (C. elegans)	202452_at	14	8.297	0.31	7	7.169	0.74	40	d	0.000334
			202448_s_at	14	2.532	0.32	7	2.632	0.67	40	u	0.708997
			202456_s_at	4	6.576	0.31	7	6.143	0.54	40	d	0.04748
			230358_at	0	2.249	0.09	7	2.323	0.17	40	u	0.286922
AP1G1	14	adaptor-related protein complex 1, gamma 1 subunit	225754_at	14	6.893	0.35	7	7.025	0.74	40	u	0.649675
			225771_at	14	9.076	0.32	7	8.253	0.68	40	d	0.003704
			203350_at	5	6.933	0.32	7	7.381	0.82	40	u	0.171079
DCBLD2	14	discoidin, CUB and LCCL domain containing 2	224911_s_at	14	8.159	0.27	7	7.229	0.91	40	d	0.000017
			213865_at	0	2.934	0.36	7	3.414	1.14	40	u	0.049424
			239446_x_at	0	7.797	0.3	7	7.457	0.35	40	d	0.023745
ZBTB39	14	zinc finger and BTB domain containing 39	205256_at	14	5.699	0.31	7	5.85	0.42	40	u	0.37781
EGR3	14	early growth response 3	206115_at	14	9.87	0.9	7	6.409	1.54	40	d	0.000001
ZCCHC14	14	zinc finger, CCHC domain containing 14	212655_at	14	7.812	0.31	7	7.127	0.96	40	d	0.00183
			215427_s_at	0	4.085	0.4	7	4.043	0.59	40	d	0.860032
			215426_at	0	2.434	0.12	7	2.479	0.12	40	u	0.372241
HIVEP2	14	human immunodeficiency virus type I enhancer binding protein 2	212642_s_at	14	8.336	0.27	7	8.077	0.56	40	d	0.245732
			212641_at	14	7.074	0.48	7	6.416	0.91	40	d	0.075025
			243254_at	0	2.479	0.09	7	2.544	0.16	40	u	0.306238
NR5A2	14	nuclear receptor subfamily 5, group A, member 2	210174_at	14	2.961	0.49	7	2.957	0.46	40	d	0.983159
			208343_s_at	8	3.119	0.32	7	3.066	0.18	40	d	0.712049
			208337_s_at	5	2.263	0.01	7	2.322	0.11	40	u	0.00202
			1560469_at	0	2.744	0.23	7	2.719	0.23	40	d	0.795976
MEX3C	14	mex-3 homolog C (C. elegans)	222567_s_at	14	5.647	0.62	7	5.92	1.35	40	u	0.6111
			218247_s_at	14	8.071	0.33	7	8.052	0.82	40	d	0.952112
			1556874_a_at	0	2.296	0.06	7	2.472	0.41	40	u	0.014847
			1556873_at	0	2.909	0.16	7	3.172	0.42	40	u	0.117443
			227275_at	0	2.733	0.35	7	2.805	0.41	40	u	0.669376

C10orf56	14	chromosome 10 open reading frame 56	212419_at	14	8.81	0.33	7	6.261	1.16	40	d	0
			212423_at	14	8.633	0.35	7	5.998	1.17	40	d	0
BSN	14	bassoon (presynaptic cytomatrix protein)	204586_at	14	2.269	0.04	7	2.418	0.58	40	u	0.119314
NIPBL	14	Nipped-B homolog (Drosophila)	212483_at	14	7.207	0.38	7	6.527	0.84	40	d	0.045528
			212469_at	14	7.511	0.38	7	7.097	0.59	40	d	0.087276
			242352_at	0	6.461	0.53	7	7.55	0.83	40	u	0.001937
			207108_s_at	0	7.368	0.28	7	7.28	0.83	40	d	0.622215
			213918_s_at	0	7.54	0.47	7	7.863	0.66	40	u	0.233666
MAPRE1	14	microtubule-associated protein, RP/EB family, member 1	200712_s_at	14	8.247	0.29	7	8.668	1.03	40	u	0.047107
			200713_s_at	14	10.45	0.16	7	10.72	0.42	40	u	0.110333
HLF	14	hepatic leukemia factor	204753_s_at	14	6.238	0.8	7	3.28	0.87	40	d	0
			204754_at	14	6.087	1.8	7	3.486	0.59	40	d	0.012818
			204755_x_at	14	6.587	0.92	7	3.283	0.99	40	d	0
RUNX1T1	14	runt-related transcription factor 1; translocated to, 1 (cyclin D-related)	205529_s_at	14	7.501	0.57	7	4.148	0.89	40	d	0
			205528_s_at	14	6.209	0.5	7	4.017	0.7	40	d	0
			1564642_at	0	2.414	0.13	7	2.523	0.18	40	u	0.146027
			216831_s_at	0	3.259	0.51	7	2.634	0.33	40	d	0.000145
			216832_at	0	3.121	0.28	7	3.201	0.4	40	u	0.623381
ZNF385	14	zinc finger protein 385	226111_s_at	14	7.332	0.4	7	7.45	0.79	40	u	0.705473
WEE1	14	WEE1 homolog (S. pombe)	212533_at	14	9.767	0.61	7	9.133	0.71	40	d	0.034439
			215711_s_at	0	7.36	0.97	7	6.669	1.43	40	d	0.233631
ZNF608	14	zinc finger protein 608	229817_at	14	7.719	0.51	7	5.727	1.3	40	d	0.000318
			232303_at	0	4.176	0.37	7	3.907	0.64	40	d	0.294822
PUM2	14	pumilio homolog 2 (Drosophila)	216221_s_at	14	10.24	0.13	7	9.92	0.45	40	d	0.001481
			201493_s_at	14	9.67	0.29	7	9.952	0.55	40	u	0.20024
PPP3R1	14	protein phosphatase 3 (formerly 2B), regulatory subunit B, alpha isoform	204506_at	14	6.309	0.45	7	6.268	0.56	40	d	0.8581
			204507_s_at	10	3.702	0.58	7	4.525	0.9	40	u	0.027121
TSC22D2	14	TSC22 domain family, member 2	215547_at	14	2.168	0.07	7	2.223	0.14	40	u	0.309058
			204094_s_at	14	8.864	0.45	7	8.453	0.74	40	d	0.168039
			210953_at	1	3.909	0.33	7	3.76	0.34	40	d	0.298571
			210954_s_at	1	3.034	0.42	7	2.792	0.32	40	d	0.09502
ACVR1	14	activin A receptor, type I	203935_at	14	8.992	0.14	7	7.957	0.71	40	d	0
SNRK	14	SNF related kinase	209481_at	14	9.265	0.29	7	8.425	0.57	40	d	0.000566
			1564514_at	0	2.735	0.09	7	2.774	0.18	40	u	0.591422
			207474_at	0	4.171	0.53	7	3.719	0.56	40	d	0.059534
HNRNPA1	14	heterogeneous nuclear ribonucleoprotein A1	213356_x_at	14	13.59	0.13	7	13.28	0.3	40	d	0.011869
			200016_x_at	14	13.78	0.09	7	13.47	0.32	40	d	0.000016
			216559_x_at	0	9.151	0.28	7	8.939	0.4	40	d	0.194273
			222040_at	0	7.829	0.35	7	7.452	1.22	40	d	0.129005
			214280_x_at	0	10.58	0.26	7	10.01	0.84	40	d	0.002248
			217353_at	0	3.871	0.22	7	4.062	0.37	40	u	0.203641
			216497_at	0	3.708	0.4	7	3.866	0.56	40	u	0.487535
AFF1	14	AF4/FMR2 family, member 1	201924_at	14	10.37	0.27	7	9.274	0.91	40	d	0.000001
			211826_s_at	0	2.473	0.21	7	2.54	0.29	40	u	0.575328
			215451_s_at	0	3.569	0.46	7	3.64	0.67	40	u	0.793724
NRP2	14	neuropilin 2	225566_at	11	5.365	0.57	7	5.004	0.77	40	d	0.252578
			229225_at	4	3.546	0.51	7	4.338	1.03	40	u	0.057786
			228102_at	1	2.172	0.04	7	2.212	0.07	40	u	0.152405
			214632_at	1	4.862	0.54	7	5.34	1.1	40	u	0.275918
			228103_s_at	1	2.106	0.08	7	2.186	0.12	40	u	0.101126
			223510_at	0	2.653	0.18	7	2.866	0.62	40	u	0.09187
			211844_s_at	0	2.579	0.16	7	2.815	0.67	40	u	0.067886
			228699_at	0	2.348	0.1	7	2.559	0.37	40	u	0.005752
			210842_at	0	2.44	0.26	7	2.37	0.05	40	d	0.53549
			1555468_at	0	3.549	0.32	7	3.6	0.38	40	u	0.742643
			230410_at	0	2.454	0.05	7	2.593	0.18	40	u	0.00044
			210841_s_at	0	4.989	0.2	7	5.159	0.51	40	u	0.402221
C1orf21	14	chromosome 1 open reading frame 21	221272_s_at	14	6.958	0.61	7	4.588	0.84	40	d	0
			223125_s_at	14	9.762	0.42	7	8.441	1.37	40	d	0.000054
			223127_s_at	14	2.301	0.28	7	2.224	0.1	40	d	0.534705
			223126_s_at	14	8.291	0.38	7	5.948	1.48	40	d	0
			237098_at	0	2.512	0.17	7	2.54	0.24	40	u	0.766332
NR3C1	14	nuclear receptor subfamily 3, group C,	201865_x_at	14	10.06	0.56	7	8.714	1.04	40	d	0.001956
			201866_s_at	14	7.039	0.54	7	6.056	0.94	40	d	0.011532

[illegible]	[illegible]	member 1 (glucocorticoid receptor)	211671_s_at	9	9.217	0.59	7	7.707	1.23	40	d	0.003121
			216321_s_at	0	9.65	0.48	7	8.172	1.06	40	d	0.0009
RIMS3	14	regulating synaptic membrane exocytosis 3	204730_at	14	5.773	0.49	7	4.174	1.53	40	d	0.000023
			210991_s_at	1	2.524	0.14	7	2.734	0.32	40	u	0.105862
HMGA2	14	high mobility group AT-hook 2	208025_s_at	14	2.264	0.08	7	2.496	0.66	40	u	0.04225
			1561633_at	0	3.126	0.06	7	3.327	0.25	40	u	0.000163
			1568286_at	0	2.896	0.19	7	2.932	0.16	40	u	0.605615
			1567223_at	0	2.783	0.09	7	2.849	0.16	40	u	0.291987
			1568287_at	0	2.773	0.11	7	2.9	0.18	40	u	0.087384
			1558683_a_at	0	2.204	0.01	7	2.368	0.23	40	u	0.000093
			1559891_at	0	2.464	0.28	7	2.484	0.31	40	u	0.8731
			1558682_at	0	4.048	0.27	7	4.322	0.41	40	u	0.098977
			1567224_at	0	3.1	0.12	7	3.337	0.3	40	u	0.052096
CBL	14	Cas-Br-M (murine) ecotropic retroviral transforming sequence	225231_at	14	7.325	0.25	7	7.05	0.67	40	d	0.302634
			225234_at	14	6.5	0.29	7	6.289	0.5	40	d	0.296284
			243475_at	2	2.027	0.14	7	2.201	0.38	40	u	0.244642
			229010_at	2	4.291	0.3	7	5.316	0.98	40	u	0.000016
			206607_at	0	2.354	0.07	7	2.599	0.34	40	u	0.000268
STK35	14	serine/threonine kinase 35	225648_at	14	7.384	0.22	7	7.52	0.5	40	u	0.494314
			225649_s_at	14	8.608	0.25	7	8.774	0.85	40	u	0.337712
			232006_at	13	1.857	0.02	7	1.896	0.03	40	u	0.001991
			1553673_at	0	2.212	0.02	7	2.302	0.19	40	u	0.007101
NPTX1	14	neuronal pentraxin I	204684_at	12	6.51	0.27	7	6.217	0.24	40	d	0.006191
ELL2	14	elongation factor, RNA polymerase II, 2	226099_at	14	7.827	0.28	7	8.253	1.27	40	u	0.074498
			214446_at	1	5.371	0.57	7	5.788	1.47	40	u	0.471638
			214445_at	1	2.048	0.02	7	2.123	0.15	40	u	0.004852
			226982_at	0	8.204	0.59	7	6.593	1.36	40	d	0.004159
			240038_at	0	6.171	0.35	7	5.113	1.59	40	d	0.000799
METTL3	14	methyltransferase like 3	209265_s_at	1	9.56	0.29	7	8.541	0.64	40	d	0.0002
			213653_at	1	8.191	0.58	7	7.17	0.7	40	d	0.000811
			242111_at	0	5.847	0.81	7	5.13	0.98	40	d	0.081224
NRXN1	13	neurexin 1	209914_s_at	17	3.915	0.41	7	3.206	0.9	40	d	0.05172
			209915_s_at	17	2.483	0.37	7	2.626	0.59	40	u	0.548886
			216096_s_at	13	2.478	0.03	7	2.688	0.32	40	u	0.000298
			228547_at	0	3.653	1.19	7	3.025	1.13	40	d	0.195821
			1558708_at	0	2.9	0.05	7	3.047	0.18	40	u	0.000352
			237535_x_at	0	2.229	0.12	7	2.287	0.19	40	u	0.436998
WDFY3	13	WD repeat and FYVE domain containing 3	212598_at	13	5.185	0.35	7	4.794	0.58	40	d	0.098945
			212606_at	13	7.885	0.25	7	6.624	0.96	40	d	0
			212602_at	13	6.781	0.37	7	6.898	0.74	40	u	0.688786
			238660_at	0	5.497	0.52	7	5.75	0.74	40	u	0.398497
C3orf58	13	chromosome 3 open reading frame 58	226464_at	5	8.62	0.36	7	7.77	1.08	40	d	0.000899
			228079_at	0	3.865	0.57	7	3.916	0.93	40	u	0.89267
			235853_at	0	2.127	0.09	7	2.164	0.12	40	u	0.435192
CHIC1	13	cysteine-rich hydrophobic domain 1	1559481_at	13	3.137	0.21	7	3.248	0.31	40	u	0.37307
			228345_at	13	5.563	0.51	7	5.015	0.82	40	d	0.100099
			1557786_s_at	4	2.877	0.11	7	3.043	0.21	40	u	0.054497
C1orf144	13	chromosome 1 open reading frame 144	212003_at	13	6.059	0.6	7	6.54	1.03	40	u	0.247035
			212004_at	13	7.635	0.14	7	7.861	0.41	40	u	0.015109
BNC2	13	basonuclin 2	235723_at	13	2.572	0.12	7	2.755	0.45	40	u	0.044263
			220272_at	3	6.235	0.28	7	5.225	1.01	40	d	0.000014
			230722_at	0	4.409	0.39	7	4.142	0.82	40	d	0.412738
			238478_at	0	4.766	0.77	7	3.911	1.08	40	d	0.054932
			229942_at	0	4.719	0.38	7	4.876	0.86	40	u	0.646878
			243445_at	0	2.574	0.28	7	2.764	0.52	40	u	0.362839
CBFA2T3	13	core-binding factor, runt domain, alpha subunit 2; translocated to, 3	208056_s_at	13	4.03	0.51	7	4.095	0.97	40	u	0.86803
NUFIP2	13	nuclear fragile X mental retardation protein interacting protein 2	224956_at	13	6.972	0.33	7	8.322	0.75	40	u	0.000034
			224958_at	13	6.812	0.25	7	7.209	0.52	40	u	0.056438
ADD3	13	adducin 3 (gamma)	201752_s_at	10	11.04	0.63	7	9.401	1.01	40	d	0.000185
			205882_x_at	5	10.48	0.75	7	8.914	1.1	40	d	0.000875
			201753_s_at	5	10.46	0.57	7	8.342	1.04	40	d	0.000006
			201034_at	1	11.78	0.54	7	9.638	1.02	40	d	0.000003
		N-deacetylase/N-sulfotransferase (heparan	202607_at	13	8.3	0.44	7	7.48	0.6	40	d	0.001406

NDST1	13	N-deacetylase/N-sulfotransferase (heparan glucosaminyl) 1	202608_s_at	7	4.251	0.61	7	4.159	0.66	40	d	0.736955
			1554010_at	0	3.73	0.54	7	4.047	0.47	40	u	0.119895
STAG2	13	stromal antigen 2	209022_at	13	10.31	0.26	7	10.09	0.73	40	d	0.176643
			209023_s_at	1	9.071	0.32	7	9.418	0.74	40	u	0.237506
			207983_s_at	0	9.546	0.21	7	9.217	0.94	40	d	0.06571
HTR2C	13	5-hydroxytryptamine (serotonin) receptor 2C	207307_at	13	2.329	0.04	7	2.399	0.07	40	u	0.01001
			211479_s_at	2	2.446	0.03	7	2.527	0.15	40	u	0.003892
DYNC1LI2	13	dynein, cytoplasmic 1, light intermediate chain 2	224614_at	13	8.096	0.14	7	8.34	0.6	40	u	0.033526
			224616_at	13	11.21	0.28	7	10.81	0.56	40	d	0.079354
			203590_at	2	9.458	0.31	7	9.3	0.65	40	d	0.537447
			213162_at	0	2.05	0.1	7	2.196	0.37	40	u	0.05068
ITPR1	13	inositol 1,4,5-triphosphate receptor, type 1	203710_at	13	9.119	0.57	7	6.904	1.14	40	d	0.000013
			216944_s_at	11	6.736	0.86	7	5.035	0.87	40	d	0.000029
			211323_s_at	4	7.58	0.82	7	5.635	1.42	40	d	0.001281
			240052_at	0	5.81	0.82	7	4.291	0.91	40	d	0.000211
CCNT2	13	cyclin T2	204645_at	13	7.984	0.28	7	7.91	0.64	40	d	0.76922
			214638_s_at	5	4.884	0.35	7	5.434	0.73	40	u	0.064249
			213743_at	0	7.912	0.4	7	7.727	0.7	40	d	0.509725
MSL2L1	13	male-specific lethal 2-like 1 (Drosophila)	218733_at	10	8.515	0.23	7	7.804	0.59	40	d	0.003595
BRUNOL6	13	bruno-like 6, RNA binding protein (Drosophila)	227775_at	8	4.313	0.38	7	3.512	0.79	40	d	0.013946
PTP4A1	13	protein tyrosine phosphatase type IVA, member 1	200730_s_at	13	7.914	0.58	7	8.371	1.53	40	u	0.449425
			200731_s_at	13	8.802	0.55	7	8.584	0.97	40	d	0.574781
			200732_s_at	13	9.85	0.41	7	9.012	0.81	40	d	0.012357
			200733_s_at	12	8.296	0.3	7	9.175	0.7	40	u	0.002504
			240329_at	0	2.273	0.16	7	2.4	0.35	40	u	0.357454
ATP2B1	13	ATPase, Ca++ transporting, plasma membrane 1	212930_at	13	5.785	0.39	7	5.122	1.31	40	d	0.017631
			215716_s_at	6	7.925	0.42	7	7.221	1.26	40	d	0.013306
			209281_s_at	1	7.99	0.36	7	7.514	1.26	40	d	0.066717
FNBP1L	13	formin binding protein 1-like	215017_s_at	13	9.324	0.29	7	9.351	0.72	40	u	0.925027
SYNGAP1	13	synaptic Ras GTPase activating protein 1 homolog (rat)	230297_x_at	10	4.888	0.29	7	5.143	0.64	40	u	0.314472
			234285_at	0	2.12	0.06	7	2.208	0.18	40	u	0.025859
SFRS1	13	splicing factor, arginine/serine-rich 1 (splicing factor 2, alternate splicing factor)	201741_x_at	13	3.938	0.3	7	4.583	0.69	40	u	0.022454
			211784_s_at	13	11.51	0.12	7	11.68	0.37	40	u	0.038547
			208863_s_at	12	9.742	0.14	7	9.72	0.4	40	d	0.802764
			201742_x_at	7	9.939	0.18	7	9.945	1.38	40	u	0.979984
			227164_at	0	7.609	0.2	7	7.994	0.45	40	u	0.035286
TAF5	13	TAF5 RNA polymerase II, TATA box binding protein (TBP)-associated factor, 100kDa	210053_at	13	5.809	0.34	7	6.429	1.05	40	u	0.008669
			1553528_a_at	10	4.759	0.24	7	5.405	0.98	40	u	0.001288
MEIS2	13	Meis homeobox 2	207480_s_at	12	7.751	0.32	7	5.047	1.42	40	d	0
C2orf31	13	chromosome 2 open reading frame 31	no probeset available									
PRDM1	13	PR domain containing 1, with ZNF domain	228964_at	13	5.73	0.7	7	6.037	1.17	40	u	0.512827
			235668_at	10	6.074	0.27	7	6.263	0.57	40	u	0.405348
			217192_s_at	0	5.524	0.37	7	5.749	0.62	40	u	0.368408
IKZF4	13	IKAROS family zinc finger 4 (Eos)	226759_at	16	4.829	0.6	7	4.454	0.72	40	d	0.206246
			226761_at	16	5.825	0.34	7	5.238	0.74	40	d	0.050406
			229752_at	7	4.519	0.47	7	4.356	0.43	40	d	0.379251
			208472_at	0	5.912	0.08	7	5.788	0.31	40	d	0.044019
PDE4B	13	phosphodiesterase 4B, cAMP-specific (phosphodiesterase E4 dunce homolog, Drosophila)	203708_at	8	9.9	0.75	7	8.652	1.81	40	d	0.084815
			211302_s_at	2	8.285	0.9	7	7.026	2.29	40	d	0.166698
			215671_at	0	4.426	0.48	7	4.471	0.72	40	u	0.874815
SGCD	13	sarcoglycan, delta (35kDa dystrophin-associated glycoprotein)	230730_at	13	5.136	0.78	7	4.745	0.81	40	d	0.253568
			228602_at	11	4.374	0.88	7	5.011	0.75	40	u	0.056149
			214492_at	0	3.62	0.31	7	3.491	0.45	40	d	0.485123
			210330_at	0	3.017	0.15	7	3.37	0.4	40	u	0.028236
			210329_s_at	0	4.922	0.37	7	4.843	0.45	40	d	0.667591
SOBP	13	sine oculis binding protein homolog (Drosophila)	229034_at	13	2.555	0.2	7	2.429	0.14	40	d	0.05367
			218974_at	10	6.909	0.59	7	4.591	1.31	40	d	0.000048
			229646_at	1	1.881	0.12	7	1.878	0.04	40	d	0.95321
			234098_at	0	3.322	0.33	7	3.024	0.2	40	d	0.002845
			1563906_at	0	2.438	0.44	7	2.389	0.55	40	d	0.828585
			233766_at	0	2.427	0.07	7	2.551	0.18	40	u	0.081363
			244279_at	0	2.543	0.28	7	2.445	0.24	40	d	0.353204
			1569351_at	0	2.071	0.03	7	2.191	0.22	40	u	0.002008

SSH2	13	slingshot homolog 2 (Drosophila)	226080_at	13	5.788	0.45	7	5.959	0.76	40	u	0.572379
			1555423_at	0	3.997	0.22	7	3.935	0.2	40	d	0.469476
			1555425_x_at	0	2.652	0.03	7	2.718	0.1	40	u	0.003531
			1554114_s_at	0	2.905	0.16	7	3.136	0.31	40	u	0.066719
TMEPAI	13	transmembrane, prostate androgen induced RNA	222450_at	13	8.234	0.53	7	8.683	1.08	40	u	0.298723
			222449_at	13	7.38	0.49	7	8.688	0.97	40	u	0.001372
			217875_s_at	2	6.74	0.47	7	7.854	0.89	40	u	0.0028
			237166_at	0	2.728	0.29	7	2.991	0.79	40	u	0.400359
NAT13	13	N-acetyltransferase 13	217745_s_at	13	9.517	0.27	7	10.08	0.61	40	u	0.023885
			222393_s_at	13	8.118	0.31	7	8.892	1.3	40	u	0.002913
LAMC1	13	laminin, gamma 1 (formerly LAMB2)	200771_at	13	10.41	0.37	7	8.908	0.99	40	d	0.000337
			200770_s_at	2	8.551	0.37	7	8.204	0.93	40	d	0.345966
SEMA6A	13	sema domain, transmembrane domain (TM), and cytoplasmic domain, (semaphorin) 6A	223449_at	13	7.007	0.49	7	4.533	1.73	40	d	0
			225660_at	5	7.569	0.25	7	5.682	1.99	40	d	0.000001
			220454_s_at	1	5.525	0.77	7	4.532	1.7	40	d	0.144907
			215028_at	0	6.083	0.56	7	4.303	1.68	40	d	0.000031
			1558338_at	0	2.304	0.26	7	2.322	0.27	40	u	0.871927
MYLIP	13	myosin regulatory light chain interacting protein	223130_s_at	11	8.994	0.28	7	7.709	1.12	40	d	0.000001
			223129_x_at	11	9.321	0.33	7	8.102	0.75	40	d	0.00016
			228097_at	0	4.942	0.56	7	3.738	0.73	40	d	0.000199
			228098_s_at	0	10.16	0.27	7	8.959	0.98	40	d	0
			227707_at	0	2.452	0.14	7	2.467	0.14	40	u	0.8006
			220319_s_at	0	8.549	0.25	7	7.218	0.97	40	d	0
ZFP91	13	zinc finger protein 91 homolog (mouse)	224631_at	13	6.36	0.26	7	7.56	0.93	40	u	0
			224636_at	13	9.238	0.21	7	8.898	0.71	40	d	0.022897
PCDH8	13	protocadherin 8	206935_at	13	2.613	0.03	7	3.038	1.01	40	u	0.012286
SRGAP3	13	SLIT-ROBO Rho GTPase activating protein 3	209794_at	13	3.61	0.72	7	2.999	0.63	40	d	0.028454
			215550_at	0	2.217	0.02	7	2.348	0.17	40	u	0.000052
			1563595_at	0	2.611	0.07	7	2.727	0.24	40	u	0.025398
			232869_at	0	3.547	0.28	7	3.501	0.33	40	d	0.732178
RPS6KA3	13	ribosomal protein S6 kinase, 90kDa, polypeptide 3	226335_at	13	7.186	0.35	7	6.696	0.89	40	d	0.168475
			203843_at	4	7.097	0.4	7	6.974	0.94	40	d	0.73753
EP300	12	E1A binding protein p300	213579_s_at	12	7.266	0.35	7	7.258	0.74	40	d	0.979429
			202221_s_at	12	6.979	0.62	7	6.687	0.64	40	d	0.281657
SUV420H1	12	suppressor of variegation 4-20 homolog 1 (Drosophila)	222566_at	12	7.417	0.38	7	6.875	0.97	40	d	0.161529
			218242_s_at	3	9.543	0.31	7	8.998	0.81	40	d	0.092368
			222759_at	0	7.964	0.47	7	7.624	0.96	40	d	0.37467
			242646_at	0	8.816	0.49	7	8.135	1.01	40	d	0.09546
OSBP	12	oxysterol binding protein	201799_s_at	12	7.876	0.35	7	7.487	0.8	40	d	0.219196
			201800_s_at	12	8.664	0.24	7	7.996	0.63	40	d	0.008935
			1563051_at	0	4.974	0.65	7	4.315	0.78	40	d	0.044409
ADAMTS6	12	ADAM metallopeptidase with thrombospondin type 1 motif, 6	237411_at	12	3.634	0.6	7	4.467	0.83	40	u	0.016171
			220866_at	0	2.464	0.1	7	2.661	0.31	40	u	0.0048
			1570351_at	0	4.006	0.43	7	4.525	0.64	40	u	0.050346
ZDHHC17	12	zinc finger, DHHC-type containing 17	212982_at	12	9.18	0.26	7	8.365	0.87	40	d	0.000062
			217486_s_at	0	4.11	0.55	7	4.295	0.75	40	u	0.543917
CBX4	12	chromobox homolog 4 (Pc class homolog, Drosophila)	227558_at	12	9.056	0.21	7	9.212	0.88	40	u	0.346976
			206724_at	0	5.996	0.54	7	5.954	1.39	40	d	0.938544
PAK7	12	p21(CDKN1A)-activated kinase 7	213990_s_at	12	2.565	0.45	7	2.062	0.17	40	d	0.035221
			210721_s_at	12	3.265	0.68	7	2.958	0.21	40	d	0.313137
FAM134C	12	family with sequence similarity 134, member C	212697_at	12	9.948	0.28	7	9.477	0.66	40	d	0.07381
ACSL1	12	acyl-CoA synthetase long-chain family member 1	201963_at	12	9.645	1.34	7	8.789	1.29	40	d	0.122007
			207275_s_at	8	9.201	1.46	7	7.967	1.5	40	d	0.054752
EN2	12	engrailed homeobox 2	207060_at	12	2.32	0.04	7	2.53	0.41	40	u	0.003237
BICD2	12	bicaudal D homolog 2 (Drosophila)	212702_s_at	12	6.95	0.26	7	6.999	0.65	40	u	0.84629
			213154_s_at	12	8.129	0.13	7	8.22	0.53	40	u	0.371648
			209203_s_at	6	5.103	0.33	7	5.484	0.98	40	u	0.076106
			1553021_s_at	1	2.209	0.08	7	2.465	0.56	40	u	0.010327
PTGFRN	12	prostaglandin F2 receptor negative regulator	224937_at	12	8.131	0.31	7	7.685	0.89	40	d	0.028162
			224950_at	12	7.145	0.38	7	7.391	0.95	40	u	0.510655
SAPS3	12	SAPS domain family, member 3	222467_s_at	12	9.252	0.21	7	9.307	0.61	40	u	0.672076
			217928_s_at	12	8.97	0.15	7	8.461	0.65	40	d	0.000151
			232312_at	0	6.719	0.45	7	5.377	1.09	40	d	0.002894

RASA1	12	RAS p21 protein activator (GTPase activating protein) 1	210621_s_at	12	8.629	0.4	7	7.719	1.17	40	d	0.001134
			202677_at	12	9.768	0.33	7	8.789	0.96	40	d	0.000066
DMD	12	dystrophin (muscular dystrophy, Duchenne and Becker types)	203881_s_at	12	8.367	0.38	7	5.929	1.94	40	d	0
			208086_s_at	2	3.735	0.43	7	3.527	1.08	40	d	0.623471
			234752_x_at	0	3.177	0.16	7	3.512	0.37	40	u	0.026392
			207660_at	0	2.828	0.73	7	2.672	0.74	40	d	0.615464
SOCS5	12	suppressor of cytokine signaling 5	209647_s_at	12	8.272	0.65	7	8.056	0.85	40	d	0.532425
			209648_x_at	12	8.962	0.51	7	8.401	0.74	40	d	0.064517
			208127_s_at	10	7.876	0.55	7	7.318	0.76	40	d	0.073778
SLC4A4	12	solute carrier family 4, sodium bicarbonate cotransporter, member 4	203908_at	12	3.353	0.54	7	3.648	1.11	40	u	0.504126
			210738_s_at	10	2.944	0.15	7	3.205	0.48	40	u	0.01265
			210739_x_at	7	3.686	0.36	7	4.224	0.73	40	u	0.067129
			211494_s_at	4	2.119	0.12	7	2.533	0.64	40	u	0.000705
			1554027_a_at	0	2.639	0.13	7	2.867	0.34	40	u	0.093642
ZBTB46	12	zinc finger and BTB domain containing 46	227329_at	4	6.281	0.52	7	5.717	0.96	40	d	0.144321
			227358_at	4	4.13	0.63	7	3.925	0.87	40	d	0.561245
			243214_at	0	2.337	0.26	7	2.342	0.2	40	u	0.951109
MYB	12	v-myb myeloblastosis viral oncogene homolog (avian)	204798_at	12	9.726	0.71	7	8.337	1.89	40	d	0.067022
			215152_at	0	2.869	0.25	7	3.044	0.43	40	u	0.313591
ZNF650	12	zinc finger protein 650	230029_x_at	12	9.001	0.2	7	8.792	0.56	40	d	0.344275
			234982_at	10	7.719	0.28	7	7.49	0.64	40	d	0.364169
			244121_at	2	2.719	0.07	7	2.901	0.2	40	u	0.00029
			235265_at	0	2.647	0.21	7	2.579	0.25	40	d	0.50418
PCTK2	12	PCTAIRE protein kinase 2	221918_at	12	9.12	0.27	7	8.965	0.81	40	d	0.369451
			206474_at	0	4.627	0.7	7	5.156	1.06	40	u	0.220838
RBM24	12	RNA binding motif protein 24	235004_at	12	3.918	0.99	7	3.884	1.75	40	d	0.960519
PRPF4B	12	PRP4 pre-mRNA processing factor 4 homolog B (yeast)	202127_at	12	8.233	0.27	7	8.068	0.72	40	d	0.561219
			202126_at	12	8.704	0.18	7	8.619	0.57	40	d	0.472908
			211090_s_at	0	7.515	0.63	7	7.526	1.92	40	u	0.978239
VLDLR	12	very low density lipoprotein receptor	209822_s_at	11	6.81	0.64	7	5.612	1.82	40	d	0.005295
SLC4A7	12	solute carrier family 4, sodium bicarbonate cotransporter, member 7	209884_s_at	12	9.441	0.57	7	8.047	0.79	40	d	0.000073
			210286_s_at	2	4.517	0.77	7	4.466	1	40	d	0.900518
			207603_at	0	2.938	0.16	7	3.027	0.37	40	u	0.544311
			207604_s_at	0	6.664	0.61	7	5.89	0.82	40	d	0.023718
PRKCE	12	protein kinase C, epsilon	226101_at	12	4.655	0.56	7	4.112	0.68	40	d	0.058178
			239011_at	0	4.664	0.32	7	4.55	0.56	40	d	0.615342
			206248_at	0	2.232	0.14	7	2.308	0.17	40	u	0.264169
			236459_at	0	2.481	0.14	7	2.604	0.29	40	u	0.291581
BMPR2	12	bone morphogenetic protein receptor, type II (serine/threonine kinase)	225144_at	12	8.969	0.21	7	8.499	0.82	40	d	0.004806
			238516_at	8	4.125	0.53	7	4.266	0.91	40	u	0.697898
			231873_at	8	7.832	0.28	7	7.74	0.7	40	d	0.740316
			210214_s_at	0	4.638	0.56	7	5.716	0.49	40	u	0.000006
			209920_at	0	6.597	0.42	7	6.968	0.67	40	u	0.170999
DAB2IP	12	DAB2 interacting protein	225020_at	12	8.783	0.28	7	7.047	0.72	40	d	0
			228942_s_at	0	3.787	0.37	7	3.622	0.62	40	d	0.506825
			235966_at	0	5.958	0.18	7	6.06	0.3	40	u	0.39204
DDX6	12	DEAD (Asp-Glu-Ala-Asp) box polypeptide 6	204909_at	0	3.654	0.18	7	4.24	0.51	40	u	0.000017
ZDHHC5	12	zinc finger, DHHC-type containing 5	224868_at	12	7.283	0.37	7	7.31	0.99	40	u	0.942978
			224858_at	12	9.081	0.31	7	9.118	0.65	40	u	0.886115
CIT	12	citron (rho-interacting, serine/threonine kinase 21)	212801_at	12	4.86	0.49	7	6.44	0.71	40	u	0.000001
			242872_at	0	2.429	0.04	7	2.537	0.15	40	u	0.000687
ROD1	12	ROD1 regulator of differentiation 1 (S. pombe)	224618_at	12	6.494	0.64	7	8.421	0.73	40	u	0
			224617_at	12	10.8	0.25	7	11.06	0.46	40	u	0.16223
			214697_s_at	3	5.086	0.68	7	6.999	1.25	40	u	0.000355
			214698_at	3	6.291	0.32	7	6.994	0.59	40	u	0.004245
			207223_s_at	0	3.753	0.55	7	5.276	1.26	40	u	0.003575
KLHL3	12	kelch-like 3 (Drosophila)	221221_s_at	12	6.652	0.55	7	5.085	1.23	40	d	0.002256
			1555110_a_at	0	3.068	0.1	7	3.208	0.2	40	u	0.07944
RNF44	12	ring finger protein 44	203286_at	12	8.026	0.31	7	7.543	0.84	40	d	0.149417
H3F3B	12	H3 histone, family 3B (H3.3B)	211999_at	12	13.25	0.22	7	12.63	0.65	40	d	0.00011
			211997_x_at	12	13.62	0.27	7	13.02	0.77	40	d	0.001352
			211998_at	12	10.37	0.59	7	7.688	1.06	40	d	0
			209069_s_at	4	14.09	0.25	7	13.62	0.7	40	d	0.005161

NEBL	12	nebulette	203962_s_at	12	9.698	0.6	7	8.796	1.31	40	d	0.085754
			203961_at	12	10.13	0.44	7	8.679	1.32	40	d	0.000017
			207279_s_at	3	4.233	0.7	7	4.83	1.05	40	u	0.164912
			216882_s_at	0	6.438	0.22	7	6.551	0.3	40	u	0.350362
			217585_at	0	4.236	0.41	7	4.116	0.49	40	d	0.55463
			241782_at	0	5.663	0.49	7	5.028	0.64	40	d	0.018223
FRYL	12	FRY-like	212548_s_at	12	8.05	0.28	7	7.61	0.91	40	d	0.025658
			212546_s_at	12	7.878	0.22	7	7.672	0.86	40	d	0.221138
			1554260_a_at	1	6.538	0.32	7	7.061	0.92	40	u	0.01461
			1562650_at	0	3.296	0.33	7	3.511	0.4	40	u	0.194202
			1562625_at	0	2.4	0.14	7	2.65	0.39	40	u	0.005842
			1563687_a_at	0	3.198	0.44	7	3.957	1.17	40	u	0.1036
PPP1R12A	12	protein phosphatase 1, regulatory (inhibitor) subunit 12A	201603_at	12	9.59	0.31	7	8.955	0.74	40	d	0.033555
			201602_s_at	12	7.108	0.46	7	7.045	0.71	40	d	0.825265
			201604_s_at	12	8.051	0.19	7	7.414	0.89	40	d	0.000286
PRRX1	12	paired related homeobox 1	226695_at	12	9.637	0.6	7	8.79	1.15	40	d	0.069479
			205991_s_at	8	6.565	0.78	7	6.026	1.54	40	d	0.380316
SCN1A	12	sodium channel, voltage-gated, type I, alpha subunit	210383_at	12	2.702	0.19	7	3.262	1.09	40	u	0.005613
			1555246_a_at	0	2.036	0.02	7	2.289	0.89	40	u	0.085796
CCDC6	12	coiled-coil domain containing 6	225010_at	12	10.21	0.29	7	10.04	0.79	40	d	0.335225
			204716_at	4	6.042	0.7	7	6.926	1.08	40	u	0.046779
NAV2	12	neuron navigator 2	222598_s_at	12	3.768	0.84	7	3.165	0.65	40	d	0.041016
			218330_s_at	12	9.012	0.66	7	6.897	1.16	40	d	0.000035
			222599_s_at	12	7.259	0.62	7	5.87	0.94	40	d	0.000632
			239141_at	0	3.813	0.43	7	4.004	0.44	40	u	0.300625
			1567358_at	0	2.865	0.08	7	2.94	0.13	40	u	0.150871
			1567357_at	0	2.562	0.03	7	2.613	0.06	40	u	0.055081
			1556606_at	0	2.43	0.15	7	2.419	0.28	40	d	0.914266
FAM78A	12	family with sequence similarity 78, member A	227002_at	12	6.251	0.57	7	6.013	0.54	40	d	0.300508
ESR1	12	estrogen receptor 1	205225_at	12	10.06	1.44	7	6.717	3.74	40	d	0.027092
			211233_x_at	0	5.251	0.62	7	5.066	1.45	40	d	0.746731
			215552_s_at	0	4.737	0.96	7	4.482	1.98	40	d	0.746006
			217190_x_at	0	6.509	0.5	7	6.153	0.91	40	d	0.328539
			211627_x_at	0	4.783	0.61	7	4.882	0.58	40	u	0.687406
			217163_at	0	2.148	0.01	7	2.185	0.04	40	u	0.014257
			211235_s_at	0	4.53	1	7	4.182	2.14	40	d	0.680659
			211234_x_at	0	5.231	0.61	7	4.943	1.17	40	d	0.538294
ARHGAP12	12	Rho GTPase activating protein 12	207606_s_at	12	8.588	0.43	7	7.916	0.76	40	d	0.03101
PDE7B	12	phosphodiesterase 7B	230109_at	12	3.873	1.17	7	2.438	0.28	40	d	0.024477
			243438_at	12	4.127	0.67	7	4.015	0.46	40	d	0.589975
			220343_at	1	2.252	0.13	7	2.232	0.1	40	d	0.644881
G3BP2	12	GTPase activating protein (SH3 domain) binding protein 2	208840_s_at	12	8.328	0.35	7	8.512	1.08	40	u	0.421033
			208841_s_at	12	9.476	0.23	7	9.81	0.52	40	u	0.111244
			206383_s_at	2	5.91	0.86	7	6.196	1.35	40	u	0.599732
CCNJ	12	cyclin J	222888_at	12	3.693	0.36	7	3.381	0.37	40	d	0.047989
			229091_s_at	12	6.057	0.39	7	6.41	0.94	40	u	0.34311
			219470_x_at	12	6.147	0.28	7	6.234	0.97	40	u	0.65365
MTMR4	12	myotubularin related protein 4	212277_at	12	7.574	0.23	7	7.764	0.89	40	u	0.272159
			214268_s_at	12	8.11	0.42	7	8.317	0.8	40	u	0.518852
AMMECR1L	12	AMME chromosomal region gene 1-like	223297_at	12	8.459	0.24	7	8.334	0.54	40	d	0.559515
MTDH	12	metadherin	212251_at	12	9.857	0.17	7	11.32	0.78	40	u	0
			212248_at	12	8.965	0.2	7	9.506	0.87	40	u	0.001695
			212250_at	12	8.879	0.17	7	10.21	0.77	40	u	0
CSNK1G1	12	casein kinase 1, gamma 1	231920_s_at	12	6.497	0.47	7	7.249	0.48	40	u	0.000505
			226888_at	4	7.567	0.36	7	7.517	0.56	40	d	0.827317
			220640_at	1	4.727	0.33	7	4.794	0.47	40	u	0.730167
			221673_s_at	1	4.108	0.22	7	4.485	0.76	40	u	0.017902
NAV3	12	neuron navigator 3	1552658_a_at	12	4.191	0.87	7	2.889	0.37	40	d	0.01125
			204823_at	9	6.236	0.74	7	3.831	1.15	40	d	0.000004
			216466_at	0	3.278	0.08	7	3.44	0.29	40	u	0.008091
			216632_at	0	2.423	0.06	7	2.501	0.16	40	u	0.034546
			1562234_a_at	0	2.492	0.22	7	2.578	0.43	40	u	0.615012
			209379_s_at	12	8.264	0.31	7	8.037	0.67	40	d	0.390396

KIAA1128	12	KIAA1128	209378_s_at	12	6.335	0.34	7	6.73	0.65	40	u	0.129191
			1554131_at	0	3.339	0.17	7	3.728	0.41	40	u	0.018526
			1554132_a_at	0	4.697	0.4	7	5.862	0.84	40	u	0.000989
LPHN2	12	latrophilin 2	206953_s_at	11	6.478	0.53	7	4.917	1.18	40	d	0.001511
PLEKHA6	12	pleckstrin homology domain containing, family A member 6	229245_at	9	7.064	0.43	7	5.446	1.48	40	d	0.000006
			205093_at	5	6.097	0.65	7	5.819	1.15	40	d	0.545084
ACSL4	12	acyl-CoA synthetase long-chain family member 4	202422_s_at	12	4.644	0.88	7	4.653	0.95	40	u	0.981739
			1557419_a_at	0	4.926	0.68	7	4.575	0.54	40	d	0.141553
			1557418_at	0	5.785	0.7	7	4.087	0.74	40	d	0.000002
MEF2D	12	myocyte enhancer factor 2D	225641_at	12	7.186	0.24	7	6.104	0.78	40	d	0
			203003_at	8	6.545	0.25	7	6.218	0.73	40	d	0.047621
			203004_s_at	2	5.809	0.41	7	5.905	0.68	40	u	0.723675
SENP1	12	SUMO1/sentrin specific peptidase 1	226619_at	12	7.138	0.29	7	7.059	0.44	40	d	0.658523
			1552812_a_at	0	3.747	0.61	7	4.998	1.08	40	u	0.005527
DACH1	12	dachshund homolog 1 (Drosophila)	228915_at	12	6.285	1.22	7	4.329	2	40	d	0.018236
			205471_s_at	3	6.231	0.71	7	4.58	1.63	40	d	0.013345
			205472_s_at	3	4.309	0.9	7	4.24	1.21	40	d	0.889419
ARNT	12	aryl hydrocarbon receptor nuclear translocator	218221_at	12	7.461	0.14	7	7.247	0.58	40	d	0.056741
			230619_at	12	6.519	0.57	7	6.039	0.79	40	d	0.141576
			231016_s_at	12	3.579	0.2	7	4.135	0.98	40	u	0.003106
			233724_at	0	4.47	0.51	7	4.379	0.44	40	d	0.634852
			218222_x_at	0	5.407	0.69	7	5.587	1.03	40	u	0.665415
			210828_s_at	0	3.281	0.22	7	3.694	0.94	40	u	0.023172
CADM2	12	cell adhesion molecule 2	1552752_a_at	12	2.905	0.27	7	3.232	0.52	40	u	0.122367
			1552754_a_at	12	2.87	0.28	7	3.196	0.79	40	u	0.06615
			1555544_a_at	0	2.507	0.03	7	2.737	0.38	40	u	0.000666
SH3KBP1	12	SH3-domain kinase binding protein 1	223082_at	12	8.005	0.52	7	8.349	1.12	40	u	0.437271
			1554168_a_at	9	7.203	0.52	7	7.703	1.27	40	u	0.321336
CD2AP	12	CD2-associated protein	203593_at	12	8.325	0.11	7	8.987	0.63	40	u	0
			236257_at	0	4.688	0.31	7	4.647	0.43	40	d	0.809733
OGT	12	O-linked N-acetylglucosamine (GlcNAc) transferase (UDP-N-acetylglucosamine:polypeptide-N-acetylglucosaminyl transferase)	209240_at	12	11.37	0.24	7	10.13	0.67	40	d	0
			229787_s_at	11	4.956	0.48	7	4.75	1.04	40	d	0.618176
			207564_x_at	8	9.408	0.35	7	8.334	0.89	40	d	0.003363
			212307_s_at	8	8.948	0.38	7	7.949	0.75	40	d	0.001502
			220594_at	0	2.864	0.21	7	3.162	0.44	40	u	0.095814
			207563_s_at	0	8.934	0.31	7	7.886	1.02	40	d	0.000022
NFASC	12	neurofascin homolog (chicken)	213438_at	12	6.288	0.75	7	4.484	1.83	40	d	0.015672
			230242_at	5	4.299	0.34	7	4.592	0.81	40	u	0.360046
			243645_at	3	2.234	0.02	7	2.426	0.48	40	u	0.017977
			214799_at	0	4.557	0.46	7	4.39	0.75	40	d	0.581043
RALA	12	v-ral simian leukemia viral oncogene homolog A (ras related)	224880_at	12	10.03	0.2	7	10.29	0.58	40	u	0.049565
			214435_x_at	0	7.381	0.29	7	9.133	0.79	40	u	0
ZMYND11	12	zinc finger, MYND domain containing 11	202136_at	12	11.64	0.2	7	11.44	0.59	40	d	0.112754
			202137_s_at	6	6.617	0.68	7	6.724	1.35	40	u	0.841207
			1554159_a_at	0	5.684	0.64	7	6.261	1.22	40	u	0.237541
			1554158_at	0	3.273	0.09	7	3.604	0.44	40	u	0.000145
MTSS1	12	metastasis suppressor 1	203037_s_at	12	8.938	0.48	7	9.402	1.38	40	u	0.128438
			203036_s_at	12	4.338	0.52	7	5.084	0.99	40	u	0.062978
			210360_s_at	0	2.84	0.18	7	3.384	1.11	40	u	0.007026
			229780_at	0	2.456	0.07	7	2.492	0.07	40	u	0.218318
			210359_at	0	2.394	0.07	7	2.907	0.91	40	u	0.001359
NPTN	12	neuroplastin	202228_s_at	2	11.28	0.14	7	10.79	0.71	40	d	0.000335
WAPAL	12	wings apart-like homolog (Drosophila)	212264_s_at	12	7.895	0.4	7	8.278	0.74	40	u	0.199925
			212267_at	12	8.76	0.39	7	8.332	0.69	40	d	0.122536
			217218_at	3	2.352	0.08	7	2.393	0.12	40	u	0.4135
			1554441_a_at	0	7.825	0.4	7	8.05	0.92	40	u	0.535122
SLMAP	11	sarcolemma associated protein	222924_at	11	4.54	0.61	7	4.619	0.73	40	u	0.789415
			225243_s_at	11	9.22	0.23	7	8.683	0.73	40	d	0.001355
			224149_x_at	0	6.483	0.62	7	5.94	1.28	40	d	0.286796
EFNB1	11	ephrin-B1	202711_at	4	5.33	0.37	7	4.882	0.54	40	d	0.045251
ST8SIA4	11	ST8 alpha-N-acetyl-neuraminide alpha-2,8-sialyltransferase 4	230836_at	11	5.075	0.75	7	5.932	0.85	40	u	0.017936
			230261_at	3	3.74	0.55	7	4.282	0.81	40	u	0.101084
			242943_at	3	3.611	0.31	7	4.657	0.99	40	u	0.000018
			206925_at	1	2.747	0.28	7	3.485	1.08	40	u	0.001135

			1554460_at	0	3.082	0.08	7	3.369	0.44	40	u	0.000515
MED14	11	mediator complex subunit 14	202611_s_at	11	6.518	0.54	7	6.399	0.78	40	d	0.704298
			202612_s_at	11	2.306	0.03	7	2.419	0.18	40	u	0.000943
			202610_s_at	10	7.571	0.32	7	8.022	0.48	40	u	0.023816
			217120_s_at	10	2.065	0.05	7	2.256	0.27	40	u	0.000306
			215167_at	0	3.571	0.33	7	4.051	0.71	40	u	0.092181
XPO4	11	exportin 4	222649_at	11	6.504	0.28	7	6.372	0.64	40	d	0.601881
			218479_s_at	0	3.107	0.51	7	3.464	0.95	40	u	0.346236
FOXN2	11	forkhead box N2	226711_at	11	7.665	0.35	7	8.071	1	40	u	0.069203
			206708_at	1	2.351	0.1	7	2.795	0.5	40	u	0.000013
ARID1B	11	AT rich interactive domain 1B (SWI1-like)	225184_at	9	7.473	0.29	7	6.671	0.67	40	d	0.003622
			225181_at	9	8.387	0.52	7	7.986	0.79	40	d	0.208311
			1566990_x_at	0	6.382	0.53	7	6.24	0.64	40	d	0.588277
			1566991_at	0	2.244	0.23	7	2.187	0.18	40	d	0.468355
			1566989_at	0	5.599	0.37	7	5.596	0.64	40	d	0.988002
			233339_s_at	0	2.303	0.16	7	2.505	0.35	40	u	0.147761
E2F7	11	E2F transcription factor 7	228033_at	11	2.73	0.28	7	4.859	1.55	40	u	0
			241725_at	0	3.281	0.19	7	3.709	0.59	40	u	0.001728
AMOTL2	11	angiomotin like 2	203002_at	11	9.605	0.44	7	7.746	1.1	40	d	0.000087
FOXO1	11	forkhead box O1	202723_s_at	5	9.216	0.43	7	7.312	0.67	40	d	0
			202724_s_at	5	7.905	0.36	7	6.241	0.91	40	d	0.000028
			228484_s_at	0	2.104	0.06	7	2.148	0.11	40	u	0.313943
CCDC117	11	coiled-coil domain containing 117	225644_at	11	8.353	0.38	7	8.764	1.01	40	u	0.304642
			235330_at	0	3.396	0.67	7	4.186	1.37	40	u	0.151615
TACC1	11	transforming, acidic coiled-coil containing protein 1	200911_s_at	11	10.48	0.32	7	8.69	1.02	40	d	0
			217433_at	7	2.201	0.07	7	2.325	0.21	40	u	0.011982
			217437_s_at	7	8.56	0.41	7	7.187	1.13	40	d	0.000011
			1554690_a_at	3	6.418	0.66	7	6.06	1.38	40	d	0.514507
			242290_at	0	5.246	0.62	7	4.82	0.97	40	d	0.276832
			1557305_at	0	3.426	0.09	7	3.662	0.56	40	u	0.019578
APLN	11	apelin, AGTRL1 ligand	222856_at	11	4.902	0.47	7	4.75	0.95	40	d	0.687837
			244166_at	0	3.827	0.61	7	3.77	0.48	40	d	0.78804
CACNB2	11	calcium channel, voltage-dependent, beta 2 subunit	213714_at	11	2.362	0.33	7	2.635	0.96	40	u	0.191268
			207776_s_at	5	3.729	0.54	7	3.728	0.68	40	d	0.998759
			1559420_x_at	0	3.323	0.36	7	3.195	0.44	40	d	0.483886
			1559419_at	0	3.598	0.28	7	3.257	0.92	40	d	0.080304
			215365_at	0	3.034	0.03	7	3.222	0.26	40	u	0.000109
			1555098_a_at	0	2.236	0.04	7	2.308	0.1	40	u	0.083544
MED12L	11	mediator complex subunit 12-like	232114_at	11	2.694	0.54	7	2.725	0.54	40	u	0.893984
			1555300_a_at	0	2.453	0.09	7	2.575	0.23	40	u	0.173663
SHANK2	11	SH3 and multiple ankyrin repeat domains 2	243681_at	11	5.544	0.81	7	4.526	1.35	40	d	0.063978
			213307_at	8	6.535	0.79	7	5.024	1.8	40	d	0.037504
			213308_at	8	7.371	0.61	7	6.775	1.46	40	d	0.302093
			1564377_at	0	2.727	0.11	7	3.022	0.45	40	u	0.001455
			215829_at	0	2.931	0.1	7	2.988	0.14	40	u	0.328815
			215830_at	0	2.604	0.29	7	2.475	0.08	40	d	0.324102
SLC39A10	11	solute carrier family 39 (zinc transporter), member 10	225295_at	11	8.415	0.27	7	8.653	0.66	40	u	0.364663
HOXA11	11	homeobox A11	213823_at	11	2.252	0.05	7	2.483	0.66	40	u	0.037526
			208493_at	0	2.168	0.01	7	2.292	0.32	40	u	0.02153
C10orf6	11	chromosome 10 open reading frame 6	203481_at	11	6.508	0.44	7	6.629	0.76	40	u	0.689124
			203482_at	11	7.47	0.37	7	6.656	0.81	40	d	0.01376
			1553909_x_at	0	3.767	0.58	7	4.189	0.68	40	u	0.137512
			235590_at	0	3.05	0.3	7	3.165	0.65	40	u	0.653455
ARID1A	11	AT rich interactive domain 1A (SWI-like)	218917_s_at	11	9.089	0.5	7	8.343	0.95	40	d	0.053259
			212152_x_at	2	9.361	0.54	7	8.534	0.88	40	d	0.022761
			210649_s_at	1	8.973	0.48	7	8.172	0.96	40	d	0.03955
			207591_s_at	0	4.544	0.36	7	4.42	0.54	40	d	0.567238
RBMS1	11	RNA binding motif, single stranded interacting protein 1	209868_s_at	0	9.698	0.46	7	8.963	1.02	40	d	0.073018
			203748_x_at	0	9.703	0.46	7	9.094	0.65	40	d	0.024855
			215127_s_at	0	8.907	0.42	7	8.861	0.67	40	d	0.864258
			225265_at	0	7.864	0.79	7	8.17	0.84	40	u	0.385018
			225269_s_at	0	10.52	0.29	7	9.651	0.63	40	d	0.000931
			207266_x_at	0	9.681	0.47	7	9.072	0.71	40	d	0.037982

LPP	11	LIM domain containing preferred translocation partner in lipoma	202822_at	11	10.1	0.4	7	9.259	0.84	40	d	0.014845
			202821_s_at	11	6.259	0.23	7	7.182	1.13	40	u	0.000049
			230996_at	0	1.961	0.09	7	2.016	0.08	40	u	0.13354
			243874_at	0	5.209	0.77	7	5.104	1	40	d	0.794731
			1558469_at	0	2.747	0.29	7	2.882	0.54	40	u	0.53017
EIF6	11	eukaryotic translation initiation factor 6	210213_s_at	0	9.172	0.07	7	9.253	0.55	40	u	0.381455
MPPED2	11	metallophosphoesterase domain containing 2	205413_at	11	6.289	0.63	7	4.188	2.14	40	d	0.000028
			215692_s_at	0	4.576	0.74	7	3.914	1.43	40	d	0.248739
CNTN4	11	contactin 4	229084_at	11	4.588	1.01	7	3.81	1.07	40	d	0.086406
			237177_at	0	3.266	0.65	7	3.087	0.96	40	d	0.643458
RAB14	11	RAB14, member RAS oncogene family	200928_s_at	11	2.981	0.55	7	2.831	0.38	40	d	0.38783
			200927_s_at	11	8.886	0.28	7	8.487	0.97	40	d	0.04709
			211503_s_at	0	9.518	0.17	7	9.801	0.55	40	u	0.018471
BCL9L	11	B-cell CLL/lymphoma 9-like	228065_at	11	7.249	0.32	7	6.962	0.69	40	d	0.297363
			227616_at	2	7.976	0.35	7	7.565	0.85	40	d	0.223171
EYA1	11	eyes absent homolog 1 (Drosophila)	214608_s_at	11	2.513	0.27	7	2.842	0.75	40	u	0.055455
EGR2	11	early growth response 2 (Krox-20 homolog, Drosophila)	205249_at	11	10.1	0.52	7	6.915	0.95	40	d	0
HOXA10	11	homeobox A10	213147_at	11	5.628	0.37	7	5.387	0.81	40	d	0.455195
			213150_at	11	3.371	0.36	7	3.329	1.16	40	d	0.861225
OXR1	11	oxidation resistance 1	222553_x_at	11	8.631	0.39	7	8.574	0.92	40	d	0.876471
			218197_s_at	5	7.78	0.42	7	8.238	0.96	40	u	0.230829
			223879_s_at	1	5.893	0.48	7	6.751	1.28	40	u	0.093937
			238409_x_at	0	2.818	0.08	7	3.422	0.74	40	u	0.000015
CDC42	11	cell division cycle 42 (GTP binding protein, 25kDa)	208728_s_at	11	10.03	0.16	7	10.12	0.41	40	u	0.548168
			226400_at	8	10.78	0.4	7	11.07	1.08	40	u	0.498782
			208727_s_at	2	7.238	1.27	7	8.36	1.06	40	u	0.018509
			210232_at	0	2.305	0.04	7	2.546	0.4	40	u	0.000742
			214230_at	0	2.939	0.14	7	3.089	0.36	40	u	0.298157
CAMK2N1	11	calcium/calmodulin-dependent protein kinase II inhibitor 1	218309_at	10	9.994	0.69	7	8.348	1.94	40	d	0.000698
			229163_at	4	5.445	0.97	7	4.96	1.6	40	d	0.450788
			228302_x_at	0	6.456	0.82	7	5.088	2.37	40	d	0.012068
HIAT1	11	hippocampus abundant transcript 1	225222_at	11	9.233	0.18	7	9.085	0.61	40	d	0.233179
LEMD3	11	LEM domain containing 3	218604_at	11	8.766	0.29	7	8.728	0.48	40	d	0.841189
SFRS2	11	splicing factor, arginine/serine-rich 2	200753_x_at	11	8.717	0.26	7	8.98	0.6	40	u	0.268736
			200754_x_at	11	12.23	0.17	7	12.08	0.36	40	d	0.275507
			214882_s_at	11	10.42	0.22	7	10.34	0.46	40	d	0.652381
OTX2	11	orthodenticle homeobox 2	242128_at	11	2.643	0.04	7	2.78	0.2	40	u	0.000503
			231731_at	1	2.322	0.03	7	2.428	0.12	40	u	0.000045
TAF1	11	TAF1 RNA polymerase II, TATA box binding protein (TBP)-associated factor, 250kDa	227205_at	0	8.383	0.24	7	8.271	0.52	40	d	0.587986
			216955_at	0	2.6	0.23	7	2.976	0.41	40	u	0.026374
			216711_s_at	0	5.699	0.63	7	4.924	0.62	40	d	0.004363
FAM116A	11	family with sequence similarity 116, member A	226965_at	11	9.332	0.27	7	8.934	0.62	40	d	0.106647
IGF1R	11	insulin-like growth factor 1 receptor	225330_at	7	9.019	0.38	7	7.683	2.02	40	d	0.000554
			203627_at	4	6.651	0.71	7	6.225	1.8	40	d	0.547402
			203628_at	4	8.607	0.91	7	7.339	2.26	40	d	0.158507
			243358_at	1	4.839	0.9	7	4.712	1.42	40	d	0.824439
			208441_at	0	1.814	0.02	7	1.864	0.08	40	u	0.00309
DCP2	11	DCP2 decapping enzyme homolog (S. cerevisiae)	235258_at	11	5.363	0.53	7	5.722	0.73	40	u	0.226804
			238779_at	3	2.058	0.01	7	2.193	0.26	40	u	0.002184
			212919_at	0	9.18	0.35	7	8.516	0.65	40	d	0.012826
			244777_at	0	6.405	0.5	7	5.936	0.89	40	d	0.191856
UBE4B	11	ubiquitination factor E4B (UFD2 homolog, yeast)	202317_s_at	4	8.499	0.27	7	7.848	0.51	40	d	0.002435
			202316_x_at	4	5.933	0.52	7	5.669	1.51	40	d	0.420255
			226746_s_at	3	4.475	0.26	7	4.634	0.58	40	u	0.488092
			210685_s_at	2	7.004	0.39	7	6.793	0.7	40	d	0.449079
			215533_s_at	0	6.227	0.49	7	5.922	1.11	40	d	0.487592
CEBPA	11	CCAAT/enhancer binding protein (C/EBP), alpha	204039_at	11	8.409	0.89	7	7.543	0.89	40	d	0.024969
XKR6	11	XK, Kell blood group complex subunit-related family, member 6	1553640_at	11	2.612	0.56	7	2.047	0.11	40	d	0.049531
			1557436_at	0	2.295	0.1	7	2.358	0.16	40	u	0.328004
		RNA binding motif, single stranded	238447_at	11	8.99	0.61	7	5.954	1.04	40	d	0
			243041_s_at	4	4.631	0.86	7	3.515	0.39	40	d	0.020153

RBMS3	11	RNA binding motif, single stranded interacting protein	237860_at	0	2.406	0.19	7	2.34	0.17	40	d	0.371252
			1560322_at	0	3.587	0.65	7	3.361	0.34	40	d	0.437195
			206767_at	0	6.958	0.49	7	4.229	0.7	40	d	0
CCDC88A	11	coiled-coil domain containing 88A	225045_at	6	5.753	0.42	7	5.757	1.25	40	u	0.987961
			238759_at	0	2.333	0.12	7	3.125	0.95	40	u	0.00001
			1562648_at	0	3.162	0.56	7	3.362	0.52	40	u	0.36821
			221078_s_at	0	3.625	0.43	7	4.431	1.17	40	u	0.084847
			239233_at	0	3.237	0.34	7	4.128	0.9	40	u	0.014477
			219387_at	0	4.425	0.38	7	5.416	0.81	40	u	0.003285
IQSEC2	11	IQ motif and Sec7 domain 2	214819_at	11	4.441	0.38	7	4.55	0.54	40	u	0.613731
			229840_at	0	5.652	0.31	7	5.532	0.54	40	d	0.579821
RALGPS1	11	Ral GEF with PH domain and SH3 binding motif 1	204199_at	11	6.027	0.48	7	5.176	1.26	40	d	0.090671
			1555452_at	0	4.606	0.2	7	4.708	0.39	40	u	0.510025
			242689_at	0	2.4	0.1	7	2.427	0.16	40	u	0.679166
			1554745_at	0	3.116	0.12	7	3.292	0.2	40	u	0.035731
			210552_s_at	0	3.627	0.51	7	3.451	0.44	40	d	0.351767
TGIF2	11	TGFB-induced factor homeobox 2	216262_s_at	11	7.302	0.47	7	7.229	1.13	40	d	0.869648
			218724_s_at	5	5.609	0.57	7	6.621	1.19	40	u	0.03658
CNOT7	11	CCR4-NOT transcription complex, subunit 7	225053_at	11	10.56	0.21	7	10.23	0.86	40	d	0.049009
			218250_s_at	1	10.95	0.24	7	10.94	0.78	40	d	0.963448
			233019_at	0	4.916	0.61	7	4.287	0.98	40	d	0.112738
			1552344_s_at	0	4.928	0.65	7	5.284	1.26	40	u	0.478585
IRS2	11	insulin receptor substrate 2	209185_s_at	11	9.789	0.58	7	8.236	0.99	40	d	0.000275
			209184_s_at	11	8.362	0.52	7	6.893	1.03	40	d	0.000721
PDE4D	11	phosphodiesterase 4D, cAMP-specific (phosphodiesterase E3 dunce homolog, Drosophila)	204491_at	11	5.609	0.47	7	5.725	0.67	40	u	0.67164
			210837_s_at	7	2.568	0.07	7	2.783	0.33	40	u	0.000835
			210836_x_at	7	2.248	0.09	7	2.391	0.23	40	u	0.125935
			1554717_a_at	0	2.624	0.07	7	3.101	0.61	40	u	0.000029
			211840_s_at	0	2.387	0.04	7	2.452	0.12	40	u	0.012316
GLCE	11	glucuronic acid epimerase	213552_at	11	6.585	0.35	7	6.332	1.13	40	d	0.283103
RERE	11	arginine-glutamic acid dipeptide (RE) repeats	200938_s_at	11	3.971	0.44	7	3.619	0.47	40	d	0.078303
			200940_s_at	11	9.506	0.56	7	7.548	1.17	40	d	0.000111
			221643_s_at	5	6.326	0.7	7	4.471	1.53	40	d	0.003411
			1557349_at	0	2.368	0.12	7	2.568	0.27	40	u	0.065376
			200939_s_at	0	4.475	0.39	7	4.228	0.54	40	d	0.265079
CACNA2D2	11	calcium channel, voltage-dependent, alpha 2/delta subunit 2	204811_s_at	11	6.467	0.32	7	6.133	1.26	40	d	0.173021
ZNF423	11	zinc finger protein 423	214761_at	11	6.291	0.66	7	4.25	0.94	40	d	0.000002
FLRT3	11	fibronectin leucine rich transmembrane protein 3	222853_at	7	5.529	0.78	7	3.993	2	40	d	0.056295
			219250_s_at	5	6.373	0.56	7	5.244	1.95	40	d	0.006326
WIPF1	11	WAS/WASL interacting protein family, member 1	202664_at	11	8.255	0.38	7	7.7	1.08	40	d	0.025256
			202663_at	11	6.159	0.25	7	6.479	0.81	40	u	0.060828
			231182_at	0	2.806	0.37	7	2.964	0.37	40	u	0.314364
			202665_s_at	0	6.278	0.44	7	7.058	0.87	40	u	0.02771
SLC24A3	11	solute carrier family 24 (sodium/potassium/calcium exchanger),	57588_at	11	6.605	0.3	7	6.371	1.36	40	d	0.354672
			219090_at	11	7.205	0.3	7	6.706	1.5	40	d	0.071117
C17orf39	11	chromosome 17 open reading frame 39	228452_at	11	7.488	0.07	7	7.469	0.83	40	d	0.88764
			220058_at	0	3.028	0.43	7	3.314	0.69	40	u	0.302187
			241465_at	0	3.312	0.18	7	3.473	0.29	40	u	0.170222
KIAA0157	11	KIAA0157	212837_at	11	7.44	0.31	7	7.282	0.57	40	d	0.488023
			212835_at	11	5.934	0.3	7	6.547	0.7	40	u	0.031766
RIMS4	11	regulating synaptic membrane exocytosis 4	227644_at	11	3.869	0.26	7	3.927	0.64	40	u	0.818575
			233299_at	6	6.282	0.19	7	6.296	0.27	40	u	0.899564
RNF11	11	ring finger protein 11	208924_at	11	9.93	0.23	7	9.629	0.56	40	d	0.175263
EPHA8	11	EPH receptor A8	231796_at	11	3.87	0.4	7	4.212	0.42	40	u	0.05488
			1554069_at	0	2.71	0.39	7	3.013	0.78	40	u	0.32877
ARL8B	11	ADP-ribosylation factor-like 8B	217852_s_at	11	10.69	0.22	7	10.68	0.7	40	d	0.940038
			222442_s_at	11	8.136	0.4	7	9.073	1.11	40	u	0.000806
VGLL4	11	vestigial like 4 (Drosophila)	212399_s_at	11	9.098	0.21	7	8.727	0.69	40	d	0.012924
			214004_s_at	1	8.932	0.26	7	9.081	0.77	40	u	0.365821
LARP5	11	La ribonucleoprotein domain family, member 5	208952_s_at	11	7.363	0.26	7	8.24	0.89	40	u	0.000026
			208954_s_at	11	7.978	0.14	7	8.005	0.72	40	u	0.83541
			208953_at	11	7.808	0.34	7	8.223	1.01	40	u	0.064129
			228196_s_at	2	4.921	0.67	7	5.805	1.22	40	u	0.073594

			214216_s_at	0	4.224	0.37	7	4.933	1.13	40	u	0.005443
			214215_s_at	0	1.944	0.07	7	1.992	0.08	40	u	0.169715
BTG1	11	B-cell translocation gene 1, anti-proliferative	1559975_at	11	2.9	0.29	7	3.002	0.45	40	u	0.571877
			200920_s_at	11	11.38	0.33	7	10.78	0.9	40	d	0.094747
			200921_s_at	11	11.32	0.18	7	10.96	0.83	40	d	0.026196
TSC22D3	11	TSC22 domain family, member 3	208763_s_at	9	10.91	0.71	7	9.8	1.31	40	d	0.037562
			212856_at	2	6.899	0.24	7	7.021	0.66	40	u	0.4021
			207001_x_at	0	6.69	0.59	7	6.228	1.01	40	d	0.254929
			235364_at	0	2.038	0.11	7	2.059	0.08	40	u	0.565121
RCAN2	11	regulator of calcineurin 2	203498_at	11	6.55	0.36	7	5.556	0.86	40	d	0.005173
PFN2	11	profilin 2	204992_s_at	11	10.61	0.33	7	10.89	1.14	40	u	0.215886
RORB	11	RAR-related orphan receptor B	206443_at	0	1.992	0.02	7	2.108	0.46	40	u	0.122659
			242385_at	0	3.113	0.14	7	3.419	0.75	40	u	0.025338
REV3L	11	REV3-like, catalytic subunit of DNA polymerase zeta (yeast)	208070_s_at	11	8.359	0.25	7	7.792	0.76	40	d	0.001447
			238736_at	0	6.758	0.61	7	6.485	1	40	d	0.496484
SLC44A1	11	solute carrier family 44, member 1	224595_at	11	9.648	0.49	7	9.55	0.61	40	d	0.695897
			224596_at	11	10.39	0.59	7	10.39	0.51	40	d	0.98611
			228486_at	0	7.302	0.67	7	5.75	1.32	40	d	0.004796
			228485_s_at	0	7.215	0.61	7	7.165	0.92	40	d	0.89275
ADAM10	11	ADAM metallopeptidase domain 10	202604_x_at	11	6.108	0.31	7	5.797	1.28	40	d	0.203599
			214895_s_at	11	7.183	0.28	7	6.541	1.36	40	d	0.0124
PDS5B	11	PDS5, regulator of cohesion maintenance, homolog B (S. cerevisiae)	229704_at	4	7.584	0.31	7	6.695	1.29	40	d	0.000745
			242302_at	0	2.456	0.04	7	2.614	0.19	40	u	0.000033
			207956_x_at	0	7.678	0.29	7	7.389	0.71	40	d	0.308654
			204742_s_at	0	5.678	0.59	7	4.739	0.83	40	d	0.007078
			1554634_at	0	3.331	0.5	7	3.367	0.37	40	u	0.826817
			1570119_at	0	2.803	0.08	7	3.091	0.29	40	u	0.000023
			215888_at	0	5.833	0.53	7	5.621	0.96	40	d	0.581587
TAF4	11	TAF4 RNA polymerase II, TATA box binding protein (TBP)-associated factor, 135kDa	213090_s_at	11	6.494	0.42	7	6.711	0.92	40	u	0.550303
			208545_x_at	0	2.625	0.1	7	2.89	0.54	40	u	0.00787
CTCF	11	CCCTC-binding factor (zinc finger protein)	202521_at	11	8.407	0.24	7	8.139	0.47	40	d	0.153535
AFF2	11	AF4/FMR2 family, member 2	206105_at	11	2	0.01	7	2.073	0.12	40	u	0.00065
			210957_s_at	5	2.698	0.2	7	2.796	0.2	40	u	0.244141
			216364_s_at	0	2.228	0.22	7	2.216	0.14	40	d	0.852053
PPARA	11	peroxisome proliferator-activated receptor alpha	226978_at	11	5.583	0.38	7	5.309	1.01	40	d	0.490621
			223437_at	4	5.67	0.57	7	5.112	1.24	40	d	0.258019
			1558631_at	4	3.467	0.49	7	3.4	0.73	40	d	0.821304
			244689_at	4	4.593	0.47	7	4.425	0.79	40	d	0.594226
			223438_s_at	4	5.872	0.36	7	5.555	1.25	40	d	0.211107
			206870_at	1	2.707	0.18	7	3.008	0.69	40	u	0.029697
			210771_at	0	3.763	0.37	7	3.997	0.37	40	u	0.138019
			1560981_a_at	0	2.941	0.09	7	3.178	0.37	40	u	0.001726
BACE1	11	beta-site APP-cleaving enzyme 1	217904_s_at	11	7.441	0.26	7	6.388	0.69	40	d	0.00032
			222462_s_at	11	6.606	0.36	7	6.373	0.7	40	d	0.406865
			222463_s_at	1	4.683	0.34	7	4.511	0.93	40	d	0.404143
			224335_s_at	0	4.023	0.52	7	4.365	1.08	40	u	0.425903
BCL2L11	11	BCL2-like 11 (apoptosis facilitator)	225606_at	11	9.257	0.34	7	8.641	0.72	40	d	0.034079
			1558143_a_at	11	6.553	0.37	7	6.627	0.96	40	u	0.844931
			222343_at	1	4.669	0.36	7	5.175	0.97	40	u	0.190036
			1553088_a_at	0	4.968	0.15	7	5.19	0.4	40	u	0.159208
			208536_s_at	0	2.523	0.21	7	2.726	0.55	40	u	0.350736
			1555372_at	0	7.588	0.7	7	6.664	1.2	40	d	0.059566
			1553096_s_at	0	5.84	0.16	7	6.048	0.59	40	u	0.076018
PLCB1	11	phospholipase C, beta 1 (phosphoinositide-specific)	213222_at	11	7.758	0.42	7	5.424	1.72	40	d	0
			215687_x_at	1	2.469	0.17	7	2.705	0.55	40	u	0.042369
			211925_s_at	0	4.25	0.44	7	4.123	0.34	40	d	0.397476
PCDH19	11	protocadherin 19	227282_at	11	3.773	0.85	7	3.186	0.83	40	d	0.099708
ANKFY1	11	ankyrin repeat and FYVE domain containing 1	224900_at	11	8.286	0.32	7	7.378	0.48	40	d	0.000021
			219868_s_at	7	5.366	0.33	7	5.077	0.77	40	d	0.343976
			239073_at	0	2.603	0.11	7	2.622	0.14	40	u	0.75152
			1562495_at	0	2.889	0.16	7	3.094	0.43	40	u	0.228735
RECK	11	reversion-inducing-cysteine-rich protein with kazal motifs	205407_at	11	7.87	0.44	7	5.473	0.93	40	d	0
			1558115_at	0	3.224	0.23	7	3.251	0.38	40	u	0.860436
			216156_at	0	2.887	0.09	7	3.109	0.34	40	u	0.002256

		kazal motifs	1558116_x_at	0	3.494	0.29	7	3.475	0.71	40	d	0.944893
			216153_x_at	0	7.957	0.28	7	8.29	0.57	40	u	0.147272
ELF2	11	E74-like factor 2 (ets domain transcription factor)	203822_s_at	11	8.617	0.16	7	8.402	0.5	40	d	0.046482
			210361_s_at	8	7.511	0.28	7	7.515	0.59	40	u	0.98717
CAB39	11	calcium binding protein 39	217873_at	11	9.865	0.15	7	9.424	0.73	40	d	0.001608
			224311_s_at	3	6.71	0.65	7	6.668	1.57	40	d	0.945852
JMJD1C	11	jumonji domain containing 1C	221763_at	11	9.299	0.39	7	8.346	0.64	40	d	0.000524
			224933_s_at	11	8.758	0.25	7	8.867	0.72	40	u	0.491328
			228793_at	0	8.262	0.62	7	7.381	1.18	40	d	0.066105
			241659_at	0	2.457	0.25	7	2.563	0.28	40	u	0.364531
			230007_at	0	5.595	0.27	7	5.339	0.4	40	d	0.120502
			241661_at	0	3.148	0.46	7	4.236	1.35	40	u	0.000856
ARID4A	11	AT rich interactive domain 4A (RBP1-like)	205062_x_at	11	6.917	0.35	7	6.595	0.64	40	d	0.211961
			230141_at	0	6.339	0.29	7	5.597	0.66	40	d	0.006248
GATAD2B	11	GATA zinc finger domain containing 2B	225393_at	11	6.501	0.48	7	7.112	0.79	40	u	0.058182
PHF21A	11	PHD finger protein 21A	203278_s_at	11	7.341	0.32	7	7.216	0.73	40	d	0.664399
			1554153_a_at	7	5.955	0.32	7	5.795	0.83	40	d	0.623355
			1558965_at	0	4.308	0.38	7	4.439	0.69	40	u	0.633449
			227090_at	0	6.372	0.32	7	5.959	0.48	40	d	0.036213
DNAJC13	11	DnaJ (Hsp40) homolog, subfamily C, member 13	212467_at	11	8.309	0.21	7	8.373	0.51	40	u	0.750033
			1564627_at	0	2.555	0.05	7	2.665	0.13	40	u	0.000513
			1560020_at	0	2.824	0.09	7	3.478	0.88	40	u	0.000052
APBB2	10	amyloid beta (A4) precursor protein-binding, family B, member 2 (Fe65-like)	213419_at	10	6.529	0.4	7	5.756	1.57	40	d	0.013985
			40148_at	7	6.564	0.41	7	6.331	1	40	d	0.555363
			216750_at	0	4.297	0.26	7	4.046	0.68	40	d	0.3544
			216747_at	0	2.822	0.11	7	2.994	0.28	40	u	0.125079
RPS6KA5	10	ribosomal protein S6 kinase, 90kDa, polypeptide 5	204635_at	10	7.024	0.4	7	6.033	1.08	40	d	0.023673
			204633_s_at	2	9.453	0.22	7	7.517	1.44	40	d	0
			1554319_at	0	2.775	0.1	7	2.896	0.24	40	u	0.208184
SLC8A1	10	solute carrier family 8 (sodium/calcium exchanger), member 1	235518_at	10	5.628	0.57	7	5.971	0.65	40	u	0.204317
			238546_at	8	2.96	0.4	7	2.966	0.43	40	u	0.970345
			241752_at	6	3.019	0.33	7	3.703	0.79	40	u	0.032018
			207053_at	2	2.248	0.19	7	2.275	0.12	40	u	0.627299
			210804_x_at	1	3.944	0.33	7	3.941	0.4	40	d	0.98399
			211805_s_at	0	4.624	0.31	7	4.818	0.41	40	u	0.250856
			1565306_a_at	0	2.133	0.07	7	2.14	0.11	40	u	0.882014
			1561614_at	0	2.858	0.18	7	3.025	0.35	40	u	0.232565
			1564985_a_at	0	2.07	0.14	7	2.074	0.09	40	u	0.906569
			1561615_s_at	0	2.807	0.29	7	3.328	0.72	40	u	0.069665
SDC2	10	syndecan 2	212158_at	10	9.416	0.63	7	9.328	1.19	40	d	0.8524
			212154_at	10	8.651	0.91	7	8.614	1.36	40	d	0.945117
			212157_at	10	7.521	0.66	7	6.966	1.19	40	d	0.243768
SPRY2	10	sprouty homolog 2 (Drosophila)	204011_at	10	8.843	0.44	7	5.672	0.88	40	d	0
KCNK10	10	potassium channel, subfamily K, member 10	220727_at	10	4.533	0.23	7	4.427	0.38	40	d	0.493722
GPATCH8	10	G patch domain containing 8	212487_at	10	6.751	0.37	7	6.863	0.7	40	u	0.689734
			212485_at	10	7.765	0.33	7	6.966	0.61	40	d	0.001871
BTBD10	10	BTB (POZ) domain containing 10	223174_at	10	8.166	0.25	7	8.185	0.52	40	u	0.925774
CUL3	10	cullin 3	201372_s_at	10	3.055	0.5	7	2.987	0.29	40	d	0.753761
			201370_s_at	10	6.761	0.42	7	6.522	0.85	40	d	0.47927
			201371_s_at	2	10.6	0.25	7	10.46	0.46	40	d	0.453737
WSB1	10	WD repeat and SOCS box-containing 1	201296_s_at	10	10.17	0.23	7	9.374	0.65	40	d	0.000011
			201294_s_at	10	8.213	0.51	7	8.067	0.98	40	d	0.708526
			210561_s_at	4	10.67	0.27	7	9.985	0.71	40	d	0.017856
			213406_at	0	2.288	0.01	7	2.587	0.62	40	u	0.004438
VAT1	10	vesicle amine transport protein 1 homolog (T. californica)	208626_s_at	10	10.13	0.22	7	9.761	0.74	40	d	0.01816
ANKRD38	10	ankyrin repeat domain 38	229125_at	2	6.215	1.14	7	5.778	2.21	40	d	0.618715
POU3F2	10	POU class 3 homeobox 2	242455_at	10	3.799	0.41	7	3.921	0.43	40	u	0.503791
			207084_at	1	4.314	0.25	7	4.396	0.32	40	u	0.530509
ADCY1	10	adenylate cyclase 1 (brain)	213245_at	10	5.371	1.59	7	4.87	1.97	40	d	0.535554
			232062_at	4	2.087	0.07	7	2.358	0.66	40	u	0.017596
			235049_at	4	4.794	1.28	7	3.951	1.58	40	d	0.196965
			215348_at	0	6.2	0.12	7	6.076	0.2	40	d	0.125111

			215340_at	0	2.202	0.18	7	2.301	0.34	40	u	0.458029
SLC6A1	10	solute carrier family 6 (neurotransmitter transporter, GABA), member 1	205152_at	10	2.445	0.09	7	2.538	0.14	40	u	0.100854
SLC17A6	10	solute carrier family 17 (sodium-dependent inorganic phosphate cotransporter), member 6	220551_at	10	2.188	0.02	7	2.309	0.18	40	u	0.000218
PADI4	10	peptidyl arginine deiminase, type IV	211413_s_at	0	2.746	0.04	7	2.925	0.33	40	u	0.002532
			220001_at	0	2.508	0.2	7	2.43	0.05	40	d	0.383013
			211412_at	0	2.434	0.02	7	2.563	0.26	40	u	0.00427
CREB1	10	cAMP responsive element binding protein 1	204313_s_at	10	7.842	0.42	7	7.916	0.73	40	u	0.800051
			204314_s_at	10	7.781	0.2	7	8.085	0.61	40	u	0.022655
			204312_x_at	10	6.914	0.64	7	7.222	1.14	40	u	0.498694
			214513_s_at	0	5.928	0.62	7	6.359	1.01	40	u	0.289056
CALCR	10	calcitonin receptor	207886_s_at	10	2.896	0.06	7	2.998	0.2	40	u	0.017992
			207887_s_at	2	2.1	0.03	7	2.205	0.17	40	u	0.000964
ATP2B4	10	ATPase, Ca++ transporting, plasma membrane 4	212135_s_at	10	9.113	0.21	7	9.13	0.82	40	u	0.916885
			212136_at	10	8.591	0.32	7	9.062	0.72	40	u	0.102819
			205410_s_at	0	2.988	0.33	7	3.347	0.9	40	u	0.081674
RAB22A	10	RAB22A, member RAS oncogene family	213405_at	10	7.793	0.2	7	7.594	0.75	40	d	0.181603
			218360_at	5	7.634	0.26	7	8.576	0.58	40	u	0.000148
TARDBP	10	TAR DNA binding protein	221264_s_at	10	8.622	0.18	7	7.214	1.01	40	d	0
			200020_at	6	10.04	0.19	7	9.994	0.4	40	d	0.789888
CLCN3	10	chloride channel 3	201735_s_at	10	8.683	0.35	7	8.782	0.85	40	u	0.768728
			201734_at	2	9.561	0.39	7	8.961	0.86	40	d	0.081717
			201732_s_at	2	6.896	0.43	7	7.656	0.82	40	u	0.022847
			201733_at	2	5.753	0.55	7	6.344	0.67	40	u	0.035929
GPAM	10	glycerol-3-phosphate acyltransferase, mitochondrial	225424_at	10	7.821	1	7	5.876	0.76	40	d	0.000001
			225420_at	10	7.883	0.88	7	6.526	0.81	40	d	0.000279
ZNF532	10	zinc finger protein 532	220617_s_at	8	9.031	0.35	7	8.438	0.96	40	d	0.121103
			225021_at	7	7.056	0.42	7	6.459	1.12	40	d	0.180173
FOSB	10	FBJ murine osteosarcoma viral oncogene homolog B	202768_at	10	10.82	1.29	7	6.344	1.6	40	d	0
JPH1	10	junctophilin 1	229139_at	9	4.044	0.49	7	5.142	1.36	40	u	0.001116
			1553533_at	0	3.279	0.27	7	3.667	0.53	40	u	0.06896
C16orf70	10	chromosome 16 open reading frame 70	223440_at	10	6.847	0.4	7	6.833	0.56	40	d	0.948383
			220601_at	0	2.554	0.04	7	2.68	0.28	40	u	0.012345
RHOBTB1	10	Rho-related BTB domain containing 1	212651_at	10	6.913	0.37	7	6.856	0.82	40	d	0.859778
YPEL2	10	yippee-like 2 (Drosophila)	227020_at	10	8.416	0.39	7	8.025	1.1	40	d	0.1145
			229060_at	3	6.331	0.45	7	6.535	0.93	40	u	0.580685
			1556420_s_at	0	5.063	0.48	7	4.645	0.77	40	d	0.179181
EDG1	10	endothelial differentiation, sphingolipid G-protein-coupled receptor, 1	204642_at	10	5.87	0.79	7	4.972	0.63	40	d	0.002058
RAP1A	10	RAP1A, member of RAS oncogene family	202362_at	10	9.443	0.28	7	9.438	0.59	40	d	0.982687
			1555339_at	0	1.967	0.02	7	2.002	0.03	40	u	0.016741
			1555340_x_at	0	2.034	0.05	7	2.047	0.03	40	u	0.360815
CTDSPL2	10	CTD (carboxy-terminal domain, RNA polymerase II, polypeptide A) small phosphatase like 2	223270_at	12	7.19	0.33	7	7.251	0.6	40	u	0.800439
			223271_s_at	9	7.665	0.43	7	8.008	0.93	40	u	0.354189
			239133_at	0	6.777	0.58	7	6.107	0.69	40	d	0.021657
			1555106_a_at	0	3.558	0.54	7	4.521	1.35	40	u	0.075523
IHPK1	10	inositol hexaphosphate kinase 1	212439_at	10	7.638	0.22	7	6.889	0.67	40	d	0.000015
MYO1C	10	myosin IC	225080_at	10	8.724	0.28	7	8.11	0.78	40	d	0.001549
			214656_x_at	10	9.022	0.23	7	8.573	0.51	40	d	0.030586
			32811_at	10	8.768	0.34	7	8.34	0.64	40	d	0.099367
HBEGF	10	heparin-binding EGF-like growth factor	38037_at	8	5.518	0.6	7	4.877	0.6	40	d	0.014378
			203821_at	8	6.467	0.76	7	5.781	0.78	40	d	0.041233
			244857_at	0	2.728	0.14	7	2.804	0.24	40	u	0.423879
			222076_at	0	2.451	0.18	7	2.556	0.18	40	u	0.174303
BCL9	10	B-cell CLL/lymphoma 9	204129_at	10	5.947	0.27	7	6.252	0.62	40	u	0.214974
BCL2L2	10	BCL2-like 2	209311_at	10	8.419	0.28	7	7.418	0.76	40	d	0.000004
			1555140_a_at	0	4.102	0.57	7	3.946	0.85	40	d	0.649187
AEBP2	10	AE binding protein 2	225889_at	10	8.248	0.3	7	8.122	0.76	40	d	0.674432
			235184_at	0	3.317	0.55	7	3.355	0.48	40	u	0.854828
HECW2	10	HECT, C2 and WW domain containing E3 ubiquitin protein ligase 2	232080_at	10	3.967	0.72	7	4.966	1.07	40	u	0.024635
			243080_at	0	2.344	0.16	7	2.426	0.16	40	u	0.235224

PLEKHA3	10	pleckstrin homology domain containing, family A (phosphoinositide binding specific) member 3	223370_at	10	7.468	0.39	7	7.817	0.56	40	u	0.128386
			227659_at	0	3.2	0.12	7	3.427	0.25	40	u	0.028684
			227658_s_at	0	6.234	0.5	7	6.8	0.74	40	u	0.062422
LARP1	10	La ribonucleoprotein domain family, member 1	212137_at	10	10.09	0.34	7	10.11	0.71	40	u	0.964565
			212193_s_at	4	8.909	0.32	7	9.167	0.83	40	u	0.434231
			210966_x_at	4	9.221	0.23	7	9.325	0.67	40	u	0.473537
			239663_x_at	0	3.618	0.16	7	3.303	0.49	40	d	0.00499
RABGAP1	10	RAB GTPase activating protein 1	204028_s_at	10	9.809	0.22	7	9.077	0.63	40	d	0.000015
			213313_at	10	9.552	0.25	7	9.035	0.55	40	d	0.020344
			215070_x_at	0	5.94	0.14	7	5.838	0.4	40	d	0.243907
PELI1	10	pellino homolog 1 (Drosophila)	218319_at	10	10.03	0.58	7	8.752	1.07	40	d	0.004224
			232304_at	0	8.51	0.35	7	6.574	1.27	40	d	0
			232213_at	0	8.722	0.41	7	6.786	0.85	40	d	0.000001
MCL1	10	myeloid cell leukemia sequence 1 (BCL2-related)	214056_at	10	5.982	0.37	7	6.42	0.79	40	u	0.164636
			200796_s_at	10	6.75	1.12	7	6.754	1.99	40	u	0.995589
			214057_at	10	5.701	0.33	7	6.013	0.54	40	u	0.155422
			200797_s_at	10	12.29	0.28	7	11.86	0.52	40	d	0.043261
			200798_x_at	3	9.845	0.75	7	9.942	1.28	40	u	0.85125
			227175_at	1	1.899	0.07	7	1.977	0.22	40	u	0.094528
CREBL2	10	cAMP responsive element binding protein-like 2	201989_s_at	10	10.17	0.22	7	9.25	0.8	40	d	0.000001
			201988_s_at	10	8.241	0.42	7	7.379	1.24	40	d	0.003139
			201990_s_at	10	8.459	0.41	7	7.505	1.18	40	d	0.000935
PPAP2B	10	phosphatidic acid phosphatase type 2B	212226_s_at	10	10.84	0.32	7	8.513	0.75	40	d	0
			212230_at	10	9.861	0.27	7	7.679	0.82	40	d	0
			209355_s_at	0	9.824	0.41	7	7.053	0.95	40	d	0
CBLN4	10	cerebellin 4 precursor	242524_at	10	3.18	0.58	7	2.963	0.32	40	d	0.407308
			234024_at	0	2.835	0.24	7	2.715	0.27	40	d	0.287008
SFRS12	10	splicing factor, arginine/serine-rich 12	212721_at	10	10.28	0.29	7	9.625	0.68	40	d	0.017996
			243361_at	0	4.271	0.54	7	4.55	0.79	40	u	0.385615
			244287_at	0	5.887	0.64	7	6.08	0.92	40	u	0.60367
			1568783_at	0	3.067	0.33	7	3.335	0.48	40	u	0.167797
FBXW11	10	F-box and WD repeat domain containing 11	209456_s_at	1	5.822	0.65	7	6.284	1.29	40	u	0.369914
			209455_at	1	8.698	0.37	7	8.554	0.7	40	d	0.604552
C18orf25	10	chromosome 18 open reading frame 25	226406_at	10	7.846	0.31	7	7.943	0.79	40	u	0.755819
			217539_at	3	3.259	0.37	7	3.654	0.81	40	u	0.21873
			217508_s_at	1	2.48	0.23	7	3.147	0.77	40	u	0.000157
			1553686_at	0	3.246	0.35	7	3.245	0.35	40	d	0.996724
RARG	10	retinoic acid receptor, gamma	204189_at	10	7.344	0.22	7	6.93	0.59	40	d	0.079422
			204188_s_at	0	5.855	0.28	7	5.768	0.77	40	d	0.773959
			217178_at	0	2.151	0.02	7	2.196	0.05	40	u	0.026098
ACVR1B	10	activin A receptor, type IB	213198_at	10	8.674	0.38	7	7.931	0.77	40	d	0.018533
			205209_at	5	5.443	0.46	7	5.627	0.64	40	u	0.481971
			208223_s_at	0	3.734	0.3	7	4.166	0.86	40	u	0.028274
			208218_s_at	0	3.438	0.29	7	3.752	0.41	40	u	0.062285
			208219_at	0	2.869	0.09	7	2.962	0.38	40	u	0.205926
			208222_at	0	2.976	0.31	7	2.994	0.37	40	u	0.905086
SYNJ1	10	synaptojanin 1	212990_at	10	6.346	0.36	7	6.288	0.64	40	d	0.823718
			207594_s_at	0	2.349	0.14	7	2.82	0.6	40	u	0.000136
			232993_at	0	3.441	0.2	7	3.651	0.25	40	u	0.04436
PHF20L1	10	PHD finger protein 20-like 1	226942_at	10	7.323	0.41	7	7.713	0.81	40	u	0.22745
			1554472_a_at	0	4.501	0.41	7	6.385	1.11	40	u	0.000084
			231967_at	0	4.747	0.64	7	5.811	1.2	40	u	0.030424
			219606_at	0	5.337	0.49	7	5.553	0.66	40	u	0.42028
			230098_at	0	6.923	0.28	7	7.704	0.93	40	u	0.000286
			227523_s_at	0	8.1	0.25	7	8.846	0.71	40	u	0.000067
			222133_s_at	0	7.329	0.28	7	7.918	0.81	40	u	0.002142
PTBP2	10	polypyrimidine tract binding protein 2	218683_at	10	7.58	0.35	7	7.572	0.93	40	d	0.983494
			1554614_a_at	1	5.298	0.49	7	4.817	1.09	40	d	0.269199
GPM6B	10	glycoprotein M6B	209169_at	10	6.568	0.38	7	5.213	1.69	40	d	0.000095
			209167_at	10	9.283	0.31	7	6.841	2.15	40	d	0
			209168_at	10	8.687	0.46	7	7.153	1.7	40	d	0.00005
			209170_s_at	0	9.214	0.44	7	7.065	2.1	40	d	0.000001
SCN2B	10	sodium channel, voltage-gated, type II, beta	235225_at	10	3.481	0.52	7	2.386	0.25	40	d	0.002355
			210363_s_at	0	2.592	0.33	7	2.409	0.07	40	d	0.229373

			210364_at	0	4.229	0.35	7	3.941	0.39	40	d	0.081124
MIB1	10	mindbomb homolog 1 (Drosophila)	224720_at	10	8.72	0.21	7	8.454	0.74	40	d	0.080598
			224725_at	10	7.071	0.39	7	7.788	0.91	40	u	0.051406
			224722_at	10	6.624	0.31	7	6.548	0.75	40	d	0.798777
			224726_at	10	8.023	0.22	7	8.833	0.73	40	u	0.000006
			1558645_at	0	2.683	0.14	7	2.946	0.49	40	u	0.011179
			236573_at	0	2.511	0.05	7	2.643	0.14	40	u	0.000228
UNC5A	10	unc-5 homolog A (C. elegans)	236448_at	10	2.341	0.64	7	3.01	2.03	40	u	0.121335
			243833_at	0	3.984	0.46	7	3.996	0.5	40	u	0.953994
PCGF3	10	polycomb group ring finger 3	212753_at	10	9.056	0.33	7	8.257	1	40	d	0.000736
			230408_at	2	6.476	0.89	7	6.122	0.79	40	d	0.301495
			1559528_at	0	3.237	0.4	7	3.517	0.49	40	u	0.165356
			204564_at	0	6.556	0.33	7	6.93	0.68	40	u	0.168665
			238084_at	0	3.931	0.64	7	4.226	0.93	40	u	0.436002
ESRRA	10	estrogen-related receptor alpha	203193_at	10	7.077	0.29	7	6.967	0.49	40	d	0.575767
			1487_at	10	6.841	0.29	7	6.671	0.7	40	d	0.537172
RAB5B	10	RAB5B, member RAS oncogene family	201276_at	10	9.034	0.14	7	8.253	0.78	40	d	0.000001
CNTFR	10	ciliary neurotrophic factor receptor	205723_at	2	5.171	0.44	7	5.131	0.42	40	d	0.820567
RARB	10	retinoic acid receptor, beta	205080_at	10	6.494	0.33	7	5.035	1.04	40	d	0
			208413_at	0	2.385	0.04	7	2.469	0.1	40	u	0.040111
			217020_at	0	2.328	0.11	7	2.503	0.28	40	u	0.118271
			208412_s_at	0	4.788	0.31	7	4.454	0.67	40	d	0.209287
			208530_s_at	0	4.34	0.71	7	3.369	1.1	40	d	0.03269
SCN3A	10	sodium channel, voltage-gated, type III, alpha subunit	210432_s_at	10	5.404	1.26	7	2.706	0.72	40	d	0.002219
			232512_at	10	2.805	0.28	7	3.032	0.8	40	u	0.200675
FRMPD4	10	FERM and PDZ domain containing 4	215052_at	10	3.149	0.08	7	3.254	0.16	40	u	0.099022
DAG1	10	dystroglycan 1 (dystrophin-associated glycoprotein 1)	212128_s_at	10	7.238	0.55	7	6.735	1	40	d	0.210562
			205417_s_at	10	8.866	0.39	7	8.05	0.86	40	d	0.019808
KLF9	10	Kruppel-like factor 9	203542_s_at	10	7.769	0.67	7	6.934	0.78	40	d	0.011973
			203541_s_at	10	2.317	0.13	7	2.315	0.11	40	d	0.962112
			203543_s_at	9	8.157	0.74	7	6.86	0.99	40	d	0.002186
			228474_s_at	0	2.214	0.08	7	2.202	0.08	40	d	0.715131
			230636_s_at	0	3.857	0.64	7	2.705	0.48	40	d	0.000003
ARID2	10	AT rich interactive domain 2 (ARID, RFX-like)	225490_at	10	7.794	0.21	7	7.673	0.67	40	d	0.383675
			225486_at	10	6.798	0.5	7	6.214	0.72	40	d	0.049989
			231090_s_at	8	5.988	0.24	7	6.503	0.71	40	u	0.001826
			1553349_at	0	7.298	0.55	7	6.372	1.16	40	d	0.049043
REEP1	10	receptor accessory protein 1	204364_s_at	10	6.973	1.6	7	6.046	2.34	40	d	0.329756
			204365_s_at	10	6.255	1.55	7	6.082	1.7	40	d	0.805805
SGK	10	serum/glucocorticoid regulated kinase	201739_at	10	10.86	0.28	7	9.863	0.8	40	d	0.000005
WDTC1	10	WD and tetratricopeptide repeats 1	215497_s_at	10	4.948	0.51	7	4.022	0.76	40	d	0.003859
			40829_at	10	8.541	0.22	7	7.771	0.41	40	d	0.000025
			216036_x_at	10	6.75	0.27	7	6.445	0.52	40	d	0.144724
FEM1C	10	fem-1 homolog c (C. elegans)	213341_at	9	6.793	0.61	7	6.838	0.96	40	u	0.906487
YWHAG	10	tyrosine 3-monooxygenase/tryptophan 5-monooxygenase activation protein, gamma polypeptide	222985_at	10	10.98	0.13	7	11.39	0.47	40	u	0.000108
TSC1	10	tuberous sclerosis 1	209390_at	10	8.61	0.29	7	7.698	0.57	40	d	0.000189
TNRC6A	10	trinucleotide repeat containing 6A	224704_at	8	8.259	0.24	7	7.34	0.76	40	d	0.000003
			234734_s_at	8	8.592	0.39	7	8.196	0.71	40	d	0.168156
			224705_s_at	8	8.506	0.13	7	8.147	0.81	40	d	0.013757
			1553346_a_at	0	4.155	0.71	7	5.108	1.16	40	u	0.045314
			243834_at	0	7.62	0.32	7	7.093	0.56	40	d	0.022289
			233836_at	0	4.693	0.48	7	4.516	0.86	40	d	0.607397
SPTY2D1	10	SPT2, Suppressor of Ty, domain containing 1 (S. cerevisiae)	229594_at	10	7.942	0.32	7	7.981	0.45	40	u	0.834218
			235440_at	6	5.822	0.25	7	6.356	0.61	40	u	0.031213
MYCN	10	v-myc myelocytomatosis viral related oncogene, neuroblastoma derived (avian)	209756_s_at	10	4.016	0.53	7	4.166	0.68	40	u	0.591057
			209757_s_at	10	3.635	0.41	7	4.682	1.59	40	u	0.001428
			242026_at	0	2.421	0.3	7	2.338	0.25	40	d	0.452346
			211377_x_at	0	2.018	0.07	7	2.1	0.28	40	u	0.131891
			234376_at	0	2.264	0.32	7	2.29	0.22	40	u	0.801958
HNRPK	10	heterogeneous nuclear ribonucleoprotein K	200097_s_at	10	11.57	0.23	7	11.37	0.48	40	d	0.292759
			200775_s_at	6	12.57	0.14	7	12.6	0.39	40	u	0.700311
PKIA	10	protein kinase (cAMP-dependent, catalytic)	204612_at	10	4.636	0.81	7	4.935	1.86	40	u	0.684699

[illegible]	[illegible]	inhibitor alpha	226864_at	0	3.218	0.41	7	3.319	1.21	40	u	0.696634
MNT	10	MAX binding protein	204206_at	10	7.193	0.46	7	6.667	0.88	40	d	0.136994
			236749_at	0	4.924	0.42	7	4.752	0.51	40	d	0.416879
ANKRD13A	10	ankyrin repeat domain 13A	224810_s_at	10	9.858	0.22	7	9.007	0.76	40	d	0.000003
			238851_at	0	2.993	0.29	7	3.341	0.54	40	u	0.111869
CD164	10	CD164 molecule, sialomucin	208653_s_at	10	8.426	0.69	7	8.828	1.3	40	u	0.439937
			208654_s_at	10	11.21	0.31	7	10.69	1.19	40	d	0.027181
			208405_s_at	10	12.31	0.24	7	11.72	0.81	40	d	0.000962
SPTBN4	10	spectrin, beta, non-erythrocytic 4	220185_at	10	6.113	0.19	7	6.12	0.27	40	u	0.950657
			224297_s_at	0	4.424	0.33	7	4.444	0.38	40	u	0.896868
			224144_at	0	4.533	0.21	7	4.62	0.35	40	u	0.539501
			224551_s_at	0	3.769	0.22	7	3.587	0.3	40	d	0.146545
			224145_s_at	0	5.239	0.27	7	5.181	0.47	40	d	0.760063
ZC3H12B	10	zinc finger CCCH-type containing 12B	229234_at	10	3.153	0.45	7	2.814	0.3	40	d	0.015799
			234878_at	0	2.373	0.07	7	2.497	0.24	40	u	0.016416
EDEM3	10	ER degradation enhancer, mannosidase alpha-like 3	223243_s_at	10	8.209	0.36	7	8.958	0.58	40	u	0.002159
			220926_s_at	5	7.797	0.38	7	8.349	0.71	40	u	0.055437
			220342_x_at	3	6.861	0.4	7	8.037	0.82	40	u	0.000723
NRP1	10	neuropilin 1	212298_at	10	8.117	0.56	7	6.988	0.88	40	d	0.002367
			210510_s_at	0	5.544	0.81	7	5.085	1.17	40	d	0.33394
			210615_at	0	2.934	0.14	7	3.191	0.36	40	u	0.072794
			1561365_at	0	2.746	0.08	7	2.851	0.15	40	u	0.083298
DICER1	10	Dicer1, Dcr-1 homolog (Drosophila)	212888_at	10	9.557	0.16	7	8.769	0.69	40	d	0
			206061_s_at	4	8.398	0.68	7	8.077	1.06	40	d	0.452107
			213229_at	4	9.553	0.32	7	9.406	0.67	40	d	0.57946
			216281_at	0	2.494	0.16	7	2.595	0.29	40	u	0.37899
			216260_at	0	2.897	0.42	7	2.778	0.27	40	d	0.347736
			216280_s_at	0	2.944	0.1	7	3.081	0.13	40	u	0.012375
BDNF	10	brain-derived neurotrophic factor	239367_at	10	2.385	0.03	7	2.63	0.36	40	u	0.000134
			206382_s_at	6	2.551	0.18	7	2.709	0.63	40	u	0.21787
LSM14A	10	LSM14A, SCD6 homolog A (S. cerevisiae)	212132_at	10	10.16	0.27	7	10.18	0.56	40	u	0.921567
			222099_s_at	10	8.004	0.18	7	7.751	0.49	40	d	0.027219
			212131_at	10	10.01	0.19	7	9.503	0.75	40	d	0.000929
FNIP1	10	folliculin interacting protein 1	228768_at	10	8.065	0.29	7	7.899	0.59	40	d	0.476675
			228250_at	1	5.02	0.51	7	5.817	0.78	40	u	0.014262
			1559060_a_at	0	3.057	0.14	7	3.285	0.35	40	u	0.10803
			223997_at	0	4.02	0.34	7	4.211	0.62	40	u	0.443209
TTYH3	10	tweety homolog 3 (Drosophila)	224674_at	10	7.089	0.34	7	7.587	0.81	40	u	0.122739
NRIP1	10	nuclear receptor interacting protein 1	202600_s_at	10	8.904	0.7	7	8.951	1.57	40	u	0.940388
			202599_s_at	10	9.75	0.49	7	9.06	1.43	40	d	0.033017
DHX40	10	DEAH (Asp-Glu-Ala-His) box polypeptide 40	222574_s_at	10	8.384	0.34	7	8.262	1.15	40	d	0.602188
			218277_s_at	10	9.305	0.29	7	9.326	0.74	40	u	0.942649
DLL1	10	delta-like 1 (Drosophila)	224215_s_at	10	7.652	0.63	7	5.529	1.07	40	d	0.00001
			227938_s_at	10	2.878	0.24	7	3.06	0.29	40	u	0.134992
EPB41L1	10	erythrocyte membrane protein band 4.1-like 1	212339_at	10	6.468	0.26	7	5.873	1.44	40	d	0.023606
			212336_at	10	6.875	0.42	7	7.136	1	40	u	0.508913
			230289_at	6	2.16	0.05	7	2.213	0.08	40	u	0.115072
			222066_at	6	3.532	0.49	7	3.533	0.55	40	u	0.995541
RAB21	10	RAB21, member RAS oncogene family	203885_at	10	8.565	0.23	7	8.4	0.72	40	d	0.276117
			226268_at	0	6.687	0.35	7	6.496	0.6	40	d	0.429955
SMAD7	10	SMAD family member 7	204790_at	10	7.264	0.55	7	7.194	0.85	40	d	0.838213
ACCN2	10	amiloride-sensitive cation channel 2, neuronal	205156_s_at	10	4.608	0.52	7	4.607	0.61	40	d	0.998105
			37953_s_at	10	4.196	0.47	7	4.249	0.44	40	u	0.777717
CLOCK	10	clock homolog (mouse)	204980_at	10	8.01	0.32	7	8.247	0.61	40	u	0.331517
			217563_at	0	1.92	0.02	7	2.043	0.22	40	u	0.001799
SORCS1	10	sortilin-related VPS10 domain containing receptor 1	228194_s_at	10	2.194	0.11	7	2.699	1.06	40	u	0.006283
			1556891_at	1	2.639	0.22	7	2.92	0.54	40	u	0.188132
MARCKS	10	myristoylated alanine-rich protein kinase C substrate	201668_x_at	10	8.098	0.61	7	8.371	1.39	40	u	0.618658
			201669_s_at	10	10.79	0.32	7	10.67	1.01	40	d	0.588681
			213002_at	10	6.787	0.26	7	7.512	1.04	40	u	0.000701
			201670_s_at	2	10.39	0.22	7	10.15	1.05	40	d	0.225077
MBD6	10	methyl-CpG binding domain protein 6	226076_s_at	10	6.454	0.48	7	6.088	1.05	40	d	0.380233
			227833_s_at	10	8.28	0.48	7	7.512	1.39	40	d	0.016222
			227832_at	10	5.374	0.2	7	5.368	0.47	40	d	0.972926

			214384_s_at	10	3.621	0.26	7	3.629	0.26	40	u	0.941671
ZDHHC9	10	zinc finger, DHHC-type containing 9	222451_s_at	3	7.053	0.25	7	7.7	0.81	40	u	0.000528
CLCN5	10	chloride channel 5 (nephrolithiasis 2, X-linked, Dent disease)	206704_at	10	2.872	0.33	7	3.848	0.72	40	u	0.001301
			226273_at	0	2.892	0.27	7	3.714	0.99	40	u	0.000149
			226274_at	0	4.251	0.4	7	4.631	1.06	40	u	0.364874
			232128_s_at	0	2.266	0.16	7	2.447	0.54	40	u	0.106172
			232127_at	0	2.42	0.14	7	3.138	0.88	40	u	0.000028
TSHZ3	10	teashirt zinc finger homeobox 3	223392_s_at	10	6.252	0.48	7	4.898	0.96	40	d	0.000893
			223393_s_at	10	6.526	0.64	7	4.726	0.8	40	d	0.000002
PRKD1	10	protein kinase D1	205880_at	10	5.919	0.46	7	4.11	1.02	40	d	0.000048
			217705_at	0	3.031	0.12	7	3.206	0.3	40	u	0.142527
WTAP	10	Wilms tumor 1 associated protein	203137_at	10	9.164	0.38	7	8.911	0.76	40	d	0.402339
			210285_x_at	2	7.926	0.54	7	7.597	1.14	40	d	0.467532
			214759_at	0	2.362	0.24	7	2.379	0.3	40	u	0.885422
			1560274_at	0	3.477	0.49	7	3.829	0.69	40	u	0.212834
			241626_at	0	2.363	0.08	7	2.523	0.31	40	u	0.011732
			229630_s_at	0	10.2	0.22	7	9.933	0.48	40	d	0.159218
ROBO2	10	roundabout, axon guidance receptor, homolog 2 (Drosophila)	226766_at	10	2.949	0.43	7	3.582	1.19	40	u	0.022951
			226709_at	10	2.223	0.05	7	2.695	1.06	40	u	0.008876
			240425_x_at	0	3.693	0.25	7	4.09	0.42	40	u	0.020973
SLITRK1	10	SLIT and NTRK-like family, member 1	236734_at	10	2.698	0.29	7	2.843	0.56	40	u	0.511301
ETV6	10	ets variant gene 6 (TEL oncogene)	225764_at	10	7.966	0.5	7	7.537	0.99	40	d	0.277305
			235056_at	4	7.869	0.52	7	7.555	0.98	40	d	0.423604
			205585_at	1	5.882	0.39	7	6.239	0.91	40	u	0.322011
			239740_at	0	8.725	0.48	7	7.515	1.05	40	d	0.005408
SNF1LK	10	SNF1-like kinase	208078_s_at	10	8.941	0.8	7	7.584	1.23	40	d	0.008218
			232470_at	0	4.889	0.24	7	4.613	0.39	40	d	0.083932
OSBPL8	9	oxysterol binding protein-like 8	212582_at	9	10.5	0.44	7	10.5	0.54	40	u	0.999109
			212585_at	9	9.316	0.27	7	9.185	0.56	40	d	0.557507
			228986_at	0	5.806	0.34	7	7.295	0.62	40	u	0
CEP350	9	centrosomal protein 350kDa	204373_s_at	9	9.024	0.25	7	9.583	0.47	40	u	0.004407
			213165_at	9	7.934	0.29	7	8.463	0.49	40	u	0.00967
			213956_at	0	7.899	0.32	7	8.889	0.66	40	u	0.00041
			213957_s_at	0	5.854	0.34	7	7.009	0.62	40	u	0.000022
RAD23B	9	RAD23 homolog B (S. cerevisiae)	223598_at	10	8.716	0.42	7	8.367	0.61	40	d	0.159852
			201222_s_at	9	10.47	0.46	7	11.16	0.77	40	u	0.028013
			201223_s_at	9	9.346	0.15	7	9.829	0.49	40	u	0.000045
			214422_at	0	3.721	0.32	7	4.175	0.68	40	u	0.094921
GRB2	9	growth factor receptor-bound protein 2	223049_at	9	9.488	0.35	7	9.69	0.74	40	u	0.492791
			228572_at	5	4.872	0.33	7	5.235	0.76	40	u	0.229436
			215075_s_at	2	7.747	0.4	7	8.738	0.7	40	u	0.000877
CACNB1	9	calcium channel, voltage-dependent, beta 1 subunit	210185_at	9	4.707	0.39	7	4.866	0.69	40	u	0.5676
			210967_x_at	0	2.758	0.35	7	2.724	0.38	40	d	0.829081
			206996_x_at	0	2.615	0.24	7	2.702	0.34	40	u	0.524796
UBE2W	9	ubiquitin-conjugating enzyme E2W (putative)	218521_s_at	9	5.805	0.46	7	7.429	0.86	40	u	0.000019
			222657_s_at	9	6.384	0.44	7	7.908	0.79	40	u	0.000014
			222656_at	9	6.115	0.66	7	6.746	0.85	40	u	0.074132
			1562458_at	0	2.479	0.1	7	2.559	0.25	40	u	0.410357
			1555217_at	0	2.457	0.05	7	2.644	0.3	40	u	0.000877
RTN4RL1	9	reticulon 4 receptor-like 1	230700_at	9	6.545	0.61	7	5.428	0.84	40	d	0.001939
TNKS2	9	tankyrase, TRF1-interacting ankyrin-related ADP-ribose polymerase 2	222562_s_at	9	6.379	1.04	7	7.194	0.94	40	u	0.047094
			218228_s_at	9	9.453	0.22	7	9.066	0.47	40	d	0.040245
			222563_s_at	2	5.705	0.48	7	6.203	0.66	40	u	0.069181
			241909_at	0	2.749	0.11	7	2.931	0.21	40	u	0.031848
MTMR12	9	myotubularin related protein 12	225232_at	9	9.945	0.32	7	9.591	0.84	40	d	0.285467
			220953_s_at	0	5.835	0.43	7	6.118	1	40	u	0.475508
LRP1B	9	low density lipoprotein-related protein 1B (deleted in tumors)	219643_at	9	2.405	0.05	7	2.677	0.8	40	u	0.040912
SLC16A2	9	solute carrier family 16, member 2 (monocarboxylic acid transporter 8)	204462_s_at	9	5.973	0.41	7	5.38	0.7	40	d	0.037805
BSDC1	9	BSD domain containing 1	222200_s_at	9	8.736	0.2	7	8.344	0.57	40	d	0.004021
			218004_at	9	8.807	0.23	7	8.127	0.5	40	d	0.001279
			1559971_at	0	3.362	0.63	7	3.375	0.58	40	u	0.958623
STX5	9	syntaxin 5	203330_s_at	9	7.225	0.21	7	6.907	0.77	40	d	0.042541

KIF1B	9	kinesin family member 1B	209234_at	9	7.382	0.28	7	6.79	0.87	40	d	0.002604
			225878_at	0	7.151	0.35	7	7.028	1.12	40	d	0.595455
			226968_at	0	8.523	0.28	7	7.759	0.94	40	d	0.000318
GRID1	9	glutamate receptor, ionotropic, delta 1	231977_at	9	2.342	0.03	7	2.392	0.05	40	u	0.016588
			1555267_at	1	1.493	0.04	7	1.553	0.18	40	u	0.071429
			1555268_a_at	1	3.025	0.18	7	2.994	0.36	40	d	0.830749
FOXG1	9	forkhead box G1	206018_at	9	2.343	0.03	7	2.819	1.12	40	u	0.011393
			207658_s_at	1	4.281	0.28	7	4.29	0.39	40	u	0.953584
CACNA1C	9	calcium channel, voltage-dependent, L type, alpha 1C subunit	211592_s_at	1	3.64	0.38	7	3.629	0.78	40	d	0.970798
			242973_at	0	4.186	0.45	7	3.847	0.32	40	d	0.023519
			208020_s_at	0	2.584	0.23	7	2.706	0.35	40	u	0.389067
			238636_at	0	4.186	0.53	7	3.416	0.62	40	d	0.004145
PAM	9	peptidylglycine alpha-amidating monooxygenase	202336_s_at	5	10.66	0.51	7	9.955	1.14	40	d	0.12562
			214620_x_at	5	8.571	0.5	7	8.307	0.85	40	d	0.43919
			212958_x_at	5	9.58	0.57	7	9.125	1.08	40	d	0.292566
KIAA0831	9	KIAA0831	204568_at	9	8.496	0.3	7	7.333	0.44	40	d	0
KIAA0240	9	KIAA0240	213208_at	9	8.321	0.56	7	7.563	0.65	40	d	0.007029
			38892_at	9	8.146	0.36	7	7.838	0.73	40	d	0.290786
CXXC6	9	CXXC finger 6	228906_at	9	3.215	0.66	7	4.357	1.38	40	u	0.040998
BIRC6	9	baculoviral IAP repeat-containing 6 (apollon)	224635_s_at	9	9.453	0.16	7	9.157	0.69	40	d	0.026088
			233093_s_at	9	8.74	0.22	7	8.619	0.76	40	d	0.430998
DDX3Y	9	DEAD (Asp-Glu-Ala-Asp) box polypeptide 3, Y-linked	205000_at	9	2.446	0.1	7	2.575	0.32	40	u	0.05894
			205001_s_at	2	2.839	0.14	7	2.97	0.28	40	u	0.236523
			1570360_s_at	0	2.023	0.02	7	2.073	0.05	40	u	0.000047
			1570359_at	0	2.867	0.09	7	3.011	0.55	40	u	0.138097
NAP1L5	9	nucleosome assembly protein 1-like 5	228063_s_at	9	7.309	0.25	7	5.888	1	40	d	0
			228062_at	9	6.471	0.53	7	4.84	1.29	40	d	0.002343
SLITRK3	9	SLIT and NTRK-like family, member 3	206732_at	9	1.958	0.03	7	2.05	0.15	40	u	0.001761
EIF3J	9	eukaryotic translation initiation factor 3, subunit J	208985_s_at	9	8.961	0.2	7	9.277	0.56	40	u	0.01573
			208264_s_at	9	7.492	0.29	7	7.689	0.55	40	u	0.367668
			217364_x_at	0	4.284	0.54	7	4.927	0.65	40	u	0.020346
KLHL18	9	kelch-like 18 (Drosophila)	212882_at	9	5.968	0.34	7	5.936	0.54	40	d	0.881628
			1557165_s_at	0	2.113	0.14	7	2.768	0.94	40	u	0.000204
			207976_at	0	3.417	0.07	7	3.489	0.16	40	u	0.262483
SLC6A8	9	solute carrier family 6 (neurotransmitter transporter, creatine), member 8	202219_at	9	7.651	0.33	7	7.9	1.1	40	u	0.270829
			213843_x_at	2	5.958	0.39	7	7.349	1.09	40	u	0.000004
			210854_x_at	2	5.836	0.72	7	7.324	1.03	40	u	0.000852
MAP3K12	9	mitogen-activated protein kinase kinase kinase 12	205447_s_at	9	4.842	0.69	7	4.361	1.27	40	d	0.343393
			205448_s_at	9	5.575	0.56	7	5.157	1.07	40	d	0.32779
SOX6	9	SRY (sex determining region Y)-box 6	223865_at	9	2.154	0.03	7	2.465	0.65	40	u	0.004987
			235526_at	0	7.209	0.3	7	6.771	0.85	40	d	0.023928
			1563454_at	0	2.413	0.16	7	2.702	0.7	40	u	0.030383
			1570486_at	0	3.62	0.25	7	3.934	0.51	40	u	0.124315
			224178_s_at	0	2.33	0.12	7	2.559	0.47	40	u	0.015297
ZCCHC2	9	zinc finger, CCHC domain containing 2	219062_s_at	9	7.23	0.23	7	7.321	1.12	40	u	0.655399
			222816_s_at	9	6.107	0.81	7	7.227	1.38	40	u	0.047263
			244034_at	0	2.325	0.24	7	2.521	0.96	40	u	0.285453
			224503_s_at	0	3.989	0.26	7	4.195	0.79	40	u	0.223091
			233425_at	0	3.966	0.62	7	4	1.17	40	u	0.942333
ZMYM2	9	zinc finger, MYM-type 2	202778_s_at	9	8.949	0.26	7	8.828	0.64	40	d	0.631141
			210282_at	0	4.257	0.49	7	4.552	0.8	40	u	0.362409
			210281_s_at	0	5.727	0.38	7	5.875	0.76	40	u	0.624217
SV2A	9	synaptic vesicle glycoprotein 2A	203069_at	9	4.795	0.47	7	3.998	1.18	40	d	0.090808
ZHX1	9	zinc fingers and homeoboxes 1	223213_s_at	0	8.874	0.2	7	8.772	0.96	40	d	0.559501
			223214_s_at	0	7.785	0.41	7	7.703	0.98	40	d	0.832041
HNRPF	9	heterogeneous nuclear ribonucleoprotein F	201376_s_at	9	9.407	0.43	7	9.479	0.87	40	u	0.834182
RSF1	9	remodeling and spacing factor 1	222540_s_at	9	8.575	0.3	7	9.466	1	40	u	0.000116
			222541_at	9	6.841	0.34	7	7.356	0.98	40	u	0.021479
			218166_s_at	9	6.203	0.34	7	7.054	1.28	40	u	0.001538
			223818_s_at	0	5.528	0.33	7	6.706	1.33	40	u	0.000039
HNRNPU	9	heterogeneous nuclear ribonucleoprotein U (scaffold attachment factor A)	221639_x_at	7	3.834	0.34	7	3.917	0.37	40	u	0.594236
			200594_x_at	7	11.88	0.2	7	11.79	0.51	40	d	0.647149
			200593_s_at	1	10.69	0.18	7	10.53	0.59	40	d	0.182595
			236244_at	0	3.118	0.61	7	3.685	1.2	40	u	0.236845

		(scaffold attachment factor A)	235603_at	0	5.156	0.46	7	4.771	0.93	40	d	0.297085
			216855_s_at	0	5.712	0.73	7	6.042	1.18	40	u	0.4856
			225805_at	0	7.562	0.18	7	7.607	0.94	40	u	0.788363
COL12A1	9	collagen, type XII, alpha 1	225664_at	9	9.695	0.35	7	9.762	1.74	40	u	0.831441
			231879_at	3	6.381	0.45	7	6.666	1.69	40	u	0.387681
			231766_s_at	1	6.408	0.55	7	7.092	1.84	40	u	0.074188
			233109_at	0	4.886	0.73	7	4.9	1.05	40	u	0.973838
			234951_s_at	0	2.685	0.27	7	3.349	0.63	40	u	0.009857
BTAF1	9	BTAF1 RNA polymerase II, B-TFIID transcription factor-associated, 170kDa (Mot1 homolog, S. cerevisiae)	209430_at	9	8.939	0.32	7	8.114	0.63	40	d	0.001906
MINK1	9	misshapen-like kinase 1 (zebrafish)	215909_x_at	10	6.909	0.34	7	7.085	0.49	40	u	0.374697
			214625_s_at	10	4.099	0.86	7	4.6	0.72	40	u	0.112909
			209241_x_at	9	6.662	0.34	7	6.156	0.65	40	d	0.054653
			214246_x_at	0	9.51	0.39	7	8.527	0.68	40	d	0.00071
SCML2	9	sex comb on midleg-like 2 (Drosophila)	206147_x_at	9	7.43	0.23	7	7.323	0.48	40	d	0.575398
ENAH	9	enabled homolog (Drosophila)	222433_at	9	10.36	0.34	7	11.32	0.65	40	u	0.000558
			222434_at	9	6.714	0.59	7	6.991	0.69	40	u	0.334248
			228310_at	9	8.088	0.55	7	7.698	0.88	40	d	0.274403
			217820_s_at	5	9.718	0.42	7	10.52	0.72	40	u	0.00775
			228553_at	0	2.354	0.08	7	2.623	0.36	40	u	0.000214
			1553672_at	0	4.009	0.14	7	4.558	0.63	40	u	0.000028
SLC12A2	9	solute carrier family 12 (sodium/potassium/chloride transporters),	225835_at	9	9.754	0.89	7	8.366	1.66	40	d	0.040508
			204404_at	1	8.675	1	7	7.909	1.31	40	d	0.156861
MAPK14	9	mitogen-activated protein kinase 14	202530_at	9	8.524	0.18	7	8.443	0.53	40	d	0.474805
			211561_x_at	3	5.456	0.32	7	6.168	1.33	40	u	0.007062
			210449_x_at	2	5.27	0.44	7	5.969	1.48	40	u	0.025308
			211087_x_at	0	3.995	0.25	7	4.507	0.95	40	u	0.00885
FZD7	9	frizzled homolog 7 (Drosophila)	203705_s_at	9	9.381	0.38	7	7.788	1.8	40	d	0.000017
			203706_s_at	9	10.36	0.46	7	8.414	1.56	40	d	0.000001
SLC38A2	9	solute carrier family 38, member 2	218041_x_at	9	11.36	0.14	7	10.5	0.89	40	d	0.000001
			222982_x_at	9	11.89	0.12	7	11.27	0.67	40	d	0.000003
			220924_s_at	9	11.71	0.15	7	10.83	0.91	40	d	0.000002
C12orf30	9	chromosome 12 open reading frame 30	225888_at	9	7.045	0.31	7	7.473	0.54	40	u	0.052961
			236816_at	0	6.839	0.49	7	6.567	0.86	40	d	0.428629
			227245_at	0	6.941	0.45	7	7.713	0.52	40	u	0.000811
NPAS4	9	neuronal PAS domain protein 4	1554299_at	9	2.106	0.04	7	2.152	0.05	40	u	0.019261
			240794_at	7	2.019	0.02	7	2.051	0.03	40	u	0.007632
TGFBR1	9	transforming growth factor, beta receptor I (activin A receptor type II-like kinase, 53kDa)	224793_s_at	9	9.879	0.63	7	9.913	0.77	40	u	0.914449
			206943_at	2	2.216	0.03	7	2.678	0.79	40	u	0.000788
			236561_at	0	7.75	0.49	7	6.358	1.52	40	d	0.00015
SFRS8	9	splicing factor, arginine/serine-rich 8 (suppressor-of-white-apricot homolog, Drosophila)	202774_s_at	3	8.58	0.33	7	8.247	0.43	40	d	0.064084
			202775_s_at	3	7.603	0.41	7	6.973	0.65	40	d	0.018823
			202773_s_at	3	6.236	0.29	7	6.345	0.43	40	u	0.534387
			240078_at	0	2.482	0.39	7	2.76	0.66	40	u	0.293064
KIF3B	9	kinesin family member 3B	225205_at	9	8.587	0.25	7	8.606	0.53	40	u	0.928726
			203943_at	2	8.439	0.28	7	8.469	0.6	40	u	0.897342
FOXP4	9	forkhead box P4	227120_at	9	6.728	0.25	7	7.762	1.06	40	u	0.000007
			229763_at	9	5.668	0.38	7	5.627	0.83	40	d	0.901117
BTBD3	9	BTB (POZ) domain containing 3	202946_s_at	9	7.909	0.25	7	7.943	0.94	40	u	0.854099
TBL1X	9	transducin (beta)-like 1X-linked	201867_s_at	9	6.126	0.61	7	6.959	1.1	40	u	0.063913
			213400_s_at	9	7.576	0.57	7	7.915	0.95	40	u	0.373119
			201869_s_at	9	5.856	0.46	7	6.276	0.82	40	u	0.20215
			201868_s_at	9	4.622	0.5	7	5.913	1.05	40	u	0.003029
			213401_s_at	9	3.297	0.25	7	3.787	0.69	40	u	0.075492
			1570293_at	0	2.54	0.02	7	2.621	0.21	40	u	0.020099
MOBKL1A	9	MOB1, Mps One Binder kinase activator-like 1A (yeast)	225997_at	9	8.326	0.29	7	8.327	0.69	40	u	0.997071
USP6	9	ubiquitin specific peptidase 6 (Tre-2 oncogene)	206405_x_at	9	6.352	0.32	7	6.651	0.83	40	u	0.364892
			1555063_at	0	4	0.34	7	4.2	0.62	40	u	0.422141
			1555065_x_at	0	3.403	0.25	7	3.674	0.79	40	u	0.109096
			242242_at	0	3.336	0.3	7	3.58	0.37	40	u	0.11864
MAPK6	9	mitogen-activated protein kinase 6	207121_s_at	9	9.448	0.41	7	9.497	0.6	40	u	0.840007
			226933_s_at	5	6.322	0.45	7	4.483	2.26	40	d	0.000047

ID4	9	inhibitor of DNA binding 4, dominant negative helix-loop-helix protein	209293_x_at	2	8.119	0.49	7	6.137	1.91	40	d	0.000004
			209292_at	0	8.787	0.38	7	5.664	2.06	40	d	0
			229386_at	0	3.359	0.26	7	3.366	0.97	40	u	0.96723
			209291_at	0	11.13	0.26	7	8.238	2.28	40	d	0
EIF5	9	eukaryotic translation initiation factor 5	208707_at	9	5.489	1.13	7	4.361	0.75	40	d	0.001877
			208705_s_at	9	11.9	0.27	7	11.34	0.52	40	d	0.008786
			208706_s_at	9	10.14	0.28	7	9.613	0.52	40	d	0.013723
			208290_s_at	2	8.314	0.23	7	8.627	0.99	40	u	0.094678
			208708_x_at	2	9.458	0.27	7	9.72	0.72	40	u	0.354444
SLC1A1	9	solute carrier family 1 (neuronal/epithelial high affinity glutamate transporter, system	213664_at	9	6.617	0.63	7	5.678	2.06	40	d	0.032424
			206396_at	0	2.63	0.24	7	2.968	0.83	40	u	0.04878
CA10	9	carbonic anhydrase X	223550_s_at	9	2.046	0.05	7	2.079	0.12	40	u	0.479794
			220889_s_at	4	2.522	0.1	7	2.684	0.35	40	u	0.023072
CTDSP1	9	CTD (carboxy-terminal domain, RNA polymerase II, polypeptide A) small phosphatase 1	217844_at	9	9.566	0.25	7	8.469	0.67	40	d	0.000135
WDR22	9	WD repeat domain 22	227251_at	9	4.964	0.35	7	4.679	0.57	40	d	0.217775
			224696_s_at	6	7.133	0.3	7	6.791	0.47	40	d	0.074497
			224697_at	6	6.104	0.35	7	5.408	0.7	40	d	0.014877
			224703_at	6	8.694	0.25	7	7.741	0.78	40	d	0.000003
			1554558_at	0	2.887	0.1	7	3.173	0.32	40	u	0.000174
MAP2	9	microtubule-associated protein 2	210015_s_at	1	2.706	0.27	7	3.648	1.23	40	u	0.000159
			225540_at	0	7.054	0.6	7	6.768	2.02	40	d	0.486794
SEMA4G	9	sema domain, immunoglobulin domain (Ig), transmembrane domain (TM) and short	203483_at	11	2.644	0.28	7	2.679	0.31	40	u	0.785606
			219194_at	9	3.222	0.22	7	3.534	0.69	40	u	0.037458
MAP3K7IP3	9	mitogen-activated protein kinase kinase kinase 7 interacting protein 3	1552927_at	9	5.011	0.46	7	5.043	0.73	40	u	0.911907
			1552928_s_at	9	2.446	0.07	7	3.11	0.95	40	u	0.000105
			1558518_at	0	2.711	0.09	7	2.842	0.21	40	u	0.120552
			227357_at	0	8.151	0.43	7	7.905	0.61	40	d	0.324548
SLC12A5	9	solute carrier family 12, (potassium-chloride transporter) member 5	210040_at	9	2.672	0.12	7	3.141	0.69	40	u	0.000312
ZFAND3	9	zinc finger, AN1-type domain 3	222493_s_at	9	5.567	0.75	7	6.691	0.85	40	u	0.002356
			218020_s_at	9	6.948	0.28	7	6.991	0.48	40	u	0.821646
PHF13	9	PHD finger protein 13	225005_at	9	9.103	0.24	7	8.502	0.57	40	d	0.009989
ZMIZ1	9	zinc finger, MIZ-type containing 1	212124_at	9	10.12	0.26	7	9.373	1.28	40	d	0.00244
			232508_at	0	3.648	0.37	7	3.563	0.41	40	d	0.622688
			233060_at	0	3.279	0.44	7	3.329	0.37	40	u	0.760652
C18orf1	9	chromosome 18 open reading frame 1	207996_s_at	9	7.281	0.68	7	6.896	1.54	40	d	0.528303
			209574_s_at	9	6.253	0.96	7	6.934	1.2	40	u	0.170108
			209573_s_at	9	5.832	0.32	7	6.022	0.77	40	u	0.531407
			233138_at	0	3.935	0.51	7	4.047	0.79	40	u	0.723521
MTMR3	9	myotubularin related protein 3	202197_at	9	8.377	0.34	7	7.571	0.59	40	d	0.001366
			226956_at	0	6.191	0.36	7	5.183	0.69	40	d	0.000595
			202198_s_at	0	4.198	0.38	7	4.151	0.47	40	d	0.805912
			211507_s_at	0	3.532	0.47	7	3.662	0.97	40	u	0.734592
KLHL24	9	kelch-like 24 (Drosophila)	226158_at	9	8.138	0.41	7	8.223	1	40	u	0.829752
			206551_x_at	0	8.599	0.25	7	8.272	0.47	40	d	0.08435
			221985_at	0	6.443	0.39	7	7.681	1.1	40	u	0.000029
			242088_at	0	3.171	0.08	7	3.876	0.76	40	u	0.000002
			221986_s_at	0	6.065	0.41	7	7.316	1.15	40	u	0.000035
ACTN4	9	actinin, alpha 4	200601_at	3	11.28	0.48	7	10.82	0.79	40	d	0.15105
ANKRD13B	9	ankyrin repeat domain 13B	227720_at	9	6.135	0.37	7	5.876	0.84	40	d	0.434149
PAPOLA	9	poly(A) polymerase alpha	212718_at	9	10.95	0.12	7	11.05	0.3	40	u	0.422859
			212720_at	9	7.234	0.45	7	8.163	1.03	40	u	0.025711
			228569_at	0	7.938	0.44	7	6.986	0.96	40	d	0.015115
			209388_at	0	7.234	0.26	7	7.635	0.86	40	u	0.028682
			222035_s_at	0	9.508	0.31	7	9.61	1.05	40	u	0.630977
			215374_at	0	4.071	0.63	7	4.277	0.72	40	u	0.490231
PALLD	9	palladin, cytoskeletal associated protein	200907_s_at	9	11.13	0.29	7	10.39	0.87	40	d	0.000394
			200906_s_at	9	8.582	0.55	7	8.392	0.85	40	d	0.578429
			200897_s_at	9	10.68	0.29	7	9.839	0.78	40	d	0.000064
			1557535_at	0	2.905	0.31	7	3.026	0.49	40	u	0.538863
HECTD2	9	HECT domain containing 2	227568_at	9	7.292	0.42	7	6.867	0.85	40	d	0.211384
			238803_at	0	3.388	0.41	7	3.569	0.72	40	u	0.529005

			1565699_at	0	3.269	0.12	7	3.37	0.16	40	u	0.12304
ADAM19	9	ADAM metallopeptidase domain 19 (meltrin beta)	209765_at	9	4.952	0.49	7	5.923	0.84	40	u	0.005737
			221128_at	0	3.399	0.37	7	3.53	0.51	40	u	0.524066
RGL1	9	ral guanine nucleotide dissociation stimulator-like 1	209568_s_at	9	8.988	0.39	7	7.596	0.71	40	d	0.000011
LIF	9	leukemia inhibitory factor (cholinergic differentiation factor)	205266_at	9	7.138	1.31	7	5.956	0.86	40	d	0.004527
OCRL	9	oculocerebrorenal syndrome of Lowe	203446_s_at	9	7.009	0.21	7	6.909	0.85	40	d	0.537261
			208316_s_at	3	5.988	0.36	7	5.854	1.16	40	d	0.574014
ZNF711	9	zinc finger protein 711	228988_at	9	7.7	0.52	7	6.803	1.44	40	d	0.008631
			207781_s_at	0	3.598	1.04	7	4.063	1.22	40	u	0.360293
SETD8	9	SET domain containing (lysine methyltransferase) 8	225118_at	9	6.423	0.33	7	6.558	0.62	40	u	0.587253
			225094_at	9	5.849	0.31	7	6.104	0.66	40	u	0.328906
			220200_s_at	0	6.185	0.6	7	6.525	0.86	40	u	0.33023
CAPN6	9	calpain 6	202966_at	9	4.555	0.8	7	3.417	1.17	40	d	0.019141
			202965_s_at	9	6.744	1.19	7	3.663	2	40	d	0.000348
			217387_at	9	2.293	0.04	7	2.361	0.1	40	u	0.079991
OSBPL3	9	oxysterol binding protein-like 3	209626_s_at	9	5.048	0.43	7	6.261	1.13	40	u	0.008812
			209627_s_at	9	6.316	0.48	7	6.139	0.94	40	d	0.634273
UBE2D3	9	ubiquitin-conjugating enzyme E2D 3 (UBC4/5 homolog, yeast)	200667_at	9	10.66	0.21	7	10.45	0.51	40	d	0.286059
			200668_s_at	4	11.62	0.22	7	11.74	0.5	40	u	0.55526
			223966_at	0	3.305	0.37	7	3.553	0.59	40	u	0.295289
			200669_s_at	0	10.49	0.36	7	10.68	0.75	40	u	0.539376
			240383_at	0	7.984	0.44	7	7.718	0.93	40	d	0.469195
CCND1	9	cyclin D1	208712_at	9	9.108	0.53	7	9.255	2.03	40	u	0.709762
			208711_s_at	0	7.624	0.17	7	8.616	2.04	40	u	0.004969
PLCXD3	9	phosphatidylinositol-specific phospholipase C, X domain containing 3	230081_at	9	3.072	0.38	7	2.813	0.25	40	d	0.029118
			239270_at	2	2.85	0.42	7	2.645	0.09	40	d	0.274853
GRM1	9	glutamate receptor, metabotropic 1	207299_s_at	9	2.299	0.03	7	2.382	0.14	40	u	0.002261
			210939_s_at	0	2.893	0.38	7	2.671	0.17	40	d	0.212225
			210940_s_at	0	3.093	0.22	7	3.012	0.15	40	d	0.227367
LRIG1	9	leucine-rich repeats and immunoglobulin-like domains 1	211596_s_at	9	11.13	0.36	7	9.15	1.31	40	d	0
			238339_x_at	0	3.473	0.87	7	3.765	1.12	40	u	0.525623
			236173_s_at	0	2.15	0.12	7	2.181	0.14	40	u	0.57606
			240140_s_at	0	2.101	0.02	7	2.315	0.55	40	u	0.02038
ITGB8	9	integrin, beta 8	205816_at	9	2.555	0.11	7	3.404	1.16	40	u	0.000063
			242982_x_at	0	4.739	0.59	7	4.933	1.12	40	u	0.663274
			211488_s_at	0	2.963	0.57	7	3.354	0.97	40	u	0.316967
			226189_at	0	8.681	0.49	7	8.057	1.69	40	d	0.072265
PRKACB	9	protein kinase, cAMP-dependent, catalytic, beta	202741_at	9	9.761	0.46	7	8.868	0.99	40	d	0.026724
			202742_s_at	7	6.817	0.86	7	6.628	1.37	40	d	0.73184
			235780_at	0	3.232	0.29	7	3.539	1.02	40	u	0.139583
DOCK4	9	dedicator of cytokinesis 4	205003_at	9	7.4	0.55	7	6.931	0.76	40	d	0.131486
			244840_x_at	0	7.661	0.36	7	6.591	0.8	40	d	0.001424
			241313_at	0	1.823	0.02	7	1.866	0.05	40	u	0.019671
			1558691_a_at	0	3.727	0.52	7	3.588	0.7	40	d	0.626505
FIGN	9	fidgetin	222956_at	9	2.234	0.06	7	2.624	0.86	40	u	0.008272
			242828_at	0	3.344	0.58	7	3.26	1.05	40	d	0.841744
YES1	9	v-yes-1 Yamaguchi sarcoma viral oncogene homolog 1	202932_at	9	8.702	0.37	7	9.087	1.27	40	u	0.139681
			202933_s_at	9	9.74	0.22	7	10.29	1.14	40	u	0.010148
			210917_at	0	2.853	0.07	7	2.9	0.14	40	u	0.392164
C1orf9	9	chromosome 1 open reading frame 9	203429_s_at	9	9.128	0.3	7	9.943	0.63	40	u	0.002064
GABRA1	9	gamma-aminobutyric acid (GABA) A receptor, alpha 1	244118_at	9	2.664	0.15	7	2.816	0.18	40	u	0.041863
			206678_at	1	2.247	0.03	7	2.333	0.16	40	u	0.005211
HAS3	9	hyaluronan synthase 3	223541_at	9	6.9	1.09	7	3.398	0.47	40	d	0.00024
			228179_at	8	2.024	0.05	7	2.167	0.38	40	u	0.030209
			228178_s_at	8	2.68	0.37	7	2.759	0.48	40	u	0.685048
			1552980_at	8	3.443	0.57	7	3.202	0.52	40	d	0.279924
POLS	9	polymerase (DNA directed) sigma	202466_at	9	8.682	0.23	7	8.355	0.68	40	d	0.030076
IGF2BP2	9	insulin-like growth factor 2 mRNA binding protein 2	218847_at	8	5.915	0.35	7	6.285	1.61	40	u	0.216274
			223963_s_at	4	2.363	0.06	7	2.68	0.72	40	u	0.010187
MAML1	9	mastermind-like 1 (Drosophila)	202360_at	9	8.529	0.29	7	8.088	0.55	40	d	0.047072
TFAP4	9	transcription factor AP-4 (activating enhancer binding protein 4)	205688_at	9	6.634	0.35	7	6.984	0.49	40	u	0.084311

CPSF6	9	cleavage and polyadenylation specific factor 6, 68kDa	202470_s_at	9	5.632	0.55	7	6.096	0.63	40	u	0.081867
			202469_s_at	9	8.49	0.32	7	9.059	0.45	40	u	0.002834
BLCAP	9	bladder cancer associated protein	201032_at	9	10.19	0.25	7	9.169	0.69	40	d	0.000001
CBX7	9	chromobox homolog 7	212914_at	9	9.806	0.5	7	7.076	0.92	40	d	0
MED13	9	mediator complex subunit 13	201986_at	9	8.989	0.17	7	8.782	0.88	40	d	0.194979
			201987_at	9	9.789	0.27	7	9.998	0.71	40	u	0.453146
			223780_s_at	0	3.752	0.44	7	4.877	1.38	40	u	0.000519
			244611_at	0	3.091	0.14	7	4	1.03	40	u	0.000005
CAMSAP1L1	9	calmodulin regulated spectrin-associated protein 1-like 1	212763_at	9	4.95	0.42	7	6.568	0.68	40	u	0
			212765_at	9	8.966	0.23	7	9.007	0.74	40	u	0.79038
			217196_s_at	7	7.825	0.28	7	8.162	0.87	40	u	0.073199
GRM7	9	glutamate receptor, metabotropic 7	207548_at	9	2.519	0.13	7	2.649	0.19	40	u	0.099329
			241049_at	0	2.281	0.19	7	2.277	0.19	40	d	0.960792
			217008_s_at	0	3.731	0.22	7	3.536	0.33	40	d	0.146191
ZFAND5	9	zinc finger, AN1-type domain 5	217741_s_at	9	9.899	0.44	7	9.75	0.93	40	d	0.685097
			210275_s_at	9	11.68	0.04	7	11.12	0.5	40	d	0
			228317_at	0	2.181	0.05	7	2.224	0.07	40	u	0.129443
			229368_s_at	0	2.796	0.38	7	2.814	0.41	40	u	0.916871
ZCCHC11	9	zinc finger, CCHC domain containing 11	212704_at	9	7.5	0.28	7	7.107	0.8	40	d	0.03145
			217594_at	0	3.587	0.26	7	3.814	0.58	40	u	0.32059
SPRY4	9	sprouty homolog 4 (Drosophila)	220983_s_at	9	4.523	0.25	7	4.607	0.49	40	u	0.664873
			221489_s_at	9	7.581	0.38	7	6.625	1.16	40	d	0.0005
GABBR2	9	gamma-aminobutyric acid (GABA) B receptor, 2	209990_s_at	9	2.125	0.03	7	3.663	2.14	40	u	0.000061
			209991_x_at	0	2.681	0.16	7	3.163	0.93	40	u	0.004783
			211679_x_at	0	2.614	0.16	7	3.302	1.24	40	u	0.001947
			217077_s_at	0	3.794	0.57	7	4.711	1.29	40	u	0.076626
HOXC8	9	homeobox C8	221350_at	0	4.897	0.48	7	5.407	0.71	40	u	0.078184
APC	9	adenomatosis polyposis coli	203527_s_at	9	4.341	0.16	7	4.275	0.34	40	d	0.630756
			203525_s_at	9	6.535	0.4	7	6.056	0.72	40	d	0.101166
			203526_s_at	5	5.335	0.41	7	5.026	0.71	40	d	0.279164
			216933_x_at	0	2.924	0.16	7	3.561	0.74	40	u	0.000031
			215310_at	0	3.99	0.49	7	3.965	0.63	40	d	0.922095
INSIG1	9	insulin induced gene 1	201625_s_at	9	6.474	0.58	7	7.149	0.95	40	u	0.08171
			201626_at	9	7.829	0.62	7	8.115	1.08	40	u	0.507274
			201627_s_at	6	7.562	0.59	7	7.762	1.04	40	u	0.63202
BRWD1	9	bromodomain and WD repeat domain containing 1	214820_at	9	5.575	0.48	7	6.396	0.64	40	u	0.002693
			231860_at	9	5.002	0.43	7	5.845	0.82	40	u	0.012506
			1553227_s_at	0	6.604	0.31	7	6.049	0.8	40	d	0.082026
			244622_at	0	5.028	0.32	7	5.589	0.54	40	u	0.0123
			231960_at	0	5.832	0.39	7	5.932	0.76	40	u	0.738331
			225446_at	0	6.653	0.27	7	6.771	0.96	40	u	0.539306
			219280_at	0	6.36	0.35	7	6.871	0.65	40	u	0.053313
ADAMTS18	9	ADAM metallopeptidase with thrombospondin type 1 motif, 18	1553234_at	0	3.375	0.48	7	3.237	0.39	40	d	0.417942
			242823_at	0	2.289	0.05	7	2.336	0.06	40	u	0.060274
			230040_at	0	2.933	0.56	7	3.029	0.73	40	u	0.748343
EXOC5	9	exocyst complex component 5	228418_at	9	5.675	0.48	7	6.061	0.93	40	u	0.299439
			225084_at	5	8.963	0.26	7	9.725	0.51	40	u	0.000455
			222726_s_at	2	6.151	0.38	7	6.653	0.86	40	u	0.144318
			218748_s_at	0	6.231	0.62	7	7.246	1.6	40	u	0.112197
PAIP2	9	poly(A) binding protein interacting protein 2	222984_at	9	11.16	0.16	7	10.92	0.65	40	d	0.056785
			222983_s_at	4	10.5	0.23	7	10.18	0.89	40	d	0.071782
			1560065_at	0	2.627	0.17	7	2.746	0.34	40	u	0.382517
SOCS6	9	suppressor of cytokine signaling 6	206020_at	9	5.708	0.56	7	6.312	0.84	40	u	0.079847
			214462_at	3	4.187	0.86	7	4.815	1.14	40	u	0.179244
			227542_at	0	7.875	0.47	7	7.736	0.87	40	d	0.691145
MYH9	9	myosin, heavy chain 9, non-muscle	211926_s_at	9	9.811	0.4	7	8.877	0.88	40	d	0.00952
RAI14	9	retinoic acid induced 14	202052_s_at	9	8.017	0.57	7	8.709	0.86	40	u	0.051378
NPAT	9	nuclear protein, ataxia-telangiectasia locus	209798_at	9	7.376	0.42	7	7.145	0.66	40	d	0.385856
			211585_at	0	2.68	0.21	7	2.964	0.58	40	u	0.034861
			211584_s_at	0	5.458	0.25	7	5.975	0.59	40	u	0.031
KRAS	9	v-Ki-ras2 Kirsten rat sarcoma viral oncogene homolog	204010_s_at	11	4.956	0.25	7	5.078	0.44	40	u	0.492511
			204009_s_at	11	8.858	0.32	7	9.641	0.67	40	u	0.004636
			214352_s_at	1	9.219	0.33	7	10.12	0.71	40	u	0.002236
			1559204_x_at	0	4.658	0.53	7	5.366	0.63	40	u	0.008984

			1559203_s_at	0	3.329	0.26	7	3.583	0.41	40	u	0.125564
SYCP1	9	synaptonemal complex protein 1	206740_x_at	0	2.429	0.06	7	2.696	0.81	40	u	0.04907
			216917_s_at	0	3.323	0.47	7	3.361	0.83	40	u	0.907656
PDPK1	9	3-phosphoinositide dependent protein kinase-1	224986_s_at	3	9.079	0.32	7	8.648	0.7	40	d	0.12698
			32029_at	1	7.672	0.23	7	7.492	0.34	40	d	0.198619
			204524_at	0	8.161	0.45	7	7.641	0.78	40	d	0.099548
CNNM4	9	cyclin M4	218900_at	9	5.729	0.8	7	5.552	0.94	40	d	0.648508
SPEN	9	spen homolog, transcriptional regulator (Drosophila)	201996_s_at	9	6.734	0.66	7	7.421	1.15	40	u	0.138382
			201997_s_at	9	8.692	0.45	7	7.579	0.93	40	d	0.003876
			1556059_s_at	5	7.632	0.45	7	6.305	1.13	40	d	0.004317
			1556058_s_at	5	4.795	0.47	7	4.149	0.65	40	d	0.017216
GALNT7	9	UDP-N-acetyl-alpha-D-galactosamine:polypeptide N-	222587_s_at	9	7.886	0.54	7	8.055	1.73	40	u	0.637753
			218313_s_at	9	9.944	0.58	7	9.371	1.45	40	d	0.318355
RALGDS	9	ral guanine nucleotide dissociation stimulator	209050_s_at	9	9.195	0.55	7	8.487	0.56	40	d	0.004267
			209051_s_at	4	7.945	0.62	7	7.238	1.1	40	d	0.113023
JOSD1	9	Josephin domain containing 1	201751_at	9	8.671	0.21	7	8.502	0.61	40	d	0.20248
INHBB	9	inhibin, beta B	205258_at	9	9.339	0.57	7	6.41	1.81	40	d	0
LIN28	9	lin-28 homolog (C. elegans)	219823_at	9	2.935	0.15	7	3.391	0.85	40	u	0.004055
SIAH1	9	seven in absentia homolog 1 (Drosophila)	202981_x_at	9	8.956	0.36	7	8.93	0.49	40	d	0.897351
			202980_s_at	9	6.857	0.21	7	7.09	0.68	40	u	0.107079
			232365_at	0	2.841	0.27	7	2.973	0.41	40	u	0.420873
CPNE8	9	copine VIII	228365_at	9	4.351	0.5	7	4.484	0.96	40	u	0.728552
			243727_at	0	2.758	0.23	7	2.947	0.56	40	u	0.394203
			241706_at	0	5.117	0.22	7	5.513	0.84	40	u	0.019581
BPTF	9	bromodomain PHD finger transcription factor	230056_at	9	3.542	0.34	7	3.712	0.48	40	u	0.379683
			209271_at	4	7.984	0.21	7	7.431	0.8	40	d	0.00111
			232909_s_at	0	8.873	0.37	7	8.589	0.73	40	d	0.328456
			207186_s_at	0	8.562	0.36	7	8.787	0.83	40	u	0.496982
			231953_at	0	3.311	0.67	7	3.92	1.1	40	u	0.171615
NFIB	9	nuclear factor I/B	209289_at	9	11.49	0.4	7	9.851	1.58	40	d	0.000003
			209290_s_at	7	12.2	0.34	7	10.72	1.5	40	d	0.000004
			213029_at	0	11.54	0.4	7	9.404	1.53	40	d	0
			213033_s_at	0	8.592	0.45	7	6.721	1.46	40	d	0.000001
			230291_s_at	0	9.794	0.69	7	8.296	1.74	40	d	0.033434
			233304_at	0	4.85	1.23	7	3.374	1.09	40	d	0.002728
			211466_at	0	7.04	0.31	7	6.403	1.83	40	d	0.052175
			233394_at	0	2.146	0.07	7	2.241	0.42	40	u	0.199899
			211467_s_at	0	9.291	0.37	7	7.282	2.42	40	d	0.000017
			213032_at	0	10.51	0.4	7	8.837	1.58	40	d	0.000003
CALU	9	calumenin	200755_s_at	9	8.434	0.28	7	9.57	0.91	40	u	0.000001
			200757_s_at	9	9.153	0.32	7	10.05	0.68	40	u	0.001594
			200756_x_at	0	7.522	0.45	7	8.676	1.31	40	u	0.000358
			238908_at	0	6.192	0.39	7	6.544	0.61	40	u	0.15436
			214845_s_at	0	8.093	0.39	7	9.272	1.49	40	u	0.00023
FNDC5	9	fibronectin type III domain containing 5	226096_at	9	3.649	0.71	7	3.248	0.52	40	d	0.092557
			226097_at	9	3.285	0.4	7	3.098	0.39	40	d	0.260817
			230646_at	0	2.767	0.2	7	2.812	0.23	40	u	0.637626
RPAIN	9	RPA interacting protein	228183_s_at	0	8.395	0.37	7	8.135	0.64	40	d	0.313816
			1564301_a_at	0	2.646	0.21	7	2.779	0.4	40	u	0.402993
			228288_at	0	2.162	0.06	7	2.221	0.19	40	u	0.144634
CADM1	9	cell adhesion molecule 1	209031_at	9	8.04	0.37	7	8.099	1.2	40	u	0.809103
			209030_s_at	9	8.54	0.32	7	8.366	0.94	40	d	0.391908
			209032_s_at	9	6.577	0.38	7	7.115	1.21	40	u	0.039683
			244345_at	0	2.218	0.1	7	2.595	0.73	40	u	0.003714
			232767_at	0	3.302	0.21	7	3.508	0.57	40	u	0.361663
CYP26B1	9	cytochrome P450, family 26, subfamily B, polypeptide 1	219825_at	9	3.642	0.85	7	3.552	1.97	40	d	0.908123
			234721_s_at	0	1.857	0.2	7	1.97	0.33	40	u	0.394048
NCALD	9	neurocalcin delta	211685_s_at	9	8.741	0.82	7	6.439	1.72	40	d	0.001458
			1562790_at	0	2.28	0.02	7	2.356	0.1	40	u	0.000072
PDS5A	9	PDS5, regulator of cohesion maintenance, homolog A (S. cerevisiae)	212140_at	9	8.575	0.22	7	8.686	0.54	40	u	0.606226
			212138_at	9	9.052	0.25	7	9.422	0.53	40	u	0.081001
			213984_at	0	3.549	0.49	7	4.396	0.92	40	u	0.024493
			213983_s_at	0	4.841	0.67	7	5.725	1.08	40	u	0.046882
			217924_at	9	5.374	0.69	7	6.516	1.5	40	u	0.05925

C6orf106	9	chromosome 6 open reading frame 106	217925_s_at	9	7.632	0.23	7	8.058	0.62	40	u	0.004401
			205457_at	0	4.836	0.72	7	6.036	1.27	40	u	0.021954
ARFGEF1	9	ADP-ribosylation factor guanine nucleotide-exchange factor 1(brefeldin A-inhibited)	202956_at	9	9.018	0.29	7	9.259	0.88	40	u	0.200361
			202955_s_at	1	7.231	0.45	7	7.561	1.25	40	u	0.237826
			216266_s_at	0	7.809	0.39	7	8.037	1.64	40	u	0.458552
EYA4	9	eyes absent homolog 4 (Drosophila)	207327_at	9	2.548	0.05	7	2.696	0.46	40	u	0.062708
			238877_at	0	2.825	0.14	7	3.218	0.91	40	u	0.01596
			1561088_at	0	2.167	0.12	7	2.295	0.17	40	u	0.074062
ST8SIA2	9	ST8 alpha-N-acetyl-neuraminide alpha-2,8-sialyltransferase 2	221285_at	0	2.266	0.03	7	2.303	0.03	40	u	0.009747
			239537_at	0	3.889	1.59	7	2.072	0.13	40	d	0.031041
TAPT1	9	transmembrane anterior posterior transformation 1	227407_at	8	8.58	0.29	7	8.009	1.03	40	d	0.008563
			226735_at	5	6.264	0.27	7	5.822	0.94	40	d	0.02424
			216507_at	0	3.868	0.38	7	3.757	0.4	40	d	0.50392
			238798_at	0	2.574	0.17	7	2.522	0.28	40	d	0.638319
			216373_at	0	2.783	0.37	7	2.896	0.4	40	u	0.496699
			242318_at	0	2.566	0.3	7	2.693	0.56	40	u	0.569256
RFX4	9	regulatory factor X, 4 (influences HLA class II expression)	223673_at	9	2.318	0.02	7	2.41	0.14	40	u	0.000336
			1552809_at	0	2.762	0.28	7	2.802	0.56	40	u	0.857492
FMNL2	9	formin-like 2	226184_at	9	9.102	0.33	7	8.905	0.84	40	d	0.550574
			235881_at	0	2.246	0.03	7	2.307	0.06	40	u	0.009615
			242665_at	0	6.298	0.42	7	6.73	1.18	40	u	0.104045
GIT2	9	G protein-coupled receptor kinase interactor 2	225558_at	9	8.521	0.19	7	7.514	0.88	40	d	0
			209876_at	3	4.544	0.24	7	4.844	0.76	40	u	0.06589
			204982_at	0	4.933	0.41	7	5.308	0.54	40	u	0.093332
KIAA0152	9	KIAA0152	200617_at	9	9.817	0.29	7	9.326	0.55	40	d	0.029031
			200616_s_at	0	9.269	0.25	7	10.05	0.74	40	u	0.000031
EIF2C4	9	eukaryotic translation initiation factor 2C, 4	227930_at	9	7.928	0.39	7	7.411	0.68	40	d	0.062047
			222842_at	9	4.682	0.39	7	4.54	0.39	40	d	0.384257
			219190_s_at	9	3.82	0.49	7	3.474	0.55	40	d	0.13526
			1569408_at	0	2.692	0.08	7	2.835	0.15	40	u	0.017372
KCNMA1	8	potassium large conductance calcium-activated channel, subfamily M, alpha member 1	228414_at	8	2.838	0.67	7	2.586	0.41	40	d	0.197351
			221583_s_at	0	3.496	0.41	7	3.904	1.08	40	u	0.339045
			221584_s_at	0	9.364	0.46	7	7.239	1.37	40	d	0
			214921_at	0	2.548	0.48	7	2.242	0.21	40	d	0.177058
ERG	8	v-ets erythroblastosis virus E26 oncogene homolog (avian)	213541_s_at	8	7.128	0.34	7	4.959	0.87	40	d	0
			211626_x_at	8	4.896	0.58	7	4.051	0.63	40	d	0.002136
			241926_s_at	3	5.09	0.58	7	4.474	0.38	40	d	0.000891
TOB2	8	transducer of ERBB2, 2	234720_s_at	8	2.494	0.24	7	2.491	0.33	40	d	0.985012
			222243_s_at	8	7.693	0.2	7	7.688	0.67	40	d	0.9695
			221496_s_at	0	6.464	0.67	7	6.395	0.96	40	d	0.859216
SRPK1	8	SFRS protein kinase 1	202199_s_at	8	5.503	0.53	7	7.089	1.22	40	u	0.001794
			202200_s_at	8	7.688	0.28	7	9.162	0.87	40	u	0
CTDSP2	8	CTD (carboxy-terminal domain, RNA polymerase II, polypeptide A) small	203445_s_at	8	10.08	0.28	7	8.986	0.6	40	d	0.000028
			208735_s_at	3	7.233	0.32	7	7.132	0.69	40	d	0.710953
WHSC1L1	8	Wolf-Hirschhorn syndrome candidate 1-like 1	218173_s_at	8	3.334	0.27	7	4.105	1.32	40	u	0.002279
			222544_s_at	8	9.901	0.25	7	9.814	1.25	40	d	0.699452
			224077_at	7	1.895	0.02	7	1.946	0.06	40	u	0.00081
			224076_s_at	7	7.254	0.39	7	7.364	1.3	40	u	0.676171
			221248_s_at	0	4.479	0.39	7	5.168	1.04	40	u	0.095717
LCOR	8	ligand dependent nuclear receptor corepressor	228454_at	8	8.125	0.32	7	7.945	0.77	40	d	0.556256
KIAA1012	8	KIAA1012	207305_s_at	4	9.672	0.16	7	9.746	0.67	40	u	0.554985
CELSR3	8	cadherin, EGF LAG seven-pass G-type receptor 3 (flamingo homolog, Drosophila)	40020_at	8	4.308	0.43	7	5.3	1.34	40	u	0.001344
			205165_at	8	3.836	0.21	7	4.523	1.2	40	u	0.002172
ITSN1	8	intersectin 1 (SH3 domain protein)	209297_at	8	8.306	0.25	7	7.298	0.55	40	d	0.000029
			209298_s_at	8	6.158	0.78	7	5.103	0.61	40	d	0.000277
			210713_at	8	3.891	0.26	7	4.059	0.32	40	u	0.204145
			35776_at	8	8.835	0.31	7	7.928	0.49	40	d	0.000028
			207322_at	0	2.259	0.05	7	2.327	0.11	40	u	0.110416
LIN28B	8	lin-28 homolog B (C. elegans)	229349_at	0	2.195	0.04	7	2.523	0.55	40	u	0.000742
BTBD14B	8	BTB (POZ) domain containing 14B	227651_at	8	8.562	0.43	7	9.266	0.73	40	u	0.019745
			235047_x_at	0	6.939	0.18	7	6.659	0.42	40	d	0.096586
NEGR1	8	neuronal growth regulator 1	239548_at	8	2.878	0.07	7	3.11	0.29	40	u	0.000126
			229461_x_at	8	4.433	0.74	7	4.452	0.93	40	u	0.959903

NEGR1	8	neuronal growth regulator 1	243357_at	6	3.853	0.67	7	3.686	0.84	40	d	0.627812
			1553194_at	2	2.942	0.37	7	3.181	0.83	40	u	0.468061
SCRT2	8	:ratch homolog 2, zinc finger protein (Drosophi	no probeset available									
PHF12	8	PHD finger protein 12	231815_at	8	7.197	0.38	7	6.661	1.19	40	d	0.036617
			234939_s_at	0	2.376	0.1	7	2.98	0.83	40	u	0.00008
BHLHB3	8	basic helix-loop-helix domain containing, class B, 3	223185_s_at	8	4.386	0.67	7	3.853	0.9	40	d	0.151703
			221530_s_at	8	9.75	0.73	7	7.11	1.41	40	d	0.000021
			231242_at	0	3.54	0.25	7	3.491	0.35	40	d	0.724186
			231243_s_at	0	4.421	1.09	7	3.775	0.93	40	d	0.11298
C6orf120	8	chromosome 6 open reading frame 120	221787_at	8	8.665	0.29	7	7.538	0.66	40	d	0.000084
			221786_at	8	7.824	0.28	7	7.368	0.81	40	d	0.013641
WDR82	8	WD repeat domain 82	201934_at	8	9.713	0.25	7	9.517	0.53	40	d	0.349479
SMURF2	8	SMAD specific E3 ubiquitin protein ligase 2	232020_at	8	5.61	0.61	7	5.533	0.78	40	d	0.810273
			205596_s_at	5	8.308	0.37	7	8.972	0.9	40	u	0.066528
			227489_at	0	7.58	0.42	7	6.841	0.81	40	d	0.025125
CC2D1B	8	coiled-coil and C2 domain containing 1B	236568_at	8	4.987	0.37	7	5.06	0.55	40	u	0.744292
			242487_at	8	2.628	0.14	7	2.66	0.22	40	u	0.723078
			225323_at	8	6.285	0.37	7	6.443	0.64	40	u	0.538645
MMP14	8	matrix metallopeptidase 14 (membrane-inserted)	202827_s_at	8	5.789	0.7	7	7.318	1.38	40	u	0.007491
			202828_s_at	8	7.264	0.27	7	7.992	0.82	40	u	0.00023
			160020_at	8	7.483	0.4	7	8.2	0.73	40	u	0.017459
			217279_x_at	1	5	0.76	7	6.022	1.13	40	u	0.029646
PAPOLG	8	poly(A) polymerase gamma	219160_s_at	8	2.491	0.2	7	2.63	0.41	40	u	0.390715
			222839_s_at	8	6.656	0.17	7	6.825	0.6	40	u	0.168397
			1556929_at	0	3.153	0.42	7	3.78	0.62	40	u	0.015745
			222273_at	0	5.745	0.65	7	5.832	0.8	40	u	0.79116
			224427_s_at	0	5.377	0.3	7	5.675	0.88	40	u	0.123096
NEO1	8	neogenin homolog 1 (chicken)	204321_at	5	6.702	0.28	7	5.894	1.19	40	d	0.000819
			225270_at	0	9.133	0.39	7	7.438	1.21	40	d	0
			229877_at	0	2.22	0.05	7	2.32	0.1	40	u	0.017223
CFL2	8	cofilin 2 (muscle)	224352_s_at	8	7.471	0.39	7	6.484	0.92	40	d	0.008708
			224663_s_at	8	7.868	0.4	7	7.211	1.08	40	d	0.126139
			233496_s_at	1	4.948	0.51	7	4.717	1.02	40	d	0.57026
TCERG1	8	transcription elongation regulator 1	202396_at	8	8.901	0.33	7	8.738	0.6	40	d	0.50078
			229706_at	0	3.463	0.67	7	4.431	1.12	40	u	0.034933
			238838_at	0	3.397	0.27	7	3.822	0.33	40	u	0.002783
UBE2H	8	ubiquitin-conjugating enzyme E2H (UBC8 homolog, yeast)	222420_s_at	8	9.542	0.31	7	9.749	0.85	40	u	0.274345
			222421_at	8	9.205	0.28	7	9.208	0.89	40	u	0.989751
			226681_at	8	2.575	0.45	7	2.672	0.53	40	u	0.656046
			222419_x_at	8	5.527	0.42	7	5.086	0.52	40	d	0.041626
			226637_at	8	3.467	0.29	7	4.443	0.98	40	u	0.000023
			217799_x_at	7	8.999	0.41	7	9.032	1.14	40	u	0.89411
			227950_at	4	2.047	0.11	7	2.206	0.35	40	u	0.036385
			221962_s_at	4	7.653	0.61	7	8.263	1.28	40	u	0.230443
CCNE2	8	cyclin E2	205034_at	8	3.346	0.49	7	7.406	1.47	40	u	0
			211814_s_at	0	2.628	0.22	7	5.45	2.06	40	u	0
PPP1CB	8	protein phosphatase 1, catalytic subunit, beta isoform	201409_s_at	8	10.4	0.49	7	10.43	0.68	40	u	0.925003
			201408_at	8	10.05	0.44	7	9.249	0.87	40	d	0.023626
			201407_s_at	8	8.308	0.65	7	9.1	0.78	40	u	0.016799
			228222_at	6	11.54	0.62	7	11.9	1.07	40	u	0.40472
C5orf30	8	chromosome 5 open reading frame 30	221823_at	8	6.876	0.41	7	6.696	0.9	40	d	0.615229
ZBTB10	8	zinc finger and BTB domain containing 10	219312_s_at	8	5.216	0.46	7	6.267	1.38	40	u	0.001182
			233899_x_at	7	5.643	0.38	7	6.591	1.33	40	u	0.00109
			222863_at	0	4.708	0.46	7	5.209	1.08	40	u	0.243428
			235491_at	0	3.221	0.34	7	3.304	0.47	40	u	0.660515
			235726_at	0	2.794	0.29	7	2.834	0.44	40	u	0.816593
PDZD2	8	PDZ domain containing 2	209493_at	8	7.51	0.63	7	5.738	1.55	40	d	0.005438
			233026_s_at	0	5.64	0.41	7	5.295	0.74	40	d	0.244306
			233025_at	0	3.996	0.76	7	3.89	0.91	40	d	0.777632
ID2	8	inhibitor of DNA binding 2, dominant negative helix-loop-helix protein	213931_at	6	7.15	0.88	7	7.712	1.5	40	u	0.352627
			201566_x_at	6	7.573	0.45	7	7.986	0.97	40	u	0.28457
			201565_s_at	1	10.53	0.39	7	10.93	1.08	40	u	0.103695
NUMB	8	numb homolog (Drosophila)	207545_s_at	8	7.389	0.41	7	7.162	0.45	40	d	0.224806
			209073_s_at	6	7.725	0.21	7	7.201	0.72	40	d	0.000995

NUMB	8	numb homolog (Drosophila)	236930_at	0	3.228	0.21	7	3.344	0.48	40	u	0.542962
			230462_at	0	5.036	0.8	7	4.431	0.71	40	d	0.051517
NMT1	8	N-myristoyltransferase 1	201158_at	8	7.898	0.29	7	7.21	0.62	40	d	0.006792
			201157_s_at	8	8.482	0.27	7	8.403	0.54	40	d	0.711992
			201159_s_at	7	6.235	0.58	7	5.614	0.76	40	d	0.049828
SMARCAD1	8	SWI/SNF-related, matrix-associated actin-dependent regulator of chromatin, subfamily a, containing DEAD/H box 1	223197_s_at	8	8.28	0.43	7	8.302	0.69	40	u	0.936044
FAM46C	8	family with sequence similarity 46, member C	226811_at	8	8.214	0.67	7	7.061	1.57	40	d	0.068073
			220306_at	3	5.731	0.46	7	5.424	1.52	40	d	0.324221
SEPT11	8	septin 11	201307_at	8	8.793	0.42	7	9.005	0.57	40	u	0.365178
			201308_s_at	1	5.193	0.69	7	6.065	0.95	40	u	0.027475
			230071_at	0	5.616	0.35	7	5.369	0.46	40	d	0.188584
			214293_at	0	5.288	0.28	7	5.429	0.55	40	u	0.520688
UNC84B	8	unc-84 homolog B (C. elegans)	212144_at	8	9.075	0.33	7	8.132	0.66	40	d	0.00076
FOXF2	8	forkhead box F2	206377_at	8	4.756	0.66	7	4.296	0.89	40	d	0.208491
FZD4	8	frizzled homolog 4 (Drosophila)	218665_at	8	8.128	0.49	7	6.312	1.06	40	d	0.000075
			224337_s_at	2	3.832	0.3	7	3.879	0.3	40	u	0.711668
XKR4	8	XK, Kell blood group complex subunit-related family, member 4	237802_at	0	2.345	0.03	7	2.568	0.49	40	u	0.008276
C20orf39	8	chromosome 20 open reading frame 39	219310_at	8	3.609	0.69	7	5.018	0.99	40	u	0.000925
CDC25A	8	cell division cycle 25 homolog A (S. pombe)	204695_at	8	5.222	0.48	7	6.292	1.16	40	u	0.023339
			1555772_a_at	2	2.476	0.11	7	3.423	1.09	40	u	0.000005
			204696_s_at	1	3.206	0.29	7	3.668	0.56	40	u	0.043482
ATRX	8	alpha thalassemia/mental retardation syndrome X-linked (RAD54 homolog, S. cerevisiae)	208859_s_at	8	8.656	0.26	7	9.128	0.66	40	u	0.072884
			208861_s_at	8	9.323	0.23	7	8.85	0.6	40	d	0.048273
			208860_s_at	6	8.993	0.39	7	8.432	0.66	40	d	0.037518
			211022_s_at	0	4.449	0.31	7	5.333	1.04	40	u	0.000222
POU4F2	8	POU class 4 homeobox 2	207725_at	8	2.025	0.04	7	2.136	0.31	40	u	0.036326
PTPN12	8	protein tyrosine phosphatase, non-receptor type 12	202006_at	7	8.49	0.32	7	8.75	0.79	40	u	0.4031
			216884_at	0	2.353	0.07	7	2.737	0.63	40	u	0.000708
			216915_s_at	0	6.809	0.33	7	7.507	1.04	40	u	0.00313
DIP2C	8	DIP2 disco-interacting protein 2 homolog C (Drosophila)	212503_s_at	8	7.739	0.35	7	7.561	0.85	40	d	0.595141
			212504_at	8	7.326	0.29	7	7.059	0.79	40	d	0.390804
			1565680_at	3	2.251	0.02	7	2.286	0.05	40	u	0.00293
			1565681_s_at	3	6.143	0.34	7	6.451	1.12	40	u	0.182855
SNIP1	8	Smad nuclear interacting protein 1	219409_at	8	6.278	0.45	7	6.048	0.84	40	d	0.491349
ARHGEF12	8	Rho guanine nucleotide exchange factor (GEF) 12	201335_s_at	8	4.104	0.32	7	3.188	0.4	40	d	0.000001
			201333_s_at	8	4.661	0.3	7	5.563	0.69	40	u	0.001755
			201334_s_at	8	10.17	0.27	7	9.345	0.67	40	d	0.002989
			1559833_at	0	2.866	0.03	7	3.006	0.16	40	u	0.000019
			1566093_at	0	3.32	0.4	7	3.735	0.49	40	u	0.043858
			233620_at	0	2.223	0.05	7	2.299	0.09	40	u	0.045539
			234541_s_at	0	2.22	0.05	7	2.374	0.33	40	u	0.008169
			234544_at	0	3.346	0.37	7	3.309	0.25	40	d	0.740305
			233621_s_at	0	1.892	0.02	7	1.931	0.04	40	u	0.009328
			234542_at	0	2.218	0.03	7	2.338	0.2	40	u	0.001031
			210741_at	0	2.473	0.06	7	2.511	0.06	40	u	0.108972
			234129_at	0	2.966	0.3	7	2.957	0.33	40	d	0.94555
			1570471_at	0	4.449	0.42	7	4.183	0.35	40	d	0.08758
C9orf150	8	chromosome 9 open reading frame 150	227443_at	8	7.123	0.62	7	6.843	1.66	40	d	0.668998
B4GALT1	8	UDP-Gal:betaGlcNAc beta 1,4-galactosyltransferase, polypeptide 1	201883_s_at	8	8.531	0.71	7	8.467	0.73	40	d	0.833698
			201882_x_at	8	3.767	0.37	7	3.545	0.26	40	d	0.060894
			229403_at	0	2.957	0.15	7	3.123	0.25	40	u	0.099806
			211631_x_at	0	4.751	0.59	7	5.104	0.89	40	u	0.32828
			238987_at	0	6.973	0.77	7	5.551	1.06	40	d	0.001753
			216627_s_at	0	3.648	0.84	7	4.133	1.25	40	u	0.3409
EPHA5	8	EPH receptor A5	215664_s_at	1	2.531	0.22	7	2.499	0.15	40	d	0.644205
			216837_at	1	2.273	0.06	7	2.332	0.27	40	u	0.228462
			237939_at	1	2.666	0.05	7	2.851	0.24	40	u	0.000095
DCUN1D3	8	DCN1, defective in cullin neddylation 1, domain containing 3 (S. cerevisiae)	239648_at	8	5.34	0.5	7	4.975	0.74	40	d	0.228052
MYO10	8	myosin X	201976_s_at	8	7.954	0.38	7	8.46	1.37	40	u	0.06937
			1554026_a_at	0	2.696	0.23	7	3.595	1.24	40	u	0.000191

			216222_s_at	0	3.548	0.27	7	4.899	1.49	40	u	0.000006
FLOT2	8	flotillin 2	201350_at	8	8.888	0.16	7	8.436	0.95	40	d	0.008936
			211299_s_at	8	5.917	0.37	7	5.511	1.14	40	d	0.096771
KIAA1217	8	KIAA1217	231807_at	5	8.827	0.41	7	7.285	0.94	40	d	0.000137
			232046_at	0	7.439	1.01	7	5.658	1.15	40	d	0.000475
			1562966_at	0	4.793	0.67	7	4.544	1.23	40	d	0.613764
			1560115_a_at	0	4.395	0.69	7	4.881	1.06	40	u	0.259604
			232762_at	0	8.374	0.72	7	5.973	1.25	40	d	0.000017
			1554438_at	0	5.063	0.53	7	5.081	0.93	40	u	0.962388
RFX1	8	regulatory factor X, 1 (influences HLA class II expression)	226786_at	8	8.447	0.3	7	8.085	0.57	40	d	0.113032
			206321_at	0	2.013	0.15	7	1.992	0.13	40	d	0.706284
ROBO1	8	roundabout, axon guidance receptor, homolog 1 (Drosophila)	213194_at	8	7.764	0.7	7	7.754	1.48	40	d	0.986722
ZBTB7A	8	zinc finger and BTB domain containing 7A	226554_at	8	8.509	0.23	7	8.25	0.65	40	d	0.076857
			219186_at	8	6.066	0.38	7	5.55	0.71	40	d	0.07325
			213299_at	1	2.603	0.11	7	2.7	0.31	40	u	0.166429
			213303_x_at	1	5.762	0.45	7	5.62	0.63	40	d	0.576926
			222082_at	0	2.461	0.23	7	2.449	0.33	40	d	0.92808
			230709_x_at	0	2.427	0.14	7	2.522	0.38	40	u	0.525856
SMEK1	8	SMEK homolog 1, suppressor of mek1 (Dictyostelium)	223160_s_at	8	8.853	0.23	7	8.523	0.56	40	d	0.136425
			220369_at	0	4.115	0.49	7	4.056	0.8	40	d	0.85473
			220368_s_at	0	8.335	0.32	7	8.378	0.66	40	u	0.871069
STYX	8	serine/threonine/tyrosine interacting protein	242408_at	7	3.488	0.3	7	3.978	0.5	40	u	0.01843
			235180_at	5	3.403	0.08	7	3.979	0.59	40	u	0.000001
ZNRF2	8	zinc and ring finger 2	226261_at	8	6.904	0.57	7	7.597	0.69	40	u	0.018454
RNF138	8	ring finger protein 138	239143_x_at	8	5.639	0.36	7	6.188	0.88	40	u	0.120463
			218738_s_at	6	9.21	0.31	7	9.651	0.9	40	u	0.028584
CAMK2G	8	calcium/calmodulin-dependent protein kinase (CaM kinase) II gamma	212757_s_at	8	7.493	0.13	7	7.431	0.51	40	d	0.530037
			212669_at	2	5.648	0.4	7	5.819	0.61	40	u	0.488732
			214322_at	0	3.225	0.38	7	3.117	0.58	40	d	0.640437
PTBP1	8	polypyrimidine tract binding protein 1	226491_x_at	8	6.482	0.11	7	6.546	0.14	40	u	0.277989
			212015_x_at	8	10.62	0.27	7	10.37	0.56	40	d	0.262288
			212016_s_at	8	8.327	0.31	7	8.228	1.32	40	d	0.691392
			211271_x_at	8	10.74	0.25	7	10.41	0.54	40	d	0.123345
			202189_x_at	8	10.67	0.29	7	10.44	0.52	40	d	0.27205
			211270_x_at	8	10.64	0.31	7	10.47	0.53	40	d	0.432777
			216306_x_at	8	10.89	0.28	7	10.59	0.52	40	d	0.156122
			236819_at	0	4.226	0.58	7	3.64	0.38	40	d	0.001662
			231158_x_at	0	5.121	0.38	7	4.956	0.36	40	d	0.281702
PRICKLE2	8	prickle homolog 2 (Drosophila)	225968_at	8	7.847	0.52	7	5.73	1.56	40	d	0.000001
CAPZA1	8	capping protein (actin filament) muscle Z-line, alpha 1	208374_s_at	8	10.82	0.17	7	11.38	0.47	40	u	0.000017
			241275_at	0	2.758	0.16	7	2.983	0.22	40	u	0.015531
			217392_at	0	2.182	0.03	7	2.268	0.14	40	u	0.00112
CUL5	8	cullin 5	203531_at	8	9.462	0.19	7	8.954	0.69	40	d	0.000707
			203533_s_at	8	7.565	0.12	7	7.395	0.52	40	d	0.087746
			230393_at	0	4.675	0.3	7	4.251	0.78	40	d	0.172042
			203532_x_at	0	5.726	0.41	7	6.607	0.68	40	u	0.002082
SNX27	8	sorting nexin family member 27	221498_at	8	7.727	0.11	7	7.878	0.48	40	u	0.096713
			221006_s_at	1	6.713	0.35	7	7.647	0.72	40	u	0.001883
ANK1	8	ankyrin 1, erythrocytic	205389_s_at	8	2.536	0.09	7	2.706	0.37	40	u	0.020499
			205390_s_at	8	2.634	0.03	7	2.86	0.67	40	u	0.0422
			207087_x_at	0	3.129	0.33	7	3.314	0.49	40	u	0.351299
			205391_x_at	0	3.538	0.26	7	3.521	0.56	40	d	0.938058
			240363_at	0	5.618	0.27	7	5.661	0.41	40	u	0.791746
			208353_x_at	0	3.152	0.24	7	3.26	0.56	40	u	0.623248
			208352_x_at	0	2.996	0.47	7	3.306	0.72	40	u	0.287415
WNK3	8	WNK lysine deficient protein kinase 3	232282_at	8	2.607	0.27	7	3.157	1.17	40	u	0.014864
			1555248_a_at	0	2.605	0.14	7	2.89	0.34	40	u	0.038794
SPRY3	8	sprouty homolog 3 (Drosophila)	232157_at	16	2.527	0.18	7	2.473	0.19	40	d	0.498501
MMD	8	monocyte to macrophage differentiation-associated	203414_at	8	8.768	0.88	7	8.669	0.98	40	d	0.807194
			244523_at	0	2.496	0.14	7	2.655	0.28	40	u	0.164417
RAB11A	8	RAB11A, member RAS oncogene family	200863_s_at	8	11.15	0.27	7	11.36	0.63	40	u	0.405497
			200864_s_at	8	8.468	0.29	7	8.696	0.67	40	u	0.391571
		eukaryotic translation initiation factor 4A	200912_s_at	8	13.1	0.03	7	12.62	0.5	40	d	0.000001

EIF4A2	8	eukaryotic translation initiation factor 4A, isoform 2	1556350_a_at	0	2.848	0.08	7	3.06	0.39	40	u	0.004197
			1555996_s_at	0	5.742	0.59	7	5.921	1.64	40	u	0.621908
ANK3	8	ankyrin 3, node of Ranvier (ankyrin G)	206385_s_at	0	9.166	0.31	7	8.455	1.24	40	d	0.004842
			207950_s_at	0	4.029	0.43	7	4.461	1.07	40	u	0.306187
			239726_at	0	6.315	0.41	7	6.046	1.17	40	d	0.296897
			209442_x_at	0	8.558	0.28	7	8.31	1.18	40	d	0.271836
MAP4K4	8	mitogen-activated protein kinase kinase kinase kinase 4	218181_s_at	8	7.653	0.36	7	7.193	0.82	40	d	0.159034
			222548_s_at	8	6.555	0.29	7	6.768	0.42	40	u	0.218698
			222547_at	8	5.154	0.45	7	5.479	0.87	40	u	0.347536
			206571_s_at	1	7.352	0.36	7	7.723	0.67	40	u	0.167386
TAS2R38	8	taste receptor, type 2, member 38	1553555_at	0	4.073	0.25	7	4.172	0.49	40	u	0.612583
YTHDC1	8	YTH domain containing 1	212455_at	8	11.06	0.25	7	10.49	0.46	40	d	0.003075
			239905_at	0	2.361	0.03	7	2.43	0.05	40	u	0.002266
			214814_at	0	6.561	0.43	7	6.544	0.7	40	d	0.950936
			228556_at	0	6.783	0.14	7	6.809	0.49	40	u	0.792212
			240459_at	0	2.67	0.13	7	2.77	0.42	40	u	0.251989
TMEM168	8	transmembrane protein 168	218962_s_at	8	8.914	0.28	7	8.729	0.69	40	d	0.49378
			234726_s_at	1	6.794	0.65	7	6.542	1.19	40	d	0.594349
INPP5A	8	inositol polyphosphate-5-phosphatase, 40kDa	203006_at	8	8.595	0.21	7	8.191	0.64	40	d	0.005475
			1554757_a_at	0	4.868	0.5	7	4.797	1.11	40	d	0.870502
MBOAT2	8	membrane bound O-acyltransferase domain containing 2	226726_at	8	8.199	0.68	7	8.866	0.9	40	u	0.07326
			213288_at	0	8.095	0.72	7	8.431	1.01	40	u	0.415359
ZNRF1	8	zinc and ring finger 1	225962_at	8	3.002	0.31	7	3.163	0.69	40	u	0.555215
			225960_at	8	4.855	0.44	7	4.286	0.41	40	d	0.002027
			225959_s_at	8	6.3	0.36	7	5.917	0.9	40	d	0.281176
			231092_s_at	6	4.218	0.64	7	4.26	0.38	40	u	0.817937
			223382_s_at	2	7.553	0.26	7	7.796	0.73	40	u	0.14029
			223383_at	2	7.69	0.27	7	8.045	0.88	40	u	0.055558
PUM1	8	pumilio homolog 1 (Drosophila)	201165_s_at	8	9.682	0.22	7	9.375	0.62	40	d	0.030331
			201166_s_at	8	10.65	0.3	7	9.984	0.48	40	d	0.001131
			201164_s_at	8	10.21	0.23	7	9.294	0.49	40	d	0.00002
CNTNAP1	8	contactin associated protein 1	219400_at	8	4.939	0.15	7	4.988	0.36	40	u	0.729403
KLHL2	8	kelch-like 2, Mayven (Drosophila)	219157_at	8	9.498	0.29	7	8.144	0.82	40	d	0
IGF1	8	insulin-like growth factor 1 (somatomedin C)	209540_at	8	8.874	0.61	7	5.287	1.43	40	d	0
			209541_at	8	9.527	0.69	7	5.905	1.42	40	d	0
			209542_x_at	3	7.544	0.84	7	5.077	0.89	40	d	0
			211577_s_at	0	7.053	0.61	7	5.131	0.81	40	d	0.000001
UBE2G1	8	ubiquitin-conjugating enzyme E2G 1 (UBC7 homolog, yeast)	209141_at	1	8.639	0.28	7	8.45	0.65	40	d	0.46145
			209142_s_at	1	8.214	0.46	7	8.269	0.95	40	u	0.884176
			226005_at	0	8.602	0.19	7	7.826	0.84	40	d	0.000012
PAIP1	8	poly(A) binding protein interacting protein 1	208051_s_at	8	8.849	0.4	7	9.089	0.76	40	u	0.43073
			209063_x_at	8	8.869	0.21	7	9.429	0.55	40	u	0.013282
			209064_x_at	8	8.888	0.31	7	9.133	0.72	40	u	0.39376
			213754_s_at	7	8.562	0.26	7	9.173	0.58	40	u	0.010001
			210283_x_at	6	8.563	0.24	7	9.295	0.62	40	u	0.004247
SH3BP4	8	SH3-domain binding protein 4	222258_s_at	8	9.925	0.43	7	9.128	0.99	40	d	0.04614
HOXA1	8	homeobox A1	214639_s_at	6	2.952	0.43	7	2.701	0.48	40	d	0.211309
INTS6	8	integrator complex subunit 6	218819_at	8	6.616	0.42	7	7.311	0.68	40	u	0.013177
			1568695_s_at	0	2.829	0.15	7	2.833	0.19	40	u	0.962269
			235283_at	0	3.168	0.19	7	3.416	0.58	40	u	0.05098
			222239_s_at	0	6.74	0.42	7	7.384	0.65	40	u	0.017153
PPP3CA	8	protein phosphatase 3 (formerly 2B), catalytic subunit, alpha isoform	202457_s_at	8	9.024	0.3	7	8.946	0.8	40	d	0.804804
			202429_s_at	8	9.735	0.25	7	9.351	0.69	40	d	0.017875
			202425_x_at	2	8.365	0.13	7	8.627	0.68	40	u	0.037311
SON	8	SON DNA binding protein	201085_s_at	8	7.619	0.47	7	8.643	0.66	40	u	0.000392
			201086_x_at	8	10.46	0.24	7	9.722	0.66	40	d	0.000035
			214988_s_at	8	10.8	0.26	7	10.24	0.64	40	d	0.030574
			213538_at	0	7.199	0.32	7	6.691	0.69	40	d	0.069669
			226465_s_at	0	10.69	0.26	7	10.73	0.57	40	u	0.867517
FAM76B	8	family with sequence similarity 76, member B	226753_at	8	7.87	0.32	7	7.195	1.05	40	d	0.003427
			232048_at	2	5.842	0.68	7	5.87	0.76	40	u	0.92661
			1553750_a_at	0	4.941	0.61	7	5.424	0.91	40	u	0.191828
			1553749_at	0	3.284	0.31	7	3.92	0.86	40	u	0.002468
			205920_at	7	3.382	0.98	7	3.516	1.1	40	u	0.769978

SLC6A6	8	solute carrier family 6 (neurotransmitter transporter, taurine), member 6	205921_s_at	3	2.568	0.08	7	2.86	0.42	40	u	0.000271
			211030_s_at	0	2.512	0.26	7	2.847	0.4	40	u	0.041391
			228754_at	0	7.66	0.56	7	7.364	0.64	40	d	0.267637
			228756_at	0	2.186	0.02	7	2.238	0.04	40	u	0.002743
DLGAP4	8	discs, large (Drosophila) homolog-associated protein 4	202571_s_at	8	4.926	0.52	7	5.375	0.93	40	u	0.228979
			202572_s_at	8	7.137	0.49	7	7.658	0.81	40	u	0.115519
			202570_s_at	8	5.528	0.44	7	6.503	0.83	40	u	0.004782
			233056_x_at	0	8.998	0.38	7	8.758	0.58	40	d	0.308118
			1556701_at	0	2.703	0.45	7	2.515	0.37	40	d	0.250836
			1557394_at	0	5.895	0.44	7	5.817	0.77	40	d	0.800619
KIAA0355	8	KIAA0355	203288_at	8	7.966	0.32	7	6.987	0.65	40	d	0.000384
LSM12	8	LSM12 homolog (S. cerevisiae)	212532_s_at	8	8.637	0.4	7	8.417	0.76	40	d	0.466268
			212529_at	8	6.068	0.71	7	6.686	0.84	40	u	0.077781
WDR20	8	WD repeat domain 20	227693_at	8	5.609	0.36	7	5.122	0.73	40	d	0.099744
			227541_at	0	5.979	0.36	7	5.997	0.71	40	u	0.947272
			1554549_a_at	0	2.955	0.28	7	2.866	0.36	40	d	0.545708
STK40	8	serine/threonine kinase 40	223852_s_at	8	7.546	0.29	7	7.123	0.54	40	d	0.05318
			225015_s_at	8	5.064	0.09	7	5.259	0.32	40	u	0.003972
ACVR2A	8	activin A receptor, type IIA	205327_s_at	8	8.284	0.56	7	7.588	0.55	40	d	0.004122
			228416_at	0	7.734	0.38	7	5.842	1.12	40	d	0
NUAK1	8	NUAK family, SNF1-like kinase, 1	204589_at	8	7.132	0.45	7	7.047	1.48	40	d	0.777844
C8orf13	8	chromosome 8 open reading frame 13	226614_s_at	8	4.591	0.36	7	4.835	0.83	40	u	0.460902
			233641_s_at	8	2.387	0.13	7	2.396	0.18	40	u	0.900924
YWHAH	8	tyrosine 3-monooxygenase/tryptophan 5-monooxygenase activation protein, eta polypeptide	201020_at	5	9.326	0.35	7	9.812	0.9	40	u	0.173841
ITGA6	8	integrin, alpha 6	201656_at	8	8.899	0.56	7	8.212	1.14	40	d	0.133723
			215177_s_at	5	8.742	0.68	7	7.424	1.74	40	d	0.058539
DMXL1	8	Dmx-like 1	203791_at	8	8.218	0.29	7	7.38	0.81	40	d	0.000077
ZIC3	8	Zic family member 3 heterotaxy 1 (odd-paired homolog, Drosophila)	207197_at	7	2.076	0.06	7	2.47	0.74	40	u	0.002398
PTPN9	8	protein tyrosine phosphatase, non-receptor type 9	202958_at	8	7.495	0.32	7	7.291	0.54	40	d	0.344711
			233226_at	0	2.911	0.49	7	3.441	0.68	40	u	0.061074
			230140_at	0	2.509	0.14	7	2.656	0.39	40	u	0.33328
FAM104A	8	family with sequence similarity 104, member A	225319_s_at	8	7.923	0.17	7	7.934	0.58	40	u	0.924196
WWP1	8	WW domain containing E3 ubiquitin protein ligase 1	212638_s_at	8	10.38	0.42	7	10.28	1.18	40	d	0.687388
			212637_s_at	8	7.303	0.52	7	7.692	1.32	40	u	0.456344
FBXO30	8	F-box protein 30	226541_at	8	7.75	0.27	7	7.638	0.74	40	d	0.493825
MKNK2	8	MAP kinase interacting serine/threonine kinase 2	218205_s_at	8	11.09	0.24	7	10.64	0.78	40	d	0.00775
			223199_at	8	9.69	0.26	7	9.005	0.77	40	d	0.000296
SASH1	8	SAM and SH3 domain containing 1	226022_at	8	9.74	0.22	7	7.885	1.03	40	d	0
			213236_at	1	8.484	0.34	7	6.776	0.92	40	d	0.000023
			41644_at	1	8.97	0.29	7	7.395	0.94	40	d	0
USP46	8	ubiquitin specific peptidase 46	203870_at	8	5.558	0.4	7	6.09	0.91	40	u	0.144818
			203869_at	8	5.409	0.29	7	6.03	0.87	40	u	0.002401
NUPL2	8	nucleoporin like 2	204003_s_at	0	6.249	0.22	7	6.534	0.74	40	u	0.063613
EIF3A	8	eukaryotic translation initiation factor 3, subunit A	200597_at	11	8.362	0.29	7	7.255	0.81	40	d	0.000002
			200596_s_at	11	9.91	0.19	7	9.566	0.49	40	d	0.079878
			200595_s_at	8	9.955	0.17	7	9.876	0.54	40	d	0.477732
RPGRIP1L	8	RPGRIP1-like	213959_s_at	8	3.918	0.27	7	4.919	1.09	40	u	0.000025
PTPRF	8	protein tyrosine phosphatase, receptor type, F	200637_s_at	8	9.201	0.49	7	8.88	1.01	40	d	0.424857
			200636_s_at	8	10.92	0.44	7	10.33	0.82	40	d	0.074812
			200635_s_at	8	9.145	0.52	7	9.15	0.92	40	u	0.988834
			215066_at	0	5.556	0.61	7	5.858	1.09	40	u	0.487864
CLIP4	8	CAP-GLY domain containing linker protein family, member 4	226425_at	8	9.164	0.35	7	7.502	1.48	40	d	0.000001
			219944_at	0	5.042	0.62	7	5.02	0.94	40	d	0.954036
UBE2Z	8	ubiquitin-conjugating enzyme E2Z	222395_s_at	8	9.21	0.16	7	9.726	0.86	40	u	0.00149
			217750_s_at	6	9.532	0.19	7	9.571	0.71	40	u	0.779484
			236107_at	0	4.938	0.63	7	5.362	1.17	40	u	0.363626
PPP6C	8	protein phosphatase 6, catalytic subunit	203529_at	8	9.46	0.15	7	9.364	0.54	40	d	0.375321
			206174_s_at	8	8.733	0.24	7	8.825	0.75	40	u	0.558331
SRPR	8	signal recognition particle receptor ('docking protein')	200917_s_at	8	6.412	0.91	7	7.03	1.02	40	u	0.148701
			200918_s_at	8	10.11	0.23	7	9.422	0.67	40	d	0.000061

FAM53B	8	family with sequence similarity 53, member B	203206_at	8	6.954	0.31	7	6.99	0.73	40	u	0.900047
			1554165_at	0	2.781	0.23	7	2.799	0.23	40	u	0.851023
			237297_at	0	2.384	0.08	7	2.457	0.26	40	u	0.192657
NRXN3	8	neurexin 3	229649_at	6	3.666	0.88	7	2.959	0.78	40	d	0.040542
			215020_at	0	2.594	0.24	7	2.542	0.24	40	d	0.603233
			205795_at	0	2.247	0.23	7	2.151	0.45	40	d	0.592446
			215021_s_at	0	2.539	0.03	7	2.559	0.09	40	u	0.307268
SP8	8	Sp8 transcription factor	237449_at	8	2.443	0.04	7	2.557	0.24	40	u	0.008306
			239743_at	0	3.55	0.31	7	3.504	0.25	40	d	0.673273
LARP4	8	La ribonucleoprotein domain family, member 4	212714_at	8	8.749	0.33	7	8.842	0.61	40	u	0.702511
			214155_s_at	6	4.821	0.43	7	6.169	0.9	40	u	0.000409
			238960_s_at	0	4.09	0.46	7	4.547	0.86	40	u	0.186148
			1555384_a_at	0	4.842	0.48	7	6.075	0.99	40	u	0.002905
			238959_at	0	5.261	0.48	7	6.413	0.87	40	u	0.001654
TESK2	8	testis-specific kinase 2	205486_at	8	6.83	0.52	7	5.444	1.12	40	d	0.002939
EPAS1	8	endothelial PAS domain protein 1	200878_at	8	10.74	0.51	7	8.788	1.34	40	d	0.000532
			200879_s_at	0	4.77	0.62	7	4.172	1.3	40	d	0.248647
			241055_at	0	2.418	0.03	7	2.487	0.06	40	u	0.007012
NRK	8	Nik related kinase	227971_at	8	4.323	0.6	7	4.394	1.06	40	u	0.867649
			232771_at	4	2.222	0.02	7	2.33	0.21	40	u	0.003579
AUTS2	8	autism susceptibility candidate 2	212599_at	8	10.66	0.54	7	9.044	1.17	40	d	0.001059
			243364_at	0	2.584	0.23	7	2.651	0.42	40	u	0.688576
			243365_s_at	0	6.356	0.33	7	5.579	0.51	40	d	0.000441
RAB8B	8	RAB8B, member RAS oncogene family	226633_at	8	8.81	0.35	7	8.239	0.97	40	d	0.012709
			222846_at	6	6.371	0.51	7	5.802	1	40	d	0.156191
			219210_s_at	2	5.497	0.24	7	5.898	0.68	40	u	0.011265
C10orf46	8	chromosome 10 open reading frame 46	227137_at	0	2.553	0.1	7	2.799	0.37	40	u	0.001768
			225192_at	0	8.34	0.33	7	8.197	0.45	40	d	0.436552
			227136_s_at	0	2.606	0.04	7	2.94	0.72	40	u	0.006185
			227257_s_at	0	6.897	0.63	7	7.882	1.23	40	u	0.048655
			227258_at	0	5.944	0.41	7	5.956	0.6	40	u	0.961212
TMEM28	8	transmembrane protein 28	206299_at	8	4.037	0.25	7	4.371	0.78	40	u	0.048123
FBN2	8	fibrillin 2 (congenital contractural arachnodactyly)	203184_at	8	3.587	0.66	7	4.682	1.97	40	u	0.01361
			215717_s_at	0	2.595	0.05	7	2.754	0.33	40	u	0.00835
			1554737_at	0	2.167	0.03	7	2.276	0.22	40	u	0.006898
ANKRD15	8	ankyrin repeat domain 15	213005_s_at	8	8.435	0.36	7	7.15	1.34	40	d	0.00002
			1565832_at	0	2.751	0.24	7	3.009	0.43	40	u	0.136524
			216763_at	0	2.491	0.04	7	2.628	0.2	40	u	0.000403
			237162_at	0	2.64	0.03	7	2.683	0.12	40	u	0.064506
			238789_at	0	4.691	0.43	7	4.832	0.69	40	u	0.612966
			216762_at	0	2.835	0.3	7	2.97	0.38	40	u	0.387183
EVI5L	8	ecotropic viral integration site 5-like	226411_at	8	6.336	0.2	7	6.196	0.54	40	d	0.516561
			242534_at	0	2.181	0.06	7	2.246	0.13	40	u	0.196489
			244500_s_at	0	2.746	0.21	7	2.699	0.45	40	d	0.787593
			244501_at	0	2.455	0.03	7	2.522	0.12	40	u	0.006064
CHST1	8	carbohydrate (keratan sulfate Gal-6) sulfotransferase 1	205567_at	8	6.601	1.75	7	6.378	2.01	40	d	0.787989
BTRC	8	beta-transducin repeat containing	224471_s_at	8	7.719	0.35	7	7.027	0.78	40	d	0.02995
			204901_at	6	4.802	0.52	7	5.062	0.58	40	u	0.285027
			1563620_at	0	2.459	0.06	7	2.562	0.08	40	u	0.002558
			222374_at	0	2.667	0.31	7	2.844	0.43	40	u	0.309094
			216091_s_at	0	3.382	0.23	7	3.65	0.64	40	u	0.068911
PIM1	8	pim-1 oncogene	209193_at	8	7.118	0.62	7	7.183	1.17	40	u	0.889359
YY1	8	YY1 transcription factor	200047_s_at	8	10.63	0.3	7	11.07	0.44	40	u	0.018054
			201901_s_at	5	10.02	0.28	7	10.2	0.38	40	u	0.243561
			201902_s_at	5	5.271	0.41	7	4.851	0.55	40	d	0.063705
			213494_s_at	2	5.154	0.58	7	6.038	0.99	40	u	0.029287
			224718_at	0	9.501	0.21	7	8.526	0.54	40	d	0.00003
			224711_at	0	8.8	0.33	7	7.861	0.59	40	d	0.000246
ZNF236	8	zinc finger protein 236	219171_s_at	8	2.919	0.37	7	3.011	0.54	40	u	0.673775
			47571_at	5	6.581	0.39	7	7.401	0.55	40	u	0.000554
			222227_at	0	2.75	0.07	7	2.95	0.2	40	u	0.000069
SNX4	8	sorting nexin 4	212652_s_at	8	8.659	0.36	7	8.776	0.57	40	u	0.611992
			205329_s_at	0	7.381	0.31	7	7.788	0.73	40	u	0.161246

IQWD1	8	IQ motif and WD repeats 1	217908_s_at	8	9.334	0.35	7	9.624	0.57	40	u	0.207483
			224372_at	0	15	0.13	7	14.89	0.36	40	d	0.164411
			241209_at	0	3.477	0.24	7	3.523	0.23	40	u	0.636251
			232349_x_at	0	7.698	0.3	7	8.338	0.62	40	u	0.011469
			224373_s_at	0	14.84	0.13	7	14.79	0.4	40	d	0.488822
TTBK1	8	tau tubulin kinase 1	230191_at	8	4.006	0.2	7	4.206	0.36	40	u	0.17096
			240643_at	0	2.617	0.57	7	2.839	0.56	40	u	0.346875
ZNF217	8	zinc finger protein 217	203739_at	0	8.532	0.44	7	9.04	0.74	40	u	0.089403
ZNF644	8	zinc finger protein 644	222580_at	8	8.991	0.59	7	9.075	0.88	40	u	0.815101
			1553725_s_at	0	5.937	0.81	7	6.479	1.56	40	u	0.384143
			229684_s_at	0	3.9	0.38	7	4.451	0.86	40	u	0.110686
SIN3A	8	SIN3 homolog A, transcription regulator (yeast)	225135_at	8	7.827	0.33	7	6.887	0.84	40	d	0.006296
			238189_at	0	2.321	0.05	7	2.398	0.1	40	u	0.052753
MAN2A1	8	mannosidase, alpha, class 2A, member 1	205105_at	4	9.19	0.34	7	8.253	1.14	40	d	0.000305
			235103_at	0	7	0.52	7	6.588	1.45	40	d	0.202979
			226538_at	0	9.238	0.3	7	8.342	1.04	40	d	0.000145
TJP1	8	tight junction protein 1 (zona occludens 1)	202011_at	8	9.57	0.2	7	8.81	0.54	40	d	0.000845
			214168_s_at	1	6.815	0.28	7	6.089	0.93	40	d	0.000531
PTCH1	8	patched homolog 1 (Drosophila)	209816_at	0	2.217	0.07	7	2.309	0.28	40	u	0.089584
			208522_s_at	0	2.802	0.22	7	2.849	0.22	40	u	0.608313
			209815_at	0	6.349	0.51	7	5.21	1.53	40	d	0.001508
			1555520_at	0	2.358	0.1	7	2.82	0.62	40	u	0.000099
FBN1	8	fibrillin 1	202766_s_at	8	9.476	0.85	7	8.765	1.53	40	d	0.248019
			202765_s_at	8	6.829	0.94	7	6.71	1.33	40	d	0.825694
			235318_at	0	4.929	0.54	7	4.322	0.97	40	d	0.123383
SPIN1	8	spindlin 1	222431_at	7	11.04	0.32	7	10.56	0.49	40	d	0.017584
			217813_s_at	3	7.6	0.3	7	8.165	0.69	40	u	0.043331
SLC7A8	8	solute carrier family 7 (cationic amino acid transporter, y+ system), member 8	202752_x_at	8	8.262	0.49	7	7.015	1.48	40	d	0.000457
			216092_s_at	8	8.629	0.39	7	7.484	1.18	40	d	0.000079
			216604_s_at	0	5.844	0.73	7	5.782	1.18	40	d	0.89608
			216603_at	0	5.26	0.35	7	5.119	1.1	40	d	0.536788
			217248_s_at	0	2.342	0.26	7	2.399	0.41	40	u	0.728261
PBX3	8	pre-B-cell leukemia homeobox 3	204082_at	8	7.434	0.7	7	6.575	1.17	40	d	0.071875
			234145_at	0	2.582	0.16	7	2.835	0.51	40	u	0.021783
PHLDB1	8	pleckstrin homology-like domain, family B, member 1	212134_at	8	8.622	0.32	7	7.652	0.52	40	d	0.000029
			216019_x_at	0	3.107	0.19	7	3.132	0.18	40	u	0.733269
			216102_at	0	2.004	0.02	7	2.049	0.05	40	u	0.014632
CMPK	8	cytidylate kinase	217870_s_at	8	10.56	0.3	7	10.32	0.69	40	d	0.384544
			222448_s_at	8	10.17	0.26	7	10.21	0.8	40	u	0.791472
			1570533_at	0	2.377	0.03	7	2.461	0.11	40	u	0.000814
ZC3H7B	8	zinc finger CCCH-type containing 7B	213323_s_at	8	4.445	0.43	7	4.778	0.46	40	u	0.086237
			205877_s_at	8	6.766	0.35	7	6.625	0.53	40	d	0.510325
			206168_at	0	1.941	0.02	7	1.981	0.05	40	u	0.053738
			206169_x_at	0	7.735	0.33	7	7.561	0.61	40	d	0.476109
			216844_at	0	3.566	0.45	7	3.501	0.39	40	d	0.699953
LRP6	8	low density lipoprotein receptor-related protein 6	34697_at	8	5.721	0.11	7	6.378	1.01	40	u	0.000307
			205606_at	8	7.73	0.19	7	8.089	0.94	40	u	0.038712
			225745_at	0	8.898	0.28	7	8.302	1.19	40	d	0.01119
LRCH2	8	leucine-rich repeats and calponin homology (CH) domain containing 2	227688_at	8	3.857	0.57	7	3.67	0.88	40	d	0.59673
MAGI2	8	membrane associated guanylate kinase, WW and PDZ domain containing 2	209737_at	8	6.938	0.75	7	5.273	1.39	40	d	0.004118
			237786_at	0	2.557	0.25	7	2.494	0.27	40	d	0.569192
			207702_s_at	0	3.227	0.55	7	2.984	0.76	40	d	0.431306
VAMP3	8	vesicle-associated membrane protein 3 (cellubrevin)	211749_s_at	8	9.697	0.49	7	9.035	1.12	40	d	0.140892
			201336_at	8	11.1	0.17	7	10.18	0.54	40	d	0
			201337_s_at	2	8.544	0.63	7	7.795	1.45	40	d	0.19575
DR1	8	down-regulator of transcription 1, TBP-binding (negative cofactor 2)	209187_at	8	8.098	0.28	7	9.026	0.73	40	u	0.002095
			207654_x_at	1	7.421	0.33	7	8.086	1.06	40	u	0.004507
			216653_at	1	2.322	0.1	7	2.401	0.11	40	u	0.093925
			216652_s_at	1	6.56	0.25	7	7.601	1.05	40	u	0.000006
			209188_x_at	1	7.37	0.37	7	8.077	1.02	40	u	0.082298
C14orf4	8	chromosome 14 open reading frame 4	223474_at	8	11.29	0.29	7	10.49	0.75	40	d	0.009361
CRK	8	v-crk sarcoma virus CT10 oncogene homolog (avian)	202224_at	8	8.606	0.29	7	8.179	0.71	40	d	0.130963
			202226_s_at	3	8.292	0.52	7	7.772	0.98	40	d	0.188537

MYRIP	8	myosin VIIA and Rab interacting protein	214156_at	8	3.783	0.3	7	3.824	0.64	40	u	0.870853
SS18L1	8	synovial sarcoma translocation gene on chromosome 18-like 1	213140_s_at	8	7.489	0.28	7	7.685	0.78	40	u	0.264637
VIPR1	8	vasoactive intestinal peptide receptor 1	205019_s_at	0	7.633	0.51	7	5.141	1.42	40	d	0
UBE3C	8	ubiquitin protein ligase E3C	201817_at	8	7.882	0.38	7	7.992	0.72	40	u	0.699479
			1554793_at	0	3.581	0.24	7	3.831	0.36	40	u	0.091672
			1554794_a_at	0	5.553	0.39	7	5.998	0.83	40	u	0.178129
			1555405_at	0	3.877	0.27	7	3.788	0.38	40	d	0.567032
RANBP2	8	RAN binding protein 2	201712_s_at	8	7.502	0.43	7	7.399	0.57	40	d	0.657603
			201711_x_at	8	9.076	0.31	7	9.651	0.77	40	u	0.062142
			201713_s_at	1	9.376	0.19	7	9.409	0.57	40	u	0.788664
			226922_at	0	7.341	0.35	7	6.583	0.99	40	d	0.001542
			242712_x_at	0	5.899	0.49	7	7.148	1.01	40	u	0.003017
UBE2Q1	8	ubiquitin-conjugating enzyme E2Q (putative) 1	222480_at	8	5.548	0.53	7	5.661	0.98	40	u	0.772751
			217978_s_at	1	9.313	0.19	7	9.597	0.67	40	u	0.040787
			231816_s_at	1	2.711	0.05	7	2.943	0.44	40	u	0.002684
RNF128	8	ring finger protein 128	219263_at	8	5.777	0.88	7	4.256	1.18	40	d	0.002667
FKBP1A	8	FK506 binding protein 1A, 12kDa	210186_s_at	0	8.54	0.4	7	8.575	1.22	40	u	0.892835
			210187_at	0	5.584	0.95	7	6.163	0.81	40	u	0.103884
			214119_s_at	0	9.559	0.3	7	9.756	0.94	40	u	0.318175
			200709_at	0	10.96	0.25	7	11.18	0.51	40	u	0.277611
TRIB2	8	tribbles homolog 2 (Drosophila)	202478_at	8	9.311	0.38	7	8.632	1.24	40	d	0.011361
			202479_s_at	1	7.084	0.36	7	7.207	1.04	40	u	0.587665
UTX	8	ubiquitously transcribed tetratricopeptide repeat, X chromosome	203991_s_at	8	6.416	0.3	7	6.411	0.91	40	d	0.978525
			203990_s_at	8	5.462	0.58	7	5.741	1.02	40	u	0.494428
			203992_s_at	6	8.455	0.28	7	7.426	0.85	40	d	0.000004
			238220_at	0	3.25	0.72	7	3.543	0.89	40	u	0.424917
RAB39	8	RAB39, member RAS oncogene family	1554800_at	0	3.395	0.37	7	3.664	0.51	40	u	0.197034
FAM38B	7	family with sequence similarity 38, member B	219602_s_at	7	5.806	1.43	7	5.548	1.23	40	d	0.627953
			1565775_at	0	2.306	0.03	7	2.431	0.25	40	u	0.005379
			222908_at	0	2.759	0.45	7	2.795	0.53	40	u	0.867502
TMSB10	7	thymosin, beta 10	217733_s_at	7	13.6	0.17	7	14.1	0.47	40	u	0.000066
IGSF3	7	immunoglobulin superfamily, member 3	202421_at	7	8.111	0.43	7	8.361	0.76	40	u	0.412909
			1556163_a_at	0	3.098	0.24	7	3.497	0.51	40	u	0.052958
			1556162_at	0	3.291	0.54	7	3.591	0.72	40	u	0.305373
			1552672_a_at	0	3.232	0.37	7	3.426	0.71	40	u	0.493028
LEP	7	leptin (obesity homolog, mouse)	207092_at	1	5.717	2.34	7	3.483	0.89	40	d	0.06034
SLK	7	STE20-like kinase (yeast)	206875_s_at	5	9.169	0.49	7	8.861	0.6	40	d	0.214984
GRK5	7	G protein-coupled receptor kinase 5	204395_s_at	7	3.639	0.58	7	3.563	0.75	40	d	0.804684
			204396_s_at	7	6.066	0.74	7	4.971	0.95	40	d	0.006851
			1569949_at	0	1.546	0.12	7	1.843	0.75	40	u	0.027402
			1557729_at	0	3.341	0.36	7	3.399	0.46	40	u	0.756671
PDCD4	7	programmed cell death 4 (neoplastic transformation inhibitor)	212593_s_at	7	12.04	0.26	7	10.84	1.04	40	d	0
			212594_at	7	8.67	0.36	7	7.782	1.25	40	d	0.001117
			202730_s_at	0	9.455	0.5	7	7.664	1.19	40	d	0.000389
			1557166_at	0	5.326	0.74	7	5.306	1.15	40	d	0.966263
			202731_at	0	10.26	0.49	7	8.348	1.46	40	d	0.000001
GFPT2	7	glutamine-fructose-6-phosphate transaminase 2	205100_at	7	6.31	0.68	7	5.909	0.96	40	d	0.304178
			237352_at	0	2.216	0.19	7	2.228	0.14	40	u	0.851352
ABR	7	active BCR-related gene	212895_s_at	7	8.038	0.33	7	7.271	0.73	40	d	0.010446
			214671_s_at	3	6.306	0.39	7	5.777	0.82	40	d	0.106345
EN1	7	engrailed homeobox 1	220559_at	7	3.709	0.66	7	6.195	3.14	40	u	0.000081
USP32	7	ubiquitin specific peptidase 32	226505_x_at	7	8.854	0.24	7	8.806	0.86	40	d	0.776672
			211702_s_at	2	6.197	0.46	7	6.608	0.69	40	u	0.14526
			244871_s_at	0	6.29	0.4	7	5.952	1.08	40	d	0.428433
GMFB	7	glia maturation factor, beta	202544_at	7	9.521	0.38	7	9.744	0.51	40	u	0.287536
			202543_s_at	2	6.94	0.75	7	8.128	0.8	40	u	0.000887
PALM2-AKAP2	7	PALM2-AKAP2	no probeset available									
C5orf5	7	chromosome 5 open reading frame 5	218518_at	7	8.822	0.22	7	7.282	1.08	40	d	0
NRF1	7	nuclear respiratory factor 1	204651_at	7	6.549	0.43	7	6.169	0.64	40	d	0.147195
			211280_s_at	7	5.71	0.44	7	5.384	0.3	40	d	0.01939
			204652_s_at	7	5.793	0.43	7	5.505	0.77	40	d	0.352993
			211279_at	7	5.573	0.17	7	5.541	0.35	40	d	0.81173
			230114_at	7	3.526	0.18	7	3.476	0.25	40	d	0.618911

HOXB8	7	homeobox B8	229667_s_at	7	2.604	0.79	7	2.389	0.84	40	d	0.53984
			221278_at	0	2.057	0.02	7	2.1	0.07	40	u	0.003246
TLK1	7	tousled-like kinase 1	202606_s_at	7	9.218	0.31	7	9.109	0.48	40	d	0.570282
			241642_x_at	0	3.578	0.14	7	3.813	0.31	40	u	0.062375
			211077_s_at	0	3.981	0.54	7	4.43	0.82	40	u	0.179478
			241643_at	0	2.576	0.06	7	2.657	0.15	40	u	0.166158
			210379_s_at	0	5.133	1.1	7	5.28	1.18	40	u	0.764933
TSHZ1	7	teashirt zinc finger homeobox 1	223282_at	7	7.539	0.51	7	5.905	1.2	40	d	0.001132
			223283_s_at	2	8.913	0.44	7	7.031	1.17	40	d	0.00017
ZBTB47	7	zinc finger and BTB domain containing 47	226484_at	7	6.785	0.49	7	6.162	0.54	40	d	0.007261
			226500_at	7	6.685	0.3	7	6.151	0.38	40	d	0.001143
			234338_s_at	1	4.255	0.78	7	3.925	0.45	40	d	0.351932
HMBOX1	7	homeobox containing 1	219269_at	3	8.209	0.5	7	7.427	1.22	40	d	0.107782
GPR124	7	G protein-coupled receptor 124	221814_at	7	8.363	0.51	7	6.067	0.73	40	d	0
			65718_at	7	5.567	0.52	7	3.778	0.62	40	d	0
RAVER1	7	ribonucleoprotein, PTB-binding 1	223425_at	7	6.945	0.45	7	6.932	0.88	40	d	0.969428
PCYT1B	7	phosphate cytidylyltransferase 1, choline, beta	232553_at	7	2.11	0.25	7	2.38	0.72	40	u	0.089987
			210456_at	1	4.642	0.51	7	4.161	0.44	40	d	0.014488
			206751_s_at	1	2.317	0.15	7	2.314	0.17	40	d	0.972929
STK39	7	serine threonine kinase 39 (STE20/SPS1 homolog, yeast)	202786_at	7	7.342	0.66	7	7.583	1.34	40	u	0.650732
SEPT7	7	septin 7	213151_s_at	7	10.81	0.29	7	10.65	0.41	40	d	0.341246
			1565823_at	0	4.356	0.37	7	5.055	0.65	40	u	0.009615
			244508_at	0	3.728	0.45	7	4.428	0.69	40	u	0.015449
SP7	7	Sp7 transcription factor	1552340_at	7	2.147	0.02	7	2.216	0.1	40	u	0.000443
EIF4E	7	eukaryotic translation initiation factor 4E	201435_s_at	7	8.512	0.32	7	8.989	0.77	40	u	0.122247
			201437_s_at	7	8.974	0.16	7	9.722	0.56	40	u	0
			201436_at	7	6.691	0.29	7	7.473	0.65	40	u	0.00359
			237718_at	0	2.346	0.08	7	2.451	0.21	40	u	0.201987
RMND5A	7	required for meiotic nuclear division 5 homolog A (S. cerevisiae)	212478_at	7	4.44	0.46	7	4.768	0.89	40	u	0.356301
			212482_at	7	8.548	0.26	7	8.435	0.72	40	d	0.478612
			212479_s_at	7	6.722	0.31	7	7.65	0.68	40	u	0.001169
USP15	7	ubiquitin specific peptidase 15	209475_at	7	7.572	0.38	7	7.015	0.75	40	d	0.066443
			210681_s_at	2	7.912	0.4	7	8.478	0.74	40	u	0.059087
			231990_at	0	5.251	0.7	7	5.097	0.89	40	d	0.670423
EPC2	7	enhancer of polycomb homolog 2 (Drosophila)	225838_at	7	8.043	0.34	7	7.863	0.63	40	d	0.478402
APPL2	7	adaptor protein, phosphotyrosine interaction, PH domain and leucine zipper containing 2	218218_at	1	8.099	0.36	7	6.742	1.26	40	d	0.000006
MYT1L	7	myelin transcription factor 1-like	210016_at	7	2.339	0.06	7	2.407	0.11	40	u	0.116707
			1554633_a_at	2	2.326	0.03	7	2.399	0.07	40	u	0.008061
			216672_s_at	0	2.308	0.03	7	2.369	0.05	40	u	0.004768
			241485_at	0	2.691	0.05	7	2.864	0.22	40	u	0.000128
STAT5B	7	signal transducer and activator of transcription 5B	212550_at	7	6.718	0.42	7	5.913	0.68	40	d	0.004889
			212549_at	7	8.878	0.23	7	7.029	0.83	40	d	0
			205026_at	3	5.79	0.42	7	5.243	0.71	40	d	0.060298
			1555086_at	0	5.142	0.59	7	5.062	0.57	40	d	0.737835
			1555088_x_at	0	5.576	0.55	7	5.771	0.57	40	u	0.418029
BTBD11	7	BTB (POZ) domain containing 11	228570_at	7	5.628	0.64	7	3.107	1.13	40	d	0.000001
			238692_at	5	2.844	0.13	7	2.796	0.26	40	d	0.638544
DIO2	7	deiodinase, iodothyronine, type II	203700_s_at	7	6.81	1.48	7	5.346	1.16	40	d	0.006004
			211215_x_at	0	3.484	0.51	7	3.595	0.66	40	u	0.683099
			203699_s_at	0	5.551	2.14	7	4.676	1.89	40	d	0.28412
			231240_at	0	7.165	1.99	7	5.359	1.79	40	d	0.02191
			210819_x_at	0	3.609	0.63	7	3.321	0.69	40	d	0.315418
ARHGAP5	7	Rho GTPase activating protein 5	233849_s_at	7	8.197	0.38	7	7.562	1.15	40	d	0.01336
			217936_at	7	8.805	0.43	7	7.989	0.78	40	d	0.011464
			233872_x_at	7	3	0.09	7	3.207	0.18	40	u	0.004987
			235635_at	0	6.02	0.7	7	4.549	0.98	40	d	0.000556
			1552627_a_at	0	4.649	0.84	7	5.388	1.4	40	u	0.191537
XRN1	7	5'-3' exoribonuclease 1	225814_at	7	9.16	0.31	7	8.857	0.51	40	d	0.142827
			233632_s_at	7	9.136	0.35	7	8.61	0.61	40	d	0.03581
			1555785_a_at	1	3.164	0.17	7	3.923	0.81	40	u	0.000006

			1570394_at	0	3.244	0.16	7	3.68	0.51	40	u	0.00029
IRF2	7	interferon regulatory factor 2	203275_at	7	7.468	0.33	7	6.92	0.93	40	d	0.012039
PPP2CA	7	protein phosphatase 2 (formerly 2A), catalytic subunit, alpha isoform	208652_at	6	10.24	0.35	7	10.29	0.57	40	u	0.816831
NAB1	7	NGFI-A binding protein 1 (EGR1 binding protein 1)	209272_at	7	9.15	0.27	7	7.963	0.95	40	d	0
			208047_s_at	3	5.508	0.78	7	5.552	1.22	40	u	0.928113
			211139_s_at	1	6.236	0.47	7	6.075	1.33	40	d	0.578138
RAP2A	7	RAP2A, member of RAS oncogene family	225585_at	7	8.926	0.37	7	9.312	0.99	40	u	0.324838
			221830_at	2	9.054	0.33	7	9.755	0.9	40	u	0.001656
			214103_s_at	0	3.081	0.52	7	2.906	0.55	40	d	0.444779
			214487_s_at	0	5.631	0.31	7	6.255	1.41	40	u	0.020602
TM9SF3	7	transmembrane 9 superfamily member 3	222399_s_at	7	10.08	0.24	7	10.21	0.91	40	u	0.447387
			217758_s_at	7	10.27	0.23	7	10.38	0.51	40	u	0.56111
			228610_at	0	2.672	0.19	7	3.704	1.11	40	u	0.000003
			239626_x_at	0	2.327	0.22	7	2.405	0.2	40	u	0.363078
RNF139	7	ring finger protein 139	209510_at	5	9.23	0.32	7	9.497	0.82	40	u	0.407691
C14orf43	7	chromosome 14 open reading frame 43	225980_at	7	7.381	0.38	7	6.425	1.12	40	d	0.000467
			235430_at	0	3.955	0.25	7	4.099	0.85	40	u	0.400613
HMG2L1	7	high-mobility group protein 2-like 1	212597_s_at	7	7.833	0.3	7	7.88	0.5	40	u	0.814125
			212596_s_at	7	7.974	0.31	7	7.971	0.63	40	d	0.9916
UNC5D	7	unc-5 homolog D (C. elegans)	231325_at	0	3.324	0.14	7	3.422	0.32	40	u	0.43242
STRA13	7	stimulated by retinoic acid 13 homolog (mouse	no probeset available									
CELSR2	7	cadherin, EGF LAG seven-pass G-type receptor 2 (flamingo homolog, Drosophila)	36499_at	7	7.664	0.66	7	6.624	1.38	40	d	0.062557
			204029_at	7	7.895	0.86	7	6.174	1.89	40	d	0.025295
MYCL1	7	v-myc myelocytomatosis viral oncogene homolog 1, lung carcinoma derived (avian)	214058_at	7	4.461	0.74	7	4.756	0.92	40	u	0.433096
			215491_at	0	2.751	0.16	7	2.796	0.35	40	u	0.743215
MKX	7	mohawk homeobox	239468_at	7	3.056	0.3	7	3.273	0.93	40	u	0.270697
			241902_at	4	2.532	0.2	7	2.655	0.33	40	u	0.362081
			236014_at	0	1.954	0.04	7	1.985	0.04	40	u	0.074808
ACTN1	7	actinin, alpha 1	208636_at	7	11.16	0.38	7	10.64	0.75	40	d	0.086229
			208637_x_at	2	10.21	0.46	7	9.507	0.92	40	d	0.058979
			211160_x_at	0	9.294	0.4	7	8.781	0.79	40	d	0.10615
			237401_at	0	4.479	0.21	7	4.402	0.35	40	d	0.592533
TLOC1	7	translocation protein 1	1552789_at	7	5.374	0.58	7	5.1	0.73	40	d	0.357826
			1552790_a_at	7	7.217	0.31	7	5.719	1.28	40	d	0
			225352_at	7	9.052	0.19	7	8.647	0.64	40	d	0.00386
			208942_s_at	6	10.1	0.16	7	10.18	0.66	40	u	0.563798
			208943_s_at	5	10.35	0.17	7	10.17	0.57	40	d	0.110836
NRBF2	7	nuclear receptor binding factor 2	221803_s_at	7	6.968	0.39	7	6.975	0.82	40	u	0.982657
			223650_s_at	3	7.272	0.53	7	7.016	0.99	40	d	0.517457
NCOA7	7	nuclear receptor coactivator 7	225344_at	7	10.88	0.63	7	10.38	1.27	40	d	0.323516
			243807_at	0	2.888	0.07	7	3.15	0.53	40	u	0.005928
			1568805_at	0	2.762	0.13	7	2.964	0.32	40	u	0.112789
SLC30A3	7	solute carrier family 30 (zinc transporter), member 3	207035_at	7	4.731	0.3	7	4.949	0.5	40	u	0.277651
NKX2-2	7	NK2 homeobox 2	206915_at	7	2.406	0.03	7	3.307	1.88	40	u	0.004738
MYO1D	7	myosin ID	212338_at	7	7.785	0.18	7	7.61	0.85	40	d	0.264893
			1555397_at	0	2.516	0.15	7	2.721	0.35	40	u	0.137676
ZADH2	7	zinc binding alcohol dehydrogenase, domain containing 2	227978_s_at	7	7.099	0.59	7	6.395	1.02	40	d	0.090493
			227977_at	7	7.415	0.31	7	6.816	0.79	40	d	0.059236
			227049_at	7	6.94	0.26	7	6.182	0.98	40	d	0.000317
			234977_at	4	4.472	0.55	7	4.131	0.87	40	d	0.332568
			237776_at	0	2.08	0.13	7	2.055	0.03	40	d	0.6741
			1554239_s_at	0	7.141	0.2	7	6.421	0.96	40	d	0.000149
			244067_x_at	0	2.991	0.2	7	3.102	0.2	40	u	0.186022
HMGB1	7	high-mobility group box 1	224734_at	7	9.051	0.31	7	8.288	0.74	40	d	0.011811
			224731_at	7	11.48	0.25	7	11.51	0.58	40	u	0.901144
			200679_x_at	6	12.13	0.15	7	12.25	0.52	40	u	0.245055
			214938_x_at	4	13.12	0.09	7	13.13	0.34	40	u	0.853424
			200680_x_at	2	13.47	0.14	7	13.55	0.35	40	u	0.572155
ZNF225	7	zinc finger protein 225	207125_at	0	3.079	0.25	7	3.088	0.38	40	u	0.956113
			229123_at	0	6.265	0.44	7	5.409	0.96	40	d	0.028316
			235702_at	0	4.26	0.48	7	4.294	0.58	40	u	0.886843
			214894_x_at	7	9.34	0.24	7	8.98	0.69	40	d	0.020994

MACF1	7	microtubule-actin crosslinking factor 1	215222_x_at	6	9.812	0.3	7	9.208	0.77	40	d	0.051443
			207358_x_at	6	8.366	0.3	7	8.076	0.75	40	d	0.326968
			232168_x_at	0	7.426	0.42	7	7.056	0.7	40	d	0.192715
			208633_s_at	0	9.035	0.23	7	8.369	0.88	40	d	0.00037
			1553407_at	0	4.436	0.56	7	4.348	0.6	40	d	0.723968
			208634_s_at	0	10.69	0.42	7	9.768	0.8	40	d	0.005729
MEX3B	7	mex-3 homolog B (C. elegans)	223627_at	2	3.672	0.75	7	3.823	0.9	40	u	0.682196
GNAI3	7	guanine nucleotide binding protein (G protein), alpha inhibiting activity polypeptide 3	201181_at	7	6.996	0.39	7	7.228	0.81	40	u	0.472263
			201180_s_at	5	10.42	0.25	7	10.86	0.65	40	u	0.091286
			201179_s_at	1	8.455	0.35	7	9.067	1.1	40	u	0.011496
CHSY1	7	carbohydrate (chondroitin) synthase 1	203044_at	7	9.815	0.35	7	9.44	0.89	40	d	0.288044
DIAPH2	7	diaphanous homolog 2 (Drosophila)	205726_at	7	6.846	0.42	7	5.984	1.07	40	d	0.046411
			205603_s_at	0	6.433	0.43	7	6.326	1.24	40	d	0.690734
			217246_s_at	0	2.71	0.21	7	2.839	0.47	40	u	0.490851
PLCG1	7	phospholipase C, gamma 1	202789_at	7	7.224	0.29	7	6.867	1.08	40	d	0.09846
			216551_x_at	4	6.885	0.21	7	7.06	0.51	40	u	0.3843
RAPH1	7	Ras association (RalGDS/AF-6) and pleckstrin homology domains 1	225188_at	7	9.442	0.44	7	8.933	0.83	40	d	0.127479
			225189_s_at	7	8.444	0.57	7	7.435	0.95	40	d	0.010764
			225186_at	7	6.432	0.57	7	6.098	1.2	40	d	0.482893
			231075_x_at	6	6.873	0.59	7	6.094	0.83	40	d	0.023992
			1552482_at	0	5.185	0.65	7	5.413	0.84	40	u	0.509729
ATP11A	7	ATPase, Class VI, type 11A	230875_s_at	7	6.86	0.39	7	6.754	0.93	40	d	0.774324
			216488_s_at	4	2.047	0.03	7	2.346	0.67	40	u	0.008726
			213582_at	4	2.996	0.23	7	3.165	0.46	40	u	0.357682
			215842_s_at	3	4.412	0.42	7	4.514	0.92	40	u	0.780352
			241604_at	0	1.98	0.03	7	2.024	0.05	40	u	0.044888
			241603_at	0	1.719	0.02	7	1.813	0.14	40	u	0.000297
ARMC1	7	armadillo repeat containing 1	218185_s_at	7	8.462	0.22	7	9.339	0.79	40	u	0.000002
			222550_at	7	9.065	0.19	7	9.697	0.67	40	u	0.000034
TIPARP	7	TCDD-inducible poly(ADP-ribose) polymerase	212665_at	7	9.082	0.6	7	8.017	0.87	40	d	0.003834
			1556829_at	0	3.455	0.42	7	3.604	0.4	40	u	0.378418
VSNL1	7	visinin-like 1	203797_at	7	6.98	0.5	7	5.506	1.21	40	d	0.003285
			203798_s_at	1	4.346	0.75	7	3.688	1.12	40	d	0.150021
ARL4C	7	ADP-ribosylation factor-like 4C	202207_at	7	9.763	0.61	7	8.615	0.94	40	d	0.003949
			202206_at	7	8.28	0.61	7	7.728	0.86	40	d	0.116176
			213759_at	1	2.154	0.03	7	2.25	0.18	40	u	0.002923
			202208_s_at	1	8.896	0.54	7	7.819	0.71	40	d	0.000509
RNF144A	7	ring finger protein 144A	204040_at	7	6.151	0.41	7	6.451	1.26	40	u	0.262282
			1570051_at	0	2.578	0.05	7	2.686	0.15	40	u	0.001571
ANTXR2	7	anthrax toxin receptor 2	225524_at	7	9.238	0.37	7	7.568	0.79	40	d	0.000002
			1555536_at	0	4.736	0.37	7	4.771	0.54	40	u	0.872932
MSI1	7	musashi homolog 1 (Drosophila)	206333_at	0	3.664	0.25	7	3.766	0.29	40	u	0.400933
UNK	7	unkempt homolog (Drosophila)	226376_at	7	7.202	0.33	7	6.761	0.44	40	d	0.017743
			1562434_at	0	4.709	0.53	7	4.818	0.6	40	u	0.661007
			228357_at	0	6.015	0.5	7	6.745	1.18	40	u	0.122476
EDNRB	7	endothelin receptor type B	204273_at	7	6.263	0.84	7	3.955	0.91	40	d	0
			206701_x_at	7	5.04	1.21	7	2.648	0.74	40	d	0
			204271_s_at	2	6.618	0.66	7	5.065	0.84	40	d	0.00004
PHF19	7	PHD finger protein 19	225533_at	7	6.393	0.15	7	6.526	0.56	40	u	0.2317
			227211_at	0	4.892	0.23	7	6.208	0.92	40	u	0
			227212_s_at	0	4.179	0.24	7	5.795	1.17	40	u	0
ZCCHC3	7	zinc finger, CCHC domain containing 3	225091_at	7	7.074	0.34	7	6.573	0.7	40	d	0.077347
			225072_at	7	2.426	0.03	7	2.51	0.09	40	u	0.00009
			229993_at	0	1.704	0.02	7	1.744	0.06	40	u	0.006945
INOC1	7	INO80 complex homolog 1 (S. cerevisiae)	229356_x_at	7	8.473	0.2	7	8.243	0.47	40	d	0.21569
			225357_s_at	7	6.483	0.24	7	6.121	0.41	40	d	0.033085
FCHSD2	7	FCH and double SH3 domains 2	203620_s_at	7	6.902	0.37	7	7.199	0.85	40	u	0.377203
			1554360_at	0	2.435	0.04	7	2.662	0.36	40	u	0.000508
KIAA0280	7	KIAA0280	36612_at	0	6.13	0.12	7	5.96	0.52	40	d	0.086911
			212357_at	0	8.161	0.15	7	7.692	0.45	40	d	0.000041
MLLT3	7	myeloid/lymphoid or mixed-lineage leukemia (trithorax homolog, Drosophila); translocated to, 3	204917_s_at	7	5.62	0.53	7	4.494	1.2	40	d	0.0208
			204918_s_at	7	4.878	0.33	7	4.858	1.08	40	d	0.931405
			1569652_at	0	5.273	0.55	7	4.353	0.95	40	d	0.018226
ARRDC4	7	arrestin domain containing 4	225283_at	7	8.815	0.51	7	6.83	1.2	40	d	0.000122

TOPORS	7	topoisomerase I binding, arginine/serine-rich	204071_s_at	7	7.75	0.26	7	7.816	0.66	40	u	0.801295
			1564403_at	0	3.904	0.18	7	3.999	0.28	40	u	0.391425
			221979_at	0	6.687	0.15	7	6.59	0.44	40	d	0.31183
			78383_at	0	6.496	0.35	7	6.349	0.52	40	d	0.481551
PCAF	7	p300/CBP-associated factor	203845_at	7	7.058	0.37	7	6.167	0.93	40	d	0.01859
DUSP6	7	dual specificity phosphatase 6	208892_s_at	7	10.84	0.47	7	8.857	1.39	40	d	0
			208891_at	7	11.36	0.57	7	9.221	1.31	40	d	0.000144
			208893_s_at	1	9.833	0.42	7	7.594	1.51	40	d	0
ELK1	7	ELK1, member of ETS oncogene family	203617_x_at	7	7.618	0.26	7	7.92	0.45	40	u	0.09905
			210376_x_at	4	5.822	0.35	7	6.2	0.94	40	u	0.30899
			210850_s_at	0	2.181	0.09	7	2.529	0.65	40	u	0.002874
UBE2R2	7	ubiquitin-conjugating enzyme E2R 2	226954_at	7	7.373	0.26	7	7.377	0.68	40	u	0.987885
			223014_at	4	9.234	0.28	7	9.846	0.92	40	u	0.002841
FOXO4	7	forkhead box O4	205451_at	7	7.604	0.16	7	7.184	0.45	40	d	0.000271
DIP2B	7	DIP2 disco-interacting protein 2 homolog B (Drosophila)	224872_at	7	8.513	0.4	7	8.377	0.76	40	d	0.651913
			1553271_at	0	3.805	0.56	7	4.005	0.6	40	u	0.430851
			242970_at	0	4.236	0.56	7	4.404	0.65	40	u	0.534867
SLC9A6	7	solute carrier family 9 (sodium/hydrogen exchanger), member 6	203909_at	7	7.71	0.31	7	6.867	1.56	40	d	0.004444
DCUN1D1	7	DCN1, defective in cullin neddylation 1, domain containing 1 (S. cerevisiae)	218583_s_at	5	7.942	0.39	7	8.513	0.54	40	u	0.012202
			222679_s_at	5	7.217	0.27	7	8.507	0.71	40	u	0.000029
			222678_s_at	5	5.325	0.56	7	6.41	1.13	40	u	0.019097
			242428_at	0	5.433	0.55	7	6.109	0.93	40	u	0.074689
GNPDA2	7	glucosamine-6-phosphate deaminase 2	227022_at	7	7.244	0.45	7	6.534	0.84	40	d	0.038873
NLGN1	7	neuroligin 1	205893_at	7	3.285	0.46	7	3.082	0.55	40	d	0.372691
			231361_at	0	2.724	0.3	7	2.816	0.44	40	u	0.600902
PDZRN4	7	PDZ domain containing RING finger 4	220595_at	7	2.566	0.15	7	2.698	0.32	40	u	0.299713
SOCS2	7	suppressor of cytokine signaling 2	203373_at	7	8.557	1.08	7	5.86	1.57	40	d	0.000101
			203372_s_at	1	6.57	1.13	7	4.128	1.38	40	d	0.000084
CSNK1G3	7	casein kinase 1, gamma 3	220768_s_at	7	7.784	0.37	7	7.694	0.55	40	d	0.682366
			229702_at	0	2.065	0.02	7	2.138	0.21	40	u	0.037149
			227767_at	0	7.739	0.36	7	7.449	0.68	40	d	0.283916
DNAJB5	7	DnaJ (Hsp40) homolog, subfamily B, member 5	212817_at	7	4.548	0.47	7	4.103	0.62	40	d	0.083206
			207453_s_at	1	2.312	0.08	7	2.398	0.24	40	u	0.096525
ST3GAL3	7	ST3 beta-galactoside alpha-2,3-sialyltransferase 3	225905_s_at	7	5.746	0.32	7	5.32	0.55	40	d	0.057787
			231659_at	6	2.042	0.02	7	2.1	0.05	40	u	0.00606
			1555171_at	3	2.678	0.15	7	2.755	0.16	40	u	0.243305
			1555181_a_at	0	6.858	0.34	7	7.376	0.39	40	u	0.002555
			1555678_at	0	2.879	0.26	7	2.916	0.34	40	u	0.785684
			1555702_a_at	0	4.136	0.51	7	4.087	0.51	40	d	0.819877
			1555703_at	0	2.76	0.09	7	2.995	0.46	40	u	0.006214
ULK2	7	unc-51-like kinase 2 (C. elegans)	204063_s_at	7	5.659	0.62	7	4.77	1.41	40	d	0.114618
			204062_s_at	7	6.795	0.33	7	6.6	1.02	40	d	0.364868
			1554112_a_at	0	3.433	0.68	7	3.546	1.29	40	u	0.826075
			215154_at	0	3.224	0.42	7	3.364	0.48	40	u	0.48274
NRCAM	7	neuronal cell adhesion molecule	204105_s_at	7	3.151	0.47	7	3.534	1.5	40	u	0.221488
			216959_x_at	0	2.14	0.1	7	2.33	0.54	40	u	0.053681
ADIPOR2	7	adiponectin receptor 2	201346_at	7	8.805	0.34	7	8.885	1.02	40	u	0.709915
KITLG	7	KIT ligand	226534_at	0	9.271	0.53	7	7.954	1.49	40	d	0.000441
			211124_s_at	0	2.699	0.2	7	3.12	0.92	40	u	0.016942
			207029_at	0	3.118	0.3	7	3.461	0.89	40	u	0.083141
STX16	7	syntaxin 16	221500_s_at	7	9.858	0.15	7	9.168	0.71	40	d	0.000004
			221499_s_at	7	8.64	0.27	7	8.173	0.6	40	d	0.053321
			1558249_s_at	5	7.17	0.35	7	7.106	0.89	40	d	0.85603
			221638_s_at	0	5.59	0.44	7	5.81	0.74	40	u	0.461378
ADAMTS5	7	ADAM metallopeptidase with thrombospondin type 1 motif, 5 (aggrecanase-2)	219935_at	7	9.741	0.52	7	6.318	1.47	40	d	0
			1558636_s_at	4	4.699	0.52	7	3.638	0.8	40	d	0.00192
			229357_at	0	9.255	0.56	7	5.413	1.46	40	d	0
			235368_at	0	9.781	0.51	7	6.843	1.27	40	d	0
ADAMTSL3	7	ADAMTS-like 3	213974_at	7	3.154	0.74	7	3.047	1.27	40	d	0.832127
			1559748_at	0	2.505	0.16	7	2.607	0.19	40	u	0.196031
POM121	7	POM121 membrane glycoprotein (rat)	212178_s_at	7	8.406	0.32	7	8.197	0.79	40	d	0.505755
			205096_at	0	5.086	0.38	7	4.748	0.36	40	d	0.029574
			213360_s_at	0	10.18	0.41	7	9.556	0.79	40	d	0.052263

GAD1	7	glutamate decarboxylase 1 (brain, 67kDa)	205278_at	7	2.271	0.13	7	2.686	1.64	40	u	0.129257
			206669_at	0	3.51	0.23	7	3.76	0.94	40	u	0.168033
			206670_s_at	0	2.127	0.07	7	2.592	1.42	40	u	0.049237
NDFIP1	7	Nedd4 family interacting protein 1	222422_s_at	7	8.507	0.34	7	8.41	0.83	40	d	0.766346
			217800_s_at	7	10.49	0.24	7	10.31	0.55	40	d	0.396009
			222423_at	7	10.07	0.35	7	8.909	0.88	40	d	0.001445
APPL1	7	adaptor protein, phosphotyrosine interaction, PH domain and leucine zipper containing 1	222538_s_at	7	7.89	0.27	7	8.017	0.71	40	u	0.648436
			218158_s_at	7	7.086	0.4	7	6.855	0.72	40	d	0.425685
ADCY9	7	adenylate cyclase 9	204497_at	7	7.552	0.33	7	6.546	1.36	40	d	0.000342
			204498_s_at	2	3.903	0.53	7	4.02	1.03	40	u	0.775032
			215400_x_at	0	5.312	0.27	7	5.19	0.32	40	d	0.358973
DPP10	7	dipeptidyl-peptidase 10	228598_at	7	2.567	0.06	7	2.85	0.63	40	u	0.008799
PRR3	7	proline rich 3	204795_at	7	5.364	0.42	7	5.354	0.66	40	d	0.970062
SNCB	7	synuclein, beta	207853_s_at	5	2.4	0.12	7	2.414	0.16	40	u	0.830232
KCTD1	7	potassium channel tetramerisation domain containing 1	226246_at	7	8.874	0.36	7	7.566	1.15	40	d	0.000006
			226245_at	7	9.746	0.25	7	8.421	1.12	40	d	0
			229183_at	0	2.171	0.11	7	2.142	0.08	40	d	0.440141
IGF2BP3	7	insulin-like growth factor 2 mRNA binding protein 3	203820_s_at	7	3.56	0.29	7	4.406	1.63	40	u	0.005007
			203819_s_at	7	2.911	0.49	7	3.908	2.07	40	u	0.014226
			1569033_at	0	3.127	0.12	7	3.368	0.44	40	u	0.009132
			216493_s_at	0	3.157	0.16	7	3.607	0.64	40	u	0.000741
FRY	7	furry homolog (Drosophila)	204072_s_at	7	8.183	0.49	7	6.21	1.49	40	d	0.000001
			214319_at	0	4.246	0.48	7	4.166	0.61	40	d	0.749174
			214318_s_at	0	5.223	0.49	7	4.532	0.86	40	d	0.048737
SHC1	7	SHC (Src homology 2 domain containing) transforming protein 1	214853_s_at	7	10.23	0.38	7	9.844	0.62	40	d	0.121261
			201469_s_at	7	8.092	0.4	7	7.977	1.07	40	d	0.78584
CEP170	7	centrosomal protein 170kDa	207719_x_at	7	7.832	0.34	7	8.643	0.71	40	u	0.00577
			212746_s_at	7	7.208	0.34	7	8	0.81	40	u	0.016948
			1552717_s_at	0	4.927	0.66	7	5.813	1.1	40	u	0.048754
PLEKHC1	7	pleckstrin homology domain containing, family C (with FERM domain) member 1	209210_s_at	7	10.18	0.44	7	8.515	1.3	40	d	0.000003
			209209_s_at	7	7.929	0.61	7	6.43	1.58	40	d	0.019536
			214212_x_at	0	7.269	0.43	7	6.057	1.04	40	d	0.004693
ATOH8	7	atonal homolog 8 (Drosophila)	228890_at	7	5.186	0.63	7	4.115	0.7	40	d	0.000617
			1558705_at	0	3.131	0.25	7	3.392	0.32	40	u	0.050061
			1558706_a_at	0	4.56	0.52	7	4.374	0.5	40	d	0.387938
SORT1	7	sortilin 1	224818_at	7	9.523	0.45	7	9.225	0.82	40	d	0.366935
			212807_s_at	2	6.748	0.46	7	7.621	0.92	40	u	0.02016
			212797_at	2	4.343	0.35	7	4.892	1.34	40	u	0.040166
STARD13	7	StAR-related lipid transfer (START) domain containing 13	213103_at	7	7.31	0.27	7	5.706	1.07	40	d	0
			242686_at	0	2.502	0.24	7	2.636	0.55	40	u	0.535379
			233159_at	0	2.118	0.18	7	2.225	0.16	40	u	0.121684
KIAA0317	7	KIAA0317	202128_at	7	7.09	0.26	7	7.027	0.58	40	d	0.784348
			244137_at	0	2.156	0.05	7	2.259	0.16	40	u	0.003543
			1554989_at	0	3.027	0.23	7	3.083	0.26	40	u	0.59769
SLAIN1	7	SLAIN motif family, member 1	225619_at	7	3.756	0.42	7	3.865	1.3	40	u	0.687263
GNAO1	7	guanine nucleotide binding protein (G protein), alpha activating activity polypeptide O	204762_s_at	7	2.214	0.36	7	2.37	0.45	40	u	0.402918
			204763_s_at	7	2.378	0.03	7	2.519	0.31	40	u	0.007899
			215912_at	0	2.343	0.09	7	2.386	0.11	40	u	0.318844
			231951_at	0	5.169	0.6	7	6.023	0.66	40	u	0.003159
ZC3H6	7	zinc finger CCCH-type containing 6	241372_at	7	4.833	0.23	7	4.808	0.52	40	d	0.904143
			227809_at	0	7.487	0.35	7	5.971	0.89	40	d	0.000077
RBM26	7	RNA binding motif protein 26	222626_at	7	5.812	0.46	7	6.266	0.73	40	u	0.126915
			218422_s_at	7	9.635	0.3	7	9.475	0.65	40	d	0.53579
			227355_at	0	3.948	0.39	7	4.604	0.76	40	u	0.034769
			215751_at	0	3.101	0.23	7	3.193	0.27	40	u	0.404122
			229433_at	0	6.033	0.3	7	6.569	0.62	40	u	0.034766
			215647_at	0	2.895	0.1	7	3.1	0.18	40	u	0.006168
			220509_at	0	2.185	0.08	7	2.326	0.33	40	u	0.027211
RIMBP2	7	RIMS binding protein 2	214811_at	7	2.347	0.05	7	2.708	1.15	40	u	0.059563
			237995_at	0	2.28	0.09	7	2.427	0.47	40	u	0.086596
			238817_at	0	2.254	0.11	7	2.375	0.17	40	u	0.089932
KIT	7	v-kit Hardy-Zuckerman 4 feline sarcoma viral oncogene homolog	205051_s_at	7	10.56	0.59	7	6.374	1.73	40	d	0
		MYST histone acetyltransferase (monocytic	202423_at	7	10.32	0.31	7	9.614	0.93	40	d	0.001157

MYST3	7	MYST histone acetyltransferase (monocytic leukemia) 3	1559142_at	0	2.873	0.07	7	3.092	0.41	40	u	0.003538
			216361_s_at	0	5.141	0.43	7	6.303	1.3	40	u	0.000234
CORO1C	7	coronin, actin binding protein, 1C	221676_s_at	7	8.516	0.53	7	9.078	1.01	40	u	0.167192
			222409_at	7	9.642	0.31	7	9.19	0.66	40	d	0.086368
HAS2	7	hyaluronan synthase 2	206432_at	7	3.377	0.87	7	3.673	0.85	40	u	0.413278
RS1	7	retinoschisis (X-linked, juvenile) 1	207363_at	1	2.944	0.08	7	3.026	0.13	40	u	0.128217
			216937_s_at	0	2.389	0.3	7	2.413	0.27	40	u	0.83501
C7orf41	7	chromosome 7 open reading frame 41	226018_at	7	8.125	0.27	7	6.145	1.33	40	d	0
			227000_at	3	3.069	0.48	7	3.313	1.03	40	u	0.547867
TARBP2P	7	TAR (HIV-1) RNA binding protein 2 pseudogen	no probeset available									
FAM107B	7	family with sequence similarity 107, member B	223058_at	7	8.718	0.64	7	8.609	1.07	40	d	0.800238
			223059_s_at	0	8.432	0.56	7	8.766	1.05	40	u	0.425184
C21orf66	7	chromosome 21 open reading frame 66	218515_at	7	8.569	0.2	7	8.345	0.59	40	d	0.087754
			1555125_at	0	4.779	0.45	7	6.143	0.87	40	u	0.000264
			240105_at	0	5.67	0.49	7	6.944	0.99	40	u	0.002186
			221158_at	0	5.929	0.39	7	6.277	0.57	40	u	0.134259
PAX2	7	paired box 2	206228_at	7	2.328	0.39	7	2.47	0.72	40	u	0.620321
			206229_x_at	6	3.43	0.42	7	3.577	0.43	40	u	0.42057
MLSTD2	7	male sterility domain containing 2	224865_at	7	7.799	0.43	7	7.009	0.71	40	d	0.008012
			224866_at	7	7.922	0.41	7	8.037	0.55	40	u	0.608411
			1558014_s_at	4	5.366	0.61	7	5.963	1.21	40	u	0.217965
			234767_at	0	2.584	0.05	7	2.723	0.16	40	u	0.0003
EPHB1	7	EPH receptor B1	230425_at	7	4.477	1.2	7	3.194	1.56	40	d	0.04905
			210753_s_at	3	2.455	0.23	7	2.481	0.88	40	u	0.875663
			211898_s_at	0	2.254	0.21	7	2.302	0.58	40	u	0.706388
SMARCA1	7	SWI/SNF related, matrix associated, actin dependent regulator of chromatin, subfamily a, member 1	203873_at	0	1.906	0.05	7	2.01	0.15	40	u	0.001952
			203874_s_at	0	8.688	0.52	7	7.079	1.74	40	d	0.000076
			215294_s_at	0	7.484	0.5	7	6.324	1.64	40	d	0.00158
			203875_at	0	7.931	0.48	7	7.006	1.2	40	d	0.05539
MDGA1	7	MAM domain containing glycosylphosphatidylinositol anchor 1	238543_x_at	7	6.004	0.39	7	6.077	0.63	40	u	0.775505
			232237_at	2	4.955	0.27	7	5.041	0.4	40	u	0.588801
			242757_at	0	2.053	0.01	7	2.082	0.03	40	u	0.016349
GPC4	7	glypican 4	204984_at	7	5.571	0.44	7	5.725	1.43	40	u	0.603453
			204983_s_at	1	4.926	0.61	7	4.523	1.69	40	d	0.285546
SAPS2	7	SAPS domain family, member 2	202792_s_at	7	6.683	0.39	7	5.79	0.74	40	d	0.003848
			202791_s_at	7	6.681	0.27	7	6.436	0.44	40	d	0.166419
			238110_at	0	3.744	0.41	7	3.599	0.44	40	d	0.429804
			1570210_x_at	0	4.659	0.54	7	4.374	0.53	40	d	0.208347
STIM2	7	stromal interaction molecule 2	225250_at	7	5.798	0.44	7	5.073	0.55	40	d	0.002157
			234140_s_at	7	7.066	0.22	7	6.279	0.84	40	d	0.000021
			225246_at	7	7.259	0.3	7	6.623	1.07	40	d	0.005156
DAZAP2	7	DAZ associated protein 2	214334_x_at	7	9.731	0.18	7	9.27	0.53	40	d	0.000319
			200794_x_at	7	11.87	0.14	7	11.32	0.59	40	d	0.000014
			212595_s_at	5	10.34	0.38	7	9.539	1.11	40	d	0.002449
			235704_at	0	2.779	0.42	7	2.783	0.62	40	u	0.988676
			238300_s_at	0	2.508	0.13	7	2.612	0.2	40	u	0.202362
ABHD13	7	abhydrolase domain containing 13	235348_at	7	4.952	0.39	7	4.901	0.6	40	d	0.833962
			234993_at	3	4.289	0.66	7	4.107	0.68	40	d	0.526714
PHF2	7	PHD finger protein 2	212726_at	7	7.923	0.25	7	7.376	0.61	40	d	0.026332
			207138_at	0	2.992	0.12	7	3.046	0.19	40	u	0.47111
PID1	7	phosphotyrosine interaction domain containing 1	219093_at	7	6.378	0.96	7	4.599	1.1	40	d	0.000301
			237867_s_at	0	3.337	0.44	7	3.143	0.58	40	d	0.411633
			237866_at	0	2.513	0.05	7	2.62	0.12	40	u	0.02487
MAP2K7	7	mitogen-activated protein kinase kinase 7	226023_at	7	6.79	0.29	7	6.617	0.69	40	d	0.526762
			226053_at	7	7.337	0.35	7	7.298	0.79	40	d	0.900319
			209951_s_at	1	1.975	0.05	7	2.148	0.82	40	u	0.199784
			209952_s_at	0	3.584	0.41	7	3.889	0.81	40	u	0.344628
			216206_x_at	0	5.13	0.49	7	5.245	0.49	40	u	0.574856
SP2	7	Sp2 transcription factor	204367_at	7	7.545	0.66	7	6.566	0.76	40	d	0.002904
			237795_s_at	0	2.765	0.09	7	2.864	0.19	40	u	0.192816
			211736_at	0	2.464	0.04	7	2.538	0.11	40	u	0.083512
KIAA1967	7	KIAA1967	225187_at	8	8.463	0.38	7	7.085	1.18	40	d	0.000005
			225193_at	8	4.729	0.4	7	4.524	0.69	40	d	0.455747
			221070_s_at	0	2.932	0.1	7	2.99	0.18	40	u	0.431017

KCNJ3	7	potassium inwardly-rectifying channel, subfamily J, member 3	207142_at	7	3.067	0.16	7	3.831	1.36	40	u	0.001649
			207141_s_at	5	2.173	0.02	7	2.725	1.31	40	u	0.012474
NTF3	7	neurotrophin 3	206706_at	7	3.789	0.62	7	3.417	0.9	40	d	0.307905
CPLX2	7	complexin 2	225815_at	7	4.073	0.23	7	4.374	0.93	40	u	0.093382
			206368_at	0	2.695	0.18	7	2.782	0.33	40	u	0.507725
RAB11FIP1	7	RAB11 family interacting protein 1 (class I)	225177_at	7	9.309	0.31	7	8.147	1.8	40	d	0.000609
			219681_s_at	5	8.857	0.11	7	8.37	1.72	40	d	0.087959
			231830_x_at	2	7.394	0.37	7	7.421	0.71	40	u	0.925489
EIF2C2	7	eukaryotic translation initiation factor 2C, 2	225827_at	7	6.855	0.46	7	8.224	1.1	40	u	0.00274
			213310_at	0	5.162	0.18	7	6.751	1.11	40	u	0
			225569_at	0	7.546	0.33	7	7.833	0.93	40	u	0.168865
GABRB3	7	gamma-aminobutyric acid (GABA) A receptor, beta 3	229724_at	7	2.98	1.19	7	3.075	1.77	40	u	0.893741
			205850_s_at	2	2.628	0.3	7	2.863	1.12	40	u	0.289665
			227830_at	0	3.379	0.83	7	3.159	1.2	40	d	0.650223
			1561316_at	0	2.649	0.24	7	2.729	0.19	40	u	0.348724
			227690_at	0	2.861	0.47	7	2.945	1.06	40	u	0.841311
TGIF1	7	TGFB-induced factor homeobox 1	203313_s_at	4	9.451	0.23	7	9.168	0.53	40	d	0.181259
			244858_at	0	4.788	0.62	7	4.638	0.67	40	d	0.589217
ZNF512B	7	zinc finger protein 512B	221869_at	7	7.213	0.23	7	6.855	0.63	40	d	0.015955
			55872_at	7	8.165	0.36	7	7.741	0.95	40	d	0.259965
BNIP3L	7	BCL2/adenovirus E1B 19kDa interacting protein 3-like	221478_at	7	10.26	0.35	7	9.066	0.72	40	d	0.000128
			221479_s_at	3	11.5	0.53	7	10.29	0.73	40	d	0.000165
DLC1	7	deleted in liver cancer 1	224822_at	7	8.644	0.54	7	6.1	1.21	40	d	0.000003
			220512_at	0	4.199	0.73	7	3.549	0.35	40	d	0.075103
			242631_x_at	0	2.972	0.19	7	3.067	0.18	40	u	0.210431
			220511_s_at	0	2.914	0.24	7	3.079	0.28	40	u	0.15987
			210762_s_at	0	9.462	0.59	7	6.952	1.13	40	d	0.000001
ASXL1	7	additional sex combs like 1 (Drosophila)	212238_at	7	6.319	0.29	7	6.292	0.63	40	d	0.912105
			212234_at	7	7.53	0.28	7	7.03	0.77	40	d	0.006596
			212237_at	7	8.382	0.25	7	7.761	0.59	40	d	0.009755
			244519_at	0	4.107	0.54	7	5.655	1.11	40	u	0.000943
WDR37	7	WD repeat domain 37	211383_s_at	7	7.34	0.29	7	6.911	0.62	40	d	0.088252
			1554869_at	0	3.763	0.3	7	3.959	0.3	40	u	0.125553
			242255_at	0	3.137	0.39	7	3.403	0.74	40	u	0.3651
			230198_at	0	3.498	0.48	7	4.202	1.1	40	u	0.108549
IGF2R	7	insulin-like growth factor 2 receptor	201392_s_at	7	7.158	0.53	7	7.224	0.78	40	u	0.833806
			201393_s_at	7	8.236	0.51	7	8.019	0.78	40	d	0.491288
ELK3	7	ELK3, ETS-domain protein (SRF accessory protein 2)	221773_at	7	9.237	0.49	7	8.742	0.74	40	d	0.10243
			206127_at	0	3.193	0.33	7	3.539	0.63	40	u	0.173972
EPHB2	7	EPH receptor B2	209588_at	7	2.762	0.53	7	2.825	0.6	40	u	0.800832
			209589_s_at	2	4.066	0.43	7	4.446	0.74	40	u	0.200793
			210651_s_at	2	4.775	0.44	7	4.794	0.51	40	u	0.929464
			211165_x_at	0	2.98	0.59	7	3.34	0.87	40	u	0.309079
ZNF652	7	zinc finger protein 652	205594_at	7	6.762	0.75	7	6.086	1.27	40	d	0.187937
			235577_at	0	7.133	0.52	7	7.145	1.24	40	u	0.979187
			225266_at	0	9.754	0.43	7	8.7	1.04	40	d	0.013266
VAMP2	7	vesicle-associated membrane protein 2 (synaptobrevin 2)	201557_at	15	8.927	0.4	7	7.478	0.66	40	d	0.000002
			214792_x_at	7	7.701	0.5	7	6.907	0.99	40	d	0.049022
			201556_s_at	7	8.161	0.28	7	7.812	0.8	40	d	0.051886
MATR3	7	matrin 3	214363_s_at	7	12.73	0.2	7	12.47	0.5	40	d	0.189352
			200626_s_at	7	10.64	0.18	7	10.4	0.69	40	d	0.079517
			200624_s_at	7	10.98	0.18	7	10.84	0.78	40	d	0.334315
			1564907_s_at	0	3.468	0.55	7	4.047	0.92	40	u	0.119151
			228012_at	0	5.46	0.49	7	5.315	0.78	40	d	0.644503
			1558093_s_at	0	7.721	0.48	7	8.306	1.39	40	u	0.059872
			242260_at	0	3.515	1.22	7	4.065	1.31	40	u	0.316757
DPYSL2	7	dihydropyrimidinase-like 2	200762_at	7	11.33	0.14	7	9.836	0.97	40	d	0
KCTD15	7	potassium channel tetramerisation domain containing 15	218553_s_at	7	5.018	0.37	7	4.956	0.95	40	d	0.866605
			222668_at	7	7.253	0.25	7	6.696	1.52	40	d	0.040112
			222664_at	7	7.182	0.36	7	6.755	1.24	40	d	0.095155
			229683_s_at	2	2.098	0.03	7	2.182	0.16	40	u	0.003546
			228683_s_at	1	2.93	0.31	7	3.073	0.6	40	u	0.544546
			242999_at	7	3.028	0.35	7	2.974	0.2	40	d	0.727899
			229642_at	1	5.552	0.54	7	4.856	0.71	40	d	0.020293

ARHGEF7	7	Rho guanine nucleotide exchange factor (GEF) 7	1562271_x_at	0	5.268	0.31	7	4.978	0.52	40	d	0.163477
			235412_at	0	3.217	1.03	7	3.786	1.31	40	u	0.29175
			202548_s_at	0	8.115	0.41	7	7.476	0.67	40	d	0.020602
			202547_s_at	0	6.107	0.36	7	6.715	0.6	40	u	0.014481
			239397_at	0	2.471	0.05	7	2.564	0.17	40	u	0.010248
			1562270_at	0	4.442	0.6	7	4.22	0.69	40	d	0.436215
ZNF704	7	zinc finger protein 704	241963_at	7	3.283	0.49	7	3.358	0.64	40	u	0.772414
			1558941_at	3	2.186	0.13	7	2.329	0.19	40	u	0.0674
C1QL3	7	omplement component 1, q subcomponent-like	no probeset available									
PHF3	7	PHD finger protein 3	217953_at	7	3.248	0.31	7	3.572	0.56	40	u	0.150322
			217952_x_at	7	9.288	0.21	7	8.823	0.54	40	d	0.034175
			217954_s_at	7	8.906	0.22	7	8.891	0.57	40	d	0.946633
			217951_s_at	7	6.315	0.6	7	7.331	0.72	40	u	0.001258
			215718_s_at	3	8.627	0.22	7	8.257	0.56	40	d	0.098609
CAPZA2	7	capping protein (actin filament) muscle Z-line, alpha 2	201237_at	7	9.437	0.31	7	9.265	0.93	40	d	0.385452
			201238_s_at	5	10.41	0.37	7	10.45	0.78	40	u	0.904214
			1569450_at	0	3.841	0.46	7	3.996	0.43	40	u	0.403591
LPGAT1	7	lysophosphatidylglycerol acyltransferase 1	202651_at	7	8.225	0.26	7	9.05	0.85	40	u	0.000049
			1555058_a_at	1	5.593	0.6	7	7.702	1.13	40	u	0.000022
GAN	7	giant axonal neuropathy (gigaxonin)	220124_at	7	2.456	0.06	7	2.644	0.3	40	u	0.000926
ATP1B1	7	ATPase, Na+/K+ transporting, beta 1 polypeptide	201243_s_at	9	11.46	0.64	7	10.7	1.12	40	d	0.095023
			201242_s_at	0	10.49	0.74	7	10.59	1.14	40	u	0.825478
TWF1	7	twinfilin, actin-binding protein, homolog 1 (Drosophila)	201745_at	7	9.093	0.33	7	9.464	0.56	40	u	0.100824
			214007_s_at	0	7.37	0.64	7	8.597	0.98	40	u	0.003085
			244199_at	0	3.289	0.51	7	3.303	0.55	40	u	0.953828
			243033_at	0	2.901	0.19	7	3.019	0.19	40	u	0.144843
			214008_at	0	2.944	0.31	7	3.915	0.83	40	u	0.00463
NUP153	7	nucleoporin 153kDa	202097_at	7	8.867	0.28	7	9.201	0.6	40	u	0.162183
			1559064_at	0	2.825	0.08	7	3.301	0.7	40	u	0.000184
			239948_at	0	4.205	0.41	7	4.234	0.58	40	u	0.903995
DCLK1	7	doublecortin-like kinase 1	205399_at	7	8.047	0.68	7	5.945	1.59	40	d	0.00157
			229800_at	0	8.091	0.79	7	6.077	1.88	40	d	0.008948
CASC3	7	cancer susceptibility candidate 3	207842_s_at	7	9.848	0.31	7	9.247	1.17	40	d	0.011907
UBXD8	7	UBX domain containing 8	212106_at	7	5.736	0.28	7	7.079	0.83	40	u	0
			212108_at	7	8.913	0.12	7	8.871	0.83	40	d	0.767934
EVI5	7	ecotropic viral integration site 5	209717_at	7	5.89	0.26	7	5.829	0.67	40	d	0.818215
			208297_s_at	0	3.894	0.46	7	4.66	0.73	40	u	0.011726
			208298_at	0	2.422	0.03	7	2.473	0.07	40	u	0.06072
LITAF	7	lipopolysaccharide-induced TNF factor	200706_s_at	7	9.807	0.27	7	9.408	1	40	d	0.048237
			1555454_at	7	2.792	0.12	7	2.955	0.27	40	u	0.1361
			200704_at	7	11.01	0.12	7	10.57	0.69	40	d	0.00056
FOXJ2	7	forkhead box J2	203734_at	7	8.18	0.37	7	7.84	0.68	40	d	0.210392
			1555305_at	0	3.173	0.18	7	3.349	0.44	40	u	0.307556
PIK3R3	7	phosphoinositide-3-kinase, regulatory subunit 3 (p55, gamma)	202743_at	7	9.23	0.48	7	9.65	0.85	40	u	0.21952
			211580_s_at	0	5.182	0.29	7	6.052	1.24	40	u	0.000571
NPTX2	7	neuronal pentraxin II	213479_at	7	2.318	0.12	7	3.209	1.62	40	u	0.001639
SNAP25	7	synaptosomal-associated protein, 25kDa	202508_s_at	7	3.15	0.67	7	3.994	1.85	40	u	0.048261
			1556629_a_at	0	3.002	0.23	7	3.406	1.32	40	u	0.086939
			202507_s_at	0	2.939	0.44	7	3.217	1.17	40	u	0.54684
SOX12	7	SRY (sex determining region Y)-box 12	204432_at	7	6.463	0.57	7	6.624	0.77	40	u	0.607128
			228358_at	7	3.76	0.37	7	3.934	0.6	40	u	0.47259
GOLGA8A	7	golgi autoantigen, golgin subfamily a, 8A	208798_x_at	7	10.13	0.29	7	7.843	1.27	40	d	0
			210424_s_at	2	6.365	0.66	7	5.242	1.08	40	d	0.012283
			213650_at	0	6.959	0.83	7	6.206	0.79	40	d	0.028842
FGFR2	7	fibroblast growth factor receptor 2 (bacteria-expressed kinase, keratinocyte growth factor receptor, craniofacial dysostosis 1, Crouzon syndrome, Pfeiffer syndrome, Jackson-Weiss syndrome)	203638_s_at	7	9.256	0.68	7	7.656	2.29	40	d	0.001558
			208228_s_at	3	7.915	0.63	7	7.031	2.13	40	d	0.047226
			203639_s_at	0	7.152	0.47	7	5.794	2.42	40	d	0.002984
			208229_at	0	3.318	0.35	7	3.797	0.65	40	u	0.068674
			211399_at	0	5.7	0.32	7	5.51	0.54	40	d	0.378598
			208225_at	0	2.675	0.11	7	2.936	0.85	40	u	0.074819
			208234_x_at	0	4.048	0.2	7	4.328	0.96	40	u	0.115579
			211398_at	0	3.308	0.3	7	3.354	0.56	40	u	0.834434
			211401_s_at	0	5.023	0.65	7	5.219	1.68	40	u	0.766959
			240913_at	0	2.776	0.2	7	3.076	1.23	40	u	0.16694

			1560859_at	0	3.345	0.17	7	3.646	1.13	40	u	0.128646
			211400_at	0	2.044	0.1	7	2.084	0.18	40	u	0.575854
MYH10	7	myosin, heavy chain 10, non-muscle	212372_at	7	10.16	0.32	7	8.92	0.74	40	d	0.000098
			213067_at	0	6.16	0.55	7	5.51	1.23	40	d	0.184751
LHX8	7	LIM homeobox 8	1569469_a_at	7	3.064	0.11	7	3.642	0.96	40	u	0.000804
PNRC1	7	proline-rich nuclear receptor coactivator 1	209034_at	7	9.253	0.23	7	8.775	0.82	40	d	0.005616
TMEM184B	7	transmembrane protein 184B	202027_at	7	8.224	0.15	7	7.918	0.68	40	d	0.0191
CLASP2	7	cytoplasmic linker associated protein 2	212308_at	7	6.43	0.4	7	5.177	1.02	40	d	0.002948
			212309_at	7	8.177	0.29	7	7.48	0.77	40	d	0.025931
			212306_at	7	8.352	0.42	7	7.951	0.79	40	d	0.209126
			238048_at	0	2.434	0.03	7	2.49	0.06	40	u	0.018185
			1558759_s_at	0	6.129	0.56	7	4.886	1.09	40	d	0.006143
			1555469_a_at	0	5.342	0.57	7	4.726	0.97	40	d	0.117533
ELOVL4	7	elongation of very long chain fatty acids (FEN1/Elo2, SUR4/Elo3, yeast)-like 4	219532_at	7	3.431	0.42	7	3.961	1.22	40	u	0.051835
UBE2D1	7	ubiquitin-conjugating enzyme E2D 1 (UBC4/5 homolog, yeast)	211764_s_at	7	7.235	0.16	7	8.072	0.64	40	u	0
			215957_at	7	3.061	0.21	7	3.112	0.21	40	u	0.566876
			214590_s_at	3	5.537	0.32	7	6.152	0.76	40	u	0.045928
FAM60A	7	family with sequence similarity 60, member A	220147_s_at	7	10.27	0.27	7	10.39	1.17	40	u	0.609324
			223038_s_at	7	7.538	0.56	7	8.686	1.27	40	u	0.025804
COPS7B	7	COP9 constitutive photomorphogenic homolog subunit 7B (Arabidopsis)	219997_s_at	7	7.276	0.19	7	6.942	0.65	40	d	0.015179
			225696_at	7	5.591	0.41	7	5.874	0.59	40	u	0.241774
			243628_at	0	2.292	0.11	7	2.356	0.32	40	u	0.362898
EIF4B	7	eukaryotic translation initiation factor 4B	211938_at	7	11.5	0.19	7	10.03	0.53	40	d	0
			211937_at	6	11.56	0.24	7	10.8	0.46	40	d	0.000124
KCNK2	7	potassium channel, subfamily K, member 2	210261_at	4	3.805	1.18	7	2.916	0.64	40	d	0.120287
NPEPPS	7	aminopeptidase puromycin sensitive	201455_s_at	7	9.234	0.14	7	8.776	0.76	40	d	0.001486
			201454_s_at	7	8.752	0.34	7	9.045	0.59	40	u	0.216883
			214101_s_at	6	4.86	0.53	7	5.007	0.94	40	u	0.697223
			242612_at	0	5.039	0.59	7	5.117	0.6	40	u	0.758661
PRKG1	7	protein kinase, cGMP-dependent, type I	207119_at	7	3.162	0.53	7	3.021	0.32	40	d	0.349358
			211380_s_at	4	2.283	0.21	7	2.376	0.31	40	u	0.455479
PERQ1	7	PERQ amino acid rich, with GYF domain 1	228755_at	3	5.236	0.6	7	5.186	0.78	40	d	0.874622
			226768_at	0	6.978	0.43	7	6.421	0.74	40	d	0.064149
SRPK2	7	SFRS protein kinase 2	203181_x_at	7	8.907	0.28	7	9.023	0.63	40	u	0.636848
			203182_s_at	7	8.76	0.31	7	8.934	0.78	40	u	0.567777
			1558254_s_at	0	6.965	0.42	7	7.376	1.19	40	u	0.122226
			214931_s_at	0	5.706	0.52	7	5.958	1.25	40	u	0.608861
			217517_x_at	0	4.238	0.2	7	4.592	0.39	40	u	0.027987
EDA	7	ectodysplasin A	206217_at	7	5.526	0.3	7	5.326	0.36	40	d	0.180403
			211127_x_at	0	2.625	0.45	7	2.414	0.13	40	d	0.30137
			211129_x_at	0	2.179	0.14	7	2.262	0.26	40	u	0.424161
			211128_at	0	2.408	0.3	7	2.248	0.14	40	d	0.245805
			211131_s_at	0	2.643	0.04	7	2.801	0.26	40	u	0.000883
			211130_x_at	0	2.351	0.14	7	2.411	0.24	40	u	0.530274
LIMCH1	7	LIM and calponin homology domains 1	212328_at	7	8.68	0.45	7	6.835	1.68	40	d	0.000002
			212325_at	7	7.116	0.53	7	6.145	1.81	40	d	0.011688
			212327_at	7	9.506	0.51	7	7.533	1.61	40	d	0.000002
			232457_at	0	5.687	0.68	7	5.185	0.65	40	d	0.074364
ELOVL6	7	ELOVL family member 6, elongation of long chain fatty acids (FEN1/Elo2, SUR4/Elo3-	204256_at	7	4.851	0.84	7	6.852	1.15	40	u	0.000088
			210868_s_at	7	3.568	0.19	7	4.5	1	40	u	0.000004
SH2B3	7	SH2B adaptor protein 3	203320_at	7	7.834	0.5	7	7.304	0.79	40	d	0.099775
KIAA1468	7	KIAA1468	225508_at	0	7.779	0.37	7	7.643	0.96	40	d	0.71923
			225506_at	0	8.557	0.27	7	8.029	0.78	40	d	0.004228
CALM3	7	calmodulin 3 (phosphorylase kinase, delta)	1563431_x_at	5	6.294	0.35	7	7.075	0.75	40	u	0.010856
			200622_x_at	5	7.646	0.35	7	8.503	0.88	40	u	0.016344
			200623_s_at	5	9.615	0.28	7	9.412	0.84	40	d	0.2564
C1orf121	7	chromosome 1 open reading frame 121	212371_at	7	9.041	0.25	7	9.834	0.84	40	u	0.000053
			219875_s_at	2	4.196	0.46	7	4.816	0.91	40	u	0.090959
			222936_s_at	2	2.466	0.04	7	2.954	0.77	40	u	0.000353
			222158_s_at	2	8.056	0.37	7	9.172	0.94	40	u	0.003917
			1558914_at	0	4.119	0.33	7	4.35	0.47	40	u	0.225892
MSTN	7	myostatin	207145_at	7	2.898	0.15	7	3.039	0.18	40	u	0.064404

ARHGDIA	7	Rho GDP dissociation inhibitor (GDI) alpha	201168_x_at	7	9.611	0.23	7	9.579	0.7	40	d	0.834367
			213606_s_at	7	6.441	0.54	7	7.455	1.61	40	u	0.006089
			211716_x_at	7	9.77	0.31	7	9.562	0.69	40	d	0.443432
			201167_x_at	0	6.954	0.6	7	7.847	1.41	40	u	0.114546
TMEM55A	7	transmembrane protein 55A	226338_at	7	5.306	0.26	7	5.217	1.03	40	d	0.653695
CLIP1	7	CAP-GLY domain containing linker protein 1	201975_at	7	9.091	0.4	7	8.464	0.53	40	d	0.00561
			210716_s_at	4	7.198	0.51	7	6.622	0.93	40	d	0.125592
			1558924_s_at	0	6.5	0.51	7	7.356	1	40	u	0.036202
			1568012_at	0	1.978	0.11	7	2.14	0.28	40	u	0.144813
			240444_x_at	0	2.743	0.14	7	2.779	0.17	40	u	0.612887
TLL1	7	tolloid-like 1	206415_at	7	2.804	0.09	7	3.001	0.27	40	u	0.00191
			1555071_at	0	2.283	0.05	7	2.496	0.38	40	u	0.002116
CD69	7	CD69 molecule	209795_at	7	4.957	0.95	7	5.219	1.72	40	u	0.703936
UBE2D2	7	ubiquitin-conjugating enzyme E2D 2 (UBC4/5 homolog, yeast)	201344_at	7	5.583	0.36	7	5.929	0.76	40	u	0.253384
			201343_at	7	8.927	0.19	7	9.322	0.49	40	u	0.047191
			201345_s_at	0	9.705	0.22	7	9.926	0.62	40	u	0.113198
PRKCA	7	protein kinase C, alpha	213093_at	7	6.181	0.41	7	5.251	1.3	40	d	0.001721
			1560074_at	4	3.127	0.18	7	3.122	0.35	40	d	0.972467
			215195_at	3	2.596	0.47	7	3.016	0.92	40	u	0.253452
			215194_at	3	2.995	0.43	7	2.836	0.24	40	d	0.407242
			206923_at	0	2.332	0.06	7	2.384	0.24	40	u	0.26199
SRF	7	serum response factor (c-fos serum response element-binding transcription	202400_s_at	7	3.853	0.65	7	4.172	1.03	40	u	0.440501
			202401_s_at	7	8.156	0.52	7	7.033	0.85	40	d	0.001736
SEMA4C	7	sema domain, immunoglobulin domain (Ig), transmembrane domain (TM) and short	219039_at	7	7.594	0.46	7	7.78	0.72	40	u	0.522283
			46665_at	7	9.068	0.23	7	9.143	0.76	40	u	0.631036
TOP1	7	topoisomerase (DNA) I	208900_s_at	7	7.5	0.29	7	8.581	0.74	40	u	0.000521
			208901_s_at	7	9.761	0.24	7	9.935	0.48	40	u	0.361756
PKNOX2	6	PBX/knotted 1 homeobox 2	222171_s_at	6	2.647	0.76	7	2.38	0.23	40	d	0.429384
			63305_at	6	3.112	0.36	7	3.218	0.42	40	u	0.53821
			222185_at	6	3.158	0.26	7	3.399	0.5	40	u	0.232564
			219046_s_at	5	2.479	0.18	7	2.681	0.33	40	u	0.129787
ZHX3	6	zinc fingers and homeoboxes 3	212545_s_at	6	3.786	0.29	7	3.795	0.38	40	u	0.950697
			217367_s_at	6	6.835	0.22	7	5.885	0.65	40	d	0
RAB1B	6	RAB1B, member RAS oncogene family	220964_s_at	6	9.251	0.09	7	8.963	0.79	40	d	0.033826
GALNT3	6	UDP-N-acetyl-alpha-D-galactosamine:polypeptide N-	203398_s_at	6	2.882	0.19	7	2.857	0.33	40	d	0.852667
			203397_s_at	6	9.438	0.35	7	8.808	1.68	40	d	0.045617
FAM134B	6	family with sequence similarity 134, member B	218532_s_at	6	7.566	0.61	7	7.323	1.28	40	d	0.631725
			218510_x_at	6	5.997	0.51	7	5.745	1.15	40	d	0.580664
MAX	6	MYC associated factor X	209332_s_at	6	10.16	0.24	7	9.603	0.52	40	d	0.009058
			209331_s_at	6	8.986	0.45	7	8.782	0.56	40	d	0.373831
			208403_x_at	0	5.598	0.85	7	5.169	1.03	40	d	0.314758
			210734_x_at	0	6.971	0.59	7	6.509	1.28	40	d	0.362961
			214108_at	0	2.442	0.08	7	2.592	0.14	40	u	0.008711
KIAA1199	6	KIAA1199	212942_s_at	6	3.341	0.57	7	5.334	1.33	40	u	0.000404
			1554685_a_at	1	2.353	0.29	7	2.573	0.31	40	u	0.093074
LHFPL2	6	lipoma HMGIC fusion partner-like 2	212658_at	6	9.003	0.34	7	8.197	0.64	40	d	0.002784
ROCK2	6	Rho-associated, coiled-coil containing protein kinase 2	202762_at	6	8.141	0.38	7	7.689	0.82	40	d	0.165918
			211504_x_at	0	6.252	0.2	7	6.485	0.52	40	u	0.254651
NCOR2	6	nuclear receptor co-repressor 2	207760_s_at	6	10.24	0.66	7	8.81	1.22	40	d	0.004904
			208889_s_at	6	4.809	0.41	7	4.324	0.59	40	d	0.047447
			208888_s_at	6	4.13	0.47	7	4.176	0.47	40	u	0.817932
			215205_x_at	1	4.586	0.47	7	3.859	0.76	40	d	0.021009
CCNE1	6	cyclin E1	213523_at	6	3.77	0.62	7	6.071	1.5	40	u	0.000313
			242105_at	0	2.514	0.12	7	2.589	0.18	40	u	0.309237
RNF4	6	ring finger protein 4	212696_s_at	6	9.173	0.25	7	8.973	0.58	40	d	0.380954
C5orf13	6	chromosome 5 open reading frame 13	201310_s_at	6	8.838	0.84	7	9.588	0.95	40	u	0.061726
			201309_x_at	2	5.83	0.86	7	7.363	1.5	40	u	0.013349
			230424_at	0	4.652	0.92	7	5.645	1.19	40	u	0.046382
DUSP5	6	dual specificity phosphatase 5	209457_at	4	8.84	0.64	7	7.741	1.45	40	d	0.060476
ATP11C	6	ATPase, Class VI, type 11C	226785_at	6	7.543	0.27	7	7.694	0.97	40	u	0.432971
			242690_at	0	2.686	0.07	7	3.462	0.95	40	u	0.000011
SLITRK4	6	SLIT and NTRK-like family, member 4	232636_at	1	3.429	0.68	7	3.238	0.78	40	d	0.554738
UBE2N	6	ubiquitin-conjugating enzyme E2N (UBC13 homolog, yeast)	212751_at	6	8.319	0.31	7	8.23	0.8	40	d	0.776707
			201523_x_at	3	8.895	0.36	7	9.262	0.88	40	u	0.292001

		homolog, yeast)	201524_x_at	3	10.25	0.24	7	10.6	0.52	40	u	0.09298
RAB38	6	RAB38, member RAS oncogene family	219412_at	6	4.909	0.59	7	4.258	1.5	40	d	0.274337
CASC4	6	cancer susceptibility candidate 4	224619_at	6	10.54	0.28	7	10.29	0.56	40	d	0.256098
			1552719_at	0	5.595	0.52	7	5.169	0.93	40	d	0.254401
			1559635_at	0	2.876	0.37	7	2.898	0.39	40	u	0.894026
SVIL	6	supervillin	202565_s_at	6	8.993	0.32	7	8.245	0.8	40	d	0.020799
			202566_s_at	0	7.975	0.41	7	6.881	1.11	40	d	0.015109
GRHL1	6	grainyhead-like 1 (Drosophila)	222830_at	6	9.659	0.46	7	9.058	1.14	40	d	0.185019
			1552685_a_at	0	7.193	0.64	7	6.444	1.69	40	d	0.262496
SET	6	SET translocation (myeloid leukemia-associated)	40189_at	6	10.79	0.16	7	10.97	0.47	40	u	0.092148
			200631_s_at	6	11.33	0.17	7	11.22	0.44	40	d	0.512789
			200630_x_at	6	11.2	0.2	7	11.34	0.35	40	u	0.318621
			213047_x_at	6	11.15	0.24	7	11.08	0.77	40	d	0.665316
			210231_x_at	1	10.98	0.23	7	11.08	0.4	40	u	0.523154
			215780_s_at	0	9.367	0.42	7	9.648	0.83	40	u	0.395169
KALRN	6	kalirin, RhoGEF kinase	206078_at	6	2.55	0.27	7	3.148	0.51	40	u	0.004731
			227750_at	0	5.926	0.38	7	5.921	0.75	40	d	0.987755
			236651_at	0	3.247	0.14	7	3.472	0.43	40	u	0.020011
			232717_at	0	2.659	0.2	7	2.719	0.32	40	u	0.640956
			205635_at	0	2.409	0.14	7	2.726	0.67	40	u	0.012779
			232756_at	0	3.431	0.12	7	3.664	0.29	40	u	0.043265
NCK2	6	NCK adaptor protein 2	203315_at	6	9.477	0.2	7	9.254	0.88	40	d	0.182235
PODXL	6	podocalyxin-like	201578_at	6	10.17	0.51	7	9.2	1.37	40	d	0.075352
FAM122B	6	family with sequence similarity 122B	225361_x_at	6	7.775	0.41	7	7.671	1.04	40	d	0.800172
			225362_at	6	4.995	0.46	7	5.549	0.95	40	u	0.146674
			222673_x_at	0	7.799	0.4	7	7.769	1.06	40	d	0.941937
CDH11	6	cadherin 11, type 2, OB-cadherin (osteoblast)	207173_x_at	6	8.802	0.31	7	8.609	1.19	40	d	0.403799
			207172_s_at	1	7.791	0.28	7	7.679	1.12	40	d	0.604747
			239769_at	0	2.904	0.51	7	3.11	0.58	40	u	0.391877
C21orf25	6	chromosome 21 open reading frame 25	212875_s_at	6	8.141	0.38	7	7.393	1.01	40	d	0.063819
DUSP10	6	dual specificity phosphatase 10	221563_at	5	7.771	0.72	7	7.29	1.42	40	d	0.39649
			215501_s_at	0	7.502	0.7	7	7.649	1.22	40	u	0.761742
CBX2	6	chromobox homolog 2 (Pc class homolog, Drosophila)	226473_at	6	3.444	0.16	7	6.622	1.62	40	u	0
			215989_at	0	2.375	0.06	7	2.923	0.68	40	u	0.000017
			224138_at	0	4.99	0.21	7	5.116	0.21	40	u	0.160744
C11orf57	6	chromosome 11 open reading frame 57	222588_s_at	6	7.173	0.26	7	6.706	0.66	40	d	0.075817
			218314_s_at	6	7.076	0.28	7	6.837	0.59	40	d	0.309441
			1555803_a_at	2	6.993	0.25	7	6.658	0.65	40	d	0.195731
			230724_s_at	2	2.902	0.51	7	3.387	0.76	40	u	0.116094
			1560029_a_at	0	4.347	0.65	7	4.383	0.62	40	u	0.890063
			1560028_at	0	2.511	0.15	7	2.746	0.38	40	u	0.119513
FBRS	6	fibrosin	218255_s_at	6	8.037	0.23	7	7.781	0.64	40	d	0.077845
			242217_s_at	2	7.449	0.23	7	7.43	0.92	40	d	0.913364
			238771_at	0	2.32	0.23	7	2.2	0.23	40	d	0.227696
CENTG2	6	centaurin, gamma 2	204066_s_at	6	7.876	0.47	7	7.429	1.01	40	d	0.267871
			235968_at	0	2.066	0.13	7	2.127	0.2	40	u	0.443398
FAM73B	6	family with sequence similarity 73, member B	226356_at	6	6.329	0.21	7	5.686	0.76	40	d	0.000144
DUSP3	6	dual specificity phosphatase 3 (vaccinia virus phosphatase VH1-related)	201536_at	6	9.063	0.22	7	8.152	0.6	40	d	0.000336
			201537_s_at	2	6.526	0.58	7	6.493	0.9	40	d	0.926381
			201538_s_at	0	5.196	0.72	7	5.022	1.25	40	d	0.727552
MIER2	6	mesoderm induction early response 1, family member 2	221863_at	6	6.062	0.39	7	5.734	0.57	40	d	0.162923
			221862_at	6	2.779	0.36	7	3.039	0.24	40	u	0.020826
			44822_s_at	6	5.685	0.38	7	5.651	0.53	40	d	0.875865
MESDC1	6	mesoderm development candidate 1	223264_at	6	8.451	0.47	7	8.698	0.71	40	u	0.388163
API5	6	apoptosis inhibitor 5	201687_s_at	6	9.08	0.35	7	9.188	0.59	40	u	0.647709
			214960_at	0	6.112	0.41	7	6.041	0.79	40	d	0.820858
			201686_x_at	0	6.092	0.58	7	6.799	1	40	u	0.083325
			214959_s_at	0	6.011	0.48	7	6.454	1.27	40	u	0.376127
			233078_at	0	5.941	0.3	7	6.261	0.83	40	u	0.090791
FAM117A	6	family with sequence similarity 117, member A	221249_s_at	6	8.075	0.45	7	6.883	0.94	40	d	0.002448
			201047_x_at	6	10.3	0.14	7	10.54	0.61	40	u	0.038255
			201045_s_at	6	5.373	0.26	7	5.459	0.64	40	u	0.735774

RAB6A	6	RAB6A, member RAS oncogene family	210406_s_at	4	10.66	0.2	7	10.91	0.62	40	u	0.066384
			201048_x_at	1	4.373	0.93	7	5.265	1.53	40	u	0.149597
			1559174_at	0	4.77	0.38	7	5.15	0.61	40	u	0.123791
PCNP	6	PEST proteolytic signal containing nuclear protein	217816_s_at	6	11.05	0.14	7	10.52	0.49	40	d	0.000007
			1554868_s_at	6	9.885	0.47	7	9.477	1.03	40	d	0.317787
			237577_at	0	4.234	0.35	7	4.682	0.59	40	u	0.063774
TRAK2	6	trafficking protein, kinesin binding 2	202124_s_at	6	7.766	0.52	7	7.659	0.67	40	d	0.699299
			202125_s_at	6	8.491	0.29	7	7.855	0.73	40	d	0.031597
ZHX2	6	zinc fingers and homeoboxes 2	203556_at	6	8.749	0.43	7	8.436	0.77	40	d	0.312253
			1557706_at	0	3.496	0.45	7	3.473	0.56	40	d	0.918481
CD28	6	CD28 molecule	206545_at	6	2.847	0.47	7	3.042	0.76	40	u	0.523095
			211856_x_at	0	2.343	0.11	7	2.419	0.27	40	u	0.470527
			211861_x_at	0	2.457	0.09	7	2.547	0.13	40	u	0.083049
RPS6KA4	6	ribosomal protein S6 kinase, 90kDa, polypeptide 4	204632_at	6	6.964	0.35	7	7.245	0.48	40	u	0.155477
			230544_at	0	2.286	0.03	7	2.381	0.12	40	u	0.000112
BAGE3	6	B melanoma antigen family, member 3	no probeset available									
SKI	6	v-ski sarcoma viral oncogene homolog (avian)	204270_at	6	3.711	0.61	7	4.162	1.08	40	u	0.300413
TMEM50B	6	transmembrane protein 50B	225182_at	6	9.633	0.24	7	9.016	0.65	40	d	0.020049
			222907_x_at	3	8.021	0.33	7	8.21	0.61	40	u	0.441485
			219600_s_at	2	8.935	0.52	7	8.975	0.95	40	u	0.915445
C1orf108	6	chromosome 1 open reading frame 108	217893_s_at	6	8.039	0.4	7	8.195	0.69	40	u	0.573261
			222459_at	6	7.288	0.44	7	7.622	0.69	40	u	0.232316
			222458_s_at	6	7.404	0.62	7	7.734	1.29	40	u	0.519487
BCAT2	6	branched chain aminotransferase 2, mitochondrial	203576_at	6	7.66	0.36	7	7.178	0.74	40	d	0.105008
			215654_at	0	2.461	0.04	7	2.56	0.21	40	u	0.013596
ABCC5	6	ATP-binding cassette, sub-family C (CFTR/MRP), member 5	209380_s_at	6	6.694	0.46	7	7.088	1.23	40	u	0.419093
			1558460_at	0	3.129	0.35	7	3.419	0.72	40	u	0.313543
			226363_at	0	5.678	0.42	7	5.558	1.68	40	d	0.709939
TBX3	6	T-box 3 (ulnar mammary syndrome)	222917_s_at	6	5.288	0.4	7	5.192	1.02	40	d	0.810912
			219682_s_at	6	9.212	0.53	7	7.332	1.78	40	d	0.000011
			225544_at	6	9.069	0.6	7	7.648	1.35	40	d	0.010514
			228344_s_at	3	2.045	0.03	7	2.072	0.06	40	u	0.247949
			229576_s_at	3	6.78	0.56	7	5.588	1.67	40	d	0.00239
			229565_x_at	3	2.18	0.02	7	2.235	0.06	40	u	0.000179
			243234_at	0	3.278	0.24	7	3.555	0.4	40	u	0.085994
PRX	6	periaxin	220024_s_at	6	5.865	0.4	7	5.348	0.4	40	d	0.003436
			222255_at	6	2.137	0.02	7	2.179	0.04	40	u	0.007859
SYNE1	6	spectrin repeat containing, nuclear envelope 1	209447_at	4	6.449	0.51	7	4.834	1.11	40	d	0.000591
			244070_at	0	2.363	0.06	7	2.501	0.36	40	u	0.028588
			215350_at	0	2.816	0.56	7	2.78	0.34	40	d	0.824053
			244144_at	0	4.021	0.27	7	3.851	0.28	40	d	0.15411
			232027_at	0	2.164	0.03	7	2.234	0.18	40	u	0.030986
WDR44	6	WD repeat domain 44	219297_at	6	7.259	0.11	7	7.007	0.81	40	d	0.072871
			235485_at	0	2.722	0.42	7	2.604	0.27	40	d	0.349424
THRA	6	thyroid hormone receptor, alpha (erythroblastic leukemia viral (v-erb-a) oncogene homolog, avian)	214883_at	6	2.783	0.42	7	2.823	0.33	40	u	0.783149
			1316_at	6	5.235	0.25	7	5.367	0.6	40	u	0.575826
			204100_at	1	4.968	0.58	7	4.392	0.78	40	d	0.074508
			35846_at	0	7.072	0.2	7	6.205	0.76	40	d	0.000001
TMEM64	6	transmembrane protein 64	225974_at	6	7.032	0.27	7	7.415	1.56	40	u	0.166645
			225972_at	6	6.918	0.36	7	6.818	1.24	40	d	0.686729
			242338_at	2	4.195	0.7	7	4.809	1.67	40	u	0.353608
NEUROD6	6	neurogenic differentiation 6	220045_at	1	2.31	0.03	7	2.45	0.2	40	u	0.000265
ITCH	6	itchy homolog E3 ubiquitin protein ligase (mouse)	236235_at	6	4.97	0.63	7	5.15	0.82	40	u	0.59237
			235057_at	6	4.13	0.69	7	4.3	0.86	40	u	0.628847
			239101_at	6	5.296	0.31	7	5.008	0.45	40	d	0.12125
			209744_x_at	1	5.244	0.23	7	7.029	0.8	40	u	0
			209743_s_at	1	4.124	0.25	7	5.802	0.98	40	u	0
			217094_s_at	0	5.654	0.44	7	7.143	0.68	40	u	0.000002
BMI1	6	BMI1 polycomb ring finger oncogene	202265_at	6	9.729	0.16	7	9.961	0.79	40	u	0.113495
UBE2V1	6	ubiquitin-conjugating enzyme E2 variant 1	no probeset available									
SCRT1	6	scratch homolog 1, zinc finger protein (Drosophila)	221026_s_at	0	2.002	0.01	7	2.066	0.09	40	u	0.000132
			228761_at	0	2.33	0.02	7	2.502	0.32	40	u	0.001845
SH3GL2	6	SH3-domain GRB2-like 2	205751_at	6	2.349	0.11	7	2.545	0.55	40	u	0.054218

SAMD4B	6	sterile alpha motif domain containing 4B	226714_at	6	8.146	0.34	7	7.998	0.7	40	d	0.595015
			220457_at	1	4.381	0.29	7	4.773	0.8	40	u	0.218729
			229871_at	0	8.487	0.5	7	7.437	1.06	40	d	0.015801
			227511_at	0	6.933	0.35	7	6.831	0.94	40	d	0.784303
MAP2K3	6	mitogen-activated protein kinase kinase 3	215499_at	6	8.535	0.33	7	7.954	0.58	40	d	0.014609
			207667_s_at	6	7.918	0.49	7	7.347	0.54	40	d	0.014274
			215498_s_at	6	8.374	0.61	7	7.895	0.67	40	d	0.088911
CCDC126	6	coiled-coil domain containing 126	228087_at	0	6.284	0.54	7	5.703	1.06	40	d	0.172168
			228061_at	0	5.294	0.65	7	5.128	0.97	40	d	0.671165
NDEL1	6	nudE nuclear distribution gene E homolog (A. nidulans)-like 1	208093_s_at	6	8.897	0.27	7	8.325	0.54	40	d	0.010717
			227553_at	0	2.152	0.2	7	2.185	0.17	40	u	0.652721
BAIAP2	6	BAI1-associated protein 2	205294_at	6	5.981	0.28	7	6.081	0.67	40	u	0.705261
			205293_x_at	3	3.366	0.51	7	3.247	0.91	40	d	0.742741
			209502_s_at	0	5.908	0.48	7	5.679	0.98	40	d	0.554135
			207832_at	0	2.611	0.23	7	2.588	0.14	40	d	0.818906
TMEM110	6	transmembrane protein 110	213851_at	6	5.62	0.31	7	5.844	0.5	40	u	0.270385
			227127_at	0	6.322	0.34	7	5.721	0.62	40	d	0.019131
			227078_at	0	6.146	0.2	7	5.737	0.68	40	d	0.004869
STARD7	6	StAR-related lipid transfer (START) domain containing 7	200028_s_at	6	11.1	0.22	7	11.19	0.43	40	u	0.600558
HECW1	6	HECT, C2 and WW domain containing E3 ubiquitin protein ligase 1	210331_at	6	2.227	0.02	7	2.35	0.16	40	u	0.000033
			215584_at	0	4.658	0.29	7	4.612	0.39	40	d	0.768722
ERBB4	6	v-erb-a erythroblastic leukemia viral oncogene homolog 4 (avian)	206794_at	6	4.627	0.55	7	4.503	0.92	40	d	0.735154
			214053_at	0	8.102	0.98	7	4.903	2.7	40	d	0.000016
			233498_at	0	8.947	1.56	7	6.448	2.4	40	d	0.012421
			233494_at	0	2.32	0.05	7	2.606	0.34	40	u	0.00001
GPHN	6	gephyrin	223319_at	6	6.44	0.26	7	5.375	1.25	40	d	0.000026
			220773_s_at	0	5.674	0.55	7	4.933	1.15	40	d	0.109039
			234941_s_at	0	5.076	0.21	7	4.624	0.75	40	d	0.004087
TFDP2	6	transcription factor Dp-2 (E2F dimerization partner 2)	203588_s_at	6	6.95	0.51	7	7.662	1.09	40	u	0.104265
			203589_s_at	6	4.825	0.42	7	5.26	0.8	40	u	0.173435
			226157_at	0	8.219	0.45	7	6.696	0.94	40	d	0.000164
FOXQ1	6	forkhead box Q1	227475_at	6	3.874	1.42	7	3.901	1.5	40	u	0.96549
PTPRE	6	protein tyrosine phosphatase, receptor type, E	221840_at	6	8.365	0.47	7	6.449	0.86	40	d	0.000001
			233978_at	0	2.529	0.06	7	2.575	0.14	40	u	0.408343
			1559018_at	0	2.942	0.31	7	3.059	0.39	40	u	0.464953
SFPQ	6	splicing factor proline/glutamine-rich (polypyrimidine tract binding protein associated)	201585_s_at	3	8.104	0.15	7	8.108	0.49	40	u	0.96235
			201586_s_at	3	10.25	0.25	7	10.5	0.46	40	u	0.174581
			221768_at	0	10.41	0.41	7	8.871	1.13	40	d	0.000002
			214016_s_at	0	10.97	0.33	7	9.595	0.96	40	d	0.000001
			226898_s_at	0	2.621	0.23	7	3.322	0.86	40	u	0.000171
TMEM127	6	transmembrane protein 127	219460_s_at	6	8.053	0.32	7	7.79	0.49	40	d	0.189809
			222887_s_at	6	6.929	0.38	7	6.698	0.66	40	d	0.38262
CACNA1E	6	calcium channel, voltage-dependent, R type, alpha 1E subunit	236013_at	6	2.273	0.05	7	2.295	0.07	40	u	0.436541
			240650_at	0	1.96	0.09	7	2.012	0.18	40	u	0.473841
			208432_s_at	0	2.22	0.21	7	2.263	0.27	40	u	0.695574
			242410_s_at	0	2.671	0.44	7	2.648	0.15	40	d	0.901855
			244256_at	0	3.544	0.18	7	3.584	0.2	40	u	0.636181
PGRMC2	6	progesterone receptor membrane component 2	201701_s_at	6	8.643	0.35	7	8.565	0.62	40	d	0.750976
			213227_at	6	8.916	0.41	7	8.336	0.77	40	d	0.06452
C14orf28	6	chromosome 14 open reading frame 28	235369_at	6	6.941	0.35	7	5.646	0.67	40	d	0.000012
			238647_at	2	4.949	0.55	7	4.118	0.9	40	d	0.025398
SLC25A1	6	solute carrier family 25 (mitochondrial carrier; citrate transporter), member 1	210010_s_at	6	8.669	0.33	7	8.58	0.89	40	d	0.797007
FAM133B	6	family with sequence similarity 133, member B	226332_at	6	8.611	0.36	7	8.457	0.42	40	d	0.382211
			235469_at	0	6.247	0.44	7	6.626	0.65	40	u	0.155184
PI4K2A	6	phosphatidylinositol 4-kinase type 2 alpha	215134_at	6	2.451	0.18	7	2.479	0.27	40	u	0.79625
			209345_s_at	6	7.15	0.18	7	6.834	0.52	40	d	0.009313
			209346_s_at	2	5.336	0.25	7	5.501	0.56	40	u	0.453854
TEX261	6	testis expressed 261	212083_at	6	9.337	0.18	7	9.046	0.47	40	d	0.119635
			212084_at	6	5.643	0.36	7	5.692	0.62	40	u	0.843158
RNF145	6	ring finger protein 145	226077_at	6	10.03	0.38	7	9.32	1.02	40	d	0.083341
			239899_at	0	1.885	0.04	7	2.003	0.47	40	u	0.130527
[illegible]	[illegible]	family with sequence similarity 135, member [illegible]	223497_at	6	6.764	0.25	7	6.465	1.03	40	d	0.130384

FAM135A	6	family with sequence similarity 135, member A	233730_at	0	3.68	0.23	7	4.02	0.53	40	u	0.10699
			221233_s_at	0	2.571	0.08	7	2.758	0.28	40	u	0.001769
GPR64	6	G protein-coupled receptor 64	206002_at	6	2.565	0.43	7	3.433	1.91	40	u	0.017816
EFNB3	6	ephrin-B3	205031_at	6	6.263	0.3	7	4.402	1.27	40	d	0
			210883_x_at	0	3.969	0.59	7	4.127	0.43	40	u	0.41481
STOX2	6	storkhead box 2	231969_at	6	4.418	0.71	7	4.298	1.35	40	d	0.82468
			234319_at	5	2.518	0.03	7	2.536	0.07	40	u	0.495165
			234317_s_at	5	4.372	0.56	7	4.235	1.07	40	d	0.74768
			226822_at	0	6.405	0.52	7	6.005	0.88	40	d	0.26022
GRSF1	6	G-rich RNA sequence binding factor 1	201501_s_at	0	8.272	0.22	7	8.85	0.72	40	u	0.000423
			221917_s_at	0	3.129	0.36	7	3.44	0.65	40	u	0.236709
			201520_s_at	0	9.658	0.21	7	10.14	0.67	40	u	0.001368
			215030_at	0	6.105	0.18	7	6.661	1.03	40	u	0.003492
CNR1	6	cannabinoid receptor 1 (brain)	213436_at	6	2.944	0.51	7	3.044	1.44	40	u	0.751646
			1560225_at	0	2.299	0.11	7	2.5	0.84	40	u	0.159454
			208243_s_at	0	2.098	0.02	7	2.25	0.35	40	u	0.009803
			207940_x_at	0	2.336	0.1	7	2.455	0.4	40	u	0.128706
CC2D1A	6	coiled-coil and C2 domain containing 1A	221888_at	6	7.239	0.44	7	6.079	0.63	40	d	0.000039
			58994_at	6	9.177	0.32	7	8.184	0.59	40	d	0.000102
			207083_s_at	0	2.771	0.33	7	2.87	0.66	40	u	0.706111
			222137_at	0	3.291	0.49	7	3.51	0.71	40	u	0.449398
TMPO	6	thymopoietin	209754_s_at	3	4.635	0.62	7	6.244	1.64	40	u	0.015968
			209753_s_at	3	4.755	0.36	7	6.298	1.1	40	u	0
			224944_at	0	9.187	0.2	7	10.06	0.69	40	u	0
			203432_at	0	6.885	0.21	7	8.427	0.87	40	u	0
UBR5	6	ubiquitin protein ligase E3 component n-recognin 5	208882_s_at	4	8.633	0.32	7	8.931	0.91	40	u	0.138318
			208883_at	4	7.667	0.27	7	7.478	0.92	40	d	0.31558
			208884_s_at	0	9.581	0.19	7	9.619	0.84	40	u	0.81043
			1555888_at	0	5.252	0.23	7	5.759	1.17	40	u	0.019758
PITX2	6	paired-like homeodomain 2	207558_s_at	6	3.347	0.14	7	3.745	0.6	40	u	0.000965
BOLA2	6	bolA homolog 2 (E. coli)	210396_s_at	3	8.336	0.42	7	9.178	0.78	40	u	0.008738
			209836_x_at	3	8.376	0.28	7	9.523	0.69	40	u	0.000114
			231500_s_at	3	3.837	0.27	7	4.608	0.69	40	u	0.006613
			241644_at	0	5.228	0.18	7	5.171	0.35	40	d	0.680865
MOBKL3	6	MOB1, Mps One Binder kinase activator-like 3 (yeast)	202919_at	6	8.943	0.35	7	8.903	0.61	40	d	0.870244
			202918_s_at	3	8.472	0.36	7	9.305	0.61	40	u	0.001258
ELN	6	elastin (supravalvular aortic stenosis, Williams-Beuren syndrome)	212670_at	6	6.946	0.79	7	5.596	1.74	40	d	0.054558
			216269_s_at	0	5.542	0.58	7	5.507	0.87	40	d	0.921407
ARHGEF3	6	Rho guanine nucleotide exchange factor (GEF) 3	218501_at	6	8.453	0.29	7	7.603	1.1	40	d	0.000302
ARHGEF18	6	rho/rac guanine nucleotide exchange factor (GEF) 18	213039_at	6	7.907	0.28	7	7.684	0.7	40	d	0.423332
CCNJL	6	cyclin J-like	219227_at	6	3.463	0.31	7	3.069	0.62	40	d	0.114681
UBE2B	6	ubiquitin-conjugating enzyme E2B (RAD6 homolog)	211763_s_at	6	8.414	0.38	7	8.115	0.62	40	d	0.232231
			202333_s_at	6	9.964	0.22	7	9.813	0.44	40	d	0.391359
			202334_s_at	6	8.76	0.36	7	8.447	0.65	40	d	0.232006
			202335_s_at	6	3.92	0.45	7	3.723	0.41	40	d	0.267188
			228588_s_at	1	7.992	0.78	7	8.571	0.86	40	u	0.109545
			239163_at	0	4.077	0.22	7	4.461	0.71	40	u	0.011962
			224118_at	0	2.26	0.22	7	2.241	0.23	40	d	0.842291
KHDRBS2	6	KH domain containing, RNA binding, signal transduction associated 2	215527_at	1	2.365	0.06	7	2.542	0.17	40	u	0.009815
PDIK1L	6	PDLIM1 interacting kinase 1 like	227255_at	6	5.836	0.37	7	6.128	0.72	40	u	0.310119
ZRANB2	6	zinc finger, RAN-binding domain containing 2	223716_s_at	6	11.06	0.24	7	10.46	0.51	40	d	0.005149
			223016_x_at	0	8.698	0.27	7	8.797	0.7	40	u	0.721818
			241345_at	0	3.103	0.32	7	3.577	0.62	40	u	0.059288
BRD1	6	bromodomain containing 1	215460_x_at	6	7.795	0.35	7	7.247	0.58	40	d	0.021087
			204520_x_at	6	7.929	0.38	7	7.363	0.54	40	d	0.01249
CA7	6	carbonic anhydrase VII	207504_at	6	2.345	0.21	7	2.506	0.37	40	u	0.27731
WDR47	6	WD repeat domain 47	203855_at	6	7.444	0.29	7	7.867	0.61	40	u	0.083144
CCDC131	6	coiled-coil domain containing 131	213065_at	6	8.843	0.21	7	8.212	0.94	40	d	0.000798
			1553736_at	0	2.936	0.2	7	3.334	0.56	40	u	0.003129
RBM12	6	RNA binding motif protein 12	212168_at	6	8.772	0.33	7	9.115	0.47	40	u	0.075479
			212170_at	6	7.289	0.38	7	6.954	0.63	40	d	0.192587

CACNB4	6	calcium channel, voltage-dependent, beta 4 subunit	207693_at	1	2.547	0.08	7	2.856	0.64	40	u	0.006426
UBL3	6	ubiquitin-like 3	201534_s_at	6	9.346	0.43	7	8.845	0.67	40	d	0.068669
			201535_at	6	9.323	0.32	7	8.163	0.75	40	d	0.000293
BCORL1	6	BCL6 co-repressor-like 1	219444_at	6	6.346	0.77	7	6.602	0.79	40	u	0.441187
			233572_s_at	0	4.134	0.44	7	4.519	0.58	40	u	0.110948
			234711_s_at	0	2.441	0.18	7	2.507	0.39	40	u	0.66364
			233574_at	0	1.788	0.02	7	1.82	0.03	40	u	0.014435
AXIN2	6	axin 2 (conductin, axil)	222695_s_at	6	2.254	0.39	7	2.17	0.32	40	d	0.546459
			222696_at	6	5.792	0.34	7	4.597	0.78	40	d	0.000333
			224498_x_at	6	3.908	0.33	7	3.924	0.38	40	u	0.920762
			224176_s_at	0	2.534	0.21	7	2.447	0.28	40	d	0.451965
FGF2	6	fibroblast growth factor 2 (basic)	204422_s_at	6	6.078	0.95	7	3.526	0.9	40	d	0
			204421_s_at	0	4.316	1.08	7	3.161	0.67	40	d	0.000559
PHYHIPL	6	phytanoyl-CoA 2-hydroxylase interacting protein-like	226623_at	6	2.562	0.09	7	2.845	0.58	40	u	0.007323
AP3M1	6	adaptor-related protein complex 3, mu 1 subunit	222517_at	6	5.906	0.76	7	5.323	0.94	40	d	0.137
			222516_at	6	8.308	0.29	7	8.194	0.85	40	d	0.530974
CAV1	6	caveolin 1, caveolae protein, 22kDa	212097_at	6	11.33	0.5	7	8.57	1.17	40	d	0
			203065_s_at	1	10.84	0.52	7	7.719	1.19	40	d	0
PATZ1	6	POZ (BTB) and AT hook containing zinc finger 1	209431_s_at	6	7.822	0.34	7	7.446	0.91	40	d	0.294484
			211392_s_at	0	6.266	0.53	7	5.983	1.22	40	d	0.558253
			210581_x_at	0	4.529	0.89	7	4.265	1	40	d	0.523759
			211393_at	0	2.915	0.29	7	2.985	0.28	40	u	0.553545
			209494_s_at	0	7.438	0.56	7	6.721	0.9	40	d	0.052385
			211391_s_at	0	4.863	0.68	7	4.305	1.24	40	d	0.261833
CNTNAP2	6	contactin associated protein-like 2	219300_s_at	6	5.174	0.78	7	5.616	2.21	40	u	0.362524
			219301_s_at	6	4.192	0.53	7	4.536	1.29	40	u	0.500716
			219302_s_at	1	2.889	0.23	7	3.328	1.02	40	u	0.025666
			215145_s_at	0	2.772	0.76	7	3.359	1.75	40	u	0.39655
CDK5R1	6	cyclin-dependent kinase 5, regulatory subunit 1 (p35)	204995_at	6	3.214	0.37	7	4.197	0.85	40	u	0.005019
			204996_s_at	0	1.782	0.03	7	1.807	0.03	40	u	0.092794
HIF1A	6	hypoxia-inducible factor 1, alpha subunit (basic helix-loop-helix transcription factor)	200989_at	6	10.68	0.35	7	10.88	0.72	40	u	0.483072
EDEM1	6	ER degradation enhancer, mannosidase alpha-like 1	203279_at	6	8.213	0.25	7	7.483	0.89	40	d	0.00023
			230659_at	5	5.476	0.19	7	5.658	0.93	40	u	0.281958
FLRT2	6	fibronectin leucine rich transmembrane protein 2	204359_at	6	8.273	0.87	7	5.667	1.1	40	d	0.000001
			204358_s_at	0	4.392	0.42	7	4.257	0.22	40	d	0.466944
ERF	6	Ets2 repressor factor	203643_at	6	7.653	0.28	7	7.523	0.71	40	d	0.641909
			230368_at	0	6.136	0.15	7	5.657	0.78	40	d	0.001414
NIPA1	6	non imprinted in Prader-Willi/Angelman syndrome 1	225752_at	6	5.675	0.3	7	5.863	0.9	40	u	0.326505
			1552696_at	0	3.675	0.25	7	3.963	0.5	40	u	0.149593
LMO3	6	LIM domain only 3 (rhombotin-like 2)	231348_s_at	6	2.17	0.1	7	2.552	0.8	40	u	0.006833
			204424_s_at	6	3.399	0.6	7	4.848	2.15	40	u	0.001647
			1565937_a_at	0	2.488	0.04	7	2.801	0.55	40	u	0.001168
			1565936_a_at	0	2.96	0.1	7	3.097	0.27	40	u	0.201123
RANBP10	6	RAN binding protein 10	221809_at	6	6.03	0.2	7	5.364	0.71	40	d	0.000035
			53987_at	6	6.636	0.23	7	5.928	0.62	40	d	0.005746
			1558773_s_at	6	2.038	0.03	7	2.133	0.23	40	u	0.01571
			232329_at	0	2.28	0.41	7	2.134	0.08	40	d	0.421363
CDV3	6	CDV3 homolog (mouse)	228746_s_at	6	7.034	0.52	7	6.643	1.01	40	d	0.33381
			212010_s_at	6	11.48	0.27	7	11.22	0.59	40	d	0.260808
			213548_s_at	2	6.898	0.89	7	6.955	1.69	40	u	0.932148
			213554_s_at	2	7.649	0.48	7	8.01	0.88	40	u	0.306231
MYCBP	6	c-myc binding protein	203359_s_at	6	8.501	0.38	7	8.488	0.69	40	d	0.962111
			203361_s_at	6	5.954	0.35	7	6.176	0.66	40	u	0.399087
			203360_s_at	1	7.347	0.54	7	7.794	0.86	40	u	0.201599
VANGL1	6	vang-like 1 (van gogh, Drosophila)	219330_at	2	5.835	0.24	7	7.037	0.96	40	u	0
			229492_at	0	4.532	0.67	7	5.785	1.21	40	u	0.011936
			229134_at	0	4.08	0.56	7	4.454	0.87	40	u	0.288913
			229997_at	0	6.977	0.24	7	8.02	0.8	40	u	0
PPM1F	6	protein phosphatase 1F (PP2C domain containing)	37384_at	6	7.236	0.51	7	6.146	0.91	40	d	0.004121
			203063_at	6	8.099	0.5	7	7.154	0.77	40	d	0.003721
			1555091_at	0	2.685	0.16	7	2.851	0.28	40	u	0.148595

			1555470_a_at	0	5.26	0.18	7	5.52	0.39	40	u	0.100758
E2F6	6	E2F transcription factor 6	203957_at	6	6.48	0.41	7	7.072	0.58	40	u	0.015153
EFNA3	6	ephrin-A3	210132_at	6	6.871	0.46	7	7.631	1.09	40	u	0.083533
RSRC2	6	arginine/serine-rich coiled-coil 2	202301_s_at	6	8.628	0.32	7	8.584	0.46	40	d	0.812093
			202302_s_at	2	10.63	0.16	7	10.01	0.42	40	d	0.000443
MAP2K1	6	mitogen-activated protein kinase kinase 1	202670_at	6	8.988	0.28	7	9.11	0.79	40	u	0.480844
PPP2R5E	6	protein phosphatase 2, regulatory subunit B', epsilon isoform	203338_at	6	8.342	0.28	7	8.779	0.53	40	u	0.041174
			229322_at	0	8.738	0.25	7	8.883	0.57	40	u	0.51902
			231101_at	0	4.162	0.47	7	4.784	0.79	40	u	0.052995
LMNA	6	lamin A/C	212086_x_at	6	10.08	0.37	7	9.089	0.93	40	d	0.009409
			212089_at	6	9.731	0.33	7	8.827	0.92	40	d	0.000162
			214213_x_at	3	8.639	0.18	7	7.915	0.71	40	d	0.000006
			203411_s_at	0	10.92	0.45	7	10.21	0.96	40	d	0.068976
			1554600_s_at	0	9.446	0.58	7	8.647	1.18	40	d	0.092234
KPNB1	6	karyopherin (importin) beta 1	208975_s_at	6	8.864	0.2	7	9.702	0.54	40	u	0.000238
			213507_s_at	1	11.15	0.18	7	11.3	0.37	40	u	0.321223
			217027_x_at	0	6.335	0.08	7	6.76	0.44	40	u	0.000002
			208974_x_at	0	10.67	0.14	7	11.25	0.39	40	u	0.000001
			213803_at	0	7.578	0.22	7	8.036	0.72	40	u	0.003964
			213574_s_at	0	9.096	0.25	7	9.457	0.64	40	u	0.158494
FOXO3	6	forkhead box O3	204131_s_at	6	10.63	0.23	7	9.72	0.88	40	d	0.000005
			204132_s_at	4	10.03	0.35	7	9.16	1.06	40	d	0.00058
			217399_s_at	0	5.437	0.31	7	5.306	0.83	40	d	0.688952
			231548_at	0	5.38	0.39	7	5.46	0.58	40	u	0.732479
			224891_at	0	10.61	0.34	7	10.09	0.81	40	d	0.110084
			210655_s_at	0	8.866	0.45	7	7.772	1.6	40	d	0.001463
			224889_at	0	8.996	0.35	7	8.638	0.84	40	d	0.280469
FAM70A	6	family with sequence similarity 70, member A	219895_at	6	4.637	1.07	7	2.911	0.52	40	d	0.008187
ST7	6	suppression of tumorigenicity 7	207871_s_at	0	6.666	0.16	7	6.722	0.65	40	u	0.655322
			207524_at	0	5.61	0.27	7	5.597	0.5	40	d	0.948071
ATG16L1	6	ATG16 autophagy related 16-like 1 (S. cerevisiae)	232612_s_at	6	7.315	0.4	7	6.718	0.72	40	d	0.040738
			220521_s_at	0	5.937	0.56	7	5.846	1.06	40	d	0.828154
KIAA1267	6	KIAA1267	225117_at	6	9.557	0.44	7	8.95	0.52	40	d	0.007017
			224489_at	0	3.693	0.89	7	3.178	0.64	40	d	0.078876
			243589_at	0	6.992	1.07	7	6.84	1.16	40	d	0.751996
			224490_s_at	0	4.282	0.75	7	3.71	0.4	40	d	0.118654
CRKL	6	v-crk sarcoma virus CT10 oncogene homolog (avian)-like	212180_at	6	8.423	0.28	7	8.32	0.47	40	d	0.582216
			206184_at	1	3.732	0.56	7	4.401	1.21	40	u	0.166909
TCBA1	6	T-cell lymphoma breakpoint associated target 1	242002_at	6	3.142	0.16	7	3.275	0.32	40	u	0.301978
			1554601_at	0	2.202	0.19	7	2.199	0.08	40	d	0.971193
BTG3	6	BTG family, member 3	213134_x_at	6	8.959	0.36	7	9.097	1.37	40	u	0.60285
			205548_s_at	5	7.902	0.43	7	7.869	1.46	40	d	0.910118
			1556213_a_at	0	2.921	0.07	7	3.05	0.39	40	u	0.063743
			215425_at	0	3.788	0.67	7	4.097	1.04	40	u	0.460818
APP	6	amyloid beta (A4) precursor protein (peptidase nexin-II, Alzheimer disease)	200602_at	6	10.48	0.42	7	9.964	0.78	40	d	0.098604
			214953_s_at	6	11.58	0.46	7	10.7	1.02	40	d	0.033125
			211277_x_at	0	7.813	0.45	7	7.652	0.6	40	d	0.514469
ORC5L	6	origin recognition complex, subunit 5-like (yeast)	204957_at	6	7.357	0.49	7	7.717	0.57	40	u	0.130298
			211212_s_at	0	5.218	0.38	7	5.933	0.68	40	u	0.011419
			211213_at	0	3.21	0.18	7	3.272	0.32	40	u	0.622471
CENTG3	6	centaurin, gamma 3	239026_x_at	6	6.41	0.14	7	6.306	0.56	40	d	0.333213
			231299_at	0	2.614	0.28	7	2.996	0.7	40	u	0.169055
			225789_at	0	8.841	0.34	7	8.295	0.92	40	d	0.136756
ATRN	6	attractin	212517_at	6	7.942	0.39	7	7.371	0.62	40	d	0.026372
			211852_s_at	0	6.861	0.41	7	5.882	0.85	40	d	0.005581
SGPP1	6	sphingosine-1-phosphate phosphatase 1	223391_at	6	8.075	0.17	7	7.527	1.13	40	d	0.006876
			221268_s_at	5	6.247	0.51	7	5.739	1.49	40	d	0.121677
CNBP	6	CCHC-type zinc finger, nucleic acid binding protein	206158_s_at	6	12.16	0.3	7	11.63	0.5	40	d	0.011004
PRKRA	6	protein kinase, interferon-inducible double stranded RNA dependent activator	237107_at	0	4.821	0.46	7	5.5	0.96	40	u	0.078102
			209139_s_at	0	8.874	0.22	7	8.944	0.54	40	u	0.742123
TLN1	6	talin 1	203254_s_at	6	8.174	0.31	7	6.977	0.97	40	d	0.000002
			232763_at	0	2.442	0.18	7	2.671	0.34	40	u	0.094747

USP47	6	ubiquitin specific peptidase 47	223117_s_at	6	8.636	0.33	7	8.119	0.5	40	d	0.01273
			223118_s_at	6	7.379	0.2	7	6.658	0.74	40	d	0.000014
			221518_s_at	6	9.472	0.24	7	9.16	0.49	40	d	0.111692
			223119_s_at	6	7.303	0.32	7	5.399	0.92	40	d	0
			223701_s_at	0	7.832	0.29	7	8.424	0.52	40	u	0.006559
BZRAP1	6	benzodiazapine receptor (peripheral) associated protein 1	205839_s_at	0	6.751	0.47	7	5.985	0.93	40	d	0.043789
B4GALNT1	6	beta-1,4-N-acetyl-galactosaminyl transferase 1	206435_at	6	2.868	0.29	7	3.151	0.63	40	u	0.263531
			1555385_at	0	2.559	0.27	7	2.799	0.58	40	u	0.293358
ARHGAP21	6	Rho GTPase activating protein 21	224764_at	6	9.488	0.35	7	9.019	1.13	40	d	0.050423
			241701_at	0	5.225	0.64	7	5.815	1	40	u	0.147936
HOOK3	6	hook homolog 3 (Drosophila)	235114_x_at	6	2.735	0.13	7	3.64	0.9	40	u	0.000001
			224359_s_at	4	4.982	0.43	7	5.126	0.83	40	u	0.661553
			1558315_s_at	0	4.177	0.5	7	4.402	0.77	40	u	0.470315
PARP16	6	poly (ADP-ribose) polymerase family, member 16	219034_at	6	7.268	0.49	7	6.93	0.49	40	d	0.109198
DLX2	6	distal-less homeobox 2	207147_at	6	2.81	0.57	7	3.368	1.55	40	u	0.359743
			215685_s_at	0	5.328	0.16	7	5.383	0.35	40	u	0.689863
C10orf38	6	chromosome 10 open reading frame 38	212771_at	6	7.999	0.45	7	7.675	1.8	40	d	0.350589
MBD5	6	methyl-CpG binding domain protein 5	227839_at	6	3.769	0.28	7	3.925	0.58	40	u	0.501856
			220195_at	0	3.716	0.43	7	4.596	0.77	40	u	0.006314
CPEB1	6	cytoplasmic polyadenylation element binding protein 1	222903_s_at	6	2.119	0.09	7	2.254	0.32	40	u	0.042386
			219578_s_at	1	5.03	0.72	7	4.054	1.22	40	d	0.050278
RASSF5	6	Ras association (RalGDS/AF-6) domain family 5	223322_at	6	7.515	0.32	7	7.276	1.05	40	d	0.270445
			1554834_a_at	0	4.536	0.78	7	4.862	1.32	40	u	0.538862
PALM2	6	paralemmin 2	1554640_at	6	4.235	0.83	7	4.285	1.05	40	u	0.907565
			1553502_a_at	0	2.987	0.1	7	3.205	0.53	40	u	0.027006
PICALM	6	phosphatidylinositol binding clathrin assembly protein	212506_at	6	10.31	0.25	7	10.07	0.45	40	d	0.188141
			212511_at	6	7.326	0.53	7	6.801	0.64	40	d	0.051592
			215236_s_at	6	8.027	0.62	7	7.899	1.7	40	d	0.732877
			215832_x_at	6	7.416	0.47	7	7.158	1.25	40	d	0.600907
			203134_at	0	7.063	0.34	7	7.014	0.55	40	d	0.823527
PJA2	6	praja 2, RING-H2 motif containing	201133_s_at	6	10.41	0.27	7	9.6	0.78	40	d	0.000047
KIAA0494	6	KIAA0494	201776_s_at	6	8.037	0.23	7	7.633	0.6	40	d	0.091353
			201775_s_at	6	8.122	0.3	7	7.72	0.65	40	d	0.120229
			201778_s_at	6	10.74	0.21	7	9.58	0.51	40	d	0.000001
			1559022_at	0	3.968	0.45	7	3.626	0.58	40	d	0.153415
			201777_s_at	0	5.906	0.44	7	6.435	0.68	40	u	0.059028
NR2C2	6	nuclear receptor subfamily 2, group C, member 2	225477_s_at	1	8.357	0.37	7	8.032	0.49	40	d	0.112212
			206038_s_at	0	3.807	0.27	7	4.345	0.69	40	u	0.051385
SEPT3	6	septin 3	223362_s_at	6	5.095	0.28	7	6.379	1.63	40	u	0.000051
ASXL2	6	additional sex combs like 2 (Drosophila)	218659_at	6	8.919	0.26	7	8.874	0.44	40	d	0.795594
			237095_at	0	4.235	0.59	7	4.515	0.48	40	u	0.183219
			240072_at	0	4.995	0.23	7	5.175	0.64	40	u	0.204775
			1555266_a_at	0	2.641	0.14	7	3.471	1.18	40	u	0.000133
NCOA3	6	nuclear receptor coactivator 3	209060_x_at	6	7.051	0.27	7	7.826	0.64	40	u	0.003373
			209061_at	6	8.386	0.2	7	8.272	0.66	40	d	0.401601
			207700_s_at	5	7.936	0.22	7	8.464	0.86	40	u	0.002579
			211352_s_at	4	6.911	0.31	7	7.532	0.96	40	u	0.004119
			1562439_at	0	2.38	0.04	7	2.456	0.1	40	u	0.065686
			209062_x_at	0	5.322	0.42	7	6.149	1.16	40	u	0.003347
PHF21B	6	PHD finger protein 21B	1560091_a_at	6	2.385	0.08	7	2.529	0.43	40	u	0.06541
			1562309_s_at	6	2.972	0.3	7	3.236	1.26	40	u	0.268433
ZFP36	6	zinc finger protein 36, C3H type, homolog (mouse)	201531_at	6	11.94	0.75	7	9.708	1	40	d	0.000002
SOLH	6	small optic lobes homolog (Drosophila)	204275_at	6	5.974	0.53	7	6.074	1.09	40	u	0.817192
			230295_at	0	3.29	0.35	7	3.319	0.57	40	u	0.900311
MAP7	6	microtubule-associated protein 7	202889_x_at	6	6.001	0.35	7	6.646	1.39	40	u	0.01952
			202890_at	6	8.374	0.29	7	8.974	0.96	40	u	0.004622
			215471_s_at	1	6.061	0.57	7	6.897	1.31	40	u	0.111946
WIPI2	6	WD repeat domain, phosphoinositide interacting 2	214699_x_at	6	5.167	0.58	7	5.367	0.72	40	u	0.500391
			226986_at	6	6.449	0.36	7	6.052	0.63	40	d	0.121608
			202031_s_at	5	8.947	0.16	7	8.847	0.56	40	d	0.373897
			204710_s_at	2	9.359	0.26	7	9.53	0.49	40	u	0.380625

			1570033_at	0	2.734	0.13	7	2.819	0.26	40	u 0.411007
CBX1	6	chromobox homolog 1 (HP1 beta homolog Drosophila)	201518_at	6	9.557	0.27	7	9.878	0.65	40	u 0.212844
SPATA2	6	spermatogenesis associated 2	204434_at	6	5.821	0.34	7	5.196	0.91	40	d 0.085968
			204433_s_at	1	4.919	0.5	7	4.919	0.94	40	d 0.998131
FAM91A1	6	family with sequence similarity 91, member A1	226294_x_at	6	8.515	0.27	7	9.282	0.61	40	u 0.002383
			237045_at	0	4.396	0.25	7	4.478	0.26	40	u 0.460506
TSPAN14	6	tetraspanin 14	221002_s_at	6	8.78	0.31	7	8.019	0.7	40	d 0.00796
			223314_at	3	7.731	0.49	7	7.093	0.61	40	d 0.014093
PTGER4	6	prostaglandin E receptor 4 (subtype EP4)	204896_s_at	6	2.728	0.47	7	2.293	0.36	40	d 0.009024
			204897_at	6	8.831	0.46	7	6.909	0.97	40	d 0.000008
SMAP1	6	stromal membrane-associated protein 1	218137_s_at	6	7.87	0.35	7	8.722	0.58	40	u 0.000648
HMGCR	6	3-hydroxy-3-methylglutaryl-Coenzyme A reductase	202540_s_at	6	7.834	0.55	7	7.671	0.77	40	d 0.600981
			202539_s_at	6	8.091	0.77	7	7.609	0.99	40	d 0.238962
FLT1	6	fms-related tyrosine kinase 1 (vascular endothelial growth factor/vascular permeability factor receptor)	204406_at	6	2.394	0.14	7	2.439	0.28	40	u 0.692376
			232809_s_at	0	6.62	0.26	7	5.907	0.64	40	d 0.006572
			210287_s_at	0	4.363	0.72	7	4.183	0.41	40	d 0.574285
			222033_s_at	0	7.03	0.67	7	6.438	0.5	40	d 0.010539
FOXJ3	6	forkhead box J3	206015_s_at	6	9.539	0.24	7	9.011	0.45	40	d 0.004631
			217310_s_at	1	2.839	0.35	7	3.941	1.21	40	u 0.000069
GATA3	6	GATA binding protein 3	209602_s_at	6	8.292	0.92	7	7.221	2.52	40	d 0.064663
			209603_at	6	8.311	1.02	7	7.006	2.42	40	d 0.175326
			209604_s_at	3	12.06	0.71	7	10.65	2.45	40	d 0.007163
BIRC4	6	baculoviral IAP repeat-containing 4	228363_at	6	8.416	0.3	7	8.722	0.69	40	u 0.265458
			225858_s_at	5	7.84	0.23	7	8.301	0.65	40	u 0.003249
			235222_x_at	5	8.271	0.39	7	8.476	0.62	40	u 0.414428
			225859_at	5	7.241	0.29	7	7.733	0.87	40	u 0.011712
			243026_x_at	5	7.637	0.34	7	7.89	0.65	40	u 0.330364
			206536_s_at	0	3.592	0.26	7	4.727	0.84	40	u 0
PLCB4	6	phospholipase C, beta 4	203895_at	6	6.175	0.45	7	6.713	1.86	40	u 0.130998
			240728_at	0	3.286	0.44	7	4.384	1.31	40	u 0.000555
			203896_s_at	0	5.482	0.64	7	7.006	1.9	40	u 0.000788
HOXB4	6	homeobox B4	231767_at	6	6.795	0.6	7	6.673	0.64	40	d 0.648619
MYLK	6	myosin, light chain kinase	224823_at	6	11.88	0.25	7	8.978	1.53	40	d 0
			202555_s_at	1	12.23	0.22	7	8.885	1.64	40	d 0
			1563466_at	0	5.253	0.62	7	3.113	0.78	40	d 0
			1568770_at	0	2.119	0.1	7	2.235	0.22	40	u 0.184152
HOXD10	6	homeobox D10	229400_at	6	5.615	0.65	7	4.956	1.11	40	d 0.142848
			207373_at	0	2.212	0.09	7	2.283	0.49	40	u 0.419158
SH3BP5	6	SH3-domain binding protein 5 (BTK-associated)	201810_s_at	6	9.065	0.37	7	8.54	0.88	40	d 0.133083
			201811_x_at	6	10.03	0.44	7	9.4	0.86	40	d 0.07292
			227591_at	6	7.772	0.35	7	6.511	0.76	40	d 0.000118
AKAP6	6	A kinase (PRKA) anchor protein 6	205359_at	6	2.048	0.19	7	2.093	0.3	40	u 0.706987
			217669_s_at	0	2.381	0.08	7	2.465	0.12	40	u 0.087744
NECAP1	6	NECAP endocytosis associated 1	209300_s_at	6	8.054	0.23	7	8.006	0.77	40	d 0.759729
CSMD2	6	CUB and Sushi multiple domains 2	1570169_at	0	2.952	0.37	7	3.005	0.54	40	u 0.808426
			233145_at	0	4.482	0.24	7	4.745	0.36	40	u 0.077182
			1557143_at	0	2.769	0.35	7	3.261	0.59	40	u 0.042509
KIAA1370	6	KIAA1370	225327_at	6	10.13	0.38	7	8.617	1.48	40	d 0.000005
SLC39A13	6	solute carrier family 39 (zinc transporter), member 13	225277_at	6	8.308	0.11	7	7.8	0.58	40	d 0.000012
			1552295_a_at	6	7.117	0.29	7	7.034	0.84	40	d 0.647268
WBS2	6	Williams-Beuren syndrome type 2	no probeset available								
SMPD3	6	sphingomyelin phosphodiesterase 3, neutral membrane (neutral sphingomyelinase II)	219695_at	6	3.011	0.64	7	3.24	1.09	40	u 0.599017
			231732_at	1	4.88	0.21	7	4.965	0.87	40	u 0.603882
LYCAT	6	lysocardiolipin acyltransferase	226996_at	6	8.464	0.3	7	9.149	0.6	40	u 0.006255
COL4A1	6	collagen, type IV, alpha 1	211980_at	6	9.605	0.44	7	9.798	1	40	u 0.624171
			211981_at	6	8.092	0.36	7	8.249	1.41	40	u 0.562816
SLC7A5P1	6	solute carrier family 7 (cationic amino acid transporter, y+ system), member 5 pseudogene 1	208118_x_at	3	5.774	0.48	7	6.338	0.57	40	u 0.019968
SEMA4F	6	sema domain, immunoglobulin domain (Ig), transmembrane domain (TM) and short cytoplasmic domain, (semaphorin) 4F	228660_x_at	6	6.165	0.43	7	6.209	0.68	40	u 0.871388
			210124_x_at	6	6.272	0.39	7	6.23	0.73	40	d 0.883905
			208124_s_at	0	2.593	0.16	7	2.802	0.36	40	u 0.145042
			201500_s_at	6	9.74	0.29	7	9.614	0.56	40	d 0.570747

PPP1R11	6	protein phosphatase 1, regulatory (inhibitor) subunit 11	1566302_at	6	3.46	0.44	7	3.817	0.49	40	u	0.083226
			1566301_at	6	2.855	0.09	7	2.924	0.18	40	u	0.338409
			1566303_s_at	6	8.719	0.34	7	8.453	1.16	40	d	0.261196
CHED1	6	ıeal endothelial dystrophy 1 (autosomal domin	no probeset available									
RAB1A	6	RAB1A, member RAS oncogene family	213440_at	6	8.298	0.14	7	8.301	0.46	40	u	0.978853
			208724_s_at	5	12.19	0.18	7	12.43	0.5	40	u	0.036592
			207791_s_at	0	9.733	0.35	7	10.08	1.17	40	u	0.147067
			239428_at	0	3.39	0.27	7	3.376	0.33	40	d	0.914729
RGS17	6	regulator of G-protein signaling 17	220334_at	4	2.549	0.18	7	2.552	0.25	40	u	0.977025
NUDT4	6	nudix (nucleoside diphosphate linked moiety X)-type motif 4	212183_at	6	7.016	0.46	7	6.58	1.19	40	d	0.35216
			212182_at	6	2.53	0.08	7	2.765	0.49	40	u	0.007605
			212181_s_at	6	9.686	0.26	7	9.319	0.98	40	d	0.060185
			206302_s_at	1	7.664	0.52	7	8.186	1.2	40	u	0.274607
			206303_s_at	1	5.698	0.58	7	6.141	1.11	40	u	0.316805
KCND2	6	potassium voltage-gated channel, Shal-related subfamily, member 2	207103_at	6	2.803	0.16	7	3.12	0.73	40	u	0.023587
GALNT1	6	UDP-N-acetyl-alpha-D-galactosamine:polypeptide N-acetylgalactosaminyltransferase 1 (GalNAc-T1)	201724_s_at	6	8.7	0.47	7	9.017	0.71	40	u	0.269106
			201722_s_at	6	9.59	0.35	7	9.856	0.64	40	u	0.298832
			201723_s_at	3	9.181	0.42	7	9.86	0.64	40	u	0.011375
			1568618_a_at	0	9.656	0.33	7	10.26	0.71	40	u	0.034835
			236746_at	0	2.472	0.12	7	2.582	0.29	40	u	0.333598
ARCN1	6	archain 1	201176_s_at	5	10.76	0.26	7	10.52	0.59	40	d	0.316769
LIMK1	6	LIM domain kinase 1	204357_s_at	6	4.027	0.33	7	4.382	0.55	40	u	0.11254
			204356_at	6	1.997	0.06	7	2.03	0.05	40	u	0.106736
			208372_s_at	0	4.24	0.15	7	4.375	0.51	40	u	0.200217
PBX2	6	pre-B-cell leukemia homeobox 2	202876_s_at	0	7.885	0.22	7	7.702	0.81	40	d	0.252512
			202875_s_at	0	6.675	0.11	7	6.43	0.92	40	d	0.117522
			211096_at	0	5.701	0.25	7	5.722	0.41	40	u	0.899565
			211097_s_at	0	5.431	0.27	7	5.698	0.94	40	u	0.163826
SOX2	6	SRY (sex determining region Y)-box 2	228038_at	6	2.585	0.63	7	3.095	1.81	40	u	0.201898
			214178_s_at	0	2.058	0.38	7	2.105	0.4	40	u	0.777381
			213721_at	0	2.647	0.15	7	3.082	1.09	40	u	0.02271
			213722_at	0	2.915	0.26	7	3.047	0.63	40	u	0.592391
SPHK2	6	sphingosine kinase 2	40273_at	6	5.556	0.53	7	5.535	0.5	40	d	0.919462
			209857_s_at	2	3.981	0.25	7	3.937	0.3	40	d	0.723583
LASS2	6	LAG1 homolog, ceramide synthase 2	222212_s_at	6	10.2	0.4	7	10.76	0.83	40	u	0.092448
DHCR24	6	24-dehydrocholesterol reductase	200862_at	6	11.19	0.88	7	9.82	1.4	40	d	0.01794
UBE2E3	6	ubiquitin-conjugating enzyme E2E 3 (UBC4/5 homolog, yeast)	210024_s_at	6	10.19	0.25	7	9.563	1.15	40	d	0.005174
SLC23A2	6	solute carrier family 23 (nucleobase transporters), member 2	209236_at	6	7.918	0.35	7	6.93	0.79	40	d	0.002724
			209237_s_at	3	3.018	0.46	7	3.227	0.8	40	u	0.511981
			211572_s_at	1	2.411	0.27	7	2.793	0.8	40	u	0.032143
			1554692_at	0	2.492	0.05	7	2.585	0.16	40	u	0.007382
GRK6	6	G protein-coupled receptor kinase 6	202849_x_at	6	6.996	0.54	7	7.151	0.57	40	u	0.520112
			210981_s_at	6	7.22	0.41	7	7.703	0.54	40	u	0.03346
			202848_s_at	6	5.949	0.36	7	6.154	0.67	40	u	0.442828
			211543_s_at	0	6.778	0.32	7	6.559	0.74	40	d	0.453398
VPS26A	6	vacuolar protein sorting 26 homolog A (S. pombe)	201807_at	6	10.6	0.15	7	10.46	0.48	40	d	0.181341
			243316_x_at	0	3.002	0.17	7	3.239	0.29	40	u	0.047765
ATP8B2	6	ATPase, Class I, type 8B, member 2	226771_at	6	7.871	0.19	7	6.998	1.18	40	d	0.0001
			216873_s_at	2	6.519	0.2	7	6.426	0.54	40	d	0.65725
DPP4	6	dipeptidyl-peptidase 4 (CD26, adenosine deaminase complexing protein 2)	203717_at	6	4.698	0.57	7	3.946	0.91	40	d	0.0446
			211478_s_at	0	4.325	0.8	7	3.751	0.86	40	d	0.115887
			203716_s_at	0	5.142	0.67	7	4.253	1.06	40	d	0.041368
HDAC5	6	histone deacetylase 5	229408_at	6	5.816	0.26	7	5.702	0.51	40	d	0.576206
			202455_at	2	6.536	0.49	7	5.82	0.9	40	d	0.051073
MARK1	6	MAP/microtubule affinity-regulating kinase 1	226653_at	6	3.545	0.85	7	3.716	1.07	40	u	0.697544
			231613_at	3	2.691	0.05	7	2.95	0.34	40	u	0.000072
			221047_s_at	1	5.686	0.43	7	5.859	1.18	40	u	0.508309
			1560407_at	0	3.713	0.2	7	4.091	0.61	40	u	0.006396
TRIM3	6	tripartite motif-containing 3	213885_at	6	6.705	0.38	7	6.454	1.3	40	d	0.339466
			213884_s_at	6	4.682	0.38	7	4.669	1.31	40	d	0.959193
			204910_s_at	1	2.725	0.31	7	2.827	0.6	40	u	0.670618
			204911_s_at	0	4.814	0.36	7	5.027	1.24	40	u	0.39407

TBC1D22B	6	TBC1 domain family, member 22B	223375_at	6	6.406	0.2	7	6.657	0.61	40	u	0.060559
			233430_at	0	4.991	0.14	7	5.14	0.49	40	u	0.133454
			216549_s_at	0	2.804	0.46	7	3.226	1.01	40	u	0.294082
SIPA1L2	6	signal-induced proliferation-associated 1 like 2	225056_at	6	7.42	0.47	7	7.773	1.03	40	u	0.389224
			233587_s_at	3	6.107	0.6	7	6.616	1.1	40	u	0.250871
			1559469_s_at	0	2.704	0.26	7	3.164	0.48	40	u	0.019022
ITSN2	6	intersectin 2	209907_s_at	6	7.233	0.33	7	7.041	0.69	40	d	0.487479
			209898_x_at	0	8.813	0.34	7	8.609	0.67	40	d	0.444595
			240941_at	0	4.429	0.19	7	4.763	0.41	40	u	0.042505
			210545_at	0	1.972	0.09	7	2.012	0.11	40	u	0.361342
CDX2	6	caudal type homeobox 2	206387_at	4	2.955	0.27	7	3.007	0.47	40	u	0.782291
HOXA3	6	homeobox A3	235521_at	5	7.019	0.64	7	4.238	1.32	40	d	0.000003
			208604_s_at	0	4.275	0.72	7	3.457	0.62	40	d	0.003438
PPP1CC	6	protein phosphatase 1, catalytic subunit, gamma isoform	200726_at	6	11.16	0.14	7	11.03	0.47	40	d	0.190456
CDC37L1	6	cell division cycle 37 homolog (S. cerevisiae)-like 1	219343_at	5	7.565	0.41	7	7.05	0.98	40	d	0.187302
			228561_at	0	5.609	0.32	7	4.838	1.19	40	d	0.002041
ZIC1	6	Zic family member 1 (odd-paired homolog, Drosophila)	206373_at	6	3.131	1.23	7	5.203	2.59	40	u	0.047779
			236896_at	0	2.566	0.21	7	2.641	0.22	40	u	0.404418
			1557154_at	0	3.5	0.28	7	4.557	1.08	40	u	0.000013
			234716_at	0	2.618	0.22	7	2.596	0.16	40	d	0.748372
			1557155_a_at	0	3.35	0.77	7	4.773	1.92	40	u	0.065188
LYST	6	lysosomal trafficking regulator	203518_at	6	7.576	0.36	7	7.064	0.92	40	d	0.160825
			1562511_at	0	3.004	0.46	7	3.394	0.47	40	u	0.054732
			215415_s_at	0	2.542	0.18	7	2.997	1.07	40	u	0.018549
			210943_s_at	0	6.845	0.58	7	7.431	0.81	40	u	0.079774
JAKMIP1	6	janus kinase and microtubule interacting protein 1	238600_at	6	2.736	0.22	7	2.902	0.4	40	u	0.293315
COPS2	6	COP9 constitutive photomorphogenic homolog subunit 2 (Arabidopsis)	209838_at	6	6.447	0.56	7	6.424	0.71	40	d	0.936805
			202467_s_at	6	10.89	0.21	7	10.39	0.51	40	d	0.015082
PRKAR1A	6	protein kinase, cAMP-dependent, regulatory, type I, alpha (tissue specific extinguisher 1)	200603_at	6	11.26	0.14	7	11.02	0.84	40	d	0.106908
			200605_s_at	4	11.46	0.17	7	10.88	1.26	40	d	0.009477
			242482_at	0	5.753	0.64	7	5.715	0.84	40	d	0.913318
			200604_s_at	0	9.649	0.57	7	9.126	1.47	40	d	0.369295
DOLPP1	6	dolichyl pyrophosphate phosphatase 1	221817_at	6	7.279	0.33	7	7.43	0.54	40	u	0.486075
B4GALT2	6	UDP-Gal:betaGlcNAc beta 1,4- galactosyltransferase, polypeptide 2	209413_at	6	7.02	0.23	7	7.335	0.79	40	u	0.055843
TMEM9B	6	TMEM9 domain family, member B	218065_s_at	6	9.641	0.33	7	9.043	0.53	40	d	0.007084
			222507_s_at	6	8.595	0.56	7	7.837	1.4	40	d	0.173608
FAM53C	6	family with sequence similarity 53, member C	218023_s_at	6	8.055	0.3	7	7.662	0.53	40	d	0.065375
PKNOX1	6	PBX/knotted 1 homeobox 1	54051_at	6	4.538	0.11	7	4.632	0.28	40	u	0.400004
			221883_at	6	6.205	0.33	7	6.17	0.61	40	d	0.886489
			216004_s_at	4	3.526	0.28	7	3.846	0.49	40	u	0.106589
			204195_s_at	4	5.499	0.48	7	5.414	0.43	40	d	0.649302
			204196_x_at	4	2.797	0.09	7	3.16	0.3	40	u	0.000001
RND3	6	Rho family GTPase 3	212724_at	6	11.14	0.28	7	9.34	1.04	40	d	0
RRBP1	6	ribosome binding protein 1 homolog 180kDa (dog)	201203_s_at	6	7.458	0.4	7	7.185	0.94	40	d	0.460644
			201204_s_at	6	9.499	0.45	7	8.915	0.9	40	d	0.108361
			213495_s_at	6	2.209	0.18	7	2.4	0.4	40	u	0.236014
			201206_s_at	3	8.299	0.42	7	7.853	1.08	40	d	0.296262
			227888_at	0	2.949	0.28	7	2.971	0.35	40	u	0.877551
			213973_at	0	4.415	0.59	7	4.536	0.73	40	u	0.684738
VAV2	6	vav 2 guanine nucleotide exchange factor	226063_at	6	7.736	0.28	7	8.185	0.55	40	u	0.045556
			205536_at	2	5.892	0.27	7	6.501	0.63	40	u	0.018252
			205537_s_at	0	4.946	0.69	7	5.198	0.67	40	u	0.372911
SLC17A7	6	solute carrier family 17 (sodium-dependent inorganic phosphate cotransporter), member	204229_at	6	2.192	0.13	7	2.194	0.1	40	u	0.974912
			204230_s_at	2	4.604	0.48	7	4.639	0.65	40	u	0.894729
TMEM20	6	transmembrane protein 20	231012_at	0	3.116	0.22	7	3.356	0.49	40	u	0.221573
			239265_at	0	2.523	0.19	7	2.767	0.64	40	u	0.069744
PRPF38B	6	PRP38 pre-mRNA processing factor 38 (yeast) domain containing B	218040_at	6	7.208	0.08	7	7.135	0.45	40	d	0.357585
			230270_at	0	8.218	0.61	7	7.974	0.83	40	d	0.469972
GRIA3	6	glutamate receptor, ionotrophic, AMPA 3	206730_at	6	2.395	0.02	7	2.455	0.06	40	u	0.000106
			1569290_s_at	0	2.332	0.01	7	2.467	0.4	40	u	0.040856

GRIA3	6	glutamate receptor, ionotrophic, AMPA 3	217565_at	0	2.769	0.11	7	2.85	0.23	40	u	0.379484
			208032_s_at	0	3.644	0.4	7	3.224	0.37	40	d	0.010773
GOPC	6	golgi associated PDZ and coiled-coil motif containing	225022_at	6	8.389	0.23	7	8.152	0.77	40	d	0.135178
			225023_at	6	6.902	0.37	7	7.503	0.73	40	u	0.043803
			227215_at	2	7.39	0.25	7	7.476	0.87	40	u	0.622952
			227214_at	2	4.961	0.48	7	5.764	1.14	40	u	0.079736
			236862_at	0	3.767	0.61	7	4.412	1.37	40	u	0.237162
NTRK3	6	neurotrophic tyrosine kinase, receptor, type 3	228849_at	6	4.187	0.61	7	4.204	0.89	40	u	0.963521
			215025_at	2	2.42	0.12	7	2.834	1.09	40	u	0.027619
			215115_x_at	0	4.515	0.5	7	4.483	0.6	40	d	0.894894
			217033_x_at	0	4.265	0.32	7	4.235	0.54	40	d	0.890918
			217377_x_at	0	5.121	0.3	7	5.02	0.55	40	d	0.640997
			1557795_s_at	0	1.922	0.07	7	2.221	0.86	40	u	0.039383
			206462_s_at	0	2.71	0.36	7	2.615	0.66	40	d	0.718691
RAPGEF4	6	Rap guanine nucleotide exchange factor (GEF) 4	205651_x_at	6	4.362	0.4	7	4.398	0.71	40	u	0.900097
BMPER	6	BMP binding endothelial regulator	241986_at	6	2.724	0.31	7	2.651	0.31	40	d	0.574423
PDCD10	6	programmed cell death 10	210907_s_at	6	10.01	0.37	7	10.83	0.74	40	u	0.007521
KIAA0284	6	KIAA0284	213242_x_at	6	8.751	0.14	7	8.068	0.66	40	d	0.000001
C11orf30	6	chromosome 11 open reading frame 30	222806_s_at	6	7.221	0.33	7	7.426	1.04	40	u	0.344821
			222807_at	6	6.785	0.31	7	6.808	1.01	40	u	0.909538
			219012_s_at	6	3.922	0.4	7	4.191	0.74	40	u	0.366689
			1569349_at	0	2.947	0.13	7	3.671	1.15	40	u	0.000492
			234669_x_at	0	7.152	0.15	7	6.92	0.53	40	d	0.036774
			242847_at	0	4.965	0.47	7	5.419	0.78	40	u	0.149612
DYRK1B	6	dual-specificity tyrosine-(Y)-phosphorylation regulated kinase 1B	204954_s_at	6	4.97	0.42	7	4.615	0.84	40	d	0.28892
			217270_s_at	0	5.778	0.27	7	5.5	0.62	40	d	0.25866
SLC25A25	6	solute carrier family 25 (mitochondrial carrier; phosphate carrier), member 25	225212_at	6	8.224	0.7	7	7.682	0.63	40	d	0.04915
BHLHB5	6	basic helix-loop-helix domain containing, class B, 5	228636_at	6	5.795	0.73	7	5.253	0.89	40	d	0.143034
			244323_at	0	3.07	0.32	7	3.269	0.4	40	u	0.229923
SLC11A2	6	solute carrier family 11 (proton-coupled divalent metal ion transporters), member 2	203123_s_at	3	8.024	0.4	7	8.192	0.89	40	u	0.635392
			203124_s_at	3	8.861	0.28	7	8.821	0.78	40	d	0.818707
			203125_x_at	1	6.078	0.68	7	6.737	1.1	40	u	0.138734
			237106_at	0	4.72	0.37	7	5.054	0.56	40	u	0.147193
			210047_at	0	5.413	0.36	7	5.506	0.58	40	u	0.687125
EPB41	6	erythrocyte membrane protein band 4.1 (elliptocytosis 1, RH-linked)	225051_at	9	8.763	0.15	7	8.635	0.65	40	d	0.297624
			207793_s_at	0	2.871	0.22	7	3.079	0.41	40	u	0.208911
			1554481_a_at	0	2.459	0.18	7	2.906	0.82	40	u	0.004691
			214530_x_at	0	2.221	0.12	7	2.642	0.7	40	u	0.00125
SACS	6	spastic ataxia of Charlevoix-Saguenay (sacsin)	213262_at	6	6.341	0.46	7	5.497	1.01	40	d	0.038436
PLK2	6	polo-like kinase 2 (Drosophila)	201939_at	6	10.01	0.46	7	8.546	1.1	40	d	0.001468
SMURF1	6	SMAD specific E3 ubiquitin protein ligase 1	212666_at	6	6.295	0.28	7	6.023	0.74	40	d	0.354193
			212668_at	6	3.42	0.32	7	3.524	0.63	40	u	0.676755
			215458_s_at	0	4.897	0.51	7	5.12	1.09	40	u	0.605998
MLLT6	6	myeloid/lymphoid or mixed-lineage leukemia (trithorax homolog, Drosophila); translocated to, 6	225628_s_at	7	8.086	0.27	7	7.298	0.82	40	d	0.000081
			224784_at	0	10.2	0.3	7	9.357	1.17	40	d	0.000574
			1555155_at	0	5.27	0.22	7	5.368	0.41	40	u	0.552002
FGF7	6	fibroblast growth factor 7 (keratinocyte growth factor)	205782_at	6	4.876	1.11	7	3.51	0.84	40	d	0.000621
			1555103_s_at	0	2.595	0.06	7	2.887	0.38	40	u	0.000047
			1555102_at	0	3.107	0.08	7	3.274	0.25	40	u	0.00258
			1554741_s_at	0	7.986	0.4	7	6.996	0.79	40	d	0.002589
FNBP4	6	formin binding protein 4	212232_at	6	9.277	0.26	7	8.715	0.74	40	d	0.001687
			235101_at	0	3.347	0.14	7	4.084	1.08	40	u	0.000212
			242472_x_at	0	5.077	0.27	7	5.182	0.65	40	u	0.68546
			229272_at	0	6.646	0.44	7	6.973	0.97	40	u	0.394455
ANKRD13C	6	ankyrin repeat domain 13C	223418_x_at	6	6.294	0.38	7	6.665	0.64	40	u	0.153103
			227375_at	6	5.545	0.5	7	5.699	0.75	40	u	0.613152
			1554471_a_at	6	6.492	0.46	7	6.712	0.78	40	u	0.484758
			238597_at	0	3.903	0.68	7	3.843	0.64	40	d	0.823064
			1556361_s_at	0	2.495	0.04	7	2.601	0.11	40	u	0.000128
SYS1	6	SYS1 Golgi-localized integral membrane protein homolog (S. cerevisiae)	224668_at	6	6.459	0.2	7	5.986	0.81	40	d	0.003633
			224670_at	6	7.215	0.26	7	7.091	0.64	40	d	0.624444

		protein homolog (S. cerevisiae)	224669_at	6	7.604	0.25	7	7.202	0.63	40	d	0.110264
DLX3	6	distal-less homeobox 3	231778_at	4	3.019	0.2	7	3.482	1.11	40	u	0.022577
TMEM188	6	transmembrane protein 188	235812_at	6	8.105	0.27	7	7.965	0.53	40	d	0.508122
LHX6	6	LIM homeobox 6	219884_at	6	5.767	0.73	7	4.882	0.84	40	d	0.013508
			224556_s_at	0	2.202	0.2	7	2.253	0.39	40	u	0.741694
STK4	6	serine/threonine kinase 4	225364_at	6	8.432	0.22	7	8.712	0.57	40	u	0.217022
			236259_at	5	7.728	0.36	7	8.087	0.49	40	u	0.078371
			205411_at	3	2.353	0.03	7	2.645	0.44	40	u	0.000173
			1569791_at	0	2.903	0.15	7	3.059	0.28	40	u	0.174422
			243981_at	0	3.545	0.37	7	3.539	0.8	40	d	0.9837
			211085_s_at	0	4.045	0.29	7	5.259	0.81	40	u	0
			223746_at	0	4.163	0.54	7	4.574	0.93	40	u	0.271847
ZNF367	6	zinc finger protein 367	229551_x_at	0	4.499	0.68	7	6.95	0.96	40	u	0
SLC9A1	6	solute carrier family 9 (sodium/hydrogen exchanger), member 1 (antiporter, Na+/H+,	209453_at	6	7.769	0.71	7	6.64	1.07	40	d	0.011669
			1554728_at	0	3.445	0.03	7	3.486	0.12	40	u	0.076614
TTYH2	6	tweety homolog 2 (Drosophila)	223741_s_at	6	6.245	0.39	7	6.142	0.76	40	d	0.731697
			232042_at	0	3.205	0.38	7	3.127	0.4	40	d	0.644202
TRIM39	6	tripartite motif-containing 39	222732_at	6	7.182	0.21	7	7.043	0.39	40	d	0.377255
ZFYVE26	6	zinc finger, FYVE domain containing 26	213073_at	6	7.199	0.23	7	6.429	0.73	40	d	0.000017
			37943_at	6	6.395	0.6	7	6.243	0.49	40	d	0.478809
HOXA9	6	homeobox A9	209905_at	6	5.531	0.81	7	4.308	1.01	40	d	0.004752
			214651_s_at	3	7.147	0.91	7	4.218	1.53	40	d	0.000017
ZC3H11A	6	zinc finger CCCH-type containing 11A	205788_s_at	6	10.66	0.22	7	10.52	0.52	40	d	0.50913
			205787_x_at	6	5.846	0.51	7	7.794	0.89	40	u	0.000002
			1565867_a_at	0	2.934	0.29	7	5.294	1.13	40	u	0
VCL	6	vinculin	200930_s_at	6	2.913	0.2	7	2.991	0.29	40	u	0.514565
			200931_s_at	6	11.27	0.36	7	10.25	0.86	40	d	0.004013
FYTTD1	6	forty-two-three domain containing 1	224641_at	6	10.04	0.22	7	10.23	0.82	40	u	0.250409
			224642_at	6	8.078	0.5	7	8.677	1.19	40	u	0.204683
RAVER2	6	ribonucleoprotein, PTB-binding 2	231851_at	6	5.796	0.51	7	5.643	0.96	40	d	0.687221
			220680_at	0	2.183	0.03	7	2.235	0.05	40	u	0.007243
MAP1B	6	microtubule-associated protein 1B	226084_at	6	8.403	0.44	7	7.433	1.29	40	d	0.0016
			214577_at	0	5.972	0.16	7	6.083	0.6	40	u	0.349267
BACH1	6	BTB and CNC homology 1, basic leucine zipper transcription factor 1	204194_at	6	7.087	0.3	7	7.201	0.78	40	u	0.710131
			210818_s_at	0	2.694	0.14	7	2.973	0.67	40	u	0.026992
			234315_at	0	2.931	0.1	7	3.153	0.35	40	u	0.003094
			234938_at	0	1.917	0.02	7	1.956	0.05	40	u	0.001009
DNAJB12	6	DnaJ (Hsp40) homolog, subfamily B, member 12	202865_at	6	6.276	0.28	7	6.4	0.46	40	u	0.497789
			202866_at	6	7.686	0.32	7	7.496	0.54	40	d	0.38217
			202867_s_at	3	7.543	0.28	7	6.962	0.78	40	d	0.061622
			214338_at	3	6.276	0.32	7	6.295	0.4	40	u	0.910022
ETV5	6	ets variant gene 5 (ets-related molecule)	203348_s_at	6	7.26	0.68	7	6.596	1.03	40	d	0.114768
			203349_s_at	6	8.325	0.6	7	6.238	1.28	40	d	0.00015
			230102_at	6	4.764	0.94	7	4.206	1.22	40	d	0.266509
			216375_s_at	1	4.859	0.64	7	4.008	1.07	40	d	0.051389
CXCL5	6	chemokine (C-X-C motif) ligand 5	214974_x_at	6	2.704	0.49	7	3.673	1.93	40	u	0.012637
			215101_s_at	0	2.497	0.15	7	3.173	1.46	40	u	0.007954
			207852_at	0	2.174	0.08	7	2.256	0.13	40	u	0.113326
PTPN3	6	protein tyrosine phosphatase, non-receptor type 3	203997_at	6	8.009	0.5	7	7.872	0.76	40	d	0.655037
			227944_at	0	5.892	0.47	7	5.234	0.75	40	d	0.033394
UBP1	6	upstream binding protein 1 (LBP-1a)	218082_s_at	6	9.213	0.38	7	8.913	0.64	40	d	0.244758
C16orf5	6	chromosome 16 open reading frame 5	218183_at	6	5.58	0.45	7	4.303	0.59	40	d	0.000003
			223960_s_at	6	7.044	0.39	7	6.028	0.91	40	d	0.006836
MYST2	6	MYST histone acetyltransferase 2	200049_at	6	7.61	0.31	7	7.656	0.79	40	u	0.882459
ATP2A2	6	ATPase, Ca++ transporting, cardiac muscle, slow twitch 2	209186_at	6	10.08	0.33	7	10.63	0.75	40	u	0.066624
			239996_x_at	0	7.564	0.26	7	7.537	0.4	40	d	0.869044
			212362_at	0	4.562	0.65	7	4.799	0.92	40	u	0.526806
			212361_s_at	0	9.166	0.12	7	7.751	1	40	d	0
FAM81A	6	family with sequence similarity 81, member A	227756_at	3	2.205	0.08	7	2.408	0.58	40	u	0.044187
			241481_at	0	2.323	0.13	7	2.446	0.22	40	u	0.160857
			241503_at	0	2.271	0.04	7	2.46	0.74	40	u	0.120279
RNF6	6	ring finger protein (C3H2C3 type) 6	203403_s_at	6	8.655	0.3	7	8.864	0.8	40	u	0.507216
			210931_at	0	2.947	0.34	7	2.971	0.31	40	u	0.857778
			210932_s_at	0	3.62	0.3	7	4.039	0.53	40	u	0.052082

SLC10A3	6	solute carrier family 10 (sodium/bile acid cotransporter family), member 3	204928_s_at	6	7.032	0.49	7	6.848	0.78	40	d	0.557093
TP63	6	tumor protein p63	209863_s_at	6	8.838	0.99	7	4.124	1.55	40	d	0
			211194_s_at	2	5.281	0.53	7	3.109	1.16	40	d	0.000019
			1555581_a_at	0	2.314	0.08	7	2.387	0.2	40	u	0.358789
			211195_s_at	0	2.425	0.46	7	2.205	1.01	40	d	0.582485
			211834_s_at	0	3.048	0.39	7	2.682	0.9	40	d	0.304116
			207382_at	0	3.739	0.78	7	2.759	0.83	40	d	0.006697
			211193_at	0	2.393	0.22	7	2.216	0.49	40	d	0.366431
C11orf9	6	chromosome 11 open reading frame 9	204073_s_at	6	2.69	0.24	7	2.801	0.55	40	u	0.610835
			217462_at	0	5.093	0.36	7	4.713	0.54	40	d	0.088496
			217463_s_at	0	2.426	0.03	7	2.483	0.06	40	u	0.018152
ST18	6	suppression of tumorigenicity 18 (breast carcinoma) (zinc finger protein)	206135_at	6	2.389	0.03	7	2.543	0.36	40	u	0.01177
			1570307_s_at	0	2.872	0.08	7	2.941	0.11	40	u	0.113683
			1557583_at	0	2.689	0.06	7	2.806	0.17	40	u	0.0037
			1570306_at	0	2.815	0.37	7	2.742	0.3	40	d	0.578529
FARP1	5	FERM, RhoGEF (ARHGEF) and pleckstrin domain protein 1 (chondrocyte-derived)	201910_at	5	8.471	0.38	7	8.035	0.77	40	d	0.158718
			227996_at	0	6.377	0.92	7	6.542	1.37	40	u	0.766255
			1562425_at	0	1.961	0.15	7	1.986	0.21	40	u	0.769025
			201911_s_at	0	9.376	0.4	7	9.111	1.01	40	d	0.50647
			239246_at	0	8.416	0.49	7	8.349	0.92	40	d	0.855688
			1562426_a_at	0	2.104	0.06	7	2.184	0.18	40	u	0.044558
ITGA5	5	integrin, alpha 5 (fibronectin receptor, alpha polypeptide)	201389_at	5	7.502	0.21	7	7.306	0.69	40	d	0.166767
CXCR4	5	chemokine (C-X-C motif) receptor 4	217028_at	5	8.742	0.4	7	10.3	1.13	40	u	0.000001
			209201_x_at	0	7.647	0.29	7	8.932	1.5	40	u	0.000018
			211919_s_at	0	7.638	0.24	7	8.96	1.65	40	u	0.000027
RTN1	5	reticulon 1	203485_at	5	6.697	0.66	7	5.599	1.79	40	d	0.123971
			210222_s_at	0	7.083	0.8	7	6.011	1.63	40	d	0.102485
KLHL14	5	kelch-like 14 (Drosophila)	228377_at	5	2.331	0.28	7	2.443	0.75	40	u	0.701443
			1554941_at	0	3.356	0.26	7	3.537	0.29	40	u	0.131377
			1554942_a_at	0	2.341	0.02	7	2.37	0.05	40	u	0.14126
LRRN1	5	leucine rich repeat neuronal 1	226884_at	2	6.439	0.49	7	5.148	1.68	40	d	0.000576
NFIA	5	nuclear factor I/A	224970_at	5	10.18	0.19	7	7.921	1.86	40	d	0
			224975_at	5	9.444	0.27	7	7.971	1.66	40	d	0.000007
			224976_at	5	8.651	0.23	7	6.427	1.91	40	d	0
			1557639_at	0	3.019	0.12	7	3.196	0.21	40	u	0.035048
CCL22	5	chemokine (C-C motif) ligand 22	207861_at	0	4.241	0.52	7	4.087	0.71	40	d	0.595789
EIF4G3	5	eukaryotic translation initiation factor 4 gamma, 3	201936_s_at	5	5.281	0.33	7	5.404	0.65	40	u	0.6371
			201935_s_at	5	7.274	0.41	7	7.178	0.83	40	d	0.76872
			1554310_a_at	0	3.406	0.6	7	3.849	0.72	40	u	0.140776
			1554309_at	0	3.376	0.61	7	3.381	0.54	40	u	0.984166
SNX16	5	sorting nexin 16	229618_at	5	6.138	0.53	7	6.579	0.91	40	u	0.23082
			219793_at	2	3.89	0.33	7	5.105	1.06	40	u	0.000006
ACBD3	5	acyl-Coenzyme A binding domain containing 3	202323_s_at	5	7.025	0.53	7	8.073	0.91	40	u	0.005901
			202324_s_at	5	9.568	0.29	7	10.1	0.54	40	u	0.016581
PRDM2	5	PR domain containing 2, with ZNF domain	205277_at	5	6.148	0.45	7	4.934	0.83	40	d	0.000566
			203056_s_at	0	5.533	0.53	7	5.703	1.01	40	u	0.673548
			216445_at	0	2.069	0.11	7	2.127	0.13	40	u	0.293758
			203057_s_at	0	8.207	0.27	7	7.817	0.43	40	d	0.029208
			216433_s_at	0	2.376	0.06	7	2.53	0.23	40	u	0.001206
WHSC1	5	Wolf-Hirschhorn syndrome candidate 1	223472_at	5	4.029	0.66	7	4.361	0.84	40	u	0.335625
			222777_s_at	4	4.358	0.56	7	5.025	0.9	40	u	0.069167
			222778_s_at	4	4.197	0.24	7	4.869	0.74	40	u	0.000163
			209053_s_at	3	5.467	0.3	7	7.678	0.7	40	u	0
			209052_s_at	3	3.538	0.25	7	5.351	1.49	40	u	0
			209054_s_at	3	6.275	0.28	7	7.072	0.55	40	u	0.000698
FAM98A	5	family with sequence similarity 98, member A	212333_at	5	8.697	0.24	7	9.05	0.64	40	u	0.16164
			239487_at	0	4.394	0.53	7	5.588	0.77	40	u	0.000377
			241832_at	0	3.625	0.28	7	4.281	0.6	40	u	0.007837
FBXO11	5	F-box protein 11	222119_s_at	5	8.792	0.21	7	8.481	0.62	40	d	0.025391
			203255_at	5	7.631	0.31	7	7.821	0.52	40	u	0.361609
			232364_at	0	2.088	0.08	7	2.102	0.04	40	u	0.664949
			219208_at	0	2.737	0.16	7	3.26	0.77	40	u	0.000556

MMP16	5	matrix metallopeptidase 16 (membrane-inserted)	207012_at	5	2.907	0.37	7	3.394	1.01	40	u	0.222788
			207013_s_at	1	4.552	0.18	7	4.854	0.52	40	u	0.011031
			208166_at	0	3.269	0.3	7	3.344	0.27	40	u	0.522507
			208167_s_at	0	3.726	0.44	7	4.011	0.51	40	u	0.181939
TAF12	5	TAF12 RNA polymerase II, TATA box binding protein (TBP)-associated factor, 20kDa	209463_s_at	0	7.493	0.31	7	7.024	0.96	40	d	0.027002
SLC9A9	5	solute carrier family 9 (sodium/hydrogen exchanger), member 9	227791_at	5	5.857	0.98	7	5.212	0.72	40	d	0.04871
			242587_at	2	3.69	0.59	7	3.401	0.45	40	d	0.153864
GNG12	5	guanine nucleotide binding protein (G protein), gamma 12	212294_at	5	10.18	0.21	7	8.743	1.13	40	d	0
			222834_s_at	2	8.268	0.83	7	7.657	1.37	40	d	0.268766
			1555240_s_at	0	6.135	1.07	7	6.021	1.09	40	d	0.804456
HDHD2	5	haloacid dehalogenase-like hydrolase domain containing 2	223155_at	5	9.208	0.25	7	8.569	0.8	40	d	0.000556
PBEF1	5	pre-B-cell colony enhancing factor 1	217738_at	5	9.68	0.46	7	9.143	0.95	40	d	0.159644
			217739_s_at	5	9.758	0.55	7	9.543	0.92	40	d	0.558179
			243296_at	0	9.656	0.8	7	8.73	1.2	40	d	0.060713
			1555167_s_at	0	7.515	0.61	7	7.585	1.85	40	u	0.857677
CAMSAP1	5	calmodulin regulated spectrin-associated protein 1	212712_at	5	6.469	0.21	7	6.922	0.72	40	u	0.003741
			212710_at	5	5.697	0.2	7	5.736	0.59	40	u	0.759946
			212711_at	5	6.568	0.29	7	6.536	0.59	40	d	0.890914
			220410_s_at	0	3.518	0.52	7	3.391	0.59	40	d	0.604615
			244682_at	0	3.371	0.29	7	3.407	0.33	40	u	0.792874
			228995_at	0	2.313	0.16	7	2.305	0.18	40	d	0.915743
			220409_at	0	2.656	0.07	7	2.914	0.42	40	u	0.000989
NFE2L2	5	nuclear factor (erythroid-derived 2)-like 2	201146_at	5	10.12	0.38	7	9.619	0.57	40	d	0.033056
			1567015_at	0	2.631	0.1	7	2.732	0.2	40	u	0.20457
			1567014_s_at	0	6.18	0.48	7	6.788	0.7	40	u	0.036831
			1567013_at	0	6.002	0.51	7	6.424	0.56	40	u	0.076649
RBBP6	5	retinoblastoma binding protein 6	212783_at	5	8.967	0.25	7	8.658	0.48	40	d	0.107985
			212781_at	5	7.425	0.22	7	7.623	0.5	40	u	0.314603
			232044_at	0	5.909	0.64	7	5.583	0.68	40	d	0.25246
			223802_s_at	0	7.75	0.19	7	7.705	0.78	40	d	0.760117
			228814_at	0	4.568	0.48	7	4.288	0.56	40	d	0.228348
			1552329_at	0	5.81	0.51	7	5.996	0.85	40	u	0.587032
			205178_s_at	0	8.028	0.36	7	7.915	0.52	40	d	0.589736
PDAP1	5	PDGFA associated protein 1	217624_at	5	2.885	0.33	7	3.155	0.7	40	u	0.336118
			202290_at	3	8.004	0.33	7	8.473	1.02	40	u	0.036048
HOXB5	5	homeobox B5	205601_s_at	5	6.168	0.43	7	5.752	0.68	40	d	0.130902
			205600_x_at	5	3.536	0.76	7	3.425	0.64	40	d	0.690686
LIMD2	5	LIM domain containing 2	218600_at	3	5.741	0.34	7	6.302	0.75	40	u	0.062944
TERF2	5	telomeric repeat binding factor 2	1555185_x_at	0	3.26	0.37	7	3.499	0.31	40	u	0.079937
			203611_at	0	8.143	0.36	7	7.829	0.53	40	d	0.149077
			229790_at	0	3.184	0.32	7	3.403	0.7	40	u	0.429206
			1555183_at	0	2.694	0.29	7	2.737	0.36	40	u	0.774737
HOXA5	5	homeobox A5	213844_at	5	8.441	0.23	7	3.798	1.34	40	d	0
PFTK1	5	PFTAIRE protein kinase 1	204604_at	5	8.586	0.45	7	8.003	1.1	40	d	0.184188
			211502_s_at	0	3.503	0.65	7	4.103	0.99	40	u	0.138891
UBE2J1	5	ubiquitin-conjugating enzyme E2, J1 (UBC6 homolog, yeast)	217824_at	5	6.803	0.34	7	6.734	0.86	40	d	0.838323
			217823_s_at	5	8.517	0.33	7	8.593	0.77	40	u	0.801596
			217826_s_at	1	7.966	0.41	7	8.606	0.91	40	u	0.080384
			217825_s_at	1	7.696	0.31	7	7.745	0.79	40	u	0.876635
			222435_s_at	1	8.151	0.41	7	8.711	0.93	40	u	0.131886
			228924_s_at	0	4.819	0.18	7	4.886	0.49	40	u	0.725973
SLC9A2	5	solute carrier family 9 (sodium/hydrogen exchanger), member 2	211116_at	0	2.338	0.17	7	2.738	0.65	40	u	0.003159
PTPN4	5	protein tyrosine phosphatase, non-receptor type 4 (megakaryocyte)	205171_at	2	6.662	0.48	7	7.188	0.73	40	u	0.078883
ELL	5	elongation factor RNA polymerase II	204095_s_at	5	3.129	0.43	7	3.067	0.47	40	d	0.75126
			204096_s_at	2	5.258	0.25	7	5.002	0.38	40	d	0.103441
			1565254_s_at	0	2.542	0.28	7	2.528	0.44	40	d	0.935992
UNKL	5	unkempt homolog (Drosophila)-like	221064_s_at	5	6.395	0.43	7	6.146	0.62	40	d	0.325965
			235087_at	0	2.724	0.5	7	2.583	0.11	40	d	0.517841
IL1RAPL1	5	interleukin 1 receptor accessory protein-like 1	220663_at	5	2.658	0.04	7	2.767	0.32	40	u	0.050041

IL1RAPL1	5	interleukin 1 receptor accessory protein-like 1	222963_s_at	1	2.18	0.08	7	2.326	0.62	40	u	0.173657
SLC35A1	5	solute carrier family 35 (CMP-sialic acid transporter), member A1	203306_s_at	4	9.537	0.28	7	8.961	0.73	40	d	0.049706
C14orf129	5	chromosome 14 open reading frame 129	223239_at	5	8.089	0.35	7	8.544	0.73	40	u	0.119156
CCDC28A	5	coiled-coil domain containing 28A	209479_at	5	8.873	0.36	7	8.073	0.85	40	d	0.02059
PPP4R1	5	protein phosphatase 4, regulatory subunit 1	201594_s_at	5	9.633	0.35	7	9.413	0.6	40	d	0.357879
			1563690_at	0	2.992	0.18	7	3.249	0.33	40	u	0.05553
KIAA1219	5	KIAA1219	221738_at	5	6.274	0.24	7	6.62	0.6	40	u	0.149045
			221736_at	5	8.758	0.24	7	8.172	0.66	40	d	0.028419
			224678_at	5	5.784	0.33	7	6.166	0.57	40	u	0.100346
FAM84B	5	family with sequence similarity 84, member B	225864_at	5	10.17	0.43	7	10.16	0.98	40	d	0.980715
			230817_at	0	1.945	0.02	7	1.983	0.03	40	u	0.006627
			228870_at	0	3.968	0.19	7	4.387	1.08	40	u	0.0327
ZFYVE9	5	zinc finger, FYVE domain containing 9	204893_s_at	5	6.015	0.33	7	5.887	0.95	40	d	0.535864
			208446_s_at	0	3.421	0.56	7	3.702	0.85	40	u	0.411694
KIF23	5	kinesin family member 23	204709_s_at	5	3.756	0.48	7	5.932	0.88	40	u	0
			244427_at	0	2.863	0.25	7	3.631	0.74	40	u	0.000042
TMEM16D	5	transmembrane protein 16D	236420_s_at	5	2.45	0.19	7	2.576	0.45	40	u	0.483407
			239883_s_at	2	3.304	0.51	7	3.512	0.57	40	u	0.383148
			229749_at	1	2.183	0.03	7	2.293	0.23	40	u	0.006021
GRM3	5	glutamate receptor, metabotropic 3	205814_at	5	2.321	0.06	7	2.372	0.08	40	u	0.123108
KIAA1600	5	KIAA1600	226155_at	5	8.054	0.16	7	7.532	0.88	40	d	0.001593
			1554288_at	0	2.579	0.07	7	2.672	0.17	40	u	0.155943
KIAA0408	5	KIAA0408	205496_at	5	2.853	0.1	7	2.894	0.2	40	u	0.597616
F13A1	5	coagulation factor XIII, A1 polypeptide	203305_at	5	8.659	1.22	7	6.494	1.36	40	d	0.000353
FVT1	5	follicular lymphoma variant translocation 1	202419_at	5	8.816	0.22	7	8.483	0.67	40	d	0.023775
UBN1	5	ubinuclein 1	207253_s_at	5	7.212	0.18	7	7.207	0.56	40	d	0.964761
			209088_s_at	5	8.135	0.4	7	8.83	0.58	40	u	0.00481
SLC31A1	5	solute carrier family 31 (copper transporters), member 1	235013_at	3	7.634	0.46	7	7.738	0.81	40	u	0.749938
			203971_at	1	6.175	0.37	7	6.932	0.63	40	u	0.004321
			236217_at	0	4.447	0.61	7	4.754	0.69	40	u	0.285533
MAP3K7IP2	5	mitogen-activated protein kinase kinase kinase 7 interacting protein 2	212184_s_at	5	9.121	0.22	7	8.963	0.61	40	d	0.249472
			210284_s_at	1	8.184	0.34	7	7.991	1.41	40	d	0.468722
SLC8A2	5	solute carrier family 8 (sodium-calcium exchanger), member 2	215267_s_at	5	2.707	0.34	7	3.17	0.94	40	u	0.033354
NDRG1	5	N-myc downstream regulated gene 1	200632_s_at	5	10.16	0.35	7	10.19	1.42	40	u	0.923032
AFAP1	5	actin filament associated protein 1	203563_at	5	3.699	0.37	7	3.798	0.44	40	u	0.583446
KIAA0423	5	KIAA0423	213304_at	5	8.223	0.32	7	7.485	0.87	40	d	0.00073
PPP2R2A	5	protein phosphatase 2 (formerly 2A), regulatory subunit B, alpha isoform	202313_at	5	9.287	0.34	7	8.931	0.63	40	d	0.161066
			236492_at	0	8.365	0.23	7	7.434	0.99	40	d	0.000011
			237636_at	0	2.113	0.07	7	2.133	0.07	40	u	0.458557
PLEKHH1	5	pleckstrin homology domain containing, family H (with MyTH4 domain) member 1	225727_at	5	6.169	0.8	7	5.032	1.16	40	d	0.018586
			225726_s_at	5	6.838	0.69	7	5.946	1.26	40	d	0.080459
RBBP7	5	retinoblastoma binding protein 7	201092_at	5	10.19	0.15	7	10.49	0.57	40	u	0.011812
USP33	5	ubiquitin specific peptidase 33	212513_s_at	5	9.715	0.12	7	9.307	0.59	40	d	0.00037
			217441_at	5	1.992	0.11	7	2.293	0.46	40	u	0.001438
			214843_s_at	0	8.453	0.31	7	8.779	0.72	40	u	0.257725
FBXL17	5	F-box and leucine-rich repeat protein 17	227203_at	5	7.744	0.33	7	7.475	0.7	40	d	0.332158
			244360_at	0	3.447	0.12	7	3.542	0.35	40	u	0.221305
			233087_at	0	3.757	0.25	7	3.59	0.46	40	d	0.362199
			242034_at	0	2.316	0.4	7	2.399	0.53	40	u	0.701838
ZBED4	5	zinc finger, BED-type containing 4	204799_at	5	4.876	0.58	7	5.811	1.06	40	u	0.03136
LRRC1	5	leucine rich repeat containing 1	218816_at	5	8.048	0.59	7	8.392	0.82	40	u	0.304096
			207790_at	0	4.87	0.55	7	5.184	0.98	40	u	0.421198
FBXL16	5	F-box and leucine-rich repeat protein 16	227641_at	5	7.334	0.54	7	7.141	1.75	40	d	0.591534
			232409_x_at	0	2.123	0.2	7	2.182	0.33	40	u	0.65375
TMEM16C	5	transmembrane protein 16C	215241_at	5	2.37	0.12	7	2.554	0.56	40	u	0.073511
OSBP2	5	oxysterol binding protein 2	223432_at	5	4.112	0.28	7	4.029	0.65	40	d	0.747396
			1569617_at	0	4.731	0.51	7	5.057	0.59	40	u	0.186035
			221237_s_at	0	4.25	0.29	7	4.258	0.37	40	u	0.956158
RAPGEF5	5	Rap guanine nucleotide exchange factor (GEF) 5	204681_s_at	5	7.484	0.2	7	7.707	0.87	40	u	0.175702
			204680_s_at	5	2.225	0.07	7	2.819	0.78	40	u	0.000038
			1561269_at	0	2.163	0.07	7	2.284	0.24	40	u	0.0154
			1563882_a_at	0	1.938	0.09	7	2.004	0.24	40	u	0.490836

KBTBD2	5	kelch repeat and BTB (POZ) domain containing 2	212447_at	5	8.549	0.31	7	8.384	0.44	40	d	0.352443
			223584_s_at	0	6.392	0.57	7	7.148	1.02	40	u	0.068573
			223585_x_at	0	6.679	0.46	7	6.794	1.4	40	u	0.69861
			234232_at	0	2.959	0.25	7	3.12	0.34	40	u	0.251568
			234793_at	0	2.273	0.02	7	2.373	0.12	40	u	0.000031
			234233_s_at	0	4.133	0.67	7	3.911	0.45	40	d	0.280125
SULF1	5	sulfatase 1	212354_at	5	8.107	0.44	7	9.455	1.51	40	u	0.000099
			212353_at	5	6.543	0.82	7	8.777	1.53	40	u	0.000611
			212344_at	5	7.104	0.43	7	8.188	1.78	40	u	0.002388
FGF13	5	fibroblast growth factor 13	205110_s_at	5	8.097	0.53	7	7.97	1.52	40	d	0.701569
MARCKSL1	5	MARCKS-like 1	200644_at	5	11.57	0.34	7	11.67	0.76	40	u	0.752271
DAGLA	5	diacylglycerol lipase, alpha	214128_at	5	3.551	0.34	7	3.349	0.46	40	d	0.280783
PAK4	5	p21(CDKN1A)-activated kinase 4	203154_s_at	5	8.426	0.34	7	8.609	0.6	40	u	0.443438
			33814_at	5	8.66	0.49	7	8.886	0.64	40	u	0.384087
			215326_at	0	2.501	0.3	7	2.513	0.36	40	u	0.936167
TBC1D15	5	TBC1 domain family, member 15	218268_at	1	8.568	0.24	7	8.585	0.51	40	u	0.93404
SIM2	5	single-minded homolog 2 (Drosophila)	206558_at	5	3.339	0.26	7	4.086	1.21	40	u	0.001575
			1557900_at	0	2.181	0.16	7	2.264	0.23	40	u	0.366069
			208157_at	0	3.142	0.24	7	3.322	0.32	40	u	0.169664
TCF20	5	transcription factor 20 (AR1)	212931_at	5	8.413	0.2	7	8.461	0.58	40	u	0.701722
			215511_at	0	3.22	0.37	7	3.549	0.72	40	u	0.252624
			238667_at	0	1.999	0.06	7	2.047	0.07	40	u	0.096298
TFEB	5	transcription factor EB	221866_at	5	7.049	0.27	7	6.877	0.6	40	d	0.473163
			50221_at	0	6.043	0.31	7	5.762	1.06	40	d	0.196178
NEK9	5	NIMA (never in mitosis gene a)- related kinase 9	214738_s_at	5	5.183	0.81	7	4.638	1.1	40	d	0.227031
			212299_at	0	8.505	0.26	7	7.423	0.71	40	d	0.000302
			230153_at	0	2.782	0.15	7	2.917	0.24	40	u	0.16314
CUTL1	5	cut-like 1, CCAAT displacement protein (Drosophila)	214743_at	5	6.386	0.47	7	6.57	1.16	40	u	0.687878
			202367_at	0	6.908	0.38	7	6.851	0.79	40	d	0.853782
SFMBT1	5	Scm-like with four mbt domains 1	213370_s_at	5	6.302	0.28	7	6.329	0.73	40	u	0.92261
			239054_at	0	3.424	0.51	7	3.777	0.73	40	u	0.236254
DEK	5	DEK oncogene (DNA binding)	200934_at	5	10.4	0.2	7	10.96	0.94	40	u	0.002097
ARHGAP24	5	Rho GTPase activating protein 24	223422_s_at	5	6.918	0.32	7	5.823	0.84	40	d	0.001655
			221030_s_at	5	2.677	0.29	7	2.597	0.33	40	d	0.556408
			230803_s_at	0	3.019	0.25	7	3.316	0.38	40	u	0.059824
LIN7C	5	lin-7 homolog C (C. elegans)	223350_x_at	5	8.666	0.2	7	8.714	0.66	40	u	0.722217
			219399_at	5	6.633	0.5	7	7.184	0.94	40	u	0.146294
			221568_s_at	5	8.603	0.23	7	8.729	0.7	40	u	0.389858
STX6	5	syntaxin 6	212799_at	5	7.469	0.35	7	8.61	0.56	40	u	0.000006
			212800_at	5	4.906	0.65	7	6.862	0.8	40	u	0
			214441_at	0	5.422	0.12	7	6.045	0.76	40	u	0.000025
			244041_at	0	3.117	0.27	7	2.898	0.4	40	d	0.178649
			1552618_at	0	6.524	0.29	7	8	0.79	40	u	0
SLC26A7	5	solute carrier family 26, member 7	239006_at	5	3.186	0.51	7	3.833	1.45	40	u	0.048155
			1552827_s_at	0	2.299	0.03	7	2.457	0.33	40	u	0.00598
			238214_at	0	4.956	0.6	7	5.383	1.19	40	u	0.368823
			1552826_at	0	2.794	0.09	7	3.123	0.38	40	u	0.000044
LBH	5	limb bud and heart development homolog (mouse)	221011_s_at	5	8.79	0.59	7	7.437	1.17	40	d	0.005406
DYNLT3	5	dynein, light chain, Tctex-type 3	203303_at	5	9.833	0.32	7	10.1	0.72	40	u	0.348995
SESTD1	5	SEC14 and spectrin domains 1	226763_at	4	9.166	0.43	7	9.339	0.76	40	u	0.566199
FAM13A1	5	family with sequence similarity 13, member A1	202973_x_at	5	9.241	0.29	7	7.579	0.97	40	d	0
			217047_s_at	5	9.099	0.46	7	7.362	0.94	40	d	0.000027
			202972_s_at	5	7.89	0.38	7	6.898	0.83	40	d	0.004092
			1569025_s_at	0	3.592	0.38	7	4.034	1.06	40	u	0.065776
			1569024_at	0	2.951	0.17	7	3.327	0.44	40	u	0.035678
PSD3	5	pleckstrin and Sec7 domain containing 3	203354_s_at	4	5.498	1.38	7	6.058	1.8	40	u	0.447736
			203355_s_at	4	8.448	0.52	7	7.868	1.68	40	d	0.10135
			218613_at	1	6.433	0.58	7	5.776	1.53	40	d	0.277868
DUSP8	5	dual specificity phosphatase 8	206374_at	5	7.693	0.45	7	6.612	0.88	40	d	0.003194
LZTS2	5	leucine zipper, putative tumor suppressor 2	224473_x_at	5	7.088	0.37	7	6.882	0.58	40	d	0.382031
			1555881_s_at	5	7.961	0.22	7	7.564	0.69	40	d	0.010116
			225013_at	5	2.098	0.11	7	2.19	0.19	40	u	0.238293
			232129_s_at	0	6.268	0.34	7	5.485	1.03	40	d	0.00116

ZDHHC2	5	zinc finger, DHHC-type containing 2	222731_at	5	7.811	0.64	7	6.457	1.22	40	d	0.007289
			222730_s_at	5	5.486	0.65	7	5.189	0.74	40	d	0.332424
			1563502_at	0	2.074	0.27	7	2.021	0.08	40	d	0.646619
C5orf24	5	chromosome 5 open reading frame 24	224876_at	5	10.33	0.24	7	10.12	0.54	40	d	0.328739
			224875_at	5	6.243	0.35	7	6.531	0.73	40	u	0.323314
			1553106_at	1	4.04	0.41	7	5.165	0.96	40	u	0.004684
			1553107_s_at	1	5.659	0.26	7	6.273	0.81	40	u	0.001081
			1553108_at	1	4.025	0.31	7	4.614	0.64	40	u	0.024992
			229098_s_at	0	2.677	0.21	7	2.674	0.16	40	d	0.970347
MSN	5	moesin	200600_at	5	9.868	0.36	7	9.744	0.98	40	d	0.747138
			233749_at	0	2.594	0.06	7	2.612	0.19	40	u	0.646156
			240960_at	0	5.992	0.78	7	4.586	1.01	40	d	0.001305
ARHGAP17	5	Rho GTPase activating protein 17	218076_s_at	5	8.58	0.21	7	8.129	0.73	40	d	0.003907
PTPRZ1	5	protein tyrosine phosphatase, receptor-type, Z polypeptide 1	204469_at	5	6.262	0.68	7	3.386	1.36	40	d	0.000003
TIMP3	5	TIMP metallopeptidase inhibitor 3 (Sorsby fundus dystrophy, pseudoinflammatory)	201147_s_at	5	9.903	0.23	7	9.078	1.35	40	d	0.001075
			201150_s_at	5	12.09	0.31	7	10.41	1.47	40	d	0
			201148_s_at	5	9.131	0.2	7	7.512	1.57	40	d	0
			201149_s_at	0	9.384	0.38	7	8.394	1.56	40	d	0.00178
HISPPD1	5	histidine acid phosphatase domain containing 1	203253_s_at	5	7.781	0.31	7	7.663	0.68	40	d	0.663202
YIPF4	5	Yip1 domain family, member 4	209551_at	5	4.77	0.39	7	5.774	0.83	40	u	0.003656
			213999_at	0	3.532	0.37	7	3.707	0.55	40	u	0.433131
PRKAR2A	5	protein kinase, cAMP-dependent, regulatory, type II, alpha	204842_x_at	5	9.076	0.36	7	9.109	0.53	40	u	0.878142
			213052_at	3	9.196	0.19	7	8.895	0.7	40	d	0.032492
			204843_s_at	1	4.505	0.49	7	3.952	0.79	40	d	0.087643
			225000_at	0	10.42	0.2	7	10.08	0.72	40	d	0.023997
			225011_at	0	9.314	0.12	7	9.18	0.7	40	d	0.279956
LRRC16	5	leucine rich repeat containing 16	219573_at	5	6.232	0.21	7	6.168	0.64	40	d	0.631877
			230793_at	5	7.534	0.47	7	7.271	1.21	40	d	0.580908
			1562939_at	0	3.583	0.35	7	3.738	0.54	40	u	0.475404
			1558931_at	0	2.315	0.07	7	2.598	0.52	40	u	0.00244
ARF4	5	ADP-ribosylation factor 4	201096_s_at	5	9.825	0.33	7	10.07	0.93	40	u	0.227986
			201097_s_at	5	11.57	0.2	7	11.7	0.58	40	u	0.310112
LRRC62	5	leucine rich repeat containing 62	1560713_a_at	5	2.774	0.05	7	2.89	0.32	40	u	0.037305
			1559072_a_at	5	3.288	0.25	7	3.542	0.68	40	u	0.345096
			1563108_at	3	3.272	0.47	7	3.174	0.59	40	d	0.683984
NEFM	5	neurofilament, medium polypeptide 150kDa	205113_at	5	2.491	0.08	7	2.574	0.21	40	u	0.320279
			223902_at	0	1.677	0.03	7	1.697	0.03	40	u	0.120314
ZBTB2	5	zinc finger and BTB domain containing 2	226284_at	5	7.049	0.3	7	7.092	0.64	40	u	0.86499
LRRC4	5	leucine rich repeat containing 4	223552_at	3	2.861	0.28	7	2.99	0.39	40	u	0.41692
ARMC8	5	armadillo repeat containing 8	219094_at	5	4.334	0.36	7	4.7	0.7	40	u	0.189635
			1555281_x_at	5	5.402	0.47	7	5.642	0.51	40	u	0.259345
			1555279_at	5	5.263	0.29	7	5.487	0.63	40	u	0.36997
			244528_at	0	2.63	0.18	7	2.613	0.15	40	d	0.799889
			203486_s_at	0	7.376	0.17	7	7.043	0.56	40	d	0.006732
			203487_s_at	0	6.942	0.28	7	6.478	0.71	40	d	0.099916
			236966_at	0	6.273	0.74	7	5.931	0.94	40	d	0.373467
CORO2B	5	coronin, actin binding protein, 2B	209789_at	5	3.572	0.72	7	2.391	0.17	40	d	0.007191
MARCH5	5	membrane-associated ring finger (C3HC4) 5	226394_at	5	6.847	0.48	7	6.447	0.62	40	d	0.116909
			218582_at	0	7.975	0.44	7	8.067	0.83	40	u	0.781466
DCLRE1B	5	DNA cross-link repair 1B (PSO2 homolog, S. cerevisiae)	222889_at	5	5.086	0.35	7	5.666	0.94	40	u	0.121779
			219490_s_at	0	4.044	0.31	7	4.807	0.87	40	u	0.00046
PRKCD	5	protein kinase C, delta	202545_at	0	6.587	0.66	7	6.884	1.18	40	u	0.528937
COL4A3	5	collagen, type IV, alpha 3 (Goodpasture antigen)	222073_at	2	3.168	0.68	7	2.93	1.06	40	d	0.576304
			216896_at	0	2.249	0.08	7	2.293	0.11	40	u	0.313004
			216368_s_at	0	2.286	0.08	7	2.426	0.12	40	u	0.005004
			216898_s_at	0	3.199	0.33	7	3.396	0.39	40	u	0.22053
			216367_at	0	2.331	0.03	7	2.405	0.06	40	u	0.003187
			216893_s_at	0	2.824	0.12	7	2.887	0.21	40	u	0.447428
			214641_at	0	2.33	0.11	7	2.515	0.47	40	u	0.038514
PRKAA1	5	protein kinase, AMP-activated, alpha 1 catalytic subunit	225984_at	5	7.008	0.3	7	6.641	0.78	40	d	0.238563
			225985_at	5	6.331	0.28	7	5.541	0.68	40	d	0.005134
			214917_at	0	4.449	0.48	7	4.251	1.03	40	d	0.6296

		catalytic subunit	209799_at	0	6.351	0.53	7	6.256	1.23	40	d	0.844604
			1555177_at	0	2.443	0.09	7	2.524	0.22	40	u	0.352238
RCC2	5	regulator of chromosome condensation 2	224578_at	5	9.188	0.33	7	9.566	0.62	40	u	0.13245
GRHL2	5	grainyhead-like 2 (Drosophila)	219388_at	5	7.425	0.36	7	8.19	0.74	40	u	0.012386
ABCC4	5	ATP-binding cassette, sub-family C (CFTR/MRP), member 4	203196_at	5	6.207	1.09	7	6.415	1.66	40	u	0.75574
			1555039_a_at	0	2.881	0.59	7	3.684	1.63	40	u	0.03419
			243928_s_at	0	2.701	0.13	7	2.999	0.56	40	u	0.006314
			1554918_a_at	0	3.236	1.06	7	3.841	1.97	40	u	0.442829
RNF2	5	ring finger protein 2	205215_at	5	6.336	0.17	7	6.461	0.38	40	u	0.410054
TMEM47	5	transmembrane protein 47	209655_s_at	5	7.562	0.52	7	6.767	1.49	40	d	0.019792
			209656_s_at	5	9.476	0.3	7	7.962	1.51	40	d	0.000001
MAP3K8	5	mitogen-activated protein kinase kinase kinase 8	205027_s_at	5	5.814	0.29	7	5.914	0.94	40	u	0.608004
			235421_at	0	5.96	0.68	7	5.404	1.27	40	d	0.273209
FRMD6	5	FERM domain containing 6	225481_at	5	10.16	0.4	7	8.184	1.16	40	d	0
			225464_at	5	10.49	0.33	7	8.555	1.09	40	d	0
PLS1	5	plastin 1 (I isoform)	205190_at	5	6.141	0.59	7	6.093	1.61	40	d	0.939181
CGGBP1	5	CGG triplet repeat binding protein 1	224600_at	5	9.306	0.26	7	8.998	0.88	40	d	0.08912
			224599_at	5	10.16	0.24	7	9.68	0.68	40	d	0.003197
			206861_s_at	0	7.108	0.58	7	7.768	0.81	40	u	0.049329
LZTS1	5	leucine zipper, putative tumor suppressor 1	219042_at	5	4.372	0.86	7	3.315	0.73	40	d	0.001514
			47550_at	5	4.325	0.61	7	3.608	0.39	40	d	0.000252
			222107_x_at	2	5.567	0.27	7	5.537	0.47	40	d	0.8738
			221722_x_at	0	1.976	0.02	7	2.033	0.08	40	u	0.000294
			221719_s_at	0	3.065	0.23	7	3.069	0.13	40	u	0.967408
			221721_s_at	0	2.144	0.03	7	2.196	0.04	40	u	0.002415
STAT3	5	signal transducer and activator of transcription 3 (acute-phase response factor)	225289_at	5	8.832	0.25	7	7.659	0.78	40	d	0
			208991_at	1	11.52	0.15	7	10.76	0.93	40	d	0.000026
			243213_at	0	4.756	0.44	7	5.12	0.98	40	u	0.34745
			208992_s_at	0	9.931	0.21	7	9.125	1.19	40	d	0.000366
PHC2	5	polyhomeotic homolog 2 (Drosophila)	200919_at	5	8.984	0.25	7	8.612	0.47	40	d	0.053206
			238131_at	0	3.068	0.4	7	3.029	0.33	40	d	0.784658
PPP2R5C	5	protein phosphatase 2, regulatory subunit B', gamma isoform	201877_s_at	4	9.379	0.3	7	8.987	0.62	40	d	0.115761
			1557718_at	0	5.053	0.66	7	4.427	0.66	40	d	0.028721
			213305_s_at	0	7.242	0.32	7	7.822	0.78	40	u	0.062717
			214083_at	0	4.255	0.76	7	4.241	0.84	40	d	0.966393
			1554364_at	0	1.814	0.02	7	1.882	0.06	40	u	0.00003
			1554365_a_at	0	7.609	0.51	7	8.305	0.88	40	u	0.053271
DPYSL5	5	dihydropyrimidinase-like 5	222797_at	5	1.947	0.02	7	2.05	0.23	40	u	0.009103
			224100_s_at	1	4.614	0.16	7	4.708	0.22	40	u	0.298296
EIF5A2	5	eukaryotic translation initiation factor 5A2	235289_at	5	3.889	0.47	7	3.704	0.63	40	d	0.469813
			235296_at	3	3.58	0.31	7	3.723	0.92	40	u	0.466177
			220198_s_at	1	2.759	0.13	7	2.979	0.26	40	u	0.035947
PTER	5	phosphotriesterase related	218967_s_at	2	7.411	0.53	7	7.304	1.23	40	d	0.827007
			222798_at	2	8.319	0.27	7	8.276	1.07	40	d	0.834551
SNX2	5	sorting nexin 2	202114_at	5	8.524	0.15	7	8.309	0.49	40	d	0.038044
			202113_s_at	0	8.721	0.3	7	8.269	0.96	40	d	0.029769
EREG	5	epiregulin	205767_at	5	5.63	1.63	7	3.66	1.36	40	d	0.001663
			1569583_at	0	2.635	0.28	7	2.562	0.51	40	d	0.722011
NDFIP2	5	Nedd4 family interacting protein 2	224799_at	5	7.558	0.45	7	7.98	1	40	u	0.289099
			224802_at	5	7.57	0.52	7	7.985	0.81	40	u	0.205806
			224801_at	5	7.533	0.51	7	6.81	0.9	40	d	0.049723
			230190_at	0	2.296	0.25	7	2.338	0.29	40	u	0.722321
PRUNE2	5	prune homolog 2 (Drosophila)	212805_at	5	6.504	0.67	7	5.8	1.32	40	d	0.182891
			212806_at	5	2.862	0.25	7	3.173	0.82	40	u	0.07234
			1552455_at	0	2.432	0.21	7	2.63	0.82	40	u	0.214587
NMT2	5	N-myristoyltransferase 2	205005_s_at	5	5.61	0.87	7	5.342	1.31	40	d	0.612755
			205006_s_at	5	5.4	0.52	7	5.107	1.04	40	d	0.479411
			215743_at	0	3.291	0.3	7	3.259	0.56	40	d	0.885947
			215069_at	0	4.29	0.48	7	4.309	0.53	40	u	0.931656
MARCH7	5	membrane-associated ring finger (C3HC4) 7	202653_s_at	5	8.923	0.27	7	9.017	0.51	40	u	0.642809
			202654_x_at	4	9.021	0.36	7	9.062	0.93	40	u	0.910653
			1557704_a_at	0	3.374	0.15	7	3.411	0.21	40	u	0.664009
			232371_at	0	6.249	0.36	7	6.317	0.56	40	u	0.758646
			1557703_at	0	2.962	0.1	7	3.128	0.22	40	u	0.060259

ATG9A	5	ATG9 autophagy related 9 homolog A (S. cerevisiae)	202492_at	5	6.626	0.34	7	6.325	0.89	40	d	0.389536
MTUS1	5	mitochondrial tumor suppressor 1	212093_s_at	5	8.309	0.47	7	6.265	1.39	40	d	0
			212095_s_at	5	7.847	0.38	7	6.948	1.02	40	d	0.028439
			212096_s_at	5	10.46	0.21	7	8.232	1.14	40	d	0
			239576_at	0	7.24	0.47	7	6.095	0.7	40	d	0.0002
ARFIP2	5	ADP-ribosylation factor interacting protein 2 (arfaptin 2)	202109_at	3	10.29	0.37	7	9.511	0.84	40	d	0.023083
SLITRK6	5	SLIT and NTRK-like family, member 6	235976_at	5	5.019	1.23	7	5.213	1.96	40	u	0.805389
			232176_at	4	6.089	1.46	7	5.77	2.55	40	d	0.75414
			232481_s_at	2	7.305	1.8	7	6.224	2.93	40	d	0.359308
KCTD9	5	potassium channel tetramerisation domain containing 9	218823_s_at	5	8.115	0.5	7	7.338	1.18	40	d	0.100775
			224316_at	0	3.466	0.64	7	3.785	0.77	40	u	0.31517
PRRT3	5	proline-rich transmembrane protein 3	1556308_at	5	4.473	0.57	7	4.462	1.13	40	d	0.980979
PRKCB1	5	protein kinase C, beta 1	209685_s_at	4	4.867	0.79	7	5.048	1.45	40	u	0.754786
			227817_at	0	4.157	0.71	7	4.307	0.83	40	u	0.66182
			207957_s_at	0	3.549	0.54	7	3.424	1.1	40	d	0.773796
			228795_at	0	2.179	0.06	7	2.339	0.24	40	u	0.001693
			230437_s_at	0	2.118	0.12	7	2.155	0.15	40	u	0.541828
			227824_at	0	2.537	0.17	7	2.674	0.18	40	u	0.075061
LYPD6	5	LY6/PLAUR domain containing 6	227763_at	5	3.211	0.95	7	2.51	0.21	40	d	0.120323
			227764_at	5	6.778	0.67	7	3.786	1.21	40	d	0
			239028_at	0	3.286	0.65	7	2.982	0.43	40	d	0.12758
PKN2	5	protein kinase N2	212629_s_at	5	8.896	0.39	7	9.488	0.78	40	u	0.060756
			212628_at	5	8.431	0.26	7	8.128	0.7	40	d	0.276684
			210969_at	0	2.88	0.26	7	3.131	0.5	40	u	0.208171
			230400_s_at	0	2.285	0.18	7	2.982	0.81	40	u	0.000035
CLDN12	5	claudin 12	223249_at	5	8.51	0.54	7	8.053	0.66	40	d	0.096588
ALS2CR13	5	amyotrophic lateral sclerosis 2 (juvenile) chromosome region, candidate 13	226431_at	8	6.039	0.38	7	6.568	1.07	40	u	0.032462
			1553220_at	5	2.746	0.25	7	3.277	0.65	40	u	0.042656
			1564868_a_at	0	2.338	0.08	7	2.368	0.18	40	u	0.666887
CREB3L2	5	cAMP responsive element binding protein 3-like 2	212345_s_at	5	9.474	0.32	7	8.894	1.07	40	d	0.010967
			228759_at	0	4.03	0.49	7	4.447	0.88	40	u	0.237435
			237819_at	0	2.987	0.23	7	3.177	0.48	40	u	0.323723
MOV10	5	Mov10, Moloney leukemia virus 10, homolog (mouse)	223849_s_at	5	7.364	0.21	7	7.298	0.65	40	d	0.629236
			233917_s_at	5	5.958	0.25	7	6.225	0.65	40	u	0.302635
LATS2	5	LATS, large tumor suppressor, homolog 2 (Drosophila)	223379_s_at	5	2.871	0.34	7	2.91	0.45	40	u	0.829059
			223380_s_at	5	8.083	0.44	7	7.385	0.9	40	d	0.0551
			227013_at	0	8.989	0.52	7	7.695	0.98	40	d	0.001676
			230348_at	0	2.77	0.21	7	2.867	0.36	40	u	0.505871
DDIT4	5	DNA-damage-inducible transcript 4	202887_s_at	5	9.414	0.65	7	10.65	1.22	40	u	0.013691
DNAJA2	5	DnaJ (Hsp40) homolog, subfamily A, member 2	226994_at	5	8.802	0.29	7	8.443	0.78	40	d	0.243606
			209157_at	4	8.756	0.33	7	8.525	0.59	40	d	0.329217
			223850_at	0	2.382	0.19	7	2.557	0.28	40	u	0.127862
PSAP	5	prosaposin (variant Gaucher disease and variant metachromatic leukodystrophy)	200871_s_at	4	12.96	0.11	7	12.83	0.52	40	d	0.182452
			200866_s_at	1	11.26	0.17	7	10.95	1.18	40	d	0.126342
GDAP2	5	ganglioside induced differentiation associated protein 2	219473_at	5	3.14	0.38	7	4.108	1.16	40	u	0.000428
			1554154_at	0	6.915	0.17	7	7.061	0.87	40	u	0.353032
KIAA0907	5	KIAA0907	202220_at	5	9.775	0.25	7	10.08	0.56	40	u	0.175719
			230028_at	0	5.42	0.41	7	6.624	0.9	40	u	0.001365
SYNC1	5	syncoilin, intermediate filament 1	221276_s_at	5	5.589	0.83	7	5.243	1.09	40	d	0.438159
			237333_at	1	6.175	0.49	7	6.568	1.12	40	u	0.377404
HCN2	5	hyperpolarization activated cyclic nucleotide-gated potassium channel 2	214893_x_at	5	4.642	0.27	7	4.594	0.4	40	d	0.771925
			207592_s_at	5	2.95	0.3	7	3.035	0.27	40	u	0.464452
SDC1	5	syndecan 1	201287_s_at	5	9.609	0.32	7	10.91	1.25	40	u	0.000004
			201286_at	5	8.908	0.46	7	9.567	1.34	40	u	0.029703
RNF144B	5	ring finger 144B	228153_at	5	9.843	0.37	7	9.255	1.05	40	d	0.015729
			239012_at	5	7.167	0.7	7	6.754	1.13	40	d	0.364469
			235549_at	2	3.656	0.39	7	3.92	1.07	40	u	0.531344
			239704_at	0	3.189	0.29	7	3.357	0.73	40	u	0.561153
HNRPH3	5	heterogeneous nuclear ribonucleoprotein H3 (2H9)	208990_s_at	5	9.976	0.3	7	9.34	0.47	40	d	0.00156
			210588_x_at	4	9.589	0.2	7	9.276	0.44	40	d	0.077743
			207127_s_at	3	9.012	0.17	7	8.877	0.53	40	d	0.2206
			210110_x_at	2	9.233	0.16	7	8.988	0.48	40	d	0.023041

ABCD1	5	ATP-binding cassette, sub-family D (ALD), member 1	205142_x_at	5	5.884	0.25	7	6.279	0.84	40	u	0.025012
			216418_at	0	3.159	0.27	7	3.177	0.36	40	u	0.905695
TMEM109	5	transmembrane protein 109	201361_at	5	9.603	0.26	7	9.044	0.76	40	d	0.001799
FAM43A	5	family with sequence similarity 43, member A	227410_at	5	7.288	0.53	7	5.735	0.95	40	d	0.000153
			1558174_at	0	2.081	0.18	7	1.996	0.15	40	d	0.199645
DYNC1I1	5	dynein, cytoplasmic 1, intermediate chain 1	205348_s_at	5	4.221	0.69	7	3.953	1.26	40	d	0.593315
			1569843_at	0	2.266	0.03	7	2.39	0.22	40	u	0.001773
C12orf53	5	chromosome 12 open reading frame 53	228165_at	5	2.822	0.6	7	2.72	0.54	40	d	0.658749
			1553566_at	0	3.04	0.48	7	3.186	0.43	40	u	0.431092
TCERG1L	5	transcription elongation regulator 1-like	231257_at	5	1.94	0.05	7	2.037	0.31	40	u	0.082136
SFRS7	5	splicing factor, arginine/serine-rich 7, 35kDa	201129_at	5	9.629	0.21	7	9.311	0.58	40	d	0.01815
			211439_at	4	2.386	0.21	7	2.665	0.32	40	u	0.035278
			213649_at	4	9.746	0.37	7	9.22	0.56	40	d	0.024623
			214141_x_at	4	11.12	0.28	7	10.97	0.38	40	d	0.366751
DSEL	5	dermatan sulfate epimerase-like	232235_at	5	5.2	0.72	7	4.74	1.49	40	d	0.436565
			232825_s_at	3	3.443	0.15	7	3.769	0.59	40	u	0.006799
			244852_at	0	2.737	0.09	7	3.212	0.79	40	u	0.000818
TMOD1	5	tropomodulin 1	203662_s_at	6	4.795	0.86	7	3.339	0.95	40	d	0.000543
			203661_s_at	1	5.056	0.69	7	4.407	1.25	40	d	0.194941
CANX	5	calnexin	208852_s_at	5	11.42	0.29	7	11.04	1.06	40	d	0.069972
			200068_s_at	5	12.26	0.18	7	12.01	0.52	40	d	0.032671
			238034_at	0	6.778	0.28	7	6.553	0.65	40	d	0.384629
			208853_s_at	0	9.79	0.52	7	9.644	1.24	40	d	0.764255
			241712_at	0	2.539	0.23	7	2.536	0.29	40	d	0.981323
AOF1	5	amine oxidase (flavin containing) domain 1	227021_at	5	6.266	0.36	7	7.673	0.76	40	u	0.000026
			1553150_at	2	3.325	0.28	7	5.252	1.46	40	u	0
ENC1	5	ectodermal-neural cortex (with BTB-like domain)	201340_s_at	0	4.886	0.45	7	6.881	1.63	40	u	0
			201341_at	0	8.047	0.43	7	8.131	1.22	40	u	0.751306
KCNMB2	5	potassium large conductance calcium-activated channel, subfamily M, beta	223823_at	5	2.821	0.53	7	2.838	0.45	40	u	0.928911
			221097_s_at	4	2.516	0.21	7	2.506	0.18	40	d	0.900126
KIAA0430	5	KIAA0430	202386_s_at	5	9.431	0.33	7	8.706	0.69	40	d	0.010277
			1558697_a_at	0	2.618	0.19	7	3.136	0.96	40	u	0.004208
C20orf108	5	chromosome 20 open reading frame 108	224693_at	5	8.635	0.47	7	8.498	0.92	40	d	0.707837
			224690_at	5	11.36	0.27	7	10.55	0.67	40	d	0.00344
JUB	5	jub, ajuba homolog (Xenopus laevis)	1553764_a_at	5	7.208	0.58	7	6.259	1.07	40	d	0.030623
			225807_at	5	5.887	0.54	7	5.455	0.57	40	d	0.073954
			225806_at	5	7.824	0.4	7	6.663	1.32	40	d	0.000151
			243446_at	0	2.313	0.18	7	2.362	0.17	40	u	0.502453
TCF7	5	transcription factor 7 (T-cell specific, HMG-box)	205255_x_at	5	7.873	0.28	7	7.178	0.64	40	d	0.008006
			205254_x_at	5	3.542	0.51	7	2.738	0.47	40	d	0.000231
STRN4	5	striatin, calmodulin binding protein 4	217903_at	3	7.384	0.32	7	7.031	0.84	40	d	0.285265
PAQR9	5	progestin and adipoQ receptor family member IX	1558322_a_at	5	2.587	0.06	7	2.693	0.15	40	u	0.072215
			1560467_at	0	2.238	0.03	7	2.363	0.24	40	u	0.003381
ZBTB5	5	zinc finger and BTB domain containing 5	203026_at	5	7.573	0.28	7	7.442	0.54	40	d	0.542845
DLX1	5	distal-less homeobox 1	242138_at	5	2.669	0.82	7	3.245	1.67	40	u	0.387423
			1560100_at	0	3.651	0.37	7	3.738	0.62	40	u	0.726551
RELN	5	reelin	205923_at	5	6.85	1.09	7	4.824	0.4	40	d	0.004027
SERBP1	5	SERPINE1 mRNA binding protein 1	210076_x_at	5	7.484	0.37	7	8.051	0.78	40	u	0.072041
			217724_at	5	11.38	0.18	7	11.18	0.65	40	d	0.118891
			217725_x_at	5	9.56	0.41	7	9.835	1.17	40	u	0.284893
			210466_s_at	4	10.74	0.15	7	11.32	0.76	40	u	0.00011
			209669_s_at	1	10.02	0.23	7	10.87	0.82	40	u	0.000008
			228129_at	0	6.618	0.33	7	6.788	0.82	40	u	0.599236
			227369_at	0	7.964	0.28	7	7.766	0.65	40	d	0.441616
PNN	5	pinin, desmosome associated protein	212037_at	5	9.708	0.28	7	9.27	0.54	40	d	0.047708
			212036_s_at	5	10.77	0.19	7	9.901	0.6	40	d	0
			1567213_at	0	5.524	0.5	7	5.136	0.91	40	d	0.290489
			1567214_a_at	0	9.667	0.2	7	9.166	0.6	40	d	0.000604
NXPH1	5	neurexophilin 1	232377_at	5	2.877	0.28	7	3.582	1.68	40	u	0.020192
ANP32B	5	acidic (leucine-rich) nuclear phosphoprotein 32 family, member B	201305_x_at	5	11.28	0.1	7	11.61	0.59	40	u	0.001894
			201306_s_at	5	11.92	0.15	7	12.04	0.61	40	u	0.282925
WAC	5	WW domain containing adaptor with coiled-coil	217742_s_at	5	10.1	0.16	7	10.26	0.53	40	u	0.139346
			222389_s_at	4	10.67	0.18	7	10.9	0.57	40	u	0.064604
			219679_s_at	0	8.381	0.51	7	8.991	1.03	40	u	0.138705

			242427_at	0	4.266	0.37	7	4.281	0.62	40	u	0.951374
PAPOLB	5	poly(A) polymerase beta (testis specific)	242158_at	1	2.329	0.02	7	2.39	0.05	40	u	0.006766
			208271_at	0	2.791	0.29	7	2.755	0.28	40	d	0.763047
SMEK2	5	SMEK homolog 2, suppressor of mek1 (Dictyostelium)	226230_at	5	8.347	0.25	7	8.438	0.78	40	u	0.575238
			233759_s_at	5	8.83	0.17	7	9.287	0.68	40	u	0.001058
			224474_x_at	2	8.46	0.33	7	9.14	0.88	40	u	0.055547
			222270_at	0	5.693	0.46	7	5.517	0.68	40	d	0.519584
			1568627_at	0	3.133	0.47	7	3.652	0.82	40	u	0.116738
			241103_at	0	2.926	0.11	7	3.14	0.38	40	u	0.00856
SFRS11	5	splicing factor, arginine/serine-rich 11	200686_s_at	5	11.3	0.25	7	11.27	0.48	40	d	0.847284
			200685_at	3	7.902	0.12	7	6.576	0.93	40	d	0
			236948_x_at	0	3.468	0.49	7	3.699	0.68	40	u	0.404389
			213742_at	0	7.146	0.46	7	7.281	0.93	40	u	0.714148
			237746_at	0	3.839	0.73	7	4.336	1.18	40	u	0.297726
SAR1B	5	SAR1 gene homolog B (S. cerevisiae)	223512_at	5	6.498	0.62	7	6.56	0.96	40	u	0.871357
			218254_s_at	5	8.304	0.82	7	8.728	0.98	40	u	0.298179
			230397_at	0	2.624	0.32	7	2.747	0.77	40	u	0.684578
			1554482_a_at	0	7.059	0.93	7	7.091	1.12	40	u	0.944069
TOB1	5	transducer of ERBB2, 1	202704_at	5	10.47	0.57	7	10.09	1.22	40	d	0.432409
			228834_at	0	9.965	0.8	7	9.865	2.21	40	d	0.837601
FCHSD1	5	FCH and double SH3 domains 1	226698_at	5	4.875	0.38	7	4.448	0.8	40	d	0.181397
			226699_at	5	2.875	0.11	7	3.006	0.3	40	u	0.263461
HAPLN1	5	hyaluronan and proteoglycan link protein 1	205523_at	5	3.143	0.37	7	3.778	1.68	40	u	0.046018
			205524_s_at	2	2.31	0.19	7	2.892	1.81	40	u	0.058953
RAB7A	5	RAB7A, member RAS oncogene family	211961_s_at	5	9.697	0.17	7	9.558	0.47	40	d	0.448461
			211960_s_at	5	8.245	0.34	7	8.953	0.83	40	u	0.035432
			217267_s_at	4	2.119	0.02	7	2.177	0.06	40	u	0.000022
			217268_at	4	2.664	0.07	7	2.779	0.22	40	u	0.014579
			227602_at	0	2.925	0.35	7	3.299	0.53	40	u	0.083578
SPOCK1	5	sparc/osteonectin, cwcv and kazal-like domains proteoglycan (testican) 1	202363_at	5	6.11	0.35	7	6.017	1.6	40	d	0.75364
RNGTT	5	RNA guanylyltransferase and 5'-phosphatase	204208_at	5	7.022	0.16	7	7.513	0.58	40	u	0.000146
			204207_s_at	2	4.74	0.23	7	6.106	1.06	40	u	0
			211387_x_at	0	4.984	0.29	7	5.341	0.66	40	u	0.176159
			211849_s_at	0	3.645	0.28	7	4.531	0.87	40	u	0.000036
TACC2	5	transforming, acidic coiled-coil containing protein 2	202289_s_at	5	8.005	0.6	7	7.097	1.52	40	d	0.133623
			211382_s_at	2	7.982	0.51	7	7.035	1.33	40	d	0.076464
			1570546_a_at	0	2.135	0.02	7	2.2	0.06	40	u	0.000112
			1570025_at	0	3.007	0.41	7	3.09	0.65	40	u	0.751436
PAK1	5	p21/Cdc42/Rac1-activated kinase 1 (STE20 homolog, yeast)	226507_at	5	7.753	0.22	7	8.161	0.91	40	u	0.021178
			209615_s_at	2	4.853	0.48	7	5.718	0.88	40	u	0.016586
			230100_x_at	0	4.96	0.52	7	4.48	1.48	40	d	0.144732
			1565772_at	0	2.675	0.61	7	3.234	0.75	40	u	0.072841
PELI2	5	pellino homolog 2 (Drosophila)	219132_at	5	6.766	0.6	7	4.735	0.91	40	d	0.000001
ZNF131	5	zinc finger protein 131	221842_s_at	5	8.119	0.21	7	8.858	0.6	40	u	0.000005
			225916_at	5	5.739	0.37	7	6.315	0.6	40	u	0.021087
			214741_at	5	7.399	0.48	7	8.039	0.78	40	u	0.044383
			1557384_at	0	6.632	0.33	7	6.625	0.96	40	d	0.971828
HOXA13	5	homeobox A13	231786_at	5	2.72	0.14	7	2.857	0.24	40	u	0.154024
AK3L1	5	adenylate kinase 3-like 1	204348_s_at	1	9.279	0.49	7	8.305	1.41	40	d	0.003682
			225342_at	0	9.769	0.34	7	7.98	1.36	40	d	0
ELOVL5	5	ELOVL family member 5, elongation of long chain fatty acids (FEN1/Elo2, SUR4/Elo3-like, yeast)	208788_at	5	12.49	0.22	7	10.96	1.02	40	d	0
			1567222_x_at	0	4.783	0.5	7	4.572	0.57	40	d	0.373107
			214153_at	0	6.392	0.49	7	5.035	0.9	40	d	0.000437
			215082_at	0	4.951	0.38	7	4.703	0.6	40	d	0.306735
HERC2	5	hect domain and RLD 2	217902_s_at	5	7.63	0.58	7	6.801	1	40	d	0.042977
			222461_s_at	5	2.038	0.02	7	2.22	0.37	40	u	0.004611
BCLAF1	5	BCL2-associated transcription factor 1	201084_s_at	5	11.16	0.17	7	10.81	0.45	40	d	0.046663
			201101_s_at	5	7.821	0.5	7	9.026	1.01	40	u	0.004139
			201083_s_at	5	6.624	0.34	7	7.595	0.59	40	u	0.000161
			214499_s_at	1	7.539	0.4	7	8.728	0.84	40	u	0.000766
			239897_at	0	5.977	0.6	7	5.539	0.65	40	d	0.111618
			229454_at	0	4.87	0.91	7	4.185	0.85	40	d	0.06412
			217600_at	5	2.712	0.16	7	2.948	0.37	40	u	0.112496

SCUBE3	5	signal peptide, CUB domain, EGF-like 3	230290_at	4	3.695	0.49	7	4.161	1.17	40	u	0.314713
			230253_at	4	4.703	0.55	7	4.418	0.5	40	d	0.188228
			228407_at	0	4.29	0.22	7	5.964	1.63	40	u	0
RTF1	5	Rtf1, Paf1/RNA polymerase II complex component, homolog (S. cerevisiae)	212302_at	5	7.395	0.25	7	7.59	0.53	40	u	0.350963
			212301_at	5	8.968	0.35	7	8.727	0.55	40	d	0.277125
			230832_at	0	2.779	0.41	7	2.733	0.43	40	d	0.798667
GATA6	5	GATA binding protein 6	210002_at	5	4.841	0.7	7	5.093	1.7	40	u	0.707652
			229282_at	0	3.473	0.35	7	3.622	0.39	40	u	0.355552
DMRT3	5	doublesex and mab-3 related transcription factor 3	233930_at	5	1.792	0.04	7	1.824	0.04	40	u	0.047051
			231800_s_at	5	2.884	0.34	7	2.917	0.53	40	u	0.877226
DENND1A	5	DENN/MADD domain containing 1A	226849_at	5	6.254	0.36	7	5.836	0.71	40	d	0.142466
			219763_at	0	2.864	0.33	7	3.239	0.81	40	u	0.246997
LTBP1	5	latent transforming growth factor beta binding protein 1	202728_s_at	5	4.472	0.76	7	6.348	1.62	40	u	0.005149
			202729_s_at	5	8.347	0.31	7	8.743	1.27	40	u	0.108029
CBFA2T2	5	core-binding factor, runt domain, alpha subunit 2; translocated to, 2	207625_s_at	5	7.406	0.49	7	7.133	0.74	40	d	0.363362
			209144_s_at	5	5.556	0.33	7	5.332	0.69	40	d	0.411411
			209145_s_at	1	2.576	0.28	7	2.746	0.42	40	u	0.316111
			1554637_a_at	0	4.578	0.63	7	4.918	0.84	40	u	0.325018
			238549_at	0	8.218	0.48	7	8.319	1.07	40	u	0.811679
MARK3	5	MAP/microtubule affinity-regulating kinase 3	202569_s_at	5	7.93	0.27	7	7.973	0.48	40	u	0.82411
			202568_s_at	5	7.618	0.17	7	7.229	0.45	40	d	0.032812
			232537_x_at	0	3.668	0.39	7	3.8	0.38	40	u	0.414522
CBX6	5	chromobox homolog 6	202047_s_at	5	8.674	0.33	7	7.624	0.89	40	d	0.004047
			202048_s_at	5	9.082	0.41	7	8.336	0.52	40	d	0.001005
NEK6	5	NIMA (never in mitosis gene a)-related kinase 6	223158_s_at	5	7.065	0.39	7	7.159	1.01	40	u	0.811691
			223159_s_at	5	6.028	0.43	7	6.227	0.78	40	u	0.522001
			223561_at	2	1.831	0.1	7	1.916	0.13	40	u	0.106995
			239267_at	0	4.694	0.46	7	4.595	0.5	40	d	0.634286
			237761_at	0	2.384	0.04	7	2.513	0.21	40	u	0.001192
PGM2L1	5	phosphoglucomutase 2-like 1	229256_at	5	6.106	0.88	7	6.425	1.17	40	u	0.503105
			238417_at	2	5.994	0.59	7	6.233	0.8	40	u	0.463288
			229553_at	0	7.013	0.75	7	7.888	1.02	40	u	0.038953
			1569448_at	0	4.356	0.45	7	4.191	0.57	40	d	0.479928
			235149_at	0	3.665	0.6	7	4.29	0.64	40	u	0.022609
KLHL9	5	kelch-like 9 (Drosophila)	213117_at	5	8.301	0.37	7	8.228	0.77	40	d	0.81002
			213233_s_at	5	9.709	0.27	7	9.303	0.83	40	d	0.026862
			237123_x_at	0	2.871	0.12	7	3.107	0.3	40	u	0.049432
			233197_at	0	3.94	0.41	7	3.742	0.89	40	d	0.573745
C6orf62	5	chromosome 6 open reading frame 62	208809_s_at	5	9.685	0.21	7	9.998	0.48	40	u	0.106712
			213875_x_at	0	7.148	0.43	7	7.745	0.98	40	u	0.128462
			222309_at	0	3.066	0.35	7	3.601	0.79	40	u	0.093184
			213872_at	0	9.833	0.57	7	9.903	2.29	40	u	0.873223
WNT3A	5	less-type MMTV integration site family, membe	no probeset available									
EDAR	5	ectodysplasin A receptor	220048_at	5	3.657	0.59	7	3.862	1	40	u	0.609451
ITGB1	5	integrin, beta 1 (fibronectin receptor, beta polypeptide, antigen CD29 includes MDF2, MSK12)	211945_s_at	5	12.37	0.24	7	12.22	0.52	40	d	0.468172
			1553678_a_at	2	11.53	0.37	7	11.23	1.34	40	d	0.258286
			216190_x_at	0	2.63	0.05	7	2.763	0.23	40	u	0.002757
			216178_x_at	0	2.711	0.06	7	3.009	0.39	40	u	0.000058
			1553530_a_at	0	10.47	0.44	7	10.45	1.22	40	d	0.931638
			215878_at	0	2.3	0.03	7	2.392	0.09	40	u	0.000015
			1561042_at	0	2.306	0.04	7	3.013	1.04	40	u	0.000143
			215879_at	0	2.675	0.22	7	2.713	0.16	40	u	0.59248
BAI1	5	brain-specific angiogenesis inhibitor 1	206083_at	5	5.182	0.28	7	4.833	0.45	40	d	0.057119
EPS15	5	epidermal growth factor receptor pathway substrate 15	217886_at	5	8.453	0.22	7	7.269	0.6	40	d	0.000007
			217887_s_at	1	9.796	0.23	7	9.561	0.53	40	d	0.266406
WNT5A	5	wingless-type MMTV integration site family, member 5A	213425_at	5	7.128	0.55	7	6.015	1.38	40	d	0.045039
			231227_at	4	4.29	0.82	7	4.453	1.02	40	u	0.697039
			205990_s_at	3	8.223	0.82	7	6.946	1.75	40	d	0.070513
TEX2	5	testis expressed 2	218099_at	5	8.547	0.36	7	8.261	1.02	40	d	0.204877
ATP6V1A	5	ATPase, H+ transporting, lysosomal 70kDa, V1 subunit A	201972_at	5	9.856	0.25	7	10.08	0.52	40	u	0.28374
			201971_s_at	2	7.88	0.43	7	7.685	1.71	40	d	0.549584
STK3	5	serine/threonine kinase 3 (STE20 homolog, yeast)	204068_at	2	8.096	0.3	7	8.795	0.88	40	u	0.000988
			211078_s_at	0	3.602	0.18	7	4.614	0.96	40	u	0
COL19A1	5	collagen, type XIX, alpha 1	211011_at	0	2.378	0.09	7	2.427	0.25	40	u	0.610616

FAM122A	5	family with sequence similarity 122A	235043_at	5	5.215	0.51	7	4.095	0.56	40	d	0.000014
			226443_at	2	5.775	0.54	7	5.138	0.89	40	d	0.080638
UBE2Q2	5	ubiquitin-conjugating enzyme E2Q (putative) 2	224747_at	5	9.326	0.2	7	9.515	0.68	40	u	0.178588
SYT7	5	synaptotagmin VII	226167_at	5	5.497	0.2	7	5.546	0.76	40	u	0.739184
			232025_at	3	3.202	0.31	7	3.417	0.64	40	u	0.401948
			1559392_s_at	0	2.725	0.17	7	2.83	0.25	40	u	0.305338
			1559956_at	0	3.269	0.17	7	3.581	0.22	40	u	0.001036
DPY19L3	5	dpy-19-like 3 (C. elegans)	225633_at	5	7.123	0.31	7	6.595	1.06	40	d	0.017792
OLIG3	5	oligodendrocyte transcription factor 3	1556371_at	5	2.417	0.05	7	2.492	0.08	40	u	0.029982
DMTF1	5	cyclin D binding myb-like transcription factor 1	203301_s_at	5	8.948	0.42	7	8.494	0.6	40	d	0.067875
EPHB3	5	EPH receptor B3	204600_at	5	8.48	0.44	7	8.249	1.13	40	d	0.604302
			1438_at	0	7.553	0.62	7	6.914	1.55	40	d	0.298007
RASGEF1A	5	RasGEF domain family, member 1A	230563_at	5	6.642	0.66	7	6.175	1.99	40	d	0.273953
			242917_at	2	2.717	0.31	7	2.878	0.47	40	u	0.396962
PCTK3	5	PCTAIRE protein kinase 3	214797_s_at	5	3.263	0.33	7	3.863	1.15	40	u	0.013267
MFAP3L	5	microfibrillar-associated protein 3-like	210843_s_at	5	2.804	0.5	7	2.758	0.99	40	d	0.905779
			205442_at	0	7.895	1.18	7	6.546	1.74	40	d	0.059744
			210493_s_at	0	3.654	0.29	7	3.775	0.72	40	u	0.669539
			210492_at	0	2.609	0.23	7	2.822	0.57	40	u	0.348803
TGFB1	5	transforming growth factor, beta 1	203085_s_at	0	6.428	0.41	7	6.579	1.18	40	u	0.555642
			203084_at	0	4.621	0.5	7	4.01	0.62	40	d	0.019417
SLC31A2	5	solute carrier family 31 (copper transporters), member 2	204204_at	5	7.75	0.73	7	7.404	0.98	40	d	0.385881
ZFAND6	5	zinc finger, AN1-type domain 6	221613_s_at	5	9.845	0.27	7	9.87	0.6	40	u	0.916063
			239757_at	0	6.118	0.43	7	6.209	1.3	40	u	0.739891
			222186_at	0	7.224	0.25	7	6.825	0.98	40	d	0.039489
IGFBP5	5	insulin-like growth factor binding protein 5	211958_at	5	7.459	0.59	7	6.917	2.03	40	d	0.190525
			211959_at	5	10.71	0.6	7	10.11	1.82	40	d	0.126863
			1555997_s_at	0	5.178	0.66	7	5.693	1.65	40	u	0.431115
			203426_s_at	0	6.004	0.4	7	5.944	1.32	40	d	0.82516
			203424_s_at	0	7.006	0.45	7	7.301	1.73	40	u	0.380205
			203425_s_at	0	6.154	0.48	7	6.445	1.48	40	u	0.350934
DLL4	5	delta-like 4 (Drosophila)	223525_at	5	6.265	0.28	7	6.154	0.43	40	d	0.51969
CTTNBP2	5	cortactin binding protein 2	232136_s_at	5	4.111	0.61	7	3.937	1.46	40	d	0.761352
ARHGAP20	5	Rho GTPase activating protein 20	1555020_a_at	5	3.123	0.21	7	3.148	0.32	40	u	0.847532
			228368_at	5	5.449	0.65	7	3.374	0.65	40	d	0
TRPM7	5	transient receptor potential cation channel, subfamily M, member 7	223324_s_at	5	7.274	0.31	7	6.958	0.75	40	d	0.290136
			223323_x_at	5	5.177	0.42	7	5.998	0.67	40	u	0.003746
			237884_x_at	0	3.792	0.4	7	4.259	0.48	40	u	0.021381
			231689_at	0	4.82	0.34	7	4.803	0.44	40	d	0.924534
			1565887_at	0	2.497	0.16	7	2.716	0.6	40	u	0.064854
TLN2	5	talin 2	212703_at	5	6.592	0.2	7	6.269	0.5	40	d	0.106164
			232625_at	0	2.12	0.05	7	2.165	0.05	40	u	0.044534
CTNNBIP1	5	catenin, beta interacting protein 1	203081_at	5	8.083	0.38	7	7.222	0.78	40	d	0.007272
TEP1	5	telomerase-associated protein 1	228670_at	0	6.588	0.28	7	6.071	0.78	40	d	0.005929
			205727_at	0	2.873	0.16	7	3.127	0.42	40	u	0.132765
GNAZ	5	guanine nucleotide binding protein (G protein), alpha z polypeptide	204993_at	5	4.306	0.64	7	4.509	1.13	40	u	0.654213
KIAA0182	5	KIAA0182	212057_at	5	9.056	0.44	7	8.768	1.33	40	d	0.309424
			212056_at	5	8.628	0.32	7	7.907	1.47	40	d	0.010267
			232988_at	0	4.572	0.4	7	4.485	0.43	40	d	0.62801
TNFSF11	5	tumor necrosis factor (ligand) superfamily, member 11	210643_at	5	4.67	1.96	7	3.644	0.67	40	d	0.251068
			211153_s_at	0	2.732	1.1	7	2.377	0.45	40	d	0.464303
OPCML	5	opioid binding protein/cell adhesion molecule-like	214111_at	5	3.229	0.42	7	3.478	0.47	40	u	0.202473
			206215_at	3	2.544	0.19	7	2.671	0.33	40	u	0.342947
PHACTR2	5	phosphatase and actin regulator 2	204047_s_at	5	4.969	0.52	7	5.593	1.01	40	u	0.125213
			204049_s_at	5	8.119	0.33	7	7.675	0.75	40	d	0.13807
			204048_s_at	5	8.253	0.48	7	8.241	0.79	40	d	0.971182
			227947_at	2	7.895	0.36	7	7.527	0.76	40	d	0.226463
SCN1B	5	sodium channel, voltage-gated, type I, beta	205508_at	4	5.497	0.24	7	5.161	0.79	40	d	0.043944
RAI2	5	retinoic acid induced 2	219440_at	5	8.181	0.51	7	5.102	1.37	40	d	0.000001

NFE2L1	5	nuclear factor (erythroid-derived 2)-like 1	200759_x_at	5	9.857	0.08	7	9.353	0.55	40	d	0.000004
			214179_s_at	5	9.086	0.14	7	8.649	0.63	40	d	0.000564
			200758_s_at	5	10.42	0.12	7	9.755	0.68	40	d	0.000002
CALN1	5	calneuron 1	223885_at	5	1.907	0.02	7	1.955	0.09	40	u	0.007468
			1555168_a_at	2	2.142	0.13	7	2.176	0.3	40	u	0.776092
LMBR1L	5	limb region 1 homolog (mouse)-like	220036_s_at	5	7.542	0.2	7	6.906	0.63	40	d	0.000041
SLC43A2	5	solute carrier family 43, member 2	226629_at	5	6.469	0.16	7	6.429	0.59	40	d	0.726537
DNM2	5	dynamin 2	202253_s_at	5	7.709	0.5	7	7.087	0.71	40	d	0.034941
			216024_at	5	2.202	0.03	7	2.273	0.08	40	u	0.000165
			1555895_at	2	2.265	0.25	7	2.406	0.47	40	u	0.450787
YTHDF2	5	YTH domain family, member 2	217812_at	5	9.539	0.22	7	9.811	0.39	40	u	0.082343
			222430_s_at	0	10.43	0.32	7	10.27	0.51	40	d	0.440761
FZD8	5	frizzled homolog 8 (Drosophila)	224325_at	5	5.207	0.87	7	4.486	1.73	40	d	0.297998
			227405_s_at	4	8.051	0.57	7	6.801	1.79	40	d	0.002104
			216587_s_at	0	1.953	0.07	7	2.542	1.51	40	u	0.020099
PCDH17	5	protocadherin 17	205656_at	2	4.922	0.45	7	6.044	0.91	40	u	0.00311
			228863_at	0	4.086	0.49	7	5.825	0.8	40	u	0.000002
			227289_at	0	4.036	0.75	7	5.483	0.9	40	u	0.000277
RIC8B	5	resistance to inhibitors of cholinesterase 8 homolog B (C. elegans)	219446_at	5	6.874	0.23	7	6.819	0.78	40	d	0.725027
			229637_at	0	6.469	0.49	7	6.008	0.68	40	d	0.098601
ADAMTS3	5	ADAM metallopeptidase with thrombospondin type 1 motif, 3	214913_at	5	3.44	0.45	7	3.155	1.09	40	d	0.506066
SMG7	5	Smg-7 homolog, nonsense mediated mRNA decay factor (C. elegans)	201793_x_at	5	3.176	0.62	7	4.398	0.87	40	u	0.001158
			201794_s_at	5	8.675	0.29	7	9.233	0.51	40	u	0.00886
			217189_s_at	0	4.013	0.54	7	5.637	1.09	40	u	0.000445
TGFBR3	5	transforming growth factor, beta receptor III	204731_at	5	10.35	0.46	7	6.35	1.71	40	d	0
SRA1	5	steroid receptor RNA activator 1	224130_s_at	1	7.47	0.29	7	7.37	0.77	40	d	0.740749
			224864_at	1	7.357	0.27	7	7.555	0.68	40	u	0.459508
MIPOL1	5	mirror-image polydactyly 1	244246_at	4	5.325	0.68	7	5.29	0.99	40	d	0.931688
			1552572_a_at	1	3.503	0.3	7	4.041	0.58	40	u	0.022872
			1552573_s_at	1	4.526	0.63	7	4.942	0.77	40	u	0.190484
			1570052_at	0	2.607	0.05	7	2.745	0.19	40	u	0.000687
RPS6KA1	5	ribosomal protein S6 kinase, 90kDa, polypeptide 1	203379_at	5	7.666	0.28	7	7.397	0.73	40	d	0.354915
ABCA2	5	ATP-binding cassette, sub-family A (ABC1), member 2	210099_at	5	2.879	0.12	7	2.879	0.21	40	d	0.996482
			212772_s_at	5	6.273	0.26	7	5.927	0.95	40	d	0.072329
			210100_s_at	1	4.752	0.55	7	4.583	1.13	40	d	0.7047
STXBP1	5	syntaxin binding protein 1	202260_s_at	5	7.597	0.39	7	5.539	1.63	40	d	0
ADM	5	adrenomedullin	202912_at	5	8.087	0.83	7	7.092	1.49	40	d	0.098383
SLCO5A1	5	solute carrier organic anion transporter family, member 5A1	220984_s_at	5	2.36	0.03	7	2.69	0.69	40	u	0.005267
RSPO3	5	R-spondin 3 homolog (Xenopus laevis)	228186_s_at	5	6.948	1.33	7	4.728	1.07	40	d	0.00002
ZNF207	5	zinc finger protein 207	200829_x_at	5	9.389	0.25	7	9.464	0.42	40	u	0.659655
			200828_s_at	5	10.45	0.31	7	10.33	0.79	40	d	0.699962
			1556035_s_at	0	8.384	0.37	7	7.43	0.97	40	d	0.01573
			228157_at	0	8.373	0.66	7	7.288	1.21	40	d	0.028378
			239937_at	0	7.097	0.4	7	7.139	0.61	40	u	0.864463
			238772_at	0	2.646	0.04	7	2.721	0.11	40	u	0.092881
			231848_x_at	0	8.774	0.52	7	7.976	0.73	40	d	0.009358
NXT2	5	nuclear transport factor 2-like export factor 2	209628_at	5	8.146	0.24	7	8.598	1	40	u	0.020458
			209629_s_at	4	4.88	1.13	7	6.032	1.71	40	u	0.098723
GOLGA8G	5	golgi autoantigen, golgin subfamily a, 8G	222149_x_at	0	5.533	0.61	7	4.941	0.62	40	d	0.025932
SBNO1	5	strawberry notch homolog 1 (Drosophila)	216161_at	0	3.432	0.57	7	3.325	0.48	40	d	0.610756
			218737_at	0	5.277	0.35	7	5.721	0.64	40	u	0.089355
			216162_at	0	2.437	0.05	7	2.57	0.18	40	u	0.000716
B4GALT5	5	UDP-Gal:betaGlcNAc beta 1,4-galactosyltransferase, polypeptide 5	221485_at	5	8.814	0.15	7	9.091	0.74	40	u	0.044122
			221484_at	5	8.394	0.48	7	9.067	0.96	40	u	0.083055
ACTG1	5	actin, gamma 1	211983_x_at	5	14.08	0.19	7	14.19	0.24	40	u	0.238368
			211970_x_at	5	13.86	0.19	7	13.91	0.25	40	u	0.60022
			224585_x_at	5	13.99	0.21	7	14.11	0.23	40	u	0.21211
			221607_x_at	5	14.2	0.2	7	14.31	0.23	40	u	0.253971
			211995_x_at	5	14.26	0.18	7	14.21	0.33	40	d	0.725516
			201550_x_at	5	14.11	0.2	7	14.15	0.25	40	u	0.714749
			213214_x_at	5	13.94	0.23	7	14.07	0.25	40	u	0.210537

			212988_x_at	5	13.94	0.2	7	14.03	0.23	40	u	0.329333
			212363_x_at	5	13.91	0.21	7	13.84	0.33	40	d	0.573772
TRIM36	5	tripartite motif-containing 36	219736_at	5	5.318	0.75	7	4.724	1.62	40	d	0.356311
			231123_at	0	2.701	0.47	7	2.624	0.63	40	d	0.765221
			1565812_at	0	1.904	0.01	7	1.937	0.03	40	u	0.012594
			1565814_at	0	1.991	0.15	7	2.075	0.2	40	u	0.306104
SLC16A1	5	solute carrier family 16, member 1 (monocarboxylic acid transporter 1)	202235_at	5	3.61	0.53	7	3.972	1.19	40	u	0.440639
			202234_s_at	5	4.48	0.89	7	5.267	1.73	40	u	0.255425
			202236_s_at	5	7.58	0.98	7	7.938	1.68	40	u	0.595073
			209900_s_at	5	4.754	0.98	7	5.537	1.68	40	u	0.24811
			1557918_s_at	0	4.05	0.97	7	5.015	1.79	40	u	0.181601
PDE4A	5	phosphodiesterase 4A, cAMP-specific (phosphodiesterase E2 dunce homolog, Drosophila)	204735_at	5	6.198	0.49	7	5.753	1.26	40	d	0.371844
			211447_s_at	0	2.691	0.28	7	3.078	0.69	40	u	0.158205
			211901_s_at	0	5.336	0.16	7	5.051	0.62	40	d	0.020423
			242435_at	0	3.164	0.51	7	3.103	0.31	40	d	0.680345
			211591_s_at	0	3.462	0.4	7	3.411	0.31	40	d	0.714137
PPM1D	5	protein phosphatase 1D magnesium-dependent, delta isoform	204566_at	5	7.248	0.35	7	7.079	1.18	40	d	0.480972
C6orf134	5	chromosome 6 open reading frame 134	218874_s_at	5	6.776	0.33	7	6.901	0.63	40	u	0.616692
			228510_at	0	4.835	0.32	7	4.517	1.1	40	d	0.158457
B3GNT2	5	UDP-GlcNAc:betaGal beta-1,3-N-acetylglucosaminyltransferase 2	222870_s_at	5	9.109	0.44	7	9.089	0.59	40	d	0.933382
			224154_at	0	2.415	0.04	7	2.507	0.07	40	u	0.003439
			219326_s_at	0	7.959	0.62	7	7.548	1.34	40	d	0.440458
RLF	5	rearranged L-myc fusion	204243_at	5	6.822	0.37	7	6.757	0.73	40	d	0.822699
RYR3	5	ryanodine receptor 3	206306_at	5	3.863	0.85	7	2.714	0.85	40	d	0.002433
CTNND2	5	catenin (cadherin-associated protein), delta 2 (neural plakophilin-related arm-repeat	209618_at	5	2.154	0.17	7	2.515	0.9	40	u	0.029037
			209617_s_at	1	5.451	0.8	7	5.345	1.17	40	d	0.822523
SETD2	5	SET domain containing 2	212493_s_at	5	8.461	0.18	7	8.083	0.62	40	d	0.004479
			220946_s_at	0	5.468	0.29	7	5.877	0.8	40	u	0.197963
			215038_s_at	0	8.411	0.16	7	7.786	0.66	40	d	0.000009
KLF11	5	Kruppel-like factor 11	1553137_s_at	3	4.352	0.45	7	4.723	1	40	u	0.348819
			218486_at	0	8.113	0.57	7	7.437	0.82	40	d	0.047233
GPR3	5	G protein-coupled receptor 3	214613_at	4	2.894	0.12	7	3.067	0.37	40	u	0.034532
GJA1	5	gap junction protein, alpha 1, 43kDa	201667_at	5	11.31	0.35	7	10.42	1.25	40	d	0.000909
GNAI2	5	guanine nucleotide binding protein (G protein), alpha inhibiting activity polypeptide 2	201040_at	2	9.691	0.41	7	9.599	0.59	40	d	0.69936
AKAP7	5	A kinase (PRKA) anchor protein 7	205772_s_at	5	2.301	0.07	7	2.412	0.15	40	u	0.068583
			205771_s_at	0	6.406	0.62	7	6.506	1.19	40	u	0.83237
			211172_x_at	0	5.866	0.24	7	5.928	0.31	40	u	0.626467
LIN54	5	lin-54 homolog (C. elegans)	235809_at	5	2.74	0.25	7	2.854	0.23	40	u	0.242879
GDF6	5	growth differentiation factor 6	no probeset available									
GGA3	5	golgi associated, gamma adaptin ear containing, ARF binding protein 3	209411_s_at	5	7.405	0.34	7	7.162	0.76	40	d	0.422445
			211815_s_at	0	3.535	0.31	7	4.071	0.95	40	u	0.011326
CDKN1B	5	cyclin-dependent kinase inhibitor 1B (p27, Kip1)	209112_at	5	9.94	0.31	7	9.916	1.03	40	d	0.909288
CSF1	5	colony stimulating factor 1 (macrophage)	209716_at	2	7.721	0.33	7	7.192	0.39	40	d	0.001925
			210557_x_at	1	3.542	0.54	7	3.654	0.7	40	u	0.694728
			211839_s_at	0	3.193	0.43	7	3.127	0.42	40	d	0.706156
			207082_at	0	2.89	0.3	7	2.998	0.39	40	u	0.503238
MXD4	5	MAX dimerization protein 4	212346_s_at	5	7.366	0.15	7	6.287	0.89	40	d	0
			212347_x_at	5	7.05	0.27	7	6.89	0.7	40	d	0.564947
			210778_s_at	2	6.978	0.17	7	6.612	0.7	40	d	0.008368
ULK1	5	unc-51-like kinase 1 (C. elegans)	209333_at	5	6.627	0.35	7	5.664	1	40	d	0.000165
ADAMTS9	5	ADAM metallopeptidase with thrombospondin type 1 motif, 9	233785_at	0	2.993	0.29	7	3.179	0.33	40	u	0.181037
			226814_at	0	5.699	1.11	7	4.648	1.01	40	d	0.018173
			220287_at	0	5.517	1.63	7	3.681	0.93	40	d	0.035749
			1554697_at	0	3.273	0.62	7	2.996	0.66	40	d	0.316434
CDH2	5	cadherin 2, type 1, N-cadherin (neuronal)	203440_at	5	4.533	0.87	7	5.171	1.21	40	u	0.197113
			203441_s_at	0	4.13	0.39	7	4.134	0.72	40	u	0.9875
ARF1	5	ADP-ribosylation factor 1	208750_s_at	5	9.135	0.29	7	9.672	1.5	40	u	0.050399
			200065_s_at	4	12.1	0.17	7	12.64	0.47	40	u	0.000024
			1565651_at	0	5.908	0.9	7	5.885	0.82	40	d	0.949239
			232175_at	0	6.653	0.43	7	6.763	0.56	40	u	0.631894

			244504_x_at	0	3.021	0.31	7	3.185	0.59	40	u	0.480922
ATP7A	5	ATPase, Cu++ transporting, alpha polypeptide (Menkes syndrome)	205197_s_at	4	6.221	1.07	7	5.919	0.67	40	d	0.340475
ACTR1A	5	ARP1 actin-related protein 1 homolog A, centractin alpha (yeast)	200720_s_at	5	8.058	0.32	7	8.195	0.75	40	u	0.642875
			200721_s_at	5	8.549	0.18	7	8.481	0.5	40	d	0.542328
			236713_at	0	3.485	0.32	7	3.278	0.35	40	d	0.162137
CNN3	5	calponin 3, acidic	228297_at	5	10.64	0.23	7	10.07	1.07	40	d	0.005507
			201445_at	3	10.16	0.32	7	8.82	1.11	40	d	0.000001
RAPGEFL1	5	Rap guanine nucleotide exchange factor (GEF)-like 1	218657_at	8	4.927	1.01	7	4.852	1.37	40	d	0.893688
NUPL1	5	nucleoporin like 1	225047_at	5	4.999	0.58	7	5.428	0.71	40	u	0.146199
			204435_at	0	6.723	0.49	7	7.239	0.92	40	u	0.16166
			241425_at	0	7.459	0.66	7	6.804	0.92	40	d	0.084148
			223984_s_at	0	4.713	0.5	7	5.68	0.83	40	u	0.005432
MYT1	5	myelin transcription factor 1	210341_at	5	3.421	0.08	7	3.701	0.88	40	u	0.058497
			215822_x_at	1	4.358	0.34	7	4.45	0.55	40	u	0.67711
			1556269_at	0	2.749	0.05	7	2.947	0.46	40	u	0.014077
GOLGA1	5	golgi autoantigen, golgin subfamily a, 1	203384_s_at	5	6.551	0.28	7	5.658	0.96	40	d	0.000053
			203383_s_at	5	6.678	0.19	7	6.795	0.53	40	u	0.31332
			214997_at	0	3.222	0.18	7	3.532	0.41	40	u	0.060082
MEA1	5	male-enhanced antigen 1	218061_at	8	10.1	0.15	7	10.49	0.68	40	u	0.003023
VAPB	5	VAMP (vesicle-associated membrane protein)-associated protein B and C	202550_s_at	5	8.348	0.32	7	9.043	0.64	40	u	0.008509
			202549_at	5	2.858	0.45	7	3.594	0.89	40	u	0.042553
LMBRD2	5	LMBR1 domain containing 2	232893_at	4	2.469	0.06	7	3.18	0.83	40	u	0.000005
CADPS	5	Ca2+-dependent secretion activator	1568604_a_at	5	2.244	0.11	7	2.366	0.2	40	u	0.127381
			204814_at	5	1.971	0.03	7	2.027	0.06	40	u	0.030639
			1568603_at	5	2.881	0.12	7	3.119	0.27	40	u	0.027142
			233950_at	0	2.175	0.04	7	2.249	0.1	40	u	0.071728
			234220_at	0	2.456	0.08	7	2.495	0.2	40	u	0.613996
CPT1B	5	carnitine palmitoyltransferase 1B (muscle)	no probeset available									
USP25	5	ubiquitin specific peptidase 25	223167_s_at	5	5.673	0.75	7	5.244	0.75	40	d	0.18051
			220419_s_at	2	9.015	0.33	7	8.577	0.89	40	d	0.212363
			1563497_at	0	6.312	0.33	7	6.07	1.07	40	d	0.275902
			1555559_s_at	0	3.536	0.44	7	4.429	0.98	40	u	0.024461
			1555558_at	0	2.903	0.09	7	3.118	0.2	40	u	0.00909
PCTK1	5	PCTAIRE protein kinase 1	208823_s_at	5	7.379	0.19	7	7.81	0.71	40	u	0.003476
			208824_x_at	1	7.337	0.27	7	8.289	0.91	40	u	0.000011
			207239_s_at	0	6.749	0.21	7	7.673	1.05	40	u	0.000013
GAMT	5	guanidinoacetate N-methyltransferase	1552473_at	0	3.103	0.4	7	3.287	0.56	40	u	0.4248
			205354_at	0	6.859	0.57	7	5.981	1.29	40	d	0.090402
			1552474_a_at	0	7.614	0.4	7	6.504	1.39	40	d	0.000325
TPM1	5	tropomyosin 1 (alpha)	210986_s_at	5	12.72	0.31	7	11.52	0.88	40	d	0.000002
			210987_x_at	4	12.65	0.44	7	11.01	0.99	40	d	0.000123
			1558532_at	0	2.02	0.25	7	2.075	0.28	40	u	0.636042
			206116_s_at	0	11.16	0.52	7	9.456	1.32	40	d	0.001935
			238688_at	0	10.01	0.51	7	7.693	1.29	40	d	0.000036
			206117_at	0	7.372	0.61	7	6.051	0.99	40	d	0.001671
TULP4	5	tubby like protein 4	218184_at	5	8.029	0.32	7	7.975	0.9	40	d	0.782304
			239742_at	0	7.196	0.5	7	8.462	1.07	40	u	0.004173
			224170_s_at	0	2.879	0.25	7	3.134	0.37	40	u	0.094016
PLEKHA1	5	pleckstrin homology domain containing, family A (phosphoinositide binding specific)	226247_at	5	8.548	0.3	7	8.899	0.59	40	u	0.139559
			219024_at	1	7.109	0.36	7	7.82	0.73	40	u	0.017163
USP2	5	ubiquitin specific peptidase 2	230965_at	5	3.736	0.25	7	3.593	0.35	40	d	0.315317
			229337_at	5	3.406	0.36	7	3.059	0.41	40	d	0.046829
			207213_s_at	0	1.83	0.02	7	2.058	0.57	40	u	0.017019
			207211_at	0	1.817	0.04	7	1.879	0.12	40	u	0.027626
CCNYL1	5	cyclin Y-like 1	227280_s_at	5	5.771	0.43	7	6.367	0.73	40	u	0.045572
			228810_at	5	4.648	0.49	7	5.44	0.91	40	u	0.033652
KIAA2026	5	KIAA2026	228446_at	5	8.351	0.36	7	7.622	0.78	40	d	0.022091
			238490_at	0	6.435	0.44	7	5.626	1.07	40	d	0.05909
PTPRT	5	protein tyrosine phosphatase, receptor type, T	205948_at	5	4.451	0.46	7	2.958	1.06	40	d	0.000845
TRIM9	5	tripartite motif-containing 9	209859_at	5	3.959	0.62	7	3.671	0.63	40	d	0.280932
			230280_at	0	2.577	0.15	7	2.584	0.17	40	u	0.920213

CXXC5	5	CXXC finger 5	233955_x_at	5	10.12	0.66	7	9.25	1.53	40	d	0.154729
			224516_s_at	5	9.959	0.64	7	8.969	1.54	40	d	0.108239
			222996_s_at	3	9.938	0.65	7	9.078	1.49	40	d	0.149637
TFRC	5	transferrin receptor (p90, CD71)	207332_s_at	5	9.763	0.29	7	10.84	1.21	40	u	0.000029
			208691_at	5	10.33	0.22	7	11.67	1.03	40	u	0
			237215_s_at	0	3.495	0.41	7	5.728	2	40	u	0
			240686_x_at	0	6.587	0.1	7	6.098	0.53	40	d	0.000005
			237214_at	0	2.501	0.1	7	2.608	0.22	40	u	0.214126
OSBPL6	5	oxysterol binding protein-like 6	238575_at	5	2.47	0.1	7	3.052	1.12	40	u	0.002923
			223805_at	2	5.715	0.81	7	5.728	1.36	40	u	0.980318
BCOR	5	BCL6 co-repressor	219433_at	5	5.007	0.79	7	4.937	0.93	40	d	0.854469
			223566_s_at	4	5.784	0.69	7	5.517	1.22	40	d	0.584645
			223915_at	0	5.768	0.49	7	5.527	1.08	40	d	0.574711
			223916_s_at	0	3.498	0.43	7	3.777	0.64	40	u	0.283827
ARNTL	5	aryl hydrocarbon receptor nuclear translocator-like	209824_s_at	5	6.914	0.37	7	6.26	0.56	40	d	0.005311
			210971_s_at	0	4.484	0.7	7	4.479	1.05	40	d	0.99086
RSBN1L	5	round spermatid basic protein 1-like	226387_at	5	7.259	0.35	7	7.403	0.62	40	u	0.556451
			232221_x_at	0	7.083	0.16	7	6.641	0.56	40	d	0.000429
ICK	5	intestinal cell (MAK-like) kinase	204569_at	5	5.811	0.45	7	6.099	0.56	40	u	0.212259
			1552837_at	0	2.713	0.08	7	2.849	0.25	40	u	0.013963
BTF3L4	5	basic transcription factor 3-like 4	225976_at	5	8.113	0.29	7	8.337	0.53	40	u	0.291937
			226963_at	3	8.994	0.23	7	9.597	0.6	40	u	0.01402
GZF1	5	GDNF-inducible zinc finger protein 1	225884_s_at	5	6.801	0.24	7	6.839	0.86	40	u	0.823808
			234055_s_at	5	3.515	0.34	7	3.69	0.62	40	u	0.478505
DNM3	5	dynamin 3	209839_at	5	2.401	0.3	7	3.097	1.46	40	u	0.011592
			1558501_at	0	2.725	0.24	7	3.206	0.79	40	u	0.005008
			1558502_s_at	0	3.022	0.25	7	3.291	0.65	40	u	0.292834
RPIA	5	ribose 5-phosphate isomerase A (ribose 5-phosphate epimerase)	212973_at	5	8.134	0.18	7	8.301	0.78	40	u	0.26058
RARA	5	retinoic acid receptor, alpha	203749_s_at	5	7.816	0.55	7	7.215	1.27	40	d	0.235707
			203750_s_at	5	6.716	0.38	7	6.19	0.77	40	d	0.09105
			216300_x_at	2	6.789	0.41	7	6.488	0.89	40	d	0.39261
			1565358_at	0	7.669	0.66	7	8.449	1.83	40	u	0.061848
			211605_s_at	0	4.904	0.95	7	4.82	1.13	40	d	0.855873
CARM1	5	coactivator-associated arginine methyltransferase 1	212512_s_at	8	6.442	0.33	7	7.241	0.94	40	u	0.000576
CBFB	5	core-binding factor, beta subunit	202370_s_at	5	9.749	0.21	7	9.789	0.56	40	u	0.856883
			206788_s_at	0	6.737	0.6	7	7.252	1.07	40	u	0.231829
SOCS7	5	suppressor of cytokine signaling 7	214015_at	0	2.258	0.08	7	2.651	0.57	40	u	0.000204
			226572_at	0	7.229	0.21	7	7.157	1.03	40	d	0.699174
KRIT1	5	KRIT1, ankyrin repeat containing	229785_at	2	5.414	0.23	7	5.394	0.63	40	d	0.883498
			216713_at	2	6.458	0.18	7	7.004	0.7	40	u	0.000246
			204738_s_at	0	5.977	0.14	7	6.141	0.39	40	u	0.065425
			34031_i_at	0	8.222	0.32	7	8.169	0.68	40	d	0.844164
SBF2	5	SET binding factor 2	226169_at	5	8.056	0.22	7	7.191	0.61	40	d	0.000001
			233914_s_at	0	6.482	0.59	7	5.559	0.69	40	d	0.002185
			242935_at	0	3.213	0.36	7	3.235	0.48	40	u	0.908918
CAPRIN1	5	cell cycle associated protein 1	200722_s_at	5	9.392	0.41	7	10.08	1.4	40	u	0.018659
			226990_at	5	7.328	0.34	7	7.389	1.1	40	u	0.789945
			243370_at	5	3.964	0.57	7	3.886	1.13	40	d	0.861619
			225340_s_at	5	10.52	0.19	7	10.95	0.75	40	u	0.004985
			200723_s_at	4	10.91	0.28	7	11.12	1.12	40	u	0.342776
			226289_at	0	5.322	0.42	7	6.06	1.15	40	u	0.007622
			226285_at	0	6.802	0.54	7	7.587	1.06	40	u	0.066059
PEA15	5	phosphoprotein enriched in astrocytes 15	200788_s_at	5	11.11	0.21	7	10.82	0.55	40	d	0.180922
			200787_s_at	1	9.434	0.37	7	9.572	1.13	40	u	0.562742
SNAP23	5	synaptosomal-associated protein, 23kDa	209130_at	5	9.694	0.15	7	9.502	0.65	40	d	0.12113
			209131_s_at	0	5.716	0.72	7	5.579	1.27	40	d	0.788237
			214544_s_at	0	6.818	0.47	7	6.573	1.36	40	d	0.406821
SPTLC2	5	serine palmitoyltransferase, long chain base subunit 2	203128_at	5	7.467	0.34	7	7.259	1.11	40	d	0.366281
			203127_s_at	2	7.244	0.5	7	7.617	0.96	40	u	0.329232
			225095_at	0	8.274	0.41	7	7.549	1.15	40	d	0.007599
			216203_at	0	2.85	0.4	7	2.983	0.49	40	u	0.510946
			216202_s_at	0	6.533	0.49	7	7.06	1.46	40	u	0.09908

FUSIP1	5	FUS interacting protein (serine/arginine-rich) 1	206095_s_at	0	9.731	0.16	7	9.752	0.55	40	u	0.855603
			204299_at	0	6.887	0.39	7	7.395	0.69	40	u	0.06823
			210178_x_at	0	8.19	0.34	7	8.262	1.02	40	u	0.741143
			213594_x_at	0	9.109	0.31	7	9.098	0.82	40	d	0.97194
PLEKHM1	5	pleckstrin homology domain containing, family M (with RUN domain) member 1	212717_at	5	7.602	0.39	7	7.007	0.38	40	d	0.00054
			212700_x_at	5	5.653	0.45	7	5.542	0.44	40	d	0.551834
			216200_at	0	3.538	0.56	7	3.356	0.35	40	d	0.266976
SPIRE1	5	spire homolog 1 (Drosophila)	225018_at	5	6.81	0.27	7	7.222	1.17	40	u	0.065954
			1554807_a_at	5	5.743	0.4	7	6.31	1.33	40	u	0.043133
			224995_at	5	8.317	0.22	7	8.206	1.13	40	d	0.584242
			1559517_a_at	1	3.403	0.34	7	4.331	1.2	40	u	0.000481
KHDRBS3	5	KH domain containing, RNA binding, signal transduction associated 3	209781_s_at	0	5.969	0.25	7	7.07	1.53	40	u	0.000149
ANKRD50	5	ankyrin repeat domain 50	225731_at	4	8.289	0.4	7	8.613	1.03	40	u	0.426591
			225735_at	4	5.828	0.26	7	6.599	1.08	40	u	0.000488
			236189_at	0	2.969	0.39	7	3.249	0.54	40	u	0.202515
SMOC1	5	SPARC related modular calcium binding 1	222783_s_at	5	1.993	0.02	7	2.35	0.81	40	u	0.00913
			222784_at	5	5.349	0.45	7	5.429	0.83	40	u	0.807608
NEUROG2	5	neurogenin 2	215632_at	0	2.607	0.03	7	2.718	0.38	40	u	0.081361
RYR2	5	ryanodine receptor 2 (cardiac)	214044_at	5	2.92	0.18	7	3.047	0.43	40	u	0.459786
			207557_s_at	5	2.72	0.39	7	2.714	0.47	40	d	0.976824
NCKIPSD	5	NCK interacting protein with SH3 domain	218697_at	5	7.491	0.33	7	6.667	0.89	40	d	0.022454
			216114_at	0	3.394	0.56	7	3.443	0.57	40	u	0.8351
			234806_at	0	2.08	0.12	7	2.109	0.11	40	u	0.52462
			216116_at	0	1.935	0.03	7	1.968	0.09	40	u	0.105301
			233165_at	0	3.502	0.36	7	3.505	0.31	40	u	0.977469
			234855_at	0	4.663	0.45	7	4.845	0.47	40	u	0.356002
KLHDC5	5	kelch domain containing 5	225732_at	5	7.989	0.25	7	7.378	0.86	40	d	0.001216
			225963_at	3	6.635	0.26	7	6.864	0.69	40	u	0.401309
			225961_at	3	7.528	0.4	7	7.18	1	40	d	0.378428
TSPAN9	5	tetraspanin 9	220968_s_at	5	8.265	0.26	7	7.799	0.75	40	d	0.00764
			205665_at	3	3.83	0.31	7	4.157	0.87	40	u	0.094964
			233435_at	0	5.602	0.46	7	4.924	1.05	40	d	0.106418
RAB30	5	RAB30, member RAS oncogene family	228003_at	5	6.975	0.58	7	6.492	1.18	40	d	0.305548
			227842_at	5	4.382	0.49	7	3.956	0.97	40	d	0.271308
			206530_at	0	2.789	0.15	7	3.11	0.99	40	u	0.066761
OSBPL11	5	oxysterol binding protein-like 11	218304_s_at	5	6.946	0.44	7	7.523	0.77	40	u	0.065036
			222586_s_at	5	6.133	0.43	7	6.883	0.76	40	u	0.01664
			229527_s_at	0	2.812	0.06	7	2.931	0.21	40	u	0.007352
GGNBP2	5	gametogenetin binding protein 2	218079_s_at	5	9.039	0.29	7	8.865	0.45	40	d	0.343184
			1554419_x_at	0	3.273	0.22	7	3.398	0.12	40	u	0.225449
			233936_s_at	0	8.773	0.2	7	8.68	0.51	40	d	0.641316
			233937_at	0	4.018	0.57	7	3.926	0.66	40	d	0.736641
TMEM30A	5	transmembrane protein 30A	217743_s_at	5	9.167	0.35	7	9.364	0.7	40	u	0.480788
			222391_at	5	9.779	0.19	7	9.368	0.64	40	d	0.003392
			232591_s_at	2	7.349	0.81	7	8.008	1.72	40	u	0.336467
PHLPP	5	PH domain and leucine rich repeat protein phosphatase	212719_at	5	7.113	0.21	7	5.873	1.47	40	d	0.000012
CUL4A	5	cullin 4A	201424_s_at	5	7.288	0.32	7	7.347	0.67	40	u	0.825731
			201423_s_at	5	8.907	0.22	7	8.758	0.57	40	d	0.508128
			232466_at	0	5.186	0.9	7	4.968	0.9	40	d	0.563688
			242194_at	0	5.229	0.33	7	5.129	0.5	40	d	0.619929
			227757_at	0	5.097	0.49	7	5.639	1.06	40	u	0.199206
SCN8A	5	sodium channel, voltage gated, type VIII, alpha subunit	207049_at	5	1.868	0.02	7	1.918	0.11	40	u	0.008994
			1561820_at	0	3.348	0.41	7	3.582	0.47	40	u	0.236497
RGMA	5	RGM domain family, member A	234406_at	5	2.019	0.02	7	2.057	0.09	40	u	0.034092
			223468_s_at	5	6.905	0.36	7	5.414	2.41	40	d	0.000772
JARID1C	5	jumonji, AT rich interactive domain 1C	202383_at	5	8.052	0.24	7	8.104	0.53	40	u	0.804796
			239207_at	0	5.802	0.61	7	5.026	0.85	40	d	0.027924
L3MBTL3	5	l(3)mbt-like 3 (Drosophila)	229393_at	5	5.796	0.39	7	5.318	0.83	40	d	0.150864
CGN	5	cingulin	223233_s_at	5	7.431	0.6	7	6.778	1.48	40	d	0.26721
			223232_s_at	5	4.917	1.02	7	5.262	1.68	40	u	0.608151
HIATL1	5	hippocampus abundant transcript-like 1	223073_at	5	8.656	0.21	7	9.024	0.67	40	u	0.012227
			233954_at	0	2.388	0.03	7	2.544	0.25	40	u	0.000448

ZNF282	5	zinc finger protein 282	212892_at	5	7.108	0.19	7	6.943	0.68	40	d	0.230201
RNF13	5	ring finger protein 13	201780_s_at	5	9.821	0.29	7	9.449	0.62	40	d	0.136704
			201779_s_at	0	10.61	0.35	7	9.898	0.6	40	d	0.004269
HMGA1	5	high mobility group AT-hook 1	206074_s_at	5	7.902	0.47	7	9.595	1.05	40	u	0.000171
			210457_x_at	1	6.235	0.29	7	6.566	1.12	40	u	0.132937
LGR4	5	leucine-rich repeat-containing G protein-coupled receptor 4	222596_s_at	5	3.741	0.4	7	3.497	0.47	40	d	0.208571
			218326_s_at	5	6.857	0.53	7	6.118	1	40	d	0.068742
			230674_at	0	2.81	0.1	7	2.874	0.28	40	u	0.297806
SCN5A	5	sodium channel, voltage-gated, type V, alpha subunit	207413_s_at	5	2.591	0.53	7	2.537	0.44	40	d	0.778203
PDGFC	5	platelet derived growth factor C	218718_at	5	9.827	0.29	7	8.313	1.1	40	d	0
			222719_s_at	0	7.124	0.35	7	5.022	1.67	40	d	0
ANXA11	5	annexin A11	214783_s_at	5	7.859	0.37	7	6.886	0.66	40	d	0.000542
			206200_s_at	0	9.721	0.23	7	8.856	0.6	40	d	0.000581
OSBPL5	5	oxysterol binding protein-like 5	223464_at	5	6.202	0.38	7	5.242	0.78	40	d	0.003287
			233734_s_at	4	7.887	0.44	7	7.27	0.66	40	d	0.025251
ELMO2	4	engulfment and cell motility 2	220363_s_at	4	6.529	0.27	7	6.537	0.89	40	u	0.964844
			55692_at	4	7.129	0.24	7	7.164	0.68	40	u	0.813289
			221528_s_at	4	7.827	0.26	7	7.744	0.81	40	d	0.627562
			1554857_at	0	2.333	0.05	7	2.394	0.08	40	u	0.071687
C15orf27	4	chromosome 15 open reading frame 27	1552400_a_at	4	2.281	0.14	7	2.37	0.24	40	u	0.365181
CADM4	4	cell adhesion molecule 4	229335_at	4	6.925	0.26	7	6.848	0.5	40	d	0.695769
			214267_s_at	0	3.546	0.22	7	3.62	0.24	40	u	0.457004
			215258_at	0	3.333	0.88	7	3.498	0.71	40	u	0.595411
			215259_s_at	0	2.525	0.33	7	2.469	0.32	40	d	0.683243
			222293_at	0	5.12	0.48	7	5.01	0.56	40	d	0.632257
DDX5	4	DEAD (Asp-Glu-Ala-Asp) box polypeptide 5	200033_at	4	13.33	0.25	7	12.93	0.66	40	d	0.128613
ZEB1	4	zinc finger E-box binding homeobox 1	210875_s_at	4	4.117	0.54	7	4.127	0.75	40	u	0.974565
			212758_s_at	0	6.294	0.33	7	6.308	0.72	40	u	0.960711
EPN2	4	epsin 2	203463_s_at	4	5.57	0.32	7	5.741	0.95	40	u	0.403021
			203464_s_at	4	7.449	0.15	7	6.784	0.79	40	d	0.000022
MET	4	met proto-oncogene (hepatocyte growth factor receptor)	203510_at	4	7.856	0.55	7	6.024	1.75	40	d	0.000021
			213807_x_at	2	4.07	0.66	7	3.654	1.02	40	d	0.314207
			213816_s_at	2	2.208	0.26	7	2.682	1.16	40	u	0.032476
			211599_x_at	1	5.348	0.66	7	4.506	1.27	40	d	0.099801
TNS3	4	tensin 3	217853_at	4	8.968	0.26	7	8.349	0.88	40	d	0.001535
			234257_at	0	2.488	0.42	7	2.463	0.29	40	d	0.849657
SCYL3	4	SCY1-like 3 (S. cerevisiae)	205607_s_at	4	7.684	0.51	7	7.836	0.76	40	u	0.619794
			41329_at	4	7.463	0.44	7	7.727	0.7	40	u	0.354025
GABPAP	4	g protein transcription factor, alpha subunit ps	no probeset available									
TAOK3	4	TAO kinase 3	221508_at	4	6.713	0.3	7	6.688	0.45	40	d	0.890794
			220761_s_at	0	8.728	0.2	7	8.506	0.68	40	d	0.109851
ITGA3	4	integrin, alpha 3 (antigen CD49C, alpha 3 subunit of VLA-3 receptor)	201474_s_at	4	7.216	0.48	7	5.499	1.08	40	d	0.0002
KIAA1033	4	KIAA1033	212794_s_at	4	9.142	0.23	7	9.202	0.55	40	u	0.782353
			212795_at	4	9.254	0.28	7	9.123	0.57	40	d	0.567789
			215936_s_at	0	8.059	0.29	7	7.877	1.12	40	d	0.402091
RUNX3	4	runt-related transcription factor 3	204198_s_at	4	7.139	0.54	7	6.615	1.35	40	d	0.327604
			204197_s_at	4	6.123	0.56	7	5.771	1.25	40	d	0.476623
			234928_x_at	0	5.893	0.3	7	5.631	0.28	40	d	0.034206
BAZ1B	4	bromodomain adjacent to zinc finger domain, 1B	208445_s_at	1	8.59	0.2	7	8.66	0.65	40	u	0.599551
			213336_at	1	6.422	0.32	7	6.931	0.71	40	u	0.076653
			211313_s_at	0	4.534	0.52	7	4.612	0.63	40	u	0.763559
			229658_at	0	2.273	0.03	7	2.349	0.16	40	u	0.008937
PPP1R15B	4	protein phosphatase 1, regulatory (inhibitor) subunit 15B	224692_at	4	9.209	0.31	7	9.302	0.57	40	u	0.683524
COL1A1	4	collagen, type I, alpha 1	1556499_s_at	4	11.63	0.97	7	12.38	1.23	40	u	0.140526
			202312_s_at	4	4.76	0.46	7	4.381	0.42	40	d	0.037415
			202310_s_at	4	11.29	1.01	7	11.71	1.61	40	u	0.520279
			202311_s_at	4	6.666	1.18	7	8.399	1.6	40	u	0.010186
			217430_x_at	0	8.397	0.71	7	9.101	1.55	40	u	0.253916
C10orf22	4	chromosome 10 open reading frame 22	212500_at	4	7.011	0.25	7	6.594	0.67	40	d	0.118081
			212502_at	4	8.081	0.25	7	8.259	0.5	40	u	0.373094

			229409_s_at	0	2.132	0.07	7	2.099	0.06	40	d	0.237446
ZDHHC6	4	zinc finger, DHHC-type containing 6	218249_at	4	9.077	0.21	7	8.81	0.53	40	d	0.204018
ALX1	4	ALX homeobox 1	no probeset available									
ING5	4	inhibitor of growth family, member 5	228287_at	4	7.063	0.46	7	6.298	0.77	40	d	0.016986
			228672_at	3	2.762	0.06	7	2.856	0.15	40	u	0.112715
			229197_at	1	2.783	0.44	7	2.764	0.65	40	d	0.940555
PPP1R3D	4	protein phosphatase 1, regulatory (inhibitor) subunit 3D	204554_at	4	5.953	0.33	7	6.567	0.74	40	u	0.041503
			204555_s_at	0	4.646	0.4	7	5.472	0.77	40	u	0.009105
EIF1	4	eukaryotic translation initiation factor 1	212130_x_at	4	12.58	0.37	7	11.73	0.61	40	d	0.001092
			202021_x_at	4	12.66	0.36	7	11.8	0.63	40	d	0.001335
			212225_at	4	7.391	0.6	7	5.889	0.68	40	d	0.000003
			212227_x_at	4	12.68	0.34	7	11.81	0.64	40	d	0.001321
			211956_s_at	3	13.69	0.17	7	13.35	0.38	40	d	0.025583
			228967_at	0	5.896	0.27	7	5.184	0.54	40	d	0.001733
GCC2	4	GRIP and coiled-coil domain containing 2	202832_at	4	8.736	0.27	7	8.247	0.67	40	d	0.067041
GAS2L1	4	growth arrest-specific 2 like 1	31874_at	4	8.346	0.32	7	7.747	1.01	40	d	0.007536
			209729_at	4	7.802	0.38	7	6.997	0.88	40	d	0.024404
			208258_s_at	0	5.202	0.48	7	4.923	0.59	40	d	0.253273
WBP4	4	WW domain binding protein 4 (formin binding protein 21)	203597_s_at	4	5.82	0.32	7	5.602	0.67	40	d	0.414333
			203599_s_at	4	7.139	0.44	7	6.454	0.71	40	d	0.02018
			203598_s_at	4	6.19	0.38	7	6.278	0.67	40	u	0.745713
HS2ST1	4	heparan sulfate 2-O-sulfotransferase 1	203284_s_at	4	7.235	0.26	7	7.821	0.63	40	u	0.0225
			203285_s_at	4	6.351	0.33	7	6.068	0.71	40	d	0.319096
			203283_s_at	4	4.654	0.32	7	5.299	0.78	40	u	0.040687
			230465_at	0	2.507	0.13	7	2.607	0.14	40	u	0.084037
PLCL2	4	phospholipase C-like 2	213309_at	4	6.916	0.52	7	5.923	1.06	40	d	0.021645
			216218_s_at	0	4.28	0.79	7	4.202	1.04	40	d	0.853738
			216217_at	0	3.844	0.24	7	4.297	0.64	40	u	0.075803
ERBB2IP	4	erbb2 interacting protein	222473_s_at	4	8.345	0.28	7	8.422	0.85	40	u	0.671042
			217941_s_at	4	9.637	0.33	7	9.601	0.59	40	d	0.878849
			232896_at	0	3.076	0.23	7	3.352	0.39	40	u	0.084597
EMX2	4	empty spiracles homeobox 2	221950_at	4	2.782	0.41	7	2.586	0.54	40	d	0.372803
SNX6	4	sorting nexin 6	217789_at	4	8.569	0.15	7	8.164	0.76	40	d	0.004973
			222410_s_at	4	9.755	0.28	7	9.924	0.72	40	u	0.550234
RAG1	4	recombination activating gene 1	206591_at	4	3.083	0.1	7	3.3	0.43	40	u	0.010543
			1554994_at	0	2.465	0.06	7	2.658	0.34	40	u	0.002352
IRS1	4	insulin receptor substrate 1	204686_at	0	9.636	0.43	7	7.745	1.54	40	d	0.000001
LRRC8A	4	leucine rich repeat containing 8 family, member A	224624_at	4	8.716	0.2	7	8.273	0.43	40	d	0.012866
			233487_s_at	1	6.828	0.3	7	6.395	0.5	40	d	0.035754
GRIK2	4	glutamate receptor, ionotropic, kainate 2	213845_at	4	2.712	0.2	7	2.76	0.37	40	u	0.741304
			1560142_at	0	2.507	0.04	7	2.705	0.25	40	u	0.000023
			1560265_at	0	3.229	0.16	7	3.651	0.35	40	u	0.003336
			1555375_at	0	2.811	0.11	7	2.919	0.23	40	u	0.234426
			215655_at	0	2.608	0.13	7	2.671	0.17	40	u	0.366197
			1563754_at	0	2.822	0.09	7	3.146	0.39	40	u	0.000057
C17orf85	4	chromosome 17 open reading frame 85	218896_s_at	4	6.038	0.35	7	5.752	0.78	40	d	0.357215
			222779_s_at	4	6.669	0.27	7	6.436	0.67	40	d	0.379222
COL7A1	4	collagen, type VII, alpha 1 (epidermolysis bullosa, dystrophic, dominant and recessive)	204136_at	4	7.6	0.39	7	6.691	0.64	40	d	0.000849
			217312_s_at	0	5.947	0.56	7	4.796	0.81	40	d	0.000913
SPG20	4	spastic paraplegia 20 (Troyer syndrome)	212526_at	4	8.673	0.32	7	7.898	1.07	40	d	0.001179
			236600_at	4	5.19	0.77	7	4.023	0.95	40	d	0.004308
			216965_x_at	0	2.844	0.28	7	3.041	0.41	40	u	0.235254
CHMP7	4	CHMP family, member 7	212313_at	4	8.255	0.11	7	7.852	0.63	40	d	0.000652
NFYA	4	nuclear transcription factor Y, alpha	204108_at	4	7.504	0.36	7	8.191	0.87	40	u	0.049937
			204107_at	4	5.465	0.3	7	6.368	1.33	40	u	0.000716
			204109_s_at	1	5.466	0.45	7	6.578	1.42	40	u	0.000721
			215720_s_at	0	4.822	0.63	7	5.543	1.26	40	u	0.155513
CHMP2B	4	chromatin modifying protein 2B	202538_s_at	4	8.521	0.43	7	8.79	0.79	40	u	0.391526
			202536_at	4	8.572	0.28	7	8.752	0.9	40	u	0.334894
			202537_s_at	0	8.24	0.38	7	8.722	0.87	40	u	0.16388
NFYC	4	nuclear transcription factor Y, gamma	202215_s_at	4	8.195	0.35	7	7.504	0.53	40	d	0.002126
			1559218_s_at	3	3.739	0.46	7	3.253	0.66	40	d	0.075976
			202216_x_at	3	7.693	0.24	7	7.199	0.68	40	d	0.002851
			211251_x_at	3	7.836	0.34	7	7.219	0.78	40	d	0.048811

			238231_at	0	3.375	0.44	7	3.176	0.49	40	d	0.332203
			1558782_a_at	0	2.329	0.36	7	2.637	0.36	40	u	0.047451
			211797_s_at	0	7.765	0.28	7	7.004	0.75	40	d	0.000122
RNF182	4	ring finger protein 182	230720_at	4	3.142	0.3	7	4.374	1.83	40	u	0.000343
PCDHGB4	4	protocadherin gamma subfamily B, 4	no probeset available									
KIAA0664	4	KIAA0664	212456_at	4	7.483	0.3	7	6.731	1.21	40	d	0.002188
			243258_at	0	5.335	0.21	7	5.54	0.39	40	u	0.189001
RDX	4	radixin	212398_at	4	6.896	0.28	7	7.124	1.24	40	u	0.325377
			212397_at	4	9.361	0.18	7	9.073	0.87	40	d	0.072887
			204969_s_at	3	5.08	0.45	7	5.78	1.25	40	u	0.016791
ADCY3	4	adenylate cyclase 3	209320_at	5	6.645	0.24	7	6.201	0.62	40	d	0.075445
			209321_s_at	5	7.41	0.6	7	7.279	1.12	40	d	0.770617
R3HDM2	4	R3H domain containing 2	203831_at	4	8.256	0.29	7	7.766	0.77	40	d	0.111709
PAQR3	4	progestin and adipoQ receptor family member III	213372_at	4	5.687	0.53	7	5.953	1.03	40	u	0.516303
			1557576_at	0	2.512	0.05	7	2.672	0.26	40	u	0.000931
TRAM2	4	translocation associated membrane protein 2	202368_s_at	4	6.828	0.32	7	6.548	0.86	40	d	0.411764
			202369_s_at	4	7.601	0.31	7	7.16	0.66	40	d	0.095049
			1554383_a_at	0	3.465	0.23	7	3.786	0.55	40	u	0.143896
RER1	4	RER1 retention in endoplasmic reticulum 1 homolog (S. cerevisiae)	202297_s_at	4	9.07	0.23	7	9.245	0.49	40	u	0.370135
			202296_s_at	0	9.743	0.28	7	9.851	0.7	40	u	0.698083
			213296_at	0	8.195	0.11	7	7.265	0.75	40	d	0
CSMD3	4	CUB and Sushi multiple domains 3	240228_at	4	3.128	0.33	7	3.265	0.43	40	u	0.434325
TMEM189	4	transmembrane protein 189	201003_x_at	6	8.975	0.29	7	8.701	0.74	40	d	0.349506
			223186_at	4	6.651	0.27	7	7.113	0.62	40	u	0.064314
NTN4	4	netrin 4	223315_at	4	10.21	0.57	7	6.808	2	40	d	0
PCDHGA10	4	protocadherin gamma subfamily A, 10	211875_x_at	0	2.699	0.05	7	3.059	0.75	40	u	0.005336
			211876_x_at	0	3.677	0.42	7	3.461	1.05	40	d	0.601847
RAB15	4	RAB15, member RAS onocogene family	221810_at	4	7.242	0.26	7	7.12	0.97	40	d	0.519739
			59697_at	4	7.585	0.19	7	7.463	1.05	40	d	0.512258
ATP6V0A2	4	ATPase, H+ transporting, lysosomal V0 subunit a2	235255_at	4	2.576	0.12	7	2.655	0.32	40	u	0.269329
			205704_s_at	2	4.629	0.62	7	5.564	0.85	40	u	0.009412
			217603_at	0	2.534	0.15	7	2.698	0.33	40	u	0.211936
			1555114_at	0	2.793	0.07	7	2.994	0.31	40	u	0.001081
RAB11FIP4	4	RAB11 family interacting protein 4 (class II)	224482_s_at	4	4.089	0.64	7	4.095	1.19	40	u	0.990241
			225739_at	0	5.055	0.4	7	5.455	0.74	40	u	0.180513
			225746_at	0	3.068	0.4	7	3.515	0.68	40	u	0.103281
APOBEC3A	4	apolipoprotein B mRNA editing enzyme, catalytic polypeptide-like 3A	210873_x_at	0	3.251	0.42	7	4.232	1.35	40	u	0.001434
TRIM63	4	tripartite motif-containing 63	236972_at	4	3.875	0.52	7	3.293	0.21	40	d	0.035511
PCDHGA5	4	protocadherin gamma subfamily A, 5	no probeset available									
NOTCH2	4	Notch homolog 2 (Drosophila)	202443_x_at	4	10.6	0.29	7	9.92	0.63	40	d	0.008565
			202445_s_at	4	5.094	0.68	7	4.599	0.66	40	d	0.081501
			212377_s_at	4	10.16	0.29	7	9.543	0.66	40	d	0.021969
			210756_s_at	0	8.934	0.38	7	7.938	1.52	40	d	0.001435
MAPK7	4	mitogen-activated protein kinase 7	207292_s_at	4	6.399	0.29	7	5.922	0.48	40	d	0.01534
			35617_at	4	5.201	0.28	7	4.807	0.67	40	d	0.140132
MAB21L2	4	mab-21-like 2 (C. elegans)	210303_at	4	3.154	0.25	7	3.322	0.27	40	u	0.134645
			210302_s_at	4	2.161	0.12	7	2.37	0.29	40	u	0.075335
TRHDE	4	thyrotropin-releasing hormone degrading enzyme	219937_at	4	2.894	0.29	7	3.083	0.38	40	u	0.221781
			1560697_at	0	2.53	0.12	7	2.583	0.16	40	u	0.418836
			1560698_a_at	0	3.145	0.32	7	2.998	0.27	40	d	0.214793
GRIN2A	4	glutamate receptor, ionotropic, N-methyl D-aspartate 2A	206534_at	4	2.5	0.05	7	2.527	0.21	40	u	0.488848
VPS37B	4	vacuolar protein sorting 37 homolog B (S. cerevisiae)	221704_s_at	4	8.362	0.42	7	7.495	0.99	40	d	0.030827
PCDHGA4	4	protocadherin gamma subfamily A, 4	1552735_at	0	6.379	0.59	7	6.075	1.32	40	d	0.559191
CBLN2	4	cerebellin 2 precursor	242301_at	4	2.455	0.07	7	3.359	1.77	40	u	0.00287
CA12	4	carbonic anhydrase XII	214164_x_at	4	9.905	0.76	7	7.579	2.68	40	d	0.000114
			203963_at	1	9.984	0.58	7	7.172	3.09	40	d	0.000007
			204508_s_at	1	8.379	0.77	7	5.896	2.94	40	d	0.000103
			210735_s_at	1	9.166	0.75	7	6.959	2.68	40	d	0.000213
			204509_at	0	4.124	0.38	7	4.103	0.63	40	d	0.935419
			215867_x_at	0	9.929	0.76	7	7.502	2.8	40	d	0.000087
			212684_at	4	7.192	0.64	7	7.736	0.81	40	u	0.105507

ZNF3	4	zinc finger protein 3	219605_at	0	2.924	0.4	7	3.214	0.45	40	u	0.123216
			232497_at	0	4.399	0.29	7	4.213	0.46	40	d	0.311896
			219604_s_at	0	2.545	0.14	7	2.912	0.55	40	u	0.001133
TMEM49	4	transmembrane protein 49	231697_s_at	0	7.314	0.89	7	8.158	1.11	40	u	0.068219
			1569003_at	0	8.1	0.76	7	8.273	1.66	40	u	0.791821
P2RY4	4	pyrimidinergic receptor P2Y, G-protein coupled, 4	221466_at	0	1.925	0.08	7	1.98	0.12	40	u	0.246646
GPBP1	4	GC-rich promoter binding protein 1	223705_s_at	4	10.47	0.24	7	10.17	0.49	40	d	0.13462
			224648_at	4	8.354	0.29	7	7.784	0.77	40	d	0.064656
STCH	4	stress 70 protein chaperone, microsome-associated, 60kDa	202557_at	4	7.769	0.28	7	8.481	0.83	40	u	0.000445
			202558_s_at	0	5.971	0.54	7	7.392	1.29	40	u	0.007155
GBA2	4	glucosidase, beta (bile acid) 2	224627_at	4	7.526	0.13	7	6.765	0.62	40	d	0
			223921_s_at	0	6.871	0.33	7	5.885	1.07	40	d	0.000094
GRIN3A	4	glutamate receptor, ionotropic, N-methyl-D-aspartate 3A	233220_at	4	2.063	0.05	7	2.171	0.2	40	u	0.009497
			233171_at	1	2.615	0.27	7	2.63	0.24	40	u	0.881364
SMOC2	4	SPARC related modular calcium binding 2	223235_s_at	4	10.18	0.5	7	6.863	1.69	40	d	0
YBX1	4	Y box binding protein 1	208628_s_at	4	12.32	0.13	7	12.7	0.84	40	u	0.011223
			208627_s_at	4	11.51	0.29	7	11.92	1.1	40	u	0.058084
PDCD6	4	programmed cell death 6	203415_at	1	8.883	0.18	7	8.888	0.72	40	u	0.970402
			222380_s_at	0	6.93	0.7	7	7.039	1.67	40	u	0.869048
			234297_at	0	4.378	0.47	7	3.914	0.79	40	d	0.145601
			1568764_x_at	0	4.104	0.44	7	4.119	0.6	40	u	0.949477
			1568763_s_at	0	8.611	0.32	7	8.599	0.93	40	d	0.952551
			1568762_at	0	3.152	0.38	7	3.158	0.2	40	u	0.970777
			229283_at	0	4.723	0.73	7	4.81	1.02	40	u	0.835072
			222381_at	0	5.603	0.47	7	5.542	0.64	40	d	0.817403
			222152_at	0	6.614	0.51	7	6.233	1.12	40	d	0.39142
DCTN4	4	dynactin 4 (p62)	233490_at	4	2.945	0.32	7	2.922	0.15	40	d	0.862105
			222488_s_at	4	9.961	0.22	7	9.573	0.64	40	d	0.00872
			218013_x_at	0	7.462	0.36	7	7.038	1.15	40	d	0.084242
AMPH	4	amphiphysin	205257_s_at	4	5.014	0.97	7	4.497	1.89	40	d	0.492479
MAN1A1	4	mannosidase, alpha, class 1A, member 1	208116_s_at	4	5.62	0.87	7	5.936	1.14	40	u	0.498975
			221760_at	4	9.475	0.56	7	8.549	1.23	40	d	0.060963
FBXO34	4	F-box protein 34	218539_at	4	8.86	0.39	7	8.538	0.57	40	d	0.165955
			1570183_at	0	2.956	0.1	7	3.129	0.2	40	u	0.031183
ZDHHC1	4	zinc finger, DHHC-type containing 1	228637_at	4	4.279	0.77	7	3.806	0.57	40	d	0.066713
			244494_at	0	4.619	0.47	7	4.333	0.55	40	d	0.207463
			237228_at	0	2.986	0.41	7	2.935	0.21	40	d	0.775297
HTR4	4	5-hydroxytryptamine (serotonin) receptor 4	207577_at	4	2.265	0.03	7	2.3	0.07	40	u	0.238199
			216939_s_at	0	2.739	0.19	7	2.775	0.32	40	u	0.777768
			207578_s_at	0	5.098	0.19	7	5.475	0.44	40	u	0.0345
ZDHHC7	4	zinc finger, DHHC-type containing 7	218606_at	4	9.775	0.11	7	9.233	0.56	40	d	0.000003
TSKU	4	tsukushin	218245_at	4	9.204	0.89	7	7.839	0.92	40	d	0.000844
SPRY1	4	sprouty homolog 1, antagonist of FGF signaling (Drosophila)	212558_at	4	9.478	0.33	7	7.317	0.73	40	d	0
CDON	4	Cdon homolog (mouse)	207230_at	4	3.139	0.21	7	3.142	0.35	40	u	0.97993
			227526_at	0	6.745	0.54	7	5.281	1.43	40	d	0.011871
GNAI1	4	guanine nucleotide binding protein (G protein), alpha inhibiting activity polypeptide	227692_at	4	7.397	0.77	7	6.08	1.12	40	d	0.005275
			209576_at	2	6.675	0.46	7	5.881	1.19	40	d	0.094655
RBM16	4	RNA binding motif protein 16	203250_at	4	8.879	0.29	7	8.566	0.62	40	d	0.205567
SNX5	4	sorting nexin 5	229980_s_at	4	10.33	0.16	7	10.68	0.53	40	u	0.002909
			217792_at	4	9.652	0.2	7	9.255	0.49	40	d	0.042668
			222417_s_at	4	9.22	0.31	7	9.536	0.78	40	u	0.307967
			229981_at	4	5.049	0.35	7	5.469	0.74	40	u	0.156548
			238433_at	0	2.75	0.25	7	2.814	0.27	40	u	0.56374
			223666_at	0	4.688	0.42	7	5.554	0.91	40	u	0.019206
ZSCAN21	4	zinc finger and SCAN domain containing 21	223424_s_at	1	7.351	0.28	7	6.854	0.77	40	d	0.007076
ABCB9	4	ATP-binding cassette, sub-family B (MDR/TAP), member 9	207321_s_at	4	5.76	0.25	7	5.757	0.44	40	d	0.986362
			214209_s_at	4	5.79	0.26	7	5.915	0.68	40	u	0.640481
			1555323_at	0	6.248	0.33	7	6.362	0.43	40	u	0.51707
HOXC4	4	homeobox C4	206194_at	4	7.657	0.38	7	7.72	1.06	40	u	0.788384
CNTN1	4	contactin 1	211203_s_at	1	3.132	0.53	7	3.056	0.61	40	d	0.763326
			227202_at	0	3.999	0.36	7	3.491	0.73	40	d	0.084085

CNTN1	4	contactin 1	1554784_at	0	3.387	0.29	7	3.432	0.3	40	u	0.714661
			227209_at	0	6.906	0.65	7	5.669	0.79	40	d	0.000395
HOXC10	4	homeobox C10	218959_at	4	7.027	0.59	7	7.388	2.43	40	u	0.435855
EML4	4	echinoderm microtubule associated protein like 4	223068_at	4	8.958	0.19	7	9.124	0.68	40	u	0.219154
			228673_s_at	0	2.97	0.3	7	3.733	0.8	40	u	0.018511
			228674_s_at	0	6.242	0.47	7	7.056	0.78	40	u	0.012129
			220386_s_at	0	6.625	0.71	7	7.399	1.41	40	u	0.171956
			1556732_at	0	4.636	0.64	7	4.372	0.67	40	d	0.350606
			223069_s_at	0	6.506	0.44	7	6.92	0.82	40	u	0.210219
			232587_at	0	5.882	0.85	7	5.511	0.75	40	d	0.254668
TTL	4	tubulin tyrosine ligase	224896_s_at	4	7.36	0.37	7	7.851	0.68	40	u	0.076433
			224908_s_at	4	6.859	0.19	7	7.672	0.68	40	u	0.000001
			1559680_at	0	2.408	0.07	7	2.552	0.19	40	u	0.001765
UBE2O	4	ubiquitin-conjugating enzyme E2O	218141_at	4	6.279	0.2	7	6.425	0.61	40	u	0.260205
			1554814_at	0	2.816	0.49	7	2.943	0.49	40	u	0.537223
ARHGAP29	4	Rho GTPase activating protein 29	203910_at	4	10.37	0.41	7	9.512	1.38	40	d	0.004052
			1560318_at	0	4.242	0.82	7	4.417	1.37	40	u	0.749696
			1554736_at	0	3.561	0.2	7	4	0.79	40	u	0.005682
TAL1	4	T-cell acute lymphocytic leukemia 1	206283_s_at	4	3.62	0.72	7	3.057	0.32	40	d	0.106919
			216928_at	2	2.353	0.06	7	2.452	0.22	40	u	0.03108
			216925_s_at	2	2.559	0.21	7	2.555	0.23	40	d	0.95942
			1561651_s_at	0	5.568	0.48	7	6.073	0.57	40	u	0.035276
PTPRG	4	protein tyrosine phosphatase, receptor type, G	204944_at	4	5.141	0.78	7	4.762	0.8	40	d	0.261644
			1569323_at	0	3.897	0.62	7	3.338	0.64	40	d	0.041719
PRDM10	4	PR domain containing 10	219515_at	4	6.788	0.53	7	6.257	0.54	40	d	0.022576
			214158_s_at	0	2.882	0.32	7	3.106	0.5	40	u	0.267607
SRGAP1	4	SLIT-ROBO Rho GTPase activating protein 1	233888_s_at	4	6.729	0.29	7	5.87	1.13	40	d	0.000342
			1569269_s_at	0	4.768	0.6	7	4.572	0.73	40	d	0.514355
			1554473_at	0	3.559	0.29	7	3.82	0.82	40	u	0.151688
ELMO1	4	engulfment and cell motility 1	204513_s_at	4	6.011	0.57	7	5.126	1.02	40	d	0.034382
SLC24A4	4	solute carrier family 24 (sodium/potassium/calcium exchanger),	243969_at	4	2.132	0.09	7	2.237	0.2	40	u	0.194445
			1568922_at	0	2.845	0.29	7	2.822	0.3	40	d	0.856433
RANBP5	4	RAN binding protein 5	211953_s_at	4	7.603	0.22	7	8.115	0.85	40	u	0.003464
			211952_at	4	6.57	0.14	7	6.519	0.76	40	d	0.701787
			211954_s_at	4	9.123	0.24	7	9.086	0.76	40	d	0.813723
			211955_at	4	9.071	0.17	7	9.075	0.79	40	u	0.978393
			236906_x_at	0	2.94	0.22	7	3.112	0.34	40	u	0.213171
C20orf23	4	chromosome 20 open reading frame 23	219570_at	4	7.66	0.9	7	7.008	0.95	40	d	0.106735
			232083_at	0	6.316	0.68	7	5.605	1.41	40	d	0.205959
RHOQ	4	ras homolog gene family, member Q	212122_at	4	5.91	0.51	7	6.002	0.79	40	u	0.770591
			212119_at	4	10.3	0.31	7	10.07	0.58	40	d	0.336041
			212120_at	4	9.03	0.37	7	8.688	0.68	40	d	0.211372
			212117_at	4	9.471	0.34	7	9.129	0.56	40	d	0.135448
			214449_s_at	3	7.825	0.37	7	7.693	0.89	40	d	0.706527
			1559582_at	0	4.457	0.64	7	4.002	0.4	40	d	0.018237
PSKH1	4	protein serine kinase H1	213141_at	4	6.779	0.28	7	6.002	0.6	40	d	0.001968
PDHX	4	pyruvate dehydrogenase complex, component X	203067_at	4	8.193	0.31	7	8.564	0.76	40	u	0.218561
CASR	4	calcium-sensing receptor (hypocalciuric hypercalcemia 1, severe neonatal hyperparathyroidism)	210577_at	4	2.48	0.15	7	2.541	0.34	40	u	0.650147
			242744_s_at	0	2.216	0.02	7	2.293	0.11	40	u	0.000162
			211384_s_at	0	2.531	0.16	7	2.572	0.19	40	u	0.598746
			240886_at	0	4.857	0.53	7	4.74	0.42	40	d	0.524452
LIN9	4	lin-9 homolog (C. elegans)	230365_at	4	3.077	0.05	7	3.416	0.52	40	u	0.000308
			235039_x_at	3	4.865	0.59	7	6.071	0.75	40	u	0.000252
			1552771_a_at	0	2.311	0.02	7	2.398	0.13	40	u	0.000551
LRP12	4	low density lipoprotein-related protein 12	219631_at	6	5.606	0.28	7	6.128	1.05	40	u	0.014208
			220253_s_at	2	3.714	0.37	7	4.688	1.34	40	u	0.000736
			220254_at	2	3.781	0.27	7	4.852	0.95	40	u	0.000002
TBC1D22A	4	TBC1 domain family, member 22A	209650_s_at	4	6.163	0.29	7	6.082	0.57	40	d	0.720772
			210144_at	4	6.405	0.24	7	5.88	0.48	40	d	0.007826
			33778_at	4	7.38	0.13	7	7.361	0.51	40	d	0.850815
FAM123A	4	family with sequence similarity 123A	235465_at	4	2.652	0.16	7	2.774	0.7	40	u	0.345612
			1553720_a_at	1	3.659	0.21	7	3.814	0.38	40	u	0.308994
			230496_at	0	2.366	0.04	7	2.781	0.83	40	u	0.003511

PEG3	4	paternally expressed 3	230068_s_at	4	3.648	0.45	7	4.013	1.36	40	u	0.213921
			209243_s_at	4	6.4	0.59	7	4.768	2.31	40	d	0.000715
			209242_at	2	8.161	0.4	7	5.606	2.76	40	d	0.000002
SYDE1	4	synapse defective 1, Rho GTPase, homolog 1 (C. elegans)	216272_x_at	4	7.115	0.29	7	7.021	0.4	40	d	0.558722
			44702_at	4	7.201	0.4	7	6.586	0.51	40	d	0.004911
			212962_at	4	4.487	0.46	7	4.252	0.72	40	d	0.419145
			216271_x_at	0	1.783	0.01	7	1.886	0.18	40	u	0.000814
FAM62B	4	family with sequence similarity 62 (C2 domain containing) member B	224699_s_at	4	8.578	0.48	7	9.332	0.81	40	u	0.023529
			224698_at	4	9.578	0.3	7	9.03	0.87	40	d	0.00675
			1558511_s_at	0	8.191	0.51	7	9.257	0.82	40	u	0.002226
			1555830_s_at	0	5.361	0.86	7	6.587	1.68	40	u	0.070414
			1555829_at	0	4.255	0.51	7	5.714	1.31	40	u	0.006778
			239334_at	0	6.335	0.26	7	6.265	0.39	40	d	0.653358
PCDHGA11	4	protocadherin gamma subfamily A, 11	211877_s_at	0	2.49	0.04	7	2.537	0.07	40	u	0.103757
RNH1	4	ribonuclease/angiogenin inhibitor 1	206050_s_at	2	10.28	0.13	7	9.378	0.53	40	d	0
			216798_at	0	2.476	0.1	7	2.556	0.24	40	u	0.395937
			1555529_at	0	2.343	0.08	7	2.434	0.15	40	u	0.134443
PRRG3	4	proline rich Gla (G-carboxyglutamic acid) 3 (transmembrane)	220433_at	4	2.014	0.09	7	2.032	0.06	40	u	0.503388
PABPL1	4	poly(A) binding protein-like 1	no probeset available									
RAB11FIP5	4	RAB11 family interacting protein 5 (class I)	210879_s_at	4	8.213	0.24	7	7.368	0.63	40	d	0.00125
FBXL3	4	F-box and leucine-rich repeat protein 3	225132_at	4	9.959	0.23	7	8.933	0.82	40	d	0
			242829_x_at	0	6.694	0.42	7	6.919	0.88	40	u	0.520085
MPHOSPH9	4	M-phase phosphoprotein 9	206205_at	4	2.926	0.43	7	4.3	0.86	40	u	0.000207
			215731_s_at	0	3.773	0.27	7	4.002	0.67	40	u	0.384793
			237158_s_at	0	2.656	0.2	7	3.916	1.11	40	u	0
			221965_at	0	5.602	0.67	7	5.971	0.73	40	u	0.231209
			1558369_at	0	5.293	0.62	7	5.903	1.07	40	u	0.158952
HCN4	4	hyperpolarization activated cyclic nucleotide-gated potassium channel 4	206946_at	4	2.033	0.02	7	2.082	0.05	40	u	0.018743
NONO	4	non-POU domain containing, octamer-binding	200057_s_at	4	12.19	0.2	7	11.87	0.48	40	d	0.092831
			210470_x_at	2	10.52	0.33	7	10.24	0.89	40	d	0.422903
			208698_s_at	1	10.19	0.36	7	10.03	0.93	40	d	0.661508
JMJD1A	4	jumonji domain containing 1A	212689_s_at	4	9.118	0.24	7	8.974	0.64	40	d	0.566696
HOXC11	4	homeobox C11	206745_at	4	5.561	0.73	7	6.16	1.16	40	u	0.204114
KLHL20	4	kelch-like 20 (Drosophila)	204176_at	4	5.106	0.46	7	5.189	0.71	40	u	0.771017
			204177_s_at	0	7.748	0.18	7	7.335	0.59	40	d	0.001754
			210635_s_at	0	4.899	0.49	7	4.685	0.85	40	d	0.531919
			210634_at	0	2.785	0.28	7	2.906	0.38	40	u	0.427783
KLHL5	4	kelch-like 5 (Drosophila)	226001_at	4	8.026	0.44	7	8.181	0.89	40	u	0.662389
			233866_at	0	3.852	0.54	7	4.076	0.59	40	u	0.364523
DLG5	4	discs, large homolog 5 (Drosophila)	201681_s_at	4	10.26	0.5	7	9.17	1.26	40	d	0.031933
			210469_at	0	7.718	1.01	7	5.046	1.93	40	d	0.001058
SH3RF1	4	SH3 domain containing ring finger 1	225590_at	4	5.962	0.43	7	6.736	1.19	40	u	0.006549
			225589_at	4	7.476	0.4	7	8.005	0.81	40	u	0.106445
ASTN1	4	astrotactin 1	213197_at	4	2.216	0.06	7	2.359	0.47	40	u	0.074877
PCDHGB3	4	protocadherin gamma subfamily B, 3	no probeset available									
PCDHGC3	4	protocadherin gamma subfamily C, 3	214563_at	0	4.338	0.26	7	4.261	0.47	40	d	0.682237
			214564_s_at	0	2.688	0.42	7	2.751	0.91	40	u	0.862185
MAPKBP1	4	mitogen activated protein kinase binding protein 1	1558839_at	4	2.256	0.05	7	2.36	0.2	40	u	0.008045
			213394_at	4	6.674	0.42	7	5.179	0.99	40	d	0.00037
UST	4	uronyl-2-sulfotransferase	205138_s_at	4	2.528	0.09	7	2.552	0.1	40	u	0.5784
			205139_s_at	4	5.673	0.7	7	4.353	1.22	40	d	0.008911
CALD1	4	caldesmon 1	201615_x_at	3	8.406	0.24	7	8.393	1.11	40	d	0.9486
			214880_x_at	3	8.533	0.33	7	8.218	0.53	40	d	0.143904
			201616_s_at	3	9.825	0.61	7	8.91	1.01	40	d	0.027335
			201617_x_at	3	9.097	0.52	7	8.488	1.27	40	d	0.226934
			212077_at	0	11.75	0.25	7	10.71	0.79	40	d	0.000001
			231881_at	0	3.34	0.86	7	2.619	0.54	40	d	0.006336
			205525_at	0	7.49	1.02	7	6.054	1.23	40	d	0.006447
			235834_at	0	7.756	0.79	7	7.088	0.79	40	d	0.049751
			215198_s_at	0	7.39	0.69	7	5.978	0.86	40	d	0.000217
			215199_at	0	7.171	1	7	5.011	1.17	40	d	0.00005

			243084_at	0	5.98	0.73	7	5.479	0.87	40	d	0.168383
NSMAF	4	neutral sphingomyelinase (N-SMase) activation associated factor	203269_at	4	8.634	0.22	7	8.767	0.84	40	u	0.414684
			240056_at	0	4.64	0.38	7	4.708	0.39	40	u	0.680868
			1558775_s_at	0	4.426	0.7	7	5.094	1.03	40	u	0.112696
			232148_at	0	6.055	0.65	7	4.999	1.11	40	d	0.021135
			232149_s_at	0	7.294	0.35	7	6.99	1.06	40	d	0.181289
HMGCS1	4	3-hydroxy-3-methylglutaryl-Coenzyme A synthase 1 (soluble)	221750_at	4	7.914	0.62	7	8.203	0.93	40	u	0.440889
			205822_s_at	0	4.896	0.99	7	5.89	1.68	40	u	0.144872
WRNIP1	4	Werner helicase interacting protein 1	218015_s_at	4	2.659	0.42	7	2.651	0.33	40	d	0.95195
			222489_s_at	4	9.04	0.26	7	9.068	0.71	40	u	0.854669
			227619_at	0	2.53	0.12	7	2.631	0.2	40	u	0.215906
C12orf23	4	chromosome 12 open reading frame 23	224759_s_at	4	9.356	0.34	7	9.684	0.81	40	u	0.3093
SEC61A2	4	Sec61 alpha 2 subunit (S. cerevisiae)	219499_at	4	3.804	0.53	7	5.009	0.83	40	u	0.000749
			230215_at	0	3.341	0.35	7	3.321	0.25	40	d	0.861302
			228747_at	0	5.582	0.35	7	6.126	0.89	40	u	0.125446
XAB1	4	XPA binding protein 1, GTPase	209313_at	4	8.176	0.27	7	8.816	0.69	40	u	0.022188
C11orf56	4	chromosome 11 open reading frame 56	223637_s_at	4	8.122	0.36	7	7.011	0.74	40	d	0.000448
CSNK2A1	4	casein kinase 2, alpha 1 polypeptide	212073_at	4	7.141	0.29	7	7.595	0.59	40	u	0.055326
			229212_at	4	3.773	0.16	7	4.076	0.4	40	u	0.061742
			212075_s_at	4	7.522	0.23	7	8.242	0.69	40	u	0.000044
			212072_s_at	4	7.854	0.16	7	8.777	0.6	40	u	0
			206075_s_at	2	7.143	0.39	7	7.799	1.08	40	u	0.010501
			229216_s_at	0	2.376	0.25	7	2.563	0.38	40	u	0.231808
TWIST1	4	twist homolog 1 (acrocephalosyndactyly 3; Saethre-Chotzen syndrome) (Drosophila)	213943_at	2	7.872	0.51	7	6.768	1.09	40	d	0.013344
TMOD3	4	tropomodulin 3 (ubiquitous)	223077_at	4	7.632	0.16	7	8.174	0.56	40	u	0.000033
			220800_s_at	3	5.304	0.67	7	5.778	1.03	40	u	0.255938
			223078_s_at	1	5.777	0.51	7	6.059	0.81	40	u	0.386857
MAB21L1	4	mab-21-like 1 (C. elegans)	206163_at	4	5.403	0.84	7	2.743	0.47	40	d	0.000278
ARL5A	4	ADP-ribosylation factor-like 5A	218150_at	4	9.305	0.13	7	9.774	0.47	40	u	0.000014
			226617_at	0	8.562	0.19	7	9.108	0.55	40	u	0.000082
SYT4	4	synaptotagmin IV	223529_at	4	3.557	0.32	7	4.042	0.95	40	u	0.023181
BAAT	4	bile acid Coenzyme A: amino acid N-acyltransferase (glycine N-choloyltransferase)	206913_at	0	2.527	0.36	7	2.426	0.25	40	d	0.37831
PCDHGA3	4	protocadherin gamma subfamily A, 3	216352_x_at	0	3.311	0.42	7	2.835	0.88	40	d	0.179085
			216343_at	0	2.118	0.01	7	2.152	0.07	40	u	0.005531
			211879_x_at	0	2.363	0.39	7	2.561	0.81	40	u	0.538311
FAM13C1	4	family with sequence similarity 13, member C1	1554547_at	10	6.104	0.41	7	4.174	0.66	40	d	0
			214914_at	0	2.548	0.07	7	2.628	0.09	40	u	0.036917
NRXN2	4	neurexin 2	209982_s_at	4	4.623	0.84	7	3.791	0.82	40	d	0.020056
			209983_s_at	4	3.814	0.5	7	3.229	0.64	40	d	0.028257
ZNF22	4	zinc finger protein 22 (KOX 15)	218005_at	0	8.894	0.27	7	8.46	0.91	40	d	0.024165
			218006_s_at	0	7.737	0.24	7	7.638	1.04	40	d	0.610142
AXUD1	4	AXIN1 up-regulated 1	225557_at	4	8.403	0.52	7	6.04	1.01	40	d	0
KLF3	4	Kruppel-like factor 3 (basic)	222913_at	3	6.542	0.18	7	6.701	0.25	40	u	0.120698
			225140_at	3	5.123	0.88	7	5.766	1.23	40	u	0.200959
			225133_at	3	8.152	0.33	7	7.734	0.76	40	d	0.167512
			219657_s_at	1	5.754	0.54	7	6.122	0.85	40	u	0.286362
KIF26B	4	kinesin family member 26B	220002_at	4	4.233	0.34	7	4.399	0.71	40	u	0.557035
HIVEP1	4	human immunodeficiency virus type I enhancer binding protein 1	204512_at	4	6.911	0.43	7	7.34	0.55	40	u	0.060269
			1560485_at	0	2.087	0.11	7	2.216	0.32	40	u	0.068646
MDFIC	4	MyoD family inhibitor domain containing	217599_s_at	3	4.205	0.29	7	4.901	0.89	40	u	0.000934
			211675_s_at	3	8.963	0.21	7	8.487	0.96	40	d	0.009835
			1559942_at	0	2.685	0.21	7	3.183	0.65	40	u	0.001014
			239172_x_at	0	3.077	0.21	7	3.153	0.33	40	u	0.57019
AXIN1	4	axin 1	212849_at	4	6.227	0.47	7	6.563	0.66	40	u	0.210534
TRIO	4	triple functional domain (PTPRF interacting)	209010_s_at	4	2.462	0.13	7	2.595	0.43	40	u	0.133313
			209012_at	4	7.526	0.29	7	6.422	0.88	40	d	0.000002
			209011_at	4	8.013	0.25	7	7.436	0.57	40	d	0.013985
			209013_x_at	3	7.103	0.37	7	6.876	0.66	40	d	0.387713
			208178_x_at	1	7.332	0.42	7	7.236	0.81	40	d	0.767963
			244527_at	0	2.474	0.04	7	2.523	0.06	40	u	0.056733

			216697_at	0	3.715	0.33	7	3.744	0.51	40	u	0.887843
			231403_at	0	5.92	0.63	7	5.167	0.92	40	d	0.046879
			216700_at	0	2.982	0.61	7	3.044	0.58	40	u	0.799363
IQCK	4	IQ motif containing K	213392_at	4	8.084	0.36	7	7.796	0.75	40	d	0.335395
			215131_at	0	6.148	0.22	7	6.132	0.55	40	d	0.940172
			215130_s_at	0	6.125	0.39	7	6.379	0.73	40	u	0.385256
			216135_at	0	3.964	0.36	7	4.161	0.55	40	u	0.371559
IRF1	4	interferon regulatory factor 1	202531_at	4	7.303	0.54	7	7.645	1.33	40	u	0.516006
ZNF230	4	zinc finger protein 230	205791_x_at	0	4.011	0.73	7	3.845	0.79	40	d	0.614924
			1570135_at	0	3.322	0.34	7	3.553	0.52	40	u	0.2723
			1557322_at	0	2.749	0.3	7	3.105	0.5	40	u	0.078736
SMARCD1	4	SWI/SNF related, matrix associated, actin dependent regulator of chromatin, subfamily	209518_at	4	8.134	0.19	7	7.825	0.52	40	d	0.132135
			203183_s_at	3	6.421	0.44	7	6.335	0.63	40	d	0.73731
CRY2	4	cryptochrome 2 (photolyase-like)	212695_at	4	8.035	0.5	7	6.593	0.59	40	d	0
BMPR1A	4	bone morphogenetic protein receptor, type IA	204832_s_at	4	7.404	0.31	7	7.09	0.91	40	d	0.117793
			213578_at	4	8.184	0.34	7	7.932	0.81	40	d	0.432583
CIB2	4	calcium and integrin binding family member 2	205007_s_at	4	3.236	0.56	7	3.457	0.97	40	u	0.570566
			205008_s_at	4	6.003	0.31	7	6.465	0.95	40	u	0.027935
			214065_s_at	0	2.675	0.03	7	2.712	0.06	40	u	0.115545
FUBP3	4	far upstream element (FUSE) binding protein 3	212824_at	4	7.295	0.22	7	7.175	0.6	40	d	0.611948
NFATC1	4	nuclear factor of activated T-cells, cytoplasmic, calcineurin-dependent 1	209664_x_at	4	2.244	0.24	7	2.354	0.46	40	u	0.549992
			211105_s_at	4	6.518	0.4	7	5.894	1.09	40	d	0.14967
			208196_x_at	2	3.881	0.5	7	3.61	0.66	40	d	0.314357
			210162_s_at	0	6.461	0.39	7	6.215	0.99	40	d	0.528288
SCOC	4	short coiled-coil protein	224786_at	4	9.98	0.26	7	10.15	0.68	40	u	0.534327
			223341_s_at	2	7.921	0.45	7	8.004	1.19	40	u	0.85921
SMAD3	4	SMAD family member 3	218284_at	3	8.296	0.44	7	7.374	0.98	40	d	0.021123
			205396_at	1	3.791	0.81	7	3.947	1.22	40	u	0.752533
			205398_s_at	0	6.088	0.44	7	5.842	0.7	40	d	0.382581
			205397_x_at	0	4.255	0.65	7	3.79	1.24	40	d	0.349349
CACNB3	4	calcium channel, voltage-dependent, beta 3 subunit	209530_at	4	7.001	0.17	7	7.631	0.91	40	u	0.000352
			34726_at	4	7.038	0.18	7	7.728	0.95	40	u	0.00019
EZH2	4	enhancer of zeste homolog 2 (Drosophila)	203358_s_at	4	4.826	0.52	7	7.925	0.91	40	u	0
CSTF3	4	cleavage stimulation factor, 3' pre-RNA, subunit 3, 77kDa	203947_at	4	8.337	0.37	7	8.594	0.67	40	u	0.336881
			229666_s_at	0	7.163	0.54	7	7.955	0.95	40	u	0.042118
			229665_at	0	6.482	0.34	7	7.326	0.97	40	u	0.000477
TXNDC1	4	thioredoxin domain containing 1	209476_at	4	9.638	0.16	7	9.958	0.73	40	u	0.020267
			208097_s_at	0	8.197	0.49	7	8.05	1.68	40	d	0.663165
PCDHGA7	4	protocadherin gamma subfamily A, 7	no probeset available									
RTN3	4	reticulon 3	224564_s_at	4	9.007	0.31	7	9.23	0.64	40	u	0.378205
			219549_s_at	4	10.46	0.38	7	9.687	0.84	40	d	0.024204
SLCO3A1	4	solute carrier organic anion transporter family, member 3A1	229776_at	4	3.794	0.65	7	3.911	1.08	40	u	0.787257
			219229_at	4	6.727	0.61	7	5.708	1.3	40	d	0.052553
			227367_at	0	6.12	0.45	7	5.553	0.95	40	d	0.137215
			210542_s_at	0	4.353	0.96	7	3.861	0.67	40	d	0.108405
PIB5PA	4	phosphatidylinositol (4,5) bisphosphate 5-phosphatase, A	213651_at	4	6.452	0.66	7	4.933	1.31	40	d	0.005128
FAT2	4	FAT tumor suppressor homolog 2 (Drosophila)	208153_s_at	4	5.181	0.89	7	3.082	0.68	40	d	0
GATAD2A	4	GATA zinc finger domain containing 2A	222526_at	4	7.087	0.26	7	6.872	0.73	40	d	0.187685
			218131_s_at	4	7.959	0.31	7	8.523	0.73	40	u	0.054949
			234294_x_at	4	8.192	0.41	7	8.768	0.71	40	u	0.046042
			229166_s_at	0	2.303	0.03	7	2.343	0.04	40	u	0.029058
			238324_at	0	5.849	0.19	7	6.023	0.54	40	u	0.142854
			236932_s_at	0	2.332	0.12	7	2.398	0.16	40	u	0.318507
GPRASP2	4	G protein-coupled receptor associated sorting protein 2	228027_at	4	8.822	0.24	7	6.436	1.19	40	d	0
CCM2	4	cerebral cavernous malformation 2	223164_at	4	7.644	0.2	7	7.427	0.58	40	d	0.090979
FAM130A2	4	family with sequence similarity 130, member A2	220462_at	4	3.374	0.73	7	2.791	0.5	40	d	0.013279
TUSC2	4	tumor suppressor candidate 2	203272_s_at	4	6.947	0.21	7	6.681	0.72	40	d	0.073324
			203273_s_at	4	7.518	0.16	7	7.134	0.6	40	d	0.002406
			211421_s_at	4	4.393	2.24	7	5.464	2.39	40	u	0.285506

RET	4	ret proto-oncogene	215771_x_at	0	3.243	1.22	7	4.218	1.77	40	u	0.177976
			205879_x_at	0	4.652	1.25	7	5.232	1.85	40	u	0.438314
ATP2C1	4	ATPase, Ca++ transporting, type 2C, member 1	212255_s_at	4	8.079	0.28	7	8.598	0.59	40	u	0.029323
			211137_s_at	4	6.677	0.52	7	6.736	0.82	40	u	0.859676
			209934_s_at	0	6.379	0.24	7	6.995	0.64	40	u	0.018297
			209935_at	0	5.361	0.52	7	6.165	0.87	40	u	0.025204
MAPK9	4	mitogen-activated protein kinase 9	203218_at	4	8.145	0.25	7	8.555	0.92	40	u	0.029617
			225781_at	0	7.183	0.4	7	7.209	0.69	40	u	0.92247
			210570_x_at	0	6.46	0.34	7	6.699	1.04	40	u	0.280405
OTX1	4	orthodenticle homeobox 1	238839_at	0	2.393	0.25	7	2.467	0.37	40	u	0.619864
NLGN2	4	neuroligin 2	226288_s_at	4	7.64	0.27	7	7.232	0.52	40	d	0.053128
			235838_at	0	2.123	0.08	7	2.199	0.26	40	u	0.163281
			1554428_s_at	0	3.854	0.65	7	4.409	0.91	40	u	0.138494
PLAGL1	4	pleiomorphic adenoma gene-like 1	207002_s_at	4	6.714	0.66	7	5.446	1.28	40	d	0.016141
			207943_x_at	4	5.867	0.85	7	4.932	1.03	40	d	0.031622
			209318_x_at	4	8.833	0.42	7	7.06	1.55	40	d	0.000001
DYRK2	4	dual-specificity tyrosine-(Y)-phosphorylation regulated kinase 2	202968_s_at	4	6.323	0.54	7	7.271	0.71	40	u	0.00196
			202971_s_at	4	6.014	0.46	7	7.948	0.88	40	u	0.000002
KLF6	4	Kruppel-like factor 6	224606_at	0	11.72	0.37	7	10.73	0.81	40	d	0.003253
			211610_at	0	6.086	0.53	7	5.484	0.99	40	d	0.133331
			208961_s_at	0	10.68	0.54	7	9.951	0.9	40	d	0.047905
			1555832_s_at	0	11.33	0.47	7	10.31	0.96	40	d	0.009953
			208960_s_at	0	8.736	0.74	7	8.135	1.28	40	d	0.24357
SRR	4	serine racemase	219204_s_at	5	6.519	0.38	7	5.633	0.7	40	d	0.002468
			219205_at	5	6.097	0.28	7	5.108	0.94	40	d	0.000012
			222844_s_at	1	4.333	0.38	7	4.093	0.71	40	d	0.39753
			235677_at	0	3.655	0.69	7	3.726	0.73	40	u	0.81541
ARPC5	4	actin related protein 2/3 complex, subunit 5, 16kDa	211963_s_at	4	10.82	0.18	7	11.34	0.75	40	u	0.000709
			1555797_a_at	0	8.792	0.24	7	9.554	0.92	40	u	0.000119
			1569325_at	0	2.403	0.15	7	2.738	0.41	40	u	0.044644
LEF1	4	lymphoid enhancer-binding factor 1	221557_s_at	0	4.524	0.44	7	4.229	0.48	40	d	0.143699
			210948_s_at	0	3.867	0.35	7	4.479	1.03	40	u	0.009229
			221558_s_at	0	5.581	1.31	7	6.143	1.95	40	u	0.476606
CCNL2	4	cyclin L2	222999_s_at	4	10.5	0.22	7	9.291	0.85	40	d	0
			232274_at	4	7.161	0.3	7	6.04	0.59	40	d	0.000017
			221427_s_at	0	8.935	0.22	7	7.716	1.02	40	d	0
			225555_x_at	0	4.525	0.26	7	4.549	0.42	40	u	0.882812
ANKS1B	4	ankyrin repeat and sterile alpha motif domain containing 1B	227439_at	4	3.727	0.61	7	3.669	0.78	40	d	0.856964
			227441_s_at	4	2.069	0.12	7	2.176	0.57	40	u	0.302334
			240292_x_at	4	3.448	0.54	7	3.445	0.35	40	d	0.985387
			227440_at	4	3.734	0.74	7	3.317	0.64	40	d	0.136741
			219989_s_at	0	2.836	0.09	7	2.868	0.12	40	u	0.524238
VANGL2	4	vang-like 2 (van gogh, Drosophila)	226029_at	4	7.676	0.34	7	7.208	2.18	40	d	0.219451
WWC1	4	WW and C2 domain containing 1	213085_s_at	4	8.062	0.41	7	6.882	0.77	40	d	0.00037
			237238_at	0	4.941	0.7	7	4.264	0.9	40	d	0.070161
			1554511_at	0	2.144	0.03	7	2.196	0.05	40	u	0.010827
			236725_at	0	7.303	0.66	7	5.553	1.17	40	d	0.000464
			243290_at	0	6.018	0.56	7	5.394	0.6	40	d	0.01555
			216074_x_at	0	5.063	0.52	7	3.812	0.65	40	d	0.000023
CUTL2	4	cut-like 2 (Drosophila)	213920_at	4	3.351	1.4	7	2.501	0.45	40	d	0.190246
PCDHGC4	4	protocadherin gamma subfamily C, 4	231753_s_at	0	2.11	0.02	7	2.148	0.03	40	u	0.006351
			231754_at	0	2.334	0.08	7	2.427	0.29	40	u	0.102315
ANGEL1	4	angel homolog 1 (Drosophila)	213099_at	4	6.611	0.32	7	6.436	0.45	40	d	0.339224
			36865_at	4	6.324	0.21	7	6.227	0.31	40	d	0.441155
KIF21A	4	kinesin family member 21A	226003_at	4	6.385	0.35	7	8.012	0.98	40	u	0
			231875_at	0	4.92	0.53	7	6.301	0.75	40	u	0.000039
RNF12	4	ring finger protein 12	222815_at	4	4.113	0.67	7	4.82	1.13	40	u	0.122166
			242121_at	0	10.68	0.53	7	9.05	1.26	40	d	0.001838
			244470_at	0	5.361	0.52	7	4.877	0.67	40	d	0.084317
			225416_at	0	8.497	0.34	7	8.665	0.59	40	u	0.477719
C15orf29	4	chromosome 15 open reading frame 29	222745_s_at	4	6.865	0.24	7	7.268	0.64	40	u	0.114154
			218791_s_at	4	6.256	0.41	7	6.201	0.63	40	d	0.827478
			1559658_at	0	2.432	0.21	7	2.599	0.53	40	u	0.426088

CRISPLD1	4	cysteine-rich secretory protein LCCL domain containing 1	223475_at	4	9.033	0.37	7	6.596	2.7	40	d	0.000003
TBC1D20	4	TBC1 domain family, member 20	226409_at	4	7.89	0.21	7	7.416	0.53	40	d	0.027318
			226664_at	1	5.463	0.29	7	5.647	0.45	40	u	0.309385
SEPT9	4	septin 9	208657_s_at	4	8.274	0.22	7	8.705	0.92	40	u	0.016238
			41220_at	4	11.22	0.25	7	11.44	0.74	40	u	0.17703
			207425_s_at	4	5.39	0.89	7	6.431	1.65	40	u	0.118737
			1559025_at	0	3.404	0.51	7	3.392	0.95	40	d	0.973128
PAK3	4	p21 (CDKN1A)-activated kinase 3	214607_at	4	6.306	0.8	7	4.755	1.3	40	d	0.004456
C1orf52	4	chromosome 1 open reading frame 52	239038_at	4	4.865	0.22	7	4.113	0.66	40	d	0.00001
			228135_at	0	9.116	0.19	7	8.836	0.61	40	d	0.031818
TSNAX	4	translin-associated factor X	203983_at	4	9.068	0.23	7	9.838	0.58	40	u	0.001487
CCBL2	4	cysteine conjugate-beta lyase 2	227748_at	4	7.175	0.34	7	6.86	0.86	40	d	0.354946
			209472_at	0	9.221	0.57	7	8.731	0.64	40	d	0.068289
TMED5	4	transmembrane emp24 protein transport domain containing 5	202194_at	4	9.787	0.37	7	9.837	0.85	40	u	0.881079
			239283_at	0	4.773	0.63	7	4.649	0.66	40	d	0.653998
			1558814_s_at	0	2.21	0.23	7	2.251	0.23	40	u	0.666912
			242263_at	0	6.309	0.4	7	6.261	1.02	40	d	0.902682
			1558813_at	0	1.89	0.02	7	1.928	0.03	40	u	0.005659
			202195_s_at	0	6.494	0.57	7	7.343	1.14	40	u	0.066495
PCDHGB2	4	protocadherin gamma subfamily B, 2	no probeset available									
PPP1R10	4	protein phosphatase 1, regulatory (inhibitor) subunit 10	201703_s_at	0	8.29	0.25	7	7.938	0.76	40	d	0.03609
			201702_s_at	0	6.163	0.33	7	6.706	0.91	40	u	0.011285
PTX3	4	pentraxin-related gene, rapidly induced by IL-1 beta	206157_at	2	6.085	0.75	7	5.745	2.46	40	d	0.502114
PCDHGA12	4	protocadherin gamma subfamily A, 12	no probeset available									
PPP1R12C	4	protein phosphatase 1, regulatory (inhibitor) subunit 12C	223152_at	4	7.797	0.15	7	7.214	0.47	40	d	0.000002
NUMBL	4	numb homolog (Drosophila)-like	224059_s_at	4	4.435	0.47	7	4.124	0.64	40	d	0.234927
			242195_x_at	0	8.817	0.29	7	8.77	0.63	40	d	0.850989
H2AFX	4	H2A histone family, member X	213344_s_at	4	3.917	0.64	7	4.859	0.72	40	u	0.002595
			205436_s_at	4	8.213	0.32	7	9.526	0.84	40	u	0.000229
			212524_x_at	4	2.088	0.16	7	2.125	0.19	40	u	0.638577
			212525_s_at	4	5.132	0.27	7	5.877	0.95	40	u	0.000367
GNB2	4	guanine nucleotide binding protein (G protein), beta polypeptide 2	200852_x_at	4	10.12	0.19	7	10.08	0.81	40	d	0.774802
MAP4K5	4	mitogen-activated protein kinase kinase kinase kinase 5	203552_at	4	8.075	0.32	7	7.977	0.6	40	d	0.679043
			203553_s_at	0	7.692	0.3	7	7.782	0.77	40	u	0.766529
			211081_s_at	0	4.589	0.46	7	5.282	0.76	40	u	0.026546
CHODL	4	chondrolectin	219867_at	4	2.959	0.36	7	4.247	1.57	40	u	0.000072
CTGF	4	connective tissue growth factor	209101_at	2	11.26	0.35	7	10.14	1.16	40	d	0.000041
USP37	4	ubiquitin specific peptidase 37	226730_s_at	4	5.8	0.33	7	5.909	0.66	40	u	0.679917
			226729_at	4	6.746	0.24	7	6.746	0.42	40	d	0.997976
			232033_at	2	5.61	0.55	7	6.497	0.68	40	u	0.002382
TMEM32	4	transmembrane protein 32	225125_at	4	9.485	0.16	7	9.041	0.97	40	d	0.012307
SUV420H2	4	suppressor of variegation 4-20 homolog 2 (Drosophila)	224431_s_at	4	7.145	0.24	7	7.251	0.44	40	u	0.551398
			1570058_at	0	2.81	0.08	7	2.977	0.29	40	u	0.005884
TNFAIP1	4	tumor necrosis factor, alpha-induced protein 1 (endothelial)	201208_s_at	4	6.856	0.21	7	7.163	0.62	40	u	0.028044
			201207_at	4	9.053	0.39	7	8.355	0.66	40	d	0.011201
XPR1	4	xenotropic and polytropic retrovirus receptor	222581_at	4	8.465	0.29	7	8.652	0.56	40	u	0.406584
THAP2	4	THAP domain containing, apoptosis associated protein 2	223588_at	4	2.515	0.11	7	2.891	0.59	40	u	0.000851
			230380_at	0	3.188	0.37	7	3.643	0.73	40	u	0.122492
RTN4	4	reticulon 4	211509_s_at	4	12.86	0.19	7	12.67	0.5	40	d	0.330795
			214629_x_at	1	12	0.2	7	11.81	0.46	40	d	0.296821
			210968_s_at	0	12.43	0.2	7	12.18	0.66	40	d	0.066579
			1556049_at	0	6.989	0.59	7	7.046	0.84	40	u	0.86885
COL3A1	4	collagen, type III, alpha 1 (Ehlers-Danlos syndrome type IV, autosomal dominant)	201852_x_at	4	12.27	0.91	7	12.43	1.27	40	u	0.763797
			211161_s_at	4	11.27	0.94	7	11.76	1.31	40	u	0.36052
			215076_s_at	0	12.61	0.91	7	13.1	1.17	40	u	0.314452
			232458_at	0	7.068	0.85	7	7.175	2.21	40	u	0.902567
UBE2NL	4	ubiquitin-conjugating enzyme E2N-like	217393_x_at	4	8.299	0.23	7	8.664	0.41	40	u	0.029197
FBXO21	4	F-box protein 21	212229_s_at	4	8.099	0.31	7	8.191	0.68	40	u	0.733659
			212231_at	4	9.094	0.32	7	8.798	0.54	40	d	0.17324

			214891_at	4	2.663	0.37	7	2.523	0.34	40	d	0.33507
DEDD	4	death effector domain containing	215158_s_at	2	8.095	0.18	7	8.559	0.53	40	u	0.00035
			211255_x_at	0	6.359	0.36	7	6.41	1.14	40	u	0.831824
			202480_s_at	0	6.779	0.2	7	6.656	0.79	40	d	0.416408
PTPN1	4	protein tyrosine phosphatase, non-receptor type 1	202716_at	4	6.771	0.52	7	6.984	0.87	40	u	0.54104
			239526_x_at	0	2.997	0.08	7	3.046	0.11	40	u	0.25147
			217686_at	0	2.571	0.05	7	2.693	0.23	40	u	0.00442
			217689_at	0	2.935	0.26	7	3.056	0.4	40	u	0.4609
VPS37C	4	vacuolar protein sorting 37 homolog C (S. cerevisiae)	219053_s_at	4	9.223	0.38	7	8.958	0.74	40	d	0.372895
			1560059_at	4	4.727	0.38	7	4.616	0.46	40	d	0.557255
			1560060_s_at	4	9.042	0.47	7	8.873	0.8	40	d	0.599784
ATP6V1B2	4	ATPase, H+ transporting, lysosomal 56/58kDa, V1 subunit B2	201089_at	3	9.506	0.24	7	9.473	0.72	40	d	0.829603
STX1A	4	syntaxin 1A (brain)	204729_s_at	4	3.941	0.55	7	4.772	1.1	40	u	0.061198
SH3BP5L	4	SH3-binding domain protein 5-like	223265_at	4	6.308	0.28	7	6.59	0.62	40	u	0.251216
SDCBP	4	syndecan binding protein (syntenin)	200958_s_at	4	11.42	0.37	7	11.2	0.87	40	d	0.524122
SNPH	4	syntaphilin	205104_at	4	6.599	0.39	7	6.181	0.7	40	d	0.138982
			215917_at	0	3.623	0.22	7	3.889	0.53	40	u	0.206097
AKAP1	4	A kinase (PRKA) anchor protein 1	201675_at	4	9.526	0.31	7	9.432	0.82	40	d	0.771582
			201674_s_at	2	9.488	0.28	7	9.292	0.96	40	d	0.315968
			210625_s_at	0	7.914	0.39	7	7.641	1.37	40	d	0.322222
			210626_at	0	3.044	0.25	7	3.296	0.56	40	u	0.255976
SEC24C	4	SEC24 related gene family, member C (S. cerevisiae)	202361_at	4	9.344	0.2	7	8.881	0.7	40	d	0.002349
SLITRK5	4	SLIT and NTRK-like family, member 5	214930_at	4	3.179	0.47	7	3.75	1.35	40	u	0.059906
HAND1	4	heart and neural crest derivatives expressed 1	220138_at	4	2.945	0.25	7	3.053	0.4	40	u	0.502727
FOS	4	v-fos FBJ murine osteosarcoma viral oncogene homolog	209189_at	3	11.81	1.36	7	8.553	1.62	40	d	0.000013
MYCBP2	4	MYC binding protein 2	201960_s_at	4	9.191	0.17	7	8.297	0.99	40	d	0.000005
			201959_s_at	4	9.82	0.16	7	8.793	0.96	40	d	0
			1557370_s_at	0	8.273	0.17	7	7.556	0.92	40	d	0.000065
BRP44L	4	brain protein 44-like	218024_at	4	8.234	0.35	7	7.905	0.73	40	d	0.26054
FOXL2	4	forkhead box L2	220102_at	4	2.72	0.21	7	2.856	0.43	40	u	0.427599
DBC1	4	deleted in bladder cancer 1	205818_at	4	5.596	0.83	7	3.317	0.87	40	d	0
			1555457_at	0	2.666	0.04	7	2.799	0.33	40	u	0.02171
MARCH6	4	membrane-associated ring finger (C3HC4) 6	201736_s_at	4	9.411	0.41	7	9.76	0.76	40	u	0.254592
			201737_s_at	4	9.532	0.38	7	9.454	0.8	40	d	0.805014
			215512_at	0	5.05	0.72	7	5.199	1.27	40	u	0.769481
GRM5	4	glutamate receptor, metabotropic 5	207235_s_at	4	2.315	0.05	7	2.392	0.16	40	u	0.024932
			214217_at	0	2.645	0.04	7	2.812	0.24	40	u	0.000225
			1565389_s_at	0	3.765	0.37	7	3.688	0.56	40	d	0.732015
CXCL12	4	chemokine (C-X-C motif) ligand 12 (stromal cell-derived factor 1)	203666_at	4	8.209	0.76	7	6.545	0.97	40	d	0.000114
			209687_at	0	12.48	0.63	7	8.53	1.48	40	d	0
DIAPH1	4	diaphanous homolog 1 (Drosophila)	213514_s_at	4	7.616	0.1	7	7.548	0.57	40	d	0.499395
			209190_s_at	4	8.198	0.22	7	7.954	0.72	40	d	0.108559
			215541_s_at	0	6.07	0.34	7	6.874	0.75	40	u	0.008716
			1560080_at	0	2.962	0.19	7	3.193	0.38	40	u	0.12592
STRBP	4	spermatid perinuclear RNA binding protein	223245_at	4	6.703	0.47	7	7.608	0.69	40	u	0.002133
			223246_s_at	1	5.381	0.44	7	6.142	0.63	40	u	0.004199
			233252_s_at	0	7.475	0.35	7	8.487	0.59	40	u	0.000085
			233251_at	0	4.061	0.33	7	4.274	0.72	40	u	0.455836
			229513_at	0	7.703	0.31	7	7.086	1.25	40	d	0.012682
ABHD3	4	abhydrolase domain containing 3	213017_at	4	7.396	0.41	7	7.544	0.64	40	u	0.565613
KIAA1409	4	KIAA1409	229550_at	3	2.061	0.05	7	2.175	0.44	40	u	0.124856
TMEM24	4	transmembrane protein 24	204758_s_at	0	2.507	0.15	7	2.532	0.15	40	u	0.692235
			204757_s_at	0	5.631	0.17	7	5.872	0.54	40	u	0.036296
PAX8	4	paired box 8	209552_at	4	4.873	0.45	7	5.031	0.71	40	u	0.579337
			121_at	4	6.795	0.18	7	7.146	0.36	40	u	0.018391
			214528_s_at	0	2.26	0.05	7	2.318	0.07	40	u	0.051676
			221990_at	0	2.665	0.39	7	2.711	0.38	40	u	0.76977
			207923_x_at	0	2.405	0.19	7	2.372	0.13	40	d	0.570807
			207921_x_at	0	2.286	0.13	7	2.31	0.05	40	u	0.675698
			207924_x_at	0	2.279	0.05	7	2.295	0.04	40	u	0.383942

			213917_at	0	3.326	0.33	7	3.477	0.54	40	u	0.486368
OTUD5	4	OTU domain containing 5	1555426_a_at	4	7.075	0.39	7	6.482	0.95	40	d	0.118537
			224745_x_at	4	9.114	0.16	7	8.987	0.34	40	d	0.349124
			233933_s_at	3	7.887	0.22	7	7.787	0.54	40	d	0.63839
KHSRP	4	KH-type splicing regulatory protein (FUSE binding protein 2)	204372_s_at	4	8.34	0.3	7	8.51	0.76	40	u	0.567271
			204371_s_at	4	5.057	0.17	7	5.253	0.65	40	u	0.123515
			227555_s_at	1	3.674	0.46	7	3.906	0.62	40	u	0.357962
C21orf91	4	chromosome 21 open reading frame 91	226109_at	4	8.301	0.31	7	8.418	1.07	40	u	0.586595
			220941_s_at	0	3.477	0.36	7	5.072	1.65	40	u	0.000005
MLLT10	4	myeloid/lymphoid or mixed-lineage leukemia (trithorax homolog, Drosophila); translocated to, 10	225992_at	4	7.798	0.21	7	7.493	0.77	40	d	0.052107
			205408_at	2	6.726	0.46	7	6.691	1.14	40	d	0.938283
			230122_at	0	5.355	0.37	7	6.241	1.08	40	u	0.000685
			238257_at	0	3.081	0.18	7	3.512	0.68	40	u	0.002159
			1563321_s_at	0	6.458	0.23	7	7.096	0.85	40	u	0.000464
			216506_x_at	0	5.095	0.31	7	5.577	0.62	40	u	0.054248
			216480_x_at	0	2.279	0.08	7	2.632	0.52	40	u	0.000295
			216509_x_at	0	3.452	0.37	7	3.376	0.43	40	d	0.669682
			216503_s_at	0	4.541	0.68	7	5.133	1.21	40	u	0.222996
HMGCLL1	4	3-hydroxymethyl-3-methylglutaryl-Coenzyme A lyase-like 1	232305_at	4	3.896	0.44	7	3.258	0.49	40	d	0.002669
			233576_at	0	3.025	0.25	7	3.119	0.22	40	u	0.323367
			1554872_a_at	0	3.121	0.68	7	2.748	0.38	40	d	0.238682
CECR6	4	cat eye syndrome chromosome region, candidate 6	224393_s_at	4	1.994	0.29	7	1.964	0.32	40	d	0.825446
ARHGEF6	4	Rac/Cdc42 guanine nucleotide exchange factor (GEF) 6	209539_at	4	9.328	0.31	7	8.018	0.95	40	d	0
GLRB	4	glycine receptor, beta	205280_at	3	4.576	0.98	7	3.878	1.29	40	d	0.187325
			244680_at	0	3.167	0.57	7	3.012	0.9	40	d	0.668111
			205279_s_at	0	5.59	1	7	4.233	1.4	40	d	0.020663
ARHGAP6	4	Rho GTPase activating protein 6	206167_s_at	4	5.197	0.79	7	3.632	0.87	40	d	0.000073
			208085_s_at	0	2.218	0.03	7	2.285	0.15	40	u	0.014253
ZFR	4	zinc finger RNA binding protein	201857_at	4	10.04	0.3	7	10.08	0.53	40	u	0.833455
			201856_s_at	2	7.455	0.43	7	7.912	0.62	40	u	0.073152
			33148_at	0	4.992	0.26	7	6.38	0.75	40	u	0
			213286_at	0	6.002	0.4	7	7.465	0.81	40	u	0.000038
FLOT1	4	flotillin 1	208749_x_at	0	10.65	0.21	7	10.67	0.52	40	u	0.916542
			210142_x_at	0	10.39	0.28	7	10.4	0.55	40	u	0.97072
			213819_s_at	0	2.178	0.26	7	2.254	0.29	40	u	0.524024
			208748_s_at	0	6.32	0.45	7	6.682	0.86	40	u	0.292147
SHROOM2	4	shroom family member 2	204967_at	4	4.793	1.07	7	5.301	1.5	40	u	0.405355
PABPC1	4	poly(A) binding protein, cytoplasmic 1	215157_x_at	0	13.17	0.21	7	13.47	0.48	40	u	0.124051
			1560770_at	0	2.718	0.12	7	2.824	0.2	40	u	0.181916
			215823_x_at	0	12.36	0.25	7	12.88	0.65	40	u	0.044412
TMEFF1	4	transmembrane protein with EGF-like and two follistatin-like domains 1	205122_at	4	3.634	0.52	7	5.134	1.37	40	u	0.007349
			205123_s_at	1	2.553	0.06	7	3.443	1.16	40	u	0.000027
CAMKK2	4	calcium/calmodulin-dependent protein kinase kinase 2, beta	212252_at	4	8.371	0.19	7	8.088	0.6	40	d	0.028856
			207359_at	0	2.513	0.32	7	2.293	0.09	40	d	0.143706
			210787_s_at	0	4.338	0.48	7	4.857	1.12	40	u	0.244247
			213812_s_at	0	7.853	0.63	7	8.434	0.68	40	u	0.043854
ANKRD27	4	ankyrin repeat domain 27 (VPS9 domain)	221522_at	4	8.201	0.25	7	8.561	0.71	40	u	0.025652
RNF19A	4	ring finger protein 19A	223085_at	4	9.186	0.45	7	9.876	0.95	40	u	0.072831
			220483_s_at	0	7.212	0.22	7	8.306	1.08	40	u	0.000001
KCTD12	4	potassium channel tetramerisation domain containing 12	212192_at	4	10.49	0.39	7	8.843	0.97	40	d	0.000083
			212188_at	4	10.33	0.3	7	9.029	0.79	40	d	0.000112
MYOHD1	4	myosin head domain containing 1	225947_at	4	7.183	0.13	7	7.696	0.52	40	u	0.000009
			219320_at	0	4.884	0.31	7	5.65	0.71	40	u	0.00905
			236022_at	0	6.849	0.31	7	6.837	0.57	40	d	0.954991
PRNPIP	4	prion protein interacting protein	208973_at	4	6.911	0.23	7	6.842	0.83	40	d	0.675916
SPRYD3	4	SPRY domain containing 3	225134_at	4	8.499	0.3	7	7.75	0.61	40	d	0.003314
PRRT2	4	proline-rich transmembrane protein 2	227192_at	4	5.447	0.35	7	5.645	1.24	40	u	0.421392
PCDHGA6	4	protocadherin gamma subfamily A, 6	no probeset available									
TMEM16A	4	transmembrane protein 16A	218804_at	2	9.519	0.72	7	6.242	1.62	40	d	0.000006
			1555269_a_at	2	8.621	0.72	7	5.894	1.37	40	d	0.000009
VAV3	4	vav 3 guanine nucleotide exchange factor	218807_at	4	9.922	0.8	7	8.594	1.78	40	d	0.06396
			218806_s_at	2	8.75	0.86	7	7.424	2.04	40	d	0.104984

			224221_s_at	0	6.563	1.32	7	6.134	1.92	40	d	0.580835
FOSL1	4	FOS-like antigen 1	204420_at	4	3.896	0.9	7	4.687	0.94	40	u	0.048432
PCDHGB1	4	protocadherin gamma subfamily B, 1	no probeset available									
DPYSL3	4	dihydropyrimidinase-like 3	201430_s_at	4	6.066	0.82	7	6.395	1.12	40	u	0.47237
			201431_s_at	4	8.389	0.84	7	7.36	1.06	40	d	0.021591
TTC7B	4	tetratricopeptide repeat domain 7B	226152_at	4	4.971	0.53	7	4.948	0.93	40	d	0.951887
WASF2	4	WAS protein family, member 2	224562_at	4	10.93	0.32	7	9.888	0.61	40	d	0.000078
			224563_at	4	4.78	0.63	7	5.866	0.97	40	u	0.007474
ELAVL3	4	ELAV (embryonic lethal, abnormal vision, Drosophila)-like 3 (Hu antigen C)	227612_at	4	2.232	0.02	7	2.405	0.32	40	u	0.001833
			206338_at	4	6.881	0.25	7	6.573	0.46	40	d	0.1007
			1555602_a_at	0	1.93	0.13	7	1.976	0.13	40	u	0.395823
PNKD	4	paroxysmal nonkinesigenic dyskinesia	233177_s_at	4	4.413	0.46	7	6.3	1.26	40	u	0.000385
			1555713_at	0	2.295	0.04	7	2.417	0.25	40	u	0.007195
			225298_at	0	8.553	0.21	7	8.206	0.62	40	d	0.013811
ITPR3	4	inositol 1,4,5-triphosphate receptor, type 3	201187_s_at	4	5.012	0.37	7	5.002	0.45	40	d	0.954692
			201189_s_at	4	8.566	0.38	7	8.662	0.94	40	u	0.796185
			201188_s_at	0	7.303	0.41	7	7.32	0.98	40	u	0.964188
WASF1	4	WAS protein family, member 1	204165_at	4	6.418	0.35	7	6.006	1.09	40	d	0.079355
GLTP	4	glycolipid transfer protein	226177_at	4	9.609	0.26	7	9.308	0.63	40	d	0.226955
			219267_at	2	7.044	0.38	7	7.035	0.69	40	d	0.9724
HERPUD2	4	HERPUD family member 2	222751_at	4	8.071	0.31	7	7.865	0.61	40	d	0.398765
			1558699_a_at	1	6.664	0.35	7	6.623	0.53	40	d	0.848164
			1552628_a_at	1	8.454	0.22	7	8.541	0.47	40	u	0.639588
			236170_x_at	0	5.063	0.35	7	5.318	0.72	40	u	0.376014
RBJ	4	rab and DnaJ domain containing	227859_at	4	5.707	0.4	7	5.043	0.99	40	d	0.093691
			223504_at	2	5.98	0.45	7	5.542	0.87	40	d	0.208505
			233368_s_at	0	2.596	0.06	7	2.788	0.3	40	u	0.0008
			223505_s_at	0	2.737	0.31	7	3.159	0.53	40	u	0.049294
CACNA2D1	4	calcium channel, voltage-dependent, alpha 2/delta subunit 1	207050_at	4	1.939	0.04	7	2.073	0.31	40	u	0.014143
TMCC3	4	transmembrane and coiled-coil domain family 3	226489_at	4	5.747	1.12	7	4.887	1.02	40	d	0.053476
FBXO42	4	F-box protein 42	221813_at	4	6.833	0.31	7	6.851	0.31	40	u	0.895938
			221812_at	4	6.517	0.3	7	6.071	0.6	40	d	0.066029
			1559566_at	0	2.813	0.11	7	3.017	0.37	40	u	0.011381
			47773_at	0	6.492	0.25	7	5.807	0.61	40	d	0.00631
PPP1R13B	4	protein phosphatase 1, regulatory (inhibitor) subunit 13B	216347_s_at	4	7.133	0.25	7	6.272	0.8	40	d	0.000014
ARL2	4	ADP-ribosylation factor-like 2	202564_x_at	4	8.341	0.27	7	7.759	0.77	40	d	0.001734
FGFR1	4	fibroblast growth factor receptor 1 (fms-related tyrosine kinase 2, Pfeiffer syndrome)	211535_s_at	2	10.16	0.28	7	8.497	1.96	40	d	0.00001
			207822_at	0	5.599	0.46	7	5.242	1.67	40	d	0.282327
			226705_at	0	8.827	0.3	7	7.092	2.04	40	d	0.00001
			210973_s_at	0	8.729	0.55	7	7.259	2.03	40	d	0.000715
			222164_at	0	7.359	0.33	7	7.196	1.79	40	d	0.609269
			215404_x_at	0	5.858	0.4	7	5.818	0.87	40	d	0.90915
			207937_x_at	0	6.394	0.34	7	6.582	1.38	40	u	0.475692
SPECC1L	4	SPECC1-like	212480_at	4	7.973	0.32	7	7.457	0.53	40	d	0.017649
C10orf12	4	chromosome 10 open reading frame 12	216393_at	4	2.555	0.22	7	2.588	0.22	40	u	0.72153
			219601_s_at	4	2.62	0.1	7	2.7	0.24	40	u	0.408443
			226892_at	0	7.024	0.41	7	6.711	0.87	40	d	0.367467
TSC22D1	4	TSC22 domain family, member 1	215111_s_at	4	12.41	0.15	7	11.42	0.81	40	d	0
			235315_at	0	4.66	0.8	7	4.891	1.46	40	u	0.690641
			243133_at	0	2.614	0.4	7	2.691	0.36	40	u	0.618272
RHOT1	4	ras homolog gene family, member T1	218323_at	4	8.771	0.26	7	8.684	0.58	40	d	0.704101
			222148_s_at	4	8.633	0.26	7	8.228	0.67	40	d	0.130561
			224044_at	0	3.253	0.36	7	3.418	0.55	40	u	0.459105
EPS8	4	epidermal growth factor receptor pathway substrate 8	202609_at	4	9.632	0.26	7	8.797	0.96	40	d	0.00008
KPNA2	4	karyopherin alpha 2 (RAG cohort 1, importin alpha 1)	201088_at	8	8.482	0.27	7	10.85	0.79	40	u	0
			211762_s_at	0	8.703	0.27	7	10.49	1.07	40	u	0
RHOC	4	ras homolog gene family, member C	200885_at	4	10.42	0.23	7	9.863	0.87	40	d	0.002048
			235742_at	0	3.372	0.68	7	3.389	0.64	40	u	0.951175
PRR16	4	proline rich 16	220014_at	4	3.112	0.32	7	3.305	0.63	40	u	0.439181
			1554867_a_at	3	3.123	0.56	7	3.48	0.81	40	u	0.280074

MGAT4B	4	mannosyl (alpha-1,3-)-glycoprotein beta-1,4-N-acetylglucosaminyltransferase, isozyme B	224598_at	4	8.711	0.21	7	9.001	0.77	40	u	0.062271
			220189_s_at	0	8.302	0.2	7	8.27	0.85	40	d	0.840084
PLEC1	4	plectin 1, intermediate filament binding protein 500kDa	201373_at	4	8.257	0.44	7	7.529	0.82	40	d	0.030459
			216971_s_at	0	6.881	0.86	7	5.534	1.72	40	d	0.053212
RIN2	4	Ras and Rab interactor 2	209684_at	4	10.17	0.34	7	9.829	0.96	40	d	0.114986
			233811_at	0	3.623	0.51	7	3.634	0.37	40	u	0.947848
PTK2	4	PTK2 protein tyrosine kinase 2	208820_at	4	9.13	0.15	7	9.799	0.69	40	u	0.000004
			207821_s_at	1	8	0.17	7	8.661	0.88	40	u	0.000129
			241453_at	0	2.899	0.53	7	4.436	1.42	40	u	0.00813
			1559529_at	0	6.507	0.58	7	6.404	1.02	40	d	0.80058
			239121_at	0	6.131	0.42	7	6.056	0.77	40	d	0.805905
YWHAQ	4	tyrosine 3-monooxygenase/tryptophan 5-monooxygenase activation protein, theta polypeptide	212426_s_at	4	11	0.16	7	11.63	0.54	40	u	0.000002
			200693_at	4	11.58	0.2	7	12.09	0.66	40	u	0.000754
			213699_s_at	4	13.13	0.15	7	13.75	0.44	40	u	0
SCP2	4	sterol carrier protein 2	201339_s_at	1	10.99	0.89	7	10.29	0.81	40	d	0.046801
			211733_x_at	1	11.02	0.73	7	10.5	0.5	40	d	0.02597
SBNO2	4	strawberry notch homolog 2 (Drosophila)	204166_at	4	7.724	0.35	7	7.476	0.7	40	d	0.376165
			215760_s_at	0	7.129	0.34	7	7.092	0.82	40	d	0.909336
ATP1B4	4	ATPase, (Na+)/K+ transporting, beta 4 polypeptide	220556_at	4	2.956	0.2	7	3.058	0.41	40	u	0.535509
RAB35	4	RAB35, member RAS oncogene family	225620_at	4	6.213	0.54	7	6.753	0.6	40	u	0.034972
			221819_at	4	7.025	0.36	7	7.099	0.48	40	u	0.700723
			205461_at	0	2.96	0.42	7	3.482	1.06	40	u	0.214827
ERLIN2	4	ER lipid raft associated 2	221542_s_at	4	8.053	0.3	7	7.343	1.58	40	d	0.015449
			221543_s_at	4	9.336	0.26	7	8.691	1.49	40	d	0.017703
			238615_at	0	3.655	0.36	7	4.196	1.44	40	u	0.055535
TMEM117	4	transmembrane protein 117	223594_at	4	5.185	0.4	7	5.13	0.7	40	d	0.842827
PPP2R5A	4	protein phosphatase 2, regulatory subunit B', alpha isoform	202186_x_at	4	2.195	0.18	7	2.292	0.29	40	u	0.400154
			202187_s_at	4	9.068	0.32	7	9.199	1.04	40	u	0.537258
POGZ	4	pogo transposable element with ZNF domain	212153_at	4	9.224	0.42	7	8.649	0.81	40	d	0.080199
			215281_x_at	0	5.826	0.55	7	5.978	0.51	40	u	0.488237
PCDHGA1	4	protocadherin gamma subfamily A, 1	215836_s_at	4	10.23	0.26	7	8.188	1.11	40	d	0
			205717_x_at	4	9.712	0.28	7	7.854	1.02	40	d	0
			209079_x_at	4	9.956	0.25	7	8.268	0.98	40	d	0
			211066_x_at	4	10.17	0.36	7	8.351	1.08	40	d	0
			211880_x_at	0	2.234	0.11	7	2.342	0.61	40	u	0.322162
SLC38A4	4	solute carrier family 38, member 4	220786_s_at	4	2.885	0.58	7	2.819	0.13	40	d	0.790018
			1555345_at	0	2.488	0.04	7	2.587	0.14	40	u	0.001024
CSDA	4	cold shock domain protein A	228634_s_at	0	2.665	0.38	7	2.876	0.65	40	u	0.422106
			213319_s_at	0	4.057	0.49	7	3.67	0.52	40	d	0.077915
			201161_s_at	0	9.387	0.37	7	8.484	1.51	40	d	0.003212
			201160_s_at	0	11.41	0.17	7	10.7	1.28	40	d	0.001958
UBQLN2	4	ubiquilin 2	215884_s_at	4	8.584	0.36	7	8.737	0.52	40	u	0.466498
PCDHGC5	4	protocadherin gamma subfamily C, 5	224537_at	0	2.079	0.02	7	2.13	0.06	40	u	0.000822
			224536_s_at	0	1.975	0.02	7	2.012	0.03	40	u	0.004309
SNN	4	stannin	218032_at	4	8.495	0.32	7	8.246	0.68	40	d	0.355997
			218033_s_at	3	3.888	0.34	7	4.061	0.88	40	u	0.617798
SSRP1	4	structure specific recognition protein 1	200956_s_at	4	8.393	0.27	7	9.044	1.06	40	u	0.002616
			200957_s_at	4	9.05	0.22	7	9.243	0.93	40	u	0.272268
RBM9	4	RNA binding motif protein 9	216215_s_at	0	9.375	0.18	7	8.177	0.58	40	d	0
			213898_at	0	2.383	0.21	7	2.586	0.31	40	u	0.110136
			212104_s_at	0	9.28	0.33	7	8.468	0.58	40	d	0.001053
			213901_x_at	0	7.652	0.34	7	6.786	1.18	40	d	0.000809
VIL2	4	villin 2 (ezrin)	208621_s_at	4	6.915	0.98	7	7.814	1.29	40	u	0.093148
			208622_s_at	4	9.651	0.65	7	9.466	1.02	40	d	0.653268
			208623_s_at	4	9.899	0.49	7	9.903	0.77	40	u	0.991187
			217234_s_at	0	7.908	0.82	7	8.595	1.18	40	u	0.153606
			217230_at	0	3.153	0.64	7	3.471	0.74	40	u	0.299292
HNRNPA0	4	heterogeneous nuclear ribonucleoprotein A0	201054_at	4	10.58	0.35	7	9.885	0.73	40	d	0.019902
			201055_s_at	1	8.549	0.19	7	8.246	0.74	40	d	0.040779
YPEL5	4	yippee-like 5 (Drosophila)	222408_s_at	4	10.42	0.36	7	9.961	0.77	40	d	0.13769
			217783_s_at	4	10.81	0.31	7	9.999	0.69	40	d	0.004486
C3orf59	4	chromosome 3 open reading frame 59	227599_at	4	7.433	0.53	7	7.102	0.96	40	d	0.391456
CHST10	4	carbohydrate sulfotransferase 10	204065_at	4	5.818	0.44	7	6.009	0.62	40	u	0.450609

LMAN2L	4	lectin, mannose-binding 2-like	221274_s_at	4	7.817	0.29	7	7.38	0.65	40	d	0.094444
SCN4B	4	sodium channel, voltage-gated, type IV, beta	236359_at	4	6.737	0.65	7	3.402	0.62	40	d	0
SLC39A9	4	solute carrier family 39 (zinc transporter), member 9	222445_at	2	9.211	0.21	7	8.75	0.62	40	d	0.001642
			217859_s_at	2	4.89	0.37	7	5.281	1	40	u	0.319838
			216011_at	0	2.081	0.05	7	2.153	0.1	40	u	0.086469
			216009_at	0	2.303	0.04	7	2.488	0.27	40	u	0.000257
EPB49	4	erythrocyte membrane protein band 4.9 (dematin)	204505_s_at	4	6.729	0.32	7	5.98	0.95	40	d	0.000862
FAM19A1	4	family with sequence similarity 19 (chemokine (C-C motif)-like), member A1	230923_at	4	2.709	0.05	7	2.922	0.69	40	u	0.066513
SH3BGRL2	4	SH3 domain binding glutamic acid-rich protein like 2	225354_s_at	4	8.03	0.35	7	5.086	1.57	40	d	0
			244635_s_at	0	2.944	0.1	7	3.172	0.26	40	u	0.031319
DNMT3A	4	DNA (cytosine-5-)-methyltransferase 3 alpha	222640_at	4	6.434	0.44	7	7.897	0.72	40	u	0.000006
			218457_s_at	0	3.677	0.71	7	4.379	1.06	40	u	0.106963
			244428_at	0	4.199	0.54	7	3.607	0.8	40	d	0.07294
SFRS10	4	splicing factor, arginine/serine-rich 10 (transformer 2 homolog, Drosophila)	200893_at	4	11.07	0.19	7	10.93	0.39	40	d	0.369479
			200892_s_at	3	9.927	0.24	7	9.925	0.71	40	d	0.99032
			210180_s_at	0	5.97	0.44	7	5.858	0.66	40	d	0.672389
MARCH3	4	membrane-associated ring finger (C3HC4) 3	213256_at	4	4.544	0.3	7	5.786	1.59	40	u	0.000072
FBXL10	4	F-box and leucine-rich repeat protein 10	226215_s_at	4	7.956	0.44	7	8.44	0.64	40	u	0.065648
SURF4	4	surfeit 4	222977_at	4	8.903	0.23	7	8.998	0.74	40	u	0.539448
			222978_at	4	10.62	0.08	7	10.69	0.54	40	u	0.473113
			222979_s_at	0	8.777	0.41	7	9.282	1.09	40	u	0.244796
ZKSCAN1	4	zinc finger with KRAB and SCAN domains 1	214670_at	4	8.526	0.45	7	8.3	0.95	40	d	0.548575
			1557953_at	4	9.151	0.5	7	8.494	0.8	40	d	0.045346
			214900_at	0	5.541	0.45	7	6.184	0.91	40	u	0.081343
GAP43	4	growth associated protein 43	204471_at	4	3.285	0.21	7	3.384	0.44	40	u	0.567724
			216967_at	1	2.134	0.02	7	2.212	0.14	40	u	0.001741
			216963_s_at	1	3.916	0.34	7	4.035	0.32	40	u	0.383587
XYLT2	4	xylosyltransferase II	231550_at	4	6.385	0.64	7	6.085	1.11	40	d	0.500875
			219401_at	4	6.747	0.21	7	6.139	1.02	40	d	0.00203
			1570528_at	0	2.859	0.18	7	3.182	0.57	40	u	0.010098
CHST2	4	carbohydrate (N-acetylglucosamine-6-O) sulfotransferase 2	203921_at	4	6.705	0.63	7	6.621	1.53	40	d	0.888851
UBE2L3	4	ubiquitin-conjugating enzyme E2L 3	200683_s_at	4	8.567	0.2	7	8.967	0.63	40	u	0.004476
			200684_s_at	4	7.26	0.17	7	7.481	0.73	40	u	0.114686
			200682_s_at	4	10.01	0.27	7	10.37	0.64	40	u	0.157854
			200676_s_at	4	6.5	0.2	7	6.264	1.01	40	d	0.195584
PLOD2	4	procollagen-lysine, 2-oxoglutarate 5-dioxygenase 2	202619_s_at	4	6.99	0.68	7	8.591	1.13	40	u	0.000889
			202620_s_at	4	7.327	0.76	7	8.897	1.05	40	u	0.000583
UBXD3	4	UBX domain containing 3	232109_at	4	4.711	0.65	7	4.447	0.76	40	d	0.400776
			238657_at	1	2.903	0.56	7	2.82	0.83	40	d	0.80474
			230092_at	0	5.395	0.47	7	5.339	0.62	40	d	0.822985
MRFAP1	4	Mof4 family associated protein 1	226091_s_at	4	13.06	0.09	7	12.79	0.51	40	d	0.003699
CREM	4	cAMP responsive element modulator	230511_at	4	5.252	0.68	7	4.602	0.59	40	d	0.01403
			209967_s_at	3	6.903	0.58	7	6.894	0.66	40	d	0.974026
			210171_s_at	0	2.47	0.09	7	2.6	0.24	40	u	0.166978
			214508_x_at	0	6.779	0.77	7	6.744	0.98	40	d	0.931332
			228092_at	0	4.732	0.29	7	5.563	1.28	40	u	0.001126
			207630_s_at	0	7.163	0.83	7	7.26	0.92	40	u	0.797387
METAP1	4	methionyl aminopeptidase 1	212673_at	4	9.07	0.22	7	8.619	0.52	40	d	0.033548
SSFA2	4	sperm specific antigen 2	202506_at	3	8.561	0.37	7	8.517	1.03	40	d	0.845923
			236207_at	0	2.412	0.35	7	3.849	1.54	40	u	0.000011
			229744_at	0	5.43	0.62	7	5.397	1.66	40	d	0.95929
NPAS2	4	neuronal PAS domain protein 2	213462_at	4	6.842	0.46	7	5.593	1.1	40	d	0.005769
			39549_at	4	5.48	0.64	7	5.129	0.93	40	d	0.351849
			39548_at	3	4.445	0.96	7	4.146	0.74	40	d	0.362916
			205460_at	0	4.097	0.17	7	4.042	0.5	40	d	0.610067
			205459_s_at	0	3.904	0.44	7	3.362	0.54	40	d	0.017437
DOC2A	4	double C2-like domains, alpha	205744_at	4	3.107	0.76	7	3.071	0.95	40	d	0.925395
CDC27	4	cell division cycle 27 homolog (S. cerevisiae)	217879_at	4	6.57	0.42	7	7.401	0.73	40	u	0.006244
			217878_s_at	4	5.873	0.34	7	7.683	0.64	40	u	0

CDC27	4	cell division cycle 27 homolog (S. cerevisiae)	217881_s_at	0	6.644	0.33	7	6.899	1.11	40	u	0.260297
			214236_at	0	2.933	0.26	7	3.565	0.84	40	u	0.000926
WNT1	4	wingless-type MMTV integration site family, member 1	208570_at	0	1.62	0.02	7	1.676	0.17	40	u	0.050318
H3F3A	4	H3 histone, family 3A	200080_s_at	4	13.28	0.15	7	13.72	0.22	40	u	0.000012
			208755_x_at	4	13.26	0.21	7	13.66	0.36	40	u	0.007131
			211940_x_at	4	13.09	0.22	7	13.52	0.42	40	u	0.011886
			213828_x_at	0	13.35	0.26	7	13.77	0.36	40	u	0.006064
GTF2H1	4	general transcription factor IIH, polypeptide 1, 62kDa	202451_at	4	8.271	0.37	7	7.988	0.79	40	d	0.369798
			202453_s_at	1	7.437	0.45	7	6.94	1.12	40	d	0.263615
PCDHGB7	4	protocadherin gamma subfamily B, 7	1552662_a_at	0	2.149	0.15	7	2.171	0.18	40	u	0.762297
			1552661_at	0	3.158	0.15	7	3.471	0.28	40	u	0.006676
INSM1	4	insulinoma-associated 1	231558_at	4	3.542	0.17	7	3.69	0.3	40	u	0.224186
			206502_s_at	4	2.225	0.06	7	3.503	1.97	40	u	0.000245
HMGN2	4	high-mobility group nucleosomal binding domain 2	208668_x_at	4	12.71	0.22	7	12.49	0.54	40	d	0.283793
YAF2	4	YY1 associated factor 2	244783_at	4	3.399	0.18	7	3.73	0.41	40	u	0.04714
			206238_s_at	0	5.067	0.31	7	5.579	0.92	40	u	0.014516
PGF	4	placental growth factor, vascular endothelial growth factor-related protein	209652_s_at	4	6.488	0.39	7	5.766	0.83	40	d	0.032907
			215179_x_at	0	7.9	0.4	7	7.624	0.62	40	d	0.270172
HNRNPA2B1	4	heterogeneous nuclear ribonucleoprotein A2/B1	205292_s_at	2	12.19	0.22	7	12.39	0.43	40	u	0.237363
			225107_at	0	10.18	0.26	7	9.327	1.32	40	d	0.000835
			244131_at	0	3.664	0.49	7	3.411	0.44	40	d	0.183231
			225932_s_at	0	12.01	0.19	7	11.15	0.9	40	d	0.000004
			235454_at	0	2.234	0.04	7	2.257	0.05	40	u	0.22953
FAM57A	4	family with sequence similarity 57, member A	218898_at	4	5.192	0.56	7	6.057	1.04	40	u	0.040651
ARHGAP1	4	Rho GTPase activating protein 1	202117_at	4	10.57	0.3	7	9.982	0.7	40	d	0.037151
			216689_x_at	1	6.799	0.86	7	6.447	1.05	40	d	0.417634
			217153_at	1	2.74	0.07	7	2.7	0.17	40	d	0.558382
			241419_at	0	4.169	0.6	7	4.278	0.77	40	u	0.729522
SLC38A3	4	solute carrier family 38, member 3	205972_at	2	2.799	0.47	7	2.796	0.68	40	d	0.992449
AKAP12	4	A kinase (PRKA) anchor protein (gravin) 12	210517_s_at	4	8.833	0.92	7	5.844	1.24	40	d	0
			1555395_at	0	2.746	0.31	7	2.656	0.25	40	d	0.414172
			227530_at	0	8.396	1.06	7	5.529	0.75	40	d	0
			227529_s_at	0	6.499	1.24	7	3.989	0.44	40	d	0.002734
			231067_s_at	0	3.988	0.95	7	3.149	0.14	40	d	0.072899
ELOVL2	4	elongation of very long chain fatty acids (FEN1/Elo2, SUR4/Elo3, yeast)-like 2	213712_at	4	5.616	1.56	7	5.333	1.89	40	d	0.715873
			220029_at	1	3.142	1.08	7	3.225	1.33	40	u	0.879275
LRP2	4	low density lipoprotein-related protein 2	205710_at	4	7.004	1.08	7	5.498	1.65	40	d	0.027108
			230863_at	0	8.465	0.7	7	5.545	2.16	40	d	0.000001
PCDHGA8	4	protocadherin gamma subfamily A, 8	210368_at	0	4.162	0.23	7	4.203	0.46	40	u	0.820003
KCMF1	4	potassium channel modulatory factor 1	217938_s_at	4	9.818	0.22	7	10.57	0.54	40	u	0.000923
			222471_s_at	4	7.224	0.21	7	7.25	0.84	40	u	0.870103
JMJD2C	4	jumonji domain containing 2C	209984_at	4	6.912	0.42	7	6.291	0.8	40	d	0.055504
			1556493_a_at	0	4.761	0.48	7	4.561	0.64	40	d	0.441702
			214861_at	0	4.619	0.33	7	4.487	0.47	40	d	0.487945
			244385_at	0	3.182	0.22	7	3.681	0.79	40	u	0.003034
SOCS1	4	suppressor of cytokine signaling 1	210001_s_at	4	5.545	0.23	7	6.103	1.25	40	u	0.01516
			210000_s_at	4	2.319	0.03	7	2.373	0.06	40	u	0.021174
			213337_s_at	4	5.524	0.26	7	5.806	0.37	40	u	0.063938
			209999_x_at	4	2.477	0.08	7	2.676	0.58	40	u	0.05004
KBTBD4	4	kelch repeat and BTB (POZ) domain containing 4	218569_s_at	4	6.497	0.46	7	6.266	0.75	40	d	0.44467
			218570_at	4	7.437	0.48	7	7.41	0.76	40	d	0.93005
			223765_s_at	0	5.271	0.4	7	5.269	0.78	40	d	0.994004
VWC2	4	von Willebrand factor C domain containing 2	no probeset available									
NTNG1	4	netrin G1	236088_at	4	2.567	0.15	7	2.574	0.2	40	u	0.933286
			206713_at	0	1.987	0.04	7	2.066	0.07	40	u	0.008027
TFAP2A	4	transcription factor AP-2 alpha (activating enhancer binding protein 2 alpha)	204653_at	4	10.7	0.55	7	9.938	1.13	40	d	0.091442
			204654_s_at	3	8.411	0.82	7	8.244	1.64	40	d	0.798117
			210669_at	0	2.346	0.13	7	2.662	0.57	40	u	0.005113
PTPLB	4	protein tyrosine phosphatase-like (proline instead of catalytic arginine), member b	212640_at	4	9.138	0.37	7	9.576	0.72	40	u	0.129926
			227741_at	1	6.359	0.4	7	7.927	1.1	40	u	0.000001
SLITRK2	4	SLIT and NTRK-like family, member 2	233051_at	0	2.864	0.32	7	3.084	0.36	40	u	0.145476

MLL5	4	myeloid/lymphoid or mixed-lineage leukemia 5 (trithorax homolog, Drosophila)	226100_at	4	8.094	0.35	7	7.527	0.78	40	d	0.072061
			223190_s_at	0	9.604	0.36	7	9.044	0.72	40	d	0.053467
			223189_x_at	0	8.716	0.48	7	8.537	0.59	40	d	0.461405
TMEM108	4	transmembrane protein 108	223524_s_at	4	3.408	0.73	7	3.43	1.54	40	u	0.97161
			223523_at	3	2.998	0.52	7	3.044	1.09	40	u	0.916228
			234818_at	0	2.847	0.26	7	2.951	0.23	40	u	0.291429
			234813_at	0	2.33	0.05	7	2.392	0.19	40	u	0.107501
VPS54	4	vacuolar protein sorting 54 homolog (S. cerevisiae)	218423_x_at	4	8.168	0.15	7	8.433	0.55	40	u	0.018428
			222627_at	4	7.889	0.34	7	8.194	0.67	40	u	0.255673
			233656_s_at	2	8.228	0.14	7	8.414	0.52	40	u	0.078686
PEX5	4	peroxisomal biogenesis factor 5	203244_at	4	8.723	0.23	7	8.443	0.8	40	d	0.085858
			215481_s_at	0	4.911	0.22	7	4.753	0.72	40	d	0.28655
SYPL1	4	synaptophysin-like 1	201260_s_at	4	9.878	0.22	7	9.474	0.74	40	d	0.011294
			201259_s_at	4	9.697	0.39	7	9.231	1.13	40	d	0.06336
MAP3K7	4	mitogen-activated protein kinase kinase kinase 7	206854_s_at	4	8.071	0.31	7	7.99	0.68	40	d	0.76312
			211537_x_at	1	7.959	0.39	7	7.76	0.9	40	d	0.578078
			206853_s_at	0	6.62	0.28	7	6.443	0.75	40	d	0.547369
			211536_x_at	0	7.809	0.38	7	7.831	0.96	40	u	0.953961
SPSB4	4	splA/ryanodine receptor domain and SOCS box containing 4	229929_at	4	2.529	0.15	7	2.676	0.26	40	u	0.167123
SLC7A1	4	solute carrier family 7 (cationic amino acid transporter, y+ system), member 1	212295_s_at	4	8.491	0.37	7	8.441	0.89	40	d	0.88577
			212292_at	4	4.666	0.25	7	4.926	0.67	40	u	0.326928
			212290_at	4	6.067	0.3	7	6.805	0.77	40	u	0.018144
			215979_s_at	4	4.469	0.09	7	4.473	0.27	40	u	0.939491
			206566_at	0	2.414	0.1	7	2.919	1.03	40	u	0.00471
C6orf85	4	chromosome 6 open reading frame 85	223194_s_at	4	7.526	0.42	7	6.07	1.6	40	d	0.000036
			1568719_s_at	0	3.759	0.3	7	3.74	0.31	40	d	0.880666
			233206_at	0	3.165	0.43	7	3.337	0.37	40	u	0.286509
			1568718_at	0	1.36	0.02	7	1.44	0.2	40	u	0.019891
NCOA2	4	nuclear receptor coactivator 2	205732_s_at	4	3.689	0.45	7	4.491	1.25	40	u	0.007143
			205731_s_at	4	5.017	0.52	7	5.64	0.96	40	u	0.109603
			215605_at	0	6.347	0.37	7	6.075	0.87	40	d	0.430015
S100PBP	4	S100P binding protein	222610_s_at	4	6.525	0.18	7	6.907	0.58	40	u	0.003402
			218370_s_at	4	7.427	0.23	7	6.75	0.87	40	d	0.000295
PCDHGB6	4	protocadherin gamma subfamily B, 6	221682_s_at	0	3.011	0.21	7	3.108	0.38	40	u	0.520565
SERF2	4	small EDRK-rich factor 2	224625_x_at	4	12.49	0.2	7	12.56	0.58	40	u	0.555939
			217756_x_at	4	11.69	0.25	7	11.7	0.56	40	u	0.971077
			226692_at	2	7.896	0.23	7	6.794	0.99	40	d	0.000001
MAPK8IP3	4	mitogen-activated protein kinase 8 interacting protein 3	213177_at	4	6.779	0.38	7	6.267	0.93	40	d	0.166344
			213178_s_at	4	7.606	0.3	7	6.936	1.02	40	d	0.002456
			216139_s_at	0	3.325	0.32	7	3.21	0.76	40	d	0.701742
			230162_s_at	0	2.286	0.04	7	2.358	0.1	40	u	0.078018
			232085_at	0	4.082	0.66	7	3.84	0.55	40	d	0.310931
			216137_s_at	0	2.362	0.26	7	2.358	0.31	40	d	0.972394
IMPDH1	4	IMP (inosine monophosphate) dehydrogenase 1	204169_at	4	6.61	0.23	7	7.533	0.77	40	u	0.000002
PRKACA	4	protein kinase, cAMP-dependent, catalytic, alpha	202801_at	4	7.996	0.15	7	7.859	0.61	40	d	0.245805
			216234_s_at	0	3.287	0.45	7	3.631	1.12	40	u	0.438881
LDB1	4	LIM domain binding 1	35160_at	2	6.365	0.44	7	6.654	0.53	40	u	0.193146
			203451_at	2	6.964	0.48	7	7.135	0.51	40	u	0.420353
TXNDC4	4	thioredoxin domain containing 4 (endoplasmic reticulum)	208958_at	4	5.422	0.24	7	5.019	0.46	40	d	0.030608
			208957_at	4	6.037	0.31	7	5.894	0.46	40	d	0.439576
			208959_s_at	1	9.789	0.17	7	9.664	0.56	40	d	0.278396
TAF15	4	TAF15 RNA polymerase II, TATA box binding protein (TBP)-associated factor, 68kDa	202840_at	4	8.332	0.33	7	8.179	0.55	40	d	0.486097
			227891_s_at	0	7.981	0.55	7	7.63	0.9	40	d	0.332442
			234168_at	0	2.208	0.05	7	2.413	0.51	40	u	0.019685
			227884_at	0	6.311	0.8	7	6.928	1.1	40	u	0.170039
LRRC57	4	leucine rich repeat containing 57	229232_at	4	5.604	0.27	7	6.105	0.74	40	u	0.004961
OBFC2A	4	oligonucleotide/oligosaccharide-binding fold containing 2A	219334_s_at	4	3.088	0.13	7	3.45	0.45	40	u	0.000379
			222872_x_at	4	6.901	0.44	7	7.456	0.82	40	u	0.092972
			233085_s_at	4	5.934	0.3	7	6.547	0.73	40	u	0.037322
R3HDM1	4	R3H domain containing 1	202754_at	4	7.411	0.33	7	8.279	0.45	40	u	0.000019
			210714_at	0	3.384	0.5	7	3.477	0.53	40	u	0.672577
MKRN1	4	makorin, ring finger protein, 1	201285_at	4	10.42	0.3	7	10.41	0.64	40	d	0.948511

MKRN1	4	makorin, ring finger protein, 1	209845_at	0	8.273	0.28	7	8.435	0.79	40	u	0.3531
ANXA7	4	annexin A7	209860_s_at	4	10.16	0.33	7	9.812	0.57	40	d	0.128259
			201366_at	4	10.14	0.21	7	10.26	0.45	40	u	0.51924
MORF4L1	4	mortality factor 4 like 1	224561_s_at	4	11.5	0.33	7	11.27	0.63	40	d	0.358674
			217982_s_at	4	12.24	0.1	7	12.08	0.47	40	d	0.068361
OLFM3	4	olfactomedin 3	1554524_a_at	4	2.547	0.11	7	2.703	0.17	40	u	0.030188
			1554526_at	4	2.988	0.13	7	3.301	0.37	40	u	0.000709
SLC39A1	4	solute carrier family 39 (zinc transporter), member 1	217778_at	4	7.745	0.21	7	8.264	0.6	40	u	0.000458
RBM25	4	RNA binding motif protein 25	212027_at	4	9.72	0.26	7	9.325	0.53	40	d	0.064854
			212030_at	4	9.184	0.3	7	8.444	0.76	40	d	0.01651
			212028_at	4	7.861	0.27	7	8.099	0.32	40	u	0.077096
			212031_at	4	9.134	0.4	7	8.757	0.61	40	d	0.129547
			212033_at	4	10.41	0.33	7	9.443	0.75	40	d	0.002002
			236613_at	0	5.826	0.59	7	6.161	0.61	40	u	0.195072
			1557081_at	0	8.143	0.52	7	7.269	1.09	40	d	0.04838
FBXO28	4	F-box protein 28	1555971_s_at	4	7.247	0.41	7	8.59	0.65	40	u	0.000005
			1555970_at	4	6.125	0.27	7	6.306	0.48	40	u	0.345998
			1555972_s_at	4	4.187	0.6	7	4.837	1.18	40	u	0.169972
			202272_s_at	4	6.64	0.62	7	7.903	0.81	40	u	0.000391
			202271_at	4	8.773	0.38	7	8.977	0.6	40	u	0.398316
AMOT	4	angiomotin	209521_s_at	4	2.803	0.33	7	2.621	0.18	40	d	0.232667
RALBP1	4	ralA binding protein 1	202844_s_at	4	9.064	0.24	7	9.409	0.85	40	u	0.047308
			202845_s_at	4	9.034	0.24	7	8.821	0.79	40	d	0.194279
			226957_x_at	4	6.355	0.29	7	6.111	0.82	40	d	0.182979
RUFY2	4	RUN and FYVE domain containing 2	238550_at	3	3.435	0.42	7	3.494	0.42	40	u	0.737606
			235345_at	3	2.772	0.19	7	2.895	0.41	40	u	0.444112
			219957_at	0	7.553	0.56	7	6.389	1.38	40	d	0.0367
			233192_s_at	0	3.91	0.34	7	4.045	0.55	40	u	0.541504
			233191_at	0	3.582	0.35	7	3.608	0.36	40	u	0.866086
			1554133_at	0	3.367	0.19	7	3.69	0.33	40	u	0.017407
			1569630_a_at	0	2.993	0.19	7	3.388	0.44	40	u	0.026118
CAPZB	4	capping protein (actin filament) muscle Z-line, beta	37012_at	4	10.2	0.25	7	10.24	0.38	40	u	0.795978
			215097_at	4	2.705	0.2	7	2.858	0.28	40	u	0.184322
			201949_x_at	4	9.443	0.18	7	9.382	0.37	40	d	0.672966
			201950_x_at	0	10.29	0.29	7	10.22	0.66	40	d	0.77183
YPEL3	4	yippee-like 3 (Drosophila)	223179_at	4	9.8	0.38	7	8.888	1.03	40	d	0.028921
			232077_s_at	0	5.105	0.44	7	4.84	1.03	40	d	0.514569
EYA2	4	eyes absent homolog 2 (Drosophila)	209692_at	4	6.984	0.78	7	5.572	1.81	40	d	0.052988
CKAP4	4	cytoskeleton-associated protein 4	200999_s_at	4	9.644	0.4	7	10.42	0.7	40	u	0.007091
			200998_s_at	4	8.526	0.29	7	9.587	0.72	40	u	0.00051
			226526_s_at	0	2.105	0.03	7	2.198	0.21	40	u	0.013593
IQGAP2	4	IQ motif containing GTPase activating protein 2	203474_at	4	8.729	0.88	7	6.314	1.22	40	d	0.000013
			241723_at	0	3.387	0.19	7	3.738	0.35	40	u	0.015667
RAC1	4	ras-related C3 botulinum toxin substrate 1 (rho family, small GTP binding protein Rac1)	208640_at	4	12.52	0.18	7	12.16	0.53	40	d	0.003871
			1567457_at	2	3.38	0.21	7	4.222	0.98	40	u	0.000026
			1567458_s_at	2	8.982	0.35	7	9.661	1.31	40	u	0.011727
			208641_s_at	0	11.44	0.29	7	11.59	0.59	40	u	0.527632
TIA1	4	TIA1 cytotoxic granule-associated RNA binding protein	201450_s_at	4	8.166	0.39	7	9.043	0.76	40	u	0.00523
			201446_s_at	2	9.058	0.31	7	9.301	0.85	40	u	0.201969
			201448_at	2	9.441	0.3	7	9.269	0.68	40	d	0.525692
			201447_at	2	8.32	0.28	7	8.116	0.63	40	d	0.412815
			201449_at	2	8.253	0.33	7	7.469	0.89	40	d	0.029874
			1554889_at	0	3.546	0.51	7	4.029	0.69	40	u	0.091832
			1554890_a_at	0	6.84	0.46	7	7.566	0.72	40	u	0.015855
SPTLC1	4	serine palmitoyltransferase, long chain base subunit 1	202277_at	4	10.18	0.28	7	10.01	0.59	40	d	0.467509
			1554053_at	0	3.671	0.23	7	3.915	0.65	40	u	0.09474
			202278_s_at	0	7.07	0.46	7	6.874	1.03	40	d	0.631268
MID1IP1	4	MID1 interacting protein 1 (gastrulation specific G12 homolog (zebrafish))	218251_at	4	6.71	0.59	7	7.593	0.84	40	u	0.012498
BAI3	4	brain-specific angiogenesis inhibitor 3	205638_at	4	2.055	0.02	7	2.11	0.06	40	u	0.022468
			211568_at	0	2.605	0.07	7	2.852	0.33	40	u	0.000189
TRIM23	4	tripartite motif-containing 23	204732_s_at	4	6.205	0.44	7	6.048	0.66	40	d	0.555225
			210994_x_at	2	4.886	0.8	7	4.712	0.86	40	d	0.627631

			210995_s_at	2	4.353	0.54	7	4.394	0.93	40	u	0.912933
ZNF219	4	zinc finger protein 219	222864_s_at	4	2.366	0.17	7	2.246	0.31	40	d	0.339733
			219314_s_at	4	7.387	0.84	7	6.093	0.8	40	d	0.000373
BASP1	4	brain abundant, membrane attached signal protein 1	202391_at	3	9.134	0.49	7	9.44	1.12	40	u	0.489956
			228589_at	0	4.019	0.34	7	4.028	0.42	40	u	0.958441
SOX21	4	SRY (sex determining region Y)-box 21	208468_at	0	1.712	0.07	7	1.727	0.06	40	u	0.559051
GRM8	4	glutamate receptor, metabotropic 8	216992_s_at	4	3.236	0.13	7	3.507	0.26	40	u	0.011568
			216255_s_at	0	2.076	0.02	7	2.123	0.05	40	u	0.012183
			216256_at	0	4.493	0.39	7	4.795	0.47	40	u	0.120903
SLC44A2	4	solute carrier family 44, member 2	224609_at	4	8.56	0.25	7	8.828	1.01	40	u	0.171738
			225175_s_at	4	8.308	0.19	7	8.285	0.98	40	d	0.897146
EPHA2	4	EPH receptor A2	203499_at	4	6.47	0.9	7	5.119	0.68	40	d	0.00005
GABPB2	4	GA binding protein transcription factor, beta subunit 2	204618_s_at	4	7.857	0.42	7	8.111	0.51	40	u	0.233091
			226900_at	0	2.667	0.24	7	2.556	0.22	40	d	0.235654
			227406_at	0	7.226	0.35	7	6.758	0.8	40	d	0.141965
			206173_x_at	0	4.324	0.45	7	5.252	0.98	40	u	0.019685
KCND1	4	potassium voltage-gated channel, Shal-related subfamily, member 1	206842_at	4	3.333	0.31	7	3.199	0.45	40	d	0.467114
CALML4	4	calmodulin-like 4	1568600_at	4	2.963	0.42	7	3.061	0.5	40	u	0.639118
			64408_s_at	0	6.419	0.23	7	6.425	0.86	40	u	0.972038
			1566149_at	0	2.759	0.04	7	2.818	0.09	40	u	0.101883
			221879_at	0	4.95	0.44	7	5.361	1.47	40	u	0.17577
			1566150_at	0	2.589	0.04	7	2.752	0.23	40	u	0.00018
C1orf218	4	chromosome 1 open reading frame 218	219696_at	4	7.712	0.79	7	8.515	1.07	40	u	0.070949
			1564164_at	0	6.855	0.6	7	7.622	1.18	40	u	0.105616
RASL10B	4	RAS-like, family 10, member B	244656_at	4	6.418	0.37	7	6.243	0.47	40	d	0.360296
			235488_at	4	2.744	0.22	7	2.846	0.46	40	u	0.575927
COL15A1	4	collagen, type XV, alpha 1	203477_at	4	9.716	0.47	7	8.157	1.27	40	d	0.002955
PCMT1	4	protein-L-isoaspartate (D-aspartate) O-methyltransferase	205202_at	4	10.14	0.18	7	10.49	0.73	40	u	0.014718
			210156_s_at	0	9.175	0.28	7	9.745	0.89	40	u	0.004161
			208857_s_at	0	9.592	0.17	7	10.23	0.78	40	u	0.000058
PIAS3	4	protein inhibitor of activated STAT, 3	203035_s_at	4	7.039	0.38	7	6.217	1.3	40	d	0.003484
CTGLF1	4	centaurin, gamma-like family, member 1	221850_x_at	8	8.629	0.3	7	8.059	0.69	40	d	0.041122
			221971_x_at	0	8.727	0.38	7	8.154	0.72	40	d	0.049603
			1565620_at	0	5.698	0.77	7	6.251	0.97	40	u	0.169871
EVI1	4	ecotropic viral integration site 1	221884_at	4	5.985	0.84	7	5.079	0.9	40	d	0.019162
			226420_at	4	4.797	0.73	7	3.877	0.72	40	d	0.003746
			215851_at	0	2.122	0.02	7	2.17	0.04	40	u	0.000054
			243277_x_at	0	3.441	0.23	7	3.694	0.33	40	u	0.0642
SLC2A3	4	solute carrier family 2 (facilitated glucose transporter), member 3	202498_s_at	4	4.834	0.78	7	4.983	0.74	40	u	0.638382
			202497_x_at	4	6.819	1.21	7	6.912	0.99	40	u	0.830483
			202499_s_at	4	7.752	0.99	7	7.118	0.99	40	d	0.133361
			216236_s_at	2	6.14	1.19	7	6.07	1.22	40	d	0.891537
			222088_s_at	2	6.217	1	7	5.888	1.05	40	d	0.457427
CPLX1	4	complexin 1	223500_at	4	6.317	0.48	7	4.616	1.43	40	d	0.000008
MYEF2	4	myelin expression factor 2	232676_x_at	4	7.44	0.52	7	7.654	1.28	40	u	0.671253
			222153_at	4	2.645	0.23	7	2.709	0.25	40	u	0.52924
			222772_at	4	5.197	0.4	7	5.394	1.2	40	u	0.44127
			222771_s_at	4	7.182	0.35	7	7.578	1.25	40	u	0.118117
			229464_at	0	5.367	0.64	7	5.548	1.48	40	u	0.755705
MARCH8	4	membrane-associated ring finger (C3HC4) 8	231933_at	4	4.749	0.44	7	4.763	0.73	40	u	0.961818
			236056_s_at	0	2.146	0.1	7	2.196	0.1	40	u	0.250762
			221824_s_at	0	8.641	0.55	7	7.235	0.73	40	d	0.000021
SYPL2	4	synaptophysin-like 2	230611_at	4	2.125	0.23	7	2.213	0.38	40	u	0.557145
TMEM161B	4	transmembrane protein 161B	227861_at	4	7.828	0.45	7	7.078	0.71	40	d	0.011789
			236227_at	1	5.343	0.38	7	5.362	0.58	40	u	0.934813
			238783_at	0	5.355	0.5	7	5.213	0.62	40	d	0.575542
			244513_at	0	3.402	0.17	7	3.635	0.46	40	u	0.199854
CCNG2	4	cyclin G2	202769_at	4	10.15	0.45	7	9.57	0.84	40	d	0.090047
			202770_s_at	4	9.156	0.5	7	8.362	1.17	40	d	0.090374
			211559_s_at	1	9.465	0.53	7	8.304	1.8	40	d	0.003132
			1555056_at	0	3.166	0.33	7	3.333	0.46	40	u	0.369211
TANC1	4	tetratricopeptide repeat, ankyrin repeat and coiled-coil containing 1	234039_at	0	4.949	0.56	7	4.797	0.94	40	d	0.686302
			225308_s_at	0	8.7	0.22	7	8.122	0.82	40	d	0.00098

		coiled-coil containing 1	234870_at	0	2.048	0.08	7	2.173	0.23	40	u	0.018274
PRDM4	4	PR domain containing 4	49485_at	3	6.916	0.35	7	6.864	0.58	40	d	0.823164
			218329_at	3	7.891	0.19	7	7.797	0.58	40	d	0.444114
C11orf58	4	chromosome 11 open reading frame 58	200084_at	4	11.08	0.12	7	10.89	0.5	40	d	0.055979
			201784_s_at	3	11.08	0.26	7	10.91	0.6	40	d	0.485955
PHOX2B	4	paired-like homeobox 2b	207009_at	4	1.883	0.09	7	1.97	0.17	40	u	0.201276
ELOVL7	4	ELOVL family member 7, elongation of long chain fatty acids (yeast)	227180_at	4	7.734	2.25	7	6.296	1.45	40	d	0.036567
VAPA	4	VAMP (vesicle-associated membrane protein)-associated protein A, 33kDa	228480_at	4	7.424	0.72	7	5.713	1.05	40	d	0.000199
			225198_at	4	9.298	0.46	7	7.97	0.86	40	d	0.000328
			208780_x_at	3	11.37	0.21	7	11.38	0.67	40	u	0.980368
			239750_x_at	0	3.653	0.22	7	3.847	0.23	40	u	0.047112
			242780_at	0	5.481	0.44	7	5.39	0.63	40	d	0.720886
MAP2K6	4	mitogen-activated protein kinase kinase 6	205699_at	4	2.592	0.29	7	2.407	0.14	40	d	0.170592
			205698_s_at	0	7.27	0.74	7	5.613	1.24	40	d	0.001614
SLBP	4	stem-loop (histone) binding protein	206052_s_at	4	8.19	0.23	7	9.168	0.72	40	u	0.000001
AP2M1	4	adaptor-related protein complex 2, mu 1 subunit	200613_at	4	10.53	0.18	7	10.57	0.57	40	u	0.722453
CSK	4	c-src tyrosine kinase	202329_at	4	7.999	0.35	7	8.148	0.63	40	u	0.551297
MORF4L2	4	mortality factor 4 like 2	201994_at	4	12.9	0.22	7	13	0.45	40	u	0.574016
			243857_at	0	4.87	0.78	7	5.629	1.11	40	u	0.096087
			243683_at	0	4.776	0.89	7	5.524	0.89	40	u	0.05057
PRKAB2	4	protein kinase, AMP-activated, beta 2 non-catalytic subunit	225278_at	4	7.264	0.52	7	7.037	0.93	40	d	0.54371
			1558027_s_at	3	6.485	0.55	7	6.99	0.88	40	u	0.158136
			214474_at	0	3.766	0.58	7	5.29	1.05	40	u	0.00065
RAF1	4	v-raf-1 murine leukemia viral oncogene homolog 1	201244_s_at	4	9.396	0.2	7	9.2	0.57	40	d	0.120918
			1557675_at	0	7.174	0.36	7	6.198	0.93	40	d	0.01051
ZNF281	4	zinc finger protein 281	218401_s_at	4	6.164	0.4	7	7.393	1.01	40	u	0.003388
			228785_at	0	7.399	0.47	7	8.265	0.86	40	u	0.014756
			222619_at	0	7.425	0.51	7	8.281	0.86	40	u	0.015941
CLDND1	4	claudin domain containing 1	1554149_at	3	8.814	0.19	7	8.639	0.57	40	d	0.152929
			208925_at	1	8.765	0.25	7	8.551	0.86	40	d	0.222253
			239146_at	0	3.677	0.43	7	3.479	0.4	40	d	0.250591
ATG5	4	ATG5 autophagy related 5 homolog (S. cerevisiae)	202511_s_at	4	8.028	0.28	7	8.724	0.91	40	u	0.000765
			202512_s_at	4	7.41	0.33	7	6.91	1.26	40	d	0.046366
			210639_s_at	0	6.555	0.42	7	7.315	1.2	40	u	0.007251
HS6ST2	4	heparan sulfate 6-O-sulfotransferase 2	1552766_at	4	3.07	0.3	7	3.231	0.6	40	u	0.501174
			230030_at	4	4.841	0.93	7	3.982	1.44	40	d	0.143612
			1552767_a_at	4	6.751	0.6	7	5.52	1.53	40	d	0.04532
KIF5A	4	kinesin family member 5A	205318_at	5	1.877	0.22	7	1.977	0.34	40	u	0.461685
			229921_at	0	2.631	0.23	7	2.886	0.7	40	u	0.093382
			223933_at	0	3.397	0.47	7	3.828	0.73	40	u	0.145136
MIDN	4	midnolin	225954_s_at	4	7.538	0.37	7	7.319	0.82	40	d	0.502083
			231072_at	0	2.53	0.44	7	2.429	0.39	40	d	0.548447
			227398_s_at	0	1.86	0.02	7	1.961	0.25	40	u	0.01697
NOX4	4	NADPH oxidase 4	219773_at	4	4.391	1.38	7	5.528	1.3	40	u	0.044807
OPRS1	4	opioid receptor, sigma 1	214484_s_at	4	7.94	0.23	7	8.095	1.08	40	u	0.436131
			201692_at	4	7.921	0.14	7	8.008	0.72	40	u	0.505058
CRTC2	4	CREB regulated transcription coactivator 2	226307_at	4	7.389	0.17	7	7.349	0.68	40	d	0.755038
NR6A1	4	nuclear receptor subfamily 6, group A, member 1	210392_x_at	4	2.647	0.25	7	2.802	0.32	40	u	0.242445
			210391_at	4	2.05	0.07	7	2.17	0.16	40	u	0.054521
			211402_x_at	4	2.533	0.1	7	2.701	0.22	40	u	0.061764
			207742_s_at	1	2.042	0.04	7	2.095	0.18	40	u	0.115609
PFKFB3	4	6-phosphofructo-2-kinase/fructose-2,6-biphosphatase 3	202464_s_at	4	10.2	0.45	7	9.407	0.92	40	d	0.033599
DIRAS2	4	DIRAS family, GTP-binding RAS-like 2	219619_at	4	2.372	0.03	7	2.619	0.65	40	u	0.022672
			240122_at	0	2.202	0.1	7	2.358	0.71	40	u	0.206022
OTP	4	orthopedia homeobox	223835_x_at	4	2.659	0.12	7	2.823	0.27	40	u	0.131736
			231488_at	0	2.214	0.04	7	2.273	0.07	40	u	0.028721
PCDHGA9	4	protocadherin gamma subfamily A, 9	211873_s_at	0	2.087	0.02	7	2.163	0.1	40	u	0.000215
PER2	4	period homolog 2 (Drosophila)	205251_at	4	9.346	0.5	7	7.245	1.09	40	d	0.000012
			208518_s_at	0	3.261	1.17	7	2.644	0.63	40	d	0.253219
RBPMS2	4	RNA binding protein with multiple splicing 2	228802_at	2	3.001	0.54	7	3.144	1.21	40	u	0.765609

AK2	4	adenylate kinase 2	205996_s_at	4	7.389	0.4	7	7.359	1.32	40	d	0.912794
			212172_at	4	5.707	0.43	7	5.673	0.6	40	d	0.890634
			212174_at	4	8.052	0.48	7	7.793	1.19	40	d	0.581235
			212173_at	4	5.749	0.49	7	5.557	1.13	40	d	0.667301
			212175_s_at	4	8.594	0.26	7	8.858	0.89	40	u	0.146603
			208967_s_at	0	9.544	0.26	7	9.849	0.89	40	u	0.09819
CSNK1A1	4	casein kinase 1, alpha 1	208866_at	5	9.418	0.4	7	8.221	0.87	40	d	0.001056
			208867_s_at	5	8.865	0.51	7	8.515	1.07	40	d	0.410703
			208865_at	5	11.51	0.33	7	11.11	0.62	40	d	0.102018
			213086_s_at	4	10.59	0.37	7	10.76	0.56	40	u	0.451357
			213860_x_at	4	10.91	0.32	7	10.98	0.52	40	u	0.717779
			243338_at	0	7.772	0.57	7	7.067	1.26	40	d	0.161093
			235464_at	0	4.251	0.81	7	4.438	0.82	40	u	0.591127
			206562_s_at	0	9.966	0.26	7	10.42	0.57	40	u	0.05128
			240221_at	0	7.768	0.41	7	7.002	1.01	40	d	0.059756
			1556007_s_at	0	9.119	0.46	7	8.498	1.25	40	d	0.210353
			1556006_s_at	0	7.591	0.49	7	6.949	1.11	40	d	0.146902
			226920_at	0	2.326	0.28	7	2.802	0.49	40	u	0.017963
LDOC1L	4	leucine zipper, down-regulated in cancer 1-like	223228_at	4	8.775	0.25	7	8.412	0.66	40	d	0.168025
PRRC1	4	proline-rich coiled-coil 1	224643_at	4	9.203	0.24	7	9.382	0.59	40	u	0.443643
			221734_at	4	7.201	0.33	7	8.202	0.73	40	u	0.001083
KPNA1	4	karyopherin alpha 1 (importin alpha 5)	202056_at	4	6.582	0.34	7	6.251	0.85	40	d	0.322987
			202057_at	4	5.087	0.31	7	4.389	0.87	40	d	0.001036
			202055_at	4	7.34	0.15	7	7.294	0.51	40	d	0.659573
			213741_s_at	4	6.052	0.25	7	7.23	0.76	40	u	0
			202059_s_at	4	7.274	0.29	7	7.557	0.57	40	u	0.212199
			202058_s_at	0	6.096	0.56	7	6.997	1.45	40	u	0.119739
M6PR	4	mannose-6-phosphate receptor (cation dependent)	200901_s_at	7	9.439	0.2	7	9.249	0.58	40	d	0.137469
			200900_s_at	7	9.016	0.29	7	9.386	0.73	40	u	0.202719
DOT1L	4	DOT1-like, histone H3 methyltransferase (S. cerevisiae)	231297_at	4	4.246	0.57	7	4.198	0.72	40	d	0.870441
			214865_at	0	1.812	0.02	7	1.844	0.04	40	u	0.029889
CLTC	4	clathrin, heavy chain (Hc)	200614_at	4	12.04	0.16	7	12.22	0.72	40	u	0.167332
			210498_at	0	3.205	0.13	7	3.323	0.3	40	u	0.322951
			239871_at	0	2.362	0.05	7	2.575	0.44	40	u	0.005422
GPR88	4	G protein-coupled receptor 88	220313_at	4	2.58	0.23	7	2.471	0.1	40	d	0.287703
COL13A1	4	collagen, type XIII, alpha 1	211343_s_at	3	4.53	0.73	7	4.752	0.8	40	u	0.505412
			208535_x_at	0	3.449	0.51	7	3.366	0.48	40	d	0.686441
			211809_x_at	0	4.464	0.4	7	4.228	0.54	40	d	0.283916
RPS15	4	ribosomal protein S15	200819_s_at	0	14.3	0.11	7	13.81	0.44	40	d	0.000001
			221126_at	0	2.594	0.11	7	2.647	0.3	40	u	0.42836
			1563014_at	0	2.364	0.12	7	2.379	0.09	40	u	0.723654
			221127_s_at	0	5.116	0.6	7	4.194	0.56	40	d	0.000316
GOLGA7	4	golgi autoantigen, golgin subfamily a, 7	217819_at	4	9.945	0.14	7	9.889	0.9	40	d	0.71772
			1554167_a_at	2	8.864	0.34	7	8.773	1.12	40	d	0.693424
DNAJC6	4	DnaJ (Hsp40) homolog, subfamily C, member 6	204720_s_at	4	3.331	0.46	7	4.102	1.19	40	u	0.104741
			204721_s_at	4	2.688	0.06	7	2.875	0.21	40	u	0.000069
SEC23A	4	Sec23 homolog A (S. cerevisiae)	212887_at	4	8.495	0.3	7	8.828	0.82	40	u	0.302774
			204344_s_at	0	6.597	0.44	7	6.64	1.16	40	u	0.925997
MLX	4	MAX-like protein X	210752_s_at	4	4.947	0.29	7	4.852	0.56	40	d	0.669302
			217909_s_at	3	7.433	0.35	7	7.475	0.79	40	u	0.892926
			213708_s_at	3	8.734	0.27	7	8.746	0.6	40	u	0.958916
			217910_x_at	3	8.038	0.25	7	7.942	0.5	40	d	0.626362
			1570347_at	0	3.168	0.34	7	3.373	0.38	40	u	0.195537
TPD52	4	tumor protein D52	201690_s_at	4	10.69	0.33	7	11.37	1.01	40	u	0.002786
			201689_s_at	4	9.003	0.49	7	10	1.37	40	u	0.002482
			201688_s_at	4	9.789	0.52	7	10.5	1.67	40	u	0.047215
			201691_s_at	4	6.439	0.54	7	7.129	1.22	40	u	0.155721
			236655_at	0	6.167	0.5	7	6.734	1.42	40	u	0.076057
GAPVD1	4	GTPase activating protein and VPS9 domains 1	212802_s_at	4	9.051	0.18	7	8.793	0.59	40	d	0.040877
			212804_s_at	4	6.405	0.17	7	7.035	0.55	40	u	0.000004
			214869_x_at	2	6.278	0.28	7	6.91	0.59	40	u	0.009364
ZDHHC18	4	zinc finger, DHHC-type containing 18	212860_at	4	6.269	0.16	7	6.464	0.59	40	u	0.099113
			1558327_at	0	3.209	0.25	7	3.556	0.62	40	u	0.157328

			231900_at	0	2.221	0.02	7	2.328	0.24	40	u	0.00857
TBC1D4	4	TBC1 domain family, member 4	203387_s_at	4	8.623	0.24	7	7.296	0.95	40	d	0
			203386_at	4	9.419	0.35	7	8.206	0.84	40	d	0.000604
PCDHGA2	4	protocadherin gamma subfamily A, 2	no probeset available									
PHIP	4	pleckstrin homology domain interacting protein	212542_s_at	1	10.19	0.15	7	9.706	0.71	40	d	0.000522
			1557363_a_at	0	2.621	0.04	7	2.797	0.17	40	u	0.000001
			1557362_at	0	2.128	0.02	7	2.155	0.06	40	u	0.028228
C15orf21	4	chromosome 15 open reading frame 21	242649_x_at	0	3.666	0.47	7	3.937	1	40	u	0.495637
SS18	4	synovial sarcoma translocation, chromosome 18	202816_s_at	4	8.009	0.46	7	8.418	0.95	40	u	0.279297
			202817_s_at	3	8.075	0.46	7	8.059	1.01	40	d	0.967818
			216684_s_at	0	6.971	0.49	7	7.319	0.8	40	u	0.28003
			217051_s_at	0	2.264	0.14	7	2.352	0.35	40	u	0.528917
			209954_x_at	0	7.189	0.52	7	7.705	1.16	40	u	0.263012
ANKRD57	4	ankyrin repeat domain 57	219496_at	4	7.991	0.54	7	7.584	0.93	40	d	0.27876
			227034_at	4	7.78	0.37	7	7.185	1.2	40	d	0.021239
PPP1R12B	4	protein phosphatase 1, regulatory (inhibitor) subunit 12B	201957_at	4	8.566	0.52	7	6.119	1.13	40	d	0.000002
			201958_s_at	1	2.258	0.19	7	2.352	0.71	40	u	0.497848
			1555444_a_at	0	3.162	0.1	7	3.44	0.23	40	u	0.003678
			1557553_at	0	5.813	0.89	7	4.427	0.89	40	d	0.000565
PLXNA2	4	plexin A2	213030_s_at	4	4.421	0.81	7	4.444	1.71	40	u	0.972957
			207290_at	4	2.936	0.56	7	3.02	0.77	40	u	0.788745
			227032_at	3	4.436	0.55	7	5.238	1.04	40	u	0.058409
MGLL	4	monoglyceride lipase	225102_at	4	9.207	0.62	7	7.688	1.41	40	d	0.008662
			211026_s_at	0	8.893	0.5	7	7.323	1.47	40	d	0.000033
OPTN	4	optineurin	202073_at	0	7.586	0.36	7	7.314	1.12	40	d	0.251353
			202074_s_at	0	8.668	0.29	7	8.884	1.09	40	u	0.31097
AFAP1L2	4	actin filament associated protein 1-like 2	226829_at	4	5.424	0.6	7	3.297	0.88	40	d	0
RAI1	4	retinoic acid induced 1	1552558_a_at	4	4.949	0.11	7	5.269	0.31	40	u	0.000099
			226143_at	4	8.969	0.36	7	8.478	0.85	40	d	0.148948
			219830_at	0	2.887	0.32	7	2.528	0.31	40	d	0.00898
PCGF5	4	polycomb group ring finger 5	229194_at	4	7.572	0.33	7	7.67	0.94	40	u	0.635123
			235331_x_at	2	4.492	0.4	7	4.951	1.03	40	u	0.261027
			226326_at	0	9.078	0.31	7	7.829	0.9	40	d	0.000001
			229996_s_at	0	2.472	0.05	7	2.558	0.1	40	u	0.033257
			227935_s_at	0	5.25	0.5	7	6.247	0.95	40	u	0.010905
CDH9	4	cadherin 9, type 2 (T1-cadherin)	207729_at	4	2.408	0.05	7	2.513	0.14	40	u	0.059552
PPM1B	4	protein phosphatase 1B (formerly 2C), magnesium-dependent, beta isoform	209296_at	4	9.195	0.17	7	9.299	0.57	40	u	0.374498
			213225_at	0	6.897	0.38	7	7.269	0.86	40	u	0.27805
PACSIN1	4	protein kinase C and casein kinase substrate in neurons 1	227053_at	4	3.822	0.33	7	3.969	0.65	40	u	0.569454
			224360_s_at	0	2.381	0.3	7	2.283	0.12	40	d	0.453718
MAP3K2	4	mitogen-activated protein kinase kinase kinase 2	226979_at	4	9.911	0.22	7	10.09	0.43	40	u	0.304446
			221695_s_at	1	5.88	0.98	7	6.613	1.25	40	u	0.1551
			235011_at	0	4.479	0.34	7	5.096	0.68	40	u	0.025863
			227073_at	0	6.144	0.44	7	6.541	0.49	40	u	0.055616
			235254_at	0	2.307	0.32	7	2.247	0.18	40	d	0.669584
LEPROTL1	4	leptin receptor overlapping transcript-like 1	202594_at	4	8.117	0.2	7	8.113	0.57	40	d	0.970676
			202595_s_at	0	8.108	0.24	7	8.453	0.81	40	u	0.043992
FGF9	4	fibroblast growth factor 9 (glia-activating factor)	206404_at	4	2.359	0.37	7	2.455	0.73	40	u	0.739815
CCNG1	4	cyclin G1	208796_s_at	4	11.19	0.2	7	9.936	0.94	40	d	0
SLC27A4	4	solute carrier family 27 (fatty acid transporter), member 4	225779_at	4	6.719	0.24	7	6.322	0.98	40	d	0.038659
ABAT	4	4-aminobutyrate aminotransferase	209460_at	4	8.498	0.71	7	6.934	1.72	40	d	0.025259
			209459_s_at	4	6.991	0.7	7	5.523	1.94	40	d	0.001998
			206527_at	0	3.272	0.58	7	3.643	0.9	40	u	0.307802
PCDHGB5	4	protocadherin gamma subfamily B, 5	211807_x_at	0	2.557	0.12	7	2.819	0.39	40	u	0.002533
SLC20A2	4	solute carrier family 20 (phosphate transporter), member 2	202744_at	2	6.498	0.39	7	6.516	0.75	40	u	0.951526
DNAJB9	4	DnaJ (Hsp40) homolog, subfamily B, member 9	202843_at	4	5.844	0.34	7	6.113	0.84	40	u	0.417499
			202842_s_at	4	9.329	0.21	7	8.949	0.81	40	d	0.020425
			1554462_a_at	1	6.772	0.43	7	6.828	0.84	40	u	0.86529
KIF1C	4	kinesin family member 1C	209245_s_at	4	3.053	0.3	7	3.066	0.35	40	u	0.93158
			209244_s_at	4	9.51	0.22	7	9.143	0.46	40	d	0.049159
LDLRAD3	4	low density lipoprotein receptor class A	244338_at	4	2.901	0.15	7	3.557	0.81	40	u	0.000039

[illegible]	[illegible]	domain containing 3	234985_at	4	8.452	0.45	7	7.996	1.28	40	d	0.110487
RAB5C	4	RAB5C, member RAS oncogene family	201156_s_at	4	8.266	0.49	7	7.963	1.11	40	d	0.492584
			201140_s_at	4	9.405	0.28	7	9.314	0.64	40	d	0.719466
PIP4K2C	4	phosphatidylinositol-5-phosphate 4-kinase, type II, gamma	218942_at	4	8.164	0.29	7	8.112	0.88	40	d	0.778602
WSCD2	4	WSC domain containing 2	229032_at	4	2.139	0.21	7	2.334	0.48	40	u	0.309353
			207637_at	0	2.404	0.08	7	2.473	0.23	40	u	0.168914
MGAT4A	4	mannosyl (alpha-1,3-)-glycoprotein beta-1,4-N-acetylglucosaminyltransferase, isozyme A	219797_at	4	3.672	0.48	7	4.835	1.43	40	u	0.000653
			226039_at	0	8.574	0.47	7	8.256	1.62	40	d	0.331931
			231283_at	0	5.736	0.42	7	6.154	1.5	40	u	0.166283
			1569136_at	0	3.153	0.19	7	3.492	0.51	40	u	0.097907
EIF4E3	4	eukaryotic translation initiation factor 4E family member 3	225941_at	4	7.815	0.48	7	6.449	1.52	40	d	0.00015
			225940_at	4	7.919	0.41	7	7.062	1.14	40	d	0.002079
			225939_at	4	4.833	0.45	7	4.6	0.71	40	d	0.415121
			238461_at	2	4.21	0.9	7	4.017	1.24	40	d	0.70193
USP48	4	ubiquitin specific peptidase 48	220078_at	4	5.938	0.47	7	5.838	0.61	40	d	0.686196
			220079_s_at	4	9.091	0.2	7	8.393	0.71	40	d	0.000024
			225925_s_at	4	7.813	0.21	7	7.608	0.78	40	d	0.186879
			232621_at	0	3.088	0.17	7	3.369	0.49	40	u	0.012071
			229812_at	0	4.933	0.57	7	5.231	0.79	40	u	0.357375
BRWD3	4	bromodomain and WD repeat domain containing 3	244738_at	3	6.758	0.18	7	6.978	0.32	40	u	0.090827
			1553252_a_at	0	4.709	0.32	7	5.969	0.91	40	u	0.000001
ZFYVE21	4	zinc finger, FYVE domain containing 21	224445_s_at	4	10.1	0.22	7	8.989	0.7	40	d	0
			219929_s_at	0	7.931	0.3	7	6.985	1.21	40	d	0.000197
HEY2	4	hairy/enhancer-of-split related with YRPW motif 2	219743_at	4	7.452	0.88	7	6.206	1.6	40	d	0.055163
			222921_s_at	0	5.378	0.33	7	5.845	1.72	40	u	0.133718
SLC39A11	4	solute carrier family 39 (metal ion transporter), member 11	227046_at	4	7.786	0.59	7	7.773	0.88	40	d	0.970225
ENPP1	4	ectonucleotide pyrophosphatase/phosphodiesterase 1	205066_s_at	0	7.912	0.75	7	8.143	1.71	40	u	0.731559
PAX3	4	paired box 3	231666_at	4	2.812	0.15	7	3.165	0.75	40	u	0.011778
			207679_at	0	2.712	0.04	7	2.823	0.24	40	u	0.009566
			207680_x_at	0	2.018	0.14	7	2.069	0.17	40	u	0.479958
			216059_at	0	2.045	0.02	7	2.101	0.1	40	u	0.003399
YAP1	4	Yes-associated protein 1, 65kDa	224895_at	4	10.29	0.31	7	8.585	1.1	40	d	0
			224894_at	4	10.33	0.15	7	8.794	0.96	40	d	0
			213342_at	0	7.187	0.24	7	7.21	1.32	40	u	0.923086
SSX2IP	4	synovial sarcoma, X breakpoint 2 interacting protein	203016_s_at	4	5.952	0.52	7	7.498	0.86	40	u	0.000045
			203015_s_at	4	3.815	0.55	7	5.323	1.42	40	u	0.009271
			203017_s_at	4	5.745	0.58	7	6.634	1.08	40	u	0.042453
			203018_s_at	4	5.229	0.31	7	6.218	0.58	40	u	0.000094
			203019_x_at	4	3.838	0.44	7	4.315	0.82	40	u	0.148188
			210871_x_at	4	4.425	0.69	7	4.997	0.96	40	u	0.146863
TMTC4	4	transmembrane and tetratricopeptide repeat containing 4	225666_at	4	6.895	0.43	7	7.065	1.02	40	u	0.672097
			1554102_a_at	0	4.051	0.45	7	4.934	1.01	40	u	0.030938
			1554101_a_at	0	2.494	0.3	7	3.164	0.97	40	u	0.002145
PFN1	4	profilin 1	200634_at	4	12.19	0.2	7	12.5	0.61	40	u	0.020362
ARL15	4	ADP-ribosylation factor-like 15	219842_at	4	3.772	0.5	7	3.841	0.75	40	u	0.81858
DUSP15	4	dual specificity phosphatase 15	230402_at	4	5.204	0.35	7	4.966	0.59	40	d	0.317843
DUSP16	4	dual specificity phosphatase 16	224336_s_at	4	7.659	0.54	7	6.74	1	40	d	0.025109
			1555399_a_at	1	5.985	0.44	7	5.428	0.71	40	d	0.055382
			224832_at	0	9.214	0.37	7	7.918	0.96	40	d	0.001178
			1563505_at	0	3.94	0.83	7	4.225	0.75	40	u	0.379441
PDZD4	4	PDZ domain containing 4	223543_at	4	4.899	0.38	7	4.336	0.77	40	d	0.069387
FZD1	4	frizzled homolog 1 (Drosophila)	204451_at	4	9.475	0.19	7	7.979	1.18	40	d	0
			204452_s_at	1	6.224	0.8	7	4.617	1.57	40	d	0.01298
TRPC4AP	4	transient receptor potential cation channel, subfamily C, member 4 associated protein	212059_s_at	4	8.167	0.17	7	8.015	0.43	40	d	0.37218
ZNFX1	4	zinc finger, NFX1-type containing 1	225076_s_at	4	8.346	0.19	7	8.334	0.7	40	d	0.932669
CCDC92	4	coiled-coil domain containing 92	218175_at	4	9.601	0.27	7	8.883	0.51	40	d	0.00095
PEX5L	4	peroxisomal biogenesis factor 5-like	222910_s_at	4	2.325	0.07	7	2.518	0.46	40	u	0.01743
			219642_s_at	4	2.067	0.03	7	2.153	0.13	40	u	0.000552
TMSB4X	4	thymosin, beta 4, X-linked	216438_s_at	0	14.39	0.18	7	14.51	0.55	40	u	0.336851
C18orf34	4	chromosome 18 open reading frame 34	1558820_a_at	4	4.198	0.68	7	2.946	0.73	40	d	0.000157

TBC1D9	4	TBC1 domain family, member 9 (with GRAM domain)	212960_at	4	8.302	0.62	7	6.756	1.86	40	d	0.000515
			212956_at	4	11.55	0.53	7	9.629	2.08	40	d	0.000025
HSPG2	4	heparan sulfate proteoglycan 2	201655_s_at	7	8.292	0.49	7	7.535	1.09	40	d	0.08419
			201654_s_at	7	5.119	0.85	7	4.334	1.14	40	d	0.095707
SMARCAL1	4	SWI/SNF related, matrix associated, actin dependent regulator of chromatin, subfamily a-like 1	218452_at	0	6.614	0.17	7	6.959	0.44	40	u	0.050919
NPAS3	4	neuronal PAS domain protein 3	220316_at	4	4.454	0.41	7	4.336	0.38	40	d	0.473251
			222172_at	0	2.479	0.05	7	2.55	0.1	40	u	0.080395
			230412_at	0	3.314	0.47	7	3.335	0.41	40	u	0.905803
			1554635_a_at	0	1.783	0.23	7	1.699	0.06	40	d	0.419832
AMD1	4	adenosylmethionine decarboxylase 1	201196_s_at	4	8.155	0.42	7	9.052	1.19	40	u	0.001834
			201197_at	4	9.357	0.28	7	9.723	0.96	40	u	0.067688
GRIA2	4	glutamate receptor, ionotropic, AMPA 2	205358_at	4	3.737	1.75	7	3.353	2.15	40	d	0.664365
			241172_at	0	2.619	0.27	7	2.668	0.27	40	u	0.670666
			236538_at	0	2.755	0.21	7	3.328	1.31	40	u	0.015207
FAM49B	4	family with sequence similarity 49, member B	217916_s_at	4	8.439	0.19	7	9.737	0.54	40	u	0
			217534_at	0	4.188	0.32	7	4.473	0.51	40	u	0.166568
			217535_at	0	3.117	0.26	7	3.19	0.37	40	u	0.630453
SNX17	4	sorting nexin 17	200991_s_at	4	9.236	0.15	7	8.933	0.53	40	d	0.006351
ARL6IP2	4	ADP-ribosylation factor-like 6 interacting protein 2	1553603_s_at	4	4.537	0.57	7	5.545	1.49	40	u	0.091135
			237968_at	0	3.654	0.47	7	4.806	1.36	40	u	0.000575
			222700_at	0	10.4	0.43	7	10.35	1.07	40	d	0.891115
PIK3IP1	4	phosphoinositide-3-kinase interacting protein 1	221757_at	4	7.356	0.47	7	7.006	0.86	40	d	0.311794
			221756_at	4	7.942	0.57	7	6.912	0.81	40	d	0.002934
			1555632_at	0	3.705	0.26	7	3.678	0.33	40	d	0.836201
FSTL1	3	follistatin-like 1	208782_at	3	11.58	0.66	7	10.33	0.83	40	d	0.000548
			240891_at	0	2.848	0.17	7	2.938	0.24	40	u	0.353809
NAGPA	3	N-acetylglucosamine-1-phosphodiester alpha-N-acetylglucosaminidase	205090_s_at	3	6.454	0.26	7	6.419	0.52	40	d	0.863398
IRAK1	3	interleukin-1 receptor-associated kinase 1	201587_s_at	3	9.744	0.31	7	10.51	0.97	40	u	0.000599
			1555784_s_at	1	4.324	0.31	7	5.386	1.44	40	u	0.000227
HNRPD	3	heterogeneous nuclear ribonucleoprotein D (AU-rich element RNA binding protein 1, 37kDa)	221481_x_at	0	11.17	0.23	7	10.73	0.52	40	d	0.034812
			239040_at	0	3.739	0.32	7	4.09	0.61	40	u	0.15482
			200073_s_at	0	11.75	0.22	7	11.36	0.51	40	d	0.055656
			205183_at	0	3.063	0.36	7	2.892	0.35	40	d	0.249876
			239052_at	0	2.106	0.03	7	2.352	0.33	40	u	0.000038
			213359_at	0	9.429	0.2	7	8.721	1.04	40	d	0.000434
			227744_s_at	0	3.971	0.6	7	3.951	0.57	40	d	0.933751
			209330_s_at	0	9.718	0.16	7	9.287	0.83	40	d	0.005561
			221480_at	0	8.057	0.34	7	7.796	0.56	40	d	0.248687
SLC10A7	3	solute carrier family 10 (sodium/bile acid cotransporter family), member 7	235143_at	3	5.965	0.33	7	6.272	0.5	40	u	0.130911
			224126_at	0	2.681	0.17	7	2.856	0.3	40	u	0.150951
			1555108_at	0	2.361	0.12	7	2.494	0.24	40	u	0.17174
DLEU7	3	deleted in lymphocytic leukemia, 7	233277_at	0	2.128	0.01	7	2.187	0.12	40	u	0.00485
TRIB3	3	tribbles homolog 3 (Drosophila)	218145_at	3	7.39	0.35	7	7.946	1.31	40	u	0.034736
			1555788_a_at	0	4.624	0.45	7	5.292	1.36	40	u	0.027471
MIER1	3	mesoderm induction early response 1 homolog (Xenopus laevis)	225475_at	3	8.113	0.34	7	8.101	0.65	40	d	0.9642
			240227_at	0	2.175	0.02	7	2.224	0.04	40	u	0.008067
			1555105_a_at	0	6.001	0.42	7	6.334	0.67	40	u	0.22279
GDA	3	guanine deaminase	224209_s_at	3	2.273	0.03	7	2.412	0.19	40	u	0.000179
			1569555_at	3	2.413	0.03	7	2.62	0.32	40	u	0.000296
PHYHIP	3	phytanoyl-CoA 2-hydroxylase interacting protein	205325_at	3	4.398	0.51	7	3.104	0.37	40	d	0
COPS7A	3	COP9 constitutive photomorphogenic homolog subunit 7A (Arabidopsis)	209029_at	3	8.532	0.27	7	8.555	0.83	40	u	0.898849
C15orf41	3	chromosome 15 open reading frame 41	224486_s_at	3	4.148	0.4	7	4.205	0.69	40	u	0.834391
			232506_s_at	0	3.506	0.34	7	4.1	0.48	40	u	0.003666
			232507_at	0	2.546	0.07	7	2.622	0.15	40	u	0.210042
ATXN10	3	ataxin 10	208832_at	3	7.308	0.28	7	6.825	0.79	40	d	0.008638
			208833_s_at	0	10.85	0.23	7	10.88	0.51	40	u	0.886464
TRPM6	3	transient receptor potential cation channel, subfamily M, member 6	240389_at	3	2.733	0.31	7	2.685	0.41	40	d	0.7765
			224412_s_at	3	2.195	0.12	7	2.449	0.68	40	u	0.039037
			221102_s_at	3	2.351	0.09	7	2.623	0.23	40	u	0.004395

			234864_s_at	0	2.621	0.14	7	2.627	0.11	40	u	0.89414
FOXK2	3	forkhead box K2	203064_s_at	3	7.389	0.17	7	8.12	0.8	40	u	0.00001
			226224_at	0	7.156	0.18	7	7.56	0.73	40	u	0.005986
			242938_s_at	0	4.317	0.47	7	5.41	1.28	40	u	0.033591
PSCD3	3	pleckstrin homology, Sec7 and coiled-coil domains 3	225147_at	3	8.54	0.23	7	7.877	0.73	40	d	0.00013
			206523_at	2	4.302	0.67	7	3.981	0.78	40	d	0.319638
			243752_s_at	0	2.138	0.19	7	2.257	0.41	40	u	0.471263
			236136_at	0	4.747	0.23	7	4.548	0.28	40	d	0.087295
PPIL1	3	peptidylprolyl isomerase (cyclophilin)-like 1	222500_at	3	7.204	0.31	7	8.295	0.71	40	u	0.000315
USP14	3	ubiquitin specific peptidase 14 (tRNA-guanine transglycosylase)	201671_x_at	3	8.267	0.37	7	8.511	0.95	40	u	0.515088
			201672_s_at	3	9.224	0.3	7	10.12	0.76	40	u	0.004156
			226567_at	0	8.962	0.36	7	9.001	0.9	40	u	0.911753
ALG2	3	asparagine-linked glycosylation 2 homolog (S. cerevisiae, alpha-1,3-	225621_at	3	8.956	0.26	7	8.704	0.66	40	d	0.334
			228166_at	0	2.191	0.52	7	2.292	0.56	40	u	0.667889
PLXNA1	3	plexin A1	221537_at	3	6.534	0.17	7	6.579	0.66	40	u	0.716438
			221538_s_at	3	8.11	0.58	7	7.316	1.03	40	d	0.05908
			1558140_at	0	2.133	0.06	7	2.37	0.51	40	u	0.007698
CHFR	3	checkpoint with forkhead and ring finger domains	218803_at	3	7.729	0.21	7	7.902	0.82	40	u	0.277426
			223931_s_at	2	6.439	0.19	7	6.662	0.68	40	u	0.108068
ID1	3	inhibitor of DNA binding 1, dominant negative helix-loop-helix protein	208937_s_at	0	8.532	1.08	7	6.349	1.54	40	d	0.00098
CDCA4	3	cell division cycle associated 4	218399_s_at	3	6.614	0.4	7	7.311	0.73	40	u	0.020788
LUC7L2	3	LUC7-like 2 (S. cerevisiae)	217842_at	3	8.165	0.27	7	7.798	0.75	40	d	0.033244
			220099_s_at	1	9.378	0.36	7	8.997	0.72	40	d	0.188057
			243852_at	0	7.46	0.37	7	7.639	0.53	40	u	0.404313
			226758_at	0	8.926	0.24	7	8.823	0.61	40	d	0.66915
CDC42EP3	3	CDC42 effector protein (Rho GTPase binding) 3	209288_s_at	3	7.603	0.3	7	7.241	1.32	40	d	0.143111
			209286_at	3	7.489	0.4	7	6.121	1.49	40	d	0.000037
			209287_s_at	0	6.229	0.39	7	5.987	1.11	40	d	0.317509
PCDH18	3	protocadherin 18	225977_at	3	4.285	0.81	7	2.972	0.69	40	d	0.000062
			225975_at	3	9.794	0.43	7	7.166	1.02	40	d	0
ATF7	3	activating transcription factor 7	206684_s_at	3	2.895	0.51	7	2.929	0.52	40	u	0.87722
			228830_s_at	0	5.689	0.63	7	5.733	0.97	40	u	0.910497
			228829_at	0	5.373	0.45	7	5.62	1	40	u	0.533326
KCNQ5	3	potassium voltage-gated channel, KQT-like subfamily, member 5	223891_at	1	2.324	0.03	7	2.461	0.28	40	u	0.004627
DOCK11	3	dedicator of cytokinesis 11	226875_at	3	7.353	0.71	7	6.884	1.13	40	d	0.306176
			238356_at	0	2.866	0.13	7	3.153	0.45	40	u	0.003371
FKBP4	3	FK506 binding protein 4, 59kDa	200895_s_at	3	9.741	0.34	7	10.03	0.93	40	u	0.161255
			200894_s_at	3	8.514	0.5	7	8.811	1.15	40	u	0.515325
AHNAK	3	AHNAK nucleoprotein	211986_at	3	12.24	0.29	7	10.1	1.17	40	d	0
			235281_x_at	0	5.48	0.45	7	5.776	0.87	40	u	0.393196
			238855_at	0	4.037	0.26	7	3.88	0.42	40	d	0.355879
			1563560_at	0	2.464	0.15	7	2.626	0.32	40	u	0.201611
			220016_at	0	6.812	1.01	7	5.259	1.36	40	d	0.006912
YIPF2	3	Yip1 domain family, member 2	221939_at	8	7.476	0.55	7	8.186	0.83	40	u	0.037737
			219075_at	3	5.611	0.47	7	6.084	0.98	40	u	0.227691
			65086_at	3	7.034	0.48	7	7.57	0.79	40	u	0.094183
CCDC109A	3	coiled-coil domain containing 109A	225320_at	3	6.068	0.42	7	6.502	0.75	40	u	0.150126
AHR	3	aryl hydrocarbon receptor	202820_at	2	9.513	0.35	7	9.018	1.04	40	d	0.032491
KCNA4	3	potassium voltage-gated channel, shaker-related subfamily, member 4	207248_at	0	2.689	0.09	7	2.774	0.13	40	u	0.120372
RBM6	3	RNA binding motif protein 6	201967_at	3	8.519	0.31	7	7.541	0.83	40	d	0.004209
			228030_at	0	8.744	0.41	7	7.864	1.55	40	d	0.005911
			1556672_a_at	0	5.451	0.51	7	5.549	0.76	40	u	0.75006
PSMF1	3	proteasome (prosome, macropain) inhibitor subunit 1 (PI31)	201052_s_at	3	8.698	0.3	7	8.691	0.64	40	d	0.978139
			201053_s_at	3	8.221	0.39	7	8.147	0.51	40	d	0.724583
LMO4	3	LIM domain only 4	209204_at	3	6.975	0.27	7	8.185	1.48	40	u	0.000032
			227155_at	3	4.533	0.33	7	4.899	1.27	40	u	0.145135
			209205_s_at	3	7.59	0.4	7	9.274	1.79	40	u	0.000008
PRDM12	3	PR domain containing 12	220894_x_at	0	2.887	0.3	7	2.689	0.24	40	d	0.067912
SCHIP1	3	schwannomin interacting protein 1	204030_s_at	3	7.619	0.79	7	7.202	1.42	40	d	0.462785
			217877_s_at	2	9.545	0.26	7	9.083	0.49	40	d	0.021868

GPBP1L1	3	GC-rich promoter binding protein 1-like 1	222452_s_at	2	9.546	0.37	7	9.221	0.67	40	d	0.229556
			1569320_at	0	6.434	0.74	7	6.291	1.08	40	d	0.743995
C19orf6	3	chromosome 19 open reading frame 6	225247_at	3	8.209	0.24	7	7.613	0.61	40	d	0.015903
			213985_s_at	0	2.825	0.26	7	2.758	0.27	40	d	0.54594
			212574_x_at	0	6.914	0.65	7	6.14	1.58	40	d	0.21878
			213986_s_at	0	7.015	0.5	7	6.104	1.63	40	d	0.009911
			212575_at	0	4.032	0.53	7	4.051	0.79	40	u	0.952817
			230089_s_at	0	3.18	0.31	7	3.265	0.41	40	u	0.61012
MBOAT5	3	membrane bound O-acyltransferase domain containing 5	202793_at	3	7.554	0.68	7	6.798	0.88	40	d	0.03965
			213615_at	0	3.787	0.48	7	3.956	0.82	40	u	0.60731
CUL4B	3	cullin 4B	202214_s_at	3	9.521	0.15	7	9.293	0.5	40	d	0.031834
			202213_s_at	3	7.851	0.53	7	7.967	1	40	u	0.771238
			210257_x_at	0	6.649	0.48	7	6.892	0.9	40	u	0.499328
			215997_s_at	0	6.518	0.4	7	6.459	1.14	40	d	0.810721
LDLRAD2	3	nsity lipoprotein receptor class A domain conta	no probeset available									
CXorf23	3	chromosome X open reading frame 23	232087_at	3	5.049	0.29	7	5.204	0.5	40	u	0.441346
APBA2	3	amyloid beta (A4) precursor protein-binding, family A, member 2 (X11-like)	209870_s_at	3	5.728	0.58	7	6.227	0.84	40	u	0.14598
			209871_s_at	3	3.343	0.51	7	3.951	1.19	40	u	0.198204
SRML2	3	spermidine synthase-like 2	no probeset available									
TMED7	3	transmembrane emp24 protein transport domain containing 7	209404_s_at	3	8.529	0.16	7	8.708	0.71	40	u	0.176577
			214658_at	3	8.143	0.26	7	8.233	0.86	40	u	0.606042
PI15	3	peptidase inhibitor 15	229947_at	3	10.88	1.29	7	5.881	2.4	40	d	0.000004
			207938_at	0	4.647	1.53	7	3.11	1.83	40	d	0.046422
SIX2	3	SIX homeobox 2	206510_at	3	3.319	0.52	7	5.212	1.76	40	u	0.000009
			206511_s_at	1	3.514	0.41	7	4.789	1.24	40	u	0.000045
FBXL20	3	F-box and leucine-rich repeat protein 20	235089_at	3	4.73	0.45	7	6.014	1.46	40	u	0.000186
			239224_at	0	3.647	0.39	7	3.59	0.9	40	d	0.872853
			239223_s_at	0	5.315	0.57	7	4.968	1.5	40	d	0.557201
HSPA2	3	heat shock 70kDa protein 2	211538_s_at	3	8.392	0.73	7	6.943	1.68	40	d	0.032844
TM9SF4	3	transmembrane 9 superfamily protein member 4	212198_s_at	3	8.232	0.38	7	8.029	0.8	40	d	0.519878
			212194_s_at	3	7.405	0.24	7	7.467	0.83	40	u	0.7073
SUMF1	3	sulfatase modifying factor 1	226850_at	3	9.376	0.44	7	8.898	0.87	40	d	0.170771
MAP4K3	3	mitogen-activated protein kinase kinase kinase kinase 3	218311_at	3	7.751	0.29	7	7.843	0.69	40	u	0.736151
SNTA1	3	syntrophin, alpha 1 (dystrophin-associated protein A1, 59kDa, acidic component)	203516_at	3	5.632	0.15	7	5.422	0.8	40	d	0.148837
KDELR1	3	KDEL (Lys-Asp-Glu-Leu) endoplasmic reticulum protein retention receptor 1	1555575_a_at	3	10.9	0.11	7	10.69	0.52	40	d	0.029525
			200922_at	0	8.715	0.17	7	8.481	1.01	40	d	0.189736
ZNF533	3	zinc finger protein 533	229019_at	3	6.448	1.03	7	5.033	0.92	40	d	0.000798
			1555800_at	1	4.804	1.96	7	2.792	0.94	40	d	0.048511
			1555801_s_at	1	4.314	1.32	7	2.967	0.52	40	d	0.048675
FAM129B	3	family with sequence similarity 129, member B	223019_at	3	8.898	0.2	7	8.585	0.61	40	d	0.022139
			233974_s_at	3	7.582	0.16	7	7.508	0.41	40	d	0.644214
ACLY	3	ATP citrate lyase	201127_s_at	3	8.065	0.36	7	8.024	0.7	40	d	0.882111
			201128_s_at	3	10.64	0.34	7	10.68	0.77	40	u	0.908674
			210337_s_at	1	8.653	0.47	7	8.428	1.32	40	d	0.436823
HPCAL4	3	hippocalcin like 4	222091_at	2	5.052	0.35	7	4.976	0.31	40	d	0.568702
			219671_at	0	2.35	0.06	7	2.518	0.29	40	u	0.002786
MEX3D	3	mex-3 homolog D (C. elegans)	91816_f_at	3	7.091	0.53	7	7.17	1.46	40	u	0.805321
			222042_x_at	0	5.192	0.32	7	5.103	0.52	40	d	0.668651
			244712_at	0	2.348	0.19	7	2.39	0.31	40	u	0.733618
KIAA0232	3	KIAA0232	212441_at	3	9.549	0.27	7	8.808	0.98	40	d	0.000474
			232366_at	3	4.602	0.68	7	4.134	1.05	40	d	0.270655
KIAA0368	3	KIAA0368	214356_s_at	3	8.997	0.28	7	8.953	0.6	40	d	0.854506
			212427_at	0	5.265	0.39	7	4.414	0.96	40	d	0.029166
			212428_at	0	8.525	0.2	7	8.419	0.61	40	d	0.413594
			236368_at	0	6.108	0.21	7	6.296	0.72	40	u	0.198518
GCH1	3	GTP cyclohydrolase 1 (dopa-responsive dystonia)	204224_s_at	3	7.193	0.41	7	7.959	1.27	40	u	0.007421
CSNK2A2	3	casein kinase 2, alpha prime polypeptide	224922_at	3	7.36	0.27	7	7.258	0.64	40	d	0.682054
			203575_at	0	7.52	0.18	7	6.857	0.87	40	d	0.000133
RNF43	3	ring finger protein 43	218704_at	3	7.217	0.58	7	6.205	1.24	40	d	0.043569
ZNF536	3	zinc finger protein 536	206403_at	3	2.133	0.04	7	2.188	0.08	40	u	0.109825
			228448_at	3	3.034	0.43	7	3.018	0.54	40	d	0.943829

MAP6	3	microtubule-associated protein 6	241980_at	1	3.257	0.58	7	3.122	0.53	40	d	0.555253
			235672_at	1	2.463	0.13	7	2.744	0.64	40	u	0.019636
			228943_at	0	4.135	0.26	7	4.202	0.51	40	u	0.740561
HN1	3	hematological and neurological expressed 1	222396_at	3	7.637	0.49	7	9.231	1.06	40	u	0.000406
			217755_at	3	7.523	0.47	7	9.92	1.2	40	u	0.000006
SUPT16H	3	suppressor of Ty 16 homolog (S. cerevisiae)	217815_at	3	9.402	0.15	7	9.241	0.49	40	d	0.114767
			233827_s_at	0	5.494	0.88	7	7.012	1.37	40	u	0.007967
SOCS3	3	suppressor of cytokine signaling 3	227697_at	3	7.106	1.32	7	6.395	1.1	40	d	0.142509
			206359_at	1	8.086	1.17	7	7.212	0.88	40	d	0.029855
			206360_s_at	1	6.366	0.96	7	5.883	0.75	40	d	0.149031
			214105_at	0	2.874	0.52	7	2.723	0.49	40	d	0.469676
OTUB2	3	OTU domain, ubiquitin aldehyde binding 2	222878_s_at	3	2.228	0.11	7	2.352	0.36	40	u	0.10171
			219369_s_at	3	3.774	0.33	7	3.958	0.64	40	u	0.469056
			1555682_at	0	1.937	0.01	7	2.021	0.21	40	u	0.016431
RAI16	3	retinoic acid induced 16	218337_at	3	7.621	0.34	7	6.007	1.03	40	d	0
			222928_s_at	0	2.414	0.02	7	2.449	0.04	40	u	0.018652
			222929_at	0	3.214	0.42	7	3.22	0.46	40	u	0.973841
AUH	3	AU RNA binding protein/enoyl-Coenzyme A hydratase	205052_at	3	8.8	0.39	7	7.615	0.93	40	d	0.00229
PANK3	3	pantothenate kinase 3	218433_at	3	5.669	0.17	7	6.345	0.8	40	u	0.000039
TRIP10	3	thyroid hormone receptor interactor 10	202734_at	3	8.115	0.44	7	7.678	0.81	40	d	0.18032
AMPD3	3	adenosine monophosphate deaminase (isoform E)	207992_s_at	3	6.496	0.42	7	6.683	0.79	40	u	0.551345
			209491_s_at	3	2.467	0.17	7	2.534	0.27	40	u	0.538837
ST6GALNAC 3	3	ST6 (alpha-N-acetyl-neuraminyl-2,3-beta-galactosyl-1,3)-N-acetylgalactosaminide alpha-2,6-sialyltransferase 3	235334_at	3	3.84	0.64	7	3.47	0.89	40	d	0.309102
MAN1A2	3	mannosidase, alpha, class 1A, member 2	217920_at	2	5.653	0.24	7	6.366	0.94	40	u	0.000355
			1552481_s_at	1	3.805	0.49	7	4.833	1.04	40	u	0.015811
			214242_at	0	2.355	0.03	7	2.407	0.07	40	u	0.003325
			217282_at	0	3.355	0.28	7	3.575	0.41	40	u	0.184396
CTDP1	3	CTD (carboxy-terminal domain, RNA polymerase II, polypeptide A) phosphatase, subunit 1	205035_at	3	6.211	0.26	7	5.94	0.66	40	d	0.303465
STMN1	3	stathmin 1/oncoprotein 18	200783_s_at	3	5.826	0.35	7	7.834	0.82	40	u	0
			217714_x_at	2	8.16	0.48	7	8.836	0.53	40	u	0.003494
GTPBP2	3	GTP binding protein 2	221050_s_at	3	6.682	0.27	7	7.15	0.89	40	u	0.014239
			223789_s_at	0	4.023	0.58	7	4.884	1.58	40	u	0.170572
SETD3	3	SET domain containing 3	212465_at	3	8.076	0.2	7	7.536	0.58	40	d	0.000201
			229940_at	0	4.005	0.4	7	4.619	0.63	40	u	0.019015
RHO	3	rhodopsin (opsin 2, rod pigment) (retinitis pigmentosa 4, autosomal dominant)	206454_s_at	3	2.281	0.03	7	2.341	0.06	40	u	0.014587
			206455_s_at	3	6.212	0.26	7	6.273	0.36	40	u	0.680835
DNAJB11	3	DnaJ (Hsp40) homolog, subfamily B, member 11	223054_at	0	9.291	0.16	7	9.819	0.7	40	u	0.000226
SLC22A5	3	solute carrier family 22 (organic cation transporter), member 5	205074_at	3	6.966	0.21	7	5.986	1.41	40	d	0.000206
C14orf24	3	chromosome 14 open reading frame 24	227029_at	3	8.292	0.3	7	8.57	0.56	40	u	0.219374
PPARD	3	peroxisome proliferator-activated receptor delta	37152_at	3	6.78	0.23	7	7.039	0.71	40	u	0.091636
			208044_s_at	3	6.51	0.17	7	6.387	0.38	40	d	0.417578
			210636_at	0	2.733	0.05	7	2.745	0.06	40	u	0.63914
ANXA2	3	annexin A2	210427_x_at	3	13.47	0.31	7	13.27	0.65	40	d	0.441551
			201590_x_at	3	13.41	0.29	7	13.23	0.65	40	d	0.474557
			213503_x_at	3	13.24	0.34	7	13.03	0.67	40	d	0.433644
			1568126_at	0	2.774	0.12	7	2.983	0.24	40	u	0.031733
HLCS	3	holocarboxylase synthetase (biotin-(proprionyl-Coenzyme A-carboxylase (ATP-hydrolysing)) ligase)	209399_at	2	5.796	0.33	7	5.585	0.45	40	d	0.249679
			241726_at	0	3.018	0.2	7	3.905	1.07	40	u	0.000032
			207833_s_at	0	3.527	0.35	7	4.261	0.74	40	u	0.015763
SYNPO2L	3	synaptopodin 2-like	243313_at	3	3.91	1.87	7	3.249	0.58	40	d	0.423136
			219804_at	3	3.34	1.68	7	2.767	0.19	40	d	0.435552
BMF	3	Bcl2 modifying factor	226530_at	3	4.792	0.55	7	5.262	0.91	40	u	0.202064
LRRC32	3	leucine rich repeat containing 32	203835_at	3	5.841	0.66	7	5.174	0.84	40	d	0.057546
LMNB1	3	lamin B1	203276_at	3	5.822	0.64	7	8.598	1.17	40	u	0
C9orf72	3	chromosome 9 open reading frame 72	225919_s_at	3	7.399	0.64	7	7.209	0.73	40	d	0.528921
			1553133_at	0	4.754	0.97	7	4.644	0.68	40	d	0.722306
			1553134_s_at	0	2.309	0.03	7	2.491	0.36	40	u	0.003557

PPP5C	3	protein phosphatase 5, catalytic subunit	201979_s_at	0	6.481	0.25	7	6.681	0.52	40	u	0.336681
			215705_at	0	4.074	0.47	7	4.013	0.51	40	d	0.775713
HRBL	3	HIV-1 Rev binding protein-like	222126_at	3	5.256	0.59	7	3.971	1.04	40	d	0.003134
			1554618_at	0	2.053	0.04	7	2.144	0.18	40	u	0.009388
			206820_at	0	4.6	0.39	7	4.432	0.49	40	d	0.406331
			206821_x_at	0	3.243	0.21	7	2.9	0.51	40	d	0.093855
			222362_at	0	2.92	0.37	7	2.611	0.36	40	d	0.045828
SH3PXD2B	3	SH3 and PX domains 2B	231823_s_at	3	8.98	0.4	7	7.882	0.63	40	d	0.00008
			1562910_at	0	2.405	0.12	7	2.434	0.18	40	u	0.690835
SERTAD4	3	SERTA domain containing 4	229674_at	3	7.953	0.65	7	8.083	1.74	40	u	0.850408
			230660_at	0	6.66	0.75	7	7.38	1.54	40	u	0.242599
KLHDC3	3	kelch domain containing 3	214383_x_at	3	9.364	0.27	7	9.351	1.2	40	d	0.953804
			208784_s_at	3	8.96	0.11	7	9.285	0.97	40	u	0.049845
VPS4B	3	vacuolar protein sorting 4 homolog B (S. cerevisiae)	218171_at	3	9.39	0.19	7	9.371	0.67	40	d	0.887669
ZDHHC3	3	zinc finger, DHHC-type containing 3	218077_s_at	3	6.003	0.49	7	5.932	0.73	40	d	0.806815
			218078_s_at	3	7.228	0.39	7	6.832	0.69	40	d	0.153959
			236507_at	0	4.064	0.27	7	4.08	0.34	40	u	0.908541
SESN1	3	sestrin 1	218346_s_at	3	8.634	0.36	7	7.006	0.9	40	d	0.000031
			236512_at	0	4.577	0.32	7	3.948	0.52	40	d	0.004344
CLASP1	3	cytoplasmic linker associated protein 1	212752_at	3	7.597	0.31	7	7.371	0.75	40	d	0.444778
			240757_at	0	3.725	0.24	7	4.237	0.94	40	u	0.007541
RASGEF1B	3	RasGEF domain family, member 1B	1553785_at	3	3.109	0.08	7	3.338	0.39	40	u	0.002464
			1554999_at	0	2.258	0.29	7	2.396	0.53	40	u	0.518848
ACOT11	3	acyl-CoA thioesterase 11	214763_at	3	5.637	0.25	7	5.723	0.76	40	u	0.594089
			1555321_at	0	3.081	0.28	7	3.031	0.35	40	d	0.722441
			216103_at	0	3.913	0.37	7	3.954	0.48	40	u	0.834841
			1554938_a_at	0	3.938	0.28	7	3.841	0.42	40	d	0.569799
CAPN2	3	calpain 2, (m/II) large subunit	208683_at	3	11.47	0.26	7	11.43	0.95	40	d	0.838914
			214888_at	0	4.4	0.59	7	4.638	0.74	40	u	0.435195
FAM134A	3	family with sequence similarity 134, member A	218037_at	3	8.807	0.26	7	8.627	0.61	40	d	0.458278
			222129_at	0	8.175	0.3	7	7.194	0.57	40	d	0.000082
			221983_at	0	8.134	0.22	7	7.858	0.71	40	d	0.069079
			221984_s_at	0	9.103	0.24	7	9.153	0.68	40	u	0.739739
SH3PX3	3	SH3 and PX domain containing 3	227151_at	3	7.213	0.21	7	6.466	0.42	40	d	0.000048
ZDHHC20	3	zinc finger, DHHC-type containing 20	243786_at	3	2.829	0.3	7	3.574	1	40	u	0.000867
			225365_at	0	9.526	0.17	7	10.14	0.72	40	u	0.00006
ITPKB	3	inositol 1,4,5-trisphosphate 3-kinase B	203723_at	3	7.932	0.52	7	7.738	1.13	40	d	0.665846
			235213_at	0	5.422	0.89	7	5.574	1.04	40	u	0.724234
			232526_at	0	2.893	0.27	7	2.795	0.39	40	d	0.541087
			232499_at	0	3.135	0.21	7	3.185	0.35	40	u	0.721903
			1554306_at	0	3.009	0.23	7	3.404	0.54	40	u	0.067991
MEOX2	3	mesenchyme homeobox 2	206202_at	3	2.339	0.02	7	2.413	0.09	40	u	0.000136
			206201_s_at	3	5.858	0.81	7	3.554	0.4	40	d	0.000486
ARHGEF4	3	Rho guanine nucleotide exchange factor (GEF) 4	205109_s_at	3	3.68	0.7	7	3.379	0.98	40	d	0.451685
			216912_at	3	2.069	0.02	7	2.107	0.05	40	u	0.075519
			211891_s_at	0	2.416	0.22	7	2.438	0.45	40	u	0.903233
PDE5A	3	phosphodiesterase 5A, cGMP-specific	227088_at	3	6.826	0.33	7	4.358	1.13	40	d	0
			206757_at	1	3.624	0.36	7	3.593	0.68	40	d	0.909615
			1553175_s_at	1	2.685	0.17	7	2.846	0.46	40	u	0.378796
			1562227_at	0	1.825	0.27	7	1.816	0.35	40	d	0.951343
			1562228_s_at	0	3.458	0.26	7	3.937	0.56	40	u	0.034252
TNFRSF21	3	tumor necrosis factor receptor superfamily, member 21	218856_at	3	9.198	0.52	7	8.457	0.94	40	d	0.053237
			214581_x_at	1	7.296	0.61	7	6.615	1.51	40	d	0.255263
CCT3	3	chaperonin containing TCP1, subunit 3 (gamma)	200910_at	3	10.94	0.14	7	11.95	0.57	40	u	0
TRIOBP	3	TRIO and F-actin binding protein	202795_x_at	3	8.139	0.19	7	7.255	0.54	40	d	0
			210276_s_at	3	9.334	0.15	7	8.022	0.74	40	d	0
			216210_x_at	3	8.825	0.25	7	7.921	0.41	40	d	0.000001
			1552334_at	0	4.676	0.66	7	4.475	0.73	40	d	0.508631
			1552332_at	0	5.48	0.26	7	5.351	0.3	40	d	0.302908
			243690_at	0	7.01	0.54	7	6.274	0.56	40	d	0.00276
IER5	3	immediate early response 5	218611_at	3	9.821	0.26	7	9.27	0.75	40	d	0.00207
SPTBN2	3	spectrin, beta, non-erythrocytic 2	205155_s_at	3	5.905	0.48	7	6.076	0.82	40	u	0.603711

STAU1	3	staufen, RNA binding protein, homolog 1 (Drosophila)	207320_x_at	3	11.01	0.2	7	11.07	0.45	40	u	0.710026
			211505_s_at	3	8.86	0.23	7	9.186	0.66	40	u	0.030797
			213037_x_at	3	11.09	0.23	7	11.13	0.48	40	u	0.79671
			208948_s_at	3	10.92	0.26	7	11	0.48	40	u	0.673217
GAS2	3	growth arrest-specific 2	205848_at	3	3.475	0.58	7	3.212	1.31	40	d	0.611331
RHOG	3	ras homolog gene family, member G (rho G)	203175_at	3	8.351	0.25	7	8.152	0.64	40	d	0.429673
TRPM3	3	transient receptor potential cation channel, subfamily M, member 3	216489_at	3	1.827	0.05	7	1.888	0.09	40	u	0.08628
			216452_at	3	1.94	0.27	7	1.874	0.06	40	d	0.57152
			1555252_a_at	0	2.418	0.12	7	2.426	0.17	40	u	0.908308
			220463_at	0	2.397	0.07	7	2.568	0.26	40	u	0.002026
			233022_at	0	4.373	0.38	7	4.345	0.41	40	d	0.869709
			1554722_at	0	2.993	0.21	7	3.297	0.36	40	u	0.037194
			211422_at	0	2.531	0.07	7	2.614	0.23	40	u	0.089918
ZMYM4	3	zinc finger, MYM-type 4	202049_s_at	3	8.336	0.24	7	8.775	0.52	40	u	0.037903
			202051_s_at	3	8.748	0.28	7	8.395	0.46	40	d	0.060794
			202050_s_at	3	8.157	0.31	7	8.061	0.48	40	d	0.613362
LRRIQ2	3	leucine-rich repeats and IQ motif containing 2	219886_at	3	3.554	0.29	7	4.137	0.68	40	u	0.033286
UBE2A	3	ubiquitin-conjugating enzyme E2A (RAD6 homolog)	201898_s_at	3	8.352	0.42	7	8.922	0.92	40	u	0.120676
			201899_s_at	3	9.388	0.22	7	9.831	0.51	40	u	0.033274
DDHD1	3	DDHD domain containing 1	225970_at	3	4.549	0.72	7	4.999	0.79	40	u	0.17446
			225965_at	3	4.272	0.44	7	5.011	0.59	40	u	0.003345
			233444_at	0	1.988	0.02	7	2.025	0.04	40	u	0.015862
			243705_at	0	3.864	0.5	7	4.079	0.49	40	u	0.304212
CCDC21	3	coiled-coil domain containing 21	227818_at	3	6.394	0.29	7	7.018	0.64	40	u	0.016992
			219611_s_at	3	6.238	0.22	7	6.663	0.56	40	u	0.05948
CBX3	3	chromobox homolog 3 (HP1 gamma homolog, Drosophila)	200037_s_at	3	11.16	0.24	7	11.82	0.37	40	u	0.000058
			201091_s_at	3	8.932	0.26	7	10.2	0.72	40	u	0
			1555920_at	0	5.768	0.59	7	7.157	0.92	40	u	0.000473
CLIP3	3	CAP-GLY domain containing linker protein 3	212358_at	3	6.936	0.25	7	5.031	0.91	40	d	0
			235243_at	0	2.57	0.29	7	2.319	0.33	40	d	0.075127
PRDM5	3	PR domain containing 5	220792_at	0	2.367	0.08	7	2.538	0.33	40	u	0.010117
			1569508_at	0	3.982	0.16	7	4.251	0.36	40	u	0.063414
PDIA6	3	protein disulfide isomerase family A, member 6	208638_at	6	10.62	0.34	7	11.12	0.65	40	u	0.061004
			208639_x_at	6	10.52	0.24	7	11.14	0.77	40	u	0.000449
			207668_x_at	6	10.59	0.26	7	11.15	0.76	40	u	0.001995
			216640_s_at	3	10.18	0.25	7	10.77	0.9	40	u	0.001793
CENPB	3	centromere protein B, 80kDa	212437_at	3	8.319	0.24	7	8.027	0.71	40	d	0.06557
GATA2	3	GATA binding protein 2	209710_at	3	6.91	0.31	7	6.073	1.17	40	d	0.000781
			210358_x_at	2	3.219	0.22	7	3.281	0.5	40	u	0.752523
			207954_at	2	3.342	0.27	7	3.489	0.35	40	u	0.304508
ADCY2	3	adenylate cyclase 2 (brain)	213219_at	3	2.392	0.09	7	2.478	0.14	40	u	0.143762
			213217_at	3	5.239	0.69	7	5.241	2.05	40	u	0.995751
			217687_at	0	2.504	0.36	7	2.978	0.63	40	u	0.064253
PPM1G	3	protein phosphatase 1G (formerly 2C), magnesium-dependent, gamma isoform	200913_at	1	8.396	0.2	7	8.81	0.69	40	u	0.005562
FZD3	3	frizzled homolog 3 (Drosophila)	219683_at	0	6.659	0.69	7	6.183	0.97	40	d	0.230489
PER1	3	period homolog 1 (Drosophila)	36829_at	3	7.676	0.56	7	5.749	0.66	40	d	0
			202861_at	3	8.164	0.69	7	5.429	0.93	40	d	0
			242832_at	0	2.187	0.29	7	2.109	0.07	40	d	0.53348
KLC2	3	kinesin light chain 2	218906_x_at	3	6.188	0.45	7	6.808	1.21	40	u	0.198306
TMEM135	3	transmembrane protein 135	222209_s_at	3	8.459	0.66	7	7.894	1.03	40	d	0.175416
			1554866_at	0	2.68	0.14	7	2.741	0.21	40	u	0.474401
DCHS1	3	dachsous 1 (Drosophila)	218892_at	3	5.461	0.36	7	3.802	1.06	40	d	0
			222101_s_at	3	7.031	0.47	7	5.693	1.27	40	d	0.00987
ADNP	3	activity-dependent neuroprotector homeobox	201773_at	3	9.69	0.4	7	9.985	0.65	40	u	0.264592
DMWD	3	dystrophia myotonica, WD repeat containing	33768_at	3	7.61	0.24	7	7.639	0.67	40	u	0.843901
			213231_at	3	5.159	0.25	7	4.995	0.77	40	d	0.316655
			1554429_a_at	3	7.347	0.35	7	7.25	0.72	40	d	0.737004
RFFL	3	ring finger and FYVE-like domain containing	1566171_at	3	5.824	0.4	7	5.821	0.45	40	d	0.98888
			228980_at	3	7.356	0.39	7	7.819	0.59	40	u	0.055469
			1552651_a_at	2	5.016	0.32	7	5.324	0.6	40	u	0.200856

[illegible]	[illegible]	1	1552649_a_at	2	2.506	0.24	7	2.523	0.48	40	u	0.928453
			237919_at	2	5.354	0.33	7	5.517	0.5	40	u	0.42282
			1562603_at	2	3.095	0.29	7	3.311	0.58	40	u	0.354498
TLK2	3	tousled-like kinase 2	212997_s_at	3	7.196	0.31	7	7.766	0.85	40	u	0.094126
			212986_s_at	3	6.66	0.32	7	7.028	1.04	40	u	0.091252
			233349_at	3	2.277	0.11	7	2.362	0.2	40	u	0.288802
			228627_at	0	5.028	0.74	7	4.941	0.91	40	d	0.816661
			232585_at	0	2.882	0.13	7	2.99	0.15	40	u	0.079039
IGFBP3	3	insulin-like growth factor binding protein 3	212143_s_at	1	8.045	0.39	7	7.444	1.3	40	d	0.029074
			210095_s_at	1	9.99	0.42	7	9.349	1.09	40	d	0.1413
PLP1	3	proteolipid protein 1 (Pelizaeus-Merzbacher disease, spastic paraplegia 2, uncomplicated)	210198_s_at	2	4.461	0.79	7	3.248	0.8	40	d	0.000702
ANKRD43	3	ankyrin repeat domain 43	230238_at	3	2.867	0.28	7	2.917	0.51	40	u	0.80612
MAP7D1	3	MAP7 domain containing 1	217943_s_at	3	8.972	0.21	7	8.29	0.68	40	d	0.000037
COL1A2	3	collagen, type I, alpha 2	202404_s_at	3	10.72	0.88	7	12.06	1.41	40	u	0.02152
			202403_s_at	3	12.53	0.5	7	12.77	1.29	40	u	0.645835
			229218_at	0	7.591	0.64	7	7.324	2.12	40	d	0.539095
TBC1D8B	3	TBC1 domain family, member 8B (with GRAM domain)	238067_at	3	4.627	0.57	7	4.753	0.84	40	u	0.713032
			219771_at	2	5.83	0.49	7	5.875	0.87	40	u	0.895708
			1563272_at	0	3.293	0.22	7	3.23	0.31	40	d	0.614665
HEPACAM	3	hepatocyte cell adhesion molecule	238003_at	3	2.679	0.46	7	2.5	0.23	40	d	0.386244
RBAK	3	RB-associated KRAB zinc finger	1553122_s_at	3	3.282	0.43	7	4.193	0.82	40	u	0.007456
			228571_at	0	7.935	0.25	7	7.847	0.74	40	d	0.582049
			241366_at	0	3.834	0.71	7	4.331	0.8	40	u	0.139045
GULP1	3	GULP, engulfment adaptor PTB domain containing 1	204237_at	3	8.461	0.55	7	6.964	1.1	40	d	0.001203
			215913_s_at	0	6.08	0.99	7	4.676	1.25	40	d	0.008166
			204235_s_at	0	7.27	0.72	7	5.753	1.31	40	d	0.005286
			215915_at	0	2.148	0.03	7	2.202	0.07	40	u	0.060702
			223837_at	0	3.788	0.49	7	3.945	0.47	40	u	0.436149
KCNS3	3	potassium voltage-gated channel, delayed-rectifier, subfamily S, member 3	205968_at	3	7.353	0.56	7	7.7	1.45	40	u	0.542594
SMAD6	3	SMAD family member 6	207069_s_at	3	5.788	0.29	7	5.82	1.02	40	u	0.876414
			213565_s_at	3	2.762	0.25	7	2.695	0.27	40	d	0.559549
			209886_s_at	3	5.481	0.45	7	5.157	0.41	40	d	0.068494
			209887_at	3	2.197	0.03	7	2.248	0.05	40	u	0.01287
ACTR2	3	ARP2 actin-related protein 2 homolog (yeast)	200728_at	3	10.45	0.21	7	10.94	0.56	40	u	0.032953
			200727_s_at	3	7.484	0.8	7	9.73	0.93	40	u	0
			200729_s_at	3	9.184	0.44	7	10.39	0.72	40	u	0.000124
			234212_at	0	2.192	0.03	7	2.266	0.08	40	u	0.015185
			1554390_s_at	0	6.903	0.79	7	8.888	1.22	40	u	0.000188
			234210_x_at	0	3.865	0.48	7	3.983	0.59	40	u	0.629904
			1558015_s_at	0	5.274	1.06	7	7.687	1.63	40	u	0.000579
PLXNA3	3	plexin A3	203623_at	3	5.49	0.29	7	5.765	1.29	40	u	0.256228
			1553139_s_at	1	4.091	0.6	7	4.817	1.43	40	u	0.201171
			1567519_at	0	2.139	0.1	7	2.186	0.14	40	u	0.40491
TBC1D19	3	TBC1 domain family, member 19	220260_at	3	4.133	0.67	7	3.827	0.87	40	d	0.391369
HECA	3	headcase homolog (Drosophila)	218603_at	3	9.223	0.32	7	9.253	0.84	40	u	0.927029
			230529_at	0	4.365	0.57	7	4.206	0.81	40	d	0.629404
			230582_at	0	2.534	0.31	7	2.454	0.22	40	d	0.425202
SLTM	3	SAFB-like, transcription modulator	217828_at	3	9.61	0.36	7	8.907	0.7	40	d	0.014518
MTM1	3	myotubularin 1	36920_at	3	5.126	0.29	7	5.349	0.56	40	u	0.317461
			204101_at	3	3.438	0.19	7	3.589	0.32	40	u	0.237322
HOXC13	3	homeobox C13	219832_s_at	3	5.251	0.55	7	6.097	1.14	40	u	0.065773
SMARCC1	3	SWI/SNF related, matrix associated, actin dependent regulator of chromatin, subfamily c, member 1	201075_s_at	3	7.705	0.35	7	7.704	1.18	40	d	0.997836
			201072_s_at	3	5.966	0.37	7	7.467	1.16	40	u	0.000001
			201074_at	3	8.935	0.39	7	9.077	0.79	40	u	0.648055
			201073_s_at	3	7.803	0.27	7	8.438	1	40	u	0.002556
USF2	3	upstream transcription factor 2, c-fos interacting	215737_x_at	3	8.661	0.21	7	8.586	0.35	40	d	0.5967
			214879_x_at	3	8.879	0.22	7	8.617	0.46	40	d	0.152456
			202152_x_at	3	8.859	0.17	7	8.666	0.48	40	d	0.306785
TPD52L2	3	tumor protein D52-like 2	201379_s_at	3	9.094	0.15	7	9.236	0.76	40	u	0.302389
RNF41	3	ring finger protein 41	201961_s_at	3	5.544	0.31	7	5.435	0.48	40	d	0.571276
			201962_s_at	3	6.874	0.35	7	6.955	0.68	40	u	0.762926

SH2D1A	3	SH2 domain protein 1A, Duncan's disease (lymphoproliferative syndrome)	210116_at	3	4.978	0.37	7	5.709	1.15	40	u	0.004692
			211211_x_at	0	2.554	0.03	7	2.752	0.35	40	u	0.001135
			211210_x_at	0	2.576	0.04	7	2.887	0.54	40	u	0.00118
			211209_x_at	0	2.563	0.05	7	2.746	0.32	40	u	0.00158
LAPTM4A	3	lysosomal-associated protein transmembrane 4 alpha	200673_at	3	12.85	0.16	7	12.84	0.36	40	d	0.910468
GPR137B	3	G protein-coupled receptor 137B	204137_at	3	7.605	0.72	7	7.619	0.99	40	u	0.973235
LAMP1	3	lysosomal-associated membrane protein 1	201552_at	3	11.01	0.21	7	10.21	0.67	40	d	0.000002
			213728_at	3	7.324	0.44	7	7.252	0.62	40	d	0.77469
			201553_s_at	3	12.9	0.2	7	12.36	0.68	40	d	0.000429
			201551_s_at	0	8.507	1.73	7	7.73	2.11	40	d	0.371459
RXRA	3	retinoid X receptor, alpha	202426_s_at	8	5.509	0.58	7	5.779	0.84	40	u	0.428022
			202449_s_at	8	8.617	0.28	7	8.114	0.74	40	d	0.090002
NOS1	3	nitric oxide synthase 1 (neuronal)	239132_at	0	1.822	0.05	7	1.935	0.19	40	u	0.004014
			1560974_s_at	0	2.77	0.21	7	2.952	0.43	40	u	0.287917
			207309_at	0	2.637	0.47	7	2.746	0.6	40	u	0.657756
			207310_s_at	0	2.212	0.3	7	2.261	0.3	40	u	0.69895
			240911_at	0	2.642	0.14	7	2.662	0.22	40	u	0.826219
CLDN11	3	claudin 11 (oligodendrocyte transmembrane protein)	228335_at	3	9.596	0.33	7	5.814	1.52	40	d	0
			206908_s_at	0	2.24	0.26	7	2.24	0.28	40	u	0.998578
YBX2	3	Y box binding protein 2	219704_at	3	5.047	0.3	7	5.89	0.67	40	u	0.002593
ABHD4	3	abhydrolase domain containing 4	218581_at	3	7.684	0.4	7	7.101	0.77	40	d	0.062925
			242023_at	0	2.423	0.17	7	2.497	0.18	40	u	0.316697
LYPLA1	3	lysophospholipase I	212449_s_at	3	9.743	0.42	7	10.61	0.87	40	u	0.015313
			203007_x_at	2	8.781	0.46	7	9.796	1.06	40	u	0.018373
TBX5	3	T-box 5	240715_at	3	6.082	0.33	7	5.749	0.63	40	d	0.184369
			207155_at	0	2.102	0.17	7	2.226	0.3	40	u	0.301731
			211886_s_at	0	3.085	0.26	7	3.365	0.52	40	u	0.175862
			1563018_at	0	1.795	0.02	7	1.844	0.06	40	u	0.000302
C14orf45	3	chromosome 14 open reading frame 45	220173_at	3	7.857	0.84	7	6.917	1.08	40	d	0.037482
DGKH	3	diacylglycerol kinase, eta	1553300_a_at	3	2.941	0.28	7	3.324	0.82	40	u	0.038077
SLC16A7	3	solute carrier family 16, member 7 (monocarboxylic acid transporter 2)	241866_at	3	2.642	0.1	7	2.786	0.44	40	u	0.080911
			210807_s_at	1	2.825	0.26	7	2.958	0.59	40	u	0.568894
			207057_at	1	4.377	0.57	7	4.705	1.01	40	u	0.418655
TSGA14	3	testis specific, 14	225485_at	3	5.45	0.49	7	6.138	0.96	40	u	0.074787
			225484_at	3	6.119	0.61	7	6.493	0.94	40	u	0.324227
			1563997_at	0	2.42	0.15	7	2.581	0.35	40	u	0.246681
			215637_at	0	2.794	0.25	7	2.921	0.31	40	u	0.319684
NEDD4	3	neural precursor cell expressed, developmentally down-regulated 4	213012_at	3	7.502	0.35	7	6.465	0.94	40	d	0.007134
KIAA0323	3	KIAA0323	212356_at	3	7.987	0.32	7	7.276	0.62	40	d	0.005438
			212355_at	3	7.676	0.33	7	7.325	0.78	40	d	0.257004
FYCO1	3	FYVE and coiled-coil domain containing 1	218204_s_at	3	7.929	0.25	7	6.759	0.81	40	d	0
			1555523_a_at	0	3.613	0.3	7	3.72	0.5	40	u	0.594105
SLC25A5	3	solute carrier family 25 (mitochondrial carrier; adenine nucleotide translocator), member 5	200657_at	3	11.9	0.29	7	12.14	0.96	40	u	0.235322
DDAH1	3	dimethylarginine dimethylaminohydrolase 1	209094_at	3	9.714	0.4	7	9.449	0.69	40	d	0.337277
			229456_s_at	1	4.417	0.4	7	4.641	0.73	40	u	0.442249
			1553565_s_at	0	4.258	0.71	7	3.958	0.91	40	d	0.421978
			243711_at	0	3.224	0.19	7	3.319	0.47	40	u	0.60553
ACBD5	3	acyl-Coenzyme A binding domain containing 5	225663_at	0	8.862	0.31	7	9.285	0.6	40	u	0.081926
			1568877_a_at	0	5.17	0.25	7	6.623	0.84	40	u	0
FAM105B	3	family with sequence similarity 105, member B	228382_at	0	4.122	0.27	7	4.542	0.82	40	u	0.021134
			229268_at	0	5.288	0.36	7	6.372	0.76	40	u	0.000713
			229470_at	0	6.433	0.66	7	5.432	0.94	40	d	0.011449
			240834_at	0	2.956	0.48	7	3.003	0.37	40	u	0.769847
JAKMIP2	3	janus kinase and microtubule interacting protein 2	205888_s_at	0	2.426	0.1	7	2.758	0.73	40	u	0.010877
			205889_s_at	0	2.183	0.12	7	2.24	0.12	40	u	0.259628
GOLPH3	3	golgi phosphoprotein 3 (coat-protein)	217803_at	3	10.02	0.21	7	10.28	0.63	40	u	0.06461
NKRF	3	NF-kappaB repressing factor	205004_at	3	6.925	0.21	7	7.181	0.37	40	u	0.088585
NNAT	3	neuronatin	204239_s_at	3	5.701	0.5	7	5.496	0.59	40	d	0.402624
CLDN2	3	claudin 2	223509_at	3	2.871	0.23	7	2.985	0.43	40	u	0.511819
C7orf42	3	chromosome 7 open reading frame 42	218008_at	3	9.326	0.2	7	9.541	0.57	40	u	0.087743

C7orf42	3	chromosome 7 open reading frame 42	224688_at	0	9.451	0.19	7	8.303	0.83	40	d	0
ETS2	3	v-ets erythroblastosis virus E26 oncogene homolog 2 (avian)	201328_at	3	7.599	0.62	7	6.365	0.8	40	d	0.00045
			241193_at	0	2.757	0.07	7	2.9	0.27	40	u	0.010797
			201329_s_at	0	7.55	0.7	7	6.509	0.86	40	d	0.004615
CS	3	citrate synthase	208660_at	3	10.78	0.24	7	10.6	0.67	40	d	0.228585
FREQ	3	frequenin homolog (Drosophila)	222570_at	3	4.834	0.61	7	5.016	0.83	40	u	0.590961
			218266_s_at	2	3.765	0.55	7	4.287	0.94	40	u	0.171427
			238753_at	0	5.196	0.44	7	5.189	0.53	40	d	0.974597
			230146_s_at	0	3.223	0.37	7	4.263	1.02	40	u	0.01205
			230138_at	0	2.374	0.13	7	2.527	0.26	40	u	0.148566
MYNN	3	myoneurin	218926_at	3	7.163	0.38	7	7.507	0.52	40	u	0.10577
			224206_x_at	1	7.26	0.42	7	7.711	0.59	40	u	0.066426
			1566108_at	0	2.551	0.06	7	2.819	0.43	40	u	0.00075
			1566109_at	0	2.114	0.08	7	2.255	0.2	40	u	0.075562
ORMDL3	3	ORM1-like 3 (S. cerevisiae)	223259_at	3	9.033	0.2	7	8.624	1.58	40	d	0.132361
			235136_at	2	7.084	0.68	7	7.029	2.14	40	d	0.901885
			240701_at	0	3.53	0.84	7	3.574	1.19	40	u	0.927028
SMC1A	3	structural maintenance of chromosomes 1A	1555677_s_at	5	3.429	0.26	7	3.654	0.71	40	u	0.158338
			217555_at	3	6.575	0.39	7	6.387	0.39	40	d	0.255844
			239688_at	2	4.948	0.62	7	4.936	0.72	40	d	0.96757
			201589_at	1	8.335	0.4	7	9.016	0.74	40	u	0.024761
RNF103	3	ring finger protein 103	202636_at	3	10.3	0.44	7	9.522	1.05	40	d	0.0654
			1568665_at	0	3.415	0.52	7	3.504	0.75	40	u	0.771127
PABPC3	3	poly(A) binding protein, cytoplasmic 3	208113_x_at	3	12.79	0.19	7	13.24	0.59	40	u	0.001147
PPTC7	3	PTC7 protein phosphatase homolog (S. cerevisiae)	235744_at	3	3.703	0.5	7	3.88	0.78	40	u	0.573036
			225213_at	2	8.863	0.44	7	8.676	0.51	40	d	0.381274
			225204_at	2	8.846	0.44	7	8.582	0.45	40	d	0.165528
ZIC4	3	Zic family member 4	236711_at	1	2.516	0.06	7	2.602	0.19	40	u	0.034309
			211463_at	0	2.681	0.14	7	2.877	0.34	40	u	0.148375
ZFP36L2	3	zinc finger protein 36, C3H type-like 2	201368_at	1	12.22	0.24	7	10.65	0.89	40	d	0
			227681_at	1	2.608	0.38	7	2.539	0.31	40	d	0.609422
			201367_s_at	1	8.404	0.8	7	6.908	1.4	40	d	0.010161
			201369_s_at	0	10.56	0.31	7	8.67	0.88	40	d	0
C12orf64	3	chromosome 12 open reading frame 64	1553746_a_at	3	2.425	0.06	7	2.616	0.43	40	u	0.012671
ARIH2	3	ariadne homolog 2 (Drosophila)	201230_s_at	3	7.328	0.42	7	6.47	0.85	40	d	0.013862
			201228_s_at	3	5.454	0.25	7	5.891	0.57	40	u	0.05564
			216008_s_at	2	6.672	0.23	7	6.852	0.52	40	u	0.383667
			201229_s_at	1	7.415	0.23	7	7.172	0.67	40	d	0.101
			1559121_s_at	0	4.77	0.54	7	3.834	0.71	40	d	0.00227
			238068_at	0	5.347	0.57	7	5.215	0.55	40	d	0.568501
DNAJC7	3	DnaJ (Hsp40) homolog, subfamily C, member 7	202416_at	4	8.319	0.35	7	8.51	0.86	40	u	0.573352
			1556053_at	0	6.977	0.42	7	6.634	1.02	40	d	0.395273
HUWE1	3	HECT, UBA and WWE domain containing 1	214673_s_at	0	2.466	0.03	7	2.638	0.28	40	u	0.000626
			208599_at	0	5.775	0.41	7	5.905	0.46	40	u	0.499181
			208598_s_at	0	9.546	0.51	7	9.081	0.76	40	d	0.134061
			207783_x_at	0	14.46	0.09	7	14.38	0.11	40	d	0.07632
			236294_at	0	3.714	0.55	7	3.425	0.45	40	d	0.143299
TSPYL2	3	TSPY-like 2	218012_at	3	8.669	0.44	7	7.343	0.79	40	d	0.000117
AP2A1	3	adaptor-related protein complex 2, alpha 1 subunit	223237_x_at	4	7.356	0.2	7	7.722	0.66	40	u	0.009929
			229617_x_at	4	6.163	0.2	7	6.235	0.48	40	u	0.703308
			234068_s_at	0	5.946	0.34	7	6.277	1.53	40	u	0.247814
			1558792_x_at	0	2.941	0.27	7	2.405	0.31	40	d	0.000122
MORC3	3	MORC family CW-type zinc finger 3	213000_at	3	7.621	0.34	7	7.598	0.52	40	d	0.91608
FA2H	3	fatty acid 2-hydroxylase	219429_at	3	3.507	0.5	7	3.608	1.06	40	u	0.809841
			234963_s_at	0	1.96	0.17	7	2.045	0.38	40	u	0.575505
TTLL7	3	tubulin tyrosine ligase-like family, member 7	219882_at	3	3.442	0.61	7	4.189	1.34	40	u	0.164941
SH3GL3	3	SH3-domain GRB2-like 3	205636_at	3	2.194	0.07	7	2.298	0.27	40	u	0.052506
			205637_s_at	3	2.063	0.03	7	2.493	1.02	40	u	0.012546
			211565_at	0	2.719	0.36	7	3.671	0.98	40	u	0.01687
RAB23	3	RAB23, member RAS oncogene family	223463_at	3	7.022	0.5	7	8.124	1.11	40	u	0.015016
			220955_x_at	1	4.711	0.52	7	5.671	1.3	40	u	0.065599
MAPRE3	3	microtubule-associated protein, RP/EB	203841_x_at	3	3.471	0.44	7	3.4	1.19	40	d	0.879493
			229682_at	3	3.985	0.27	7	3.939	0.44	40	d	0.790329

[illegible]	[illegible]	family, member 3	203842_s_at	3	7.33	0.18	7	6.668	0.61	40	d	0.000008
			214270_s_at	0	3.726	0.75	7	3.526	1.08	40	d	0.646932
DBT	3	dihydrolipoamide branched chain transacylase E2	205371_s_at	3	4.256	0.63	7	5.151	0.96	40	u	0.024093
			205369_x_at	3	5.368	0.24	7	6.057	0.96	40	u	0.000583
			205370_x_at	3	10.5	0.29	7	10.29	0.7	40	d	0.450528
			244687_at	0	4.275	0.37	7	4.219	0.93	40	d	0.878424
			231919_at	0	4.377	0.37	7	4.516	0.83	40	u	0.671308
			211196_at	0	2.198	0.03	7	2.256	0.05	40	u	0.006787
SAMD8	3	sterile alpha motif domain containing 8	242062_at	3	3.013	0.36	7	3.404	0.63	40	u	0.121995
CSNK1G2	3	casein kinase 1, gamma 2	202573_at	3	8.747	0.36	7	9.022	0.62	40	u	0.273174
			202574_s_at	0	7.317	0.08	7	7.785	0.64	40	u	0.000076
GRIN2B	3	glutamate receptor, ionotropic, N-methyl D-aspartate 2B	210412_at	3	2.014	0.01	7	2.095	0.08	40	u	0.000001
			210411_s_at	0	2.513	0.35	7	2.626	0.33	40	u	0.418746
MPP2	3	membrane protein, palmitoylated 2 (MAGUK p55 subfamily member 2)	213270_at	3	3.815	0.58	7	3.644	0.85	40	d	0.618526
			207984_s_at	3	4.232	0.49	7	3.923	0.79	40	d	0.332035
MATN3	3	matrilin 3	206091_at	3	3.45	0.27	7	4.515	1.07	40	u	0.000007
TNK2	3	tyrosine kinase, non-receptor, 2	203839_s_at	3	8.183	0.34	7	7.471	0.87	40	d	0.042825
			1555557_a_at	3	2.289	0.04	7	2.399	0.28	40	u	0.026287
			203838_s_at	3	2.757	0.24	7	3.044	0.63	40	u	0.252349
			216439_at	0	4.325	0.28	7	4.04	0.52	40	d	0.172457
TMEM169	3	transmembrane protein 169	228981_at	2	2.192	0.08	7	2.263	0.2	40	u	0.359716
ABI3BP	3	ABI gene family, member 3 (NESH) binding protein	223395_at	3	8.318	0.45	7	5.304	1.84	40	d	0
			1559077_at	0	2.924	0.04	7	3.065	0.12	40	u	0.000009
			220518_at	0	2.303	0.11	7	2.485	0.51	40	u	0.060329
CDK5R2	3	cyclin-dependent kinase 5, regulatory subunit 2 (p39)	205852_at	3	4.519	0.42	7	4.485	0.46	40	d	0.858361
SLC30A4	3	solute carrier family 30 (zinc transporter), member 4	238845_at	3	2.708	0.1	7	2.804	0.22	40	u	0.268835
			207362_at	1	2.103	0.02	7	2.196	0.14	40	u	0.000371
WDR42A	3	WD repeat domain 42A	216885_s_at	3	7.614	0.18	7	7.052	0.64	40	d	0.000079
			202249_s_at	3	8.326	0.37	7	8.561	0.55	40	u	0.289628
			202250_s_at	3	8.47	0.27	7	7.878	0.83	40	d	0.002086
			243318_at	0	8.245	0.52	7	8.006	0.69	40	d	0.398163
			238340_at	0	4.259	0.45	7	4.784	0.53	40	u	0.019551
			232339_at	0	2.336	0.04	7	2.453	0.21	40	u	0.00321
			1554049_s_at	0	2.943	0.42	7	3.333	0.77	40	u	0.206591
			233637_at	0	4.155	0.32	7	4.495	0.54	40	u	0.116939
ITM2C	3	integral membrane protein 2C	221004_s_at	3	8.811	0.27	7	8.251	1.1	40	d	0.010812
SCAMP5	3	secretory carrier membrane protein 5	212699_at	3	4.892	0.3	7	5.011	1.17	40	u	0.597143
			234568_at	0	3.208	0.23	7	3.154	0.15	40	d	0.419845
MAP7D2	3	MAP7 domain containing 2	228262_at	0	2.354	0.25	7	2.688	1	40	u	0.085862
PCGF2	3	polycomb group ring finger 2	213551_x_at	3	7.42	0.36	7	6.8	1.08	40	d	0.011333
			203793_x_at	3	7.064	0.27	7	6.702	0.87	40	d	0.050113
			214239_x_at	3	8.154	0.25	7	7.502	1.25	40	d	0.005866
			203792_x_at	0	7.583	0.29	7	7.625	0.91	40	u	0.824688
			240752_at	0	3.708	0.55	7	3.442	0.41	40	d	0.150539
TMEM183A	3	transmembrane protein 183A	212164_at	3	6.522	0.56	7	6.454	0.96	40	d	0.858745
			212165_at	3	9.211	0.26	7	9.985	0.58	40	u	0.00146
PYGO2	3	pygopus homolog 2 (Drosophila)	225370_at	3	7.764	0.3	7	8.161	0.52	40	u	0.062126
			239666_at	0	4.892	0.23	7	4.948	0.31	40	u	0.654481
VDAC3	3	voltage-dependent anion channel 3	208845_at	3	11.04	0.13	7	11.69	0.82	40	u	0.000034
			208846_s_at	3	9.348	0.26	7	9.957	0.9	40	u	0.00177
			208844_at	3	3.609	0.43	7	4.406	0.67	40	u	0.004909
PRKAG2	3	protein kinase, AMP-activated, gamma 2 non-catalytic subunit	222582_at	3	7.635	0.25	7	7.439	0.58	40	d	0.391573
			233748_x_at	1	6.36	0.34	7	6.623	0.93	40	u	0.473298
			218292_s_at	1	6.27	0.43	7	6.464	0.94	40	u	0.600728
			215231_at	0	4.38	0.59	7	4.05	0.41	40	d	0.08374
			231224_x_at	0	4.149	0.46	7	4.268	0.54	40	u	0.593912
H2AFZ	3	H2A histone family, member Z	200853_at	3	10.69	0.19	7	12.06	0.73	40	u	0
			213911_s_at	3	11.13	0.17	7	12.62	0.61	40	u	0
PLOD3	3	procollagen-lysine, 2-oxoglutarate 5-dioxygenase 3	202185_at	3	7.816	0.33	7	8.27	0.91	40	u	0.206575
AYTL2	3	acyltransferase like 2	201818_at	3	7.906	0.42	7	8.815	1	40	u	0.024912
POLQ	3	polymerase (DNA directed), theta	219510_at	3	3.445	0.48	7	5.092	0.95	40	u	0.000071
			207746_at	0	2.85	0.35	7	4.171	0.98	40	u	0.000002

UBE1L2	3	ubiquitin-activating enzyme E1-like 2	222602_at	3	8.413	0.48	7	8.978	0.61	40	u	0.026898
			218340_s_at	3	6.45	1.15	7	7.554	1.63	40	u	0.099478
			222600_s_at	3	6.969	0.41	7	8.423	0.75	40	u	0.000013
			222601_at	3	7.706	0.32	7	8.31	0.74	40	u	0.043327
			1555441_at	0	2.938	0.17	7	3.116	0.31	40	u	0.158853
			220869_at	0	2.993	0.32	7	3.339	0.64	40	u	0.178764
WDFY1	3	WD repeat and FYVE domain containing 1	224800_at	3	8.415	0.28	7	8.475	0.68	40	u	0.819699
			233559_s_at	1	6.294	0.28	7	7.178	0.65	40	u	0.001152
ZBTB6	3	zinc finger and BTB domain containing 6	206098_at	0	3.126	0.37	7	3.751	0.83	40	u	0.06124
TXNIP	3	thioredoxin interacting protein	201010_s_at	3	13.42	0.28	7	11.34	0.93	40	d	0
			201009_s_at	3	12.42	0.46	7	10.39	0.97	40	d	0.000003
			201008_s_at	3	11.48	0.5	7	9.56	1.28	40	d	0.000383
OSGIN2	3	oxidative stress induced growth inhibitor family member 2	204024_at	3	4.801	0.56	7	5.532	0.96	40	u	0.062441
			1554414_a_at	0	6.632	0.63	7	7.822	1.39	40	u	0.035055
			214161_at	0	2.61	0.12	7	2.956	0.5	40	u	0.000757
			41553_at	0	2.55	0.08	7	2.77	0.35	40	u	0.001813
HMGB2	3	high-mobility group box 2	208808_s_at	3	9.624	0.42	7	11.2	1.06	40	u	0.000469
			243368_at	0	2.255	0.02	7	2.365	0.19	40	u	0.001051
			236091_at	0	2.759	0.25	7	2.785	0.32	40	u	0.842783
FBXL2	3	F-box and leucine-rich repeat protein 2	214436_at	3	7.068	0.33	7	7.141	0.99	40	u	0.726716
C5orf23	3	chromosome 5 open reading frame 23	219054_at	3	5.838	1.18	7	6.559	1.57	40	u	0.263685
POLR2D	3	polymerase (RNA) II (DNA directed) polypeptide D	203664_s_at	3	5.689	0.23	7	6.661	0.7	40	u	0
			214144_at	2	5.182	0.42	7	5.866	0.51	40	u	0.001954
HNRNPC	3	heterogeneous nuclear ribonucleoprotein C (C1/C2)	200014_s_at	3	10.55	0.17	7	10.71	0.41	40	u	0.314098
			200751_s_at	3	10.84	0.22	7	11.04	1.08	40	u	0.325494
			212626_x_at	3	10.86	0.27	7	11.02	0.41	40	u	0.336011
			214737_x_at	2	10.84	0.28	7	10.96	0.38	40	u	0.442042
			216302_at	0	4.856	0.75	7	5.01	0.73	40	u	0.621145
			235500_at	0	4.701	0.57	7	3.434	0.88	40	d	0.000825
			227110_at	0	8.392	0.41	7	8.019	0.62	40	d	0.137399
SF1	3	splicing factor 1	208313_s_at	3	10.98	0.34	7	9.917	0.62	40	d	0.000081
			210172_at	0	6.816	0.74	7	6.605	1.17	40	d	0.655502
			1555749_at	0	3.321	0.32	7	3.318	0.64	40	d	0.988364
RGS2	3	regulator of G-protein signaling 2, 24kDa	202388_at	3	11.97	0.81	7	10.14	1.36	40	d	0.001569
KIAA1737	3	KIAA1737	225623_at	3	8.752	0.45	7	8.059	0.73	40	d	0.021289
PPT2	3	palmitoyl-protein thioesterase 2	209490_s_at	3	7.152	0.16	7	6.884	0.57	40	d	0.024414
KIAA1324L	3	KIAA1324-like	235301_at	3	6.194	0.73	7	5.671	1.4	40	d	0.347751
			244317_at	0	5.658	0.6	7	5.456	1.15	40	d	0.659407
PPP1R2	3	protein phosphatase 1, regulatory (inhibitor) subunit 2	202165_at	3	8.27	0.31	7	8.328	0.77	40	u	0.846941
			202166_s_at	3	8.312	0.56	7	9.11	1.15	40	u	0.085561
PRDM8	3	PR domain containing 8	219835_at	0	2.047	0.04	7	2.076	0.08	40	u	0.351269
P4HA2	3	procollagen-proline, 2-oxoglutarate 4-dioxygenase (proline 4-hydroxylase), alpha polypeptide II	202733_at	3	7.328	0.65	7	8.097	0.8	40	u	0.023328
TMEM9	3	transmembrane protein 9	222987_s_at	3	8.822	0.29	7	9.122	0.83	40	u	0.105186
			222988_s_at	3	9.333	0.24	7	9.484	0.76	40	u	0.339429
ZNF580	3	zinc finger protein 580	220748_s_at	3	8.378	0.25	7	7.658	0.65	40	d	0.00678
EZH1	3	enhancer of zeste homolog 1 (Drosophila)	203249_at	3	8.399	0.35	7	6.722	0.72	40	d	0
			32259_at	3	7.544	0.41	7	6.197	0.7	40	d	0.000016
			239197_s_at	0	5.656	0.23	7	5.774	0.33	40	u	0.3737
			239198_at	0	2.444	0.26	7	2.459	0.3	40	u	0.906265
			211310_at	0	2.523	0.37	7	2.496	0.25	40	d	0.813684
LRRTM4	3	leucine rich repeat transmembrane neuronal 4	220345_at	3	2.948	0.08	7	3.117	0.23	40	u	0.001711
ZNF512	3	zinc finger protein 512	225050_at	3	9.056	0.25	7	8.283	1.02	40	d	0.000286
			1553218_a_at	2	8.391	0.14	7	7.589	0.84	40	d	0.000002
LRRC55	3	leucine rich repeat containing 55	232856_at	0	2.611	0.14	7	2.944	0.89	40	u	0.034898
ONECUT1	3	one cut homeobox 1	210745_at	3	2.303	0.1	7	2.341	0.11	40	u	0.392783
STX17	3	syntaxin 17	222708_s_at	3	8.937	0.15	7	8.422	0.45	40	d	0.000014
			218666_s_at	3	5.164	0.46	7	5.486	0.58	40	u	0.182905
CCNL1	3	cyclin L1	220046_s_at	3	10.23	0.3	7	9.028	0.66	40	d	0.000034
			1555411_a_at	0	10.44	0.32	7	9.145	0.75	40	d	0.000066
			241495_at	0	3.351	0.62	7	3.818	0.92	40	u	0.21281
			1555827_at	0	6.057	0.78	7	6.033	0.96	40	d	0.952776

NAP1L3	3	nucleosome assembly protein 1-like 3	204749_at	3	5.284	0.62	7	4.065	0.79	40	d	0.000462
PCDH11Y	3	protocadherin 11 Y-linked	217049_x_at	0	3.976	0.3	7	3.881	0.28	40	d	0.418253
			211227_s_at	0	2.911	0.05	7	3.008	0.13	40	u	0.061591
KCNH8	3	potassium voltage-gated channel, subfamily H (eag-related), member 8	1552742_at	3	3.596	0.79	7	2.937	0.74	40	d	0.041582
CNIH2	3	cornichon homolog 2 (Drosophila)	230070_at	3	3.097	0.48	7	3.37	0.7	40	u	0.340882
DSCAM	3	Down syndrome cell adhesion molecule	237268_at	3	2.782	0.23	7	2.819	0.31	40	u	0.765793
			211484_s_at	2	2.612	0.07	7	2.724	0.22	40	u	0.015833
INA	3	internexin neuronal intermediate filament protein, alpha	204465_s_at	3	2.618	0.13	7	2.711	0.28	40	u	0.402265
PIGA	3	phosphatidylinositol glycan anchor biosynthesis, class A (paroxysmal nocturnal hemoglobinuria)	205281_s_at	3	6.577	0.46	7	6.607	0.77	40	u	0.921279
RREB1	3	ras responsive element binding protein 1	228487_s_at	0	7.946	0.31	7	7.27	1.24	40	d	0.006627
			215620_at	0	4.775	0.41	7	4.886	0.55	40	u	0.620769
			216648_s_at	0	2.299	0.02	7	2.32	0.04	40	u	0.184111
			216649_at	0	2.213	0.01	7	2.25	0.1	40	u	0.03007
			217411_s_at	0	2.574	0.21	7	2.596	0.22	40	u	0.812697
			215032_at	0	7.408	0.24	7	6.632	0.67	40	d	0.000017
			242297_at	0	6.568	0.39	7	5.93	0.82	40	d	0.054348
ENPP5	3	ectonucleotide pyrophosphatase/phosphodiesterase 5	227803_at	3	7.867	0.78	7	7.084	1.53	40	d	0.201958
			237054_at	0	4.5	0.7	7	4.439	1.27	40	d	0.904627
BOLL	3	bol, boule-like (Drosophila)	236954_at	2	2.012	0.02	7	2.073	0.07	40	u	0.000029
SYT3	3	synaptotagmin III	223901_at	2	2.445	0.28	7	2.617	0.41	40	u	0.297627
PPME1	3	protein phosphatase methylesterase 1	49077_at	3	8.067	0.22	7	8.359	0.6	40	u	0.036287
			217841_s_at	3	8.419	0.18	7	8.613	0.69	40	u	0.151864
NUP160	3	nucleoporin 160kDa	212709_at	3	6.72	0.28	7	7.432	0.84	40	u	0.000422
			214963_at	0	4.114	0.16	7	4.37	0.56	40	u	0.026043
			214962_s_at	0	5.142	0.56	7	5.747	0.93	40	u	0.11089
DAZL	3	deleted in azoospermia-like	206588_at	3	2.888	0.16	7	3.091	0.38	40	u	0.176723
TDG	3	thymine-DNA glycosylase	203742_s_at	3	6.516	0.42	7	7.219	0.69	40	u	0.014477
			203743_s_at	3	8.725	0.24	7	9.342	0.69	40	u	0.000296
IQGAP1	3	IQ motif containing GTPase activating protein 1	200791_s_at	3	11.38	0.27	7	10.56	0.91	40	d	0.000089
			210840_s_at	3	9.846	0.24	7	10.12	0.91	40	u	0.124784
			213446_s_at	0	6.859	0.45	7	8.437	1.38	40	u	0.000008
NPEPL1	3	aminopeptidase-like 1	218822_s_at	3	6.487	0.13	7	6.568	0.73	40	u	0.528727
			218821_at	3	7.289	0.17	7	7.138	1.05	40	d	0.41461
			235033_at	0	4.542	0.45	7	5.517	0.91	40	u	0.009546
			89476_r_at	0	8.508	0.19	7	8.502	0.36	40	d	0.966714
XPO1	3	exportin 1 (CRM1 homolog, yeast)	208775_at	3	10.81	0.37	7	11.13	0.47	40	u	0.102271
LATS1	3	LATS, large tumor suppressor, homolog 1 (Drosophila)	219813_at	3	3.724	0.19	7	4.191	0.52	40	u	0.026535
			1570231_at	0	2.908	0.1	7	3.086	0.17	40	u	0.010028
			1570425_s_at	0	2.577	0.08	7	2.807	0.38	40	u	0.001602
CAMK2A	3	calcium/calmodulin-dependent protein kinase (CaM kinase) II alpha	213108_at	3	2.674	0.03	7	2.764	0.14	40	u	0.0012
			207613_s_at	0	2.271	0.2	7	2.326	0.28	40	u	0.626824
SPOCK2	3	sparc/osteonectin, cwcv and kazal-like domains proteoglycan (testican) 2	202523_s_at	3	2.577	0.27	7	3.081	1.31	40	u	0.039611
			202524_s_at	3	6.055	0.68	7	6.584	1.52	40	u	0.379147
TFAP2C	3	transcription factor AP-2 gamma (activating enhancer binding protein 2 gamma)	205286_at	3	10.81	0.53	7	9.506	1.01	40	d	0.001959
			205287_s_at	0	6.343	0.63	7	4.584	1.4	40	d	0.002488
TRAF4	3	TNF receptor-associated factor 4	206837_at	4	1.975	0.06	7	2.698	1.17	40	u	0.000451
			202871_at	3	7.999	0.5	7	8.365	0.82	40	u	0.266454
			211899_s_at	0	5.546	0.64	7	6.394	1.4	40	u	0.131273
			235688_s_at	0	5.661	0.55	7	5.808	1.07	40	u	0.729928
			242473_at	0	3.889	0.45	7	4.158	0.65	40	u	0.310298
LGI1	3	leucine-rich, glioma inactivated 1	206349_at	3	2.193	0.03	7	2.363	0.71	40	u	0.142138
SLC20A1	3	solute carrier family 20 (phosphate transporter), member 1	201920_at	3	9.022	0.42	7	9.4	0.68	40	u	0.171872
EHD3	3	EH-domain containing 3	218935_at	3	6.326	0.39	7	5.099	1.03	40	d	0.00392
			241267_at	0	3.558	0.27	7	3.573	0.29	40	u	0.903416
PURG	3	purine-rich element binding protein G	220860_at	3	2.569	0.1	7	2.713	0.19	40	u	0.066706
			235634_at	0	3.595	0.35	7	3.532	0.64	40	d	0.804786
DGAT2	3	diacylglycerol O-acyltransferase homolog 2 (mouse)	224327_s_at	3	7.017	1.18	7	6.413	1.42	40	d	0.30343
			226064_s_at	3	5.934	1.33	7	5.716	1.79	40	d	0.765379
			225548_at	0	9.27	0.43	7	8.09	0.82	40	d	0.000697

SHROOM3	3	shroom family member 3	228400_at	0	8.819	0.41	7	7.187	1.04	40	d	0.000227
			243653_at	0	2.099	0.05	7	2.111	0.06	40	u	0.640547
LRRC7	3	leucine rich repeat containing 7	1552666_a_at	3	2.385	0.04	7	2.513	0.14	40	u	0.000081
			233499_at	3	2.538	0.23	7	2.63	0.26	40	u	0.388395
			242050_at	0	2.473	0.23	7	2.38	0.05	40	d	0.369064
WNT2B	3	wingless-type MMTV integration site family, member 2B	206458_s_at	3	2.828	0.06	7	2.867	0.13	40	u	0.468661
			206459_s_at	0	2.106	0.09	7	2.163	0.12	40	u	0.264436
PC	3	pyruvate carboxylase	204476_s_at	3	6.345	0.35	7	5.805	0.96	40	d	0.016461
MON2	3	MON2 homolog (S. cerevisiae)	212755_at	3	5.498	0.45	7	5.184	0.69	40	d	0.263295
			212754_s_at	3	8.65	0.27	7	8.254	0.67	40	d	0.13782
FRK	3	fyn-related kinase	207178_s_at	3	2.8	0.26	7	3.398	0.81	40	u	0.001455
RASD1	3	RAS, dexamethasone-induced 1	223467_at	3	9.754	1.3	7	8.142	1.45	40	d	0.009745
SLC41A2	3	solute carrier family 41, member 2	243894_at	3	4.45	0.87	7	4.766	0.71	40	u	0.308708
			223798_at	2	4.067	1.14	7	3.994	1.1	40	d	0.875793
CDC73	3	cell division cycle 73, Paf1/RNA polymerase II complex component, homolog (S. cerevisiae)	218578_at	2	6.579	0.34	7	8.047	0.62	40	u	0
LOXL3	3	lysyl oxidase-like 3	228253_at	0	4.194	0.68	7	4.501	0.73	40	u	0.31441
C3orf57	3	chromosome 3 open reading frame 57	238702_at	3	4.501	0.44	7	4.687	0.54	40	u	0.406349
NRIP3	3	nuclear receptor interacting protein 3	219557_s_at	3	6.224	1.39	7	5.812	1.42	40	d	0.491059
LRRN3	3	leucine rich repeat neuronal 3	209840_s_at	3	4.824	1.82	7	2.915	1.43	40	d	0.003846
			209841_s_at	3	5.193	1.53	7	4.322	1.15	40	d	0.093331
PTPRO	3	protein tyrosine phosphatase, receptor type, O	208121_s_at	3	3.245	0.51	7	3.943	1	40	u	0.084163
			1554199_at	0	2.726	0.08	7	2.982	0.42	40	u	0.001539
ARID3B	3	AT rich interactive domain 3B (BRIGHT-like)	218964_at	3	4.697	0.37	7	4.968	0.52	40	u	0.203986
CDKN1C	3	cyclin-dependent kinase inhibitor 1C (p57, Kip2)	219533_at	3	2.051	0.07	7	2.106	0.09	40	u	0.123791
			216894_x_at	3	7.83	0.78	7	5.969	0.92	40	d	0.000011
			219534_x_at	3	7.823	0.64	7	5.861	1.11	40	d	0.000054
			213182_x_at	3	7.376	0.78	7	5.292	1.11	40	d	0.000028
			213183_s_at	3	5.352	0.79	7	4.49	0.84	40	d	0.017119
			213348_at	2	8.948	0.68	7	6.774	1.15	40	d	0.000021
NAT11	3	N-acetyltransferase 11	218734_at	3	6.596	0.29	7	6.578	0.86	40	d	0.92086
SYNPR	3	synaptoporin	230303_at	3	2.464	0.08	7	2.625	0.27	40	u	0.005332
RGAG1	3	retrotransposon gag domain containing 1	232785_at	3	2.832	0.35	7	2.874	0.33	40	u	0.768998
UBQLN1	3	ubiquilin 1	222989_s_at	3	6.944	0.37	7	7.999	0.75	40	u	0.000916
			222990_at	3	10.04	0.18	7	10	0.64	40	d	0.739202
			222991_s_at	1	8.039	0.41	7	8.814	0.62	40	u	0.003279
PPP2R5D	3	protein phosphatase 2, regulatory subunit B', delta isoform	202513_s_at	3	7.376	0.25	7	7.586	0.69	40	u	0.173337
			211159_s_at	0	6.966	0.29	7	6.91	1.02	40	d	0.780937
RBMX	3	RNA binding motif protein, X-linked	213762_x_at	3	11.01	0.23	7	10.75	0.56	40	d	0.23097
			225310_at	2	10.69	0.37	7	10.18	0.66	40	d	0.059343
			1556336_at	0	6.145	0.74	7	7.435	0.64	40	u	0.000026
ITGA11	3	integrin, alpha 11	222899_at	3	6.85	0.52	7	6.843	0.84	40	d	0.983825
			1554819_a_at	1	4.637	0.46	7	4.737	0.61	40	u	0.687055
GAS7	3	growth arrest-specific 7	202191_s_at	3	8.477	0.73	7	6.691	1.1	40	d	0.000189
			202192_s_at	3	8.116	0.73	7	6.161	0.81	40	d	0.000001
			210872_x_at	1	3.551	1.23	7	2.713	0.62	40	d	0.152302
			211067_s_at	1	5.045	0.77	7	3.661	1.27	40	d	0.008673
			207704_s_at	0	4.478	1.13	7	3.734	0.77	40	d	0.038867
FCP1	3	F-cell production 1	no probeset available									
NOG	3	noggin	231798_at	3	2.384	0.22	7	2.335	0.11	40	d	0.608654
KLF15	3	Kruppel-like factor 15	231015_at	3	5.874	0.36	7	5.58	0.58	40	d	0.210555
			221302_at	0	2.056	0.03	7	2.155	0.16	40	u	0.001133
ARHGEF17	3	Rho guanine nucleotide exchange factor (GEF) 17	203756_at	3	8.127	0.43	7	7.032	0.78	40	d	0.000953
CHD5	3	chromodomain helicase DNA binding protein 5	213965_s_at	2	2.469	0.19	7	2.517	0.29	40	u	0.683806
			217250_s_at	1	1.94	0.02	7	2.026	0.13	40	u	0.000333
RPGR	3	retinitis pigmentosa GTPase regulator	207624_s_at	0	5.572	0.49	7	5.559	0.55	40	d	0.954082
COL6A3	3	collagen, type VI, alpha 3	201438_at	3	11.46	0.54	7	10.79	1.54	40	d	0.056466
EGLN3	3	egl nine homolog 3 (C. elegans)	219232_s_at	3	4.817	0.9	7	5.302	1.37	40	u	0.381428
			222847_s_at	3	5.777	0.61	7	6.361	1.43	40	u	0.303612
ST13	3	suppression of tumorigenicity 13 (colon carcinoma) (Hsp70 interacting protein)	208666_s_at	1	9.245	0.29	7	8.914	0.96	40	d	0.099358
			208667_s_at	1	10.7	0.21	7	10.13	0.68	40	d	0.000251

		carcinoma) (Hsp70 interacting protein)	207040_s_at	0	12.05	0.26	7	11.33	0.59	40	d	0.002956
SHC4	3	SHC (Src homology 2 domain containing) family, member 4	235238_at	3	3.936	0.8	7	5.02	2.48	40	u	0.044322
			230538_at	3	4.979	0.44	7	5.267	2.56	40	u	0.523348
GPRC5B	3	G protein-coupled receptor, family C, group 5, member B	225511_at	7	5.184	0.63	7	3.669	1.14	40	d	0.001636
			203632_s_at	3	8.652	0.48	7	7.156	1.79	40	d	0.000132
			203631_s_at	1	3.987	0.66	7	3.407	0.89	40	d	0.115055
			228313_at	0	2.391	0.1	7	2.399	0.14	40	u	0.90149
HDGF	3	hepatoma-derived growth factor (high-mobility group protein 1-like)	216484_x_at	3	8.731	0.17	7	9.515	0.63	40	u	0
			200896_x_at	3	10.75	0.22	7	11.58	0.71	40	u	0.000003
LHFPL3	3	lipoma HMGIC fusion partner-like 3	236761_at	0	2.233	0.1	7	2.352	0.3	40	u	0.077993
SPARC	3	secreted protein, acidic, cysteine-rich (osteonectin)	212667_at	3	10.35	0.39	7	9.444	1.15	40	d	0.001003
			200665_s_at	2	12.39	0.42	7	12.85	0.87	40	u	0.179976
ASB7	3	ankyrin repeat and SOCS box-containing 7	219996_at	0	4.31	0.34	7	4.997	0.76	40	u	0.026339
			233807_at	0	2.535	0.24	7	2.639	0.29	40	u	0.378097
ZNF579	3	zinc finger protein 579	229140_at	3	7.186	0.23	7	7.058	0.55	40	d	0.556491
			239703_at	0	2.295	0.19	7	2.396	0.41	40	u	0.537579
F3	3	coagulation factor III (thromboplastin, tissue factor)	204363_at	3	8.811	0.39	7	6.823	0.95	40	d	0.000003
KIF21B	3	kinesin family member 21B	204411_at	3	3.911	0.33	7	3.898	0.47	40	d	0.943997
SAPS1	3	SAPS domain family, member 1	209229_s_at	3	7.358	0.21	7	7.607	0.54	40	u	0.248761
WNT5B	3	wingless-type MMTV integration site family, member 5B	223537_s_at	3	4.083	0.46	7	4.095	0.61	40	u	0.961447
			230299_s_at	3	2	0.02	7	2.063	0.12	40	u	0.002683
			221029_s_at	3	4.787	0.8	7	3.769	1.26	40	d	0.049746
KHDRBS1	3	KH domain containing, RNA binding, signal transduction associated 1	200040_at	3	10.61	0.24	7	10.34	0.43	40	d	0.124027
			214185_at	1	5.281	0.44	7	5.623	0.51	40	u	0.111086
			201488_x_at	1	10.22	0.23	7	10.34	0.47	40	u	0.51033
IFT20	3	intraflagellar transport 20 homolog (Chlamydomonas)	210312_s_at	3	9.467	0.22	7	9.297	0.71	40	d	0.245417
GPC6	3	glypican 6	223730_at	2	4.226	0.67	7	5.146	1.25	40	u	0.070617
			227059_at	0	5.758	0.81	7	6.68	1.36	40	u	0.094746
HIP1	3	huntingtin interacting protein 1	226364_at	3	6.815	0.5	7	6.63	1.01	40	d	0.642913
			205425_at	3	6.397	0.46	7	6.554	1.13	40	u	0.726859
			205426_s_at	1	5.011	0.47	7	5.164	1.11	40	u	0.72755
			1560317_s_at	0	2.72	0.09	7	2.796	0.21	40	u	0.347311
LHX2	3	LIM homeobox 2	206140_at	3	2.361	0.09	7	2.586	0.65	40	u	0.046708
			211219_s_at	0	2.032	0.23	7	2.026	0.32	40	d	0.962928
STK38	3	serine/threonine kinase 38	202951_at	3	8.947	0.14	7	9.132	0.55	40	u	0.088413
			1553117_a_at	1	7.322	0.25	7	7.516	1.18	40	u	0.372428
			1557145_at	0	5.223	0.26	7	5.559	0.6	40	u	0.158586
			216727_at	0	2.637	0.18	7	2.665	0.31	40	u	0.823395
			216451_at	0	3.504	0.48	7	3.529	0.51	40	u	0.907716
RUNDC3A	3	RUN domain containing 3A	206196_s_at	3	2.36	0.16	7	2.926	1.44	40	u	0.022903
			213439_x_at	3	5.317	0.24	7	5.363	0.93	40	u	0.799815
FJX1	3	four jointed box 1 (Drosophila)	219522_at	3	6.602	0.29	7	6.633	0.97	40	u	0.875967
CLCF1	3	cardiotrophin-like cytokine factor 1	219500_at	3	5.988	0.44	7	5.847	0.65	40	d	0.591426
RP		3 th dementia, seizures, ataxia, proximal muscle	no probeset available									
RAB43	3	RAB43, member RAS oncogene family	225632_s_at	3	6.596	0.18	7	5.955	0.61	40	d	0.00001
			229894_s_at	3	5.215	0.29	7	5.405	0.62	40	u	0.439653
NOTCH1	3	Notch homolog 1, translocation-associated (Drosophila)	218902_at	3	8.128	0.55	7	7.19	0.88	40	d	0.01036
			223508_at	0	3.952	0.23	7	3.994	0.26	40	u	0.695808
SNAP91	3	synaptosomal-associated protein, 91kDa homolog (mouse)	204953_at	0	2.632	0.56	7	2.627	0.96	40	d	0.988579
STXBP3	3	syntaxin binding protein 3	203310_at	3	8.953	0.47	7	9.067	0.65	40	u	0.66535
			1560486_at	0	3.252	0.39	7	3.841	0.8	40	u	0.068164
DAAM1	3	dishevelled associated activator of morphogenesis 1	216060_s_at	3	8.628	0.34	7	8.423	0.96	40	d	0.333498
			232552_at	0	7.78	0.63	7	6.554	1.11	40	d	0.00789
			244062_at	0	5.734	0.8	7	5.343	1.1	40	d	0.385242
GOLT1B	3	golgi transport 1 homolog B (S. cerevisiae)	222552_at	3	8.665	0.24	7	9.378	0.84	40	u	0.00016
			218193_s_at	0	6.724	0.75	7	8.488	0.94	40	u	0.000034
RASSF1	3	Ras association (RalGDS/AF-6) domain family 1	204346_s_at	3	7.911	0.4	7	7.294	0.61	40	d	0.014693
			240278_at	0	2.218	0.29	7	2.278	0.48	40	u	0.754656
PITPNB	3	phosphatidylinositol transfer protein, beta	202522_at	0	9.491	0.2	7	9.42	0.46	40	d	0.69509
USP1	3	ubiquitin specific peptidase 1	202412_s_at	3	7.459	0.41	7	9.299	1.13	40	u	0.000131
			202413_s_at	3	9.633	0.27	7	10.31	0.99	40	u	0.001193

RBL1	3	retinoblastoma-like 1 (p107)	205296_at	3	3.591	0.2	7	4.855	0.91	40	u	0
			1555003_at	0	4.275	0.14	7	4.459	0.53	40	u	0.077428
			1559307_s_at	0	2.658	0.05	7	2.946	0.47	40	u	0.000587
			1555004_a_at	0	3.149	0.21	7	3.964	0.76	40	u	0.000005
ELMOD1	3	ELMO/CED-12 domain containing 1	231930_at	3	2.235	0.07	7	2.345	0.21	40	u	0.01874
TMEM178	3	transmembrane protein 178	229302_at	3	6.601	0.63	7	3.222	1.04	40	d	0
HLX	3	H2.0-like homeobox	214438_at	3	4.38	0.79	7	4.179	0.52	40	d	0.40643
APBA1	3	amyloid beta (A4) precursor protein-binding, family A, member 1 (X11)	206679_at	3	4.231	0.23	7	4.356	0.47	40	u	0.498836
RAB2A	3	RAB2A, member RAS oncogene family	208732_at	3	9.029	0.41	7	8.857	0.9	40	d	0.629536
			208730_x_at	3	9.648	0.51	7	8.871	0.49	40	d	0.000503
			208731_at	3	10.64	0.26	7	11.03	0.86	40	u	0.032028
			208733_at	3	4.394	0.52	7	4.45	0.99	40	u	0.885934
			208734_x_at	0	9.866	0.22	7	10.78	0.79	40	u	0.000002
			221960_s_at	0	4.313	0.72	7	6.176	1.47	40	u	0.002451
SLC7A6	3	solute carrier family 7 (cationic amino acid transporter, y+ system), member 6	203579_s_at	3	5.192	0.2	7	6.045	0.73	40	u	0.000001
			203578_s_at	3	3.589	0.52	7	4.268	0.64	40	u	0.013069
			203580_s_at	3	6.515	0.19	7	7.107	0.61	40	u	0.000052
			229153_at	2	6.157	0.31	7	5.599	0.62	40	d	0.026532
ADAM12	3	ADAM metallopeptidase domain 12 (meltrin alpha)	202952_s_at	3	5.814	0.92	7	6.349	1.14	40	u	0.255407
			215613_at	0	4.188	0.72	7	3.522	0.51	40	d	0.00579
			204943_at	0	2.843	0.42	7	2.858	0.52	40	u	0.943346
SIDT1	3	SID1 transmembrane family, member 1	219734_at	3	7.019	0.78	7	5.855	1.89	40	d	0.123084
SLC33A1	3	solute carrier family 33 (acetyl-CoA transporter), member 1	203165_s_at	3	5.645	0.41	7	6.32	1.02	40	u	0.099376
			1554148_a_at	0	6.412	0.42	7	6.99	0.9	40	u	0.110576
			203164_at	0	7.32	0.37	7	7.987	0.7	40	u	0.020961
C13orf23	3	chromosome 13 open reading frame 23	225887_at	3	7.045	0.34	7	6.965	0.74	40	d	0.782783
			218420_s_at	0	6.445	0.43	7	6.566	0.69	40	u	0.663182
SLC16A14	3	solute carrier family 16, member 14 (monocarboxylic acid transporter 14)	238029_s_at	3	6.623	0.76	7	6.128	1.83	40	d	0.49406
RAB40B	3	RAB40B, member RAS oncogene family	204547_at	3	9.137	0.35	7	8.355	0.96	40	d	0.001093
			217597_x_at	0	7.021	0.37	7	5.974	0.6	40	d	0.00007
DKK2	3	dickkopf homolog 2 (Xenopus laevis)	219908_at	3	4.034	0.91	7	2.751	0.31	40	d	0.013889
			224199_at	2	3.233	0.45	7	3.5	0.55	40	u	0.240151
LBX1	3	ladybird homeobox 1	208380_at	3	2.312	0.03	7	2.361	0.04	40	u	0.005785
CAND1	3	cullin-associated and neddylation-dissociated 1	208839_s_at	3	8.14	0.25	7	8.7	0.72	40	u	0.001184
			208838_at	3	8.796	0.2	7	9.346	0.73	40	u	0.000489
			239771_at	0	5.125	0.61	7	6.201	0.93	40	u	0.005958
			207483_s_at	0	8.601	0.22	7	9.285	0.64	40	u	0.000038
NASP	3	nuclear autoantigenic sperm protein (histone-binding)	201969_at	3	6.592	0.3	7	6.251	0.88	40	d	0.078774
			201970_s_at	1	9.857	0.19	7	9.896	0.78	40	u	0.790251
			242918_at	0	3.039	0.51	7	4.417	1.25	40	u	0.007424
CNN1	3	calponin 1, basic, smooth muscle	203951_at	3	9.821	0.64	7	5.017	1.23	40	d	0
HISPPD2A	3	histidine acid phosphatase domain containing 2A	204578_at	3	6.415	0.63	7	5.688	1.08	40	d	0.097098
			1555255_a_at	0	5.998	0.45	7	5.691	0.76	40	d	0.314984
ZIC2	3	Zic family member 2 (odd-paired homolog, Drosophila)	223642_at	3	2.661	0.13	7	3.389	1.03	40	u	0.00012
ZCCHC5	3	zinc finger, CCHC domain containing 5	1552935_at	3	2.493	0.19	7	2.619	0.42	40	u	0.446112
MKNK1	3	MAP kinase interacting serine/threonine kinase 1	209467_s_at	3	8.192	0.2	7	7.823	0.34	40	d	0.009388
			1560720_at	0	3.178	0.29	7	3.181	0.21	40	u	0.973285
			243256_at	0	6.497	0.54	7	5.795	0.91	40	d	0.059652
NKD1	3	naked cuticle homolog 1 (Drosophila)	1553115_at	3	2.522	0.06	7	2.553	0.13	40	u	0.534632
HTR7	3	5-hydroxytryptamine (serotonin) receptor 7 (adenylate cyclase-coupled)	236281_x_at	3	3.688	0.28	7	3.58	0.27	40	d	0.350405
			207927_at	0	3.667	0.38	7	3.549	0.49	40	d	0.555959
			207818_s_at	0	2.993	0.11	7	3.057	0.25	40	u	0.521877
			216098_s_at	0	2.209	0.02	7	2.274	0.06	40	u	0.011766
YTHDF1	3	YTH domain family, member 1	221741_s_at	3	9.712	0.21	7	9.786	0.53	40	u	0.721068
CABP1	3	calcium binding protein 1	208321_s_at	3	4.695	0.45	7	4.448	0.53	40	d	0.264588
			208320_at	3	2.237	0.09	7	2.318	0.14	40	u	0.146118
			210181_s_at	0	2.705	0.23	7	2.555	0.28	40	d	0.200062
EMP2	3	epithelial membrane protein 2	204975_at	1	10.38	0.46	7	9.429	1.21	40	d	0.050905
			225079_at	0	9.771	0.35	7	9.256	1.11	40	d	0.03139
			238500_at	0	2.918	0.28	7	3.189	0.55	40	u	0.220082
			225078_at	0	10.06	0.43	7	9.316	1	40	d	0.064563

HYOU1	3	hypoxia up-regulated 1	200825_s_at	3	10.13	0.32	7	9.912	0.79	40	d	0.477215
ZNF25	3	zinc finger protein 25	228185_at	3	7.082	0.22	7	6.612	0.78	40	d	0.00424
			235164_at	1	4.028	0.33	7	4.029	0.67	40	u	0.996549
TPCN1	3	two pore segment channel 1	1557185_at	3	5.577	0.24	7	5.35	0.44	40	d	0.201705
			217914_at	3	9.339	0.35	7	7.826	1.05	40	d	0
			1557186_s_at	3	5.205	0.45	7	4.756	0.71	40	d	0.121223
PCBP4	3	poly(rC) binding protein 4	209361_s_at	3	7.581	0.33	7	7.051	0.82	40	d	0.106043
ABHD2	3	abhydrolase domain containing 2	228490_at	3	5.066	0.28	7	6.778	1.34	40	u	0
			87100_at	0	4.9	0.53	7	5.775	1.71	40	u	0.017638
			205566_at	0	5.047	0.34	7	6.307	1.4	40	u	0.000028
			225337_at	0	7.959	0.24	7	7.47	1.3	40	d	0.039048
			221815_at	0	6.396	0.16	7	7.015	1.31	40	u	0.007378
			63825_at	0	6.124	0.31	7	6.515	1.51	40	u	0.159383
PDZRN3	3	PDZ domain containing RING finger 3	212915_at	3	8.934	0.47	7	7.411	1.06	40	d	0.000672
			238165_at	0	2.801	0.4	7	2.578	0.21	40	d	0.227254
			1555587_at	0	3.333	0.3	7	3.415	0.37	40	u	0.594222
SNF1LK2	3	SNF1-like kinase 2	1556056_at	3	2.488	0.25	7	2.421	0.26	40	d	0.540445
			223430_at	3	7.325	0.23	7	6.254	0.61	40	d	0.00005
			215752_at	0	2.251	0.03	7	2.313	0.06	40	u	0.018791
PPFIA3	3	protein tyrosine phosphatase, receptor type, f polypeptide (PTPRF), interacting protein	213368_x_at	3	4.479	0.5	7	4.709	0.78	40	u	0.466123
			215280_s_at	3	3.166	0.4	7	3.212	0.85	40	u	0.890563
FNDC8	3	fibronectin type III domain containing 8	220499_at	3	3.769	0.27	7	3.844	0.44	40	u	0.672313
PIP4K2B	3	phosphatidylinositol-5-phosphate 4-kinase, type II, beta	201081_s_at	3	5.831	0.22	7	5.859	0.86	40	u	0.864679
			201080_at	3	8.172	0.37	7	7.591	0.96	40	d	0.127781
			1553047_at	0	4.847	0.62	7	5.161	0.5	40	u	0.154559
			1553048_a_at	0	4.084	0.36	7	4.583	0.97	40	u	0.195087
BAMBI	3	BMP and activin membrane-bound inhibitor homolog (Xenopus laevis)	203304_at	3	9.267	0.71	7	9.306	1.92	40	u	0.959713
TRIP12	3	thyroid hormone receptor interactor 12	201546_at	3	9.972	0.16	7	10.17	0.5	40	u	0.066456
			244659_at	0	6.242	0.52	7	6.337	0.93	40	u	0.799168
SENP7	3	SUMO1/sentrin specific peptidase 7	223444_at	3	7.599	0.49	7	7.401	0.69	40	d	0.482202
			220735_s_at	3	4.946	0.54	7	4.355	1.1	40	d	0.182103
MOBKL2B	3	MOB1, Mps One Binder kinase activator-like 2B (yeast)	226844_at	3	7.964	0.71	7	5.78	1.44	40	d	0.000386
			229568_at	3	5.579	0.58	7	3.596	0.85	40	d	0.000001
			219265_at	3	4.458	0.37	7	4.681	0.55	40	u	0.319134
			1563521_at	0	4.293	0.45	7	4.382	0.56	40	u	0.700102
ZNF395	3	zinc finger protein 395	218149_s_at	3	8.838	0.39	7	7.177	0.86	40	d	0.000012
			222536_s_at	3	5.529	0.59	7	4.516	0.65	40	d	0.000453
			232694_at	0	3.138	0.69	7	2.521	0.33	40	d	0.073567
			221123_x_at	0	8.157	0.6	7	6.232	0.91	40	d	0.000004
TMEM33	3	transmembrane protein 33	218465_at	3	7.461	0.48	7	7.985	0.68	40	u	0.062577
			222642_s_at	1	6.705	0.69	7	7.377	0.86	40	u	0.060747
GDAP1L1	3	ganglioside-induced differentiation-associated protein 1-like 1	219668_at	0	3.522	0.33	7	3.433	0.33	40	d	0.530622
PIP5K3	3	phosphatidylinositol-3-phosphate/phosphatidylinositol 5-kinase, type III	213111_at	3	8.731	0.11	7	8.118	0.73	40	d	0.000014
			1553917_at	0	3.099	0.31	7	3.428	0.56	40	u	0.148242
			1557719_at	0	3.562	0.42	7	3.918	0.86	40	u	0.297124
ARL2BP	3	ADP-ribosylation factor-like 2 binding protein	202092_s_at	3	8.603	0.28	7	8.639	0.64	40	u	0.888403
			202091_at	3	2.553	0.06	7	2.738	0.41	40	u	0.011594
PRRG1	3	proline rich Gla (G-carboxyglutamic acid) 1	205618_at	3	5.7	0.61	7	6.043	1.17	40	u	0.461263
EFHA2	3	EF-hand domain family, member A2	238458_at	3	5.089	0.57	7	3.194	0.47	40	d	0
MED12	3	mediator complex subunit 12	211342_x_at	3	7.68	0.52	7	6.548	0.94	40	d	0.003957
			216071_x_at	3	7.673	0.48	7	6.713	0.69	40	d	0.001147
			203506_s_at	0	7.189	0.6	7	6.151	0.96	40	d	0.00926
			214275_at	0	1.999	0.02	7	2.031	0.02	40	u	0.001851
C9orf25	3	chromosome 9 open reading frame 25	225146_at	3	7.525	0.29	7	7.664	0.74	40	u	0.634163
NKTR	3	natural killer-tumor recognition sequence	202380_s_at	2	9.46	0.18	7	8.232	0.84	40	d	0
			202379_s_at	2	10.74	0.25	7	10.22	0.71	40	d	0.002184
			1557736_at	0	4.648	0.64	7	3.965	0.68	40	d	0.019946
			1557737_s_at	0	4.023	0.37	7	4.278	0.76	40	u	0.397431
			231235_at	0	3.436	0.59	7	4.869	1.2	40	u	0.004133
			1570342_at	0	2.953	0.14	7	3.329	0.44	40	u	0.000291
			215338_s_at	0	8.335	0.35	7	8.544	0.7	40	u	0.451271

			215339_at	0	4.387	0.5	7	4.643	0.79	40	u	0.421892
RHOBTB2	3	Rho-related BTB domain containing 2	209441_at	3	4.953	0.91	7	4.575	0.69	40	d	0.218721
			1554586_a_at	2	3.839	0.54	7	3.777	0.59	40	d	0.799954
TBC1D9B	3	TBC1 domain family, member 9B (with GRAM domain)	212052_s_at	3	9.16	0.17	7	8.831	0.61	40	d	0.009512
			206431_x_at	3	7.088	0.25	7	6.603	0.63	40	d	0.056329
			215994_x_at	3	8.369	0.27	7	7.972	0.49	40	d	0.046315
			212054_x_at	3	8.821	0.35	7	8.248	0.65	40	d	0.031355
AP3B1	3	adaptor-related protein complex 3, beta 1 subunit	203141_s_at	3	6.927	0.2	7	7.375	0.47	40	u	0.020443
			203142_s_at	1	8.235	0.34	7	8.086	0.61	40	d	0.540697
FUBP1	3	far upstream element (FUSE) binding protein 1	214093_s_at	0	9.68	0.39	7	9.003	0.61	40	d	0.00756
			203091_at	0	8.355	0.12	7	7.996	0.78	40	d	0.010876
			214094_at	0	4.58	0.72	7	4.394	0.78	40	d	0.569807
			212847_at	0	7.615	0.43	7	7.413	0.72	40	d	0.485164
YWHAZ	3	tyrosine 3-monooxygenase/tryptophan 5-monooxygenase activation protein, zeta polypeptide	200638_s_at	3	11.55	0.29	7	12.36	0.87	40	u	0.000162
			200640_at	3	12.05	0.21	7	12.58	0.67	40	u	0.000708
			200639_s_at	3	12.22	0.21	7	13	0.49	40	u	0.000219
			200641_s_at	0	10.39	0.6	7	11.18	1.39	40	u	0.150959
YIPF6	3	Yip1 domain family, member 6	212340_at	3	6.112	0.52	7	6.971	0.87	40	u	0.017101
			212341_at	3	9.808	0.23	7	9.788	0.64	40	d	0.888595
			212342_at	3	6.808	0.36	7	7.092	0.65	40	u	0.273591
			212343_at	3	8.228	0.25	7	7.405	0.89	40	d	0.000046
TMEM25	3	transmembrane protein 25	226647_at	3	7.011	0.48	7	5.885	1.16	40	d	0.016714
			238514_at	0	5.417	0.42	7	4.617	0.68	40	d	0.005122
BMP6	3	bone morphogenetic protein 6	206176_at	4	2.413	0.23	7	2.542	0.87	40	u	0.447037
			215042_at	1	2.247	0.13	7	2.274	0.14	40	u	0.652646
			241141_at	0	2.376	0.11	7	2.321	0.11	40	d	0.250953
OSBPL10	3	oxysterol binding protein-like 10	219073_s_at	3	7.311	0.55	7	7.336	1.1	40	u	0.953989
			222818_at	3	2.309	0.07	7	2.374	0.19	40	u	0.138864
			231656_x_at	0	2.409	0.12	7	2.666	0.53	40	u	0.012434
			216755_at	0	5.803	0.5	7	5.907	0.51	40	u	0.627234
			217017_at	0	2.756	0.04	7	2.806	0.08	40	u	0.114749
C20orf77	3	chromosome 20 open reading frame 77	225024_at	3	7.36	0.16	7	7.311	0.57	40	d	0.667842
			230801_at	0	3.485	0.26	7	3.455	0.41	40	d	0.856762
MEIS1	3	Meis homeobox 1	1559477_s_at	3	5.071	0.93	7	3.2	0.76	40	d	0.000001
			204069_at	3	7.679	0.63	7	5.317	1.11	40	d	0.000003
			242172_at	0	3.802	0.4	7	3.405	0.51	40	d	0.063524
COL23A1	3	collagen, type XXIII, alpha 1	229168_at	3	2.494	0.86	7	2.065	0.08	40	d	0.268231
AMMECR1	3	Alport syndrome, mental retardation, midface hypoplasia and elliptocytosis chromosomal region, gene 1	204976_s_at	3	6.718	0.68	7	7.77	1.08	40	u	0.018911
			226421_at	3	5.543	0.66	7	6.406	1.08	40	u	0.050846
			1553219_a_at	2	2.176	0.12	7	2.946	1.18	40	u	0.00028
			236760_at	0	3.034	0.22	7	3.364	0.51	40	u	0.104663
C6orf60	3	chromosome 6 open reading frame 60	220150_s_at	3	5.567	0.59	7	4.794	0.85	40	d	0.028556
			1558523_at	3	4.007	0.82	7	4.077	0.98	40	u	0.861481
FYN	3	FYN oncogene related to SRC, FGR, YES	210105_s_at	3	8.245	0.54	7	7.681	0.88	40	d	0.11577
			212486_s_at	2	4.176	0.75	7	3.675	0.97	40	d	0.20948
			1559101_at	0	4.886	0.66	7	4.881	0.6	40	d	0.983336
			216033_s_at	0	7.455	0.57	7	6.724	1.03	40	d	0.079737
SNX13	3	sorting nexin 13	213292_s_at	3	7.053	0.32	7	7.713	0.72	40	u	0.023337
			1553148_a_at	3	4.674	0.68	7	4.803	1.05	40	u	0.761071
			215820_x_at	0	4.08	0.31	7	5.098	1.11	40	u	0.000047
			215366_at	0	2.816	0.17	7	3.107	0.41	40	u	0.076277
MEMO1	3	mediator of cell motility 1	219065_s_at	3	8.859	0.24	7	9.923	0.66	40	u	0.000156
			1555522_s_at	0	7.032	0.37	7	8.204	0.68	40	u	0.000076
NFKBIZ	3	nuclear factor of kappa light polypeptide gene enhancer in B-cells inhibitor, zeta	223217_s_at	3	10.08	0.56	7	8.844	1.18	40	d	0.010903
			223218_s_at	3	10.27	0.48	7	8.715	1.44	40	d	0.000024
			1556153_s_at	0	2.73	0.05	7	2.887	0.24	40	u	0.000784
CENTB2	3	centaurin, beta 2	212477_at	3	2.249	0.04	7	2.571	0.41	40	u	0.000018
			212476_at	3	8.727	0.34	7	9.069	0.66	40	u	0.1958
			1552472_a_at	0	3.95	0.24	7	5.603	1.17	40	u	0
MCFD2	3	multiple coagulation factor deficiency 2	212245_at	3	9.214	0.18	7	9.131	0.54	40	d	0.467561
			212246_at	3	7.344	0.49	7	7.786	0.72	40	u	0.134187
			1559309_at	0	2.524	0.16	7	2.559	0.26	40	u	0.742767
ATXN7	3	ataxin 7	204516_at	3	8.159	0.31	7	7.487	0.57	40	d	0.004793
			209964_s_at	0	7.337	0.31	7	6.253	0.87	40	d	0.000008

BRUNOL4	3	bruno-like 4, RNA binding protein (Drosophila)	231220_at	0	3.251	0.17	7	3.625	0.66	40	u	0.005663
			232719_at	0	2.226	0.05	7	2.234	0.03	40	u	0.735027
			223653_x_at	0	2.09	0.12	7	2.115	0.12	40	u	0.630941
			223654_s_at	0	2.683	0.03	7	3.202	0.84	40	u	0.000437
			238966_at	0	2.364	0.12	7	2.791	0.83	40	u	0.00413
KIAA0241	3	KIAA0241	212475_at	0	5.931	0.43	7	6.462	0.64	40	u	0.045769
			212471_at	0	6.017	0.23	7	6.191	0.46	40	u	0.340752
			1555055_at	0	2.758	0.05	7	2.888	0.16	40	u	0.000628
			212474_at	0	8.496	0.26	7	8.761	0.62	40	u	0.282598
CCNK	3	cyclin K	225824_at	3	8.892	0.22	7	8.573	0.57	40	d	0.161674
			219273_at	0	2.462	0.24	7	2.967	0.93	40	u	0.007268
C1GALT1	3	core 1 synthase, glycoprotein-N-acetylgalactosamine 3-beta-galactosyltransferase, 1	219439_at	3	6.088	0.33	7	7.028	1.24	40	u	0.000385
HNF4G	3	hepatocyte nuclear factor 4, gamma	232271_at	3	3.184	0.31	7	3.677	0.76	40	u	0.104778
			207456_at	2	2.109	0.02	7	2.257	0.24	40	u	0.000511
LRCH4	3	leucine-rich repeats and calponin homology (CH) domain containing 4	37796_at	3	6.226	0.16	7	6.277	0.39	40	u	0.739663
			90610_at	3	7.308	0.2	7	7.072	0.66	40	d	0.086175
			204692_at	3	6.307	0.38	7	6.013	0.74	40	d	0.316613
			222017_x_at	3	4.366	0.64	7	3.93	0.67	40	d	0.124907
			221956_at	2	7.393	0.29	7	7.041	0.38	40	d	0.026037
ZNF740	3	zinc finger protein 740	1558277_at	3	3.574	0.47	7	3.907	0.52	40	u	0.1308
ATXN2	3	ataxin 2	202622_s_at	2	8.156	0.21	7	7.417	0.81	40	d	0.000035
NET1	3	neuroepithelial cell transforming gene 1	201829_at	3	9.129	0.35	7	10.13	0.84	40	u	0.003741
			201830_s_at	1	8.827	0.41	7	10.32	1.16	40	u	0.000003
SGCZ	3	sarcoglycan zeta	1552971_at	3	2.279	0.04	7	2.341	0.08	40	u	0.04254
SH2D3C	3	SH2 domain containing 3C	226673_at	3	6.554	0.28	7	5.929	0.53	40	d	0.004382
			215639_at	0	2.235	0.02	7	2.306	0.1	40	u	0.000256
			1552667_a_at	0	4.987	0.91	7	4.179	0.74	40	d	0.015416
NFATC3	3	nuclear factor of activated T-cells, cytoplasmic, calcineurin-dependent 3	207416_s_at	3	6.581	0.23	7	6.229	0.4	40	d	0.030223
			210555_s_at	0	7.729	0.32	7	7.48	0.79	40	d	0.427435
			210556_at	0	3.126	0.52	7	2.98	0.31	40	d	0.528825
PDE3A	3	phosphodiesterase 3A, cGMP-inhibited	206388_at	3	2.21	0.05	7	2.251	0.11	40	u	0.366135
			206389_s_at	3	2.602	0.07	7	2.84	0.63	40	u	0.028631
PCDH11X	3	protocadherin 11 X-linked	210292_s_at	3	2.831	0.09	7	3.099	0.46	40	u	0.00253
			208366_at	0	2.871	0.08	7	2.95	0.18	40	u	0.272841
			1557880_at	0	2.069	0.02	7	2.153	0.15	40	u	0.001691
ZNF354A	3	zinc finger protein 354A	205427_at	3	6.022	0.24	7	6.439	0.85	40	u	0.019054
CDR2L	3	cerebellar degeneration-related protein 2-like	213230_at	3	7.757	0.35	7	7.883	0.86	40	u	0.709583
PITPNM2	3	phosphatidylinositol transfer protein, membrane-associated 2	1552924_a_at	3	2.61	0.23	7	2.709	0.39	40	u	0.527379
			1552923_a_at	3	2.643	0.2	7	2.804	0.38	40	u	0.28644
			232950_s_at	3	4.127	0.39	7	4.051	0.74	40	d	0.794977
SLC7A11	3	solute carrier family 7, (cationic amino acid transporter, y+ system) member 11	209921_at	0	4.878	0.57	7	6.382	1.25	40	u	0.003836
			217678_at	0	4.052	0.81	7	5.437	1.1	40	u	0.003156
			207528_s_at	0	1.926	0.06	7	2.105	0.29	40	u	0.001645
DERL2	3	Der1-like domain family, member 2	218333_at	3	9.256	0.25	7	8.916	0.59	40	d	0.146412
BRWD2	3	bromodomain and WD repeat domain containing 2	218090_s_at	3	8.709	0.31	7	8.608	0.97	40	d	0.618949
			229694_at	0	7.523	0.41	7	7.443	1.26	40	d	0.762419
SMCR7	3	Smith-Magenis syndrome chromosome region, candidate 7	235896_s_at	3	6.296	0.54	7	5.767	0.95	40	d	0.167391
RUFY1	3	RUN and FYVE domain containing 1	218243_at	3	8.365	0.36	7	7.762	0.69	40	d	0.031214
			229179_at	0	2.227	0.05	7	2.31	0.16	40	u	0.020284
			233380_s_at	0	7.097	0.28	7	6.63	0.73	40	d	0.109287
			233381_at	0	4.518	0.43	7	4.264	0.59	40	d	0.290798
TXNDC12	3	thioredoxin domain containing 12 (endoplasmic reticulum)	223017_at	3	9.509	0.15	7	9.575	0.52	40	u	0.530508
PCSK2	3	proprotein convertase subtilisin/kexin type 2	204869_at	3	2.646	0.11	7	2.919	0.83	40	u	0.056718
			204870_s_at	0	2.277	0.05	7	2.588	1	40	u	0.061561
TSPAN2	3	tetraspanin 2	227236_at	3	4.375	0.57	7	4.991	1.72	40	u	0.099316
			227233_at	3	3.394	0.38	7	3.686	0.75	40	u	0.332808
			214606_at	1	3.448	0.19	7	3.989	0.62	40	u	0.000165
RIPK5	3	receptor interacting protein kinase 5	229017_s_at	3	7.267	0.33	7	7.201	0.82	40	d	0.838019
			214663_at	3	6.648	0.42	7	6.703	0.81	40	u	0.86372

RIPK3	3	receptor interacting protein kinase 3	211514_at	0	3.962	0.22	7	4.003	0.26	40	u	0.702054
			211515_s_at	0	4.435	0.61	7	4.857	0.85	40	u	0.2233
PIP5K1C	3	phosphatidylinositol-4-phosphate 5-kinase, type I, gamma	212518_at	3	8.046	0.14	7	7.185	0.77	40	d	0
RAB4B	3	RAB4B, member RAS oncogene family	233385_x_at	3	7.195	0.18	7	7.043	0.54	40	d	0.194493
			228521_s_at	3	7.046	0.18	7	6.847	0.71	40	d	0.153146
			219807_x_at	3	6.635	0.29	7	6.399	0.69	40	d	0.383901
			237257_at	0	4.218	0.29	7	4.445	0.71	40	u	0.418817
HUNK	3	hormonally upregulated Neu-associated kinase	1555935_s_at	3	4.174	0.55	7	3.68	1.02	40	d	0.228667
			219535_at	3	4.535	0.32	7	4.341	0.42	40	d	0.262557
GNA13	3	guanine nucleotide binding protein (G protein), alpha 13	224761_at	2	10.44	0.25	7	10.87	0.86	40	u	0.018751
			206917_at	0	4.046	0.68	7	5.866	2.03	40	u	0.000229
PDK4	3	pyruvate dehydrogenase kinase, isozyme 4	225207_at	3	9.893	1.16	7	6.329	1.46	40	d	0
			205960_at	2	5.929	1.67	7	4.184	1.04	40	d	0.000791
			1562321_at	0	4.507	0.86	7	3.5	0.28	40	d	0.029729
SSBP2	3	single-stranded DNA binding protein 2	203787_at	3	8.943	0.24	7	7.892	1.14	40	d	0.000008
			210829_s_at	0	8.69	0.22	7	7.019	1.26	40	d	0
SPAST	3	spastin	209748_at	3	5.661	0.5	7	6.717	0.82	40	u	0.002399
			207724_s_at	1	4.23	0.22	7	5.53	1.14	40	u	0
PLXND1	3	plexin D1	212235_at	3	6.938	0.41	7	6.38	0.71	40	d	0.053646
			38671_at	3	7.839	0.52	7	7.555	0.83	40	d	0.394216
			1563657_at	0	3.592	0.33	7	3.945	0.34	40	u	0.015506
CCDC47	3	coiled-coil domain containing 47	217814_at	3	9.825	0.18	7	9.722	0.79	40	d	0.48778
			222432_s_at	2	9.541	0.42	7	9.566	1.11	40	u	0.953628
PARP8	3	poly (ADP-ribose) polymerase family, member 8	219033_at	3	6.559	0.27	7	6.303	1.13	40	d	0.235662
BIN1	3	bridging integrator 1	202931_x_at	3	7.31	0.52	7	6.225	0.6	40	d	0.000062
			214643_x_at	3	3.2	0.74	7	3.115	0.59	40	d	0.741879
			210201_x_at	3	7.535	0.45	7	6.498	0.6	40	d	0.000096
			214439_x_at	3	7.41	0.53	7	6.235	0.79	40	d	0.000539
			210202_s_at	2	6.723	0.45	7	5.732	0.82	40	d	0.003879
TIMM8A	3	translocase of inner mitochondrial membrane 8 homolog A (yeast)	205217_at	3	3.891	0.3	7	4.48	0.93	40	u	0.005012
			210800_at	0	3.78	0.28	7	4.364	0.57	40	u	0.012894
UCHL3	3	ubiquitin carboxyl-terminal esterase L3 (ubiquitin thiolesterase)	204616_at	2	8.694	0.54	7	8.552	0.9	40	d	0.696917
NCOA5	3	nuclear receptor coactivator 5	234471_s_at	3	6.392	0.33	7	6.115	0.96	40	d	0.185267
			225145_at	3	7.922	0.38	7	7.794	0.55	40	d	0.562627
VASH2	3	vasohibin 2	219740_at	3	3.892	0.69	7	5.426	2.09	40	u	0.001729
			1555092_at	0	2.188	0.16	7	2.456	0.55	40	u	0.020629
ZNF664	3	zinc finger protein 664	224593_at	3	11.08	0.28	7	10.85	0.7	40	d	0.412396
			1569935_at	0	3.608	0.57	7	4.034	0.59	40	u	0.090324
			1558816_at	0	4.558	0.49	7	4.916	0.79	40	u	0.262485
			1569936_a_at	0	2.379	0.03	7	2.451	0.06	40	u	0.002142
COL5A3	3	collagen, type V, alpha 3	52255_s_at	3	4.888	0.96	7	4.768	1.08	40	d	0.787719
			218975_at	3	5.843	0.71	7	5.64	1.02	40	d	0.621941
C11orf59	3	chromosome 11 open reading frame 59	223009_at	3	9.975	0.13	7	9.679	0.65	40	d	0.015369
EXT1	3	exostoses (multiple) 1	201995_at	2	7.95	0.28	7	8.413	0.8	40	u	0.013033
SMAD2	3	SMAD family member 2	203077_s_at	3	8.371	0.16	7	8.414	0.59	40	u	0.715382
			203075_at	3	8.699	0.11	7	8.427	0.74	40	d	0.036274
			235598_at	3	4.388	0.42	7	4.926	0.85	40	u	0.11584
			226563_at	2	8.16	0.37	7	7.541	0.92	40	d	0.093203
			203076_s_at	2	8.852	0.35	7	8.803	0.71	40	d	0.860674
			239271_at	0	3.893	0.43	7	3.979	0.65	40	u	0.74102
PALM	3	paralemmin	203859_s_at	3	8.18	0.38	7	6.661	0.99	40	d	0.000297
CDC14B	3	CDC14 cell division cycle 14 homolog B (S. cerevisiae)	211348_s_at	4	3.652	0.43	7	3.423	0.51	40	d	0.280387
			211347_at	4	2.431	0.11	7	2.415	0.13	40	d	0.768082
			221556_at	3	7.207	0.28	7	5.765	0.87	40	d	0
			221555_x_at	3	6.299	0.43	7	5.586	0.87	40	d	0.042583
			208022_s_at	2	6.91	0.21	7	5.883	0.89	40	d	0
			234740_at	0	3.082	0.11	7	3.109	0.23	40	u	0.762985
			234605_at	0	2.951	0.39	7	3.214	1	40	u	0.50303
			1553320_s_at	0	3.044	0.27	7	3.121	0.2	40	u	0.381282
			230887_at	0	2.865	0.35	7	3.024	0.57	40	u	0.487712
PHC1	3	polyhomeotic homolog 1 (Drosophila)	225958_at	7	6.804	0.44	7	6.378	0.73	40	d	0.147733

PHC1	3	polyhomeotic homolog 1 (Drosophila)	218338_at	1	7.813	0.34	7	7.097	0.83	40	d	0.03231
PPP3CB	3	protein phosphatase 3 (formerly 2B), catalytic subunit, beta isoform	202432_at	3	9.814	0.22	7	9.088	0.67	40	d	0.000022
			209817_at	0	6.929	0.39	7	7.275	0.93	40	u	0.346683
SSBP3	3	single stranded DNA binding protein 3	217991_x_at	3	6.877	0.38	7	6.674	1.01	40	d	0.611361
			222482_at	3	7.633	0.42	7	6.861	1.12	40	d	0.084508
			223635_s_at	3	7.759	0.7	7	7.652	1.15	40	d	0.817632
MMD2	3	monocyte to macrophage differentiation-associated 2	230826_at	3	4.538	0.39	7	4.429	0.42	40	d	0.536319
			1569998_at	0	2.915	0.08	7	3.11	0.22	40	u	0.000337
KLHL1	3	kelch-like 1 (Drosophila)	223810_at	3	2.482	0.03	7	2.696	0.75	40	u	0.083546
			216391_s_at	0	2.339	0.15	7	2.247	0.14	40	d	0.12692
HN1L	3	hematological and neurological expressed 1-like	212115_at	2	9.677	0.45	7	10.32	0.57	40	u	0.007386
			212109_at	2	7.413	0.44	7	7.738	0.64	40	u	0.209503
			216031_x_at	0	5.925	0.39	7	6.43	0.68	40	u	0.069677
GADD45A	3	growth arrest and DNA-damage-inducible, alpha	203725_at	3	8.631	0.66	7	8.496	0.98	40	d	0.734067
DKK3	3	dickkopf homolog 3 (Xenopus laevis)	214247_s_at	3	10.64	0.34	7	8.174	1.14	40	d	0
			202196_s_at	3	7.986	0.34	7	5.589	1.1	40	d	0
			230508_at	0	3.9	0.6	7	2.975	0.38	40	d	0.000004
PPP1R14B	3	protein phosphatase 1, regulatory (inhibitor) subunit 14B	226138_s_at	3	2.854	0.26	7	2.798	0.36	40	d	0.708127
			212680_x_at	3	8.814	0.15	7	9.878	0.92	40	u	0
			230374_at	0	5.778	0.21	7	5.759	0.36	40	d	0.89478
PSD	3	pleckstrin and Sec7 domain containing	208102_s_at	3	3.937	0.21	7	3.668	0.32	40	d	0.041661
RASL12	3	RAS-like, family 12	219167_at	3	6.292	0.6	7	5.191	1.43	40	d	0.056429
ANKHD1	3	ankyrin repeat and KH domain containing 1	208772_at	2	8.889	0.36	7	8.45	0.63	40	d	0.088256
			208773_s_at	2	9.033	0.26	7	8.421	0.64	40	d	0.018849
			219081_at	0	6.786	0.45	7	7.004	0.68	40	u	0.424387
			229457_at	0	6.535	0.42	7	5.809	0.67	40	d	0.00966
			233292_s_at	0	6.883	0.44	7	6.139	1.47	40	d	0.017287
VPS37A	3	vacuolar protein sorting 37 homolog A (S. cerevisiae)	225378_at	3	6.487	0.47	7	6.687	0.88	40	u	0.567191
			228024_at	3	6.87	0.46	7	6.3	0.92	40	d	0.125042
			216176_at	0	5.269	0.79	7	5.441	1.62	40	u	0.788195
			216174_at	0	2.638	0.15	7	3.093	0.98	40	u	0.009466
CFL1	3	cofilin 1 (non-muscle)	200021_at	3	13.01	0.18	7	13.47	0.36	40	u	0.00238
			1555730_a_at	0	11.26	0.18	7	11.71	0.91	40	u	0.009355
HMG20A	3	high-mobility group 20A	218152_at	3	7.954	0.33	7	7.72	0.58	40	d	0.3185
ITFG3	3	integrin alpha FG-GAP repeat containing 3	224749_at	3	8.068	0.25	7	7.853	0.77	40	d	0.189666
C10orf137	3	chromosome 10 open reading frame 137	213410_at	3	7.582	0.2	7	7.722	0.71	40	u	0.320744
			215072_x_at	0	2.818	0.07	7	3.067	0.39	40	u	0.00075
			208115_x_at	0	2.144	0.22	7	2.279	0.39	40	u	0.385363
TNKS1BP1	3	tankyrase 1 binding protein 1, 182kDa	224792_at	0	7.735	0.38	7	7.015	0.87	40	d	0.041016
C1orf83	3	chromosome 1 open reading frame 83	238737_at	3	3.585	0.59	7	3.701	0.63	40	u	0.661756
			1562470_at	1	2.236	0.03	7	2.335	0.13	40	u	0.000343
NELF	3	nasal embryonic LHRH factor	221214_s_at	3	6.867	0.32	7	6.821	0.85	40	d	0.890552
NRGN	3	neurogranin (protein kinase C substrate, RC3)	204081_at	3	5.901	0.3	7	5.721	0.66	40	d	0.493206
DPF1	3	D4, zinc and double PHD fingers family 1	206531_at	3	3.244	0.42	7	3.138	0.39	40	d	0.526832
GSPT1	3	G1 to S phase transition 1	215438_x_at	3	8.791	0.23	7	9.765	0.5	40	u	0.000011
			201912_s_at	3	9.16	0.26	7	9.808	0.64	40	u	0.013394
			240452_at	0	6.943	0.45	7	6.501	1.41	40	d	0.138713
RGL2	3	ral guanine nucleotide dissociation stimulator-like 2	209110_s_at	0	9.609	0.29	7	8.861	0.84	40	d	0.000312
EXTL3	3	exostoses (multiple)-like 3	209202_s_at	3	5.175	0.38	7	4.96	0.67	40	d	0.425113
			211051_s_at	0	2.656	0.35	7	2.99	0.68	40	u	0.219796
EGLN2	3	egl nine homolog 2 (C. elegans)	220956_s_at	3	9.08	0.18	7	8.825	0.62	40	d	0.048516
			223083_s_at	3	4.921	0.57	7	4.8	0.64	40	d	0.647747
			227147_s_at	0	1.948	0.02	7	1.998	0.06	40	u	0.000767
NPLOC4	3	nuclear protein localization 4 homolog (S. cerevisiae)	217796_s_at	3	7.808	0.21	7	7.732	0.63	40	d	0.572593
FAM76A	3	family with sequence similarity 76, member A	228638_at	3	7.238	0.29	7	7.394	1.11	40	u	0.470104
			216897_s_at	0	2.132	0.01	7	2.175	0.05	40	u	0.000025
ERLIN1	3	ER lipid raft associated 1	202441_at	3	9.105	0.04	7	8.825	0.48	40	d	0.000895
			202444_s_at	0	5.728	0.58	7	5.895	1.5	40	u	0.776634
REPS1	3	RALBP1 associated Eps domain containing 1	224366_s_at	3	8.152	0.34	7	8.225	0.9	40	u	0.837334
			215201_at	0	5.615	0.39	7	5.238	1.01	40	d	0.344789

			215922_at	0	3.45	0.49	7	3.42	0.61	40	d	0.904763
TMED10	3	transmembrane emp24-like trafficking protein 10 (yeast)	212352_s_at	3	11.76	0.14	7	11.14	0.59	40	d	0.000002
			200929_at	2	10.9	0.42	7	10.88	0.83	40	d	0.962691
			238886_at	0	6.489	0.36	7	5.906	0.8	40	d	0.070618
MAFB	3	v-maf musculoaponeurotic fibrosarcoma oncogene homolog B (avian)	218559_s_at	3	9.675	0.43	7	9.014	0.8	40	d	0.04221
			222670_s_at	3	9.175	0.29	7	8.599	0.64	40	d	0.027848
KTELC1	3	KTEL (Lys-Tyr-Glu-Leu) containing 1	222681_at	3	7.204	0.24	7	6.972	0.77	40	d	0.151906
			218587_s_at	3	7.29	0.24	7	7.533	0.76	40	u	0.134001
STK19	3	serine/threonine kinase 19	36019_at	3	7.846	0.18	7	7.82	0.47	40	d	0.890414
			204090_at	3	8.376	0.23	7	8.325	0.46	40	d	0.782442
OTUB1	3	OTU domain, ubiquitin aldehyde binding 1	201246_s_at	3	7.18	0.21	7	7.264	0.86	40	u	0.605649
			38710_at	3	8.848	0.26	7	8.997	0.69	40	u	0.585616
			201245_s_at	3	8.986	0.12	7	8.786	0.82	40	d	0.161514
SH3BGRL3	3	SH3 domain binding glutamic acid-rich protein like 3	221269_s_at	3	9.249	0.3	7	9.547	0.74	40	u	0.306067
VASP	3	vasodilator-stimulated phosphoprotein	202205_at	3	8.011	0.3	7	8.125	0.57	40	u	0.616419
FNTA	3	farnesyltransferase, CAAX box, alpha	200090_at	3	10.87	0.17	7	10.94	0.89	40	u	0.647754
			230808_at	0	4.393	0.24	7	5.072	0.85	40	u	0.000279
			209471_s_at	0	9.564	0.22	7	9.627	0.89	40	u	0.707013
BAG4	3	BCL2-associated athanogene 4	219624_at	3	3.356	0.22	7	4.319	1.25	40	u	0.000071
			222909_s_at	0	4.251	0.33	7	5.624	1.79	40	u	0.000082
			228189_at	0	8.832	0.26	7	9.281	1.2	40	u	0.047792
LAMP2	3	lysosomal-associated membrane protein 2	200821_at	3	9.315	0.33	7	9.465	0.68	40	u	0.576545
			226671_at	0	7.752	0.5	7	7.021	1.26	40	d	0.145859
			203041_s_at	0	9.874	0.44	7	9.726	1.05	40	d	0.719143
			203042_at	0	8.284	0.32	7	8.444	0.85	40	u	0.63212
PTPRM	3	protein tyrosine phosphatase, receptor type, M	1555578_at	3	2.773	0.1	7	2.965	0.19	40	u	0.014056
			1555579_s_at	3	7.719	0.19	7	6.745	1.26	40	d	0.000048
			203329_at	3	7.596	0.2	7	6.789	1.09	40	d	0.000129
LAMC2	3	laminin, gamma 2	202267_at	3	7.884	0.44	7	6.425	1.42	40	d	0.000026
			207517_at	0	3.792	0.4	7	3.346	0.27	40	d	0.000735
USP5	3	ubiquitin specific peptidase 5 (isopeptidase T)	206031_s_at	3	6.864	0.37	7	7.323	0.58	40	u	0.054411
PREPL	3	prolyl endopeptidase-like	212216_at	3	7.862	0.51	7	7.949	0.85	40	u	0.799166
			212215_at	3	9.661	0.28	7	9.262	0.69	40	d	0.147521
			212217_at	3	8.695	0.31	7	8.224	0.73	40	d	0.106586
			243542_at	0	2.996	0.09	7	3.228	0.27	40	u	0.000399
ZMAT2	3	zinc finger, matrin type 2	224782_at	3	9.663	0.23	7	9.471	0.62	40	d	0.429383
EIF4H	3	eukaryotic translation initiation factor 4H	206621_s_at	3	11.29	0.25	7	10.98	0.51	40	d	0.129291
KCNH7	3	potassium voltage-gated channel, subfamily H (eag-related), member 7	224099_at	3	2.279	0.07	7	2.405	0.33	40	u	0.045636
			1555316_a_at	0	2.712	0.16	7	2.744	0.18	40	u	0.677311
USO1	3	USO1 homolog, vesicle docking protein (yeast)	201832_s_at	0	10.41	0.17	7	10.24	0.55	40	d	0.132787
			201831_s_at	0	8.752	0.32	7	9.725	0.86	40	u	0.005876
FASTK	3	Fas-activated serine/threonine kinase	202676_x_at	3	7.783	0.12	7	7.686	0.58	40	d	0.364981
			210975_x_at	3	7.583	0.18	7	7.496	0.66	40	d	0.502168
			214114_x_at	3	8.296	0.14	7	8.249	0.62	40	d	0.68452
PCBP2	3	poly(rC) binding protein 2	213264_at	9	8.508	0.33	7	7.444	0.82	40	d	0.001892
			213263_s_at	9	8.991	0.29	7	7.835	1.06	40	d	0.000003
			204031_s_at	2	12.38	0.25	7	11.35	0.65	40	d	0.000224
			213517_at	0	7.917	0.52	7	8.224	1.07	40	u	0.470801
			237813_at	0	3.808	0.5	7	3.952	0.42	40	u	0.427777
			229467_at	0	6.114	0.74	7	6.415	1.16	40	u	0.521381
RBP3	3	retinol binding protein 3, interstitial	210318_at	0	3.446	0.28	7	3.399	0.19	40	d	0.589194
UBFD1	3	ubiquitin family domain containing 1	205687_at	3	5.464	0.62	7	7.131	0.78	40	u	0.000004
			224878_at	0	8.47	0.27	7	8.59	0.73	40	u	0.672866
PIP5K1B	3	phosphatidylinositol-4-phosphate 5-kinase, type I, beta	217477_at	3	1.992	0.08	7	2.052	0.13	40	u	0.238921
			205632_s_at	3	5.603	0.49	7	4.654	1.26	40	d	0.059807
AFTPH	3	aftiphilin	222472_at	3	8.709	0.24	7	8.803	0.69	40	u	0.533995
			217939_s_at	0	9.727	0.26	7	9.66	0.49	40	d	0.732147
ZMPSTE24	3	zinc metallopeptidase (STE24 homolog, S. cerevisiae)	202939_at	1	10	0.25	7	10.25	0.49	40	u	0.206019
AGBL5	3	ATP/GTP binding protein-like 5	231857_s_at	0	6.787	0.39	7	6.964	0.89	40	u	0.61554
			218480_at	0	6.292	0.31	7	6.351	0.99	40	u	0.77405
			238889_at	0	3.101	0.28	7	3.349	0.43	40	u	0.159722

KCNIP2	3	Kv channel interacting protein 2	223727_at	3	6.403	1.43	7	4.688	0.66	40	d	0.02802
			1555230_a_at	0	2.744	0.73	7	2.178	0.09	40	d	0.10535
			224528_s_at	0	3.35	0.31	7	3.364	0.25	40	u	0.897175
			221321_s_at	0	4.617	0.37	7	4.471	0.35	40	d	0.326275
TSSK2	3	testis-specific serine kinase 2	214371_at	6	2.33	0.03	7	2.372	0.05	40	u	0.036324
			217275_at	0	2.258	0.16	7	2.28	0.26	40	u	0.83373
RAB18	3	RAB18, member RAS oncogene family	223336_s_at	3	10.41	0.32	7	10.37	0.7	40	d	0.88191
			224787_s_at	3	10.52	0.34	7	10.5	0.97	40	d	0.896343
			224377_s_at	2	9.117	0.52	7	9.353	0.82	40	u	0.475164
			229398_at	0	6.14	0.26	7	6.429	0.91	40	u	0.120005
TXLNB	3	taxilin beta	227834_at	3	4.482	0.51	7	4.854	0.85	40	u	0.278487
KIAA0427	3	KIAA0427	204302_s_at	3	5.91	0.2	7	5.719	0.32	40	d	0.14195
			204303_s_at	3	6.403	0.27	7	5.585	0.72	40	d	0.005765
LPIN1	3	lipin 1	212274_at	3	7.447	0.54	7	7.699	1.25	40	u	0.609252
			212272_at	3	4.018	1.2	7	5.175	2.01	40	u	0.154539
			212276_at	3	7.658	0.6	7	7.796	1.25	40	u	0.778977
KIAA1598	3	KIAA1598	221802_s_at	3	8.652	0.62	7	9.012	0.92	40	u	0.337177
PMP22	3	peripheral myelin protein 22	210139_s_at	3	11.44	0.35	7	9.985	1.23	40	d	0.000001
FAM108B1	3	family with sequence similarity 108, member B1	227551_at	3	7.321	0.37	7	7.538	0.68	40	u	0.424108
			228872_at	0	2.776	0.09	7	2.815	0.13	40	u	0.459389
			220285_at	0	4.294	0.7	7	4.882	1.11	40	u	0.192847
PLEKHA5	3	pleckstrin homology domain containing, family A member 5	220952_s_at	3	8.87	0.11	7	8.137	1.2	40	d	0.000609
			233040_at	0	2.993	0.11	7	3.261	0.36	40	u	0.001146
USP28	3	ubiquitin specific peptidase 28	230623_x_at	3	5.353	0.5	7	5.669	0.66	40	u	0.245778
			231837_at	1	5.876	0.45	7	5.926	0.65	40	u	0.847451
			1552678_a_at	0	5.207	0.45	7	5.289	0.75	40	u	0.783819
MTCH2	3	mitochondrial carrier homolog 2 (C. elegans)	222403_at	3	7.384	0.46	7	7.717	0.81	40	u	0.305329
			217772_s_at	1	9.085	0.26	7	9.968	0.78	40	u	0.000013
TFF1	3	trefoil factor 1	205009_at	0	10.4	1.98	7	8.147	3.61	40	d	0.123182
PHLDB2	3	pleckstrin homology-like domain, family B, member 2	225688_s_at	3	8.85	0.55	7	7.422	1.37	40	d	0.010922
			1554778_at	0	3.222	0.6	7	3.184	0.28	40	d	0.883484
			1557578_at	0	5.629	0.87	7	4.332	1.02	40	d	0.003388
			1554779_s_at	0	2.269	0.02	7	2.364	0.12	40	u	0.000052
			238419_at	0	3.308	0.39	7	3.728	0.92	40	u	0.252021
MAP3K11	3	mitogen-activated protein kinase kinase kinase 11	203652_at	3	8.489	0.35	7	8.345	0.8	40	d	0.64864
			1558984_at	0	2.334	0.23	7	2.349	0.22	40	u	0.872748
ZKSCAN2	3	zinc finger with KRAB and SCAN domains 2	232019_at	3	4.362	0.19	7	4.328	0.74	40	d	0.81042
PIM2	3	pim-2 oncogene	204269_at	3	5.949	0.5	7	5.908	0.94	40	d	0.912608
SLC17A5	3	solute carrier family 17 (anion/sugar transporter), member 5	223441_at	3	8.764	0.38	7	8.453	0.84	40	d	0.351744
			221041_s_at	3	7.207	0.69	7	6.651	1.4	40	d	0.318217
NKIRAS2	3	NFKB inhibitor interacting Ras-like 2	218240_at	3	7.551	0.25	7	7.549	0.68	40	d	0.991737
			222105_s_at	3	7	0.37	7	7.367	0.68	40	u	0.181051
TRMT5	3	TRM5 tRNA methyltransferase 5 homolog (S. cerevisiae)	227653_at	3	6.59	0.33	7	6.773	0.71	40	u	0.518806
			221952_x_at	3	11.48	0.24	7	11.24	0.63	40	d	0.326321
C14orf100	3	chromosome 14 open reading frame 100	223547_at	3	7.16	0.3	7	7.044	0.97	40	d	0.567047
			223215_s_at	3	9.503	0.21	7	8.97	0.82	40	d	0.001631
CACNA1G	3	calcium channel, voltage-dependent, T type, alpha 1G subunit	207869_s_at	3	3.833	0.33	7	3.679	0.31	40	d	0.246668
			210380_s_at	3	4.745	0.78	7	4.341	0.53	40	d	0.101424
			211315_s_at	0	4.28	0.31	7	4.164	0.34	40	d	0.418648
			211314_at	0	2.968	0.22	7	2.742	0.41	40	d	0.174032
			211802_x_at	0	2.33	0.32	7	2.124	0.27	40	d	0.081549
SLC19A2	3	solute carrier family 19 (thiamine transporter), member 2	209681_at	3	8.497	0.49	7	8.05	1.63	40	d	0.183069
FNBP1	3	formin binding protein 1	212288_at	3	9.236	0.39	7	8.437	0.76	40	d	0.010813
			213940_s_at	0	7.864	0.28	7	7.666	0.68	40	d	0.462649
			230086_at	0	4.882	0.64	7	5.078	1.4	40	u	0.723275
			230389_at	0	8.431	0.44	7	7.52	1.05	40	d	0.032399
TMTC2	3	transmembrane and tetratricopeptide repeat containing 2	235775_at	3	5.367	0.73	7	5.414	1.02	40	u	0.909391
			228574_at	0	5.265	0.44	7	4.838	0.96	40	d	0.262979
TLX3	3	T-cell leukemia homeobox 3	208495_at	0	2.191	0.03	7	2.283	0.19	40	u	0.005718
HMGB3	3	high-mobility group box 3	203744_at	3	6.089	0.65	7	8.67	1.01	40	u	0
			225601_at	0	6.323	0.47	7	8.187	1.07	40	u	0.000062
			202761_s_at	3	9.358	0.36	7	8.222	0.8	40	d	0.000799

SYNE2	3	spectrin repeat containing, nuclear envelope 2	1558392_at	0	3.097	0.33	7	3.348	0.51	40	u	0.223775
			242774_at	0	8.091	0.38	7	7.299	1.67	40	d	0.014375
			240777_at	0	2.698	0.1	7	2.854	0.3	40	u	0.021124
			243841_at	0	3.422	0.25	7	3.901	0.59	40	u	0.04528
NETO2	3	neuropilin (NRP) and tolloid (TLL)-like 2	222774_s_at	3	5.156	0.4	7	5.713	1.54	40	u	0.068872
			218888_s_at	2	7.172	0.37	7	7.105	1.58	40	d	0.821196
VCPIP1	3	valosin containing protein (p97)/p47 complex interacting protein 1	234988_at	3	3.382	0.24	7	3.941	0.64	40	u	0.030653
			1556227_at	0	3.418	0.3	7	3.45	0.38	40	u	0.837898
			1556228_a_at	0	5.285	0.32	7	5.596	0.7	40	u	0.266113
			219810_at	0	3.527	0.32	7	3.664	0.56	40	u	0.543074
GABARAP	3	GABA(A) receptor-associated protein	200645_at	3	12.7	0.12	7	12.03	0.47	40	d	0
SOX13	3	SRY (sex determining region Y)-box 13	209736_at	3	7.177	0.26	7	6.868	1.07	40	d	0.13435
			38918_at	3	7.276	0.27	7	6.789	1.26	40	d	0.040274
			1569111_at	0	3.821	0.75	7	3.806	0.87	40	d	0.965274
DNAJB1	3	DnaJ (Hsp40) homolog, subfamily B, member 1	200666_s_at	3	10.11	0.56	7	9.789	0.7	40	d	0.262134
			200664_s_at	3	9.505	0.74	7	9.296	0.99	40	d	0.603838
IKZF5	3	IKAROS family zinc finger 5 (Pegasus)	226680_at	3	7.194	0.44	7	6.769	0.57	40	d	0.073279
			220086_at	0	5.886	0.42	7	6.168	0.57	40	u	0.230753
C20orf111	3	chromosome 20 open reading frame 111	209020_at	3	8.004	0.29	7	7.864	0.67	40	d	0.597307
			221954_at	0	6.874	0.28	7	6.563	0.71	40	d	0.270771
TBK1	3	TANK-binding kinase 1	218520_at	2	9.122	0.34	7	9.036	0.47	40	d	0.653374
			1554391_at	0	3.075	0.1	7	3.145	0.18	40	u	0.341485
TMEM86A	3	transmembrane protein 86A	238710_at	3	2.585	0.78	7	2.613	0.7	40	u	0.925308
			227570_at	3	5.962	0.42	7	5.533	0.6	40	d	0.08253
			242103_at	1	5.489	0.48	7	4.682	0.83	40	d	0.017848
CLCN6	3	chloride channel 6	203950_s_at	3	5.181	0.83	7	4.295	0.76	40	d	0.008388
C12orf34	3	chromosome 12 open reading frame 34	226487_at	3	4.945	0.85	7	5.275	1.33	40	u	0.538742
UBE2F	3	ubiquitin-conjugating enzyme E2F (putative)	225783_at	3	8.13	0.31	7	9.129	0.64	40	u	0.000245
			225787_at	3	8.311	0.27	7	9.058	0.7	40	u	0.008698
			231948_s_at	2	6.452	0.33	7	7.365	0.57	40	u	0.000221
DVL2	3	dishevelled, dsh homolog 2 (Drosophila)	57532_at	3	7.175	0.19	7	6.557	0.86	40	d	0.000357
			218759_at	3	6.598	0.32	7	6.006	0.54	40	d	0.008089
ZNF148	3	zinc finger protein 148	203318_s_at	3	9.463	0.26	7	9.554	0.58	40	u	0.690569
			203319_s_at	0	8.767	0.28	7	8.512	0.79	40	d	0.147061
MFAP3	3	microfibrillar-associated protein 3	214588_s_at	3	4.066	0.28	7	4.174	0.51	40	u	0.597542
			1552312_a_at	3	4.504	1.09	7	4.977	1.13	40	u	0.320072
			213123_at	0	8.279	0.14	7	7.664	0.63	40	d	0.000005
DACT3	3	dapper, antagonist of beta-catenin, homolog 3 (Xenopus laevis)	228228_at	3	5.402	0.37	7	5.273	1.11	40	d	0.586877
			229745_x_at	0	3.026	0.32	7	2.809	0.09	40	d	0.146346
TRPV6	3	transient receptor potential cation channel, subfamily V, member 6	206827_s_at	3	7.465	0.46	7	7.054	0.8	40	d	0.204718
			1559405_a_at	0	4.005	0.58	7	3.84	0.49	40	d	0.438595
WDR1	3	WD repeat domain 1	210935_s_at	3	7.76	0.57	7	8.024	1.51	40	u	0.656245
			210936_at	3	3.239	0.21	7	3.342	0.28	40	u	0.37107
			200609_s_at	2	10.98	0.21	7	10.42	0.66	40	d	0.000238
			200611_s_at	1	10.51	0.33	7	10.38	0.66	40	d	0.602932
			244672_at	0	2.387	0.12	7	2.461	0.17	40	u	0.272666
			240282_at	0	7.79	0.86	7	6.134	1.1	40	d	0.000563
MOSPD2	3	motile sperm domain containing 2	64883_at	3	6.886	0.5	7	6.941	0.47	40	u	0.784573
			221895_at	3	6.403	0.53	7	6.73	0.69	40	u	0.250952
REEP3	3	receptor accessory protein 3	235016_at	3	4.247	0.93	7	4.39	1.16	40	u	0.763588
			225785_at	0	9.098	0.37	7	8.774	0.75	40	d	0.280188
PAK6	3	p21(CDKN1A)-activated kinase 6	219461_at	3	4.369	0.83	7	4.332	1.25	40	d	0.94035
			1555310_a_at	3	6.15	0.54	7	6.012	1.25	40	d	0.778562
EDC3	3	enhancer of mRNA decapping 3 homolog (S. cerevisiae)	226042_at	3	5.808	0.38	7	5.672	0.69	40	d	0.62252
			219207_at	1	5.762	0.32	7	6.046	0.8	40	u	0.369987
PPAP2A	3	phosphatidic acid phosphatase type 2A	210946_at	3	10.2	0.36	7	8.302	0.93	40	d	0.000004
			209147_s_at	0	9.814	0.31	7	7.647	1.11	40	d	0
ALS2CR2	3	amyotrophic lateral sclerosis 2 (juvenile) chromosome region, candidate 2	223266_at	3	7.636	0.36	7	6.702	0.83	40	d	0.006296
UHRF2	3	ubiquitin-like, containing PHD and RING finger domains, 2	225610_at	3	9.649	0.33	7	9.432	0.7	40	d	0.432454
CSDE1	3	cold shock domain containing E1, RNA-binding	202646_s_at	4	10.22	0.19	7	9.651	0.67	40	d	0.000158
			222975_s_at	3	11.12	0.22	7	11.2	0.67	40	u	0.607605
			219939_s_at	3	11.45	0.13	7	11.27	0.63	40	d	0.126405

RAD9A	3	RAD9 homolog A (S. pombe)	204828_at	3	6.682	0.45	7	6.542	0.86	40	d	0.68047
			1562022_s_at	0	3.814	0.53	7	4.324	0.99	40	u	0.199263
AGPAT3	3	1-acylglycerol-3-phosphate O-acyltransferase 3	225440_at	2	8.777	0.5	7	8.867	0.61	40	u	0.722563
			219723_x_at	1	4.937	0.92	7	5.629	0.77	40	u	0.042892
			223184_s_at	1	6.622	0.31	7	6.407	0.54	40	d	0.318366
			223183_at	0	6.163	0.22	7	6.047	0.73	40	d	0.430923
			224282_s_at	0	3.842	0.43	7	4.297	0.64	40	u	0.08414
			223182_s_at	0	6.998	0.37	7	6.855	1.05	40	d	0.531707
CAMK2D	3	calcium/calmodulin-dependent protein kinase (CaM kinase) II delta	224994_at	3	7.395	0.29	7	7.573	1.09	40	u	0.401678
			225019_at	3	8.867	0.34	7	8.974	0.89	40	u	0.760698
			231042_s_at	0	2.781	0.06	7	3.464	1.01	40	u	0.000167
			231793_s_at	0	6.537	0.28	7	6.738	1.14	40	u	0.352737
			228555_at	0	6.659	0.5	7	5.752	1.2	40	d	0.059378
			230749_s_at	0	3.09	0.32	7	3.016	0.33	40	d	0.596872
ARHGEF2	3	rho/rac guanine nucleotide exchange factor (GEF) 2	209435_s_at	3	8.891	0.11	7	9.098	0.69	40	u	0.087418
			207629_s_at	1	6.706	0.39	7	6.907	1.12	40	u	0.411414
			235595_at	0	6.564	0.31	7	6.927	0.58	40	u	0.122586
			1554783_s_at	0	2.199	0.07	7	2.529	0.63	40	u	0.00307
PRKD3	3	protein kinase D3	218236_s_at	3	8.14	0.26	7	8.104	1.32	40	d	0.882774
			222565_s_at	3	5.328	0.21	7	5.832	1.28	40	u	0.028298
			1554910_at	0	2.837	0.17	7	3.082	0.48	40	u	0.027658
			242549_at	0	3.223	0.23	7	3.568	0.56	40	u	0.126743
			211084_x_at	0	6.672	0.34	7	6.697	0.84	40	u	0.939146
BNIP2	3	BCL2/adenovirus E1B 19kDa interacting protein 2	209308_s_at	3	8.916	0.34	7	8.739	0.5	40	d	0.381926
PPARG	3	peroxisome proliferator-activated receptor gamma	208510_s_at	0	4.932	0.8	7	3.336	0.97	40	d	0.000209
RHOA	3	ras homolog gene family, member A	200059_s_at	2	12.19	0.13	7	12.04	0.72	40	d	0.222106
			1555814_a_at	0	11.38	0.3	7	10.75	1.62	40	d	0.032998
			240337_at	0	2.172	0.02	7	2.244	0.12	40	u	0.001453
KIAA0174	3	KIAA0174	200851_s_at	3	10.29	0.18	7	9.73	0.46	40	d	0.003269
			215585_at	0	3.34	0.35	7	3.324	0.2	40	d	0.917274
DTX1	3	deltex homolog 1 (Drosophila)	227336_at	3	5.458	0.31	7	5.064	0.57	40	d	0.086011
NRAS	3	neuroblastoma RAS viral (v-ras) oncogene homolog	202647_s_at	1	5.274	0.89	7	6.947	1.52	40	u	0.008107
MGAT5B	3	mannosyl (alpha-1,6-)-glycoprotein beta-1,6-N-acetyl-glucosaminyltransferase, isozyme B	238445_x_at	3	5.483	0.47	7	5.214	0.46	40	d	0.171122
			1563137_at	0	1.85	0.01	7	1.895	0.07	40	u	0.000717
KCTD5	3	potassium channel tetramerisation domain containing 5	222645_s_at	3	6.871	0.36	7	7.272	0.94	40	u	0.28372
			218474_s_at	3	7.161	0.46	7	8.092	0.63	40	u	0.000652
			229837_s_at	2	4.019	0.49	7	4.008	0.45	40	d	0.950687
WDR40B	3	WD repeat domain 40B	238205_at	3	2.688	0.34	7	2.513	0.52	40	d	0.404134
MTHFD2	3	methylenetetrahydrofolate dehydrogenase (NADP+ dependent) 2, methenyltetrahydrofolate cyclohydrolase	201761_at	3	9.108	0.25	7	10.97	0.69	40	u	0
CBLB	3	Cas-Br-M (murine) ecotropic retroviral transforming sequence b	209682_at	3	8.276	0.29	7	8.093	0.7	40	d	0.505988
			208348_s_at	0	2.318	0.03	7	2.429	0.18	40	u	0.001247
			227900_at	0	6.059	0.22	7	5.292	0.9	40	d	0.000063
TLL2	3	tolloid-like 2	215008_at	3	2.696	0.12	7	2.857	0.3	40	u	0.181787
			208027_s_at	1	2.514	0.07	7	2.618	0.13	40	u	0.053388
			215843_s_at	0	2.259	0.03	7	2.304	0.05	40	u	0.03237
ZYX	3	zyxin	200808_s_at	3	9.761	0.43	7	8.716	0.74	40	d	0.000944
			215706_x_at	3	9.237	0.49	7	7.991	0.86	40	d	0.000664
DRD2	3	dopamine receptor D2	216938_x_at	3	3.818	0.5	7	3.284	0.65	40	d	0.047578
			206590_x_at	3	1.985	0.09	7	2.14	0.46	40	u	0.068084
			216924_s_at	3	2.559	0.38	7	2.509	0.35	40	d	0.740491
			211624_s_at	0	2.475	0.07	7	2.538	0.19	40	u	0.146589
SDC4	3	syndecan 4	202071_at	3	10.85	0.49	7	9.926	0.82	40	d	0.006636
BZW1	3	basic leucine zipper and W2 domains 1	200777_s_at	3	10.79	0.27	7	10.86	0.61	40	u	0.789041
			200776_s_at	3	10.39	0.51	7	10.74	0.91	40	u	0.340471
C1orf34	3	chromosome 1 open reading frame 34	210652_s_at	3	8.641	0.64	7	7.392	1.81	40	d	0.003779
			215562_at	0	2.089	0.12	7	2.15	0.13	40	u	0.264008
			217101_at	0	3.453	0.15	7	3.448	0.24	40	d	0.952761
PPP2CB	3	protein phosphatase 2 (formerly 2A), catalytic subunit, beta isoform	201375_s_at	3	11.45	0.26	7	10.73	0.64	40	d	0.005909
			201374_x_at	3	3.248	0.49	7	2.654	0.39	40	d	0.001174

RAB6B	3	RAB6B, member RAS oncogene family	210127_at	3	2.331	0.03	7	2.931	0.99	40	u	0.000535
			225259_at	0	3.28	0.52	7	3.727	1.16	40	u	0.329964
			221792_at	0	5.035	0.46	7	4.791	0.82	40	d	0.455833
FAM62A	3	family with sequence similarity 62 (C2 domain containing), member A	208858_s_at	3	9.214	0.2	7	8.513	0.56	40	d	0.000008
			244234_at	0	3.252	0.46	7	3.326	0.49	40	u	0.717811
CCDC100	3	coiled-coil domain containing 100	226449_at	3	7.535	0.23	7	6.91	0.78	40	d	0.000372
			1554606_at	0	5.233	0.56	7	5.204	0.89	40	d	0.93535
PCYOX1	3	prenylcysteine oxidase 1	203803_at	3	7.896	0.41	7	7.075	0.94	40	d	0.031543
SPRED1	3	sprouty-related, EVH1 domain containing 1	226837_at	3	7.406	0.43	7	7.326	0.8	40	d	0.804371
			235074_at	0	7.68	0.39	7	7.126	0.65	40	d	0.037316
			244439_at	0	4.102	0.96	7	4.489	1.36	40	u	0.481776
TBKBP1	3	TBK1 binding protein 1	205424_at	3	6.595	0.25	7	6.117	0.55	40	d	0.032967
CABLES2	3	Cdk5 and Abl enzyme substrate 2	226004_at	3	5.78	0.34	7	6.689	0.88	40	u	0.011694
ABCG4	3	ATP-binding cassette, sub-family G (WHITE), member 4	207593_at	3	2.388	0.03	7	2.455	0.08	40	u	0.000829
MTMR10	3	myotubularin related protein 10	225810_at	3	7.805	0.18	7	7.004	0.77	40	d	0.000002
			220286_at	0	1.986	0.09	7	2.086	0.14	40	u	0.077988
MAT2B	3	methionine adenosyltransferase II, beta	217993_s_at	3	10.3	0.18	7	9.679	0.71	40	d	0.000046
			229284_at	3	2.087	0.07	7	2.338	0.47	40	u	0.003499
CSNK1D	3	casein kinase 1, delta	207945_s_at	3	8.326	0.4	7	8.381	0.8	40	u	0.86244
			208774_at	0	9.498	0.24	7	8.782	0.82	40	d	0.000117
ANUBL1	3	AN1, ubiquitin-like, homolog (Xenopus laevis)	223624_at	3	3.641	0.49	7	3.587	0.51	40	d	0.801645
			244206_at	0	2.555	0.13	7	2.898	0.42	40	u	0.000348
PLEKHH2	3	pleckstrin homology domain containing, family H (with MyTH4 domain) member 2	227148_at	3	7.042	0.26	7	4.774	0.92	40	d	0
			243650_at	0	2.834	0.24	7	2.97	0.49	40	u	0.481695
			239568_at	0	3.365	0.74	7	2.262	0.67	40	d	0.000347
TES	3	testis derived transcript (3 LIM domains)	202720_at	3	10.77	0.38	7	10.34	0.87	40	d	0.209269
			244870_at	0	2.34	0.08	7	2.429	0.12	40	u	0.070974
			202719_s_at	0	8.754	0.36	7	8.624	0.91	40	d	0.718504
SLC6A14	3	solute carrier family 6 (amino acid transporter), member 14	219795_at	3	7.263	0.84	7	4.902	2.86	40	d	0.000257
KIAA1853	3	KIAA1853	230010_at	3	3.235	0.19	7	3.473	0.54	40	u	0.052685
			236151_at	3	2.256	0.32	7	2.519	0.49	40	u	0.186296
			237828_at	3	2.155	0.01	7	2.231	0.18	40	u	0.011347
FREM2	3	FRAS1 related extracellular matrix protein 2	230964_at	3	2.674	0.21	7	3.185	1.24	40	u	0.021987
TBX15	3	T-box 15	230438_at	3	6.452	0.24	7	5.584	0.52	40	d	0.000098
MAZ	3	MYC-associated zinc finger protein (purine-binding transcription factor)	212064_x_at	3	9.346	0.37	7	10.1	0.63	40	u	0.00386
			228798_x_at	3	6.635	0.23	7	6.44	0.38	40	d	0.210445
			229807_s_at	0	2.477	0.34	7	2.467	0.41	40	d	0.954011
			207824_s_at	0	6.596	0.38	7	7.441	1.62	40	u	0.007931
GALNT4	3	UDP-N-acetyl-alpha-D-galactosamine:polypeptide N-	231832_at	3	5.868	0.34	7	5.737	0.99	40	d	0.542505
			220442_at	0	2.7	0.35	7	3.093	0.86	40	u	0.251556
ANGPT1	3	angiopoietin 1	205609_at	3	4.305	0.54	7	4.05	0.95	40	d	0.501863
			205608_s_at	1	3.712	0.74	7	3.527	1.52	40	d	0.75951
			1552939_at	0	2.694	0.04	7	2.911	0.33	40	u	0.000255
LMTK2	3	lemur tyrosine kinase 2	235307_at	3	2.361	0.09	7	2.406	0.06	40	u	0.125205
			206223_at	0	2.064	0.08	7	2.145	0.18	40	u	0.269526
CASKIN1	3	CASK interacting protein 1	1552689_at	4	2.85	0.4	7	3.105	0.57	40	u	0.272317
			1569737_a_at	2	2.334	0.34	7	2.433	0.41	40	u	0.555515
			239189_at	0	2.132	0.15	7	2.119	0.08	40	d	0.840053
ING2	3	inhibitor of growth family, member 2	205981_s_at	3	6.995	0.33	7	7.332	0.74	40	u	0.253237
			213544_at	3	2.031	0.06	7	2.312	0.61	40	u	0.007329
KCNJ14	3	potassium inwardly-rectifying channel, subfamily J, member 14	220776_at	3	2.6	0.25	7	2.87	0.26	40	u	0.017168
C1orf149	3	chromosome 1 open reading frame 149	228517_at	3	6.011	0.22	7	6.012	0.7	40	u	0.994743
			218165_at	2	9.828	0.46	7	9.663	0.57	40	d	0.482866
SMCR7L	3	Smith-Magenis syndrome chromosome region, candidate 7-like	204593_s_at	3	6.739	0.33	7	6.969	0.74	40	u	0.435387
			204594_s_at	3	6.838	0.42	7	6.801	0.74	40	d	0.902171
			221516_s_at	3	8.006	0.37	7	8.001	0.69	40	d	0.986907
			224319_s_at	2	7.088	0.27	7	7.069	0.62	40	d	0.939231
COTL1	3	coactosin-like 1 (Dictyostelium)	224583_at	3	7.238	0.49	7	8.766	1.34	40	u	0.005386
			221059_s_at	0	7.722	0.28	7	8.991	1.16	40	u	0.000001
DLG2	3	discs, large homolog 2, chapsyn-110	228973_at	20	3.618	0.42	7	3.032	0.6	40	d	0.019662

DLG2	3	(Drosophila)	206253_at	3	2.906	0.09	7	2.991	0.19	40	u	0.267927
YPEL4	3	yippee-like 4 (Drosophila)	235916_at	3	2.678	0.22	7	2.609	0.14	40	d	0.292097
ZDHHC8	3	zinc finger, DHHC-type containing 8	225744_at	3	7.268	0.29	7	6.809	0.71	40	d	0.106061
SERTAD3	3	SERTA domain containing 3	219382_at	3	8.483	0.42	7	7.176	1.09	40	d	0.003734
PTPRD	3	protein tyrosine phosphatase, receptor type, D	205712_at	3	2.413	0.09	7	2.72	0.51	40	u	0.001355
			214043_at	0	3.178	0.34	7	3.287	0.8	40	u	0.729698
			242493_at	0	2.629	0.23	7	2.669	0.15	40	u	0.573344
			213362_at	0	2.697	0.18	7	2.976	0.4	40	u	0.081242
GRASP	3	GRP1 (general receptor for phosphoinositides 1)-associated scaffold protein	228263_at	3	6.973	0.35	7	6.061	0.67	40	d	0.001218
TADA1L	3	transcriptional adaptor 1 (HFI1 homolog, yeast)-like	225455_at	3	6.784	0.5	7	7.636	0.66	40	u	0.002742
			1559289_at	0	2.544	0.06	7	2.752	0.34	40	u	0.001007
RNF26	3	ring finger protein 26	224947_at	3	7.763	0.29	7	7.493	0.58	40	d	0.246323
			224338_s_at	0	5.956	0.26	7	5.832	0.5	40	d	0.534746
WIPF2	3	WAS/WASL interacting protein family, member 2	212051_at	3	8.023	0.37	7	7.708	0.89	40	d	0.36973
			212050_at	3	8.462	0.22	7	7.956	1	40	d	0.008891
			212049_at	3	8.168	0.36	7	7.845	0.62	40	d	0.195832
			231251_at	0	3.81	0.61	7	3.672	0.94	40	d	0.715002
ADRA2B	3	adrenergic, alpha-2B-, receptor	208544_at	0	3.264	0.53	7	3.061	0.48	40	d	0.323886
SUV39H1	3	suppressor of variegation 3-9 homolog 1 (Drosophila)	218619_s_at	3	5.109	0.56	7	5.747	0.9	40	u	0.081761
SLC35D3	3	solute carrier family 35, member D3	231341_at	3	2.352	0.14	7	2.523	0.39	40	u	0.263015
MXI1	3	MAX interactor 1	202364_at	3	10.36	0.3	7	8.927	0.86	40	d	0
STX12	3	syntaxin 12	212112_s_at	3	9.542	0.35	7	8.848	0.56	40	d	0.003356
			212111_at	3	9.15	0.26	7	8.323	0.54	40	d	0.000361
NIN	3	ninein (GSK3B interacting protein)	225921_at	3	8.378	0.25	7	7.976	0.77	40	d	0.017336
			234299_s_at	2	3.283	0.59	7	4.041	0.9	40	u	0.04087
			224303_x_at	0	3.755	0.45	7	3.691	0.53	40	d	0.769955
			219285_s_at	0	4.635	0.43	7	4.525	0.43	40	d	0.54057
			224304_x_at	0	4.759	0.47	7	4.726	0.86	40	d	0.923263
			223981_at	0	2.457	0.21	7	2.377	0.13	40	d	0.201004
OSR1	3	odd-skipped related 1 (Drosophila)	228399_at	3	7.641	0.48	7	4.348	1.43	40	d	0
ENSA	3	endosulfine alpha	221486_at	3	9.092	0.3	7	8.95	0.78	40	d	0.641826
			202596_at	0	11.5	0.25	7	11.74	0.77	40	u	0.164467
			228851_s_at	0	7.88	0.45	7	8.518	1.34	40	u	0.033478
			221487_s_at	0	7.216	0.44	7	7.578	1.19	40	u	0.440127
			228852_at	0	6.072	0.44	7	6.966	1	40	u	0.028031
RHOB	3	ras homolog gene family, member B	212099_at	3	11.94	0.49	7	10.8	1.34	40	d	0.000748
			1553965_x_at	1	2.724	0.07	7	2.797	0.14	40	u	0.193204
			1553963_at	1	2.571	0.06	7	2.642	0.09	40	u	0.044227
			1553962_s_at	1	8.532	0.72	7	7.411	1.92	40	d	0.141695
			226417_at	0	2.108	0.02	7	2.157	0.04	40	u	0.00602
DOCK10	3	dedicator of cytokinesis 10	219279_at	3	6.355	0.61	7	5.703	1.34	40	d	0.221232
			215151_at	0	3.717	0.79	7	3.735	0.55	40	u	0.942236
ZBTB9	3	zinc finger and BTB domain containing 9	226163_at	13	6.161	0.24	7	6.302	0.58	40	u	0.540047
COL2A1	3	collagen, type II, alpha 1 (primary osteoarthritis, spondyloepiphyseal dysplasia,	213492_at	3	2.814	0.35	7	4.443	3.07	40	u	0.002705
			217404_s_at	0	2.497	0.19	7	3.925	2.54	40	u	0.001329
ST8SIA3	3	ST8 alpha-N-acetyl-neuraminide alpha-2,8-sialyltransferase 3	208065_at	3	3.054	0.17	7	3.295	0.41	40	u	0.140007
			208064_s_at	3	2.305	0.08	7	2.455	0.25	40	u	0.006647
			230262_at	0	2.35	0.05	7	2.433	0.14	40	u	0.011053
BFAR	3	bifunctional apoptosis regulator	218056_at	1	7.605	0.28	7	8.527	0.55	40	u	0.000117
PPIF	3	peptidylprolyl isomerase F (cyclophilin F)	201489_at	3	9.812	0.95	7	9.859	0.97	40	u	0.908865
			201490_s_at	0	8.416	1.08	7	8.348	1.66	40	d	0.919081
MICALCL	3	MICAL C-terminal like	243611_at	0	2.709	0.51	7	2.684	0.58	40	d	0.914832
BCL2L1	3	BCL2-like 1	212312_at	3	7.557	0.43	7	7.44	0.82	40	d	0.720191
			215037_s_at	0	7.197	0.44	7	6.998	1.12	40	d	0.650979
			206665_s_at	0	6.577	0.4	7	6.531	1.22	40	d	0.858947
			231228_at	0	5.295	0.32	7	5.44	0.67	40	u	0.583856
USP34	3	ubiquitin specific peptidase 34	212066_s_at	3	10.05	0.19	7	9.681	0.57	40	d	0.00531
			212065_s_at	3	6.623	0.55	7	6.893	1.04	40	u	0.513753
			212980_at	0	8.127	0.62	7	7.149	1.18	40	d	0.041531
			215013_s_at	0	4.776	0.34	7	4.967	0.51	40	u	0.35538
			207365_x_at	0	9.43	0.32	7	9.217	0.63	40	d	0.39476

			233595_at	0	8.534	0.45	7	8.56	0.91	40	u	0.94334
			242647_at	0	3.372	0.21	7	4.526	1.13	40	u	0.000001
APLP2	3	amyloid beta (A4) precursor-like protein 2	228520_s_at	3	8.164	0.46	7	7.524	0.88	40	d	0.073669
			208248_x_at	3	12.42	0.34	7	10.72	0.88	40	d	0.00001
			208701_at	3	2.53	0.3	7	3.243	1.33	40	u	0.006051
			208704_x_at	3	12.45	0.36	7	10.64	0.88	40	d	0.000004
			208703_s_at	3	11.23	0.41	7	9.277	1.16	40	d	0
			208702_x_at	3	10.9	0.6	7	9.484	1.07	40	d	0.001651
			211404_s_at	0	9.762	0.66	7	8.545	1.06	40	d	0.006051
			214875_x_at	0	9.177	0.62	7	7.699	1.1	40	d	0.001457
CELSR1	3	cadherin, EGF LAG seven-pass G-type receptor 1 (flamingo homolog, Drosophila)	41660_at	3	7.582	0.88	7	6.868	1.36	40	d	0.196807
			204539_s_at	3	2.378	0.03	7	2.514	0.21	40	u	0.000472
			217262_s_at	0	2.231	0.05	7	2.403	0.26	40	u	0.000515
THBS2	3	thrombospondin 2	203083_at	3	9.739	0.6	7	9.497	1.29	40	d	0.63514
TGOLN2	3	trans-golgi network protein 2	212040_at	3	8.42	0.22	7	7.583	0.82	40	d	0.000007
			203833_s_at	3	7.518	0.26	7	8.24	0.67	40	u	0.008314
			212043_at	3	10.88	0.2	7	10.26	0.67	40	d	0.000064
			203834_s_at	3	6.65	0.48	7	6.606	0.84	40	d	0.896163
			1554608_at	0	6.162	0.28	7	6.206	0.83	40	u	0.801328
PIGS	3	phosphatidylinositol glycan anchor biosynthesis, class S	223148_at	0	8.901	0.22	7	8.542	0.65	40	d	0.013992
HSP90B1	3	heat shock protein 90kDa beta (Grp94), member 1	200599_s_at	3	12.2	0.18	7	12.33	0.46	40	u	0.470285
			200598_s_at	3	10.3	0.29	7	11.23	0.6	40	u	0.00027
			216450_x_at	0	5.526	0.81	7	7.379	0.94	40	u	0.000019
			216449_x_at	0	9.002	0.24	7	9.665	0.52	40	u	0.002291
HOXB6	3	homeobox B6	205366_s_at	3	6.198	1.03	7	5.756	1.41	40	d	0.441959
			205365_at	3	2.567	0.17	7	2.682	0.29	40	u	0.321377
SORD	3	sorbitol dehydrogenase	201563_at	3	9.212	0.74	7	9.351	1.27	40	u	0.783401
			201562_s_at	3	7.483	0.83	7	7.888	1.03	40	u	0.340591
RCE1	3	RCE1 homolog, prenyl protein peptidase (S. cerevisiae)	205332_at	3	5.084	0.19	7	4.946	0.32	40	d	0.287874
			205333_s_at	3	7.285	0.25	7	7.381	0.5	40	u	0.626732
SEMA3D	3	sema domain, immunoglobulin domain (Ig), short basic domain, secreted, (semaphorin) 3D	215324_at	0	2.56	0.11	7	2.573	0.11	40	u	0.779124
AP3D1	3	adaptor-related protein complex 3, delta 1 subunit	206592_s_at	3	9.777	0.31	7	9.414	0.62	40	d	0.141752
			208710_s_at	3	6.767	0.35	7	7.318	0.78	40	u	0.079984
			210974_s_at	0	3.171	0.27	7	3.793	1.08	40	u	0.004442
HIF1AN	3	hypoxia-inducible factor 1, alpha subunit inhibitor	218525_s_at	3	6.516	0.26	7	6.245	0.7	40	d	0.327978
			59999_at	3	6.746	0.25	7	6.583	0.7	40	d	0.291651
RAD17	3	RAD17 homolog (S. pombe)	207405_s_at	3	7.957	0.29	7	7.937	0.7	40	d	0.940548
			210826_x_at	0	8.325	0.36	7	7.874	0.6	40	d	0.064676
			211228_s_at	0	8.268	0.32	7	7.786	0.74	40	d	0.103512
RNF214	3	ring finger protein 214	227114_at	0	5.748	0.17	7	5.616	0.55	40	d	0.247512
ACIN1	3	apoptotic chromatin condensation inducer 1	201715_s_at	3	8.598	0.23	7	8.207	0.4	40	d	0.017219
SMAP1L	3	stromal membrane-associated protein 1-like	225282_at	3	6.802	0.3	7	6.578	0.73	40	d	0.435988
TCF3	3	transcription factor 3 (E2A immunoglobulin enhancer binding factors E12/E47)	213809_x_at	3	1.462	0.28	7	1.425	0.15	40	d	0.76304
			210776_x_at	3	8.304	0.36	7	8.757	0.78	40	u	0.147962
			213811_x_at	3	8.277	0.27	7	8.616	0.84	40	u	0.060659
			209151_x_at	3	3.626	0.55	7	4.384	1.4	40	u	0.173702
			209152_s_at	3	6.517	0.39	7	6.915	0.85	40	u	0.239324
			213731_s_at	3	2.339	0.21	7	2.292	0.13	40	d	0.44915
			228052_x_at	3	1.972	0.17	7	2.015	0.15	40	u	0.510125
			213732_at	3	2.506	0.33	7	2.44	0.3	40	d	0.604765
			213730_x_at	3	8.119	0.38	7	8.721	0.75	40	u	0.048977
			209153_s_at	3	7.875	0.39	7	8.077	1.01	40	u	0.613117
			215260_s_at	3	6.012	0.35	7	6.282	0.97	40	u	0.212954
			216647_at	0	6.445	0.31	7	6.132	0.54	40	d	0.151917
FAM125B	3	family with sequence similarity 125, member B	221687_s_at	3	5.089	0.45	7	4.878	0.58	40	d	0.376895
			221828_s_at	3	2.644	0.38	7	2.422	0.44	40	d	0.224327
			52975_at	3	4.919	0.31	7	4.616	0.45	40	d	0.100607
			1554065_at	0	2.478	0.14	7	2.56	0.17	40	u	0.239982
TXNDC11	3	thioredoxin domain containing 11	223325_at	3	8.109	0.21	7	7.961	0.52	40	d	0.473086

B4GALT3	3	UDP-Gal:betaGlcNAc beta 1,4-galactosyltransferase, polypeptide 3	210243_s_at	3	7.953	0.3	7	8.627	0.66	40	u	0.012714
			238312_s_at	0	2.968	0.23	7	3.086	0.35	40	u	0.407887
PRPF38A	3	PRP38 pre-mRNA processing factor 38 (yeast) domain containing A	223230_at	3	8.036	0.27	7	7.704	0.54	40	d	0.127442
			1553709_a_at	1	8.114	0.26	7	8.258	0.65	40	u	0.577707
EDARADD	3	EDAR-associated death domain	1553430_a_at	3	4.359	0.87	7	4.194	1.72	40	d	0.808863
ATP6V1C2	3	ATPase, H+ transporting, lysosomal 42kDa, V1 subunit C2	1552532_a_at	6	2.919	0.32	7	3.446	1	40	u	0.016961
			1553989_a_at	3	2.334	0.41	7	3.592	1.75	40	u	0.000405
LRRFIP2	3	leucine rich repeat (in FLII) interacting protein 2	218364_at	3	8.064	0.32	7	8.148	0.83	40	u	0.796801
			220610_s_at	0	6.558	0.43	7	6.981	0.79	40	u	0.182586
			232673_at	0	2.363	0.03	7	2.442	0.09	40	u	0.000396
			232704_s_at	0	5.98	0.4	7	5.51	0.6	40	d	0.058141
			232705_at	0	4.067	0.87	7	3.973	0.79	40	d	0.783594
AKAP13	3	A kinase (PRKA) anchor protein 13	224884_at	3	8.397	0.3	7	7.327	0.85	40	d	0.000005
			227039_at	1	6.993	0.23	7	7.252	0.67	40	u	0.077566
			222023_at	0	5.067	0.41	7	5.202	0.59	40	u	0.569916
			222024_s_at	0	9.014	0.22	7	8.786	0.68	40	d	0.117399
			232188_at	0	3.612	0.26	7	3.758	0.37	40	u	0.326677
			221718_s_at	0	9.53	0.18	7	8.72	0.69	40	d	0.000001
			213516_at	0	2.57	0.06	7	2.645	0.11	40	u	0.10268
			209534_x_at	0	8.448	0.27	7	8.169	0.59	40	d	0.238783
			208325_s_at	0	6.725	0.31	7	7.53	0.83	40	u	0.017082
ANGPTL2	3	angiopoietin-like 2	213001_at	4	8.811	0.5	7	6.532	1.08	40	d	0.000003
			213004_at	4	7.557	0.72	7	6.012	0.67	40	d	0.000002
			219514_at	0	5.984	0.76	7	4.331	0.89	40	d	0.000045
DENND4C	3	DENN/MADD domain containing 4C	226867_at	3	9.542	0.16	7	8.724	0.69	40	d	0
			205684_s_at	0	8.842	0.15	7	8.151	0.81	40	d	0.00002
			234968_at	0	2.749	0.08	7	2.999	0.41	40	u	0.001583
NEDD9	3	neural precursor cell expressed, developmentally down-regulated 9	202149_at	3	9.36	0.61	7	7.711	1.13	40	d	0.000598
			1569020_at	0	4.285	0.81	7	3.979	0.63	40	d	0.273337
			202150_s_at	0	7.908	0.62	7	6.746	1	40	d	0.005508
MORC4	3	MORC family CW-type zinc finger 4	219038_at	3	7.102	0.38	7	6.906	1.34	40	d	0.462562
CDC42BPB	3	CDC42 binding protein kinase beta (DMPK-like)	217849_s_at	3	8.817	0.39	7	8.115	0.79	40	d	0.029421
			1557963_at	0	2.662	0.07	7	2.807	0.25	40	u	0.004786
RIMS2	3	regulating synaptic membrane exocytosis 2	206137_at	0	2.273	0.45	7	2.951	1.43	40	u	0.027792
			215478_at	0	2.805	0.19	7	3.3	0.66	40	u	0.000591
TMEM145	3	transmembrane protein 145	1553479_at	3	2.104	0.09	7	2.532	1.23	40	u	0.038673
KCTD16	3	potassium channel tetramerisation domain containing 16	233234_at	3	2.365	0.03	7	2.551	0.33	40	u	0.001225
SPCS2	3	signal peptidase complex subunit 2 homolog (S. cerevisiae)	201240_s_at	3	11.36	0.12	7	11.34	0.5	40	d	0.843786
			201239_s_at	3	10.28	0.2	7	10.13	0.84	40	d	0.347986
ITPKC	3	inositol 1,4,5-trisphosphate 3-kinase C	213076_at	3	7.247	0.81	7	6.722	0.73	40	d	0.099542
LRP4	3	low density lipoprotein receptor-related protein 4	212850_s_at	3	4.054	0.38	7	3.125	1.11	40	d	0.000573
RAD21	3	RAD21 homolog (S. pombe)	200607_s_at	3	8.941	0.52	7	10.9	1.1	40	u	0.000044
			200608_s_at	3	10.32	0.29	7	11.63	0.71	40	u	0.000025
POMT2	3	protein-O-mannosyltransferase 2	225560_at	3	6.912	0.26	7	6.164	0.51	40	d	0.00056
			220632_s_at	0	5.12	0.29	7	5.008	0.52	40	d	0.59055
			239535_at	0	2.344	0.14	7	2.545	0.37	40	u	0.172075
BCL3	3	B-cell CLL/lymphoma 3	204907_s_at	3	4.08	0.68	7	4.805	1.42	40	u	0.201837
			204908_s_at	3	8.625	0.41	7	8.447	1.06	40	d	0.669133
SMARCA2	3	SWI/SNF related, matrix associated, actin dependent regulator of chromatin, subfamily a, member 2	212258_s_at	3	3.733	0.37	7	3.844	0.43	40	u	0.536045
			217707_x_at	3	8.993	0.19	7	7.995	0.6	40	d	0
			212257_s_at	3	7.651	0.41	7	6.844	1.48	40	d	0.008828
			206542_s_at	1	9.259	0.23	7	8.066	0.72	40	d	0
			206543_at	1	3.37	0.2	7	3.71	0.41	40	u	0.04168
			206544_x_at	0	9.146	0.21	7	7.575	1.24	40	d	0
			228926_s_at	0	2.001	0.19	7	1.966	0.06	40	d	0.657994
ADAMTS10	3	ADAM metallopeptidase with thrombospondin type 1 motif, 10	230341_x_at	3	7.517	0.18	7	7.559	0.47	40	u	0.820583
			232133_at	0	4.452	0.54	7	4.259	0.72	40	d	0.510469
F11R	3	F11 receptor	224097_s_at	3	7.348	0.56	7	8.708	0.89	40	u	0.000385
			221664_s_at	3	9.308	0.5	7	10.25	0.9	40	u	0.011425
			223000_s_at	3	11.18	0.31	7	11.84	0.63	40	u	0.010652
			226482_s_at	0	10.89	0.49	7	10.52	1.04	40	d	0.369574
SPAG7	3	sperm associated antigen 7	200053_at	3	9.26	0.28	7	8.679	0.62	40	d	0.02131

ZZZ3	3	zinc finger, ZZ-type containing 3	212893_at	3	8.355	0.39	7	8.231	0.56	40	d	0.589163
			229325_at	0	7.62	0.34	7	6.619	0.64	40	d	0.000284
HOXC6	3	homeobox C6	206858_s_at	3	9.226	0.4	7	8.516	1.47	40	d	0.018329
CTNNA2	3	catenin (cadherin-associated protein), alpha 2	205373_at	3	2.845	0.6	7	2.598	0.18	40	d	0.356581
ASCL1	3	achaete-scute complex homolog 1 (Drosophila)	209985_s_at	3	2.555	0.27	7	2.836	0.78	40	u	0.105458
			209988_s_at	3	2.303	0.05	7	2.969	2.11	40	u	0.055908
			213768_s_at	2	2.524	0.07	7	2.957	1.34	40	u	0.052646
			209987_s_at	0	1.864	0.03	7	2.455	1.99	40	u	0.071682
FAM131B	3	family with sequence similarity 131, member B	205368_at	3	3.145	0.22	7	3.384	0.63	40	u	0.089199
ZXDB	3	zinc finger, X-linked, duplicated B	228005_at	3	6.334	0.37	7	6.407	0.71	40	u	0.798723
			216013_at	0	3.44	0.32	7	3.46	0.43	40	u	0.909673
MGEA5	3	meningioma expressed antigen 5 (hyaluronidase)	200898_s_at	3	9.306	0.24	7	8.981	0.76	40	d	0.048342
			200899_s_at	3	10.17	0.24	7	9.291	0.73	40	d	0.000004
			214972_at	0	4.689	0.56	7	4.989	0.8	40	u	0.356957
			235868_at	0	2.577	0.36	7	2.948	0.88	40	u	0.287755
			223494_at	0	9.234	0.46	7	8.363	0.92	40	d	0.020392
CEBPB	3	CCAAT/enhancer binding protein (C/EBP), beta	212501_at	3	11.94	0.26	7	12.01	0.88	40	u	0.716184
CAST	3	calpastatin	207467_x_at	3	9.391	0.45	7	9.526	0.74	40	u	0.648807
			212586_at	3	10.92	0.29	7	9.864	0.78	40	d	0.001157
			208908_s_at	1	9.185	0.41	7	9.241	0.76	40	u	0.852508
			1564010_at	0	2.895	0.44	7	2.757	0.22	40	d	0.47944
STRN	3	striatin, calmodulin binding protein	205520_at	1	2.186	0.09	7	3.045	1.07	40	u	0.000015
			1569815_x_at	0	3.076	0.2	7	3.459	0.52	40	u	0.067979
			1569813_at	0	2.954	0.15	7	3.254	0.4	40	u	0.063991
GAB1	3	GRB2-associated binding protein 1	225998_at	3	6.991	0.43	7	6.564	1.06	40	d	0.308717
			207112_s_at	1	5.199	0.43	7	5.144	1.07	40	d	0.896399
			1557030_at	0	3.281	0.13	7	3.501	0.27	40	u	0.044129
			1560382_at	0	3.427	0.63	7	3.545	0.75	40	u	0.702591
TGFBI	3	transforming growth factor, beta-induced, 68kDa	201506_at	3	9.953	0.54	7	9.903	0.81	40	d	0.878248
DGCR2	3	DiGeorge syndrome critical region gene 2	214198_s_at	3	8.354	0.33	7	7.843	0.77	40	d	0.099663
			202099_s_at	3	6.195	0.34	7	5.506	1.16	40	d	0.005703
			227028_s_at	1	3.746	0.46	7	3.569	0.33	40	d	0.235185
TAOK1	3	TAO kinase 1	227454_at	3	5.98	0.56	7	7.929	0.98	40	u	0.000009
			216310_at	0	6.289	0.35	7	6.237	0.6	40	d	0.826492
CASK	3	calcium/calmodulin-dependent serine protein kinase (MAGUK family)	207620_s_at	3	6.556	0.37	7	7.008	1.28	40	u	0.086085
			211208_s_at	3	8.335	0.23	7	8.976	0.62	40	u	0.010874
			238698_at	0	2.672	0.17	7	2.996	0.44	40	u	0.068277
			238699_s_at	0	4.968	0.48	7	5.733	1.34	40	u	0.015126
LRRC14	3	leucine rich repeat containing 14	203495_at	3	3.704	0.4	7	3.813	0.62	40	u	0.663887
			32062_at	3	6.899	0.34	7	6.994	0.37	40	u	0.537735
GGA2	3	golgi associated, gamma adaptin ear containing, ARF binding protein 2	210658_s_at	3	8.02	0.26	7	7.822	1.06	40	d	0.329023
			208913_at	1	7.402	0.17	7	7.398	0.73	40	d	0.976425
			208914_at	1	7.83	0.27	7	6.845	1.06	40	d	0.000021
			208915_s_at	0	8.063	0.23	7	8.039	0.9	40	d	0.88696
			214233_at	0	4.144	0.57	7	4.319	0.6	40	u	0.486206
			213772_s_at	0	7.118	0.18	7	6.937	0.78	40	d	0.218981
			214190_x_at	0	4.911	0.54	7	5.185	1.13	40	u	0.543384
			214189_s_at	0	2.608	0.04	7	2.687	0.11	40	u	0.002344
FMN2	3	formin 2	1555471_a_at	3	2.973	0.05	7	3.162	0.21	40	u	0.000024
			223618_at	3	2.575	0.17	7	2.673	0.39	40	u	0.525294
			230946_at	0	6.831	0.17	7	6.712	0.23	40	d	0.202914
			1559244_at	0	2.346	0.02	7	2.434	0.21	40	u	0.014685
NDRG3	3	NDRG family member 3	221082_s_at	3	6.256	0.42	7	6.367	0.96	40	u	0.770061
			217286_s_at	3	9.114	0.22	7	8.806	0.56	40	d	0.170975
			224368_s_at	3	7.436	0.51	7	7.16	1.19	40	d	0.557923
DACT1	3	dapper, antagonist of beta-catenin, homolog 1 (Xenopus laevis)	219179_at	3	3.824	0.91	7	3.838	1.36	40	u	0.980135
BET1	3	BET1 homolog (S. cerevisiae)	202710_at	3	8.399	0.23	7	8.615	0.82	40	u	0.191925
USP3	3	ubiquitin specific peptidase 3	221654_s_at	3	8.36	0.23	7	8.298	0.85	40	d	0.711296
			226652_at	0	7.353	0.49	7	6.727	1.05	40	d	0.136047

C14orf101	3	chromosome 14 open reading frame 101	225675_at	3	7.62	0.2	7	7.064	0.5	40	d	0.006645
			219757_s_at	3	7.498	0.23	7	7.415	0.6	40	d	0.722807
PLSCR3	3	phospholipid scramblase 3	218828_at	3	8.427	0.21	7	8.105	0.45	40	d	0.077841
			56197_at	3	8.683	0.24	7	8.239	0.49	40	d	0.025167
XPO5	3	exportin 5	223055_s_at	3	5.844	0.33	7	6.786	1.06	40	u	0.000177
			223056_s_at	3	7.062	0.22	7	7.861	0.79	40	u	0.000013
			223057_s_at	2	6.557	0.27	7	7.165	1.18	40	u	0.008106
KIAA0802	3	KIAA0802	213358_at	3	4.886	0.58	7	5.306	1.88	40	u	0.282813
			240448_at	0	2.806	0.1	7	2.881	0.15	40	u	0.213914
CD44	2	CD44 molecule (Indian blood group)	212063_at	2	11.25	0.41	7	10.62	1.03	40	d	0.128628
			1565868_at	0	4.595	0.54	7	5.165	0.95	40	u	0.137518
			212014_x_at	0	9.102	0.56	7	9.256	1.36	40	u	0.774248
			234411_x_at	0	5.568	0.52	7	5.603	0.83	40	u	0.916318
			209835_x_at	0	9.118	0.6	7	9.318	1.23	40	u	0.682829
			217523_at	0	9.329	0.72	7	8.99	1.36	40	d	0.532714
			210916_s_at	0	7.611	0.59	7	7.697	1.46	40	u	0.881506
			1557905_s_at	0	7.881	0.67	7	8.195	1.43	40	u	0.580641
			234418_x_at	0	3.058	0.09	7	3.279	0.43	40	u	0.007183
			204490_s_at	0	8.544	0.6	7	8.769	1.26	40	u	0.650917
			204489_s_at	0	9.391	0.52	7	9.127	1.34	40	d	0.617889
			216056_at	0	2.843	0.65	7	3.769	1.27	40	u	0.072278
KLHL13	2	kelch-like 13 (Drosophila)	227875_at	2	8.737	0.5	7	4.762	1.72	40	d	0
TUT1	2	terminal uridylyl transferase 1, U6 snRNA-specific	218965_s_at	2	6.988	0.32	7	6.437	0.47	40	d	0.005706
EDN1	2	endothelin 1	222802_at	2	7.34	0.4	7	6.197	1.29	40	d	0.000161
			1564630_at	2	2.507	0.48	7	2.651	0.61	40	u	0.565305
			218995_s_at	1	7.538	0.59	7	6.331	1.06	40	d	0.006153
VPS4A	2	vacuolar protein sorting 4 homolog A (S. cerevisiae)	217913_at	0	8.466	0.15	7	8.377	0.57	40	d	0.421287
ABCB7	2	ATP-binding cassette, sub-family B (MDR/TAP), member 7	209620_s_at	2	7.181	0.33	7	6.698	0.59	40	d	0.043299
SLC25A3	2	solute carrier family 25 (mitochondrial carrier; phosphate carrier), member 3	200030_s_at	2	12.55	0.13	7	12.05	0.55	40	d	0.000027
LMAN1	2	lectin, mannose-binding, 1	203293_s_at	2	6.171	0.48	7	7.258	1.59	40	u	0.001993
			203294_s_at	1	5.742	0.42	7	6.932	1.51	40	u	0.000322
			224629_at	0	11.09	0.18	7	11.07	0.65	40	d	0.909245
SUMO1	2	SMT3 suppressor of mif two 3 homolog 1 (S. cerevisiae)	208762_at	2	5.196	0.34	7	5.848	0.76	40	u	0.033593
			211069_s_at	2	10.9	0.16	7	11.32	0.36	40	u	0.004171
			208761_s_at	1	9.843	0.26	7	10.31	0.64	40	u	0.069393
GARNL1	2	GTPase activating Rap/RanGAP domain-like 1	213049_at	2	8.807	0.29	7	8.07	0.75	40	d	0.016179
			214855_s_at	1	8.088	0.35	7	7.397	0.76	40	d	0.025705
			1566271_x_at	0	3.148	0.3	7	3.626	0.74	40	u	0.105548
			1566269_at	0	3.162	0.29	7	3.659	0.67	40	u	0.065153
			235524_at	0	2.251	0.02	7	2.319	0.15	40	u	0.011343
			234923_at	0	6.577	0.35	7	6.373	0.49	40	d	0.304345
			215162_at	0	5.049	0.41	7	4.802	0.38	40	d	0.128707
			1566272_at	0	2.688	0.06	7	2.968	0.35	40	u	0.00004
CCNI	2	cyclin I	208655_at	2	12.11	0.18	7	11.73	0.49	40	d	0.049189
			227299_at	0	5.369	0.29	7	5.437	0.91	40	u	0.719137
			208656_s_at	0	12.1	0.18	7	11.73	0.55	40	d	0.003944
RDH10	2	retinol dehydrogenase 10 (all-trans)	1552379_at	2	2.914	0.05	7	2.983	0.11	40	u	0.115671
			1552378_s_at	2	4.378	0.89	7	5.606	1.66	40	u	0.068486
			226021_at	2	7.562	0.85	7	8.579	1.79	40	u	0.156875
			227467_at	0	6.845	0.86	7	7.618	1.56	40	u	0.21764
UTY	2	ubiquitously transcribed tetratricopeptide repeat gene, Y-linked	211149_at	2	3.429	0.3	7	3.635	0.32	40	u	0.13079
			208067_x_at	0	3.628	0.59	7	4.405	0.62	40	u	0.004526
			210322_x_at	0	2.868	0.2	7	2.956	0.29	40	u	0.455311
CHORDC1	2	cysteine and histidine-rich domain (CHORD)-containing 1	218566_s_at	2	7.948	0.38	7	8.735	0.8	40	u	0.016157
			239436_at	0	2.272	0.12	7	2.425	0.22	40	u	0.090479
COL4A5	2	collagen, type IV, alpha 5 (Alport syndrome)	213110_s_at	2	9.525	0.62	7	7.028	1.93	40	d	0.000001
			234387_at	0	2.754	0.26	7	2.819	0.3	40	u	0.601665
PROSC	2	proline synthetase co-transcribed homolog (bacterial)	209384_at	2	8.85	0.2	7	8.779	1.11	40	d	0.719196
			214545_s_at	0	6.915	0.3	7	7.394	1.32	40	u	0.057379
			209385_s_at	0	9.211	0.15	7	9.463	1.06	40	u	0.168711

		(bacterial)	227315_x_at	0	2.005	0.03	7	2.069	0.21	40	u	0.078848
			216519_s_at	0	2.013	0.02	7	2.123	0.35	40	u	0.058747
TMEM132E	2	transmembrane protein 132E	243708_at	2	5.993	0.22	7	5.876	0.41	40	d	0.478354
SORL1	2	sortilin-related receptor, L(DLR class) A repeats-containing	203509_at	2	9.651	0.53	7	8.475	1.13	40	d	0.011476
			230707_at	0	6.306	0.47	7	5.662	1.13	40	d	0.151568
ARL6IP1	2	ADP-ribosylation factor-like 6 interacting protein 1	211935_at	2	11.07	0.17	7	11.93	0.54	40	u	0
CTAGE5	2	CTAGE family, member 5	204055_s_at	2	4.587	0.5	7	3.92	0.74	40	d	0.029966
			215930_s_at	2	6.868	0.41	7	6.9	0.34	40	u	0.828688
			235790_at	0	2.501	0.42	7	2.599	0.34	40	u	0.50595
MAPKAPK2	2	mitogen-activated protein kinase-activated protein kinase 2	201460_at	2	9.477	0.36	7	9.992	0.69	40	u	0.065131
			215050_x_at	1	6.428	0.34	7	7.211	0.83	40	u	0.020933
			201461_s_at	1	6.735	0.47	7	7.728	1.14	40	u	0.031252
DIP2A	2	DIP2 disco-interacting protein 2 homolog A (Drosophila)	227199_at	2	7.828	0.34	7	7.879	0.67	40	u	0.84696
			1552677_a_at	2	4.041	0.44	7	4.347	0.54	40	u	0.173678
			1556127_at	0	2.061	0.18	7	2.074	0.16	40	u	0.845568
			1561286_a_at	0	3.191	0.21	7	3.536	0.52	40	u	0.099862
			215529_x_at	0	8.469	0.28	7	8.497	0.39	40	u	0.861372
			1554967_at	0	2.907	0.3	7	3.101	0.3	40	u	0.127598
			1554969_x_at	0	3.052	0.12	7	3.255	0.31	40	u	0.101812
			1555301_a_at	0	3.262	0.04	7	3.665	0.63	40	u	0.000327
CDC25B	2	cell division cycle 25 homolog B (S. pombe)	201853_s_at	2	7.801	0.37	7	8.626	0.9	40	u	0.023972
USP52	2	ubiquitin specific peptidase 52	203117_s_at	2	8.14	0.33	7	6.58	1.27	40	d	0
GTPBP1	2	GTP binding protein 1	226359_at	2	5.347	0.52	7	5.339	0.71	40	d	0.978371
			205275_at	0	3.965	0.18	7	3.884	0.23	40	d	0.39411
			219357_at	0	6.786	0.37	7	6.573	0.66	40	d	0.423074
			205276_s_at	0	4.994	0.24	7	4.821	0.63	40	d	0.486531
			205274_at	0	2.181	0.01	7	2.212	0.14	40	u	0.171318
AP1S2	2	adaptor-related protein complex 1, sigma 2 subunit	230413_s_at	2	4.885	0.59	7	5.406	0.9	40	u	0.154224
			203299_s_at	2	5.052	0.48	7	5.256	0.55	40	u	0.370919
			230264_s_at	2	8.418	0.57	7	8.604	0.89	40	u	0.602592
			203300_x_at	1	7.478	0.69	7	7.859	0.79	40	u	0.248393
RAPGEF6	2	Rap guanine nucleotide exchange factor (GEF) 6	219112_at	2	8.017	0.42	7	7.688	0.69	40	d	0.238063
			1555247_a_at	2	7.411	0.39	7	6.94	1.12	40	d	0.06211
			230078_at	0	8.616	0.35	7	8.316	0.75	40	d	0.313118
			239438_at	0	4.228	0.19	7	4.493	0.58	40	u	0.039723
ARHGEF9	2	Cdc42 guanine nucleotide exchange factor (GEF) 9	203263_s_at	2	5.294	0.61	7	6.07	1.5	40	u	0.191931
			203264_s_at	2	7.426	0.31	7	6.836	1.42	40	d	0.028009
NAT8L	2	N-acetyltransferase 8-like	235316_at	2	4.361	0.71	7	4.67	0.71	40	u	0.305655
C19orf43	2	chromosome 19 open reading frame 43	223003_at	2	11.01	0.1	7	10.78	0.62	40	d	0.034033
			230213_at	0	6.539	0.46	7	6.436	0.91	40	d	0.777566
NEFH	2	neurofilament, heavy polypeptide 200kDa	204412_s_at	2	4.775	0.54	7	4.783	0.98	40	u	0.982822
			33767_at	2	3.1	0.24	7	3.242	1.46	40	u	0.578283
PGBD5	2	piggyBac transposable element derived 5	219225_at	2	2.893	0.31	7	4.684	2.26	40	u	0.000027
CCNY	2	cyclin Y	224651_at	2	8.719	0.32	7	9.109	0.84	40	u	0.23989
			224652_at	2	8.063	0.11	7	8.537	0.74	40	u	0.000542
			1554694_at	0	2.406	0.04	7	2.498	0.18	40	u	0.009456
ETNK2	2	ethanolamine kinase 2	219268_at	0	6.537	0.48	7	5.985	1.41	40	d	0.077143
CCAR1	2	cell division cycle and apoptosis regulator 1	224736_at	2	9.156	0.18	7	9.715	0.49	40	u	0.005966
			224737_x_at	2	6.936	0.23	7	7.461	0.44	40	u	0.004436
			239014_at	0	5.269	0.33	7	5.525	1.02	40	u	0.238884
TOR3A	2	torsin family 3, member A	218459_at	2	7.414	0.31	7	8.208	0.69	40	u	0.005666
			233851_s_at	0	6.767	0.26	7	7.2	0.9	40	u	0.022326
SYT12	2	synaptotagmin XII	215860_at	0	4.677	0.33	7	4.586	0.47	40	d	0.630509
			215865_at	0	2.415	0.37	7	2.472	0.41	40	u	0.742857
			228072_at	0	7.041	0.71	7	6.385	1.49	40	d	0.270119
SETDB1	2	SET domain, bifurcated 1	203155_at	2	7.685	0.24	7	7.737	0.68	40	u	0.727481
			214197_s_at	0	5.99	0.3	7	6.185	0.73	40	u	0.498406
EIF1AX	2	eukaryotic translation initiation factor 1A, X-linked	201018_at	2	9.391	0.28	7	8.922	0.75	40	d	0.116664
			201016_at	2	8.106	0.23	7	9.046	0.78	40	u	0.000001
			201017_at	2	9.139	0.23	7	8.814	0.83	40	d	0.054906
NDUFS4	2	NADH dehydrogenase (ubiquinone) Fe-S protein 4, 18kDa (NADH-coenzyme Q	209303_at	2	9.969	0.18	7	9.273	0.73	40	d	0.000014
			1555057_at	0	4.279	0.63	7	4.202	0.67	40	d	0.782963

CLCN4	2	chloride channel 4	205148_s_at	2	2.418	0.41	7	2.719	0.87	40	u	0.381307
			205149_s_at	2	1.975	0.01	7	2.158	0.34	40	u	0.002017
			231066_s_at	0	1.943	0.02	7	2.016	0.14	40	u	0.004392
MMP15	2	matrix metallopeptidase 15 (membrane-inserted)	203365_s_at	2	5.519	0.39	7	5.815	1.17	40	u	0.240947
			243883_at	0	3.444	0.27	7	3.638	0.32	40	u	0.144131
SEC23IP	2	SEC23 interacting protein	209175_at	2	7.751	0.25	7	7.806	0.6	40	u	0.817612
			209176_at	2	5.326	0.22	7	5.488	0.4	40	u	0.319819
			216392_s_at	0	7.549	0.38	7	7.816	0.61	40	u	0.276554
RGS4	2	regulator of G-protein signaling 4	204337_at	2	5.245	0.73	7	5.509	1.85	40	u	0.717256
			204339_s_at	1	3.732	0.55	7	4.525	0.98	40	u	0.048245
			204338_s_at	0	3.697	0.47	7	4.043	1.05	40	u	0.406872
CNOT1	2	CCR4-NOT transcription complex, subunit 1	200861_at	2	8.302	0.18	7	8.086	0.5	40	d	0.062005
			200860_s_at	1	9.688	0.21	7	9.624	0.47	40	d	0.731161
			1554052_at	0	2.93	0.45	7	3.843	1.13	40	u	0.044659
ZBTB11	2	zinc finger and BTB domain containing 11	204847_at	2	7.96	0.12	7	8.128	0.59	40	u	0.119107
			242433_at	0	3.595	0.4	7	3.863	0.64	40	u	0.299866
TMEM1	2	transmembrane protein 1	208184_s_at	2	6.394	0.27	7	6.565	0.49	40	u	0.37955
			209412_at	2	6.013	0.51	7	6.188	0.76	40	u	0.566896
			215269_at	0	5.104	0.88	7	5.132	1.07	40	u	0.949661
			1555446_s_at	0	3.582	0.65	7	4.14	0.9	40	u	0.133125
TMEM104	2	transmembrane protein 104	220097_s_at	0	4.863	0.27	7	4.729	0.74	40	d	0.415864
PTPN5	2	protein tyrosine phosphatase, non-receptor type 5 (striatum-enriched)	236456_at	2	2.564	0.2	7	2.65	0.34	40	u	0.524729
			233471_at	0	2.175	0.02	7	2.258	0.13	40	u	0.000489
			233470_at	0	2.139	0.19	7	2.187	0.21	40	u	0.579333
PAQR8	2	progestin and adipoQ receptor family member VIII	227626_at	2	4.448	0.43	7	5.138	1.05	40	u	0.100924
			226423_at	2	5.408	0.44	7	5.844	0.76	40	u	0.155485
IGSF11	2	immunoglobulin superfamily, member 11	228375_at	2	2.262	0.03	7	2.381	0.21	40	u	0.001515
SNX25	2	sorting nexin 25	230066_at	0	1.998	0.09	7	2.035	0.09	40	u	0.309108
			1555388_s_at	0	6.109	0.53	7	5.57	0.73	40	d	0.07237
			227408_s_at	0	7.425	0.38	7	7.069	0.54	40	d	0.106171
			232425_at	0	2.938	0.15	7	3.143	0.33	40	u	0.123451
			227311_at	0	2.238	0.17	7	2.292	0.22	40	u	0.547493
ZNF687	2	zinc finger protein 687	234924_s_at	2	6.315	0.27	7	6.736	0.96	40	u	0.032613
ANKRD49	2	ankyrin repeat domain 49	219069_at	2	8.258	0.36	7	7.851	0.98	40	d	0.295316
MAPK10	2	mitogen-activated protein kinase 10	204813_at	2	5.331	0.78	7	4.88	1.54	40	d	0.461843
LRRTM3	2	leucine rich repeat transmembrane neuronal 3	no probeset available									
GLIS2	2	GLIS family zinc finger 2	223378_at	2	6.689	0.45	7	6.468	0.94	40	d	0.554199
HABP4	2	hyaluronan binding protein 4	209819_at	4	3.536	0.35	7	3.517	0.25	40	d	0.866884
			209818_s_at	4	7.369	0.3	7	6.846	0.27	40	d	0.00005
			232341_x_at	4	4.708	0.68	7	4.129	0.56	40	d	0.020611
			233919_s_at	0	5.799	0.37	7	5.279	0.56	40	d	0.024874
CHRM1	2	cholinergic receptor, muscarinic 1	231783_at	2	4.013	0.99	7	3.574	0.68	40	d	0.161701
DNAJA3	2	DnaJ (Hsp40) homolog, subfamily A, member 3	1554078_s_at	2	8.861	0.26	7	9.212	0.53	40	u	0.102111
			205963_s_at	1	8.226	0.24	7	8.49	0.47	40	u	0.165879
ZBTB20	2	zinc finger and BTB domain containing 20	205383_s_at	2	10.28	0.31	7	8.199	1.2	40	d	0
			222357_at	0	8.593	0.31	7	6.501	1.13	40	d	0
			235308_at	0	9.835	0.35	7	8.113	1.13	40	d	0
IPO13	2	importin 13	203546_at	0	6.927	0.33	7	7.281	0.53	40	u	0.100993
KLHL7	2	kelch-like 7 (Drosophila)	223250_at	2	8.856	0.19	7	8.489	0.78	40	d	0.01703
			220239_at	0	5.159	0.39	7	6.575	1.32	40	u	0.000008
			220238_s_at	0	5.121	0.34	7	6.411	1.32	40	u	0.000011
DCUN1D5	2	DCN1, defective in cullin neddylation 1, domain containing 5 (S. cerevisiae)	223151_at	2	8.296	0.31	7	9.055	0.82	40	u	0.02168
SLC39A5	2	solute carrier family 39 (metal ion transporter), member 5	231667_at	2	2.399	0.2	7	2.34	0.13	40	d	0.328513
			1552281_at	2	4.674	0.44	7	5.034	0.46	40	u	0.068775
PTPN2	2	protein tyrosine phosphatase, non-receptor type 2	204935_at	2	3.187	0.28	7	3.785	0.66	40	u	0.026063
			241622_at	0	2.348	0.1	7	2.385	0.14	40	u	0.528553
			241623_at	0	2.801	0.29	7	3.104	0.49	40	u	0.127526
			213137_s_at	0	8.264	0.23	7	8.401	0.94	40	u	0.447865
			240843_at	0	5.852	0.38	7	5.549	0.86	40	d	0.377603
			213136_at	0	8.678	0.29	7	8.499	0.82	40	d	0.317032
			241983_at	0	3.831	0.42	7	3.712	0.63	40	d	0.642236
			231961_at	3	3.026	0.47	7	2.658	0.21	40	d	0.106182
			207837_at	2	4.184	0.68	7	3.826	0.42	40	d	0.071809

RBPMS	2	RNA binding protein with multiple splicing	207836_s_at	2	8.869	0.27	7	6.877	1.32	40	d	0
			209487_at	0	8.787	0.24	7	7.295	1.43	40	d	0
			209488_s_at	0	9.688	0.28	7	7.606	1.49	40	d	0
NMNAT2	2	nicotinamide nucleotide adenylyltransferase 2	209755_at	2	2.291	0.56	7	2.918	1.72	40	u	0.090589
			1556029_s_at	2	1.91	0.01	7	2.442	1.24	40	u	0.010563
			1552712_a_at	1	2.962	0.35	7	3.437	1.25	40	u	0.06354
			1562818_at	0	2.013	0.09	7	2.049	0.16	40	u	0.580783
TRAF7	2	TNF receptor-associated factor 7	223031_s_at	5	7.145	0.23	7	7.629	0.59	40	u	0.041076
			223029_s_at	5	6.683	0.27	7	6.925	0.47	40	u	0.203672
			223030_at	5	2.645	0.12	7	2.698	0.13	40	u	0.326124
CXCR7	2	chemokine (C-X-C motif) receptor 7	212977_at	2	7.168	1.54	7	6.38	1.25	40	d	0.154093
			232746_at	0	4.901	0.85	7	4.75	0.81	40	d	0.662037
			1559114_a_at	0	2.568	0.18	7	2.726	0.22	40	u	0.090051
FSTL5	2	follistatin-like 5	232010_at	2	2.486	0.03	7	2.555	0.08	40	u	0.000329
BRD4	2	bromodomain containing 4	202103_at	2	5.814	0.32	7	5.832	0.93	40	u	0.930135
			202102_s_at	2	9.768	0.51	7	9.434	0.97	40	d	0.388444
			240360_at	0	3.118	0.22	7	3.148	0.35	40	u	0.825692
			239000_at	0	4.469	0.28	7	4.264	0.53	40	d	0.333789
			226054_at	0	6.798	0.45	7	7.176	1.16	40	u	0.409108
ZNF146	2	zinc finger protein 146	200050_at	2	9.865	0.22	7	10.35	0.65	40	u	0.001348
			1554433_a_at	0	7.743	0.48	7	8.069	1.41	40	u	0.28675
DOK4	2	docking protein 4	209691_s_at	2	6.697	0.2	7	5.935	0.68	40	d	0.000005
			209690_s_at	2	3.403	0.6	7	3.184	0.41	40	d	0.245866
			207747_s_at	0	6.145	0.41	7	5.842	0.52	40	d	0.158914
MTMR6	2	myotubularin related protein 6	214429_at	2	8.681	0.29	7	8.704	0.59	40	u	0.920549
			228789_at	0	3.562	0.3	7	3.617	0.57	40	u	0.807933
CUGBP1	2	CUG triplet repeat, RNA binding protein 1	209489_at	2	9.113	0.22	7	8.947	0.69	40	d	0.254516
			204113_at	0	4.989	0.45	7	5.605	0.98	40	u	0.116817
			235297_at	0	2.684	0.27	7	2.937	0.49	40	u	0.200511
			221743_at	0	10.51	0.23	7	10.54	0.43	40	u	0.859342
			1555467_a_at	0	6.994	0.38	7	7.141	1.19	40	u	0.556637
			221742_at	0	8.405	0.36	7	8.449	0.45	40	u	0.812814
			235865_at	0	3.036	0.23	7	3.223	0.38	40	u	0.227557
ENTPD7	2	ectonucleoside triphosphate diphosphohydrolase 7	220153_at	2	3.131	0.37	7	4.411	0.74	40	u	0.000071
PDGFRB	2	platelet-derived growth factor receptor, beta polypeptide	202273_at	1	7.991	0.63	7	6.717	1.01	40	d	0.002713
SAMD10	2	sterile alpha motif domain containing 10	233001_at	2	3.137	0.5	7	2.836	0.57	40	d	0.207965
			227773_at	2	4.759	0.26	7	4.827	0.39	40	u	0.669772
EDG2	2	endothelial differentiation, lysophosphatidic acid G-protein-coupled receptor, 2	204037_at	2	8.119	0.43	7	6.749	0.8	40	d	0.000083
			204036_at	2	8.31	0.38	7	6.639	1.27	40	d	0
			204038_s_at	0	5.497	0.76	7	5.099	0.67	40	d	0.17254
MYF5	2	myogenic factor 5	207424_at	2	3.092	0.34	7	3.124	0.27	40	u	0.788816
DLST	2	dihydrolipoamide S-succinyltransferase (E2 component of 2-oxo-glutarate complex)	215210_s_at	0	7.93	0.19	7	7.805	0.63	40	d	0.341475
LDLR	2	low density lipoprotein receptor (familial hypercholesterolemia)	202067_s_at	2	6.956	0.87	7	6.646	1.33	40	d	0.564535
			202068_s_at	2	8.781	0.64	7	7.995	1.58	40	d	0.209956
			217103_at	1	2.8	0.05	7	2.9	0.12	40	u	0.03743
			217005_at	1	3.7	0.27	7	3.765	0.4	40	u	0.689043
			217183_at	0	2.372	0.14	7	2.387	0.14	40	u	0.790491
			217173_s_at	0	5.735	0.66	7	5.471	1.39	40	d	0.631736
SCRN1	2	secernin 1	201462_at	2	8.907	0.4	7	9.034	1.08	40	u	0.764133
ZNF384	2	zinc finger protein 384	212369_at	2	8.615	0.4	7	8.349	0.7	40	d	0.342467
KLF2	2	Kruppel-like factor 2 (lung)	226645_at	2	2.41	0.15	7	2.38	0.22	40	d	0.736107
			219371_s_at	2	10.32	0.9	7	8.602	1.08	40	d	0.000339
			226646_at	2	3.187	0.78	7	2.758	0.49	40	d	0.064511
RPN1	2	ribophorin I	201011_at	2	10.16	0.2	7	10.06	0.71	40	d	0.51616
			230862_at	0	2.394	0.19	7	2.537	0.22	40	u	0.118307
WDR16	2	WD repeat domain 16	239916_at	2	2.982	0.17	7	3.098	0.39	40	u	0.453296
			237755_s_at	1	2.512	0.03	7	2.631	0.14	40	u	0.000024
PGM1	2	phosphoglucomutase 1	201968_s_at	2	10.02	0.22	7	9.486	0.84	40	d	0.002549
SNRPC	2	small nuclear ribonucleoprotein polypeptide C	201342_at	2	8.747	0.22	7	9.427	0.53	40	u	0.002131

SNCAIP	2	synuclein, alpha interacting protein (synphilin)	219511_s_at	2	5.456	0.74	7	4.435	1.02	40	d	0.017384
			237834_at	0	3.28	0.12	7	3.327	0.19	40	u	0.540993
			237833_s_at	0	5.176	0.36	7	4.418	0.66	40	d	0.005895
PELI3	2	pellino homolog 3 (Drosophila)	235431_s_at	2	6.675	0.22	7	6.172	0.88	40	d	0.004739
			244214_at	0	2.364	0.14	7	2.646	0.46	40	u	0.005029
FAM120A	2	family with sequence similarity 120A	200774_at	2	11.18	0.27	7	11.36	0.54	40	u	0.405005
			1555944_at	1	3.582	0.74	7	4.078	0.86	40	u	0.168069
			1555947_at	1	2.121	0.07	7	2.35	0.44	40	u	0.004716
			1555945_s_at	1	7.031	0.43	7	7.999	0.51	40	u	0.000031
			1555948_s_at	1	7.51	0.48	7	8.301	0.94	40	u	0.03802
			200767_s_at	0	7.195	0.33	7	7.711	0.53	40	u	0.019724
			1555908_at	0	2.698	0.2	7	2.911	0.44	40	u	0.22875
			1559711_at	0	2.378	0.07	7	2.482	0.22	40	u	0.030895
			210516_at	0	2.87	0.09	7	3.078	0.22	40	u	0.020798
THPO	2	thrombopoietin (myeloproliferative leukemia virus oncogene ligand, megakaryocyte growth and development factor)	211154_at	2	2.692	0.15	7	2.718	0.18	40	u	0.730806
			211155_s_at	2	2.732	0.09	7	2.846	0.22	40	u	0.184457
			211831_s_at	0	3.053	0.12	7	3.303	0.31	40	u	0.042702
JAK2	2	Janus kinase 2 (a protein tyrosine kinase)	205841_at	2	4.256	0.44	7	4.218	0.78	40	d	0.902115
			205842_s_at	0	6.544	0.45	7	6.6	1.29	40	u	0.839838
			1562031_at	0	6.178	0.4	7	5.77	0.73	40	d	0.167419
RAB5A	2	RAB5A, member RAS oncogene family	209089_at	2	10.7	0.27	7	10.74	0.49	40	u	0.849031
			240990_at	0	3.602	0.37	7	3.931	0.41	40	u	0.057364
			206113_s_at	0	7.889	0.77	7	7.992	1.33	40	u	0.846008
AQP11	2	aquaporin 11	229526_at	0	2.675	0.61	7	2.999	1.35	40	u	0.544603
NF1	2	neurofibromin 1 (neurofibromatosis, von Recklinghausen disease, Watson disease)	204325_s_at	2	3.413	0.49	7	3.78	0.91	40	u	0.31299
			211914_x_at	0	2.562	0.28	7	2.925	0.84	40	u	0.050406
			216115_at	0	6.432	0.55	7	5.55	0.86	40	d	0.014043
			211095_at	0	3.255	0.15	7	3.599	0.44	40	u	0.000919
			212678_at	0	7.972	0.27	7	7.026	0.84	40	d	0.000008
			212676_at	0	7.936	0.25	7	7.602	0.64	40	d	0.18676
			211094_s_at	0	5.4	0.38	7	5.76	1.22	40	u	0.158965
			210631_at	0	3.199	0.44	7	3.176	0.38	40	d	0.889034
			204323_x_at	0	4.227	0.53	7	4.756	0.92	40	u	0.156872
IMPAD1	2	inositol monophosphatase domain containing 1	222654_at	2	6.934	0.36	7	8.388	0.9	40	u	0.000154
			227774_s_at	0	2.218	0.08	7	2.271	0.11	40	u	0.261461
			224743_at	0	9.374	0.3	7	9.318	0.91	40	d	0.772757
			218516_s_at	0	6.889	0.33	7	7.753	1.03	40	u	0.000409
			224744_at	0	7.162	0.29	7	8.05	0.81	40	u	0.000042
TAOK2	2	TAO kinase 2	204878_s_at	2	6.453	0.42	7	5.97	0.51	40	d	0.025915
			204877_s_at	2	6.025	0.38	7	5.602	0.71	40	d	0.13602
			204986_s_at	0	5.534	0.42	7	5.658	1.03	40	u	0.757767
SEPHS1	2	selenophosphate synthetase 1	208940_at	2	7.096	0.39	7	7.361	1.11	40	u	0.279737
			208939_at	2	8.484	0.19	7	8.706	0.83	40	u	0.156662
			208941_s_at	0	7.904	0.28	7	8.624	1.18	40	u	0.002381
LYPD3	2	LY6/PLAUR domain containing 3	204952_at	2	7.733	0.52	7	6.681	1.69	40	d	0.004771
CACNG2	2	calcium channel, voltage-dependent, gamma subunit 2	214495_at	1	2.376	0.09	7	2.461	0.22	40	u	0.325899
ELF1	2	E74-like factor 1 (ets domain transcription factor)	212418_at	2	9.658	0.28	7	8.968	0.75	40	d	0.023393
			212420_at	2	8.665	0.36	7	8.987	0.77	40	u	0.294695
CLIP2	2	CAP-GLY domain containing linker protein 2	211031_s_at	2	5.251	0.5	7	4.967	0.91	40	d	0.434259
C19orf22	2	chromosome 19 open reading frame 22	55705_at	2	9.386	0.12	7	9.456	0.52	40	u	0.473702
			221764_at	2	8.514	0.29	7	8.363	1.01	40	d	0.459416
CD93	2	CD93 molecule	202877_s_at	2	6.521	0.72	7	5.975	0.83	40	d	0.118684
			202878_s_at	2	8.882	0.67	7	7.773	0.9	40	d	0.00375
ADD1	2	adducin 1 (alpha)	208030_s_at	2	8.072	0.3	7	7.257	0.73	40	d	0.006894
			238939_at	0	4.49	0.53	7	4.395	0.82	40	d	0.773284
			214726_x_at	0	8.096	0.36	7	7.766	0.69	40	d	0.232823
			214736_s_at	0	9.599	0.22	7	8.469	0.74	40	d	0
TOR2A	2	torsin family 2, member A	227972_at	2	4.956	0.5	7	4.953	0.59	40	d	0.988069
			244155_x_at	0	2.307	0.06	7	2.369	0.14	40	u	0.269826
KIAA1546	2	KIAA1546	227624_at	0	7.378	0.27	7	7.339	0.59	40	d	0.86848
			1569385_s_at	0	4.074	0.21	7	4.927	0.85	40	u	0.000004
			235461_at	0	3.409	0.64	7	4.148	0.75	40	u	0.020221

ANP32A	2	acidic (leucine-rich) nuclear phosphoprotein 32 family, member A	201043_s_at	0	7.875	0.27	7	8.526	0.68	40	u	0.017745
			208571_at	0	2.539	0.04	7	2.628	0.11	40	u	0.001078
			201038_s_at	0	8.655	0.27	7	8.425	0.54	40	d	0.283285
			201051_at	0	10.05	0.23	7	10.12	0.59	40	u	0.765926
BTBD14A	2	BTB (POZ) domain containing 14A	230050_at	0	2.331	0.18	7	2.637	0.48	40	u	0.108261
DNAJC1	2	DnaJ (Hsp40) homolog, subfamily C, member 1	218409_s_at	2	7.52	0.21	7	8.074	1.08	40	u	0.006434
			222620_s_at	2	10.33	0.25	7	10.94	1.22	40	u	0.00807
			222621_at	2	8.645	0.2	7	9.218	1.27	40	u	0.012545
SMS	2	spermine synthase	202043_s_at	2	9.534	0.15	7	10.45	0.8	40	u	0
FBXO3	2	F-box protein 3	218432_at	2	7.864	0.34	7	7.547	1.28	40	d	0.208084
			229955_at	0	5.574	0.7	7	5.564	0.88	40	d	0.978283
			242791_at	0	3.328	0.13	7	3.596	0.42	40	u	0.003879
			232815_at	0	2.74	0.25	7	3.171	0.95	40	u	0.023881
			238686_at	0	6.101	0.71	7	6.668	1.28	40	u	0.26814
			232851_at	0	3.817	0.37	7	4.143	0.46	40	u	0.090491
FLI1	2	Friend leukemia virus integration 1	204236_at	2	6.209	0.78	7	5.427	0.88	40	d	0.036482
			210786_s_at	0	5.673	0.49	7	5.382	0.77	40	d	0.352446
			237722_at	0	2.382	0.05	7	2.508	0.27	40	u	0.012008
			211825_s_at	0	4.852	0.89	7	4.513	1	40	d	0.414991
DGKQ	2	diacylglycerol kinase, theta 110kDa	226605_at	2	6.367	0.33	7	6.449	0.86	40	u	0.808682
			207562_at	0	2.137	0.05	7	2.185	0.16	40	u	0.162185
MEGF11	2	multiple EGF-like-domains 11	1552439_s_at	2	2.576	0.06	7	2.735	0.21	40	u	0.000397
			1560334_at	0	2.147	0.03	7	2.213	0.07	40	u	0.000293
			1569879_a_at	0	4.039	0.43	7	4.026	0.69	40	d	0.960491
IL15RA	2	interleukin 15 receptor, alpha	207375_s_at	2	5.921	0.46	7	5.645	1.16	40	d	0.545991
SNCA	2	synuclein, alpha (non A4 component of amyloid precursor)	204466_s_at	2	6.555	0.46	7	4.497	0.86	40	d	0
			204467_s_at	2	4.812	0.18	7	4.352	0.4	40	d	0.0058
			211546_x_at	0	7.561	0.35	7	7.049	0.24	40	d	0.00003
			207827_x_at	0	7.551	0.26	7	7.005	0.5	40	d	0.008049
CLIC5	2	chloride intracellular channel 5	219866_at	2	6.216	0.45	7	5.868	0.39	40	d	0.043119
			243917_at	0	1.986	0.04	7	2.088	0.18	40	u	0.003619
			234329_at	0	2.194	0.02	7	2.244	0.05	40	u	0.000165
			213317_at	0	4.579	0.79	7	3.223	0.72	40	d	0.000057
			217628_at	0	2.762	0.27	7	2.936	0.32	40	u	0.186046
ARHGAP26	2	Rho GTPase activating protein 26	205068_s_at	2	6.099	0.36	7	5.827	0.66	40	d	0.303516
			205069_s_at	2	3.791	0.4	7	3.701	0.3	40	d	0.496545
			226576_at	0	5.253	0.62	7	3.209	0.74	40	d	0
			215955_x_at	0	2.689	0.27	7	2.673	0.39	40	d	0.915646
EPB41L3	2	erythrocyte membrane protein band 4.1-like 3	211776_s_at	0	7.122	0.62	7	6.423	1.2	40	d	0.147698
			206710_s_at	0	7.361	0.83	7	6.582	1.41	40	d	0.171765
			212681_at	0	6.023	0.57	7	5.631	1.12	40	d	0.380643
SAE2	2	SUMO1 activating enzyme subunit 2	229587_at	2	5.289	0.34	7	5.505	0.51	40	u	0.300013
			201177_s_at	2	9.605	0.21	7	10.36	0.7	40	u	0.00001
CDS2	2	CDP-diacylglycerol synthase (phosphatidate cytidylyltransferase) 2	212862_at	2	7.045	0.36	7	7.118	0.89	40	u	0.83382
			212864_at	2	8.01	0.4	7	7.767	0.77	40	d	0.424932
			233630_at	0	2.708	0.11	7	2.849	0.42	40	u	0.095967
ITGA2	2	integrin, alpha 2 (CD49B, alpha 2 subunit of VLA-2 receptor)	205032_at	2	6.944	0.72	7	5.91	1.1	40	d	0.023331
			227314_at	0	8.604	0.5	7	7.162	1.36	40	d	0.000069
MME	2	membrane metallo-endopeptidase	203434_s_at	0	8.856	0.6	7	4.362	1.18	40	d	0
			203435_s_at	0	8.705	0.78	7	5.14	0.81	40	d	0
KCTD2	2	potassium channel tetramerisation domain containing 2	34858_at	2	7.453	0.2	7	6.919	0.64	40	d	0.000319
			212564_at	2	7.341	0.25	7	6.691	0.66	40	d	0.014815
CCNA2	2	cyclin A2	213226_at	2	5.13	0.6	7	7.826	0.89	40	u	0
			203418_at	2	2.848	0.38	7	6.661	1.71	40	u	0
GBX2	2	gastrulation brain homeobox 2	210560_at	0	2.68	0.09	7	2.729	0.11	40	u	0.308908
C10orf114	2	chromosome 10 open reading frame 114	1555923_a_at	2	3.049	0.19	7	2.964	0.27	40	d	0.443796
			1555922_at	2	4.61	0.41	7	4.831	0.53	40	u	0.311758
GPD1L	2	glycerol-3-phosphate dehydrogenase 1-like	212510_at	2	9.911	0.53	7	8.09	1.49	40	d	0.000008
KLHDC2	2	kelch domain containing 2	217906_at	2	10.83	0.32	7	9.723	0.92	40	d	0.000008
PIAS1	2	protein inhibitor of activated STAT, 1	217863_at	4	7.625	0.33	7	6.873	0.55	40	d	0.00134
			217862_at	4	8.154	0.34	7	7.167	0.84	40	d	0.004426
			217864_s_at	2	9.078	0.26	7	8.508	0.61	40	d	0.020883
[illegible]		tRNA splicing endonuclease 54 homolog (S	1558304_s_at	2	7.409	0.16	7	7.969	0.55	40	u	0.000017

TSEN54	2	tRNA splicing endonuclease 54 homolog (S. cerevisiae)	225879_at	2	7.026	0.15	7	7.588	0.69	40	u	0.000059
			241402_at	0	6.245	0.37	7	6.057	0.62	40	d	0.455376
FSD1	2	fibronectin type III and SPRY domain containing 1	219170_at	2	3.177	0.16	7	3.477	0.84	40	u	0.051315
TMEM134	2	transmembrane protein 134	218531_at	2	8.81	0.22	7	8.771	0.71	40	d	0.793909
			233018_at	0	2.143	0.03	7	2.162	0.04	40	u	0.226354
			1558712_at	0	2.257	0.19	7	2.202	0.27	40	d	0.614659
SEC61A1	2	Sec61 alpha 1 subunit (S. cerevisiae)	217716_s_at	2	9.289	0.29	7	9.184	0.58	40	d	0.647711
			222385_x_at	0	7.498	0.58	7	7.885	1.7	40	u	0.294964
CRTAP	2	cartilage associated protein	227138_at	2	4.563	0.34	7	4.01	0.6	40	d	0.025971
			226656_at	0	8.023	0.49	7	6.739	0.92	40	d	0.001001
			1555889_a_at	0	10.81	0.21	7	9.66	0.64	40	d	0
			1554464_a_at	0	7.787	0.84	7	6.789	1.47	40	d	0.095195
			201380_at	0	8.027	0.39	7	7.265	0.7	40	d	0.008852
WNT16	2	wingless-type MMTV integration site family, member 16	224022_x_at	2	2.82	0.09	7	2.925	0.16	40	u	0.096573
			221113_s_at	0	2.423	0.14	7	2.445	0.18	40	u	0.771383
SNW1	2	SNW domain containing 1	202781_s_at	2	6.689	0.33	7	6.24	0.64	40	d	0.080497
			202782_s_at	2	7.457	0.16	7	6.953	0.71	40	d	0.000402
			201575_at	0	9.949	0.13	7	9.732	0.37	40	d	0.009947
			228509_at	0	3.38	1.13	7	2.618	1.08	40	d	0.10187
			222183_x_at	0	4.31	0.24	7	4.796	0.93	40	u	0.009853
			215424_s_at	0	9.092	0.14	7	8.774	0.7	40	d	0.016057
EFCAB4A	2	EF-hand calcium binding domain 4A	227429_at	2	9.565	0.45	7	7.359	1.46	40	d	0
GALNT10	2	UDP-N-acetyl-alpha-D-galactosamine:polypeptide N-acetylgalactosaminyltransferase 10 (GalNAc-T10)	212256_at	2	8.078	0.68	7	6.864	1.59	40	d	0.057832
			207357_s_at	1	5.844	0.56	7	6.122	1.32	40	u	0.593991
			220296_at	0	6.69	0.72	7	6.598	0.96	40	d	0.814516
			230906_at	0	6.081	0.62	7	5.442	1.61	40	d	0.315295
C20orf112	2	chromosome 20 open reading frame 112	225224_at	0	7.424	0.35	7	7.384	1.25	40	d	0.869308
			234031_at	0	2.882	0.34	7	3.19	0.43	40	u	0.085777
			230954_at	0	2.453	0.09	7	2.798	0.87	40	u	0.022006
			230955_s_at	0	3.145	0.43	7	3.222	0.48	40	u	0.698589
			241830_at	0	1.936	0.07	7	1.936	0.11	40	u	0.995343
PABPN1	2	poly(A) binding protein, nuclear 1	201545_s_at	2	8.932	0.24	7	8.587	0.56	40	d	0.120905
			201544_x_at	2	10.9	0.13	7	10.63	0.41	40	d	0.00401
			213046_at	0	7.746	0.3	7	7.451	0.91	40	d	0.132276
ASPN	2	asporin	219087_at	2	8.178	1.18	7	9.019	1.72	40	u	0.228698
			224396_s_at	0	4.64	1.59	7	5.79	2.06	40	u	0.176305
CABP7	2	calcium binding protein 7	243173_at	2	3.267	0.15	7	3.536	0.69	40	u	0.040882
			234796_at	0	1.937	0.03	7	1.973	0.05	40	u	0.07422
KIAA1429	2	KIAA1429	238818_at	2	3.917	0.37	7	4.429	1.05	40	u	0.032028
			223110_at	1	8.208	0.27	7	8.561	0.68	40	u	0.192196
			243927_x_at	0	3.667	0.28	7	4.047	0.52	40	u	0.069706
VNN3	2	vanin 3	220528_at	2	2.482	0.17	7	2.718	0.58	40	u	0.053766
			1553513_at	0	2.853	0.35	7	3.153	0.48	40	u	0.130422
			1553514_a_at	0	3.109	0.2	7	3.418	0.23	40	u	0.00196
HORMAD2	2	HORMA domain containing 2	237781_at	2	2.596	0.12	7	2.886	0.45	40	u	0.001987
			244442_at	1	2.018	0.1	7	2.115	0.28	40	u	0.375175
ABCD3	2	ATP-binding cassette, sub-family D (ALD), member 3	202850_at	2	10.19	0.41	7	10.32	0.88	40	u	0.720921
			1554878_a_at	0	6.15	0.68	7	7.046	1.89	40	u	0.040122
FAM20B	2	family with sequence similarity 20, member B	202916_s_at	2	7.239	0.34	7	7.923	0.54	40	u	0.002612
			202915_s_at	2	7.417	0.25	7	8.585	0.66	40	u	0.000048
			217320_at	0	4.383	1.05	7	3.711	1.33	40	d	0.220738
EGR4	2	early growth response 4	207768_at	2	2.546	0.09	7	2.914	1.23	40	u	0.075054
			207767_s_at	2	4.102	0.44	7	4.145	0.86	40	u	0.901104
			231575_at	1	2.229	0.07	7	2.261	0.08	40	u	0.344543
FAM3C	2	family with sequence similarity 3, member C	201889_at	2	9.657	0.32	7	9.537	0.68	40	d	0.653285
			240062_at	0	2.815	0.08	7	3.046	0.3	40	u	0.00045
			236316_at	0	3.366	0.15	7	3.839	0.54	40	u	0.000068
NUTF2	2	nuclear transport factor 2	202397_at	2	8.444	0.47	7	8.508	0.96	40	u	0.865548
			228379_at	0	6.303	0.19	7	6.195	0.57	40	d	0.377875
ADRBK1	2	adrenergic, beta, receptor kinase 1	201402_at	2	6.173	0.32	7	6.11	0.32	40	d	0.642801
			38447_at	2	5.228	0.22	7	5.191	0.34	40	d	0.785073
			201401_s_at	0	6.951	0.31	7	6.765	0.89	40	d	0.338465
HOXD8	2	homeobox D8	231906_at	2	5.713	0.71	7	5.022	1.04	40	d	0.106675

C1orf96	2	chromosome 1 open reading frame 96	225904_at	2	7.53	0.32	7	8.144	0.75	40	u	0.042314
			1553698_a_at	1	2.81	0.18	7	3.94	1.33	40	u	0.000009
			1553697_at	1	3.209	0.23	7	4.154	0.78	40	u	0.000001
			1555145_at	0	2.429	0.12	7	2.567	0.28	40	u	0.211158
FOXP2	2	forkhead box P2	1552902_a_at	2	2.349	0.18	7	2.318	0.07	40	d	0.702597
			1555647_a_at	0	2.564	0.04	7	2.639	0.1	40	u	0.052688
			1562139_a_at	0	2.789	0.13	7	2.885	0.17	40	u	0.174213
			1564876_s_at	0	5.614	0.13	7	5.633	0.22	40	u	0.821714
			1555648_at	0	3.001	0.14	7	3.298	0.37	40	u	0.046024
			1555352_at	0	2.594	0.3	7	2.555	0.42	40	d	0.820127
			1555516_at	0	2.269	0.15	7	2.362	0.46	40	u	0.351294
ATP11B	2	ATPase, Class VI, type 11B	212536_at	2	7.988	0.36	7	7.788	1.05	40	d	0.379493
			1564063_a_at	0	6.941	0.28	7	7.735	0.59	40	u	0.001405
			1564064_a_at	0	5.826	0.58	7	7.085	1.31	40	u	0.018447
			1554556_a_at	0	4.021	0.48	7	4.791	0.81	40	u	0.020719
			1554557_at	0	3.658	0.44	7	4.572	0.98	40	u	0.021849
			238811_at	0	3.754	0.57	7	4.951	1.28	40	u	0.0211
ENO1P	2	enolase 1, (alpha) pseudogene	no probeset available									
CSDC2	2	cold shock domain containing C2, RNA binding	209981_at	2	3.061	0.44	7	3.041	0.62	40	d	0.934103
GSC	2	goosecoid homeobox	1552338_at	2	1.932	0.11	7	1.939	0.24	40	u	0.939827
CRHR1	2	corticotropin releasing hormone receptor 1	214619_at	2	2.573	0.19	7	2.609	0.29	40	u	0.758996
			208593_x_at	0	2.327	0.23	7	2.259	0.21	40	d	0.456718
			211897_s_at	0	2.064	0.04	7	2.159	0.21	40	u	0.012052
SLC35F5	2	solute carrier family 35, member F5	225872_at	0	8.691	0.32	7	8.941	0.83	40	u	0.445228
			220123_at	0	3.03	0.17	7	3.373	0.36	40	u	0.018784
MAF1	2	MAF1 homolog (S. cerevisiae)	222998_at	2	8.73	0.23	7	8.922	0.74	40	u	0.215971
SLC5A7	2	solute carrier family 5 (choline transporter), member 7	222967_at	2	2.673	0.71	7	2.341	0.11	40	d	0.297527
			220722_s_at	0	2.244	0.08	7	2.377	0.32	40	u	0.031424
ST7L	2	suppression of tumorigenicity 7 like	236123_at	2	2.814	0.26	7	2.847	0.5	40	u	0.869083
			219964_at	1	3.734	0.34	7	3.784	0.61	40	u	0.834315
			230079_at	0	5.082	0.38	7	5.045	0.49	40	d	0.85407
			1552739_s_at	0	4.49	0.61	7	4.711	0.63	40	u	0.406977
			233141_s_at	0	2.681	0.24	7	2.716	0.33	40	u	0.793428
			1552738_a_at	0	3.557	0.27	7	4.058	0.5	40	u	0.015564
BLMH	2	bleomycin hydrolase	202179_at	1	7.014	0.38	7	7.281	1	40	u	0.497598
ADCYAP1	2	adenylate cyclase activating polypeptide 1 (pituitary)	206281_at	4	2.19	0.15	7	2.505	0.82	40	u	0.033932
KIAA0513	2	KIAA0513	204546_at	2	6.467	0.25	7	6.207	0.86	40	d	0.140874
			1554440_at	0	2.382	0.05	7	2.59	0.27	40	u	0.000067
AGRN	2	agrin	212283_at	2	3.099	0.31	7	2.922	0.49	40	d	0.369485
			212285_s_at	2	9.983	0.66	7	9.133	1.08	40	d	0.05472
			217410_at	2	4.913	0.31	7	4.417	0.58	40	d	0.035785
			217419_x_at	2	9.249	0.72	7	8.519	1.04	40	d	0.086319
CLIC4	2	chloride intracellular channel 4	201560_at	2	11.16	0.32	7	11.25	0.75	40	u	0.759928
			201559_s_at	1	7.109	0.94	7	7.94	1.94	40	u	0.283443
			221881_s_at	1	6.275	0.98	7	7.995	1.49	40	u	0.005961
LRP1	2	low density lipoprotein-related protein 1 (alpha-2-macroglobulin receptor)	200785_s_at	2	9.547	0.48	7	7.805	0.63	40	d	0
			200784_s_at	2	7.992	0.32	7	6.527	0.79	40	d	0.000022
			1555353_at	0	3.81	0.44	7	3.795	0.26	40	d	0.938479
			1569042_at	0	2.264	0.05	7	2.314	0.06	40	u	0.059712
NKX2-3	2	NK2 transcription factor related, locus 3 (Drosophila)	1553808_a_at	2	2.175	0.02	7	2.41	0.77	40	u	0.063159
SCG2	2	secretogranin II (chromogranin C)	204035_at	1	3.094	0.29	7	3.642	1.17	40	u	0.018665
DYNC1LI1	2	dynein, cytoplasmic 1, light intermediate chain 1	217976_s_at	2	7.055	0.49	7	7.523	0.88	40	u	0.18911
			222479_s_at	2	8.257	0.3	7	8.693	0.73	40	u	0.136236
BIVM	2	basic, immunoglobulin-like variable motif containing	229478_x_at	2	2.965	0.32	7	3.068	0.3	40	u	0.420353
			229589_x_at	2	4.489	0.55	7	4.577	0.66	40	u	0.744703
			222761_at	2	7.828	0.25	7	6.998	1.06	40	d	0.000141
			233255_s_at	1	6.073	0.37	7	5.755	0.94	40	d	0.392822
			1569289_at	0	2.325	0.1	7	2.513	0.24	40	u	0.055049
IPPK	2	inositol 1,3,4,5,6-pentakisphosphate 2-kinase	222823_at	3	4.158	0.47	7	4.479	0.67	40	u	0.238269
			219092_s_at	3	6.893	0.3	7	7.05	0.5	40	u	0.432844
EFHD2	2	EF-hand domain family, member D2	217992_s_at	2	7.479	0.54	7	7.421	0.87	40	d	0.868265

EFHD2	2	EF-hand domain family, member D2	222483_at	2	6.033	0.53	7	6.45	0.83	40	u	0.214175
RAB2B	2	RAB2B, member RAS oncogene family	225074_at	2	8.822	0.16	7	7.899	0.63	40	d	0
EGFR	2	epidermal growth factor receptor (erythroblastic leukemia viral (v-erb-b) oncogene homolog, avian)	201983_s_at	2	9.077	0.85	7	5.849	1.98	40	d	0.000148
			201984_s_at	2	5.81	0.82	7	5.078	1.24	40	d	0.14703
			1565483_at	0	2.905	0.42	7	4.4	1.38	40	u	0.000008
			211607_x_at	0	4.197	0.52	7	3.961	0.9	40	d	0.513594
			1565484_x_at	0	2.428	0.46	7	3.599	1.14	40	u	0.01175
			211550_at	0	2.805	0.07	7	2.841	0.09	40	u	0.335308
			210984_x_at	0	4.136	0.67	7	3.647	1.05	40	d	0.250995
			211551_at	0	2.872	0.36	7	2.932	0.47	40	u	0.755513
FAM19A2	2	family with sequence similarity 19 (chemokine (C-C motif)-like), member A2	241399_at	2	3.7	0.22	7	4.053	0.48	40	u	0.066445
TNPO1	2	transportin 1	207657_x_at	3	9.542	0.26	7	9.809	0.55	40	u	0.225692
			221829_s_at	3	11.18	0.2	7	11.46	0.6	40	u	0.033962
			209226_s_at	1	9.598	0.18	7	9.847	0.72	40	u	0.077098
			209225_x_at	1	9.188	0.22	7	9.732	0.57	40	u	0.018028
			1557278_s_at	0	3.394	0.18	7	3.932	0.54	40	u	0.00006
PACS1	2	phosphofurin acidic cluster sorting protein 1	224658_x_at	2	7.777	0.21	7	7.618	0.57	40	d	0.474837
			220557_s_at	2	4.683	0.42	7	5.069	0.61	40	u	0.122433
GLT8D1	2	glycosyltransferase 8 domain containing 1	218147_s_at	2	8.165	0.35	7	8.066	0.7	40	d	0.722505
			218146_at	2	9.531	0.26	7	9.061	0.67	40	d	0.078364
LRRTM2	2	leucine rich repeat transmembrane neuronal 2	206408_at	0	3.138	0.35	7	3.007	0.24	40	d	0.235715
OSBPL7	2	oxysterol binding protein-like 7	227946_at	2	4.885	0.31	7	3.997	0.77	40	d	0.005062
			208163_s_at	0	2.335	0.26	7	2.304	0.17	40	d	0.696242
			210344_at	0	2.408	0.05	7	2.422	0.11	40	u	0.737094
SKIV2L2	2	superkiller viralicidic activity 2-like 2 (S. cerevisiae)	212896_at	5	8.486	0.25	7	8.136	0.64	40	d	0.167048
			227447_at	3	9.037	0.26	7	8.499	0.63	40	d	0.034834
			1562142_at	0	3.476	0.38	7	3.776	0.47	40	u	0.121825
NAT5	2	N-acetyltransferase 5	223040_at	2	9.663	0.3	7	9.877	0.67	40	u	0.418529
RNF152	2	ring finger protein 152	1553722_s_at	2	2.608	0.56	7	2.663	0.33	40	u	0.820935
			1553721_at	2	2.718	0.07	7	3.018	0.29	40	u	0.000004
LRP8	2	low density lipoprotein receptor-related protein 8, apolipoprotein e receptor	205282_at	2	3.314	0.37	7	4.922	1.18	40	u	0
			208433_s_at	2	5.408	0.42	7	7.037	1.1	40	u	0.000469
TAGLN3	2	transgelin 3	204743_at	2	3.524	0.35	7	3.616	0.55	40	u	0.678242
AKR1D1	2	aldo-keto reductase family 1, member D1 (delta 4-3-ketosteroid-5-beta-reductase)	207102_at	2	4.075	2.21	7	2.671	0.21	40	d	0.171497
CLPTM1L	2	CLPTM1-like	223020_at	2	10.06	0.17	7	10.22	0.61	40	u	0.194465
			226935_s_at	2	9.268	0.1	7	9.401	0.62	40	u	0.224856
			229416_at	0	2.112	0.06	7	2.196	0.23	40	u	0.066811
UBOX5	2	U-box domain containing 5	215544_s_at	2	5.461	0.41	7	5.284	0.77	40	d	0.567538
			204598_at	2	5.763	0.28	7	5.416	0.46	40	d	0.06297
TOM1L1	2	target of myb1 (chicken)-like 1	204485_s_at	3	8.523	0.32	7	8.766	1.38	40	u	0.350112
			240261_at	0	5.601	0.5	7	6.194	1.11	40	u	0.180389
EIF4ENIF1	2	eukaryotic translation initiation factor 4E nuclear import factor 1	218626_at	2	7.38	0.35	7	7.086	0.76	40	d	0.332672
			242291_at	0	2.527	0.12	7	2.573	0.15	40	u	0.452531
VIP	2	vasoactive intestinal peptide	206577_at	2	2.941	0.2	7	3.066	0.22	40	u	0.172237
ARF5	2	ADP-ribosylation factor 5	201526_at	2	9.122	0.19	7	9.305	0.58	40	u	0.142048
RBM5	2	RNA binding motif protein 5	201395_at	2	9.826	0.31	7	8.579	0.73	40	d	0.000078
			201394_s_at	0	10.13	0.25	7	8.544	0.85	40	d	0
			209936_at	0	5.997	0.54	7	6.146	0.78	40	u	0.639824
TMEM85	2	transmembrane protein 85	223043_at	2	10.11	0.24	7	9.973	0.69	40	d	0.356798
			223857_x_at	2	10.33	0.22	7	10.16	0.59	40	d	0.478682
KIAA0922	2	KIAA0922	209760_at	1	6.198	0.32	7	6.402	0.7	40	u	0.459989
			235674_at	0	5.959	0.61	7	5.25	0.63	40	d	0.010057
PTH	2	parathyroid hormone	206977_at	2	2.601	0.14	7	2.647	0.1	40	u	0.287981
CRB3	2	crumbs homolog 3 (Drosophila)	232609_at	2	6.637	0.36	7	6.919	0.81	40	u	0.379377
METAP2	2	methionyl aminopeptidase 2	213899_at	2	6.773	0.42	7	6.908	0.7	40	u	0.632586
			209861_s_at	2	9.476	0.21	7	9.597	0.64	40	u	0.374737
			227993_at	0	6.916	0.33	7	6.632	0.83	40	d	0.387595
MAP3K4	2	mitogen-activated protein kinase kinase kinase 4	204089_x_at	2	8.343	0.28	7	7.824	0.7	40	d	0.064403
			216199_s_at	2	8.514	0.22	7	7.9	0.71	40	d	0.000211
CDC37	2	cell division cycle 37 homolog (S. cerevisiae)	209953_s_at	0	9.111	0.14	7	8.785	0.7	40	d	0.013659

SOX1	2	SRY (sex determining region Y)-box 1	230982_at	1	4.931	0.22	7	4.899	0.23	40	d	0.746164
			237472_at	1	2.87	0.25	7	2.859	0.23	40	d	0.913639
			208533_at	0	2.313	0.27	7	2.237	0.1	40	d	0.522254
INTS2	2	integrator complex subunit 2	224308_s_at	2	5.45	0.45	7	5.855	0.98	40	u	0.298986
ADORA2B	2	adenosine A2b receptor	205891_at	2	4.41	0.69	7	4.022	1.7	40	d	0.563849
NR2F6	2	nuclear receptor subfamily 2, group F, member 6	209261_s_at	2	5.399	0.28	7	6.08	1.27	40	u	0.005906
			209262_s_at	2	8.53	0.25	7	8.807	0.9	40	u	0.125655
			213354_s_at	0	2.054	0.08	7	2.086	0.08	40	u	0.38124
C20orf117	2	chromosome 20 open reading frame 117	207711_at	0	5.595	1.04	7	4.161	1.36	40	d	0.012366
			225473_at	0	7.298	0.66	7	6.806	1.1	40	d	0.266692
			215852_x_at	0	3.05	0.18	7	3.113	0.25	40	u	0.541554
			241362_at	0	3.393	0.12	7	3.549	0.31	40	u	0.201408
DDX17	2	DEAD (Asp-Glu-Ala-Asp) box polypeptide 17	208718_at	2	12.98	0.22	7	12.21	0.49	40	d	0.000253
			208151_x_at	1	8.19	1.23	7	7.789	1.13	40	d	0.409984
			213998_s_at	0	9.186	0.76	7	9.247	1.03	40	u	0.882654
			230180_at	0	8.769	0.75	7	9.058	1.17	40	u	0.540146
			208719_s_at	0	7.714	1.14	7	6.775	1.5	40	d	0.129307
CAMTA2	2	calmodulin binding transcription activator 2	212948_at	2	6.5	0.4	7	5.529	0.73	40	d	0.001551
FILIP1L	2	filamin A interacting protein 1-like	204135_at	2	9.07	0.74	7	6.934	1.23	40	d	0.000074
			1554965_at	0	2.429	0.08	7	2.525	0.25	40	u	0.067502
			1554966_a_at	0	8.734	0.73	7	6.651	1.21	40	d	0.000081
TMUB1	2	transmembrane and ubiquitin-like domain containing 1	223153_x_at	2	7.463	0.12	7	7.604	0.62	40	u	0.204244
CXCL10	2	chemokine (C-X-C motif) ligand 10	204533_at	2	5.159	0.57	7	9.357	2.32	40	u	0
CA8	2	carbonic anhydrase VIII	220234_at	0	5.351	0.74	7	4.828	1.72	40	d	0.443074
			1555445_at	0	2.326	0.05	7	2.389	0.07	40	u	0.023116
SLC35C1	2	solute carrier family 35, member C1	222647_at	2	6.623	0.37	7	6.956	0.72	40	u	0.246056
			218485_s_at	2	5.254	0.24	7	5.465	0.87	40	u	0.221403
ARG2	2	arginase, type II	203945_at	2	3.508	0.28	7	3.434	0.49	40	d	0.702517
			203946_s_at	2	5.054	1.34	7	5.146	1.16	40	u	0.854764
BRMS1L	2	breast cancer metastasis-suppressor 1-like	224484_s_at	2	5.019	0.24	7	5.636	0.71	40	u	0.000314
			226580_at	0	7.75	0.22	7	7.558	0.65	40	d	0.173993
ALKBH5	2	alkB, alkylation repair homolog 5 (E. coli)	234302_s_at	2	9.456	0.21	7	9.176	0.42	40	d	0.099165
			1553101_a_at	1	8.4	0.26	7	8.258	0.75	40	d	0.38906
			228034_x_at	0	3.251	0.43	7	3.189	0.41	40	d	0.719839
JUP	2	junction plakoglobin	201015_s_at	2	10.54	0.46	7	9.948	0.87	40	d	0.093089
EHD1	2	EH-domain containing 1	222221_x_at	2	7.168	0.23	7	7.214	0.43	40	u	0.790156
			209038_s_at	2	6.251	0.51	7	6.448	0.56	40	u	0.396151
			209039_x_at	2	7.433	0.3	7	7.405	0.51	40	d	0.89259
			208112_x_at	2	7.705	0.25	7	7.777	0.49	40	u	0.711592
			209037_s_at	2	6.187	0.48	7	6.716	0.78	40	u	0.096462
CDKN1A	2	cyclin-dependent kinase inhibitor 1A (p21, Cip1)	202284_s_at	2	9.116	0.81	7	8.34	0.79	40	d	0.02427
			1555186_at	0	2.218	0.02	7	2.292	0.13	40	u	0.00195
KCTD8	2	ssium channel tetramerisation domain containi	no probeset available									
SLC2A4RG	2	SLC2A4 regulator	1555500_s_at	5	2.95	0.45	7	3.65	0.87	40	u	0.046945
			227362_at	1	2.774	0.17	7	2.897	0.36	40	u	0.384161
			222650_s_at	1	6.958	0.35	7	7.162	1.13	40	u	0.384806
			218494_s_at	1	8.647	0.27	7	8.789	0.95	40	u	0.453578
ARPC5L	2	actin related protein 2/3 complex, subunit 5-like	220966_x_at	2	9.513	0.18	7	10.08	0.6	40	u	0.00006
			226914_at	2	7.552	0.36	7	8.17	0.59	40	u	0.012236
			226915_s_at	2	8.386	0.23	7	8.982	0.59	40	u	0.013118
			223101_s_at	0	7.418	0.26	7	8.129	0.7	40	u	0.013002
MBP	2	myelin basic protein	210136_at	2	8.085	0.77	7	7.514	1.47	40	d	0.330486
			1554544_a_at	0	7.341	0.6	7	7.034	1.13	40	d	0.496299
			207323_s_at	0	3.891	0.61	7	3.92	0.63	40	u	0.912625
			225407_at	0	7.417	0.42	7	6.623	1.31	40	d	0.006817
			236324_at	0	2.09	0.02	7	2.138	0.05	40	u	0.009173
			225408_at	0	5.191	0.28	7	5.282	0.83	40	u	0.608178
			228938_at	0	1.979	0.09	7	2.12	0.33	40	u	0.038977
			209072_at	0	2.598	0.36	7	2.501	0.58	40	d	0.675497
STAM2	2	signal transducing adaptor molecule (SH3 domain and ITAM motif) 2	209649_at	2	7.442	0.34	7	7.667	0.7	40	u	0.4212
			215044_s_at	0	5.414	0.52	7	6.385	0.62	40	u	0.000372
			208194_s_at	0	3.391	0.19	7	4.17	0.55	40	u	0.000001
			242569_at	0	6.178	0.76	7	5.999	0.86	40	d	0.614693

GPC3	2	glypican 3	209220_at	0	8.803	0.49	7	4.453	0.88	40	d	0
			243243_at	0	2.646	0.36	7	2.944	0.38	40	u	0.067396
C18orf19	2	chromosome 18 open reading frame 19	235022_at	2	5.745	0.46	7	6.753	1.06	40	u	0.019997
HELZ	2	helicase with zinc finger	203674_at	2	8.318	0.26	7	7.523	0.71	40	d	0.000033
			225910_at	0	8.072	0.24	7	7.232	0.63	40	d	0.001349
PLEKHJ1	2	pleckstrin homology domain containing, family J member 1	218290_at	2	7.567	0.21	7	7.711	0.57	40	u	0.523028
NFYB	2	nuclear transcription factor Y, beta	244704_at	2	4.201	0.39	7	4.167	0.57	40	d	0.882622
			218127_at	2	8.638	0.23	7	8.248	0.72	40	d	0.014406
			218128_at	2	7.298	0.28	7	7.172	0.7	40	d	0.648161
			218129_s_at	0	7.021	0.35	7	7.056	1.04	40	u	0.874438
SH3BGR	2	SH3 domain binding glutamic acid-rich protein	204979_s_at	2	6.782	0.45	7	6.232	1.55	40	d	0.085997
OVOL1	2	ovo-like 1(Drosophila)	229396_at	2	6.271	1.04	7	6.281	0.98	40	u	0.980215
			206604_at	1	2.85	0.55	7	2.882	0.61	40	u	0.897681
ASF1A	2	ASF1 anti-silencing function 1 homolog A (S. cerevisiae)	203428_s_at	2	7.194	0.37	7	8.168	1.32	40	u	0.000713
			213561_at	2	4.206	0.29	7	4.71	0.72	40	u	0.079327
			203427_at	1	8.184	0.24	7	8.514	0.93	40	u	0.071591
GNB1	2	guanine nucleotide binding protein (G protein), beta polypeptide 1	200745_s_at	2	10.59	0.32	7	10.8	0.72	40	u	0.454768
			200746_s_at	2	11.3	0.21	7	11.25	0.49	40	d	0.808251
			200744_s_at	2	8.774	0.41	7	9.431	0.86	40	u	0.058491
			1570108_at	0	3.496	0.49	7	3.653	0.6	40	u	0.526536
UPF3B	2	UPF3 regulator of nonsense transcripts homolog B (yeast)	218757_s_at	2	6.667	0.28	7	7.367	0.76	40	u	0.02259
C13orf34	2	chromosome 13 open reading frame 34	219544_at	1	5.358	0.38	7	6.956	0.96	40	u	0.0001
SPG3A	2	spastic paraplegia 3A (autosomal dominant)	223340_at	2	7.703	0.29	7	6.397	1.13	40	d	0.000001
SKIL	2	SKI-like oncogene	232379_at	2	2.039	0.02	7	2.109	0.1	40	u	0.00033
			206675_s_at	1	3.775	0.53	7	4.318	0.71	40	u	0.065325
			217591_at	0	6.434	0.45	7	6.629	0.71	40	u	0.495694
			215889_at	0	2.309	0.06	7	2.479	0.31	40	u	0.003912
POLR3F	2	polymerase (RNA) III (DNA directed) polypeptide F, 39 kDa	205218_at	2	5.569	0.34	7	5.894	0.61	40	u	0.187706
CNNM3	2	cyclin M3	220739_s_at	2	7.417	0.52	7	7.121	0.74	40	d	0.32753
RBM32A	2	RNA binding motif protein 32A	no probeset available									
HSF5	2	heat shock transcription factor family member 5	230718_at	2	2.389	0.05	7	2.481	0.24	40	u	0.043144
SUCLG2	2	succinate-CoA ligase, GDP-forming, beta subunit	214835_s_at	2	9.051	0.19	7	8.826	0.8	40	d	0.142789
			212459_x_at	2	9.225	0.21	7	8.722	0.75	40	d	0.001838
			215772_x_at	2	9.166	0.2	7	8.619	0.78	40	d	0.000756
COL11A1	2	collagen, type XI, alpha 1	37892_at	2	3.052	0.69	7	8.394	2.2	40	u	0
			204320_at	2	3.715	0.66	7	8.36	1.8	40	u	0
			229271_x_at	0	3.133	0.25	7	3.857	0.7	40	u	0.000084
UCP3	2	uncoupling protein 3 (mitochondrial, proton carrier)	219827_at	2	4.638	0.67	7	4.405	0.5	40	d	0.295454
			207349_s_at	0	2.396	0.12	7	2.583	0.27	40	u	0.081506
KCTD3	2	potassium channel tetramerisation domain containing 3	217894_at	1	8.906	0.42	7	8.774	0.84	40	d	0.691319
DOCK7	2	dedicator of cytokinesis 7	225384_at	2	8.221	0.33	7	8.293	0.93	40	u	0.724727
			237470_at	0	3.38	0.23	7	3.805	0.6	40	u	0.07558
KCNJ16	2	potassium inwardly-rectifying channel, subfamily J, member 16	219564_at	2	5.007	0.63	7	2.286	0.21	40	d	0.000044
			222901_s_at	0	2.624	0.04	7	2.675	0.14	40	u	0.062773
STMN2	2	stathmin-like 2	203000_at	2	3.235	0.59	7	3.251	0.89	40	u	0.965017
			203001_s_at	2	2.859	0.32	7	3.01	0.62	40	u	0.542687
TNPO2	2	transportin 2 (importin 3, karyopherin beta 2b)	226428_at	2	8.532	0.2	7	8.456	0.53	40	d	0.718873
			221507_at	1	8.494	0.22	7	8.464	0.69	40	d	0.834855
			221506_s_at	1	8.573	0.23	7	8.413	0.83	40	d	0.335955
			215844_at	0	6.444	0.44	7	5.888	0.47	40	d	0.006969
			210585_s_at	0	3.361	0.36	7	3.591	0.8	40	u	0.465255
SFXN5	2	sideroflexin 5	226373_at	2	6.687	0.39	7	6.641	0.67	40	d	0.863403
			241999_at	0	2.901	0.18	7	3.152	0.37	40	u	0.087889
			232691_at	0	3.343	0.25	7	3.487	0.28	40	u	0.222257
			1563708_at	0	3.931	0.38	7	4.019	0.5	40	u	0.666661
OMG	2	oligodendrocyte myelin glycoprotein	207093_s_at	2	2.579	0.05	7	2.796	0.32	40	u	0.000309
CALCOCO1	2	calcium binding and coiled-coil domain 1	209002_s_at	2	9.955	0.21	7	8.356	0.6	40	d	0

KTN1	2	kinectin 1 (kinesin receptor)	200915_x_at	2	11.23	0.39	7	10.89	0.55	40	d	0.133465
			200914_x_at	2	10.37	0.27	7	10.26	0.65	40	d	0.664361
			214709_s_at	2	10.94	0.41	7	10.75	0.59	40	d	0.442896
RELL2	2	RELT-like 2	1564031_a_at	6	4.748	0.5	7	5.594	0.64	40	u	0.002149
FKBP2	2	FK506 binding protein 2, 13kDa	203391_at	1	8.771	0.12	7	8.268	0.6	40	d	0.000027
XPO7	2	exportin 7	212166_at	2	8.716	0.19	7	8.099	0.68	40	d	0.000054
			208459_s_at	0	6.759	0.57	7	6.416	1.42	40	d	0.541632
NEK2	2	NIMA (never in mitosis gene a)-related kinase 2	204641_at	2	3.709	0.65	7	8.465	1.05	40	u	0
			211080_s_at	0	4.214	0.2	7	6.385	1.45	40	u	0
RRAGB	2	Ras-related GTP binding B	205540_s_at	0	4.088	0.31	7	3.816	0.52	40	d	0.193066
RCN2	2	reticulocalbin 2, EF-hand calcium binding domain	201486_at	2	9.964	0.28	7	10.57	0.61	40	u	0.014736
			201485_s_at	0	9.368	0.31	7	9.78	0.84	40	u	0.216684
ALPL	2	alkaline phosphatase, liver/bone/kidney	215783_s_at	1	6.535	0.65	7	4.627	0.63	40	d	0
			1557924_s_at	0	3.552	0.65	7	3.31	0.33	40	d	0.403148
ABLIM1	2	actin binding LIM protein 1	200965_s_at	2	10.75	0.12	7	8.789	1.19	40	d	0
			210461_s_at	2	7.826	0.31	7	7.395	1.08	40	d	0.053183
MAP3K9	2	mitogen-activated protein kinase kinase kinase 9	214969_at	2	3.229	0.34	7	3.351	0.54	40	u	0.574376
			213927_at	0	4.49	0.49	7	4.413	1.05	40	d	0.853166
RBM35B	2	RNA binding motif protein 35B	219395_at	1	7.994	0.44	7	7.71	1.03	40	d	0.486131
			225137_at	0	5.314	0.14	7	6.109	0.67	40	u	0
			225139_at	0	6.706	0.27	7	6.347	0.69	40	d	0.188481
			225141_at	0	7.502	0.15	7	7.767	0.52	40	u	0.016232
VEGFB	2	vascular endothelial growth factor B	203683_s_at	0	6.84	0.48	7	6.429	0.91	40	d	0.258646
MAEA	2	macrophage erythroblast attacher	207922_s_at	2	9.077	0.11	7	8.648	0.6	40	d	0.000186
CPSF2	2	cleavage and polyadenylation specific factor 2, 100kDa	225994_at	2	4.907	0.45	7	6.039	0.83	40	u	0.001176
			225986_x_at	2	7.876	0.33	7	8.375	0.54	40	u	0.025243
			233208_x_at	0	4.471	0.42	7	5.655	0.83	40	u	0.000798
SAFB	2	scaffold attachment factor B	213635_s_at	2	3.367	0.09	7	3.41	0.12	40	u	0.382014
			201747_s_at	2	4.851	0.63	7	4.08	0.85	40	d	0.029037
			201748_s_at	2	9.016	0.33	7	8.255	0.63	40	d	0.003754
MAPK8IP1	2	mitogen-activated protein kinase 8 interacting protein 1	213014_at	2	2.236	0.23	7	2.333	0.43	40	u	0.577123
			213013_at	2	6.561	0.55	7	6.176	0.84	40	d	0.257552
C19orf50	2	chromosome 19 open reading frame 50	200076_s_at	2	8.549	0.13	7	8.436	0.61	40	d	0.316428
LBR	2	lamin B receptor	201795_at	2	8.622	0.5	7	9.504	0.98	40	u	0.028128
PTGER3	2	prostaglandin E receptor 3 (subtype EP3)	210834_s_at	2	2.868	0.24	7	2.895	0.33	40	u	0.841316
			210831_s_at	0	5.394	0.52	7	4.676	0.61	40	d	0.006419
			213933_at	0	7.215	0.92	7	5.047	1.54	40	d	0.000938
			210374_x_at	0	5.673	0.83	7	5.28	0.79	40	d	0.241669
			210375_at	0	3.543	0.82	7	3.307	0.44	40	d	0.516705
			210832_x_at	0	3.382	0.59	7	3.064	0.73	40	d	0.289586
			211265_at	0	2.875	0.25	7	2.97	0.25	40	u	0.357847
			208169_s_at	0	3.096	0.35	7	3.15	0.18	40	u	0.726515
			210833_at	0	3.351	0.55	7	3.243	0.37	40	d	0.524654
			211909_x_at	0	8.872	0.35	7	8.288	0.49	40	d	0.00465
CHPT1	2	choline phosphotransferase 1	221675_s_at	2	10.38	0.47	7	9.366	1.33	40	d	0.001815
			230364_at	0	7.106	0.79	7	6.222	1.69	40	d	0.190772
			1559739_at	0	7.492	0.86	7	6.164	1.24	40	d	0.010867
BCL2L12	2	BCL2-like 12 (proline rich)	233110_s_at	0	6.256	0.31	7	6.626	0.74	40	u	0.207631
RAB6IP1	2	RAB6 interacting protein 1	212561_at	2	9.006	0.18	7	8.199	0.43	40	d	0.000016
EIF4EBP2	2	eukaryotic translation initiation factor 4E binding protein 2	208769_at	2	4.148	0.96	7	4.218	1.16	40	u	0.88309
			208770_s_at	2	9.324	0.22	7	9.005	0.56	40	d	0.15579
			224645_at	0	8.316	0.32	7	6.873	0.73	40	d	0.000008
			1570454_at	0	2.282	0.03	7	2.382	0.17	40	u	0.001827
			224653_at	0	8.868	0.22	7	8.119	0.61	40	d	0.00278
CXCL14	2	chemokine (C-X-C motif) ligand 14	222484_s_at	2	12.48	0.34	7	8.148	2.23	40	d	0
			218002_s_at	0	11.7	0.5	7	6.969	2.36	40	d	0
			237038_at	0	2.539	0.28	7	2.877	1.02	40	u	0.097408
MMP13	2	matrix metallopeptidase 13 (collagenase 3)	205959_at	2	4.451	0.39	7	6.402	1.37	40	u	0
KIAA0329	2	KIAA0329	204307_at	2	5.648	0.44	7	4.646	0.6	40	d	0.000157
			204308_s_at	2	6.223	0.36	7	4.688	0.77	40	d	0.000008
C16orf14	2	chromosome 16 open reading frame 14	1553715_s_at	2	6.814	0.33	7	7.835	1.05	40	u	0.000057
			225861_at	2	6.776	0.31	7	7.754	0.73	40	u	0.001338
			231516_at	0	5.579	0.55	7	5.297	0.57	40	d	0.242745

SLC25A37	2	solute carrier family 25, member 37	222528_s_at	2	7.777	0.68	7	6.836	1.56	40	d	0.13064
			222529_at	2	8.181	0.28	7	7.764	1.21	40	d	0.071176
			221920_s_at	2	7.97	0.59	7	6.622	1.14	40	d	0.004614
			226928_x_at	2	4.445	0.27	7	4.478	0.54	40	u	0.877909
			242335_at	2	4.013	0.51	7	4.21	1.23	40	u	0.683925
			218136_s_at	0	7.538	0.67	7	6.7	1.19	40	d	0.083502
			228527_s_at	0	2.625	0.25	7	2.474	0.78	40	d	0.353561
			231274_s_at	0	7.5	0.74	7	6.208	1.69	40	d	0.057176
			218978_s_at	0	6.377	0.48	7	5.778	0.68	40	d	0.03359
			226179_at	0	10.03	0.47	7	8.372	1.14	40	d	0.000542
FAM43B	2	family with sequence similarity 43, member B	1569256_a_at	2	2.584	0.31	7	2.44	0.08	40	d	0.299965
FHL3	2	four and a half LIM domains 3	218818_at	2	5.639	0.07	7	5.516	0.31	40	d	0.039066
CD4	2	CD4 molecule	203547_at	2	6.458	0.48	7	6.515	0.87	40	u	0.869332
			216424_at	0	2.587	0.34	7	2.556	0.31	40	d	0.818862
PTK2B	2	PTK2B protein tyrosine kinase 2 beta	203110_at	2	5.883	0.46	7	5.48	0.69	40	d	0.150689
			203111_s_at	1	4.576	0.62	7	4.56	0.63	40	d	0.951701
SERINC3	2	serine incorporator 3	221473_x_at	1	10.16	0.39	7	9.775	0.68	40	d	0.161483
			221472_at	0	9.571	0.15	7	8.987	0.53	40	d	0.000003
			211769_x_at	0	10.07	0.43	7	9.671	0.75	40	d	0.190964
			221471_at	0	9.308	0.29	7	9.13	0.6	40	d	0.452495
C11orf75	2	chromosome 11 open reading frame 75	219806_s_at	2	8.33	0.57	7	8.049	1.19	40	d	0.551822
TPP2	2	tripeptidyl peptidase II	203374_s_at	2	8.14	0.46	7	8.325	0.74	40	u	0.534582
			203375_s_at	2	8.932	0.16	7	8.163	0.7	40	d	0.000001
			1569857_s_at	0	4.067	0.31	7	4.737	0.99	40	u	0.002746
			1569856_at	0	2.54	0.07	7	2.689	0.27	40	u	0.006201
SLC39A14	2	solute carrier family 39 (zinc transporter), member 14	212110_at	2	8.343	0.32	7	8.357	1.09	40	u	0.950328
			1555433_at	0	2.063	0.02	7	2.156	0.15	40	u	0.001045
			1555434_a_at	0	3.505	0.56	7	4.347	1.24	40	u	0.091542
PSMA1	2	proteasome (prosome, macropain) subunit, alpha type, 1	211746_x_at	2	11.1	0.19	7	11.13	0.49	40	u	0.872083
			201676_x_at	2	10.85	0.17	7	10.9	0.5	40	u	0.68398
			210759_s_at	0	10.47	0.18	7	10.3	0.67	40	d	0.204184
MAP3K5	2	mitogen-activated protein kinase kinase kinase 5	203837_at	2	6.434	0.24	7	6.254	1.03	40	d	0.355615
			203836_s_at	1	7.104	0.19	7	6.743	1.32	40	d	0.115378
PRL	2	prolactin	205445_at	0	2.212	0.12	7	2.284	0.16	40	u	0.284446
FAM5B	2	family with sequence similarity 5, member B	214822_at	2	2.95	0.88	7	3.053	1.18	40	u	0.831093
DSCAML1	2	Down syndrome cell adhesion molecule like 1	232059_at	2	3.187	0.21	7	3.378	0.45	40	u	0.282112
			234908_s_at	0	2.297	0.13	7	2.301	0.06	40	u	0.942682
GLUD2	2	glutamate dehydrogenase 2	210447_at	2	2.204	0.05	7	2.577	0.83	40	u	0.008495
			215794_x_at	0	8.524	0.57	7	7.369	1.16	40	d	0.014973
LNPEP	2	leucyl/cystinyl aminopeptidase	207904_s_at	2	3.163	0.28	7	3.972	0.87	40	u	0.000115
			236728_at	0	2.89	0.38	7	2.996	0.37	40	u	0.503034
			231866_at	0	8.01	0.4	7	7.912	1.07	40	d	0.816008
ZNF513	2	zinc finger protein 513	225753_at	6	6.628	0.53	7	6.257	0.6	40	d	0.142879
PLP2	2	proteolipid protein 2 (colonic epithelium-enriched)	201136_at	2	9.531	0.46	7	9.74	0.87	40	u	0.543833
TAGLN2	2	transgelin 2	200916_at	2	9.878	0.39	7	10.66	0.79	40	u	0.016372
			210978_s_at	0	9.819	0.58	7	10.45	1.64	40	u	0.089622
THAP11	2	THAP domain containing 11	212910_at	2	7.892	0.19	7	7.788	0.51	40	d	0.36459
			226788_at	0	2.259	0.19	7	2.179	0.06	40	d	0.347185
ITGB4	2	integrin, beta 4	204989_s_at	2	7.093	0.54	7	5.563	1.71	40	d	0.00017
			214292_at	2	3.66	0.48	7	2.894	0.32	40	d	0.000004
			204990_s_at	2	9.273	0.33	7	7.77	1.05	40	d	0
			230704_s_at	0	2.428	0.06	7	2.454	0.13	40	u	0.629289
			211905_s_at	0	3.368	0.44	7	3.086	0.99	40	d	0.473027
UBE2I	2	ubiquitin-conjugating enzyme E2I (UBC9 homolog, yeast)	208760_at	2	8.04	0.6	7	6.464	1.09	40	d	0.000687
			213535_s_at	2	10.33	0.22	7	10.69	0.63	40	u	0.011712
			213536_s_at	2	2.76	0.15	7	3.486	0.59	40	u	0
			1558088_a_at	0	4.894	0.5	7	5.183	0.59	40	u	0.240195
			233360_at	0	3.5	0.49	7	3.679	0.52	40	u	0.409622
			211008_s_at	0	2.981	0.36	7	2.859	0.43	40	d	0.493135
ARL5B	2	ADP-ribosylation factor-like 5B	242727_at	2	3.281	0.12	7	4.963	1.32	40	u	0
SYT5	2	synaptotagmin V	206161_s_at	2	2.988	0.22	7	3.076	0.4	40	u	0.576037

[illegible]	2	synaptotagmin v	206162_x_at	2	4.11	0.28	7	4.277	0.38	40	u	0.284729
CNKSR3	2	CNKSR family member 3	227481_at	2	5.89	0.35	7	6.254	1.95	40	u	0.295711
			242394_at	1	2.934	0.39	7	3.435	1.1	40	u	0.045894
MGAT1	2	mannosyl (alpha-1,3-)-glycoprotein beta-1,2-N-acetylglucosaminyltransferase	201126_s_at	2	8.595	0.27	7	8.337	0.53	40	d	0.220501
C14orf121	2	chromosome 14 open reading frame 121	229561_at	2	7.724	0.25	7	7.499	0.39	40	d	0.156818
SLC35B2	2	solute carrier family 35, member B2	224716_at	2	9.156	0.29	7	9.39	0.81	40	u	0.193354
TNFRSF11B	2	tumor necrosis factor receptor superfamily, member 11b (osteoprotegerin)	204932_at	2	4.226	1.09	7	4.901	1.63	40	u	0.307389
			204933_s_at	0	4.587	1.23	7	4.992	2	40	u	0.61473
SUHW3	2	suppressor of hairy wing homolog 3 (Drosophila)	235520_at	2	3.044	0.17	7	3.315	0.54	40	u	0.019919
CLK2	2	CDC-like kinase 2	203229_s_at	2	8.447	0.25	7	8.148	0.53	40	d	0.157343
HMGN3	2	high mobility group nucleosomal binding domain 3	209377_s_at	2	11.07	0.15	7	10.67	0.79	40	d	0.007133
DHDDS	2	dehydrodolichyl diphosphate synthase	218547_at	2	7.203	0.28	7	6.913	0.5	40	d	0.151051
			224064_s_at	0	5.058	0.41	7	5.046	0.45	40	d	0.945154
USP10	2	ubiquitin specific peptidase 10	209136_s_at	3	6.197	0.49	7	7.421	1.03	40	u	0.004233
			209137_s_at	2	7.295	0.36	7	7.932	0.7	40	u	0.025978
SLC25A44	2	solute carrier family 25, member 44	32091_at	2	8.275	0.21	7	8.572	0.45	40	u	0.097575
			212683_at	2	7.396	0.22	7	7.607	0.46	40	u	0.251106
HMCN1	2	hemicentin 1	235944_at	2	6.838	0.66	7	5.563	1.12	40	d	0.006244
TCEAL8	2	transcription elongation factor A (SII)-like 8	224819_at	2	8.488	0.21	7	8.574	0.93	40	u	0.618315
PDXK	2	pyridoxal (pyridoxine, vitamin B6) kinase	218018_at	2	7.907	0.16	7	9.333	1.01	40	u	0
			218019_s_at	2	8.882	0.31	7	8.645	1.21	40	d	0.313352
			222492_at	2	6.32	0.19	7	7.226	1.03	40	u	0.00001
			202671_s_at	0	7.261	0.22	7	8.645	1.16	40	u	0
			1566671_a_at	0	3.205	0.46	7	3.408	0.59	40	u	0.4005
			1566670_at	0	4.249	0.26	7	4.132	0.33	40	d	0.393344
SCN4A	2	sodium channel, voltage-gated, type IV, alpha subunit	206981_at	2	2.716	0.75	7	2.315	0.2	40	d	0.239662
ECE2	2	endothelin converting enzyme 2	206728_at	2	2.966	0.82	7	2.691	0.31	40	d	0.449931
			227103_s_at	0	4.365	0.41	7	6.545	1.15	40	u	0
			1555306_a_at	0	4.686	0.57	7	4.411	0.47	40	d	0.187537
ERMP1	2	endoplasmic reticulum metallopeptidase 1	222603_at	2	8.187	0.8	7	8.397	1.15	40	u	0.651214
			218342_s_at	0	7.695	0.69	7	7.808	1.19	40	u	0.812811
UBQLNL	2	ubiquilin-like	236965_at	2	2.512	0.22	7	2.585	0.83	40	u	0.648727
ZNF641	2	zinc finger protein 641	235179_at	2	4.608	0.36	7	5.701	0.82	40	u	0.001414
			1554958_at	2	3.427	0.28	7	3.803	0.69	40	u	0.168562
			226509_at	0	7.735	0.28	7	7.697	0.62	40	d	0.874032
			229897_at	0	6.22	0.58	7	5.717	1	40	d	0.212013
LASS6	2	LAG1 homolog, ceramide synthase 6	212442_s_at	2	10.23	0.52	7	9.976	1.02	40	d	0.538938
			212446_s_at	2	8.992	0.32	7	9.095	1.07	40	u	0.632203
			242019_at	2	3.625	0.2	7	5.055	1.31	40	u	0
			235463_s_at	0	5.678	0.63	7	6.83	1.4	40	u	0.04172
HIRA	2	HIR histone cell cycle regulation defective homolog A (S. cerevisiae)	217427_s_at	2	6.092	0.65	7	7.096	1.13	40	u	0.030416
			1569560_at	0	2.851	0.2	7	2.849	0.16	40	d	0.986466
CCRN4L	2	CCR4 carbon catabolite repression 4-like (S. cerevisiae)	1554283_at	2	3.291	0.27	7	3.738	0.66	40	u	0.091376
			220671_at	0	2.988	0.28	7	3.187	0.37	40	u	0.195129
DULLARD	2	dullard homolog (Xenopus laevis)	200035_at	2	9.503	0.12	7	8.988	0.48	40	d	0.000002
SMYD5	2	SMYD family member 5	209516_at	2	6.679	0.27	7	6.622	0.66	40	d	0.825993
MASTL	2	microtubule associated serine/threonine kinase-like	228468_at	2	5.475	0.53	7	7.022	1.01	40	u	0.000341
ALDH1A2	2	aldehyde dehydrogenase 1 family, member A2	207016_s_at	2	5.533	1.41	7	2.436	0.36	40	d	0.001762
			207015_s_at	0	3.07	0.37	7	2.931	0.23	40	d	0.197273
POGK	2	pogo transposable element with KRAB domain	218229_s_at	2	9.22	0.3	7	9.689	0.89	40	u	0.019601
			222564_at	2	4.375	0.44	7	4.266	0.39	40	d	0.516952
CRYGEP1	2	crystallin, gamma E pseudogene 1	no probeset available									
FTL	2	ferritin, light polypeptide	212788_x_at	2	13.89	0.24	7	14.21	0.31	40	u	0.015986
			213187_x_at	0	12.78	0.5	7	13.1	0.62	40	u	0.21949
MTF1	2	metal-regulatory transcription factor 1	205323_s_at	2	7.182	0.24	7	7.304	0.56	40	u	0.582653
			205322_s_at	2	7.561	0.22	7	7.396	0.67	40	d	0.254291
			227150_at	0	7.128	0.28	7	6.832	0.68	40	d	0.272866
ASPH	2	aspartate beta-hydroxylase	209135_at	2	9.047	0.44	7	8.598	1.21	40	d	0.101474
			210896_s_at	2	9.628	0.62	7	8.912	1.53	40	d	0.237547

ASPH	2	aspartate beta-hydroxylase	205808_at	0	4.49	0.71	7	4.031	1.49	40	d	0.43723
			207284_s_at	0	3.165	0.38	7	3.751	0.92	40	u	0.1131
LYRM5	2	LYR motif containing 5	225469_at	2	8.967	0.36	7	8.504	0.69	40	d	0.099036
			231640_at	0	2.879	0.69	7	2.946	0.59	40	u	0.793027
ABCA8	2	ATP-binding cassette, sub-family A (ABC1), member 8	204719_at	2	9.321	1.03	7	4.068	1.07	40	d	0
			1565780_at	0	2.043	0.05	7	2.052	0.06	40	u	0.714516
			1565778_at	0	2.937	0.41	7	2.841	0.28	40	d	0.460226
ILK	2	integrin-linked kinase	201234_at	2	9.775	0.12	7	8.911	0.59	40	d	0
			235135_at	0	3.412	0.28	7	3.413	0.39	40	u	0.995855
			235906_at	0	2.788	0.35	7	2.744	0.25	40	d	0.697068
SERPINB2	2	serpin peptidase inhibitor, clade B (ovalbumin), member 2	204614_at	2	2.471	0.09	7	3.091	1.45	40	u	0.01157
DYNLL2	2	dynein, light chain, LC8-type 2	229106_at	2	4.944	0.38	7	5.216	1.05	40	u	0.246345
CAMKK1	2	calcium/calmodulin-dependent protein kinase kinase 1, alpha	223460_at	2	4.727	0.46	7	4.222	0.63	40	d	0.052754
			1552768_at	0	2.222	0.11	7	2.27	0.14	40	u	0.406851
SLC25A13	2	solute carrier family 25, member 13 (citrin)	203775_at	2	6.805	0.35	7	7.077	0.65	40	u	0.296462
			229061_s_at	0	5.717	0.34	7	6.273	0.64	40	u	0.033251
			229081_at	0	3.604	0.26	7	3.697	0.32	40	u	0.480122
GPR126	2	G protein-coupled receptor 126	213094_at	2	5.508	0.26	7	6.232	1.68	40	u	0.016011
			233887_at	0	2.683	0.17	7	3.3	0.9	40	u	0.000373
			1553025_at	0	2.223	0.04	7	2.333	0.17	40	u	0.001092
DLG4	2	discs, large homolog 4 (Drosophila)	204592_at	2	4.284	0.43	7	4.372	0.59	40	u	0.716096
			210684_s_at	0	4.159	0.38	7	3.86	0.55	40	d	0.180694
SPOCK3	2	sparc/osteonectin, cwcv and kazal-like domains proteoglycan (testican) 3	235342_at	2	3.043	0.41	7	3.171	0.37	40	u	0.416632
			206434_at	1	3.133	0.13	7	3.298	0.21	40	u	0.05395
			1554418_s_at	0	2.393	0.03	7	2.502	0.14	40	u	0.000164
			206433_s_at	0	2.734	0.07	7	2.826	0.15	40	u	0.117497
GARNL4	2	GTPase activating Rap/RanGAP domain-like 4	213280_at	2	6.365	0.57	7	5.987	1.21	40	d	0.432195
EFNA1	2	ephrin-A1	202023_at	2	9.813	0.59	7	10.02	0.96	40	u	0.598397
VIM	2	vimentin	1555938_x_at	2	5.86	0.76	7	5.669	1.31	40	d	0.716107
			201426_s_at	2	13.74	0.25	7	12.93	0.82	40	d	0.00003
MEPCE	2	methylphosphate capping enzyme	219798_s_at	0	8.731	0.38	7	8.167	0.58	40	d	0.019704
RRAGA	2	Ras-related GTP binding A	201628_s_at	2	10.56	0.14	7	9.786	0.73	40	d	0
ABCE1	2	ATP-binding cassette, sub-family E (OABP), member 1	201872_s_at	2	8.647	0.38	7	9.452	0.73	40	u	0.007826
			201873_s_at	2	7.563	0.4	7	8.087	0.55	40	u	0.022312
KIAA1244	2	KIAA1244	231856_at	2	7.92	1	7	7.397	1.66	40	d	0.432956
			227479_at	0	6.131	0.17	7	6.374	0.68	40	u	0.065555
PEX13	2	peroxisome biogenesis factor 13	1556009_at	2	5.176	0.49	7	5.993	0.88	40	u	0.022987
			205246_at	2	6.356	0.44	7	6.948	0.87	40	u	0.090183
			1558163_at	0	2.937	0.08	7	3.328	0.61	40	u	0.000418
			1558164_s_at	0	2.784	0.37	7	3.84	1.41	40	u	0.000414
SCD	2	stearoyl-CoA desaturase (delta-9-desaturase)	200832_s_at	2	10.47	1.32	7	10.37	1.31	40	d	0.856725
			200831_s_at	2	9.565	1.37	7	8.838	1.12	40	d	0.142985
			211708_s_at	0	5.471	1.99	7	5.32	2.06	40	d	0.861372
			211162_x_at	0	5.318	1.84	7	5.191	1.78	40	d	0.866021
LIN7A	2	lin-7 homolog A (C. elegans)	206440_at	2	2.886	0.2	7	3.131	1.2	40	u	0.246897
			240027_at	0	3.144	0.39	7	3.288	1.06	40	u	0.728068
			241652_x_at	0	2.949	0.08	7	3.404	1.03	40	u	0.009439
MPZL1	2	myelin protein zero-like 1	201875_s_at	2	9.562	0.36	7	9.066	0.73	40	d	0.090151
			201874_at	2	10	0.42	7	9.427	0.79	40	d	0.072189
			210594_x_at	0	6.967	0.8	7	7.159	1.05	40	u	0.652114
			210087_s_at	0	7.531	0.69	7	7.885	1.24	40	u	0.474654
			210210_at	0	7.696	0.5	7	7.192	0.61	40	d	0.050611
TMUB2	2	transmembrane and ubiquitin-like domain containing 2	218419_s_at	2	7.754	0.23	7	7.392	0.51	40	d	0.078635
APAF1	2	apoptotic peptidase activating factor 1	204859_s_at	2	6.69	0.34	7	6.923	0.63	40	u	0.357461
			211553_x_at	0	2.474	0.04	7	2.637	0.21	40	u	0.000066
			211554_s_at	0	2.128	0.13	7	2.513	0.51	40	u	0.000353
ZNF289	2	zinc finger protein 289, ID1 regulated	211975_at	2	9.369	0.19	7	8.778	0.71	40	d	0.000134
CAP1	2	CAP, adenylate cyclase-associated protein 1 (yeast)	200625_s_at	2	11.07	0.24	7	11.27	0.49	40	u	0.303119
			213798_s_at	0	11.14	0.27	7	11.4	0.6	40	u	0.27417
			223458_at	2	6.044	0.5	7	6.087	1.01	40	u	0.913691
			233337_s_at	2	6.555	0.79	7	5.87	1.83	40	d	0.346826

SEZ6L2	2	seizure related 6 homolog (mouse)-like 2	218720_x_at	2	5.713	1.04	7	5.219	1.76	40	d	0.484586
			238406_x_at	0	6.598	0.37	7	6.245	0.74	40	d	0.234666
			238404_x_at	0	6.7	0.13	7	6.512	0.44	40	d	0.042338
INSM2	2	insulinoma-associated 2	240841_at	2	2.637	0.24	7	2.623	0.2	40	d	0.874462
SHPRH	2	SNF2 histone linker PHD RING helicase	226366_at	2	7.188	0.24	7	6.391	0.98	40	d	0.000118
ATXN3	2	ataxin 3	205415_s_at	2	7.048	0.18	7	7.441	0.68	40	u	0.004553
			205416_s_at	2	4.787	0.48	7	5.679	0.82	40	u	0.008557
			235240_at	0	6.152	0.29	7	5.519	0.53	40	d	0.004272
			238723_at	0	4.805	0.37	7	4.37	0.48	40	d	0.030563
			233182_x_at	0	5.008	0.35	7	4.988	0.4	40	d	0.904251
			217321_x_at	0	3.153	0.39	7	3.262	0.32	40	u	0.434598
			216657_at	0	3.415	0.48	7	3.421	0.32	40	u	0.970537
SIPA1L1	2	signal-induced proliferation-associated 1 like 1	202254_at	2	6.922	0.47	7	4.859	0.94	40	d	0.000001
			202255_s_at	2	7.073	0.23	7	6.84	0.53	40	d	0.269164
IER5L	2	immediate early response 5-like	226552_at	2	6.433	0.47	7	6.965	1.04	40	u	0.199483
			226559_at	2	8.022	0.27	7	8.133	0.64	40	u	0.661038
ATP1B2	2	ATPase, Na+/K+ transporting, beta 2 polypeptide	204311_at	1	3.084	0.57	7	3.141	0.55	40	u	0.810297
ANKRD54	2	ankyrin repeat domain 54	231847_at	2	7.116	0.43	7	7.424	0.53	40	u	0.162862
MADD	2	MAP-kinase activating death domain	210252_s_at	2	7.492	0.26	7	6.971	0.77	40	d	0.003717
			38398_at	2	7.19	0.41	7	6.844	0.64	40	d	0.186319
ARRB1	2	arrestin, beta 1	222912_at	2	7.056	0.77	7	5.531	1.16	40	d	0.001982
			222756_s_at	2	7.724	0.25	7	7.136	0.56	40	d	0.010389
			218832_x_at	0	5.54	0.66	7	3.81	1.39	40	d	0.002838
LRRC15	2	leucine rich repeat containing 15	213909_at	2	4.199	0.91	7	6.932	1.92	40	u	0.000742
			1552960_at	0	2.87	0.3	7	2.941	0.36	40	u	0.6329
USP45	2	ubiquitin specific peptidase 45	238057_at	2	6.344	0.43	7	6.566	0.8	40	u	0.484104
			224441_s_at	0	3.05	0.23	7	3.733	0.88	40	u	0.000282
FRAP1	2	FK506 binding protein 12-rapamycin associated protein 1	202288_at	2	6.065	0.33	7	5.678	0.55	40	d	0.081237
			215381_at	0	2.81	0.37	7	2.778	0.35	40	d	0.832111
TNS1	2	tensin 1	218864_at	2	4.024	0.64	7	3.487	0.59	40	d	0.037086
			221748_s_at	0	10.89	0.46	7	8.08	1.19	40	d	0
			221246_x_at	0	8.291	0.49	7	6.596	0.69	40	d	0
			221747_at	0	9.349	0.53	7	6.826	0.73	40	d	0
			218863_s_at	0	6.103	0.31	7	5.754	0.33	40	d	0.013411
			232750_at	0	3.536	0.18	7	3.621	0.65	40	u	0.511383
MDM1	2	Mdm4, transformed 3T3 cell double minute 1, p53 binding protein (mouse)	220397_at	2	3.103	0.25	7	3.453	0.78	40	u	0.038185
			213761_at	0	6.926	0.54	7	6.99	0.98	40	u	0.870381
RBL2	2	retinoblastoma-like 2 (p130)	212331_at	2	9.739	0.19	7	9.153	0.59	40	d	0.00006
			212332_at	2	7.379	0.32	7	7.585	0.75	40	u	0.488122
LCA5	2	Leber congenital amaurosis 5	242006_at	2	4.383	0.66	7	3.386	0.54	40	d	0.000111
			229953_x_at	2	3.788	0.52	7	3.567	0.45	40	d	0.260023
			244401_at	0	5.541	0.45	7	5.558	0.7	40	u	0.952289
TMED2	2	transmembrane emp24 domain trafficking protein 2	200087_s_at	1	12.37	0.13	7	12.62	0.47	40	u	0.00793
			204426_at	0	9.31	0.44	7	9.607	1.25	40	u	0.279241
			204427_s_at	0	10.23	0.43	7	10.36	1.38	40	u	0.643943
LYPLA2	2	lysophospholipase II	202292_x_at	2	7.702	0.23	7	8.032	0.48	40	u	0.08856
			215568_x_at	2	5.661	0.23	7	5.725	0.48	40	u	0.738177
			215566_x_at	2	7.357	0.23	7	7.548	0.59	40	u	0.409638
SMAD4	2	SMAD family member 4	202527_s_at	2	8.181	0.34	7	7.984	1.02	40	d	0.365088
			202526_at	2	3.137	0.29	7	3.267	0.57	40	u	0.571579
			1565702_at	0	2.236	0.21	7	2.333	0.53	40	u	0.640906
			1565703_at	0	6.11	0.77	7	5.631	1.16	40	d	0.310155
C9orf58	2	chromosome 9 open reading frame 58	223074_s_at	2	5.772	0.51	7	5.66	1.12	40	d	0.801647
			223075_s_at	2	9.239	0.56	7	8.364	1.6	40	d	0.017589
GOSR1	2	golgi SNAP receptor complex member 1	213020_at	2	7.128	0.41	7	7.015	0.61	40	d	0.644505
			213021_at	2	7.224	0.18	7	7.48	0.88	40	u	0.113817
			204630_s_at	0	9.071	0.26	7	8.813	0.69	40	d	0.344588
			1555199_at	0	3.019	0.22	7	3.083	0.31	40	u	0.603359
TMEM63B	2	transmembrane protein 63B	225271_at	2	7.766	0.27	7	8.157	0.53	40	u	0.068694
ELMOD2	2	ELMO/CED-12 domain containing 2	1553928_at	2	5.829	0.59	7	6.874	0.93	40	u	0.007354
			1557837_a_at	0	2.775	0.08	7	2.98	0.34	40	u	0.002641
			226502_at	0	8.416	0.35	7	8.358	0.62	40	d	0.812017
			1557836_at	0	3.846	0.29	7	4.157	0.5	40	u	0.127393

GTF3C2	2	general transcription factor IIIC, polypeptide 2, beta 110kDa	212429_s_at	2	8.852	0.34	7	8.94	0.57	40	u	0.698018
			204366_s_at	2	8.841	0.31	7	8.972	0.56	40	u	0.560346
			210620_s_at	0	5.551	0.4	7	5.683	1.12	40	u	0.591286
ACHE	2	acetylcholinesterase (Yt blood group)	205378_s_at	2	2.301	0.15	7	2.305	0.08	40	u	0.951816
			205377_s_at	2	2.179	0.07	7	2.357	0.32	40	u	0.004841
TNFRSF12A	2	tumor necrosis factor receptor superfamily, member 12A	218368_s_at	2	8.402	1.03	7	8.365	1.01	40	d	0.931442
PLAA	2	phospholipase A2-activating protein	209532_at	2	4.185	0.46	7	4.585	0.82	40	u	0.227074
			209533_s_at	2	6.502	0.23	7	7.037	1.06	40	u	0.008415
PPP1R3A	2	protein phosphatase 1, regulatory (inhibitor) subunit 3A (glycogen and sarcoplasmic	206895_at	2	1.988	0.07	7	2.063	0.17	40	u	0.273235
			211169_s_at	0	2.503	0.04	7	2.635	0.18	40	u	0.000381
TSPAN5	2	tetraspanin 5	225388_at	2	7.177	0.57	7	6.144	1.43	40	d	0.072233
			225387_at	2	8.364	0.6	7	6.708	1.33	40	d	0.002837
			209890_at	2	7.81	0.68	7	6.699	1.34	40	d	0.042007
AMIGO1	2	adhesion molecule with Ig-like domain 1	226718_at	2	5.755	0.38	7	5.083	0.85	40	d	0.049721
MTMR9	2	myotubularin related protein 9	213278_at	2	7.53	0.3	7	6.37	0.74	40	d	0.000248
			204837_at	0	7.907	0.16	7	6.865	1.01	40	d	0
			233101_at	0	7.19	0.25	7	6.923	0.44	40	d	0.133227
ZNF335	2	zinc finger protein 335	221890_at	2	6.925	0.35	7	5.883	0.85	40	d	0.003033
			78330_at	0	2.432	0.19	7	2.475	0.25	40	u	0.67457
			222059_at	0	3.118	0.31	7	2.972	0.33	40	d	0.294342
CPT2	2	carnitine palmitoyltransferase II	204263_s_at	0	7.232	0.35	7	7.287	0.47	40	u	0.772853
			204264_at	0	6.248	0.36	7	6.276	0.51	40	u	0.893351
KLHL32	2	kelch-like 32 (Drosophila)	1553765_a_at	2	3.116	0.24	7	3.264	0.29	40	u	0.211629
ZMYM3	2	zinc finger, MYM-type 3	207559_s_at	2	7.767	0.43	7	7.376	0.68	40	d	0.157553
			1554172_a_at	0	3.84	0.44	7	4.161	0.6	40	u	0.191254
			1554171_at	0	6.318	0.44	7	6.066	0.49	40	d	0.21764
FAM120C	2	family with sequence similarity 120C	220685_at	0	2.434	0.38	7	2.736	0.56	40	u	0.188505
ASB15	2	ankyrin repeat and SOCS box-containing 15	1564679_at	0	2.463	0.18	7	2.484	0.2	40	u	0.796696
YWHAB	2	tyrosine 3-monooxygenase/tryptophan 5-monooxygenase activation protein, beta polypeptide	217717_s_at	2	10.16	0.37	7	11.1	0.63	40	u	0.000458
			217718_s_at	2	12.39	0.17	7	12.65	0.4	40	u	0.101154
			208743_s_at	0	10.52	0.35	7	11.31	0.55	40	u	0.000773
ENTPD5	2	ectonucleoside triphosphate diphosphohydrolase 5	205757_at	2	6.754	1.24	7	5.449	0.96	40	d	0.00323
			1554094_at	0	2.783	0.98	7	2.481	0.26	40	d	0.481071
CUL1	2	cullin 1	228899_at	0	4.348	0.5	7	5.162	1.15	40	u	0.077989
			238509_at	0	6.011	0.44	7	5.592	0.49	40	d	0.044044
			207614_s_at	0	8.18	0.12	7	8.318	0.59	40	u	0.199504
PDIA5	2	protein disulfide isomerase family A, member 5	203857_s_at	2	8.056	0.49	7	8.251	0.79	40	u	0.53871
SLC7A2	2	solute carrier family 7 (cationic amino acid transporter, y+ system), member 2	225516_at	2	9.334	0.77	7	6.065	2.8	40	d	0.000001
			207626_s_at	0	2.208	0.25	7	2.463	0.74	40	u	0.117254
			230658_at	0	2.573	0.08	7	2.654	0.19	40	u	0.266693
KCTD20	2	potassium channel tetramerisation domain containing 20	223176_at	2	7.531	0.36	7	8.194	1.12	40	u	0.00835
			214849_at	0	4.009	0.58	7	5.507	1.44	40	u	0.010731
			228299_at	0	9.004	0.17	7	9.307	0.51	40	u	0.009792
DALRD3	2	DALR anticodon binding domain containing 3	218808_at	1	6.086	0.15	7	5.636	0.66	40	d	0.000647
			221934_s_at	1	8.682	0.21	7	7.873	1.07	40	d	0.000119
IRF4	2	interferon regulatory factor 4	204562_at	2	5.102	0.66	7	5.22	1.01	40	u	0.771527
			216986_s_at	0	2.255	0.07	7	2.341	0.13	40	u	0.107056
			216987_at	0	2.684	0.06	7	2.838	0.28	40	u	0.003776
L1CAM	2	L1 cell adhesion molecule	204584_at	2	4.655	0.54	7	5.493	1.51	40	u	0.017457
			204585_s_at	2	2.46	0.17	7	2.623	0.39	40	u	0.291404
FASLG	2	Fas ligand (TNF superfamily, member 6)	210865_at	2	2.418	0.05	7	2.488	0.13	40	u	0.189261
			211333_s_at	0	2.194	0.05	7	2.306	0.2	40	u	0.005377
PTMA	2	prothymosin, alpha (gene sequence 28)	200773_x_at	2	13.1	0.11	7	13.47	0.22	40	u	0.000094
			211921_x_at	0	13.1	0.22	7	13.91	0.49	40	u	0.000101
			216515_x_at	0	12.36	0.12	7	12.78	0.27	40	u	0.000258
			200772_x_at	0	13.15	0.25	7	13.84	0.46	40	u	0.000373
			208549_x_at	0	10.94	0.24	7	11.77	0.45	40	u	0.000028
ARHGEF11	2	Rho guanine nucleotide exchange factor (GEF) 11	202913_at	2	7.133	0.28	7	7.356	0.48	40	u	0.247893
			202914_s_at	1	5.414	0.49	7	5.707	1.11	40	u	0.507058
			238730_at	0	2.057	0.08	7	2.113	0.11	40	u	0.204083
			201362_at	2	8.772	0.31	7	8.732	0.62	40	d	0.870205

IVNS1ABP	2	influenza virus NS1A binding protein	201363_s_at	2	8.676	0.27	7	8.905	0.56	40	u	0.308594
			206245_s_at	1	9.745	0.3	7	10.27	0.63	40	u	0.039555
			244235_at	0	3.411	0.3	7	3.815	0.41	40	u	0.018197
			239152_at	0	3.078	0.31	7	3.329	0.51	40	u	0.227399
CD163	2	CD163 molecule	215049_x_at	0	7.37	1.21	7	7.398	1.12	40	u	0.95422
			216233_at	0	2.636	0.13	7	2.883	0.29	40	u	0.036412
			203645_s_at	0	7.82	1.19	7	7.855	1.06	40	u	0.938637
PRKAR2B	2	protein kinase, cAMP-dependent, regulatory, type II, beta	203680_at	1	8.779	0.72	7	8.289	1.52	40	d	0.417176
DNAJC14	2	DnaJ (Hsp40) homolog, subfamily C, member 14	223420_at	3	7.437	0.35	7	7.102	0.91	40	d	0.350696
			1554451_s_at	2	5.962	0.63	7	5.632	1.24	40	d	0.505084
			224914_s_at	0	8.646	0.29	7	8.736	0.61	40	u	0.713494
HNRPUL1	2	heterogeneous nuclear ribonucleoprotein U-like 1	208713_at	2	9.299	0.34	7	9.316	0.56	40	u	0.938719
			209675_s_at	1	8.709	0.4	7	8.716	0.75	40	u	0.983381
INSIG2	2	insulin induced gene 2	209566_at	2	8.49	0.58	7	8.103	0.76	40	d	0.213531
SEMA5B	2	sema domain, seven thrombospondin repeats (type 1 and type 1-like), transmembrane domain (TM) and short cytoplasmic domain, (semaphorin) 5B	223610_at	2	5.082	0.32	7	5.758	0.61	40	u	0.007313
TGFB3	2	transforming growth factor, beta 3	209747_at	2	7.928	0.52	7	6.88	1.18	40	d	0.029024
			1555540_at	0	2.753	0.03	7	2.734	0.2	40	d	0.580565
KLF7	2	Kruppel-like factor 7 (ubiquitous)	1555420_a_at	2	6.009	0.45	7	6.095	1.35	40	u	0.764866
			204334_at	2	7.375	0.51	7	6.966	1.04	40	d	0.324522
			238482_at	0	2.734	0.36	7	3.162	0.67	40	u	0.113488
SERPINE2	2	serpin peptidase inhibitor, clade E (nexin, plasminogen activator inhibitor type 1),	212190_at	2	8.359	0.69	7	8.819	1.68	40	u	0.487727
			227487_s_at	0	2.234	0.04	7	2.407	0.48	40	u	0.033808
GCC1	2	GRIP and coiled-coil domain containing 1	218912_at	2	7.337	0.29	7	7.217	0.7	40	d	0.664115
			243306_s_at	0	4.643	0.28	7	4.483	0.58	40	d	0.484599
			243437_at	0	2.312	0.04	7	2.371	0.12	40	u	0.017359
P2RY5	2	purinergic receptor P2Y, G-protein coupled, 5	218589_at	0	9.843	0.45	7	8.197	1.05	40	d	0.000231
			1557763_at	0	2.556	0.19	7	2.73	0.36	40	u	0.235109
SYT6	2	synaptotagmin VI	244227_at	1	3.434	0.29	7	3.526	0.54	40	u	0.668296
DGKI	2	diacylglycerol kinase, iota	206806_at	2	3.096	0.81	7	3.056	0.51	40	d	0.867019
FAM136A	2	family with sequence similarity 136, member A	215947_s_at	2	8.17	0.23	7	8.791	0.63	40	u	0.015659
TPP1	2	tripeptidyl peptidase I	200742_s_at	2	9.186	0.17	7	8.335	0.87	40	d	0.000002
			200743_s_at	2	9.005	0.23	7	8.376	0.73	40	d	0.000213
			214196_s_at	0	7.031	0.33	7	6.507	0.97	40	d	0.01731
			214195_at	0	4.564	0.42	7	4.624	0.47	40	u	0.758733
ANKFN1	2	ankyrin-repeat and fibronectin type III domain containing 1	1553211_at	2	2.243	0.03	7	2.295	0.06	40	u	0.035257
			1559640_at	0	2.281	0.03	7	2.34	0.08	40	u	0.063989
NME7	2	non-metastatic cells 7, protein expressed in (nucleoside-diphosphate kinase)	219553_at	9	6.903	0.19	7	7.382	0.87	40	u	0.004401
			227556_at	7	10.68	0.74	7	10.18	1.35	40	d	0.361772
LHX3	2	LIM homeobox 3	221670_s_at	2	3.527	0.25	7	3.922	0.45	40	u	0.033163
PIK3CA	2	phosphoinositide-3-kinase, catalytic, alpha polypeptide	231854_at	0	3.856	0.29	7	3.643	0.39	40	d	0.184443
			204369_at	0	7.914	0.35	7	7.699	0.83	40	d	0.511224
			235980_at	0	5.602	0.49	7	5.371	0.55	40	d	0.310106
TNPO3	2	transportin 3	212317_at	1	6.535	0.32	7	7.242	0.75	40	u	0.020401
			212318_at	1	8.717	0.29	7	9.032	0.63	40	u	0.210331
			214550_s_at	1	7.458	0.27	7	8.042	0.62	40	u	0.021326
RAB3D	2	RAB3D, member RAS oncogene family	225001_at	2	8.29	0.32	7	8.157	0.97	40	d	0.519551
			208466_at	0	4.255	0.57	7	4.237	0.8	40	d	0.955152
TBR1	2	T-box, brain, 1	220025_at	2	2.053	0.07	7	2.134	0.1	40	u	0.042732
PMP2	2	peripheral myelin protein 2	206826_at	2	3.265	0.13	7	3.523	0.48	40	u	0.008592
			235127_at	2	2.857	0.08	7	2.982	0.23	40	u	0.017729
TBC1D1	2	TBC1 (tre-2/USP6, BUB2, cdc16) domain family, member 1	227945_at	2	5.303	0.6	7	4.673	1.03	40	d	0.129552
			212350_at	0	7.837	0.29	7	8.407	0.81	40	u	0.003364
			1568713_a_at	0	2.961	0.23	7	3.069	0.37	40	u	0.467071
			214013_s_at	0	2.097	0.04	7	2.134	0.07	40	u	0.170017
			1569566_at	0	2.483	0.16	7	2.559	0.29	40	u	0.506865
EMD	2	emerin (Emery-Dreifuss muscular dystrophy)	209477_at	2	7.539	0.14	7	7.391	0.6	40	d	0.196982
TUBB6	2	tubulin, beta 6	209191_at	2	10.11	0.49	7	9.436	1.1	40	d	0.126326
			209448_at	2	8.181	0.42	7	9.254	0.7	40	u	0.000391

HTATIP2	2	HIV-1 Tat interactive protein 2, 30kDa	229102_at	0	2.005	0.02	7	2.093	0.18	40	u	0.004193
			210253_at	0	5.117	0.6	7	5.668	1.05	40	u	0.191027
			207180_s_at	0	6.726	0.49	7	7.658	1.04	40	u	0.027486
SACM1L	2	SAC1 suppressor of actin mutations 1-like (yeast)	202797_at	2	9.423	0.14	7	9.255	0.63	40	d	0.158734
ZNF784	2	zinc finger protein 784	228364_at	2	4.549	0.7	7	4.365	0.58	40	d	0.468715
CX3CL1	2	chemokine (C-X3-C motif) ligand 1	823_at	2	10.19	0.69	7	6.954	1.32	40	d	0
			203687_at	2	9.541	0.62	7	6.3	1.4	40	d	0
LDB2	2	LIM domain binding 2	206481_s_at	2	8.8	0.47	7	6.175	0.92	40	d	0
			242360_at	0	2.122	0.03	7	2.212	0.18	40	u	0.008274
IPO7	2	importin 7	200993_at	0	9.533	0.23	7	9.561	0.65	40	u	0.843601
			200992_at	0	9.615	0.28	7	9.852	0.64	40	u	0.348783
			200994_at	0	9.226	0.26	7	9.466	0.56	40	u	0.277315
			200995_at	0	7.605	0.22	7	7.754	0.61	40	u	0.271636
CEBPG	2	CCAAT/enhancer binding protein (C/EBP), gamma	225527_at	2	7.823	0.35	7	8.208	0.75	40	u	0.198913
			204203_at	0	6.814	0.74	7	7.972	1.07	40	u	0.010166
GALNTL1	2	UDP-N-acetyl-alpha-D-galactosamine:polypeptide N-acetylgalactosaminyltransferase-like 1	230417_at	2	5.981	0.72	7	4.675	0.99	40	d	0.002141
			230418_s_at	2	5.965	1.01	7	2.865	1.73	40	d	0.000047
			232548_at	2	3.8	0.15	7	3.748	0.46	40	d	0.597757
BAG2	2	BCL2-associated athanogene 2	230879_at	0	3.157	0.32	7	3.828	0.69	40	u	0.017086
			209406_at	0	4.473	0.93	7	5.752	1.84	40	u	0.085579
TUBG1	2	tubulin, gamma 1	201714_at	2	7.195	0.14	7	8.092	0.8	40	u	0
IRX2	2	iroquois homeobox 2	228462_at	0	10.2	0.42	7	7.563	2.11	40	d	0
			228404_at	0	4.208	0.43	7	4.056	0.7	40	d	0.589716
ELOF1	2	elongation factor 1 homolog (S. cerevisiae)	225156_at	2	8.307	0.14	7	8.082	0.59	40	d	0.046998
SNRPN	2	small nuclear ribonucleoprotein polypeptide N	201522_x_at	4	10.8	0.19	7	9.529	1.23	40	d	0
			216850_at	3	4.83	0.38	7	4.425	0.69	40	d	0.143279
			206042_x_at	0	9.385	0.28	7	8.076	1.22	40	d	0.000001
			1559342_a_at	0	3.067	0.11	7	3.141	0.37	40	u	0.335714
			1559545_at	0	3.789	0.27	7	3.902	0.38	40	u	0.468913
			1559546_s_at	0	3.49	0.08	7	3.564	0.22	40	u	0.140758
			1559343_at	0	3.195	0.37	7	3.907	0.92	40	u	0.055288
			1560741_at	0	7.114	0.8	7	5.629	0.96	40	d	0.000456
CDKN2AIP	2	CDKN2A interacting protein	218929_at	2	7.609	0.43	7	7.491	0.8	40	d	0.711596
NIT1	2	nitrilase 1	202891_at	2	8.19	0.16	7	8.101	0.61	40	d	0.455526
			241395_at	0	4.482	0.52	7	4.045	0.7	40	d	0.131511
CHD4	2	chromodomain helicase DNA binding protein 4	201184_s_at	2	8.613	0.34	7	8.409	0.66	40	d	0.43573
			201182_s_at	2	6.05	0.31	7	7.159	0.87	40	u	0.000004
			201183_s_at	2	8.91	0.29	7	9.148	0.55	40	u	0.282656
			1560419_at	0	3.205	0.18	7	3.327	0.26	40	u	0.254232
SLC2A4	2	solute carrier family 2 (facilitated glucose transporter), member 4	206603_at	2	2.539	0.03	7	2.624	0.17	40	u	0.007017
LRRC59	2	leucine rich repeat containing 59	222231_s_at	2	9.054	0.25	7	9.867	0.93	40	u	0.00008
			234812_at	0	1.815	0.04	7	1.859	0.06	40	u	0.060572
			1569371_at	0	2.221	0.02	7	2.31	0.17	40	u	0.004493
KCNIP4	2	Kv channel interacting protein 4	236783_at	2	2.62	0.29	7	2.638	0.2	40	u	0.846025
			240591_at	0	2.458	0.07	7	2.599	0.1	40	u	0.00122
			224530_s_at	0	3.048	0.24	7	3.195	0.35	40	u	0.298481
VSX1	2	visual system homeobox 1	222972_at	2	2.25	0.02	7	2.335	0.12	40	u	0.000366
			221124_s_at	0	2.569	0.12	7	2.534	0.16	40	d	0.588443
			224074_at	0	3.219	0.17	7	3.308	0.25	40	u	0.377673
			224075_s_at	0	2.564	0.03	7	2.6	0.06	40	u	0.164414
CAMK1D	2	calcium/calmodulin-dependent protein kinase ID	235626_at	2	6.072	0.43	7	6.807	0.88	40	u	0.039812
			220246_at	0	3.202	0.27	7	3.755	1.17	40	u	0.015201
PPP4C	2	protein phosphatase 4 (formerly X), catalytic subunit	208932_at	2	9.022	0.25	7	9.442	0.61	40	u	0.083492
ST14	2	suppression of tumorigenicity 14 (colon carcinoma)	202005_at	2	8.894	0.42	7	8.899	1.01	40	u	0.989865
			216905_s_at	2	7.839	0.39	7	7.959	1.1	40	u	0.620762
			216906_at	2	2.423	0.04	7	2.532	0.17	40	u	0.001451
FGD5	2	FYVE, RhoGEF and PH domain containing 5	226984_at	2	5.068	0.26	7	4.919	0.52	40	d	0.473805
			226985_at	2	7.15	0.66	7	6.185	0.97	40	d	0.017325
AKT1S1	2	AKT1 substrate 1 (proline-rich)	1555821_a_at	2	4.268	0.52	7	4.375	0.72	40	u	0.715199
			224982_at	2	6.902	0.31	7	6.729	1.05	40	d	0.420962
			213433_at	2	5.079	0.47	7	4.967	0.49	40	d	0.588277

ARL3	2	ADP-ribosylation factor-like 3	1568590_at	0	3.605	0.26	7	3.718	0.36	40	u	0.440588
			202641_at	0	9.809	0.35	7	9.718	0.59	40	d	0.700982
C7orf43	2	chromosome 7 open reading frame 43	220659_s_at	2	7.181	0.42	7	6.963	0.6	40	d	0.37241
OTUD7B	2	OTU domain containing 7B	220031_at	2	2.822	0.11	7	2.924	0.31	40	u	0.14565
			229488_at	0	5.978	0.31	7	6.137	0.65	40	u	0.53674
			1555139_a_at	0	3.178	0.29	7	3.501	0.52	40	u	0.121624
			238994_at	0	6.35	0.56	7	6.291	0.85	40	d	0.863947
			227436_at	0	6.872	0.17	7	6.907	0.87	40	u	0.82664
ZNF295	2	zinc finger protein 295	225539_at	2	7.448	0.68	7	6.712	0.76	40	d	0.022615
			233952_s_at	2	7.329	0.54	7	7.173	0.76	40	d	0.611615
SUFU	2	suppressor of fused homolog (Drosophila)	222749_at	2	7.706	0.26	7	7.07	0.68	40	d	0.020887
			224201_s_at	0	2.584	0.21	7	2.514	0.14	40	d	0.288157
			224202_at	0	2.018	0.04	7	2.073	0.13	40	u	0.051564
			224203_at	0	2.418	0.19	7	2.458	0.25	40	u	0.693142
C11orf24	2	chromosome 11 open reading frame 24	52164_at	2	7.282	0.19	7	7.712	0.69	40	u	0.002922
			218299_at	2	6.496	0.22	7	6.787	0.66	40	u	0.045697
TMEM128	2	transmembrane protein 128	225462_at	0	7.765	0.38	7	7.446	0.87	40	d	0.355881
RABL3	2	RAB, member of RAS oncogene family-like 3	213970_at	2	7.256	0.46	7	6.532	0.67	40	d	0.010297
			226090_x_at	2	6.866	0.31	7	6.823	0.56	40	d	0.847586
			226089_at	2	5.839	0.55	7	6.157	0.79	40	u	0.324075
INSR	2	insulin receptor	213792_s_at	2	9.181	0.48	7	8.742	1.17	40	d	0.342154
			226216_at	2	7.013	0.54	7	7.243	1.28	40	u	0.649795
			226212_s_at	2	5.147	0.41	7	4.906	1.03	40	d	0.551557
			207851_s_at	1	4.939	1.18	7	4.335	0.78	40	d	0.098568
			226450_at	0	7.9	0.31	7	7.413	0.81	40	d	0.131734
MARK4	2	MAP/microtubule affinity-regulating kinase 4	55065_at	2	7.496	0.28	7	7.535	0.77	40	u	0.818955
			221560_at	2	7.259	0.28	7	7.121	0.74	40	d	0.633902
PPP1R16B	2	protein phosphatase 1, regulatory (inhibitor) subunit 16B	41577_at	2	4.166	0.87	7	3.777	1.02	40	d	0.359447
			212750_at	2	4.792	0.68	7	4.398	1.06	40	d	0.357951
			233813_at	0	3.509	0.89	7	3.449	0.86	40	d	0.868431
MTERFD2	2	MTERF domain containing 2	214364_at	1	5.971	0.35	7	5.476	0.34	40	d	0.001298
			226486_at	1	6.047	0.3	7	5.497	0.53	40	d	0.012031
			1557966_x_at	0	7.706	0.22	7	7.51	0.56	40	d	0.377418
			1557965_at	0	8.072	0.15	7	7.719	0.6	40	d	0.003586
FOXN1	2	forkhead box N1	207683_at	2	2.086	0.01	7	2.106	0.04	40	u	0.024265
RASAL2	2	RAS protein activator like 2	222810_s_at	2	7.708	0.34	7	7.515	0.85	40	d	0.563403
			234343_s_at	1	3.289	0.34	7	3.614	0.6	40	u	0.178738
			219026_s_at	1	3.383	0.24	7	3.643	0.57	40	u	0.251557
			234910_at	1	2.966	0.33	7	3.105	0.32	40	u	0.31226
			217201_at	0	3.429	0.43	7	3.512	0.4	40	u	0.627396
			217194_at	0	2.985	0.12	7	3.248	0.27	40	u	0.015288
			1557432_at	0	6.75	0.52	7	6.003	1.05	40	d	0.077307
KCTD7	2	potassium channel tetramerisation domain containing 7	1553717_at	2	3.996	0.4	7	4.332	0.65	40	u	0.200589
			1555569_a_at	2	2.152	0.1	7	2.327	0.28	40	u	0.007957
SNX1	2	sorting nexin 1	214531_s_at	2	8.262	0.33	7	8.034	0.96	40	d	0.277606
			201716_at	1	9.421	0.29	7	8.924	0.74	40	d	0.094621
			213364_s_at	0	9.277	0.37	7	7.156	1.09	40	d	0
			216357_at	0	2.266	0.16	7	2.27	0.17	40	u	0.954939
ELF5	2	E74-like factor 5 (ets domain transcription factor)	220625_s_at	2	9.232	1.39	7	7.844	3.02	40	d	0.248704
			220624_s_at	0	6.731	1.87	7	5.494	2.99	40	d	0.305767
SETD7	2	SET domain containing (lysine methyltransferase) 7	224928_at	2	10.25	0.22	7	9.387	0.6	40	d	0.000664
ITGA9	2	integrin, alpha 9	206009_at	2	2.851	0.14	7	3.057	0.85	40	u	0.168456
			1555336_a_at	0	2.164	0.19	7	2.296	0.52	40	u	0.521279
			227297_at	0	7.924	0.51	7	5.561	1.54	40	d	0
			1555335_at	0	3.004	0.2	7	2.967	0.23	40	d	0.698566
KCNB1	2	potassium voltage-gated channel, Shab-related subfamily, member 1	211006_s_at	2	2.929	0.2	7	3.148	0.95	40	u	0.214483
UGP2	2	UDP-glucose pyrophosphorylase 2	205480_s_at	1	11.45	0.28	7	11.13	0.75	40	d	0.286229
RNF125	2	ring finger protein 125	207735_at	2	3.641	0.3	7	3.812	0.61	40	u	0.478567
			235199_at	0	5.025	0.34	7	4.644	0.68	40	d	0.162483
ZC3H10	2	zinc finger CCCH-type containing 10	227430_at	2	6.767	0.29	7	6.401	0.46	40	d	0.053214
M6PRBP1	2	mannose-6-phosphate receptor binding protein 1	202122_s_at	0	9.751	0.32	7	9.56	0.79	40	d	0.54148

FAT	2	FAT tumor suppressor homolog 1 (Drosophila)	201579_at	2	9.903	0.44	7	9.016	1.36	40	d	0.004039
ZFHX3	2	zinc finger homeobox 3	208033_s_at	2	5.737	0.37	7	5.122	1.32	40	d	0.023879
			233751_at	0	2.251	0.02	7	2.359	0.18	40	u	0.00089
			243323_s_at	0	2.985	0.11	7	3.015	0.26	40	u	0.774542
			233752_s_at	0	2.499	0.07	7	2.587	0.35	40	u	0.17952
			226137_at	0	7.408	0.34	7	6.701	1.44	40	d	0.012128
			242738_s_at	0	6.887	0.26	7	5.92	1.32	40	d	0.00019
GRM4	2	glutamate receptor, metabotropic 4	210234_at	1	5.685	0.37	7	5.926	0.45	40	u	0.198369
RRAGD	2	Ras-related GTP binding D	221524_s_at	2	6.651	0.43	7	7.396	1.33	40	u	0.011442
			221523_s_at	0	6.532	0.71	7	5.954	1.34	40	d	0.280557
EIF2S2	2	eukaryotic translation initiation factor 2, subunit 2 beta, 38kDa	208725_at	2	4.708	0.24	7	5.233	0.85	40	u	0.003516
			208726_s_at	0	9.9	0.24	7	10.66	0.65	40	u	0.00466
RNF130	2	ring finger protein 130	217865_at	2	9.777	0.24	7	9.391	0.86	40	d	0.029301
			1563975_at	0	3.436	0.39	7	4.271	0.56	40	u	0.000594
			236292_at	0	5.997	0.45	7	5.939	0.74	40	d	0.845519
ZBTB44	2	zinc finger and BTB domain containing 44	226148_at	0	10.84	0.18	7	9.472	0.88	40	d	0
			225845_at	0	9.85	0.3	7	8.499	1	40	d	0
			1554469_at	0	5.722	0.5	7	5.532	0.91	40	d	0.599553
			1554470_s_at	0	7.464	0.17	7	6.451	1.11	40	d	0.000003
			220243_at	0	5.551	0.87	7	3.93	0.98	40	d	0.000214
TIMP2	2	TIMP metallopeptidase inhibitor 2	231579_s_at	2	11.91	0.49	7	10.51	0.8	40	d	0.000069
			224560_at	2	11.48	0.36	7	9.908	0.91	40	d	0.000067
			203167_at	0	7.052	0.41	7	7.619	0.93	40	u	0.126204
ZMAT4	2	zinc finger, matrin type 4	219877_at	2	2.717	0.43	7	2.971	1.13	40	u	0.56766
MAK	2	male germ cell-associated kinase	1555315_a_at	2	2.036	0.02	7	2.138	0.23	40	u	0.010454
			220302_at	2	2.861	0.38	7	2.974	0.9	40	u	0.750598
STC2	2	stanniocalcin 2	203439_s_at	2	9.54	0.91	7	5.789	1.37	40	d	0
			203438_at	2	10.72	0.61	7	6.148	1.49	40	d	0
PPP1R14C	2	protein phosphatase 1, regulatory (inhibitor) subunit 14C	226907_at	2	6.659	0.91	7	6.817	2.37	40	u	0.8653
STMN4	2	stathmin-like 4	221236_s_at	2	3.247	0.42	7	3.16	0.51	40	d	0.678833
WNT4	2	wingless-type MMTV integration site family, member 4	208606_s_at	0	3.9	1.55	7	2.467	1.05	40	d	0.004349
			1556689_a_at	0	3.247	0.5	7	3.14	0.19	40	d	0.62128
DLAT	2	dihydrolipoamide S-acetyltransferase (E2 component of pyruvate dehydrogenase complex)	213149_at	2	7.122	0.2	7	7.254	0.67	40	u	0.334271
			212568_s_at	0	7.083	0.39	7	7.632	0.86	40	u	0.110014
			211150_s_at	0	5.986	0.34	7	6.635	1.2	40	u	0.009953
C10orf54	2	chromosome 10 open reading frame 54	225372_at	2	3.908	0.5	7	3.026	0.54	40	d	0.00028
			225373_at	2	8.3	0.6	7	6.728	0.71	40	d	0.000003
JUN	2	jun oncogene	201464_x_at	2	11.86	0.66	7	10.18	0.73	40	d	0.000001
			201466_s_at	2	10.77	0.72	7	8.855	0.88	40	d	0.000003
			213281_at	2	7.657	0.9	7	6.866	0.77	40	d	0.0211
			201465_s_at	0	9.445	1.29	7	8.195	1	40	d	0.006553
CSF1R	2	colony stimulating factor 1 receptor, formerly McDonough feline sarcoma viral (v-fms) oncogene homolog	203104_at	2	8.48	0.49	7	7.204	0.93	40	d	0.00113
ATP1B3	2	ATPase, Na+/K+ transporting, beta 3 polypeptide	208836_at	2	9.919	0.28	7	10.76	0.8	40	u	0.000054
			229709_at	0	2.411	0.17	7	2.406	0.15	40	d	0.942879
			226570_at	0	3.4	0.29	7	3.719	0.55	40	u	0.145858
AP4E1	2	adaptor-related protein complex 4, epsilon 1 subunit	241174_at	2	2.152	0.08	7	2.192	0.12	40	u	0.414919
			228164_at	2	6.289	0.27	7	6.31	0.64	40	u	0.934227
			220228_at	1	1.966	0.06	7	2.013	0.12	40	u	0.310842
			220229_s_at	1	2.705	0.05	7	2.919	0.27	40	u	0.00005
SFRP1	2	secreted frizzled-related protein 1	202037_s_at	2	13	0.28	7	8.756	2.67	40	d	0
			202035_s_at	2	10.24	0.45	7	6.714	2.66	40	d	0
			202036_s_at	0	11.79	0.49	7	8.623	2.61	40	d	0
			228413_s_at	0	2.645	0.27	7	2.82	0.62	40	u	0.473738
DAPK1	2	death-associated protein kinase 1	203139_at	2	6.037	0.93	7	7.299	0.98	40	u	0.003451
			211214_s_at	0	2.595	0.09	7	3.111	0.75	40	u	0.000155
CDH5	2	cadherin 5, type 2, VE-cadherin (vascular epithelium)	204677_at	2	8.025	0.57	7	5.956	1.1	40	d	0.000019
SMARCE1	2	SWI/SNF related, matrix associated, actin dependent regulator of chromatin, subfamily e, member 1	211989_at	2	9.213	0.14	7	9.572	0.69	40	u	0.006112
			211988_at	2	10.44	0.21	7	10.12	0.82	40	d	0.050469
			229511_at	0	6.251	0.57	7	5.914	1.19	40	d	0.476829

ADD2	2	adducin 2 (beta)	237336_at	2	2.964	0.27	7	3.049	0.28	40	u	0.469299
			206807_s_at	1	4.751	0.13	7	4.629	0.31	40	d	0.315775
			205268_s_at	0	2.436	0.08	7	3.161	1.43	40	u	0.003285
LRIG2	2	leucine-rich repeats and immunoglobulin-like domains 2	205953_at	2	6.46	0.54	7	6.223	0.61	40	d	0.350377
			242165_at	0	3.05	0.24	7	3.071	0.27	40	u	0.847535
			242164_s_at	0	2.99	0.05	7	3.111	0.17	40	u	0.000988
PISD	2	phosphatidylserine decarboxylase	202392_s_at	2	8.255	0.16	7	7.408	0.83	40	d	0.000001
NPTXR	2	neuronal pentraxin receptor	213040_s_at	2	4.736	0.58	7	4.459	0.92	40	d	0.455941
			217041_at	2	2.533	0.25	7	2.49	0.29	40	d	0.719527
SCN9A	2	sodium channel, voltage-gated, type IX, alpha subunit	206950_at	2	3.389	0.32	7	3.223	0.23	40	d	0.114616
UBC	2	ubiquitin C	211296_x_at	2	14.3	0.12	7	14.23	0.31	40	d	0.592053
			208980_s_at	2	13.9	0.17	7	13.9	0.48	40	d	0.971461
CEP250	2	centrosomal protein 250kDa	210894_s_at	0	5.024	0.22	7	4.994	0.46	40	d	0.868162
			209495_at	0	5.268	0.27	7	5.604	0.63	40	u	0.181108
MUM1L1	2	melanoma associated antigen (mutated) 1-like 1	229160_at	2	4.843	1.47	7	4.352	1.9	40	d	0.527628
CRTAM	2	cytotoxic and regulatory T cell molecule	206914_at	2	3.104	0.26	7	3.479	0.78	40	u	0.029478
ITPK1	2	inositol 1,3,4-triphosphate 5/6 kinase	210740_s_at	2	7.663	0.19	7	7.466	0.74	40	d	0.171839
			210197_at	0	2.256	0.08	7	2.36	0.22	40	u	0.040769
			217710_x_at	0	5.649	0.26	7	5.869	0.36	40	u	0.132998
G3BP1	2	GTPase activating protein (SH3 domain) binding protein 1	201503_at	2	9.837	0.13	7	10.22	0.54	40	u	0.000578
			1557350_at	0	5.697	0.56	7	5.31	0.58	40	d	0.116695
			244396_at	0	3.673	0.19	7	4.127	0.66	40	u	0.001574
			201514_s_at	0	8.153	0.29	7	8.361	0.81	40	u	0.24636
			222187_x_at	0	4.665	0.36	7	4.831	0.51	40	u	0.419312
CYP39A1	2	cytochrome P450, family 39, subfamily A, polypeptide 1	220432_s_at	2	5.724	0.85	7	4.894	1.56	40	d	0.185757
			1553977_a_at	2	5.274	0.52	7	4.233	1.41	40	d	0.065216
			244407_at	0	3.339	0.86	7	3.372	1.34	40	u	0.950922
GLULD1	2	glutamate-ammonia ligase (glutamine synthetase) domain containing 1	220393_at	2	2.594	0.11	7	2.818	0.63	40	u	0.046809
DAZAP1	2	DAZ associated protein 1	229813_x_at	2	8.845	0.23	7	8.808	0.59	40	d	0.87549
			218443_s_at	2	8.916	0.19	7	9.322	0.49	40	u	0.039703
			226620_x_at	2	9.837	0.28	7	9.974	0.56	40	u	0.543171
GYS1	2	glycogen synthase 1 (muscle)	201673_s_at	2	7.223	0.16	7	6.978	0.9	40	d	0.127341
TAF9B	2	TAF9B RNA polymerase II, TATA box binding protein (TBP)-associated factor, 31kDa	228483_s_at	0	6.379	0.37	7	6.486	0.77	40	u	0.726307
			226037_s_at	0	8.87	0.26	7	8.337	0.69	40	d	0.053146
			221618_s_at	0	5.612	0.81	7	5.364	1.42	40	d	0.661791
			221617_at	0	5.303	0.44	7	5.357	0.58	40	u	0.819762
			221616_s_at	0	2.804	0.46	7	3.134	0.79	40	u	0.300451
PI4KB	2	phosphatidylinositol 4-kinase, catalytic, beta	206139_at	2	7.102	0.25	7	7.259	0.62	40	u	0.519176
			206138_s_at	2	8.95	0.08	7	9.037	0.59	40	u	0.385734
			210417_s_at	0	8.417	0.19	7	8.379	0.98	40	d	0.82923
ANP32E	2	acidic (leucine-rich) nuclear phosphoprotein 32 family, member E	221505_at	2	9.269	0.28	7	10.23	1.18	40	u	0.000101
			229128_s_at	0	7.182	0.45	7	8.087	2.4	40	u	0.039191
			208103_s_at	0	3.593	0.39	7	6.597	1.84	40	u	0
OAZ2	2	ornithine decarboxylase antizyme 2	201365_at	2	8.69	0.23	7	8.212	0.55	40	d	0.033037
			201364_s_at	0	9.034	0.3	7	8.934	0.67	40	d	0.705042
			238024_at	0	2.553	0.33	7	2.594	0.45	40	u	0.822239
LRFN5	2	leucine rich repeat and fibronectin type III domain containing 5	230644_at	2	4.552	0.72	7	3.509	0.33	40	d	0.012957
ITM2B	2	integral membrane protein 2B	217731_s_at	2	11.77	0.16	7	10.95	0.57	40	d	0
			217732_s_at	0	12.7	0.2	7	11.78	0.76	40	d	0
KRT1	2	keratin 1 (epidermolytic hyperkeratosis)	205900_at	1	2.058	0.02	7	2.166	0.24	40	u	0.008929
SDAD1	2	SDA1 domain containing 1	218607_s_at	2	8.437	0.16	7	8.697	0.57	40	u	0.027747
			228408_s_at	0	8.144	0.17	7	8.909	0.76	40	u	0.000003
			242190_at	0	5.374	0.27	7	5.519	0.41	40	u	0.381116
CPNE5	2	copine V	227189_at	2	4.047	0.41	7	4.207	1.17	40	u	0.529861
CLTA	2	clathrin, light chain (Lca)	200960_x_at	2	11.58	0.15	7	11.38	0.7	40	d	0.128657
			204050_s_at	2	11.1	0.19	7	10.89	0.83	40	d	0.170002
			216296_at	1	3.687	0.35	7	4.03	0.63	40	u	0.174161
			216295_s_at	1	11.38	0.2	7	11.33	0.75	40	d	0.72865
			216293_at	1	6.657	0.49	7	6.573	0.33	40	d	0.575938
			1560434_x_at	0	3.762	0.51	7	3.465	0.58	40	d	0.21736

DCP1A	2	DCP1 decapping enzyme homolog A (S. cerevisiae)	218508_at	2	7.753	0.24	7	7.075	0.61	40	d	0.006855
ANLN	2	anillin, actin binding protein	222608_s_at	2	3.503	0.5	7	8.109	1.4	40	u	0
			1552619_a_at	0	2.984	0.56	7	7.178	1.61	40	u	0
RBED1	2	RNA binding motif and ELMO/CED-12 domain 1	226286_at	3	6.611	0.4	7	5.832	0.46	40	d	0.000162
GHR	2	growth hormone receptor	205498_at	2	8.275	0.9	7	4.884	1.64	40	d	0.000005
HS3ST1	2	heparan sulfate (glucosamine) 3-O-sulfotransferase 1	205465_x_at	2	2.093	0.02	7	2.155	0.12	40	u	0.006889
			205466_s_at	2	4.717	0.95	7	4.432	1.05	40	d	0.515755
			213991_s_at	0	2.457	0.18	7	2.526	0.19	40	u	0.381333
RBPJ	2	recombination signal binding protein for immunoglobulin kappa J region	211974_x_at	2	9.968	0.18	7	9.991	0.52	40	u	0.838632
			207785_s_at	2	9.218	0.2	7	9.025	0.65	40	d	0.156335
			229540_at	0	3.646	0.54	7	3.712	0.56	40	u	0.779421
FRMD5	2	FERM domain containing 5	1569470_a_at	2	3.401	0.26	7	3.567	0.4	40	u	0.307445
RNF217	2	ring finger protein 217	235492_at	2	4.145	0.31	7	4.507	1.08	40	u	0.100954
USP7	2	ubiquitin specific peptidase 7 (herpes virus-associated)	201499_s_at	2	9.648	0.2	7	9.599	0.54	40	d	0.683904
			230967_s_at	0	3.206	0.62	7	2.673	0.44	40	d	0.009566
			201498_at	0	8.874	0.25	7	8.811	0.82	40	d	0.7078
			222032_s_at	0	7.301	0.27	7	7.044	0.28	40	d	0.031328
SLC25A35	2	solute carrier family 25, member 35	236064_at	2	5.584	0.35	7	5.541	0.56	40	d	0.847831
			228836_at	0	4.109	0.71	7	4.469	0.84	40	u	0.303712
KL	2	klotho	205978_at	2	3.939	0.58	7	3.269	0.78	40	d	0.039289
LRRC4C	2	leucine rich repeat containing 4C	232226_at	2	5.847	0.8	7	5.491	1.55	40	d	0.562954
			241585_at	0	2.392	0.02	7	2.503	0.22	40	u	0.003814
EDNRA	2	endothelin receptor type A	204463_s_at	2	5.52	0.79	7	5.839	1.16	40	u	0.497062
			204464_s_at	2	7.791	0.92	7	7.263	1.14	40	d	0.260052
			216235_s_at	0	5.409	0.46	7	5.575	0.93	40	u	0.651301
PTMS	2	parathymosin	218044_x_at	2	2.395	0.12	7	2.576	0.32	40	u	0.15431
			218045_x_at	2	8.57	0.38	7	8.741	0.82	40	u	0.598841
PHF16	2	PHD finger protein 16	204866_at	2	6.62	0.71	7	7.379	1.01	40	u	0.068567
RYR1	2	ryanodine receptor 1 (skeletal)	205485_at	2	3.793	1.24	7	3.179	1.86	40	d	0.413966
BACE2	2	beta-site APP-cleaving enzyme 2	222446_s_at	2	7.601	0.53	7	8.404	1.47	40	u	0.019002
			217867_x_at	0	7.828	0.41	7	8.646	1.58	40	u	0.010677
ACTC1	2	actin, alpha, cardiac muscle 1	205132_at	2	3.398	0.12	7	3.429	0.21	40	u	0.708144
ERO1L	2	ERO1-like (S. cerevisiae)	218498_s_at	2	6.7	0.45	7	7.159	0.73	40	u	0.121375
			222646_s_at	2	6.997	0.3	7	7.985	1.24	40	u	0.000147
ACOT7	2	acyl-CoA thioesterase 7	208002_s_at	2	6.389	0.47	7	7.711	1.08	40	u	0.003067
			215728_s_at	0	5.659	0.26	7	6.2	0.72	40	u	0.061904
FGFR3	2	fibroblast growth factor receptor 3 (achondroplasia, thanatophoric dwarfism)	204379_s_at	2	5.682	1.27	7	6.32	2.45	40	u	0.513633
			204380_s_at	0	2.564	0.34	7	3.038	0.58	40	u	0.045829
POU6F1	2	POU class 6 homeobox 1	216330_s_at	2	4.585	0.41	7	4.244	0.6	40	d	0.165113
			205878_at	2	6.926	0.31	7	6.139	0.33	40	d	0.000001
			216332_at	2	2.51	0.25	7	2.381	0.21	40	d	0.164559
PLD1	2	phospholipase D1, phosphatidylcholine-specific	226636_at	2	7.066	0.59	7	6.04	0.84	40	d	0.004099
			215723_s_at	0	3.603	0.57	7	3.527	1.19	40	d	0.870881
			232530_at	0	3.62	1.33	7	3.557	1.07	40	d	0.893897
			205203_at	0	5.333	0.56	7	4.655	0.92	40	d	0.070489
			1557126_a_at	0	3.607	0.67	7	2.955	0.57	40	d	0.011306
			177_at	0	4.315	0.59	7	4.021	0.79	40	d	0.365096
			215724_at	0	3.248	0.17	7	3.371	0.26	40	u	0.251194
VASH1	2	vasohibin 1	203940_s_at	2	6.172	0.45	7	5.54	0.72	40	d	0.031899
			239810_at	0	4.866	0.45	7	4.876	0.45	40	u	0.956945
			230546_at	0	4.709	0.33	7	4.748	0.37	40	u	0.803144
			242566_at	0	2.851	0.5	7	2.663	0.23	40	d	0.397747
			1556423_at	0	4.586	0.72	7	3.923	0.92	40	d	0.082379
CNIH	2	cornichon homolog (Drosophila)	201653_at	2	9.337	0.33	7	9.668	0.67	40	u	0.217578
VTCN1	2	V-set domain containing T cell activation inhibitor 1	219768_at	0	10.34	0.81	7	8.781	2.4	40	d	0.004935
KIF2A	2	kinesin heavy chain member 2A	203087_s_at	2	6.225	0.41	7	7.289	0.9	40	u	0.004389
			203086_at	0	3.984	0.4	7	4.565	0.78	40	u	0.066398
PNOC	2	prepronociceptin	205901_at	2	2.545	0.35	7	2.656	0.81	40	u	0.72988
AGGF1	2	angiogenic factor with G patch and FHA domains 1	218534_s_at	2	7.427	0.3	7	7.561	0.76	40	u	0.656787
			222661_at	2	7.904	0.32	7	7.169	0.72	40	d	0.012364
			210710_at	0	2.434	0.19	7	2.599	0.4	40	u	0.298802

			208042_at	0	5.364	0.33	7	6.029	0.68	40	u	0.017023
TMED9	2	transmembrane emp24 protein transport domain containing 9	208757_at	2	9.539	0.25	7	9.828	0.68	40	u	0.279973
			205812_s_at	0	10.88	0.21	7	11.16	0.79	40	u	0.078791
			239627_at	0	5.416	0.21	7	5.444	0.33	40	u	0.83221
MYH1	2	myosin, heavy chain 1, skeletal muscle, adult	205951_at	2	3.098	0.39	7	3.027	0.18	40	d	0.674928
UTP14C	2	UTP14, U3 small nucleolar ribonucleoprotein, homolog C (yeast)	203614_at	2	8.62	0.25	7	8.217	0.77	40	d	0.018369
PNPLA6	2	patatin-like phospholipase domain containing 6	203718_at	2	7.793	0.32	7	7.565	0.76	40	d	0.449915
CDH24	2	cadherin-like 24	230157_at	2	5.326	0.41	7	6.156	0.59	40	u	0.001025
			1553166_at	0	3.133	0.08	7	3.277	0.44	40	u	0.066754
VAMP4	2	vesicle-associated membrane protein 4	211760_s_at	2	4.734	0.51	7	5.536	0.86	40	u	0.023403
			207350_s_at	1	5.765	0.48	7	6.5	0.66	40	u	0.008063
			213480_at	1	8.087	0.32	7	7.825	0.58	40	d	0.259215
EML5	2	echinoderm microtubule associated protein like 5	1568777_at	2	2.968	0.39	7	2.831	0.35	40	d	0.36577
			1570393_at	0	2.068	0.02	7	2.133	0.09	40	u	0.000503
			242443_at	0	3.578	0.62	7	3.186	0.45	40	d	0.054809
SORCS2	2	sortilin-related VPS10 domain containing receptor 2	228720_at	0	4.87	0.5	7	4.851	0.62	40	d	0.939737
HEXIM1	2	hexamethylene bis-acetamide inducible 1	202815_s_at	2	7.594	0.2	7	6.527	0.64	40	d	0
			202814_s_at	2	8.118	0.31	7	7.723	0.82	40	d	0.223845
			214188_at	0	5.895	0.66	7	4.607	0.9	40	d	0.000979
			238442_at	0	2.691	0.4	7	2.641	0.22	40	d	0.771981
HNRPH1	2	heterogeneous nuclear ribonucleoprotein H1 (H)	201031_s_at	2	11.69	0.21	7	11.49	0.6	40	d	0.139069
			213619_at	2	12.07	0.35	7	12.02	0.64	40	d	0.852988
			213470_s_at	0	8.268	0.94	7	7.774	1.25	40	d	0.334954
			213472_at	0	4.301	0.79	7	4.841	1.01	40	u	0.193291
SNAI1	2	snail homolog 1 (Drosophila)	219480_at	2	4.988	0.64	7	5.062	0.54	40	u	0.752814
DEF8	2	differentially expressed in FDCP 8 homolog (mouse)	225637_at	2	8.213	0.1	7	7.437	0.89	40	d	0.000005
			219646_at	0	5.598	0.27	7	6.082	1.01	40	u	0.018859
CCDC113	2	coiled-coil domain containing 113	222890_at	2	6.296	0.26	7	6.179	1.17	40	d	0.593428
TOR1AIP1	2	torsin A interacting protein 1	212409_s_at	2	7.408	0.56	7	7.506	1.05	40	u	0.813158
			212408_at	2	10.47	0.38	7	9.967	0.61	40	d	0.043984
			216100_s_at	2	5.974	0.68	7	6.26	1.09	40	u	0.516824
ADC	2	arginine decarboxylase	228000_at	0	6.384	0.34	7	6.27	0.51	40	d	0.577568
			1554393_a_at	0	4.644	0.81	7	5.32	0.88	40	u	0.069569
MLL3	2	myeloid/lymphoid or mixed-lineage leukemia 3	235859_at	0	2.011	0.04	7	2.202	0.37	40	u	0.003999
			222414_at	0	8.358	0.42	7	8.038	0.82	40	d	0.328328
			222413_s_at	0	7.543	0.51	7	7.779	1.09	40	u	0.584343
			222415_at	0	9.69	0.44	7	9.039	1.02	40	d	0.111606
			1557158_s_at	0	4.257	0.22	7	4.916	0.89	40	u	0.000389
			232940_s_at	0	5.304	0.52	7	6.426	0.96	40	u	0.005023
MOSPD1	2	motile sperm domain containing 1	218853_s_at	2	7.092	0.49	7	7.593	0.84	40	u	0.140192
			1557455_s_at	0	5.022	0.32	7	6.18	1.08	40	u	0.000009
KIAA1161	2	KIAA1161	232244_at	2	3.758	0.34	7	4.248	0.82	40	u	0.135433
			231185_at	0	2.337	0.16	7	2.335	0.14	40	d	0.974877
PXN	2	paxillin	201087_at	2	8.059	0.33	7	6.723	0.83	40	d	0.000164
			211823_s_at	0	5.524	0.71	7	4.544	1.38	40	d	0.078497
C1orf198	2	chromosome 1 open reading frame 198	223063_at	2	9.716	0.34	7	9.839	0.85	40	u	0.715111
NGFR	2	nerve growth factor receptor (TNFR superfamily, member 16)	205858_at	2	6.614	1.24	7	4.115	0.91	40	d	0
PCBP1	2	poly(rC) binding protein 1	208620_at	1	10.85	0.19	7	10.33	0.68	40	d	0.000457
NGFRAP1	2	nerve growth factor receptor (TNFRSF16) associated protein 1	217963_s_at	2	12.35	0.25	7	11.74	1.09	40	d	0.004904
NEDD4L	2	neural precursor cell expressed, developmentally down-regulated 4-like	212445_s_at	2	7.649	0.49	7	6.361	1.01	40	d	0.002279
			212448_at	2	8.128	0.76	7	6.196	1.05	40	d	0.00004
SEC13	2	SEC13 homolog (S. cerevisiae)	207707_s_at	2	10.17	0.18	7	10.58	0.65	40	u	0.002783
TUBA1A	2	tubulin, alpha 1a	209118_s_at	2	12.15	0.26	7	12.03	0.79	40	d	0.465656
PRMT8	2	protein arginine methyltransferase 8	230839_at	2	3.044	0.32	7	3.069	0.59	40	u	0.914684
			207772_s_at	0	2.526	0.12	7	2.606	0.16	40	u	0.233337
BRSK1	2	BR serine/threonine kinase 1	1552504_a_at	2	4.758	0.39	7	4.965	0.65	40	u	0.426927
MAP1LC3B	2	microtubule-associated protein 1 light chain 3 beta	208786_s_at	2	9.644	0.23	7	9.159	0.67	40	d	0.002513
			208785_s_at	2	8.334	0.36	7	8.856	0.76	40	u	0.086211

LRBA	2	LPS-responsive vesicle trafficking, beach and anchor containing	214109_at	2	7.359	0.84	7	7.296	1.3	40	d	0.903262
			212692_s_at	2	9.043	0.48	7	8.646	1.04	40	d	0.337543
NAPB	2	N-ethylmaleimide-sensitive factor attachment protein, beta	225111_s_at	2	6.827	0.29	7	6.385	1	40	d	0.034828
			1570441_at	0	2.926	0.34	7	2.935	0.34	40	u	0.946646
FNTB	2	farnesyltransferase, CAAX box, beta	225851_at	2	7.153	0.31	7	6.949	0.52	40	d	0.33045
			204764_at	0	4.804	0.47	7	4.471	0.91	40	d	0.360095
			233283_at	0	3.02	0.06	7	3.034	0.3	40	u	0.796953
			1568865_at	0	3.303	0.63	7	3.572	0.86	40	u	0.44145
			1773_at	0	4.097	0.47	7	4.133	0.7	40	u	0.899008
ZNF294	2	zinc finger protein 294	215596_s_at	2	8.869	0.25	7	8.847	0.47	40	d	0.904632
			233819_s_at	0	5.017	0.32	7	6.984	0.89	40	u	0
			233818_at	0	2.407	0.05	7	2.584	0.3	40	u	0.001456
HAND2	2	heart and neural crest derivatives expressed 2	220480_at	2	2.252	0.26	7	2.19	0.07	40	d	0.585487
ANTXR1	2	anthrax toxin receptor 1	224694_at	2	10.43	0.47	7	9.658	1.1	40	d	0.080287
			234832_at	0	3.508	0.4	7	3.653	0.42	40	u	0.406681
			220092_s_at	0	4.963	0.63	7	5.265	0.89	40	u	0.405951
			227660_at	0	5.708	0.6	7	6.172	0.71	40	u	0.119628
			234430_at	0	2.214	0.09	7	2.319	0.14	40	u	0.065089
			220093_at	0	3.611	1	7	3.573	0.57	40	d	0.931444
PANK1	2	pantothenate kinase 1	226649_at	2	4.531	0.39	7	5.812	0.98	40	u	0.001676
GPR23	2	G protein-coupled receptor 23	206960_at	2	3.517	0.21	7	3.487	0.29	40	d	0.798073
C19orf47	2	chromosome 19 open reading frame 47	234999_at	2	4.486	0.5	7	4.572	0.59	40	u	0.72462
			1554475_a_at	1	4.366	0.26	7	4.511	0.42	40	u	0.389924
WBP11	2	WW domain binding protein 11	217822_at	2	9.027	0.3	7	9.508	0.77	40	u	0.118432
			217821_s_at	0	8.181	0.3	7	8.335	1.2	40	u	0.502831
CTNNB1	2	catenin (cadherin-associated protein), beta 1, 88kDa	201533_at	2	9.942	0.4	7	9.684	0.6	40	d	0.293071
			1554411_at	1	6.205	0.92	7	5.101	1.06	40	d	0.014993
			1570507_at	0	5.176	0.55	7	5.345	0.91	40	u	0.645332
			223679_at	0	8.953	0.34	7	7.991	0.86	40	d	0.006329
GALNT2	2	UDP-N-acetyl-alpha-D-galactosamine:polypeptide N-acetylgalactosaminyltransferase 2 (GalNAc-T2)	217787_s_at	2	5.429	0.4	7	6.432	1	40	u	0.01406
			217788_s_at	2	8.225	0.33	7	8.265	0.87	40	u	0.907404
			223991_s_at	0	5.551	0.27	7	6.21	0.93	40	u	0.001237
			239930_at	0	5.674	0.52	7	5.71	0.99	40	u	0.927382
ANKRD17	2	ankyrin repeat domain 17	212211_at	2	8.5	0.2	7	8.297	0.74	40	d	0.170029
			225852_at	0	9.474	0.24	7	9.525	0.58	40	u	0.824075
RNF150	2	ring finger protein 150	227657_at	2	4.836	0.8	7	3.532	1.57	40	d	0.041129
EAF1	2	ELL associated factor 1	226952_at	2	6.804	0.25	7	7.569	0.7	40	u	0.000042
PSIP1	2	PC4 and SFRS1 interacting protein 1	209337_at	2	8.367	0.37	7	8.022	1.09	40	d	0.147744
			205961_s_at	1	7.506	0.25	7	7.473	1.17	40	d	0.880824
			1554933_at	0	3.54	0.18	7	3.905	0.53	40	u	0.002999
			210758_at	0	5.595	0.18	7	6.626	0.99	40	u	0.000001
EIF2C3	2	eukaryotic translation initiation factor 2C, 3	219426_at	2	6.797	0.5	7	7.761	0.75	40	u	0.00256
C11orf61	2	chromosome 11 open reading frame 61	221208_s_at	2	7.123	0.4	7	5.975	1.07	40	d	0.008746
PGGT1B	2	protein geranylgeranyltransferase type I, beta subunit	206288_at	2	4.334	0.55	7	4.294	0.88	40	d	0.908679
			235615_at	0	7.542	0.4	7	7.677	0.78	40	u	0.66204
			242844_at	0	7.362	0.37	7	7.607	1.1	40	u	0.301535
			244684_at	0	2.3	0.19	7	2.332	0.22	40	u	0.72971
KCNN3	2	potassium intermediate/small conductance calcium-activated channel, subfamily N,	205902_at	2	3.748	0.52	7	3.362	0.41	40	d	0.037862
			205903_s_at	0	2.59	0.07	7	2.685	0.4	40	u	0.176389
CD79B	2	CD79b molecule, immunoglobulin-associated beta	205297_s_at	2	5.092	0.49	7	5.251	0.95	40	u	0.673351
			1555746_at	0	4.24	0.49	7	3.838	0.4	40	d	0.026018
			1555748_x_at	0	4.544	0.16	7	4.335	0.41	40	d	0.197617
TNFRSF19	2	tumor necrosis factor receptor superfamily, member 19	223827_at	2	2.069	0.06	7	2.198	0.25	40	u	0.008065
			224090_s_at	0	2.482	0.06	7	2.451	0.13	40	d	0.532826
			227812_at	0	5.945	0.73	7	5.104	1.25	40	d	0.097459
AADACL1	2	arylacetamide deacetylase-like 1	225847_at	2	5.67	0.55	7	6.173	1.23	40	u	0.303499
LUZP2	2	leucine zipper protein 2	215323_at	2	3.915	0.54	7	4.186	0.47	40	u	0.18578
KCNAB1	2	potassium voltage-gated channel, shaker-related subfamily, beta member 1	210471_s_at	2	4.377	1.03	7	3.507	0.41	40	d	0.087511
			210079_x_at	2	2.238	0.02	7	2.284	0.08	40	u	0.003943
			210078_s_at	2	4.776	0.77	7	4.232	0.74	40	d	0.08637
			208213_s_at	1	2.731	0.34	7	2.709	0.32	40	d	0.872191

ERC2	2	ELKS/RAB6-interacting/CAST family member 2	213938_at	2	2.403	0.08	7	2.696	0.55	40	u	0.003052
PAPD4	2	PAP associated domain containing 4	225761_at	2	8.026	0.27	7	7.495	0.7	40	d	0.057802
			238706_at	0	7.978	0.42	7	7.855	0.87	40	d	0.722623
			1556277_a_at	0	5.89	0.7	7	6.323	0.94	40	u	0.260086
PRDM13	2	PR domain containing 13	221168_at	2	2.517	0.03	7	2.837	0.61	40	u	0.002231
FRS3	2	fibroblast growth factor receptor substrate 3	219907_at	2	5.132	0.34	7	5.232	0.55	40	u	0.649443
C22orf9	2	chromosome 22 open reading frame 9	212421_at	2	6.75	0.14	7	6.304	0.68	40	d	0.000827
			227144_at	2	6.355	0.31	7	6.072	0.61	40	d	0.245036
			217118_s_at	2	8.906	0.31	7	8.625	0.69	40	d	0.305526
PPP1R14D	2	protein phosphatase 1, regulatory (inhibitor) subunit 14D	220082_at	2	1.949	0.05	7	2.044	0.17	40	u	0.009926
PIK3CD	2	phosphoinositide-3-kinase, catalytic, delta polypeptide	203879_at	2	6.914	0.48	7	5.919	1.33	40	d	0.00232
			211230_s_at	1	5.93	0.33	7	5.695	0.45	40	d	0.203396
ALG11	2	osylation 11 homolog (S. cerevisiae, alpha-1,2	no probeset available									
ARPC1A	2	actin related protein 2/3 complex, subunit 1A, 41kDa	200950_at	0	10.19	0.21	7	10.25	0.81	40	u	0.658283
FZD10	2	frizzled homolog 10 (Drosophila)	219764_at	1	3.017	0.45	7	3.003	1.09	40	d	0.972858
PTGIR	2	prostaglandin I2 (prostacyclin) receptor (IP)	206187_at	2	5.417	0.4	7	5.485	0.37	40	u	0.668876
			1555620_a_at	0	2.408	0.38	7	2.58	0.36	40	u	0.264718
NLGN3	2	neuroligin 3	219726_at	2	3.559	0.6	7	3.503	0.67	40	d	0.840221
			234751_s_at	0	2.874	0.33	7	2.764	0.31	40	d	0.404808
LMX1A	2	LIM homeobox transcription factor 1, alpha	1553541_at	1	1.986	0.02	7	2.045	0.08	40	u	0.000229
			237595_at	0	3.745	0.73	7	3.726	0.72	40	d	0.94959
PPP1R3F	2	protein phosphatase 1, regulatory (inhibitor) subunit 3F	229365_at	2	5.732	0.26	7	5.849	0.39	40	u	0.463229
			231197_at	0	2.636	0.16	7	2.633	0.24	40	d	0.977049
			232211_at	0	5.505	0.18	7	5.299	0.44	40	d	0.240528
CALM2	2	calmodulin 2 (phosphorylase kinase, delta)	207243_s_at	2	13.44	0.2	7	13.79	0.29	40	u	0.005181
FUT8	2	fucosyltransferase 8 (alpha (1,6) fucosyltransferase)	203988_s_at	2	7.467	0.44	7	6.649	1.4	40	d	0.00809
			1554930_a_at	0	6.613	0.46	7	5.987	1.34	40	d	0.037913
SNURF	2	SNRPN upstream reading frame	no probeset available									
JMJD2A	2	jumonji domain containing 2A	203205_at	2	6.812	0.2	7	6.724	0.55	40	d	0.472029
			203204_s_at	0	6.596	0.35	7	7.402	0.75	40	u	0.008678
CASP8AP2	2	CASP8 associated protein 2	222201_s_at	2	7.032	0.37	7	7.615	0.79	40	u	0.068789
			1570001_at	0	3.343	0.43	7	3.646	0.55	40	u	0.181157
COLEC12	2	collectin sub-family member 12	221019_s_at	1	7.997	0.74	7	6.304	1.04	40	d	0.000214
SLC2A1	2	solute carrier family 2 (facilitated glucose transporter), member 1	201249_at	2	2.599	0.32	7	2.67	0.38	40	u	0.651025
			201250_s_at	2	5.153	0.39	7	6.328	0.97	40	u	0.00336
RGS1	2	regulator of G-protein signaling 1	216834_at	2	8.497	0.67	7	9.927	1.08	40	u	0.001824
			202988_s_at	2	6.656	0.83	7	8.083	1.37	40	u	0.012026
			202989_at	2	3.19	0.09	7	3.392	0.26	40	u	0.001511
SRRM1	2	serine/arginine repetitive matrix 1	201224_s_at	2	7.73	0.34	7	7.647	0.6	40	d	0.729157
			201225_s_at	2	10.53	0.23	7	9.855	0.48	40	d	0.000909
PPP1CA	2	protein phosphatase 1, catalytic subunit, alpha isoform	200846_s_at	3	10.05	0.26	7	10.47	0.7	40	u	0.011957
TRIP11	2	thyroid hormone receptor interactor 11	209778_at	2	6.47	0.23	7	6.377	0.65	40	d	0.516143
			210760_x_at	0	2.339	0.11	7	2.391	0.14	40	u	0.366651
ECT2	2	epithelial cell transforming sequence 2 oncogene	219787_s_at	2	5.922	0.78	7	8.798	0.93	40	u	0
			234992_x_at	0	2.762	0.5	7	4.317	1.41	40	u	0.000031
			237241_at	0	2.344	0.06	7	2.672	0.54	40	u	0.000698
SYP	2	synaptophysin	213200_at	0	5.297	0.29	7	5.546	0.7	40	u	0.368713
SLC25A42	2	solute carrier family 25, member 42	242274_x_at	0	2.849	0.2	7	2.997	0.28	40	u	0.200135
			226737_at	0	6.128	0.26	7	5.891	0.28	40	d	0.047172
			228921_at	0	2.091	0.14	7	2.231	0.42	40	u	0.12725
			214029_at	0	1.937	0.02	7	1.982	0.09	40	u	0.009727
C10orf119	2	chromosome 10 open reading frame 119	217905_at	2	4.779	0.39	7	5.284	0.63	40	u	0.048567
			233028_at	0	2.361	0.06	7	2.483	0.11	40	u	0.007249
			222464_s_at	0	8.229	0.29	7	8.942	0.75	40	u	0.019066
B3GALT2	2	UDP-Gal:betaGlcNAc beta 1,3-galactosyltransferase, polypeptide 2	210121_at	2	2.396	0.04	7	2.496	0.14	40	u	0.001147
			217452_s_at	2	2.943	0.11	7	3.138	0.27	40	u	0.075359
GREM2	2	gremlin 2, cysteine knot superfamily, homolog (Xenopus laevis)	235504_at	2	2.341	0.21	7	2.317	0.13	40	d	0.70233
			220794_at	1	3.739	1.01	7	2.578	0.63	40	d	0.000252

		homolog (Xenopus laevis)	240509_s_at	0	3.037	0.53	7	2.869	0.39	40	d	0.332424
CTR9	2	Ctr9, Paf1/RNA polymerase II complex component, homolog (S. cerevisiae)	202060_at	2	9.186	0.22	7	8.736	0.54	40	d	0.040051
			228190_at	0	6.629	0.15	7	6.78	0.8	40	u	0.291927
FOXD2	2	forkhead box D2	207653_at	0	2.707	0.23	7	2.736	0.37	40	u	0.846104
PTTG1IP	2	pituitary tumor-transforming 1 interacting protein	200677_at	2	11.67	0.2	7	11.68	0.61	40	u	0.978158
ADARB1	2	adenosine deaminase, RNA-specific, B1 (RED1 homolog rat)	203865_s_at	2	6.778	0.47	7	5.883	1.12	40	d	0.048239
			207999_s_at	0	3.995	0.34	7	4.2	0.36	40	u	0.17338
			234539_at	0	2.607	0.51	7	2.535	0.41	40	d	0.690248
			234799_at	0	2.226	0.22	7	2.231	0.3	40	u	0.966929
			209979_at	0	5.535	0.41	7	4.854	0.61	40	d	0.008337
COL4A2	2	collagen, type IV, alpha 2	211966_at	2	8.427	0.65	7	7.518	1.68	40	d	0.172798
			211964_at	2	10.41	0.52	7	9.638	1.07	40	d	0.074564
FAM126A	2	family with sequence similarity 126, member A	244115_at	2	3.214	1.03	7	2.831	0.6	40	d	0.410768
			223625_at	0	4.222	0.64	7	3.75	1.2	40	d	0.32808
			227239_at	0	9.304	0.35	7	6.988	1.11	40	d	0
			231396_s_at	0	2.716	0.54	7	3.045	0.92	40	u	0.374809
CGNL1	2	cingulin-like 1	225817_at	2	9.14	0.32	7	6.294	1.52	40	d	0
			1569847_at	0	3.144	0.18	7	3.115	0.3	40	d	0.807197
COL11A2	2	collagen, type XI, alpha 2	213870_at	0	3.424	0.41	7	4.181	1.47	40	u	0.01283
			216993_s_at	0	6.173	0.2	7	6.431	0.63	40	u	0.055733
C1orf119	2	chromosome 1 open reading frame 119	222495_at	4	9.796	0.28	7	9.362	0.65	40	d	0.097812
SLC2A14	2	rier family 2 (facilitated glucose transporter), m	no probeset available									
IL17RD	2	interleukin 17 receptor D	229263_at	2	4.986	0.5	7	4.642	0.67	40	d	0.208513
			227997_at	0	9.443	0.9	7	7.035	1.38	40	d	0.000073
ZNF365	2	zinc finger protein 365	206448_at	2	3.075	0.72	7	3.713	1.26	40	u	0.208391
			1570121_at	0	2.268	0.03	7	2.326	0.04	40	u	0.002027
RLBP1L1	2	retinaldehyde binding protein 1-like 1	230217_at	0	2.032	0.02	7	2.086	0.06	40	u	0.000299
KCTD4	2	potassium channel tetramerisation domain containing 4	239787_at	2	2.59	0.36	7	2.551	0.28	40	d	0.749415
			240512_x_at	2	3.011	0.36	7	3.075	0.29	40	u	0.611693
PLCH1	2	phospholipase C, eta 1	214745_at	2	5.53	0.79	7	6.364	1.33	40	u	0.123217
			216634_at	0	2.442	0.04	7	2.54	0.11	40	u	0.022991
			216633_s_at	0	3.122	0.17	7	3.357	0.56	40	u	0.045371
MPZ	2	myelin protein zero (Charcot-Marie-Tooth neuropathy 1B)	210280_at	0	2.593	0.17	7	2.804	0.84	40	u	0.170234
HS3ST3B1	2	heparan sulfate (glucosamine) 3-O-sulfotransferase 3B1	221062_at	2	2.769	0.09	7	2.845	0.13	40	u	0.148973
			227361_at	0	4.71	0.22	7	5.019	1.04	40	u	0.108548
			1561908_a_at	0	3.783	0.27	7	4.171	0.59	40	u	0.103474
CTNS	2	cystinosis, nephropathic	36566_at	2	6.04	0.21	7	5.788	0.54	40	d	0.242236
			204925_at	2	5.274	0.11	7	4.918	0.49	40	d	0.000337
			215459_at	0	2.529	0.1	7	2.594	0.21	40	u	0.428579
FAM102A	2	family with sequence similarity 102, member A	212400_at	2	8.964	0.32	7	8.775	0.85	40	d	0.569836
NEUROD2	2	neurogenic differentiation 2	210271_at	2	2.089	0.07	7	2.213	0.14	40	u	0.030063
			1552953_a_at	2	3.202	0.27	7	3.384	0.35	40	u	0.201468
BRPF1	2	bromodomain and PHD finger containing, 1	204481_at	2	6.696	0.34	7	6.574	0.69	40	d	0.653832
AMFR	2	autocrine motility factor receptor	202203_s_at	2	7.395	1.57	7	5.834	1.82	40	d	0.042896
			202204_s_at	1	7.345	0.48	7	6.459	1.07	40	d	0.040917
MAPK1	2	mitogen-activated protein kinase 1	1552264_a_at	0	6.166	0.53	7	6.873	1.24	40	u	0.152538
			212271_at	0	8.186	0.34	7	8.763	1.09	40	u	0.015103
			208351_s_at	0	5.872	0.55	7	6.809	1.34	40	u	0.081027
			1552263_at	0	4.478	0.39	7	5.053	0.79	40	u	0.070775
			224621_at	0	10.19	0.27	7	10.01	0.61	40	d	0.454225
			224620_at	0	2.767	0.18	7	2.822	0.27	40	u	0.61225
			229847_at	0	3.359	0.45	7	3.209	0.56	40	d	0.512519
ZNF322A	2	zinc finger protein 322A	227980_at	0	8.798	0.27	7	8.633	0.57	40	d	0.464369
TNRC4	2	trinucleotide repeat containing 4	206817_x_at	0	4.221	0.69	7	3.741	0.59	40	d	0.064929
			215045_at	0	4.709	0.48	7	4.883	0.64	40	u	0.504698
SYBL1	2	synaptobrevin-like 1	202829_s_at	4	9.267	0.32	7	8.992	1.13	40	d	0.229159
TMEM166	2	transmembrane protein 166	227828_s_at	2	5.207	0.6	7	5.101	0.96	40	d	0.783186
SPOP	2	speckle-type POZ protein	238923_at	2	3.38	0.45	7	3.662	0.96	40	u	0.457425
			208927_at	1	8.816	0.4	7	9.033	0.88	40	u	0.531608
			204640_s_at	0	9.224	0.2	7	8.907	0.79	40	d	0.043299

ZNF706	2	zinc finger protein 706	227132_at	0	6.882	0.36	7	6.914	1.2	40	u	0.895413
			218059_at	0	10.5	0.29	7	10.88	0.71	40	u	0.176622
SMAD1	2	SMAD family member 1	227798_at	2	7.45	0.46	7	7.911	0.6	40	u	0.063514
			210993_s_at	0	9.124	0.5	7	9.056	0.73	40	d	0.817152
			208015_at	0	5.008	0.87	7	4.605	0.99	40	d	0.328709
PLEKHK1	2	pleckstrin homology domain containing, family K member 1	230469_at	2	2.897	0.21	7	3.538	0.83	40	u	0.000277
			242047_at	0	2.854	0.18	7	3.101	0.34	40	u	0.072572
CCPG1	2	cell cycle progression 1	222156_x_at	2	6.625	0.59	7	5.908	0.98	40	d	0.072025
			221511_x_at	2	8.524	0.36	7	7.619	0.83	40	d	0.007816
			214151_s_at	1	7.441	0.44	7	6.478	1.04	40	d	0.022434
			214152_at	1	7.641	0.26	7	7.038	0.83	40	d	0.001386
			221156_x_at	0	6.342	0.42	7	6.116	0.65	40	d	0.387613
IL17F	2	interleukin 17F	234408_at	0	2.258	0.02	7	2.322	0.08	40	u	0.000367
C1QTNF6	2	C1q and tumor necrosis factor related protein 6	223571_at	2	4.44	0.4	7	4.952	0.73	40	u	0.084206
			242444_at	0	3.765	0.7	7	4.182	0.8	40	u	0.210841
MARK2	2	MAP/microtubule affinity-regulating kinase 2	203942_s_at	2	6.15	0.47	7	5.965	0.86	40	d	0.588131
			211082_x_at	0	3.318	0.4	7	3.899	0.89	40	u	0.101897
C14orf32	2	chromosome 14 open reading frame 32	212497_at	2	4.243	0.61	7	3.707	0.92	40	d	0.153306
			212644_s_at	2	8.746	0.27	7	8.405	0.61	40	d	0.159618
			212643_at	2	10.63	0.21	7	10.15	0.56	40	d	0.031799
			212499_s_at	2	7.76	0.21	7	7.963	0.63	40	u	0.134252
			225643_at	0	7.833	0.29	7	7.081	0.61	40	d	0.003213
ATF2	2	activating transcription factor 2	205446_s_at	2	6.029	0.5	7	6.103	0.9	40	u	0.835126
			212984_at	0	8.664	0.26	7	8.652	0.54	40	d	0.956934
			1555146_at	0	2.628	0.06	7	2.787	0.2	40	u	0.000432
EHD4	2	EH-domain containing 4	233660_at	2	4.489	0.72	7	4.104	0.81	40	d	0.254546
			209536_s_at	2	8.609	0.3	7	8.069	0.52	40	d	0.012369
			1556608_a_at	0	4.523	0.73	7	3.324	0.46	40	d	0.000001
			1556607_at	0	6.527	0.59	7	5.609	0.59	40	d	0.00054
SYNCRIP	2	synaptotagmin binding, cytoplasmic RNA interacting protein	209025_s_at	2	8.318	0.37	7	8.999	0.67	40	u	0.013254
			209024_s_at	2	9.404	0.2	7	10.68	0.77	40	u	0
			217834_s_at	0	6.994	0.35	7	8.56	0.91	40	u	0.000061
			236146_at	0	4.101	0.64	7	4.098	0.93	40	d	0.992459
			217832_at	0	9.229	0.26	7	9.696	0.72	40	u	0.006746
			1555427_s_at	0	6.065	0.51	7	6.498	1.03	40	u	0.291886
			217833_at	0	10.11	0.48	7	9.488	0.91	40	d	0.090948
WNT11	2	wingless-type MMTV integration site family, member 11	206737_at	2	3.213	0.68	7	3.458	1.07	40	u	0.570111
MTAP	2	methylthioadenosine phosphorylase	231984_at	2	5.779	0.4	7	5.865	1.28	40	u	0.745318
			204956_at	0	4.375	0.33	7	5.216	1	40	u	0.000437
			216685_s_at	0	6.241	0.23	7	6.487	1.09	40	u	0.224031
			217134_at	0	2.723	0.11	7	2.897	0.41	40	u	0.037302
			211364_at	0	2.054	0.05	7	2.267	0.38	40	u	0.001705
			211363_s_at	0	2.835	0.36	7	3.454	0.97	40	u	0.109051
EBF2	2	early B-cell factor 2	220392_at	2	2.481	0.2	7	2.429	0.08	40	d	0.545391
			1562477_at	0	2.927	0.08	7	2.965	0.19	40	u	0.608222
HSP90AB1	2	heat shock protein 90kDa alpha (cytosolic), class B member 1	214359_s_at	2	11.7	0.18	7	11.75	1.29	40	u	0.812887
			1557910_at	0	11.39	0.46	7	11.57	1.75	40	u	0.591586
			200064_at	0	12.99	0.14	7	12.98	0.74	40	d	0.914917
SLC25A20	2	solute carrier family 25 (carnitine/acylcarnitine translocase), member 20	203658_at	2	7.764	0.35	7	7.034	0.74	40	d	0.016005
ERO1LB	2	ERO1-like beta (S. cerevisiae)	231944_at	2	7.383	0.55	7	6.18	1.13	40	d	0.009488
			220012_at	0	4.224	0.61	7	4.048	0.86	40	d	0.614671
PMPCB	2	peptidase (mitochondrial processing) beta	201682_at	2	9.623	0.16	7	9.353	0.57	40	d	0.023348
NID2	2	nidogen 2 (osteonidogen)	204114_at	2	6.049	0.71	7	6.265	1.21	40	u	0.65526
EHMT1	2	euchromatic histone-lysine N-methyltransferase 1	225461_at	2	7.259	0.46	7	7.227	0.78	40	d	0.920006
			222873_s_at	0	5.075	0.51	7	5.656	0.99	40	u	0.145128
			219339_s_at	0	4.411	0.48	7	4.591	0.63	40	u	0.48388
PI4K2B	2	phosphatidylinositol 4-kinase type 2 beta	222631_at	2	7.405	0.46	7	8.173	0.75	40	u	0.013954
SRCRB4D	2	scavenger receptor cysteine rich domain containing, group B (4 domains)	236529_at	2	6.333	0.33	7	6.074	0.38	40	d	0.106193
ST5	2	suppression of tumorigenicity 5	202440_s_at	2	8.937	0.57	7	7.219	0.99	40	d	0.000072
		membrane protein, palmitoylated 5 (MAGUK	226092_at	2	8.053	0.34	7	7.323	0.95	40	d	0.001679

MPP5	2	membrane protein, palmitoylated 5 (MAGUK p55 subfamily member 5)	219321_at	0	5.981	0.56	7	6.167	1.15	40	u	0.68499
			235864_at	0	4.394	0.37	7	4.874	0.35	40	u	0.002168
RB1	2	retinoblastoma 1 (including osteosarcoma)	203132_at	2	8.512	0.23	7	8.282	1.16	40	d	0.273904
			211540_s_at	0	4.301	0.18	7	4.459	0.7	40	u	0.249104
COL16A1	2	collagen, type XVI, alpha 1	204345_at	2	8.882	0.4	7	6.88	0.96	40	d	0.000003
RNF24	2	ring finger protein 24	204669_s_at	2	7.653	0.31	7	7.24	1.07	40	d	0.061294
			204668_at	2	2.724	0.35	7	3.416	0.68	40	u	0.013594
			210706_s_at	2	8.125	0.21	7	7.863	1.04	40	d	0.170262
PDXDC1	2	pyridoxal-dependent decarboxylase domain containing 1	1555347_at	0	3.882	0.42	7	3.897	0.62	40	u	0.951511
			212053_at	0	9.553	0.42	7	9.926	0.78	40	u	0.230581
			232288_at	0	4.76	0.61	7	5.563	0.85	40	u	0.023564
			237354_at	0	2.311	0.23	7	2.375	0.25	40	u	0.536195
			1560014_s_at	0	4.737	0.5	7	4.737	0.98	40	d	0.999461
			1560013_at	0	4.881	0.45	7	5.061	0.86	40	u	0.600219
MYST4	2	MYST histone acetyltransferase (monocytic leukemia) 4	212462_at	2	8.157	0.28	7	7.595	1.1	40	d	0.011372
			212452_x_at	2	5.888	0.47	7	5.446	1.03	40	d	0.280903
			214496_x_at	0	6.286	0.38	7	5.997	1.06	40	d	0.22165
			211874_s_at	0	5.788	0.53	7	5.222	1.16	40	d	0.220623
			1562236_at	0	4.075	0.31	7	4.32	0.46	40	u	0.190658
FBXL7	2	F-box and leucine-rich repeat protein 7	213249_at	2	7.591	0.31	7	6.965	1.18	40	d	0.009253
PPRC1	2	peroxisome proliferator-activated receptor gamma, coactivator-related 1	203737_s_at	2	7.737	0.37	7	7.386	0.71	40	d	0.217753
CBARA1	2	calcium binding atopy-related autoantigen 1	216903_s_at	2	8.56	0.14	7	8.042	0.43	40	d	0.000004
TMEM87A	2	transmembrane protein 87A	212202_s_at	2	10.6	0.18	7	10.08	0.83	40	d	0.001455
			212204_at	2	11	0.19	7	10.46	0.73	40	d	0.000432
			223771_at	0	3.197	0.15	7	3.343	0.4	40	u	0.356694
			223772_s_at	0	2.52	0.08	7	2.817	0.5	40	u	0.001203
P4HA1	2	procollagen-proline, 2-oxoglutarate 4-dioxygenase (proline 4-hydroxylase), alpha polypeptide I	207543_s_at	2	8.653	0.28	7	9.035	0.71	40	u	0.174118
GLS	2	glutaminase	203159_at	2	8.817	0.33	7	7.793	0.99	40	d	0.000039
			203158_s_at	1	5.354	0.44	7	5.349	0.89	40	d	0.987557
			203157_s_at	1	6.458	0.38	7	5.572	1.19	40	d	0.001234
			223080_at	0	6.181	0.65	7	5.871	0.66	40	d	0.268107
			221510_s_at	0	7.407	0.46	7	8.12	1.18	40	u	0.129085
			223079_s_at	0	5.26	0.69	7	6.82	1.66	40	u	0.02101
			211414_at	0	2.325	0.03	7	2.548	0.31	40	u	0.000099
			241034_at	0	2.328	0.04	7	2.398	0.1	40	u	0.087611
SEC11A	2	SEC11 homolog A (S. cerevisiae)	201290_at	2	12	0.15	7	11.72	0.53	40	d	0.013155
			216274_s_at	0	11.32	0.19	7	10.87	0.75	40	d	0.00291
SHROOM4	2	shroom family member 4	244825_at	4	4.035	0.44	7	3.491	0.68	40	d	0.050234
EPS8L2	2	EPS8-like 2	218180_s_at	2	8.597	0.2	7	7.804	1.4	40	d	0.001888
			222546_s_at	2	3.549	0.61	7	3.676	1.64	40	u	0.842733
			229020_x_at	0	2.055	0.16	7	2.069	0.14	40	u	0.812809
C9orf82	2	chromosome 9 open reading frame 82	231995_at	2	4.532	0.58	7	4.23	0.72	40	d	0.307142
			219276_x_at	2	8.006	0.39	7	7.648	1	40	d	0.365899
EMILIN2	2	elastin microfibril interfacer 2	221980_at	2	3.644	0.14	7	3.704	0.2	40	u	0.454068
			224374_s_at	2	6.34	0.83	7	5.443	1.2	40	d	0.069032
			242288_s_at	0	5.35	0.39	7	4.934	0.53	40	d	0.05884
OR51E2	2	olfactory receptor, family 51, subfamily E, member 2	236121_at	2	2.023	0.03	7	2.102	0.12	40	u	0.001021
			221424_s_at	0	2.126	0.02	7	2.166	0.05	40	u	0.062099
			232482_at	0	2.423	0.11	7	2.463	0.13	40	u	0.446423
TMEM115	2	transmembrane protein 115	216267_s_at	2	7.135	0.31	7	6.406	0.85	40	d	0.000652
WDR45L	2	WDR45-like	209076_s_at	2	10.32	0.33	7	10.22	0.58	40	d	0.66751
DZIP1L	2	DAZ interacting protein 1-like	239785_at	2	5.909	0.28	7	5.374	0.72	40	d	0.062956
SETD1A	2	SET domain containing 1A	213202_at	2	6.316	0.3	7	5.699	0.59	40	d	0.011771
HNF1B	2	HNF1 homeobox B	208135_at	2	2.244	0.02	7	2.316	0.15	40	u	0.007679
			205313_at	0	3.208	0.37	7	3.768	0.53	40	u	0.011307
GTF2I	2	general transcription factor II, i	210891_s_at	2	11.91	0.26	7	11.55	0.66	40	d	0.159169
			201065_s_at	2	11.1	0.34	7	11.09	0.68	40	d	0.971026
			210892_s_at	0	6.87	0.61	7	7.495	1.35	40	u	0.245615
ZNF746	2	zinc finger protein 746	225848_at	2	7.009	0.22	7	6.912	0.77	40	d	0.53139
			1570373_at	0	2.008	0.05	7	2.268	0.68	40	u	0.022917

GORASP2	2	golgi reassembly stacking protein 2, 55kDa	208842_s_at	2	9.061	0.2	7	9.283	0.75	40	u	0.13467
			207812_s_at	2	9.35	0.15	7	9.695	0.57	40	u	0.003533
			208843_s_at	2	9.307	0.29	7	9.632	0.57	40	u	0.156287
GABRG2	2	gamma-aminobutyric acid (GABA) A receptor, gamma 2	1568612_at	2	2.554	0.12	7	2.607	0.12	40	u	0.288127
			206849_at	0	2.139	0.1	7	2.225	0.35	40	u	0.226865
FAM78B	2	family with sequence similarity 78, member B	1558113_at	2	3.876	0.66	7	3.895	0.55	40	u	0.935886
NDRG4	2	NDRG family member 4	209159_s_at	2	3.722	0.3	7	3.99	1.2	40	u	0.247781
LCP1	2	lymphocyte cytosolic protein 1 (L-plastin)	208885_at	2	8.036	0.52	7	8.398	1.22	40	u	0.453828
TRAK1	2	trafficking protein, kinesin binding 1	226013_at	2	6.831	0.61	7	6.211	1.08	40	d	0.155369
			214924_s_at	2	8.298	0.44	7	7.402	1.1	40	d	0.042873
			201283_s_at	0	6.189	0.69	7	5.603	1.12	40	d	0.194796
			202079_s_at	0	7.074	0.42	7	6.531	1.01	40	d	0.179322
			202080_s_at	0	8.408	0.44	7	7.502	0.92	40	d	0.016154
TCTEX1D1	2	Tctex1 domain containing 1	1553635_s_at	2	3.235	0.27	7	3.385	0.37	40	u	0.322314
RHPN2	2	rhophilin, Rho GTPase binding protein 2	227196_at	2	7.568	0.73	7	7.701	1.17	40	u	0.776982
C9orf40	2	chromosome 9 open reading frame 40	222781_s_at	2	6.34	0.36	7	7.02	1.03	40	u	0.004851
			218904_s_at	2	4.261	0.52	7	4.793	0.89	40	u	0.13873
RBM39	2	RNA binding motif protein 39	208720_s_at	2	11.48	0.2	7	10.9	0.55	40	d	0.000059
			207941_s_at	2	11.89	0.24	7	11.4	0.66	40	d	0.002324
			226404_at	0	4.2	0.4	7	5.015	0.88	40	u	0.022754
			238357_at	0	2.222	0.01	7	2.321	0.16	40	u	0.000368
			227223_at	0	8.127	0.7	7	8.686	0.91	40	u	0.136364
			238355_at	0	2.628	0.07	7	2.759	0.13	40	u	0.013217
MLL2	2	myeloid/lymphoid or mixed-lineage leukemia 2	227528_s_at	0	6.938	0.64	7	6.564	0.9	40	d	0.307903
			227527_at	0	8.161	0.56	7	7.093	1.39	40	d	0.055903
			211790_s_at	0	2.751	0.26	7	3.046	0.66	40	u	0.261162
			231974_at	0	7.472	0.39	7	7.489	0.6	40	u	0.942372
STX1B	2	syntaxin 1B	230691_at	2	2.08	0.07	7	2.09	0.1	40	u	0.801267
NFXL1	2	nuclear transcription factor, X-box binding-like 1	227220_at	2	7.431	0.13	7	7.593	0.59	40	u	0.140793
FBXO38	2	F-box protein 38	221257_x_at	2	8.199	0.41	7	7.753	0.99	40	d	0.257997
			224369_s_at	2	9.044	0.3	7	8.615	0.71	40	d	0.130199
			219608_s_at	0	4.596	0.55	7	5.013	0.81	40	u	0.204819
NHLH1	2	nescient helix loop helix 1	214628_at	1	2.884	0.45	7	3.02	0.49	40	u	0.50667
VGF	2	VGF nerve growth factor inducible	205586_x_at	2	5.242	0.07	7	5.381	0.36	40	u	0.037646
RBM15B	2	RNA binding motif protein 15B	226987_at	2	7.231	0.43	7	6.583	0.75	40	d	0.035141
			202689_at	1	8.077	0.29	7	7.903	0.61	40	d	0.472812
IL12B	2	interleukin 12B (natural killer cell stimulatory factor 2, cytotoxic lymphocyte maturation factor 2, p40)	207901_at	2	3.328	0.21	7	3.534	0.47	40	u	0.270159
FAM57B	2	family with sequence similarity 57, member B	227781_x_at	2	5.855	0.54	7	5.747	0.53	40	d	0.630385
ITGA7	2	integrin, alpha 7	216331_at	2	7.694	0.59	7	5.219	1.04	40	d	0
			209663_s_at	0	6.371	0.77	7	3.913	0.82	40	d	0
EXOC7	2	exocyst complex component 7	215413_at	2	2.935	0.27	7	2.98	0.3	40	u	0.716335
			212026_s_at	2	6.188	0.34	7	5.191	1.16	40	d	0.000163
			212034_s_at	2	9.675	0.15	7	9.123	0.57	40	d	0.000016
			212035_s_at	2	7.145	0.21	7	6.956	0.91	40	d	0.271774
			216510_x_at	0	7.166	1.37	7	6.632	2.69	40	d	0.61765
			214916_x_at	0	8.161	1.44	7	7.117	2.26	40	d	0.252793
			214802_at	0	7.26	0.51	7	6.637	0.92	40	d	0.094018
PRELP	2	proline/arginine-rich end leucine-rich repeat protein	228224_at	2	6.044	0.87	7	4.296	1.12	40	d	0.000382
			37022_at	1	6.558	0.6	7	5.732	0.69	40	d	0.005711
			204223_at	0	7.462	0.77	7	5.745	1.25	40	d	0.001284
UBTF	2	upstream binding transcription factor, RNA polymerase I	225982_at	2	7.824	0.32	7	7.656	0.48	40	d	0.38767
			202692_s_at	1	8.133	0.29	7	7.676	0.37	40	d	0.003836
			1558215_s_at	0	6.351	0.37	7	6.588	0.77	40	u	0.435274
			214881_s_at	0	6.905	0.3	7	7.451	0.47	40	u	0.006039
PHB	2	prohibitin	200658_s_at	2	9.396	0.28	7	9.656	0.91	40	u	0.170055
			200659_s_at	2	8.972	0.18	7	8.492	0.84	40	d	0.003301
SEMA6B	2	sema domain, transmembrane domain (TM), and cytoplasmic domain, (semaphorin) 6B	220778_x_at	2	4.711	0.47	7	4.426	0.57	40	d	0.223024
			223567_at	2	3.97	0.39	7	3.904	0.74	40	d	0.820291
			224182_x_at	2	3.22	0.28	7	2.701	0.54	40	d	0.019028

MLLT11	2	myeloid/lymphoid or mixed-lineage leukemia (trithorax homolog, Drosophila); translocated to, 11	211071_s_at	2	6.759	0.6	7	7.918	1.55	40	u	0.061989
CNNM2	2	cyclin M2	209874_x_at	2	3.149	0.56	7	3.304	0.55	40	u	0.507479
			206818_s_at	2	3.033	0.48	7	3.147	0.58	40	u	0.632734
			1554523_a_at	0	3.066	0.18	7	3.22	0.44	40	u	0.379729
			1554522_at	0	2.635	0.15	7	2.806	0.28	40	u	0.127745
ADNP2	2	ADNP homeobox 2	203321_s_at	2	7.214	0.4	7	7.613	0.62	40	u	0.116027
			203322_at	2	8.521	0.33	7	8.112	0.61	40	d	0.099945
C4orf16	2	chromosome 4 open reading frame 16	219023_at	1	6.938	0.44	7	7.702	0.88	40	u	0.03234
			242753_x_at	0	3.634	0.3	7	4.394	0.97	40	u	0.000655
			231010_at	0	2.143	0.13	7	2.39	0.42	40	u	0.007605
AKAP11	2	A kinase (PRKA) anchor protein 11	203156_at	2	9.629	0.16	7	8.66	0.73	40	d	0
			215336_at	0	6.656	0.3	7	5.924	0.62	40	d	0.004688
SGK3	2	serum/glucocorticoid regulated kinase family, member 3	227627_at	2	10.12	0.58	7	8.897	1.09	40	d	0.006784
			220038_at	2	7.948	0.75	7	7.159	1.56	40	d	0.205388
GCN1L1	2	GCN1 general control of amino-acid synthesis 1-like 1 (yeast)	212139_at	2	7.666	0.33	7	7.432	0.78	40	d	0.451645
			216232_s_at	2	7.642	0.36	7	7.225	0.58	40	d	0.076803
ETV1	2	ets variant gene 1	221911_at	9	7.628	0.56	7	5.536	1.46	40	d	0.000649
			206501_x_at	2	4.654	0.63	7	3.809	0.87	40	d	0.020872
			217061_s_at	0	4.917	0.36	7	3.991	0.91	40	d	0.012493
			217053_x_at	0	3.406	0.68	7	2.657	0.63	40	d	0.007628
IGSF22	2	immunoglobulin superfamily, member 22	1553857_at	5	4.608	0.46	7	4.369	0.63	40	d	0.354479
ERGIC2	2	ERGIC and golgi 2	226418_at	2	3.336	0.49	7	3.883	0.56	40	u	0.022162
			226422_at	2	8.381	0.19	7	9.336	0.62	40	u	0
			218135_at	1	9.027	0.25	7	9.812	0.77	40	u	0.000038
			240172_at	0	6.249	0.3	7	5.675	0.82	40	d	0.003922
CYBRD1	2	cytochrome b reductase 1	222453_at	2	10.92	0.59	7	8.752	1.29	40	d	0.0001
			217889_s_at	0	7.897	0.89	7	6.671	1.82	40	d	0.093684
CTTNBP2NL	2	CTTNBP2 N-terminal like	226000_at	2	8.519	0.24	7	8.593	0.65	40	u	0.774981
			231822_at	2	6.116	0.49	7	6.243	1.23	40	u	0.792591
			239525_at	0	3.695	0.34	7	4.177	0.67	40	u	0.07708
			240868_at	0	3.558	0.49	7	3.719	0.52	40	u	0.462965
			214731_at	0	6.827	0.6	7	5.87	1.13	40	d	0.038251
PPP2R3A	2	protein phosphatase 2 (formerly 2A), regulatory subunit B'', alpha	207749_s_at	2	6.542	0.47	7	6.932	1.11	40	u	0.373557
			209632_at	2	7.112	0.33	7	7.167	0.86	40	u	0.870812
			209633_at	2	8.186	0.37	7	7.805	0.8	40	d	0.23538
SPATA8	2	spermatogenesis associated 8	231006_at	2	2.499	0.03	7	2.611	0.2	40	u	0.002264
MUM1	2	melanoma associated antigen (mutated) 1	223347_at	0	7.847	0.28	7	6.982	0.83	40	d	0.000038
			229033_s_at	0	6.724	0.47	7	6.054	0.98	40	d	0.088326
			223348_x_at	0	6.834	0.33	7	6.037	0.75	40	d	0.009354
			221290_s_at	0	6.942	0.26	7	5.991	0.84	40	d	0.000005
			230461_s_at	0	4.352	0.32	7	4.754	0.86	40	u	0.239246
FAM45A	2	family with sequence similarity 45, member A	221804_s_at	2	9.379	0.29	7	9.432	0.47	40	u	0.776596
			225351_at	2	7.502	0.28	7	7.942	0.46	40	u	0.021802
			1563920_at	0	3.061	0.15	7	3.172	0.13	40	u	0.054554
			222955_s_at	0	7.152	0.3	7	7.363	0.69	40	u	0.443228
			1562919_at	0	2.626	0.03	7	2.709	0.09	40	u	0.00008
RANBP9	2	RAN binding protein 9	202582_s_at	2	9.705	0.26	7	10.26	0.57	40	u	0.015629
			202583_s_at	0	7.892	0.36	7	8.361	1.4	40	u	0.089155
			216125_s_at	0	6.229	0.58	7	7.162	1.7	40	u	0.015773
SYNGR1	2	synaptogyrin 1	204287_at	2	6.415	0.33	7	5.744	1.05	40	d	0.004338
			210613_s_at	0	7.137	0.38	7	6.353	1.78	40	d	0.020138
			213854_at	0	4.371	0.5	7	4.266	0.89	40	d	0.769201
HSPA12A	2	heat shock 70kDa protein 12A	214434_at	2	5.236	0.65	7	4.405	0.94	40	d	0.032428
			220763_at	0	2.143	0.02	7	2.201	0.14	40	u	0.021526
REPIN1	2	replication initiator 1	219041_s_at	2	9.664	0.27	7	9.528	0.94	40	d	0.471618
			222501_s_at	2	3.587	0.35	7	4.496	1.53	40	u	0.00279
			228815_s_at	2	1.948	0.02	7	2.027	0.17	40	u	0.007767
XYLT1	2	xylosyltransferase I	232574_at	2	2.288	0.03	7	2.486	0.41	40	u	0.00529
			213725_x_at	0	6.42	0.46	7	6.672	1.46	40	u	0.407907
SYAP1	2	synapse associated protein 1, SAP47 homolog (Drosophila)	225154_at	2	8.827	0.41	7	9.486	0.74	40	u	0.029201
UCK2	2	uridine-cytidine kinase 2	209825_s_at	1	7.07	0.27	7	8.439	1.07	40	u	0

CDYL	2	chromodomain protein, Y-like	203098_at	2	7.903	0.18	7	8.102	0.73	40	u	0.15731
			203100_s_at	0	6.649	0.23	7	7.126	1.08	40	u	0.019209
			203099_s_at	0	6.812	0.17	7	7.162	0.76	40	u	0.016744
MLXIP	2	MLX interacting protein	202519_at	2	8.47	0.57	7	7.719	0.72	40	d	0.013667
			211789_s_at	0	2.604	0.51	7	2.947	0.88	40	u	0.333846
			1554886_a_at	0	3.225	0.12	7	3.524	0.46	40	u	0.001562
			225157_at	0	7.924	0.58	7	7.276	0.79	40	d	0.048513
PLOD1	2	procollagen-lysine 1, 2-oxoglutarate 5-dioxygenase 1	200827_at	2	8.105	0.25	7	8.243	0.89	40	u	0.433535
ADRA2C	2	adrenergic, alpha-2C-, receptor	206128_at	2	6.871	0.18	7	6.786	0.43	40	d	0.61361
MYO1B	2	myosin IB	212364_at	2	8.763	0.27	7	8.361	0.82	40	d	0.026521
			212365_at	2	8.228	0.32	7	8.171	0.83	40	d	0.859392
			215074_at	0	3.805	0.4	7	3.759	0.58	40	d	0.844363
VAMP8	2	vesicle-associated membrane protein 8 (endobrevin)	202546_at	0	10.92	0.33	7	10.78	0.75	40	d	0.633132
ZSCAN18	2	zinc finger and SCAN domain containing 18	218312_s_at	2	9.622	0.18	7	8.031	1.08	40	d	0
			217593_at	0	6.965	0.52	7	5.82	0.59	40	d	0.000025
			232866_at	0	2.362	0.22	7	2.418	0.45	40	u	0.757849
PLAU	2	plasminogen activator, urokinase	205479_s_at	2	7.64	0.51	7	8.188	1.08	40	u	0.205426
			211668_s_at	0	6.869	0.7	7	7.434	1.6	40	u	0.373119
ZNF189	2	zinc finger protein 189	207513_s_at	2	8.563	0.19	7	8.069	0.65	40	d	0.00067
COL22A1	2	collagen, type XXII, alpha 1	228873_at	2	2.563	0.24	7	3.301	1.32	40	u	0.002901
			243301_at	0	2.389	0.03	7	2.545	0.31	40	u	0.003962
FAM5C	2	family with sequence similarity 5, member C	217562_at	2	3.291	0.76	7	3.836	1.94	40	u	0.476801
DCK	2	deoxycytidine kinase	203302_at	2	6.934	0.47	7	8.177	0.87	40	u	0.000737
SLC6A4	2	solute carrier family 6 (neurotransmitter transporter, serotonin), member 4	207519_at	2	2.345	0.17	7	2.829	1.06	40	u	0.011355
PHLDA3	2	pleckstrin homology-like domain, family A, member 3	218634_at	0	7.155	0.36	7	6.127	0.99	40	d	0.011158
GOLM1	2	golgi membrane protein 1	217771_at	2	9.053	0.45	7	8.577	1.06	40	d	0.25809
			1558248_at	0	2.974	0.1	7	3.086	0.26	40	u	0.277748
GK	2	glycerol kinase	214681_at	2	5.437	0.41	7	5.804	0.78	40	u	0.237831
			207387_s_at	0	3.159	0.28	7	3.83	0.94	40	u	0.001379
			215977_x_at	0	3.755	0.52	7	4.698	0.95	40	u	0.015863
			216316_x_at	0	3.144	0.41	7	3.54	0.69	40	u	0.156524
			217167_x_at	0	4.445	0.36	7	5.017	0.82	40	u	0.083014
			215975_x_at	0	2.186	0.17	7	2.375	0.45	40	u	0.290801
AK3	2	adenylate kinase 3	224655_at	2	10.55	0.26	7	9.872	0.7	40	d	0.016344
			224151_s_at	0	8.266	0.34	7	8.144	0.72	40	d	0.670658
RBM35A	2	RNA binding motif protein 35A	219121_s_at	2	7.787	0.47	7	9.404	1.17	40	u	0.000963
			225846_at	0	9.057	0.42	7	10.25	0.83	40	u	0.000708
MDGA2	2	MAM domain containing glycosylphosphatidylinositol anchor 2	1570114_at	0	2.24	0.02	7	2.338	0.18	40	u	0.002548
			239935_at	0	2.986	0.08	7	3.241	0.34	40	u	0.000245
HES5	2	hairy and enhancer of split 5 (Drosophila)	239230_at	2	2.18	0.01	7	2.265	0.15	40	u	0.001028
BCAN	2	brevican	221623_at	2	1.763	0.03	7	1.82	0.09	40	u	0.008231
			223632_s_at	0	2.275	0.05	7	2.417	0.37	40	u	0.028056
			242843_at	0	2.405	0.13	7	2.412	0.09	40	u	0.873117
			223633_s_at	0	3.005	0.27	7	2.931	0.4	40	d	0.646718
			219107_at	0	4.206	0.61	7	4.347	0.6	40	u	0.579098
			91920_at	0	4.174	0.4	7	4.332	0.58	40	u	0.499005
FGF11	2	fibroblast growth factor 11	227271_at	2	5.02	0.45	7	4.978	0.93	40	d	0.907727
			231803_at	0	2.079	0.04	7	2.129	0.19	40	u	0.16741
STX2	2	syntaxin 2	213434_at	2	6.102	0.51	7	6.175	0.63	40	u	0.779242
			207346_at	0	2.531	0.24	7	2.699	0.39	40	u	0.291086
FAM46B	2	family with sequence similarity 46, member B	229518_at	2	6.913	0.71	7	5.196	1.41	40	d	0.003391
PKD2	2	polycystic kidney disease 2 (autosomal dominant)	203688_at	2	9.156	0.28	7	7.328	0.67	40	d	0
SQSTM1	2	sequestosome 1	201471_s_at	1	10.53	0.26	7	10.35	0.8	40	d	0.285117
			213112_s_at	1	5.787	0.32	7	5.744	0.62	40	d	0.864619
			217252_at	0	2.169	0.19	7	2.167	0.09	40	d	0.974469
			217255_at	0	3.073	0.29	7	3.099	0.36	40	u	0.860212
			244804_at	0	6.11	0.46	7	6.489	0.93	40	u	0.30674

			239004_at	0	4.887	0.34	7	4.873	0.61	40	d	0.952391
PROK2	2	prokineticin 2	232629_at	2	3.152	0.2	7	3.245	0.33	40	u	0.481097
MTHFR	2	5,10-methylenetetrahydrofolate reductase (NADPH)	226929_at	2	6.293	0.72	7	5.364	0.95	40	d	0.019545
			206800_at	1	4.126	0.32	7	4.233	0.49	40	u	0.586622
			217071_s_at	0	4.508	0.2	7	4.519	0.45	40	u	0.948826
			217070_at	0	5.781	0.19	7	5.849	0.34	40	u	0.611013
			239035_at	0	5.785	0.4	7	5.388	0.55	40	d	0.078689
RNF208	2	ring finger protein 208	221273_s_at	0	2.912	0.56	7	3.318	0.72	40	u	0.173141
PTPN11	2	protein tyrosine phosphatase, non-receptor type 11 (Noonan syndrome 1)	209895_at	2	7.32	0.35	7	7.975	0.93	40	u	0.07704
			209896_s_at	2	8.652	0.39	7	8.855	0.73	40	u	0.483169
			212610_at	2	10.16	0.24	7	10.32	0.49	40	u	0.416838
			205867_at	0	3.601	0.13	7	4.127	0.64	40	u	0.000041
			205868_s_at	0	3.919	0.41	7	4.737	1.1	40	u	0.063936
			1552637_at	0	2.086	0.12	7	2.108	0.08	40	u	0.540499
PLK3	2	polo-like kinase 3 (Drosophila)	204958_at	2	7.308	0.69	7	5.86	0.79	40	d	0.000053
			229825_at	0	2.345	0.08	7	2.399	0.11	40	u	0.221764
			215462_at	0	6.904	0.37	7	6.158	0.7	40	d	0.00962
ATF3	2	activating transcription factor 3	202672_s_at	2	10.8	0.9	7	8.286	1.36	40	d	0.000035
			1554420_at	1	5.31	1.11	7	4.658	0.96	40	d	0.120035
			1554980_a_at	0	7.974	1.01	7	6.433	1.17	40	d	0.002438
SLC2A13	2	solute carrier family 2 (facilitated glucose transporter), member 13	1552695_a_at	2	3.171	0.23	7	3.84	0.6	40	u	0.006585
			1552694_at	2	3.241	0.14	7	3.39	0.2	40	u	0.075462
			234268_at	0	2.707	0.09	7	2.953	0.45	40	u	0.004296
			234561_at	0	2.004	0.09	7	2.086	0.21	40	u	0.329598
			227176_at	0	5.367	0.67	7	5.401	1.05	40	u	0.936131
ZDHHC16	2	zinc finger, DHHC-type containing 16	223212_at	2	7.806	0.38	7	8.16	0.63	40	u	0.163511
RFTN1	2	raftlin, lipid raft linker 1	212646_at	2	8.455	0.81	7	8.778	1.38	40	u	0.559838
BTBD12	2	BTB (POZ) domain containing 12	232147_at	2	2.899	0.51	7	3.038	0.56	40	u	0.550034
			239687_at	0	3.995	0.27	7	3.933	0.43	40	d	0.718063
HIF3A	2	hypoxia inducible factor 3, alpha subunit	219319_at	2	3.254	0.39	7	3.011	0.49	40	d	0.226214
			222124_at	1	3.234	0.47	7	3.619	0.63	40	u	0.138989
			222123_s_at	1	3.177	0.44	7	2.774	0.24	40	d	0.072451
			1556069_s_at	0	2.993	0.64	7	2.84	0.62	40	d	0.559846
			233517_s_at	0	2.388	0.08	7	2.449	0.16	40	u	0.347042
			232669_at	0	2.304	0.02	7	2.365	0.07	40	u	0.000072
			1555318_at	0	3.349	0.45	7	3.436	0.51	40	u	0.680313
SRI	2	sorcin	208920_at	2	7.71	0.46	7	6.931	1.02	40	d	0.058274
			208921_s_at	1	10.55	0.32	7	10.43	0.73	40	d	0.663401
SGPL1	2	sphingosine-1-phosphate lyase 1	212322_at	2	8.192	0.38	7	8.858	0.66	40	u	0.015196
			212321_at	2	8.024	0.29	7	7.616	0.79	40	d	0.190758
			208381_s_at	0	1.865	0.02	7	2.107	0.49	40	u	0.003668
CREG2	2	cellular repressor of E1A-stimulated genes 2	1552714_at	2	2.408	0.04	7	2.513	0.27	40	u	0.027255
C5orf15	2	chromosome 5 open reading frame 15	203024_s_at	2	9.088	0.2	7	8.761	0.56	40	d	0.013298
			229260_at	0	2.691	0.16	7	2.848	0.51	40	u	0.141292
SEMA3A	2	sema domain, immunoglobulin domain (Ig), short basic domain, secreted, (semaphorin) 3A	206805_at	2	2.873	0.24	7	2.811	0.32	40	d	0.63184
			244849_at	0	2.412	0.03	7	2.498	0.08	40	u	0.006854
			244163_at	0	3.128	0.13	7	3.342	0.22	40	u	0.018887
STEAP3	2	STEAP family member 3	218424_s_at	2	8.098	0.37	7	7.739	1.07	40	d	0.127784
			1554830_a_at	0	5.038	0.48	7	5.43	1.3	40	u	0.182088
YEATS2	2	YEATS domain containing 2	221203_s_at	2	7.394	0.2	7	7.903	0.62	40	u	0.000474
			1557047_at	0	5.163	0.4	7	5.621	0.51	40	u	0.032979
KRTAP11-1	2	keratin associated protein 11-1	1564803_at	2	2.571	0.15	7	2.584	0.24	40	u	0.890408
MBOAT1	2	membrane bound O-acyltransferase domain containing 1	227379_at	2	8.534	0.56	7	8.409	1.32	40	d	0.811255
TEAD3	2	TEA domain family member 3	209454_s_at	2	8.398	0.18	7	7.615	0.65	40	d	0.000001
ACOX1	2	acyl-Coenzyme A oxidase 1, palmitoyl	213501_at	2	5.036	0.4	7	6.019	0.97	40	u	0.013209
			209600_s_at	1	6.563	0.47	7	7.465	0.76	40	u	0.004765
			209601_at	1	2.436	0.09	7	2.474	0.15	40	u	0.517803
			227962_at	0	7.448	0.26	7	7.019	0.63	40	d	0.091006
			207656_s_at	0	2.747	0.15	7	2.829	0.13	40	u	0.139013
KIF5B	2	kinesin family member 5B	201991_s_at	0	11.22	0.16	7	11.46	0.58	40	u	0.044411
			201992_s_at	0	7.142	0.65	7	8.12	0.92	40	u	0.011397
			224662_at	0	10.45	0.25	7	10.63	0.61	40	u	0.454694

HAO1	2	hydroxyacid oxidase (glycolate oxidase) 1	220224_at	2	2.248	0.02	7	2.514	0.82	40	u	0.050952
SFRS16	2	splicing factor, arginine/serine-rich 16	204978_at	2	8.35	0.6	7	7.93	0.64	40	d	0.120439
ANGPTL7	2	angiopoietin-like 7	206423_at	2	2.596	0.43	7	2.521	0.97	40	d	0.843708
NTNG2	2	netrin G2	233072_at	2	3.4	0.2	7	3.376	0.34	40	d	0.865608
APOB	2	apolipoprotein B (including Ag(x) antigen)	205108_s_at	2	2.17	0.03	7	2.281	0.16	40	u	0.000366
			223579_s_at	0	1.96	0.02	7	2.001	0.04	40	u	0.004849
FLNC	2	filamin C, gamma (actin binding protein 280)	207876_s_at	2	5.336	0.34	7	4.541	0.49	40	d	0.000233
C14orf147	2	chromosome 14 open reading frame 147	213508_at	2	8.863	0.42	7	8.231	1.18	40	d	0.021163
			212460_at	2	8.957	0.44	7	9.296	1.23	40	u	0.216756
SPTAN1	2	spectrin, alpha, non-erythrocytic 1 (alpha-fodrin)	215235_at	2	8.807	0.31	7	7.818	0.76	40	d	0.001689
			208611_s_at	2	9.785	0.27	7	8.591	0.69	40	d	0.000056
			214926_at	0	2.088	0.15	7	2.164	0.32	40	u	0.54449
			241150_at	0	4.495	0.3	7	4.433	0.42	40	d	0.715229
			214925_s_at	0	6.928	0.35	7	5.793	0.92	40	d	0.002893
RLBP1L2	2	retinaldehyde binding protein 1-like 2	1562443_at	2	2.697	0.22	7	2.951	0.36	40	u	0.080485
HSPA4	2	heat shock 70kDa protein 4	208814_at	2	5.15	0.53	7	5.276	1.17	40	u	0.784028
			211016_x_at	2	5.35	0.69	7	6.669	1.27	40	u	0.012096
			208815_x_at	2	9.737	0.23	7	10.3	0.62	40	u	0.025461
			211015_s_at	0	8.541	0.3	7	9.382	0.76	40	u	0.006995
HIP2	2	huntingtin interacting protein 2	202346_at	2	7.349	0.26	7	8.109	0.66	40	u	0.005297
			202347_s_at	2	9.128	0.13	7	9.728	0.54	40	u	0.000001
PSCD1	2	pleckstrin homology, Sec7 and coiled-coil domains 1(cytohesin 1)	202880_s_at	2	7.856	0.18	7	8.024	0.72	40	u	0.230257
			202879_s_at	2	5.899	0.3	7	6.224	0.59	40	u	0.171111
PLXNC1	2	plexin C1	206470_at	2	4.601	0.55	7	5.337	1.03	40	u	0.07653
			206471_s_at	2	4.166	0.38	7	5.397	0.87	40	u	0.000752
			213241_at	0	7.435	0.47	7	7.39	1.24	40	d	0.927526
			235328_at	0	3.759	0.73	7	3.666	0.66	40	d	0.74185
STBD1	2	starch binding domain 1	203986_at	2	6.874	0.78	7	6.463	1.35	40	d	0.447114
			1570398_at	0	2.42	0.22	7	2.56	0.23	40	u	0.147698
			1557575_at	0	2.652	0.22	7	2.863	0.42	40	u	0.211578
SFRS3	2	splicing factor, arginine/serine-rich 3	208673_s_at	2	10.22	0.33	7	9.823	0.44	40	d	0.030823
			208672_s_at	1	12.08	0.18	7	12.1	0.3	40	u	0.886154
			232392_at	1	5.626	0.6	7	6.451	1.21	40	u	0.090654
			202899_s_at	0	10.64	0.38	7	10.54	0.69	40	d	0.721553
CENPO	2	centromere protein O	219472_at	5	5.398	0.59	7	5.32	0.63	40	d	0.766364
			226118_at	2	4.132	0.7	7	5.351	0.9	40	u	0.001783
FBXW2	2	F-box and WD repeat domain containing 2	218941_at	2	6.496	0.32	7	6.563	0.6	40	u	0.776646
			209630_s_at	1	9.567	0.32	7	9.077	0.7	40	d	0.080172
			241736_at	0	2.471	0.19	7	2.684	0.3	40	u	0.083725
			235195_at	0	4.701	0.48	7	4.483	0.85	40	d	0.5184
			1560752_at	0	2.807	0.35	7	2.728	0.15	40	d	0.601242
CRY1	2	cryptochrome 1 (photolyase-like)	209674_at	2	8.242	0.53	7	8.163	0.61	40	d	0.756135
CPNE2	2	copine II	225129_at	2	7.169	0.41	7	6.966	0.91	40	d	0.573462
PBRM1	2	polybromo 1	223899_at	2	4.131	0.35	7	4.59	0.55	40	u	0.041972
			223900_s_at	2	2.684	0.13	7	2.858	0.39	40	u	0.045595
			232613_at	0	5.708	0.54	7	5.771	0.83	40	u	0.849803
			220355_s_at	0	8.031	0.23	7	7.871	0.58	40	d	0.483766
			221212_x_at	0	3.631	0.28	7	3.874	0.32	40	u	0.071833
			223238_s_at	0	8.122	0.27	7	8.238	0.55	40	u	0.592038
			224152_s_at	0	5.052	0.54	7	4.855	0.81	40	d	0.547237
			223400_s_at	0	3.811	0.51	7	4.061	0.71	40	u	0.388148
			223399_x_at	0	2.397	0.13	7	2.868	0.69	40	u	0.000385
PUNC	2	putative neuronal cell adhesion molecule	232037_at	2	4.169	0.88	7	3.96	0.84	40	d	0.55693
			230960_at	0	3.043	0.45	7	2.996	0.54	40	d	0.835292
GRLF1	2	glucocorticoid receptor DNA binding factor 1	202044_at	2	5.607	0.3	7	5.855	0.45	40	u	0.173883
			202045_s_at	2	6.755	0.35	7	6.339	0.57	40	d	0.073004
			229394_s_at	2	9.721	0.4	7	8.696	0.78	40	d	0.001667
			229397_s_at	2	3.985	0.64	7	4.283	0.85	40	u	0.391646
			202046_s_at	0	4.958	0.57	7	5.657	1.25	40	u	0.163547
USP9X	2	ubiquitin specific peptidase 9, X-linked	201099_at	2	8.617	0.28	7	8.378	0.7	40	d	0.386594
			201100_s_at	2	10.58	0.16	7	10.58	0.51	40	d	0.969084
			229573_at	0	6.839	0.27	7	6.883	0.47	40	u	0.814887

PAXIP1	2	PAX interacting (with transcription-activation domain) protein 1	212825_at	2	7.658	0.42	7	8.19	0.84	40	u	0.116594
SERINC2	2	serine incorporator 2	224762_at	2	8.548	0.44	7	7.677	1.2	40	d	0.002936
ARHGAP28	2	Rho GTPase activating protein 28	227911_at	2	4.34	0.74	7	4.239	0.93	40	d	0.791686
			220382_s_at	0	2.333	0.03	7	2.501	0.42	40	u	0.017652
			220381_at	0	2.091	0.02	7	2.18	0.18	40	u	0.005056
CCDC45	2	coiled-coil domain containing 45	225705_at	2	7.882	0.51	7	7.436	0.95	40	d	0.240671
			223815_at	0	4.258	0.45	7	4.41	0.6	40	u	0.530543
THTPA	2	thiamine triphosphatase	218540_at	2	7.746	0.28	7	7.222	0.71	40	d	0.06758
STK38L	2	serine/threonine kinase 38 like	212572_at	2	8.376	0.53	7	9.101	1.25	40	u	0.145849
			212565_at	2	7.199	0.17	7	7.79	1.26	40	u	0.008476
CCDC120	2	coiled-coil domain containing 120	239403_at	2	3.405	0.27	7	3.35	0.43	40	d	0.75013
			232996_at	0	2.149	0.05	7	2.316	0.26	40	u	0.000707
SLC27A1	2	solute carrier family 27 (fatty acid transporter), member 1	226728_at	2	8.069	0.36	7	6.246	1.09	40	d	0
STAC2	2	SH3 and cysteine rich domain 2	228888_at	2	8.506	0.81	7	6.438	0.82	40	d	0
CDK6	2	cyclin-dependent kinase 6	224851_at	0	6.927	0.38	7	6.842	1.88	40	d	0.805363
			224847_at	0	7.481	0.42	7	7.045	1.75	40	d	0.191371
			224848_at	0	5.772	0.25	7	5.728	1.35	40	d	0.85453
			243000_at	0	3.551	0.25	7	4.181	1.05	40	u	0.0027
			207143_at	0	2.792	0.25	7	3.337	0.9	40	u	0.004308
			235287_at	0	2.971	0.22	7	3.593	1.18	40	u	0.005018
			231198_at	0	2.884	0.41	7	2.673	0.6	40	d	0.383387
THC2	2	thrombocytopenia 2 (autosomal dominant)	no probeset available									
ZXDA	2	zinc finger, X-linked, duplicated A	215263_at	2	2.933	0.37	7	3.159	0.44	40	u	0.221145
			216014_s_at	0	2.32	0.18	7	2.364	0.2	40	u	0.593095
TMTC3	2	transmembrane and tetratricopeptide repeat containing 3	226600_at	2	7.316	0.39	7	7.84	0.8	40	u	0.105193
			226604_at	2	7.963	0.34	7	7.668	0.89	40	d	0.401884
			1560017_at	0	3.481	0.19	7	4.245	0.68	40	u	0.000002
AFF4	2	AF4/FMR2 family, member 4	225229_at	8	9.267	0.24	7	9.009	0.57	40	d	0.255495
			219199_at	2	2.666	0.31	7	3.363	1.07	40	u	0.002522
			1555436_a_at	0	5.294	0.45	7	5.24	0.67	40	d	0.841935
			232865_at	0	8.457	0.43	7	7.426	1.04	40	d	0.015095
			232864_s_at	0	7.408	0.47	7	6.724	0.55	40	d	0.004228
			243487_at	0	2.876	0.29	7	3.449	0.98	40	u	0.006506
			239439_at	0	3.742	0.61	7	4.929	1.37	40	u	0.033
			1555435_at	0	3.901	0.44	7	4.383	0.59	40	u	0.048497
RAG1AP1	2	recombination activating gene 1 activating protein 1	219125_s_at	2	8.292	0.47	7	8.867	0.73	40	u	0.05519
LMOD1	2	leiomodin 1 (smooth muscle)	211562_s_at	2	5.86	0.44	7	3.981	0.58	40	d	0
			203766_s_at	2	8.953	0.38	7	4.689	1.27	40	d	0
FHL2	2	four and a half LIM domains 2	202949_s_at	2	11.09	0.64	7	9.146	1.54	40	d	0.002408
SUGT1	2	SGT1, suppressor of G2 allele of SKP1 (S. cerevisiae)	223330_s_at	2	5.232	0.48	7	5.988	0.89	40	u	0.038046
			223329_x_at	2	9.545	0.24	7	10.03	0.68	40	u	0.002994
			224309_s_at	0	8.703	0.07	7	9.17	0.67	40	u	0.000142
			234686_at	0	3.533	0.24	7	3.683	0.37	40	u	0.319291
TMEM112B	2	transmembrane protein 112B	212682_s_at	2	6.643	0.27	7	6.232	0.56	40	d	0.070179
			31837_at	2	7.908	0.2	7	7.687	0.51	40	d	0.273621
ITGAV	2	integrin, alpha V (vitronectin receptor, alpha polypeptide, antigen CD51)	202351_at	2	10.04	0.3	7	10.15	0.84	40	u	0.55304
RDBP	2	RD RNA binding protein	209219_at	0	9.091	0.21	7	9.211	0.67	40	u	0.390448
RBBP4	2	retinoblastoma binding protein 4	217301_x_at	7	9.809	0.31	7	9.407	1.2	40	d	0.090426
			210371_s_at	6	10.24	0.2	7	10.15	0.7	40	d	0.515986
			239071_at	0	7.665	0.3	7	7.57	0.7	40	d	0.728885
B4GALT6	2	UDP-Gal:betaGlcNAc beta 1,4-galactosyltransferase, polypeptide 6	206233_at	2	3.948	0.63	7	3.942	0.89	40	d	0.986126
			235333_at	0	5.451	0.48	7	5.218	0.96	40	d	0.543142
			206232_s_at	0	2.014	0.05	7	2.095	0.17	40	u	0.020327
ZRANB1	2	zinc finger, RAN-binding domain containing 1	225131_at	2	7.352	0.21	7	7.421	0.62	40	u	0.606887
			225138_at	2	7.89	0.24	7	7.339	0.61	40	d	0.026811
			225130_at	2	5.074	0.44	7	4.304	0.63	40	d	0.004173
FAM80A	2	family with sequence similarity 80, member A	235948_at	2	3.033	0.34	7	3.299	0.53	40	u	0.218401
			221726_at	2	11.85	0.17	7	10.52	0.81	40	d	0
			221775_x_at	0	13.89	0.1	7	13.52	0.4	40	d	0.000026

RPL22	2	ribosomal protein L22	208768_x_at	0	13.87	0.08	7	13.5	0.4	40	d	0.000006
			220960_x_at	0	12.92	0.12	7	12.69	0.32	40	d	0.004315
			214042_s_at	0	12.55	0.14	7	11.84	0.6	40	d	0
XKR7	2	blood group complex subunit-related family, m	no probeset available									
KIAA1147	2	KIAA1147	223162_s_at	2	9.234	0.35	7	9.083	0.67	40	d	0.56965
			223161_at	2	6.065	0.54	7	7.154	0.96	40	u	0.006388
MFSD2	2	major facilitator superfamily domain containing 2	225316_at	2	5.147	0.6	7	5.435	1.02	40	u	0.480744
			1553386_at	0	3.034	0.28	7	3.17	0.59	40	u	0.563506
HR	2	hairless homolog (mouse)	241355_at	2	5.481	0.35	7	5.689	1.32	40	u	0.421829
			210086_at	0	5.693	0.33	7	5.71	0.91	40	u	0.931015
			220163_s_at	0	4.762	0.24	7	4.728	0.58	40	d	0.883185
C14orf130	2	chromosome 14 open reading frame 130	218108_at	2	8.525	0.27	7	8.449	0.65	40	d	0.765687
TFAP2D	2	transcription factor AP-2 delta (activating enhancer binding protein 2 delta)	233987_at	0	4.426	0.29	7	4.388	0.41	40	d	0.822636
CTBP1	2	C-terminal binding protein 1	213980_s_at	2	9.978	0.28	7	9.924	0.56	40	d	0.80654
			203392_s_at	1	9.683	0.43	7	9.077	0.97	40	d	0.120845
			1557714_at	0	1.949	0.03	7	1.994	0.06	40	u	0.067077
			212863_x_at	0	10.91	0.31	7	10.41	0.64	40	d	0.053413
ASCC2	2	activating signal cointegrator 1 complex subunit 2	215684_s_at	2	7.572	0.12	7	7.376	0.57	40	d	0.066177
PLEKHA7	2	pleckstrin homology domain containing, family A member 7	228450_at	2	5.677	0.42	7	4.501	0.68	40	d	0.000089
REV1	2	REV1 homolog (S. cerevisiae)	218428_s_at	2	8.707	0.13	7	8.081	0.4	40	d	0
			222629_at	0	4.899	0.64	7	4.561	0.7	40	d	0.25014
			243592_at	0	3.739	0.38	7	3.692	0.38	40	d	0.769832
			222628_s_at	0	8.547	0.31	7	8.261	0.47	40	d	0.139852
PRPS1	2	phosphoribosyl pyrophosphate synthetase 1	209440_at	2	8.345	0.31	7	8.488	0.86	40	u	0.451063
			208447_s_at	0	5.322	0.65	7	6.055	1.34	40	u	0.172453
PPP2R2C	2	protein phosphatase 2 (formerly 2A), regulatory subunit B, gamma isoform	228010_at	2	3.406	0.44	7	5.153	2.31	40	u	0.000111
			228137_s_at	0	2.262	0.02	7	2.313	0.07	40	u	0.000573
			228025_s_at	0	2.288	0.02	7	2.353	0.08	40	u	0.00034
			228140_s_at	0	3.212	0.69	7	2.956	0.36	40	d	0.406347
			223573_s_at	0	3.321	0.55	7	4.04	1.48	40	u	0.220303
			223574_x_at	0	3.474	0.51	7	5.224	2.34	40	u	0.000202
AGPAT4	2	1-acylglycerol-3-phosphate O-acyltransferase 4 (lysophosphatidic acid acyltransferase,	228667_at	2	4.183	0.55	7	3.511	0.74	40	d	0.030483
			219693_at	2	3.825	0.48	7	3.827	0.75	40	u	0.99414
TBX1	2	T-box 1	236926_at	2	6.353	0.54	7	6.057	1.16	40	d	0.518721
			207662_at	0	5.906	0.41	7	5.922	0.39	40	u	0.922413
			211274_at	0	2.786	0.27	7	2.748	0.11	40	d	0.74074
			242941_x_at	0	5.051	0.35	7	4.82	0.33	40	d	0.105452
			211273_s_at	0	2.499	0.13	7	3.1	1.09	40	u	0.001895
LRFN2	2	leucine rich repeat and fibronectin type III domain containing 2	232697_at	2	2.511	0.03	7	2.578	0.1	40	u	0.001421
SFRS2IP	2	splicing factor, arginine/serine-rich 2, interacting protein	209376_x_at	2	9.318	0.29	7	9.351	0.46	40	u	0.860832
			206989_s_at	2	9.021	0.18	7	8.787	0.68	40	d	0.082651
			213850_s_at	0	9.262	0.29	7	9.38	0.47	40	u	0.528525
			235579_at	0	6.559	0.36	7	6.874	0.45	40	u	0.093957
			232597_x_at	0	9.186	0.47	7	8.597	0.82	40	d	0.079044
			225336_at	0	9.22	0.12	7	8.695	0.52	40	d	0.000003
NRG2	2	neuregulin 2	206879_s_at	2	4.308	0.46	7	3.847	0.52	40	d	0.036478
			208062_s_at	0	4.343	1.36	7	3.132	0.93	40	d	0.006187
STK33	2	serine/threonine kinase 33	228035_at	2	1.743	0.02	7	1.839	0.19	40	u	0.003972
			228086_at	2	1.833	0.1	7	2.259	0.82	40	u	0.003301
TXNL1	2	thioredoxin-like 1	201588_at	2	10.88	0.2	7	10.99	0.6	40	u	0.422634
			243664_at	0	4.514	0.34	7	4.698	0.85	40	u	0.583176
MAP3K10	2	mitogen-activated protein kinase kinase kinase 10	206362_x_at	2	5.647	0.28	7	5.84	0.29	40	u	0.124035
JPH4	2	junctophilin 4	226918_at	2	3.419	0.29	7	3.337	0.36	40	d	0.577184
FAM118A	2	family with sequence similarity 118, member A	219629_at	2	4.359	1.34	7	4.356	1.09	40	d	0.995581
			226475_at	0	6.238	0.65	7	5.291	1.15	40	d	0.043116
ADRB2	2	adrenergic, beta-2-, receptor, surface	206170_at	2	5.872	0.7	7	4.04	0.94	40	d	0.000018
KY	2	kyphoscoliosis peptidase	1556411_s_at	2	2.604	0.29	7	2.415	0.27	40	d	0.103015
			1564254_at	2	4.475	0.18	7	4.483	0.2	40	u	0.924737
			1569991_at	0	2.499	0.2	7	2.53	0.44	40	u	0.859449

C20orf46	2	chromosome 20 open reading frame 46	219958_at	2	3.048	0.6	7	3.544	0.88	40	u	0.168533
MANEAL	2	mannosidase, endo-alpha-like	226132_s_at	2	5.979	0.6	7	6.789	1.55	40	u	0.187971
			231564_at	0	2.589	0.06	7	2.665	0.1	40	u	0.059356
ABHD7	2	abhydrolase domain containing 7	239579_at	2	2.812	0.2	7	3.451	0.97	40	u	0.0008
B3GNT1	2	UDP-GlcNAc:betaGal beta-1,3-N-acetylglucosaminyltransferase 1	203188_at	2	8.006	0.17	7	6.768	0.93	40	d	0
			228203_at	0	2.759	0.29	7	2.602	0.24	40	d	0.137768
SEMA4B	2	sema domain, immunoglobulin domain (Ig), transmembrane domain (TM) and short cytoplasmic domain, (semaphorin) 4B	234725_s_at	2	8.876	0.39	7	8.217	0.96	40	d	0.088131
CCNC	2	cyclin C	201955_at	2	10.23	0.31	7	10.62	0.79	40	u	0.214022
			1569126_at	0	3.6	0.39	7	4.366	0.66	40	u	0.005614
UBAP1	2	ubiquitin associated protein 1	46270_at	2	7.875	0.27	7	7.554	0.47	40	d	0.091442
			221490_at	2	7.881	0.39	7	7.63	0.38	40	d	0.120574
GAB2	2	GRB2-associated binding protein 2	203853_s_at	2	7.13	0.3	7	6.914	1.17	40	d	0.339815
HSDL1	2	hydroxysteroid dehydrogenase like 1	223248_at	2	5.648	0.37	7	5.843	0.5	40	u	0.335599
CCL2	2	chemokine (C-C motif) ligand 2	216598_s_at	2	9.138	1.14	7	8.021	1.36	40	d	0.050973
WHSC2	2	Wolf-Hirschhorn syndrome candidate 2	203112_s_at	2	7.36	0.18	7	7.089	0.61	40	d	0.033517
			34225_at	2	7.221	0.31	7	6.937	0.6	40	d	0.234457
CCND3	2	cyclin D3	201700_at	2	9.525	0.47	7	8.785	0.81	40	d	0.026073
			1562028_at	0	3.444	0.91	7	2.766	0.35	40	d	0.122651
CHMP4B	2	chromatin modifying protein 4B	225498_at	2	11.06	0.23	7	10.35	0.56	40	d	0.002454
			225119_at	0	5.582	0.61	7	6.865	1.12	40	u	0.00592
GRAMD1A	2	GRAM domain containing 1A	224807_at	2	6.632	0.16	7	6.727	0.94	40	u	0.565804
			244808_at	0	6.36	0.26	7	6.103	0.89	40	d	0.156248
PSME4	2	proteasome (prosome, macropain) activator subunit 4	212222_at	2	9.014	0.38	7	8.561	0.79	40	d	0.152137
			212219_at	2	8.992	0.43	7	9.596	0.69	40	u	0.033512
			212220_at	2	5.455	0.53	7	7.666	1.05	40	u	0.000003
			237180_at	0	6.844	0.61	7	6.289	0.95	40	d	0.149042
ANXA5	2	annexin A5	200782_at	2	11.76	0.29	7	11.42	0.59	40	d	0.157961
ATF7IP	2	activating transcription factor 7 interacting protein	207728_at	0	3.474	0.44	7	3.601	0.52	40	u	0.554588
			216197_at	0	6.207	0.35	7	6.461	0.85	40	u	0.448778
			218987_at	0	7.623	0.28	7	8.333	0.57	40	u	0.00297
			231825_x_at	0	9.906	0.28	7	9.745	0.58	40	d	0.483698
			216198_at	0	3.202	0.45	7	3.478	0.47	40	u	0.167856
CHAC1	2	ChaC, cation transport regulator homolog 1 (E. coli)	219270_at	2	4.212	0.41	7	4.843	1	40	u	0.113416
LIMK2	2	LIM domain kinase 2	210582_s_at	2	8.279	0.29	7	7.833	0.76	40	d	0.141022
			202193_at	2	8.101	0.29	7	7.94	1.05	40	d	0.443224
			217475_s_at	0	4.886	0.39	7	4.873	1.21	40	d	0.958318
EIF4G1	2	eukaryotic translation initiation factor 4 gamma, 1	208624_s_at	1	6.938	0.64	7	8.321	0.98	40	u	0.001027
			208625_s_at	1	10.53	0.28	7	10.38	0.61	40	d	0.547269
RANBP3	2	RAN binding protein 3	210120_s_at	2	7.668	0.14	7	7.641	0.45	40	d	0.765635
			202639_s_at	2	7.48	0.35	7	7.284	0.69	40	d	0.47331
			202640_s_at	2	6.861	0.5	7	6.201	0.93	40	d	0.080323
			208272_at	0	4.439	0.63	7	3.915	0.4	40	d	0.007183
SLC4A10	2	solute carrier family 4, sodium bicarbonate transporter-like, member 10	206830_at	2	2.668	0.06	7	2.836	0.29	40	u	0.002269
PARP1	2	poly (ADP-ribose) polymerase family, member 1	208644_at	2	8.506	0.22	7	9.704	0.69	40	u	0
SLC35A4	2	solute carrier family 35, member A4	224626_at	2	8.377	0.21	7	7.792	0.68	40	d	0.000232
JAG2	2	jagged 2	209784_s_at	2	8.965	0.68	7	7.378	0.79	40	d	0.000012
			32137_at	2	8.598	0.72	7	7.045	0.92	40	d	0.000142
CD34	2	CD34 molecule	209543_s_at	2	7.169	0.98	7	4.998	0.52	40	d	0.001844
STX3	2	syntaxin 3	209238_at	3	7.469	0.31	7	7.919	0.82	40	u	0.164817
			216985_s_at	0	3.745	0.72	7	4.853	1.52	40	u	0.070834
TAGLN	2	transgelin	1555724_s_at	2	12.64	0.3	7	10.03	1.47	40	d	0
			205547_s_at	2	13.29	0.25	7	10.87	1.39	40	d	0
			226523_at	0	8.383	0.49	7	5.692	0.9	40	d	0
WDR81	2	WD repeat domain 81	226738_at	2	7.456	0.22	7	6.792	0.46	40	d	0.0006
GLUD1	2	glutamate dehydrogenase 1	200946_x_at	2	9.887	0.59	7	8.857	0.79	40	d	0.002404
			200947_s_at	2	10.93	0.44	7	9.758	0.83	40	d	0.000855
			244802_at	0	2.821	0.22	7	3.453	1.06	40	u	0.001997
LSM11	2	LSM11, U7 small nuclear RNA associated [illegible]	242112_at	2	2.504	0.11	7	2.782	0.46	40	u	0.002544

LSM11	2	LSM11, U7 small nuclear RNA associated	241599_at	0	2.294	0.18	7	2.526	0.44	40	u	0.189566
RFXDC1	2	regulatory factor X domain containing 1	1552673_at	2	2.777	0.82	7	2.334	0.14	40	d	0.233618
CNNM1	2	cyclin M1	220166_at	2	2.509	0.19	7	2.719	0.61	40	u	0.104151
PRKRIR	2	protein-kinase, interferon-inducible double stranded RNA dependent inhibitor, repressor of (P58 repressor)	209323_at	2	9.565	0.21	7	9.858	1.03	40	u	0.12127
TMEM158	2	transmembrane protein 158	213338_at	2	5.36	1.43	7	7.436	2.44	40	u	0.037857
TRABD	2	TraB domain containing	221807_s_at	2	6.092	0.25	7	6.483	0.58	40	u	0.092353
			232706_s_at	2	7.607	0.21	7	7.387	0.57	40	d	0.090928
			225360_at	2	6.543	0.24	7	6.66	0.58	40	u	0.604939
PDE2A	2	phosphodiesterase 2A, cGMP-stimulated	204134_at	2	7.047	0.55	7	4.217	0.69	40	d	0
DAB2	2	disabled homolog 2, mitogen-responsive phosphoprotein (Drosophila)	201278_at	0	9.231	0.45	7	7.382	1.04	40	d	0.000042
			232898_at	0	5.911	0.64	7	4.384	1.21	40	d	0.002625
			240873_x_at	0	4.354	0.6	7	3.991	0.59	40	d	0.150043
			201279_s_at	0	9.158	0.72	7	7.725	0.91	40	d	0.000339
			201280_s_at	0	9.277	0.56	7	7.732	0.94	40	d	0.000148
			210757_x_at	0	8.965	0.62	7	7.504	1.01	40	d	0.000725
			1559370_at	0	2.378	0.08	7	2.546	0.16	40	u	0.011807
DVL3	2	dishevelled, dsh homolog 3 (Drosophila)	201908_at	2	9.016	0.26	7	8.518	0.77	40	d	0.004967
			201907_x_at	1	5.561	0.17	7	5.856	0.77	40	u	0.043189
MN1	2	meningioma (disrupted in balanced translocation) 1	205330_at	2	4.506	1.24	7	3.629	1.12	40	d	0.072852
PLDN	2	pallidin homolog (mouse)	222826_at	2	8.83	0.3	7	8.694	0.72	40	d	0.632759
			224892_at	2	9.134	0.25	7	8.93	0.59	40	d	0.383074
			224883_at	2	7.429	0.3	7	7.413	0.69	40	d	0.951586
MOSPD3	2	motile sperm domain containing 3	219070_s_at	2	7.944	0.24	7	8	0.55	40	u	0.797496
CD24	2	CD24 molecule	266_s_at	2	11.28	0.55	7	12.07	1.33	40	u	0.134629
			216379_x_at	2	13.28	0.43	7	13.63	1.15	40	u	0.448999
			209771_x_at	2	13.18	0.43	7	13.54	1.09	40	u	0.401464
			208650_s_at	2	11.22	0.73	7	12.41	1.73	40	u	0.086128
			208651_x_at	0	10.47	0.52	7	11.14	1.43	40	u	0.041585
			209772_s_at	0	9.778	0.88	7	10.71	2.07	40	u	0.256247
TPM4	2	tropomyosin 4	1559989_at	2	2.935	0.12	7	3.183	0.32	40	u	0.001639
			212481_s_at	2	9.098	0.64	7	9.746	1.12	40	u	0.151042
			209344_at	2	9.814	0.52	7	10.3	1.24	40	u	0.321346
			1567107_s_at	0	7.938	0.63	7	9.138	1.04	40	u	0.005939
HERPUD1	2	homocysteine-inducible, endoplasmic reticulum stress-inducible, ubiquitin-like domain member 1	217168_s_at	2	11.06	0.35	7	10.42	0.71	40	d	0.029277
C2orf29	2	chromosome 2 open reading frame 29	224695_at	2	8.423	0.26	7	8.764	0.52	40	u	0.104489
ATAD2	2	ATPase family, AAA domain containing 2	222740_at	2	6.095	0.41	7	8.154	1.05	40	u	0.000009
			228401_at	0	5.309	0.39	7	6.995	0.83	40	u	0.000006
			235266_at	0	4.113	0.56	7	6.293	1.19	40	u	0.000028
			218782_s_at	0	5.355	0.35	7	8.016	1.32	40	u	0
LRRK1	2	leucine-rich repeat kinase 1	219441_s_at	2	5.255	0.28	7	5.181	0.6	40	d	0.754892
DENR	2	density-regulated protein	221509_at	2	9.83	0.31	7	10.08	0.55	40	u	0.25933
			234915_s_at	2	6.244	0.58	7	6.964	0.98	40	u	0.0705
			234347_s_at	2	2.914	0.25	7	3.065	0.6	40	u	0.52026
			231896_s_at	2	10.89	0.16	7	10.83	0.57	40	d	0.61471
			238982_at	0	3.815	0.49	7	4.188	0.6	40	u	0.131298
ADAMTS1	2	ADAM metallopeptidase with thrombospondin type 1 motif, 1	222162_s_at	2	9.34	0.65	7	7.263	1.41	40	d	0.000532
			222486_s_at	0	7.113	1.02	7	5.453	1.43	40	d	0.006117
ELAVL4	2	ELAV (embryonic lethal, abnormal vision, Drosophila)-like 4 (Hu antigen D)	206051_at	2	2.686	0.21	7	2.799	0.35	40	u	0.421129
			234904_x_at	0	2.926	0.06	7	3.086	0.23	40	u	0.000919
CADM3	2	cell adhesion molecule 3	213948_x_at	2	2.688	0.35	7	2.482	0.2	40	d	0.211699
			211677_x_at	2	5.581	0.5	7	4.84	0.27	40	d	0.012282
			221921_s_at	1	5.417	0.51	7	4.705	0.43	40	d	0.00037
			216535_at	0	1.955	0.02	7	2.023	0.2	40	u	0.041975
PCF11	2	PCF11, cleavage and polyadenylation factor subunit, homolog (S. cerevisiae)	203378_at	2	9.013	0.34	7	8.137	0.71	40	d	0.003026
			227622_at	0	8.571	0.38	7	8.328	0.75	40	d	0.413641
T	2	T, brachyury homolog (mouse)	206524_at	2	2.859	0.24	7	3.074	0.33	40	u	0.113069
SORBS1	2	sorbin and SH3 domain containing 1	218087_s_at	2	9.103	0.7	7	5.569	1.36	40	d	0
			222513_s_at	2	9.009	0.92	7	6.925	1.12	40	d	0.000037
			211819_s_at	0	3.647	0.85	7	2.947	0.41	40	d	0.093884

			211705_s_at	0	3.918	0.38	7	3.89	0.44	40	d	0.877484
TRIM13	2	tripartite motif-containing 13	230192_at	2	6.337	0.34	7	6.23	0.95	40	d	0.606917
			229943_at	0	7.429	0.34	7	7.017	1.16	40	d	0.086086
			203659_s_at	0	7.916	0.21	7	7.526	0.7	40	d	0.009498
			1569142_at	0	3.579	0.35	7	4.172	0.72	40	u	0.042463
CYGB	2	cytoglobin	226632_at	2	7.622	0.58	7	5.869	0.71	40	d	0
			1570410_at	1	5.295	0.49	7	5.211	0.55	40	d	0.711697
			1553572_a_at	0	4.487	0.89	7	3.739	0.52	40	d	0.090429
GPR4	2	G protein-coupled receptor 4	206236_at	2	4.165	0.3	7	3.758	0.56	40	d	0.071412
			211266_s_at	0	2.788	0.11	7	2.854	0.15	40	u	0.287031
CLINT1	2	clathrin interactor 1	201769_at	2	9.149	0.26	7	9.045	0.72	40	d	0.510583
			201768_s_at	0	7.834	0.54	7	7.951	1.04	40	u	0.77627
			230609_at	0	3.675	0.59	7	3.849	1.02	40	u	0.669887
SEMA6C	2	sema domain, transmembrane domain (TM), and cytoplasmic domain, (semaphorin) 6C	208100_x_at	2	5.745	0.24	7	5.762	0.4	40	u	0.915625
FOXA1	2	forkhead box A1	204667_at	2	9.348	0.71	7	6.87	3.16	40	d	0.000129
SLC1A4	2	solute carrier family 1 (glutamate/neutral amino acid transporter), member 4	212810_s_at	0	7.04	0.84	7	7.03	1.2	40	d	0.982832
			244377_at	0	3.921	0.47	7	4.37	0.92	40	u	0.221985
			209610_s_at	0	8.179	0.7	7	8.301	1.36	40	u	0.821297
			209611_s_at	0	5.042	0.47	7	5.323	1.18	40	u	0.546924
			212811_x_at	0	7.826	0.62	7	7.935	1.21	40	u	0.821644
HOMER1	2	homer homolog 1 (Drosophila)	226651_at	2	7.715	0.47	7	6.981	0.84	40	d	0.032703
			213793_s_at	0	7.84	0.51	7	7.517	0.84	40	d	0.340854
ENY2	2	enhancer of yellow 2 homolog (Drosophila)	218482_at	2	9.495	0.19	7	10.39	0.68	40	u	0
			226775_at	0	6.973	0.49	7	7.141	0.92	40	u	0.648434
			226776_at	0	4.301	0.34	7	4.411	0.76	40	u	0.716573
SCN2A	2	sodium channel, voltage-gated, type II, alpha subunit	206381_at	2	2.63	0.57	7	2.663	1.06	40	u	0.936962
			229057_at	0	4.398	1.14	7	2.885	1.25	40	d	0.005421
WNT7A	2	wingless-type MMTV integration site family, member 7A	210248_at	2	2.471	0.2	7	2.542	0.35	40	u	0.605898
SLC18A2	2	solute carrier family 18 (vesicular monoamine), member 2	1553328_a_at	2	2.605	0.28	7	2.608	0.33	40	u	0.98172
			205857_at	0	3.63	0.51	7	3.576	0.76	40	d	0.861819
GNAQ	2	guanine nucleotide binding protein (G protein), q polypeptide	202615_at	2	10.8	0.29	7	10.22	0.65	40	d	0.029396
			224861_at	0	8.905	0.2	7	7.887	0.69	40	d	0
			224862_at	0	7.595	0.26	7	7.539	0.76	40	d	0.732562
			211426_x_at	0	6.274	0.48	7	6.19	0.57	40	d	0.718753
			224863_at	0	7.414	0.17	7	7.65	0.44	40	u	0.180438
GNPNAT1	2	glucosamine-phosphate N-acetyltransferase 1	225853_at	2	8.225	0.24	7	8.287	0.81	40	u	0.705197
TROVE2	2	TROVE domain family, member 2	212839_s_at	2	3.112	0.45	7	3.928	0.61	40	u	0.001875
			207520_at	2	3.093	0.27	7	3.56	0.44	40	u	0.011234
			212852_s_at	2	11.32	0.12	7	11.62	0.53	40	u	0.003616
			210438_x_at	0	10.32	0.09	7	10.5	0.54	40	u	0.073603
			213027_at	0	8.121	0.21	7	8.338	0.62	40	u	0.109214
METRNL	2	meteorin, glial cell differentiation regulator-like	225955_at	2	7.974	0.72	7	8.114	0.8	40	u	0.673122
RASSF2	2	Ras association (RalGDS/AF-6) domain family 2	203185_at	2	7.028	0.62	7	7.335	1.07	40	u	0.472983
ZNF346	2	zinc finger protein 346	222884_at	2	6.872	0.34	7	6.4	1.07	40	d	0.040568
			236267_at	2	6.26	0.47	7	5.591	0.91	40	d	0.068893
			232753_at	0	3.818	0.49	7	3.958	0.8	40	u	0.664839
KIAA0195	2	KIAA0195	222210_at	3	3.166	0.2	7	3.121	0.48	40	d	0.8116
			202650_s_at	2	6.89	0.37	7	6.631	0.78	40	d	0.402664
KIAA1333	2	KIAA1333	223256_at	2	6.661	0.54	7	7.051	0.84	40	u	0.252065
			223258_s_at	2	5.757	0.44	7	6.687	0.97	40	u	0.019036
			223255_at	2	6.609	0.3	7	7.478	0.67	40	u	0.001878
			223254_s_at	2	5.803	0.46	7	7.202	0.74	40	u	0.000021
			223257_at	2	5.432	0.25	7	5.914	0.81	40	u	0.006339
SCARB2	2	scavenger receptor class B, member 2	201647_s_at	2	7.578	0.74	7	7.916	1.64	40	u	0.602587
			224983_at	2	11.58	0.19	7	10.97	0.65	40	d	0.000067
			201646_at	2	9.168	0.64	7	9.154	1.3	40	d	0.978258
			215754_at	0	6.403	0.42	7	6.081	0.75	40	d	0.28795
MAPK3	2	mitogen-activated protein kinase 3	212046_x_at	0	8.685	0.26	7	8.546	0.5	40	d	0.485733

ZDHHC23	2	zinc finger, DHHC-type containing 23	226912_at	2	6.139	0.37	7	5.721	1	40	d	0.289027
CHIC2	2	cysteine-rich hydrophobic domain 2	219492_at	2	8.415	0.63	7	7.841	0.69	40	d	0.04994
CA2	2	carbonic anhydrase II	209301_at	2	7.93	1.34	7	7.211	2.45	40	d	0.463365
KIAA1467	2	KIAA1467	213232_at	2	1.798	0.02	7	1.841	0.07	40	u	0.002141
			213234_at	2	8.203	0.77	7	6.562	1.71	40	d	0.01861
			1559302_at	0	2.314	0.08	7	2.342	0.09	40	u	0.443159
TPO	2	thyroid peroxidase	210342_s_at	0	2.14	0.32	7	2.05	0.1	40	d	0.513208
PAFAH1B2	2	platelet-activating factor acetylhydrolase, isoform Ib, beta subunit 30kDa	210160_at	2	5.618	0.29	7	5.81	0.42	40	u	0.258356
			224777_s_at	0	9.976	0.15	7	9.749	0.57	40	d	0.04406
			1569431_at	0	2.46	0.04	7	2.542	0.15	40	u	0.010002
ETS1	2	v-ets erythroblastosis virus E26 oncogene homolog 1 (avian)	224833_at	16	9.13	0.57	7	9.067	0.85	40	d	0.854256
			1555355_a_at	2	4.577	0.5	7	5.163	1.01	40	u	0.148724
			214447_at	0	2.41	0.04	7	2.784	0.6	40	u	0.000388
WBP2	2	WW domain binding protein 2	209117_at	0	8.715	0.21	7	8.351	0.9	40	d	0.035438
GTDC1	2	glycosyltransferase-like domain containing 1	219770_at	2	6.816	0.34	7	6.844	0.71	40	u	0.920657
			239390_at	0	3.324	0.56	7	3.097	0.55	40	d	0.328774
			220853_at	0	2.073	0.1	7	2.109	0.18	40	u	0.62637
			238585_at	0	2.838	0.58	7	3.749	1.09	40	u	0.040178
TRIM44	2	tripartite motif-containing 44	217759_at	2	10.08	0.22	7	9.595	0.62	40	d	0.001508
			217760_at	2	8.43	0.22	7	8.2	0.65	40	d	0.109523
SUHW2	2	suppressor of hairy wing homolog 2 (Drosophila)	230789_at	2	2.638	0.08	7	3.132	0.89	40	u	0.001525
			215048_at	0	2.726	0.1	7	2.936	0.23	40	u	0.024484
			229360_at	0	2.584	0.38	7	3.636	1.41	40	u	0.000525
TGFA	2	transforming growth factor, alpha	205016_at	2	6.674	0.7	7	6.333	1.64	40	d	0.597869
			211258_s_at	1	2.947	0.19	7	3.422	0.86	40	u	0.004277
			205015_s_at	1	2.616	0.35	7	2.92	1.17	40	u	0.205366
AP3S1	2	adaptor-related protein complex 3, sigma 1 subunit	202442_at	2	10.71	0.18	7	10.66	0.72	40	d	0.687253
SNX3	2	sorting nexin 3	210648_x_at	2	11.77	0.13	7	11.59	0.69	40	d	0.155094
			213545_x_at	1	9.278	0.16	7	9.131	0.78	40	d	0.306465
			208781_x_at	0	10.59	0.19	7	10.41	0.92	40	d	0.289726
			200067_x_at	0	10.42	0.1	7	10.31	0.73	40	d	0.399272
DST	2	dystonin	212253_x_at	2	8.934	0.48	7	7.119	0.99	40	d	0.000029
			215016_x_at	2	10.29	0.26	7	8.561	0.98	40	d	0
			212254_s_at	2	10.34	0.28	7	8.549	0.96	40	d	0
			204455_at	0	11.68	0.34	7	6.093	1.49	40	d	0
			1553191_at	0	2.432	0.03	7	2.544	0.16	40	u	0.000386
			232098_at	0	7.99	0.87	7	6.048	1.33	40	d	0.000695
			216918_s_at	0	11.54	0.34	7	6.834	0.98	40	d	0
			220154_at	0	2.503	0.24	7	2.579	0.54	40	u	0.721627
BAP1	2	BRCA1 associated protein-1 (ubiquitin carboxy-terminal hydrolase)	201419_at	2	8.243	0.33	7	7.663	0.77	40	d	0.059943
			1555735_a_at	1	6.029	0.23	7	5.533	1.17	40	d	0.022778
CYLD	2	cylindromatosis (turban tumor syndrome)	60084_at	0	2.607	0.07	7	2.822	0.22	40	u	0.000071
			39582_at	0	7.449	0.22	7	6.579	0.86	40	d	0.000006
			222142_at	0	2.953	0.27	7	3.073	0.27	40	u	0.303333
			213295_at	0	8.262	0.19	7	7.249	1	40	d	0.000001
			214272_at	0	3.582	0.29	7	3.87	0.49	40	u	0.145207
			221905_at	0	7.683	0.35	7	7.578	0.79	40	d	0.736102
			221903_s_at	0	7.14	0.21	7	7.616	0.78	40	u	0.003303
MTMR2	2	myotubularin related protein 2	203211_s_at	2	8.038	0.3	7	8.52	0.98	40	u	0.021297
			203212_s_at	2	6.138	0.34	7	6.124	0.87	40	d	0.967321
			214649_s_at	1	5.984	0.37	7	6.582	1.02	40	u	0.013052
OLFML2B	2	olfactomedin-like 2B	213125_at	2	7.31	0.53	7	7.277	0.94	40	d	0.92959
KCNRG	2	potassium channel regulator	239098_at	2	2.855	0.35	7	2.842	0.32	40	d	0.928934
			240288_at	2	3.828	0.42	7	3.975	0.44	40	u	0.430201
RGS9	2	regulator of G-protein signaling 9	206518_s_at	2	3.994	0.69	7	3.733	0.72	40	d	0.386862
TMEM163	2	transmembrane protein 163	1552626_a_at	2	4.921	0.85	7	4.452	0.89	40	d	0.212031
			223503_at	2	4.991	0.86	7	4.35	0.99	40	d	0.122363
GNB5	2	guanine nucleotide binding protein (G protein), beta 5	204000_at	2	7.489	0.24	7	6.152	0.9	40	d	0
			211871_x_at	0	5.449	0.67	7	4.264	0.62	40	d	0.000045
			1554346_at	0	2.56	0.42	7	2.63	0.36	40	u	0.650216
			207124_s_at	0	6.256	0.68	7	4.756	1.28	40	d	0.004756
IPO11	2	importin 11	222659_at	2	7.268	0.27	7	7.19	0.55	40	d	0.720807
			234304_s_at	1	6.3	0.4	7	6.568	0.83	40	u	0.418633

IPO11	2	importin 11	1557770_at	0	2.438	0.07	7	2.791	0.44	40	u	0.000036
			238488_at	0	4.639	0.95	7	3.134	0.53	40	d	0.009131
UBTD2	2	ubiquitin domain containing 2	224827_at	0	8.538	0.25	7	8.478	0.85	40	d	0.727643
			224834_at	0	8.851	0.41	7	8.74	0.7	40	d	0.692466
ZSWIM7	2	zinc finger, SWIM-type containing 7	228719_at	0	5.967	0.45	7	5.507	0.49	40	d	0.027439
			229119_s_at	0	8.15	0.28	7	7.428	1.06	40	d	0.00127
ARC	2	activity-regulated cytoskeleton-associated protein	210090_at	1	3.522	0.82	7	2.676	0.57	40	d	0.001792
PORCN	2	porcupine homolog (Drosophila)	219483_s_at	0	5.841	0.28	7	5.697	0.55	40	d	0.512839
GLT25D2	2	glycosyltransferase 25 domain containing 2	209883_at	2	3.143	0.42	7	2.883	0.51	40	d	0.22236
RGS6	2	regulator of G-protein signaling 6	210270_at	2	3.252	0.14	7	3.327	0.28	40	u	0.492896
			211448_s_at	2	2.917	0.29	7	2.951	0.33	40	u	0.804112
			214538_x_at	0	2.435	0.52	7	2.435	0.43	40	u	0.999026
TBC1D10B	2	TBC1 domain family, member 10B	220947_s_at	2	7.621	0.35	7	7.877	0.75	40	u	0.391354
ACCN1	2	amiloride-sensitive cation channel 1, neuronal (degenerin)	206690_at	2	2.628	0.22	7	2.731	0.44	40	u	0.558834
SLC45A3	2	solute carrier family 45, member 3	228696_at	2	5.452	0.32	7	5.412	0.59	40	d	0.866371
			238499_at	0	2.752	0.26	7	2.829	0.37	40	u	0.602798
KIAA2013	2	KIAA2013	224708_at	2	7.846	0.16	7	7.984	0.58	40	u	0.227815
			224706_at	2	8.725	0.27	7	8.65	0.58	40	d	0.743907
			1555933_at	0	5.424	0.3	7	5.663	0.45	40	u	0.193539
RBBP5	2	retinoblastoma binding protein 5	205169_at	2	6.121	0.43	7	5.896	0.85	40	d	0.505342
PPFIA1	2	protein tyrosine phosphatase, receptor type, f polypeptide (PTPRF), interacting protein (liprin), alpha 1	202066_at	2	8.888	0.33	7	9.174	0.9	40	u	0.419628
			202065_s_at	2	6.108	0.3	7	6.902	0.9	40	u	0.000292
			210236_at	0	3.767	0.36	7	4.553	0.98	40	u	0.045005
			210235_s_at	0	6.569	0.42	7	6.749	1.37	40	u	0.522324
ORC2L	2	origin recognition complex, subunit 2-like (yeast)	204853_at	2	6.965	0.18	7	7.223	0.49	40	u	0.024528
CUL2	2	cullin 2	203079_s_at	2	7.309	0.27	7	7.887	0.72	40	u	0.045547
			203078_at	0	4.36	0.31	7	4.935	0.66	40	u	0.031453
CAMKV	2	CaM kinase-like vesicle-associated	219365_s_at	2	3.513	0.35	7	3.455	0.64	40	d	0.820511
AUP1	2	ancient ubiquitous protein 1	220525_s_at	2	9.316	0.18	7	9.484	0.53	40	u	0.151475
GSTO1	2	glutathione S-transferase omega 1	201470_at	0	10.33	0.17	7	10.32	0.63	40	d	0.99297
			1557915_s_at	0	9.999	0.18	7	9.916	0.74	40	d	0.5548
CCDC128	2	coiled-coil domain containing 128	1553955_at	2	7.971	0.31	7	8.091	0.63	40	u	0.630053
			1554145_a_at	0	5.525	0.5	7	6.059	0.92	40	u	0.149988
LSM1	2	LSM1 homolog, U6 small nuclear RNA associated (S. cerevisiae)	203534_at	2	9.079	0.19	7	9.846	1.16	40	u	0.000444
SCAMP2	2	secretory carrier membrane protein 2	224921_at	2	8.543	0.27	7	8.069	0.57	40	d	0.040365
			218143_s_at	2	7.964	0.24	7	7.892	0.55	40	d	0.740108
SGSM2	2	small G protein signaling modulator 2	212319_at	2	7.927	0.24	7	6.434	1.1	40	d	0
			36129_at	2	9.909	0.2	7	8.535	0.88	40	d	0
			217538_at	0	5.36	0.52	7	4.4	0.86	40	d	0.007334
SART3	2	squamous cell carcinoma antigen recognized by T cells 3	200069_at	2	7.295	0.25	7	7.168	0.78	40	d	0.439203
			209128_s_at	2	8.327	0.27	7	8.068	0.58	40	d	0.265989
			209127_s_at	2	7.46	0.44	7	8.266	0.81	40	u	0.015813
			1554276_at	0	2.082	0.02	7	2.143	0.1	40	u	0.001276
FAM114A1	2	family with sequence similarity 114, member A1	226697_at	2	6.712	0.29	7	8.19	0.78	40	u	0.000014
			213455_at	0	9.198	0.26	7	9.083	0.52	40	d	0.576284
TJP2	2	tight junction protein 2 (zona occludens 2)	202085_at	2	8.292	0.34	7	7.977	0.91	40	d	0.381897
			242991_at	0	3.579	0.3	7	3.541	0.3	40	d	0.766311
			232017_at	0	6.528	0.67	7	6.015	1.15	40	d	0.268336
			237132_at	0	2.184	0.13	7	2.268	0.23	40	u	0.352018
RAB27A	2	RAB27A, member RAS oncogene family	209514_s_at	2	6.703	0.49	7	7.553	0.88	40	u	0.019051
			209515_s_at	2	8.679	0.18	7	8.436	0.82	40	d	0.114237
			210951_x_at	0	6.991	0.46	7	7.548	1.02	40	u	0.172263
PRKAB1	2	protein kinase, AMP-activated, beta 1 non-catalytic subunit	201834_at	2	6.639	0.33	7	6.181	0.69	40	d	0.098508
			201835_s_at	0	5.679	0.47	7	4.884	0.98	40	d	0.044828
KIF20A	2	kinesin family member 20A	218755_at	2	3.062	0.76	7	6.897	1.47	40	u	0
ZNF654	2	zinc finger protein 654	222851_at	2	4.578	0.18	7	4.814	0.86	40	u	0.141756
			219239_s_at	2	6.14	0.54	7	6.826	1.13	40	u	0.128759
			1556744_a_at	0	6.74	0.35	7	6.785	0.95	40	u	0.905833
			1556743_at	0	3.806	0.36	7	4.125	0.42	40	u	0.068691

			241348_at	0	6.203	0.74	7	6.455	0.86	40	u	0.478124
			242017_at	0	3.31	0.31	7	3.572	0.55	40	u	0.234384
SYVN1	2	synovial apoptosis inhibitor 1, synoviolin	225090_at	2	8.187	0.27	7	7.436	0.97	40	d	0.000402
			244522_at	0	2.447	0.16	7	2.422	0.08	40	d	0.725638
ABLIM3	2	actin binding LIM protein family, member 3	205730_s_at	2	7.587	0.31	7	5.026	1.56	40	d	0
C2orf42	2	chromosome 2 open reading frame 42	219128_at	2	6.157	0.36	7	5.848	0.55	40	d	0.170123
BRF2	2	BRF2, subunit of RNA polymerase III transcription initiation factor, BRF1-like	218955_at	2	7.679	0.13	7	8.094	1.11	40	u	0.030029
			218954_s_at	0	5.991	0.24	7	6.143	1.5	40	u	0.5598
NUMA1	2	nuclear mitotic apparatus protein 1	214251_s_at	2	7.313	0.58	7	6.233	1.56	40	d	0.08361
			214250_at	2	5.967	0.41	7	5.911	1.08	40	d	0.894699
			200747_s_at	2	10.15	0.45	7	8.75	0.88	40	d	0.000222
			1554663_a_at	0	2.74	1.21	7	2.502	0.67	40	d	0.654734
C11orf31	2	chromosome 11 open reading frame 31	228331_at	2	5.453	0.47	7	5.264	0.79	40	d	0.549113
			1557801_x_at	2	6.133	0.24	7	6.551	0.72	40	u	0.010514
			224677_x_at	2	9.674	0.21	7	9.798	0.7	40	u	0.387023
			228332_s_at	2	10.89	0.14	7	11.21	0.66	40	u	0.010395
			231045_x_at	2	10.62	0.15	7	10.75	0.69	40	u	0.297677
			1557799_at	2	5.842	0.25	7	6.473	0.59	40	u	0.008659
ZNF770	2	zinc finger protein 770	225517_at	2	8.75	0.22	7	8.963	0.61	40	u	0.375197
			238687_x_at	0	4.116	0.2	7	4.897	0.72	40	u	0.000004
ZNF436	2	zinc finger protein 436	226113_at	2	8.448	0.43	7	7.679	0.7	40	d	0.008887
			226114_at	2	7.013	0.36	7	6.056	1.21	40	d	0.00046
KCNK5	2	potassium channel, subfamily K, member 5	219615_s_at	2	4.64	0.36	7	5.607	1.7	40	u	0.003265
KLF10	2	Kruppel-like factor 10	202393_s_at	2	9.97	0.58	7	8.812	0.79	40	d	0.000751
PSME3	2	proteasome (prosome, macropain) activator subunit 3 (PA28 gamma; Ki)	209852_x_at	2	8.844	0.21	7	8.964	0.67	40	u	0.394576
			200988_s_at	2	6.076	0.3	7	5.673	0.83	40	d	0.035834
			200987_x_at	2	7.718	0.41	7	8.143	1.07	40	u	0.313308
			209853_s_at	0	6.605	0.33	7	7.166	1.12	40	u	0.01805
CXorf45	2	chromosome X open reading frame 45	205583_s_at	2	8.052	0.42	7	6.994	1.17	40	d	0.000389
			205584_at	2	7.18	0.48	7	5.998	1.13	40	d	0.01079
			232435_at	0	2.591	0.28	7	3.136	0.65	40	u	0.036868
			1559057_at	0	3.609	0.3	7	3.614	0.47	40	u	0.976792
ING1	2	inhibitor of growth family, member 1	209808_x_at	2	6.031	0.38	7	6.261	0.72	40	u	0.420817
			208415_x_at	2	8.069	0.29	7	7.961	0.62	40	d	0.657806
			1557179_s_at	0	2.3	0.06	7	2.429	0.22	40	u	0.004456
			244817_at	0	3.061	0.06	7	3.171	0.16	40	u	0.076036
			210350_x_at	0	5.112	0.26	7	5.251	0.66	40	u	0.593128
			237555_at	0	3.156	0.4	7	3.27	0.59	40	u	0.631549
PHOSPHO1	2	phosphatase, orphan 1	236218_at	0	2.173	0.03	7	2.314	0.37	40	u	0.024331
SALL3	2	sal-like 3 (Drosophila)	1553411_s_at	2	2.487	0.16	7	2.697	0.66	40	u	0.1005
			232847_at	0	2.694	0.03	7	2.922	0.34	40	u	0.000187
TMED1	2	transmembrane emp24 protein transport domain containing 1	203679_at	2	7.491	0.32	7	7.189	0.68	40	d	0.264408
CAPN7	2	calpain 7	203356_at	2	7.456	0.41	7	7.572	0.71	40	u	0.683878
			203357_s_at	0	8.017	0.5	7	7.727	1.44	40	d	0.354157
ZSCAN22	2	zinc finger and SCAN domain containing 22	228769_at	2	4.188	0.67	7	4.327	0.86	40	u	0.691346
SYT9	2	synaptotagmin IX	1563658_a_at	2	2.409	0.05	7	2.529	0.66	40	u	0.270346
			232445_at	2	3.198	0.6	7	3.202	0.56	40	u	0.988448
			1555052_a_at	0	2.189	0.35	7	2.096	0.52	40	d	0.657493
			1555053_at	0	2.668	0.07	7	2.824	0.19	40	u	0.043493
JPH3	2	junctophilin 3	229294_at	2	2.418	0.08	7	2.712	0.81	40	u	0.033539
			220188_at	1	2.33	0.04	7	2.518	0.31	40	u	0.000805
			234736_at	0	2.451	0.1	7	2.448	0.23	40	d	0.976865
			243679_at	0	2.271	0.26	7	2.232	0.16	40	d	0.605118
FAM38A	2	family with sequence similarity 38, member A	202771_at	2	8.659	0.36	7	8.097	1.16	40	d	0.024471
CCDC71	2	coiled-coil domain containing 71	219893_at	0	5.627	0.23	7	5.658	0.37	40	u	0.835699
ABHD6	2	abhydrolase domain containing 6	45288_at	2	5.401	0.63	7	4.636	0.75	40	d	0.016301
			221552_at	2	5.541	1.08	7	4.404	0.87	40	d	0.004248
			221678_at	0	2.729	0.18	7	2.773	0.24	40	u	0.654185
			221679_s_at	0	5.028	0.7	7	4.565	0.75	40	d	0.144085
			201171_at	2	4.425	0.58	7	4.764	1.02	40	u	0.409214

ATP6V0E1	2	ATPase, H+ transporting, lysosomal 9kDa, V0 subunit e1	214150_x_at	2	11.4	0.18	7	11.28	0.51	40	d	0.295024
			214149_s_at	2	6.899	0.31	7	7.883	0.78	40	u	0.002311
			201172_x_at	2	11.47	0.19	7	11.15	0.67	40	d	0.02332
			214244_s_at	2	5.242	0.46	7	5.825	0.88	40	u	0.10107
			200096_s_at	2	11.45	0.21	7	11.32	0.69	40	d	0.373709
NCOA4	2	nuclear receptor coactivator 4	210774_s_at	2	12.15	0.22	7	11.5	0.74	40	d	0.000155
FXYD3	2	FXYD domain containing ion transport regulator 3	202488_s_at	2	7.412	0.36	7	7.624	1.01	40	u	0.338983
			202489_s_at	0	10.78	0.38	7	10.61	1.36	40	d	0.529028
RNF183	2	ring finger protein 183	235153_at	2	5.194	1.17	7	4.319	1.32	40	d	0.115895
GRAMD3	2	GRAM domain containing 3	218706_s_at	2	10.44	0.25	7	7.91	0.85	40	d	0
			238049_at	0	7.897	0.5	7	5.249	0.89	40	d	0
CACNA1I	2	calcium channel, voltage-dependent, T type, alpha 1I subunit	221631_at	2	4.71	0.76	7	4.355	0.69	40	d	0.231884
			211830_s_at	0	2.298	0.28	7	2.237	0.1	40	d	0.614613
			208299_at	0	2.972	0.6	7	2.74	0.51	40	d	0.297333
ARMCX2	2	armadillo repeat containing, X-linked 2	203404_at	0	9.507	0.48	7	8.575	1.2	40	d	0.053152
VDAC2	2	voltage-dependent anion channel 2	211662_s_at	2	10.67	0.21	7	10.8	0.55	40	u	0.543765
SNX21	2	sorting nexin family member 21	1553961_s_at	2	5.868	0.56	7	5.448	0.72	40	d	0.157757
			226595_at	2	5.083	0.45	7	4.309	0.77	40	d	0.01533
			1553960_at	2	5.725	0.76	7	5.322	1.13	40	d	0.381196
FAM55C	2	family with sequence similarity 55, member C	235030_at	2	3.776	0.31	7	3.594	0.78	40	d	0.553864
			243011_at	0	3.892	0.46	7	4.625	0.93	40	u	0.052936
TMEM16F	2	transmembrane protein 16F	224906_at	2	9.3	0.42	7	8.484	0.45	40	d	0.000067
			240947_at	0	2.684	0.16	7	3.08	0.55	40	u	0.000902
ZNF622	2	zinc finger protein 622	225152_at	2	8.356	0.19	7	8.317	0.59	40	d	0.75201
			1560075_at	0	2.911	0.12	7	3.09	0.3	40	u	0.128743
CSF2	2	colony stimulating factor 2 (granulocyte-macrophage)	210228_at	2	4.773	0.15	7	4.701	0.36	40	d	0.607426
			210229_s_at	0	2.441	0.19	7	2.6	0.34	40	u	0.236358
RAB8A	2	RAB8A, member RAS oncogene family	208819_at	2	9.634	0.14	7	9.637	0.7	40	u	0.984184
CREB3L1	1	cAMP responsive element binding protein 3-like 1	213059_at	1	6.111	0.62	7	6.194	1.63	40	u	0.896137
			213498_at	0	2.542	0.23	7	2.828	0.81	40	u	0.082241
MMP2	1	matrix metallopeptidase 2 (gelatinase A, 72kDa gelatinase, 72kDa type IV collagenase)	201069_at	1	10.51	0.4	7	8.641	1.23	40	d	0
			1566678_at	0	3.354	0.19	7	3.441	0.31	40	u	0.482094
			1566677_at	0	2.54	0.04	7	2.637	0.13	40	u	0.00083
ABCA3	1	ATP-binding cassette, sub-family A (ABC1), member 3	204343_at	1	6.536	1.04	7	6.091	1.31	40	d	0.409037
C9orf39	1	chromosome 9 open reading frame 39	220095_at	1	2.952	0.57	7	3.307	0.9	40	u	0.329087
			1559005_s_at	0	3.304	0.52	7	3.708	0.99	40	u	0.309048
			241696_at	0	3.026	0.18	7	3.65	0.82	40	u	0.000151
TUBD1	1	tubulin, delta 1	231853_at	1	7.124	0.38	7	7.123	0.88	40	d	0.999151
			221326_s_at	0	4.146	0.36	7	4.707	0.82	40	u	0.090093
			210389_x_at	0	5.951	0.42	7	5.937	1.18	40	d	0.956977
NARG2	1	NMDA receptor regulated 2	228960_at	1	6.96	0.55	7	6.39	0.81	40	d	0.086172
			218713_at	1	7.702	0.28	7	7.646	0.69	40	d	0.839548
			235189_at	0	4.846	0.75	7	5.797	1.39	40	u	0.090654
SALL1	1	sal-like 1 (Drosophila)	229273_at	1	2.721	0.06	7	3.01	0.33	40	u	0.000014
			206893_at	1	2.319	0.03	7	2.455	0.13	40	u	0.000003
ZNF646	1	zinc finger protein 646	204876_at	1	4.668	0.45	7	4.539	0.55	40	d	0.571941
LCORL	1	ligand dependent nuclear receptor corepressor-like	232293_at	1	4.71	0.32	7	5.05	0.53	40	u	0.1131
			235970_at	0	5.228	0.53	7	5.656	0.85	40	u	0.214171
			240592_at	0	6.896	0.27	7	7.133	0.8	40	u	0.171012
MYO9B	1	myosin IXB	214780_s_at	1	7.231	0.34	7	7.134	0.54	40	d	0.656008
			217297_s_at	1	6.918	0.17	7	6.887	0.84	40	d	0.838266
			208452_x_at	0	4.786	0.66	7	4.947	0.78	40	u	0.618432
CENPQ	1	centromere protein Q	219294_at	1	3.291	0.31	7	4.16	0.9	40	u	0.000137
C9orf165	1	chromosome 9 open reading frame 165	228403_at	1	3.908	0.47	7	4.215	0.83	40	u	0.358245
CCDC97	1	coiled-coil domain containing 97	225257_at	1	6.501	0.42	7	5.941	0.74	40	d	0.06298
			225254_at	1	6.837	0.28	7	6.47	0.63	40	d	0.143468
			235495_at	0	2.414	0.08	7	2.509	0.24	40	u	0.06543
KCNK3	1	potassium channel, subfamily K, member 3	205952_at	1	3.374	1.23	7	3.281	2.01	40	d	0.908617
AQP9	1	aquaporin 9	205568_at	1	3.587	0.32	7	4.664	1.35	40	u	0.000126
AGPAT1	1	1-acylglycerol-3-phosphate O-acyltransferase 1 (lysophosphatidic acid acyltransferase,	32836_at	0	8.438	0.23	7	8.232	0.68	40	d	0.168979
			215535_s_at	0	8.569	0.22	7	8.403	0.7	40	d	0.257224
			217847_s_at	1	5.855	0.36	7	5.217	0.97	40	d	0.097953

THRAP3	1	thyroid hormone receptor associated protein 3	222439_s_at	0	8.153	0.36	7	8.377	0.68	40	u	0.412111
			222440_s_at	0	9.734	0.21	7	9.072	0.34	40	d	0.000014
			242163_at	0	2.857	0.23	7	3.607	0.67	40	u	0.000021
RNF185	1	ring finger protein 185	224750_at	1	7.396	0.29	7	6.695	0.62	40	d	0.005933
			207280_at	1	1.833	0.02	7	1.872	0.03	40	u	0.00466
IPO9	1	importin 9	217885_at	1	9.277	0.27	7	9.788	0.6	40	u	0.035606
			213785_at	0	5.217	0.44	7	5.098	0.74	40	d	0.691189
			244703_x_at	0	3.595	0.25	7	4.046	0.52	40	u	0.031665
			242561_at	0	3.254	0.33	7	3.682	0.53	40	u	0.05059
PPCS	1	phosphopantothenoylcysteine synthetase	218341_at	1	9.327	0.28	7	9.363	0.59	40	u	0.877016
ZFYVE1	1	zinc finger, FYVE domain containing 1	223387_at	1	8.289	0.37	7	7.209	0.64	40	d	0.000108
			223388_s_at	0	3.472	0.53	7	3.238	0.91	40	d	0.521718
SLC25A14	1	solute carrier family 25 (mitochondrial carrier, brain), member 14	204587_at	1	7.359	0.3	7	7.253	0.53	40	d	0.61898
			211855_s_at	0	5.828	0.62	7	5.647	0.84	40	d	0.59316
C20orf27	1	chromosome 20 open reading frame 27	50314_i_at	1	7.063	0.39	7	7.693	0.81	40	u	0.055746
			218081_at	1	6.653	0.57	7	7.223	0.7	40	u	0.051609
ACSM3	1	acyl-CoA synthetase medium-chain family member 3	210377_at	0	5.528	1.79	7	3.135	0.62	40	d	0.017416
			205942_s_at	0	4.713	2.11	7	2.231	0.47	40	d	0.028246
POU3F1	1	POU class 3 homeobox 1	210475_at	1	5.35	0.28	7	5.39	0.32	40	u	0.761927
			208345_s_at	1	3.085	0.19	7	3.108	0.27	40	u	0.837732
EED	1	embryonic ectoderm development	210656_at	1	3.935	0.69	7	4.522	1.24	40	u	0.236776
			209572_s_at	1	8.511	0.23	7	8.588	0.63	40	u	0.577844
RAB33B	1	RAB33B, member RAS oncogene family	221014_s_at	1	7.429	0.22	7	7.289	0.53	40	d	0.503365
ZNF212	1	zinc finger protein 212	203985_at	1	8.06	0.33	7	7.798	0.45	40	d	0.156834
KIAA0528	1	KIAA0528	212943_at	1	9.323	0.23	7	8.998	0.84	40	d	0.05349
FAM73A	1	family with sequence similarity 73, member A	235125_x_at	1	6.625	0.47	7	6.298	0.82	40	d	0.321074
			243042_at	1	5.536	0.67	7	4.89	0.98	40	d	0.10666
LGI2	1	leucine-rich repeat LGI family, member 2	219699_at	1	4.369	0.44	7	4.719	0.62	40	u	0.166656
C16orf45	1	chromosome 16 open reading frame 45	212736_at	1	7.125	0.44	7	5.769	1.83	40	d	0.000329
			1565641_at	0	5.611	0.43	7	5.693	0.61	40	u	0.741747
RBM10	1	RNA binding motif protein 10	217221_x_at	1	7.701	0.28	7	7.25	0.61	40	d	0.065287
			208984_x_at	1	7.765	0.34	7	7.474	0.63	40	d	0.247312
			215089_s_at	1	7.946	0.33	7	7.609	0.7	40	d	0.224994
DUSP4	1	dual specificity phosphatase 4	204015_s_at	1	9.759	0.67	7	7.607	1.85	40	d	0.000021
			204014_at	1	9.997	0.73	7	7.533	2.24	40	d	0.000015
ROR2	1	receptor tyrosine kinase-like orphan receptor 2	205578_at	1	5.019	0.48	7	4.654	1.01	40	d	0.362901
FBXL14	1	F-box and leucine-rich repeat protein 14	1553683_s_at	1	3.217	0.15	7	3.278	0.34	40	u	0.650453
			1553682_at	1	2.383	0.32	7	2.354	0.41	40	d	0.864602
			213145_at	0	8.039	0.11	7	8.388	0.76	40	u	0.010161
TUBB2C	1	tubulin, beta 2C	213726_x_at	1	11.12	0.1	7	11.29	0.42	40	u	0.028704
			208977_x_at	1	11.02	0.16	7	11.26	0.48	40	u	0.026579
HSPD1	1	heat shock 60kDa protein 1 (chaperonin)	200806_s_at	1	10.78	0.44	7	11.51	1.09	40	u	0.094732
			200807_s_at	1	12.56	0.24	7	13.13	0.51	40	u	0.00697
			241716_at	0	3.533	0.32	7	4.207	0.83	40	u	0.043156
			243372_at	0	3.284	0.44	7	3.328	0.67	40	u	0.870217
FAM19A4	1	family with sequence similarity 19 (chemokine (C-C motif)-like), member A4	242348_at	1	2.564	0.14	7	2.773	0.38	40	u	0.166865
CETN3	1	centrin, EF-hand protein, 3 (CDC31 homolog, yeast)	209662_at	1	8.831	0.25	7	8.616	0.7	40	d	0.166996
CCDC43	1	coiled-coil domain containing 43	225607_at	1	6.927	0.24	7	7.49	0.61	40	u	0.023651
COQ9	1	coenzyme Q9 homolog (S. cerevisiae)	212228_s_at	1	8.805	0.24	7	8.835	0.65	40	u	0.906964
			235822_at	0	4.093	0.25	7	4.233	0.75	40	u	0.386779
LYZL4	1	lysozyme-like 4	231306_at	1	2.059	0.17	7	2.091	0.21	40	u	0.703591
FNDC1	1	fibronectin type III domain containing 1	226930_at	1	5.135	0.81	7	7.353	1.24	40	u	0.000052
PREP	1	prolyl endopeptidase	37950_at	1	6.366	0.18	7	6.343	0.7	40	d	0.861945
			204117_at	1	7.071	0.25	7	7.102	0.76	40	u	0.847899
			229644_at	0	5.172	0.5	7	5.646	0.82	40	u	0.155832
CRELD2	1	cysteine-rich with EGF-like domains 2	218358_at	1	8.891	0.22	7	9.22	0.68	40	u	0.028369
COPS4	1	COP9 constitutive photomorphogenic homolog subunit 4 (Arabidopsis)	218042_at	1	9.279	0.27	7	9.208	0.58	40	d	0.756978
			237649_at	0	3.027	0.06	7	3.176	0.2	40	u	0.001138
BCHE	1	butyrylcholinesterase	205433_at	1	3.558	0.88	7	2.893	0.66	40	d	0.028012
LIM2	1	lens intrinsic membrane protein 2, 19kDa	220519_s_at	1	2.121	0.02	7	2.175	0.08	40	u	0.000604
GCLM	1	glutamate-cysteine ligase, modifier subunit	203925_at	1	7.972	0.41	7	8.598	0.9	40	u	0.082662

GCLM	1	glutamate-cysteine ligase, modifier subunit	236140_at	1	5.586	0.39	7	5.811	1.05	40	u	0.586114
SNX19	1	sorting nexin 19	202359_s_at	1	7.129	0.24	7	6.851	0.67	40	d	0.068539
			202358_s_at	1	7.662	0.27	7	7.66	0.66	40	d	0.994697
			1561710_at	0	2.335	0.05	7	2.428	0.14	40	u	0.098936
			1554986_a_at	0	3.474	0.66	7	3.838	1.05	40	u	0.391199
MLF1	1	myeloid leukemia factor 1	204783_at	1	6.632	0.81	7	7.111	1.03	40	u	0.259052
			204784_s_at	1	7.713	0.67	7	8.679	0.97	40	u	0.017162
SEC14L1	1	SEC14-like 1 (S. cerevisiae)	202084_s_at	1	9.765	0.29	7	8.731	0.63	40	d	0.000126
			202082_s_at	1	7.095	0.31	7	7.282	0.78	40	u	0.540754
			202083_s_at	1	4.746	0.35	7	4.647	0.79	40	d	0.747803
			240036_at	0	2.41	0.17	7	2.614	0.33	40	u	0.121348
COL18A1	1	collagen, type XVIII, alpha 1	1555953_at	1	2.579	0.11	7	2.874	0.39	40	u	0.000621
			209081_s_at	1	9.36	0.64	7	8.352	1.17	40	d	0.035403
			209082_s_at	1	8.245	0.55	7	7.229	1.3	40	d	0.051764
TNXB	1	tenascin XB	217413_s_at	0	3.265	0.62	7	3.023	0.61	40	d	0.352694
			206093_x_at	0	8.928	0.66	7	5.982	0.48	40	d	0
			216654_at	0	2.866	0.18	7	2.914	0.3	40	u	0.691487
			216333_x_at	0	9.035	0.48	7	5.918	0.4	40	d	0
			1565242_at	0	2.548	0.09	7	2.648	0.24	40	u	0.300319
			213451_x_at	0	9.19	0.49	7	6.017	0.57	40	d	0
C1QL1	1	complement component 1, q subcomponent-like 1	205575_at	1	5.086	0.57	7	5.052	0.75	40	d	0.911579
			214346_at	0	2.028	0.02	7	2.07	0.03	40	u	0.002848
C2orf44	1	chromosome 2 open reading frame 44	219120_at	1	4.739	0.2	7	5.218	0.66	40	u	0.001247
TOMM34	1	translocase of outer mitochondrial membrane 34	201870_at	1	7.398	0.21	7	7.841	0.52	40	u	0.035586
XRN2	1	5'-3' exoribonuclease 2	223002_s_at	1	8.079	0.22	7	8.023	0.92	40	d	0.747431
			233878_s_at	0	7.855	0.41	7	7.919	1.48	40	u	0.828274
FUS	1	fusion (involved in t(12;16) in malignant liposarcoma)	200959_at	1	10.37	0.34	7	10.08	0.59	40	d	0.218931
			215744_at	1	3.811	0.54	7	3.9	0.53	40	u	0.692482
			217370_x_at	0	7.825	0.5	7	7.927	0.99	40	u	0.794521
			1565717_s_at	0	8.128	0.88	7	8.283	1.77	40	u	0.826129
			1565715_at	0	2.232	0.05	7	2.286	0.13	40	u	0.303243
			1565716_at	0	2.601	0.6	7	2.444	0.78	40	d	0.6234
			231108_at	0	5.985	0.56	7	6.015	1	40	u	0.938794
CRHBP	1	corticotropin releasing hormone binding protein	205984_at	0	2.166	0.08	7	2.231	0.07	40	u	0.042729
CHST11	1	carbohydrate (chondroitin 4) sulfotransferase 11	219634_at	0	4.024	0.33	7	5.092	1.42	40	u	0.000235
			226368_at	0	4.826	0.72	7	6.287	1.2	40	u	0.003718
			226372_at	0	5.165	0.56	7	6.398	1.29	40	u	0.019205
RNF39	1	ring finger protein 39	219916_s_at	0	8.165	0.73	7	4.886	0.89	40	d	0
MYBPC1	1	myosin binding protein C, slow type	214087_s_at	1	8.687	2.03	7	5.04	2.74	40	d	0.001922
MTMR14	1	myotubularin related protein 14	222143_s_at	1	7.643	0.26	7	7.563	0.68	40	d	0.764984
			232342_at	0	4.144	0.26	7	3.926	0.4	40	d	0.179795
DNAL4	1	dynein, axonemal, light chain 4	204008_at	1	6.147	0.38	7	6.178	0.75	40	u	0.91823
CDC42BPA	1	CDC42 binding protein kinase alpha (DMPK-like)	203794_at	1	7.529	0.3	7	7.313	0.88	40	d	0.258653
			213595_s_at	1	6.1	0.23	7	6.448	0.48	40	u	0.070725
			214464_at	0	8.453	0.34	7	9.395	1.1	40	u	0.000224
			240735_at	0	3.718	0.31	7	4.072	0.55	40	u	0.11411
			215296_at	0	6.044	0.29	7	5.684	0.69	40	d	0.188268
C14orf141	1	chromosome 14 open reading frame 141	no probeset available									
ADAM23	1	ADAM metallopeptidase domain 23	1559268_at	1	2.459	0.08	7	2.742	0.69	40	u	0.018757
			206046_at	1	3.378	0.41	7	3.408	1.07	40	u	0.943473
TFR2	1	transferrin receptor 2	210215_at	1	2.807	0.17	7	3.052	0.48	40	u	0.026475
			215863_at	0	5.101	0.26	7	4.894	0.38	40	d	0.184231
			207883_s_at	0	3.876	0.33	7	3.627	0.33	40	d	0.078622
NCDN	1	neurochondrin	209556_at	1	5.913	0.39	7	5.903	0.96	40	d	0.977528
			209557_s_at	0	2.964	0.5	7	3.147	0.87	40	u	0.598187
			237002_at	0	3.197	0.51	7	3.358	0.71	40	u	0.578322
HSF2	1	heat shock transcription factor 2	209657_s_at	1	7.277	0.21	7	7.334	0.79	40	u	0.711802
			211220_s_at	0	4.224	0.47	7	4.781	0.9	40	u	0.125104
NUCB1	1	nucleobindin 1	200646_s_at	1	8.354	0.79	7	7.617	1.06	40	d	0.092453
			200649_at	1	9.715	0.17	7	8.801	0.65	40	d	0
SNAPC1	1	small nuclear RNA activating complex, polypeptide 1, 43kDa	205443_at	0	6.753	0.38	7	7.301	0.74	40	u	0.0658

SFRS5	1	splicing factor, arginine/serine-rich 5	203380_x_at	1	11.38	0.33	7	9.421	0.78	40	d	0
			210077_s_at	0	5.965	0.42	7	5.68	0.76	40	d	0.352024
			212266_s_at	0	12.31	0.39	7	10.33	0.9	40	d	0.000001
CAPN1	1	calpain 1, (mu/l) large subunit	200752_s_at	1	8.075	0.21	7	7.647	0.99	40	d	0.022044
			232012_at	0	7.226	0.37	7	7.757	0.7	40	u	0.060705
DDHD2	1	DDHD domain containing 2	212690_at	1	9.009	0.36	7	8.666	1.45	40	d	0.218122
GBGT1	1	globoside alpha-1,3-N-acetylgalactosaminyltransferase 1	231780_at	1	5.201	0.42	7	5.232	0.7	40	u	0.913401
UNC13B	1	unc-13 homolog B (C. elegans)	202893_at	1	7.879	0.33	7	7.184	0.84	40	d	0.039245
			231206_at	0	2.02	0.03	7	2.103	0.22	40	u	0.032151
DPYD	1	dihydropyrimidine dehydrogenase	204646_at	1	8.272	0.42	7	6.827	1.12	40	d	0.001875
			1554534_at	0	2.402	0.4	7	2.414	0.43	40	u	0.944312
			1554536_at	0	2.461	0.05	7	2.592	0.12	40	u	0.008617
CNTN2	1	contactin 2 (axonal)	206970_at	1	2.404	0.11	7	2.47	0.24	40	u	0.487579
			230045_at	1	2.212	0.06	7	2.36	0.53	40	u	0.096948
IL6R	1	interleukin 6 receptor	226333_at	1	5.967	0.36	7	5.997	1.07	40	u	0.898166
			205945_at	1	5.785	0.9	7	5.66	1.38	40	d	0.821373
			217489_s_at	0	2.712	0.25	7	2.883	0.49	40	u	0.384894
VPS25	1	vacuolar protein sorting 25 homolog (S. cerevisiae)	224608_s_at	1	9.268	0.23	7	9.158	0.78	40	d	0.486348
FUT4	1	fucosyltransferase 4 (alpha (1,3) fucosyltransferase, myeloid-specific)	209893_s_at	1	4.218	0.44	7	4.576	0.88	40	u	0.307247
			209892_at	1	4.264	0.63	7	4.136	1.09	40	d	0.769375
CAPN12	1	calpain 12	228705_at	7	3.249	0.38	7	3.687	0.72	40	u	0.133803
TFAP2B	1	transcription factor AP-2 beta (activating enhancer binding protein 2 beta)	214451_at	1	9.27	0.59	7	5.583	3.17	40	d	0
			1553394_a_at	0	7.497	0.8	7	4.54	2.6	40	d	0.000005
			215686_x_at	0	4.313	0.58	7	3.852	0.8	40	d	0.159814
QRICH1	1	glutamine-rich 1	209174_s_at	1	8.334	0.25	7	7.724	0.51	40	d	0.004154
			229806_at	1	2.354	0.22	7	2.475	0.24	40	u	0.238453
TPBG	1	trophoblast glycoprotein	203476_at	1	10.4	0.4	7	9.288	1.25	40	d	0.000203
OSR2	1	odd-skipped related 2 (Drosophila)	213568_at	1	6.737	0.49	7	6.236	1.21	40	d	0.29747
SLC9A3R1	1	solute carrier family 9 (sodium/hydrogen exchanger), member 3 regulator 1	201349_at	1	9.897	0.87	7	9.302	1.37	40	d	0.282767
STAT5A	1	signal transducer and activator of transcription 5A	203010_at	1	7.636	0.4	7	6.686	0.75	40	d	0.002447
SMTNL2	1	smoothelin-like 2	229730_at	1	2.413	0.18	7	2.507	0.31	40	u	0.451439
FAM18B	1	family with sequence similarity 18, member B	218446_s_at	1	8.378	0.14	7	8.664	0.73	40	u	0.032761
HCFC1	1	host cell factor C1 (VP16-accessory protein)	202473_x_at	1	5.237	0.39	7	5.275	0.47	40	u	0.843798
			202474_s_at	1	7.652	0.52	7	7.08	0.87	40	d	0.10506
			231177_at	0	1.858	0.08	7	1.855	0.07	40	d	0.910309
FAM108A1	1	family with sequence similarity 108, member A1	221267_s_at	1	9.949	0.21	7	9.528	0.58	40	d	0.003024
C6orf35	1	chromosome 6 open reading frame 35	230457_at	0	2.817	0.37	7	2.784	0.64	40	d	0.896616
			218453_s_at	0	2.603	0.51	7	2.736	0.53	40	u	0.551141
			222638_s_at	0	7.715	0.12	7	7.611	0.42	40	d	0.224107
FHL1	1	four and a half LIM domains 1	210299_s_at	1	9.284	1.15	7	6.175	1.06	40	d	0
			214505_s_at	1	9.484	1.51	7	6.286	1.21	40	d	0
			201539_s_at	1	7.823	1.85	7	4.425	1.14	40	d	0
			201540_at	1	11.25	1	7	7.582	1.43	40	d	0
			210298_x_at	0	9.075	1.56	7	5.868	1.05	40	d	0
HK3	1	hexokinase 3 (white cell)	205936_s_at	1	2.907	0.58	7	3.273	0.72	40	u	0.219767
MYLK2	1	myosin light chain kinase 2, skeletal muscle	231792_at	1	2.872	0.08	7	2.939	0.2	40	u	0.405109
			1568926_x_at	0	3.747	0.45	7	3.739	0.32	40	d	0.957646
			1568925_at	0	4.302	0.48	7	4.367	0.29	40	u	0.640263
			1562411_at	0	2.43	0.17	7	2.515	0.28	40	u	0.44091
ATP10A	1	ATPase, Class V, type 10A	214256_at	1	2.277	0.15	7	2.371	0.18	40	u	0.20858
			214255_at	1	5.894	0.19	7	6.165	0.55	40	u	0.030598
			1568743_at	0	2.689	0.22	7	2.868	0.26	40	u	0.10325
DPH4	1	DPH4, JJJ3 homolog (S. cerevisiae)	213853_at	1	7.822	0.26	7	7.792	0.64	40	d	0.90539
			242562_at	0	3.814	0.35	7	4.142	0.53	40	u	0.128715
			1570048_at	0	4.632	0.86	7	4.339	0.78	40	d	0.382217
IRX3	1	iroquois homeobox 3	229638_at	1	11.4	0.33	7	10.76	0.77	40	d	0.039826
COQ10B	1	coenzyme Q10 homolog B (S. cerevisiae)	219397_at	1	8.454	0.42	7	8.296	0.58	40	d	0.505036
			1561948_at	0	2.633	0.09	7	2.709	0.15	40	u	0.217622

SORBS2	1	sorbin and SH3 domain containing 2	204288_s_at	1	9.746	0.23	7	7.918	1.88	40	d	0.000001
			1558815_at	0	6.409	0.2	7	5.946	1.04	40	d	0.016436
			225728_at	0	9.855	0.3	7	7.729	2.12	40	d	0
			233720_at	0	4.896	0.41	7	3.729	0.93	40	d	0.002452
			1555472_at	0	2.367	0.03	7	2.459	0.07	40	u	0.002548
			237285_at	0	5.132	0.62	7	4.975	0.44	40	d	0.429518
			220858_at	0	6.341	0.64	7	5.547	0.87	40	d	0.027989
SKAP2	1	src kinase associated phosphoprotein 2	225639_at	1	8.616	0.38	7	7.554	1.21	40	d	0.000197
			216899_s_at	0	7.734	0.62	7	6.508	1.64	40	d	0.062898
			204361_s_at	0	6.305	0.37	7	5.398	1.21	40	d	0.000931
			204362_at	0	9.151	0.33	7	7.925	1.17	40	d	0.000008
			241331_at	0	2.237	0.07	7	2.412	0.38	40	u	0.011123
KRCC1	1	lysine-rich coiled-coil 1	233329_s_at	1	8.184	0.32	7	8.334	0.86	40	u	0.655816
			218303_x_at	1	9.882	0.3	7	9.583	0.78	40	d	0.332344
			222585_x_at	1	8.301	0.32	7	8.131	0.82	40	d	0.598207
TAGAP	1	T-cell activation GTPase activating protein	229723_at	1	4.964	0.76	7	5.392	1.4	40	u	0.445298
			1552542_s_at	0	2.36	0.33	7	2.648	0.61	40	u	0.236252
			1552541_at	0	6.025	0.26	7	6.384	0.48	40	u	0.067571
			234050_at	0	2.605	0.2	7	2.889	0.39	40	u	0.074119
HS1BP3	1	HCLS1 binding protein 3	219020_at	1	7.341	0.28	7	7.894	0.6	40	u	0.022687
			237407_at	0	2.677	0.11	7	2.734	0.19	40	u	0.450292
			1568734_a_at	0	2.569	0.27	7	2.733	0.26	40	u	0.139728
			236530_at	0	2.486	0.1	7	2.644	0.23	40	u	0.094199
OLFML2A	1	olfactomedin-like 2A	213075_at	1	8.656	0.4	7	6.11	1.2	40	d	0
HAPLN4	1	hyaluronan and proteoglycan link protein 4	235420_at	1	1.905	0.02	7	1.973	0.21	40	u	0.05599
SFRS6	1	splicing factor, arginine/serine-rich 6	208804_s_at	1	11.19	0.26	7	9.77	0.93	40	d	0
			206108_s_at	1	6.528	1.92	7	6.226	1.74	40	d	0.684473
UIMC1	1	ubiquitin interaction motif containing 1	220746_s_at	1	7.841	0.27	7	7.166	0.91	40	d	0.000796
SFRP2	1	secreted frizzled-related protein 2	223122_s_at	1	11.38	0.29	7	9.734	1.95	40	d	0.000012
			223121_s_at	1	10.66	0.47	7	9.169	1.86	40	d	0.000165
RIC3	1	resistance to inhibitors of cholinesterase 3 homolog (C. elegans)	220282_at	1	2.857	0.3	7	2.762	0.67	40	d	0.716556
C10orf104	1	chromosome 10 open reading frame 104	224665_at	1	9.899	0.19	7	9.234	0.61	40	d	0.000011
			229145_at	1	8.861	0.23	7	8.002	0.7	40	d	0.000003
			224664_at	1	11.34	0.15	7	10.98	0.54	40	d	0.002096
			224667_x_at	1	9.063	0.3	7	8.79	0.56	40	d	0.227824
CD151	1	CD151 molecule (Raph blood group)	204306_s_at	1	10.44	0.37	7	9.748	0.73	40	d	0.020312
FAM26B	1	family with sequence similarity 26, member B	221565_s_at	1	7.723	0.34	7	7.211	0.62	40	d	0.041866
			57715_at	1	8.133	0.38	7	7.586	0.58	40	d	0.023757
EIF4E2	1	eukaryotic translation initiation factor 4E family member 2	209393_s_at	1	7.636	0.29	7	8.176	0.52	40	u	0.011397
			213571_s_at	0	8.081	0.2	7	8.758	0.52	40	u	0.001741
			226734_at	0	7.046	0.41	7	6.908	0.66	40	d	0.603773
			213570_at	0	4.748	0.28	7	4.954	0.83	40	u	0.248084
ZNF324	1	zinc finger protein 324	205182_s_at	1	6.917	0.22	7	6.21	0.63	40	d	0.000028
PRR6	1	proline rich 6	226610_at	1	6.213	0.48	7	6.465	2.1	40	u	0.519754
			226611_s_at	1	6.496	0.49	7	6.342	2.34	40	d	0.718459
			230436_s_at	0	1.93	0.02	7	1.973	0.05	40	u	0.02317
PITX1	1	paired-like homeodomain 1	208502_s_at	0	3.851	0.48	7	6.332	1.85	40	u	0
			209587_at	0	3.286	0.41	7	3.815	0.98	40	u	0.176121
JUND	1	jun D proto-oncogene	203751_x_at	1	8.121	0.6	7	6.837	1.26	40	d	0.012909
			203752_s_at	1	12.94	0.21	7	11.98	0.65	40	d	0
			214326_x_at	0	8.054	0.61	7	6.808	1.07	40	d	0.005272
			229117_s_at	0	2.167	0.2	7	2.14	0.14	40	d	0.666619
NDRG2	1	NDRG family member 2	206453_s_at	1	11.38	0.37	7	8.753	1.38	40	d	0
			214279_s_at	0	8.96	0.79	7	6.052	1.99	40	d	0.000521
			214278_s_at	0	2.705	0.27	7	2.63	0.22	40	d	0.442514
C19orf2	1	chromosome 19 open reading frame 2	210157_at	1	4.62	0.53	7	4.798	1.02	40	u	0.660724
			214173_x_at	1	9.874	0.17	7	9.9	0.69	40	u	0.845322
			222266_at	0	8.71	0.29	7	8.524	1.02	40	d	0.358021
			211563_s_at	0	7.681	0.24	7	7.518	0.95	40	d	0.37303
MS4A13	1	rane-spanning 4-domains, subfamily A, memb	no probeset available									
SEMA3G	1	sema domain, immunoglobulin domain (Ig), short basic domain, secreted, (semaphorin) 3G	219689_at	1	8.266	0.4	7	5.413	0.83	40	d	0

IL23A	1	interleukin 23, alpha subunit p19	220054_at	1	3.151	0.2	7	3.33	0.55	40	u	0.405963
			1568513_x_at	0	8.823	0.21	7	9.259	0.64	40	u	0.002835
			217328_at	0	2.248	0.18	7	2.233	0.25	40	d	0.880383
			213193_x_at	0	7.332	0.97	7	7.539	1.62	40	u	0.749852
			217326_x_at	0	2.66	0.59	7	2.873	0.64	40	u	0.42353
			240102_at	0	1.901	0.01	7	2.155	0.49	40	u	0.002391
			240766_at	0	2.378	0.08	7	2.462	0.27	40	u	0.141781
			234377_at	0	2.898	0.31	7	2.998	0.31	40	u	0.439129
			234883_x_at	0	2.334	0.18	7	2.361	0.16	40	u	0.703464
			234865_at	0	2.515	0.12	7	2.606	0.19	40	u	0.248525
SEPT12	1	septin 12	230947_at	1	2.514	0.36	7	2.318	0.2	40	d	0.24044
TCP1	1	t-complex 1	222010_at	1	7.749	0.27	7	8.347	0.86	40	u	0.002103
			222011_s_at	1	6.613	0.51	7	7.357	0.87	40	u	0.037165
			208778_s_at	0	10.59	0.17	7	10.61	0.69	40	u	0.905712
PSD2	1	pleckstrin and Sec7 domain containing 2	223536_at	1	2.698	0.17	7	2.652	0.31	40	d	0.711864
PAWR	1	PRKC, apoptosis, WT1, regulator	204004_at	1	10.12	0.4	7	9.092	1.06	40	d	0.017403
			204005_s_at	1	7.37	0.52	7	7.855	0.96	40	u	0.210943
			214237_x_at	0	4.592	0.29	7	4.248	0.61	40	d	0.160369
			229515_at	0	7.039	0.58	7	6.057	0.93	40	d	0.011535
			214090_at	0	2.408	0.1	7	2.477	0.1	40	u	0.119749
DLGAP1	1	discs, large (Drosophila) homolog-associated protein 1	210750_s_at	1	2.833	0.14	7	2.915	0.19	40	u	0.299533
			206489_s_at	0	2.273	0.14	7	2.503	0.54	40	u	0.032006
			206490_at	0	3.652	0.27	7	3.951	0.57	40	u	0.192339
KLHL28	1	kelch-like 28 (Drosophila)	235727_at	1	8.259	0.34	7	7.661	0.71	40	d	0.036586
			228328_at	0	6.703	0.48	7	6.358	0.76	40	d	0.258563
			220374_at	0	3.624	0.28	7	3.77	0.33	40	u	0.278698
			243982_at	0	3.684	0.44	7	3.759	0.72	40	u	0.795495
RCAN1	1	regulator of calcineurin 1	208370_s_at	1	11.12	0.53	7	9.024	1.54	40	d	0.000001
			215253_s_at	0	8.076	0.89	7	6.281	1.65	40	d	0.008489
			215254_at	0	2.094	0.18	7	2.062	0.25	40	d	0.751981
DNAJB4	1	DnaJ (Hsp40) homolog, subfamily B, member 4	203810_at	1	8.614	0.24	7	7.788	0.92	40	d	0.000038
			203811_s_at	0	6.753	0.34	7	6.305	1.23	40	d	0.072269
NAB2	1	NGFI-A binding protein 2 (EGR1 binding protein 2)	212803_at	1	7.35	0.25	7	6.81	0.72	40	d	0.001874
			216017_s_at	0	5.897	0.46	7	5.709	0.85	40	d	0.577223
TYRP1	1	tyrosinase-related protein 1	205694_at	1	2.104	0.02	7	2.778	1.22	40	u	0.001426
RBM7	1	RNA binding motif protein 7	218379_at	1	8.148	0.42	7	7.911	0.87	40	d	0.493964
			235045_at	0	4.866	0.45	7	4.725	0.72	40	d	0.625664
DPYSL4	1	dihydropyrimidinase-like 4	205492_s_at	1	2.572	0.21	7	2.786	0.84	40	u	0.190057
			205493_s_at	1	2.63	0.28	7	2.911	1.3	40	u	0.246314
			214301_s_at	1	2.862	0.39	7	2.639	0.39	40	d	0.175258
NKX2-8	1	NK2 homeobox 8	207451_at	1	2.847	0.55	7	2.603	0.27	40	d	0.326377
PTHR1	1	parathyroid hormone receptor 1	205911_at	1	3.617	0.52	7	3.09	0.44	40	d	0.007848
TNFRSF1A	1	tumor necrosis factor receptor superfamily, member 1A	207643_s_at	1	9.922	0.2	7	9.324	0.8	40	d	0.000321
ACADVL	1	acyl-Coenzyme A dehydrogenase, very long chain	200710_at	1	10.74	0.35	7	8.915	0.74	40	d	0
TLX1	1	T-cell leukemia homeobox 1	207179_at	1	2.61	0.25	7	2.775	0.82	40	u	0.331525
DOPEY2	1	dopey family member 2	215219_at	0	2.856	0.16	7	3.018	0.29	40	u	0.162177
			205248_at	0	4.391	0.69	7	4.987	1.11	40	u	0.184685
OSM	1	oncostatin M	230170_at	1	4.63	0.8	7	4.776	0.85	40	u	0.681294
			214637_at	0	3.614	0.42	7	3.572	0.51	40	d	0.841139
EIF5A	1	eukaryotic translation initiation factor 5A	213753_x_at	1	8.478	0.54	7	7.886	0.76	40	d	0.059516
			213757_at	1	8.676	0.63	7	9.413	0.98	40	u	0.065569
			201122_x_at	0	8.412	0.67	7	7.575	0.85	40	d	0.02026
			201123_s_at	0	9.381	2.18	7	6.879	2.65	40	d	0.025451
PPFIBP1	1	PTPRF interacting protein, binding protein 1 (liprin beta 1)	203736_s_at	1	5.345	0.8	7	4.726	0.64	40	d	0.031568
			203735_x_at	1	7.416	0.27	7	7.166	0.83	40	d	0.157465
			214375_at	0	7.268	0.46	7	6.493	1.51	40	d	0.01745
			1569464_at	0	2.857	0.29	7	3.049	0.43	40	u	0.275351
			214374_s_at	0	7.185	0.32	7	6.77	1.01	40	d	0.055468
EHD2	1	EH-domain containing 2	221870_at	1	8.866	0.41	7	6.379	1.22	40	d	0
			45297_at	1	8.335	0.32	7	5.937	0.99	40	d	0
			205341_at	0	6.751	0.38	7	5.563	0.71	40	d	0.000117
BCL7A	1	B-cell CLL/lymphoma 7A	203795_s_at	1	6.457	0.51	7	6.209	0.81	40	d	0.450764

BCL7A	1	B-cell CLL/lymphoma 7A	203796_s_at	1	6.623	0.31	7	6.89	0.77	40	u	0.380561
FGF12	1	fibroblast growth factor 12	214589_at	1	3.138	0.17	7	3.309	0.22	40	u	0.05785
			207501_s_at	0	2.702	0.52	7	2.836	0.71	40	u	0.644026
SLMO2	1	slowmo homolog 2 (Drosophila)	222441_x_at	1	7.745	0.56	7	8.866	0.87	40	u	0.002283
			217851_s_at	1	7.333	0.38	7	8.489	0.8	40	u	0.00069
GABRE	1	gamma-aminobutyric acid (GABA) A receptor, epsilon	204537_s_at	1	5.96	1.14	7	3.985	1.82	40	d	0.009177
GRPEL2	1	GrpE-like 2, mitochondrial (E. coli)	226881_at	1	7.709	0.21	7	8.274	0.52	40	u	0.007915
			238427_at	0	3.144	0.27	7	3.866	0.91	40	u	0.000444
ELF4	1	E74-like factor 4 (ets domain transcription factor)	31845_at	1	5.71	0.34	7	6.685	0.84	40	u	0.004887
			203490_at	1	5.077	0.2	7	5.785	0.64	40	u	0.000009
ZNF524	1	zinc finger protein 524	238683_at	1	6.275	0.14	7	5.786	0.53	40	d	0.000033
CD83	1	CD83 molecule	204440_at	1	6.911	0.48	7	7.545	0.77	40	u	0.043833
GALNT9	1	UDP-N-acetyl-alpha-D-galactosamine:polypeptide N-acetylgalactosaminyltransferase 9 (GalNAc-T9)	229451_at	1	4.273	0.47	7	3.896	0.49	40	d	0.071055
SAFB2	1	scaffold attachment factor B2	203280_at	1	6.887	0.21	7	6.229	0.48	40	d	0.001021
			32099_at	1	8.623	0.36	7	7.515	0.83	40	d	0.001362
NPY	1	neuropeptide Y	206001_at	0	2.378	0.23	7	3.01	2	40	u	0.064957
TMEM77	1	transmembrane protein 77	225228_at	1	7.498	0.09	7	7.327	1.02	40	d	0.315527
			225230_at	1	9.03	0.25	7	8.846	0.99	40	d	0.333619
GK5	1	glycerol kinase 5 (putative)	241384_x_at	1	3.344	0.28	7	3.526	0.53	40	u	0.394391
			238121_at	0	4.93	0.48	7	5.032	0.73	40	u	0.730274
			1553772_at	0	2.98	0.19	7	3.212	0.31	40	u	0.067734
E2F4	1	E2F transcription factor 4, p107/p130-binding	38707_r_at	1	6.851	0.14	7	7.078	0.31	40	u	0.065173
			202248_at	1	2.585	0.26	7	2.693	0.36	40	u	0.464022
TAC1	1	tachykinin, precursor 1 (substance K, substance P, neurokinin 1, neurokinin 2, neuromedin L, neurokinin alpha, neuropeptide K, neuropeptide gamma)	206552_s_at	1	5.459	1.27	7	3	0.69	40	d	0.003582
TRPC1	1	transient receptor potential cation channel, subfamily C, member 1	205803_s_at	1	5.902	0.56	7	4.473	1.01	40	d	0.000859
			211602_s_at	1	3.861	0.79	7	3.072	0.37	40	d	0.052766
			205802_at	1	5.68	0.58	7	4.228	0.7	40	d	0.000007
SOST	1	sclerosteosis	223869_at	1	2.015	0.12	7	2.054	0.12	40	u	0.435123
TRAIP	1	TRAF interacting protein	205598_at	0	2.195	0.07	7	2.337	0.38	40	u	0.037357
FKBP5	1	FK506 binding protein 5	224856_at	1	9.283	1.45	7	7.545	1.63	40	d	0.01297
			224840_at	1	10.43	1.34	7	8.958	1.23	40	d	0.007298
			204560_at	1	6.537	2	7	5.274	1.55	40	d	0.070097
RRAGC	1	Ras-related GTP binding C	222514_at	1	8.605	0.32	7	8.907	0.63	40	u	0.230559
			218088_s_at	1	8.552	0.24	7	9.001	0.71	40	u	0.005811
			242531_at	0	3.732	0.48	7	4.028	0.69	40	u	0.291719
NUDT22	1	nudix (nucleoside diphosphate linked moiety X)-type motif 22	224464_s_at	1	7.469	0.21	7	7.354	0.73	40	d	0.437245
			1558848_at	1	2.999	0.15	7	3.037	0.25	40	u	0.707807
WIF1	1	WNT inhibitory factor 1	204712_at	1	9.519	1	7	4.916	2.15	40	d	0.000002
TMOD2	1	tropomodulin 2 (neuronal)	219701_at	1	2.625	0.1	7	2.875	0.41	40	u	0.002742
VPRBP	1	Vpr (HIV-1) binding protein	226476_s_at	2	6.314	0.32	7	6.143	0.6	40	d	0.471473
			226481_at	2	8.046	0.21	7	8.045	0.6	40	d	0.992056
			226477_at	2	5.339	0.1	7	5.631	0.69	40	u	0.018059
			204377_s_at	1	4.531	0.47	7	3.915	0.27	40	d	0.020253
			204376_at	1	3.48	1.01	7	2.628	0.56	40	d	0.089133
HERC3	1	hect domain and RLD 3	206183_s_at	1	4.441	0.57	7	3.693	0.42	40	d	0.000216
			1554290_at	0	3.501	0.43	7	3.53	0.54	40	u	0.895276
SDK2	1	sidekick homolog 2 (chicken)	220628_s_at	1	5.722	0.31	7	5.726	0.36	40	u	0.981221
			242064_at	1	5.279	1.07	7	3.822	1.16	40	d	0.003929
			232061_at	1	6.114	0.34	7	6.128	0.45	40	u	0.935976
			233585_at	0	4.592	0.21	7	4.803	0.41	40	u	0.202255
RABGGTB	1	Rab geranylgeranyltransferase, beta subunit	209181_s_at	1	10.3	0.22	7	10.5	0.56	40	u	0.35504
			209180_at	1	8.502	0.23	7	8.529	0.64	40	u	0.849005
			213704_at	1	6.726	0.18	7	6.833	0.71	40	u	0.434776
ANKRD40	1	ankyrin repeat domain 40	211717_at	1	5.466	0.25	7	6.257	0.57	40	u	0.000965
			241032_at	0	3.238	0.15	7	3.206	0.18	40	d	0.666549
			227064_at	0	7.769	0.19	7	7.455	0.71	40	d	0.028317
FBXL5	1	F-box and leucine-rich repeat protein 5	209005_at	1	7.642	0.34	7	6.834	0.83	40	d	0.016341

FBXL5	1	F-box and leucine-rich repeat protein 5	209004_s_at	1	9.755	0.19	7	9.108	0.86	40	d	0.000205
NPNT	1	nephronectin	225911_at	1	8.172	0.76	7	8.677	1.68	40	u	0.447711
			244747_at	0	4.151	0.41	7	4.437	1.05	40	u	0.490767
CD72	1	CD72 molecule	215925_s_at	0	4.179	0.46	7	4.854	1.02	40	u	0.099703
FAM96A	1	family with sequence similarity 96, member A	224779_s_at	1	9.119	0.29	7	9.994	0.63	40	u	0.000925
SETBP1	1	SET binding protein 1	205933_at	1	8.224	0.47	7	5.61	1.59	40	d	0
DMBX1	1	diencephalon/mesencephalon homeobox 1	234776_at	0	2.831	0.21	7	2.957	0.32	40	u	0.327683
NRSN1	1	neurensin 1	239293_at	1	3.287	0.26	7	3.378	0.43	40	u	0.594562
AP2S1	1	adaptor-related protein complex 2, sigma 1 subunit	202120_x_at	1	9.957	0.21	7	9.984	0.68	40	u	0.846934
			211047_x_at	1	10.82	0.26	7	10.73	0.71	40	d	0.741615
			208074_s_at	1	9.712	0.28	7	9.712	0.83	40	u	0.999022
KRT34	1	keratin 34	206969_at	1	1.926	0.03	7	1.976	0.05	40	u	0.014914
TOMM22	1	translocase of outer mitochondrial membrane 22 homolog (yeast)	229076_s_at	1	6.142	0.27	7	6.264	0.52	40	u	0.553769
			222474_s_at	1	8.793	0.2	7	8.543	0.67	40	d	0.074544
			217960_s_at	0	8.531	0.22	7	8.169	0.75	40	d	0.020227
MALL	1	mal, T-cell differentiation protein-like	209373_at	1	7.29	0.61	7	6.811	1.5	40	d	0.418497
TMEM132A	1	transmembrane protein 132A	218834_s_at	1	7.166	0.21	7	8.093	1	40	u	0.000007
			222758_s_at	1	3.756	0.39	7	4.946	1.35	40	u	0.000098
			229486_s_at	0	3.972	0.44	7	3.965	0.46	40	d	0.971827
			232419_at	0	3.805	0.23	7	4.034	0.56	40	u	0.306432
ACPL2	1	acid phosphatase-like 2	226925_at	1	7.97	0.38	7	7.709	0.76	40	d	0.390624
			239475_at	0	2.824	0.04	7	2.866	0.25	40	u	0.357299
SPNS1	1	spinster homolog 1 (Drosophila)	223173_at	1	6.28	0.29	7	6.674	0.8	40	u	0.033923
EPN1	1	epsin 1	221141_x_at	1	6.92	0.27	7	6.621	0.42	40	d	0.083821
			226667_x_at	1	7.537	0.13	7	7.347	0.57	40	d	0.08079
NKX2-1	1	NK2 homeobox 1	211024_s_at	1	2.895	0.12	7	3.04	0.27	40	u	0.181117
			231315_at	1	2.445	0.06	7	2.533	0.11	40	u	0.046057
			210673_x_at	0	2.143	0.04	7	2.204	0.09	40	u	0.102476
COCH	1	coagulation factor C homolog, cochlin (Limulus polyphemus)	1554242_a_at	4	2.842	0.43	7	4.027	1.88	40	u	0.001621
			1554241_at	4	8.94	0.27	7	8.659	0.84	40	d	0.11513
			205229_s_at	1	3.723	1.19	7	4.954	2.44	40	u	0.207322
C1orf32	1	chromosome 1 open reading frame 32	no probeset available									
RDH11	1	retinol dehydrogenase 11 (all-trans/9-cis/11-cis)	217775_s_at	1	7.078	0.28	7	7.691	0.48	40	u	0.002324
			217776_at	1	7.962	0.29	7	8.398	0.55	40	u	0.050895
			217042_at	0	3.152	0.52	7	3.241	0.56	40	u	0.704279
			232359_at	0	3.482	0.05	7	3.513	0.19	40	u	0.399968
SP4	1	Sp4 transcription factor	206663_at	1	2.173	0.18	7	2.317	0.33	40	u	0.267836
			236265_at	0	7.94	0.48	7	7.654	0.63	40	d	0.265825
SLC25A39	1	solute carrier family 25, member 39	223649_s_at	0	8.679	0.19	7	9.361	0.7	40	u	0.000017
RALB	1	v-ral simian leukemia viral oncogene homolog B (ras related; GTP binding protein)	202100_at	1	8.765	0.19	7	8.301	0.55	40	d	0.000557
			202101_s_at	0	7.55	0.39	7	7.626	0.72	40	u	0.789381
MBD2	1	methyl-CpG binding domain protein 2	202485_s_at	0	2.337	0.14	7	2.417	0.23	40	u	0.384271
			202484_s_at	0	9.189	0.21	7	8.934	0.59	40	d	0.058122
			214396_s_at	0	1.974	0.2	7	2.254	0.75	40	u	0.060714
			214397_at	0	2.203	0.12	7	2.571	0.88	40	u	0.017304
ALS2CR4	1	amyotrophic lateral sclerosis 2 (juvenile) chromosome region, candidate 4	1553956_at	1	7.012	0.61	7	6.925	0.63	40	d	0.741966
			228255_at	0	8.164	0.7	7	5.534	1.01	40	d	0
PSRC1	1	proline/serine-rich coiled-coil 1	201896_s_at	1	4.755	0.77	7	6.498	1.08	40	u	0.000232
GCLC	1	glutamate-cysteine ligase, catalytic subunit	202923_s_at	1	7.94	0.4	7	7.993	0.81	40	u	0.868247
			202922_at	1	7.69	0.33	7	7.269	0.67	40	d	0.11571
			1555330_at	0	3.052	0.42	7	2.89	0.49	40	d	0.419765
ZC3H14	1	zinc finger CCCH-type containing 14	213063_at	1	7.176	0.31	7	6.864	0.45	40	d	0.091701
			213064_at	1	7.854	0.27	7	7.234	0.6	40	d	0.011209
			204216_s_at	0	8.028	0.16	7	7.643	0.5	40	d	0.000896
ALDH5A1	1	aldehyde dehydrogenase 5 family, member A1 (succinate-semialdehyde dehydrogenase)	203609_s_at	1	2.129	0.12	7	2.29	0.32	40	u	0.206031
			203608_at	1	6.873	0.43	7	6.463	1.08	40	d	0.337763
TWSG1	1	twisted gastrulation homolog 1 (Drosophila)	219201_s_at	1	6.242	0.26	7	6.464	0.92	40	u	0.229869
			225406_at	1	8.791	0.27	7	8.689	0.72	40	d	0.715575
UPF1	1	UPF1 regulator of nonsense transcripts homolog (yeast)	211168_s_at	1	8.229	0.61	7	7.446	0.99	40	d	0.054178
			208947_s_at	0	5.363	0.59	7	5.356	1.05	40	d	0.986453
ZSWIM4	1	zinc finger, SWIM-type containing 4	231915_at	1	3.837	0.38	7	3.614	0.53	40	d	0.301155
CDC40	1	cell division cycle 40 homolog (S. cerevisiae)	203376_at	1	8.518	0.21	7	8.468	0.73	40	d	0.733375
			203377_s_at	0	4.162	0.47	7	5.106	1.13	40	u	0.038023

PQLC3	1	PQ loop repeat containing 3	225579_at	1	9.374	0.28	7	9.006	1.01	40	d	0.071861
			216458_at	0	5.207	0.2	7	5.289	0.33	40	u	0.533227
BANF1	1	barrier to autointegration factor 1	210125_s_at	1	10.73	0.22	7	10.85	0.62	40	u	0.377559
RPS6KA2	1	ribosomal protein S6 kinase, 90kDa, polypeptide 2	212912_at	1	7.762	0.21	7	6.056	1.09	40	d	0
			204906_at	1	8.055	0.27	7	7.135	0.66	40	d	0.000859
			1557970_s_at	1	6.267	0.34	7	6.123	0.55	40	d	0.517128
HMBS	1	hydroxymethylbilane synthase	203040_s_at	1	7.312	0.24	7	7.724	0.64	40	u	0.107854
SLC25A24	1	solute carrier family 25 (mitochondrial carrier; phosphate carrier), member 24	204342_at	1	8.6	0.69	7	9.048	0.62	40	u	0.095127
			1569121_at	0	2.299	0.13	7	2.394	0.18	40	u	0.201478
			244481_at	0	4.472	0.34	7	4.401	0.57	40	d	0.756023
KLHDC1	1	kelch domain containing 1	1552733_at	1	6.423	0.83	7	5.365	0.87	40	d	0.005358
			1564273_at	0	2.463	0.05	7	2.569	0.12	40	u	0.025053
			1564272_a_at	0	3.231	0.19	7	3.404	0.26	40	u	0.108035
ZNF287	1	zinc finger protein 287	220055_at	1	2.876	0.24	7	2.917	0.42	40	u	0.805238
			216453_at	0	2.189	0.02	7	2.285	0.15	40	u	0.000715
			216710_x_at	0	3.432	0.16	7	3.924	0.48	40	u	0.000039
WIBG	1	within bgcn homolog (Drosophila)	224465_s_at	1	8.008	0.24	7	7.622	0.66	40	d	0.013553
			1569428_at	0	2.724	0.18	7	2.847	0.31	40	u	0.327811
			242304_at	0	6.375	0.6	7	5.962	0.75	40	d	0.180525
FMNL1	1	formin-like 1	204789_at	1	5.953	0.43	7	5.723	0.58	40	d	0.328655
			1569257_at	0	3.779	0.24	7	3.834	0.29	40	u	0.649386
GRAMD1C	1	GRAM domain containing 1C	219313_at	1	6.723	0.44	7	4.996	1.23	40	d	0.000001
USP38	1	ubiquitin specific peptidase 38	223288_at	1	7.267	0.43	7	8.206	0.82	40	u	0.005653
			223289_s_at	0	7.02	0.31	7	7.568	1.18	40	u	0.021436
ARMCX3	1	armadillo repeat containing, X-linked 3	222444_at	1	9.068	0.5	7	8.458	0.86	40	d	0.079473
			217858_s_at	0	9.752	0.45	7	9.452	0.83	40	d	0.36437
NDE1	1	nudE nuclear distribution gene E homolog 1 (A. nidulans)	222625_s_at	1	5.27	0.23	7	5.683	0.67	40	u	0.007544
			218414_s_at	1	7.186	0.45	7	7.575	0.8	40	u	0.224285
			227249_at	0	5.974	0.33	7	7.321	0.99	40	u	0.000001
			230219_at	0	4.272	0.27	7	4.679	0.7	40	u	0.144434
			227843_at	0	1.884	0.03	7	2.021	0.35	40	u	0.01979
MTF2	1	metal response element binding transcription factor 2	203345_s_at	1	7.969	0.27	7	8.137	0.82	40	u	0.339536
			203347_s_at	1	6.625	0.47	7	7.319	0.76	40	u	0.026847
			209705_at	1	7.317	0.23	7	7.084	0.6	40	d	0.323952
			203346_s_at	0	7.848	0.32	7	8.014	0.75	40	u	0.572946
ATP6V1F	1	ATPase, H+ transporting, lysosomal 14kDa, V1 subunit F	201527_at	1	10.52	0.17	7	11.01	0.58	40	u	0.000167
DUS2L	1	dihydrouridine synthase 2-like, SMM1 homolog (S. cerevisiae)	47105_at	1	4.967	0.15	7	4.861	0.31	40	d	0.385369
			219486_at	1	6.261	0.28	7	6.533	0.39	40	u	0.08779
LMO7	1	LIM domain 7	202674_s_at	1	2.616	0.15	7	2.78	0.33	40	u	0.212973
			1556377_s_at	0	4.041	0.4	7	4.035	0.49	40	d	0.979249
			241140_at	0	2.731	0.15	7	2.932	0.55	40	u	0.067806
			242722_at	0	5.987	0.41	7	6.566	0.98	40	u	0.140706
C10orf97	1	chromosome 10 open reading frame 97	1554509_a_at	1	5.914	0.34	7	5.727	0.9	40	d	0.599168
			218297_at	1	7.617	0.35	7	7.623	0.69	40	u	0.982206
SLC23A1	1	solute carrier family 23 (nucleobase transporters), member 1	223732_at	0	2.414	0.13	7	2.733	0.72	40	u	0.015771
RBM4B	1	RNA binding motif protein 4B	209497_s_at	0	7.553	0.32	7	7.005	0.7	40	d	0.051276
C12orf49	1	chromosome 12 open reading frame 49	218867_s_at	1	5.762	0.25	7	6.364	0.74	40	u	0.000663
			222767_s_at	1	6.268	0.42	7	6.891	0.71	40	u	0.032404
CCDC49	1	coiled-coil domain containing 49	222706_at	1	6.793	0.37	7	5.913	1.13	40	d	0.000833
			218655_s_at	1	6.629	0.33	7	6.66	0.95	40	u	0.879926
STARD8	1	StAR-related lipid transfer (START) domain containing 8	206868_at	1	5.142	0.37	7	4.578	0.39	40	d	0.001035
ABHD5	1	abhydrolase domain containing 5	218739_at	1	6.633	0.41	7	6.682	0.98	40	u	0.898532
			213935_at	0	6.804	0.52	7	6.403	0.49	40	d	0.060659
			213805_at	0	5.138	0.5	7	5.096	0.91	40	d	0.905915
			232862_at	0	2.595	0.42	7	2.493	0.15	40	d	0.580045
EXOC4	1	exocyst complex component 4	224926_at	1	8.266	0.24	7	8.325	0.58	40	u	0.793768
			240528_s_at	0	6.214	0.7	7	6.185	1.25	40	d	0.952355
TMBIM1	1	transmembrane BAX inhibitor motif containing 1	217730_at	1	9.816	0.25	7	9.335	0.52	40	d	0.023248
PSCD2	1	pleckstrin homology, Sec7 and coiled-coil domains 2 (cytohesin-2)	209158_s_at	1	8.374	0.28	7	7.784	0.59	40	d	0.014291
			237234_at	0	4.676	0.44	7	4.456	0.69	40	d	0.430013

TCHP	1	trichoplein, keratin filament binding	223455_at	1	7.113	0.35	7	6.68	0.81	40	d	0.179515
			223456_s_at	0	4.444	0.36	7	4.36	0.38	40	d	0.600746
F2RL2	1	coagulation factor II (thrombin) receptor-like 2	230147_at	1	6.608	0.69	7	5.401	1.47	40	d	0.042353
			206795_at	0	3.935	0.27	7	3.962	0.31	40	u	0.837879
EMB	1	embigin homolog (mouse)	no probeset available									
TRAF6	1	TNF receptor-associated factor 6	205558_at	0	6.124	0.24	7	5.963	0.54	40	d	0.448988
AXL	1	AXL receptor tyrosine kinase	202686_s_at	1	8.193	0.41	7	7.045	0.9	40	d	0.002188
			202685_s_at	1	3.422	0.7	7	3.279	0.66	40	d	0.609506
CD276	1	CD276 molecule	224859_at	1	7.673	0.32	7	7.952	0.75	40	u	0.347343
			1552914_a_at	0	3.98	0.69	7	4.539	1.36	40	u	0.302581
			1559583_at	0	2.562	0.29	7	2.774	0.45	40	u	0.243909
CSNK1E	1	casein kinase 1, epsilon	225756_at	0	8.2	0.51	7	7.701	0.59	40	d	0.044443
			202332_at	0	8.52	0.31	7	7.852	0.6	40	d	0.007241
			222015_at	0	6.856	0.14	7	6.789	0.42	40	d	0.448207
			226858_at	0	6.749	0.37	7	6.595	0.85	40	d	0.64732
KRT72	1	keratin 72	1564435_a_at	1	2.31	0.03	7	2.369	0.06	40	u	0.014674
RAGE	1	renal tumor antigen	205130_at	1	7.021	0.35	7	6.51	1.17	40	d	0.038756
OGN	1	osteoglycin	222722_at	1	10.33	0.67	7	4.933	1.89	40	d	0
			218730_s_at	1	8.339	0.73	7	3.741	1.12	40	d	0
XPO6	1	exportin 6	214784_x_at	1	7.358	0.22	7	8.025	0.51	40	u	0.0016
			211982_x_at	1	7.976	0.24	7	8.478	0.61	40	u	0.042463
PIK3C2B	1	phosphoinositide-3-kinase, class 2, beta polypeptide	204484_at	1	7.784	0.33	7	7.298	0.89	40	d	0.170883
TUBB2B	1	tubulin, beta 2B	214023_x_at	1	6.097	0.79	7	6.187	1.95	40	u	0.906892
CEND1	1	cell cycle exit and neuronal differentiation 1	219591_at	1	4.379	0.58	7	4.02	0.56	40	d	0.13563
ENTPD6	1	ectonucleoside triphosphate diphosphohydrolase 6 (putative function)	234946_at	1	3.226	0.36	7	3.325	0.38	40	u	0.538962
			201704_at	1	8.601	0.21	7	8.065	1.07	40	d	0.007587
RAD23A	1	RAD23 homolog A (S. cerevisiae)	201039_s_at	1	7.952	0.32	7	8.774	0.85	40	u	0.01708
			201046_s_at	1	8.675	0.22	7	9.013	0.79	40	u	0.037793
CTNNA1	1	catenin (cadherin-associated protein), alpha 1, 102kDa	215883_at	1	4.514	1	7	3.865	0.76	40	d	0.05895
			200765_x_at	1	10.85	0.25	7	10.41	0.77	40	d	0.011634
			200764_s_at	1	9.901	0.27	7	9.552	0.73	40	d	0.226574
			210844_x_at	0	11.31	0.25	7	10.87	0.72	40	d	0.008287
			1558214_s_at	0	6.416	0.74	7	6.659	1.83	40	u	0.736131
			217366_at	0	3.297	0.09	7	3.435	0.32	40	u	0.038791
IRAK2	1	interleukin-1 receptor-associated kinase 2	231779_at	1	4.684	0.28	7	4.902	0.92	40	u	0.255072
			1553740_a_at	0	4.321	0.4	7	4.598	0.47	40	u	0.156191
			1553739_at	0	2.482	0.04	7	2.584	0.13	40	u	0.000337
GHITM	1	growth hormone inducible transmembrane protein	209248_at	1	10.2	0.16	7	10.19	0.51	40	d	0.962779
			209249_s_at	0	10.81	0.23	7	10.84	0.52	40	u	0.889819
			1554510_s_at	0	9.365	0.28	7	9.338	0.71	40	d	0.923606
CCDC3	1	coiled-coil domain containing 3	223316_at	1	7.839	1.13	7	5.718	1.08	40	d	0.000028
NTRK2	1	neurotrophic tyrosine kinase, receptor, type 2	236095_at	1	3.518	0.59	7	3.029	0.69	40	d	0.091861
			207152_at	0	2.75	0.27	7	2.766	0.38	40	u	0.921469
			214680_at	0	7.637	0.84	7	3.432	1.93	40	d	0.000001
			221796_at	0	11.28	0.64	7	5.265	2.43	40	d	0
			221795_at	0	10.54	0.67	7	5.881	2.1	40	d	0
			229463_at	0	3.873	0.63	7	3.519	0.83	40	d	0.298701
C17orf49	1	chromosome 17 open reading frame 49	224574_at	1	9.269	0.39	7	9.108	0.77	40	d	0.599156
DHH	1	desert hedgehog homolog (Drosophila)	1552730_at	1	2.357	0.05	7	2.468	0.23	40	u	0.012785
RNF167	1	ring finger protein 167	212047_s_at	1	8.887	0.12	7	8.477	0.56	40	d	0.000231
ETV3	1	ets variant gene 3	1552423_at	1	4.721	0.36	7	5.288	0.72	40	u	0.051483
			214480_at	0	2.621	0.11	7	2.617	0.18	40	d	0.949414
KCNS2	1	potassium voltage-gated channel, delayed-rectifier, subfamily S, member 2	232401_at	1	2.364	0.1	7	2.385	0.1	40	u	0.627212
			1554680_s_at	1	2.387	0.05	7	2.45	0.16	40	u	0.065984
			1554212_s_at	1	5.136	0.17	7	5.493	0.5	40	u	0.002663
APBB3	1	amyloid beta (A4) precursor protein-binding, family B, member 3	204650_s_at	1	7.132	0.24	7	5.742	0.81	40	d	0
RNF126	1	ring finger protein 126	205748_s_at	1	7.181	0.43	7	7.273	0.84	40	u	0.782003
			215031_x_at	1	6.47	0.41	7	6.248	0.67	40	d	0.409037
			223332_x_at	1	6.786	0.34	7	6.834	0.68	40	u	0.856817
WDR92	1	WD repeat domain 92	235071_at	0	6.597	0.3	7	6.765	0.5	40	u	0.40494
			206655_s_at	1	4.542	0.74	7	4.329	1.13	40	d	0.639523

GP1BB	1	glycoprotein Ib (platelet), beta polypeptide	209768_s_at	1	4.569	0.4	7	4.3	0.51	40	d	0.200074
			209767_s_at	1	7.647	0.36	7	7.562	0.56	40	d	0.704425
			209769_s_at	1	5.392	0.28	7	4.969	0.58	40	d	0.06953
SAMHD1	1	SAM domain and HD domain 1	204502_at	1	6.852	0.47	7	7.477	1.08	40	u	0.148133
			1559883_s_at	0	3.773	0.82	7	4.669	1.84	40	u	0.222222
			1559882_at	0	3.142	0.68	7	3.15	0.58	40	u	0.976345
ATP13A2	1	ATPase type 13A2	218608_at	1	7.378	0.25	7	6.862	0.8	40	d	0.003871
CAPNS1	1	calpain, small subunit 1	200001_at	1	11.92	0.17	7	11.32	0.8	40	d	0.000174
LHX4	1	LIM homeobox 4	1553157_at	1	2.431	0.09	7	2.608	0.33	40	u	0.010987
			1553156_at	1	2.204	0.08	7	2.222	0.07	40	u	0.529163
SLC25A4	1	solute carrier family 25 (mitochondrial carrier; adenine nucleotide translocator), member 4	202825_at	1	8.386	0.27	7	7.894	0.83	40	d	0.008471
			1557631_at	0	3.084	0.17	7	3.245	0.34	40	u	0.234765
CARD10	1	caspase recruitment domain family, member 10	210026_s_at	1	7.053	0.67	7	5.491	1.21	40	d	0.002077
			210025_s_at	1	5.505	0.46	7	4.996	0.6	40	d	0.040874
			214207_s_at	1	2.549	0.57	7	2.517	0.35	40	d	0.846548
SOD1	1	superoxide dismutase 1, soluble (amyotrophic lateral sclerosis 1 (adult))	200642_at	0	12.36	0.22	7	12.35	0.56	40	d	0.960886
BCR	1	breakpoint cluster region	202315_s_at	1	7.327	0.33	7	7.235	0.87	40	d	0.788566
			217223_s_at	0	6.219	0.38	7	6.017	0.75	40	d	0.502363
			226602_s_at	0	7.429	0.24	7	6.769	0.93	40	d	0.000736
RNF146	1	ring finger protein 146	223886_s_at	1	9.468	0.17	7	9.135	0.54	40	d	0.006269
			221430_s_at	0	7.505	0.34	7	7.139	1.16	40	d	0.123932
TIAM2	1	T-cell lymphoma invasion and metastasis 2	222942_s_at	1	3.527	0.78	7	3.467	1.21	40	d	0.901147
			219950_s_at	1	2.225	0.18	7	2.378	0.42	40	u	0.362448
UBE1C	1	ubiquitin-activating enzyme E1C (UBA3 homolog, yeast)	209115_at	1	9.302	0.26	7	9.421	0.57	40	u	0.595167
FZD5	1	frizzled homolog 5 (Drosophila)	221245_s_at	13	4.74	0.41	7	4.512	0.81	40	d	0.479903
			206136_at	1	3.438	0.64	7	3.343	0.51	40	d	0.672266
RPL15	1	ribosomal protein L15	221475_s_at	1	13.14	0.1	7	12.45	0.48	40	d	0
			221476_s_at	1	13.43	0.13	7	12.23	0.66	40	d	0
			240806_at	0	4.605	0.9	7	3.791	0.59	40	d	0.004168
			217266_at	0	7.217	0.22	7	7.088	0.72	40	d	0.391666
PRC1	1	protein regulator of cytokinesis 1	218009_s_at	1	5.316	0.86	7	9.217	0.98	40	u	0
TMPRSS3	1	transmembrane protease, serine 3	220177_s_at	1	6.526	0.7	7	6.215	2.59	40	d	0.541614
			223949_at	0	5.375	1.15	7	5.965	1.75	40	u	0.403689
			223948_s_at	0	6.137	0.53	7	6.337	1.76	40	u	0.580071
C4orf32	1	chromosome 4 open reading frame 32	227856_at	1	8.59	0.41	7	7.102	2.03	40	d	0.000197
			240465_at	0	3.574	0.28	7	3.673	0.47	40	u	0.597868
KCNA3	1	potassium voltage-gated channel, shaker-related subfamily, member 3	207237_at	1	2.829	0.26	7	2.963	0.61	40	u	0.579117
DIABLO	1	diablo homolog (Drosophila)	219350_s_at	1	9.26	0.28	7	9.111	0.5	40	d	0.454459
SEPT2	1	septin 2	200778_s_at	1	10.68	0.36	7	10.67	0.87	40	d	0.972824
			200015_s_at	1	11.56	0.16	7	11.21	0.51	40	d	0.001788
			1559038_at	0	7.542	0.31	7	7.432	0.93	40	d	0.577334
			232637_at	0	4.32	0.22	7	4.583	0.61	40	u	0.058788
			1554747_a_at	0	8.79	0.43	7	8.778	1.09	40	d	0.979135
CHRDL2	1	chordin-like 2	223987_at	1	2.328	0.08	7	2.796	1.31	40	u	0.033487
PALMD	1	palmdelphin	218736_s_at	1	8.818	0.45	7	6.271	1.19	40	d	0.000002
			222725_s_at	1	5.593	0.45	7	4.779	0.85	40	d	0.019056
			232187_at	1	3.384	0.9	7	2.682	0.38	40	d	0.108669
PRPF8	1	PRP8 pre-mRNA processing factor 8 homolog (S. cerevisiae)	200000_s_at	1	9.955	0.52	7	8.984	0.83	40	d	0.005382
RPL28	1	ribosomal protein L28	200003_s_at	1	13.63	0.16	7	13.41	0.39	40	d	0.159033
			213223_at	1	5.271	0.23	7	4.646	1.09	40	d	0.003052
GLIS3	1	GLIS family zinc finger 3	230258_at	1	2.856	0.19	7	3.488	0.55	40	u	0.000015
			229435_at	0	3.456	0.5	7	3.585	0.69	40	u	0.645485
BIN2	1	bridging integrator 2	219191_s_at	1	3.989	1.04	7	4.119	1.06	40	u	0.772519
DBNDD1	1	dysbindin (dystrobrevin binding protein 1) domain containing 1	222234_s_at	1	5.966	0.84	7	6.366	1.07	40	u	0.362042
G6PD	1	glucose-6-phosphate dehydrogenase	202275_at	1	7.942	1.49	7	7.734	1.16	40	d	0.684366
DNAJC5B	1	DnaJ (Hsp40) homolog, subfamily C, member 5 beta	1554258_a_at	1	2.676	0.24	7	2.888	0.75	40	u	0.181375
			232798_at	0	3.684	0.31	7	3.902	0.5	40	u	0.284489
WHDC1	1	WAS protein homology region 2 domain containing 1	228953_at	1	6.708	0.38	7	5.817	0.88	40	d	0.013291
			222367_at	0	2.443	0.05	7	2.512	0.09	40	u	0.057542

BCKDHA	1	branched chain keto acid dehydrogenase E1, alpha polypeptide	202331_at	1	7.899	0.28	7	7.586	0.71	40	d	0.264518
EFCBP1	1	EF-hand calcium binding protein 1	233305_at	0	2.365	0.03	7	2.648	0.6	40	u	0.005383
ABL1	1	v-abl Abelson murine leukemia viral oncogene homolog 1	202123_s_at	1	9.379	0.37	7	8.257	0.74	40	d	0.000384
TNRC15	1	trinucleotide repeat containing 15	212260_at	1	7.867	0.17	7	7.175	0.6	40	d	0.000002
			212261_at	1	8.328	0.27	7	7.941	0.41	40	d	0.02128
			1558305_at	0	2.864	0.16	7	3.374	0.98	40	u	0.004586
			1560133_at	0	4.671	0.6	7	4.58	0.5	40	d	0.675128
SPINK4	1	serine peptidase inhibitor, Kazal type 4	207214_at	1	3.016	0.66	7	2.973	0.56	40	d	0.858726
CCDC136	1	coiled-coil domain containing 136	226972_s_at	1	4.425	0.4	7	3.756	0.71	40	d	0.022486
			232989_s_at	1	3.39	0.32	7	3.345	0.39	40	d	0.780759
			226971_at	1	3.083	0.32	7	2.942	0.5	40	d	0.481514
BARHL1	1	BarH-like homeobox 1	224092_at	1	3.114	0.37	7	2.94	0.3	40	d	0.192859
RAD50	1	RAD50 homolog (S. cerevisiae)	209349_at	1	6.435	0.55	7	5.767	1.21	40	d	0.168192
			208393_s_at	0	7.924	0.22	7	7.919	0.79	40	d	0.970599
CMTM4	1	CKLF-like MARVEL transmembrane domain containing 4	1554677_s_at	1	2.303	0.28	7	2.498	0.58	40	u	0.398696
			225009_at	0	9.134	0.39	7	8.901	0.97	40	d	0.545114
			224998_at	0	8.183	0.39	7	7.991	0.84	40	d	0.561828
MTHFD1L	1	methylenetetrahydrofolate dehydrogenase (NADP+ dependent) 1-like	225520_at	1	5.204	0.66	7	6.87	1.3	40	u	0.002285
			1561125_at	0	2.413	0.11	7	2.546	0.29	40	u	0.243394
			231094_s_at	0	5.267	0.6	7	6.28	0.92	40	u	0.008343
NCL	1	nucleolin	200610_s_at	1	12.22	0.16	7	11.99	0.4	40	d	0.162687
SSR2	1	signal sequence receptor, beta (translocon-associated protein beta)	200652_at	1	11.55	0.15	7	11.58	0.69	40	u	0.828157
IL25	1	interleukin 25	220971_at	1	2.096	0.02	7	2.159	0.1	40	u	0.001223
CRELD1	1	cysteine-rich with EGF-like domains 1	203368_at	1	6.791	0.36	7	6.081	0.97	40	d	0.066348
PRKRIP1	1	PRKR interacting protein 1 (IL11 inducible)	218378_s_at	1	7.278	0.35	7	6.798	0.67	40	d	0.075073
			222615_s_at	1	5.906	0.19	7	5.841	0.39	40	d	0.672976
			1564166_s_at	0	2.957	0.17	7	3.512	0.54	40	u	0.000028
			1564165_at	0	4.866	0.14	7	4.944	0.31	40	u	0.529128
GDF11	1	growth differentiation factor 11	216854_at	0	2.587	0.15	7	2.539	0.15	40	d	0.454023
			216860_s_at	0	2.562	0.23	7	2.922	0.66	40	u	0.017751
MED21	1	mediator complex subunit 21	209363_s_at	1	6.366	0.59	7	6.987	1.14	40	u	0.173361
			209362_at	1	7.754	0.28	7	7.927	0.95	40	u	0.37162
CEP192	1	centrosomal protein 192kDa	218827_s_at	1	5.875	0.19	7	5.876	0.97	40	u	0.990928
WISP1	1	WNT1 inducible signaling pathway protein 1	206796_at	1	2.642	0.34	7	4.137	1	40	u	0
			211312_s_at	0	2.235	0.08	7	2.407	0.36	40	u	0.013026
SLC24A1	1	solute carrier family 24 (sodium/potassium/calcium exchanger), member 1	211842_s_at	0	2.568	0.19	7	2.85	0.39	40	u	0.075141
			210420_at	0	3.991	0.28	7	4.089	0.42	40	u	0.562245
			210421_s_at	0	3.061	0.39	7	2.766	0.27	40	d	0.020086
			206081_at	0	6.56	0.31	7	5.526	0.8	40	d	0.001919
CHMP1A	1	chromatin modifying protein 1A	201933_at	1	7.998	0.34	7	7.99	0.62	40	d	0.974406
TNIP1	1	TNFAIP3 interacting protein 1	207196_s_at	1	8.88	0.21	7	8.529	0.67	40	d	0.016671
STXBP4	1	syntaxin binding protein 4	244404_at	1	3.605	0.33	7	4.205	1.02	40	u	0.008699
			1554329_x_at	0	2.853	0.13	7	3.275	0.71	40	u	0.001549
			237706_at	0	4.216	0.18	7	4.56	0.66	40	u	0.011949
			1554328_at	0	2.864	0.15	7	3.058	0.58	40	u	0.088219
TM9SF2	1	transmembrane 9 superfamily member 2	201078_at	0	10.99	0.23	7	10.89	0.8	40	d	0.522277
MESP1	1	mesoderm posterior 1 homolog (mouse)	224476_s_at	1	5.443	0.5	7	5.01	1.2	40	d	0.362859
			229195_at	1	4.243	0.58	7	4.168	0.67	40	d	0.789748
			243387_at	0	3.324	0.23	7	2.967	0.48	40	d	0.06576
TOX2	1	TOX high mobility group box family member 2	228737_at	1	3.13	0.27	7	3.066	0.57	40	d	0.77861
MED15	1	mediator complex subunit 15	222175_s_at	1	7.547	0.34	7	6.984	0.6	40	d	0.022661
CDK2	1	cyclin-dependent kinase 2	204252_at	1	7.649	0.15	7	7.741	0.73	40	u	0.485784
			211803_at	0	2.508	0.22	7	2.444	0.1	40	d	0.508045
			211804_s_at	0	6.301	0.38	7	6.086	1.24	40	d	0.403999
RHOJ	1	ras homolog gene family, member J	235489_at	1	7.754	0.63	7	4.15	1.01	40	d	0
			238905_at	0	6.164	0.74	7	4.287	0.53	40	d	0
			1555233_at	0	3.98	1	7	2.173	0.44	40	d	0.004857
			1555234_a_at	0	4.531	0.97	7	3.51	0.59	40	d	0.000634
			243481_at	0	4.133	0.7	7	3.145	0.26	40	d	0.014518
			235131_at	0	6.221	0.45	7	5.497	0.59	40	d	0.003999

			238906_s_at	0	5.816	0.74	7	3.862	0.74	40	d	0
VSTM2A	1	V-set and transmembrane domain containing 2A	1554530_at	1	2.016	0.03	7	2.516	1.4	40	u	0.032134
SRPX	1	sushi-repeat-containing protein, X-linked	204955_at	1	10.2	0.59	7	6.962	1.06	40	d	0
ZNF474	1	zinc finger protein 474	1556907_at	1	2.838	0.29	7	2.823	0.19	40	d	0.866
MBNL3	1	muscleblind-like 3 (Drosophila)	219814_at	1	2.326	0.06	7	2.561	0.29	40	u	0.000073
SYNJ2BP	1	synaptojanin 2 binding protein	219156_at	1	8.077	0.33	7	7.597	0.78	40	d	0.1219
			235722_at	0	3.707	0.39	7	4.494	0.89	40	u	0.02918
HIP1R	1	huntingtin interacting protein 1 related	209559_at	1	7.402	0.25	7	6.915	0.32	40	d	0.000511
			38340_at	1	8.386	0.31	7	7.734	0.7	40	d	0.022257
			209558_s_at	1	7.401	0.37	7	6.6	0.92	40	d	0.030987
RSPH10B	1	radial spoke head 10 homolog B (Chlamydomonas)	1555272_at	1	5.265	0.3	7	5.877	0.7	40	u	0.031448
			1557417_s_at	1	2.426	0.06	7	2.488	0.14	40	u	0.256853
MED31	1	mediator complex subunit 31	219318_x_at	1	2.639	0.13	7	2.721	0.23	40	u	0.383992
			222867_s_at	1	7.754	0.26	7	7.361	0.6	40	d	0.102057
			243319_at	0	2.167	0.09	7	2.309	0.23	40	u	0.130365
			236241_at	0	4.301	0.73	7	4.239	0.87	40	d	0.86184
ZNF263	1	zinc finger protein 263	203707_at	1	7.386	0.31	7	7.361	0.64	40	d	0.922464
ATP6AP2	1	ATPase, H+ transporting, lysosomal accessory protein 2	201443_s_at	1	11.18	0.18	7	11.38	0.58	40	u	0.096094
			201442_s_at	1	2.446	0.38	7	2.382	0.16	40	d	0.70177
			201444_s_at	0	10.34	0.23	7	10.4	1.04	40	u	0.766477
ASB14	1	ankyrin repeat and SOCS box-containing 14	1553453_at	0	3.244	0.3	7	3.515	0.55	40	u	0.217491
			237785_at	0	2.351	0.03	7	2.428	0.17	40	u	0.011632
KIF1A	1	kinesin family member 1A	225482_at	1	2.385	0.35	7	3.24	1.3	40	u	0.001808
			203849_s_at	0	3.041	0.25	7	4.209	1.63	40	u	0.000144
			203850_s_at	0	2.754	0.06	7	2.923	0.15	40	u	0.007521
VCAN	1	versican	204619_s_at	1	7.068	0.85	7	8.03	1.31	40	u	0.073077
			204620_s_at	1	9.714	0.75	7	10.49	1.24	40	u	0.1226
			221731_x_at	1	10.17	0.72	7	10.7	1.28	40	u	0.304029
			211571_s_at	0	7.414	0.8	7	7.926	1.76	40	u	0.46309
			215646_s_at	0	7.723	0.66	7	8.418	1.6	40	u	0.273755
C16orf58	1	chromosome 16 open reading frame 58	217891_at	1	8.267	0.17	7	7.86	0.8	40	d	0.008018
			222190_s_at	1	8.109	0.25	7	7.775	0.52	40	d	0.112088
ZDHHC21	1	zinc finger, DHHC-type containing 21	235068_at	1	6.087	0.48	7	6.255	0.77	40	u	0.590567
			241946_at	1	4.294	0.45	7	4.989	1.03	40	u	0.092981
			243550_at	1	3.639	0.42	7	4.078	0.81	40	u	0.175022
			229240_at	0	6.523	0.37	7	6.557	0.78	40	u	0.912271
			233247_x_at	0	3.461	0.28	7	3.543	0.29	40	u	0.499523
			233216_at	0	2.501	0.08	7	2.705	0.3	40	u	0.001131
			233215_s_at	0	2.016	0.1	7	2.096	0.43	40	u	0.325743
			229923_at	0	4.261	0.45	7	3.768	0.73	40	d	0.096804
			243835_at	0	6.163	0.41	7	6.747	1.11	40	u	0.183917
FAM112B	1	family with sequence similarity 112, member B	227711_at	0	2.246	0.23	7	2.775	1.25	40	u	0.020816
SLC1A3	1	solute carrier family 1 (glial high affinity glutamate transporter), member 3	202800_at	1	6.303	0.73	7	6.71	0.95	40	u	0.295568
			1569054_at	0	2.765	0.19	7	2.878	0.38	40	u	0.449798
HAO2	1	hydroxyacid oxidase 2 (long chain)	220801_s_at	1	2.713	0.12	7	2.871	0.24	40	u	0.103565
RPL35A	1	ribosomal protein L35a	225190_x_at	1	13.25	0.18	7	13.1	0.32	40	d	0.250896
			213687_s_at	1	13.95	0.2	7	13.53	0.52	40	d	0.046838
			215208_x_at	0	7.025	0.46	7	7.123	0.46	40	u	0.61491
			238026_at	0	7.084	0.46	7	6.655	0.72	40	d	0.145057
			215249_at	0	3.052	0.41	7	3.853	0.52	40	u	0.000414
RAB40C	1	RAB40C, member RAS oncogene family	227698_s_at	1	6.836	0.44	7	7.002	0.97	40	u	0.666688
			227269_s_at	1	2.722	0.25	7	2.591	0.22	40	d	0.167263
			1569396_at	0	5.641	0.37	7	5.644	0.6	40	u	0.989243
			213466_at	0	3.242	0.69	7	3.102	0.6	40	d	0.590305
KIF3A	1	kinesin family member 3A	213623_at	1	5.364	0.36	7	6.081	0.86	40	u	0.038629
			228680_at	0	6.969	0.3	7	6.569	0.86	40	d	0.040233
CASKIN2	1	CASK interacting protein 2	221846_s_at	3	7.525	0.43	7	6.593	0.86	40	d	0.008864
			61297_at	1	6.911	0.5	7	6.131	0.8	40	d	0.018837
NBL1	1	neuroblastoma, suppression of tumorigenicity 1	37005_at	1	10.44	0.49	7	9.768	1.03	40	d	0.101623
			201621_at	1	9.463	0.53	7	8.613	1.16	40	d	0.069593
SOHLH1	1	spermatogenesis and oogenesis specific basic helix-loop-helix 1	1561403_at	1	2.465	0.05	7	2.642	0.33	40	u	0.003348

NCK1	1	NCK adaptor protein 1	211063_s_at	1	8.511	0.31	7	9.025	0.9	40	u	0.013688
			229895_s_at	0	1.83	0.11	7	1.966	0.31	40	u	0.048341
			204725_s_at	0	8.163	0.38	7	8.456	1.25	40	u	0.25433
NEURL	1	neuralized homolog (Drosophila)	204888_s_at	1	2.34	0.43	7	2.253	0.48	40	d	0.662695
			204889_s_at	0	3.846	0.63	7	4.356	1.03	40	u	0.220026
LPL	1	lipoprotein lipase	203549_s_at	1	9.188	1.29	7	7.038	1.8	40	d	0.004929
			203548_s_at	1	9.283	1.62	7	6.627	1.91	40	d	0.001468
CLEC2D	1	C-type lectin domain family 2, member D	233500_x_at	0	5.15	0.49	7	5.184	1.03	40	u	0.932635
			228426_at	0	3.213	0.28	7	3.743	0.94	40	u	0.008867
			220132_s_at	0	4.459	0.09	7	4.84	0.8	40	u	0.00654
			235522_at	0	4.224	0.52	7	4.524	0.76	40	u	0.331126
SH2D4B	1	SH2 domain containing 4B	1563849_at	1	2.254	0.05	7	2.361	0.2	40	u	0.007192
CXorf1	1	chromosome X open reading frame 1	206711_at	1	2.237	0.04	7	2.376	0.22	40	u	0.000902
DIO1	1	deiodinase, iodothyronine, type I	206457_s_at	1	2.499	0.54	7	2.705	1.24	40	u	0.672956
C20orf11	1	chromosome 20 open reading frame 11	225376_at	1	8.531	0.29	7	8.054	0.78	40	d	0.122922
			218448_at	0	8.566	0.23	7	9.136	0.59	40	u	0.017331
HS3ST2	1	heparan sulfate (glucosamine) 3-O-sulfotransferase 2	219697_at	1	3.318	0.31	7	3.791	0.85	40	u	0.0177
SH3BP1	1	SH3-domain binding protein 1	213633_at	1	5.567	0.48	7	5.654	0.72	40	u	0.766899
MGAT3	1	mannosyl (beta-1,4-)-glycoprotein beta-1,4-N-acetylglucosaminyltransferase	208058_s_at	1	3.301	0.27	7	3.277	0.34	40	d	0.860802
			209764_at	0	5.513	0.59	7	5.365	0.66	40	d	0.588939
			244104_at	0	4.276	0.18	7	4.463	0.48	40	u	0.329729
TCEAL3	1	transcription elongation factor A (SII)-like 3	227279_at	1	9.029	0.51	7	8.305	0.94	40	d	0.058348
SOSTDC1	1	sclerostin domain containing 1	213456_at	1	6.585	1.28	7	4.353	1.97	40	d	0.006933
KIRREL	1	kin of IRRE like (Drosophila)	220825_s_at	1	3.49	0.67	7	3.253	0.49	40	d	0.286447
			232467_at	1	2.88	0.3	7	2.924	0.51	40	u	0.829623
			225303_at	0	7.758	0.7	7	5.544	1.16	40	d	0.000019
C4orf34	1	chromosome 4 open reading frame 34	224990_at	1	10.41	0.3	7	10.09	1.23	40	d	0.179356
			222336_at	0	4.651	0.58	7	4.586	0.99	40	d	0.869177
SLC46A3	1	solute carrier family 46, member 3	214719_at	1	7.376	0.59	7	5.838	1.16	40	d	0.001558
FRMPD1	1	FERM and PDZ domain containing 1	206774_at	1	2.737	0.25	7	2.795	0.27	40	u	0.606338
STAMBP	1	STAM binding protein	202811_at	1	8.081	0.25	7	8.544	0.6	40	u	0.056021
			227515_at	0	6.057	0.47	7	5.859	0.69	40	d	0.476552
HADHA	1	hydroxyacyl-Coenzyme A dehydrogenase/3-ketoacyl-Coenzyme A thiolase/enoyl-Coenzyme A hydratase (trifunctional protein),	208630_at	1	9.537	0.13	7	9.042	0.41	40	d	0.000003
			208629_s_at	1	9.415	0.37	7	9.573	0.61	40	u	0.515412
			208631_s_at	1	10.25	0.13	7	9.512	0.59	40	d	0
HDAC1	1	histone deacetylase 1	201209_at	1	10.26	0.29	7	10.01	0.51	40	d	0.206264
RNMT	1	RNA (guanine-7-) methyltransferase	202683_s_at	1	8.062	0.41	7	7.774	0.73	40	d	0.327767
			202684_s_at	0	3.64	0.54	7	4.582	0.93	40	u	0.014487
MYH4	1	myosin, heavy chain 4, skeletal muscle	208148_at	1	3.341	0.18	7	3.392	0.31	40	u	0.678842
GPR63	1	G protein-coupled receptor 63	220993_s_at	1	2.471	0.49	7	2.934	0.65	40	u	0.085047
AIFM3	1	apoptosis-inducing factor, mitochondrion-associated, 3	244084_at	1	2.799	0.46	7	3.077	0.58	40	u	0.243893
CAV2	1	caveolin 2	203323_at	1	8.678	0.43	7	6.296	1.16	40	d	0.000004
			213426_s_at	0	2.703	0.13	7	2.718	0.37	40	u	0.859495
			203324_s_at	0	9.249	0.47	7	7.528	1.17	40	d	0.000477
TOMM40	1	translocase of outer mitochondrial membrane 40 homolog (yeast)	202264_s_at	1	8.065	0.22	7	8.389	0.58	40	u	0.164267
TRIM46	1	tripartite motif-containing 46	238147_at	1	4.29	0.47	7	4.722	0.61	40	u	0.089551
			220909_at	0	2.43	0.05	7	2.542	0.22	40	u	0.007624
C22orf15	1	chromosome 22 open reading frame 15	1558333_at	1	2.503	0.04	7	2.587	0.21	40	u	0.036092
			1558334_a_at	1	2.77	0.23	7	2.959	0.51	40	u	0.354091
APBA3	1	amyloid beta (A4) precursor protein-binding, family A, member 3 (X11-like 2)	205146_x_at	1	6.59	0.18	7	6.505	0.61	40	d	0.494421
			215148_s_at	1	7.801	0.09	7	7.869	0.66	40	u	0.544511
ADSS	1	adenylosuccinate synthase	221761_at	1	8.869	0.28	7	9.555	0.49	40	u	0.001014
			227310_at	0	2.517	0.05	7	2.9	0.5	40	u	0.000035
ATP2B3	1	ATPase, Ca++ transporting, plasma membrane 3	242036_x_at	1	2.85	0.05	7	2.98	0.2	40	u	0.001768
			215911_x_at	0	3.722	0.13	7	3.801	0.28	40	u	0.475694
			207026_s_at	0	1.877	0.03	7	1.895	0.06	40	u	0.432679
			216312_at	0	2.22	0.04	7	2.277	0.08	40	u	0.080803
RPN2	1	ribophorin II	213491_x_at	1	11.59	0.26	7	11.96	0.56	40	u	0.104048
			213399_x_at	1	11.56	0.27	7	11.96	0.56	40	u	0.071756
			208689_s_at	0	11.23	0.29	7	11.66	0.53	40	u	0.045204
ZNF2	1	zinc finger protein 2	223792_at	1	5.312	0.42	7	5.189	0.67	40	d	0.64797

ST3GAL5	1	ST3 beta-galactoside alpha-2,3-sialyltransferase 5	203217_s_at	1	6.366	0.5	7	5.979	1.07	40	d	0.360044
			239755_at	0	4.375	0.42	7	4.417	0.7	40	u	0.880209
ZBTB7B	1	zinc finger and BTB domain containing 7B	205853_at	1	3.677	0.59	7	3.633	0.74	40	d	0.884548
			235145_at	0	8.625	0.12	7	8.538	0.55	40	d	0.392801
TSSK6	1	testis-specific serine kinase 6	239524_at	1	2.408	0.12	7	2.476	0.17	40	u	0.331166
			224409_s_at	1	2.913	0.18	7	2.905	0.22	40	d	0.930275
			230989_s_at	0	2.15	0.13	7	2.147	0.19	40	d	0.966745
COL24A1	1	collagen, type XXIV, alpha 1	238732_at	1	3.549	0.52	7	3.689	0.39	40	u	0.42161
CNOT3	1	CCR4-NOT transcription complex, subunit 3	203239_s_at	1	7.576	0.37	7	7.369	0.69	40	d	0.453156
			211141_s_at	1	3.603	0.42	7	3.551	0.86	40	d	0.877815
			229143_at	1	3.863	0.31	7	4.052	0.68	40	u	0.485125
NFKBIA	1	nuclear factor of kappa light polypeptide gene enhancer in B-cells inhibitor, alpha	201502_s_at	1	11.25	0.41	7	11.05	0.85	40	d	0.551537
			231699_at	0	2.477	0.15	7	2.658	0.35	40	u	0.196743
SDF2L1	1	stromal cell-derived factor 2-like 1	218681_s_at	1	6.902	0.37	7	7.201	1.06	40	u	0.200771
SFT2D2	1	SFT2 domain containing 2	214838_at	1	5.333	0.45	7	6.624	0.97	40	u	0.00154
HOXA6	1	homeobox A6	208557_at	0	2.827	0.44	7	2.558	0.41	40	d	0.124855
SORBS3	1	sorbin and SH3 domain containing 3	209253_at	1	8.277	0.3	7	7.373	0.67	40	d	0.001235
			207788_s_at	0	7.732	0.47	7	6.284	1.38	40	d	0.000045
DOPEY1	1	dopey family member 1	213271_s_at	1	7.938	0.32	7	7.004	0.6	40	d	0.000271
			213267_at	1	3.831	0.73	7	3.93	0.51	40	u	0.671664
			40612_at	1	5.894	0.35	7	5.599	0.6	40	d	0.221028
			1564387_at	0	2.847	0.12	7	3.192	0.4	40	u	0.00019
LMO1	1	LIM domain only 1 (rhombotin 1)	206718_at	1	2.626	0.06	7	2.873	0.46	40	u	0.002541
DCN	1	decorin	209335_at	1	11.72	0.67	7	8.56	1.29	40	d	0
			240556_at	0	3.436	0.5	7	2.992	0.26	40	d	0.077647
			211813_x_at	0	13.73	0.61	7	10.78	1.33	40	d	0.000001
			201893_x_at	0	13.89	0.42	7	11.22	1.45	40	d	0
			211896_s_at	0	13.67	0.65	7	10.61	1.49	40	d	0.000004
UBAP2	1	ubiquitin associated protein 2	221839_s_at	1	8.248	0.28	7	8.534	0.69	40	u	0.293399
			219192_at	1	7.406	0.36	7	8.066	0.85	40	u	0.053889
AMPD2	1	adenosine monophosphate deaminase 2 (isoform L)	212360_at	1	7.581	0.2	7	7.495	0.6	40	d	0.49516
SLC13A3	1	solute carrier family 13 (sodium-dependent dicarboxylate transporter), member 3	205244_s_at	1	4.47	0.47	7	4.456	0.72	40	d	0.961813
			205243_at	1	2.637	0.11	7	2.825	0.46	40	u	0.036039
			230686_s_at	0	3.005	0.44	7	3.406	0.76	40	u	0.189849
			230687_at	0	2.637	0.27	7	2.81	0.37	40	u	0.254049
C20orf174	1	chromosome 20 open reading frame 174	1558827_a_at	1	2.762	0.22	7	2.984	0.38	40	u	0.145205
			1558826_at	1	2.154	0.09	7	2.254	0.16	40	u	0.118667
CYB5A	1	cytochrome b5 type A (microsomal)	207843_x_at	1	10.55	0.6	7	9.498	1.22	40	d	0.034746
			215726_s_at	1	10.08	0.72	7	9.121	1.41	40	d	0.090301
			209366_x_at	1	10.93	0.66	7	9.772	1.24	40	d	0.022147
			217021_at	0	2.774	0.09	7	2.946	0.35	40	u	0.016135
SPRED2	1	sprouty-related, EVH1 domain containing 2	212466_at	1	4.445	0.48	7	3.927	1.01	40	d	0.198139
			212458_at	1	8.29	0.2	7	7.067	1.62	40	d	0.000049
			214026_s_at	1	2.053	0.02	7	2.127	0.17	40	u	0.013787
MON1A	1	MON1 homolog A (yeast)	224500_s_at	1	5.081	0.06	7	5.237	0.75	40	u	0.209718
ZSWIM3	1	zinc finger, SWIM-type containing 3	228223_at	1	5.065	0.29	7	5.36	0.5	40	u	0.142011
ZNF804A	1	zinc finger protein 804A	215767_at	1	2.83	0.14	7	2.953	0.3	40	u	0.302287
SLC25A31	1	solute carrier family 25 (mitochondrial carrier; adenine nucleotide translocator), member 31	221037_s_at	1	2.941	0.05	7	3.148	0.16	40	u	0.000002
KCNMB4	1	potassium large conductance calcium-activated channel, subfamily M, beta	222857_s_at	1	4.535	0.81	7	3.837	1.2	40	d	0.153764
			219287_at	1	3.887	0.58	7	3.795	0.65	40	d	0.733549
KCNJ11	1	potassium inwardly-rectifying channel, subfamily J, member 11	231740_at	1	2.93	0.46	7	2.702	0.51	40	d	0.282479
DNAJB14	1	DnaJ (Hsp40) homolog, subfamily B, member 14	219237_s_at	1	8.27	0.25	7	8.352	0.63	40	u	0.743141
			222850_s_at	1	7.269	0.54	7	7.731	0.74	40	u	0.126677
WIT1	1	Wilms tumor upstream neighbor 1	206954_at	1	2.526	0.05	7	2.754	0.35	40	u	0.000453
EXOC3L	1	exocyst complex component 3-like	1554937_x_at	0	3.693	0.71	7	3.668	0.45	40	d	0.903444
			1559272_at	0	4.904	0.26	7	4.958	0.22	40	u	0.563536
BRD3	1	bromodomain containing 3	203825_at	1	9.022	0.38	7	9.03	0.83	40	u	0.981465
			1555028_at	0	2.266	0.04	7	2.323	0.08	40	u	0.084595
HIST1H4A	1	histone cluster 1, H4a	208046_at	1	2.501	0.1	7	2.504	0.08	40	u	0.925496
			204779_s_at	1	5.815	0.52	7	6.825	1.47	40	u	0.003928

HOXB7	1	homeobox B7	204778_x_at	1	3.683	0.31	7	4.209	0.99	40	u	0.015028
			216973_s_at	1	5.909	0.39	7	6.981	1.28	40	u	0.000255
MAGED1	1	melanoma antigen family D, 1	209014_at	1	10.53	0.49	7	10.97	0.78	40	u	0.165182
CENTG1	1	centaurin, gamma 1	206152_at	1	5.409	0.2	7	5.428	0.49	40	u	0.920912
			215080_s_at	0	1.985	0.02	7	2.021	0.04	40	u	0.031494
UGT8	1	UDP glycosyltransferase 8 (UDP-galactose ceramide galactosyltransferase)	228956_at	1	4.605	1.08	7	5.133	2.47	40	u	0.589053
			208358_s_at	0	3.494	0.58	7	4.522	1.99	40	u	0.014328
BMP2K	1	BMP2 inducible kinase	59644_at	1	6.037	1.01	7	5.598	0.64	40	d	0.143227
			219546_at	1	5.623	0.92	7	5.229	0.72	40	d	0.219076
			232688_at	0	3.884	1.05	7	3.121	0.21	40	d	0.125095
			214716_at	0	3.581	0.54	7	3.739	0.56	40	u	0.500492
			37170_at	0	7.899	0.74	7	7.629	0.58	40	d	0.291285
			226853_at	0	8.14	1.08	7	7.428	0.65	40	d	0.024159
SLC25A23	1	solute carrier family 25 (mitochondrial carrier; phosphate carrier), member 23	226010_at	1	7.29	0.26	7	7.218	0.81	40	d	0.671888
			220182_at	0	2.788	0.42	7	2.931	0.6	40	u	0.558966
USP39	1	ubiquitin specific peptidase 39	217829_s_at	0	7.546	0.18	7	7.964	0.47	40	u	0.028964
ATXN2L	1	ataxin 2-like	207798_s_at	1	4.455	0.49	7	4.495	0.58	40	u	0.867289
			201806_s_at	1	7.255	0.22	7	7.462	0.44	40	u	0.239948
AP2A2	1	adaptor-related protein complex 2, alpha 2 subunit	215764_x_at	1	7.136	0.21	7	6.709	0.53	40	d	0.043558
			212159_x_at	1	8.719	0.29	7	8.221	0.62	40	d	0.047467
			211779_x_at	1	8.771	0.23	7	8.189	0.62	40	d	0.019721
			212161_at	1	5.186	0.38	7	4.709	0.62	40	d	0.060784
CYR61	1	cysteine-rich, angiogenic inducer, 61	201289_at	1	11.75	0.39	7	9.637	1.1	40	d	0
			210764_s_at	0	11.71	0.59	7	8.935	1.46	40	d	0.000015
MAFF	1	v-maf musculoaponeurotic fibrosarcoma oncogene homolog F (avian)	36711_at	1	9.688	0.79	7	7.807	1.18	40	d	0.000245
			205193_at	1	8.73	0.73	7	7.192	0.73	40	d	0.000009
EEA1	1	early endosome antigen 1	225885_at	9	8.316	0.49	7	7.882	0.85	40	d	0.208134
			204840_s_at	1	4.632	0.45	7	5.173	0.7	40	u	0.059721
			204841_s_at	0	3.396	0.43	7	4.013	0.76	40	u	0.047221
IL1R1	1	interleukin 1 receptor, type I	202948_at	1	9.207	0.53	7	8.089	1.13	40	d	0.01572
			215561_s_at	0	4.485	0.51	7	4.486	0.75	40	u	0.996027
CTTN	1	cortactin	201059_at	1	9.67	0.24	7	9.505	1.01	40	d	0.384865
			227473_at	0	9.343	0.38	7	9.301	1.41	40	d	0.878306
			214074_s_at	0	5.485	0.33	7	6.119	1.12	40	u	0.008493
			1567277_at	0	2.91	0.27	7	2.93	0.26	40	u	0.859468
			214782_at	0	4.209	0.49	7	4.632	1.01	40	u	0.292813
			214073_at	0	4.259	0.7	7	5.662	1.65	40	u	0.035068
MYBBP1A	1	MYB binding protein (P160) 1a	219098_at	1	6.962	0.38	7	6.24	0.76	40	d	0.019858
			233803_s_at	1	6.175	0.5	7	5.452	0.54	40	d	0.002443
C20orf59	1	chromosome 20 open reading frame 59	233328_x_at	1	2.826	0.54	7	3.26	1.15	40	u	0.341644
			219559_at	1	5.26	0.29	7	5.939	0.95	40	u	0.001456
			232922_s_at	1	5.998	0.48	7	5.879	0.88	40	d	0.733365
ZNF503	1	zinc finger protein 503	227195_at	1	6.812	0.38	7	6.313	1.36	40	d	0.071429
			228684_at	0	2.911	0.22	7	2.92	0.46	40	u	0.96178
			232178_at	0	1.607	0.03	7	1.667	0.19	40	u	0.070629
NR1D1	1	nuclear receptor subfamily 1, group D, member 1	31637_s_at	1	7.496	0.78	7	7.341	0.72	40	d	0.613954
			217476_at	1	4.013	0.63	7	3.99	0.54	40	d	0.918652
			204760_s_at	1	2.618	0.82	7	2.417	0.52	40	d	0.407384
C1orf162	1	chromosome 1 open reading frame 162	228532_at	1	6.588	0.47	7	6.69	0.95	40	u	0.788867
HIST2H2BE	1	histone cluster 2, H2be	202708_s_at	1	8.069	0.6	7	8.459	1.85	40	u	0.319859
FLVCR2	1	feline leukemia virus subgroup C cellular receptor family, member 2	222866_s_at	1	2.757	0.5	7	3.403	0.82	40	u	0.055439
			219316_s_at	1	4.05	0.12	7	4.541	0.54	40	u	0.000012
			1569536_at	0	5.172	0.2	7	5.335	0.47	40	u	0.383944
AP1S1	1	adaptor-related protein complex 1, sigma 1 subunit	209635_at	1	7.032	0.38	7	8.223	0.8	40	u	0.000458
			205195_at	0	4.822	0.24	7	6.075	1.07	40	u	0
			205196_s_at	0	5.717	0.32	7	6.657	1.16	40	u	0.000226
SAV1	1	salvador homolog 1 (Drosophila)	222573_s_at	1	8.738	0.49	7	7.758	0.9	40	d	0.008824
			218276_s_at	1	9.207	0.16	7	8.02	0.9	40	d	0
			234491_s_at	0	8.061	0.2	7	7.471	0.72	40	d	0.000184
ADAMTS7	1	ADAM metallopeptidase with thrombospondin type 1 motif, 7	228911_at	1	3.048	0.22	7	3.127	0.34	40	u	0.561268
			220705_s_at	0	3.933	0.83	7	3.708	0.82	40	d	0.517689
			220706_at	0	3.244	0.33	7	3.033	0.29	40	d	0.098468
ANKRD25	1	ankyrin repeat domain 25	218418_s_at	1	8.818	0.4	7	6.919	1.01	40	d	0.000018
			221068_at	0	5.1	0.37	7	4.489	0.49	40	d	0.003779

TTC26	1	tetratricopeptide repeat domain 26	219758_at	1	2.705	0.16	7	3.218	0.57	40	u	0.00007
			233999_s_at	0	3.058	0.44	7	3.805	1.06	40	u	0.080009
			1554672_at	0	2.616	0.06	7	2.756	0.16	40	u	0.000616
AGR2	1	anterior gradient homolog 2 (Xenopus laevis)	228969_at	1	8.273	0.98	7	7.111	2.93	40	d	0.070364
			209173_at	0	11.18	1.04	7	8.677	4.48	40	d	0.004596
HDLBP	1	high density lipoprotein binding protein (vigilin)	225012_at	1	7.925	0.23	7	7.325	0.92	40	d	0.00151
			200643_at	1	9.302	0.27	7	9.28	0.53	40	d	0.915145
			221767_x_at	1	10.52	0.28	7	10.59	0.56	40	u	0.754409
			235624_at	0	2.404	0.15	7	2.488	0.16	40	u	0.219617
			219674_s_at	0	3.56	0.2	7	3.361	0.52	40	d	0.329303
			240152_at	0	3.375	0.32	7	3.487	0.27	40	u	0.339212
			222916_s_at	0	5.924	0.55	7	5.314	1.01	40	d	0.136167
GNB3	1	guanine nucleotide binding protein (G protein), beta polypeptide 3	206047_at	1	3.996	0.3	7	3.844	0.31	40	d	0.246619
ANKZF1	1	ankyrin repeat and zinc finger domain containing 1	218274_s_at	1	7.029	0.3	7	6.41	0.67	40	d	0.02283
ARL6	1	ADP-ribosylation factor-like 6	235320_at	0	4.924	0.74	7	4.782	0.84	40	d	0.682605
			223735_at	0	2.782	0.37	7	3.363	0.83	40	u	0.081251
RAP1GDS1	1	RAP1, GTP-GDP dissociation stimulator 1	209444_at	1	7.889	0.39	7	8.751	0.72	40	u	0.003938
			217457_s_at	0	7.526	0.29	7	8.431	0.74	40	u	0.003236
			237856_at	0	3.525	0.49	7	3.844	0.55	40	u	0.167617
DPH1	1	DPH1 homolog (S. cerevisiae)	202632_at	1	9.079	0.14	7	8.561	0.55	40	d	0.000021
			222041_at	0	5.684	0.46	7	4.793	0.71	40	d	0.002851
CREBL1	1	cAMP responsive element binding protein-like 1	203168_at	0	5.794	0.36	7	5.626	0.59	40	d	0.481465
			211611_s_at	0	3.683	0.68	7	3.427	0.61	40	d	0.327685
			216208_s_at	0	3.359	0.65	7	3.44	0.66	40	u	0.771317
			1554487_a_at	0	2.688	0.4	7	2.637	0.5	40	d	0.802609
B3GNT5	1	UDP-GlcNAc:betaGal beta-1,3-N-acetylglucosaminyltransferase 5	225612_s_at	1	6.928	0.34	7	7.282	2.17	40	u	0.349351
			1554835_a_at	0	2.861	0.28	7	4.375	1.95	40	u	0.000043
			1556134_a_at	0	2.078	0.11	7	2.22	0.4	40	u	0.077994
DBNL	1	drebrin-like	222429_at	1	8.481	0.15	7	8.37	0.73	40	d	0.40555
ARHGEF10L	1	Rho guanine nucleotide exchange factor (GEF) 10-like	221656_s_at	1	7.688	0.29	7	7.077	0.65	40	d	0.020038
			1570511_at	0	4.912	0.41	7	4.528	0.65	40	d	0.145864
STT3A	1	STT3, subunit of the oligosaccharyltransferase complex, homolog A (S. cerevisiae)	202223_at	1	8.222	0.45	7	7.323	1.22	40	d	0.065606
C17orf70	1	chromosome 17 open reading frame 70	225322_s_at	1	4.502	0.49	7	4.31	0.9	40	d	0.594018
			221800_s_at	1	6.662	0.17	7	6.43	0.76	40	d	0.104561
PTN	1	pleiotrophin (heparin binding growth factor 8, neurite growth-promoting factor 1)	209465_x_at	1	11.15	0.98	7	6.537	1.22	40	d	0
			211737_x_at	0	11.85	1.15	7	6.908	1.53	40	d	0
			209466_x_at	0	11.75	1.22	7	6.99	1.38	40	d	0
			208408_at	0	2.3	0.04	7	2.371	0.22	40	u	0.062239
C1orf106	1	chromosome 1 open reading frame 106	219010_at	1	5.356	0.66	7	7.374	2.04	40	u	0.000059
RAE1	1	RAE1 RNA export 1 homolog (S. pombe)	201558_at	1	8.037	0.31	7	8.931	0.76	40	u	0.004264
			211318_s_at	0	7.501	0.38	7	8.225	0.73	40	u	0.015462
RNF122	1	ring finger protein 122	219897_at	1	4.308	0.61	7	4.564	0.6	40	u	0.312054
SOX7	1	SRY (sex determining region Y)-box 7	228698_at	1	5.746	1.07	7	4.428	0.62	40	d	0.02601
			224013_s_at	0	4.047	1.1	7	2.762	0.24	40	d	0.02942
HSPB6	1	heat shock protein, alpha-crystallin-related, B6	226304_at	1	7.708	1.21	7	4.439	1.12	40	d	0
			214767_s_at	1	6.904	1.2	7	4.127	1.27	40	d	0.000004
IER2	1	immediate early response 2	202081_at	1	12.36	0.54	7	10.95	0.8	40	d	0.00007
COX5A	1	cytochrome c oxidase subunit Va	203663_s_at	0	10.67	0.18	7	11.39	0.61	40	u	0.000002
			217451_at	0	2.293	0.05	7	2.343	0.07	40	u	0.100583
			229426_at	0	4.697	0.3	7	4.956	0.44	40	u	0.147979
SEC24D	1	SEC24 related gene family, member D (S. cerevisiae)	202375_at	1	6.539	0.31	7	7.468	0.86	40	u	0.000045
			215641_at	0	2.676	0.13	7	3.018	0.45	40	u	0.000541
			215209_at	0	5.798	0.55	7	5.23	1.14	40	d	0.213113
TGFB2	1	transforming growth factor, beta 2	209908_s_at	1	2.499	0.24	7	2.766	0.89	40	u	0.132469
			220407_s_at	1	3.222	0.36	7	3.815	1.47	40	u	0.039012
			220406_at	1	2.637	0.03	7	2.675	0.06	40	u	0.124734
CHRNE	1	cholinergic receptor, nicotinic, epsilon	215916_at	10	4.36	0.23	7	4.399	0.3	40	u	0.751813
			207274_at	9	2.537	0.26	7	2.484	0.1	40	d	0.645195
			229547_s_at	1	5.837	0.25	7	5.343	0.48	40	d	0.011726
			1557536_at	1	3.593	0.45	7	3.272	0.46	40	d	0.101166

WNK2	1	WNK lysine deficient protein kinase 2	227217_at	0	5.195	0.6	7	4.814	0.6	40	d	0.136532
			1562026_at	0	4.227	0.21	7	4.293	0.3	40	u	0.59121
			1560866_at	0	2.851	0.36	7	2.958	0.39	40	u	0.512198
MTA2	1	metastasis associated 1 family, member 2	203444_s_at	1	2.505	0.1	7	2.606	0.35	40	u	0.155619
GAL3ST3	1	galactose-3-O-sulfotransferase 3	1553257_at	1	2.774	0.28	7	2.834	0.24	40	u	0.569274
ARVCF	1	armadillo repeat gene deletes in velocardiofacial syndrome	1556149_at	0	4.526	0.29	7	4.768	0.33	40	u	0.08432
			205784_x_at	0	6.409	0.25	7	6.097	0.38	40	d	0.044063
			216204_at	0	2.447	0.07	7	2.484	0.06	40	u	0.187771
			217516_x_at	0	7.219	0.25	7	6.75	0.54	40	d	0.032021
ATXN7L1	1	ataxin 7-like 1	214342_at	0	2.593	0.15	7	2.59	0.17	40	d	0.961134
			214343_s_at	0	2.389	0.14	7	2.49	0.38	40	u	0.228213
			232265_at	0	1.968	0.02	7	2.167	0.55	40	u	0.030216
			227732_at	0	6.41	0.29	7	6.088	0.68	40	d	0.234038
ID3	1	inhibitor of DNA binding 3, dominant negative helix-loop-helix protein	207826_s_at	0	8.951	1	7	8.111	1.02	40	d	0.055237
TUBGCP3	1	tubulin, gamma complex associated protein 3	203690_at	1	6.818	0.21	7	6.657	0.69	40	d	0.259375
			1554086_at	0	2.601	0.16	7	3.164	0.75	40	u	0.00018
			215739_s_at	0	4.855	0.63	7	4.988	0.84	40	u	0.697935
CSNK1A1L	1	casein kinase 1, alpha 1-like	no probeset available									
SMARCC2	1	SWI/SNF related, matrix associated, actin dependent regulator of chromatin, subfamily c, member 2	201321_s_at	1	8.401	0.3	7	7.584	0.75	40	d	0.007641
			201320_at	1	8.143	0.29	7	7.384	0.71	40	d	0.009381
			1561973_at	0	3.206	0.47	7	3.327	0.57	40	u	0.604038
SLC37A3	1	solute carrier family 37 (glycerol-3-phosphate transporter), member 3	223304_at	1	7.872	0.26	7	7.887	0.51	40	u	0.94344
ZNF248	1	zinc finger protein 248	213269_at	1	7.209	0.28	7	6.561	1.09	40	d	0.00364
			1569557_at	0	3.371	0.29	7	3.438	0.67	40	u	0.79996
C12orf12	1	chromosome 12 open reading frame 12	236968_at	0	2.853	0.06	7	2.971	0.2	40	u	0.005276
TSPAN3	1	tetraspanin 3	200972_at	1	10.07	0.34	7	9.89	0.77	40	d	0.550125
			200973_s_at	0	9.262	0.37	7	8.771	1.31	40	d	0.066034
C8orf58	1	chromosome 8 open reading frame 58	227263_at	1	6.242	0.34	7	5.318	0.47	40	d	0.000015
			236296_x_at	0	5.108	0.5	7	5.154	0.57	40	u	0.846044
SLC9A5	1	solute carrier family 9 (sodium/hydrogen exchanger), member 5	220650_s_at	1	4.421	0.62	7	3.993	0.72	40	d	0.153554
			1559092_at	0	6.581	0.31	7	6.605	0.32	40	u	0.856519
			232564_at	0	3.511	0.27	7	3.516	0.29	40	u	0.96313
PTPLAD1	1	protein tyrosine phosphatase-like A domain containing 1	217777_s_at	1	8.399	0.91	7	8.724	1.37	40	u	0.557995
			222405_at	1	11.82	0.59	7	11.47	0.7	40	d	0.23296
			222404_x_at	1	11.25	0.68	7	11.21	0.7	40	d	0.886786
			234000_s_at	0	10.91	0.66	7	10.9	0.71	40	d	0.959229
CALB1	1	calbindin 1, 28kDa	205626_s_at	1	2.758	0.07	7	2.996	0.57	40	u	0.016477
			205625_s_at	1	2.359	0.03	7	2.595	0.71	40	u	0.044232
PKD1	1	polycystic kidney disease 1 (autosomal dominant)	202328_s_at	1	8.104	0.54	7	7.384	1	40	d	0.075914
			202327_s_at	1	3.7	0.3	7	3.633	0.57	40	d	0.77075
			216949_s_at	0	6.417	0.31	7	6.269	0.87	40	d	0.435568
FOXR2	1	forkhead box R2	1569669_at	1	2.711	0.19	7	2.775	0.35	40	u	0.640397
KCNIP3	1	Kv channel interacting protein 3, calsenilin	228269_x_at	1	4.705	0.25	7	4.133	0.45	40	d	0.00274
			231774_at	0	4.849	0.7	7	4.148	0.84	40	d	0.047416
			1555694_a_at	0	2.808	0.36	7	2.793	0.4	40	d	0.927401
			233688_at	0	2.386	0.18	7	2.359	0.12	40	d	0.610954
			1569355_at	0	2.526	0.14	7	2.69	0.3	40	u	0.170605
			234647_at	0	2.225	0.05	7	2.259	0.13	40	u	0.491471
			234198_at	0	1.923	0.07	7	2.079	0.3	40	u	0.006762
RGS16	1	regulator of G-protein signaling 16	209324_s_at	1	6.984	0.67	7	7.861	1.2	40	u	0.072314
			209325_s_at	0	6.04	0.58	7	7.139	1.23	40	u	0.028444
LASS1	1	LAG1 homolog, ceramide synthase 1	229448_at	0	4.367	0.31	7	4.061	0.54	40	d	0.159533
NFATC4	1	nuclear factor of activated T-cells, cytoplasmic, calcineurin-dependent 4	213345_at	1	6.955	0.3	7	6.803	0.84	40	d	0.414185
			236270_at	1	3.856	0.73	7	3.129	0.63	40	d	0.009653
			205897_at	0	5.074	0.25	7	5.292	0.97	40	u	0.246646
KIAA1166	1	KIAA1166	220040_x_at	1	4.714	0.22	7	4.566	1.06	40	d	0.445721
			225784_s_at	1	3.911	0.7	7	3.846	1.46	40	d	0.911006
			234809_at	1	2.35	0.07	7	2.413	0.16	40	u	0.326238
ACACA	1	acetyl-Coenzyme A carboxylase alpha	212186_at	1	8.527	1.01	7	7.904	1	40	d	0.145438
			214358_at	0	5.394	0.88	7	5.189	1.1	40	d	0.648474
ABCG1	1	ATP-binding cassette, sub-family G (WHITE), member 1	204567_s_at	1	6.09	0.37	7	6.937	1.07	40	u	0.00107
			211113_s_at	0	5.181	0.46	7	5.609	0.98	40	u	0.272333

RGS3	1	regulator of G-protein signaling 3	203823_at	1	8.312	0.49	7	7.59	0.71	40	d	0.015605
			220300_at	0	2.436	0.1	7	2.544	0.31	40	u	0.097762
ODF1	1	outer dense fiber of sperm tails 1	214485_at	1	2.83	0.62	7	2.856	0.56	40	u	0.912982
C4orf31	1	chromosome 4 open reading frame 31	219747_at	1	5.228	1.18	7	3.165	0.63	40	d	0.005636
			230319_at	0	3.746	0.74	7	3.006	0.21	40	d	0.049859
			215059_at	0	3.593	0.38	7	3.275	0.21	40	d	0.091041
SEPT10	1	septin 10	212698_s_at	1	10.06	0.29	7	9.255	0.86	40	d	0.000145
			214720_x_at	0	7.341	0.64	7	6.545	1.22	40	d	0.106422
FBXO27	1	F-box protein 27	235169_at	1	3.695	0.39	7	4.25	1.01	40	u	0.166637
SLC18A3	1	solute carrier family 18 (vesicular acetylcholine), member 3	207150_at	1	2.319	0.11	7	2.309	0.1	40	d	0.814885
INTU	1	inturned planar cell polarity effector homolog (Drosophila)	228946_at	1	6.955	0.51	7	5.286	1.02	40	d	0.000142
SYNGR3	1	synaptogyrin 3	205691_at	1	2.746	0.47	7	3.603	1.07	40	u	0.046455
NUP93	1	nucleoporin 93kDa	202188_at	1	7.241	0.36	7	7.775	0.73	40	u	0.071784
HMOX2	1	heme oxygenase (decycling) 2	218121_at	1	6.058	0.18	7	6.444	0.56	40	u	0.002769
			218120_s_at	0	8.279	0.3	7	8.466	0.43	40	u	0.28353
MRVI1	1	murine retrovirus integration site 1 homolog	226047_at	1	7.625	0.44	7	6.401	1.07	40	d	0.005668
			230214_at	1	5.631	0.69	7	5.714	0.85	40	u	0.809873
			224550_s_at	0	3.95	0.6	7	3.893	0.98	40	d	0.885762
PTGDR	1	prostaglandin D2 receptor (DP)	234165_at	1	2.381	0.08	7	2.45	0.19	40	u	0.363463
			215894_at	0	2.496	0.18	7	2.483	0.21	40	d	0.884917
			215937_at	0	2.874	0.03	7	2.951	0.11	40	u	0.00079
ZBTB22	1	zinc finger and BTB domain containing 22	213081_at	0	7.182	0.46	7	6.412	0.73	40	d	0.011064
GJB6	1	gap junction protein, beta 6	231771_at	1	2.255	0.03	7	2.734	1.28	40	u	0.024911
			211644_x_at	0	10.31	1.18	7	8.568	2.7	40	d	0.106918
ELOVL3	1	elongation of very long chain fatty acids (FEN1/Elo2, SUR4/Elo3, yeast)-like 3	234513_at	0	1.874	0.02	7	2.012	0.39	40	u	0.03623
MEPE	1	matrix, extracellular phosphoglycoprotein with ASARM motif (bone)	221150_at	1	3.681	0.56	7	3.505	0.44	40	d	0.36383
RERG	1	RAS-like, estrogen-regulated, growth inhibitor	227758_at	1	9.776	0.75	7	7.689	1.91	40	d	0.007689
			244745_at	0	8.236	1.07	7	5.994	2.3	40	d	0.0169
GALNT13	1	UDP-N-acetyl-alpha-D-galactosamine:polypeptide N-acetylgalactosaminyltransferase 13 (GalNAc-	243779_at	1	2.495	0.06	7	2.688	0.28	40	u	0.000611
			236536_at	1	3.666	0.37	7	4.079	0.69	40	u	0.135866
			234472_at	0	3.028	0.2	7	3.703	1.13	40	u	0.001417
CCDC141	1	coiled-coil domain containing 141	1553645_at	0	3.533	0.76	7	3.313	0.25	40	d	0.504969
SYT10	1	synaptotagmin X	no probeset available									
OSTF1	1	osteoclast stimulating factor 1	204479_at	1	7.406	0.4	7	7.099	0.75	40	d	0.303594
DNAJA4	1	DnaJ (Hsp40) homolog, subfamily A, member 4	225061_at	1	8.271	0.48	7	8.378	1.23	40	u	0.825002
			1554333_at	0	3.841	0.41	7	4.449	1.08	40	u	0.156922
			220395_at	0	4.398	0.26	7	4.756	0.62	40	u	0.150438
			1554334_a_at	0	7.079	0.52	7	7.781	0.98	40	u	0.078211
DOCK8	1	dedicator of cytokinesis 8	232842_at	1	2.688	0.21	7	2.909	0.27	40	u	0.050421
			232843_s_at	1	6.088	0.4	7	4.973	1.04	40	d	0.008603
			225502_at	1	7.104	0.35	7	6.086	1.34	40	d	0.000358
			239027_at	0	4.065	0.37	7	4.025	0.4	40	d	0.813428
GRB7	1	growth factor receptor-bound protein 7	210761_s_at	0	7.446	0.62	7	7.676	2.12	40	u	0.590795
C3orf64	1	chromosome 3 open reading frame 64	221935_s_at	1	8.387	0.41	7	6.965	0.88	40	d	0.000154
C1orf63	1	chromosome 1 open reading frame 63	209007_s_at	1	10.13	0.37	7	9.27	0.68	40	d	0.002442
			209006_s_at	0	8.347	0.59	7	8.019	0.99	40	d	0.408995
GLRA2	1	glycine receptor, alpha 2	207462_at	1	2.295	0.08	7	2.425	0.28	40	u	0.028126
C17orf59	1	chromosome 17 open reading frame 59	222882_s_at	1	3.012	0.5	7	2.974	0.55	40	d	0.866891
			219417_s_at	1	6.557	0.14	7	6.001	0.5	40	d	0.000003
SPRN	1	shadow of prion protein homolog (zebrafish)	238333_s_at	1	4.296	0.36	7	4.799	1.01	40	u	0.029706
			238331_at	1	3.548	0.29	7	3.395	0.41	40	d	0.357251
			238334_at	1	4.291	0.38	7	4.13	0.63	40	d	0.526722
SLC15A4	1	solute carrier family 15, member 4	225043_at	1	8.02	0.24	7	7.854	0.37	40	d	0.267046
			225057_at	1	7.57	0.14	7	7.19	0.53	40	d	0.000782
			229503_at	0	2.601	0.2	7	2.688	0.23	40	u	0.355918
PRTFDC1	1	phosphoribosyl transferase domain containing 1	222803_at	1	7.018	0.64	7	6.658	2.13	40	d	0.409678
LEPRE1	1	leucine proline-enriched proteoglycan (leprecan) 1	220750_s_at	1	6.404	0.33	7	6.562	0.52	40	u	0.452901
ZC3H3	1	zinc finger CCCH-type containing 3	213445_at	1	5.564	0.52	7	5.918	0.86	40	u	0.3068

COMMD2	1	COMM domain containing 2	223491_at	1	5.985	0.37	7	6.846	0.99	40	u	0.031911
SPRYD4	1	SPRY domain containing 4	225616_at	1	6.413	0.32	7	6.149	0.66	40	d	0.313284
SAMD13	1	sterile alpha motif domain containing 13	229402_at	1	5.268	0.34	7	4.628	1.17	40	d	0.01023
CHEK1	1	CHK1 checkpoint homolog (S. pombe)	205393_s_at	1	4.344	0.29	7	5.88	1.14	40	u	0
			205394_at	1	3.874	0.62	7	5.773	1.31	40	u	0.000623
KIF11	1	kinesin family member 11	204444_at	1	3.578	0.49	7	6.961	1.21	40	u	0
RASD2	1	RASD family, member 2	223634_at	1	4.464	0.22	7	5.612	1.73	40	u	0.000284
SLC40A1	1	solute carrier family 40 (iron-regulated transporter), member 1	223044_at	1	10.92	0.59	7	9.316	1.5	40	d	0.008754
			233123_at	0	6.542	0.26	7	5.948	0.98	40	d	0.00352
RNF34	1	ring finger protein 34	219035_s_at	2	7.886	0.29	7	7.981	0.66	40	u	0.7163
			229855_at	0	2.802	0.35	7	2.939	0.45	40	u	0.461483
			236288_at	0	4.243	0.35	7	4.635	0.53	40	u	0.071392
UBLCP1	1	ubiquitin-like domain containing CTD phosphatase 1	227413_at	1	7.856	0.31	7	7.662	0.69	40	d	0.479905
			243916_x_at	0	7.483	0.49	7	8.208	0.62	40	u	0.006379
CCT2	1	chaperonin containing TCP1, subunit 2 (beta)	201947_s_at	1	11.37	0.18	7	11.94	0.73	40	u	0.000211
			201946_s_at	1	9.374	0.35	7	10.3	0.91	40	u	0.012908
ZPBP2	1	zona pellucida binding protein 2	231442_at	1	2.064	0.01	7	2.24	0.44	40	u	0.017303
GPSN2	1	glycoprotein, synaptic 2	208336_s_at	1	9.206	0.27	7	8.278	0.9	40	d	0.00002
			231556_at	0	2.382	0.07	7	2.487	0.12	40	u	0.037496
			1569392_at	0	3.892	0.55	7	4.01	0.63	40	u	0.650904
SH3GLB1	1	SH3-domain GRB2-like endophilin B1	210101_x_at	1	9.658	0.35	7	9.75	0.84	40	u	0.781052
			209091_s_at	0	9.815	0.29	7	9.966	0.9	40	u	0.427058
			209090_s_at	0	9.598	0.26	7	8.708	0.71	40	d	0.000008
SECISBP2	1	SECIS binding protein 2	218265_at	1	8.221	0.38	7	7.584	0.64	40	d	0.015257
			224250_s_at	0	8.763	0.36	7	8.814	0.66	40	u	0.846873
SLC12A6	1	solute carrier family 12 (potassium/chloride transporters), member 6	226741_at	2	7.803	0.21	7	7.522	0.35	40	d	0.05214
			223596_at	1	2.992	0.28	7	2.933	0.36	40	d	0.685303
			220740_s_at	1	3.165	0.29	7	3.29	0.59	40	u	0.590651
KBTBD6	1	kelch repeat and BTB (POZ) domain containing 6	226479_at	1	6.346	0.46	7	6.258	1.02	40	d	0.826992
			1553111_a_at	0	4.966	0.74	7	4.806	0.96	40	d	0.684797
GNG10	1	guanine nucleotide binding protein (G protein), gamma 10	201921_at	1	10.14	0.31	7	10.56	0.64	40	u	0.101336
C1QTNF5	1	C1q and tumor necrosis factor related protein 5	223499_at	1	7.339	0.53	7	7.343	0.91	40	u	0.991527
ANKRD28	1	ankyrin repeat domain 28	213035_at	1	6.851	0.42	7	5.959	0.87	40	d	0.012565
			1561079_at	0	6.781	0.37	7	6.435	0.74	40	d	0.242774
			229307_at	0	6.955	0.97	7	6.507	0.75	40	d	0.180087
			226025_at	0	9.16	0.36	7	9.075	0.68	40	d	0.751342
C9orf41	1	chromosome 9 open reading frame 41	228324_at	0	6.594	0.46	7	6.902	0.55	40	u	0.178182
			241781_at	0	3.119	0.25	7	3.54	0.51	40	u	0.041556
FAM40A	1	family with sequence similarity 40, member A	225226_at	1	6.666	0.16	7	6.386	0.5	40	d	0.010805
TRAF3	1	TNF receptor-associated factor 3	208315_x_at	1	4.63	0.61	7	5.257	0.73	40	u	0.041535
			221571_at	0	6.745	0.42	7	6.331	0.78	40	d	0.18654
GALNT5	1	UDP-N-acetyl-alpha-D-galactosamine:polypeptide N-	236129_at	0	3.03	0.35	7	3.334	0.53	40	u	0.156854
			229555_at	0	5.315	0.3	7	5.605	0.51	40	u	0.157086
CHD3	1	chromodomain helicase DNA binding protein 3	208806_at	1	7.01	0.28	7	6.495	0.71	40	d	0.071966
			208807_s_at	1	7.982	0.38	7	7.12	0.89	40	d	0.017684
CHD6	1	chromodomain helicase DNA binding protein 6	225026_at	1	7.035	0.49	7	6.591	0.86	40	d	0.200613
			225031_at	1	7.049	0.48	7	6.489	1.35	40	d	0.066356
			234681_s_at	0	2.162	0.22	7	2.29	0.34	40	u	0.345547
			1555463_a_at	0	2.55	0.12	7	2.636	0.3	40	u	0.470136
ZMYND8	1	zinc finger, MYND-type containing 8	209048_s_at	1	8.293	0.43	7	8.162	1.11	40	d	0.766437
			209049_s_at	1	8.568	0.39	7	8.656	0.91	40	u	0.804024
			230533_at	0	4.502	0.32	7	5.237	0.7	40	u	0.010908
			207130_at	0	3.353	0.35	7	3.682	0.65	40	u	0.205983
			243814_at	0	3.747	0.12	7	3.948	0.35	40	u	0.012759
			1558571_at	0	2.387	0.03	7	2.515	0.2	40	u	0.00047
			214795_at	0	5.069	0.54	7	4.741	0.88	40	d	0.355404
PTGES3	1	prostaglandin E synthase 3 (cytosolic)	200627_at	1	12.18	0.16	7	12.25	0.66	40	u	0.536996
C6orf166	1	chromosome 6 open reading frame 166	223145_s_at	1	8.076	0.25	7	7.699	0.71	40	d	0.021489
			223143_s_at	0	3.425	0.48	7	3.402	0.95	40	d	0.949998
			223144_s_at	0	7.521	0.28	7	7.345	0.74	40	d	0.545966
NDNL2	1	necdin-like 2	226008_at	1	6.19	0.3	7	5.635	0.52	40	d	0.0099

ZNF318	1	zinc finger protein 318	203521_s_at	1	6.349	0.44	7	6.311	0.94	40	d	0.918737
			203520_s_at	1	6.1	0.24	7	6.03	1.05	40	d	0.719415
			231388_at	0	5.656	0.24	7	5.786	0.2	40	u	0.133004
EHMT2	1	euchromatic histone-lysine N-methyltransferase 2	207484_s_at	0	5.342	0.42	7	5.461	0.62	40	u	0.632867
			202326_at	0	7.361	0.4	7	7.415	0.88	40	u	0.875671
			229079_at	0	5.145	0.29	7	5.28	0.42	40	u	0.425148
INTS8	1	integrator complex subunit 8	218905_at	1	8.361	0.23	7	9.649	0.75	40	u	0
FOLR1	1	folate receptor 1 (adult)	204437_s_at	1	8.876	0.34	7	5.77	2.46	40	d	0
			211074_at	0	9.805	0.29	7	9.854	0.83	40	u	0.78739
TMCC2	1	transmembrane and coiled-coil domain family 2	213096_at	1	5.381	0.32	7	5.81	0.85	40	u	0.201841
			1555503_a_at	0	3.992	0.24	7	4.228	0.66	40	u	0.113278
HACE1	1	HECT domain and ankyrin repeat containing, E3 ubiquitin protein ligase 1	227471_at	1	6.528	0.3	7	6.199	1.02	40	d	0.116384
SNRPF	1	small nuclear ribonucleoprotein polypeptide F	203832_at	1	9.272	0.36	7	9.467	0.68	40	u	0.471332
UNC84A	1	unc-84 homolog A (C. elegans)	212074_at	1	9.676	0.13	7	9.321	0.65	40	d	0.004059
			230210_at	0	2.568	0.2	7	2.686	0.25	40	u	0.252125
			206487_at	0	5.476	0.67	7	4.754	0.95	40	d	0.065731
SGIP1	1	SH3-domain GRB2-like (endophilin) interacting protein 1	223672_at	1	3.27	0.29	7	3.652	0.84	40	u	0.045559
ATG4B	1	ATG4 autophagy related 4 homolog B (S. cerevisiae)	204902_s_at	2	6.471	0.33	7	6.217	0.56	40	d	0.261657
			204903_x_at	2	7.653	0.15	7	7.452	0.74	40	d	0.136535
AQP4	1	aquaporin 4	226228_at	1	2.543	0.05	7	2.938	1.47	40	u	0.101551
			210067_at	0	2.493	0.04	7	2.75	0.88	40	u	0.076428
			210906_x_at	0	2.624	0.14	7	2.865	1.1	40	u	0.198716
			210068_s_at	0	2.111	0.02	7	2.443	1.26	40	u	0.107577
			210066_s_at	0	2.471	0.05	7	2.733	0.85	40	u	0.063177
HDAC7A	1	histone deacetylase 7A	217937_s_at	1	8.377	0.23	7	7.751	0.72	40	d	0.000256
			236326_at	0	2.2	0.02	7	2.274	0.11	40	u	0.000426
CCL7	1	chemokine (C-C motif) ligand 7	208075_s_at	1	4.416	0.18	7	5.04	1.14	40	u	0.002754
KRTAP17-1	1	keratin associated protein 17-1	234680_at	1	2.357	0.38	7	2.381	0.72	40	u	0.933605
DSC2	1	desmocollin 2	226817_at	1	9.51	0.35	7	9.585	1.3	40	u	0.76853
			204750_s_at	1	5.308	1.01	7	7.658	2.11	40	u	0.006966
			204751_x_at	1	7.596	0.61	7	7.979	1.77	40	u	0.317416
RPS13	1	ribosomal protein S13	200018_at	1	13.82	0.04	7	13.57	0.33	40	d	0.000035
OLFM1	1	olfactomedin 1	213131_at	1	6.348	0.57	7	5.049	2.41	40	d	0.006519
			210924_at	0	3.487	0.3	7	3.526	0.37	40	u	0.798852
			205591_at	0	4.681	0.66	7	4.062	1.62	40	d	0.335875
TMEM74	1	transmembrane protein 74	1552520_at	1	2.905	0.15	7	3.481	0.69	40	u	0.00005
			1552521_a_at	1	2.771	0.11	7	2.982	0.23	40	u	0.023386
			1555159_at	0	2.473	0.23	7	2.654	0.35	40	u	0.209535
C14orf138	1	chromosome 14 open reading frame 138	218940_at	1	6.951	0.2	7	6.862	0.8	40	d	0.566871
			237640_at	0	4.13	0.54	7	4.258	0.48	40	u	0.536723
APBA2BP	1	amyloid beta (A4) precursor protein-binding, family A, member 2 binding protein	210720_s_at	1	6.774	0.34	7	6.742	1.23	40	d	0.897451
			223954_x_at	0	6.932	0.18	7	6.975	0.72	40	u	0.756637
SNX10	1	sorting nexin 10	218404_at	1	6.779	0.4	7	8.104	1.13	40	u	0.000016
GABRG1	1	gamma-aminobutyric acid (GABA) A receptor, gamma 1	241805_at	1	4.267	0.81	7	4.019	0.43	40	d	0.489991
			1552943_at	1	2.098	0.03	7	2.372	0.64	40	u	0.011441
CROT	1	carnitine O-octanoyltransferase	204573_at	1	8.354	0.47	7	7.259	1.02	40	d	0.009129
			231102_at	0	3.34	0.42	7	3.836	0.88	40	u	0.159652
GLP1R	1	glucagon-like peptide 1 receptor	208400_at	1	3.744	0.18	7	3.686	0.28	40	d	0.612692
			208391_s_at	1	1.986	0.03	7	2.027	0.07	40	u	0.168211
			208401_s_at	1	6.52	0.2	7	6.446	0.39	40	d	0.634281
			211232_x_at	1	5.096	0.24	7	5.147	0.24	40	u	0.619522
			208390_s_at	1	5.433	0.33	7	5.605	0.38	40	u	0.27815
TRIM37	1	tripartite motif-containing 37	1554001_at	2	3.613	0.22	7	4.118	0.66	40	u	0.001101
			213009_s_at	1	8.073	0.27	7	8.881	0.7	40	u	0.005201
IREB2	1	iron-responsive element binding protein 2	214666_x_at	1	5.305	0.24	7	5.603	0.75	40	u	0.066567
			225892_at	0	7.855	0.17	7	7.928	0.68	40	u	0.575241
			1555476_at	0	3.612	0.62	7	3.599	0.51	40	d	0.954333
LASS5	1	LAG1 homolog, ceramide synthase 5	224951_at	1	8.781	0.23	7	8.384	0.61	40	d	0.102014
			239491_at	0	2.05	0.02	7	2.106	0.05	40	u	0.000024
PDCD7	1	programmed cell death 7	231809_x_at	1	8.241	0.25	7	7.972	0.45	40	d	0.134609
			230182_at	0	2.227	0.16	7	2.34	0.22	40	u	0.215876

PSMC6	1	proteasome (prosome, macropain) 26S subunit, ATPase, 6	201699_at	1	9.92	0.15	7	10.14	0.61	40	u	0.071287
PCNX	1	pecanex homolog (Drosophila)	229287_at	0	8.312	0.29	7	6.25	0.93	40	d	0
			213159_at	0	6.219	0.28	7	5.868	0.51	40	d	0.087139
			235082_at	0	3.086	0.17	7	3.13	0.37	40	u	0.763249
			238792_at	0	4.135	0.44	7	3.983	0.44	40	d	0.412928
			215175_at	0	6.175	1.21	7	4.788	0.9	40	d	0.001128
			213173_at	0	4.894	0.52	7	4.828	0.3	40	d	0.774578
SLC32A1	1	solute carrier family 32 (GABA vesicular transporter), member 1	240532_at	1	2.062	0.03	7	2.105	0.04	40	u	0.010268
RRAS2	1	related RAS viral (r-ras) oncogene homolog 2	212589_at	1	8.617	0.38	7	7.764	1.1	40	d	0.001259
			212590_at	1	7.541	0.55	7	7.018	1.26	40	d	0.297498
			208456_s_at	0	6.591	0.63	7	6.172	1.4	40	d	0.450697
LTBP2	1	latent transforming growth factor beta binding protein 2	223690_at	1	9.617	0.64	7	6.877	0.94	40	d	0
			204682_at	0	10.07	0.63	7	7.359	1.25	40	d	0.000002
SLC22A8	1	solute carrier family 22 (organic anion transporter), member 8	231352_at	1	3.341	0.48	7	3.484	0.6	40	u	0.562754
			221298_s_at	0	2.708	0.03	7	2.706	0.14	40	d	0.94166
FABP3	1	fatty acid binding protein 3, muscle and heart (mammary-derived growth inhibitor)	205738_s_at	1	4.403	0.35	7	4.289	0.89	40	d	0.745397
			214285_at	1	3.268	0.16	7	3.279	0.69	40	u	0.933876
CDC42SE1	1	CDC42 small effector 1	222537_s_at	1	6.99	0.34	7	7.646	0.72	40	u	0.025458
			223674_s_at	1	3.683	0.43	7	3.757	0.7	40	u	0.793269
			229120_s_at	1	9.034	0.25	7	9.177	0.55	40	u	0.509301
			218157_x_at	1	9.25	0.3	7	9.357	0.54	40	u	0.618239
MYPN	1	myopalladin	235367_at	1	3.282	0.39	7	3.66	0.37	40	u	0.020552
ANXA6	1	annexin A6	200982_s_at	1	9.821	0.47	7	9.494	0.88	40	d	0.350931
			244250_at	0	3.449	0.34	7	3.595	0.49	40	u	0.464712
EMID1	1	EMI domain containing 1	213779_at	1	6.672	0.56	7	6.717	0.76	40	u	0.884003
			1564251_at	0	2.029	0.04	7	2.099	0.12	40	u	0.012935
EIF2S3	1	eukaryotic translation initiation factor 2, subunit 3 gamma, 52kDa	224935_at	1	10.5	0.26	7	10.51	0.81	40	u	0.966658
			224936_at	1	10.48	0.21	7	10.24	0.67	40	d	0.092998
			205321_at	0	8.764	1.14	7	7.577	1.62	40	d	0.075557
ABTB2	1	ankyrin repeat and BTB (POZ) domain containing 2	213497_at	1	3.918	0.84	7	4.726	1.22	40	u	0.107273
			232624_at	0	1.893	0.05	7	1.927	0.07	40	u	0.258616
SOX30	1	SRY (sex determining region Y)-box 30	207678_s_at	1	2.662	0.06	7	2.899	0.47	40	u	0.004876
			1559235_a_at	0	2.071	0	7	2.137	0.06	40	u	0
PPHLN1	1	periphilin 1	227025_at	1	5.448	0.33	7	5.586	0.77	40	u	0.651629
			225073_at	0	9.809	0.25	7	10.01	0.49	40	u	0.308799
			1555350_at	0	3.071	0.34	7	3.262	0.43	40	u	0.283237
			1555351_s_at	0	5.293	0.39	7	5.536	0.36	40	u	0.121821
			234459_at	0	3.686	0.56	7	3.791	0.47	40	u	0.606304
CCDC32	1	coiled-coil domain containing 32	225397_at	1	7.274	0.45	7	6.939	0.8	40	d	0.298394
			1558648_at	1	4.002	0.39	7	4.217	0.62	40	u	0.392924
			1560814_a_at	0	4.245	1.21	7	3.88	1.06	40	d	0.426576
AKAP10	1	A kinase (PRKA) anchor protein 10	236007_at	0	6.197	0.41	7	6.094	0.85	40	d	0.758168
			236006_s_at	0	3.641	0.2	7	3.787	0.32	40	u	0.264691
			205045_at	0	5.073	0.5	7	5.018	0.57	40	d	0.81663
DGKZ	1	diacylglycerol kinase, zeta 104kDa	207556_s_at	1	8.636	0.35	7	8.666	0.83	40	u	0.928982
			239342_at	0	3.512	0.34	7	4.172	0.94	40	u	0.003741
MYH3	1	myosin, heavy chain 3, skeletal muscle, embryonic	205940_at	1	2.856	0.32	7	2.792	0.17	40	d	0.644336
SENP6	1	SUMO1/sentrin specific peptidase 6	202319_at	1	6.473	0.38	7	6.097	0.76	40	d	0.217682
			202318_s_at	1	9.196	0.21	7	9.063	0.54	40	d	0.533695
			214790_at	0	6.631	0.53	7	5.563	0.83	40	d	0.002502
RIF1	1	RAP1 interacting factor homolog (yeast)	226503_at	1	7.23	0.23	7	7.618	0.71	40	u	0.01357
			214700_x_at	1	6.512	0.51	7	7.17	0.72	40	u	0.029165
			233781_s_at	0	5.192	0.36	7	5.643	0.57	40	u	0.053325
			236620_at	0	7.491	0.27	7	8.447	0.7	40	u	0.001051
			233780_at	0	3.332	0.37	7	3.67	0.61	40	u	0.171592
			214872_at	0	2.623	0.13	7	2.734	0.28	40	u	0.318993
			241820_at	0	5.367	0.57	7	6.116	1.23	40	u	0.129905
			240098_at	0	3.169	0.27	7	3.367	0.58	40	u	0.393078
VEGFC	1	vascular endothelial growth factor C	209946_at	0	6.822	0.37	7	5.838	0.9	40	d	0.007985
EXOC3L2	1	exocyst complex component 3-like 2	1552889_a_at	1	6.877	0.24	7	7.184	0.38	40	u	0.05049
C21orf63	1	chromosome 21 open reading frame 63	227188_at	1	7.451	0.87	7	5.696	1.29	40	d	0.001485

PDGFB	1	platelet-derived growth factor beta polypeptide (simian sarcoma viral (v-sis) oncogene homolog)	204200_s_at	0	6.468	0.69	7	6.477	0.95	40	u	0.982618
			216055_at	0	2.309	0.01	7	2.372	0.21	40	u	0.074052
			216061_x_at	0	6.792	0.39	7	6.841	0.65	40	u	0.850926
			217112_at	0	1.996	0.02	7	2.035	0.07	40	u	0.007307
MPI	1	mannose phosphate isomerase	202472_at	1	5.828	0.24	7	4.986	0.67	40	d	0.000005
			230524_at	0	3.077	0.28	7	3.278	0.4	40	u	0.218455
SUMO3	1	SMT3 suppressor of mif two 3 homolog 3 (S. cerevisiae)	200740_s_at	1	10.51	0.26	7	11.1	0.56	40	u	0.010376
			200739_s_at	1	8.815	0.44	7	9.425	1.02	40	u	0.13265
HPS3	1	Hermansky-Pudlak syndrome 3	227139_s_at	1	7.862	0.39	7	8.616	0.69	40	u	0.008279
			238539_at	0	4.623	0.46	7	5.181	1.24	40	u	0.253891
			231121_at	0	3.307	0.41	7	4.165	0.8	40	u	0.009736
LDHA	1	lactate dehydrogenase A	200650_s_at	1	12.6	0.38	7	13.18	0.42	40	u	0.001778
RHEBL1	1	Ras homolog enriched in brain like 1	1553713_a_at	1	4.246	0.34	7	4.773	0.71	40	u	0.067095
			1570253_a_at	1	2.319	0.08	7	2.708	0.71	40	u	0.001951
MAN1C1	1	mannosidase, alpha, class 1C, member 1	218918_at	1	5.948	0.36	7	4.578	1.6	40	d	0.000037
			214180_at	0	4.538	0.45	7	4.264	0.9	40	d	0.44299
KLHDC6	1	kelch domain containing 6	244771_at	1	2.927	0.04	7	3.214	0.42	40	u	0.00014
C8orf55	1	chromosome 8 open reading frame 55	218500_at	1	7.192	0.18	7	7.419	1.19	40	u	0.272524
RCVRN	1	recoverin	206203_at	1	2.179	0.04	7	2.265	0.17	40	u	0.013268
SHANK1	1	SH3 and multiple ankyrin repeat domains 1	220563_s_at	1	2.273	0.1	7	2.422	0.28	40	u	0.022938
ST3GAL6	1	ST3 beta-galactoside alpha-2,3-sialyltransferase 6	210942_s_at	1	5.336	0.55	7	5.661	1.81	40	u	0.383054
			213335_s_at	1	3.698	0.23	7	3.812	0.48	40	u	0.554357
			213355_at	1	3.947	0.37	7	4.807	1.61	40	u	0.006505
ATN1	1	atrophin 1	40489_at	1	7.834	0.56	7	7.534	1.16	40	d	0.51671
			208871_at	1	7.155	0.62	7	6.611	1.22	40	d	0.26458
			229265_at	0	9.318	0.28	7	8.517	0.78	40	d	0.0001
			1555754_s_at	0	4.462	0.19	7	4.69	0.64	40	u	0.089197
			211076_x_at	0	2.58	0.26	7	2.661	0.31	40	u	0.525678
TSSK3	1	testis-specific serine kinase 3	228451_at	1	2.584	0.23	7	2.382	0.2	40	d	0.0223
			1552395_at	1	2.037	0.04	7	2.139	0.16	40	u	0.002351
			1556560_a_at	0	3.085	0.58	7	2.877	0.56	40	d	0.382888
DDX52	1	DEAD (Asp-Glu-Ala-Asp) box polypeptide 52	212834_at	1	4.774	0.37	7	5.138	1.01	40	u	0.359945
			210320_s_at	1	5.262	0.38	7	5.836	1.12	40	u	0.022352
			1570415_at	0	2.425	0.03	7	2.603	0.51	40	u	0.037099
NEK1	1	NIMA (never in mitosis gene a)-related kinase 1	213331_s_at	1	7.001	0.47	7	6.633	0.71	40	d	0.202023
			216213_at	1	1.78	0.03	7	1.854	0.25	40	u	0.07555
			213328_at	1	7.013	0.27	7	7.32	0.7	40	u	0.268016
			1564093_at	0	2.971	0.23	7	3.285	0.34	40	u	0.02487
			211086_x_at	0	4.388	0.6	7	4.677	0.72	40	u	0.331643
AGK	1	acylglycerol kinase	218568_at	1	5.947	0.43	7	6.252	0.77	40	u	0.323321
			222132_s_at	1	7.626	0.3	7	7.76	0.55	40	u	0.536235
			1555610_at	0	2.489	0.04	7	2.613	0.14	40	u	0.000107
MCCD1	1	mitochondrial coiled-coil domain 1	no probeset available									
ANKRD11	1	ankyrin repeat domain 11	238538_at	1	8.571	0.42	7	7.997	0.87	40	d	0.10026
			234701_at	0	5.64	0.55	7	5.575	1.04	40	d	0.87655
			219437_s_at	0	7.615	0.58	7	6.941	1.01	40	d	0.099946
			226012_at	0	7.807	0.15	7	7.207	0.47	40	d	0.000001
			228356_at	0	5.892	0.31	7	5.799	0.58	40	d	0.685033
			231999_at	0	5.98	0.18	7	5.959	0.66	40	d	0.874164
DUSP1	1	dual specificity phosphatase 1	201044_x_at	1	8.355	1.19	7	6.497	0.99	40	d	0.000078
			226578_s_at	1	2.998	0.62	7	2.839	0.52	40	d	0.483019
			201041_s_at	1	13.07	0.69	7	10.96	1.11	40	d	0.000019
EGFLAM	1	EGF-like, fibronectin type III and laminin G domains	226911_at	1	4.993	0.65	7	2.59	0.77	40	d	0
CCDC98	1	coiled-coil domain containing 98	226521_s_at	1	7.351	0.28	7	7.38	0.59	40	u	0.900521
			233543_s_at	0	4.585	0.53	7	4.925	0.73	40	u	0.253892
DGKB	1	diacylglycerol kinase, beta 90kDa	216307_at	1	2.664	0.11	7	2.906	0.34	40	u	0.001904
			1553524_at	0	2.996	0.08	7	3.252	0.37	40	u	0.000591
DPYS	1	dihydropyrimidinase	206065_s_at	1	2.556	0.05	7	2.584	0.07	40	u	0.352896
GCN5L2	1	GCN5 general control of amino-acid synthesis 5-like 2 (yeast)	202182_at	1	8.008	0.34	7	7.513	0.64	40	d	0.056295
MRC2	1	mannose receptor, C type 2	209280_at	1	7.472	0.36	7	6.826	0.63	40	d	0.01271
			37408_at	1	9.124	0.34	7	8.315	0.75	40	d	0.008794

GARNL3	1	GTPase activating Rap/RanGAP domain-like 3	223604_at<br>216165_at<br>1563907_at	1<br>0<br>0	5.128<br>3.099<br>2.274	0.69<br>0.33<br>0.13	7<br>7<br>7	4.491<br>3.136<br>2.32	0.83<br>0.36<br>0.18	40<br>40<br>40	d<br>u<br>u	0.068317<br>0.805407<br>0.52642
TGM3	1	transglutaminase 3 (E polypeptide, protein-glutamine-gamma-glutamyltransferase)	206004_at	1	2.24	0.14	7	2.629	0.78	40	u	0.006788
MTCH1	1	mitochondrial carrier homolog 1 (C. elegans)	221619_s_at	1	11.73	0.17	7	11.63	0.52	40	d	0.330536
FBXW9	1	F-box and WD repeat domain containing 9	223419_at	1	6.911	0.37	7	6.644	0.77	40	d	0.384369
SFMBT2	1	Scm-like with four mbt domains 2	232938_at	1	1.958	0.07	7	1.957	0.08	40	d	0.976539
AOF2	1	amine oxidase (flavin containing) domain 2	212348_s_at<br>1555897_at<br>238652_at	1<br>0<br>0	8.766<br>3.91<br>6.22	0.14<br>0.44<br>0.53	7<br>7<br>7	8.585<br>3.254<br>5.164	0.73<br>0.51<br>0.53	40<br>40<br>40	d<br>d<br>d	0.171065<br>0.003113<br>0.000023
SETD5	1	SET domain containing 5	221806_s_at<br>1569105_at<br>244691_at<br>222575_at<br>1569106_s_at	1<br>0<br>0<br>0<br>0	9.783<br>5.179<br>3.31<br>7.294<br>7.82	0.28<br>0.26<br>0.31<br>0.22<br>0.39	7<br>7<br>7<br>7<br>7	9.496<br>5.591<br>3.735<br>7.226<br>7.693	0.65<br>0.46<br>0.64<br>0.52<br>1.51	40<br>40<br>40<br>40<br>40	d<br>u<br>u<br>d<br>d	0.265137<br>0.030313<br>0.100064<br>0.737163<br>0.665321
EBF1	1	early B-cell factor 1	232204_at<br>233261_at	1<br>0	8.554<br>3.126	0.78<br>0.15	7<br>7	5.783<br>3.199	0.79<br>0.24	40<br>40	d<br>u	0<br>0.444058
MRPL35	1	mitochondrial ribosomal protein L35	222775_s_at<br>218890_x_at	0<br>0	6.813<br>7.57	0.55<br>0.31	7<br>7	7.058<br>8.426	0.63<br>0.62	40<br>40	u<br>u	0.350575<br>0.001022
C2orf24	1	chromosome 2 open reading frame 24	200070_at<br>207511_s_at	1<br>0	8.396<br>7.85	0.15<br>0.17	7<br>7	8.387<br>7.803	0.62<br>0.67	40<br>40	d<br>d	0.934777<br>0.716251
MYO18A	1	myosin XVIIIA	202039_at	1	8.245	0.39	7	7.271	1.07	40	d	0.000367
TUB	1	tubby homolog (mouse)	228883_at<br>228882_at<br>208431_s_at<br>210737_at	2<br>2<br>0<br>0	3.816<br>6.586<br>2.1<br>2.951	0.37<br>0.27<br>0.01<br>0.31	7<br>7<br>7<br>7	3.945<br>5.74<br>2.159<br>2.856	0.47<br>1.4<br>0.05<br>0.22	40<br>40<br>40<br>40	u<br>d<br>u<br>d	0.498772<br>0.001543<br>0.000001<br>0.345519
KCNK1	1	potassium channel, subfamily K, member 1	204679_at<br>204678_s_at	1<br>0	7.551<br>6.027	0.76<br>0.99	7<br>7	8.995<br>7.359	1.54<br>1.65	40<br>40	u<br>u	0.02178<br>0.048708
RUNDC3B	1	RUN domain containing 3B	215321_at<br>241703_at	1<br>1	4.099<br>4.52	0.58<br>0.64	7<br>7	3.49<br>3.305	0.46<br>0.95	40<br>40	d<br>d	0.004194<br>0.002652
POLR3E	1	polymerase (RNA) III (DNA directed) polypeptide E (80kD)	218016_s_at<br>233459_at<br>233458_at<br>222490_at	1<br>0<br>0<br>0	9.063<br>1.808<br>4.03<br>7.442	0.27<br>0.02<br>0.67<br>0.31	7<br>7<br>7<br>7	8.82<br>1.855<br>3.806<br>7.172	0.63<br>0.1<br>0.74<br>0.79	40<br>40<br>40<br>40	d<br>u<br>d<br>d	0.32665<br>0.012706<br>0.466008<br>0.384563
IPMK	1	inositol polyphosphate multikinase	239878_at	1	3.808	0.46	7	4.171	0.55	40	u	0.11277
CDCA7	1	cell division cycle associated 7	224428_s_at<br>230060_at	1<br>0	5.522<br>3.021	0.73<br>0.08	7<br>7	7.725<br>3.752	1.75<br>0.89	40<br>40	u<br>u	0.002412<br>0.000011
GRHL3	1	grainyhead-like 3 (Drosophila)	232116_at	1	2.221	0.25	7	2.782	1.03	40	u	0.006322
C14orf166	1	chromosome 14 open reading frame 166	217768_at	1	11.31	0.19	7	11.22	0.51	40	d	0.634048
OVOL2	1	ovo-like 2 (Drosophila)	206048_at<br>211778_s_at	1<br>1	5.971<br>7.759	0.52<br>0.34	7<br>7	5.755<br>7.409	0.62<br>0.96	40<br>40	d<br>d	0.399036<br>0.104761
CASP7	1	caspase 7, apoptosis-related cysteine peptidase	207181_s_at	1	7.544	0.28	7	7.782	0.72	40	u	0.403493
CSE1L	1	CSE1 chromosome segregation 1-like (yeast)	201111_at<br>201112_s_at<br>210766_s_at<br>210765_at	1<br>0<br>0<br>0	8.618<br>9.797<br>8.786<br>4.978	0.15<br>0.14<br>0.2<br>0.54	7<br>7<br>7<br>7	9.785<br>10.71<br>9.826<br>5.416	0.72<br>0.71<br>0.69<br>0.64	40<br>40<br>40<br>40	u<br>u<br>u<br>u	0<br>0<br>0<br>0.100377
KCNJ10	1	potassium inwardly-rectifying channel, subfamily J, member 10	228581_at<br>206692_at	1<br>0	3.399<br>1.942	0.18<br>0.03	7<br>7	3.654<br>2.01	0.44<br>0.19	40<br>40	u<br>u	0.145094<br>0.039966
LENG8	1	leukocyte receptor cluster (LRC) member 8	224673_at	1	9.529	0.58	7	7.948	1.11	40	d	0.000751
LACE1	1	lactation elevated 1	1552490_at<br>1563671_at	1<br>0	2.673<br>2.378	0.26<br>0.03	7<br>7	2.924<br>2.507	0.47<br>0.19	40<br>40	u<br>u	0.18458<br>0.000432
KIAA1274	1	KIAA1274	231887_s_at	1	5.96	0.38	7	5.223	0.69	40	d	0.010213
C4orf33	1	chromosome 4 open reading frame 33	1552372_at<br>1552373_s_at<br>1552370_at	1<br>1<br>1	2.978<br>2.697<br>6.404	0.12<br>0.07<br>0.46	7<br>7<br>7	3.163<br>2.895<br>5.976	0.23<br>0.25<br>0.75	40<br>40<br>40	u<br>u<br>d	0.046031<br>0.000358<br>0.161185
DOK6	1	docking protein 6	236290_at<br>241729_at	1<br>0	3.213<br>2.232	0.23<br>0.17	7<br>7	3.374<br>2.292	0.44<br>0.37	40<br>40	u<br>u	0.363636<br>0.680973
SLC35B3	1	solute carrier family 35, member B3	222691_at<br>231003_at	1<br>0	8.832<br>4.838	0.34<br>0.61	7<br>7	9.123<br>4.543	0.74<br>0.64	40<br>40	u<br>d	0.323236<br>0.271236
G0S2	1	G0/G1switch 2	213524_s_at	1	9.81	1.79	7	7.289	1.24	40	d	0.000048

PFKM	1	phosphofructokinase, muscle	210976_s_at	1	8.74	0.11	7	8.161	0.99	40	d	0.001074
SLC6A20	1	solute carrier family 6 (proline IMINO transporter), member 20	219614_s_at	1	2.535	0.08	7	2.624	0.22	40	u	0.305334
			234291_s_at	0	3.115	0.32	7	3.162	0.39	40	u	0.771109
STAT6	1	signal transducer and activator of transcription 6, interleukin-4 induced	201331_s_at	1	10.24	0.22	7	9.277	0.81	40	d	0.000001
			201332_s_at	0	7.009	0.26	7	6.067	1.15	40	d	0.000069
GNL1	1	guanine nucleotide binding protein-like 1	203307_at	0	8.391	0.17	7	7.869	0.42	40	d	0.002495
			233544_at	0	2.583	0.11	7	2.675	0.25	40	u	0.364801
PER3	1	period homolog 3 (Drosophila)	221045_s_at	1	7.555	0.57	7	6.383	0.65	40	d	0.000068
			1555130_at	0	3.591	0.34	7	3.892	0.34	40	u	0.041634
			1569701_at	0	3.295	1.2	7	3.297	0.8	40	u	0.996171
			1555131_a_at	0	3.338	0.76	7	3.353	0.93	40	u	0.968848
TRIAP1	1	TP53 regulated inhibitor of apoptosis 1	218403_at	1	9.021	0.17	7	8.922	0.59	40	d	0.402388
ADCY8	1	adenylate cyclase 8 (brain)	206811_at	1	2.656	0.14	7	2.779	0.41	40	u	0.166259
TTC7A	1	tetratricopeptide repeat domain 7A	224912_at	1	6.756	0.23	7	6.501	0.68	40	d	0.084452
			224923_at	1	6.538	0.21	7	6.465	0.63	40	d	0.591756
			224924_at	1	2.439	0.41	7	2.699	0.75	40	u	0.389385
			240369_at	0	2.226	0.07	7	2.325	0.23	40	u	0.041748
ELP3	1	elongation protein 3 homolog (S. cerevisiae)	227075_at	1	7.453	0.38	7	6.679	0.82	40	d	0.020313
			221094_s_at	0	7.58	0.31	7	7.406	0.64	40	d	0.489882
C2orf58	1	chromosome 2 open reading frame 58	1553829_at	1	2.179	0.08	7	2.146	0.07	40	d	0.27165
			233410_at	0	2.773	0.32	7	2.974	0.41	40	u	0.231797
			233285_at	0	3.357	0.23	7	3.747	0.41	40	u	0.019516
C8orf34	1	chromosome 8 open reading frame 34	231380_at	1	2.767	0.29	7	2.765	0.31	40	d	0.989183
			1562301_at	0	3.087	0.1	7	3.349	0.31	40	u	0.000347
KERA	1	keratocan	220504_at	1	3.802	0.64	7	3.178	0.41	40	d	0.001772
SLC35C2	1	solute carrier family 35, member C2	225037_at	1	8.464	0.27	7	8.334	0.63	40	d	0.603565
			219447_s_at	0	7.218	0.2	7	7.381	0.45	40	u	0.361683
FRMPD2	1	FERM and PDZ domain containing 2	239275_at	2	2.163	0.16	7	2.183	0.15	40	u	0.74891
MUC4	1	mucin 4, cell surface associated	217109_at	1	3.61	1.27	7	3.322	0.53	40	d	0.604552
			204895_x_at	1	2.512	0.78	7	2.227	0.05	40	d	0.405299
			217110_s_at	1	2.491	0.62	7	2.316	0.12	40	d	0.520378
			1561785_at	0	4.08	0.49	7	4.147	0.62	40	u	0.791397
			235055_x_at	0	6.526	0.49	7	6.411	0.49	40	d	0.575102
ABLIM2	1	actin binding LIM protein family, member 2	236087_at	1	5.179	0.31	7	4.953	0.43	40	d	0.19637
			228132_at	1	4.888	0.35	7	4.881	0.82	40	d	0.981802
			1567622_at	0	3.469	0.16	7	3.72	0.36	40	u	0.08454
			242624_at	0	2.4	0.05	7	2.511	0.22	40	u	0.008816
			1567623_at	0	3.258	0.25	7	3.542	0.52	40	u	0.170611
PLEKHQ1	1	pleckstrin homology domain containing, family Q member 1	204436_at	1	7.566	0.66	7	7.18	0.59	40	d	0.131859
CCDC130	1	coiled-coil domain containing 130	208094_s_at	1	7.874	0.26	7	7.373	0.67	40	d	0.063947
C2orf7	1	chromosome 2 open reading frame 7	223312_at	1	6.793	0.29	7	6.88	0.79	40	u	0.617054
KLHL25	1	kelch-like 25 (Drosophila)	210307_s_at	0	5.147	0.44	7	5.268	0.64	40	u	0.639045
HCN3	1	hyperpolarization activated cyclic nucleotide-gated potassium channel 3	228741_s_at	1	4.446	0.3	7	4.509	0.46	40	u	0.730607
SOAT1	1	sterol O-acyltransferase (acyl-Coenzyme A: cholesterol acyltransferase) 1	221561_at	1	3.811	0.39	7	6.012	0.94	40	u	0
C10orf130	1	chromosome 10 open reading frame 130	243534_at	1	3.259	0.27	7	3.161	0.35	40	d	0.495563
LASP1	1	LIM and SH3 protein 1	200618_at	1	11.4	0.45	7	11.03	1	40	d	0.344545
GDPD1	1	glycerophosphodiester phosphodiesterase domain containing 1	1560227_at	1	2.889	0.22	7	3.053	0.23	40	u	0.097819
			238681_at	0	5.168	0.55	7	5.679	0.95	40	u	0.18311
			1555606_a_at	0	2.883	0.41	7	3.473	0.77	40	u	0.057499
SHMT1	1	serine hydroxymethyltransferase 1 (soluble)	224954_at	1	8.155	0.15	7	8.212	0.47	40	u	0.556131
			217304_at	0	2.768	0.28	7	2.682	0.37	40	d	0.572885
			209980_s_at	0	6.723	0.44	7	6.182	0.84	40	d	0.109451
			231701_s_at	0	2.752	0.2	7	2.948	0.36	40	u	0.176872
POPDC2	1	popeye domain containing 2	219647_at	1	3.698	0.72	7	2.677	0.44	40	d	0.000011
C1orf113	1	chromosome 1 open reading frame 113	220193_at	0	6.401	0.24	7	5.723	0.48	40	d	0.000851
KLK15	1	kallikrein-related peptidase 15	233477_at	1	2.327	0.02	7	2.389	0.04	40	u	0.000429
			221462_x_at	0	2.404	0.3	7	2.337	0.08	40	d	0.60109
			234495_at	0	6.948	0.16	7	6.743	0.46	40	d	0.050334
			234966_at	0	3.176	0.61	7	3.059	0.41	40	d	0.536881
GABPA	1	GA binding protein transcription factor, alpha subunit 60kDa	227428_at	1	7.398	0.62	7	7.419	0.56	40	u	0.930756
			210188_at	0	4.112	0.71	7	5.27	1.11	40	u	0.012466

POLB	1	polymerase (DNA directed), beta	203616_at	1	9.081	0.32	7	9.625	0.92	40	u	0.0114
C3orf27	1	chromosome 3 open reading frame 27	208440_at	1	2.344	0.12	7	2.468	0.2	40	u	0.12994
SNX7	1	sorting nexin 7	205573_s_at	1	8.577	0.41	7	8.362	1.35	40	d	0.437732
SEC31A	1	SEC31 homolog A (S. cerevisiae)	210616_s_at	1	10.49	0.16	7	10.49	0.47	40	d	0.976355
			200945_s_at	1	10.61	0.22	7	10.59	0.5	40	d	0.924992
			215009_s_at	0	8.232	0.17	7	8.432	0.83	40	u	0.191208
TOR1B	1	torsin family 1, member B (torsin B)	209593_s_at	1	7.403	0.2	7	7.565	0.64	40	u	0.229534
MTX3	1	metaxin 3	226528_at	1	7.382	0.27	7	6.358	0.95	40	d	0.000005
			241779_at	0	5.398	0.18	7	5.522	0.43	40	u	0.461295
C1QL4	1	›mplement component 1, q subcomponent-like	no probeset available									
CASP3	1	caspase 3, apoptosis-related cysteine peptidase	202763_at	1	6.98	0.28	7	7.724	0.82	40	u	0.000221
			236729_at	0	2.766	0.11	7	2.891	0.13	40	u	0.026387
ITGB3BP	1	integrin beta 3 binding protein (beta3-endonexin)	205176_s_at	0	7.76	0.77	7	7.708	0.74	40	d	0.868813
CCRL2	1	chemokine (C-C motif) receptor-like 2	211434_s_at	2	3.324	0.67	7	3.682	0.75	40	u	0.253755
C1orf19	1	chromosome 1 open reading frame 19	225400_at	1	5.618	0.46	7	6.712	1.13	40	u	0.017028
			225399_at	1	7.537	0.3	7	8.475	0.87	40	u	0.000037
			230257_s_at	0	6.468	0.36	7	7.362	0.74	40	u	0.00372
MRPL43	1	mitochondrial ribosomal protein L43	224332_s_at	0	8.657	0.37	7	8.451	0.54	40	d	0.343382
			230026_at	0	6.431	1.12	7	5.626	0.94	40	d	0.053821
			230027_s_at	0	8.313	0.29	7	7.296	1.12	40	d	0.000035
ACE	1	angiotensin I converting enzyme (peptidyl-dipeptidase A) 1	209749_s_at	1	3.982	0.5	7	3.998	0.8	40	u	0.958679
			227463_at	0	3.163	0.38	7	2.989	0.71	40	d	0.538694
ZNF213	1	zinc finger protein 213	227207_x_at	1	7.519	0.32	7	7.302	0.71	40	d	0.441479
EML2	1	echinoderm microtubule associated protein like 2	204398_s_at	1	7.126	0.4	7	7.016	0.75	40	d	0.714583
			204399_s_at	1	3.324	0.56	7	3.42	0.87	40	u	0.782873
			204397_at	1	2.322	0.21	7	2.426	0.29	40	u	0.377429
			234021_at	0	6.571	0.35	7	6.271	0.36	40	d	0.049609
			234026_at	0	5.315	0.35	7	5.301	0.4	40	d	0.930541
			1569086_at	0	2.893	0.08	7	2.982	0.18	40	u	0.217461
DTD1	1	D-tyrosyl-tRNA deacylase 1 homolog (S. cerevisiae)	234107_s_at	1	9.053	0.3	7	9.124	0.71	40	u	0.799466
			1552718_at	0	2.617	0.1	7	2.698	0.31	40	u	0.220794
RPL34P1	1	ribosomal protein L34 pseudogene 1	no probeset available									
GFRA3	1	GDNF family receptor alpha 3	229936_at	1	2.573	0.32	7	3.625	1.44	40	u	0.000309
			214479_at	0	2.575	0.03	7	2.69	0.37	40	u	0.064038
CACNA1D	1	calcium channel, voltage-dependent, L type, alpha 1D subunit	207998_s_at	1	3.399	0.36	7	3.484	0.74	40	u	0.771824
DUSP19	1	dual specificity phosphatase 19	1552708_a_at	1	3.001	0.26	7	3.151	0.36	40	u	0.308053
			1552705_at	1	3.416	0.23	7	3.631	0.36	40	u	0.137331
			1555598_a_at	0	2.581	0.21	7	2.765	0.3	40	u	0.139435
PACRG	1	PARK2 co-regulated	214204_at	1	2.252	0.74	7	1.976	0.07	40	d	0.397149
			215472_at	0	2.785	0.25	7	2.836	0.34	40	u	0.711171
GRB10	1	growth factor receptor-bound protein 10	209409_at	1	9.394	0.49	7	8.436	1.06	40	d	0.026469
			215248_at	0	7.191	0.71	7	6.162	1.1	40	d	0.024025
			210999_s_at	0	7.539	0.26	7	7.525	0.98	40	d	0.94458
			209410_s_at	0	6.473	0.5	7	6.628	1.07	40	u	0.716292
CLSTN1	1	calsyntenin 1	201561_s_at	1	10.05	0.4	7	9.059	0.86	40	d	0.005421
RFWD3	1	ring finger and WD repeat domain 3	218564_at	1	5.06	0.57	7	6.377	0.97	40	u	0.001415
EDIL3	1	EGF-like repeats and discoidin I-like domains 3	225275_at	1	8.302	0.53	7	8.085	1.81	40	d	0.553422
			1558643_s_at	0	3.032	0.38	7	3.492	1.39	40	u	0.100494
			207379_at	0	3.456	0.64	7	4.213	1.49	40	u	0.202407
DSC3	1	desmocollin 3	206033_s_at	0	6.564	0.69	7	4.918	2.3	40	d	0.001322
			206032_at	0	5.505	0.77	7	5.045	2	40	d	0.558967
SAP130	1	Sin3A-associated protein, 130kDa	220367_s_at	1	6.764	0.48	7	6.263	0.87	40	d	0.15408
TMEM65	1	transmembrane protein 65	241342_at	0	6.941	0.45	7	8.449	0.95	40	u	0.000211
			238045_at	0	5.018	0.23	7	6.137	1	40	u	0.000001
PCDH1	1	protocadherin 1	215277_at	1	2.724	0.38	7	2.968	0.54	40	u	0.268211
			203918_at	0	7.587	0.17	7	7.406	0.76	40	d	0.19977
CD59	1	CD59 molecule, complement regulatory protein	228748_at	1	6.491	0.63	7	5.256	1.3	40	d	0.020036
			200984_s_at	0	11.76	0.3	7	10.81	1.17	40	d	0.000147
			200985_s_at	0	12.28	0.27	7	11.27	1.07	40	d	0.000019
			200983_x_at	0	12.04	0.33	7	11.33	1.21	40	d	0.004564
			212463_at	0	11.14	0.26	7	9.557	1.11	40	d	0
		cyclin-dependent kinase 9 (CDC2-related	203198_at	1	7.36	0.2	7	6.732	0.88	40	d	0.000407

CDK9	1	cyclin-dependent kinase 9 (CDC2-related kinase)	236023_at	1	3.974	0.41	7	3.482	0.5	40	d	0.019783
			228901_at	0	2.336	0.02	7	2.405	0.07	40	u	0.000057
ARHGAP18	1	Rho GTPase activating protein 18	225173_at	1	7.794	0.33	7	8.617	0.83	40	u	0.014807
			225166_at	1	4.291	0.62	7	3.848	0.76	40	d	0.160289
			225171_at	1	8.184	0.39	7	8.306	0.65	40	u	0.640393
TTR	1	transthyretin (prealbumin, amyloidosis type I)	209660_at	0	3.891	0.37	7	3.855	0.5	40	d	0.862777
NUS1	1	nuclear undecaprenyl pyrophosphate synthase 1 homolog (S. cerevisiae)	225070_at	1	8.709	0.37	7	8.766	1.13	40	u	0.811448
			225071_at	1	7.669	0.25	7	8.499	1.04	40	u	0.000142
UVRAG	1	UV radiation resistance associated gene	203241_at	1	7.96	0.26	7	7.914	0.68	40	d	0.864608
C3orf63	1	chromosome 3 open reading frame 63	209285_s_at	1	9.023	0.28	7	8.029	0.74	40	d	0.001232
			209284_s_at	1	7.595	0.31	7	7.807	0.67	40	u	0.423327
			227460_at	0	2.378	0.12	7	2.443	0.28	40	u	0.561549
C5orf25	1	chromosome 5 open reading frame 25	228805_at	1	6.125	0.26	7	6.573	0.97	40	u	0.023867
LYN	1	v-yes-1 Yamaguchi sarcoma viral related oncogene homolog	202626_s_at	1	7.198	0.85	7	7.398	1.48	40	u	0.735021
			202625_at	1	6.658	0.71	7	7.135	1.25	40	u	0.342017
			210754_s_at	0	7.319	0.71	7	7.599	1.47	40	u	0.632688
TIMM9	1	translocase of inner mitochondrial membrane 9 homolog (yeast)	218316_at	1	7.473	0.25	7	7.375	0.55	40	d	0.653719
DNASE2	1	deoxyribonuclease II, lysosomal	209831_x_at	0	6.993	0.27	7	7.488	0.61	40	u	0.043797
			214992_s_at	0	6.348	0.38	7	6.835	1.13	40	u	0.053356
MGAT2	1	mannosyl (alpha-1,6-)-glycoprotein beta-1,2-N-acetylglucosaminyltransferase	203102_s_at	1	8.305	0.22	7	8.71	0.74	40	u	0.011053
			203101_s_at	1	4.112	0.25	7	3.939	0.67	40	d	0.510158
			211061_s_at	0	7.407	0.32	7	7.458	1.05	40	u	0.813607
SUMO2	1	SMT3 suppressor of mif two 3 homolog 2 (S. cerevisiae)	213881_x_at	1	12.67	0.1	7	12.96	0.4	40	u	0.000403
			208739_x_at	1	11.63	0.16	7	11.68	0.88	40	u	0.744281
			208738_x_at	0	11.27	0.14	7	11.79	0.48	40	u	0.000004
NUDT11	1	nudix (nucleoside diphosphate linked moiety X)-type motif 11	219855_at	1	5.34	0.63	7	4.846	1.42	40	d	0.379883
MTA3	1	metastasis associated 1 family, member 3	223311_s_at	1	7.537	0.37	7	7.902	0.54	40	u	0.101233
			226852_at	0	5.911	0.36	7	5.429	1.08	40	d	0.043887
SLC16A6	1	solute carrier family 16, member 6 (monocarboxylic acid transporter 7)	207038_at	1	5.902	0.48	7	5.72	1.94	40	d	0.623158
			230748_at	0	6.813	0.8	7	6.249	2.29	40	d	0.260776
IL9R	1	interleukin 9 receptor	214950_at	2	2.541	0.26	7	2.964	0.52	40	u	0.043105
			208164_s_at	0	2.523	0.28	7	2.478	0.26	40	d	0.682989
			217212_s_at	0	4.253	0.32	7	4.327	0.55	40	u	0.739784
NLN	1	neurolysin (metallopeptidase M3 family)	225943_at	1	5.803	0.35	7	6.25	1.06	40	u	0.054474
			225944_at	1	6.507	0.37	7	7.253	0.54	40	u	0.001175
			225942_at	1	4.764	0.6	7	4.917	0.64	40	u	0.565504
			224063_at	0	2.881	0.16	7	3.069	0.26	40	u	0.078593
			234762_x_at	0	9.301	0.35	7	8.987	0.82	40	d	0.33337
UTS2	1	urotensin 2	220785_at	1	2.276	0.05	7	2.385	0.19	40	u	0.004311
			220784_s_at	1	2.931	0.3	7	3.046	0.33	40	u	0.398198
FRMPD2L1	1	FERM and PDZ domain containing 2 like 1	no probeset available									
TOX4	1	TOX high mobility group box family member 4	201684_s_at	1	8.661	0.25	7	8.559	0.45	40	d	0.570927
			201685_s_at	1	7.816	0.44	7	7.11	0.6	40	d	0.005352
			201683_x_at	1	7.204	0.33	7	7.443	0.53	40	u	0.262731
			217448_s_at	0	5.575	0.49	7	5.373	0.63	40	d	0.432012
			232097_at	0	6.709	0.56	7	6.193	0.71	40	d	0.081896
HCCS	1	holocytochrome c synthase (cytochrome c heme-lyase)	203745_at	1	6.572	0.41	7	6.719	0.67	40	u	0.585816
			203746_s_at	0	7.19	0.47	7	7.755	0.83	40	u	0.09357
LSM5	1	LSM5 homolog, U6 small nuclear RNA associated (S. cerevisiae)	202903_at	1	4.989	0.51	7	5.501	0.97	40	u	0.187788
			211747_s_at	1	10.04	0.22	7	10.61	0.66	40	u	0.0004
			202904_s_at	1	5.606	0.23	7	6.695	0.88	40	u	0
ZBTB1	1	zinc finger and BTB domain containing 1	205092_x_at	1	4.057	0.42	7	4.665	0.73	40	u	0.040785
			1557036_at	0	3.916	0.35	7	4.308	0.6	40	u	0.108729
			213376_at	0	8.893	0.22	7	8.502	0.55	40	d	0.075618
CXorf57	1	chromosome X open reading frame 57	219355_at	1	2.902	0.49	7	3.086	1.25	40	u	0.709119
			215803_at	0	2.602	0.06	7	2.699	0.13	40	u	0.062908
IRF2BP1	1	interferon regulatory factor 2 binding protein 1	213771_at	1	7.687	0.32	7	7.545	0.64	40	d	0.578357
NDST2	1	N-deacetylase/N-sulfotransferase (heparan glucosaminyl) 2	203916_at	1	6.323	0.38	7	5.625	0.81	40	d	0.03464
			1559701_s_at	0	2.301	0.09	7	2.449	0.22	40	u	0.09376
AP1G2	1	adaptor-related protein complex 1, gamma 2	214341_at	1	3.698	0.13	7	3.337	0.34	40	d	0.009293

[illegible]	1	subunit	201613_s_at	0	8.789	0.15	7	7.86	1.05	40	d	0.000005
ERBB3	1	v-erb-b2 erythroblastic leukemia viral oncogene homolog 3 (avian)	202454_s_at	1	10.3	0.58	7	9.397	1.34	40	d	0.094732
			226213_at	1	11.01	0.53	7	10.34	1.39	40	d	0.225762
			1563252_at	0	4.188	0.5	7	3.842	0.53	40	d	0.122212
			215638_at	0	2.741	0.52	7	2.768	0.52	40	u	0.902443
			1563253_s_at	0	6.403	0.59	7	6.585	1.21	40	u	0.703906
USP21	1	ubiquitin specific peptidase 21	218367_x_at	1	8.2	0.3	7	8.134	0.82	40	d	0.716821
			234735_s_at	1	7.616	0.28	7	7.496	0.93	40	d	0.528222
			232219_x_at	1	7.931	0.23	7	7.867	0.62	40	d	0.793137
ATP6V1H	1	ATPase, H+ transporting, lysosomal 50/57kDa, V1 subunit H	221504_s_at	1	8.881	0.4	7	8.782	0.99	40	d	0.79897
			1557585_at	0	4.052	0.43	7	4.034	0.71	40	d	0.949305
			1557586_s_at	0	5.452	0.49	7	5.421	1.13	40	d	0.945293
FGA	1	fibrinogen alpha chain	231671_at	1	2.51	0.15	7	2.668	0.21	40	u	0.068018
			205649_s_at	1	2.526	0.04	7	2.746	0.32	40	u	0.000168
			205650_s_at	0	2.558	0.12	7	3.029	0.79	40	u	0.001177
ORMDL2	1	ORM1-like 2 (S. cerevisiae)	218556_at	0	8.778	0.4	7	8.9	0.78	40	u	0.694682
DHX57	1	DEAH (Asp-Glu-Ala-Asp/His) box polypeptide 57	213420_at	1	6.249	0.32	7	6.226	0.6	40	d	0.92224
			244784_at	0	3.295	0.14	7	3.551	0.37	40	u	0.079702
KIFC2	1	kinesin family member C2	226791_at	1	5.883	0.45	7	7.331	1.32	40	u	0.000023
			226792_s_at	1	8.124	0.13	7	8.572	0.82	40	u	0.002748
PTPN21	1	protein tyrosine phosphatase, non-receptor type 21	40524_at	1	3.199	0.62	7	2.891	0.34	40	d	0.279226
			205438_at	0	5.073	0.57	7	4.296	0.54	40	d	0.001366
			222092_at	0	1.957	0.04	7	1.984	0.05	40	u	0.161293
			1320_at	0	2.736	0.31	7	2.71	0.16	40	d	0.84967
IFNG	1	interferon, gamma	210354_at	0	2.627	0.15	7	2.831	0.41	40	u	0.032913
C14orf1	1	chromosome 14 open reading frame 1	202563_at	1	4.575	0.53	7	4.724	0.52	40	u	0.499079
			202562_s_at	1	8.659	0.35	7	8.214	0.67	40	d	0.102149
			217188_s_at	0	7.672	0.44	7	6.853	1.47	40	d	0.009822
ENTPD4	1	ectonucleoside triphosphate diphosphohydrolase 4	204076_at	1	8.695	0.29	7	8.143	0.91	40	d	0.00667
			204077_x_at	1	4.99	0.4	7	4.788	0.68	40	d	0.456754
			1555358_a_at	0	3.885	0.37	7	4.384	0.57	40	u	0.033314
PUS7	1	pseudouridylate synthase 7 homolog (S. cerevisiae)	218984_at	1	7.426	0.26	7	8.426	0.85	40	u	0.000002
C1orf2	1	chromosome 1 open reading frame 2	203550_s_at	1	7.862	0.28	7	8.436	0.92	40	u	0.004628
			1555515_a_at	1	2.639	0.16	7	3.509	1.3	40	u	0.000251
LARP2	1	La ribonucleoprotein domain family, member 2	226750_at	1	6.163	0.5	7	6.557	0.82	40	u	0.235157
			231824_at	0	4.215	0.47	7	4.682	0.64	40	u	0.076833
			231011_at	0	4.181	0.63	7	5.051	0.84	40	u	0.014031
			233764_s_at	0	4.006	0.23	7	4.4	0.47	40	u	0.039926
			1554038_at	0	2.533	0.18	7	2.79	0.38	40	u	0.095427
SLC7A9	1	solute carrier family 7 (cationic amino acid transporter, y+ system), member 9	220135_s_at	1	2.964	0.62	7	3.005	0.61	40	u	0.873512
FXYD7	1	FXYD domain containing ion transport regulator 7	220131_at	1	2.338	0.16	7	2.306	0.2	40	d	0.692006
MED1	1	mediator complex subunit 1	203497_at	1	8.421	0.36	7	8.884	1.51	40	u	0.110628
			225452_at	0	6.293	0.65	7	7.635	1.55	40	u	0.032181
			203496_s_at	0	5.389	0.33	7	6.424	1.75	40	u	0.001867
			225456_at	0	7.081	0.15	7	7.221	1.54	40	u	0.582832
PABPC4	1	poly(A) binding protein, cytoplasmic 4 (inducible form)	201064_s_at	0	9.73	0.21	7	9.389	0.64	40	d	0.017185
ING4	1	inhibitor of growth family, member 4	218234_at	1	7.542	0.21	7	6.936	0.73	40	d	0.000213
			48825_at	1	7.216	0.28	7	6.974	0.79	40	d	0.169828
C4orf17	1	chromosome 4 open reading frame 17	223990_at	1	4.141	0.36	7	4.168	0.59	40	u	0.907608
KCTD6	1	potassium channel tetramerisation domain containing 6	238077_at	1	7.865	0.44	7	7.66	1.68	40	d	0.529439
			238001_at	0	5.111	0.69	7	4.739	1.75	40	d	0.589796
ZNF313	1	zinc finger protein 313	200867_at	1	8.121	0.29	7	8.309	0.67	40	u	0.477334
			200868_s_at	0	8.722	0.29	7	8.983	0.84	40	u	0.161447
			211678_s_at	0	8.972	0.21	7	9.346	0.5	40	u	0.064355
RRM2	1	ribonucleotide reductase M2 polypeptide	201890_at	1	4.944	1.06	7	9.865	0.97	40	u	0
			209773_s_at	1	3.68	1.24	7	8.94	1.16	40	u	0
GPSM3	1	G-protein signaling modulator 3 (AGS3-like, C. elegans)	204265_s_at	0	6.76	0.29	7	7.083	0.75	40	u	0.279026
			214847_s_at	0	5.733	0.33	7	5.663	0.98	40	d	0.735312
KLK5	1	kallikrein-related peptidase 5	222242_s_at	0	8.766	0.84	7	5.075	2.7	40	d	0
SPTB	1	spectrin, beta, erythrocytic (includes	214145_s_at	1	5.239	0.16	7	5.233	0.37	40	d	0.967801

SPTB	1	spherocytosis, clinical type I)	208416_s_at	0	2.515	0.17	7	2.635	0.42	40	u	0.469675
SF3B1	1	splicing factor 3b, subunit 1, 155kDa	201071_x_at	1	12.08	0.17	7	11.52	0.43	40	d	0.001685
			211185_s_at	1	11.3	0.11	7	11.04	0.46	40	d	0.004025
			201070_x_at	1	8.648	0.41	7	9.318	0.5	40	u	0.002067
			214305_s_at	0	8.867	0.43	7	9.654	0.51	40	u	0.000473
NMNAT3	1	nicotinamide nucleotide adenylyltransferase 3	228090_at	1	6.224	0.62	7	5.657	0.8	40	d	0.086299
			243738_at	0	5.957	0.33	7	5.656	0.33	40	d	0.034134
TSG101	1	tumor susceptibility gene 101	201758_at	1	9.652	0.21	7	9.39	0.65	40	d	0.061453
HPRT1	1	hypoxanthine phosphoribosyltransferase 1 (Lesch-Nyhan syndrome)	202854_at	1	8.601	0.37	7	9.594	0.93	40	u	0.00885
			1565446_at	0	3.292	0.09	7	3.629	0.45	40	u	0.000163
FANCI	1	Fanconi anemia, complementation group I	213008_at	1	4.73	0.43	7	7.268	0.91	40	u	0
			213007_at	1	5.325	0.62	7	7.947	0.99	40	u	0
			223785_at	0	3.185	0.15	7	5.2	1.31	40	u	0
RBM22	1	RNA binding motif protein 22	224068_x_at	1	8.788	0.33	7	8.193	0.62	40	d	0.019241
			218134_s_at	1	8.743	0.34	7	8.344	0.62	40	d	0.113384
			222527_s_at	1	7.249	0.41	7	7.211	0.61	40	d	0.877505
			236872_at	0	2.421	0.13	7	2.443	0.17	40	u	0.758003
FAM130A1	1	family with sequence similarity 130, member A1	221260_s_at	1	7.86	0.33	7	7.841	0.75	40	d	0.948077
			225042_s_at	1	7.068	0.33	7	7.418	0.62	40	u	0.159452
CORIN	1	corin, serine peptidase	220356_at	1	2.685	0.2	7	2.892	0.5	40	u	0.294709
			239261_s_at	0	3.014	0.52	7	3.302	0.55	40	u	0.215496
			239260_at	0	2.718	0.13	7	2.782	0.14	40	u	0.267443
MSI2	1	musashi homolog 2 (Drosophila)	243010_at	1	3.077	0.39	7	4.265	1.25	40	u	0.000074
			225237_s_at	0	8.182	0.39	7	7.802	1.28	40	d	0.153105
			239232_at	0	5.471	0.75	7	5.315	1.21	40	d	0.748751
			243579_at	0	7.176	0.34	7	7.127	0.99	40	d	0.817644
			225240_s_at	0	9.353	0.26	7	9.225	0.85	40	d	0.466056
			1552364_s_at	0	3.794	0.65	7	4.634	1.16	40	u	0.073344
			225238_at	0	6.42	0.95	7	6.568	1.22	40	u	0.765548
			225233_at	0	3.727	0.72	7	4.197	1.17	40	u	0.319712
TXNDC14	1	thioredoxin domain containing 14	201175_at	1	9.941	0.13	7	9.919	0.79	40	d	0.870341
IL6ST	1	interleukin 6 signal transducer (gp130, oncostatin M receptor)	204863_s_at	1	7.518	0.71	7	7.772	1.12	40	u	0.574963
			204864_s_at	1	4.925	1.12	7	4.963	1.7	40	u	0.956333
			211000_s_at	0	6.723	0.86	7	7.029	1.31	40	u	0.563734
			212196_at	0	9.833	0.62	7	8.513	1.41	40	d	0.021776
			234474_x_at	0	3.054	0.1	7	3.21	0.24	40	u	0.10193
			212195_at	0	12.29	0.27	7	10.78	1.26	40	d	0
			234967_at	0	2.118	0.02	7	2.18	0.05	40	u	0.002805
ZNF319	1	zinc finger protein 319	228460_at	1	4.155	0.6	7	3.645	0.45	40	d	0.014005
MRPL49	1	mitochondrial ribosomal protein L49	201717_at	1	9.443	0.27	7	9.307	0.69	40	d	0.616229
POLR1D	1	polymerase (RNA) I polypeptide D, 16kDa	224857_s_at	1	8.192	0.18	7	8.347	0.83	40	u	0.31397
			224874_at	1	8.575	0.33	7	8.755	0.81	40	u	0.571602
			218258_at	0	9.904	0.25	7	9.296	0.77	40	d	0.00079
EPM2A	1	epilepsy, progressive myoclonus type 2A, Lafora disease (laforin)	205231_s_at	1	5.69	0.4	7	5.729	0.59	40	u	0.869356
			216079_at	1	1.95	0.07	7	2.029	0.17	40	u	0.244375
			210870_s_at	0	3.525	0.62	7	3.481	0.58	40	d	0.857421
SETDB2	1	SET domain, bifurcated 2	238731_at	1	3.216	0.23	7	3.496	0.54	40	u	0.194086
			235339_at	0	6.97	0.44	7	6.41	0.85	40	d	0.101232
			1564970_at	0	2.281	0.03	7	2.379	0.15	40	u	0.000714
			1564972_x_at	0	2.353	0.06	7	2.482	0.25	40	u	0.007861
			235338_s_at	0	6.511	0.29	7	6.312	0.61	40	d	0.413654
SGMS2	1	sphingomyelin synthase 2	242963_at	1	3.521	0.85	7	3.604	0.91	40	u	0.828452
			227038_at	0	6.641	0.66	7	6.323	1.09	40	d	0.467395
			243141_at	0	4.04	0.48	7	3.977	0.64	40	d	0.810052
CARTPT	1	CART prepropeptide	206339_at	1	3.04	0.08	7	3.309	1.29	40	u	0.206318
TNFRSF10B	1	tumor necrosis factor receptor superfamily, member 10b	209295_at	1	9.035	0.31	7	8.277	0.82	40	d	0.022698
			209294_x_at	0	4.839	0.68	7	5.027	1.16	40	u	0.686199
			210405_x_at	0	3.667	0.73	7	4.023	1.01	40	u	0.387117
TBX19	1	T-box 19	206838_at	1	4.214	0.5	7	4.366	0.74	40	u	0.612473
DBN1	1	drebrin 1	202806_at	1	7.731	0.53	7	8.057	0.81	40	u	0.31835
			217025_s_at	0	7.422	0.47	7	7.558	0.78	40	u	0.662879
FEZF2	1	FEZ family zinc finger 2	221086_s_at	1	2.324	0.03	7	2.422	0.13	40	u	0.000314
			233897_at	0	1.938	0.02	7	1.982	0.04	40	u	0.011316
			233972_s_at	0	2.314	0.03	7	2.376	0.08	40	u	0.053445

BIRC8	1	baculoviral IAP repeat-containing 8	no probeset available									
SLC26A9	1	solute carrier family 26, member 9	242271_at	1	2.991	0.25	7	3.371	1.11	40	u	0.072207
IBTK	1	inhibitor of Bruton agammaglobulinemia tyrosine kinase	210970_s_at	1	8.682	0.3	7	8.864	0.68	40	u	0.497661
			215086_at	0	2.95	0.44	7	2.87	0.31	40	d	0.568626
TCEB1P	1	ion elongation factor B (SIII), polypeptide 1 ps	no probeset available									
PANX2	1	pannexin 2	239067_s_at	1	4.56	0.38	7	4.652	0.69	40	u	0.739253
			244471_x_at	1	2.108	0.06	7	2.124	0.05	40	u	0.468032
			1552944_a_at	0	2.364	0.09	7	2.726	0.32	40	u	0.000002
CFTR	1	cystic fibrosis transmembrane conductance regulator (ATP-binding cassette sub-family C, member 7)	205043_at	1	2.486	0.27	7	2.561	0.72	40	u	0.790688
			234702_x_at	0	2.275	0.03	7	2.367	0.1	40	u	0.000025
			234706_x_at	0	2.759	0.07	7	2.943	0.13	40	u	0.001181
			215703_at	0	2.868	0.08	7	3.024	0.18	40	u	0.030975
			215702_s_at	0	2.768	0.17	7	2.807	0.15	40	u	0.539154
			217026_at	0	2.653	0.05	7	2.801	0.27	40	u	0.003745
KLF14	1	Kruppel-like factor 14	1552814_a_at	1	2.447	0.1	7	2.522	0.12	40	u	0.133038
			1552813_at	1	2.127	0.2	7	2.14	0.2	40	u	0.876829
C6orf195	1	chromosome 6 open reading frame 195	1553363_at	1	2.706	0.13	7	2.841	0.22	40	u	0.130511
C15orf24	1	chromosome 15 open reading frame 24	217898_at	1	9.629	0.23	7	9.376	0.66	40	d	0.084387
			227535_at	0	2.7	0.07	7	2.957	0.21	40	u	0.000003
CD48	1	CD48 molecule	204118_at	1	6.97	0.78	7	7.107	1.73	40	u	0.840817
			237759_at	0	3.426	0.42	7	3.387	0.44	40	d	0.830108
GDI2	1	GDP dissociation inhibitor 2	200009_at	1	12.18	0.14	7	12.42	0.78	40	u	0.095129
			200008_s_at	0	10.4	0.39	7	10.61	1.45	40	u	0.468413
SCML1	1	sex comb on midleg-like 1 (Drosophila)	222747_s_at	1	5.734	0.39	7	5.013	1.11	40	d	0.00572
			218793_s_at	1	7.24	0.46	7	5.742	1.31	40	d	0.00002
REXO1L1	1	REX1, RNA exonuclease 1 homolog (S. cerevisiae)-like 1	1553009_s_at	1	2.203	0.06	7	2.245	0.08	40	u	0.214779
CRYGS	1	crystallin, gamma S	223643_at	1	2.648	0.34	7	2.873	0.68	40	u	0.409198
			223644_s_at	0	4.039	0.59	7	3.793	0.82	40	d	0.458725
C14orf131	1	chromosome 14 open reading frame 131	221709_s_at	1	6.65	0.37	7	5.903	0.77	40	d	0.017722
			219086_at	1	4.707	0.56	7	4.464	0.76	40	d	0.430221
			230223_at	0	4.565	0.45	7	4.201	0.46	40	d	0.063921
PHF14	1	PHD finger protein 14	204525_at	1	2.968	0.22	7	3.226	0.58	40	u	0.262635
			228095_at	0	8.494	0.21	7	9.031	0.8	40	u	0.001436
SAP30BP	1	SAP30 binding protein	222475_at	1	6.945	0.1	7	6.977	0.77	40	u	0.805699
			217965_s_at	0	7.879	0.19	7	7.806	0.75	40	d	0.613753
GPRASP1	1	G protein-coupled receptor associated sorting protein 1	204793_at	0	7.751	0.6	7	5.118	0.96	40	d	0
ACBD4	1	acyl-Coenzyme A binding domain containing 4	219413_at	1	6.095	0.17	7	5.061	0.84	40	d	0
HSPH1	1	heat shock 105kDa/110kDa protein 1	208744_x_at	1	8.011	0.26	7	9.323	0.8	40	u	0
			206976_s_at	1	9.792	0.29	7	10.27	0.66	40	u	0.068468
FAM133A	1	family with sequence similarity 133, member A	231131_at	1	2.574	0.11	7	2.815	0.42	40	u	0.004866
			239481_at	0	3.156	0.23	7	3.221	0.31	40	u	0.601802
ZDHHC14	1	zinc finger, DHHC-type containing 14	219247_s_at	1	6.233	0.41	7	5.344	0.82	40	d	0.008535
			1556967_at	0	2.151	0.02	7	2.205	0.08	40	u	0.001506
ACTB	1	actin, beta	213867_x_at	1	14.71	0.14	7	14.75	0.22	40	u	0.597059
			200801_x_at	1	14.71	0.13	7	14.76	0.24	40	u	0.582819
			224594_x_at	0	14.71	0.15	7	14.72	0.24	40	u	0.932934
KIF13B	1	kinesin family member 13B	202962_at	1	7.52	0.56	7	5.246	1.44	40	d	0.000208
MKRN3	1	makorin, ring finger protein, 3	206585_at	1	3.107	0.12	7	3.356	0.65	40	u	0.034965
IMPG1	1	interphotoreceptor matrix proteoglycan 1	207054_at	1	2.533	0.05	7	2.642	0.15	40	u	0.002995
TTYH1	1	tweety homolog 1 (Drosophila)	219415_at	1	4.134	1.15	7	3.575	2.28	40	d	0.53809
C3orf18	1	chromosome 3 open reading frame 18	219114_at	1	6.699	0.3	7	6.273	0.48	40	d	0.030563
EXTL1	1	exostoses (multiple)-like 1	206329_at	1	4.191	0.25	7	4.159	0.64	40	d	0.899619
C9orf24	1	chromosome 9 open reading frame 24	229012_at	1	2.196	0.02	7	2.294	0.2	40	u	0.005834
CAP2	1	CAP, adenylate cyclase-associated protein, 2 (yeast)	212554_at	1	7.185	0.71	7	6.858	1.27	40	d	0.518928
			212551_at	1	6.57	0.86	7	6.859	1.28	40	u	0.575924
MOBKL2C	1	MOB1, Mps One Binder kinase activator-like 2C (yeast)	227066_at	1	6.884	0.4	7	6.157	0.68	40	d	0.009997
			243052_at	1	3.58	0.57	7	3.604	0.74	40	u	0.935825
FAM83D	1	family with sequence similarity 83, member D	225687_at	1	3.698	0.61	7	7.92	1.45	40	u	0
MBIP	1	MAP3K12 binding inhibitory protein 1	218411_s_at	1	6.333	0.58	7	6.084	0.78	40	d	0.43503
MATN2	1	matrilin 2	202350_s_at	1	9.779	0.44	7	6.109	2.01	40	d	0

PRELID1	1	PRELI domain containing 1	224232_s_at	1	7.58	0.17	7	7.794	0.84	40	u	0.165359
			223032_x_at	0	10.25	0.24	7	10.49	0.73	40	u	0.141652
SMG6	1	Smg-6 homolog, nonsense mediated mRNA decay factor (C. elegans)	214940_s_at	1	6.428	0.35	7	5.404	0.75	40	d	0.001124
			220347_at	0	2.302	0.21	7	2.346	0.28	40	u	0.699881
TGFBR2	1	transforming growth factor, beta receptor II (70/80kDa)	207334_s_at	1	5.177	0.87	7	3.827	0.86	40	d	0.00052
			208944_at	1	11.51	0.34	7	8.821	0.96	40	d	0
IL33	1	interleukin 33	209821_at	1	6.19	0.36	7	3.357	0.79	40	d	0
METT5D1	1	methyltransferase 5 domain containing 1	238773_at	1	7.047	0.26	7	6.936	0.59	40	d	0.635927
			242247_at	1	4.743	0.67	7	4.921	0.7	40	u	0.541249
			1562830_at	0	2.201	0.04	7	2.454	0.34	40	u	0.000067
			1565900_at	0	2.947	0.34	7	3.122	0.51	40	u	0.393482
			1565898_at	0	2.987	0.36	7	3.162	0.41	40	u	0.301884
COBL	1	cordon-bleu homolog (mouse)	1552496_a_at	1	7.219	0.67	7	6.46	1.8	40	d	0.287592
			213050_at	1	8.371	0.56	7	7.108	2	40	d	0.003107
SLC35A3	1	solute carrier family 35 (UDP-N-acetylglucosamine (UDP-GlcNAc) transporter), member A3	226894_at	1	8.57	0.27	7	8.729	0.93	40	u	0.394027
			206770_s_at	1	8.129	0.5	7	8.49	0.99	40	u	0.361305
			209865_at	1	6.926	0.31	7	7.028	1.18	40	u	0.657422
CLEC16A	1	C-type lectin domain family 16, member A	212786_at	1	5.676	0.36	7	5.282	0.82	40	d	0.226796
			231221_at	0	3.267	0.3	7	3.111	0.37	40	d	0.30094
ZNF607	1	zinc finger protein 607	223680_at	1	5.213	0.24	7	5.54	0.65	40	u	0.206213
IER3IP1	1	immediate early response 3 interacting protein 1	211406_at	1	5.283	0.2	7	6.368	0.85	40	u	0
			223071_at	1	9.92	0.3	7	10.14	0.76	40	u	0.45697
SLC38A1	1	solute carrier family 38, member 1	218237_s_at	1	11.15	0.65	7	10.19	0.95	40	d	0.0157
			224579_at	0	11.3	0.51	7	10.43	0.9	40	d	0.018167
			236857_at	0	2.457	0.26	7	2.397	0.22	40	d	0.524666
			224580_at	0	7.598	0.74	7	7.438	0.91	40	d	0.668597
TOMM40L	1	translocase of outer mitochondrial membrane 40 homolog (yeast)-like	226059_at	1	4.252	0.46	7	5.171	0.96	40	u	0.018927
MCM3AP	1	minichromosome maintenance complex component 3 associated protein	212269_s_at	1	8.622	0.4	7	8.44	0.76	40	d	0.547896
			214514_at	1	2.176	0.06	7	2.195	0.07	40	u	0.507502
			215580_at	0	2.878	0.18	7	3.008	0.34	40	u	0.344624
			215581_s_at	0	2.576	0.48	7	3.587	1.38	40	u	0.002284
			215582_x_at	0	5.007	0.61	7	5.097	0.77	40	u	0.77728
PPP2R2B	1	protein phosphatase 2 (formerly 2A), regulatory subunit B, beta isoform	213849_s_at	1	4.186	0.34	7	3.858	0.67	40	d	0.22275
			205643_s_at	1	2.393	0.1	7	2.533	0.26	40	u	0.17213
TCF7L2	1	transcription factor 7-like 2 (T-cell specific, HMG-box)	212759_s_at	1	7.793	0.54	7	6.893	1.12	40	d	0.047786
			212761_at	1	10.03	0.41	7	8.399	0.93	40	d	0.000055
			212762_s_at	1	8.863	0.42	7	7.483	1.07	40	d	0.001859
			216035_x_at	1	8.806	0.35	7	7.102	0.99	40	d	0
			216037_x_at	1	8.533	0.57	7	6.952	0.83	40	d	0.00002
			216511_s_at	1	8.754	0.41	7	7.148	1.13	40	d	0.000001
			236094_at	0	7.234	0.51	7	5.499	0.95	40	d	0.000035
WSB2	1	WD repeat and SOCS box-containing 2	201760_s_at	1	9.339	0.37	7	10.04	0.59	40	u	0.004707
TBC1D25	1	TBC1 domain family, member 25	226173_at	1	4.533	0.54	7	4.578	0.57	40	u	0.850199
			227820_at	0	6.188	0.11	7	6.269	0.27	40	u	0.449892
C2orf25	1	chromosome 2 open reading frame 25	217883_at	1	11.57	0.23	7	11.63	0.49	40	u	0.73474
GPD2	1	glycerol-3-phosphate dehydrogenase 2 (mitochondrial)	225447_at	6	8.803	0.46	7	8.926	1	40	u	0.757006
			210007_s_at	1	5.226	0.56	7	6.089	1.28	40	u	0.091526
			211613_s_at	0	2.523	0.31	7	3.186	0.78	40	u	0.036082
HOXB9	1	homeobox B9	226461_at	1	3.948	0.28	7	3.994	0.75	40	u	0.87718
			216417_x_at	0	2.74	0.52	7	2.842	0.61	40	u	0.684511
ACSL6	1	acyl-CoA synthetase long-chain family member 6	213683_at	1	2.285	0.11	7	2.74	1.08	40	u	0.014579
			211207_s_at	1	2.905	0.1	7	3.529	0.98	40	u	0.000398
			229725_at	0	2.773	0.09	7	3.347	0.99	40	u	0.000996
			216409_at	0	2.004	0.32	7	1.94	0.12	40	d	0.648359
			223918_at	0	2.595	0.09	7	2.745	0.22	40	u	0.08476
KIAA1328	1	KIAA1328	232358_at	1	3.87	0.47	7	3.738	0.59	40	d	0.58296
DAP	1	death-associated protein	201095_at	1	9.095	0.48	7	8.991	0.76	40	d	0.734933
ENPP6	1	ectonucleotide pyrophosphatase/phosphodiesterase 6	229916_at	1	2.715	0.18	7	2.995	0.67	40	u	0.038659
ZNF462	1	zinc finger protein 462	226575_at	1	7.462	0.32	7	6.99	1.45	40	d	0.08357
			232393_at	0	2.641	0.26	7	2.595	0.13	40	d	0.678102
CUEDC1	1	CUE domain containing 1	227667_at	0	5.618	0.21	7	5.996	1.1	40	u	0.058345
			219468_s_at	0	6.519	0.26	7	7.01	1.19	40	u	0.030589

CLDN8	1	claudin 8	214598_at	1	8.968	1.04	7	4.978	1.96	40	d	0.000006
LYSMD1	1	LysM, putative peptidoglycan-binding, domain containing 1	232283_at	1	3.887	0.57	7	4.426	1.03	40	u	0.193444
TSR1	1	TSR1, 20S rRNA accumulation, homolog (S. cerevisiae)	218155_x_at	5	8.027	0.33	7	7.792	0.49	40	d	0.233392
			218156_s_at	4	7.378	0.27	7	8.131	0.86	40	u	0.000183
			221987_s_at	1	5.991	0.21	7	6.829	0.81	40	u	0.000005
			239042_at	1	5.382	0.48	7	5.214	0.63	40	d	0.514666
SCRIB	1	scribbled homolog (Drosophila)	212556_at	1	7.39	0.32	7	7.443	1.35	40	u	0.834711
FAM115A	1	family with sequence similarity 115, member A	204403_x_at	1	8.093	0.4	7	7.818	0.63	40	d	0.283329
			212981_s_at	0	7.224	0.39	7	7.558	0.93	40	u	0.363598
			212979_s_at	0	7.117	0.38	7	8.031	1.02	40	u	0.026396
			210529_s_at	0	6.456	0.57	7	6.346	1.46	40	d	0.84845
GPX3	1	glutathione peroxidase 3 (plasma)	201348_at	1	10.94	1.06	7	7.758	1.24	40	d	0
			214091_s_at	1	10.14	0.99	7	7.306	1.02	40	d	0
CPSF4	1	cleavage and polyadenylation specific factor 4, 30kDa	206688_s_at	1	7.732	0.15	7	7.633	0.53	40	d	0.35414
TMEM93	1	transmembrane protein 93	221255_s_at	1	8.056	0.3	7	8.387	0.65	40	u	0.204385
PNO1	1	partner of NOB1 homolog (S. cerevisiae)	211697_x_at	1	7.661	0.32	7	8.346	0.76	40	u	0.025696
			203622_s_at	1	7.842	0.47	7	8.832	0.81	40	u	0.003596
			242905_at	1	4.688	0.52	7	5.53	0.59	40	u	0.001156
			242627_at	0	3.8	0.36	7	4.126	0.43	40	u	0.071436
HPS5	1	Hermansky-Pudlak syndrome 5	204544_at	0	8.055	0.43	7	7.489	0.5	40	d	0.008109
CDH12	1	cadherin 12, type 2 (N-cadherin 2)	207149_at	1	2.742	0.66	7	2.652	0.59	40	d	0.722824
SNX24	1	sorting nexin 24	222716_s_at	1	7.229	0.85	7	7.185	0.97	40	d	0.912433
			233883_at	1	3.731	0.39	7	3.656	0.28	40	d	0.547486
			218705_s_at	1	5.898	0.66	7	6.164	0.91	40	u	0.472283
			239693_at	0	2.537	0.07	7	2.691	0.28	40	u	0.006224
			239739_at	0	2.26	0.04	7	2.39	0.16	40	u	0.000112
RARS	1	arginyl-tRNA synthetase	201330_at	1	9.118	0.12	7	9.103	0.37	40	d	0.846123
ARRDC1	1	arrestin domain containing 1	226405_s_at	0	8.276	0.32	7	8.069	0.92	40	d	0.30759
ACOT4	1	acyl-CoA thioesterase 4	229534_at	1	6.869	0.78	7	6.794	1.17	40	d	0.873651
YIPF5	1	Yip1 domain family, member 5	224949_at	1	8.593	0.26	7	8.567	1.06	40	d	0.897814
			224953_at	1	8.279	0.21	7	8.231	0.68	40	d	0.726573
			224934_at	1	9.454	0.28	7	9.759	0.55	40	u	0.166804
			221423_s_at	0	7.718	0.49	7	7.389	1.41	40	d	0.288608
IL11	1	interleukin 11	206926_s_at	1	5.363	0.39	7	5.259	0.41	40	d	0.544333
			206924_at	1	2.501	0.12	7	2.53	0.17	40	u	0.678263
PRSS8	1	protease, serine, 8	202525_at	1	8.648	0.6	7	8.404	1.39	40	d	0.656506
HSD11B1	1	hydroxysteroid (11-beta) dehydrogenase 1	205404_at	1	4.85	0.95	7	4.295	0.72	40	d	0.088513
SUMO4	1	SMT3 suppressor of mif two 3 homolog 4 (S. cerevisiae)	215452_x_at	1	9.091	0.08	7	9.484	0.46	40	u	0.000017
SETX	1	senataxin	201965_s_at	0	7.577	0.33	7	7.68	0.48	40	u	0.598904
			201964_at	0	7.777	0.3	7	7.119	0.81	40	d	0.043592
			232229_at	0	3	0.36	7	3.866	0.98	40	u	0.028286
CBX5	1	chromobox homolog 5 (HP1 alpha homolog, Drosophila)	209715_at	1	5.502	0.87	7	6.843	0.82	40	u	0.000356
			242069_at	0	2.192	0.19	7	2.37	0.57	40	u	0.155666
WWP2	1	WW domain containing E3 ubiquitin protein ligase 2	204022_at	1	7.825	0.47	7	7.741	1.36	40	d	0.777431
			1552737_s_at	0	3.132	0.36	7	3.458	1.46	40	u	0.243287
			1554580_a_at	0	3.287	0.33	7	3.977	0.58	40	u	0.004269
			210200_at	0	4.875	0.32	7	5.267	0.45	40	u	0.036081
COL5A1	1	collagen, type V, alpha 1	212488_at	8	6.977	0.65	7	7.429	1.84	40	u	0.265949
			212489_at	8	6.88	0.6	7	7.619	1.45	40	u	0.201502
			203325_s_at	1	6.667	0.62	7	7.412	1.57	40	u	0.231103
PAF1	1	Paf1, RNA polymerase II associated factor, homolog (S. cerevisiae)	202093_s_at	1	8.312	0.14	7	7.655	0.73	40	d	0.00001
DERL1	1	Der1-like domain family, member 1	222543_at	1	8.699	0.13	7	9.68	0.68	40	u	0
			219402_s_at	1	8.484	0.32	7	9.644	0.7	40	u	0.000111
			218172_s_at	1	7.893	0.42	7	8.898	0.88	40	u	0.005953
			225488_at	1	5.227	0.32	7	5.113	0.49	40	d	0.563658
TMEM2	1	transmembrane protein 2	218113_at	1	7.353	0.54	7	6.578	1.01	40	d	0.058097
MOAP1	1	modulator of apoptosis 1	212508_at	1	9.293	0.31	7	8.703	0.66	40	d	0.027505
MFNG	1	MFNG O-fucosylpeptide 3-beta-N-acetylglucosaminyltransferase	204153_s_at	1	6.681	0.68	7	6.164	0.91	40	d	0.169287
			204152_s_at	1	2.623	0.39	7	2.508	0.63	40	d	0.648056
			213783_at	0	2.785	0.2	7	2.904	0.34	40	u	0.381524

CASQ2	1	calsequestrin 2 (cardiac muscle)	207317_s_at	1	3.376	0.57	7	3.189	1.15	40	d	0.682657
KPRP	1	keratinocyte proline-rich protein	no probeset available									
ZNF12	1	zinc finger protein 12	226015_at	1	9.103	0.2	7	8.673	0.63	40	d	0.002722
			1559881_s_at	0	8.629	0.31	7	7.848	1	40	d	0.000661
			219571_s_at	0	9.328	0.15	7	8.811	0.55	40	d	0.000027
			1554021_a_at	0	9.272	0.13	7	8.84	0.58	40	d	0.000272
PSEN1	1	presenilin 1 (Alzheimer disease 3)	203460_s_at	1	8.048	0.19	7	7.944	0.6	40	d	0.406471
			207782_s_at	0	7.219	0.36	7	7.092	1.37	40	d	0.635863
			1567443_x_at	0	3.284	0.25	7	3.445	0.31	40	u	0.209736
			1559206_at	0	2.895	0.16	7	2.968	0.18	40	u	0.320638
			1567440_at	0	2.024	0.12	7	2.132	0.19	40	u	0.157684
CTHRC1	1	collagen triple helix repeat containing 1	225681_at	1	8.463	0.99	7	11.55	1.04	40	u	0
OLA1	1	Obg-like ATPase 1	224525_s_at	1	4.356	0.89	7	4.265	0.76	40	d	0.781542
			219293_s_at	0	10.81	0.21	7	11.16	0.45	40	u	0.051412
MAMDC2	1	MAM domain containing 2	228885_at	1	8.836	0.47	7	3.951	0.84	40	d	0
TFPI2	1	tissue factor pathway inhibitor 2	209277_at	1	5.6	1.56	7	4.224	1.37	40	d	0.023284
			209278_s_at	1	7.268	2.51	7	4.414	2.37	40	d	0.006515
SHKBP1	1	SH3KBP1 binding protein 1	224846_at	1	6.353	0.23	7	6.677	0.46	40	u	0.081835
NAGK	1	N-acetylglucosamine kinase	218231_at	1	9.543	0.22	7	9.795	0.55	40	u	0.246095
NOTCH3	1	Notch homolog 3 (Drosophila)	203237_s_at	1	3.227	0.33	7	3.215	0.45	40	d	0.949186
			203238_s_at	1	8.148	0.54	7	7.525	1.39	40	d	0.25946
TMEM101	1	transmembrane protein 101	225004_at	1	7.494	0.55	7	6.309	0.96	40	d	0.003326
FAM58A	1	family with sequence similarity 58, member A	226466_s_at	1	7.332	0.21	7	7.846	0.75	40	u	0.001539
CEP57	1	centrosomal protein 57kDa	209862_s_at	1	7.295	0.19	7	7.021	1.12	40	d	0.170896
			203493_s_at	1	7.073	0.48	7	6.99	0.9	40	d	0.815494
			203494_s_at	1	8.459	0.17	7	7.825	0.89	40	d	0.000267
			203491_s_at	1	6.796	0.37	7	7.461	0.99	40	u	0.091695
			203492_x_at	1	7.71	0.27	7	7.338	1.02	40	d	0.067022
AR	1	androgen receptor (dihydrotestosterone receptor; testicular feminization; spinal and	211110_s_at	1	7.56	0.86	7	5.129	2.57	40	d	0.000128
			211621_at	1	6.775	0.7	7	5.425	1.85	40	d	0.068968
MYO18B	1	myosin XVIIIB	1554579_a_at	0	2.298	0.21	7	2.453	0.58	40	u	0.227846
EPB41L5	1	erythrocyte membrane protein band 4.1 like 5	220977_x_at	1	2.25	0.12	7	2.34	0.4	40	u	0.275961
			225855_at	1	6.559	0.52	7	5.147	1.27	40	d	0.006833
			229292_at	0	6.986	0.53	7	7.392	0.85	40	u	0.238834
RND1	1	Rho family GTPase 1	210056_at	1	4.29	1.14	7	4.864	1.71	40	u	0.406948
DHX36	1	DEAH (Asp-Glu-Ala-His) box polypeptide 36	223139_s_at	1	9.997	0.13	7	9.87	0.53	40	d	0.214144
			223138_s_at	1	7.941	0.2	7	8.435	0.65	40	u	0.000825
			223140_s_at	1	7.611	0.27	7	7.527	0.74	40	d	0.773958
			1559039_at	0	5.089	0.45	7	4.755	0.56	40	d	0.150733
NUDCD1	1	NudC domain containing 1	225438_at	1	3.879	0.23	7	5.069	0.99	40	u	0
			225439_at	1	7.505	0.35	7	8.906	0.88	40	u	0.000191
FGD6	1	FYVE, RhoGEF and PH domain containing 6	219901_at	1	5.533	0.48	7	6.14	1.29	40	u	0.234225
			1555137_a_at	0	5.113	0.7	7	5.9	1.37	40	u	0.153368
			1555136_at	0	4.567	0.46	7	4.613	0.69	40	u	0.870616
USP44	1	ubiquitin specific peptidase 44	224048_at	1	3.09	0.36	7	2.975	0.27	40	d	0.339254
GOLGA4	1	golgi autoantigen, golgin subfamily a, 4	201567_s_at	1	9.553	0.23	7	9.501	0.63	40	d	0.712153
			215203_at	0	8.054	0.61	7	7.36	0.85	40	d	0.049129
IWS1	1	IWS1 homolog (S. cerevisiae)	224775_at	1	5.856	0.23	7	6.485	0.64	40	u	0.015268
			224768_at	1	8.084	0.26	7	8.057	0.47	40	d	0.88534
PCGF1	1	polycomb group ring finger 1	210022_at	1	7.546	0.15	7	7.649	0.56	40	u	0.347354
			210023_s_at	1	8.049	0.28	7	8.183	0.52	40	u	0.515166
PTPN13	1	protein tyrosine phosphatase, non-receptor type 13 (APO-1/CD95 (Fas)-associated	204201_s_at	1	7.763	0.21	7	7.402	0.93	40	d	0.041815
			243792_x_at	0	4.979	0.57	7	4.955	0.96	40	d	0.950633
AGPAT7	1	1-acylglycerol-3-phosphate O-acyltransferase 7 (lysophosphatidic acid acyltransferase, eta)	213078_x_at	1	4.022	0.39	7	3.414	0.68	40	d	0.029731
			40472_at	1	7.319	0.31	7	6.032	0.96	40	d	0.000001
			239608_at	0	4.394	0.63	7	4.267	0.58	40	d	0.610815
			239609_s_at	0	4.299	0.18	7	4.013	0.4	40	d	0.075939
			215409_at	0	4.457	0.2	7	4.381	0.54	40	d	0.716188
RPS6KC1	1	ribosomal protein S6 kinase, 52kDa, polypeptide 1	218909_at	1	7.041	0.42	7	7.712	0.66	40	u	0.014138
NPC1	1	Niemann-Pick disease, type C1	202679_at	1	8.01	0.47	7	7.777	0.77	40	d	0.453566
			217584_at	0	3.826	0.41	7	3.841	0.85	40	u	0.964759

ALDH9A1	1	aldehyde dehydrogenase 9 family, member A1	201612_at	1	10.5	0.17	7	10.28	0.78	40	d	0.132453
B3GAT3	1	beta-1,3-glucuronyltransferase 3 (glucuronosyltransferase I)	35179_at	1	7.365	0.2	7	7.231	0.57	40	d	0.286276
			203452_at	1	6.593	0.23	7	6.584	0.88	40	d	0.956
PKD2L2	1	polycystic kidney disease 2-like 2	221118_at	1	3.434	0.29	7	3.582	0.32	40	u	0.27221
			224226_at	0	2.996	0.14	7	3.143	0.24	40	u	0.134216
AARS	1	alanyl-tRNA synthetase	201000_at	1	9.379	0.26	7	9.591	0.85	40	u	0.228443
ARHGAP27	1	Rho GTPase activating protein 27	229424_s_at	1	2.493	0.07	7	2.518	0.11	40	u	0.572016
			225618_at	1	6.545	0.22	7	6.61	0.5	40	u	0.737667
			227057_at	0	2.423	0.36	7	2.774	0.65	40	u	0.182658
LRP11	1	low density lipoprotein receptor-related protein 11	225060_at	1	8.096	0.25	7	8.452	1.06	40	u	0.079994
URM1	1	ubiquitin related modifier 1 homolog (S. cerevisiae)	208101_s_at	1	7.616	0.23	7	7.434	0.53	40	d	0.383735
CLIC6	1	chloride intracellular channel 6	227742_at	1	6.555	1.86	7	4.462	2.59	40	d	0.050891
			242913_at	0	4.401	0.77	7	4.157	1.04	40	d	0.562796
ZNF420	1	zinc finger protein 420	238937_at	1	5.448	0.4	7	5.699	1.1	40	u	0.308216
			242761_s_at	0	7.077	0.29	7	6.653	0.94	40	d	0.034449
PAK2	1	p21 (CDKN1A)-activated kinase 2	208878_s_at	1	7.033	0.27	7	7.649	0.61	40	u	0.013742
			208877_at	1	8.59	0.32	7	8.588	0.57	40	d	0.993258
			208876_s_at	1	5.913	0.19	7	6.986	0.84	40	u	0
			208875_s_at	1	6.285	0.42	7	8.123	1.09	40	u	0.000087
			236283_x_at	0	5.57	0.32	7	5.701	0.54	40	u	0.546467
			205962_at	0	3.476	0.3	7	3.493	0.41	40	u	0.919898
			1559052_s_at	0	5.284	0.48	7	6.495	1.32	40	u	0.000332
GLS2	1	glutaminase 2 (liver, mitochondrial)	205531_s_at	1	4.289	0.53	7	3.638	1.13	40	d	0.149431
			1564707_x_at	0	2.443	0.27	7	2.701	0.41	40	u	0.126583
			1564705_at	0	3.144	0.07	7	3.322	0.26	40	u	0.001614
			1564706_s_at	0	2.448	0.14	7	2.564	0.7	40	u	0.357903
MNX1	1	motor neuron and pancreas homeobox 1	214614_at	1	4.394	0.55	7	5.161	1.02	40	u	0.063788
TCEAL1	1	transcription elongation factor A (SII)-like 1	204045_at	1	9.296	0.51	7	8.425	0.95	40	d	0.025713
ATF7IP2	1	activating transcription factor 7 interacting protein 2	219870_at	1	5.859	0.84	7	5.783	0.86	40	d	0.832439
			228381_at	0	5.175	0.44	7	5.306	1.09	40	u	0.760803
SLC25A43	1	solute carrier family 25, member 43	1557411_s_at	1	5.567	0.47	7	6.349	1.27	40	u	0.12113
PTGIS	1	prostaglandin I2 (prostacyclin) synthase	208131_s_at	1	8.502	1.19	7	6.367	1.47	40	d	0.00089
			211892_s_at	0	2.147	0.12	7	2.325	0.71	40	u	0.158001
			210702_s_at	0	2.642	0.27	7	2.738	0.51	40	u	0.639036
UBE2J2	1	ubiquitin-conjugating enzyme E2, J2 (UBC6 homolog, yeast)	225209_s_at	1	8.958	0.27	7	9.078	0.41	40	u	0.473447
			224347_x_at	1	5.968	0.4	7	5.949	0.61	40	d	0.938353
			226704_at	1	6.037	0.47	7	5.846	0.6	40	d	0.437287
HNRPH2	1	heterogeneous nuclear ribonucleoprotein H2 (H')	201132_at	1	10.31	0.31	7	9.738	0.81	40	d	0.079296
			243201_at	0	3.84	0.39	7	3.305	0.18	40	d	0.016059
ARNT2	1	aryl-hydrocarbon receptor nuclear translocator 2	202986_at	1	5.24	0.64	7	6.409	2.02	40	u	0.009078
HPSE2	1	heparanase 2	220927_s_at	1	3.268	0.64	7	2.603	0.27	40	d	0.046031
INPP5E	1	inositol polyphosphate-5-phosphatase, 72 kDa	204706_at	1	7.675	0.26	7	7.317	0.59	40	d	0.126344
HSPB1	1	heat shock 27kDa protein 1	201841_s_at	1	11.52	0.43	7	11.47	1.64	40	d	0.890739
ARRB2	1	arrestin, beta 2	203388_at	1	7.763	0.35	7	7.733	0.63	40	d	0.907096
AHDC1	1	AT hook, DNA binding motif, containing 1	205002_at	0	6.931	0.47	7	6.033	0.78	40	d	0.005922
			243793_at	0	3.239	0.22	7	3.368	0.28	40	u	0.259858
			229379_at	0	2.275	0.01	7	2.31	0.07	40	u	0.006787
TMEM167	1	transmembrane protein 167	226276_at	1	8.513	0.42	7	9.52	0.75	40	u	0.001522
			224702_at	0	10.01	0.2	7	9.797	0.66	40	d	0.1185
ZNF572	1	zinc finger protein 572	1553229_at	1	2.952	0.14	7	3.485	0.64	40	u	0.000049
POLK	1	polymerase (DNA directed) kappa	223261_at	1	6.46	0.46	7	6.282	0.61	40	d	0.473568
			223260_s_at	0	6.483	0.52	7	6.24	0.89	40	d	0.495967
			1555317_at	0	3.27	0.17	7	3.328	0.16	40	u	0.401565
RBM15	1	RNA binding motif protein 15	1555762_s_at	1	6.714	0.27	7	7.457	0.52	40	u	0.000756
			219286_s_at	1	8.005	0.23	7	8.424	0.53	40	u	0.047801
			1555761_x_at	0	5.987	0.19	7	6.26	0.49	40	u	0.158283
			228455_at	0	5.939	0.43	7	5.875	1.37	40	d	0.819096
			232971_at	0	2.847	0.07	7	3.389	0.57	40	u	0.000001
			1555760_a_at	0	5.832	0.3	7	6.243	0.84	40	u	0.034966

ADCY7	1	adenylate cyclase 7	203741_s_at	1	5.853	0.56	7	6.823	1.01	40	u	0.020099
			1554827_a_at	0	1.742	0.03	7	1.791	0.09	40	u	0.170481
GNAL	1	guanine nucleotide binding protein (G protein), alpha activating activity polypeptide,	206355_at	1	2.963	0.23	7	3.006	0.46	40	u	0.810167
			206356_s_at	0	2.304	0.24	7	2.431	0.5	40	u	0.518212
NSMCE4A	1	non-SMC element 4 homolog A (S. cerevisiae)	227387_at	0	2.333	0.08	7	2.521	0.64	40	u	0.089768
			211376_s_at	0	8.889	0.3	7	8.377	1	40	d	0.016217
			219067_s_at	0	9.847	0.21	7	9.038	1.03	40	d	0.000093
			228506_at	0	6.51	0.7	7	4.544	1.62	40	d	0.003375
ARRDC2	1	arrestin domain containing 2	226055_at	1	6.696	0.51	7	6.149	0.82	40	d	0.103352
NDUFAB1	1	NADH dehydrogenase (ubiquinone) 1, alpha/beta subcomplex, 1, 8kDa	202077_at	0	10.95	0.14	7	11.42	0.57	40	u	0.000103
BAK1	1	BCL2-antagonist/killer 1	203728_at	1	5.644	0.28	7	6.276	0.79	40	u	0.00108
FXYD6	1	FXYD domain containing ion transport regulator 6	217897_at	0	8.483	0.55	7	7.118	1.57	40	d	0.000449
CARKL	1	carbohydrate kinase-like	219713_at	1	6.119	0.26	7	6.117	0.47	40	d	0.990919
			219712_s_at	1	2.505	0.31	7	2.617	0.47	40	u	0.556964
GOT1	1	glutamic-oxaloacetic transaminase 1, soluble (aspartate aminotransferase 1)	208813_at	1	7.756	0.61	7	8.05	0.73	40	u	0.328739
TMEM39A	1	transmembrane protein 39A	222690_s_at	1	7.167	0.33	7	7.564	0.59	40	u	0.09701
			218615_s_at	1	6.673	0.44	7	6.94	0.66	40	u	0.315647
			241392_at	0	3.502	0.45	7	3.804	0.78	40	u	0.336538
ARF6	1	ADP-ribosylation factor 6	224788_at	1	10.38	0.29	7	10.14	0.58	40	d	0.307407
			203312_x_at	1	9.47	0.31	7	9.67	0.57	40	u	0.377456
			214182_at	1	8.588	0.17	7	9.315	0.82	40	u	0.000014
			203311_s_at	0	8.125	0.46	7	7.601	0.94	40	d	0.165731
LRRN2	1	leucine rich repeat neuronal 2	205154_at	1	5.469	0.32	7	5.592	1.2	40	u	0.599671
			216167_at	0	3.219	0.26	7	3.334	0.32	40	u	0.38663
			216164_at	0	3.21	0.31	7	2.984	0.36	40	d	0.131393
B3GAT1	1	beta-1,3-glucuronyltransferase 1 (glucuronosyltransferase P)	219521_at	0	3.571	1.79	7	2.518	0.75	40	d	0.204333
BET1L	1	blocked early in transport 1 homolog (S. cerevisiae)-like	220470_at	1	3.686	0.51	7	3.565	1.02	40	d	0.763887
			223023_at	1	8.858	0.23	7	8.083	0.64	40	d	0.000008
HMGN1	1	high-mobility group nucleosome binding domain 1	200943_at	1	12.41	0.17	7	12.59	0.53	40	u	0.117167
			200944_s_at	1	11.97	0.22	7	12.2	0.42	40	u	0.167028
FADS1	1	fatty acid desaturase 1	208962_s_at	1	6.529	0.78	7	7.384	1.3	40	u	0.106891
			208963_x_at	1	6.24	0.75	7	6.849	1.06	40	u	0.159449
			208964_s_at	1	6.162	0.74	7	6.976	0.92	40	u	0.035608
ZNF182	1	zinc finger protein 182	235810_at	1	5.303	0.33	7	5.197	0.88	40	d	0.759715
			244024_at	0	7.418	0.33	7	6.869	0.58	40	d	0.022154
PUF60	1	poly-U binding splicing factor 60KDa	209899_s_at	1	9.815	0.28	7	10.17	0.78	40	u	0.046021
ARL4A	1	ADP-ribosylation factor-like 4A	205020_s_at	0	10.12	0.43	7	8.31	0.86	40	d	0.000003
ZNF673	1	zinc finger protein 673	206583_at	1	7.534	0.36	7	6.894	0.68	40	d	0.02135
			1554465_s_at	1	5.02	0.4	7	4.945	0.57	40	d	0.743584
IRX5	1	iroquois homeobox 5	210239_at	0	9.609	0.41	7	8.458	1.57	40	d	0.000541
HSD11B2	1	hydroxysteroid (11-beta) dehydrogenase 2	204130_at	1	3.296	0.41	7	3.633	0.86	40	u	0.325483
PAPSS2	1	3'-phosphoadenosine 5'-phosphosulfate synthase 2	203058_s_at	1	8.302	1.09	7	7.888	1.63	40	d	0.529401
			203060_s_at	1	9.577	1.19	7	9.093	1.6	40	d	0.459215
			203059_s_at	0	5.107	1.09	7	4.654	1.25	40	d	0.383203
CLCC1	1	chloride channel CLIC-like 1	213628_at	1	7.667	0.28	7	7.621	0.47	40	d	0.808737
			207855_s_at	1	6.765	0.26	7	7.248	0.67	40	u	0.071423
			230002_at	1	5.251	0.58	7	6.682	0.97	40	u	0.000546
			1555543_a_at	0	6.058	0.32	7	5.932	1.07	40	d	0.562356
SMO	1	smoothened homolog (Drosophila)	218629_at	0	6.492	0.22	7	6.436	0.74	40	d	0.707671
ACSL3	1	acyl-CoA synthetase long-chain family member 3	201660_at	1	9.68	0.65	7	8.794	1.03	40	d	0.037591
			201662_s_at	1	10.3	1.37	7	9.498	0.89	40	d	0.058045
			201661_s_at	1	9.573	0.69	7	9.152	0.94	40	d	0.274969
CDC14A	1	CDC14 cell division cycle 14 homolog A (S. cerevisiae)	205288_at	1	4.651	0.51	7	4.069	0.66	40	d	0.034302
			210440_s_at	0	2.93	0.26	7	2.647	0.29	40	d	0.022977
			210742_at	0	4.729	0.62	7	4.044	0.92	40	d	0.069484
			210441_at	0	2.145	0.02	7	2.244	0.19	40	u	0.002738
			210743_s_at	0	3.295	0.23	7	3.194	0.68	40	d	0.493202
SIX5	1	SIX homeobox 5	229009_at	1	7.855	0.3	7	7.1	0.59	40	d	0.002126
			217661_x_at	0	3.272	0.34	7	3.106	0.47	40	d	0.383425
			241822_at	0	3.317	0.42	7	3.365	0.61	40	u	0.843423

NT5M	1	5',3'-nucleotidase, mitochondrial	219708_at	1	2.624	0.2	7	2.661	0.29	40	u	0.755288
TMCO3	1	transmembrane and coiled-coil domains 3	226050_at	1	9.424	0.31	7	8.986	1.01	40	d	0.040938
			230317_x_at	0	2.147	0.12	7	2.176	0.11	40	u	0.534654
			220240_s_at	0	7.074	0.43	7	6.716	1.5	40	d	0.239308
			220241_at	0	3.047	0.15	7	3.431	0.64	40	u	0.002465
C15orf52	1	chromosome 15 open reading frame 52	227272_at	1	6.832	0.59	7	5.985	1.24	40	d	0.089583
CPLX3	1	complexin 3	219775_s_at	1	5.484	0.31	7	5.451	0.56	40	d	0.880992
			222927_s_at	1	2.67	0.16	7	2.676	0.3	40	u	0.956696
SPIRE2	1	spire homolog 2 (Drosophila)	227706_at	1	4.151	0.19	7	4.196	0.96	40	u	0.795081
			243594_x_at	1	4.197	0.26	7	4.397	0.62	40	u	0.417642
			1569723_a_at	0	3.364	0.24	7	3.809	0.65	40	u	0.088692
			1568916_at	0	3.666	0.24	7	4.142	0.46	40	u	0.011342
PLEKHO1	1	pleckstrin homology domain containing, family O member 1	218223_s_at	0	7.144	0.61	7	7.555	0.94	40	u	0.279533
KIAA1715	1	KIAA1715	225718_at	1	7.648	0.22	7	7.7	0.62	40	u	0.699534
			225717_at	1	4.479	0.12	7	5.38	0.8	40	u	0
			229173_at	0	6.186	0.22	7	6.456	0.68	40	u	0.068405
KIAA2018	1	KIAA2018	242513_x_at	1	2.804	0.09	7	2.919	0.15	40	u	0.059457
			227433_at	0	7.2	0.34	7	6.516	0.76	40	d	0.026268
			242508_at	0	1.991	0.05	7	2.128	0.25	40	u	0.003381
			227435_at	0	7.518	0.36	7	7.666	0.64	40	u	0.560811
			1559601_at	0	2.864	0.28	7	2.908	0.19	40	u	0.61538
TMEM63C	1	transmembrane protein 63C	227733_at	1	5.717	0.87	7	5.229	1.57	40	d	0.438399
RING1	1	ring finger protein 1	208371_s_at	1	8.957	0.2	7	8.419	0.32	40	d	0.00014
			35685_at	1	9.294	0.11	7	8.722	0.35	40	d	0
LRRC8D	1	leucine rich repeat containing 8 family, member D	218684_at	1	8.639	0.32	7	9.006	0.9	40	u	0.070697
TPI1	1	triosephosphate isomerase 1	200822_x_at	1	11.17	0.13	7	11.76	0.61	40	u	0.000003
			213011_s_at	1	10.99	0.11	7	11.73	0.62	40	u	0
			210050_at	1	3.051	0.15	7	3.611	0.58	40	u	0.000015
MSH6	1	mutS homolog 6 (E. coli)	202911_at	6	8.232	0.15	7	9.213	0.65	40	u	0
			240148_at	0	4.245	0.57	7	4.869	0.64	40	u	0.022922
			211450_s_at	0	7.049	0.31	7	7.876	1.64	40	u	0.006964
			211449_at	0	2.449	0.04	7	2.584	0.12	40	u	0.000032
C4orf15	1	chromosome 4 open reading frame 15	210054_at	0	5.949	0.4	7	6.972	0.63	40	u	0.0002
			242804_at	0	3.006	0.54	7	2.771	0.4	40	d	0.190873
LMOD3	1	leiomodin 3 (fetal)	1564469_at	1	3.173	0.12	7	3.427	0.27	40	u	0.01989
			243346_at	1	3.054	0.25	7	2.987	0.16	40	d	0.38176
			1555567_s_at	0	3.358	0.19	7	3.582	0.23	40	u	0.021926
IPO4	1	importin 4	218305_at	1	7.327	0.39	7	7.381	0.74	40	u	0.854354
FIGF	1	c-fos induced growth factor (vascular endothelial growth factor D)	206742_at	1	7.708	0.99	7	2.383	0.31	40	d	0.000012
ZBTB8	1	zinc finger and BTB domain containing 8	235142_at	1	7.248	0.16	7	7.44	0.8	40	u	0.186701
SLCO2A1	1	solute carrier organic anion transporter family, member 2A1	204368_at	1	7.105	0.71	7	6.017	1.36	40	d	0.048542
FOXA2	1	forkhead box A2	40284_at	0	2.629	0.09	7	2.867	0.6	40	u	0.025261
			210103_s_at	0	2.098	0.02	7	2.225	0.32	40	u	0.017664
			214312_at	0	2.37	0.2	7	2.384	0.18	40	u	0.859867
CYorf15B	1	chromosome Y open reading frame 15B	223645_s_at	1	2.087	0.11	7	2.161	0.15	40	u	0.228603
			223646_s_at	1	2.037	0.06	7	2.161	0.18	40	u	0.00336
			214131_at	0	3.304	0.23	7	3.444	0.32	40	u	0.287782
ATAD1	1	ATPase family, AAA domain containing 1	224850_at	1	8.761	0.35	7	8.621	0.5	40	d	0.492493
LAMP3	1	lysosomal-associated membrane protein 3	205569_at	1	3.06	0.38	7	5.8	1.95	40	u	0
KIAA1622	1	KIAA1622	233002_at	1	2.905	0.46	7	3.248	0.93	40	u	0.353614
			220672_at	0	2.716	0.04	7	2.874	0.2	40	u	0.000085
			220673_s_at	0	2.224	0.04	7	2.416	0.33	40	u	0.000999
MFSD11	1	major facilitator superfamily domain containing 11	223242_s_at	1	7.916	0.45	7	7.944	0.95	40	u	0.939615
			221192_x_at	1	7.384	0.13	7	7.363	0.5	40	d	0.828142
HNRNPR	1	heterogeneous nuclear ribonucleoprotein R	208765_s_at	1	10.04	0.23	7	10.05	0.65	40	u	0.955327
			208766_s_at	1	10.59	0.09	7	10.8	0.46	40	u	0.014617
			232004_at	0	7.377	0.27	7	6.723	0.71	40	d	0.022959
CDH10	1	cadherin 10, type 2 (T2-cadherin)	220115_s_at	1	2.665	0.07	7	2.912	0.31	40	u	0.00009
GNAT1	1	guanine nucleotide binding protein (G protein), alpha transducing activity	207514_s_at	1	2.193	0.14	7	2.23	0.17	40	u	0.585532
			214286_at	1	2.426	0.14	7	2.558	0.42	40	u	0.149708

SLC35D2	1	solute carrier family 35, member D2	213083_at	1	7.45	0.18	7	7.063	0.71	40	d	0.006977
			213082_s_at	1	6.753	0.22	7	6.691	0.66	40	d	0.659756
			233325_at	1	3.901	0.3	7	3.561	0.44	40	d	0.063685
			231437_at	0	6.165	0.33	7	5.697	0.57	40	d	0.045972
ALCAM	1	activated leukocyte cell adhesion molecule	201951_at	12	9.738	0.66	7	8.915	1.49	40	d	0.167113
			201952_at	12	11.36	0.62	7	9.783	1.59	40	d	0.015113
			1569362_at	0	5.051	0.97	7	4.873	1.18	40	d	0.713787
SC4MOL	1	sterol-C4-methyl oxidase-like	209146_at	1	9.249	1.24	7	9.106	1.08	40	d	0.758743
TRIM29	1	tripartite motif-containing 29	202504_at	1	11.07	0.54	7	7.641	2.1	40	d	0
			211002_s_at	1	8.55	0.45	7	6.594	1.24	40	d	0
			211001_at	1	2.79	0.29	7	2.727	0.32	40	d	0.632401
PSMD3	1	proteasome (prosome, macropain) 26S subunit, non-ATPase, 3	201388_at	1	8.599	0.35	7	9.48	1.41	40	u	0.002152
GPD1	1	glycerol-3-phosphate dehydrogenase 1 (soluble)	213706_at	1	6.45	1.94	7	3.368	0.75	40	d	0.008561
			1563035_x_at	0	2.946	0.19	7	2.873	0.19	40	d	0.354659
			1563034_at	0	3.355	0.28	7	3.253	0.26	40	d	0.361872
			204997_at	0	5.269	1.74	7	2.523	0.34	40	d	0.008408
C1orf168	1	chromosome 1 open reading frame 168	238625_at	1	5.482	1.47	7	3.572	1.15	40	d	0.000447
			231073_at	0	2.923	0.78	7	2.456	0.25	40	d	0.19519
PRPS2	1	phosphoribosyl pyrophosphate synthetase 2	203401_at	1	7.536	0.28	7	8.042	0.85	40	u	0.008866
			230352_at	0	5.041	0.54	7	6.467	1.28	40	u	0.006911
DNMT3B	1	DNA (cytosine-5-)-methyltransferase 3 beta	220668_s_at	1	4.776	0.54	7	6.022	1.12	40	u	0.00697
TSPYL4	1	TSPY-like 4	212928_at	1	8.91	0.17	7	8.342	0.84	40	d	0.000511
ZNF24	1	zinc finger protein 24	203248_at	1	7.446	0.4	7	7.256	0.97	40	d	0.620841
			1554045_at	0	5.543	0.17	7	5.801	0.56	40	u	0.032129
			242210_at	0	4.369	0.82	7	5.203	1.16	40	u	0.0801
			203247_s_at	0	9.827	0.29	7	9.677	0.82	40	d	0.404399
GPR19	1	G protein-coupled receptor 19	207183_at	1	2.219	0.35	7	3.541	1.2	40	u	0.000005
HVCN1	1	hydrogen voltage-gated channel 1	226879_at	1	5.349	0.55	7	5.909	0.81	40	u	0.091075
TAF10	1	TAF10 RNA polymerase II, TATA box binding protein (TBP)-associated factor, 30kDa	200055_at	1	10.66	0.14	7	10.25	0.75	40	d	0.003959
NDUFA2	1	NADH dehydrogenase (ubiquinone) 1 alpha subcomplex, 2, 8kDa	209223_at	1	2.018	0.04	7	2.068	0.12	40	u	0.04759
			222971_at	0	2.139	0.02	7	2.268	0.21	40	u	0.000641
			209224_s_at	0	10.05	0.22	7	9.91	0.64	40	d	0.310049
PHLDA1	1	pleckstrin homology-like domain, family A, member 1	217999_s_at	0	6.404	0.6	7	5.555	0.98	40	d	0.035652
			218000_s_at	0	5.919	0.55	7	5.795	0.8	40	d	0.700271
			217996_at	0	7.56	0.68	7	7.343	1.52	40	d	0.718941
			217998_at	0	6.741	0.79	7	6.334	0.92	40	d	0.287792
			225842_at	0	8.737	0.8	7	7.279	1.28	40	d	0.006503
			217997_at	0	6.595	0.75	7	6.359	1.53	40	d	0.697382
OXTR	1	oxytocin receptor	206825_at	1	10.3	0.65	7	5.253	0.66	40	d	0
USP19	1	ubiquitin specific peptidase 19	214674_at	1	6.643	0.24	7	6.162	0.6	40	d	0.048093
			226357_at	0	8.219	0.2	7	7.62	0.57	40	d	0.000072
TMEM87B	1	transmembrane protein 87B	no probeset available									
WNT3	1	wingless-type MMTV integration site family, member 3	221455_s_at	0	2.057	0.03	7	2.171	0.14	40	u	0.000043
			229103_at	0	4.417	0.27	7	4.474	0.75	40	u	0.846728
			231743_at	0	2.733	0.13	7	2.762	0.28	40	u	0.797725
KIAA1632	1	KIAA1632	227638_at	1	7.161	0.29	7	6.248	1.13	40	d	0.000163
			234048_s_at	0	2.707	0.32	7	2.773	0.35	40	u	0.651349
			228453_at	0	5.617	0.58	7	5.205	1.26	40	d	0.409303
			236108_at	0	3.457	0.25	7	3.547	0.75	40	u	0.574975
			1563471_at	0	3.165	0.4	7	3.327	0.5	40	u	0.43451
			232030_at	0	5	0.62	7	4.551	0.96	40	d	0.250043
			232031_s_at	0	5.406	0.65	7	4.571	1.04	40	d	0.051275
ATF1	1	activating transcription factor 1	222103_at	1	8.58	0.35	7	8.275	0.65	40	d	0.241964
			1558233_s_at	1	6.285	0.3	7	6.677	0.76	40	u	0.19629
			1565269_s_at	0	6.278	0.46	7	6.463	0.82	40	u	0.574562
CLSTN2	1	calsyntenin 2	219414_at	1	4.88	1.32	7	4.941	1.75	40	u	0.932038
ARIH1	1	ariadne homolog, ubiquitin-conjugating enzyme E2 binding protein, 1 (Drosophila)	201881_s_at	1	6.467	0.35	7	6.869	1	40	u	0.072381
			201880_at	0	9.252	0.11	7	8.751	0.68	40	d	0.000103
			201879_at	0	8.064	0.33	7	8.217	0.69	40	u	0.573382
			201878_at	0	7.107	0.25	7	7.421	0.68	40	u	0.24585

WDR6	1	WD repeat domain 6	217734_s_at	3	9.528	0.41	7	8.151	1.03	40	d	0.001325
			233573_s_at	1	3.61	0.9	7	3.548	0.98	40	d	0.879993
KDELC1	1	KDEL (Lys-Asp-Glu-Leu) containing 1	219479_at	1	5.581	0.34	7	5.643	1.01	40	u	0.773193
XRCC6BP1	1	XRCC6 binding protein 1	227678_at	1	5.164	0.5	7	6.12	0.96	40	u	0.015646
			1554349_at	0	1.991	0.04	7	2.03	0.09	40	u	0.267671
LEMD2	1	LEM domain containing 2	224980_at	1	6.269	0.19	7	6.579	0.66	40	u	0.024398
AKNA	1	AT-hook transcription factor	225701_at	1	6.484	0.44	7	6.108	0.93	40	d	0.310361
			237715_at	0	5.187	0.34	7	4.757	0.48	40	d	0.031644
HSPA8	1	heat shock 70kDa protein 8	221891_x_at	1	13.21	0.25	7	13.59	0.56	40	u	0.09215
			224187_x_at	1	13.21	0.25	7	13.42	0.63	40	u	0.421376
			210338_s_at	0	12.62	0.25	7	12.77	1.22	40	u	0.514261
			208687_x_at	0	13.32	0.23	7	13.5	0.71	40	u	0.225931
DIDO1	1	death inducer-obliterator 1	213213_at	1	6.483	0.26	7	6.15	1.09	40	d	0.111859
			222595_s_at	0	6.376	0.26	7	5.874	0.72	40	d	0.003711
			218325_s_at	0	8.41	0.34	7	8.007	0.86	40	d	0.236679
			227335_at	0	6.104	0.42	7	5.941	0.72	40	d	0.572597
CEL	1	carboxyl ester lipase (bile salt-stimulated lipase)	1553970_s_at	1	3.449	1.39	7	3.454	1.25	40	u	0.992512
			205910_s_at	1	3.725	1.02	7	3.674	1.12	40	d	0.912886
DHX15	1	DEAH (Asp-Glu-Ala-His) box polypeptide 15	201385_at	1	11.27	0.29	7	11.41	0.48	40	u	0.49027
			201386_s_at	0	10.36	0.24	7	9.969	0.93	40	d	0.037215
DPF2	1	D4, zinc and double PHD fingers family 2	202116_at	1	8.632	0.22	7	8.17	0.62	40	d	0.002
HOXD13	1	homeobox D13	207397_s_at	0	2.916	0.15	7	3.187	0.72	40	u	0.045896
			236681_at	0	3.687	0.29	7	3.805	0.86	40	u	0.517413
			207398_at	0	2.076	0.02	7	2.203	0.38	40	u	0.047263
ZNF322B	1	zinc finger protein 322B	219376_at	1	6.275	0.55	7	6.791	0.88	40	u	0.149564
FAM54B	1	family with sequence similarity 54, member B	224280_s_at	1	8.606	0.49	7	7.443	0.7	40	d	0.000165
VSIG8	1	V-set and immunoglobulin domain containing 8	no probeset available									
MYH2	1	myosin, heavy chain 2, skeletal muscle, adult	204631_at	1	3.087	0.15	7	3.181	0.31	40	u	0.451028
NAALADL2	1	N-acetylated alpha-linked acidic dipeptidase-like 2	1557998_at	1	3.765	0.35	7	3.706	0.33	40	d	0.672122
PHF1	1	PHD finger protein 1	202928_s_at	1	8.141	0.24	7	6.755	0.67	40	d	0
			40446_at	1	8.927	0.19	7	8.054	0.5	40	d	0.000059
KIAA0892	1	KIAA0892	212505_s_at	1	7.755	0.42	7	7.36	0.64	40	d	0.131398
			227465_at	1	8.713	0.47	7	8.185	0.85	40	d	0.124407
			216926_s_at	0	2.912	0.48	7	3.2	0.85	40	u	0.398186
			217652_at	0	2.165	0.02	7	2.213	0.07	40	u	0.000347
KCNJ13	1	potassium inwardly-rectifying channel, subfamily J, member 13	210179_at	1	2.532	0.05	7	2.65	0.11	40	u	0.011574
			211427_s_at	1	2.559	0.17	7	2.736	0.23	40	u	0.059156
C11orf68	1	chromosome 11 open reading frame 68	221534_at	1	8.233	0.26	7	7.849	0.6	40	d	0.106958
AHCYL1	1	S-adenosylhomocysteine hydrolase-like 1	200849_s_at	1	9.562	0.26	7	9.053	0.58	40	d	0.030256
			200848_at	1	8.524	0.26	7	8.433	0.58	40	d	0.69292
			200850_s_at	1	10.67	0.23	7	10.13	0.47	40	d	0.00502
			207464_at	0	2.898	0.59	7	2.905	0.48	40	u	0.973742
IGLL1	1	immunoglobulin lambda-like polypeptide 1	206660_at	1	3.014	0.32	7	2.923	0.27	40	d	0.442585
PERP	1	PERP, TP53 apoptosis effector	222392_x_at	1	12.38	0.23	7	11.93	0.98	40	d	0.016887
			217744_s_at	0	10.77	0.43	7	10.48	1.43	40	d	0.327151
			1556990_at	0	2.643	0.03	7	2.688	0.09	40	u	0.017383
TNFSF12	1	tumor necrosis factor (ligand) superfamily, member 12	205611_at	1	7.09	0.55	7	5.779	0.63	40	d	0.000007
MTR	1	5-methyltetrahydrofolate-homocysteine methyltransferase	203774_at	1	7.812	0.25	7	7.819	0.51	40	u	0.972002
GANC	1	glucosidase, alpha; neutral C	236077_at	0	4.812	0.39	7	4.956	0.57	40	u	0.531638
			235340_at	0	2.408	0.25	7	2.663	0.47	40	u	0.175238
RSPO2	1	R-spondin 2 homolog (Xenopus laevis)	1554012_at	1	2.414	0.03	7	2.571	0.49	40	u	0.054942
PHACTR1	1	phosphatase and actin regulator 1	213638_at	0	2.345	0.16	7	2.998	0.84	40	u	0.000084
			232045_at	0	2.2	0.16	7	2.25	0.15	40	u	0.445936
BCAP29	1	B-cell receptor-associated protein 29	225677_at	1	8.196	0.35	7	7.831	0.71	40	d	0.196369
			225674_at	1	7.668	0.36	7	8.065	0.76	40	u	0.192036
			230150_at	0	5.303	0.59	7	4.962	0.68	40	d	0.227167
			236970_s_at	0	2.783	0.1	7	2.986	0.17	40	u	0.00541
			205084_at	0	6.733	0.43	7	7.291	0.67	40	u	0.041721
			217657_at	0	3.967	0.15	7	4.146	0.36	40	u	0.209381

GPR132	1	G protein-coupled receptor 132	223887_at	1	3.592	0.47	7	3.389	0.55	40	d	0.372538
			221140_s_at	1	2.845	0.17	7	2.821	0.24	40	d	0.804894
DGKD	1	diacylglycerol kinase, delta 130kDa	208072_s_at	1	8.398	0.5	7	7.732	1.06	40	d	0.116501
LOXL1	1	lysyl oxidase-like 1	203570_at	1	8.413	0.82	7	8.543	1.26	40	u	0.797812
C6orf159	1	chromosome 6 open reading frame 159	236771_at	1	2.49	0.02	7	2.587	0.23	40	u	0.015697
MMP23B	1	matrix metallopeptidase 23B	no probeset available									
PRPF39	1	PRP39 pre-mRNA processing factor 39 homolog (S. cerevisiae)	220553_s_at	1	7.617	0.31	7	7.199	0.71	40	d	0.139205
PROX1	1	prospero homeobox 1	207401_at	1	3.081	0.6	7	2.944	1.11	40	d	0.756564
			228656_at	1	3.588	0.76	7	3.051	0.98	40	d	0.182568
PPP1R1B	1	protein phosphatase 1, regulatory (inhibitor) subunit 1B (dopamine and cAMP regulated phosphoprotein, DARPP-32)	225165_at	1	8.461	0.7	7	7.078	2.93	40	d	0.01609
TIAF1	1	TGFB1-induced anti-apoptotic factor 1	1560553_at	0	2.989	0.12	7	3.133	0.21	40	u	0.088172
SPCS3	1	signal peptidase complex subunit 3 homolog (S. cerevisiae)	222753_s_at	0	7.014	0.67	7	8.052	0.89	40	u	0.005959
			218817_at	0	4.597	0.65	7	6.001	1.1	40	u	0.002425
RASSF4	1	Ras association (RalGDS/AF-6) domain family 4	226436_at	1	6.735	0.34	7	7.168	1.32	40	u	0.094757
			49306_at	0	5.799	0.26	7	6.204	1	40	u	0.04188
			237294_at	0	2.327	0.04	7	2.416	0.17	40	u	0.007345
			221578_at	0	4.803	0.32	7	5.032	0.7	40	u	0.407378
FCMD	1	Fukuyama type congenital muscular dystrophy (fukutin)	205283_at	1	6.643	0.46	7	6.264	0.77	40	d	0.220707
SEC63	1	SEC63 homolog (S. cerevisiae)	201914_s_at	1	8.318	0.29	7	9.116	0.94	40	u	0.000246
			235395_at	1	2.877	0.48	7	3.142	0.76	40	u	0.388445
			201915_at	1	6.481	0.21	7	6.464	0.9	40	d	0.917604
			201916_s_at	1	9.556	0.23	7	9.55	0.71	40	d	0.96758
C9orf7	1	chromosome 9 open reading frame 7	61874_at	1	7.414	0.27	7	7.546	0.52	40	u	0.522371
			219223_at	1	7.023	0.34	7	6.808	0.83	40	d	0.513743
FTH1	1	ferritin, heavy polypeptide 1	214211_at	1	10.53	0.28	7	10.05	0.78	40	d	0.009488
			200748_s_at	1	13.7	0.18	7	13.77	0.66	40	u	0.547083
SYTL4	1	synaptotagmin-like 4 (granuphilin-a)	227703_s_at	1	6.693	0.61	7	5.443	1.51	40	d	0.039694
			229991_s_at	0	5.831	0.59	7	5.75	0.49	40	d	0.703055
			1563513_at	0	3.266	0.41	7	3.456	0.55	40	u	0.393813
C5orf21	1	chromosome 5 open reading frame 21	212936_at	1	7.951	0.17	7	6.574	0.99	40	d	0
			223882_at	0	4.332	0.34	7	4.723	0.75	40	u	0.190554
FANCA	1	Fanconi anemia, complementation group A	203805_s_at	1	2.693	0.48	7	3.989	0.93	40	u	0.001012
			203806_s_at	1	2.636	0.2	7	3.347	0.67	40	u	0.000013
			215530_at	0	5.323	0.17	7	5.49	0.32	40	u	0.190397
			236976_at	0	2.472	0.19	7	3.157	0.85	40	u	0.00008
GANAB	1	glucosidase, alpha; neutral AB	214626_s_at	1	9.926	0.33	7	9.719	0.73	40	d	0.475648
			211934_x_at	1	10.23	0.33	7	9.824	1	40	d	0.062568
TIMM17A	1	translocase of inner mitochondrial membrane 17 homolog A (yeast)	215168_at	1	3.066	0.13	7	3.268	0.28	40	u	0.078082
			215171_s_at	1	9.66	0.26	7	10.87	0.62	40	u	0.000011
			201821_s_at	1	8.315	0.34	7	9.65	0.86	40	u	0.000252
			201822_at	1	4.396	0.64	7	4.798	0.46	40	u	0.055633
PGAM1	1	phosphoglycerate mutase 1 (brain)	200886_s_at	1	11.17	0.21	7	11.44	0.47	40	u	0.151533
KLHL10	1	kelch-like 10 (Drosophila)	1552424_at	1	2.649	0.04	7	2.785	0.2	40	u	0.000521
			1552425_a_at	1	2.354	0.14	7	2.431	0.24	40	u	0.418353
IL1A	1	interleukin 1, alpha	210118_s_at	1	2.588	0.05	7	2.73	0.24	40	u	0.001711
			208200_at	1	3.083	0.39	7	3.094	0.72	40	u	0.970369
PDGFD	1	platelet derived growth factor D	219304_s_at	1	10.11	0.54	7	6.131	1.42	40	d	0
			222860_s_at	1	6.001	0.76	7	4.862	0.59	40	d	0.000061
CACNA1H	1	calcium channel, voltage-dependent, T type, alpha 1H subunit	222960_at	0	5.16	0.36	7	4.772	0.5	40	d	0.061925
			205845_at	0	2.374	0.21	7	2.772	1.22	40	u	0.068585
CAV3	1	caveolin 3	208204_s_at	1	2.938	0.16	7	2.905	0.33	40	d	0.797666
LRRC42	1	leucine rich repeat containing 42	215084_s_at	1	7.18	0.19	7	7.454	0.7	40	u	0.053119
BVES	1	blood vessel epicardial substance	228783_at	1	3.044	0.47	7	3.222	0.83	40	u	0.591133
			223853_at	0	2.067	0.02	7	2.199	0.19	40	u	0.000128
COL5A2	1	collagen, type V, alpha 2	221729_at	1	8.928	0.47	7	10.26	1.3	40	u	0.000101
			221730_at	1	8.414	0.47	7	9.669	1.34	40	u	0.000206
CSPP1	1	centrosome and spindle pole associated protein 1	221925_s_at	1	6.381	0.59	7	6.232	0.79	40	d	0.644512
			227105_at	1	7.356	0.58	7	7.458	1.04	40	u	0.806667
			1554873_at	0	4.121	0.44	7	4.413	0.75	40	u	0.334757

		protein 1	242041_at	0	4.47	0.54	7	5.196	0.97	40	u	0.064484
			220072_at	0	7.957	0.39	7	8.101	0.57	40	u	0.533895
FAM100A	1	family with sequence similarity 100, member A	225874_at	1	7.697	0.35	7	7.582	0.84	40	d	0.72948
			1558975_at	0	4.601	0.64	7	3.963	0.56	40	d	0.010977
			1558976_x_at	0	3.896	0.56	7	3.864	0.56	40	d	0.89223
RABGEF1	1	RAB guanine nucleotide exchange factor (GEF) 1	218310_at	1	7.566	0.22	7	7.015	0.63	40	d	0.000415
THY1	1	Thy-1 cell surface antigen	213869_x_at	1	7.382	0.58	7	7.838	0.97	40	u	0.243427
			208850_s_at	1	7.024	0.68	7	7.924	0.85	40	u	0.012691
			208851_s_at	1	7.974	0.52	7	8.351	0.76	40	u	0.221297
DCLK2	1	doublecortin-like kinase 2	227666_at	1	2.994	0.52	7	3.189	0.58	40	u	0.420054
			216724_at	0	2.123	0.07	7	2.28	0.29	40	u	0.007926
			216725_at	0	5.116	0.43	7	5.106	0.61	40	d	0.969139
DSE	1	dermatan sulfate epimerase	218854_at	1	8.243	0.41	7	8.287	0.9	40	u	0.901236
			1558868_a_at	0	2.724	0.09	7	2.764	0.17	40	u	0.562102
			1558867_at	0	3.231	0.2	7	3.398	0.4	40	u	0.296681
SPAG9	1	sperm associated antigen 9	212468_at	1	6.392	0.43	7	7.159	0.83	40	u	0.024082
			212470_at	1	7.556	0.33	7	8.28	0.8	40	u	0.025491
			225339_at	0	8.467	0.29	7	8.15	0.77	40	d	0.299613
			206748_s_at	0	5.753	0.61	7	6.548	1.32	40	u	0.134121
			1554543_at	0	4.297	1.21	7	4.322	1	40	u	0.954572
NKIRAS1	1	NFKB inhibitor interacting Ras-like 1	225930_at	1	6.936	0.45	7	6.695	0.77	40	d	0.433911
SPP1	1	secreted phosphoprotein 1 (osteopontin, bone sialoprotein I, early T-lymphocyte	1568574_x_at	0	2.409	0.08	7	3.108	0.95	40	u	0.000057
			209875_s_at	0	8.817	1.1	7	11.02	1.83	40	u	0.004093
MIP	1	major intrinsic protein of lens fiber	220863_at	1	2.753	0.14	7	2.87	0.28	40	u	0.290494
IL13RA1	1	interleukin 13 receptor, alpha 1	201888_s_at	1	7.529	0.48	7	8.062	0.79	40	u	0.096418
			211612_s_at	1	7.495	0.73	7	7.835	1.32	40	u	0.518263
			201887_at	1	9.72	0.45	7	9.31	0.7	40	d	0.150399
			210904_s_at	0	7.195	0.82	7	7.71	1.56	40	u	0.407305
GNS	1	glucosamine (N-acetyl)-6-sulfatase (Sanfilippo disease IIID)	212334_at	1	10.08	0.16	7	9.588	0.58	40	d	0.000111
			212335_at	1	9.025	0.29	7	8.903	0.77	40	d	0.688212
			203676_at	0	6.784	0.59	7	6.93	1.2	40	u	0.759018
FDFT1	1	farnesyl-diphosphate farnesyltransferase 1	208647_at	1	10.97	0.81	7	10.1	0.75	40	d	0.008152
			210950_s_at	0	11.4	0.94	7	10.01	0.82	40	d	0.000254
			241954_at	0	7.203	0.78	7	6.399	0.84	40	d	0.025346
BMP7	1	bone morphogenetic protein 7 (osteogenic protein 1)	209590_at	1	4.682	0.39	7	4.628	1.18	40	d	0.830476
			211260_at	0	5.946	0.28	7	5.897	0.34	40	d	0.729851
			211259_s_at	0	3.717	0.45	7	3.637	0.4	40	d	0.643015
			209591_s_at	0	2.635	0.26	7	3.54	1.49	40	u	0.001246
LRP5	1	low density lipoprotein receptor-related protein 5	229591_at	0	2.276	0.13	7	2.257	0.12	40	d	0.702944
			209468_at	0	6.468	0.33	7	6.296	1.11	40	d	0.446192
ARFGEF2	1	ADP-ribosylation factor guanine nucleotide-exchange factor 2 (brefeldin A-inhibited)	222518_at	1	8.523	0.38	7	8.875	0.67	40	u	0.193359
			218098_at	1	9.846	0.48	7	9.805	0.72	40	d	0.889326
			215931_s_at	0	3.725	0.65	7	4.898	1.19	40	u	0.016989
			215927_at	0	3.066	0.13	7	3.153	0.34	40	u	0.515294
NKHD1-EIF4EB	1	ANKHD1-EIF4EBP3	no probeset available									
DND1	1	dead end homolog 1 (zebrafish)	57739_at	1	7.007	0.22	7	7.152	0.44	40	u	0.404649
			222070_at	1	6.497	0.23	7	6.533	0.37	40	u	0.806869
CHN2	1	chimerin (chimaerin) 2	207486_x_at	1	4.111	0.24	7	4.439	0.54	40	u	0.126477
			213385_at	1	3.554	0.78	7	3.78	1.36	40	u	0.679105
			211419_s_at	1	3.949	0.24	7	4.183	0.48	40	u	0.221194
			1557769_at	0	2.379	0.19	7	2.533	0.4	40	u	0.336934
REPS2	1	RALBP1 associated Eps domain containing 2	242571_at	0	3.518	0.63	7	4.119	1.38	40	u	0.273643
			205645_at	0	4.761	0.39	7	5.208	0.96	40	u	0.241722
			227425_at	0	6.374	0.41	7	5.678	1.71	40	d	0.036227
VEZT	1	vezatin, adherens junctions transmembrane protein	223090_x_at	1	8.917	0.39	7	9.17	0.82	40	u	0.436604
			223089_at	1	5.365	0.9	7	5.477	1.58	40	u	0.859563
			223675_s_at	1	7.7	0.4	7	8.275	0.72	40	u	0.050676
			207263_x_at	0	4.721	0.59	7	4.686	0.65	40	d	0.896407
WNK4	1	WNK lysine deficient protein kinase 4	229158_at	1	5.441	1.26	7	4.072	1.73	40	d	0.056475
			244344_at	0	3.18	0.7	7	3.071	0.77	40	d	0.732449
GAD2	1	glutamate decarboxylase 2 (pancreatic islets and brain, 65kDa)	206780_at	1	3.281	0.67	7	3.324	0.71	40	u	0.885945
			216651_s_at	0	2.205	0.03	7	2.318	0.23	40	u	0.00526
			1568601_at	0	2.357	0.04	7	2.533	0.23	40	u	0.000063

		and brain, 65kDa)	211264_at	0	3.044	0.07	7	3.233	0.23	40	u	0.000372
			243265_at	0	2.18	0.04	7	2.22	0.06	40	u	0.096167
NKAP	1	NFKB activating protein	223439_at	1	6.274	0.34	7	6.857	0.77	40	u	0.061271
ZNF468	1	zinc finger protein 468	214751_at	1	7.02	0.28	7	7.398	0.78	40	u	0.036349
RIC8A	1	resistance to inhibitors of cholinesterase 8 homolog A (C. elegans)	221647_s_at	1	9.473	0.16	7	9.145	0.53	40	d	0.004708
SLC30A7	1	solute carrier family 30 (zinc transporter), member 7	240277_at	1	4.895	0.5	7	6.302	0.88	40	u	0.000222
			226217_at	1	8.534	0.25	7	8.625	0.65	40	u	0.721472
			226601_at	0	6.649	0.3	7	7.029	0.66	40	u	0.151492
			239596_at	0	3.856	0.15	7	4.941	0.86	40	u	0
C7orf28A	1	chromosome 7 open reading frame 28A	208310_s_at	3	8.64	0.34	7	9.027	1.04	40	u	0.08602
			201974_s_at	2	3.444	0.21	7	3.826	0.44	40	u	0.030676
			201973_s_at	2	11.01	0.16	7	11.37	0.58	40	u	0.002881
RRAS	1	related RAS viral (r-ras) oncogene homolog	212647_at	1	7.769	0.34	7	7.291	0.78	40	d	0.125801
NPY1R	1	neuropeptide Y receptor Y1	205440_s_at	1	7.405	1.61	7	5.615	3.24	40	d	0.167865
PCDH15	1	protocadherin 15	1553344_at	1	2.332	0.1	7	2.465	0.41	40	u	0.090041
			1560330_at	0	2.656	0.04	7	2.831	0.23	40	u	0.000138
GCNT2	1	glucosaminyl (N-acetyl) transferase 2, I-branching enzyme (I blood group)	230788_at	1	8.049	0.9	7	7.512	2.05	40	d	0.508948
			211020_at	0	2.865	0.73	7	3.51	1.51	40	u	0.285402
LRRC19	1	leucine rich repeat containing 19	220376_at	1	2.693	0.08	7	2.901	0.42	40	u	0.008191
NUDCD2	1	NudC domain containing 2	226643_s_at	1	3.847	0.25	7	4.221	0.9	40	u	0.040211
			226642_s_at	1	8.261	0.3	7	8.529	0.84	40	u	0.153768
			1558124_at	0	3.107	0.08	7	3.188	0.11	40	u	0.069286
CDK4	1	cyclin-dependent kinase 4	202246_s_at	1	9.783	0.16	7	10.2	0.78	40	u	0.005299
KCTD17	1	potassium channel tetramerisation domain containing 17	205561_at	1	5.625	0.21	7	5.72	0.3	40	u	0.43607
			1554566_at	1	2.586	0.28	7	2.663	0.37	40	u	0.605737
TPPP3	1	tubulin polymerization-promoting protein family member 3	218876_at	1	5.213	1.68	7	3.883	1.34	40	d	0.028016
CILP2	1	cartilage intermediate layer protein 2	no probeset available									
PPM2C	1	protein phosphatase 2C, magnesium-dependent, catalytic subunit	222572_at	1	9.14	0.5	7	8.783	0.98	40	d	0.360455
			218273_s_at	0	7.013	0.6	7	7.376	1	40	u	0.367104
RHOV	1	ras homolog gene family, member V	241990_at	0	7.5	0.47	7	6.958	1.09	40	d	0.210555
TRIM42	1	tripartite motif-containing 42	1553620_at	1	2.24	0.02	7	2.296	0.05	40	u	0.003462
			1566852_at	0	2.654	0.11	7	2.912	0.19	40	u	0.001649
			1566851_at	0	1.826	0.04	7	1.865	0.08	40	u	0.225593
RAPSN	1	receptor-associated protein of the synapse	211570_s_at	1	2.304	0.08	7	2.394	0.12	40	u	0.072076
AQP12A	1	aquaporin 12A	1559575_a_at	1	2.154	0.04	7	2.183	0.07	40	u	0.268525
			1554344_s_at	0	5.944	0.26	7	6.32	0.43	40	u	0.034133
TSPYL5	1	TSPY-like 5	213122_at	1	8.711	0.4	7	8.546	1.75	40	d	0.616503
XPOT	1	exportin, tRNA (nuclear export receptor for tRNAs)	212160_at	1	8.529	0.21	7	10	0.72	40	u	0
CRAT	1	carnitine acetyltransferase	205843_x_at	1	7.306	0.54	7	6.923	0.62	40	d	0.143056
			209522_s_at	1	8.306	1.12	7	7.28	1.51	40	d	0.099067
TRUB1	1	TruB pseudouridine (psi) synthase homolog 1 (E. coli)	226339_at	1	8.204	0.32	7	8.659	0.55	40	u	0.042504
			235447_at	0	3.205	0.2	7	3.805	0.74	40	u	0.000176
			236020_s_at	0	1.903	0.04	7	1.932	0.04	40	u	0.12746
			241606_s_at	0	5.456	0.1	7	5.538	0.51	40	u	0.380769
			241605_at	0	3.105	0.22	7	3.083	0.22	40	d	0.807641
ENPP2	1	ectonucleotide pyrophosphatase/phosphodiesterase 2	209392_at	1	9.946	0.65	7	7.189	1.43	40	d	0.000013
			210839_s_at	0	8.641	0.77	7	5.774	1.44	40	d	0.000009
LRRC8E	1	leucine rich repeat containing 8 family, member E	239433_at	1	5.205	0.61	7	5.1	1.06	40	d	0.803393
			220174_at	1	5.32	0.59	7	5.424	0.96	40	u	0.789005
EOMES	1	eomesodermin homolog (Xenopus laevis)	231776_at	1	3.857	0.62	7	4.411	1.28	40	u	0.275506
PSEN2	1	presenilin 2 (Alzheimer disease 4)	204261_s_at	1	3.813	0.36	7	4.167	0.6	40	u	0.14647
			204262_s_at	1	4.494	0.59	7	5.503	0.75	40	u	0.0019
			211373_s_at	1	6.184	0.24	7	6.765	0.8	40	u	0.001111
ATP6V1E1	1	ATPase, H+ transporting, lysosomal 31kDa, V1 subunit E1	208678_at	1	10.42	0.15	7	10.07	0.71	40	d	0.008275
TMEFF2	1	transmembrane protein with EGF-like and two follistatin-like domains 2	233910_at	1	2.902	0.1	7	3.076	0.23	40	u	0.059323
			223557_s_at	0	2.579	0.08	7	2.723	0.14	40	u	0.013097
			224321_at	0	6.698	0.54	7	7.236	1.58	40	u	0.120798
PIGK	1	phosphatidylinositol glycan anchor biosynthesis, class K	227639_at	1	8.341	0.41	7	7.546	0.74	40	d	0.009371
			1555394_at	0	2.868	0.12	7	3.01	0.25	40	u	0.161231

		biosynthesis, class K	209707_at	0	7.932	0.37	7	7.861	0.84	40	d	0.831417
PLS3	1	plastin 3 (T isoform)	201215_at	1	10.63	0.39	7	10.27	1.16	40	d	0.153011
SLC9A3R2	1	solute carrier family 9 (sodium/hydrogen exchanger), member 3 regulator 2	209830_s_at	0	5.475	0.25	7	5.619	0.69	40	u	0.351633
TPST2	1	tyrosylprotein sulfotransferase 2	204079_at	0	6.648	0.65	7	6.443	0.96	40	d	0.596438
MRPL9	1	mitochondrial ribosomal protein L9	209609_s_at	1	9.668	0.22	7	10.53	0.78	40	u	0.000003
			211594_s_at	0	8.9	0.14	7	9.435	0.93	40	u	0.001741
MRC1	1	mannose receptor, C type 1	204438_at	2	8.295	0.98	7	6.018	1.27	40	d	0.00006
KCNIP1	1	Kv channel interacting protein 1	221307_at	0	2.221	0.04	7	2.269	0.2	40	u	0.180735
MDFI	1	MyoD family inhibitor	205375_at	1	6.57	0.7	7	6.938	1.28	40	u	0.470477
POU2F2	1	POU class 2 homeobox 2	228343_at	1	3.195	0.38	7	3.17	0.53	40	d	0.904909
			211771_s_at	1	3.645	0.59	7	3.498	0.58	40	d	0.550504
			211660_at	0	5.765	0.19	7	5.745	0.28	40	d	0.860532
			229750_at	0	5.71	0.38	7	5.434	0.5	40	d	0.177992
C10orf83	1	chromosome 10 open reading frame 83	228354_at	1	6.545	0.41	7	6.171	0.66	40	d	0.163196
			236813_at	0	4.673	0.41	7	4.461	0.65	40	d	0.418091
TRMT11	1	tRNA methyltransferase 11 homolog (S. cerevisiae)	218877_s_at	1	8.277	0.43	7	8.287	0.85	40	u	0.976039
RHBDL1	1	rhomboid, veinlet-like 1 (Drosophila)	207383_s_at	1	3.06	0.3	7	3.106	0.55	40	u	0.833935
NME4	1	non-metastatic cells 4, protein expressed in	212739_s_at	1	9.494	0.1	7	10.12	0.75	40	u	0.000013
HABP2	1	hyaluronan binding protein 2	206010_at	1	2.966	0.41	7	3.109	0.6	40	u	0.554164
ACCN4	1	amiloride-sensitive cation channel 4, pituitary	221226_s_at	1	4.599	0.35	7	4.427	0.32	40	d	0.209552
FHOD3	1	formin homology 2 domain containing 3	218980_at	1	6.583	0.7	7	4.026	1.3	40	d	0.00001
ZFP91-CNTF	1	ZFP91-CNTF	no probeset available									
BBX	1	bobby sox homolog (Drosophila)	223134_at	1	7.835	0.27	7	8.229	0.73	40	u	0.171072
			232008_s_at	0	8.743	0.22	7	8.611	0.63	40	d	0.340636
			223135_s_at	0	8.428	0.17	7	8.25	0.79	40	d	0.221955
EFEMP1	1	EGF-containing fibulin-like extracellular matrix protein 1	201842_s_at	1	11.7	0.36	7	8.971	1.53	40	d	0
			201843_s_at	1	8.533	0.47	7	5.728	1.7	40	d	0
			228421_s_at	0	1.748	0.03	7	1.834	0.42	40	u	0.209633
TNRC6B	1	trinucleotide repeat containing 6B	213254_at	1	7.628	0.26	7	6.718	0.71	40	d	0.001979
			238468_at	0	6.661	0.76	7	6.238	0.88	40	d	0.246706
			230779_at	0	6.526	0.66	7	6.043	1.06	40	d	0.25973
			229036_at	0	7.67	0.44	7	7.079	0.81	40	d	0.073669
			240044_x_at	0	5.373	0.68	7	4.902	0.77	40	d	0.146041
			228998_at	0	5.249	0.37	7	5.124	0.85	40	d	0.709317
			1558142_at	0	8.055	0.42	7	7.594	0.82	40	d	0.160333
ALS2	1	amyotrophic lateral sclerosis 2 (juvenile)	226291_at	1	7.316	0.25	7	7.03	0.77	40	d	0.086922
			1555284_at	0	2.79	0.21	7	3.103	0.42	40	u	0.063675
			232184_at	0	3.99	0.48	7	4.254	0.65	40	u	0.318753
PTDSS1	1	phosphatidylserine synthase 1	201433_s_at	1	9.563	0.2	7	10.2	0.86	40	u	0.000306
COX6A1	1	cytochrome c oxidase subunit VIa polypeptide 1	200925_at	1	12.69	0.19	7	12.92	0.44	40	u	0.20569
			224344_at	0	1.981	0.04	7	2.024	0.09	40	u	0.245456
			234876_at	0	1.996	0.1	7	2.048	0.12	40	u	0.293129
MYO1E	1	myosin IE	203072_at	1	6.087	0.24	7	5.817	0.77	40	d	0.097202
NUP35	1	nucleoporin 35kDa	225470_at	1	8.061	0.3	7	8.165	0.52	40	u	0.619699
KLHDC8A	1	kelch domain containing 8A	219331_s_at	1	2.421	0.52	7	2.518	0.88	40	u	0.781914
			222871_at	1	4.159	0.19	7	4.567	0.94	40	u	0.02075
CENTD1	1	centaurin, delta 1	213618_at	1	6.501	0.55	7	6.387	1.21	40	d	0.81124
			1561407_at	0	2.582	0.1	7	2.745	0.18	40	u	0.029257
			214102_at	0	7.451	0.76	7	5.971	0.79	40	d	0.000049
IQGAP3	1	IQ motif containing GTPase activating protein 3	229538_s_at	1	3.863	0.68	7	6.715	1.18	40	u	0
			1569061_at	1	3.555	0.34	7	3.62	0.43	40	u	0.71136
			1569062_s_at	1	2.196	0.02	7	2.359	0.47	40	u	0.038552
			241939_at	0	3.444	0.25	7	3.442	0.33	40	d	0.986359
LYPLA3	1	lysophospholipase 3 (lysosomal phospholipase A2)	204458_at	1	5.406	0.29	7	5.364	0.67	40	d	0.873427
PDZK1IP1	1	PDZK1 interacting protein 1	219630_at	1	7.966	0.48	7	6.113	2.38	40	d	0.000088
			1553589_a_at	0	7.524	0.43	7	6.121	2.05	40	d	0.000517
INPPL1	1	inositol polyphosphate phosphatase-like 1	201598_s_at	1	7.316	0.15	7	6.877	0.72	40	d	0.001612
SKP1A	1	S-phase kinase-associated protein 1A (p19A)	200719_at	1	8.75	0.38	7	7.938	0.66	40	d	0.003495
			200718_s_at	1	12.8	0.15	7	12.57	0.61	40	d	0.059987

SKP1A	1	S-phase kinase-associated protein 1A (p19A)	200711_s_at	1	10.35	0.26	7	9.805	0.65	40	d	0.037617
			207974_s_at	0	11.83	0.23	7	11.3	0.76	40	d	0.001703
DDEF2	1	development and differentiation enhancing factor 2	206414_s_at	1	7.397	0.22	7	7.718	1.08	40	u	0.107949
COL9A1	1	collagen, type IX, alpha 1	1555527_at	0	2.092	0.02	7	2.164	0.11	40	u	0.000884
			222008_at	0	2.124	0.02	7	2.407	0.83	40	u	0.040033
VPS35	1	vacuolar protein sorting 35 homolog (S. cerevisiae)	217727_x_at	1	11.29	0.14	7	11.29	0.52	40	u	0.976976
			222387_s_at	1	7.875	0.45	7	8.995	1.02	40	u	0.007836
			233864_s_at	1	10.2	0.17	7	10.45	0.56	40	u	0.036594
			222388_s_at	1	10.02	0.26	7	10.38	0.61	40	u	0.142182
			1561146_at	0	2.487	0.1	7	2.586	0.28	40	u	0.112434
ASNA1	1	arsA arsenite transporter, ATP-binding, homolog 1 (bacterial)	202024_at	1	8.339	0.18	7	8.471	0.89	40	u	0.416829
TTC19	1	tetratricopeptide repeat domain 19	217964_at	1	10	0.17	7	9.219	0.69	40	d	0.000001
TCF7L1	1	transcription factor 7-like 1 (T-cell specific, HMG-box)	221016_s_at	1	8.082	0.66	7	6.803	1.78	40	d	0.071911
GFPT1	1	glutamine-fructose-6-phosphate transaminase 1	202721_s_at	1	7.032	0.53	7	8.131	1.12	40	u	0.016383
			202722_s_at	1	8.45	0.26	7	9.499	0.8	40	u	0.000001
HMOX1	1	heme oxygenase (decycling) 1	203665_at	1	5.858	0.77	7	7.364	1.21	40	u	0.003254
DTX2	1	deltex homolog 2 (Drosophila)	215732_s_at	1	6.183	0.33	7	6.321	0.58	40	u	0.555284
DYRK3	1	dual-specificity tyrosine-(Y)-phosphorylation regulated kinase 3	210151_s_at	0	6.333	0.67	7	6.03	1.04	40	d	0.468686
			1560785_at	0	2.611	0.12	7	2.708	0.15	40	u	0.127165
PKIG	1	protein kinase (cAMP-dependent, catalytic) inhibitor gamma	202732_at	1	9.095	0.3	7	8.039	0.94	40	d	0.000008
NUCKS1	1	nuclear casein kinase and cyclin-dependent kinase substrate 1	222424_s_at	1	8.685	0.42	7	9.059	0.75	40	u	0.216942
			217802_s_at	0	11.06	0.31	7	11.56	0.47	40	u	0.010639
			229353_s_at	0	10.3	0.22	7	11.17	0.68	40	u	0.000001
			222027_at	0	5.398	0.22	7	6.624	0.73	40	u	0
			226880_at	0	10.87	0.18	7	11.3	0.86	40	u	0.008092
GALNTL4	1	UDP-N-acetyl-alpha-D-galactosamine:polypeptide N-	1554079_at	1	6.351	0.55	7	6.864	1.13	40	u	0.25647
			233188_at	0	2.633	0.09	7	2.755	0.12	40	u	0.014747
TMEM68	1	transmembrane protein 68	226483_at	1	6.203	0.29	7	7.028	0.97	40	u	0.000203
			227936_at	0	6.231	0.53	7	6.496	1.12	40	u	0.549681
NXN	1	nucleoredoxin	219489_s_at	0	8.928	0.58	7	8.717	1.23	40	d	0.667204
ZIC5	1	Zic family member 5 (odd-paired homolog, Drosophila)	1552938_at	1	2.669	0.12	7	2.696	0.11	40	u	0.588171
BPNT1	1	3'(2'), 5'-bisphosphate nucleotidase 1	1554575_a_at	1	4.823	0.49	7	6.002	1.02	40	u	0.005306
			232103_at	1	5.782	0.44	7	6.854	0.75	40	u	0.000834
			219391_at	1	2.571	0.33	7	2.563	0.25	40	d	0.943942
CXorf15	1	chromosome X open reading frame 15	219969_at	1	3.606	0.21	7	3.839	1.04	40	u	0.218894
			227520_at	0	7.854	0.22	7	7.672	0.65	40	d	0.197693
			1557954_at	0	7.662	0.3	7	7.439	0.8	40	d	0.476206
ACTR3B	1	ARP3 actin-related protein 3 homolog B (yeast)	218868_at	1	6.699	0.52	7	7.627	1.64	40	u	0.010506
			1555487_a_at	0	5.181	0.58	7	5.894	1.8	40	u	0.067244
			223874_at	0	5.002	0.63	7	5.086	0.71	40	u	0.776289
ARX	1	aristaless related homeobox	238878_at	1	2.625	0.79	7	2.905	1.69	40	u	0.675383
			231020_at	1	2.68	0.03	7	2.792	0.18	40	u	0.000688
ANGPTL4	1	angiopoietin-like 4	221009_s_at	1	7.013	0.85	7	5.568	1.26	40	d	0.006235
			223333_s_at	0	6.431	0.8	7	4.957	0.82	40	d	0.00009
VKORC1L1	1	vitamin K epoxide reductase complex, subunit 1-like 1	1553679_s_at	1	4.052	0.31	7	4.897	0.98	40	u	0.000254
			224881_at	0	6.887	0.29	7	7.196	0.84	40	u	0.099162
TBCEL	1	tubulin folding cofactor E-like	231997_at	0	3.073	0.39	7	3.187	0.51	40	u	0.585556
NGEF	1	neuronal guanine nucleotide exchange factor	227240_at	1	5.546	0.4	7	5.985	0.84	40	u	0.190759
			243556_at	0	1.745	0.02	7	1.788	0.07	40	u	0.003413
GLRA1	1	glycine receptor, alpha 1 (startle disease/hyperekplexia)	207972_at	1	2.121	0.02	7	2.173	0.07	40	u	0.000312
STK36	1	serine/threonine kinase 36, fused homolog (Drosophila)	234005_x_at	1	6.735	0.21	7	6.412	1.06	40	d	0.096594
			226166_x_at	1	7.281	0.35	7	6.819	1.23	40	d	0.0671
			231806_s_at	1	7.452	0.26	7	6.926	1.18	40	d	0.02003
TRPC5	1	transient receptor potential cation channel, subfamily C, member 5	220552_at	1	2.658	0.07	7	2.815	0.22	40	u	0.00124
C1orf26	1	chromosome 1 open reading frame 26	223548_at	1	6.066	0.48	7	6.162	0.57	40	u	0.685199
TULP1	1	tubby like protein 1	206705_at	1	2.055	0.07	7	2.158	0.16	40	u	0.116422
GTF2A2	1	general transcription factor IIA, 2, 12kDa	202678_at	1	9.259	0.25	7	9.407	0.61	40	u	0.535396

SPI1	1	spleen focus forming virus (SFFV) proviral integration oncogene spi1	205312_at	1	5.303	0.44	7	5.773	0.72	40	u	0.108295
EID1	1	EP300 interacting inhibitor of differentiation 1	211698_at	1	8.757	0.15	7	7.995	1.24	40	d	0.000645
			208669_s_at	0	10.88	0.15	7	9.707	1.3	40	d	0.000003
			208670_s_at	0	8.387	0.22	7	7.493	1.35	40	d	0.000402
C8orf46	1	chromosome 8 open reading frame 46	229430_at	1	3.102	0.34	7	3.878	1.67	40	u	0.014115
			228701_at	1	2.498	0.09	7	2.709	0.66	40	u	0.064378
			228695_at	1	2.3	0.08	7	3.187	1.57	40	u	0.001169
PPP2R5B	1	protein phosphatase 2, regulatory subunit B', beta isoform	635_s_at	1	5.961	0.29	7	5.711	0.63	40	d	0.320972
			204611_s_at	1	7.425	0.19	7	6.969	0.54	40	d	0.000696
ATG2A	1	ATG2 autophagy related 2 homolog A (S. cerevisiae)	213300_at	1	8.19	0.28	7	7.258	0.9	40	d	0.000021
SEC24B	1	SEC24 related gene family, member B (S. cerevisiae)	202798_at	1	9.201	0.23	7	8.904	0.59	40	d	0.202015
PPFIA2	1	protein tyrosine phosphatase, receptor type, f polypeptide (PTPRF), interacting protein (liprin), alpha 2	206973_at	0	2.359	0.05	7	2.418	0.11	40	u	0.191925
			232073_at	0	2.77	0.12	7	2.982	0.27	40	u	0.050939
			1558295_a_at	0	2.229	0.16	7	2.331	0.21	40	u	0.243646
ADAM28	1	ADAM metallopeptidase domain 28	205997_at	0	6.483	0.75	7	5.866	1.62	40	d	0.33744
			241446_at	0	2.686	0.08	7	2.998	0.46	40	u	0.000352
			208269_s_at	0	4.937	0.64	7	5.269	0.63	40	u	0.213182
			208268_at	0	4.119	0.58	7	4.283	1.24	40	u	0.738518
ODZ1	1	odz, odd Oz/ten-m homolog 1(Drosophila)	1553007_a_at	1	2.206	0.02	7	2.335	0.2	40	u	0.000304
THBS4	1	thrombospondin 4	204776_at	1	6.996	0.88	7	5.948	1	40	d	0.014046
DOK1	1	docking protein 1, 62kDa (downstream of tyrosine kinase 1)	216835_s_at	1	7.294	0.2	7	7.051	0.39	40	d	0.118634
			211121_s_at	0	5.744	0.4	7	5.657	0.78	40	d	0.780138
B4GALNT3	1	beta-1,4-N-acetyl-galactosaminyl transferase 3	1553727_at	1	5.26	0.3	7	5.395	0.31	40	u	0.299607
			229909_at	1	5.486	0.52	7	5.948	0.88	40	u	0.192831
			229203_at	0	3.794	0.29	7	3.888	0.39	40	u	0.556683
UNC5C	1	unc-5 homolog C (C. elegans)	206189_at	1	2.334	0.25	7	2.337	0.22	40	u	0.970675
			240958_at	0	2.791	0.21	7	2.766	0.29	40	d	0.837439
			1555342_a_at	0	2.02	0.04	7	2.082	0.06	40	u	0.012238
			1555341_at	0	2.332	0.05	7	2.404	0.17	40	u	0.042075
ARL4D	1	ADP-ribosylation factor-like 4D	203586_s_at	1	6.603	0.36	7	5.746	0.43	40	d	0.000014
			203587_at	1	2.544	0.03	7	2.63	0.17	40	u	0.005758
CTLA4	1	cytotoxic T-lymphocyte-associated protein 4	231794_at	1	4.867	0.6	7	4.767	0.65	40	d	0.710793
			234362_s_at	0	2.883	0.16	7	3.161	0.61	40	u	0.021618
			221331_x_at	0	2.569	0.38	7	2.989	0.66	40	u	0.115733
			234895_at	0	2.419	0.02	7	2.536	0.2	40	u	0.000952
			236341_at	0	2.39	0.33	7	2.884	0.9	40	u	0.019221
ACSBG1	1	acyl-CoA synthetase bubblegum family member 1	206466_at	1	2.997	0.22	7	3.164	0.31	40	u	0.185924
			206465_at	1	2.357	0.73	7	1.981	0.03	40	d	0.255469
UBAC2	1	UBA domain containing 2	224298_s_at	1	9.026	0.22	7	8.852	0.7	40	d	0.235703
SIRPA	1	signal-regulatory protein alpha	202897_at	1	7.794	0.47	7	6.361	1.1	40	d	0.001746
			202896_s_at	1	6.629	0.38	7	6.338	1.06	40	d	0.216161
			202895_s_at	0	5.603	0.57	7	5.439	0.92	40	d	0.655902
			217024_x_at	0	3.508	0.41	7	3.705	0.77	40	u	0.522017
PLCD1	1	phospholipase C, delta 1	205125_at	1	8.154	0.28	7	8.07	0.65	40	d	0.743331
ARHGEF15	1	Rho guanine nucleotide exchange factor (GEF) 15	205507_at	1	4.772	1.29	7	3.972	0.6	40	d	0.184763
			217348_x_at	0	4.562	0.18	7	4.362	0.42	40	d	0.227621
RAB3GAP2	1	RAB3 GTPase activating protein subunit 2 (non-catalytic)	202373_s_at	1	10.16	0.18	7	9.973	0.61	40	d	0.135515
			243851_at	0	4.729	0.36	7	4.817	0.82	40	u	0.786229
			202374_s_at	0	7.724	0.26	7	7.729	0.98	40	u	0.978972
			216057_at	0	3.639	0.31	7	3.838	0.58	40	u	0.386397
SLC37A2	1	solute carrier family 37 (glycerol-3-phosphate transporter), member 2	238638_at	1	5.961	0.16	7	5.931	0.42	40	d	0.856749
SORCS3	1	sortilin-related VPS10 domain containing receptor 3	215522_at	1	2.565	0.06	7	2.769	0.4	40	u	0.004796
NAPSA	1	napsin A aspartic peptidase	223806_s_at	0	2.883	0.43	7	2.907	0.54	40	u	0.915127
IL4	1	interleukin 4	207538_at	1	2.375	0.05	7	2.464	0.11	40	u	0.04015
			207539_s_at	1	2.32	0.09	7	2.416	0.24	40	u	0.310073
C2orf43	1	chromosome 2 open reading frame 43	222192_s_at	1	5.971	0.36	7	6.211	0.96	40	u	0.525022
			222193_at	1	4.129	0.82	7	4.074	1.07	40	d	0.899061
			219008_at	1	6.059	0.39	7	6.046	1.15	40	d	0.958153
GPRIN1	1	G protein regulated inducer of neurite	227975_at	1	2.309	0.43	7	3.174	1.19	40	u	0.002853

[illegible]	[illegible]	outgrowth 1	229922_at	0	2.211	0.02	7	2.277	0.08	40	u	0.000149
BRD7	1	bromodomain containing 7	222737_s_at	1	9.069	0.19	7	9.242	0.64	40	u	0.184486
			238545_at	0	3.158	0.21	7	3.188	0.44	40	u	0.862531
			221093_at	0	2.598	0.07	7	2.677	0.13	40	u	0.120145
			221776_s_at	0	8.211	0.18	7	8.441	0.51	40	u	0.049199
NISCH	1	nischarin	201591_s_at	1	10.62	0.34	7	8.794	0.84	40	d	0.000001
			227392_at	0	3.634	0.47	7	3.081	0.62	40	d	0.032599
TCEB1	1	transcription elongation factor B (SIII), polypeptide 1 (15kDa, elongin C)	202824_s_at	1	9.529	0.26	7	10.55	0.74	40	u	0.000001
			202823_at	0	6.133	0.32	7	7.275	0.84	40	u	0.001116
LINGO2	1	leucine rich repeat and Ig domain containing 2	232720_at	1	2.547	0.35	7	2.544	0.35	40	d	0.986358
WDR7	1	WD repeat domain 7	212880_at	1	7.261	0.21	7	6.924	0.78	40	d	0.033381
AMBN	1	ameloblastin (enamel matrix protein)	221114_at	0	2.283	0.03	7	2.438	0.22	40	u	0.000169
DYNLL1	1	dynein, light chain, LC8-type 1	200703_at	1	11.36	0.19	7	11.84	0.41	40	u	0.004293
KRTAP4-4	1	keratin associated protein 4-4	234684_s_at	1	2.313	0.05	7	2.371	0.1	40	u	0.156835
MYF6	1	myogenic factor 6 (herculin)	206372_at	1	2.596	0.14	7	2.776	0.29	40	u	0.125881
DNAJC8	1	DnaJ (Hsp40) homolog, subfamily C, member 8	205545_x_at	1	9.566	0.21	7	9.129	0.52	40	d	0.037851
			212491_s_at	0	7.874	0.19	7	7.339	0.62	40	d	0.000208
			212490_at	0	5.205	0.45	7	5.001	0.48	40	d	0.308715
GOLGA2	1	golgi autoantigen, golgin subfamily a, 2	225672_at	1	8.566	0.23	7	8.521	0.52	40	d	0.827767
			211059_s_at	0	7.998	0.19	7	7.841	0.46	40	d	0.39367
			35436_at	0	8.698	0.26	7	8.795	0.72	40	u	0.54351
			204384_at	0	6.632	0.43	7	6.666	0.84	40	u	0.919075
PIK3R2	1	phosphoinositide-3-kinase, regulatory subunit 2 (p85 beta)	229392_s_at	1	4.507	0.4	7	4.649	0.49	40	u	0.482782
			207105_s_at	1	5.492	0.42	7	5.753	0.81	40	u	0.417518
			1568629_s_at	0	8.629	0.39	7	8.997	0.72	40	u	0.204144
ANKRD44	1	ankyrin repeat domain 44	235777_at	0	2.676	0.05	7	2.958	0.38	40	u	0.000061
			235778_s_at	0	2.556	0.06	7	2.674	0.22	40	u	0.01041
			233361_at	0	2.745	0.23	7	3.073	0.42	40	u	0.053199
BMP1	1	bone morphogenetic protein 1	202701_at	1	6.69	0.23	7	6.373	0.54	40	d	0.142646
			1569002_x_at	0	2.53	0.41	7	2.442	0.19	40	d	0.622288
			205574_x_at	0	5.607	0.68	7	6.006	0.86	40	u	0.258137
			1569001_at	0	2.799	0.2	7	2.555	0.29	40	d	0.03941
			207595_s_at	0	5.267	0.55	7	5.665	0.47	40	u	0.053051
			206725_x_at	0	4.922	0.4	7	5.079	0.44	40	u	0.395691
			1570383_at	0	2.29	0.02	7	2.339	0.05	40	u	0.00009
CPNE6	1	copine VI (neuronal)	215700_x_at	1	3.658	0.53	7	3.791	0.51	40	u	0.543181
			210408_s_at	1	2.329	0.23	7	2.26	0.19	40	d	0.416761
RASL11B	1	RAS-like, family 11, member B	219142_at	1	4.577	0.72	7	5.188	1.24	40	u	0.221499
			234102_at	0	1.942	0.04	7	2.107	0.59	40	u	0.092873
UXS1	1	UDP-glucuronate decarboxylase 1	225583_at	1	7.747	0.21	7	8.105	0.64	40	u	0.011632
			219675_s_at	1	7.298	0.11	7	7.704	0.74	40	u	0.002594
CHST7	1	carbohydrate (N-acetylglucosamine 6-O) sulfotransferase 7	206756_at	1	3.862	0.68	7	3.457	0.62	40	d	0.132691
ZCCHC17	1	zinc finger, CCHC domain containing 17	223108_s_at	1	7.535	0.33	7	7.634	0.54	40	u	0.647764
			223107_s_at	1	8.413	0.26	7	8.209	0.66	40	d	0.436007
KIAA1754	1	KIAA1754	225582_at	1	6.532	0.46	7	6.018	0.71	40	d	0.07529
GTF2B	1	general transcription factor IIB	208066_s_at	1	8.791	0.35	7	8.396	0.79	40	d	0.210712
DMRT2	1	doublesex and mab-3 related transcription factor 2	223704_s_at	1	2.818	0.27	7	2.623	0.22	40	d	0.04439
			223986_x_at	0	2.653	0.12	7	2.698	0.23	40	u	0.6263
DHRS4L2	1	drogenase/reductase (SDR family) member 4	no probeset available									
GLDC	1	glycine dehydrogenase (decarboxylating)	204836_at	1	3.741	0.41	7	4.922	1.59	40	u	0.000419
TMEM97	1	transmembrane protein 97	214283_at	3	6.111	0.55	7	6.935	0.47	40	u	0.000196
			212279_at	1	7.479	0.65	7	8.667	1.13	40	u	0.011128
			212281_s_at	1	7.389	0.48	7	8.439	1.05	40	u	0.014763
			212282_at	1	7.451	0.56	7	8.395	1.02	40	u	0.024712
SUZ12	1	suppressor of zeste 12 homolog (Drosophila)	212287_at	1	8.886	0.23	7	9.451	0.72	40	u	0.000699
			1566191_at	0	3.488	0.42	7	4.106	0.77	40	u	0.047502
			1566190_at	0	2.974	0.17	7	3.098	0.24	40	u	0.206228
			213971_s_at	0	7.729	0.22	7	8.529	0.57	40	u	0.000768
EXTL2	1	exostoses (multiple)-like 2	209537_at	1	8.034	0.52	7	7.471	0.74	40	d	0.066082
VARS	1	valyl-tRNA synthetase	201797_s_at	0	6.868	0.43	7	7.115	0.92	40	u	0.500067
			201796_s_at	0	5.642	0.53	7	5.834	1.08	40	u	0.653423
RAX	1	retina and anterior neural fold homeobox	208242_at	0	2.489	0.16	7	2.452	0.15	40	d	0.565404

CCL1	1	chemokine (C-C motif) ligand 1	207533_at	1	2.258	0.07	7	2.38	0.45	40	u	0.123397
MFAP2	1	microfibrillar-associated protein 2	203417_at	1	6.516	0.68	7	8.713	1.25	40	u	0.000057
C10orf118	1	chromosome 10 open reading frame 118	227701_at	1	7.382	0.34	7	6.923	0.7	40	d	0.103462
			229399_at	0	5.759	0.52	7	6.967	1.07	40	u	0.006348
			219844_at	0	4.347	0.42	7	5.279	1.03	40	u	0.025926
ARPC4	1	actin related protein 2/3 complex, subunit 4, 20kDa	217817_at	1	9.286	0.18	7	9.314	0.74	40	u	0.841921
			211672_s_at	0	8.429	0.57	7	8.03	1.49	40	d	0.497869
			217818_s_at	0	7.156	0.3	7	6.761	1.26	40	d	0.103755
CAMK4	1	calcium/calmodulin-dependent protein kinase IV	241871_at	1	2.986	0.11	7	3.476	0.62	40	u	0.00005
			210349_at	1	2.568	0.17	7	2.675	0.21	40	u	0.216384
C6orf174	1	chromosome 6 open reading frame 174	233050_at	1	2.054	0.03	7	2.176	0.28	40	u	0.010559
			1569146_s_at	0	1.906	0.04	7	1.95	0.07	40	u	0.119298
CLPX	1	ClpX caseinolytic peptidase X homolog (E. coli)	204809_at	1	7.02	0.34	7	7.583	0.6	40	u	0.022024
			223507_at	0	8.117	0.31	7	7.936	0.52	40	d	0.383718
C22orf32	1	chromosome 22 open reading frame 32	227648_at	1	2.824	0.11	7	2.873	0.18	40	u	0.505844
			225794_s_at	1	10.67	0.24	7	9.82	0.69	40	d	0.000006
			225795_at	1	10.42	0.35	7	9.547	0.75	40	d	0.004701
			228117_at	0	3.896	0.65	7	3.667	0.52	40	d	0.319119
ZFP2	1	zinc finger protein 2 homolog (mouse)	207757_at	1	3.564	0.58	7	2.954	0.38	40	d	0.001128
BZW2	1	basic leucine zipper and W2 domains 2	217809_at	1	10.05	0.27	7	10.53	0.6	40	u	0.048802
PTPRN	1	protein tyrosine phosphatase, receptor type, N	204945_at	1	3.523	0.22	7	3.597	0.79	40	u	0.635434
HTATSF1	1	HIV-1 Tat specific factor 1	202601_s_at	1	5.927	0.29	7	6.935	0.94	40	u	0.000015
			202602_s_at	1	9.219	0.24	7	9.638	0.83	40	u	0.016382
C20orf103	1	chromosome 20 open reading frame 103	219463_at	1	4.256	1.01	7	3.918	1.67	40	d	0.614816
FAM40B	1	family with sequence similarity 40, member B	231880_at	1	2.731	0.12	7	2.957	0.42	40	u	0.010921
			1555292_at	0	2.399	0.11	7	2.585	0.37	40	u	0.01661
GLRX	1	glutaredoxin (thioltransferase)	209276_s_at	0	8.085	0.36	7	8.766	1.33	40	u	0.013003
			206662_at	0	8.875	0.23	7	9.449	0.92	40	u	0.002288
SLC29A1	1	solute carrier family 29 (nucleoside transporters), member 1	201802_at	1	8.612	0.39	7	8.108	1.53	40	d	0.093862
			201801_s_at	0	8.631	0.43	7	8.13	1.84	40	d	0.151036
ALG8	1	asparagine-linked glycosylation 8 homolog (S. cerevisiae, alpha-1,3-glucosyltransferase)	203545_at	1	8.49	0.21	7	8.777	1.19	40	u	0.177178
ST3GAL4	1	ST3 beta-galactoside alpha-2,3-sialyltransferase 4	203759_at	1	5.662	0.28	7	6.33	0.98	40	u	0.001568
			215710_at	0	2.66	0.1	7	2.738	0.16	40	u	0.226139
SHOC2	1	soc-2 suppressor of clear homolog (C. elegans)	202777_at	1	9.133	0.34	7	9.036	0.71	40	d	0.732618
MRPL27	1	mitochondrial ribosomal protein L27	224330_s_at	1	9.488	0.21	7	9.741	0.95	40	u	0.154533
VASN	1	vasorin	225867_at	1	6.718	0.49	7	6.175	1.1	40	d	0.217184
SSR1	1	signal sequence receptor, alpha (translocon-associated protein alpha)	200889_s_at	1	8.614	0.46	7	9.679	0.96	40	u	0.007343
			200891_s_at	1	11.51	0.17	7	11.98	0.54	40	u	0.000257
			200890_s_at	1	8.477	0.5	7	9.356	0.93	40	u	0.020987
			225435_at	0	9.091	0.25	7	8.406	0.98	40	d	0.000754
			201894_s_at	0	10	0.25	7	10.25	0.67	40	u	0.351159
C9orf97	1	chromosome 9 open reading frame 97	213161_at	6	8.326	0.23	7	7.785	0.45	40	d	0.003798
			1555430_at	0	2.298	0.2	7	2.337	0.18	40	u	0.620396
OSBPL9	1	oxysterol binding protein-like 9	218047_at	1	10.75	0.26	7	9.65	0.64	40	d	0.000065
IDH3G	1	isocitrate dehydrogenase 3 (NAD+) gamma	202471_s_at	1	9.525	0.16	7	9.104	0.7	40	d	0.002251
			214333_x_at	1	8.165	0.12	7	7.825	0.53	40	d	0.001384
MAPRE2	1	microtubule-associated protein, RP/EB family, member 2	202501_at	1	8.265	0.44	7	7.853	1.14	40	d	0.360068
			211556_at	0	2.655	0.04	7	2.759	0.11	40	u	0.000108
			213489_at	0	6.192	0.21	7	5.361	0.87	40	d	0.000012
IL1RN	1	interleukin 1 receptor antagonist	212657_s_at	1	4.877	0.87	7	6.033	1.54	40	u	0.064604
			212659_s_at	1	4.104	0.4	7	4.563	0.91	40	u	0.203496
			216245_at	0	2.063	0.21	7	2.111	0.2	40	u	0.57307
			216243_s_at	0	3.026	0.22	7	3.559	1.09	40	u	0.009364
			216244_at	0	2.885	0.21	7	2.936	0.19	40	u	0.531583
ADIPOR1	1	adiponectin receptor 1	217748_at	1	9.602	0.25	7	9.958	0.53	40	u	0.091412
SNF8	1	SNF8, ESCRT-II complex subunit, homolog (S. cerevisiae)	242790_at	0	5.702	0.32	7	5.461	0.7	40	d	0.387778
			218391_at	0	8.7	0.13	7	8.9	0.69	40	u	0.107907
HAT1	1	histone acetyltransferase 1	203138_at	1	10.08	0.24	7	10.25	0.54	40	u	0.443652
C14orf118	1	chromosome 14 open reading frame 118	219720_s_at	1	4.667	0.26	7	4.753	0.6	40	u	0.71952
			229520_s_at	0	6.443	0.42	7	6.296	0.93	40	d	0.689577

C14orf118	1	chromosome 14 open reading frame 118	231970_at	0	3.834	0.42	7	4.177	0.69	40	u	0.217431
			229514_at	0	5.537	0.63	7	4.601	0.81	40	d	0.006578
OAZ3	1	ornithine decarboxylase antizyme 3	222075_s_at	1	5.005	0.68	7	5.818	1.32	40	u	0.126768
			213863_s_at	0	2.416	0.04	7	2.573	0.37	40	u	0.013448
CA3	1	carbonic anhydrase III, muscle specific	204865_at	1	4.059	0.73	7	3.855	0.7	40	d	0.492239
ZNF143	1	zinc finger protein 143	206428_s_at	3	1.604	0.02	7	1.634	0.04	40	u	0.059314
			221873_at	1	7.047	0.16	7	7.045	0.49	40	d	0.989252
HOXD3	1	homeobox D3	206601_s_at	1	2.847	0.51	7	2.922	0.7	40	u	0.790632
			206602_s_at	0	1.962	0.04	7	2	0.05	40	u	0.095381
BFSP1	1	beaded filament structural protein 1, filensin	206746_at	1	3.721	0.46	7	3.899	0.8	40	u	0.581618
LRRC20	1	leucine rich repeat containing 20	218550_s_at	1	4.89	0.37	7	5.299	0.73	40	u	0.160221
RNPS1	1	RNA binding protein S1, serine-rich domain	200060_s_at	1	10.49	0.27	7	10.64	0.51	40	u	0.470812
			207939_x_at	1	8.612	0.19	7	8.597	0.49	40	d	0.937159
ISOC1	1	isochorismatase domain containing 1	218170_at	1	8.92	0.48	7	9.321	0.99	40	u	0.311193
EXDL2	1	exonuclease 3'-5' domain-like 2	218363_at	1	7.345	0.36	7	7.281	0.54	40	d	0.767324
			1555808_a_at	0	5.168	0.29	7	5.157	0.74	40	d	0.968956
APPBP1	1	amyloid beta precursor protein binding protein 1	202268_s_at	1	9.796	0.18	7	9.789	0.66	40	d	0.95512
ICA1	1	islet cell autoantigen 1, 69kDa	207949_s_at	1	7.982	0.28	7	7.695	0.73	40	d	0.318299
			204002_s_at	1	4.609	0.59	7	4.118	0.85	40	d	0.158812
			210547_x_at	1	7.877	0.46	7	7.458	0.99	40	d	0.288091
			214191_at	0	4.171	0.41	7	4.026	0.65	40	d	0.580454
			211740_at	0	3.259	0.27	7	3.549	0.49	40	u	0.141579
LOXL4	1	lysyl oxidase-like 4	227145_at	1	6.847	0.55	7	5.808	1.31	40	d	0.048502
			1570219_at	0	2.095	0.08	7	2.319	0.37	40	u	0.00205
FIZ1	1	FLT3-interacting zinc finger 1	226967_at	1	6.721	0.27	7	6.766	0.56	40	u	0.837862
			228663_x_at	0	3.36	0.62	7	3.523	0.82	40	u	0.624406
YARS	1	tyrosyl-tRNA synthetase	212048_s_at	1	8.138	0.29	7	8.677	0.82	40	u	0.005367
			238760_at	0	6.514	0.23	7	6.146	0.5	40	d	0.065815
BAG5	1	BCL2-associated athanogene 5	202984_s_at	1	6.578	0.69	7	7.089	0.75	40	u	0.106002
			230427_s_at	1	5.16	0.63	7	4.823	0.8	40	d	0.304177
			202985_s_at	1	9.15	0.25	7	8.774	0.58	40	d	0.103004
C6orf49	1	chromosome 6 open reading frame 49	218233_s_at	1	11.18	0.23	7	11.56	0.62	40	u	0.009356
			223516_s_at	1	10.09	0.23	7	10.44	0.71	40	u	0.02283
ZNF615	1	zinc finger protein 615	241827_at	1	7.287	0.37	7	6.379	0.86	40	d	0.009712
KCNN2	1	potassium intermediate/small conductance calcium-activated channel, subfamily N, member 2	220116_at	1	3.124	0.36	7	3.689	0.9	40	u	0.116758
CENPP	1	centromere protein P	no probeset available									
BRD2	1	bromodomain containing 2	208685_x_at	1	10.17	0.21	7	10.17	0.53	40	d	0.992984
			208686_s_at	1	9.372	0.19	7	8.75	0.71	40	d	0.000087
			214911_s_at	0	10.17	0.3	7	10.29	0.56	40	u	0.579828
C16orf80	1	chromosome 16 open reading frame 80	217957_at	1	9.049	0.18	7	8.889	0.66	40	d	0.225528
SPSB1	1	splA/ryanodine receptor domain and SOCS box containing 1	219677_at	1	5.811	0.84	7	6.736	0.68	40	u	0.002966
			226075_at	1	6.823	0.75	7	7.37	0.72	40	u	0.078272
CNOT8	1	CCR4-NOT transcription complex, subunit 8	202162_s_at	1	8.477	0.29	7	8.4	0.96	40	d	0.697637
			202164_s_at	1	8.519	0.38	7	8.301	1.02	40	d	0.586093
			202163_s_at	1	8.048	0.23	7	8.199	0.64	40	u	0.283234
TEGT	1	testis enhanced gene transcript (BAX inhibitor 1)	200804_at	1	11.37	0.34	7	10.76	0.76	40	d	0.046164
			200803_s_at	0	10.9	0.5	7	10.87	0.78	40	d	0.928645
ATP10D	1	ATPase, Class V, type 10D	213238_at	1	7.676	0.42	7	6.306	1.24	40	d	0.000021
PRKCH	1	protein kinase C, eta	218764_at	1	8.198	0.63	7	6.558	0.95	40	d	0.000087
			206099_at	0	6.851	0.88	7	6.106	0.76	40	d	0.027705
WDR48	1	WD repeat domain 48	221735_at	1	8.605	0.18	7	7.934	0.67	40	d	0.00001
			222157_s_at	0	6.896	0.35	7	6.62	1.07	40	d	0.227941
			56919_at	0	7.222	0.27	7	6.92	0.63	40	d	0.227936
			65591_at	0	6.871	0.4	7	6.682	0.95	40	d	0.614003
AGPAT5	1	1-acylglycerol-3-phosphate O-acyltransferase 5 (lysophosphatidic acid acyltransferase,	218096_at	1	8.139	0.31	7	8.418	0.85	40	u	0.403592
			232007_at	0	4.35	0.46	7	4.431	0.92	40	u	0.825438
ABCB6	1	ATP-binding cassette, sub-family B (MDR/TAP), member 6	203192_at	1	6.506	0.6	7	6.104	0.96	40	d	0.300975
			203191_at	1	5.505	0.19	7	5.309	0.32	40	d	0.131746
MRPS25	1	mitochondrial ribosomal protein S25	224873_s_at	1	6.494	0.37	7	6.401	0.75	40	d	0.755144
			224869_s_at	1	7.661	0.41	7	7.823	0.53	40	u	0.455919

			224015_s_at	0	5.401	0.59	7	5.429	0.67	40	u	0.919969
PPIC	1	peptidylprolyl isomerase C (cyclophilin C)	204517_at	1	9.619	0.42	7	9.313	1.13	40	d	0.491788
			204518_s_at	0	8.257	0.45	7	7.682	1.2	40	d	0.22781
IDH1	1	isocitrate dehydrogenase 1 (NADP+), soluble	201193_at	1	11.3	0.54	7	10.82	0.74	40	d	0.116653
			1555037_a_at	1	10.31	0.63	7	9.959	0.95	40	d	0.35627
			242001_at	0	2.663	0.06	7	2.846	0.21	40	u	0.000072
			242956_at	0	4.716	0.8	7	4.861	0.59	40	u	0.583564
BOC	1	Boc homolog (mouse)	225990_at	1	9.255	0.43	7	5.987	1.77	40	d	0
			224184_s_at	0	8.97	0.52	7	6.367	1.29	40	d	0.000006
			230035_at	0	5.49	0.42	7	5.757	0.54	40	u	0.232345
SEMA4A	1	sema domain, immunoglobulin domain (Ig), transmembrane domain (TM) and short	219259_at	1	7.062	0.3	7	7.101	0.66	40	u	0.880943
			234072_at	0	3.588	0.9	7	3.149	0.51	40	d	0.285978
NUAK2	1	NUAK family, SNF1-like kinase, 2	no probeset available									
RAB9A	1	RAB9A, member RAS oncogene family	221808_at	1	9.121	0.34	7	8.895	0.98	40	d	0.29212
CLDN1	1	claudin 1	222549_at	1	6.643	1.33	7	6.372	2.35	40	d	0.772725
			218182_s_at	0	4.396	1.1	7	5.207	1.95	40	u	0.301241
NFKB1	1	nuclear factor of kappa light polypeptide gene enhancer in B-cells 1 (p105)	209239_at	1	8.019	0.26	7	7.672	0.77	40	d	0.042035
VRK3	1	vaccinia related kinase 3	218022_at	0	7.546	0.3	7	7.401	0.69	40	d	0.591199
			221999_at	0	5.66	0.56	7	5.845	0.65	40	u	0.49462
			1566700_at	0	2.03	0.02	7	2.084	0.04	40	u	0.001057
			1566701_at	0	2.298	0.05	7	2.442	0.17	40	u	0.000339
			239190_at	0	2.055	0.03	7	2.276	0.45	40	u	0.004382
			221998_s_at	0	8.153	0.33	7	8.274	0.66	40	u	0.646471
GFRA1	1	GDNF family receptor alpha 1	205696_s_at	1	5.311	1.89	7	4.89	2.24	40	d	0.64872
GDF1	1	growth differentiation factor 1	206397_x_at	0	2.341	0.16	7	2.348	0.28	40	u	0.952322
ZKSCAN3	1	zinc finger with KRAB and SCAN domains 3	211773_s_at	1	4.962	0.46	7	5.222	0.39	40	u	0.128196
			1562303_at	0	2.363	0.03	7	2.564	0.28	40	u	0.00008
			1562305_x_at	0	2.673	0.22	7	3.065	0.49	40	u	0.050007
TDRD7	1	tudor domain containing 7	213361_at	1	7.498	0.21	7	7.815	0.88	40	u	0.063427
SNX15	1	sorting nexin 15	205482_x_at	1	6.939	0.46	7	6.938	0.41	40	d	0.995364
BMX	1	BMX non-receptor tyrosine kinase	206464_at	1	2.698	0.58	7	2.275	0.22	40	d	0.130483
PTPRU	1	protein tyrosine phosphatase, receptor type, U	211320_s_at	1	6.83	0.33	7	6.881	0.97	40	u	0.805307
ADPGK	1	ADP-dependent glucokinase	224455_s_at	1	6.262	0.41	7	6.173	1.12	40	d	0.717902
			220980_s_at	1	8.756	0.21	7	8.782	0.48	40	u	0.889609
ZBTB3	1	zinc finger and BTB domain containing 3	1553858_at	1	5.378	0.48	7	5.126	0.65	40	d	0.341253
			220391_at	0	4.031	0.38	7	4.157	0.56	40	u	0.575455
NCAN	1	neurocan	205143_at	1	2.56	0.22	7	3.416	1.91	40	u	0.010269
TOP2B	1	topoisomerase (DNA) II beta 180kDa	211987_at	1	9.424	0.23	7	9.453	0.57	40	u	0.893489
RPS25	1	ribosomal protein S25	200091_s_at	0	13.48	0.11	7	12.95	0.47	40	d	0
SHOX2	1	short stature homeobox 2	208443_x_at	1	4.212	0.37	7	3.991	0.48	40	d	0.262624
			210134_x_at	1	2.568	0.33	7	2.562	0.27	40	d	0.954385
			210135_s_at	1	5.348	1.12	7	4.219	1.11	40	d	0.019061
TXN2	1	thioredoxin 2	209077_at	1	8.715	0.22	7	8.239	0.57	40	d	0.036898
			209078_s_at	0	8.426	0.21	7	8.193	0.62	40	d	0.08808
TSPAN4	1	tetraspanin 4	209263_x_at	1	8.121	0.35	7	7.148	0.57	40	d	0.000107
			209264_s_at	0	7.734	0.39	7	6.34	0.98	40	d	0.000718
TPST1	1	tyrosylprotein sulfotransferase 1	204140_at	1	6.773	0.54	7	6.213	0.83	40	d	0.100432
PFKFB4	1	6-phosphofructo-2-kinase/fructose-2,6-biphosphatase 4	228499_at	1	4.747	0.6	7	5.472	0.67	40	u	0.012281
			206246_at	0	3.554	0.52	7	3.419	0.37	40	d	0.416018
SREBF1	1	sterol regulatory element binding transcription factor 1	1558875_at	4	4.818	0.93	7	4.735	1.2	40	d	0.865983
			202308_at	1	7.971	0.67	7	7.083	1.36	40	d	0.104509
SCMH1	1	sex comb on midleg homolog 1 (Drosophila)	221216_s_at	1	8.128	0.32	7	7.461	0.69	40	d	0.018579
FUK	1	fucokinase	226072_at	1	6.971	0.28	7	6.613	0.75	40	d	0.225715
HTR2A	1	5-hydroxytryptamine (serotonin) receptor 2A	207135_at	1	2.344	0.23	7	2.219	0.43	40	d	0.474111
			211616_s_at	0	2.155	0.03	7	2.272	0.38	40	u	0.064645
ARMC5	1	armadillo repeat containing 5	227738_s_at	1	7.404	0.37	7	6.75	0.47	40	d	0.001336
AKR1A1	1	aldo-keto reductase family 1, member A1 (aldehyde reductase)	201900_s_at	1	10.48	0.79	7	9.965	0.81	40	d	0.134687
			241978_at	0	2.763	0.64	7	2.891	0.67	40	u	0.647856
TNP1	1	transition protein 1 (during histone to protamine replacement)	206568_at	1	3.009	0.07	7	3.087	0.16	40	u	0.231211
ERRFI1	1	ERBB receptor feedback inhibitor 1	224657_at	1	10.79	0.37	7	9.897	1.07	40	d	0.000626

ZC3H15	1	zinc finger CCCH-type containing 15	201595_s_at	1	9.334	0.21	7	9.83	0.56	40	u	0.029445
			201593_s_at	1	10.41	0.15	7	10.85	0.55	40	u	0.000255
INADL	1	InaD-like (Drosophila)	232041_at	1	2.231	0.14	7	2.356	0.28	40	u	0.263016
			223681_s_at	1	8.912	0.36	7	8.162	0.74	40	d	0.013413
			214705_at	0	6.297	0.52	7	5.653	0.94	40	d	0.091584
			214493_s_at	0	6.602	0.61	7	6.323	0.66	40	d	0.311297
			239173_at	0	4.668	0.89	7	4.774	1.05	40	u	0.806386
SEC61B	1	Sec61 beta subunit	203133_at	0	11.27	0.18	7	11.34	0.5	40	u	0.710882
			244700_at	0	2.505	0.33	7	2.558	0.42	40	u	0.759846
PRPF40B	1	PRP40 pre-mRNA processing factor 40 homolog B (S. cerevisiae)	226966_at	1	6.361	0.34	7	6.037	0.58	40	d	0.168706
GRINL1A	1	glutamate receptor, ionotropic, N-methyl D-aspartate-like 1A	212241_at	1	6.629	0.51	7	6.718	0.53	40	u	0.688786
			212243_at	1	7.748	0.38	7	7.446	0.57	40	d	0.195233
			212244_at	1	8.809	0.14	7	8.168	0.45	40	d	0
MMP19	1	matrix metallopeptidase 19	204575_s_at	1	5.119	0.54	7	4.751	0.58	40	d	0.131185
			204574_s_at	1	4.863	0.23	7	4.84	0.4	40	d	0.882445
TMTC1	1	transmembrane and tetratricopeptide repeat containing 1	226322_at	1	9.725	0.3	7	6.27	1.49	40	d	0
			226931_at	0	9.055	1.25	7	5.778	1.26	40	d	0
			224397_s_at	0	5.187	0.68	7	4.451	0.96	40	d	0.064128
			244506_at	0	2.503	0.27	7	2.444	0.38	40	d	0.704084
LDOC1	1	leucine zipper, down-regulated in cancer 1	204454_at	1	8.637	0.36	7	8.309	0.86	40	d	0.336156
REG3A	1	regenerating islet-derived 3 alpha	205815_at	0	2.286	0.28	7	2.207	0.04	40	d	0.519003
			234280_at	0	3.162	0.22	7	3.076	0.3	40	d	0.471473
TRAFD1	1	TRAF-type zinc finger domain containing 1	202837_at	1	6.839	0.28	7	6.698	0.56	40	d	0.529068
			242497_at	1	3.538	0.16	7	3.553	0.24	40	u	0.877303
			35254_at	1	8.057	0.3	7	7.991	0.67	40	d	0.805325
			243196_s_at	1	6.662	0.26	7	6.679	0.42	40	u	0.921535
ZFY	1	zinc finger protein, Y-linked	207246_at	0	2.489	0.08	7	2.597	0.14	40	u	0.065275
			230760_at	0	3.098	0.05	7	3.328	0.22	40	u	0.000001
FSD1L	1	fibronectin type III and SPRY domain containing 1-like	242807_at	1	3.919	0.55	7	5.068	0.87	40	u	0.00181
			242586_at	0	3.355	0.29	7	4.524	1.08	40	u	0.000003
			230904_at	0	3.402	0.56	7	4.139	0.72	40	u	0.014948
			223985_at	0	2.964	0.13	7	3.391	0.62	40	u	0.000456
RPRM	1	reprimo, TP53 dependent G2 arrest mediator candidate	219370_at	1	2.37	0.13	7	2.825	0.74	40	u	0.001103
HAP1	1	huntingtin-associated protein 1 (neuroan 1)	211222_s_at	1	4.238	0.22	7	4.406	0.38	40	u	0.27339
			238275_at	0	3.301	0.31	7	3.29	0.33	40	d	0.938474
			211604_x_at	0	3.179	0.26	7	3.108	0.33	40	d	0.600879
PPARGC1B	1	peroxisome proliferator-activated receptor gamma, coactivator 1 beta	1563943_at	1	2.202	0.04	7	2.286	0.12	40	u	0.079983
			1555282_a_at	0	2.495	0.32	7	2.516	0.44	40	u	0.907176
CCDC78	1	coiled-coil domain containing 78	236745_at	1	4.569	0.5	7	5.137	1.22	40	u	0.239266
AGTR2	1	angiotensin II receptor, type 2	222321_at	1	3.303	0.08	7	3.276	0.21	40	d	0.745034
			207294_at	1	3.22	0.12	7	3.356	0.39	40	u	0.096422
			207293_s_at	0	1.955	0.02	7	2.049	0.3	40	u	0.057174
SRRM2	1	serine/arginine repetitive matrix 2	1554671_a_at	1	2.891	0.32	7	3.184	0.52	40	u	0.165313
			207435_s_at	1	10.59	0.52	7	9.748	1.21	40	d	0.082488
			208610_s_at	1	10.11	0.52	7	9.382	1.34	40	d	0.169895
			216629_at	0	5.424	0.38	7	5.096	1.09	40	d	0.171467
AMIGO2	1	adhesion molecule with Ig-like domain 2	222108_at	1	11.36	0.53	7	7.996	1.65	40	d	0
LRTM2	1	leucine-rich repeats and transmembrane domains 2	1558530_at	1	3.909	0.2	7	4.016	0.54	40	u	0.613548
LNX1	1	ligand of numb-protein X 1	223612_s_at	1	3.555	0.5	7	3.466	0.5	40	d	0.674275
			223611_s_at	1	6.992	0.68	7	7.1	1.19	40	u	0.821738
WDR23	1	WD repeat domain 23	216389_s_at	1	6.307	0.37	7	6.359	0.92	40	u	0.884798
			201886_at	1	7.65	0.29	7	7.569	0.6	40	d	0.735668
PPP1R7	1	protein phosphatase 1, regulatory (inhibitor) subunit 7	201213_at	1	5.335	0.28	7	5.234	0.42	40	d	0.549146
			201214_s_at	1	8.25	0.28	7	7.919	0.61	40	d	0.174016
			213465_s_at	0	8.931	0.23	7	8.793	0.73	40	d	0.361258
CYP1B1	1	cytochrome P450, family 1, subfamily B, polypeptide 1	202435_s_at	1	8.354	0.49	7	8.443	1.62	40	u	0.787545
			202437_s_at	1	8.585	0.51	7	8.336	1.79	40	d	0.484756
			202434_s_at	1	3.657	0.29	7	4.163	1.53	40	u	0.070208
			202436_s_at	1	9.097	0.53	7	8.746	1.68	40	d	0.318319
			212412_at	1	10.78	0.33	7	10.13	0.87	40	d	0.066147
			241208_at	0	2.348	0.47	7	2.298	0.27	40	d	0.807474

PDLIM5	1	PDZ and LIM domain 5	216804_s_at	0	8.854	0.68	7	8.891	1.29	40	u	0.942649
			213684_s_at	0	7.293	0.68	7	5.92	1.14	40	d	0.004017
			216803_at	0	4.071	0.53	7	3.942	0.51	40	d	0.550849
			221994_at	0	3.966	0.4	7	4.174	0.98	40	u	0.59032
			211681_s_at	0	6.497	0.78	7	6.197	1.83	40	d	0.677626
			203242_s_at	0	8.396	0.63	7	8.347	1.39	40	d	0.92939
			203243_s_at	0	10.23	0.28	7	9.512	1.05	40	d	0.001113
RAD51L1	1	RAD51-like 1 (S. cerevisiae)	1554496_at	1	4.578	0.3	7	4.737	0.91	40	u	0.408646
			210255_at	0	4.66	1.01	7	3.29	0.66	40	d	0.000043
			216880_at	0	2.828	0.48	7	2.642	0.21	40	d	0.386334
LETMD1	1	LETM1 domain containing 1	207170_s_at	1	9.294	0.26	7	7.932	0.71	40	d	0
TMEM54	1	transmembrane protein 54	225536_at	1	7.35	0.37	7	8.062	1.11	40	u	0.005128
			202090_s_at	0	11.21	0.21	7	11.24	0.62	40	u	0.823969
PHF23	1	PHD finger protein 23	223081_at	1	8.221	0.19	7	7.9	0.61	40	d	0.014633
			1555789_s_at	0	7.537	0.34	7	7.285	0.81	40	d	0.433828
MTFR1	1	mitochondrial fission regulator 1	203207_s_at	1	6.667	0.69	7	8.147	1.38	40	u	0.009438
			203208_s_at	1	7.981	0.41	7	9.407	0.86	40	u	0.00012
			232314_at	0	2.256	0.02	7	2.363	0.14	40	u	0.00007
ANKS1A	1	ankyrin repeat and sterile alpha motif domain containing 1A	212747_at	1	8.048	0.32	7	7.377	0.65	40	d	0.011675
COBLL1	1	COBL-like 1	203642_s_at	1	7.638	0.18	7	6.945	0.88	40	d	0.000086
			203641_s_at	1	5.882	0.4	7	5.176	1.41	40	d	0.016537
			215393_s_at	0	2.9	0.2	7	3.061	0.33	40	u	0.232033
			211032_at	0	2.739	0.07	7	2.906	0.23	40	u	0.001136
			229598_at	0	2.925	0.19	7	3.035	0.48	40	u	0.557722
E2F8	1	E2F transcription factor 8	219990_at	1	3.917	0.7	7	5.56	0.91	40	u	0.000056
CCDC25	1	coiled-coil domain containing 25	222525_s_at	1	8.403	0.36	7	7.723	0.78	40	d	0.030913
			218125_s_at	1	7.55	0.32	7	6.832	0.66	40	d	0.007977
RNF135	1	ring finger protein 135	223592_s_at	1	9.392	0.23	7	8.773	0.75	40	d	0.000381
			223591_at	1	8.389	0.33	7	7.894	0.71	40	d	0.084336
ARHGAP11A	1	Rho GTPase activating protein 11A	204492_at	1	2.611	0.16	7	3.045	0.63	40	u	0.000891
EHF	1	ets homologous factor	222932_at	1	6.399	0.97	7	5.757	1.69	40	d	0.343221
			219850_s_at	1	8.044	0.69	7	7.566	2.39	40	d	0.323762
			225645_at	1	11.9	0.5	7	10.06	2.2	40	d	0.000052
			224189_x_at	1	8.241	0.77	7	7.705	2.22	40	d	0.268156
			241397_at	0	6.649	1.04	7	6.407	1.35	40	d	0.661175
			232361_s_at	0	8.771	0.69	7	7.127	2	40	d	0.00071
			232360_at	0	8.5	0.76	7	6.863	1.42	40	d	0.005434
ZNF364	1	zinc finger protein 364	212742_at	1	8.142	0.14	7	8.382	0.68	40	u	0.056889
PTEN	1	phosphatase and tensin homolog (mutated in multiple advanced cancers 1)	204054_at	4	5.914	0.4	7	5.546	0.59	40	d	0.129614
			211711_s_at	1	7.411	0.25	7	7.547	0.59	40	u	0.5611
			222176_at	0	2.489	0.11	7	2.632	0.27	40	u	0.180515
			233314_at	0	4.71	0.46	7	4.173	0.84	40	d	0.115477
			233254_x_at	0	6.996	0.27	7	6.401	0.65	40	d	0.02384
			242622_x_at	0	3.93	0.61	7	4.058	0.48	40	u	0.546283
			217492_s_at	0	8.28	0.36	7	8.729	0.74	40	u	0.129813
			204053_x_at	0	8.551	0.29	7	8.678	0.68	40	u	0.637553
			225363_at	0	11.13	0.22	7	10.03	0.55	40	d	0.000007
			227469_at	0	2.704	0.1	7	2.788	0.18	40	u	0.239527
TBC1D14	1	TBC1 domain family, member 14	224622_at	1	9.123	0.25	7	8.439	0.6	40	d	0.005436
ATP1A3	1	ATPase, Na+/K+ transporting, alpha 3 polypeptide	214432_at	1	2.894	0.19	7	2.929	0.31	40	u	0.776535
ATXN7L2	1	ataxin 7-like 2	230038_at	1	2.113	0.06	7	2.122	0.05	40	u	0.669371
PACSIN2	1	protein kinase C and casein kinase substrate in neurons 2	201651_s_at	1	10.46	0.33	7	9.746	0.71	40	d	0.014323
			233705_at	0	2.49	0.15	7	2.515	0.2	40	u	0.751464
			1554691_a_at	0	7.562	0.59	7	7.299	0.82	40	d	0.429882
C17orf63	1	chromosome 17 open reading frame 63	218464_s_at	1	8.14	0.23	7	8.29	0.74	40	u	0.332063
			222641_s_at	1	7.631	0.38	7	7.56	0.7	40	d	0.79855
			229418_at	0	6.277	0.44	7	6.06	0.72	40	d	0.452332
HLTF	1	helicase-like transcription factor	202983_at	1	9.108	0.27	7	9.934	0.71	40	u	0.004568
PTPN18	1	protein tyrosine phosphatase, non-receptor type 18 (brain-derived)	203555_at	1	8.188	0.42	7	7.56	0.61	40	d	0.013217
			1569552_at	0	3.455	0.31	7	3.588	0.37	40	u	0.383685
			213521_at	0	8.51	0.32	7	8.21	0.52	40	d	0.156455
TOPBP1	1	topoisomerase (DNA) II binding protein 1	202633_at	1	8.186	0.2	7	8.705	0.59	40	u	0.000326

KCNAB3	1	potassium voltage-gated channel, shaker-related subfamily, beta member 3	221413_at	0	2.182	0.25	7	2.191	0.14	40	u	0.937337
GPR180	1	G protein-coupled receptor 180	231871_at	1	4.72	0.48	7	5.943	1.07	40	u	0.005529
			232912_at	0	3.269	0.21	7	4.156	0.86	40	u	0.000004
HD	1	huntingtin (Huntington disease)	202390_s_at	1	6.494	0.4	7	6.15	0.72	40	d	0.236299
			202389_s_at	0	6.49	0.31	7	6.282	0.76	40	d	0.487337
ARL1	1	ADP-ribosylation factor-like 1	201657_at	1	7.799	0.31	7	8.364	0.79	40	u	0.073734
			201658_at	1	9.412	0.19	7	9.031	0.69	40	d	0.008384
			201659_s_at	0	8.638	0.28	7	9.16	0.71	40	u	0.067864
KLC4	1	kinesin light chain 4	225596_at	1	6.457	0.28	7	6.398	0.58	40	d	0.798019
			1553675_at	0	2.165	0.33	7	2.1	0.17	40	d	0.653794
EIF3B	1	eukaryotic translation initiation factor 3, subunit B	203462_x_at	1	9.717	0.31	7	9.979	0.62	40	u	0.292146
			208688_x_at	1	9.915	0.25	7	9.993	0.66	40	u	0.763064
			211501_s_at	1	8.804	0.37	7	8.998	0.65	40	u	0.452271
			236274_at	0	7.353	0.4	7	6.756	0.92	40	d	0.104582
			242550_at	0	7.007	0.6	7	6.357	0.84	40	d	0.060119
			237457_at	0	2.773	0.2	7	2.742	0.21	40	d	0.728052
ALPK3	1	alpha-kinase 3	214846_s_at	1	3.333	0.65	7	2.982	0.51	40	d	0.122825
			228342_s_at	0	2.488	0.38	7	2.31	0.27	40	d	0.145481
KIAA0141	1	KIAA0141	201978_s_at	1	7.256	0.28	7	6.258	0.63	40	d	0.000205
			201977_s_at	1	6.851	0.3	7	6.187	0.91	40	d	0.001579
			227056_at	0	6.533	0.62	7	5.863	0.84	40	d	0.055135
VSTM2L	1	V-set and transmembrane domain containing 2 like	226973_at	1	4.144	0.9	7	4.048	1.19	40	d	0.842658
CDH13	1	cadherin 13, H-cadherin (heart)	204726_at	1	3.053	0.48	7	3.218	0.77	40	u	0.594786
MED29	1	mediator complex subunit 29	225708_at	1	7.846	0.3	7	7.797	0.65	40	d	0.845908
MRPS14	1	mitochondrial ribosomal protein S14	203800_s_at	0	7.941	0.25	7	8.627	0.73	40	u	0.00016
			203801_at	0	7.311	0.4	7	6.318	0.97	40	d	0.012472
FBXO8	1	F-box protein 8	1554806_a_at	1	7.972	0.43	7	7.273	0.95	40	d	0.067752
			223240_at	1	6.82	0.22	7	6.663	0.63	40	d	0.253636
SFXN2	1	sideroflexin 2	227560_at	1	7.527	0.62	7	7.492	0.82	40	d	0.917707
RAB3GAP1	1	RAB3 GTPase activating protein subunit 1 (catalytic)	213531_s_at	1	9.747	0.17	7	9.556	0.48	40	d	0.076996
			212932_at	1	7.823	0.18	7	7.26	0.76	40	d	0.000289
			213530_at	1	7.205	0.17	7	6.901	0.55	40	d	0.011431
			239527_at	0	2.784	0.07	7	2.959	0.2	40	u	0.000433
SLC25A26	1	solute carrier family 25, member 26	225862_at	10	8.001	0.5	7	7.175	0.87	40	d	0.020852
GPX7	1	glutathione peroxidase 7	213170_at	1	6.179	0.33	7	6.252	1.46	40	u	0.786011
STAM	1	signal transducing adaptor molecule (SH3 domain and ITAM motif) 1	203544_s_at	1	7.169	0.28	7	7.794	0.68	40	u	0.02292
ACTL6A	1	actin-like 6A	202666_s_at	1	8.582	0.28	7	9.945	0.77	40	u	0.00004
LYRM1	1	LYR motif containing 1	203897_at	1	8.981	0.19	7	8.732	0.77	40	d	0.095866
MANBAL	1	mannosidase, beta A, lysosomal-like	224689_at	1	9.125	0.22	7	8.552	0.69	40	d	0.000383
MPPE1	1	metallophosphoesterase 1	213924_at	1	6.426	0.94	7	4.591	1.21	40	d	0.000524
			206764_x_at	1	6.93	0.31	7	6.7	0.51	40	d	0.265189
			214071_at	1	4.71	0.88	7	3.726	1.25	40	d	0.056405
			209858_x_at	1	7.974	0.36	7	7.586	0.64	40	d	0.13543
			213727_x_at	1	8.03	0.27	7	7.634	0.7	40	d	0.156232
PIGZ	1	phosphatidylinositol glycan anchor biosynthesis, class Z	220041_at	1	5.334	0.52	7	5.15	0.84	40	d	0.584374
PHACTR3	1	phosphatase and actin regulator 3	227949_at	1	2.43	0.05	7	2.585	0.26	40	u	0.001773
KIAA0562	1	KIAA0562	204075_s_at	1	5.427	0.42	7	5.249	0.61	40	d	0.473468
			204074_s_at	1	7.39	0.34	7	7.697	0.55	40	u	0.167518
			243696_at	0	3.488	0.25	7	3.838	0.55	40	u	0.113998
BIN3	1	bridging integrator 3	222199_s_at	1	8.066	0.23	7	6.804	0.5	40	d	0
			1557582_at	0	5.115	0.63	7	4.568	0.63	40	d	0.044776
HPS6	1	Hermansky-Pudlak syndrome 6	219052_at	1	6.829	0.27	7	6.692	0.52	40	d	0.51022
			230371_at	0	4.223	0.47	7	3.957	0.36	40	d	0.099939
ZNF197	1	zinc finger protein 197	233070_at	0	3.568	0.51	7	3.441	0.55	40	d	0.579214
			205855_at	0	4.889	0.23	7	5.149	0.36	40	u	0.073651
C7orf26	1	chromosome 7 open reading frame 26	47083_at	1	7.617	0.14	7	7.315	0.55	40	d	0.006423
			219261_at	1	7.332	0.31	7	6.993	0.7	40	d	0.225287
			222002_at	0	2.695	0.14	7	2.808	0.15	40	u	0.070849
CARD11	1	caspase recruitment domain family, member 11	223514_at	1	5.44	0.47	7	5.426	0.69	40	d	0.961009

KIAA1958	1	KIAA1958	230166_at	1	3.776	0.65	7	4.282	1.09	40	u	0.247506
ZC3H7A	1	zinc finger CCCH-type containing 7A	218348_s_at	1	8.281	0.22	7	7.977	0.5	40	d	0.127912
			226897_s_at	1	7.472	0.3	7	7.762	0.63	40	u	0.249481
GEMIN8	1	gem (nuclear organelle) associated protein 8	222854_s_at	1	6.584	0.28	7	5.976	0.79	40	d	0.001537
			219252_s_at	1	5.163	0.47	7	4.84	0.58	40	d	0.182996
			230758_at	0	6.466	0.32	7	5.922	0.83	40	d	0.101473
FAM44A	1	family with sequence similarity 44, member A	225821_s_at	1	8.683	0.19	7	7.588	0.83	40	d	0
			242594_at	0	2.762	0.12	7	3.002	0.45	40	u	0.010044
			235009_at	0	6.338	0.5	7	6.708	0.9	40	u	0.307257
KCNA2	1	potassium voltage-gated channel, shaker-related subfamily, member 2	208564_at	0	2.776	0.26	7	2.701	0.26	40	d	0.491005
			239118_at	0	3.022	0.03	7	3.163	0.37	40	u	0.022954
PCYT1A	1	phosphate cytidylyltransferase 1, choline, alpha	204209_at	0	5.904	0.68	7	6.552	0.84	40	u	0.066121
			225069_at	0	8.138	0.13	7	8.145	0.52	40	u	0.946384
			204210_s_at	0	3.652	0.5	7	4.072	0.47	40	u	0.040452
SEC16B	1	SEC16 homolog B (S. cerevisiae)	228150_at	1	5.356	0.84	7	4.946	0.84	40	d	0.249855
			1552880_at	1	3.285	0.26	7	3.231	0.31	40	d	0.677375
			1564423_a_at	0	3.705	0.6	7	3.335	0.46	40	d	0.074932
			239618_at	0	2.98	0.11	7	3.162	0.3	40	u	0.009743
			1555289_at	0	2.164	0.03	7	2.32	0.25	40	u	0.000453
			241607_at	0	7.08	0.38	7	5.677	1.15	40	d	0.000004
			239279_at	0	3.295	0.13	7	3.516	0.44	40	u	0.018674
LGALS1	1	lectin, galactoside-binding, soluble, 1 (galectin 1)	201105_at	1	12.46	0.34	7	12.18	0.9	40	d	0.428605
			216405_at	0	4.014	0.25	7	4.032	0.34	40	u	0.89425
CYFIP1	1	cytoplasmic FMR1 interacting protein 1	208923_at	1	10.89	0.13	7	10.35	0.55	40	d	0.000004
			212133_at	1	4.556	0.23	7	4.932	0.72	40	u	0.01695
ALK	1	anaplastic lymphoma kinase (Ki-1)	208211_s_at	1	2.83	0.1	7	2.829	0.26	40	d	0.99489
			208212_s_at	1	5.558	0.38	7	5.663	0.45	40	u	0.571534
VAMP1	1	vesicle-associated membrane protein 1 (synaptobrevin 1)	213326_at	1	7.354	0.41	7	6.7	1.11	40	d	0.137136
			207100_s_at	1	6.594	0.42	7	6.065	0.71	40	d	0.06753
			207101_at	1	4.936	0.42	7	4.704	0.42	40	d	0.193186
EVC2	1	Ellis van Creveld syndrome 2 (limbin)	229974_at	1	4.691	0.55	7	3.735	0.53	40	d	0.000105
			1555256_at	0	2.202	0.08	7	2.234	0.08	40	u	0.330887
FLT3LG	1	fms-related tyrosine kinase 3 ligand	210607_at	1	4.918	0.51	7	4.614	0.38	40	d	0.07698
			206980_s_at	0	3.02	0.59	7	2.721	0.44	40	d	0.129387
YWHAE	1	tyrosine 3-monooxygenase/tryptophan 5-monooxygenase activation protein, epsilon polypeptide	210996_s_at	1	11.56	0.28	7	11.91	0.64	40	u	0.175089
			213655_at	1	10.72	0.79	7	10.88	0.94	40	u	0.685549
			210317_s_at	0	9.466	0.34	7	9.566	1.51	40	u	0.719842
AFG3L2	1	AFG3 ATPase family gene 3-like 2 (yeast)	202486_at	1	7.307	0.29	7	7.777	0.86	40	u	0.015722
			232919_at	0	5.691	0.37	7	5.091	0.45	40	d	0.002311
			1557820_at	0	9.613	0.29	7	10.04	0.79	40	u	0.168315
ADAT2	1	adenosine deaminase, tRNA-specific 2, TAD2 homolog (S. cerevisiae)	227213_at	1	5.604	0.35	7	5.265	0.78	40	d	0.27544
			236817_at	0	4.002	0.2	7	4.051	0.52	40	u	0.809676
EIF4EBP3	1	eukaryotic translation initiation factor 4E binding protein 3	214919_s_at	3	8.646	0.38	7	7.031	0.96	40	d	0.000086
IQCH	1	IQ motif containing H	220361_at	1	2.254	0.08	7	2.441	0.39	40	u	0.011443
			224165_s_at	1	3.244	0.55	7	3.06	0.5	40	d	0.391637
			1569610_at	0	2.603	0.09	7	2.704	0.13	40	u	0.068157
			1569611_a_at	0	3.047	0.1	7	3.266	0.33	40	u	0.002936
			239812_s_at	0	2.968	0.22	7	2.939	0.16	40	d	0.685017
			239813_at	0	2.465	0.04	7	2.514	0.15	40	u	0.092647
TFCP2L1	1	transcription factor CP2-like 1	219735_s_at	1	3.796	0.97	7	4.124	1.6	40	u	0.609722
			227642_at	0	7.404	0.91	7	6.095	1.88	40	d	0.083762
			229341_at	0	5.407	1.56	7	4.955	1.41	40	d	0.454193
THBS3	1	thrombospondin 3	209561_at	1	6.938	0.26	7	6.605	0.93	40	d	0.077956
SMARCB1	1	SWI/SNF related, matrix associated, actin dependent regulator of chromatin, subfamily	212167_s_at	1	7.743	0.36	7	7.76	0.6	40	u	0.943977
			228898_s_at	0	3.8	0.24	7	3.813	0.39	40	u	0.934846
GMCL1	1	germ cell-less homolog 1 (Drosophila)	218458_at	1	6.51	0.53	7	7.246	0.79	40	u	0.025513
			234488_s_at	0	4.991	0.4	7	6.308	0.7	40	u	0.000023
TMEM16H	1	transmembrane protein 16H	1563549_a_at	1	5.827	0.3	7	5.509	1.03	40	d	0.130917
DYNLT1	1	dynein, light chain, Tctex-type 1	201999_s_at	1	11.49	0.29	7	11.65	0.62	40	u	0.516016
MYO5B	1	myosin VB	225301_s_at	1	8.061	0.63	7	8.283	1.14	40	u	0.624547
			225299_at	1	7.029	0.58	7	6.723	1.08	40	d	0.475658
			1570141_at	0	2.743	0.1	7	2.818	0.14	40	u	0.192532

GNG5	1	guanine nucleotide binding protein (G protein), gamma 5	207157_s_at	0	11.35	0.15	7	11.48	0.62	40	u	0.282936
FEM1B	1	fem-1 homolog b (C. elegans)	212367_at	1	8.585	0.18	7	8.275	0.7	40	d	0.026874
			212373_at	1	7.638	0.39	7	7.333	0.82	40	d	0.349849
			212374_at	1	5.092	0.65	7	4.97	1	40	d	0.762079
LBP	1	lipopolysaccharide binding protein	214461_at	1	2.721	1.56	7	2.745	1.88	40	u	0.974812
			211652_s_at	0	3.418	1.21	7	3.292	1.21	40	d	0.803795
C3orf23	1	chromosome 3 open reading frame 23	226688_at	1	5.653	0.36	7	5.378	0.81	40	d	0.391187
			1555906_s_at	0	6.442	0.4	7	7.496	0.96	40	u	0.007647
			241666_at	0	3.965	0.36	7	4.306	0.85	40	u	0.31442
			1555905_a_at	0	3.625	0.38	7	4.572	1.11	40	u	0.000505
IDH2	1	isocitrate dehydrogenase 2 (NADP+), mitochondrial	210046_s_at	1	9.477	0.5	7	10.19	0.99	40	u	0.075869
			210045_at	0	5.729	0.45	7	5.695	0.96	40	d	0.927952
PCGF6	1	polycomb group ring finger 6	224326_s_at	1	6.839	0.27	7	7.026	0.62	40	u	0.448251
TMCO2	1	transmembrane and coiled-coil domains 2	231264_at	1	2.187	0.08	7	2.256	0.1	40	u	0.090231
SLC4A3	1	solute carrier family 4, anion exchanger, member 3	205918_at	1	4.644	0.54	7	4.503	1.33	40	d	0.787847
COPZ1	1	coatomer protein complex, subunit zeta 1	222386_s_at	1	9.44	0.25	7	9.609	0.79	40	u	0.302053
			217726_at	1	10.05	0.13	7	9.551	0.71	40	d	0.00024
STIM1	1	stromal interaction molecule 1	202764_at	1	6.913	0.31	7	6.101	0.84	40	d	0.000202
TBX2	1	T-box 2	213417_at	1	2.849	0.32	7	2.636	0.41	40	d	0.206595
			40560_at	1	4.869	0.36	7	4.433	0.53	40	d	0.045158
			205993_s_at	0	5.506	0.27	7	5.05	0.5	40	d	0.024848
SRPK3	1	SFRS protein kinase 3	206216_at	1	4.155	0.37	7	4.889	1.43	40	u	0.011448
CXorf9	1	chromosome X open reading frame 9	204923_at	1	5.992	0.6	7	6.481	1.21	40	u	0.312284
PPAPDC3	1	phosphatidic acid phosphatase type 2 domain containing 3	224506_s_at	1	3.813	0.53	7	2.992	0.64	40	d	0.002786
COIL	1	coilin	203654_s_at	1	8.851	0.26	7	9.054	0.71	40	u	0.470894
			203653_s_at	1	6.365	0.28	7	7.086	0.79	40	u	0.00027
PIGR	1	polymeric immunoglobulin receptor	226147_s_at	1	8.933	1.53	7	3.554	1.41	40	d	0
			229659_s_at	1	8.084	1.25	7	4.226	0.91	40	d	0
			204213_at	0	5.199	2.12	7	3.549	0.37	40	d	0.105913
DHX30	1	DEAH (Asp-Glu-Ala-His) box polypeptide 30	204355_at	1	7.746	0.19	7	7.463	0.73	40	d	0.0517
			212674_s_at	1	8.083	0.22	7	7.842	0.66	40	d	0.093201
			239772_x_at	0	4.842	0.35	7	5.005	0.55	40	u	0.468006
			230871_at	0	5.584	0.36	7	6.397	0.83	40	u	0.016857
			1556144_at	0	2.723	0.39	7	2.993	0.61	40	u	0.273892
			236844_at	0	2.827	0.55	7	2.516	0.18	40	d	0.220471
PTPRB	1	protein tyrosine phosphatase, receptor type, B	205846_at	0	4.045	0.45	7	3.117	0.63	40	d	0.000714
			230250_at	0	6.926	0.53	7	5.349	0.82	40	d	0.000017
			1560105_at	0	2.19	0.04	7	2.273	0.13	40	u	0.004461
AIM1L	1	absent in melanoma 1-like	220289_s_at	0	2.462	0.16	7	2.789	0.61	40	u	0.008656
			220290_at	0	4.898	0.44	7	5.017	0.5	40	u	0.566351
ZRSR2	1	zinc finger (CCCH type), RNA-binding motif and serine/arginine rich 2	213876_x_at	0	7.937	0.21	7	7.781	0.58	40	d	0.225602
			208174_x_at	0	7.965	0.27	7	7.765	0.6	40	d	0.402635
NOTUM	1	notum pectinacetylesterase homolog (Drosophila)	228649_at	1	6.475	0.25	7	6.516	0.35	40	u	0.772478
PPP2R1B	1	protein phosphatase 2 (formerly 2A), regulatory subunit A, beta isoform	202886_s_at	1	5.791	0.62	7	6.103	0.83	40	u	0.35601
			202884_s_at	0	5.615	0.47	7	5.591	0.75	40	d	0.936006
			222351_at	0	7.301	0.56	7	6.621	0.42	40	d	0.000704
			202883_s_at	0	7.562	0.26	7	7.29	0.79	40	d	0.111615
			202885_s_at	0	2.369	0.05	7	2.454	0.14	40	u	0.007762
SLC1A5	1	solute carrier family 1 (neutral amino acid transporter), member 5	208916_at	0	9.093	0.34	7	8.812	0.77	40	d	0.356984
CACNG4	1	calcium channel, voltage-dependent, gamma subunit 4	221585_at	1	3.83	0.7	7	3.778	1.37	40	d	0.924614
			231737_at	1	2.765	0.39	7	2.967	0.82	40	u	0.534255
			62987_r_at	1	8.407	0.2	7	8.579	0.55	40	u	0.423944
MYBL2	1	v-myb myeloblastosis viral oncogene homolog (avian)-like 2	201710_at	1	4.766	0.48	7	7.076	0.84	40	u	0
GFI1B	1	growth factor independent 1B (potential regulator of CDKN1A, translocated in CML)	237403_at	1	2.591	0.45	7	2.727	0.41	40	u	0.437636
			208501_at	0	2.056	0.03	7	2.095	0.03	40	u	0.007085
ZNF407	1	zinc finger protein 407	227768_at	1	6.89	0.35	7	6.477	0.62	40	d	0.102904
			220835_s_at	0	3.141	0.13	7	3.216	0.28	40	u	0.498604
			220836_at	0	2.758	0.17	7	2.981	0.42	40	u	0.180286

			234844_at	0	2.757	0.17	7	2.922	0.24	40	u	0.095598
MAPT	1	microtubule-associated protein tau	225379_at	3	7.174	0.58	7	4.808	1.73	40	d	0.000001
			203930_s_at	1	6.026	0.61	7	3.955	1.49	40	d	0.000936
			203929_s_at	1	9.645	0.68	7	6.42	2.47	40	d	0
			203928_x_at	1	7.785	0.93	7	6.169	1.7	40	d	0.020969
			206401_s_at	0	7.782	1.12	7	5.804	1.81	40	d	0.008809
HSPA4L	1	heat shock 70kDa protein 4-like	205543_at	1	3.778	0.73	7	4.177	1.34	40	u	0.45408
HOXD4	1	homeobox D4	1552337_s_at	1	2.376	0.41	7	2.555	0.52	40	u	0.406193
			205522_at	0	4.777	0.96	7	3.819	1.01	40	d	0.026754
TRIM55	1	tripartite motif-containing 55	236175_at	1	2.535	0.07	7	2.684	0.28	40	u	0.008812
			232721_at	1	2.221	0.02	7	2.332	0.32	40	u	0.037287
TTBK2	1	tau tubulin kinase 2	213922_at	1	4.344	0.2	7	4.798	0.58	40	u	0.001276
			1554293_at	0	5.365	0.62	7	5.331	0.8	40	d	0.916087
			240011_at	0	2.166	0.1	7	2.214	0.12	40	u	0.324452
			1554294_s_at	0	2.824	0.11	7	2.86	0.27	40	u	0.735913
			1554295_x_at	0	2.998	0.3	7	2.925	0.49	40	d	0.71111
			231610_at	0	2.518	0.32	7	2.553	0.27	40	u	0.768169
			1557073_s_at	0	5.939	0.46	7	4.929	0.56	40	d	0.000069
RASGEF1C	1	RasGEF domain family, member 1C	236748_at	1	2.749	0.34	7	2.922	0.68	40	u	0.52374
CTXN1	1	cortexin 1	228126_x_at	1	5.505	0.82	7	7.655	1.4	40	u	0.000369
SIX3	1	SIX homeobox 3	206634_at	1	3.058	0.21	7	4.646	1.96	40	u	0.000015
DUSP26	1	dual specificity phosphatase 26 (putative)	219144_at	1	3.042	0.31	7	2.865	0.34	40	d	0.219556
ZNF410	1	zinc finger protein 410	209944_at	1	9.648	0.26	7	9.561	0.54	40	d	0.683385
			202010_s_at	1	8.479	0.25	7	8.266	0.46	40	d	0.252741
TMEM165	1	transmembrane protein 165	218095_s_at	1	9.024	0.29	7	9.49	0.68	40	u	0.08746
			222515_x_at	1	2.665	0.7	7	2.444	0.53	40	d	0.347121
			226827_at	0	3.046	0.25	7	3.338	0.4	40	u	0.075659
			226825_s_at	0	9.732	0.69	7	9.815	1.23	40	u	0.865929
			230512_x_at	0	2.691	0.46	7	2.514	0.16	40	d	0.385596
UHMK1	1	U2AF homology motif (UHM) kinase 1	227740_at	1	7.273	0.48	7	9.57	0.74	40	u	0
			235003_at	1	6.14	0.44	7	8.041	0.87	40	u	0.000001
			1552656_s_at	0	4.456	0.35	7	6.125	1.23	40	u	0
RNF20	1	ring finger protein 20	222683_at	1	9.19	0.17	7	9.039	0.54	40	d	0.188408
PPP3CC	1	protein phosphatase 3 (formerly 2B), catalytic subunit, gamma isoform	207000_s_at	1	5.936	0.51	7	5.633	0.74	40	d	0.314132
			209697_at	1	1.819	0.12	7	1.892	0.16	40	u	0.267267
			32541_at	1	6.457	0.4	7	5.757	0.79	40	d	0.029074
			32540_at	0	3.876	0.17	7	4.057	0.3	40	u	0.133101
			213950_s_at	0	2.772	0.06	7	2.909	0.24	40	u	0.006514
ZBTB43	1	zinc finger and BTB domain containing 43	204180_s_at	1	6.368	0.23	7	6.133	0.66	40	d	0.10902
			204182_s_at	1	6.125	0.33	7	5.501	0.83	40	d	0.059649
			204181_s_at	1	7.509	0.26	7	7.212	0.51	40	d	0.145298
CORO1B	1	coronin, actin binding protein, 1B	221754_s_at	1	7.278	0.26	7	6.948	0.72	40	d	0.046603
			64486_at	1	8.312	0.19	7	8.146	0.81	40	d	0.274431
LEFTY1	1	left-right determination factor 1	206268_at	1	2.709	0.4	7	2.622	0.14	40	d	0.612474
FSTL4	1	follistatin-like 4	214859_at	1	2.176	0.19	7	2.3	0.21	40	u	0.157838
			1564511_a_at	0	2.643	0.22	7	2.868	0.5	40	u	0.255353
			1565910_at	0	2.107	0.02	7	2.16	0.04	40	u	0.004313
EMP1	1	epithelial membrane protein 1	201324_at	1	10.89	0.63	7	9.486	1.19	40	d	0.004705
			213895_at	1	5.616	1.35	7	4.113	1	40	d	0.001438
			201325_s_at	1	10.28	0.72	7	8.853	1.1	40	d	0.002363
			1564796_at	0	7.004	1.12	7	5.924	0.86	40	d	0.006378
AKR1B1P2	1	keto reductase family 1, member B1 pseudoge	no probeset available									
ZC3HAV1	1	zinc finger CCCH-type, antiviral 1	225634_at	1	8.803	0.39	7	8.149	0.54	40	d	0.004677
			213051_at	0	8.669	0.47	7	8.092	0.69	40	d	0.042108
			220104_at	0	5.349	0.41	7	5.766	0.86	40	u	0.22251
CDCA7L	1	cell division cycle associated 7-like	225081_s_at	1	8.075	0.54	7	7.623	1.34	40	d	0.395717
UBL4A	1	ubiquitin-like 4A	221746_at	1	7.243	0.23	7	7.919	0.65	40	u	0.000061
KCNB2	1	potassium voltage-gated channel, Shab-related subfamily, member 2	208123_at	1	2.907	0.19	7	2.867	0.31	40	d	0.745575
			208172_s_at	0	2.219	0.05	7	2.288	0.13	40	u	0.196436
FAM110A	1	family with sequence similarity 110, member A	226584_s_at	1	6.806	0.38	7	6.999	0.98	40	u	0.61937
SLC19A3	1	solute carrier family 19, member 3	239345_at	1	4.232	0.92	7	3.509	0.52	40	d	0.108415
			220736_at	0	3.44	1.06	7	2.359	0.37	40	d	0.047816
RCOR2	1	REST corepressor 2	236030_at	1	6.332	0.19	7	6.533	0.4	40	u	0.202548

DDX42	1	DEAD (Asp-Glu-Ala-Asp) box polypeptide 42	201788_at	1	9.457	0.24	7	9.051	0.66	40	d	0.009436
			1559953_at	0	3.302	0.07	7	3.691	0.49	40	u	0.000028
			1559954_s_at	0	7.177	0.46	7	7.704	1.2	40	u	0.268444
LIMA1	1	LIM domain and actin binding 1	217892_s_at	1	10.59	0.35	7	8.855	1.16	40	d	0
			222456_s_at	1	4.681	0.81	7	4.532	0.96	40	d	0.704831
			222457_s_at	0	9.814	0.3	7	8.462	1.38	40	d	0.000004
VPS39	1	vacuolar protein sorting 39 homolog (S. cerevisiae)	212156_at	1	8.342	0.18	7	7.558	0.69	40	d	0.000001
ZNF76	1	zinc finger protein 76 (expressed in testis)	207494_s_at	1	7.434	0.23	7	7.114	0.54	40	d	0.135989
SUB1	1	SUB1 homolog (S. cerevisiae)	212857_x_at	1	11.82	0.26	7	12.17	0.56	40	u	0.110454
			221727_at	1	9.496	0.48	7	8.823	1.1	40	d	0.125843
			214512_s_at	1	11.27	0.38	7	11.37	0.79	40	u	0.766195
			224586_x_at	1	11.71	0.27	7	12	0.57	40	u	0.193523
			224587_at	1	10.27	0.46	7	10.12	1.12	40	d	0.729718
			237784_at	0	2.671	0.08	7	3.175	1.1	40	u	0.007611
AKTIP	1	AKT interacting protein	223894_s_at	1	7.655	0.32	7	7.364	1.03	40	d	0.17933
			218373_at	1	8.72	0.26	7	8.526	0.61	40	d	0.418954
UQCRFS1	1	ubiquinol-cytochrome c reductase, Rieske iron-sulfur polypeptide 1	208909_at	1	11.58	0.24	7	11.39	0.69	40	d	0.224609
ARL10	1	ADP-ribosylation factor-like 10	1553207_at	1	3.657	0.35	7	3.97	0.46	40	u	0.10247
			1553208_s_at	1	2.808	0.09	7	2.944	0.38	40	u	0.064551
			242411_at	0	2.807	0.26	7	2.629	0.24	40	d	0.089399
APOA5	1	apolipoprotein A-V	224244_s_at	1	2.161	0.08	7	2.208	0.17	40	u	0.484821
			224243_at	1	3.861	0.19	7	4.155	0.37	40	u	0.051983
NAT6	1	N-acetyltransferase 6	210874_s_at	1	4.3	0.33	7	3.817	0.75	40	d	0.108824
SMOX	1	spermine oxidase	217074_at	1	4.003	0.29	7	3.897	0.47	40	d	0.574934
			210357_s_at	1	6.324	0.27	7	6.566	0.66	40	u	0.355856
			1555680_a_at	0	3.522	0.63	7	3.685	0.71	40	u	0.580131
KRT76	1	keratin 76	207878_at	1	2.408	0.29	7	2.389	0.17	40	d	0.882238
PRUNE	1	prune homolog (Drosophila)	209599_s_at	1	6.113	0.6	7	6.911	1.32	40	u	0.131925
			209586_s_at	1	9.203	0.31	7	9.623	0.75	40	u	0.160361
			210988_s_at	0	6.755	0.35	7	7.245	1.16	40	u	0.045166
			232490_s_at	0	4.287	0.34	7	4.917	1.38	40	u	0.021504
RHBDD2	1	rhomboid domain containing 2	222995_s_at	1	9.336	0.29	7	8.923	0.84	40	d	0.02998
			232053_x_at	0	7.224	0.41	7	6.966	1.31	40	d	0.34456
HIGD1A	1	HIG1 domain family, member 1A	221896_s_at	1	10.46	0.41	7	10.21	0.84	40	d	0.460073
			217845_x_at	1	10.87	0.36	7	10.76	0.74	40	d	0.715204
WARS	1	tryptophanyl-tRNA synthetase	200629_at	1	8.34	0.25	7	8.92	1.16	40	u	0.009272
			200628_s_at	1	6.973	0.22	7	7.444	1.48	40	u	0.070793
MAGIX	1	MAGI family member, X-linked	219965_s_at	1	2.817	0.2	7	2.89	0.27	40	u	0.514633
			222944_s_at	1	2.518	0.06	7	2.66	0.22	40	u	0.002516
			1565562_at	0	5.164	0.11	7	5.422	0.35	40	u	0.001466
			1563318_s_at	0	4.143	0.41	7	4.351	0.75	40	u	0.490847
			234927_s_at	0	2.108	0.1	7	2.29	0.3	40	u	0.008553
			1565559_at	0	3.299	0.48	7	3.311	0.57	40	u	0.95942
LENG4	1	leukocyte receptor cluster (LRC) member 4	209179_s_at	1	8.722	0.35	7	8.612	0.98	40	d	0.613565
			211037_s_at	0	3.914	0.51	7	4.23	0.78	40	u	0.313683
PIP5K1A	1	phosphatidylinositol-4-phosphate 5-kinase, type I, alpha	207391_s_at	1	7.469	0.41	7	8.301	0.8	40	u	0.011336
			210256_s_at	0	4.753	0.35	7	5.593	1.43	40	u	0.003709
			211205_x_at	0	4.762	0.68	7	6.124	1.46	40	u	0.021803
BTBD2	1	BTB (POZ) domain containing 2	207722_s_at	1	8.143	0.4	7	7.322	0.95	40	d	0.032958
CSF3	1	colony stimulating factor 3 (granulocyte)	207442_at	1	5.253	0.36	7	5.212	0.49	40	d	0.835004
ACRBP	1	acrosin binding protein	230833_at	1	4.468	0.24	7	4.204	0.49	40	d	0.182101
			223718_at	1	2.215	0.02	7	2.266	0.06	40	u	0.000269
			223717_s_at	1	2.888	0.56	7	2.72	0.4	40	d	0.35261
SARM1	1	sterile alpha and TIR motif containing 1	213259_s_at	1	5.63	0.71	7	5.258	0.88	40	d	0.305226
			213257_at	1	3.897	0.64	7	4.048	0.73	40	u	0.617554
CYC1	1	cytochrome c-1	201066_at	1	9.664	0.25	7	10.43	0.97	40	u	0.000219
CHUK	1	conserved helix-loop-helix ubiquitous kinase	209666_s_at	1	6.885	0.39	7	7.406	0.58	40	u	0.031667
RAB33A	1	RAB33A, member RAS oncogene family	206039_at	1	2.423	0.16	7	2.555	0.4	40	u	0.40607
ETV4	1	ets variant gene 4 (E1A enhancer binding protein, E1AF)	211603_s_at	1	6.613	0.66	7	6.364	1.02	40	d	0.545019
			1554576_a_at	0	5	0.5	7	4.373	1.32	40	d	0.231457
CYBASC3	1	cytochrome b, ascorbate dependent 3	224735_at	1	8.313	0.19	7	8.458	0.62	40	u	0.257119

CCDC86	1	coiled-coil domain containing 86	203119_at	1	7.044	0.31	7	7.287	0.83	40	u	0.459521
ARMC7	1	armadillo repeat containing 7	219096_at	1	7.393	0.26	7	7.299	0.42	40	d	0.576306
			229906_at	0	4.735	0.43	7	4.876	0.57	40	u	0.54352
TMEM150	1	transmembrane protein 150	226239_at	1	6.908	0.15	7	6.931	0.39	40	u	0.87963
ARG1	1	arginase, liver	206177_s_at	1	2.917	0.1	7	3.197	0.45	40	u	0.001692
			231662_at	0	2.458	0.15	7	2.563	0.17	40	u	0.146559
			231665_at	0	2.555	0.07	7	2.698	0.18	40	u	0.05087
			231663_s_at	0	2.184	0.09	7	2.239	0.17	40	u	0.425859
CENTD3	1	centaurin, delta 3	218950_at	1	6.953	0.57	7	5.634	0.76	40	d	0.000089
			235806_at	0	2.044	0.02	7	2.09	0.03	40	u	0.001608
CNTNAP4	1	contactin associated protein-like 4	232388_at	1	3.312	0.22	7	3.489	0.36	40	u	0.226401
			1553441_at	0	2.816	0.08	7	3.034	0.37	40	u	0.002689
			1553442_a_at	0	2.424	0.03	7	2.551	0.27	40	u	0.00702
			1554377_a_at	0	2.293	0.15	7	2.305	0.09	40	u	0.85097
CHST14	1	carbohydrate (N-acetylgalactosamine 4-0) sulfotransferase 14	226314_at	1	7.446	0.31	7	6.714	0.75	40	d	0.016672
MCHR1	1	melanin-concentrating hormone receptor 1	230498_at	1	2.151	0.04	7	2.455	0.7	40	u	0.010445
			221354_s_at	0	1.797	0.04	7	1.853	0.11	40	u	0.018703
			223855_s_at	0	2.701	0.11	7	2.743	0.22	40	u	0.629253
ATF5	1	activating transcription factor 5	204998_s_at	1	7.518	0.37	7	8.019	0.89	40	u	0.157436
			204999_s_at	0	5.704	0.25	7	6.468	1.09	40	u	0.000524
			230938_x_at	0	6.99	0.14	7	6.93	0.32	40	d	0.63171
			217389_s_at	0	5.795	0.23	7	5.791	0.31	40	d	0.979852
GABARAPL1	1	GABA(A) receptor-associated protein like 1	208869_s_at	1	8.657	0.37	7	7.871	0.65	40	d	0.003646
			208868_s_at	1	8.313	0.4	7	7.439	0.62	40	d	0.000968
			211458_s_at	0	9.965	0.38	7	8.702	0.89	40	d	0.000795
HTRA3	1	HtrA serine peptidase 3	226944_at	1	6.697	0.78	7	6.541	1.04	40	d	0.711868
			228580_at	0	4.985	0.42	7	4.556	0.49	40	d	0.040155
BMP4	1	bone morphogenetic protein 4	211518_s_at	0	5.881	0.69	7	4.484	1.16	40	d	0.00421
OSTM1	1	osteopetrosis associated transmembrane protein 1	243287_s_at	1	2.931	0.12	7	3.222	0.63	40	u	0.013166
			235198_at	1	5.307	0.41	7	5.797	1.01	40	u	0.220053
			218196_at	1	7.301	0.3	7	7.379	0.8	40	u	0.804929
			235197_s_at	1	4.147	0.24	7	4.591	0.74	40	u	0.007985
DPY19L2	1	dpy-19-like 2 (C. elegans)	230158_at	1	3.905	0.73	7	3.73	0.86	40	d	0.620362
			238784_at	0	3.068	0.51	7	3.216	1.03	40	u	0.717967
			1557290_at	0	2.148	0.01	7	2.271	0.25	40	u	0.00387
IL1RAP	1	interleukin 1 receptor accessory protein	205227_at	1	5.841	0.17	7	5.905	0.55	40	u	0.568366
			210233_at	0	3.071	0.1	7	3.699	0.75	40	u	0.000011
PRDX5	1	peroxiredoxin 5	1560587_s_at	1	11.07	0.13	7	10.61	0.78	40	d	0.001589
			222994_at	0	10.12	0.15	7	9.655	0.75	40	d	0.001265
VDAC1	1	voltage-dependent anion channel 1	212038_s_at	1	10.27	0.35	7	10.5	0.67	40	u	0.404089
			217140_s_at	0	9.406	0.39	7	9.6	1.03	40	u	0.63134
			217139_at	0	2.389	0.06	7	2.575	0.21	40	u	0.000067
TRIB1	1	tribbles homolog 1 (Drosophila)	202241_at	1	10.22	0.33	7	9.482	0.94	40	d	0.00121
			235641_at	0	2.014	0.04	7	2.087	0.14	40	u	0.015104
			239818_x_at	0	3.37	1.44	7	3.189	1.49	40	d	0.771251
TXNDC5	1	thioredoxin domain containing 5	221253_s_at	1	11.77	0.21	7	11.5	0.78	40	d	0.086234
PRMT6	1	protein arginine methyltransferase 6	223275_at	1	6.741	0.39	7	6.501	1.08	40	d	0.318676
CCK	1	cholecystokinin	205827_at	1	2.266	0.08	7	2.548	0.89	40	u	0.060915
TYSND1	1	trypsin domain containing 1	225873_at	1	3.18	0.24	7	3.24	0.36	40	u	0.679445
			231422_x_at	1	4.078	0.39	7	4.04	0.37	40	d	0.807791
			225877_at	1	6.578	0.29	7	6.507	0.68	40	d	0.790664
			238802_at	0	6.101	0.57	7	5.32	0.77	40	d	0.015637
TBP	1	TATA box binding protein	203135_at	1	7.362	0.3	7	6.963	0.44	40	d	0.029407
SLC24A2	1	solute carrier family 24 (sodium/potassium/calcium exchanger), member 2	220867_s_at	1	2.367	0.14	7	2.432	0.21	40	u	0.43971
LRRTM1	1	leucine rich repeat transmembrane neuronal 1	238815_at	1	2.447	0.1	7	2.475	0.16	40	u	0.659079
GGTL3	1	gamma-glutamyltransferase-like 3	226470_at	1	7.33	0.52	7	6.21	1	40	d	0.006659
			226471_at	1	4.874	0.42	7	4.518	0.43	40	d	0.053432
			226469_s_at	1	5.062	0.64	7	4.549	0.57	40	d	0.041438
RHCG	1	Rh family, C glycoprotein	219554_at	0	2.195	0.02	7	2.355	0.33	40	u	0.005221
DDX46	1	DEAD (Asp-Glu-Ala-Asp) box polypeptide 46	228039_at	1	6.539	0.47	7	6.377	0.7	40	d	0.568975

DDX46	1	DEAD (Asp-Glu-Ala-Asp) box polypeptide 46	202462_s_at	0	9.058	0.21	7	9.013	0.45	40	d	0.801006
VPS13A	1	vacuolar protein sorting 13 homolog A (S. cerevisiae)	227988_s_at	1	7.651	0.3	7	7.004	0.77	40	d	0.037991
			231585_at	1	4.157	0.46	7	4.14	0.36	40	d	0.913044
			227987_at	1	6.497	0.26	7	6.391	0.68	40	d	0.693849
			214785_at	0	7.399	0.26	7	7.267	0.82	40	d	0.439553
			214852_x_at	0	5.073	0.36	7	5.06	0.48	40	d	0.947193
CMTM6	1	CKLF-like MARVEL transmembrane domain containing 6	223047_at	1	9.803	0.4	7	9.643	0.78	40	d	0.606579
			217947_at	1	11.27	0.22	7	10.94	0.66	40	d	0.025816
			1565657_at	0	3.317	0.35	7	3.397	0.22	40	u	0.440553
GRB14	1	growth factor receptor-bound protein 14	206204_at	1	2.892	0.37	7	4.677	2.53	40	u	0.000162
ILF3	1	interleukin enhancer binding factor 3, 90kDa	217805_at	1	8.584	0.41	7	8.865	0.65	40	u	0.283108
			217804_s_at	1	8.822	0.37	7	8.556	0.71	40	d	0.35137
			211375_s_at	0	9.489	0.36	7	9.909	0.61	40	u	0.089161
			208931_s_at	0	8.761	0.41	7	8.748	0.83	40	d	0.968177
			208930_s_at	0	5.896	0.38	7	7.321	0.94	40	u	0.000358
RP2	1	retinitis pigmentosa 2 (X-linked recessive)	205191_at	1	5.267	0.48	7	5.894	0.69	40	u	0.029845
			1568782_at	0	2.048	0.02	7	2.178	0.17	40	u	0.000056
ZNF444	1	zinc finger protein 444	218707_at	1	7.722	0.22	7	7.512	0.51	40	d	0.302395
			50376_at	1	8.501	0.23	7	8.24	0.62	40	d	0.286221
SGCG	1	sarcoglycan, gamma (35kDa dystrophin-associated glycoprotein)	207302_at	1	2.621	0.18	7	2.659	0.35	40	u	0.782257
DCDC2	1	doublecortin domain containing 2	222925_at	1	5.927	2.15	7	3.206	0.96	40	d	0.022542
			222926_at	1	2.458	0.06	7	2.54	0.16	40	u	0.197315
NANP	1	N-acetylneuraminic acid phosphatase	228073_at	1	6.842	0.25	7	7.58	0.74	40	u	0.000082
TYRO3	1	TYRO3 protein tyrosine kinase	211432_s_at	1	5.634	0.3	7	5.84	0.76	40	u	0.489557
			211431_s_at	0	2.432	0.31	7	2.488	0.44	40	u	0.756306
BAT3	1	HLA-B associated transcript 3	230513_at	0	2.978	0.48	7	2.905	0.41	40	d	0.681171
			213318_s_at	0	9.981	0.34	7	9.622	0.75	40	d	0.231779
			210208_x_at	0	9.735	0.27	7	9.385	0.71	40	d	0.215066
			201255_x_at	0	9.506	0.26	7	9.135	0.73	40	d	0.027608
CHKA	1	choline kinase alpha	204233_s_at	1	7.768	0.55	7	7.327	1.02	40	d	0.28009
			204266_s_at	1	8.281	0.56	7	7.699	0.84	40	d	0.090339
RAB31	1	RAB31, member RAS oncogene family	217763_s_at	1	8.149	0.87	7	8.865	1.2	40	u	0.147581
			217764_s_at	1	8.721	0.87	7	9.303	1.18	40	u	0.230034
			217762_s_at	1	9.149	0.96	7	9.925	1.16	40	u	0.108237
ANKRD9	1	ankyrin repeat domain 9	230972_at	1	7.306	0.4	7	6.691	1.07	40	d	0.149216
ALDOA	1	aldolase A, fructose-bisphosphate	214687_x_at	1	12.65	0.25	7	12.49	0.8	40	d	0.349854
			200966_x_at	1	12.75	0.27	7	12.54	0.81	40	d	0.216983
			238996_x_at	0	6.898	0.17	7	7.187	0.72	40	u	0.038087
THAP3	1	THAP domain containing, apoptosis associated protein 3	217658_at	0	5.36	0.54	7	4.934	0.52	40	d	0.058605
			213192_at	0	4.063	0.34	7	4.234	0.44	40	u	0.339735
SPATS2	1	spermatogenesis associated, serine-rich 2	222594_s_at	1	4.833	0.36	7	5.774	0.74	40	u	0.002419
			222593_s_at	1	7.351	0.33	7	8.524	0.69	40	u	0.00009
			218324_s_at	1	6.679	0.45	7	7.451	0.65	40	u	0.004948
			1570130_at	0	3.221	0.4	7	3.665	0.5	40	u	0.036007
IL12A	1	interleukin 12A (natural killer cell stimulatory factor 1, cytotoxic lymphocyte maturation factor 1, p35)	207160_at	0	2.354	0.13	7	2.454	0.57	40	u	0.344232
FUZ	1	fuzzy homolog (Drosophila)	221187_s_at	4	7.359	0.41	7	6.909	0.53	40	d	0.043766
KIAA1539	1	KIAA1539	211433_x_at	1	7.307	0.17	7	7.366	0.54	40	u	0.602538
			207765_s_at	1	7.052	0.31	7	7.197	0.5	40	u	0.474395
RFXANK	1	regulatory factor X-associated ankyrin-containing protein	202758_s_at	2	7.904	0.27	7	8.107	0.8	40	u	0.236871
SPATA7	1	spermatogenesis associated 7	219583_s_at	1	6.32	0.35	7	5.803	0.75	40	d	0.086383
			234929_s_at	1	5.418	0.2	7	5.059	0.46	40	d	0.053708
EIF2AK3	1	eukaryotic translation initiation factor 2-alpha kinase 3	218696_at	1	8.6	0.22	7	8.682	0.7	40	u	0.573681
KCNK4	1	potassium channel, subfamily K, member 4	224552_s_at	0	1.969	0.02	7	2.006	0.03	40	u	0.00408
CORO6	1	coronin 6	1552301_a_at	1	4.809	0.79	7	4.449	1.01	40	d	0.384238
RAN	1	RAN, member RAS oncogene family	200749_at	1	9.682	0.24	7	9.39	0.76	40	d	0.070921
			200750_s_at	1	11.32	0.15	7	11.94	0.78	40	u	0.000067
FAM131A	1	family with sequence similarity 131, member A	221904_at	1	7.527	0.18	7	7.139	1.22	40	d	0.068916
			232807_at	0	4.924	0.22	7	4.613	0.45	40	d	0.087038

			232806_s_at	0	3.642	0.53	7	3.36	0.8	40	d	0.384697
EEF1A1	1	eukaryotic translation elongation factor 1 alpha 1	213477_x_at	1	15.23	0.07	7	15.15	0.11	40	d	0.090335
			206559_x_at	1	15.04	0.09	7	14.86	0.17	40	d	0.013896
			213614_x_at	1	15.17	0.1	7	15.09	0.14	40	d	0.16648
			227708_at	0	9.388	0.32	7	7.238	1.18	40	d	0
			213583_x_at	0	14.89	0.08	7	14.8	0.15	40	d	0.137761
			204892_x_at	0	15.03	0.11	7	14.83	0.18	40	d	0.00667
			1557120_at	0	3.079	0.26	7	3.061	0.3	40	d	0.88472
GMEB2	1	glucocorticoid modulatory element binding protein 2	222251_s_at	1	6.927	0.42	7	7.1	0.68	40	u	0.52529
			44146_at	1	7.752	0.26	7	7.649	0.54	40	d	0.630371
TEF	1	thyrotrophic embryonic factor	225840_at	1	7.636	0.6	7	6.172	0.62	40	d	0.000001
			215675_at	0	2.542	0.16	7	2.548	0.29	40	u	0.952917
			210167_s_at	0	2.545	0.23	7	2.582	0.22	40	u	0.689157
EPHA1	1	EPH receptor A1	215804_at	1	2.319	0.19	7	2.287	0.27	40	d	0.770057
			205977_s_at	1	6.122	0.59	7	5.979	0.67	40	d	0.605672
C1orf109	1	chromosome 1 open reading frame 109	218712_at	1	6.356	0.37	7	6.585	0.68	40	u	0.398656
ANGPT2	1	angiopoietin 2	205572_at	1	4.716	0.84	7	4.823	1.02	40	u	0.797485
			211148_s_at	0	4.369	0.53	7	4.276	0.8	40	d	0.772798
SEL1L	1	sel-1 suppressor of lin-12-like (C. elegans)	202063_s_at	1	2.5	0.13	7	2.68	0.59	40	u	0.105858
			202064_s_at	1	6.229	0.33	7	6.555	0.79	40	u	0.298903
			202061_s_at	1	10.04	0.22	7	9.408	0.68	40	d	0.000128
			230265_at	0	4.592	0.52	7	4.794	0.99	40	u	0.610199
			202062_s_at	0	6.451	0.57	7	6.311	0.98	40	d	0.720936
TMSB4Y	1	thymosin, beta 4, Y-linked	206769_at	0	6.613	0.37	7	6.726	0.42	40	u	0.521402
AP1GBP1	1	AP1 gamma subunit binding protein 1	222715_s_at	1	5.393	0.3	7	5.503	1.02	40	u	0.597135
			232387_at	1	2.535	0.41	7	2.597	0.42	40	u	0.726074
			232981_s_at	0	5.265	0.32	7	5.229	0.74	40	d	0.900106
			1553165_at	0	3.897	0.57	7	4.05	0.77	40	u	0.624058
FAM71C	1	family with sequence similarity 71, member C	1552526_at	1	2.106	0.12	7	2.111	0.11	40	u	0.904166
NSUN2	1	NOL1/NOP2/Sun domain family, member 2	223076_s_at	1	8.543	0.95	7	8.463	0.64	40	d	0.785357
ARPC2	1	actin related protein 2/3 complex, subunit 2, 34kDa	213513_x_at	1	9.441	0.25	7	10.05	0.37	40	u	0.000158
			208679_s_at	0	11.73	0.39	7	12.38	0.6	40	u	0.009249
			207988_s_at	0	10.48	0.37	7	11.12	0.4	40	u	0.000352
ZNF329	1	zinc finger protein 329	219765_at	1	6.899	0.3	7	6.416	0.97	40	d	0.021294
P2RY13	1	purinergic receptor P2Y, G-protein coupled, 13	220005_at	1	4.2	0.99	7	2.939	0.77	40	d	0.000506
NMUR1	1	neuromedin U receptor 1	221383_at	0	2.82	0.3	7	2.914	0.25	40	u	0.390642
MARCH2	1	membrane-associated ring finger (C3HC4) 2	210075_at	1	7.8	0.23	7	7.161	1.13	40	d	0.002999
SMYD4	1	SET and MYND domain containing 4	229175_at	1	5.353	0.44	7	5.225	0.72	40	d	0.655777
MEN1	1	multiple endocrine neoplasia I	202645_s_at	1	7.512	0.33	7	7.266	0.82	40	d	0.449775
CLDN15	1	claudin 15	1552845_at	1	1.488	0.03	7	1.51	0.04	40	u	0.133691
			219640_at	1	5.208	0.32	7	5.055	0.49	40	d	0.437477
SFRP5	1	secreted frizzled-related protein 5	207468_s_at	1	5.405	0.26	7	5.548	0.56	40	u	0.519214
IKZF2	1	IKAROS family zinc finger 2 (Helios)	220567_at	1	2.276	0.11	7	2.397	0.29	40	u	0.300057
			1555060_a_at	1	2.257	0.06	7	2.366	0.19	40	u	0.011538
			231929_at	0	6.537	0.59	7	6.32	1.63	40	d	0.548204
EBAG9	1	estrogen receptor binding site associated, antigen, 9	204274_at	1	9.889	0.19	7	10.02	0.75	40	u	0.359254
			204278_s_at	0	8.056	0.36	7	8.769	0.8	40	u	0.027768
SESN3	1	sestrin 3	235683_at	1	3.538	0.26	7	4.053	1.17	40	u	0.021847
			235684_s_at	1	3.02	0.1	7	3.265	0.36	40	u	0.001499
			1553869_at	0	2.189	0.07	7	2.351	0.27	40	u	0.004569
DPP3	1	dipeptidyl-peptidase 3	218567_x_at	1	6.757	0.42	7	7.901	0.97	40	u	0.004196
			232510_s_at	0	7.234	0.41	7	8.39	0.92	40	u	0.002496
TUBB2A	1	tubulin, beta 2A	204141_at	1	10.6	0.17	7	9.968	0.99	40	d	0.000657
			209372_x_at	1	7.1	0.28	7	6.999	0.98	40	d	0.605457
TTLL12	1	tubulin tyrosine ligase-like family, member 12	216251_s_at	1	6.789	0.31	7	7.375	1.01	40	u	0.008128
			1552257_a_at	1	8.13	0.32	7	8.783	0.94	40	u	0.003337
SERPINH1	1	serpin peptidase inhibitor, clade H (heat shock protein 47), member 1, (collagen binding protein 1)	207714_s_at	1	8.822	0.42	7	9.406	0.85	40	u	0.089331
			227918_s_at	1	4.385	0.88	7	4.901	1.09	40	u	0.25127

ZYG11B	1	zyg-11 homolog B (C. elegans)	225350_s_at	1	8.755	0.1	7	8.498	0.51	40	d	0.00706
			225338_at	1	7.639	0.27	7	7.584	0.55	40	d	0.803282
TLE1	1	transducin-like enhancer of split 1 (E(sp1) homolog, Drosophila)	203221_at	1	8.238	0.34	7	7.818	1.05	40	d	0.064504
			203220_s_at	1	4.682	0.44	7	4.539	1	40	d	0.717161
			203222_s_at	0	7.221	0.32	7	6.652	1.17	40	d	0.017477
			228284_at	0	7.079	0.39	7	7.095	1.33	40	u	0.952563
			227130_s_at	0	2.297	0.02	7	2.385	0.1	40	u	0.000013
LAYN	1	layilin	228080_at	0	7.239	0.57	7	4.938	1.17	40	d	0.000009
			1556885_at	0	2.445	0.13	7	2.515	0.25	40	u	0.487865
			1556886_a_at	0	2.185	0.02	7	2.237	0.07	40	u	0.001629
LEFTY2	1	left-right determination factor 2	206012_at	1	3.106	0.32	7	3.473	1.36	40	u	0.157303
PIAS2	1	protein inhibitor of activated STAT, 2	37433_at	1	3.658	0.3	7	4.06	0.73	40	u	0.166972
			214593_at	0	2.966	0.17	7	3.1	0.44	40	u	0.444593
			214442_s_at	0	6.74	0.42	7	7.092	0.84	40	u	0.295655
			1555514_a_at	0	4.822	0.31	7	5.47	0.86	40	u	0.001988
			1555513_at	0	2.553	0.05	7	2.731	0.19	40	u	0.000026
			243624_at	0	3.229	0.23	7	3.51	0.82	40	u	0.09163
PNRC2	1	proline-rich nuclear receptor coactivator 2	217779_s_at	1	11.4	0.15	7	10.63	0.51	40	d	0
			222406_s_at	1	8.343	0.43	7	8.321	0.68	40	d	0.935786
DENND2C	1	DENN/MADD domain containing 2C	233294_at	1	2.211	0.02	7	2.273	0.12	40	u	0.005241
			230769_at	1	5.725	0.59	7	5.105	0.75	40	d	0.049272
GPR37	1	G protein-coupled receptor 37 (endothelin receptor type B-like)	209631_s_at	1	4.41	0.33	7	5.472	1.55	40	u	0.0005
			214586_at	1	2.969	0.07	7	3.411	0.62	40	u	0.000115
SLC22A17	1	solute carrier family 22 (organic cation transporter), member 17	218675_at	1	6.862	0.38	7	5.68	1.07	40	d	0.000031
			221106_at	0	2.112	0.19	7	2.043	0.1	40	d	0.425127
KCNJ1	1	potassium inwardly-rectifying channel, subfamily J, member 1	210402_at	1	2.481	0.03	7	2.646	0.29	40	u	0.001412
			210403_s_at	0	2.028	0.03	7	2.141	0.2	40	u	0.001711
SFRS15	1	splicing factor, arginine/serine-rich 15	226082_s_at	1	6.141	0.42	7	5.954	0.66	40	d	0.481389
			222311_s_at	0	6.678	0.3	7	5.927	0.84	40	d	0.000404
			243759_at	0	5.994	0.55	7	5.175	0.95	40	d	0.034859
			233753_at	0	2.345	0.05	7	2.563	0.26	40	u	0.00003
			222310_at	0	5.879	0.31	7	5.297	0.94	40	d	0.006748
EIF1B	1	eukaryotic translation initiation factor 1B	201738_at	1	9.835	0.18	7	9.101	0.53	40	d	0.000001
			237988_at	0	2.99	0.1	7	3.243	0.37	40	u	0.001259
ATP1A2	1	ATPase, Na+/K+ transporting, alpha 2 (+) polypeptide	203296_s_at	1	4.91	1.54	7	2.893	0.19	40	d	0.018688
			203295_s_at	1	3.969	1.05	7	3.013	0.41	40	d	0.069628
ACAD9	1	acyl-Coenzyme A dehydrogenase family, member 9	224160_s_at	0	8.012	0.22	7	8.111	0.61	40	u	0.464322
			239976_at	0	2.546	0.23	7	2.78	0.4	40	u	0.146448
INHBA	1	inhibin, beta A	204926_at	1	3.828	0.54	7	4.44	0.71	40	u	0.038913
			210511_s_at	0	5.852	0.39	7	8.962	1.39	40	u	0
METTL9	1	methyltransferase like 9	222447_at	1	7.532	0.42	7	7.823	0.87	40	u	0.401926
			217868_s_at	1	10.2	0.21	7	10.76	0.56	40	u	0.014134
			226220_at	1	3.001	0.24	7	3.315	0.85	40	u	0.067182
			230395_at	0	5.806	0.59	7	6.192	1.28	40	u	0.447255
MAP2K5	1	mitogen-activated protein kinase kinase 5	204756_at	1	6.089	0.22	7	5.614	0.65	40	d	0.001689
			216765_at	0	3.499	0.41	7	3.422	0.42	40	d	0.662507
			210482_x_at	0	5.615	0.76	7	5.436	0.79	40	d	0.591375
			216435_at	0	2.313	0.02	7	2.378	0.07	40	u	0.000148
			211370_s_at	0	6.709	0.2	7	5.757	0.78	40	d	0
			211371_at	0	2.684	0.04	7	2.75	0.08	40	u	0.038818
IGF2	1	insulin-like growth factor 2 (somatomedin A)	202409_at	1	11.7	0.54	7	6.449	1.78	40	d	0
			210881_s_at	0	5.252	1.67	7	2.365	0.35	40	d	0.005583
			202410_x_at	0	7.086	1.21	7	3.29	0.99	40	d	0
SENP2	1	SUMO1/sentrin/SMT3 specific peptidase 2	222523_at	1	7.397	0.35	7	7.479	0.59	40	u	0.728476
			218122_s_at	1	6.361	0.26	7	6.19	0.89	40	d	0.341998
RASGRP1	1	RAS guanyl releasing protein 1 (calcium and DAG-regulated)	205590_at	1	5.502	0.55	7	6.578	1.71	40	u	0.005172
PLXDC2	1	plexin domain containing 2	227276_at	1	7.096	0.37	7	6.817	1.17	40	d	0.255049
GSPT2	1	G1 to S phase transition 2	205541_s_at	1	7.19	0.26	7	5.38	1.77	40	d	0
LIFR	1	leukemia inhibitory factor receptor alpha	205876_at	1	3.544	0.51	7	3.167	0.85	40	d	0.272705
			227771_at	0	7.301	0.67	7	5.077	1.26	40	d	0.000052
			225571_at	0	7.249	0.72	7	4.783	1.21	40	d	0.000006
			225575_at	0	8.933	0.36	7	5.468	1.38	40	d	0
ZBTB33	1	zinc finger and BTB domain containing 33	226255_at	1	9.527	0.32	7	9.294	0.81	40	d	0.464516

ZBTB33	1	zinc finger and BTB domain containing 33	214631_at	0	3.422	0.09	7	3.815	0.49	40	u	0.000043
CHST9	1	carbohydrate (N-acetylgalactosamine 4-0) sulfotransferase 9	223737_x_at	1	3.652	0.75	7	2.838	0.99	40	d	0.048495
			224400_s_at	1	3.491	0.86	7	2.973	0.93	40	d	0.185575
EIF4A1	1	eukaryotic translation initiation factor 4A, isoform 1	211787_s_at	1	12.17	0.15	7	12.07	0.47	40	d	0.293189
			201530_x_at	1	12.59	0.12	7	12.46	0.44	40	d	0.134193
			214805_at	0	7.38	0.35	7	5.787	0.93	40	d	0.000071
FLT3	1	fms-related tyrosine kinase 3	206674_at	1	3.159	0.67	7	3.026	0.79	40	d	0.681729
ABI1	1	abl-interactor 1	209027_s_at	1	9.093	0.36	7	9.662	1.07	40	u	0.018052
			209028_s_at	1	9.156	0.35	7	9.454	0.71	40	u	0.290119
			1559890_a_at	0	3.224	0.21	7	3.627	0.47	40	u	0.034409
CACNA1A	1	calcium channel, voltage-dependent, P/Q type, alpha 1A subunit	210770_s_at	1	3.51	0.66	7	3.8	0.95	40	u	0.452231
			214933_at	1	5.514	0.47	7	5.708	1.09	40	u	0.652393
			206399_x_at	1	6.168	0.34	7	5.758	0.43	40	d	0.023533
			1558945_s_at	0	2.609	0.05	7	2.865	0.49	40	u	0.002929
			1558944_at	0	3.802	0.35	7	3.86	0.39	40	u	0.719178
SPG21	1	spastic paraplegia 21 (autosomal recessive, Mast syndrome)	217827_s_at	1	10.38	0.27	7	10.47	0.56	40	u	0.670611
			215383_x_at	0	7.244	0.24	7	7.121	0.46	40	d	0.500765
CDH1	1	cadherin 1, type 1, E-cadherin (epithelial)	201131_s_at	1	11.76	0.36	7	10.93	1.31	40	d	0.002446
			201130_s_at	0	7.921	0.75	7	6.581	2.11	40	d	0.007479
ZNF302	1	zinc finger protein 302	218490_s_at	1	8.657	0.2	7	8.085	0.7	40	d	0.000258
			228393_s_at	0	7.108	0.54	7	6.804	1.05	40	d	0.469384
			228392_at	0	7.832	0.43	7	7.441	0.81	40	d	0.23001
GRIN1	1	glutamate receptor, ionotropic, N-methyl D-aspartate 1	205914_s_at	1	2.213	0.29	7	2.218	0.18	40	u	0.950333
			211125_x_at	1	2.038	0.16	7	2.017	0.07	40	d	0.771407
			210781_x_at	1	2.148	0.1	7	2.119	0.11	40	d	0.542439
			210782_x_at	1	6.214	0.25	7	5.989	0.35	40	d	0.114336
			205915_x_at	0	2.082	0.03	7	2.136	0.07	40	u	0.046539
BPIL1	1	bactericidal/permeability-increasing protein-like 1	232698_at	1	3.858	0.19	7	4.114	1.04	40	u	0.170389
CREG1	1	cellular repressor of E1A-stimulated genes 1	201200_at	1	11.01	0.16	7	10.66	0.79	40	d	0.015778
HSPA5	1	heat shock 70kDa protein 5 (glucose-regulated protein, 78kDa)	230031_at	1	6.524	0.39	7	5.651	0.82	40	d	0.009549
			211936_at	1	11.71	0.35	7	12.04	0.69	40	u	0.236119
JUNB	1	jun B proto-oncogene	201473_at	1	10.4	1.02	7	9.249	0.97	40	d	0.007064
H6PD	1	hexose-6-phosphate dehydrogenase (glucose 1-dehydrogenase)	226160_at	1	7.619	0.63	7	7.29	0.68	40	d	0.25114
			206933_s_at	1	1.99	0.17	7	2.058	0.18	40	u	0.368215
			1555685_at	0	2.401	0.08	7	2.497	0.12	40	u	0.057785
			221892_at	0	7.222	0.41	7	6.576	0.59	40	d	0.00925
FAIM2	1	Fas apoptotic inhibitory molecule 2	203618_at	1	4.328	0.59	7	3.961	0.48	40	d	0.086426
			203619_s_at	0	3.236	0.37	7	3.482	1.26	40	u	0.338307
HOOK2	1	hook homolog 2 (Drosophila)	218780_at	1	8.804	0.32	7	8.163	0.85	40	d	0.059177
HNRPAB	1	heterogeneous nuclear ribonucleoprotein A/B	201277_s_at	1	10.88	0.17	7	11.3	0.6	40	u	0.001402
			231060_at	0	2.545	0.07	7	2.599	0.08	40	u	0.105508
			235669_at	0	1.965	0.15	7	1.992	0.18	40	u	0.717717
MTMR7	1	myotubularin related protein 7	217292_at	0	2.473	0.12	7	2.607	0.23	40	u	0.145857
			243804_at	0	2.529	0.06	7	2.735	0.52	40	u	0.022282
			215793_at	0	2.412	0.14	7	2.452	0.11	40	u	0.416639
HADH	1	hydroxyacyl-Coenzyme A dehydrogenase	201036_s_at	1	8.864	0.08	7	8.076	0.71	40	d	0
			211569_s_at	1	8.958	0.09	7	7.979	0.91	40	d	0
			201035_s_at	0	8.568	0.28	7	7.997	0.72	40	d	0.047595
PPP2R1A	1	protein phosphatase 2 (formerly 2A), regulatory subunit A , alpha isoform	200695_at	0	9.337	0.33	7	8.948	0.71	40	d	0.169512
CACNG3	1	calcium channel, voltage-dependent, gamma subunit 3	206384_at	1	2.244	0.03	7	2.344	0.16	40	u	0.000679
PFDN6	1	prefoldin subunit 6	233588_x_at	1	6.568	0.45	7	7.492	0.78	40	u	0.004464
			222029_x_at	1	6.641	0.41	7	7.454	0.81	40	u	0.01427
			222019_at	0	2.912	0.32	7	3.404	0.62	40	u	0.050912
			242048_at	0	4.154	0.34	7	4.158	0.56	40	u	0.986997
CNTNAP3	1	contactin associated protein-like 3	223796_at	1	5.24	0.37	7	4	0.84	40	d	0.000467
			233202_at	0	2.661	0.24	7	2.629	0.2	40	d	0.711244
IFI30	1	interferon, gamma-inducible protein 30	201422_at	1	9.378	0.24	7	10.97	1.05	40	u	0
MDK	1	midkine (neurite growth-promoting factor 2)	209035_at	1	8.648	1	7	8.471	1.56	40	d	0.778198
UBE2E1	1	ubiquitin-conjugating enzyme E2E 1 (UBC4/5 homolog, yeast)	212519_at	1	11.36	0.18	7	11.7	0.57	40	u	0.007322

MICAL2	1	microtubule associated monoxygenase, calponin and LIM domain containing 2	212473_s_at	1	7.878	0.48	7	8.736	1.11	40	u	0.054477
			212472_at	1	6.965	0.53	7	7.455	1.01	40	u	0.227407
			206275_s_at	1	4.246	0.39	7	4.396	0.54	40	u	0.496923
			236475_at	0	5.303	0.32	7	5.065	0.67	40	d	0.37336
PRR15	1	proline rich 15	226961_at	1	6.822	0.57	7	6.433	1.83	40	d	0.306882
GJB2	1	gap junction protein, beta 2, 26kDa	223278_at	1	3.849	0.55	7	7.138	1.56	40	u	0
SOX8	1	SRY (sex determining region Y)-box 8	226913_s_at	1	4.327	1.09	7	5.002	2.77	40	u	0.535893
PARP10	1	poly (ADP-ribose) polymerase family, member 10	229350_x_at	1	7.274	0.26	7	7.391	0.94	40	u	0.531814
			228669_x_at	0	2.135	0.22	7	2.103	0.12	40	d	0.736703
			226401_at	0	5.877	0.51	7	5.832	1.32	40	d	0.930642
			238083_at	0	2.217	0.06	7	2.299	0.14	40	u	0.142127
TMEM35	1	transmembrane protein 35	219685_at	1	2.13	0.08	7	2.372	0.91	40	u	0.114223
BRSK2	1	BR serine/threonine kinase 2	223715_at	1	1.944	0.07	7	2.065	0.2	40	u	0.009666
			215010_s_at	0	2.552	0.07	7	2.706	0.37	40	u	0.024084
RAB3IL1	1	RAB3A interacting protein (rabin3)-like 1	219579_at	1	6.287	0.47	7	6.64	0.66	40	u	0.189615
ABCF3	1	ATP-binding cassette, sub-family F (GCN20), member 3	202394_s_at	1	6.683	0.11	7	6.868	0.53	40	u	0.060226
DLX5	1	distal-less homeobox 5	213707_s_at	1	2.131	0.12	7	2.683	1.26	40	u	0.011224
PRR7	1	proline rich 7 (synaptic)	219742_at	1	3.905	0.49	7	4.693	0.88	40	u	0.028781
KIAA1826	1	KIAA1826	227418_at	1	7.432	0.37	7	7.012	1.18	40	d	0.094655
			223799_at	0	2.655	0.31	7	2.855	0.67	40	u	0.451109
RANBP17	1	RAN binding protein 17	219661_at	1	5.199	0.43	7	5.919	0.99	40	u	0.070374
GBAS	1	glioblastoma amplified sequence	201816_s_at	1	9.543	0.36	7	9.655	0.75	40	u	0.704521
FGD1	1	FYVE, RhoGEF and PH domain containing 1 (faciogenital dysplasia)	204819_at	1	6.341	0.25	7	6.724	0.79	40	u	0.025866
MRPS27	1	mitochondrial ribosomal protein S27	212145_at	1	8.631	0.2	7	7.884	0.64	40	d	0.000004
			1563970_at	0	2.414	0.15	7	2.64	0.42	40	u	0.019236
C10orf78	1	chromosome 10 open reading frame 78	236027_at	1	7.43	0.39	7	7.536	0.53	40	u	0.622254
			238794_at	0	3.995	0.61	7	3.874	0.79	40	d	0.708605
TCEAL5	1	transcription elongation factor A (SII)-like 5	no probeset available									
BCAS1	1	breast carcinoma amplified sequence 1	204378_at	1	6.106	0.57	7	6.354	0.94	40	u	0.512546
			234780_at	0	3.029	0.14	7	3.431	0.94	40	u	0.016645
			234221_at	0	2.389	0.04	7	2.454	0.07	40	u	0.031795
GPR146	1	G protein-coupled receptor 146	228770_at	1	4.848	0.92	7	3.654	0.37	40	d	0.019679
SLC25A33	1	solute carrier family 25, member 33	223296_at	1	7.582	0.63	7	7.659	0.95	40	u	0.840742
CHCHD8	1	coiled-coil-helix-coiled-coil-helix domain containing 8	220647_s_at	1	8.362	0.15	7	8.559	0.49	40	u	0.052685
PAPSS1	1	3'-phosphoadenosine 5'-phosphosulfate synthase 1	209043_at	1	10.84	0.21	7	10.92	1.13	40	u	0.712147
POLN	1	polymerase (DNA directed) nu	233454_at	1	4.722	0.16	7	4.599	0.26	40	d	0.247557
HOXA4	1	homeobox A4	206289_at	1	4.59	0.72	7	3.62	0.66	40	d	0.001254
SCAMP1	1	secretory carrier membrane protein 1	212425_at	1	5.227	0.65	7	5.034	0.64	40	d	0.474152
			212416_at	1	10.37	0.23	7	9.898	0.64	40	d	0.064092
			212417_at	1	7.823	0.29	7	8.441	0.71	40	u	0.030781
			1552978_a_at	0	7.283	0.43	7	7.452	1.09	40	u	0.69331
			206668_s_at	0	7.467	0.29	7	7.598	0.89	40	u	0.484093
			206667_s_at	0	6.21	0.85	7	6.569	1.41	40	u	0.526189
KIAA1913	1	KIAA1913	234994_at	1	6.125	0.53	7	6.18	1.43	40	u	0.922781
BANP	1	BTG3 associated nuclear protein	233186_s_at	1	6.824	0.3	7	6.725	0.58	40	d	0.667381
			219966_x_at	1	8.678	0.25	7	8.44	0.61	40	d	0.325164
			228928_x_at	1	8.177	0.29	7	7.831	0.61	40	d	0.158641
PRKACG	1	protein kinase, cAMP-dependent, catalytic, gamma	207228_at	1	4.037	0.23	7	3.892	0.38	40	d	0.348833
SLC36A2	1	rier family 36 (proton/amino acid symporter), r	no probeset available									
ZNF569	1	zinc finger protein 569	1553696_s_at	1	4.966	0.53	7	5.386	0.79	40	u	0.190863
			1569366_a_at	0	3.293	0.37	7	3.936	1.26	40	u	0.015838
FGFR1OP	1	FGFR1 oncogene partner	205587_at	1	1.784	0.03	7	1.825	0.04	40	u	0.020616
			1568678_s_at	1	6.595	0.3	7	6.95	0.78	40	u	0.251955
			205588_s_at	1	6.478	0.38	7	6.847	0.68	40	u	0.180704
MED28	1	mediator complex subunit 28	222636_at	1	6.429	0.39	7	6.384	0.81	40	d	0.88945
			218438_s_at	1	7.964	0.24	7	7.82	0.9	40	d	0.418643
			222635_s_at	1	8.136	0.26	7	8.037	0.59	40	d	0.671898
			224416_s_at	1	6.125	0.44	7	6.192	0.94	40	u	0.85575
			228992_at	0	9.127	0.2	7	8.81	0.66	40	d	0.025219

			214831_at	0	5.279	0.47	7	5.779	0.84	40	u	0.141155
			243668_at	0	2.39	0.09	7	2.447	0.12	40	u	0.240289
PTPRA	1	protein tyrosine phosphatase, receptor type, A	213799_s_at	1	6.189	0.44	7	6.966	0.66	40	u	0.00514
			213795_s_at	1	7.581	0.28	7	7.453	0.73	40	d	0.657594
			239419_at	0	2.704	0.3	7	2.676	0.26	40	d	0.801422
			233776_at	0	3.857	0.27	7	3.748	0.33	40	d	0.423014
TSC22D4	1	TSC22 domain family, member 4	208104_s_at	1	7.395	0.16	7	7.117	0.5	40	d	0.01212
			1554501_at	0	6.094	0.38	7	6.052	0.74	40	d	0.885454
CHRM2	1	cholinergic receptor, muscarinic 2	221330_at	0	1.754	0.03	7	1.794	0.05	40	u	0.034806
DKK1	1	dickkopf homolog 1 (Xenopus laevis)	204602_at	1	4.103	1.22	7	5.777	2.93	40	u	0.152385
CCNH	1	cyclin H	204093_at	1	8.299	0.48	7	7.614	0.74	40	d	0.025634
RASL10A	1	RAS-like, family 10, member A	206850_at	1	5.166	1.02	7	3.557	1.01	40	d	0.000433
PA2G4	1	proliferation-associated 2G4, 38kDa	208676_s_at	1	8.918	0.16	7	9.442	0.52	40	u	0.00003
			214794_at	1	7.666	0.25	7	7.757	0.86	40	u	0.602374
			216422_at	0	4.712	0.35	7	5.093	0.73	40	u	0.191423
NEDD8	1	neural precursor cell expressed, developmentally down-regulated 8	201840_at	1	10.62	0.14	7	10.42	0.56	40	d	0.060079
GPC2	1	glypican 2	239422_at	1	5.821	0.44	7	6.089	0.72	40	u	0.356043
PDE11A	1	phosphodiesterase 11A	224224_s_at	1	2.503	0.11	7	2.563	0.13	40	u	0.272383
			237248_at	0	2.774	0.44	7	2.718	0.47	40	d	0.775063
			224223_s_at	0	3.909	0.3	7	3.882	0.24	40	d	0.798992
			221110_x_at	0	2.805	0.39	7	2.924	0.45	40	u	0.524918
THUMPD1	1	THUMP domain containing 1	213025_at	1	8.968	0.21	7	8.373	0.7	40	d	0.000234
			206555_s_at	0	9.59	0.14	7	9.199	0.61	40	d	0.001418
PPAPDC2	1	phosphatidic acid phosphatase type 2 domain containing 2	227385_at	1	7.748	0.37	7	6.99	0.81	40	d	0.021275
MAT1A	1	methionine adenosyltransferase I, alpha	205813_s_at	1	3.035	0.39	7	3.461	0.83	40	u	0.200463
OPRM1	1	opioid receptor, mu 1	211359_s_at	0	2.131	0.07	7	2.228	0.17	40	u	0.143914
			207994_s_at	0	2.524	0.26	7	2.648	0.21	40	u	0.170852
FAM84A	1	family with sequence similarity 84, member A	225667_s_at	1	5.967	0.35	7	6.207	1.9	40	u	0.477816
			234335_s_at	0	2.946	0.22	7	3.349	0.59	40	u	0.004897
			228459_at	0	2.293	0.05	7	2.463	0.39	40	u	0.013755
			234331_s_at	0	5.587	0.65	7	5.505	1.58	40	d	0.894312
			228319_at	0	5.644	0.31	7	5.412	0.34	40	d	0.109009
SDHC	1	succinate dehydrogenase complex, subunit C, integral membrane protein, 15kDa	215088_s_at	1	9.531	0.22	7	10.28	0.54	40	u	0.000993
			202004_x_at	1	9.842	0.35	7	10.4	0.64	40	u	0.032383
			210131_x_at	1	9.958	0.27	7	10.48	0.71	40	u	0.065717
			216591_s_at	0	8.565	0.33	7	8.905	1.36	40	u	0.189603
			238056_at	0	5.299	0.71	7	6.6	0.86	40	u	0.000604
FMO2	1	flavin containing monooxygenase 2 (non-functional)	228268_at	0	9.871	0.75	7	5.612	1.95	40	d	0.000001
			211726_s_at	0	11.66	0.77	7	7.475	1.91	40	d	0.000001
GBF1	1	golgi-specific brefeldin A resistance factor 1	201439_at	1	7.085	0.51	7	6.582	0.84	40	d	0.139997
IL16	1	interleukin 16 (lymphocyte chemoattractant factor)	209828_s_at	1	4.772	0.17	7	4.86	0.34	40	u	0.512364
			209827_s_at	1	6.566	0.29	7	6.47	0.82	40	d	0.593991
			1555016_at	0	3.214	0.56	7	3.317	0.5	40	u	0.628898
CCDC88C	1	coiled-coil domain containing 88C	227228_s_at	1	7.481	0.53	7	7.145	1.14	40	d	0.456474
			243869_at	0	5.174	0.45	7	5.129	0.59	40	d	0.85137
			231288_at	0	3.914	0.38	7	4.013	0.72	40	u	0.731736
			215343_at	0	2.902	0.44	7	3.284	0.69	40	u	0.171883
SERPINE1	1	serpin peptidase inhibitor, clade E (nexin, plasminogen activator inhibitor type 1), member 1	202627_s_at	1	5.948	0.92	7	6.596	1.04	40	u	0.136602
			202628_s_at	1	6.556	0.93	7	6.586	1.35	40	u	0.955648
			1568765_at	0	4.737	0.46	7	5.042	0.72	40	u	0.293295
POU2F3	1	POU class 2 homeobox 3	207109_at	1	3.628	0.56	7	3.868	0.88	40	u	0.498012
			215355_at	0	2.641	0.28	7	2.627	0.47	40	d	0.9438
RNF40	1	ring finger protein 40	206845_s_at	1	7.513	0.27	7	7.404	0.64	40	d	0.66742
			217642_at	0	2.609	0.33	7	2.499	0.2	40	d	0.456576
EWSR1	1	Ewing sarcoma breakpoint region 1	209214_s_at	1	10.49	0.28	7	10.43	0.5	40	d	0.746795
			210011_s_at	0	8.27	0.12	7	7.936	0.5	40	d	0.000974
			210012_s_at	0	4.426	0.61	7	4.443	0.61	40	u	0.945555
			229966_at	0	6.937	0.54	7	7.222	0.86	40	u	0.412185
TMEM151	1	transmembrane protein 151	235614_at	1	2.552	0.24	7	2.443	0.48	40	d	0.564309
PLEKHH3	1	pleckstrin homology domain containing, family H (with MyTH4 domain) member 3	227248_at	1	6.52	0.28	7	5.662	0.68	40	d	0.002285
			220259_at	1	2.367	0.05	7	2.342	0.12	40	d	0.59596

CHRD	1	chordin	211248_s_at	1	6.296	0.38	7	5.882	1.28	40	d	0.119453
			221674_s_at	0	2.256	0.1	7	2.4	0.37	40	u	0.053122
KLC1	1	kinesin light chain 1	212878_s_at	1	8.789	0.29	7	8.205	0.69	40	d	0.036301
			212877_at	1	6.12	0.36	7	6.362	1.03	40	u	0.284448
			232036_at	0	2.86	0.08	7	3.025	0.28	40	u	0.005093
			213656_s_at	0	9.983	0.33	7	8.803	0.5	40	d	0.000001
C13orf1	1	chromosome 13 open reading frame 1	214668_at	1	3.047	0.46	7	3.125	0.6	40	u	0.74985
			230151_at	1	5.418	0.35	7	5.118	0.78	40	d	0.333021
OAF	1	OAF homolog (Drosophila)	228939_at	1	3.094	0.59	7	3.28	0.54	40	u	0.419956
			225510_at	1	8.987	0.58	7	7.69	0.47	40	d	0
			1553569_at	0	14.69	0.23	7	14.8	0.32	40	u	0.416198
			1553570_x_at	0	14.76	0.22	7	14.88	0.29	40	u	0.287939
REEP4	1	receptor accessory protein 4	218777_at	1	5.14	0.16	7	5.487	0.68	40	u	0.009504
HTRA2	1	HtrA serine peptidase 2	211152_s_at	4	7.067	0.29	7	7.048	0.5	40	d	0.923368
			203089_s_at	4	8.526	0.26	7	8.489	0.63	40	d	0.880775
CDK5	1	cyclin-dependent kinase 5	204247_s_at	0	6.55	0.44	7	7.332	0.87	40	u	0.027355
LY6E	1	lymphocyte antigen 6 complex, locus E	202145_at	1	9.76	0.67	7	9.929	1.39	40	u	0.757822
GCG	1	glucagon	206422_at	1	2.289	0.04	7	2.348	0.17	40	u	0.066245
CORO7	1	coronin 7	219040_at	1	6.324	0.29	7	6.314	0.53	40	d	0.961524
NOLC1	1	nucleolar and coiled-body phosphoprotein 1	211949_s_at	1	5.956	0.22	7	6.883	0.84	40	u	0.000001
			211951_at	1	9.558	0.21	7	9.684	0.63	40	u	0.352529
			205895_s_at	1	6.893	0.34	7	7.257	1.06	40	u	0.10697
RHOBTB3	1	Rho-related BTB domain containing 3	202975_s_at	1	8.236	0.44	7	8.403	1.57	40	u	0.591558
			202976_s_at	1	8.5	0.54	7	8.216	1.69	40	d	0.420187
			216049_at	0	2.87	0.16	7	3.194	0.74	40	u	0.021614
			240111_at	0	5.018	0.35	7	5.245	0.94	40	u	0.538673
			225202_at	0	9.132	0.44	7	8.768	1.52	40	d	0.237075
			216048_s_at	0	5.303	0.67	7	6.067	1.91	40	u	0.075184
P2RX4	1	purinergic receptor P2X, ligand-gated ion channel, 4	204088_at	0	8.284	0.23	7	7.67	1.07	40	d	0.00301
COX11	1	COX11 homolog, cytochrome c oxidase assembly protein (yeast)	203551_s_at	3	7.017	0.32	7	6.927	0.74	40	d	0.755169
			211727_s_at	0	8.719	0.32	7	8.992	1.12	40	u	0.227704
			214277_at	0	2.953	0.12	7	3.627	0.95	40	u	0.000121
FSTL3	1	follistatin-like 3 (secreted glycoprotein)	203592_s_at	1	5.605	0.49	7	5.302	0.76	40	d	0.325475
QSOX1	1	quiescin Q6 sulfhydryl oxidase 1	201482_at	1	8.326	0.55	7	7.646	1.07	40	d	0.115697
PCP4	1	Purkinje cell protein 4	205549_at	1	3.293	0.4	7	3.744	1.81	40	u	0.184078
NPY2R	1	neuropeptide Y receptor Y2	210729_at	1	4.27	2.1	7	2.746	0.22	40	d	0.12569
			208379_x_at	0	2.044	0.02	7	2.109	0.12	40	u	0.002475
			210730_s_at	0	2.912	1.38	7	2.302	0.37	40	d	0.321986
PLD3	1	phospholipase D family, member 3	201050_at	1	8.687	0.27	7	8.035	0.81	40	d	0.000779
SYT17	1	synaptotagmin XVII	205613_at	0	6.632	0.86	7	5.865	1.81	40	d	0.288452
			229053_at	0	5.045	0.83	7	4.781	1.75	40	d	0.70387
EPHB6	1	EPH receptor B6	204718_at	1	6.143	0.22	7	5.329	0.66	40	d	0.000004
POFUT1	1	protein O-fucosyltransferase 1	212349_at	1	7.401	0.3	7	7.149	0.5	40	d	0.213067
			210433_at	0	2.652	0.21	7	2.77	0.25	40	u	0.247414
PDLIM3	1	PDZ and LIM domain 3	209621_s_at	1	9.507	0.66	7	6.931	1.86	40	d	0.000001
			210170_at	0	7.836	0.56	7	4.768	0.96	40	d	0
			238592_at	0	5.415	1.36	7	3.363	0.8	40	d	0.011301
NT5C3L	1	5'-nucleotidase, cytosolic III-like	225044_at	1	9.427	0.32	7	9.302	0.73	40	d	0.664911
VPS24	1	vacuolar protein sorting 24 homolog (S. cerevisiae)	217837_s_at	1	9.961	0.26	7	9.771	0.67	40	d	0.472901
			222437_s_at	1	10.87	0.27	7	10.3	0.66	40	d	0.032253
			222436_s_at	1	9.767	0.33	7	9.518	0.68	40	d	0.355756
NAPRT1	1	nicotinate phosphoribosyltransferase domain containing 1	226707_at	1	7.724	0.22	7	7.571	1.14	40	d	0.458089
			226708_at	1	2.234	0.05	7	2.283	0.06	40	u	0.039659
CDCP1	1	CUB domain containing protein 1	218451_at	1	5.671	0.5	7	6.133	0.94	40	u	0.221737
			234932_s_at	1	4.287	0.51	7	4.92	1.02	40	u	0.123657
			1554110_at	0	3.726	0.22	7	4.254	0.63	40	u	0.000622
ARF3	1	ADP-ribosylation factor 3	200734_s_at	1	9.117	0.3	7	9.558	0.71	40	u	0.11806
			200011_s_at	1	10.45	0.2	7	10.23	0.71	40	d	0.12719
			211622_s_at	0	7.534	0.32	7	7.925	0.94	40	u	0.059743
PEX19	1	peroxisomal biogenesis factor 19	201707_at	1	7.403	0.45	7	6.94	0.77	40	d	0.135869
			201706_s_at	1	8.079	0.23	7	7.981	0.65	40	d	0.496169
CITED4	1	Cbp/p300-interacting transactivator, with Glu/Asp-rich carboxy-terminal domain, 4	228625_at	1	9.064	0.63	7	8.317	1.12	40	d	0.099656

CNP	1	2',3'-cyclic nucleotide 3' phosphodiesterase	1557943_at	1	3.729	0.66	7	3.569	0.55	40	d	0.506981
			208912_s_at	0	8.976	0.32	7	8.907	0.65	40	d	0.787289
PPP1R3C	1	protein phosphatase 1, regulatory (inhibitor) subunit 3C	204284_at	1	7.991	0.77	7	6.023	2.37	40	d	0.000442
			240187_at	0	2.12	0.04	7	2.335	0.68	40	u	0.06031
CD164L2	1	CD164 sialomucin-like 2	no probeset available									
HIVEP3	1	human immunodeficiency virus type I enhancer binding protein 3	232581_x_at	1	4.309	0.75	7	4.198	0.86	40	d	0.754482
			220042_x_at	1	5.681	0.67	7	5.609	0.62	40	d	0.785359
PDE8B	1	phosphodiesterase 8B	213228_at	1	5.918	0.68	7	5.271	1.34	40	d	0.22824
			235115_at	0	2.002	0.02	7	2.067	0.1	40	u	0.000966
			1554789_a_at	0	4.033	0.74	7	3.606	1.24	40	d	0.391852
SF3B4	1	splicing factor 3b, subunit 4, 49kDa	209044_x_at	1	8.78	0.35	7	9.005	0.8	40	u	0.477452
SEMA5A	1	sema domain, seven thrombospondin repeats (type 1 and type 1-like), transmembrane domain (TM) and short	205405_at	1	7.778	0.63	7	4.613	0.57	40	d	0
			229427_at	0	5.383	0.38	7	3.028	0.62	40	d	0
			213169_at	0	8.294	0.59	7	4.541	1.14	40	d	0
C16orf77	1	chromosome 16 open reading frame 77	237046_x_at	1	6.373	0.34	7	6.111	0.63	40	d	0.29703
CABLES1	1	Cdk5 and Abl enzyme substrate 1	225531_at	1	6.555	0.26	7	6.271	0.73	40	d	0.083361
			225532_at	1	7.364	0.31	7	6.902	1.01	40	d	0.03299
			1558621_at	0	3.38	0.55	7	3.377	0.87	40	d	0.992161
C13orf15	1	chromosome 13 open reading frame 15	218723_s_at	1	9.679	0.96	7	8.141	1.33	40	d	0.006403
			228193_s_at	1	3.191	0.45	7	2.95	0.55	40	d	0.286818
			239827_at	0	2.423	0.03	7	2.492	0.06	40	u	0.003529
ALKBH8	1	alkB, alkylation repair homolog 8 (E. coli)	235713_at	0	5.639	0.19	7	5.956	0.7	40	u	0.025567
			235610_at	0	3.783	0.3	7	4.004	0.62	40	u	0.372616
SUPT6H	1	suppressor of Ty 6 homolog (S. cerevisiae)	208830_s_at	1	7.979	0.22	7	7.355	0.66	40	d	0.00015
			208420_x_at	1	8.625	0.29	7	7.963	0.71	40	d	0.020784
			208831_x_at	1	8.311	0.23	7	7.588	0.79	40	d	0.000059
			1554311_a_at	0	2.862	0.24	7	3.183	0.83	40	u	0.061352
PI16	1	peptidase inhibitor 16	228312_at	1	7.406	0.95	7	6.448	0.32	40	d	0.050178
C9orf19	1	chromosome 9 open reading frame 19	225604_s_at	1	7.669	0.79	7	7.579	1.54	40	d	0.88315
			225602_at	1	8.135	0.68	7	8.036	1.34	40	d	0.851336
WBSCR18	1	Williams Beuren syndrome chromosome region 18	223367_at	1	7.527	0.23	7	7.286	0.69	40	d	0.107781
			239194_at	1	3.255	0.5	7	3.495	0.44	40	u	0.206072
			239018_at	1	4.236	0.76	7	4.279	0.84	40	u	0.902813
BECN1	1	beclin 1 (coiled-coil, myosin-like BCL2 interacting protein)	208945_s_at	1	8.83	0.26	7	8.694	0.49	40	d	0.482551
			208946_s_at	1	9.475	0.22	7	8.996	0.71	40	d	0.002374
			1561652_at	0	3.765	0.19	7	3.82	0.42	40	u	0.743491
MKL1	1	megakaryoblastic leukemia (translocation) 1	1555763_x_at	1	4.345	0.46	7	4.569	0.47	40	u	0.263195
			212748_at	1	6.897	0.39	7	5.929	0.64	40	d	0.000402
			1559902_at	0	3.176	0.36	7	3.259	0.37	40	u	0.593599
			215291_at	0	4.867	0.14	7	4.749	0.34	40	d	0.384311
			215292_s_at	0	3.892	0.26	7	4.062	0.53	40	u	0.422338
GPT2	1	glutamic pyruvate transaminase (alanine aminotransferase) 2	224839_s_at	1	5.67	0.49	7	6.742	1.24	40	u	0.032855
C7orf28B	1	chromosome 7 open reading frame 28B	no probeset available									
C6orf47	1	chromosome 6 open reading frame 47	204968_at	0	6.202	0.21	7	6.14	0.3	40	d	0.605851
TRIT1	1	tRNA isopentenyltransferase 1	218617_at	1	7.935	0.39	7	8.042	0.52	40	u	0.617825
B3GALNT2	1	beta-1,3-N-acetylgalactosaminyltransferase 2	226233_at	0	8.441	0.13	7	9.214	0.57	40	u	0
			1562391_at	0	4.38	0.33	7	5.091	0.75	40	u	0.019365
			1553691_at	0	3.344	0.3	7	4.334	1.01	40	u	0.000029
			1561714_a_at	0	2.582	0.17	7	3.068	0.7	40	u	0.000649
CLNS1A	1	chloride channel, nucleotide-sensitive, 1A	209143_s_at	0	9.49	0.24	7	9.958	0.91	40	u	0.01226
BCAT1	1	branched chain aminotransferase 1, cytosolic	225285_at	1	5.986	0.84	7	7.267	1.3	40	u	0.017454
			226517_at	1	5.499	1.2	7	7.268	1.47	40	u	0.0051
			214452_at	0	3.141	0.16	7	3.939	1.22	40	u	0.000366
			214390_s_at	0	2.522	0.38	7	3.373	1.26	40	u	0.002323
MUTED	1	muted homolog (mouse)	226543_at	1	3.566	0.34	7	4.535	0.93	40	u	0.010685
			226544_x_at	1	8.164	0.29	7	8.16	0.43	40	d	0.982034
CHAC2	1	ChaC, cation transport regulator homolog 2 (E. coli)	235117_at	1	3.403	0.21	7	5.2	1.2	40	u	0
EMILIN3	1	elastin microfibril interfacer 3	228307_at	1	5.188	0.35	7	5.067	0.72	40	d	0.669296
ZNF398	1	zinc finger protein 398	228145_s_at	1	7.624	0.33	7	7.032	0.95	40	d	0.0078
			226202_at	1	6.333	0.51	7	6.067	0.84	40	d	0.430769
			1555015_a_at	0	5.055	0.51	7	5.421	0.61	40	u	0.149122
		REX2, RNA exonuclease 2 homolog (S	218194_at	1	10.75	0.27	7	10.22	0.79	40	d	0.00393

REXO2	1	REX2, RNA exonuclease 2 homolog (S. cerevisiae)	223989_s_at	0	7.314	0.55	7	6.891	1.01	40	d	0.297647
			239616_at	0	1.92	0.02	7	1.956	0.04	40	u	0.033325
ANXA4	1	annexin A4	201302_at	1	10.15	0.39	7	9.736	0.76	40	d	0.176224
			201301_s_at	0	9.909	0.4	7	9.724	0.89	40	d	0.600823
ATOH7	1	atonal homolog 7 (Drosophila)	1552879_a_at	1	2.594	0.04	7	2.728	0.26	40	u	0.00405
			1552878_at	1	2.367	0.07	7	2.468	0.12	40	u	0.046357
NPL	1	N-acetylneuraminate pyruvate lyase (dihydrodipicolinate synthase)	221210_s_at	1	5.123	0.38	7	6.187	1.28	40	u	0.000277
			223405_at	1	5.372	0.39	7	6.403	0.79	40	u	0.001774
			240440_at	0	3.412	0.44	7	3.749	0.78	40	u	0.284828
			243066_at	0	2.955	0.1	7	3.131	0.2	40	u	0.034434
FAM110B	1	family with sequence similarity 110, member B	228790_at	1	5.937	0.62	7	5.41	1.94	40	d	0.198794
			227800_at	1	2.766	0.12	7	2.758	0.31	40	d	0.945349
			221959_at	1	5.52	0.72	7	5.276	1.4	40	d	0.662208
C15orf37	1	chromosome 15 open reading frame 37	1556588_at	1	3.203	0.5	7	3.809	0.82	40	u	0.068989
GABARAPL2	1	GABA(A) receptor-associated protein-like 2	209046_s_at	1	11.52	0.2	7	10.93	0.69	40	d	0.000152
KCNN4	1	potassium intermediate/small conductance calcium-activated channel, subfamily N, member 4	204401_at	0	8.761	0.52	7	7.546	1.73	40	d	0.00158
FLNA	1	filamin A, alpha (actin binding protein 280)	214752_x_at	1	10.49	0.51	7	9.382	0.89	40	d	0.00308
			213746_s_at	1	10.1	0.48	7	9.113	0.88	40	d	0.007339
			200859_x_at	0	11.21	0.43	7	9.975	0.87	40	d	0.000789
LHX9	1	LIM homeobox 9	1565407_at	0	2.147	0.12	7	2.171	0.3	40	u	0.835355
			1560253_at	0	2.292	0.02	7	2.356	0.06	40	u	0.012525
			1562736_at	0	2.441	0.05	7	2.554	0.12	40	u	0.022202
			1565406_a_at	0	1.758	0.07	7	1.814	0.1	40	u	0.167621
FUNDC1	1	FUN14 domain containing 1	235346_at	1	8.367	0.52	7	8.369	0.78	40	u	0.993655
C1orf130	1	chromosome 1 open reading frame 130	no probeset available									
TRPC3	1	transient receptor potential cation channel, subfamily C, member 3	206425_s_at	1	2.651	0.07	7	3.096	0.69	40	u	0.000377
			210814_at	1	3.106	0.15	7	3.577	0.69	40	u	0.000544
MARCH1	1	membrane-associated ring finger (C3HC4) 1	219574_at	1	3.369	0.32	7	4.537	1.01	40	u	0.000005
			1562338_at	0	2.704	0.11	7	2.767	0.13	40	u	0.239622
TRIM8	1	tripartite motif-containing 8	223132_s_at	1	9.556	0.3	7	9.027	0.73	40	d	0.072406
			223131_s_at	1	5.684	0.56	7	5.369	1.07	40	d	0.462328
			221012_s_at	1	9.029	0.2	7	7.928	0.85	40	d	0
			228015_s_at	0	2.228	0.13	7	2.249	0.14	40	u	0.735232
SEMA3C	1	sema domain, immunoglobulin domain (Ig), short basic domain, secreted, (semaphorin)	203789_s_at	1	11.37	0.36	7	8.78	1.8	40	d	0
			203788_s_at	1	6.868	0.74	7	5.655	1.22	40	d	0.01596
VAV1	1	vav 1 guanine nucleotide exchange factor	206219_s_at	1	3.71	0.46	7	3.906	0.82	40	u	0.550545
ZNF192	1	zinc finger protein 192	206579_at	1	1.976	0.02	7	2.245	0.47	40	u	0.000986
SEC22A	1	SEC22 vesicle trafficking protein homolog A (S. cerevisiae)	218703_at	1	5.718	0.21	7	5.993	0.56	40	u	0.215622
ATP5G1	1	ATP synthase, H+ transporting, mitochondrial F0 complex, subunit C1 (subunit 9)	208972_s_at	1	8.429	0.21	7	8.531	0.83	40	u	0.52459
PNPLA2	1	patatin-like phospholipase domain containing 2	212705_x_at	1	9.163	0.3	7	7.694	0.7	40	d	0.000003
			39854_r_at	1	9.556	0.18	7	9.015	0.51	40	d	0.000037
			213862_at	0	2.307	0.04	7	2.378	0.09	40	u	0.0514
VPS13B	1	vacuolar protein sorting 13 homolog B (yeast)	220400_at	0	2.619	0.11	7	3.069	0.67	40	u	0.000347
			213243_at	0	8.292	0.16	7	7.933	1.16	40	d	0.075748
			236254_at	0	8.288	0.4	7	9.187	0.88	40	u	0.012947
			1553852_at	0	3.362	0.28	7	4.992	1.55	40	u	0
HCLS1	1	hematopoietic cell-specific Lyn substrate 1	202957_at	1	8.234	0.68	7	8.143	1.22	40	d	0.851842
RXFP2	1	relaxin/insulin-like family peptide receptor 2	1553326_at	1	2.145	0.02	7	2.196	0.05	40	u	0.019169
EXOC6	1	exocyst complex component 6	226259_at	1	7.076	0.5	7	6.89	0.91	40	d	0.609359
			233924_s_at	0	5.94	0.59	7	5.736	1.37	40	d	0.705328
			232599_at	0	3.107	0.58	7	2.959	0.56	40	d	0.536415
CTSC	1	cathepsin C	225646_at	1	7.124	0.37	7	7.836	0.99	40	u	0.072703
			225647_s_at	1	7.501	1.22	7	8.193	1.67	40	u	0.310776
			231234_at	0	4.248	0.56	7	4.64	0.8	40	u	0.230116
			201487_at	0	9.298	0.68	7	9.698	1.08	40	u	0.356674
			237701_at	1	2.479	0.19	7	2.508	0.13	40	u	0.622495

C12orf54	1	chromosome 12 open reading frame 54	240353_s_at	1	4.529	0.65	7	4.452	0.55	40	d	0.747893
			240354_at	1	2.612	0.37	7	2.756	0.49	40	u	0.476379
Probesets not assigned to genes			238476_at	30	6.772	0.54	7	5.778	0.81	40	d	0.0036
			225956_at	30	8.513	0.22	7	7.284	0.91	40	d	0
			225957_at	30	7.509	0.54	7	6.563	0.81	40	d	0.005794
			222494_at	24	9.124	0.3	7	7.985	0.6	40	d	0.000019
			218031_s_at	19	8.287	0.42	7	7.151	0.83	40	d	0.001184
			238400_at	14	2.033	0.01	7	2.088	0.14	40	u	0.017581
			238402_s_at	14	5.626	0.22	7	5.371	0.48	40	d	0.184687
			203629_s_at	14	7.399	0.28	7	8.048	0.61	40	u	0.009463
			222630_at	14	8.134	0.29	7	7.739	0.7	40	d	0.157219
			223925_s_at	14	3.262	0.14	7	3.68	0.63	40	u	0.000799
			238401_at	14	4.476	0.36	7	4.51	0.7	40	u	0.904192
			209469_at	14	3.026	0.37	7	3.466	1.8	40	u	0.182267
			225512_at	13	8.789	0.31	7	8.057	0.84	40	d	0.031011
			202629_at	13	8.516	0.24	7	8.437	0.86	40	d	0.642811
			202630_at	13	8.46	0.22	7	7.67	0.82	40	d	0.000017
			213496_at	13	2.9	0.29	7	3.079	0.72	40	u	0.528571
			200004_at	12	12.36	0.2	7	12.33	0.35	40	d	0.825178
			218692_at	12	8.648	0.32	7	6.446	1.8	40	d	0
			215081_at	12	2.878	0.32	7	3.454	0.48	40	u	0.004359
			211240_x_at	11	10.06	0.36	7	9.302	0.79	40	d	0.018592
			215228_at	11	2.473	0.04	7	2.673	0.52	40	u	0.02222
			222709_at	11	5.13	0.43	7	4.872	0.43	40	d	0.159772
			1565644_at	11	3.494	0.2	7	3.354	0.18	40	d	0.07353
			208862_s_at	11	10.54	0.35	7	9.801	0.68	40	d	0.009066
			208407_s_at	11	9.959	0.38	7	8.473	0.85	40	d	0.000057
			221483_s_at	10	10.34	0.17	7	9.817	0.64	40	d	0.00016
			204447_at	10	6.485	0.42	7	6.797	0.92	40	u	0.394454
			212289_at	10	7.674	0.28	7	6.931	0.82	40	d	0.000248
			226058_at	10	5.884	0.18	7	5.483	0.78	40	d	0.008716
			228248_at	10	9.103	0.2	7	8.664	0.69	40	d	0.003134
			212286_at	10	7.364	0.59	7	7.3	1.05	40	d	0.877804
			226820_at	10	9.004	0.46	7	8.305	0.69	40	d	0.015178
			210148_at	9	5.061	1.03	7	5.402	1.74	40	u	0.625888
			225658_at	9	9.286	0.53	7	8.541	1.01	40	d	0.06895
			244435_at	9	3.201	0.23	7	3.637	0.9	40	u	0.015474
			202033_s_at	9	9.175	0.39	7	9.703	0.97	40	u	0.172517
			202034_x_at	9	8.121	0.2	7	8.55	0.78	40	u	0.007135
			218091_at	9	8.131	0.51	7	8.555	0.56	40	u	0.071258
			224732_at	9	9.087	0.22	7	8.77	0.55	40	d	0.145856
			225659_at	9	7.209	0.66	7	7.232	0.76	40	u	0.942161
			212814_at	9	5.59	0.46	7	5.828	0.74	40	u	0.425241
			200970_s_at	9	10.85	0.43	7	10.66	0.68	40	d	0.483567
			200969_at	9	10.89	0.36	7	10.59	0.58	40	d	0.202764
			1555630_a_at	8	9.534	0.29	7	8.809	1.2	40	d	0.002746
			224710_at	8	9.587	0.27	7	8.905	1.15	40	d	0.003
			244030_at	8	4.923	0.64	7	4.967	1.03	40	u	0.916501
			239725_at	8	5.325	0.79	7	4.531	0.78	40	d	0.019243
			211341_at	8	2.648	0.13	7	3.187	0.95	40	u	0.001579
			239650_at	8	3.322	0.27	7	3.595	0.66	40	u	0.295687
			219126_at	8	8.734	0.23	7	8.032	0.91	40	d	0.000246
			241984_at	8	3.347	0.58	7	3.473	0.65	40	u	0.64024
			206940_s_at	8	2.894	0.05	7	3.281	0.97	40	u	0.01736
			223859_at	8	2.687	0.32	7	2.636	0.24	40	d	0.631754
			204496_at	8	6.335	0.43	7	6.744	0.74	40	u	0.171458
			203694_s_at	8	8.138	0.22	7	7.792	0.58	40	d	0.137499
			212911_at	8	6.274	0.5	7	6.284	0.76	40	u	0.972463
			231813_s_at	8	2.494	0.16	7	2.623	0.2	40	u	0.124458
			212908_at	8	7.157	0.35	7	6.679	0.73	40	d	0.101841
			222652_s_at	7	6.657	0.26	7	7.055	0.64	40	u	0.118658
			213224_s_at	7	7.851	0.24	7	7.303	0.85	40	d	0.00249
			227404_s_at	7	13	0.52	7	9.861	1.43	40	d	0
			226694_at	7	9.394	0.53	7	8.28	0.81	40	d	0.001339

209470_s_at	7	3.562	0.68	7	3.734	2.02	40	u	0.68991
224992_s_at	7	7.433	0.36	7	7.717	0.67	40	u	0.291659
201170_s_at	7	11.91	0.62	7	10.53	1.44	40	d	0.017926
201694_s_at	7	12.27	0.4	7	8.72	1.29	40	d	0
202038_at	7	10.21	0.32	7	9.492	0.62	40	d	0.005282
203804_s_at	7	10.39	0.2	7	9.894	0.82	40	d	0.002791
201693_s_at	7	11.02	0.78	7	6.813	1.67	40	d	0
208797_s_at	7	6.903	0.39	7	6.583	0.7	40	d	0.255971
219218_at	7	5.06	0.64	7	4.651	1.16	40	d	0.377968
221867_at	7	6.898	0.47	7	5.613	0.79	40	d	0.000189
208835_s_at	7	10.33	0.35	7	10.57	0.77	40	u	0.441964
228993_s_at	7	9.437	0.37	7	8.677	0.9	40	d	0.037563
208979_at	7	8.588	0.39	7	8.538	0.65	40	d	0.845783
220044_x_at	7	10.09	0.3	7	9.797	0.7	40	d	0.284986
218230_at	7	8.809	0.25	7	8.483	0.69	40	d	0.03928
213220_at	7	7.864	0.37	7	7.624	0.64	40	d	0.351667
1566833_x_at	7	2.287	0.02	7	2.352	0.14	40	u	0.010881
241801_at	7	3.949	0.27	7	3.609	0.61	40	d	0.163414
218506_x_at	7	8.168	0.25	7	8.336	0.47	40	u	0.37386
224991_at	7	6.967	0.33	7	7.468	0.9	40	u	0.163112
1566832_at	7	2.28	0.03	7	2.365	0.19	40	u	0.011738
223231_at	7	9.119	0.4	7	9.512	0.9	40	u	0.273384
206070_s_at	7	2.681	0.07	7	3.145	1.2	40	u	0.022043
201001_s_at	6	8.352	0.37	7	9.269	0.55	40	u	0.000133
227566_at	6	5.259	1.09	7	6.146	1.33	40	u	0.109902
207318_s_at	6	8.342	0.16	7	8.418	0.51	40	u	0.478918
211202_s_at	6	8.906	0.41	7	9.034	1	40	u	0.747558
201548_s_at	6	7.561	0.38	7	8.582	0.88	40	u	0.004767
217225_x_at	6	10.88	0.43	7	10.97	0.67	40	u	0.759139
236532_at	6	2.297	0.05	7	2.344	0.06	40	u	0.071902
201547_at	6	2.492	0.21	7	2.382	0.12	40	d	0.266883
224728_at	6	6.735	0.26	7	5.814	0.76	40	d	0.000008
201549_x_at	6	8.575	0.45	7	8.711	1.07	40	u	0.745867
201002_s_at	6	10.19	0.19	7	9.961	0.49	40	d	0.248566
224888_at	6	7.975	0.28	7	8.537	0.64	40	u	0.030368
227870_at	6	4.129	0.94	7	3.194	0.75	40	d	0.006634
205192_at	6	6.107	0.48	7	5.085	0.57	40	d	0.00007
214665_s_at	6	10.41	0.21	7	9.908	0.55	40	d	0.025083
201642_at	6	9.928	0.2	7	9.819	0.52	40	d	0.595584
224724_at	6	8.876	0.59	7	8.833	1.35	40	d	0.936706
221853_s_at	6	9.078	0.53	7	9.035	0.75	40	d	0.88773
212677_s_at	6	8.652	0.45	7	7.466	0.7	40	d	0.00012
214951_at	6	3.357	0.98	7	2.938	0.84	40	d	0.25344
1555274_a_at	6	5.141	0.35	7	7.553	0.75	40	u	0
226165_at	6	10.43	0.21	7	10.76	0.68	40	u	0.025875
228957_at	6	2.374	0.11	7	2.5	0.18	40	u	0.087494
201854_s_at	6	7.996	0.26	7	8.286	0.58	40	u	0.209417
227784_s_at	6	7.966	0.3	7	7.442	0.8	40	d	0.100738
1557944_s_at	6	9.199	0.38	7	8.463	1.34	40	d	0.009178
219599_at	6	6.761	0.65	7	6.329	1.12	40	d	0.338767
224729_s_at	6	8.282	0.32	7	8.145	0.6	40	d	0.567711
225450_at	5	8.974	0.51	7	7.25	1.28	40	d	0.001289
212451_at	5	7.993	0.44	7	8.279	0.91	40	u	0.429087
225665_at	5	9.618	0.37	7	8.483	0.89	40	d	0.002198
226415_at	5	3.057	0.33	7	3.17	0.66	40	u	0.667203
208398_s_at	5	7.384	0.33	7	8.024	0.92	40	u	0.003559
238462_at	5	3.077	0.14	7	3.716	0.99	40	u	0.000461
202759_s_at	5	9.341	0.39	7	8.008	0.85	40	d	0.000231
226178_at	5	7.624	0.29	7	7.674	0.51	40	u	0.804854
224899_s_at	5	10.47	0.2	7	10.2	0.91	40	d	0.117704
226608_at	5	4.675	0.51	7	6.01	0.82	40	u	0.000186
207880_at	5	2.285	0.1	7	2.393	0.22	40	u	0.226142
238047_at	5	6.508	2.62	7	2.978	1.45	40	d	0.01799
221482_s_at	5	7.68	0.39	7	8.12	0.69	40	u	0.113831

242555_at	5	2.385	0.06	7	2.827	0.59	40	u	0.000049
212906_at	5	5.278	0.29	7	5.301	0.34	40	u	0.874104
225662_at	5	8.75	0.41	7	8.11	0.76	40	d	0.038642
219732_at	5	3.901	0.68	7	2.761	0.4	40	d	0.00704
218469_at	5	6.628	1.48	7	6.854	1.54	40	u	0.726347
212450_at	5	7.976	0.28	7	7.597	0.72	40	d	0.184065
209357_at	5	9.109	0.65	7	8.812	1.15	40	d	0.519777
218765_at	5	7.587	0.27	7	6.754	0.63	40	d	0.001526
220359_s_at	5	2.066	0.02	7	2.162	0.16	40	u	0.000759
204347_at	5	7.104	0.27	7	6.948	0.81	40	d	0.366307
224842_at	5	9.535	0.29	7	9.644	0.65	40	u	0.673673
56256_at	5	9.043	0.31	7	7.777	1.05	40	d	0.000001
225459_at	5	5.202	0.53	7	4.327	1.13	40	d	0.054678
218661_at	4	6.793	0.25	7	6.625	0.68	40	d	0.528639
243430_at	4	4.043	0.38	7	4.287	0.75	40	u	0.413002
210425_x_at	4	9.637	0.35	7	7.537	1.15	40	d	0
243070_at	4	4.487	0.46	7	4.942	0.52	40	u	0.040579
208754_s_at	4	9.501	0.3	7	9.83	0.69	40	u	0.232723
225185_at	4	8.126	0.38	7	6.832	1.35	40	d	0.000029
227855_at	4	3.705	0.3	7	2.495	0.31	40	d	0
204601_at	4	5.239	0.26	7	5.921	0.67	40	u	0.01214
213188_s_at	4	7.984	0.28	7	8.019	0.93	40	u	0.854966
212842_x_at	4	9.817	0.13	7	8.853	0.62	40	d	0
213189_at	4	7.975	0.39	7	8.373	1.07	40	u	0.34516
205078_at	4	7.319	0.27	7	7.504	0.88	40	u	0.30912
1563346_at	4	1.87	0.08	7	1.923	0.1	40	u	0.202901
226573_at	4	4.743	0.51	7	5.212	1.18	40	u	0.317412
216270_at	4	2.551	0.03	7	2.597	0.13	40	u	0.05723
208752_x_at	4	12.06	0.18	7	11.71	0.51	40	d	0.004488
225945_at	4	10.08	0.17	7	8.766	1.18	40	d	0
214030_at	4	8.48	0.25	7	6.922	0.8	40	d	0
227286_at	4	8.757	0.17	7	8.269	0.74	40	d	0.001046
232672_x_at	4	2.419	0.05	7	2.554	0.19	40	u	0.000693
212544_at	4	8.673	0.35	7	8.371	0.67	40	d	0.262838
211324_s_at	4	2.945	0.13	7	3.409	0.6	40	u	0.000131
218416_s_at	4	5.719	0.45	7	4.653	0.66	40	d	0.000202
230298_at	4	6.468	0.26	7	5.515	0.81	40	d	0.000004
213102_at	4	9.632	0.34	7	10.64	0.49	40	u	0.000005
218430_s_at	4	4.59	0.37	7	5.187	0.63	40	u	0.021041
227890_at	4	5.236	0.44	7	5.328	1	40	u	0.817126
220326_s_at	4	7.266	0.42	7	5.412	1.05	40	d	0.000046
218092_s_at	4	6.961	0.4	7	8.235	0.7	40	u	0.000039
202936_s_at	4	10.33	0.49	7	9.163	1.45	40	d	0.00086
205077_s_at	4	8.368	0.29	7	8.449	0.63	40	u	0.745643
210676_x_at	4	7.896	0.31	7	7.723	0.73	40	d	0.546701
202935_s_at	4	10.25	0.49	7	9.442	1.8	40	d	0.026533
226106_at	4	9.526	0.38	7	8.71	0.84	40	d	0.017531
216995_x_at	4	2.198	0.13	7	2.308	0.16	40	u	0.109532
205956_x_at	4	5.706	0.3	7	6.086	0.49	40	u	0.057143
220990_s_at	4	10.77	0.49	7	10.73	0.92	40	d	0.904614
229044_at	4	5.703	0.43	7	5.928	0.47	40	u	0.250851
213469_at	4	5.26	0.38	7	4.866	1.07	40	d	0.099701
1554726_at	4	5.301	0.55	7	5.236	1.05	40	d	0.876483
212150_at	4	8.728	0.36	7	9.047	0.76	40	u	0.290888
242100_at	4	4.676	0.51	7	4.825	0.55	40	u	0.517576
204794_at	4	6.922	1.46	7	5.891	0.83	40	d	0.143229
1557203_at	4	3.886	0.2	7	4.143	0.32	40	u	0.05023
214825_at	4	2.369	0.04	7	2.491	0.19	40	u	0.000961
209570_s_at	4	5.499	0.39	7	3.692	0.47	40	d	0
213101_s_at	4	11.36	0.27	7	12.34	0.48	40	u	0.000006
209569_x_at	4	4.895	0.49	7	3.353	0.89	40	d	0.000076
212916_at	4	7.165	0.37	7	6.64	0.88	40	d	0.135629
45526_g_at	4	8.855	0.25	7	8.87	0.6	40	u	0.950003
212149_at	4	9.973	0.29	7	10.42	0.83	40	u	0.021053

222510_s_at	4	5.813	0.42	7	5.969	1.31	40	u	0.572848
32069_at	4	6.665	0.23	7	7.556	0.56	40	u	0.000194
1560633_a_at	4	2.709	0.07	7	2.893	0.4	40	u	0.011616
226703_at	3	5.423	0.62	7	5.01	0.59	40	d	0.106067
201855_s_at	3	7.992	0.35	7	7.735	0.69	40	d	0.350001
226321_at	3	7.949	0.48	7	7.813	0.71	40	d	0.638154
208855_s_at	3	10.77	0.31	7	10.81	0.7	40	u	0.882011
225468_at	3	6.99	0.31	7	7.251	1.16	40	u	0.255219
1553422_s_at	3	1.963	0.11	7	2.055	0.18	40	u	0.193983
218030_at	3	7.92	0.24	7	8.055	0.46	40	u	0.45834
37996_s_at	3	6.765	0.6	7	6.519	1.11	40	d	0.57935
209563_x_at	3	11.29	0.09	7	11.48	0.49	40	u	0.027328
218389_s_at	3	9.889	0.37	7	10.21	0.9	40	u	0.366741
209524_at	3	8.625	0.35	7	8.928	1.2	40	u	0.212164
210057_at	3	4.663	0.33	7	5.689	0.72	40	u	0.000716
204290_s_at	3	8.877	0.45	7	7.507	1.12	40	d	0.00313
208922_s_at	3	9.868	0.2	7	8.741	0.54	40	d	0.000003
200971_s_at	3	11.71	0.29	7	11.82	0.58	40	u	0.628982
228523_at	3	5.208	1.07	7	5.19	1.59	40	d	0.977915
1555408_at	3	1.793	0.18	7	2.748	1.16	40	u	0.000022
1554619_at	3	3.148	0.33	7	3.422	0.36	40	u	0.07518
221553_at	3	8.192	0.41	7	8.571	0.77	40	u	0.223048
208330_at	3	2.03	0.07	7	2.099	0.11	40	u	0.129685
226727_at	3	7.943	0.25	7	8.03	0.99	40	u	0.646935
212580_at	3	2.957	0.73	7	2.475	0.39	40	d	0.164596
213951_s_at	3	5.806	0.32	7	6.691	0.7	40	u	0.002383
209525_at	3	4.594	0.48	7	5.113	0.79	40	u	0.108367
218035_s_at	3	10.54	0.43	7	9.981	1.19	40	d	0.043678
222485_at	3	5.015	0.3	7	4.985	0.28	40	d	0.800899
2028_s_at	3	3.555	0.16	7	4.23	0.6	40	u	0.000002
226590_at	3	6.796	0.4	7	6.456	0.69	40	d	0.22378
225325_at	3	8.708	0.3	7	8.548	0.78	40	d	0.602744
241383_at	3	4.06	0.13	7	4.148	1.11	40	u	0.635247
213688_at	3	6.536	0.23	7	7.082	0.63	40	u	0.032044
203413_at	3	5.782	1.48	7	6.642	2.05	40	u	0.30389
222154_s_at	3	9.49	0.24	7	9.663	1.06	40	u	0.385305
230727_at	3	5.081	0.49	7	5.37	1.04	40	u	0.483921
222496_s_at	3	8.926	0.39	7	8.426	1.38	40	d	0.075643
219214_s_at	3	5.805	0.34	7	5.588	0.49	40	d	0.274259
241619_at	3	3.996	0.31	7	3.887	0.49	40	d	0.578158
238366_at	3	2.408	0.25	7	2.241	0.22	40	d	0.081603
209701_at	3	4.48	0.31	7	5.526	0.73	40	u	0.000683
201836_s_at	3	5.738	0.57	7	5.024	0.95	40	d	0.064589
225809_at	3	7.439	0.57	7	5.78	1.92	40	d	0.000166
208854_s_at	3	8.678	0.35	7	8.455	1.06	40	d	0.326444
226198_at	3	8.343	0.4	7	6.993	0.82	40	d	0.000127
238974_at	3	8.871	0.22	7	9.504	0.47	40	u	0.001411
1563792_at	3	2.658	0.2	7	2.8	0.29	40	u	0.228834
238365_s_at	3	3.05	0.24	7	3.083	0.53	40	u	0.874478
235070_at	3	3.411	0.53	7	3.617	0.59	40	u	0.405225
200961_at	3	8.867	0.46	7	9.193	0.66	40	u	0.225032
225901_at	3	6.837	0.48	7	6.551	1.03	40	d	0.483833
204289_at	3	2.904	0.14	7	2.797	0.2	40	d	0.194053
213398_s_at	3	9.01	0.25	7	8.205	0.7	40	d	0.000022
226592_at	3	6.983	0.56	7	6.16	0.93	40	d	0.031134
223530_at	3	4.034	0.79	7	5.974	1.32	40	u	0.00059
218417_s_at	3	6.656	0.09	7	5.891	0.63	40	d	0
204947_at	3	5.841	0.2	7	6.45	0.79	40	u	0.000287
226267_at	3	8.721	0.44	7	7.74	0.84	40	d	0.004781
200968_s_at	3	11.6	0.29	7	11.14	0.68	40	d	0.093523
203066_at	3	8.563	0.33	7	7.824	1.34	40	d	0.005721
232057_at	3	5.673	0.37	7	6.296	0.6	40	u	0.012515
1555409_a_at	3	3.048	0.2	7	4.049	0.96	40	u	0.000001
227386_s_at	3	6.407	0.31	7	5.879	1.22	40	d	0.029709

225162_at	3	9.517	0.29	7	7.419	0.63	40	d	0
228526_at	3	2.563	0.04	7	2.618	0.06	40	u	0.038447
227292_at	3	5.382	0.54	7	6.157	0.86	40	u	0.02964
205021_s_at	3	4.584	0.7	7	4.746	0.95	40	u	0.675383
225466_at	3	7.624	0.28	7	7.519	0.78	40	d	0.541963
235460_at	3	5.193	0.29	7	5.304	0.56	40	u	0.619289
207433_at	3	2.651	0.06	7	2.781	0.19	40	u	0.002016
200655_s_at	3	10.83	0.13	7	10.6	0.65	40	d	0.05372
48106_at	3	7.343	0.27	7	6.63	0.63	40	d	0.0059
200967_at	3	11.95	0.22	7	11.76	0.58	40	d	0.417065
203753_at	3	10.47	0.37	7	9.503	0.91	40	d	0.009271
242305_at	3	4.178	0.48	7	3.971	0.43	40	d	0.26284
1562253_at	3	2.484	0.19	7	2.503	0.21	40	u	0.826988
238367_s_at	3	2.684	0.34	7	2.571	0.08	40	d	0.455839
238587_at	3	4.053	0.28	7	4.214	0.74	40	u	0.581328
220493_at	3	2.805	0.08	7	3.034	0.44	40	u	0.004938
229521_at	3	7.288	0.51	7	7.466	1.13	40	u	0.689157
225039_at	2	7.479	0.17	7	8.089	0.5	40	u	0.000006
219049_at	2	8.021	0.45	7	6.521	1.2	40	d	0.00248
208277_at	2	3.091	0.35	7	2.921	0.43	40	d	0.338816
235815_at	2	4.89	0.47	7	4.501	0.47	40	d	0.055479
230052_s_at	2	4.241	0.34	7	4.002	0.53	40	d	0.264095
200653_s_at	2	11.15	0.19	7	11.15	0.57	40	d	0.993825
212081_x_at	2	8.154	0.29	7	7.716	0.72	40	d	0.129597
1566884_at	2	2.951	0.16	7	3.108	0.44	40	u	0.369284
212280_x_at	2	8.488	0.15	7	8.215	0.78	40	d	0.056106
229535_at	2	6.328	0.26	7	6.274	0.65	40	d	0.834659
214201_x_at	2	7.594	0.34	7	7.019	0.73	40	d	0.049952
221080_s_at	2	4.544	0.39	7	4.864	0.56	40	u	0.159651
221834_at	2	9.808	0.54	7	7.832	1.29	40	d	0.000309
239983_at	2	2.176	0.02	7	2.781	1.43	40	u	0.011587
222868_s_at	2	4.682	0.6	7	5.033	1.04	40	u	0.400895
233065_at	2	5.499	0.46	7	6.274	0.47	40	u	0.000293
52731_at	2	6.456	0.25	7	6.501	0.64	40	u	0.858281
210791_s_at	2	6.356	0.74	7	5.773	1.29	40	d	0.26143
235605_at	2	6.977	0.3	7	6.442	0.73	40	d	0.068039
205253_at	2	7.229	0.34	7	7.979	1.42	40	u	0.007654
201609_x_at	2	7.992	0.3	7	8.83	0.6	40	u	0.001013
1554668_a_at	2	1.826	0.07	7	1.99	0.84	40	u	0.23858
218752_at	2	6.189	0.2	7	6.27	0.53	40	u	0.694626
228353_x_at	2	2.822	0.13	7	3.084	0.38	40	u	0.003121
224639_at	2	9.17	0.25	7	8.686	0.58	40	d	0.03904
225040_s_at	2	7.699	0.13	7	8.335	0.55	40	u	0
205022_s_at	2	9.281	0.32	7	8.238	0.55	40	d	0.00002
226041_at	2	5.774	0.54	7	6.149	0.82	40	u	0.261328
218509_at	2	7.083	0.17	7	6.58	0.76	40	d	0.000836
231397_at	2	2.248	0.08	7	2.282	0.06	40	u	0.222104
201220_x_at	2	10.6	0.25	7	10.62	0.55	40	u	0.922718
219383_at	2	3.641	0.35	7	4.037	0.67	40	u	0.143796
231643_s_at	2	3.772	0.36	7	3.844	0.52	40	u	0.733385
203419_at	2	7.786	0.33	7	7.253	0.71	40	d	0.063034
201611_s_at	2	7.69	0.2	7	7.266	0.63	40	d	0.002917
242922_at	2	6.097	0.42	7	5.647	0.72	40	d	0.125104
1558662_s_at	2	2.668	0.21	7	3.09	1.04	40	u	0.028619
205505_at	2	4.635	0.45	7	5.01	1.29	40	u	0.187585
213062_at	2	7.128	0.31	7	7.123	0.78	40	d	0.986815
1555550_at	2	3.688	0.41	7	3.675	0.53	40	d	0.95099
1558420_at	2	3.123	0.42	7	2.601	0.47	40	d	0.009505
224925_at	2	8.191	0.47	7	6.54	1.67	40	d	0.000017
203166_at	2	8.818	0.12	7	8.478	0.74	40	d	0.011669
209850_s_at	2	6.602	0.35	7	5.755	0.51	40	d	0.000153
210332_at	2	5.75	0.31	7	5.549	0.32	40	d	0.136286
224740_at	2	9.428	0.22	7	9.385	0.62	40	d	0.751647
212275_s_at	2	7.378	0.35	7	6.826	0.64	40	d	0.035688

233475_at	2	2.333	0.15	7	2.42	0.19	40	u	0.273033
219667_s_at	2	3.043	0.76	7	3.401	1.29	40	u	0.49135
218526_s_at	2	7.549	0.2	7	6.922	0.95	40	d	0.000708
64899_at	2	7.559	0.4	7	7.556	0.66	40	d	0.989479
202395_at	2	8.739	0.17	7	8.777	0.7	40	u	0.778092
228359_at	2	2.37	0.17	7	2.79	0.89	40	u	0.011432
222146_s_at	2	9.278	0.5	7	7.92	1.11	40	d	0.00333
224886_at	2	7.857	0.23	7	7.428	0.54	40	d	0.047636
226839_at	2	6.454	0.3	7	7.035	0.79	40	u	0.066541
64438_at	2	5.71	0.47	7	5.782	0.6	40	u	0.771316
1558711_at	2	3.82	0.67	7	3.579	0.67	40	d	0.39503
223452_s_at	2	6.267	0.42	7	7.172	1.12	40	u	0.044942
200996_at	2	9.928	0.31	7	11.09	0.76	40	u	0.000318
204124_at	2	5.612	1.33	7	5.305	1.66	40	d	0.651254
214771_x_at	2	9.486	0.23	7	9.213	0.65	40	d	0.062077
204634_at	2	6.939	0.29	7	7.299	0.68	40	u	0.1863
209452_s_at	2	9.973	0.19	7	9.435	0.65	40	d	0.000263
202175_at	2	7.37	0.32	7	7.419	0.6	40	u	0.839373
224909_s_at	2	8.554	0.64	7	7.246	1.83	40	d	0.002716
227871_at	2	8.102	0.49	7	7.735	0.56	40	d	0.117344
233049_x_at	2	9.961	0.12	7	9.891	0.64	40	d	0.538436
214694_at	2	2.669	0.52	7	2.838	0.43	40	u	0.369317
223070_at	2	10.69	0.2	7	10.34	0.59	40	d	0.010578
235032_at	2	7.157	0.31	7	7.548	0.72	40	u	0.17329
1553042_a_at	2	3.146	0.46	7	2.902	0.47	40	d	0.221189
1557162_at	2	2.882	0.43	7	2.901	0.31	40	u	0.892088
1569640_s_at	2	2.486	0.28	7	2.773	0.38	40	u	0.066187
225631_at	2	4.762	0.47	7	4.274	0.38	40	d	0.0053
228082_at	2	5.648	0.87	7	4.354	0.95	40	d	0.001851
230893_at	2	6.274	0.47	7	6.38	0.84	40	u	0.750185
204666_s_at	2	3.348	0.42	7	4.742	1.41	40	u	0.000026
224480_s_at	2	5.518	0.56	7	5.369	1.45	40	d	0.794728
226583_at	2	6.463	0.25	7	6.2	0.83	40	d	0.125834
203431_s_at	2	8.578	0.64	7	7.42	1.59	40	d	0.069127
225630_at	2	4.447	0.58	7	3.574	0.52	40	d	0.000268
47553_at	2	6.193	0.53	7	5.955	1.23	40	d	0.62436
224640_at	2	7.921	0.25	7	7.691	0.54	40	d	0.285913
222915_s_at	2	3.258	0.62	7	3.594	0.8	40	u	0.306833
213097_s_at	2	8.836	0.24	7	9.575	0.61	40	u	0.003229
221833_at	2	8.618	0.71	7	6.756	1.56	40	d	0.004074
1553362_at	2	2.301	0.02	7	2.449	0.42	40	u	0.036737
208132_x_at	2	8.075	0.26	7	7.648	0.73	40	d	0.012018
205414_s_at	2	5.004	0.54	7	4.272	0.84	40	d	0.034497
209526_s_at	2	7.897	0.45	7	7.986	1.24	40	u	0.855211
230561_s_at	2	3.991	0.24	7	4.169	0.54	40	u	0.407206
223554_s_at	2	5.833	0.21	7	5.524	0.49	40	d	0.115885
215116_s_at	2	7.621	0.44	7	6.112	1.4	40	d	0.000014
201610_at	2	3.234	0.23	7	3.271	0.2	40	u	0.663798
209197_at	2	3.082	0.84	7	3.79	1.05	40	u	0.103886
212197_x_at	2	9.357	0.19	7	9.008	0.73	40	d	0.018559
1566885_at	2	2.127	0.23	7	2.14	0.28	40	u	0.91591
214194_at	2	5.238	0.69	7	4.992	0.73	40	d	0.423407
221887_s_at	2	6.76	0.43	7	6.507	1.02	40	d	0.532575
232859_s_at	2	6.416	0.28	7	5.867	0.57	40	d	0.018706
218648_at	2	9.147	0.18	7	8.818	0.65	40	d	0.01449
221762_s_at	2	7.095	0.3	7	6.742	0.6	40	d	0.145235
212787_at	2	8.908	0.17	7	8.048	0.72	40	d	0
223775_at	2	2.434	0.08	7	2.584	0.31	40	u	0.015898
1558421_a_at	2	2.683	0.51	7	2.284	0.11	40	d	0.10386
219141_s_at	2	6.675	0.1	7	6.501	0.46	40	d	0.04349
224638_at	2	6.386	0.45	7	5.804	0.56	40	d	0.015002
1558987_at	2	2.532	0.18	7	2.418	0.18	40	d	0.145115
229421_s_at	2	3.402	0.44	7	3.441	0.51	40	u	0.853329
221705_s_at	2	5.795	0.36	7	7.52	0.91	40	u	0.000015

226859_at	2	6.301	0.28	7	6.235	0.55	40	d	0.763009
221770_at	2	7.413	0.34	7	8.2	0.75	40	u	0.010426
217934_x_at	2	9.11	0.13	7	9.049	0.58	40	d	0.575294
214014_at	2	3.699	0.69	7	3.034	0.65	40	d	0.019308
227876_at	2	5.309	0.6	7	5.898	0.72	40	u	0.05121
213061_s_at	2	8.36	0.51	7	8.476	0.67	40	u	0.668848
239761_at	2	5.948	0.54	7	6.77	1.38	40	u	0.136098
219254_at	2	4.875	0.47	7	4.67	0.88	40	d	0.559774
239844_x_at	2	5.6	0.27	7	5.464	0.26	40	d	0.214307
213667_at	2	5.731	0.5	7	6.458	0.91	40	u	0.049825
53071_s_at	2	7.481	0.32	7	7.594	1.02	40	u	0.594511
224129_s_at	2	9.905	0.26	7	10.5	0.57	40	u	0.010807
234952_s_at	2	6.817	0.41	7	6.464	0.49	40	d	0.084806
214812_s_at	2	10.26	0.25	7	10.06	0.61	40	d	0.426589
204665_at	2	1.958	0.02	7	2.242	0.37	40	u	0.000026
223453_s_at	2	2.854	0.34	7	2.963	0.42	40	u	0.532001
215952_s_at	2	12.31	0.21	7	12.23	0.57	40	d	0.733954
1552722_at	2	2.895	0.07	7	3.104	0.29	40	u	0.00051
222189_at	2	2.049	0.05	7	2.076	0.05	40	u	0.212297
218849_s_at	2	8.174	0.36	7	7.012	1.05	40	d	0.000027
202631_s_at	2	5.653	0.47	7	6.828	0.92	40	u	0.002284
228477_at	1	9.917	0.5	7	8.897	0.73	40	d	0.001132
221190_s_at	1	7.143	0.36	7	7.382	0.71	40	u	0.39965
202760_s_at	1	8.793	0.59	7	8.118	0.85	40	d	0.053607
228530_at	1	2.263	0.08	7	2.63	0.64	40	u	0.001352
222115_x_at	1	5.919	0.27	7	5.986	0.88	40	u	0.712504
227617_at	1	4.977	0.37	7	4.798	0.62	40	d	0.472403
220134_x_at	1	7.97	0.56	7	6.83	1.03	40	d	0.007498
201871_s_at	1	9.738	0.23	7	8.983	0.69	40	d	0.000019
219659_at	1	3.508	0.66	7	3.606	1.27	40	u	0.845545
233971_at	1	2.312	0.18	7	2.258	0.31	40	d	0.668163
221435_x_at	1	7.189	0.27	7	6.043	0.74	40	d	0
203071_at	1	6.158	0.45	7	5.277	1.55	40	d	0.007331
224902_at	1	7.896	0.55	7	7.176	0.96	40	d	0.065713
207583_at	1	2.623	0.08	7	2.781	0.3	40	u	0.009864
230329_s_at	1	6.308	0.43	7	6.101	0.83	40	d	0.530966
212946_at	1	7.323	0.41	7	6.322	0.78	40	d	0.002194
218584_at	1	7.68	0.53	7	6.901	0.93	40	d	0.039619
208753_s_at	1	9.762	0.17	7	10.04	0.59	40	u	0.020947
228865_at	1	5.702	1.36	7	4.648	1.65	40	d	0.125158
203366_at	1	8.562	0.2	7	8.628	0.39	40	u	0.664923
210623_at	1	5.356	0.27	7	5.331	0.51	40	d	0.903415
231287_s_at	1	2.386	0.27	7	2.374	0.12	40	d	0.917835
213375_s_at	1	8.962	0.21	7	7.146	1.19	40	d	0
218517_at	1	8.333	0.48	7	7.5	0.73	40	d	0.006883
212129_at	1	9.318	0.29	7	9.723	0.6	40	u	0.091669
211452_x_at	1	10.68	0.33	7	10.28	0.67	40	d	0.13975
1555486_a_at	1	4.901	0.33	7	5.482	0.74	40	u	0.050333
208308_s_at	1	9.873	0.17	7	10.26	0.86	40	u	0.015348
203959_s_at	1	7.852	0.36	7	7.254	0.52	40	d	0.006687
236995_x_at	1	3.365	0.2	7	3.813	0.44	40	u	0.012136
208091_s_at	1	8.757	0.28	7	9.608	0.9	40	u	0.000063
1554468_s_at	1	3.462	0.21	7	3.833	0.62	40	u	0.008453
218067_s_at	1	11.1	0.17	7	10.01	0.78	40	d	0
219169_s_at	1	6.666	0.3	7	6.878	0.67	40	u	0.422298
222498_at	1	7.796	0.28	7	7.119	0.8	40	d	0.000569
52169_at	1	7.801	0.27	7	7.481	0.79	40	d	0.066725
218215_s_at	1	7.214	0.28	7	6.617	0.48	40	d	0.00324
215207_x_at	1	4.839	0.5	7	5.611	1.21	40	u	0.111073
220358_at	1	4.549	0.6	7	4.632	0.64	40	u	0.757695
201861_s_at	1	8.716	0.32	7	8.59	0.66	40	d	0.628895
221052_at	1	2.611	0.21	7	2.969	0.86	40	u	0.034343
1555360_a_at	1	6.005	0.45	7	6.214	0.71	40	u	0.46587
200056_s_at	1	8.247	0.14	7	8.507	0.69	40	u	0.043127

226631_at	1	5.867	0.41	7	6.267	0.61	40	u	0.107128
227625_s_at	1	11.46	0.14	7	11.44	0.63	40	d	0.850693
224904_at	1	7.606	0.63	7	6.857	0.92	40	d	0.049557
225103_at	1	8.524	0.36	7	8.466	0.93	40	d	0.873872
237205_at	1	2.934	0.12	7	3.228	0.33	40	u	0.02645
226499_at	1	6.949	0.35	7	7.063	0.8	40	u	0.717941
231407_s_at	1	4.417	0.36	7	4.622	0.45	40	u	0.269861
210835_s_at	1	10.49	0.29	7	10.62	0.59	40	u	0.563165
221053_s_at	1	2.199	0.02	7	2.467	0.66	40	u	0.015138
243386_at	1	6.625	0.75	7	5.404	1.49	40	d	0.043797
230106_at	1	3.362	0.57	7	4.071	0.84	40	u	0.041695
229054_at	1	6.082	0.3	7	5.798	0.71	40	d	0.31409
222125_s_at	1	8.498	0.24	7	7.39	1.53	40	d	0.000125
203958_s_at	1	4.661	0.3	7	4.936	0.53	40	u	0.199655
203443_at	1	2.047	0.16	7	2.101	0.19	40	u	0.499113
219569_s_at	1	4.289	0.34	7	4.255	1	40	d	0.872188
222776_at	1	2.403	0.19	7	2.696	0.72	40	u	0.043482
214725_at	1	4.36	0.42	7	4.16	0.84	40	d	0.550377
226108_at	1	6.783	0.26	7	6.696	0.49	40	d	0.657798
220341_s_at	1	6.358	0.57	7	5.116	0.82	40	d	0.000449
217774_s_at	1	11.42	0.18	7	11.28	0.56	40	d	0.205176
225578_at	1	7.921	0.36	7	8.679	0.7	40	u	0.008958
213493_at	1	4.5	0.53	7	4.054	0.77	40	d	0.158011
219660_s_at	1	2.27	0.15	7	2.387	0.47	40	u	0.233292
233134_at	1	3.292	0.1	7	3.323	0.12	40	u	0.540602
37079_at	1	3.282	0.26	7	4.308	1	40	u	0.000005
1552621_at	1	8.188	0.46	7	8.258	0.8	40	u	0.825934
229626_at	1	2.248	0.02	7	2.329	0.22	40	u	0.032038
1552279_a_at	1	5.837	0.62	7	5.635	0.54	40	d	0.388515
233098_s_at	1	2.505	0.14	7	2.537	0.11	40	u	0.523351
212823_s_at	1	6.256	0.36	7	5.862	0.54	40	d	0.074943
209679_s_at	1	7.069	0.23	7	7.161	1.16	40	u	0.658604
227752_at	1	4.669	0.53	7	4.181	1.12	40	d	0.274613
51158_at	1	8.268	0.44	7	7.309	1.09	40	d	0.029828
221922_at	1	6.7	0.5	7	8.483	0.99	40	u	0.000037
1553159_at	1	3.364	0.3	7	3.407	0.36	40	u	0.770419
226634_at	1	5.164	0.4	7	4.529	1.03	40	d	0.122579
232674_at	1	3.507	0.48	7	3.19	0.59	40	d	0.1957
219045_at	1	5.029	0.19	7	5.545	0.6	40	u	0.000239
209608_s_at	1	7.556	0.48	7	8.255	0.81	40	u	0.034848
35148_at	1	7.346	0.43	7	6.968	1.3	40	d	0.178455
1554798_at	1	3.092	0.47	7	3.015	0.55	40	d	0.733598
228234_at	1	5.81	0.42	7	6.546	0.81	40	u	0.027106
227595_at	1	5.448	0.42	7	5.25	0.63	40	d	0.433341
222524_s_at	1	5.723	0.32	7	5.277	0.47	40	d	0.022028
220183_s_at	1	5.172	0.41	7	4.97	0.87	40	d	0.557724
214553_s_at	1	6.29	0.56	7	6.712	0.93	40	u	0.260526
238337_s_at	1	7.756	0.23	7	8.12	0.56	40	u	0.101922
218429_s_at	1	8.19	0.27	7	7.6	1.08	40	d	0.006805
221586_s_at	1	5.755	0.19	7	7.067	1.36	40	u	0.000001
212821_at	1	5.264	0.37	7	5.025	0.65	40	d	0.362092
212314_at	1	7.172	0.52	7	7.375	1.37	40	u	0.707489
222559_s_at	1	8.226	0.33	7	8.244	1.14	40	u	0.939117
227268_at	1	6.751	0.33	7	7.282	0.95	40	u	0.014838
220987_s_at	1	4.484	0.4	7	5.454	0.89	40	u	0.008318
1558519_at	1	2.687	0.06	7	2.877	0.25	40	u	0.000189
221554_at	1	6.976	0.3	7	6.746	0.72	40	d	0.421666
217945_at	1	10.25	0.26	7	10.51	0.64	40	u	0.318621
212311_at	1	5.666	0.69	7	5.781	1.8	40	u	0.870653
228122_at	1	5.295	0.38	7	4.849	0.87	40	d	0.197147
218623_at	1	3.006	0.24	7	3.159	0.76	40	u	0.334934
1555288_s_at	1	6.466	0.16	7	6.837	0.54	40	u	0.001518
226206_at	1	8.286	0.55	7	7.07	0.83	40	d	0.00067
226290_at	1	9.376	0.42	7	8.679	0.84	40	d	0.04009

223769_x_at	1	7.606	0.24	7	6.443	0.88	40	d	0
218662_s_at	1	6.467	0.49	7	8.532	0.8	40	u	0
201219_at	1	8.192	0.34	7	7.68	0.71	40	d	0.074486
205556_at	1	3.65	0.42	7	3.372	0.27	40	d	0.03047
1552622_s_at	1	7.536	0.54	7	7.506	1.15	40	d	0.947048
230353_at	1	6.507	0.38	7	5.175	0.4	40	d	0
226207_at	1	7.603	0.23	7	6.803	0.67	40	d	0.000007
213488_at	1	6.896	0.45	7	5.995	1.01	40	d	0.028347
228565_at	1	5.179	0.45	7	5.979	1.49	40	u	0.012232
222114_x_at	1	4.504	0.38	7	4.083	0.66	40	d	0.113772
227594_at	1	4.676	0.24	7	5.304	0.64	40	u	0.01642
202929_s_at	1	8.585	0.49	7	8.219	1.03	40	d	0.372389
1562568_at	1	2.436	0.41	7	2.315	0.11	40	d	0.496596
210069_at	1	7.368	0.49	7	6.57	1.26	40	d	0.11163
209791_at	1	9.136	0.79	7	7.951	1.52	40	d	0.054446
207771_at	1	4.494	0.53	7	4.563	0.35	40	u	0.662972
225561_at	1	7.452	0.25	7	7.449	0.69	40	d	0.990019
223459_s_at	1	5.467	0.27	7	5.451	0.61	40	d	0.94665
208905_at	1	10.76	0.3	7	11.58	0.54	40	u	0.000367
222508_s_at	1	4.892	0.7	7	4.57	0.81	40	d	0.341521
218117_at	1	10.48	0.21	7	10.75	0.6	40	u	0.052123
1562640_at	1	2.621	0.04	7	2.818	0.31	40	u	0.000449
1555491_a_at	1	6.274	0.38	7	6.061	0.98	40	d	0.58093
201497_x_at	1	10.82	0.73	7	6.02	1.09	40	d	0
36030_at	1	6.311	0.41	7	5.37	0.78	40	d	0.003928
210319_x_at	1	7.11	0.76	7	5.755	1.33	40	d	0.013991
214226_at	1	5.27	0.11	7	5.094	0.63	40	d	0.119155
222558_at	1	5.328	0.42	7	5.692	1.08	40	u	0.39336
228019_s_at	1	9.148	0.31	7	9.134	0.61	40	d	0.95409
222085_at	1	2.132	0.02	7	2.187	0.04	40	u	0.002738
238613_at	1	7.339	0.59	7	6.556	0.82	40	d	0.022238
218894_s_at	1	4.798	0.38	7	6.7	1.19	40	u	0
205634_x_at	1	6.243	0.45	7	6.777	0.93	40	u	0.152608
227253_at	1	6.369	1.74	7	6.856	2.35	40	u	0.610866
223754_at	1	3.136	0.44	7	3.15	0.38	40	u	0.931958
204528_s_at	1	10.41	0.24	7	10.45	0.52	40	u	0.828336
225953_at	1	8.664	0.36	7	8.951	0.79	40	u	0.362455
38766_at	1	7.08	0.36	7	7.578	0.64	40	u	0.055666
222986_s_at	1	9.719	0.13	7	9.636	0.68	40	d	0.497311
227862_at	1	6.923	0.39	7	5.642	0.98	40	d	0.001757
214099_s_at	1	3.727	0.59	7	4.37	0.88	40	u	0.076537
203053_at	1	9.7	0.24	7	9.688	0.72	40	d	0.935759
229712_at	1	3.146	0.29	7	3.428	0.68	40	u	0.296076
213778_x_at	1	5.992	0.19	7	5.625	0.62	40	d	0.006541
203442_x_at	1	8.148	0.2	7	7.438	0.64	40	d	0.000009
221710_x_at	1	8.036	0.53	7	7.029	0.99	40	d	0.013497
205825_at	1	3.411	1.16	7	3.414	1.6	40	u	0.996313
239431_at	1	4.019	0.34	7	4.238	0.55	40	u	0.326038
239697_x_at	1	3.291	0.1	7	3.768	0.71	40	u	0.000301
218209_s_at	1	6.127	0.55	7	7.674	1.05	40	u	0.000547
212767_at	1	6.987	0.21	7	7.014	0.68	40	u	0.845579
238336_s_at	1	3.935	0.21	7	4.276	0.4	40	u	0.037065
226410_at	1	6.52	0.27	7	6.639	0.43	40	u	0.493952
238335_at	1	2.486	0.1	7	3.236	0.98	40	u	0.000035
227106_at	1	7.048	0.14	7	5.783	0.63	40	d	0
214773_x_at	1	8.429	0.22	7	9.59	0.73	40	u	0
1552278_a_at	1	2.551	0.26	7	2.965	0.76	40	u	0.015971
218663_at	1	3.519	0.32	7	6.794	1.07	40	u	0
202553_s_at	1	9.774	0.13	7	9.134	0.41	40	d	0
231986_at	1	2.773	0.7	7	2.555	0.8	40	d	0.509418
215046_at	1	4.286	0.21	7	4.491	0.78	40	u	0.181777
226796_at	1	4.393	0.34	7	4.327	0.61	40	d	0.787591
214840_at	1	4.967	0.61	7	4.735	0.66	40	d	0.400898
222948_s_at	1	2.556	0.18	7	2.685	0.21	40	u	0.137006

226909_at	1	6.797	0.76	7	6.945	1.28	40	u	0.772092
1556136_at	1	4.249	0.23	7	4.22	0.52	40	d	0.890921
216242_x_at	1	8.452	0.39	7	8.217	0.76	40	d	0.43724
220103_s_at	1	3.319	0.28	7	3.706	0.44	40	u	0.033138
221007_s_at	1	7.57	0.12	7	7.871	0.54	40	u	0.004546
201862_s_at	1	9.331	0.31	7	9.185	0.9	40	d	0.452005
225820_at	1	4.378	0.33	7	4.424	0.87	40	u	0.892948
215505_s_at	1	3.558	0.93	7	3.297	0.64	40	d	0.371399
218507_at	1	7.544	0.71	7	8.785	1.04	40	u	0.004757
223967_at	1	2.421	0.11	7	2.5	0.14	40	u	0.170973
209721_s_at	1	6.516	0.53	7	6.17	0.52	40	d	0.116686
1558139_at	1	6.536	0.58	7	6.281	0.93	40	d	0.498569
1554424_at	1	5.119	0.28	7	5.016	0.56	40	d	0.643904
244546_at	1	3.776	0.27	7	4.046	0.76	40	u	0.116826
227250_at	1	6.487	0.39	7	5.379	1.06	40	d	0.00008
207007_at	1	2.331	0.18	7	2.338	0.19	40	u	0.927169
220999_s_at	1	4.951	0.58	7	5.344	1.17	40	u	0.399618
227937_at	1	5.97	0.39	7	5.998	0.71	40	u	0.920928
235210_s_at	1	7.87	0.92	7	5.702	2.21	40	d	0.016098
219476_at	1	6.596	0.66	7	4.779	2.12	40	d	0.000258
1562778_at	1	4.003	0.4	7	3.974	0.54	40	d	0.893697
239470_at	1	2.441	0.06	7	2.605	0.28	40	u	0.00317
1556131_s_at	1	5.105	0.27	7	5.268	0.49	40	u	0.406642
1557303_at	1	4.065	0.24	7	4.177	0.47	40	u	0.546659
228133_s_at	1	4.77	0.34	7	2.672	0.34	40	d	0
221880_s_at	1	7.718	0.57	7	6.467	1.3	40	d	0.017889
223622_s_at	1	7.261	0.19	7	5.968	0.91	40	d	0
224407_s_at	1	4.703	0.71	7	6.421	1.63	40	u	0.010015
210070_s_at	1	7.087	0.36	7	6.286	1.13	40	d	0.001858
231922_at	1	5.219	0.38	7	4.555	0.61	40	d	0.009443
224573_at	1	11.46	0.17	7	10.8	0.67	40	d	0.000008
210554_s_at	1	10.5	0.25	7	10.62	0.58	40	u	0.581841
243875_at	1	3.102	0.41	7	2.748	0.28	40	d	0.006974
212406_s_at	1	9.496	0.32	7	9.097	0.97	40	d	0.059913
1557458_s_at	1	6.756	0.35	7	7.274	0.81	40	u	0.110866
228743_at	1	5.568	0.34	7	5.467	0.97	40	d	0.632165
201218_at	1	9.567	0.44	7	9.802	0.61	40	u	0.340498
229415_at	1	5.862	0.33	7	5.154	0.7	40	d	0.0138
1554017_at	1	2.974	0.35	7	3	0.3	40	u	0.838883
228075_x_at	1	6.733	0.22	7	7.017	0.61	40	u	0.041477
205240_at	1	5.569	0.72	7	7.532	1.45	40	u	0.001316
235390_at	1	6.263	0.7	7	5.978	0.8	40	d	0.393055
221614_s_at	1	6.335	0.47	7	5.403	0.81	40	d	0.0057
1555952_at	1	1.991	0.02	7	2.035	0.06	40	u	0.000773
201496_x_at	1	7.223	0.93	7	3.692	0.51	40	d	0.000099
227043_at	1	6.806	0.23	7	5.797	0.74	40	d	0
219714_s_at	1	3.029	0.36	7	2.975	0.55	40	d	0.804313
1554417_s_at	1	7.884	0.63	7	8.201	1.81	40	u	0.419548
228134_at	1	1.991	0.13	7	2.041	0.15	40	u	0.408551
225816_at	1	7.341	0.55	7	6.541	0.79	40	d	0.014923
1553677_a_at	1	6.691	0.29	7	8.157	0.71	40	u	0.000003
225172_at	1	7.611	0.3	7	6.964	0.7	40	d	0.023513
203070_at	1	2.709	0.16	7	2.723	0.19	40	u	0.860607
235209_at	1	7.558	0.98	7	6.004	1.84	40	d	0.038085
227225_at	1	4.679	0.39	7	4.306	0.41	40	d	0.035321
1558041_a_at	1	4.894	0.53	7	4.596	0.9	40	d	0.408672
217226_s_at	1	8.106	0.26	7	7.701	0.71	40	d	0.016007
1558703_at	1	6.85	0.85	7	5.642	0.97	40	d	0.004142
218021_at	1	8.85	0.25	7	7.954	0.69	40	d	0.001674
222812_s_at	1	2.8	0.28	7	2.807	0.56	40	u	0.973966
218499_at	1	7.665	0.4	7	9.243	1.22	40	u	0.000001
53720_at	1	8.176	0.23	7	7.749	0.89	40	d	0.016913
222811_at	1	7.622	0.41	7	7.784	0.64	40	u	0.533794
201495_x_at	1	2.551	0.51	7	2.298	0.21	40	d	0.273444

	228845_at	1	5.687	0.25	7	5.046	0.73	40	d	0.000343
	226353_at	1	8.953	0.48	7	9.14	0.8	40	u	0.562231
	227717_at	1	9.057	0.59	7	7.221	0.99	40	d	0.000027
	1557091_at	1	3.113	0.25	7	3.627	0.42	40	u	0.003524
	230468_s_at	1	2.705	0.08	7	2.788	0.14	40	u	0.14647
