# Supplementary material for: Increased MicroRNA Activity in Human Cancers
Source: PLoS One. 2009 Jun 25;4(6):e6045. doi: 10.1371/journal.pone.0006045 (PMC2698213; doi:10.1371/journal.pone.0006045)
Supplement: Table S6 — GO annotation enrichment analysis for the targets of the 77 miR-seeds upregulated in breast cancer (0.04 MB PDF) [file pone.0006045.s007.pdf]

Supplemental Table 6. GO annotation enrichment analysis for the targets of the 77 miR-seeds upregulated in breast cancer

A. Most enriched GO annotations in the 9542 probe-sets corresponding to genes that carry target sites for the 77 miR-seeds undergoing the most significant increase of activity in breast cancer (compared to annotations available for the 40,549 probe-sets assigned to genes on the array)

GO Annotation (biological process)	p-value	FDR	N	n	K	k
regulation of transcription, DNA-dependent	1.48E-49	3.09E-46	40539	4611	9542	1499
transcription	2.39E-39	2.49E-36	40539	4237	9542	1350
multicellular organismal development	3.05E-37	2.12E-34	40539	1907	9542	690
ubiquitin cycle	2.37E-25	1.23E-22	40539	1032	9542	390
small GTPase mediated signal transduction	5.96E-25	2.49E-22	40539	552	9542	239
regulation of transcription	1.01E-24	3.51E-22	40539	999	9542	378
protein transport	1.10E-24	3.26E-22	40539	1123	9542	415
nervous system development	2.20E-24	5.72E-22	40539	780	9542	310
transport	3.96E-24	9.17E-22	40539	3427	9542	1053
cell differentiation	1.61E-22	3.36E-20	40539	940	9542	353
endocytosis	1.42E-19	2.70E-17	40539	274	9542	133
protein amino acid phosphorylation	4.72E-18	8.19E-16	40539	1403	9542	470
regulation of transcription from RNA polymerase II promoter	7.19E-18	1.15E-15	40539	536	9542	215
regulation of translation	1.29E-14	1.92E-12	40539	111	9542	64
Wnt receptor signaling pathway	1.44E-14	2.01E-12	40539	244	9542	112
protein modification process	1.64E-14	2.14E-12	40539	603	9542	225
positive regulation of transcription from RNA polymerase II promoter	4.03E-14	4.94E-12	40539	286	9542	125
positive regulation of transcription, DNA-dependent	2.13E-13	2.46E-11	40539	243	9542	109
negative regulation of transcription, DNA-dependent	1.13E-11	1.24E-09	40539	169	9542	80
regulation of progression through cell cycle	2.00E-10	2.09E-08	40539	624	9542	216
cell fate determination	2.12E-10	2.10E-08	40539	41	9542	29
intracellular protein transport	1.29E-09	1.22E-07	40539	466	9542	167
anterior/posterior pattern formation	1.37E-09	1.24E-07	40539	88	9542	47
chromatin modification	1.48E-09	1.29E-07	40539	389	9542	144
ubiquitin-dependent protein catabolic process	5.82E-09	4.85E-07	40539	349	9542	130
transcription from RNA polymerase II promoter	8.20E-09	6.57E-07	40539	487	9542	170
vesicle-mediated transport	1.07E-08	8.26E-07	40539	342	9542	127
response to stress	3.20E-08	2.38E-06	40539	216	9542	87
anatomical structure morphogenesis	9.84E-08	7.07E-06	40539	263	9542	100
cell motility	1.28E-07	8.88E-06	40539	338	9542	122

B. Most enriched GO annotations among probe-sets regulated by the 77 miRNAs and are downregulated in tumors (compared to the 9542 predicted target probe-sets)

GO Annotation (biological process)	p-value	FDR	N	n	K	k
cell cycle arrest	1.15E-05	1.85E-02	9542	78	5069	60
regulation of cell shape	1.07E-04	8.64E-02	9542	46	5069	37
organ morphogenesis	1.53E-04	8.19E-02	9542	98	5069	70
cell-matrix adhesion	1.56E-04	6.29E-02	9542	39	5069	32
membrane fusion	1.90E-04	6.10E-02	9542	18	5069	17
response to nutrient	2.66E-04	7.14E-02	9542	13	5069	13
regulation of transcription, DNA-dependent	3.39E-04	7.78E-02	9542	1499	5069	857
transcription	3.61E-04	7.25E-02	9542	1350	5069	775
negative regulation of cell proliferation	6.17E-04	1.10E-01	9542	144	5069	96
signal transduction	9.60E-04	1.55E-01	9542	915	5069	531
cell surface receptor linked signal transduction	1.30E-03	1.89E-01	9542	101	5069	69
insulin receptor signaling pathway	1.89E-03	2.54E-01	9542	39	5069	30
heart development	3.29E-03	4.07E-01	9542	67	5069	47
epidermal growth factor receptor signaling pathway	4.94E-03	5.67E-01	9542	28	5069	22
neural crest cell migration	6.33E-03	6.79E-01	9542	8	5069	8
histone acetylation	6.33E-03	6.36E-01	9542	8	5069	8
response to stress	6.98E-03	6.61E-01	9542	87	5069	58
